# Characteristics that modify the effect of small-quantity lipid-based nutrient supplementation on child anemia and micronutrient status: an individual participant data meta-analysis of randomized controlled trials

**DOI:** 10.1093/ajcn/nqab276

**Published:** 2021-09-29

**Authors:** K Ryan Wessells, Charles D Arnold, Christine P Stewart, Elizabeth L Prado, Souheila Abbeddou, Seth Adu-Afarwuah, Benjamin F Arnold, Per Ashorn, Ulla Ashorn, Elodie Becquey, Kenneth H Brown, Kendra A Byrd, Rebecca K Campbell, Parul Christian, Lia C H Fernald, Yue-Mei Fan, Emanuela Galasso, Sonja Y Hess, Lieven Huybregts, Josh M Jorgensen, Marion Kiprotich, Emma Kortekangas, Anna Lartey, Agnes Le Port, Jef L Leroy, Audrie Lin, Kenneth Maleta, Susana L Matias, Mduduzi N N Mbuya, Malay K Mridha, Kuda Mutasa, Abu M Naser, Rina R Paul, Harriet Okronipa, Jean-Bosco Ouédraogo, Amy J Pickering, Mahbubur Rahman, Kerry Schulze, Laura E Smith, Ann M Weber, Amanda Zongrone, Kathryn G Dewey

**Affiliations:** Institute for Global Nutrition and Department of Nutrition, University of California, Davis, Davis, CA, USA; Institute for Global Nutrition and Department of Nutrition, University of California, Davis, Davis, CA, USA; Institute for Global Nutrition and Department of Nutrition, University of California, Davis, Davis, CA, USA; Institute for Global Nutrition and Department of Nutrition, University of California, Davis, Davis, CA, USA; Public Health Nutrition, Department of Public Health and Primary Care, University of Ghent, Ghent, Belgium; Department of Nutrition and Food Science, University of Ghana, Legon, Accra, Ghana; Francis I Proctor Foundation, University of California, San Francisco, San Francisco, CA, USA; Center for Child Health Research, Faculty of Medicine and Health Technology, Tampere University, Tampere, Finland; Department of Paediatrics, Tampere University Hospital, Tampere, Finland; Center for Child Health Research, Faculty of Medicine and Health Technology, Tampere University, Tampere, Finland; Poverty, Health, and Nutrition Division, International Food Policy Research Institute, Washington, DC, USA; Institute for Global Nutrition and Department of Nutrition, University of California, Davis, Davis, CA, USA; Helen Keller International, New York, NY, USA; WorldFish, Bayan Lepas, Penang, Malaysia; Division of Epidemiology and Biostatistics, University of Illinois at Chicago School of Public Health, Chicago, IL, USA; Program in Human Nutrition, Department of International Health, Johns Hopkins Bloomberg School of Public Health, Baltimore, MD, USA; School of Public Health, University of California, Berkeley, Berkeley, CA, USA; Center for Child Health Research, Faculty of Medicine and Health Technology, Tampere University, Tampere, Finland; Development Research Group, World Bank, Washington, DC, USA; Institute for Global Nutrition and Department of Nutrition, University of California, Davis, Davis, CA, USA; Poverty, Health, and Nutrition Division, International Food Policy Research Institute, Washington, DC, USA; Nutrition Program, College of Public Health and Human Sciences, Oregon State University, Corvallis, OR, USA; One Acre Fund, Nairobi, Kenya; Center for Child Health Research, Faculty of Medicine and Health Technology, Tampere University, Tampere, Finland; Department of Nutrition and Food Science, University of Ghana, Legon, Accra, Ghana; Independent consultant, Dakar, Senegal; Poverty, Health, and Nutrition Division, International Food Policy Research Institute, Washington, DC, USA; School of Public Health, University of California, Berkeley, Berkeley, CA, USA; Department of Public Health, School of Public Health and Family Medicine, College of Medicine, University of Malawi, Blantyre, Malawi; Department of Nutritional Sciences and Toxicology, University of California, Berkeley, Berkeley, CA, USA; Zvitambo Institute for Maternal and Child Health Research, Harare, Zimbabwe; Global Alliance for Improved Nutrition, Washington, DC, USA; Center for Non-communicable Diseases and Nutrition, BRAC James P Grant School of Public Health, Dhaka, Bangladesh; Zvitambo Institute for Maternal and Child Health Research, Harare, Zimbabwe; International Center for Diarrheal Diseases Research (icddr,b), Dhaka, Bangladesh; Gangarosa Department of Environmental Health, Rollins School of Public Health, Emory University, Atlanta, GA, USA; Center for Non-communicable Diseases and Nutrition, BRAC James P Grant School of Public Health, Dhaka, Bangladesh; Department of Population Medicine and Diagnostic Sciences, Cornell University, Ithaca, NY, USA; Health Sciences Research Institute (IRSS), Bobo-Dioulasso, Burkina Faso; School of Engineering, Tufts University, Medford, MA, USA; International Center for Diarrheal Diseases Research (icddr,b), Dhaka, Bangladesh; Program in Human Nutrition, Department of International Health, Johns Hopkins Bloomberg School of Public Health, Baltimore, MD, USA; Department of Epidemiology and Environmental Health, School of Public Health and Health Professions, The State University of New York at Buffalo, Buffalo, NY, USA; Division of Epidemiology, School of Community Health Sciences, University of Nevada, Reno, Reno, NV, USA; Independent consultant, Washington, DC, USA; Institute for Global Nutrition and Department of Nutrition, University of California, Davis, Davis, CA, USA

**Keywords:** anemia, iron deficiency, micronutrient status, child undernutrition, complementary feeding, nutrient supplements, home fortification

## Abstract

**Background:**

Small-quantity lipid-based nutrient supplements (SQ-LNSs) have been shown to reduce the prevalence of child anemia and iron deficiency, but effects on other micronutrients are less well known. Identifying subgroups who benefit most from SQ-LNSs could support improved program design.

**Objectives:**

We aimed to identify study-level and individual-level modifiers of the effect of SQ-LNSs on child hemoglobin (Hb), anemia, and inflammation-adjusted micronutrient status outcomes.

**Methods:**

We conducted a 2-stage meta-analysis of individual participant data from 13 randomized controlled trials of SQ-LNSs provided to children 6–24 mo of age (*n* = 15,946). We generated study-specific and subgroup estimates of SQ-LNSs compared with control, and pooled the estimates using fixed-effects models. We used random-effects meta-regression to examine potential study-level effect modifiers.

**Results:**

SQ-LNS provision decreased the prevalence of anemia (Hb < 110 g/L) by 16% (relative reduction), iron deficiency (plasma ferritin < 12 µg/L) by 56%, and iron deficiency anemia (IDA; Hb < 110 g/L and plasma ferritin <12 µg/L) by 64%. We observed positive effects of SQ-LNSs on hematological and iron status outcomes within all subgroups of the study- and individual-level effect modifiers, but effects were larger in certain subgroups. For example, effects of SQ-LNSs on anemia and iron status were greater in trials that provided SQ-LNSs for >12 mo and provided 9 (as opposed to <9) mg Fe/d, and among later-born (than among first-born) children. There was no effect of SQ-LNSs on plasma zinc or retinol, but there was a 7% increase in plasma retinol-binding protein (RBP) and a 56% reduction in vitamin A deficiency (RBP < 0.70 µmol/L), with little evidence of effect modification by individual-level characteristics.

**Conclusions:**

SQ-LNSs can substantially reduce the prevalence of anemia, iron deficiency, and IDA among children across a range of individual, population, and study design characteristics. Policy-makers and program planners should consider SQ-LNSs within intervention packages to prevent anemia and iron deficiency.

This trial was registered at www.crd.york.ac.uk/PROSPERO as CRD42020156663.

## Introduction

Micronutrient deficiencies are estimated to affect 2 billion people globally (1, 2) and roughly one-third of the world's population is anemic (3). Infants and young children in low- and middle-income countries are particularly vulnerable to micronutrient undernutrition, owing in part to low micronutrient stores at birth, inadequate dietary intake of bioavailable micronutrients, and increased micronutrient requirements due to infection or malabsorption (4, 5). Deficiencies of iron, zinc, and vitamin A in these populations are associated with increased morbidity and mortality and delayed psychomotor and neurocognitive development (1).

Nutritional strategies to prevent micronutrient deficiencies include dietary diversification and modification, provision of supplements, fortification (i.e., large-scale food fortification, biofortification, and targeted in-home fortification), and supplemental feeding [e.g., fortified blended foods and lipid-based nutrient supplements (LNSs)]. Small-quantity (SQ)-LNSs have been designed to prevent malnutrition and provide multiple micronutrients embedded in a food base that also provides energy (100–120 kcal/d), protein, and essential fatty acids. This combination of macro- and micro-nutrients in SQ-LNSs has the potential to address multiple nutritional deficiencies simultaneously, thus reducing undernutrition (6).

A 2019 Cochrane systematic review and meta-analysis showed that SQ-LNSs and medium-quantity (MQ)-LNSs (∼125–250 kcal/d) given during the period of complementary feeding (6–24 mo) significantly reduced the prevalence of anemia by 21% (RR: 0.79; 95% CI: 0.69, 0.90; 5 studies; anemia as defined by trialists) compared with no intervention (7). Similarly, a 2020 meta-analysis demonstrated a 16% reduction in the prevalence of anemia (RR: 0.84; 95% CI: 0.75, 0.93; 8 studies; anemia as defined by trialists) among children who received SQ- or MQ-LNSs in comparison with a control group (8). To date, no meta-analysis has assessed the impact of SQ-LNSs provided during the period of complementary feeding on the micronutrient status of infants and young children. However, individual studies have demonstrated significant reductions in the prevalence of iron deficiency and iron deficiency anemia (IDA) (9–16), although there is heterogeneity in the magnitude of effects. Some, but not all, studies have shown significant improvements in vitamin A, vitamin B-12, and folate status (11–13, 15, 17); no studies have reported an effect of SQ-LNSs on zinc status (9, 11, 15).

Differences in study design and context and characteristics of study participants may modify the effect of SQ-LNSs on anemia and biomarkers of micronutrient status. The identification of subgroups of infants and young children who experience greater benefits from SQ-LNSs, or who are more likely to respond to the intervention, is useful in informing the development of public health policies and programs (18). Thus, we conducted an individual participant data (IPD) meta-analysis of randomized controlled trials (RCTs) of SQ-LNSs provided to infants and young children 6–24 mo of age. The objectives of this analysis were to *1*) generate pooled estimates of the effects of SQ-LNSs provided to infants and young children 6–24 mo of age on hemoglobin (Hb) concentration and selected biomarkers of micronutrient status, and *2*) identify study- and individual-level modifiers of the effect of SQ-LNSs on these outcomes in the same populations. Two companion articles report results for other outcome domains, growth (19) and development (20), from the same IPD meta-analysis.

## Methods

Methods broadly followed those presented in a companion article (19). This systematic review and IPD meta-analysis was preregistered through PROSPERO (CRD42020156663) (21). A detailed protocol was posted to Open Science Framework before analysis and updated after consultations with co-investigators before finalizing the analysis plan (22), and we have reported results according to Preferred Reporting Items for Systematic Reviews and Meta-Analyses (PRISMA)-IPD guidelines (23). The analyses were approved by the institutional review board of the University of California, Davis (1463609-1). All individual trial protocols were approved by their relevant institutional ethics committees.

### Inclusion and exclusion criteria for this systematic review and IPD meta-analysis

Trials were eligible for inclusion if they were individual or cluster RCTs with either longitudinal follow-up or repeated cross-sectional data collection, were conducted in low- or middle-income countries (24), provided SQ-LNSs (< ∼125 kcal/d) to study participants for ≥3 mo during any part of the age range between 6 and 24 mo of age, and reported ≥1 outcome of interest.

We excluded trials in which SQ-LNS was used for the treatment, not prevention, of malnutrition (i.e., only children with moderate-to-severe malnutrition were eligible to participate), as well as studies specifically conducted in hospitalized populations or among children with a pre-existing disease. Trials were excluded if the only available comparison group received other types of non-LNS child supplementation [e.g., multiple micronutrient powder (MNP), fortified blended food], or if SQ-LNS provision was combined with an additional supplemental food or additional nutrients for the child within a single arm (e.g., SQ-LNS + food rations compared with control) and there was no appropriate comparison group (e.g., food rations alone) that would allow separation of the SQ-LNS effect from the effects of the other food or nutrients provided.

Trials in which there were multiple relevant SQ-LNS interventions (e.g., varying dosages or formulations of SQ-LNS in different arms), which combined provision of child SQ-LNS with provision of maternal LNS, or which included other nonnutritional interventions [e.g., water, sanitation, and hygiene (WASH)] were eligible for inclusion. In such trials, all arms that provided child SQ-LNS were combined into 1 group, and all non-LNS arms (i.e., no LNS for mother or child) were combined into a single comparison group for each trial (herein labeled “Control”), excluding intervention arms that received non-LNS child supplementation (e.g., MNP, fortified blended food). We also conducted a sensitivity analysis restricting the comparison to specified contrasts of intervention arms within multiple intervention trials (see below).

Individual children were included in the analyses if their age at baseline allowed them to receive ≥3 mo of intervention (supplementation or control group components) between 6 and 24 mo of age, and if biochemical outcome assessment occurred within 3 mo of the intended, trial-defined, end of supplementation.

### Search methods and identification of studies

We identified potential studies for inclusion in the IPD analysis from a recent systematic review and meta-analysis of LNSs (7). We then repeated the database search strategy used by Das et al. (7), using the same keyword and controlled vocabulary search terms, to capture additional studies (both completed and ongoing) indexed in 1 of 23 international or regional electronic databases and 2 trial registers between 1 July, 2018 and 1 May, 2019 (see **Supplemental Methods**). One of the authors (KRW) reviewed the titles and abstracts of all studies included in the 2019 Cochrane review, as well as the additional studies identified by the repeated search strategy, to select all potentially relevant studies for full-text review. The full-text reports of all potentially relevant records were reviewed. Trials were assessed against the aforementioned inclusion and exclusion criteria. In September 2019, the same author reviewed the previously identified ongoing studies that met the inclusion and exclusion criteria to determine if results for outcomes of interest had been subsequently published.

### Data collection and harmonization

We invited all principal investigators of eligible studies to participate in this IPD meta-analysis. Individual investigators were asked to provide de-identified IPD (primary data) for prespecified variables (defined above and detailed in a data dictionary provided to investigators). The IPD analyst (CDA) collected and managed the IPD, and communicated with investigators to request any missing variables or other information.

### IPD integrity

We conducted a complete-case, intention-to-treat analysis (25). We checked data for completeness by crosschecking sample sizes with study protocols and publications. We also crosschecked the data provided against reported values for each trial to ensure consistency. Variables were assessed for outliers and low-frequency categories. Relevant model assumptions were assessed (e.g., Shapiro–Wilk normality testing and Breusch–Pagan heteroscedasticity testing) and outcome variables were appropriately transformed before subsequent analyses, as necessary.

### Assessment of risk of bias in each study and quality of evidence across studies

Two independent reviewers (KRW and CDA) assessed risk of bias using the criteria outlined in the Cochrane Handbook for Systematic Reviews of Interventions version 5.1.0 (26). We assessed each trial against the following criteria: random sequence generation and allocation concealment (selection bias), blinding of participants and personnel (performance bias), blinding of outcome assessment (detection bias), incomplete outcome data (attrition bias), selective reporting (reporting bias), and other sources of bias. Reviewers also assessed the quality of evidence for each primary and secondary outcome across all trials based on the 5 Grading of Recommendations Assessment, Development and Evaluation (GRADE) criteria: risk of bias, inconsistency of effect, imprecision, indirectness, and publication bias (27). Any discrepancies were resolved by discussion or consultation with the core working group, as needed.

### Specification of outcomes and effect measures

We specified outcomes a priori in the statistical analysis plan (22). **Box 1** lists outcomes of interest. For the estimation of the main effects, we prespecified Hb concentration, anemia, moderate-to-severe anemia, and biomarkers of iron status as primary outcomes in the statistical analysis plan, and present results for all outcomes. Hb concentrations were adjusted for altitude, as necessary (28). Ferritin, soluble transferrin receptor (sTfR), zinc protoporphyrin (ZPP), zinc, retinol, and retinol-binding protein (RBP) concentrations were adjusted for inflammation [i.e., C-reactive protein (CRP) and/or α-1-acid glycoprotein (AGP) concentrations, as available], using a regression correction approach adapted from the Biomarkers Reflecting Inflammation and Nutritional Determinants of Anemia (BRINDA) project and described in detail elsewhere (29); results are presented for inflammation-adjusted concentrations.

Box 1. Specification and definitions of outcome variables^1^

**Table:** 

Outcome variables	Definitions of dichotomous outcome variables
Hb, g/L	
Anemia	Hb <110 g/L (86)
Moderate-to-severe anemia	Hb <100 g/L (86)
Plasma ferritin, µg/L	
Iron deficiency (low plasma ferritin concentration)	Plasma ferritin <12 µg/L (87)
Iron deficiency anemia	Plasma ferritin <12 µg/L and Hb <110 g/L (28)
Plasma sTfR concentration, mg/L	
Elevated plasma sTfR concentration	Plasma sTfR >8.3 mg/L (88)
Erythrocyte ZPP concentration, µmol/mol heme	
Elevated ZPP	Erythrocyte ZPP >70 µmol/mol heme (89)
Plasma zinc concentration, µg/dL	
Low plasma zinc concentration	Plasma zinc <65 µg/dL (90)
Plasma retinol concentration, µmol/L	
Low retinol	Plasma retinol <0.70 µmol/L (60)
Marginal retinol	Plasma retinol <1.05 µmol/L (60)
Plasma RBP concentration, µmol/L	
Low RBP	Plasma RBP <0.70 µmol/L^2^ (61)
Marginal RBP	Plasma RBP <1.05 µmol/L^2^ (91)

1 Plasma hepcidin concentration (ng/mL), low hepcidin (<5.5 ng/mL) (92), plasma folate concentration (nmol/L), low folate (<10 nmol/L) (93), plasma vitamin B-12 concentration (pmol/L), low vitamin B-12 (<150 pmol/L), and depleted vitamin B-12 (<221 pmol/L) (94) were also considered as outcome variables; however, data were not yet available from a sufficient number of trials to be included in the present analyses. Hb, hemoglobin; RBP, retinol-binding protein; sTfR, soluble transferrin receptor; ZPP, zinc protoporphyrin.

2 There is currently no internationally established cutoff for RBP that reflects a serum retinol concentration of <0.70 µmol/L or <1.05 µmol/L, and it has been recommended that trials determine the relation between retinol and RBP in a subsample of the trial population to establish study-specific RBP cutoffs (61). This was not done in all of the trials included in these analyses, therefore we elected to use plasma RBP <0.7 µmol/L and <1.05 µmol/L to define low and marginal status, respectively (91).

The principal measure of effect for normally distributed continuous outcomes was the mean difference (MD) between the intervention and comparison groups at endline, defined as the principal postintervention time point as reported by each longitudinal trial or at the age closest to the end of the supplementation period for cross-sectional samples. For ln-transformed outcomes, the principal measure of effect was the ratio of geometric means (GMR) between the 2 groups at endline, expressed as a relative percentage increase or decrease in the SQ-LNS group compared with the control. For binary outcomes, the principal measure of effect was the prevalence ratio (PR; relative difference in proportions between groups) at endline. We also estimated prevalence differences (PDs; in absolute percentage points) because of their importance for estimating public health impact, but considered them as secondary assessments of binary outcomes because such estimates are less consistent than PRs (26).

The treatment comparison of interest was child SQ-LNS compared with control. In the intervention groups, child SQ-LNSs may have been provided along with other nutrition-sensitive co-interventions (e.g., WASH or child morbidity monitoring and treatment). The control groups consisted of passive or active comparison arms which provided no intervention or an intervention without any type of LNS or other child supplement (e.g., WASH). Several trials (or arms within trials) have delivered SQ-LNSs to children whose mothers received maternal SQ-LNSs during pregnancy and postpartum. We had originally planned to include trial arms that provided both maternal and child SQ-LNSs in a sensitivity analysis only (i.e., the all-trials analysis), because maternal supplementation may have an additive effect on child outcomes when SQ-LNS is provided both to mothers and to their children. However, to maximize study inclusion and participant sample size, and to allow for sufficient numbers of trials to examine effect modification for certain outcomes, we decided after initial registration of the protocol but before completing statistical analyses that if the main effects did not differ between the child-LNS-only analysis and the all-trials analysis (including maternal plus child LNS arms) by >20% for MDs or by >0.05 for GMRs or PRs, the results of the all-trials analyses would be presented as the principal findings. Two additional sensitivity analyses were also conducted, as described below.

### Synthesis methods and exploration of variation in effects

We separately investigated *1*) full sample main effects of the intervention, *2*) effect modification by study-level characteristics, and *3*) effect modification by individual-level characteristics. For all 3 sets of analyses, we used a 2-stage approach, which is preferred when the analysis includes cluster-randomized trials (30). In the first stage, we generated intervention effect estimates within each individual study. For longitudinal studies, we controlled for baseline status of the outcome variable, if available, to gain efficiency. For cluster-randomized trials, we used robust SEs with randomization clusters as the independent unit (31). In the second stage, we pooled the first-stage estimates using inverse-variance weighted fixed effects. A fixed-effect approach generates estimates viewed as a typical intervention effect from the studies included in the analysis. This was prespecified in our statistical analysis plan because we anticipated similar intervention effects and similar individual-level effect modification patterns across studies. As a robustness check of this assumption, we also conducted sensitivity analyses in which we pooled estimates using inverse-variance weighted random effects (32). Pooled estimates were only generated if ≥3 study or substudy SQ-LNS against control comparisons were available for inclusion in the pooled estimate (e.g., ≥3 comparisons were represented within a study-level effect modification category).

1. *Full sample main effects of the intervention:* We first estimated the intervention effect for each study. We then pooled the first-stage estimates to generate a pooled point estimate, 95% CI, and corresponding *P* value.
2. *Effect modification by study-level characteristics:* We identified potential study-level effect modifiers before receipt of data, and categorized individual studies based on the distribution of effect modifier values across all studies (**Box 2**). We used random-effects meta-regression to test the association between each effect modifier and the intervention. The random-effects approach is used when exploring heterogeneity across studies. In the first stage of analysis, we estimated the parameter corresponding to the intervention effect as aforementioned. In the second stage, we used a bivariate random-effects meta-regression to test the association between the study-specific intervention effect and study-level characteristics and also generated strata-level pooled estimates to aid interpretation.
3. *Effect modification by individual-level characteristics:* We identified potential individual-level characteristics based on a comprehensive review of effect modifiers considered by individual trials (either listed a priori in statistical analysis plans or as published) or selected based on biological plausibility (Box 2). We estimated the parameter corresponding to the interaction term of the effect modifier and the intervention for each study (31), as follows. For categorical effect modifiers, we first recoded them to create binary variables if needed, and then determined the interaction between the intervention and the binary effect modifier. We transformed continuous effect modifiers into binary variables by modeling the relation within each study using splines and then pooling the first-stage estimates to generate a pooled, fitted line. We defined programmatically useful dichotomous cutoffs based on the pooled fitted spline results and relevant context. We then generated pooled intervention effect estimates within each category to determine how the intervention effect in 1 subgroup differed from the intervention effect in the specified reference subgroup.

Box 2. Potential effect modifiers^1^

**Table:** 

Study-level effect modifiers	Individual-level effect modifiers
• Geographic region (WHO region: African vs. South-East Asia Region)• Anemia burden (country-specific, closest in time to the study: <60% vs. ≥60%)^2^• Malaria prevalence (country-specific, closest in time to the study: <10% vs. ≥10%)^3^• Inflammation prevalence (study-specific:, elevated CRP ≤25% and/or elevated AGP ≤50% vs. elevated CRP >25% and/or elevated AGP >50%)^4^• Water quality (study-specific: <75% vs. ≥75% prevalence of improved drinking water)^5^• Sanitation (study specific: <50% vs. ≥50% prevalence of improved sanitation)^6^• Duration of child supplementation (study target: >12 mo vs. ≤12 mo)• Iron dose in the SQ-LNS product (9 mg/d vs. <9 mg/d)^7^• Child age at baseline or endline• Frequency of contact for intervention delivery or outcome assessments during the study (weekly vs. monthly)• Compliance (average percentage compliance in LNS group ≥80% vs. <80%)^8^	• Maternal BMI (<20 kg/m^2^ vs. ≥20 kg/m^2^)• Maternal age (<25 y vs. ≥25 y)• Maternal education (no formal or incomplete primary vs. complete primary or greater)• Child sex (female vs. male)• Child birth order (first born vs. later born)• Child baseline acute malnutrition (WLZ <−2 SD or MUAC <125 mm vs. WLZ ≥−2 SD and MUAC ≥125 mm; if MUAC not measured, WLZ <−2 SD vs. WLZ ≥−2 SD)• Child baseline anemia status (Hb ≥110 g/L vs. <110 g/L); iron outcomes only• Child high-dose vitamin A supplementation (receipt within 6 mo before outcome assessment vs. nonreceipt)• Child inflammation at time of outcome assessment (CRP ≤5 mg/L and AGP ≤1 g/L vs. not)• Household socioeconomic status (< study median vs. ≥ study median)^9^• Food security (moderate-to-severe food insecurity vs. mild food insecurity to secure)• Water quality (unimproved vs. improved)^6^• Sanitation (unimproved vs. improved)^7^• Season at the time of outcome assessment (rainy vs. dry)^10^

1 AGP, α-1-acid glycoprotein; CRP, C-reactive protein; Hb, hemoglobin; LNS, lipid-based nutrient supplement; MUAC, midupper arm circumference; SQ, small-quantity; WASH, water, sanitation, and hygiene; WLZ, weight-for-length *z* score.

2 Country-specific prevalence of anemia among children 6–59 mo old, based on national surveys (see Supplemental Table 3); cutoff chosen based on the median across trials.

3 Country-specific prevalence of malaria, based on the World Malaria Report 2018 (see Supplemental Table 3) (51); cutoff chosen based on the median across trials.

4 Elevated CRP defined as >5 mg/L, elevated AGP defined as >1 g/L. Based on data at outcome assessment because baseline data were not available for all trials; cutoff chosen based on the median across trials.

5 Improved water source includes piped water, boreholes or tubewells, protected dug wells or springs, rainwater, and packaged or delivered water (see Supplemental Table 3) (95); based on baseline data, excluding arms that received WASH interventions; cutoff chosen at approximately the median across trials.

6 Improved sanitation includes flush/pour flush to piped sewer system, septic tanks, or pit latrines; ventilated improved pit latrines, composting toilets, or pit latrines with slabs (see Supplemental Table 3) (96); based on baseline data, excluding arms that received WASH interventions; cutoff chosen based on the median across trials.

7 Study-specific (see Supplemental Tables 2 and 3).

8 Study-specific, because reported based on a study-defined indicator (see Supplemental Table 3); cutoff chosen based on the median across trials.

9 Based on a study-defined, study-specific assets index.

10 Rainy compared with dry, based on study- and child-specific average rainfall during the month of measurement and 2 mo prior (see Supplemental Methods and Supplemental Table 4).

In all analyses, heterogeneity was assessed using *I*^2^ and tau^2^ statistics, within strata when relevant (33). We used a *P* value <0.05 for main effects and a *P*-diff or *P*-interaction < 0.10 for effect modification by study-level or individual-level characteristics, respectively. Because biochemical outcomes are interrelated and the effect modification analyses are inherently exploratory, we did not adjust for multiple hypothesis testing because doing so may be unnecessary and counterproductive (34).

To aid in interpretation of effect modification, we evaluated the results for binary outcomes to identify what we will call the “cutoff effect.” The distribution of the continuous outcome relative to the cutoff for the corresponding binary outcome (e.g., distribution of Hb around the 110 g/L anemia cutoff) in the 2 effect modifier subgroups can influence the PR and PD. When the mean in each of the 2 subgroups falls in a different location relative to the cutoff, the proportion of children close to the cutoff may be different between subgroups. This can lead to a greater reduction in the adverse binary outcome within one subgroup than within the other even if the shift in the mean value due to SQ-LNS is the same in both subgroups. To examine this, we simulated what would happen if we shifted the distribution of the nonreference effect modification subgroup to align with the reference subgroup (see Box 2), while maintaining the observed intervention effect MD in the continuous outcome within each subgroup. Based on ad hoc pragmatic criteria, if the *P*-interaction shifted from <0.1 to >0.2, we concluded that the cutoff effect explained the apparent effect modification. In these situations, if there was no significant effect modification for the continuous outcome, we interpreted the results as evidence that both subgroups benefited and/or responded similarly. If the *P*-interaction shifted from <0.1 to >0.1 but <0.2, and there was no significant effect modification for the continuous outcome, we concluded that the cutoff effect partially contributed to the apparent effect modification.

### Additional sensitivity analyses

For the child SQ-LNS only and all-trials analyses, we combined all non-LNS arms into a “Control” group for that trial. However, substantial variations in trial design (e.g., integration of SQ-LNS supplementation with WASH interventions or enhanced morbidity monitoring and treatment, use of passive compared with active control arms) might influence the effect size of the estimates. Therefore, we conducted sensitivity analyses based on child-LNS-only analyses in which we *1*) separated the comparisons within trials that contained multicomponent arms, thus restricting the comparisons to pairs of arms with the same nonnutrition components (e.g., SQ-LNS + WASH compared with WASH and SQ-LNS compared with control), or *2*) excluded passive control arms, i.e., restricting the comparison to SQ-LNS against active control arms (**Supplemental Table 1**). These analyses are considered exploratory.

In addition, we conducted post hoc analyses to examine effects within subgroups of trials based on 2 aspects of the intervention design: *1*) whether the trial was or was not conducted within an existing program, and *2*) the extent of the social and behavior change communication (SBCC) on infant and young child feeding (IYCF) that was provided (minimal compared with expanded in all trial arms compared with expanded in SQ-LNS intervention arms only).

## Results

### Literature search and trial characteristics

**FIGURE 1:**
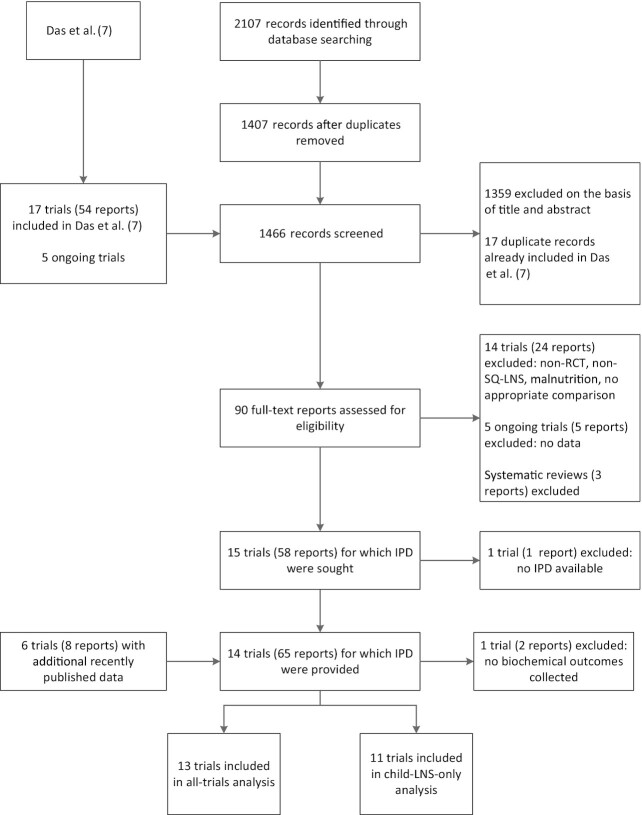
Study flow diagram. IPD, individual participant data; LNS, lipid-based nutrient supplement; RCT, randomized controlled trial; SQ, small-quantity.

We identified 15 trials that met our inclusion criteria for the child SQ-LNS only and/or all-trials analyses, 13 of which (35–48) provided individual participant biochemical data and are included in this analysis (9–13, 15–17, 39, 46–48) (**Figure 1**). In 1 study biochemical data were not collected (49), and investigators for 1 trial were unable to participate (14). In that trial Hb concentrations and the prevalence of anemia were reported; therefore, we examined pooled main effects on those 2 outcomes both without and with that trial, by calculating Hedges’ *g* (50) based on endline values extracted from the published report. **Table 1** provides an overview of each trial included in the IPD analysis; Supplemental Table 1 provides additional details. One trial was designed a priori to present results separately for HIV-exposed and HIV-unexposed children (47, 48); therefore we considered it herein as 2 comparisons in all analyses and presentation of results. The PROMIS trials in Burkina Faso and Mali each included a longitudinal cohort and repeated cross-sectional surveys (at baseline and endline); however, biochemical samples were collected only in the cross-sectional surveys (39, 46).

**TABLE 1:** Characteristics of trials included in the IPD analysis^1^

	Child SQ-LNS supplementation			Participants with data available,^2^*n*			Prevalence of deficiency in the control group at endline, %	
Country, years of study, study name, trial design, references^3^	Age at start	Duration	Maternal SQ-LNS supplement	Endline assessment	Hb subsample	MN subsample	Anemia (Hb < 110 g/L)	Iron deficiency (ferritin < 12 µg/L)
Bangladesh, 2012–2014, JiVitA-4, cluster RCT, longitudinal follow-up, Christian et al. (35) and Campbell et al. (15)	6 mo	12 mo	N	4568	603	600	15.8	22.2
Bangladesh, 2011–2015, RDNS, cluster RCT, longitudinal follow-up, Dewey et al. (36) and Matias et al. (10)	6 mo	18 mo	Y/N	2567	821	822	41.9	22.1
Bangladesh, 2012–2015, WASH-Benefits, cluster RCT, cross-sectional surveys, Luby et al. (37) and Stewart et al. (12)	6 mo	18 mo	N	4824	420	390	16.1	35.4
Burkina Faso, 2010–2012, iLiNS-ZINC, cluster RCT, longitudinal follow-up, Hess et al. (38) and Abbeddou et al. (11)	9 mo	9 mo	N	2647	2621	456	91.1	58.5
Burkina Faso, 2015–2017, PROMIS, cluster RCT, cross-sectional surveys, Becquey et al. (39)	6 mo	12 mo	N	1157	1155	0	70.1	—
Ghana, 2004–2005, RCT, longitudinal follow-up, Adu-Afarwuah et al. (40, 9)	6 mo	6 mo	N	194	194	167	58.3	56.1
Ghana, 2009–2014, iLiNS-DYAD-G, RCT, longitudinal follow-up, Adu-Afarwuah et al. (41, 16)	6 mo	12 mo	Y	1113	989	304	44.9	—
Kenya, 2012–2016, WASH-Benefits, cluster RCT, cross-sectional surveys, Null et al. (42) and Stewart et al. (12)	6 mo	18 mo	N	6815	650	631	47.3	59.1
Madagascar, 2014–2016, MAHAY, cluster RCT, longitudinal follow-up, Galasso et al. (43) and Stewart et al. (13)	6–11 mo	6–12 mo	Y/N	3438	1188	134	64.8	51.0
Malawi, 2011–2014, iLiNS-DYAD-M, RCT, longitudinal follow-up, Ashorn et al. (44)	6 mo	12 mo	Y	675	642	582	51.9	—
Malawi, 2009–2012, iLiNS-DOSE, RCT, longitudinal follow-up, Maleta et al. (45)^4^	6 mo	12 mo	N	1018	325	268	72.0	—
Mali, 2015–2017, PROMIS, cluster RCT, cross-sectional surveys, Huybregts et al. (46)	6 mo	18 mo	N	1927	1923	0	86.2	—
Zimbabwe, 2013–2017, SHINE,^5^ cluster RCT, longitudinal follow-up, Humphrey et al. (47) and Prendergast (48)	6 mo	12 mo	N	4347	3867	0	35.3^6^	—

1 Hb, hemoglobin; IPD, individual participant data; MN, micronutrient; RCT, randomized controlled trial; SQ-LNS, small-quantity lipid-based nutrient supplement.

2 Endline assessment (*n*) includes the number of children for whom any data were available for ≥1 outcome included in the IPD analyses (growth, development, and/or biochemical). MN subsample includes the number of children for whom any data were available for ≥1 MN status outcome, plus C-reactive protein and/or α-1-acid glycoprotein such that values could be adjusted for inflammation. A total of 263 children in the MN subsample did not have data available for Hb. Data on the baseline status of outcome variables were available for 5 of 13 trials (36, 38, 41, 44, 45).

3 The first citations refer to the main publication for each trial. The second citations refer to an additional publication specific to hematological and micronutrient status outcomes.

4 Trial is cited as Kumwenda 2014 in Das et al. (7).

5 Trial was designed a priori to present results separately for HIV-exposed and -unexposed children; thus considered as 2 comparisons in all analyses and the presentation of results.

6 Data are for the HIV-unexposed cohort only. The prevalence of anemia in the HIV-exposed cohort was 36.8%.

The 13 trials in these analyses were conducted in 9 countries in Sub-Saharan Africa and South Asia, and included a total of 15,946 infants and young children with biochemical data. Eleven trials began child supplementation with SQ-LNSs at 6 mo of age; for the remaining 2 trials, SQ-LNS supplementation began when infants were 9 mo of age in one (38) and between 6 and 11 mo of age in the other (43). Supplementation occurred for 6–18 mo in duration, depending on the trial. Four trials included intervention arms that also provided SQ-LNSs to mothers during pregnancy and the first 6 mo postpartum (36, 41, 43, 44). The majority of trials provided a peanut- and milk-based SQ-LNS providing ∼120 kcal/d and 1 RDA of most micronutrients (**Supplemental Table 2**). However, the iron dose per day varied among trials. Four trials provided 9 mg Fe/d (36, 37, 40, 42), 8 trials provided 6 mg Fe/d (38, 39, 41, 43–48), and 1 trial provided 3.3–9 mg Fe/d depending on study arm and child age (35). Trials varied with regard to whether they were conducted within an existing program or not, the extent of SBCC on IYCF, and other aspects of study design (Supplemental Table 1).

Categorizations and descriptive information for potential study- and individual-level effect modifiers, by trial, are presented in **Supplemental Tables 3** and **4**, respectively. Characteristics ranged widely across trials. Based on data from national surveys, the prevalence of anemia among children 6–59 mo old ranged from 36% (Kenya) to 88% (Burkina Faso), and the prevalence of malaria (presumed and confirmed cases) ranged from <1% (Bangladesh) to 59% (Burkina Faso) (51). Based on data from individual trials, the study-level prevalence of elevated CRP ranged from 11% (WASH-Benefits, Bangladesh) to 34% (iLiNS-DOSE, Malawi) and the prevalence of elevated AGP ranged from 29% (WASH-Benefits, Bangladesh) to 68% (iLiNS-DYAD, Malawi).

All trials measured Hb concentration (*n* = 15,398), and 10 reported ≥1 biomarker of iron status (*n* = 1542–3078) (Supplemental Tables 1, **5**). Fewer studies assessed zinc (3 studies; *n* = 1133) or vitamin A (9 studies; *n* = 1236–2314) status. Assessments of hepcidin, folate, and vitamin B-12 concentrations were planned in 3–4 studies each, but data were not yet available from a sufficient number of trials to be included in the present analysis. Median biomarker concentrations and prevalence of dichotomous study outcomes among control groups by trial are presented in Table 1 (anemia and iron deficiency) and **Supplemental Table 6** (all outcomes). In control groups at endline, the prevalence of anemia ranged from 16% (JiVitA-4, Bangladesh) to 91% (iLiNS-Zinc, Burkina Faso), the prevalence of iron deficiency ranged from 22% (RDNS, Bangladesh) to 59% (WASH-Benefits, Kenya), and the prevalence of IDA ranged from 9% (JiVitA-4, Bangladesh) to 55% (iLiNS-Zinc, Burkina Faso).

In general, trials were considered to have a low risk of bias (19), with the exception of lack of masking of participants owing to the nature of the intervention (**Supplemental Table 7**, **Supplemental Figure 1**).

### Main effects of SQ-LNSs on anemia and micronutrient status

Results from the child-LNS-only and all-trials analyses, inclusive of maternal + child SQ-LNS trials/arms, were similar; for nearly all outcomes, the MDs, PRs, and PDs for intervention compared with control groups were almost identical or slightly less favorable when the maternal LNS trials/arms were included (**Supplemental Figure 2**A–D). Therefore, results from the all-trials analyses are presented below and in **Table 2**. Sample sizes for the all-trials analyses were considerably larger than for the child-LNS-only analyses: for example, the total pooled sample sizes for Hb concentration were 15,398 and 13,190, respectively. Forest plots for all main effects by outcome and individual study are presented below for selected outcomes and as supplemental materials for all other outcomes (**Supplemental Figure 3**A–AA).

**TABLE 2:** The effect of small-quantity LNSs on Hb and micronutrient status^1^

Outcomes	Participants (comparisons), *n*	MD/GMR/PR (95% CI)	*P* value^2^	Heterogeneity *I*^2^ (*P*-heterogeneity)^2^	Quality of the evidence (GRADE)
Hb,^3^ g/L	15,398 (14)	2.77 (2.30, 3.25)^4^^,^^5^	<0.001	0.72 (<0.001)	High
Ferritin,^3^^,^^6^ µg/L	3078 (7)	1.56 (1.48, 1.64)^7^	<0.001	0.53 (0.072)	High
sTfR,^3^^,^^6^ mg/L	2480 (6)	0.83 (0.80, 0.85)^7^	<0.001	0.46 (0.098)	High
ZPP,^3,6^ µmol/mol heme	1542 (4)	0.80 (0.75, 0.85)^7^	<0.001	0.79 (0.001)	Moderate
Plasma zinc,^6^ µg/dL	1133 (3)	1.00 (0.97, 1.02)^7^	0.734	0.00 (0.416)	Low
Retinol,^6^ µmol/L	1236 (4)	1.04 (1.00, 1.08)^7^	0.057	0.08 (0.360)	Moderate
RBP,^6^ µmol/L	2314 (5)	1.07 (1.04, 1.09)^7^	<0.001	0.68 (0.017)	Moderate
Anemia (Hb <110 g/L)^3^	15,398 (14)	0.84 (0.81, 0.87)^5^^,^^8^	<0.001	0.65 (<0.001)	High
Moderate-to-severe anemia (Hb <100 g/L)^3^	14,375 (12)	0.72 (0.68, 0.76)^8^	<0.001	0.64 (0.001)	High
Iron deficiency (ferritin <12 µg/L)^3^	3078 (7)	0.44 (0.39, 0.50)^8^	<0.001	0.66 (0.009)	High
Iron deficiency anemia (Hb <110 g/L, ferritin <12 µg/L)^3^	2702 (6)	0.36 (0.30, 0.44)^8^	<0.001	0.71 (0.004)	High
Elevated sTfR (>8.3 mg/L)^3^	2480 (6)	0.64 (0.59, 0.70)^8^	<0.001	0.73 (<0.001)	High
Elevated ZPP (>70 µmol/mol heme)^3^	1542 (4)	0.70 (0.61, 0.79)^8^	<0.001	0.00 (0.881)	Moderate
Zinc deficiency (plasma zinc <65 µg/dL)	537 (2)	—	—	—	—
Low vitamin A status (retinol <0.70 µmol/L)	663 (3)	1.02 (0.62, 1.69)^8^	0.938	0.01 (0.581)	Low
Marginal vitamin A status (retinol <1.05 µmol/L)	1236 (4)	0.98 (0.85, 1.13)^8^	0.829	0.35 (0.206)	Moderate
Low vitamin A status (RBP <0.70 µmol/L)	1790 (3)	0.44 (0.27, 0.70)^8^	0.001	0.00 (0.713)	Moderate
Marginal vitamin A status (RBP <1.05 µmol/L)	2314 (5)	0.78 (0.70, 0.87)^8^	<0.001	0.00 (0.765)	Moderate

1 GMR, ratio of geometric means; GRADE, Grading of Recommendations Assessment, Development and Evaluation; Hb, hemoglobin; LNS, lipid-based nutrient supplement; MD, mean difference; PR, prevalence ratio; RBP, retinol-binding protein; sTfR, soluble transferrin receptor; ZPP, zinc protoporphyrin.

2 The *P* value column corresponds to the pooled main effect 2-sided superiority testing of the intervention effect estimate and 95% CI presented in the preceding column. *I*^2^ describes the percentage of variability in effect estimates that may be due to heterogeneity rather than chance. Roughly, 0.3–0.6 may be considered moderate heterogeneity, <0.6 may be considered high heterogeneity. *P* value from chi-square test for heterogeneity. *P* < 0.05 indicates statistically significant evidence of heterogeneity of intervention effects beyond chance.

3 Prespecified as primary outcomes in the statistical analysis plan (22).

4 MD: LNS − control (95% CI).

5 The MD for Hb was 2.77 g/L (95% CI: 2.31, 3.23 g/L), and the PR for anemia was 0.83 (0.80, 0.85) when results from the 1 trial that did not participate in the IPD analyses were included (14).

6 Ferritin, sTfR, ZPP, zinc, retinol, and RBP concentrations were adjusted for inflammation (i.e., C-reactive protein and/or α-1-acid glycoprotein concentrations, as available), using a regression correction approach adapted from the Biomarkers Reflecting Inflammation and Nutritional Determinants of Anemia (BRINDA) project (29).

7 GMR: LNS/control (95% CI).

8 PR: LNS/control (95% CI).

**FIGURE 2:**
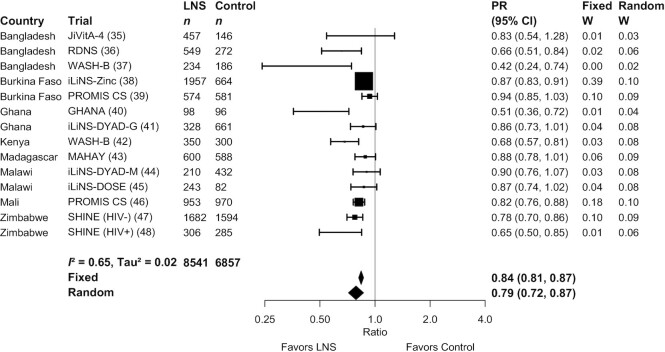
Forest plot of the effect of small-quantity LNSs on anemia prevalence. Individual study estimates were generated from log-binomial regression controlling for baseline measure when available and with clustered observations using robust SEs for cluster-randomized trials. Pooled estimates were generated using inverse-variance weighting in both fixed- and random-effects models. LNS, lipid-based nutrient supplement; PR, prevalence ratio.

**FIGURE 3:**
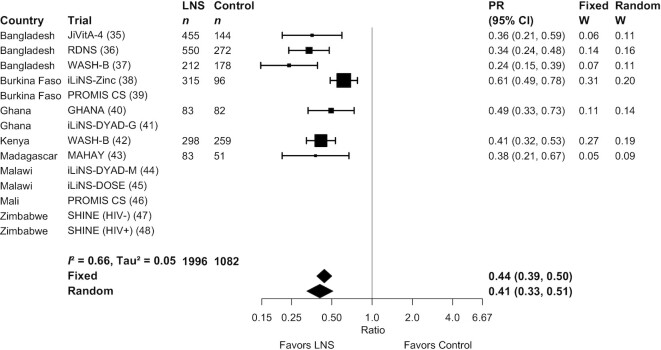
Forest plot of the effect of small-quantity LNSs on prevalence of iron deficiency (ferritin <12 µg/L). Ferritin concentrations were adjusted for inflammation (i.e., C-reactive protein and/or α-1-acid glycoprotein concentrations, as available), using a regression correction approach adapted from the Biomarkers Reflecting Inflammation and Nutritional Determinants of Anemia (BRINDA) project (29). Individual study estimates were generated from log-binomial regression controlling for baseline measure when available and with clustered observations using robust SEs for cluster-randomized trials. Pooled estimates were generated using inverse-variance weighting in both fixed- and random-effects models. LNS, lipid-based nutrient supplement; PR, prevalence ratio.

SQ-LNSs had a significant positive effect on all biomarkers of hematological and iron status (Table 2, **Figures 2**, **3**). Compared with control children, Hb concentrations were 2.77 g/L higher among children who received SQ-LNSs; in addition, ferritin concentrations were 56% higher, and sTfR and ZPP concentrations were 17% lower, among children who received SQ-LNSs as opposed to control. SQ-LNSs reduced the prevalence of anemia by 16% (10 percentage points), iron deficiency by 56% (22 percentage points), and IDA by 64% (14 percentage points). Differences in plasma zinc and retinol concentrations between those who received SQ-LNSs and those who did not were not significant. RBP concentrations were 7% higher among children who received SQ-LNSs than among the control group. SQ-LNSs reduced the prevalences of low and marginal vitamin A status, as measured by RBP, by 56% (3 percentage points) and 22% (8 percentage points), respectively; there was no effect of SQ-LNSs on vitamin A status as measured by retinol. We rated the quality of evidence for Hb, ferritin, and sTfR concentrations, as well as anemia, moderate-to-severe anemia, iron deficiency, elevated sTfR, and IDA, as high, based on the GRADE criteria listed in the Methods: ≥6 RCTs were available for these outcomes and the total sample size was roughly >2500, the risk of bias was low, all trials were directly aimed at evaluating SQ-LNSs, funnel plots revealed no indication of publication bias, and the direction of the effect was consistent even though the magnitude of the effect across trials differed (i.e., moderate to high heterogeneity). For all other outcomes, we rated the quality of the evidence as low to moderate, primarily because of limited availability of data, i.e., fewer trials and participants.

MDs, GMRs, and PRs for all outcomes were either identical or greater in random- compared with fixed-effects models (Supplemental Figure 3A–AA). The point estimates of the main effects were similar in the sensitivity analyses in which multicomponent arms (e.g., WASH + SQ-LNS) were compared with reference groups that had the same components without SQ-LNS and in which passive control trials were excluded (Supplemental Figure 2A–D). For example, the PRs for anemia ranged from 0.82–0.84 and those for iron deficiency were 0.36–0.44. There were no differences in statistical significance between models in which outcomes were adjusted compared with not adjusted for inflammation (data not shown). In addition, effects of SQ-LNSs on anemia were evident in both the program-based trials and the trials in which all activities were conducted by the research teams (**Supplemental Figure 4**), and also when trials were stratified by the extent of SBCC for IYCF (**Supplemental Figure 5**).

### Effect modification by study-level characteristics

**FIGURE 4:**
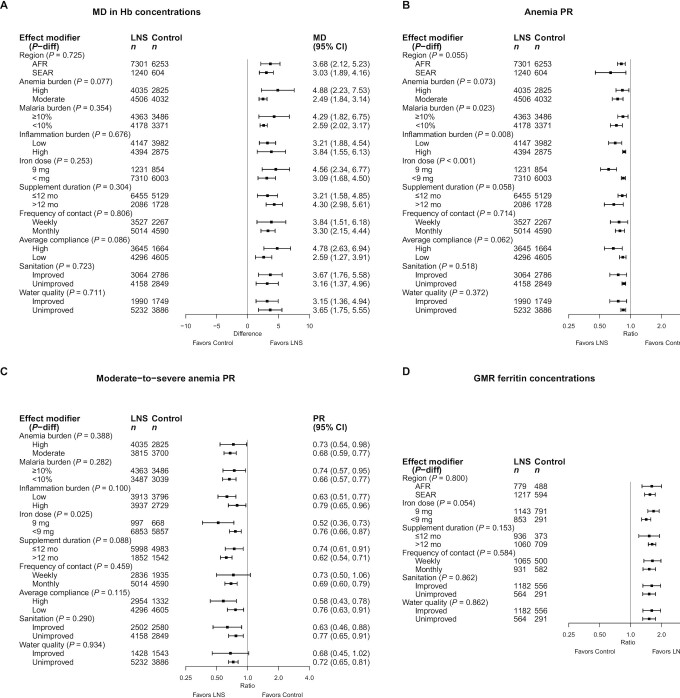

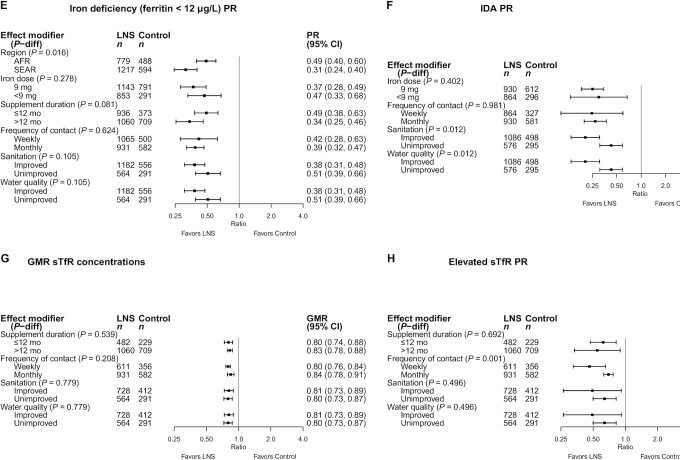
The effect of small-quantity LNSs provided to children 6–24 mo of age compared with a control on Hb concentration (A), anemia prevalence (B), moderate-to-severe anemia prevalence (C), ferritin concentration (D), iron deficiency (E), IDA (F), sTfR concentration (G), and elevated sTfR (H), stratified by study-level characteristics. Ferritin and sTfR concentrations were adjusted for inflammation (i.e., C-reactive protein and/or α-1-acid glycoprotein concentrations, as available), using a regression correction approach adapted from the Biomarkers Reflecting Inflammation and Nutritional Determinants of Anemia (BRINDA) project (29). *P*-diff was estimated using random-effects meta-regression with the indicated effect modifier as the predictor of intervention effect size; stratified pooled estimates are presented for each stratum. Owing to the limited number of studies, we were unable to examine study-level effect modification on all outcomes for all potential modifiers of interest. AFR, African Region; GMR, ratio of geometric means; Hb, hemoglobin; IDA, iron deficiency anemia; LNS, lipid-based nutrient supplement; MD, mean difference; *P*-diff, *P* value for the difference in the effect of small-quantity lipid-based nutrient supplement between the 2 levels of the effect modifier; PR, prevalence ratio; SEAR, South-East Asia Region; sTfR, soluble transferrin receptor.

**FIGURE 5:**
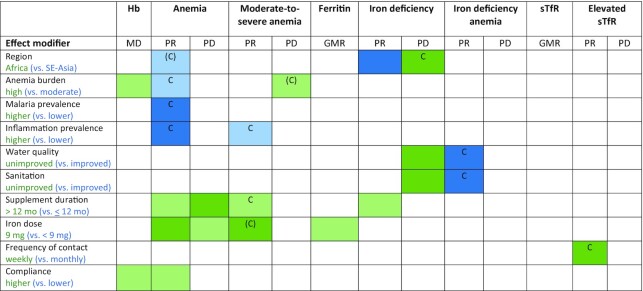
Overview of study-level effect modification. The reference subgroup is the group expected to have the greatest potential to benefit. Green indicates a stronger effect in the reference subgroup; blue indicates a stronger effect in the opposite subgroup. Box 2 provides subgroup definitions. Dark color indicates *P*-interaction <0.05; light color indicates 0.05< *P* <0.1. The letter “C” indicates that the apparent effect modification is due to the cutoff effect; when “C” is in parentheses, it is partially explained by the cutoff effect. For iron status biomarkers (ferritin and sTfR), water quality and sanitation subgroups captured the same trials, so these characteristics are considered as a single effect modifier at the study level. Water quality and sanitation subgroups captured different trials for Hb and anemia outcomes. GMR, ratio of geometric means; Hb, hemoglobin; MD, mean difference; PD, prevalence difference; PR, prevalence ratio; sTfR, soluble transferrin receptor.

**Figure 4**A–H presents the effects of SQ-LNSs on Hb, anemia, and biomarkers of iron status, stratified by study-level characteristics, and **Figure 5** presents an overview of these results. **Supplemental Figure 6**A–AA presents forest plots for all outcomes stratified by study-level effect modifiers. We were unable to generate pooled estimates for effect modification by any study-level characteristics for ZPP and biomarkers of zinc and vitamin A status (i.e., retinol and RBP) owing to the limited number of studies that measured these biomarkers. For iron status biomarkers (ferritin and sTfR), water quality and sanitation subgroups captured the same trials, so these characteristics are considered as a single effect modifier at the study level. Effect modification results were generally consistent across all sensitivity analyses (**Supplemental Figure 7**A–AA) and between models in which outcomes were inflammation-adjusted or not (data not shown); the results presented below refer to the all-trials analyses of inflammation-adjusted outcomes.

In all analyses of effect modification by study-level characteristics, the 95% CI of the mean Hb difference, and the GMRs for ferritin and sTfR concentrations, excluded the null for both strata, indicating that there were significant improvements in Hb, ferritin, and sTfR concentrations among children receiving SQ-LNSs regardless of study-level characteristics. Similarly, the upper bound of the 95% CI was <1 for the PR and <0 for the PD for both categories in all comparisons, indicating that there were significant reductions in the prevalence of anemia, iron deficiency, and IDA among children receiving SQ-LNSs regardless of study-level characteristics. However, there were some significant interactions, which are described below.

#### Hb concentrations and anemia

Reductions in anemia prevalence due to SQ-LNSs were greater among studies conducted in South Asia than in Africa (PR: 0.64 compared with 0.81) (Figure 4B, Supplemental Figure 6B1). We observed a greater effect of SQ-LNSs on Hb concentrations among high anemia burden countries than among countries with a moderate burden of anemia (MD: 4.88 g/L compared with 2.49 g/L) (Figure 4A, Supplemental Figure 6A2), although the effect of SQ-LNSs on anemia prevalence was lower in high anemia burden countries than in moderate anemia burden countries (PR: 0.83 compared with 0.75) (Figure 4B, Supplemental Figure 6B2). In addition, there was a greater effect of SQ-LNSs on the prevalence of anemia among studies conducted in countries with a low than with a high malaria prevalence (PR: 0.73 compared with 0.84) (Figure 4B, Supplemental Figure 6B3) and at sites with a low inflammation prevalence than at those with a higher inflammation prevalence (PR: 0.71 compared with 0.87) (Figure 4B, Supplemental Figure 6B4). Study-level access to improved sanitation or water quality did not significantly modify the effects of SQ-LNSs on Hb concentrations (Figure 4A, Supplemental Figure 6A5, 6A6) or the prevalence of anemia (Figure 4B, Supplemental Figure 6B5, 6B6).

Among studies that provided supplements for longer than 12 mo compared with ≤12 mo, there was a greater effect of SQ-LNS on the prevalence of both anemia (PR: 0.69 compared with 0.83) (Figure 4B, Supplemental Figure 6B7) and moderate-to-severe anemia (PR: 0.62 compared with 0.74) (Figure 4C, Supplemental Figure 6D7). Similarly, among studies that provided a higher iron dosage (9 mg/d) compared with those providing a lower dosage (<9 mg/d), we observed a greater effect of SQ-LNSs on the prevalence of anemia (PR: 0.61 compared with 0.85) (Figure 4B, Supplemental Figure 6B8) and moderate-to-severe anemia (PR: 0.52 compared with 0.76) (Supplemental Figure 6D8). There was no effect modification by frequency of contact (weekly compared with monthly), but we did observe a greater effect of SQ-LNSs on Hb concentrations and the prevalence of anemia among studies that reported high compliance than among those reporting lower compliance (MD: 4.78 g/L compared with 2.59 g/L; Figure 4A, Supplemental Figure 6A10; and PR: 0.68 compared with 0.84; Figure 4B, Supplemental Figure 6B10, respectively).

#### Biomarkers of iron status and prevalence of deficiency

The effect of SQ-LNSs on the prevalence of iron deficiency (inflammation-adjusted ferritin concentration < 12 µg/L) was greater among trials conducted in South Asia than in Africa (PR: 0.31 compared with 0.49) (Figure 4E, Supplemental Figure 6G1), although the percentage point reduction in iron deficiency associated with SQ-LNSs was lower among sites in South Asia (than in Africa) (18 compared with 30 percentage points; *P* = 0.026) (Supplemental Figure 6H1). Study-level sanitation and water quality did not significantly modify the effects of SQ-LNSs on ferritin or sTfR concentrations or the relative reductions in the prevalences of iron deficiency and high sTfR (Figure 4D, E, G, H, Supplemental Figure 6F5, F6, G5, G6, K5, K6, L5, L6). We did observe greater effects of SQ-LNSs on the relative reduction in the prevalence of IDA among trials conducted in sites where a greater proportion of households had access to improved sanitation and water quality, than in sites with lower sanitation and water quality (PR: 0.20 compared with 0.43) (Figure 4F, Supplemental Figure 6I5, I6), consistent with the nonsignificant differences in the relative reductions in the prevalence of iron deficiency (PR: 0.38 compared with 0.51; *P*-diff = 0.105) (Figure 4E, Supplemental Figure 6G6). However, the percentage point reduction in iron deficiency due to SQ-LNSs was actually greater among trials conducted in sites where a lower (as opposed to higher) proportion of households had access to improved sanitation and water quality (28 compared with 18 percentage points; *P*-diff = 0.006) (Supplemental Figure 6H5, H6).

We observed a greater effect of SQ-LNSs on the prevalence of iron deficiency among studies that provided SQ-LNSs for longer than 12 mo than in studies of shorter durations (PR: 0.34 compared with 0.49) (Figure 4E, Supplemental Figure 6G7). In addition, we observed a greater effect of SQ-LNSs on ferritin concentrations among studies that provided 9 mg Fe/d than among studies that provided <9 mg/d (GMR: 1.67 compared with 1.42) (Figure 4D, Supplemental Figure 6F8). Frequency of contact did not significantly modify the effects of SQ-LNSs on ferritin or sTfR concentrations, nor on prevalences of iron deficiency or IDA. However, the effect of SQ-LNSs on elevated sTfR (indicative of impaired functional iron status) was greater among studies in which children received weekly as opposed to monthly study visits (PR: 0.46 compared with 0.69) (Figure 4H, Supplemental Figure 6L9).

#### Overview of study-level effect modification

Figure 5 shows that numerous characteristics of study context and design (e.g., anemia burden, iron dose, duration of supplementation) modified the effect of SQ-LNSs on hematological and iron status biomarkers. When there is no significant effect modification for a continuous outcome (e.g., Hb concentration) but there is for the PR or PD for the corresponding binary outcome (e.g., anemia), the results could be due to the “cutoff effect,” as described in the Methods and in a companion overview article (52). Our simulations identified several cutoff effects (Figure 5). For example, we observed greater effects of SQ-LNSs on the prevalence of anemia in sites with a moderate (compared with high) burden of anemia, a low (compared with high) malaria burden, and a low (compared with high) inflammation burden, 3 study-level characteristics that tended to cluster together. However, the effects of SQ-LNSs on Hb concentrations were greater in studies with a higher burden of anemia and mean Hb differences due to SQ-LNSs did not differ significantly by malaria or inflammation burden. Simulations indicated that the observed effect modification by study-level anemia, malaria, and inflammation burden with respect to the PR for anemia appears to be due to the cutoff effect (i.e., differences in the population distribution of Hb between subgroups). Among control group children, mean Hb concentrations were higher in sites with lower burdens of anemia, malaria, and inflammation than in sites with higher prevalences of these factors (∼114 g/L compared with ∼100 g/L). As a result, a greater proportion of children who received SQ-LNSs shifted across the cutoff from anemic to nonanemic in the former sites than in the latter. The cutoff effect also appeared to contribute, at least partially, to the apparent effect modification by *1*) region, for the prevalence of anemia and the PD for iron deficiency; *2*) improved sanitation and water quality, for the prevalence of IDA; *3*) supplementation duration and iron dose, for the prevalence of moderate-to-severe anemia; and *4*) frequency of contact, for the prevalence of elevated sTfR.

### Effect modification by individual-level characteristics

**FIGURE 6:**
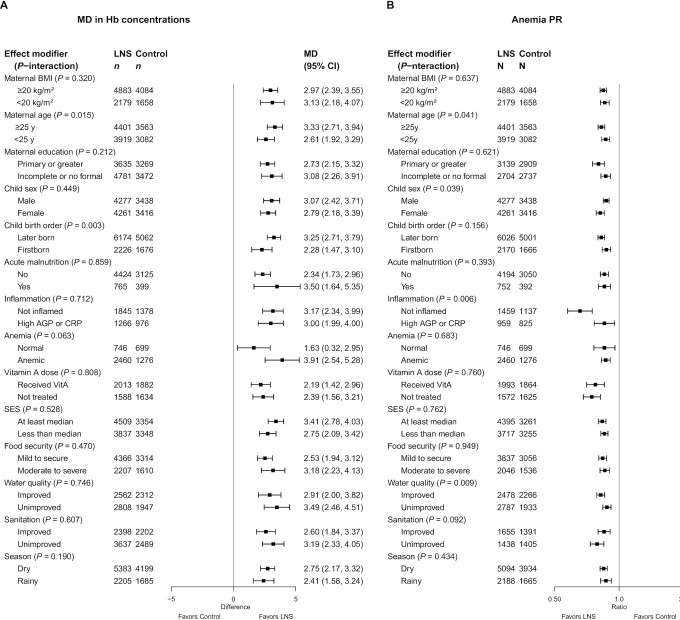

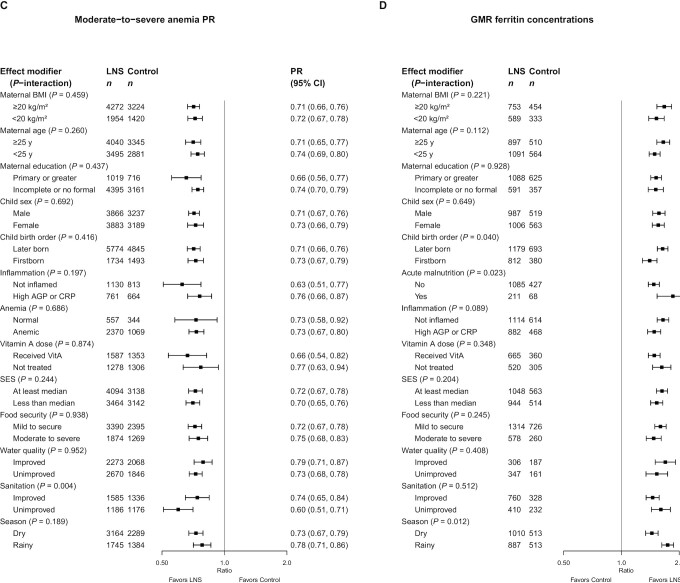

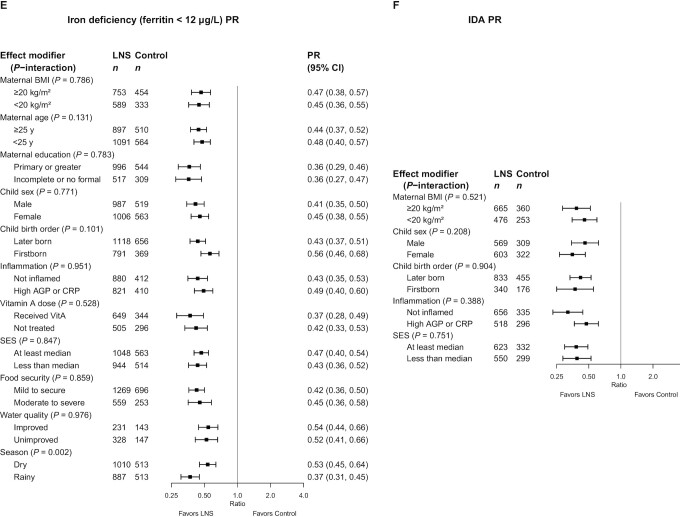

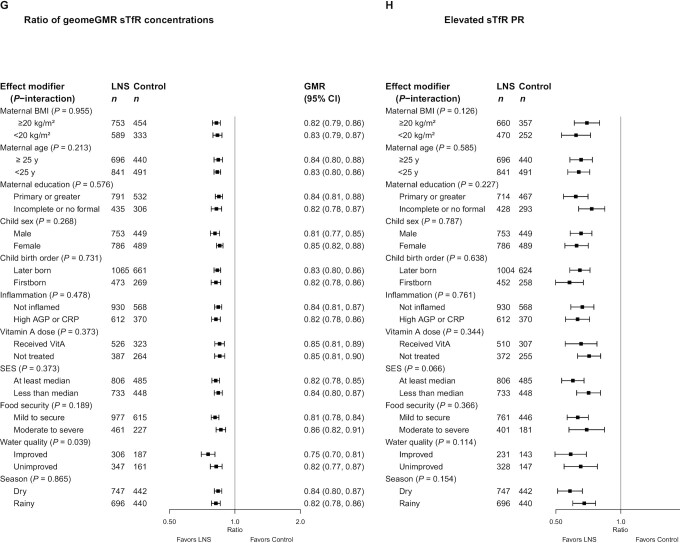
The effect of small-quantity LNSs provided to children 6–24 mo of age compared with a control on hemoglobin concentration (A), anemia prevalence (B), moderate-to-severe anemia prevalence (C), ferritin concentration (D), iron deficiency (E), IDA (F), sTfR concentration (G), and elevated sTfR (H), stratified by individual-level maternal, child, and household characteristics. Ferritin and sTfR concentrations were adjusted for inflammation (i.e., CRP and/or AGP concentrations, as available), using a regression correction approach adapted from the Biomarkers Reflecting Inflammation and Nutritional Determinants of Anemia (BRINDA) project (29). Individual study estimates for interaction effect were generated from log-binomial regression controlled for baseline measure when available and with clustered observations using robust SEs for cluster-randomized trials. Pooled subgroup estimates and statistical testing of the pooled interaction terms were generated using inverse-variance weighting fixed effects. For some biomarkers of micronutrient status, we were unable to generate pooled estimates for effect modification by certain potential individual-level effect modifiers owing to an insufficient number of comparisons. AGP, α-1-acid glycoprotein; CRP, C-reactive protein; GMR, ratio of geometric means; LNS, lipid-based nutrient supplement; MD, mean difference; *P*-interaction, *P* value for the interaction indicating the difference in effects of small-quantity lipid-based nutrient supplements between the 2 levels of the effect modifier; PR, prevalence ratio; SES, socioeconomic status; sTfR, soluble transferrin receptor.

**FIGURE 7:**
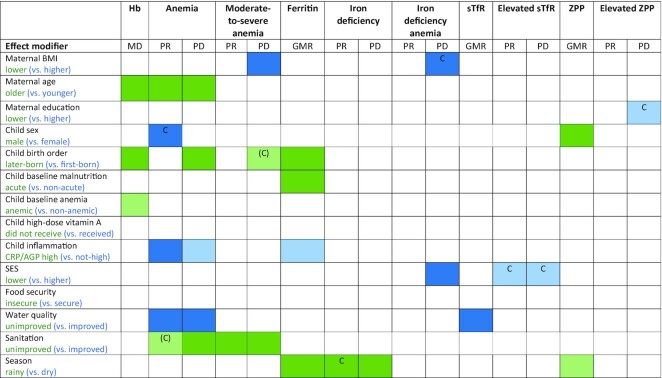
Overview of individual-level effect modification. The reference subgroup is the group expected to have the greatest potential to benefit. Green indicates a stronger effect in the reference subgroup; blue indicates a stronger effect in the opposite subgroup. Dark color indicates *P*-interaction <0.05; light color indicates 0.05< *P* <0.1. The letter “C” indicates that the apparent effect modification is due to the cutoff effect; when “C” is in parentheses, it is partially explained by the cutoff effect. AGP, α-1-acid glycoprotein; CRP, C-reactive protein; GMR, ratio of geometric means; Hb, hemoglobin; MD, mean difference; PD, prevalence difference; PR, prevalence ratio; SES, socioeconomic status; sTfR, soluble transferrin receptor; ZPP, zinc protoporphyrin.

**Figure 6**A–H presents the effects of SQ-LNSs on Hb (anemia) and biomarkers of iron status, stratified by individual-level (i.e., child, maternal, and household) characteristics, and **Figure 7** provides an overview of individual-level effect modification. Forest plots of all outcomes by potential individual-level effect modifiers are presented in **Supplemental Figures 8**A–AA and **9**A–AA. For some biomarkers of micronutrient status, we were unable to generate pooled estimates for effect modification by certain potential individual-level effect modifiers because <3 trials (comparisons) assessed both the outcome and the effect modifier of interest and/or there were too few individuals with the outcome categorized into each stratum (e.g., ferritin concentration by baseline anemia status or household sanitation). Effect modification results were generally consistent across all sensitivity analyses and between the fixed- and random-effects models (**Supplemental Figure 10**A–AA). Because all-trials analyses maximized the sample size, some potential effect modifiers were significant in the all-trials analyses, but not in the sensitivity analyses. However, the directionality of effect modification was consistent across all analyses. In addition, results were generally consistent between models in which outcomes were inflammation-adjusted or not (data not shown). Thus, the results presented below refer to the fixed-effects models for all-trials analyses of inflammation-adjusted outcomes.

#### Hb concentration and prevalence of anemia

In general, maternal characteristics did not significantly modify the effect of SQ-LNSs on Hb concentration or anemia, with 2 exceptions: there was a greater absolute reduction in the prevalence of moderate-to-severe anemia (8 compared with 6 percentage points, *P*-interaction = 0.047) (Supplemental Figure 8E1) among children of mothers with a higher (as opposed to lower) BMI, and there was a greater effect of SQ-LNSs on Hb concentration and anemia prevalence among children of older as opposed to younger mothers (MD: 3.33 g/L compared with 2.61 g/L and PR: 0.83 compared with 0.87, respectively; Figure 6A, B, Supplemental Figure 8A2, B2).

Regarding child characteristics, SQ-LNSs had a greater effect among later-born than among first-born children on Hb concentrations (MD: 3.25 g/L compared with 2.28 g/L) (Figure 6A, Supplemental Figure 8A5) and the percentage point reduction in the prevalence of anemia (12 compared with 7 percentage points; *P*-interaction = 0.024) (Supplemental Figure 8C5), although not on the relative reduction in anemia prevalence (Figure 6B, Supplemental Figure 8B5). The effect of SQ-LNSs on Hb concentration did not differ by child sex (Figure 6A, Supplemental Figure 8A4) but we observed a greater effect of SQ-LNSs on the prevalence of anemia among female than among male children (PR: 0.82 compared with 0.87) (Figure 6B, Supplemental Figure 8B4). SQ-LNSs had a greater effect on Hb concentrations, but not the prevalence of anemia, among children who were anemic at baseline (than among those who were not) (MD: 3.91 g/L compared with 1.63 g/L) (Figure 6A, B, Supplemental Figure 8A7, B7). The effect of SQ-LNSs on Hb concentration did not differ by inflammation status at endline (Figure 6A, Supplemental Figure 8A9); however, SQ-LNSs had a greater effect on the prevalence of anemia among children without (as opposed to with) inflammation at endline (PR: 0.66 compared with 0.85) (Figure 6B, Supplemental Figure 8B9). Child acute malnutrition at baseline and receipt of high-dose vitamin A supplements did not significantly modify the effects of SQ-LNSs on Hb concentrations or the prevalence of anemia.

Household water quality and sanitation did not significantly modify the effects of SQ-LNSs on Hb concentrations (Figure 6A, Supplemental Figure 9A3, A4). However, there was a greater effect of SQ-LNSs on anemia prevalence among children in households with improved water quality than among children in households with unimproved water quality (PR: 0.82 compared with 0.88) (Figure 6B, Supplemental Figure 9B3). Conversely, the effects of SQ-LNSs on prevalences of anemia and moderate-to-severe anemia were greater among children in households with unimproved than improved sanitation (Figure 6B, C, Supplemental Figure 9B4, D4). Socioeconomic status (SES), food security, and season of outcome measurement did not significantly modify the effects of SQ-LNSs on Hb concentrations or the prevalence of anemia.

#### Biomarkers of iron status and prevalence of iron deficiency and IDA

In general, maternal characteristics (i.e., BMI, age, and education) did not significantly modify the effect of SQ-LNSs on iron status biomarkers (i.e., ferritin, sTfR, or ZPP), with the exception of a greater absolute reduction in the prevalence of IDA (21 compared with 13 percentage points, *P*-interaction = 0.023) (Supplemental Figure 8J1) among children of mothers with a higher (as opposed to lower) BMI, and greater absolute reductions in the prevalence of elevated ZPP among children of mothers with more (as opposed to less) formal education (14 compared with 9 percentage points, *P*-interaction = 0.080) (Supplemental Figure 8P3).

We observed a greater effect of SQ-LNSs on ferritin concentrations among later-born children than among first-born children (GMR: 1.64 compared with 1.40) (Figure 6D, Supplemental Figure 8F5), among children who were acutely malnourished (as opposed to nonmalnourished) at baseline (GMR: 1.85 compared with 1.47) (Figure 6D, Supplemental Figure 8F6), and among children without (as opposed to with) inflammation at endline (GMR: 1.65 compared with 1.48) (Figure 6D, Supplemental Figure 8F9). In addition, we observed a greater effect of SQ-LNSs on ZPP concentrations in male than in female children (GMR: 0.76 compared with 0.82) (Supplemental Figure 8N4). No individual-level child characteristics modified the effect of SQ-LNSs on the prevalence of iron deficiency, elevated sTfR or ZPP, or IDA.

Household characteristics (i.e., SES, food security, water quality, and sanitation) did not modify the effect of SQ-LNSs on ferritin or ZPP concentrations or the prevalence of iron deficiency, elevated ZPP, or IDA, with the exception of greater absolute reductions in the prevalence of IDA among children of higher (as opposed to lower) SES (20 compared with 12 percentage points, *P*-interaction = 0.039) (Supplemental Figure 9J1). We observed a greater effect of SQ-LNSs on the prevalence of elevated sTfR among children in households with SES above (as opposed to below) the study-specific median (PR: 0.60 compared with 0.71) (Figure 6H, Supplemental Figure 9L1). In addition, SQ-LNSs had a greater effect on sTfR concentrations among children in households with improved (as opposed to unimproved) water quality (GMR: 0.75 compared with 0.82) (Figure 6G, Supplemental Figure 9K3).

There was a greater effect of SQ-LNSs on ferritin concentrations, and the prevalence of iron deficiency, in the rainy season than in the dry season (GMR: 1.74 compared with 1.44; PR: 0.37 compared with 0.53, respectively) (Figure 6D, E, Supplemental Figure 9F5, 9G5). In addition, there was a greater effect of SQ-LNSs on ZPP concentrations in the rainy season than in the dry season (GMR: 0.76 compared with 0.84) (Supplemental Figure 9N5).

#### Biomarkers of zinc and vitamin A status

The effect of SQ-LNSs on plasma zinc concentrations was greater among children of older than among children of younger mothers (GMR: 1.03 compared with 0.96) (Supplemental Figure 8Q2), and in the rainy season than the dry season (GMR: 1.02 compared with 0.99) (Supplemental Figure 9Q5). However, there was no main effect of SQ-LNSs on plasma zinc concentrations, and in both aforementioned cases of effect modification, the CIs around the estimates in both subgroups included 1; therefore, it is likely that there was no true effect on plasma zinc concentration in either subgroup. No other maternal, child, or household characteristics significantly modified the effect of SQ-LNSs on plasma zinc concentrations.

Maternal characteristics did not modify the effect of SQ-LNSs on vitamin A status. There was a greater effect of SQ-LNSs on RBP concentrations among children with (as opposed to without) inflammation at endline (GMR: 1.10 compared with 1.04) (Supplemental Figure 8W9). There was also a greater effect of SQ-LNSs on the prevalence of marginal vitamin A status (RBP < 1.05 µmol/L) among children in households with higher (as opposed to lower) SES (PR: 0.70 compared with 0.87) (Supplemental Figure 9Z1). In addition, we observed a greater effect of SQ-LNSs on retinol concentrations among children who were acutely malnourished (as opposed to nonmalnourished) at baseline (GMR: 1.14 compared with 1.02) (Supplemental Figure 8R6) and among children in mildly food-insecure or food-secure (compared with moderately to severely food-insecure) households (GMR: 1.06 compared with 0.99) (Supplemental Figure 9R2), although there was no main effect of SQ-LNSs on plasma retinol concentrations.

#### Overview of individual-level effect modification

Figure 7 and **Supplemental Figure 11** show that some characteristics (e.g., birth order, household water quality and sanitation, season, and child inflammation) modified the effect of SQ-LNSs on a number of hematological and iron status biomarkers, whereas others (e.g., maternal education, child sex, child anemia at baseline, high-dose vitamin A supplementation, and household food security) exhibited effect modification for only a few outcomes.

Figure 7 and Supplemental Figure 11 indicate the results of the simulations to identify cutoff effects. For example, among children who received SQ-LNSs compared with control, the prevalence of anemia was reduced by 18% among females and by 13% among males. However, there was no significant effect modification for the continuous outcome (i.e., Hb concentration). Mean Hb concentrations in the control group at endline were greater among females than males (107 compared with 105 g/L) and effect modification by sex became nonsignificant for anemia prevalence in the simulation models; therefore, the cutoff effect is the most likely explanation for these findings. The cutoff effect appeared to explain effect modification by maternal BMI with regard to the PD for IDA, and by maternal education with regard to the PD for elevated ZPP. For the prevalence of elevated sTfR (ratio and difference), the cutoff effect appeared to explain effect modification by SES. The cutoff effect also appeared to contribute, at least partially, to the apparent effect modification by sanitation for relative anemia prevalence (but not the PD for anemia, nor the PR or PD for moderate-to-severe anemia).

For maternal age, however, there was significant effect modification for Hb concentrations and both the PR and PD for anemia, so this was not due to the cutoff effect. In addition, the cutoff effect did not explain effect modification for anemia by maternal BMI, birth order, inflammation, or water quality; for iron deficiency by season of outcome assessment; or for IDA and vitamin A deficiency by SES.

## Discussion

This IPD analysis included 13 RCTs in 9 different countries with a total sample size of ∼15,000 children. SQ-LNSs substantially reduced the prevalence of anemia, moderate-to-severe anemia, iron deficiency, and IDA among infants and young children who received SQ-LNSs for 3–18 mo, relative to control children. The beneficial effects of SQ-LNSs on Hb appeared to be greater among studies that were conducted in countries with a high burden of anemia (>60%); greater beneficial effects of SQ-LNSs on anemia and iron status were observed among studies that provided SQ-LNSs with a higher dose of iron and for a longer duration. Several of the individual-level characteristics also appeared to modify the effects of SQ-LNSs on anemia and iron status. However, even when the magnitude of effect differed between subgroups, the magnitude of the modifying effects was generally small and we observed positive effects of SQ-LNSs within all subgroups, indicating the potential of SQ-LNSs to provide benefits across a range of individual, population, and study design characteristics. This was also the case for the growth (19) and development (20) domains of this meta-analysis.

### Main effects of SQ-LNSs on anemia and micronutrient status

Children who received SQ-LNSs had significantly higher Hb concentrations (2.77 g/L) relative to the control, and SQ-LNSs reduced the prevalence of anemia and moderate-to-severe anemia by 16% (10 percentage points) and 28% (7 percentage points), respectively. Although these effects are smaller than what was reported in the 2019 Cochrane systematic review and meta-analysis of LNSs (SQ- or MQ-LNSs) for Hb (MD: 5.78 g/L; 95% CI: 2.27, 9.30 g/L) and anemia (RR: 0.79; 95% CI: 0.69, 0.90) (7), our meta-analysis includes a larger number of trials and individual participants (*n* = 13 trials and 15,562 participants) than the Das et al. meta-analysis (*n* = 4–5 trials and 2332–4518 participants), which increases the precision of the effect estimates. Furthermore, we report estimates for iron deficiency and IDA, which were not included in the previous meta-analysis of SQ- and MQ-LNSs. SQ-LNSs reduced the prevalence of iron deficiency (plasma ferritin < 12 µg/L) by 56% (22 percentage points) and the prevalence of IDA by 64% (14 percentage points). At endline, the prevalence of IDA across all studies among children in the control groups was 23.5%, compared with 7.9% among children who received SQ-LNSs. These large relative and absolute reductions in the prevalence of iron deficiency and IDA due to SQ-LNSs are important given that iron deficiency is the most common documented micronutrient deficiency globally (4) and IDA is associated with compromised mental, motor, socio-emotional, and neural development (53).

The findings of the present SQ-LNS meta-analysis are similar to those reported in a recent Cochrane systematic review and meta-analysis of the effect of MNPs on Hb (MD: 2.74 g/L; 95% CI: 1.95, 3.53 g/L) and anemia (RR: 0.82; 95% CI: 0.76, 0.90) (*n* = 16–20 trials and 9927–10,509 participants) (54). Suchdev et al. (54) also reported a 53% reduction in the prevalence of iron deficiency (as defined by trialists) by MNPs (*n* = 7 trials and 1634 participants); the effect of MNPs on the prevalence of IDA was not reported. Another meta-analysis of MNP efficacy trials by Tam et al. (8) reported relative reductions in the prevalence of iron deficiency and IDA of 50% and 55%, respectively. Of note, in most of the MNP trials, the dosage of elemental iron was 10–12.5 mg/d (primarily as ferrous fumarate), whereas the SQ-LNS trials in our analyses provided only 6–9 mg/d (primarily as ferrous sulfate).

In the present analyses, there was considerable heterogeneity in the effect of SQ-LNSs on anemia (reductions ranging from 3 to 29 percentage points), iron deficiency (reductions ranging from 14 to 35 percentage points), and IDA (reductions ranging from 8 to 29 percentage points). This heterogeneity may be due to differences in population characteristics (e.g., baseline prevalence of anemia, iron deficiency, and IDA; proportion of anemia attributable to nutritional deficiencies compared with underlying burdens of infection and other causes) and study-design characteristics. The more modest relative reduction in the prevalence of anemia than in IDA may reflect the influence of nonnutritional causes of anemia in these populations (e.g., genetic Hb disorders and infection and inflammation including malaria, intestinal parasites, and schistosomiasis). In addition, although SQ-LNSs substantially reduced the prevalence of IDA, they did not completely eliminate IDA, possibly reflecting factors such as poor iron absorption (due to antinutritional compounds, gastric pH, or intestinal or systemic inflammation) (1) or an Hb cutoff for anemia that is set inappropriately high for infants and young children, especially in African populations (55–57). However, it is noteworthy that the endline prevalence of iron deficiency among children who received SQ-LNSs was ∼16% (compared with 40% among children in the control groups), which is comparable with the prevalence of iron deficiency among children 12–36 mo of age in the United States (13.5%) (58).

We observed significant increases in RBP concentrations among children who received SQ-LNSs, although the relative increase was small (7%). The difference in plasma retinol was in the same direction, but not statistically significant. Our ability to draw conclusions was limited owing to small sample sizes (*n* = 663–1236). However, in populations with adequate vitamin A stores, serum retinol may not respond to an intervention owing to homeostatic regulation (59). At endline, the prevalence of vitamin A deficiency (retinol or RBP < 0.70 µmol/L) in the control groups ranged from 1.1% to 16.2%; in 3 of 4 trials that assessed serum retinol, vitamin A deficiency was considered to be a mild public health problem (≥2% to <10%) (60). Although there is not an established cutoff to define vitamin A deficiency based on RBP (61), all 5 trials that assessed RBP reported <10% prevalence of RBP < 0.70 µmol/L. In the majority of studies in this analysis that assessed vitamin A status (7 of 9), more than half of study participants had received high-dose vitamin A supplementation (100,000–200,000 IU) within the 6 mo before outcome assessment. Therefore, in some study contexts, the daily low dose of vitamin A provided in SQ-LNSs may not have provided an additive benefit. However, SQ-LNSs did reduce the prevalence of vitamin A deficiency (RBP < 0.70 µmol/L) by 56% (corresponding to a modest 3 percentage point reduction, due to the low overall prevalence of deficiency), indicating the potential of this intervention to improve the status of those at-risk.

There was no overall effect of the intervention on plasma zinc concentrations, consistent with results from zinc-fortified food trials (62). Individual and population mean plasma zinc concentrations have been shown to increase significantly after zinc supplementation, regardless of initial concentrations of plasma zinc (63). Based on this and other considerations, plasma zinc concentration has been recommended by international zinc expert groups as the only valid biochemical indicator currently available to assess both the risk of zinc deficiency in populations and population-level exposure to zinc supplementation (64). The lack of effect of SQ-LNSs on plasma zinc concentrations may potentially be due to the low bioavailability of zinc in SQ-LNSs when provided as part of a food matrix that contains phytate (65), or differences in postabsorptive metabolism between zinc supplements and zinc-fortified foods (64).

### Effect modification

For both study- and individual-level effect modifiers, it is important to distinguish between the potential to benefit and the potential to respond (18). The potential to benefit is more likely when a population or an individual child is more vulnerable, e.g., owing to a high prevalence of anemia or micronutrient deficiencies in the study population at baseline. However, in some cases, children who are more vulnerable may actually be less likely to respond to a nutritional intervention because of other constraints on nutritional status such as infection and inflammation or inadequate care. Thus, we will attempt to frame the following discussion of effect modifiers in this context.

#### Effect modification by study-level characteristics

Studies included in this analysis were conducted in a variety of geographic and environmental contexts (e.g., different regions, different burdens of anemia and infection including malaria, different community-level access to improved water, sanitation, and hygiene) and used varying intervention designs (e.g., durations of supplementation, iron dose, frequency of contact between participants and study staff) and this enables us to examine whether these study context and design features modify the effects of SQ-LNSs. However, it is difficult to disentangle the impact attributable to one effect modifier from the influence of other characteristics of the study and thus these results should be interpreted with caution. For example, studies that provided a higher iron dose were more likely to provide SQ-LNSs for a longer duration, be conducted in South Asia, and have a higher average compliance with supplementation (>80%), thus making it difficult to disentangle the impact attributable to each specific study-level characteristic. In addition, it is important to note that owing to concerns about adverse effects of iron supplementation in malaria-endemic areas (66, 67), a number of the SQ-LNS studies in these analyses were purposefully designed to provide a lower iron dose in countries with a high burden of malaria (6).

The effect of SQ-LNSs on Hb concentrations was greater in studies with a higher burden of anemia (>60%), suggesting a greater potential to benefit from SQ-LNSs in such populations. Similarly, the effect of MNPs on Hb concentrations has been shown to be greater in populations that were anemic at baseline than in populations with mixed/unknown baseline anemia status (MD: 4.53 g/L compared with 3.05 g/L) (54). However, even in populations with a lower burden of anemia, both SQ-LNSs and MNPs significantly increased Hb concentrations and reduced the prevalence of anemia. In the present analyses, increases in Hb concentrations due to SQ-LNSs were not significantly different between populations with and without a high burden of malaria (4.29 g/L and 2.59 g/L, respectively) or inflammation (3.84 g/L and 3.21 g/L, respectively). Thus, these results, coupled with the observation that statistically significant effects of SQ-LNSs were found in both subgroups, do not support the hypothesis that populations with a high burden of malaria and inflammation have less of a potential to respond to iron interventions owing to increased hepcidin concentrations and downregulation of iron absorption (68, 69).

Effect modification by study-level water quality and sanitation did not reach statistical significance for the majority of hematological and iron status outcomes, with the exception of a greater percentage point reduction in iron deficiency due to SQ-LNSs in study sites with a lower proportion of households with access to improved water quality and sanitation. This is likely due to a higher prevalence of iron deficiency among children in the study sites with less access to improved water quality and sanitation; among control group children, prevalence of iron deficiency was 57% in those study sites, compared with 28% in sites with greater access.

Greater reductions in the prevalence of anemia and iron deficiency were observed in trials that provided SQ-LNSs for longer than 12 mo, than in those of a shorter duration (6–12 mo). In addition, studies that reported higher average compliance (>80%) showed greater increases in Hb concentrations and reductions in the prevalence of anemia, but we did not observe significant effect modification by frequency of contact with programmatic or study staff. Finally, we observed greater effects of SQ-LNSs on the prevalence of anemia and increase in ferritin concentrations among studies that provided 9 mg Fe/d as opposed to <9 mg Fe/d. Although it may be difficult to disentangle the impact attributable to each of these aforementioned study-level characteristics, they all suggest a greater effect among studies in which children received a higher total iron dose, either through a longer duration of supplementation, by higher compliance, or a higher daily iron dose. Consistent with this, meta-analyses of the effectiveness of MNP and iron supplements in infants and young children have also shown higher iron doses to have a greater impact on iron and hematological status (8, 54).

Differences in trial design (program-based compared with not) and SBCC for IYCF across trials were not considered as formal study-level effect modifiers. Five of the 13 trials in this IPD analysis were conducted within existing community- or clinic-based programs (36, 39, 43, 46–48), with the rest implemented entirely by research teams. There were no significant differences in effect sizes for anemia between the former and the latter. Thus, the findings reflect the impact of SQ-LNSs across the spectrum from efficacy trials to effectiveness studies in a real-world context. In addition, the effects of SQ-LNSs on anemia were evident regardless of whether the trial simply reinforced the normal IYCF messages already promoted in that setting (36, 38, 40, 41, 44, 45), or the trial provided expanded SBCC for IYCF—either in the SQ-LNS intervention arms only (37, 39, 42, 47, 48), or in both the intervention and control arms (35, 43, 46).

#### Effect modification by individual-level characteristics

The effects of SQ-LNSs on Hb concentrations and the prevalence of anemia were greater among children of older mothers than among those of younger mothers (relative reductions in anemia of 17% and 13%, respectively). Similarly, SQ-LNSs had a larger effect on both Hb and ferritin concentrations among later-born than among first-born children, and reduced the prevalence of anemia in these subgroups by 12 and 7 percentage points, respectively. SQ-LNSs also had a larger effect on stunting, underweight, and midupper arm circumference among later-born than among first-born children (19). These results likely indicate a greater potential to benefit among later-born children. For example, Hb concentrations among control group children in this IPD analysis were lower among later-born than first-born children (105.5 compared with 108.4 g/L). Later-born children, who have ≥1 older siblings, may compete more for caregiving and family resources than first-born children, making them more vulnerable to anemia and iron deficiency and therefore more likely to benefit from nutritional supplementation targeted specifically to young children. In addition, later-born children, who are more likely to be born to older mothers, may be more vulnerable to micronutrient deficiencies due to poor maternal physiological status owing to high fertility rates and short interpregnancy intervals (70, 71).

The effects of SQ-LNSs on biomarkers of hematological and iron status (i.e., ferritin, sTfR, ZPP) did not differ by child sex, with the exception of greater reductions in ZPP concentrations due to SQ-LNSs among males than among females (24% compared with 18%). The former may have had a greater potential to benefit, given that median ZPP concentrations were higher among control group males than females in this analysis (62.0 µmol/mol heme compared with 53.1 µmol/mol heme), and males tend to be more vulnerable to iron deficiency in infancy (72). However, both sexes responded positively to the intervention, with a 10 percentage point reduction in the prevalence of anemia and a 20–23 percentage point reduction in the prevalence of iron deficiency. Children who were anemic at baseline had greater increases in Hb concentrations due to SQ-LNSs than children who were not anemic at baseline, indicating a greater potential to benefit. A recent MNP trial also showed greater increases in Hb concentrations due to MNP among children who were anemic as opposed to nonanemic at baseline (73). In addition, these results are consistent with our study-level effect modification results, which indicated that increases in Hb due to SQ-LNSs were greater in populations with a high than in those with a moderate burden of anemia. Increases in ferritin concentrations due to SQ-LNSs were larger among children who were acutely malnourished at baseline than among those who were not. Among children in the control groups, median ferritin concentrations were lower among children with than among those without acute malnutrition (15.5 compared with 19.5 µg/L), also suggestive of a greater potential to benefit.

Although differences in the ability of populations to benefit from or respond to SQ-LNSs by the study-level burden of malaria or inflammation appeared to be explained by the cutoff effect, we did observe some differences at the individual level. SQ-LNSs reduced the prevalence of anemia by 34% (a 12 percentage point difference) among children without concurrent inflammation compared with 15% (a 5 percentage point difference) among children with elevated CRP and/or AGP concentrations, indicating a greater potential to respond among those without inflammation. Because inflammation is itself a cause of anemia (i.e., anemia of chronic disease) and can inhibit absorption of iron (68), it is likely that a higher proportion of anemia in the subgroup of noninflamed children was amenable to nutritional supplementation. Among children with inflammation, nonnutritional causes of anemia may have been more prevalent (e.g., anemia attributable to acute or chronic infection and inflammation) (29, 74). In the present analyses, the proportion of anemia not due to iron deficiency was greater (54% compared with 35%) among children with inflammation than among those without. In addition, it is possible that children without inflammation had a greater fractional absorption of iron relative to those with inflammation (75). Hepcidin, an important regulator of systemic iron balance, is elevated by inflammation, thereby inhibiting iron absorption (68). Although a few trials measured hepcidin, we had insufficient data to examine it in this analysis. However, greater increases in ferritin concentrations due to SQ-LNSs were observed among children without inflammation (65% compared with 48%), although there were no differences in the effect of SQ-LNSs on iron deficiency by inflammation status.

Greater effects of SQ-LNSs on reductions in the prevalence of anemia, and on reductions in sTfR concentrations, were seen among children in households with improved (as opposed to unimproved) water quality. Poor-quality water or sanitation may increase the risk of anemia due to infection with soil-transmitted helminths or environmental enteric dysfunction, which may increase inflammation or reduce nutrient absorption. Among children in the control group, the prevalence of anemia was higher among children without (than with) access to improved water quality (69% compared with 56%). Thus, this effect modification might be seen as consistent with the aforementioned inflammation effect modification results. Children with less exposure to gastrointestinal pathogens through contaminated water supplies may have less inflammation and thus may be more able to respond to the SQ-LNS intervention. However, these findings are in contrast with the observed greater effects of SQ-LNSs on anemia and moderate-to-severe anemia among children in households with unimproved (as opposed to improved) sanitation. This may indicate a greater potential to benefit, given that the prevalences of anemia and moderate-to-severe anemia were higher among control group children in households with unimproved sanitation (by 7 and 10 percentage points, respectively). The contradictory effect modification results for water quality and sanitation are difficult to explain. It is notable, though, that water quality and sanitation did not modify the effect of SQ-LNSs on Hb concentrations, and there was evidence of beneficial effects of SQ-LNSs in all subgroups of children.

Greater effects of SQ-LNSs on ferritin concentrations and the prevalence of iron deficiency were seen among children for whom biomarkers were assessed in the rainy season. Among children in the control groups in this analysis, median ferritin concentrations were lower (13.9 compared with 16.1 µg/L), and the prevalence of iron deficiency higher (by 6 percentage points), when outcome assessments occurred during the rainy (as opposed to the dry) season. Thus, it is possible that this represents a greater potential to benefit, whereby the supplemental iron provided by SQ-LNSs compensates for seasonal differences in dietary intake and/or constraints on iron absorption (e.g., infection and inflammation). However, iron status at a given point in time reflects the cumulative effect of intake, absorption, and utilization over many months, so it is unclear whether effect modification by season is due to short-term, acute phenomena or longer-term exposure to adverse conditions that is correlated with season at outcome assessment. Other household-level characteristics, including SES and food security, did not generally modify the effect of SQ-LNSs on anemia or iron biomarker outcomes.

With regard to individual-level effect modification of outcomes related to zinc and vitamin A status, observed differences between the stratum-specific point estimates were generally small even when there were statistically significant *P* values. In the latter situation, results were generally consistent with the effect modification for hematological and iron status biomarkers, whereby children of older mothers, children who were acutely malnourished at baseline, children in households of higher SES and with greater food security, and children assessed during the rainy season had greater increases in biomarker concentrations or reductions in the prevalence of deficiency than those in the respective comparison subgroups.

### Strengths and limitations

Strengths of the present analyses include the large sample size, the substantial number of high-quality RCTs available, and the availability of IPD for all but 1 of the eligible trials. In addition, we were able to report the effects of SQ-LNSs on multiple indicators of micronutrient status that had not been included in prior meta-analyses. The majority of trials used the same analytical laboratory and platform for several biomarkers (ferritin, sTfR, RBP; Vit-Min Lab) or standardized analytical methods (ZPP), lending strength to the findings. The trial sites were diverse in terms of study context (e.g., burdens of anemia, malaria, and inflammation) and design (e.g., iron dose and duration of supplementation), which provided heterogeneity for exploration of study-level effect modifiers. We presented results in terms of MDs for continuous outcomes, as well as both PRs and PDs for binary outcomes. Triangulating the findings across these different estimates of impact aided in interpretation; the absolute PDs are particularly important for understanding potential public health impact (76). The findings were generally consistent across sensitivity analyses, as well as between fixed- and random-effects models, adding strength to the conclusions. For example, the “separation of multicomponent arms” sensitivity analysis limited comparisons to pairs of arms with the same nonnutrition components, and also excluded the maternal LNS trials/arms; results were nearly identical to those of the all-trials analysis. The consistency across sensitivity analyses indicates that the all-trials analysis, which includes a larger sample size and broader group of trials, presents a valid estimate of the causal effect.

Several limitations must be considered. Bangladesh was the only country represented in these analyses outside of Sub-Saharan Africa. Although Hb data were available for all 13 trials, fewer studies assessed biomarkers of iron, zinc, and vitamin A status (3–7 studies) and sample sizes for each of those biomarkers were smaller (1133–3078 compared with 15,398 for Hb). In addition, data were not yet available from a sufficient number of studies to be able to investigate the effects of SQ-LNSs on additional biomarkers, including plasma hepcidin, folate, and vitamin B-12 concentrations. Furthermore, not all trials assessed both CRP and AGP in all children at endline, potentially limiting the accuracy of adjustments for inflammation and/or reducing the available sample size. It should also be noted that a single assessment of inflammation may not adequately characterize the inflammation status of individual children over time or the effects that average inflammation status over the entire period of supplementation may have on micronutrient status.

Owing to limitations in the data (e.g., number of studies that assessed biomarker outcomes or effect modifiers of interest, or a low prevalence of the binary outcome or proportion of children within 1 of the effect modifier subgroups), we were unable to generate effect estimates of SQ-LNSs on all biomarkers by all potential effect modifiers for all trials. Overall, statistical power for study-level effect modification was constrained by the limited number of trials, so there may be meaningful differences in effect estimates between categories of trials even if the *P*-diff for interaction was not significant. On the other hand, the individual-level effect modification analyses involved multiple effect modifiers and numerous outcomes, so several of the significant *P*-interaction values are likely due to chance. As stated in the Methods section, we did not adjust for multiple hypothesis testing because the effect modification analyses are inherently exploratory. Finally, effect modification results should be interpreted with caution, because potential effect modifiers may be interrelated or confounded by unmeasured variables. This is particularly important for study-level characteristics because, as previously noted, it was not possible to completely disentangle the impact attributable to a specific effect modifier from the impact attributable to other characteristics of the study (e.g., study design, context, implementation).

### Programmatic implications

The present findings suggest that policy-makers and program planners should consider SQ-LNSs in the mix of interventions to prevent anemia and iron deficiency, and potentially also vitamin A deficiency. The overall effects of SQ-LNSs on iron deficiency (56% reduction) and IDA (64% reduction) were substantial and may improve child neurological development and immune function (77). Although the overall effects on anemia and moderate-to-severe anemia were more modest (16% and 28% reductions, respectively), this likely reflects the presence of anemias in these populations that are not nutrition responsive (e.g., genetic Hb disorders and infection and inflammation). Alternative interventions, including micronutrient supplementation, MNP, and food fortification, are also effective in reducing anemia and the prevalence of iron deficiency (8, 54); however, SQ-LNS provides the added benefits of reducing mortality (78), stunting, and wasting (19) and improving developmental outcomes (20).

In terms of program design, the effect modification results herein suggest that a greater impact of SQ-LNSs on hematological and iron status outcomes may be obtained by providing formulations with the higher dosage of iron (9 mg/d) and/or providing SQ-LNSs for the entire window from 6 to 24 mo. The iron dose of fortified products has been of concern because of evidence that iron-containing supplements and MNPs may increase susceptibility to malaria in endemic regions, as well as respiratory and gastrointestinal infections (66, 79–81), although 2 systematic reviews and meta-analyses did not indicate any increase in the risk of diarrhea or malaria from these interventions (54, 82). The present analysis did not examine potential adverse impacts (e.g., morbidity or mortality) of SQ-LNSs by iron dose, malaria burden, or other potential effect modifiers. However, most published trials have not reported differences in diarrheal or malarial morbidity between SQ-LNS and control groups (40, 42–44, 47, 48, 83–85), and some reported beneficial effects of SQ-LNSs on diarrheal prevalence (37) and duration of pneumonia, diarrhea, and dysentery (35). In addition, a recent meta-analysis reported an overall 27% reduction in the risk of mortality among children who received SQ-LNSs compared with control (78). The majority of studies providing the higher iron dose were in lower malaria burden sites (36, 37, 42) and, as such, we cannot recommend universal use of higher iron dose SQ-LNSs at this time. In areas with a low prevalence of malaria and inflammation, or in malaria-endemic areas with a well-integrated control program with appropriate surveillance and the prevention and management of malaria, providing the higher iron dosage (9 mg/d) in SQ-LNSs may be appropriate and have a greater impact on iron status and hematological parameters. Provision of SQ-LNSs containing 9 mg Fe/d for 18 mo would cumulatively provide ∼4925 mg Fe, compared with the 2200 mg Fe provided by 12 mo of SQ-LNSs containing 6 mg Fe/d, but would increase programmatic costs.

The effect modification results for biochemical outcomes generally did not provide a strong rationale for *targeting* SQ-LNSs only to the most vulnerable children or populations. Even in situations that were indicative of a greater potential to benefit among one subgroup than among another (e.g., greater increases in Hb concentrations among later-born than among first-born children and among anemic than among nonanemic children, greater increases in ferritin concentrations among acutely malnourished than among nonmalnourished children), both subgroups responded positively and appeared to benefit from the intervention. However, some of the results suggested that a greater impact of SQ-LNSs may be obtained by *combining* supplementation with interventions that address factors related to the potential to respond. Integrating the provision of SQ-LNSs with interventions to address the prevention and control of infection and inflammation (e.g., household- and/or community-level improvements in water, sanitation, and hygiene; use of insecticide-treated bed nets; and surveillance and treatment of diarrhea and malaria) may increase nutrient (iron) absorption, and thus increase the efficacy of the supplement.

### Conclusions

There is now substantial evidence demonstrating the efficacy of SQ-LNSs for prevention of anemia and iron deficiency. However, further research is needed to investigate the efficacy of SQ-LNSs for prevention of other vitamin and mineral deficiencies. In the present analysis, there was insufficient evidence to assess the effect of SQ-LNSs on folate and vitamin B-12 status, and no trials assessed status regarding other B-vitamins, vitamin C, vitamin D, and vitamin E, or minerals other than iron and zinc. Additional research is also needed on the optimal iron dose in SQ-LNSs and duration of supplementation, with regard to not only iron status and anemia but also morbidity, the costs and benefits of shorter compared with longer supplementation, and the potential to align the intervention with periods of increased vulnerability. Finally, further research would be useful to determine the optimal doses and formulations of micronutrients to include in SQ-LNSs, and to investigate the effect of additional compounds, such as phytase or galacto-oligosaccharides, that may improve mineral bioavailability.

## Supplementary Material

nqab276_Supplemental_Files

## Data Availability

Data described in the article, code book, and analytic code will not be made available because they are compiled from 13 different trials, and access is under the control of the investigators of each of those trials.

## Notes

Supported by Bill & Melinda Gates Foundation grant OPP49817 (to KGD).

Supplemental Methods, Supplemental Figures 1–11, and Supplemental Tables 1–7 are available from the “Supplementary data” link in the online posting of the article and from the same link in the online table of contents at https://academic.oup.com/ajcn/.

Published in a supplement to *The American Journal of Clinical Nutrition*. Publication costs for this supplement were defrayed in part by the payment of page charges. The Guest Editor for this supplement was Chris Sudfeld who reports no disclosures. The Supplement Coordinator for the supplement publication was Kathryn Dewey, University of California, Davis, Davis, CA. Kathryn Dewey has received funding from the Bill & Melinda Gates Foundation and the US Agency for International Development (via the Food and Nutrition Technical Assistance Project) for research on lipid-based nutrient supplements. The opinions expressed in this publication are those of the authors and are not attributable to the sponsors or the publisher, Editor, or Editorial Board of *The American Journal of Clinical Nutrition*.

Abbreviations used: AGP, α-1-acid glycoprotein; CRP, C-reactive protein; GMR, ratio of geometric means; GRADE, Grading of Recommendations Assessment, Development and Evaluation; Hb, hemoglobin; IDA, iron deficiency anemia; IPD, individual participant data; IYCF, infant and young child feeding; LNS, lipid-based nutrient supplement; MD, mean difference; MNP, multiple micronutrient powder; MQ, medium-quantity; PD, prevalence difference; PR, prevalence ratio; RBP, retinol-binding protein; RCT, randomized controlled trial; SBCC, social and behavior change communication; SES, socioeconomic status; SQ, small-quantity; sTfR, soluble transferrin receptor; WASH, water, sanitation, and hygiene; WLZ, weight-for-length *z* score; ZPP, zinc protoporphyrin.
