# Supplementary material for: Characteristics that modify the effect of small-quantity lipid-based nutrient supplementation on child anemia and micronutrient status: an individual participant data meta-analysis of randomized controlled trials
Source: Am J Clin Nutr. 2021 Sep 29;114(Suppl 1):68S–94S. doi: 10.1093/ajcn/nqab276 (PMC8560313; doi:10.1093/ajcn/nqab276)
Supplement: nqab276_Supplemental_Files [file nqab276_supplemental_files.zip › 4_ipdb_suppfig_table_of_contents_2021-03-18.pdf]

**Online Supplemental Material**

Characteristics that modify the effect of small-quantity lipid-based nutrient supplementation on child anemia and micronutrient status: an individual participant data meta-analysis of randomized controlled trials

Wessells *et al.* (2021)

**Table of Contents: Supplemental Figures**

Supplemental Figure 1: Summary risk of bias as a percentage of all included studies for the effects of SQ-LNS on biochemical outcomes

Supplemental Figure 2: Sensitivity analyses of main effects of SQ-LNS on biochemical outcomes

Supplemental Figure 3: Forest plots for all main effects of SQ-LNS on biochemical outcomes

Supplemental Figure 4: Forest plots for effects of SQ-LNS on biochemical outcomes stratified by study implementation within an existing program vs. not program-based

Supplemental Figure 5: Forest plots for effects of SQ-LNS on biochemical outcomes stratified by extent of social and behavioral change communication (SBCC) for infant and young child feeding (IYCF) provided by the study

Supplemental Figure 6: Forest plots for effects of SQ-LNS on growth outcomes stratified by study-level effect modifiers

Supplemental Figure 7: Sensitivity analyses of effect modification of SQ-LNS on biochemical outcomes by study-level effect modifiers

Supplemental Figure 8: Forest plots for effects of SQ-LNS on biochemical outcomes stratified by individual-level maternal and child effect modifiers

Supplemental Figure 9: Forest plots for effects of SQ-LNS on biochemical outcomes stratified by individual-level household effect modifiers

Supplemental Figure 10: Sensitivity analyses of effect modification of SQ-LNS on biochemical outcomes by individual-level effect modifiers

Supplemental Figure 11: Overview of individual-level effect modification
