# Supplementary material for: Characteristics that modify the effect of small-quantity lipid-based nutrient supplementation on child anemia and micronutrient status: an individual participant data meta-analysis of randomized controlled trials
Source: Am J Clin Nutr. 2021 Sep 29;114(Suppl 1):68S–94S. doi: 10.1093/ajcn/nqab276 (PMC8560313; doi:10.1093/ajcn/nqab276)

Online Supplemental Material: Characteristics that modify the effect of small-quantity lipid-based nutrient supplementation on child anemia and micronutrient status: an individual participant data meta-analysis of randomized controlled trials. Wessells et al. (2021)

Supplemental Figure 1: Summary risk of bias as a percentage of all included studies for the effects of SQ-LNS on biochemical outcomes

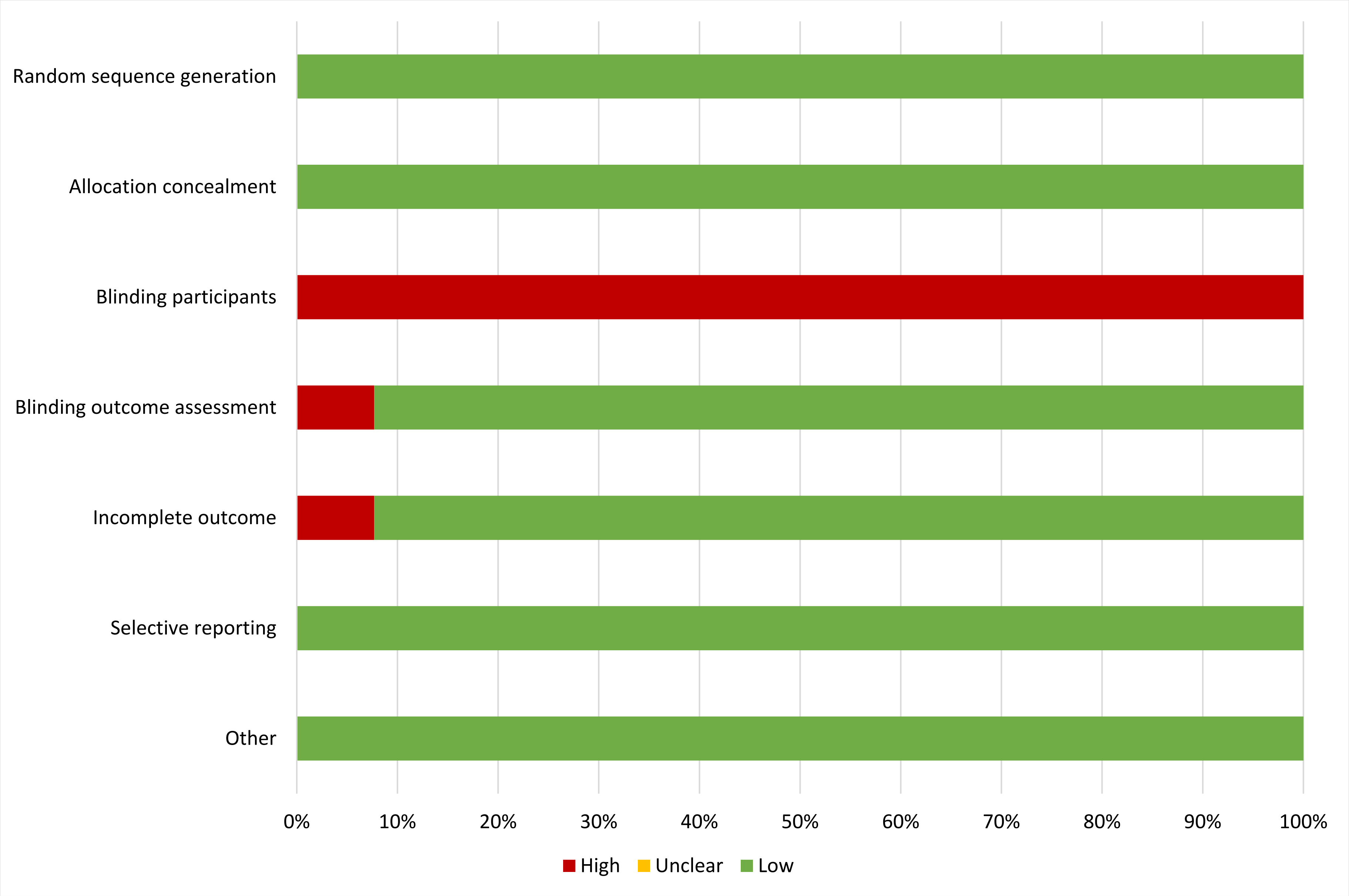

Supplement: nqab276_Supplemental_Files [file nqab276_supplemental_files.zip › 5_ipdb_suppfig1_20210401.pdf]
