# Supplementary material for: Characteristics that modify the effect of small-quantity lipid-based nutrient supplementation on child anemia and micronutrient status: an individual participant data meta-analysis of randomized controlled trials
Source: Am J Clin Nutr. 2021 Sep 29;114(Suppl 1):68S–94S. doi: 10.1093/ajcn/nqab276 (PMC8560313; doi:10.1093/ajcn/nqab276)

Online Supplemental Material: Characteristics that modify the effect of small-quantity lipid-based nutrient supplementation on child anemia and micronutrient status: an individual participant data meta-analysis of randomized controlled trials.
Wessells et al. (2021)

Supplemental figure 2: Sensitivity analyses of main effects of SQ-LNS on biochemical outcomes

Contents

Supplemental figure 2A: Mean differences in hemoglobin concentration **2**

Supplemental figure 2B:Geometric mean ratios for log transformed continuous outcomes **3**
Supplemental figure 2B1: Geometric mean ratios for ferritin, sTfR, and ZPP . . . 3
Supplemental figure 2B2: Geometric mean ratios for plasma zinc, retinol, and RBP . . . 4

Supplemental figure 2C: Prevalence ratios for dichotomous outcomes **5**
Supplemental figure 2C1: Prevalence ratios for anemia, moderate-to-severe anemia, iron deficiency, iron deficiency anemia, elevated sTfR, and elevated ZPP . . . 5
Supplemental figure 2C2: Prevalence ratios for low and marginal vitamin A status . . . 6

Supplemental figure 2D: Prevalence differences for dichotomous outcomes **7**
Supplemental figure 2D1: Prevalence differences for anemia, moderate-to-severe anemia, iron deficiency, iron deficiency anemia, elevated sTfR, and elevated ZPP . . . 7
Supplemental figure 2D2: Prevalence ratios for low and marginal vitamin A status . . . 8

These figures show the pooled estimates of intervention effects by different pooling methods and different sensitivity analyses. For continuous outcomes, the intervention effect is measured by the difference in mean of the LNS group minus control. For log transformed continuous outcomes, the intervention effect is measured by the ratio of geometric means, the effect estimate is the geometric mean in the LNS group divided by the geometric mean in the control group. For dichotomous outcomes analyzed via prevalence ratios, the effect estimate is the prevalence in the LNS group divided by the prevalence in the control group. For dichotomous outcomes analyzed via prevalence differences, the effect estimate is the prevalence in the LNS group minus the prevalence in the control group.
The labels on the left y-axis indicate which outcome is assessed. The different columns correspond to sensitivity analyses in which intervention group categorization differs. All-trial analysis includes all trials; Child-LNS-only excludes trial arms that provided both maternal and child LNS; Multi-component analysis separates comparisons within trials that included multi-component interventions, so that the SQ-LNS vs. no SQ-LNS comparisons were conducted separately between pairs of arms that included the same non-nutrition components (e.g. SQ-LNS+WASH vs. WASH; SQ-LNS vs. Control); Passive arms excluded analysis excludes passive control arms. Depending on the sensitivity analysis, there may not have been enough comparisons available to generate a pooled estimate.
Ferritin, sTfR, ZPP, zinc, retinol and RBP concentrations were adjusted for inflammation (i.e., C-reactive protein (CRP) and/or α-1-acid glycoprotein (AGP) concentrations, as available), using a regression correction approach adapted from the Biomarkers Reflecting Inflammation and Nutritional Determinants of Anemia (BRINDA) project (28)
sTfR, soluble transferrin receptor; ZPP, zinc protoporphyrin; RBP, retinol binding protein.

Supplemental figure 2A: Mean differences in hemoglobin concentration

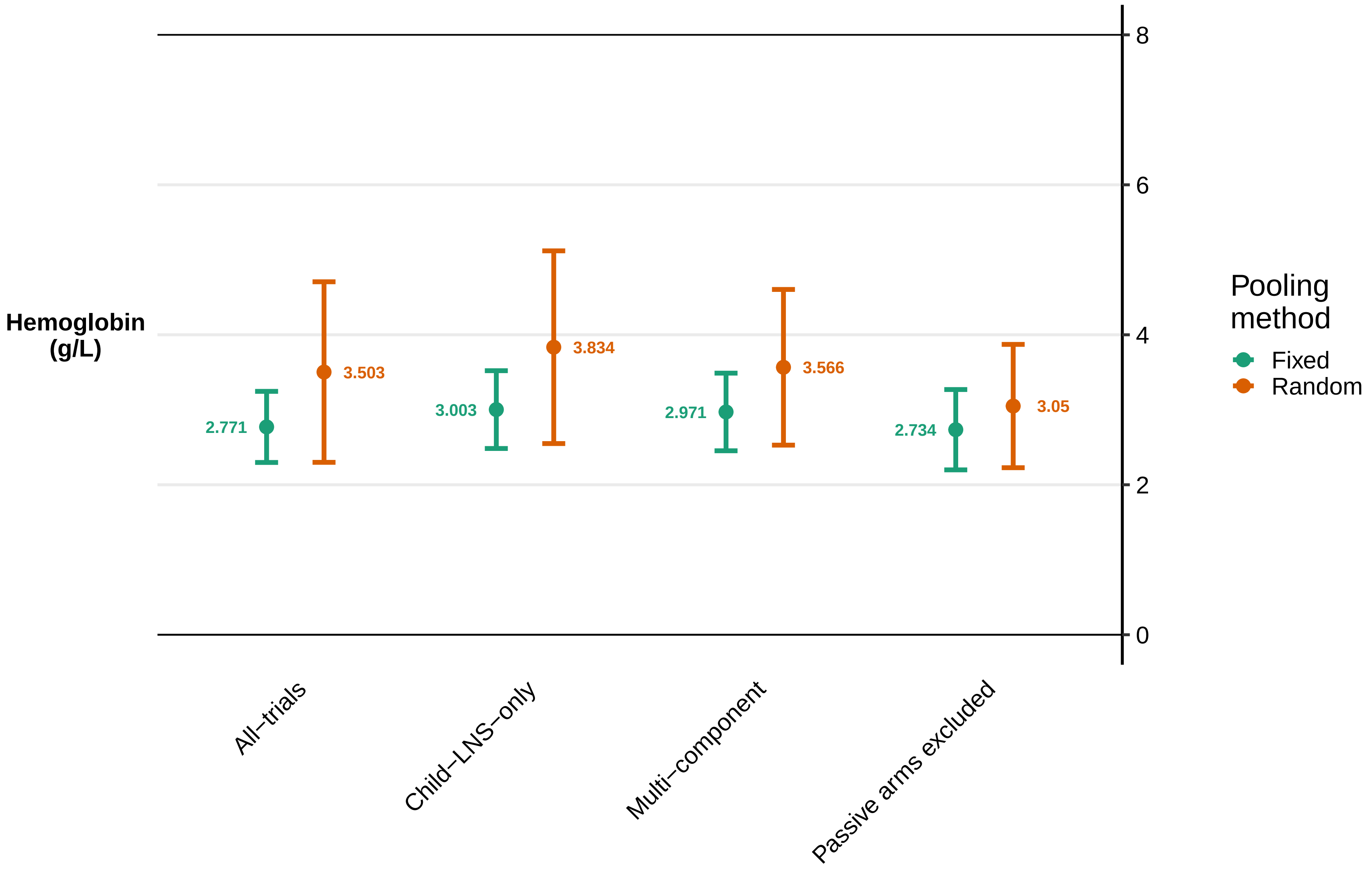

Supplemental figure 2B:Geometric mean ratios for log transformed continuous outcomes

Supplemental figure 2B1: Geometric mean ratios for ferritin, sTfR, and ZPP

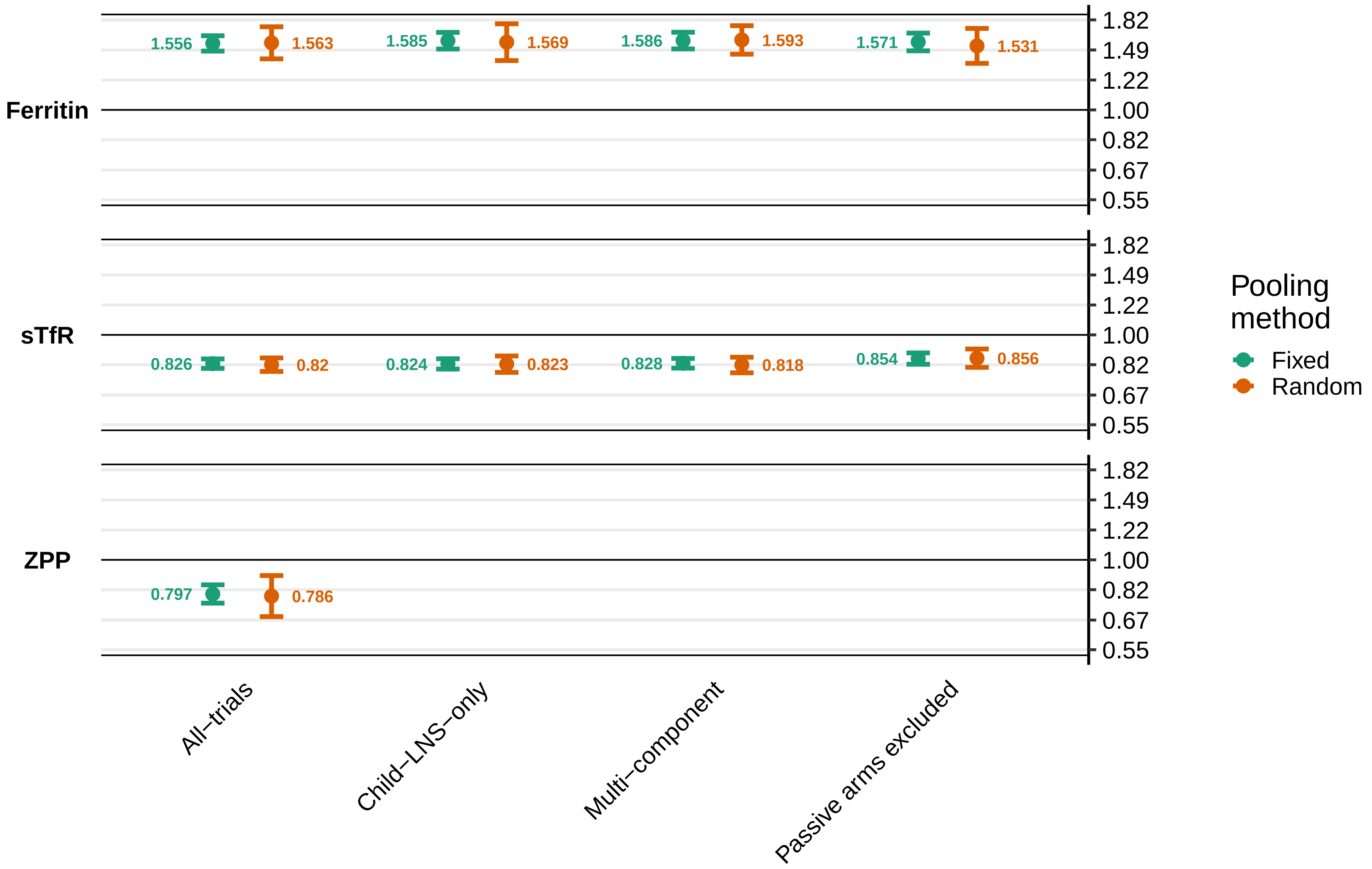

Supplemental figure 2B2: Geometric mean ratios for plasma zinc, retinol, and RBP

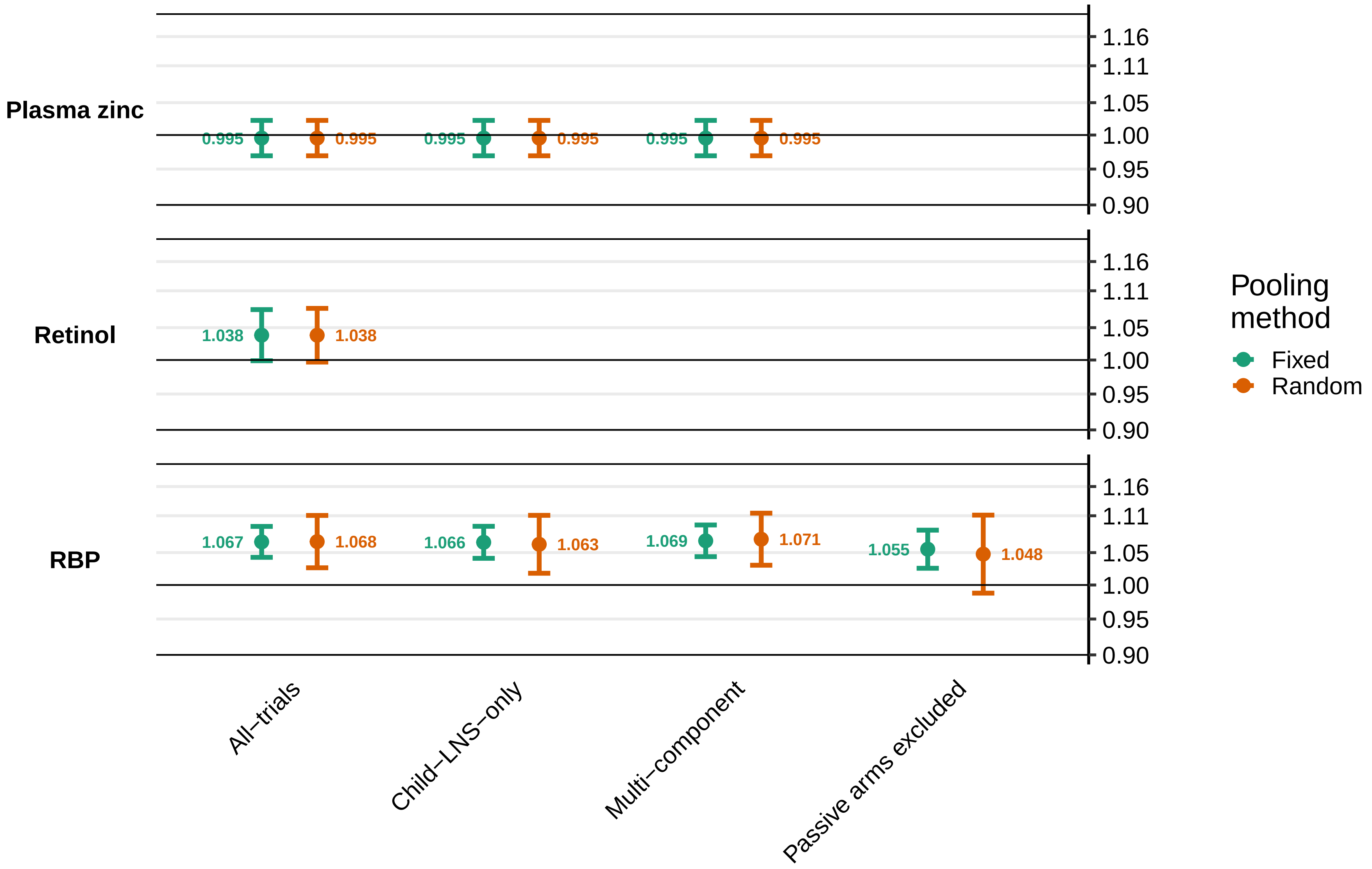

Supplemental figure 2C: Prevalence ratios for dichotomous outcomes

Supplemental figure 2C1: Prevalence ratios for anemia, moderate-to-severe anemia, iron deficiency, iron deficiency anemia, elevated sTfR, and elevated ZPP

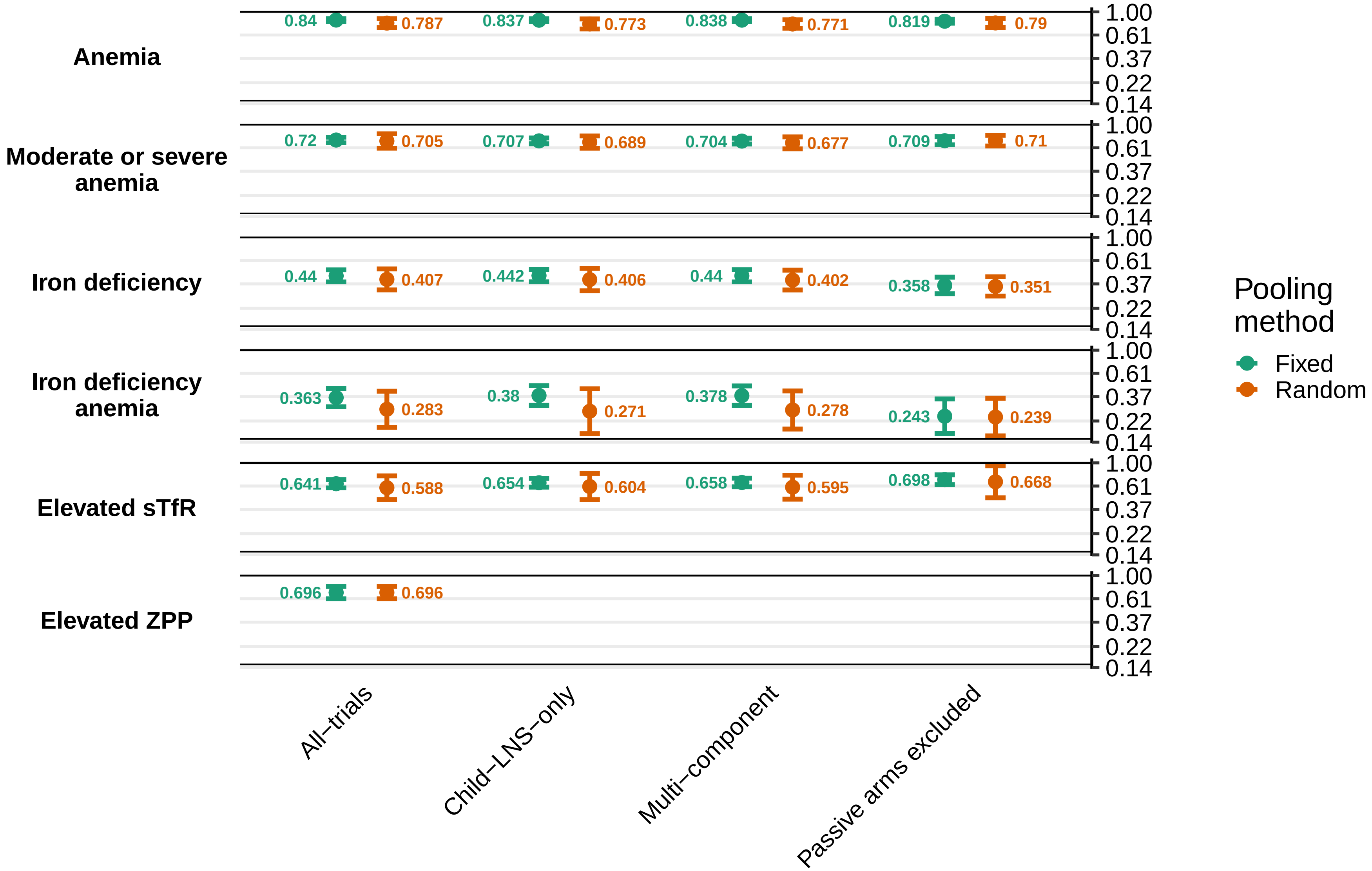

Supplemental figure 2C2: Prevalence ratios for low and marginal vitamin A status

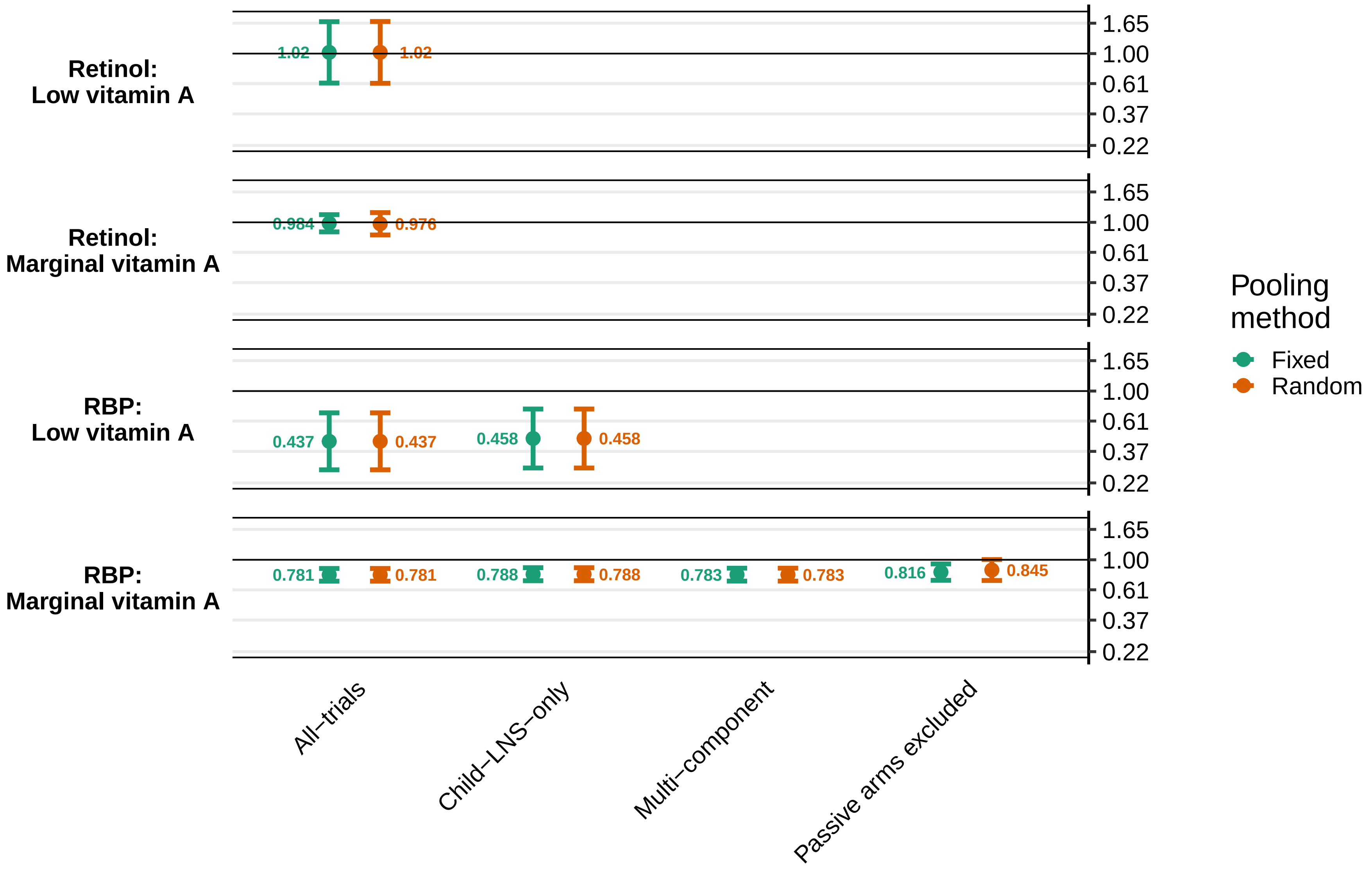

Supplemental figure 2D: Prevalence differences for dichotomous outcomes

Supplemental figure 2D1: Prevalence differences for anemia, moderate-to-severe anemia, iron deficiency, iron deficiency anemia, elevated sTfR, and elevated ZPP

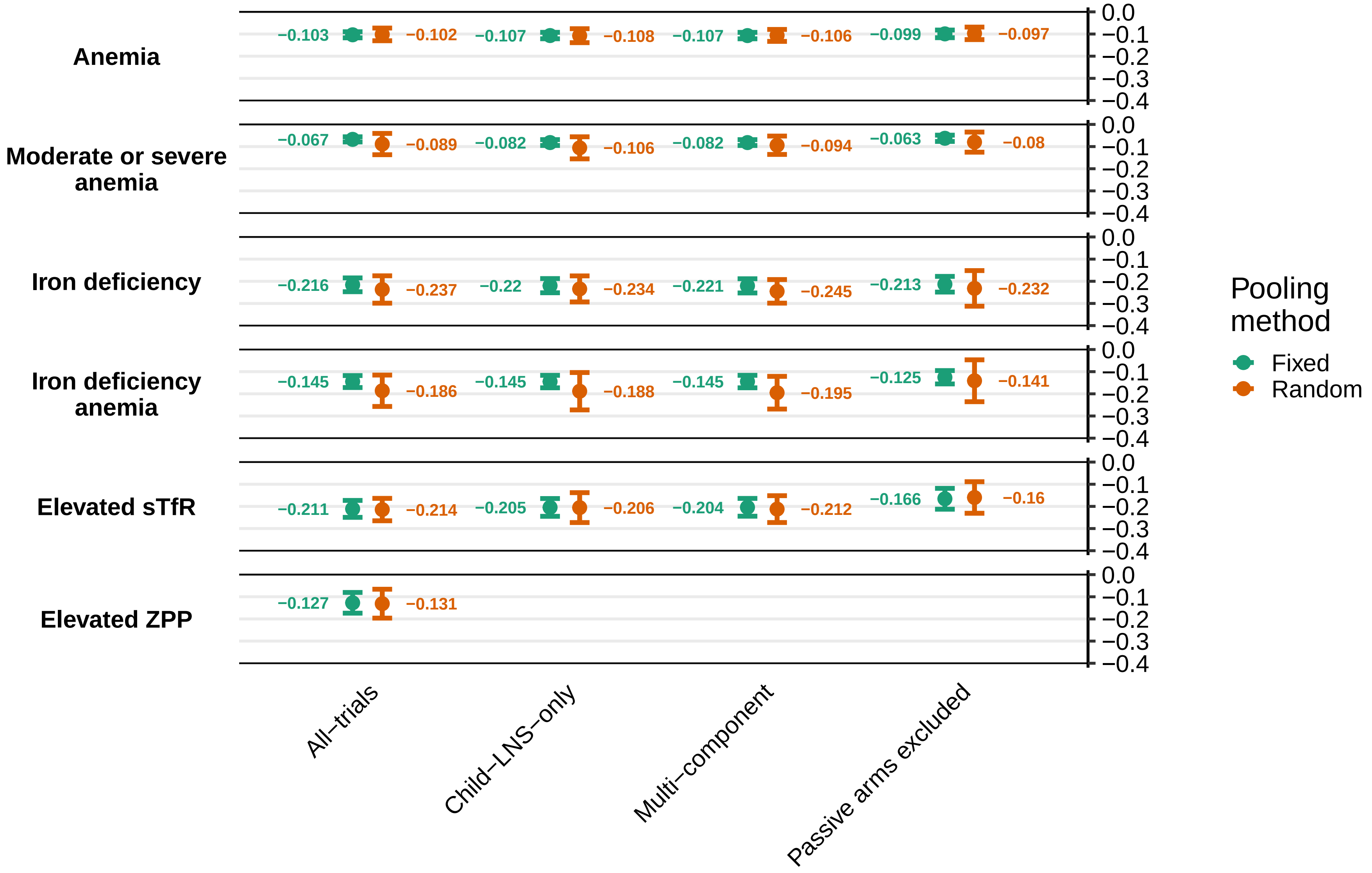

Supplemental figure 2D2: Prevalence ratios for low and marginal vitamin A status

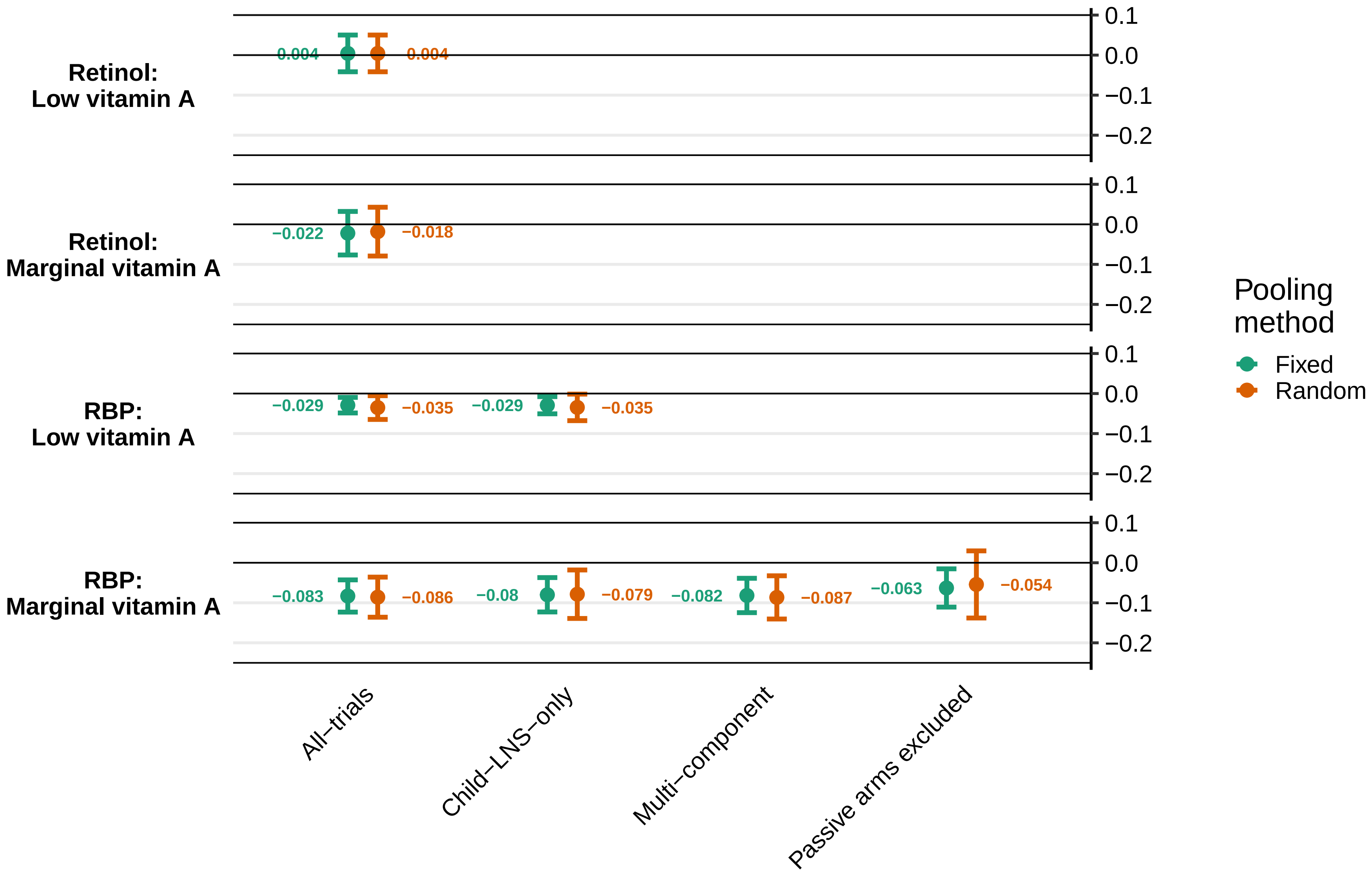

Supplement: nqab276_Supplemental_Files [file nqab276_supplemental_files.zip › 6_ipdb_suppfig2_20210331.pdf]
