# Supplementary material for: Characteristics that modify the effect of small-quantity lipid-based nutrient supplementation on child anemia and micronutrient status: an individual participant data meta-analysis of randomized controlled trials
Source: Am J Clin Nutr. 2021 Sep 29;114(Suppl 1):68S–94S. doi: 10.1093/ajcn/nqab276 (PMC8560313; doi:10.1093/ajcn/nqab276)

Supplemental figure 3: Forest plots for all main effects of SQ-LNS on biochemical outcomes

Contents

Supplemental figure 3A: Mean difference in hemoglobin concentration **3**

Supplemental figure 3B: Anemia prevalence ratio **4**

Supplemental figure 3C: Anemia prevalence difference **5**

Supplemental figure 3D: Moderate-to-severe anemia prevalence ratio **6**

Supplemental figure 3E: Moderate-to-severe anemia prevalence difference **7**

Supplemental figure 3F: Geometric mean ratio of ferritin concentration **8**

Supplemental figure 3G: Iron deficiency (ferritin < 12 µg/L) prevalence ratio **9**

Supplemental figure 3H: Iron deficiency (ferritin < 12 µg/L) prevalence difference **10**

Supplemental figure 3I: Iron deficiency anemia prevalence ratio **11**

Supplemental figure 3J: Iron deficiency anemia prevalence difference **12**

Supplemental figure 3K: Geometric mean ratio of soluble transferrin receptor concentration **13**

Supplemental figure 3L: Elevated soluble transferrin receptor prevalence ratio **14**

Supplemental figure 3M: Elevated soluble transferrin receptor prevalence difference **15**

Supplemental figure 3N: Geometric mean ratio of zinc protoporphyrin concentration **16**

Supplemental figure 3O: Elevated zinc protoporphyrin prevalence ratio **17**

Supplemental figure 3P: Elevated zinc protoporphyrin prevalence difference **18**

Supplemental figure 3Q: Geometric mean ratio of plasma zinc concentration **19**

Supplemental figure 3R: Geometric mean ratio of retinol concentration **20**

Supplemental figure 3S: Low vitamin A (retinol < 0.70 µmol/L) prevalence ratio **21**

Supplemental figure 3T: Low vitamin A (retinol < 0.70 µmol/L) prevalence difference **22**

Supplemental figure 3U: Marginal vitamin A (retinol < 1.05 µmol/L) prevalence ratio **23**

Supplemental figure 3V: Marginal vitamin A (retinol < 1.05 µmol/L) prevalence difference **24**

Supplemental figure 3W: Geometric mean ratio of retinol binding protein concentration **25**

Supplemental figure 3X: Low vitamin A status (RBP < 0.70 µmol/L) prevalence ratio **26**

Supplemental figure 3Y: Low vitamin A status (RBP < 0.70 µmol/L) prevalence difference **27**

Supplemental figure 3Z: Marginal vitamin A status (RBP < 1.05 µmol/L) prevalence ratio **28**

These figures are forest plots showing the study-level estimates of intervention effect with the pooled estimate in the bottom summary rows. Individual study estimates were generated from log binomial regression for dichotomous outcomes and simple linear regression for continuous outcomes; controlling for baseline measure when available and with clustered observations using robust standard errors for cluster-randomized trials. Pooled estimates were generated using inverse variance weighting in both fixed and random effects models. For continuous outcomes the intervention effect is measured by the difference in mean of the LNS group minus control. For log transformed continuous outcomes, the intervention effect is measured by the ratio of geometric means, the effect estimate is the geometric mean in the LNS group divided by the geometric mean in the control group. For dichotomous outcomes analyzed via prevalence ratios the effect estimate is the prevalence in the LNS group divided by the prevalence in the control group. For dichotomous outcomes analyzed via prevalence differences the effect estimate is the prevalence in the LNS group minus the prevalence in the control group. The labels on the left y-axis correspond to trial level information. The values on the right indicate the study level effect estimate, confidence interval, and weighting for deriving the pooled estimate.

Ferritin, sTfR, ZPP, zinc, retinol and RBP concentrations were adjusted for inflammation (i.e., C-reactive protein (CRP) and/or α-1-acid glycoprotein (AGP) concentrations, as available), using a regression correction approach adapted from the Biomarkers Reflecting Inflammation and Nutritional Determinants of Anemia (BRINDA) project (28).

RBP, retinol binding protein.

Supplemental figure 3A: Mean difference in hemoglobin concentration

Country	Trial	LNS N	Control N	MD (95% CI)	Fixed W	Random W
Bangladesh	JiVitA−4 (35)	457	146	2.21 (0.34, 4.07)	0.06	0.08
Bangladesh	RDNS (36)	549	272	4.10 (1.86, 6.34)	0.04	0.07
Bangladesh	WASH−B (37)	234	186	3.09 (1.22, 4.97)	0.06	0.08
Burkina Faso	iLiNS−Zinc (38)	1957	664	8.47 (5.47, 11.46)	0.03	0.06
Burkina Faso	PROMIS CS (39)	574	581	1.77 (−0.11, 3.64)	0.06	0.08
Ghana	GHANA (40)	98	96	8.84 (4.87, 12.81)	0.01	0.05
Ghana	iLiNS−DYAD−G (41)	328	661	1.24 (−0.14, 2.61)	0.12	0.09
Kenya	WASH−B (42)	350	300	3.94 (2.05, 5.84)	0.06	0.08
Madagascar	MAHAY (43)	600	588	2.77 (−0.12, 5.67)	0.03	0.06
Malawi	iLiNS−DYAD−M (44)	210	432	1.12 (−1.45, 3.69)	0.03	0.07
Malawi	iLiNS−DOSE (45)	243	82	3.76 (0.02, 7.51)	0.02	0.05
Mali	PROMIS CS (46)	953	970	6.25 (4.16, 8.34)	0.05	0.08
Zimbabwe	SHINE (HIV−) (47)	1682	1594	2.13 (1.36, 2.91)	0.37	0.09
Zimbabwe	SHINE (HIV+) (48)	306	285	2.78 (0.38, 5.19)	0.04	0.07
	**I² = 0.72, Tau² = 3.88**	**8541**	**6857**			
	**Fixed**			**2.77 (2.30, 3.25)**		
	**Random**			**3.50 (2.30, 4.71)**		

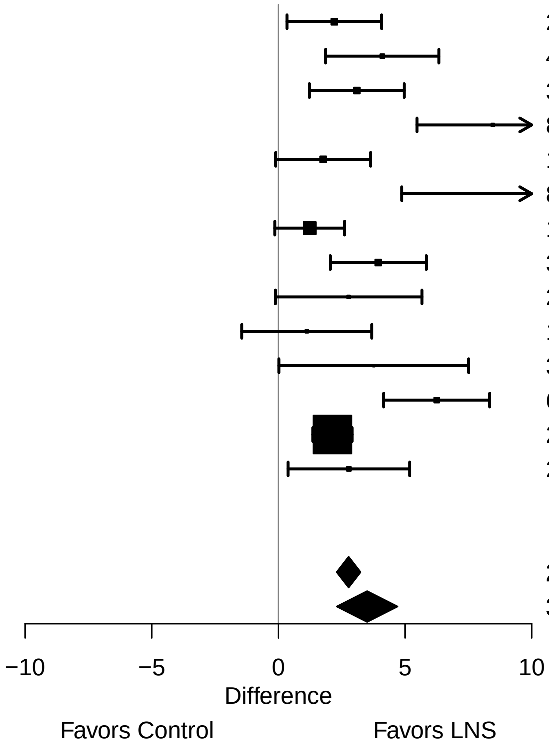

Supplemental figure 3B: Anemia prevalence ratio

Country	Trial	LNS N	Control N	PR (95% CI)	Fixed W	Random W
Bangladesh	JiVitA−4 (35)	457	146	0.83 (0.54, 1.28)	0.01	0.03
Bangladesh	RDNS (36)	549	272	0.66 (0.51, 0.84)	0.02	0.06
Bangladesh	WASH−B (37)	234	186	0.42 (0.24, 0.74)	0.00	0.02
Burkina Faso	iLiNS−Zinc (38)	1957	664	0.87 (0.83, 0.91)	0.39	0.10
Burkina Faso	PROMIS CS (39)	574	581	0.94 (0.85, 1.03)	0.10	0.09
Ghana	GHANA (40)	98	96	0.51 (0.36, 0.72)	0.01	0.04
Ghana	iLiNS−DYAD−G (41)	328	661	0.86 (0.73, 1.01)	0.04	0.08
Kenya	WASH−B (42)	350	300	0.68 (0.57, 0.81)	0.03	0.08
Madagascar	MAHAY (43)	600	588	0.88 (0.78, 1.01)	0.06	0.09
Malawi	iLiNS−DYAD−M (44)	210	432	0.90 (0.76, 1.07)	0.03	0.08
Malawi	iLiNS−DOSE (45)	243	82	0.87 (0.74, 1.02)	0.04	0.08
Mali	PROMIS CS (46)	953	970	0.82 (0.76, 0.88)	0.18	0.10
Zimbabwe	SHINE (HIV−) (47)	1682	1594	0.78 (0.70, 0.86)	0.10	0.09
Zimbabwe	SHINE (HIV+) (48)	306	285	0.65 (0.50, 0.85)	0.01	0.06
	**I² = 0.65, Tau² = 0.02**	**8541**	**6857**			
	**Fixed**			**0.84 (0.81, 0.87)**		
	**Random**			**0.79 (0.72, 0.87)**		

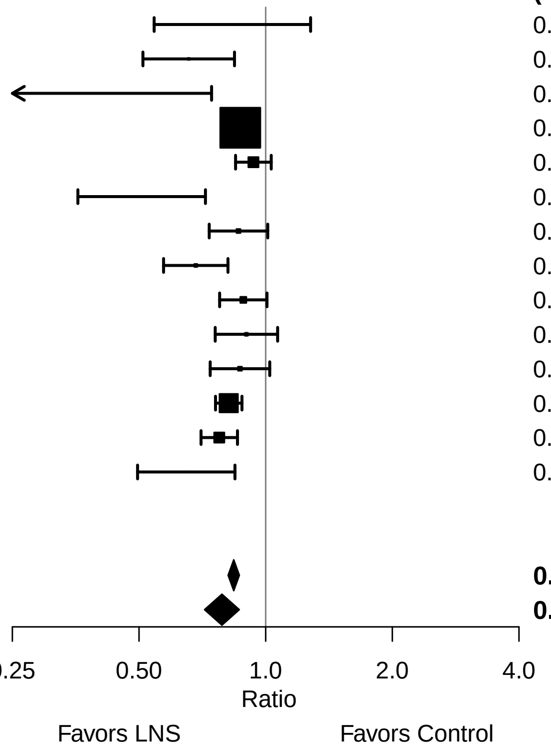

Supplemental figure 3C: Anemia prevalence difference

Country	Trial	LNS N	Control N	PD (95% CI)	Fixed W	Random W
Bangladesh	JiVitA−4 (35)	457	146	−0.03 (−0.09, 0.04)	0.04	0.07
Bangladesh	RDNS (36)	549	272	−0.14 (−0.21, −0.07)	0.04	0.07
Bangladesh	WASH−B (37)	234	186	−0.09 (−0.16, −0.03)	0.05	0.07
Burkina Faso	iLiNS−Zinc (38)	1957	664	−0.12 (−0.15, −0.09)	0.24	0.10
Burkina Faso	PROMIS CS (39)	574	581	−0.05 (−0.10, 0.01)	0.07	0.08
Ghana	GHANA (40)	98	96	−0.29 (−0.42, −0.15)	0.01	0.03
Ghana	iLiNS−DYAD−G (41)	328	661	−0.06 (−0.13, 0.00)	0.04	0.07
Kenya	WASH−B (42)	350	300	−0.15 (−0.23, −0.08)	0.03	0.06
Madagascar	MAHAY (43)	600	588	−0.07 (−0.13, −0.02)	0.06	0.08
Malawi	iLiNS−DYAD−M (44)	210	432	−0.05 (−0.13, 0.03)	0.03	0.06
Malawi	iLiNS−DOSE (45)	243	82	−0.10 (−0.21, 0.02)	0.01	0.04
Mali	PROMIS CS (46)	953	970	−0.16 (−0.19, −0.12)	0.14	0.10
Zimbabwe	SHINE (HIV−) (47)	1682	1594	−0.08 (−0.11, −0.05)	0.19	0.10
Zimbabwe	SHINE (HIV+) (48)	306	285	−0.13 (−0.20, −0.06)	0.03	0.06
	**I² = 0.64, Tau² = 0.00**	**8541**	**6857**			
	**Fixed**			**−0.10 (−0.12, −0.09)**		
	**Random**			**−0.10 (−0.13, −0.07)**		

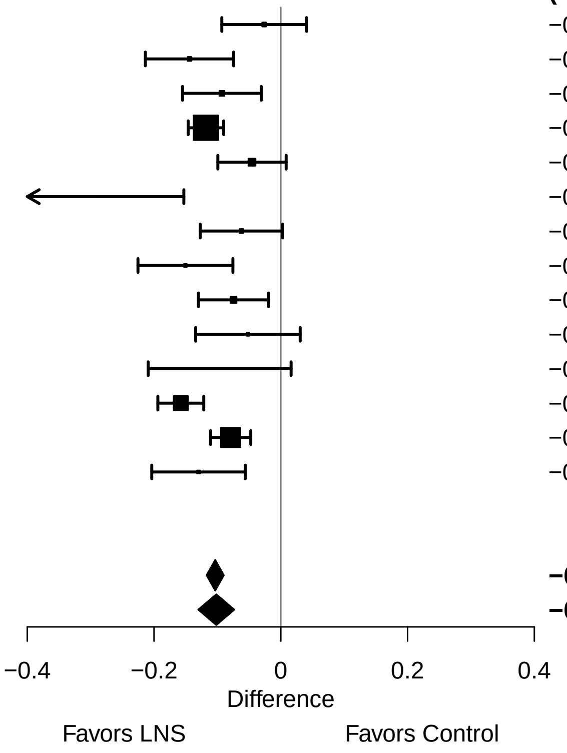

Supplemental figure 3D: Moderate-to-severe anemia prevalence ratio

Country	Trial	LNS N	Control N	PR (95% CI)	Fixed W	Random W
Bangladesh	JiVitA−4 (35)					
Bangladesh	RDNS (36)	549	272	0.54 (0.38, 0.76)	0.03	0.08
Bangladesh	WASH−B (37)					
Burkina Faso	iLiNS−Zinc (38)	1957	664	0.72 (0.65, 0.78)	0.44	0.12
Burkina Faso	PROMIS CS (39)	574	581	0.88 (0.73, 1.07)	0.10	0.11
Ghana	GHANA (40)	98	96	0.32 (0.16, 0.61)	0.01	0.04
Ghana	iLiNS−DYAD−G (41)	328	661	0.84 (0.47, 1.51)	0.01	0.04
Kenya	WASH−B (42)	350	300	0.63 (0.45, 0.88)	0.03	0.08
Madagascar	MAHAY (43)	600	588	0.81 (0.64, 1.03)	0.06	0.10
Malawi	iLiNS−DYAD−M (44)	210	432	1.15 (0.87, 1.52)	0.05	0.09
Malawi	iLiNS−DOSE (45)	243	82	0.72 (0.54, 0.96)	0.04	0.09
Mali	PROMIS CS (46)	953	970	0.63 (0.54, 0.74)	0.15	0.11
Zimbabwe	SHINE (HIV−) (47)	1682	1594	0.67 (0.53, 0.84)	0.07	0.10
Zimbabwe	SHINE (HIV+) (48)	306	285	0.51 (0.30, 0.89)	0.01	0.05
	**I² = 0.64, Tau² = 0.05**	**7850**	**6525**			
	**Fixed**			**0.72 (0.68, 0.76)**		
	**Random**			**0.70 (0.60, 0.82)**		

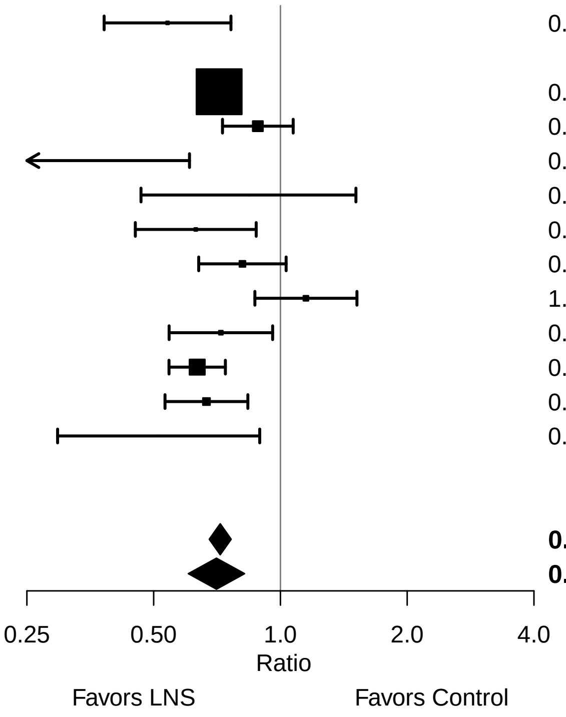

Supplemental figure 3E: Moderate-to-severe anemia prevalence difference

Country	Trial	LNS N	Control N	PD (95% CI)	Fixed W	Random W
Bangladesh	JiVitA−4 (35)					
Bangladesh	RDNS (36)	549	272	−0.08 (−0.13, −0.03)	0.05	0.09
Bangladesh	WASH−B (37)					
Burkina Faso	iLiNS−Zinc (38)	1957	664	−0.21 (−0.25, −0.17)	0.09	0.09
Burkina Faso	PROMIS CS (39)	574	581	−0.04 (−0.10, 0.01)	0.05	0.09
Ghana	GHANA (40)	98	96	−0.22 (−0.33, −0.11)	0.01	0.06
Ghana	iLiNS−DYAD−G (41)	328	661	−0.01 (−0.04, 0.02)	0.17	0.09
Kenya	WASH−B (42)	350	300	−0.08 (−0.14, −0.02)	0.04	0.08
Madagascar	MAHAY (43)	600	588	−0.06 (−0.11, −0.01)	0.05	0.09
Malawi	iLiNS−DYAD−M (44)	210	432	0.04 (−0.04, 0.11)	0.03	0.08
Malawi	iLiNS−DOSE (45)	243	82	−0.13 (−0.25, −0.01)	0.01	0.06
Mali	PROMIS CS (46)	953	970	−0.22 (−0.26, −0.18)	0.07	0.09
Zimbabwe	SHINE (HIV−) (47)	1682	1594	−0.04 (−0.05, −0.02)	0.36	0.09
Zimbabwe	SHINE (HIV+) (48)	306	285	−0.05 (−0.09, −0.01)	0.07	0.09
	**I² = 0.92, Tau² = 0.01**	**7850**	**6525**			
	**Fixed**			**−0.07 (−0.08, −0.06)**		
	**Random**			**−0.09 (−0.14, −0.04)**		

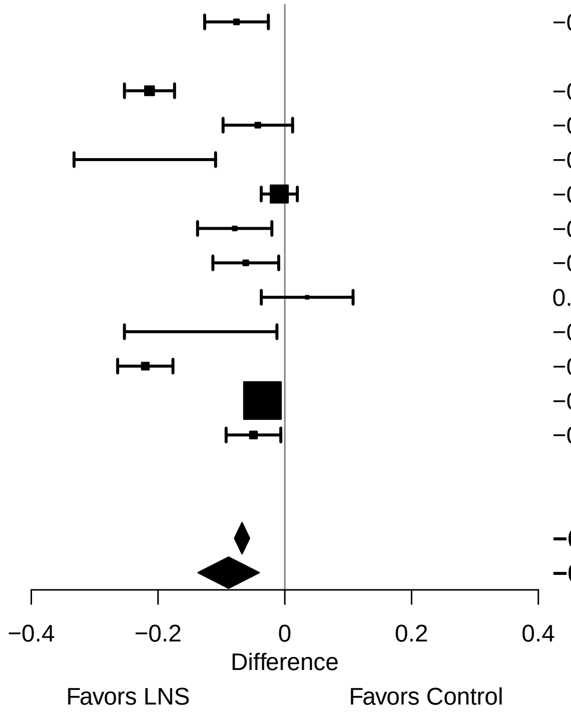

Supplemental figure 3F: Geometric mean ratio of ferritin concentration

Country	Trial	LNS N	Control N	GMR (95% CI)	Fixed W	Random W
Bangladesh	JiVitA−4 (35)	455	144	1.39 (1.17, 1.64)	0.09	0.15
Bangladesh	RDNS (36)	550	272	1.52 (1.40, 1.64)	0.44	0.21
Bangladesh	WASH−B (37)	212	178	1.74 (1.50, 2.01)	0.12	0.16
Burkina Faso	iLiNS−Zinc (38)	315	96	1.55 (1.31, 1.84)	0.09	0.15
Burkina Faso	PROMIS CS (39)					
Ghana	GHANA (40)	83	82	2.52 (1.49, 4.25)	0.01	0.04
Ghana	iLiNS−DYAD−G (41)					
Kenya	WASH−B (42)	298	259	1.69 (1.50, 1.91)	0.19	0.18
Madagascar	MAHAY (43)	83	51	1.29 (1.05, 1.59)	0.06	0.13
Malawi	iLiNS−DYAD−M (44)					
Malawi	iLiNS−DOSE (45)					
Mali	PROMIS CS (46)					
Zimbabwe	SHINE (HIV−) (47)					
Zimbabwe	SHINE (HIV+) (48)					
	**I² = 0.53, Tau² = 0.01**	**1996**	**1082**			
	**Fixed**			**1.56 (1.48, 1.64)**		
	**Random**			**1.56 (1.40, 1.74)**		

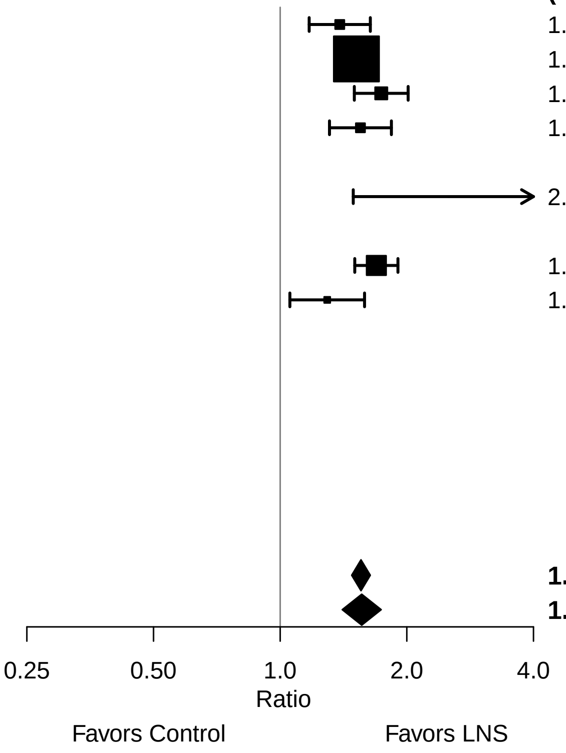

Supplemental figure 3G: Iron deficiency (ferritin < 12 μg/L) prevalence ratio

Country	Trial	LNS N	Control N	PR (95% CI)	Fixed W	Random W
Bangladesh	JiVitA−4 (35)	455	144	0.36 (0.21, 0.59)	0.06	0.11
Bangladesh	RDNS (36)	550	272	0.34 (0.24, 0.48)	0.14	0.16
Bangladesh	WASH−B (37)	212	178	0.24 (0.15, 0.39)	0.07	0.11
Burkina Faso	iLiNS−Zinc (38)	315	96	0.61 (0.49, 0.78)	0.31	0.20
Burkina Faso	PROMIS CS (39)					
Ghana	GHANA (40)	83	82	0.49 (0.33, 0.73)	0.11	0.14
Ghana	iLiNS−DYAD−G (41)					
Kenya	WASH−B (42)	298	259	0.41 (0.32, 0.53)	0.27	0.19
Madagascar	MAHAY (43)	83	51	0.38 (0.21, 0.67)	0.05	0.09
Malawi	iLiNS−DYAD−M (44)					
Malawi	iLiNS−DOSE (45)					
Mali	PROMIS CS (46)					
Zimbabwe	SHINE (HIV−) (47)					
Zimbabwe	SHINE (HIV+) (48)					
	**I² = 0.66, Tau² = 0.05**	**1996**	**1082**			
	**Fixed**			**0.44 (0.39, 0.50)**		
	**Random**			**0.41 (0.33, 0.51)**		

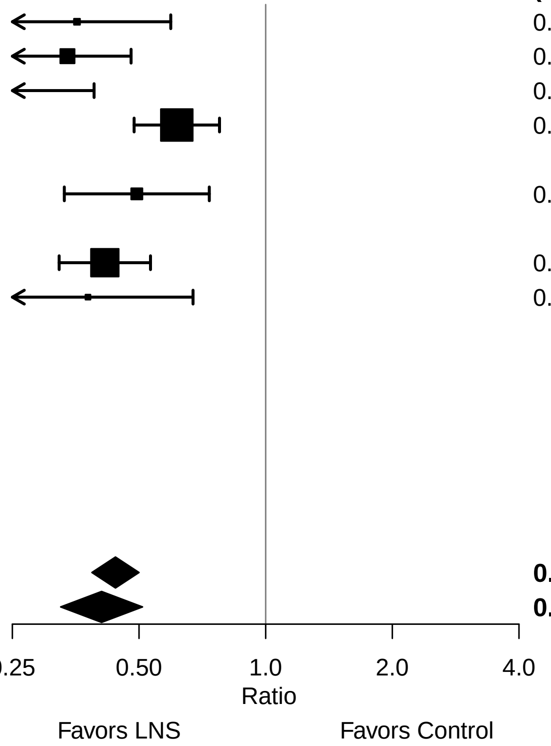

Supplemental figure 3H: Iron deficiency (ferritin < 12 µg/L) prevalence difference

Country	Trial	LNS N	Control N	PD (95% CI)	Fixed W	Random W
Bangladesh	JiVitA−4 (35)	455	144	−0.14 (−0.22, −0.07)	0.19	0.17
Bangladesh	RDNS (36)	550	272	−0.15 (−0.20, −0.09)	0.33	0.19
Bangladesh	WASH−B (37)	212	178	−0.27 (−0.35, −0.19)	0.15	0.16
Burkina Faso	iLiNS−Zinc (38)	315	96	−0.23 (−0.33, −0.12)	0.08	0.13
Burkina Faso	PROMIS CS (39)					
Ghana	GHANA (40)	83	82	−0.28 (−0.43, −0.14)	0.05	0.10
Ghana	iLiNS−DYAD−G (41)					
Kenya	WASH−B (42)	298	259	−0.35 (−0.42, −0.27)	0.16	0.16
Madagascar	MAHAY (43)	83	51	−0.32 (−0.48, −0.15)	0.04	0.09
Malawi	iLiNS−DYAD−M (44)					
Malawi	iLiNS−DOSE (45)					
Mali	PROMIS CS (46)					
Zimbabwe	SHINE (HIV−) (47)					
Zimbabwe	SHINE (HIV+) (48)					
	**I² = 0.76, Tau² = 0.00**	**1996**	**1082**			
	**Fixed**			**−0.22 (−0.25, −0.18)**		
	**Random**			**−0.24 (−0.30, −0.18)**		

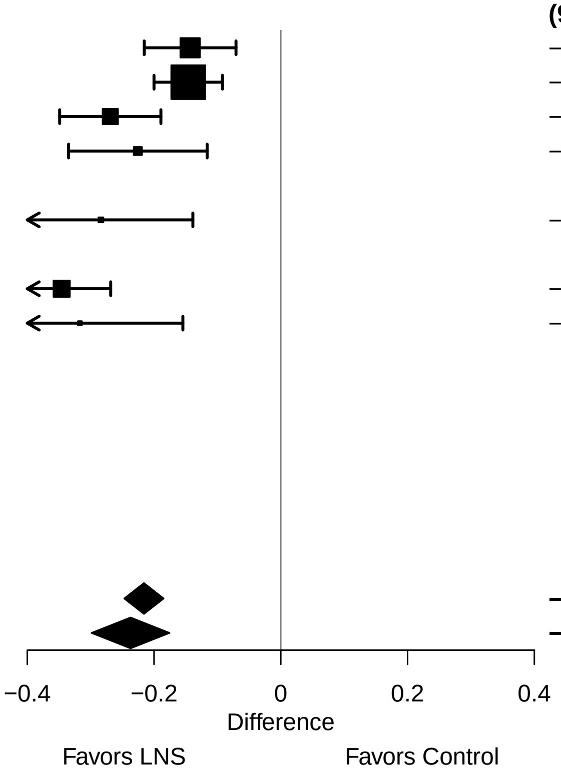

Supplemental figure 3I: Iron deficiency anemia prevalence ratio

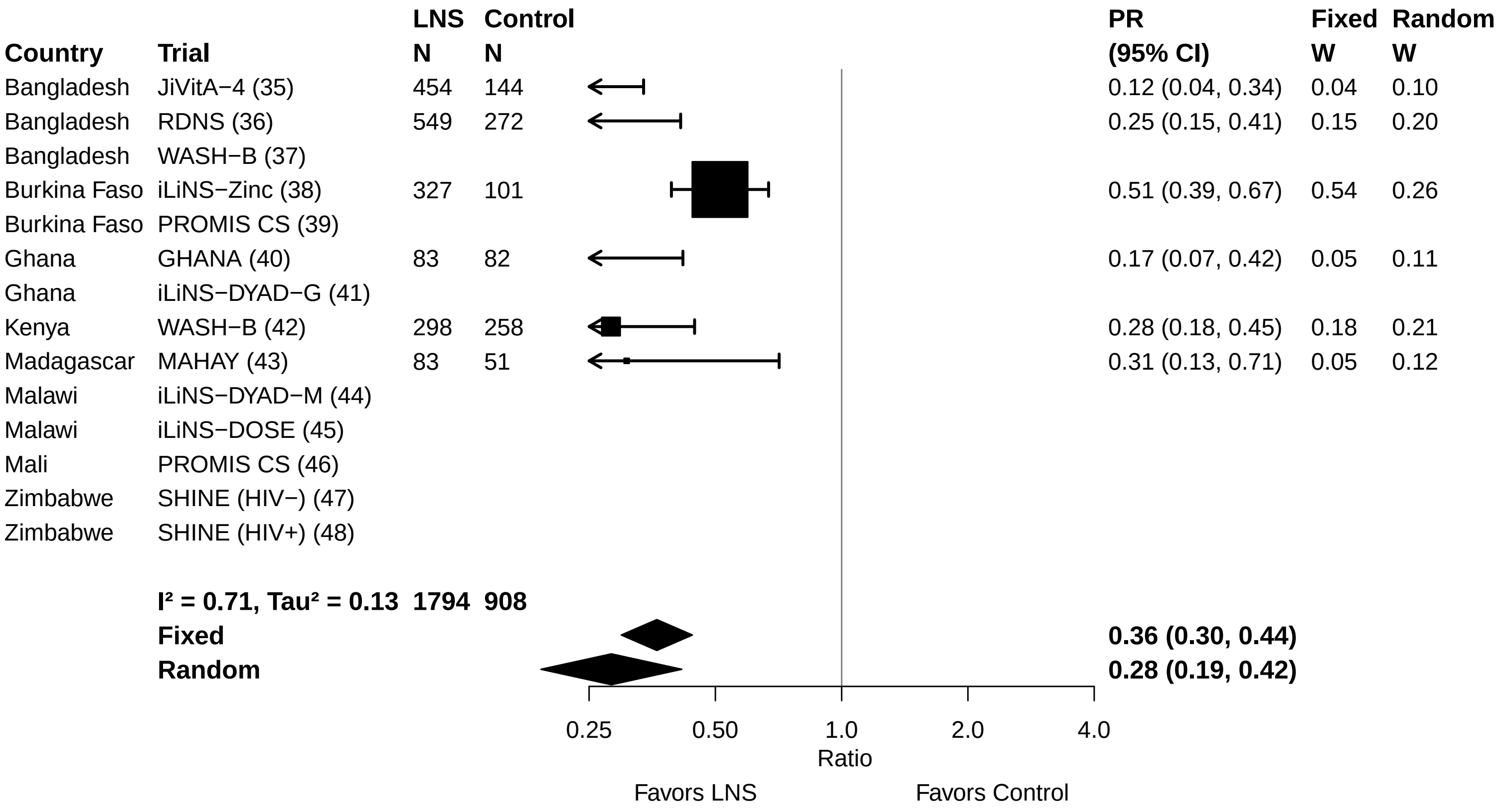

Supplemental figure 3J: Iron deficiency anemia prevalence difference

Country	Trial	LNS N	Control N	PD (95% CI)	Fixed W	Random W
Bangladesh	JiVitA−4 (35)	454	144	−0.08 (−0.13, −0.03)	0.32	0.20
Bangladesh	RDNS (36)	549	272	−0.11 (−0.16, −0.07)	0.36	0.20
Bangladesh	WASH−B (37)					
Burkina Faso	iLiNS−Zinc (38)	327	101	−0.27 (−0.37, −0.16)	0.06	0.15
Burkina Faso	PROMIS CS (39)					
Ghana	GHANA (40)	83	82	−0.29 (−0.41, −0.18)	0.05	0.14
Ghana	iLiNS−DYAD−G (41)					
Kenya	WASH−B (42)	298	258	−0.24 (−0.31, −0.17)	0.17	0.19
Madagascar	MAHAY (43)	83	51	−0.19 (−0.33, −0.05)	0.04	0.12
Malawi	iLiNS−DYAD−M (44)					
Malawi	iLiNS−DOSE (45)					
Mali	PROMIS CS (46)					
Zimbabwe	SHINE (HIV−) (47)					
Zimbabwe	SHINE (HIV+) (48)					
	**I² = 0.83, Tau² = 0.01**	**1794**	**908**			
	**Fixed**			**−0.14 (−0.17, −0.12)**		
	**Random**			**−0.19 (−0.26, −0.12)**		

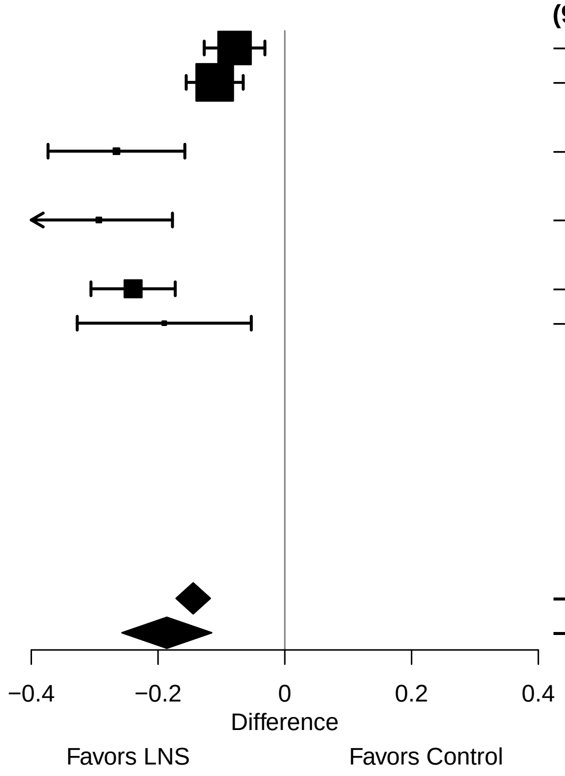

Supplemental figure 3K: Geometric mean ratio of soluble transferrin receptor concentration

Country	Trial	LNS N	Control N	GMR (95% CI)	Fixed W	Random W
Bangladesh	JiVitA−4 (35)					
Bangladesh	RDNS (36)	550	272	0.87 (0.83, 0.92)	0.35	0.26
Bangladesh	WASH−B (37)	212	178	0.82 (0.76, 0.87)	0.23	0.22
Burkina Faso	iLiNS−Zinc (38)	315	96	0.76 (0.68, 0.84)	0.09	0.13
Burkina Faso	PROMIS CS (39)					
Ghana	GHANA (40)	84	82	0.80 (0.72, 0.89)	0.09	0.12
Ghana	iLiNS−DYAD−G (41)					
Kenya	WASH−B (42)	298	259	0.79 (0.74, 0.85)	0.20	0.21
Madagascar	MAHAY (43)	83	51	0.90 (0.77, 1.05)	0.04	0.07
Malawi	iLiNS−DYAD−M (44)					
Malawi	iLiNS−DOSE (45)					
Mali	PROMIS CS (46)					
Zimbabwe	SHINE (HIV−) (47)					
Zimbabwe	SHINE (HIV+) (48)					
	**I^2 = 0.46, Tau^2 = 0.00**	**1542**	**938**			
	**Fixed**			**0.83 (0.80, 0.85)**		
	**Random**			**0.82 (0.78, 0.86)**		

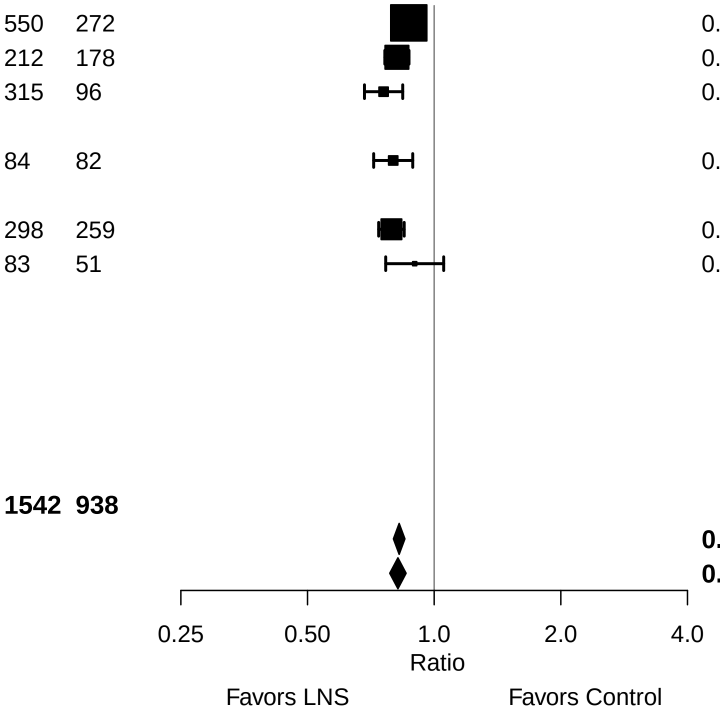

Supplemental figure 3L: Elevated soluble transferrin receptor prevalence ratio

Country	Trial	LNS N	Control N	PR (95% CI)	Fixed W	Random W
Bangladesh	JiVitA−4 (35)					
Bangladesh	RDNS (36)	550	272	0.70 (0.59, 0.84)	0.24	0.19
Bangladesh	WASH−B (37)	212	178	0.31 (0.21, 0.47)	0.05	0.14
Burkina Faso	iLiNS−Zinc (38)	315	96	0.51 (0.38, 0.68)	0.09	0.16
Burkina Faso	PROMIS CS (39)					
Ghana	GHANA (40)	84	82	0.57 (0.41, 0.80)	0.07	0.15
Ghana	iLiNS−DYAD−G (41)					
Kenya	WASH−B (42)	298	259	0.67 (0.59, 0.77)	0.46	0.20
Madagascar	MAHAY (43)	83	51	0.82 (0.60, 1.12)	0.08	0.16
Malawi	iLiNS−DYAD−M (44)					
Malawi	iLiNS−DOSE (45)					
Mali	PROMIS CS (46)					
Zimbabwe	SHINE (HIV−) (47)					
Zimbabwe	SHINE (HIV+) (48)					
	**I² = 0.73, Tau² = 0.08**	**1542**	**938**			
	**Fixed**			**0.64 (0.59, 0.70)**		
	**Random**			**0.59 (0.46, 0.76)**		

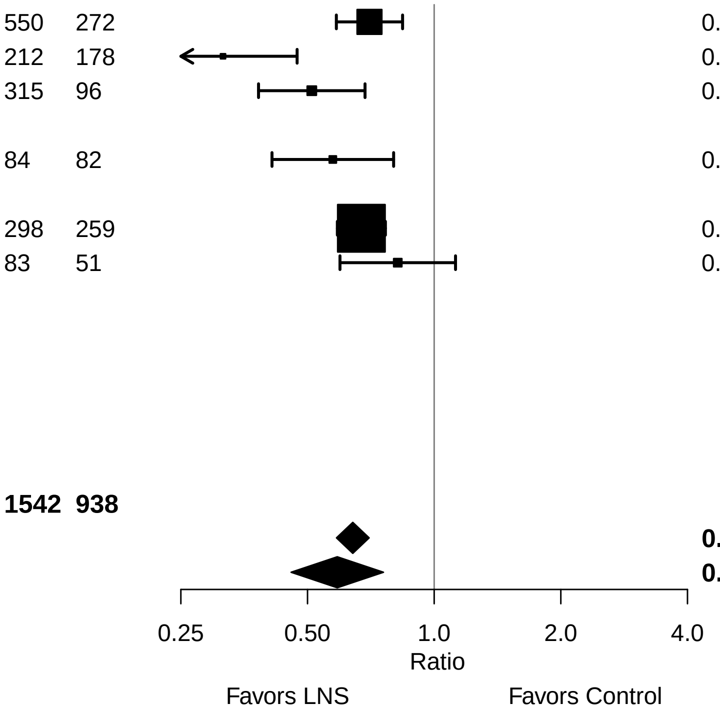

Supplemental figure 3M: Elevated soluble transferrin receptor prevalence difference

Country	Trial	LNS N	Control N	PD (95% CI)	Fixed W	Random W
Bangladesh	JiVitA−4 (35)					
Bangladesh	RDNS (36)	550	272	−0.15 (−0.22, −0.08)	0.28	0.24
Bangladesh	WASH−B (37)	212	178	−0.22 (−0.29, −0.14)	0.23	0.22
Burkina Faso	iLiNS−Zinc (38)	315	96	−0.30 (−0.40, −0.19)	0.13	0.16
Burkina Faso	PROMIS CS (39)					
Ghana	GHANA (40)	84	82	−0.26 (−0.41, −0.12)	0.07	0.09
Ghana	iLiNS−DYAD−G (41)					
Kenya	WASH−B (42)	298	259	−0.24 (−0.32, −0.16)	0.24	0.22
Madagascar	MAHAY (43)	83	51	−0.11 (−0.28, 0.07)	0.05	0.07
Malawi	iLiNS−DYAD−M (44)					
Malawi	iLiNS−DOSE (45)					
Mali	PROMIS CS (46)					
Zimbabwe	SHINE (HIV−) (47)					
Zimbabwe	SHINE (HIV+) (48)					
	**I² = 0.37, Tau² = 0.00**	**1542**	**938**			
	**Fixed**			**−0.21 (−0.25, −0.17)**		
	**Random**			**−0.21 (−0.26, −0.16)**		

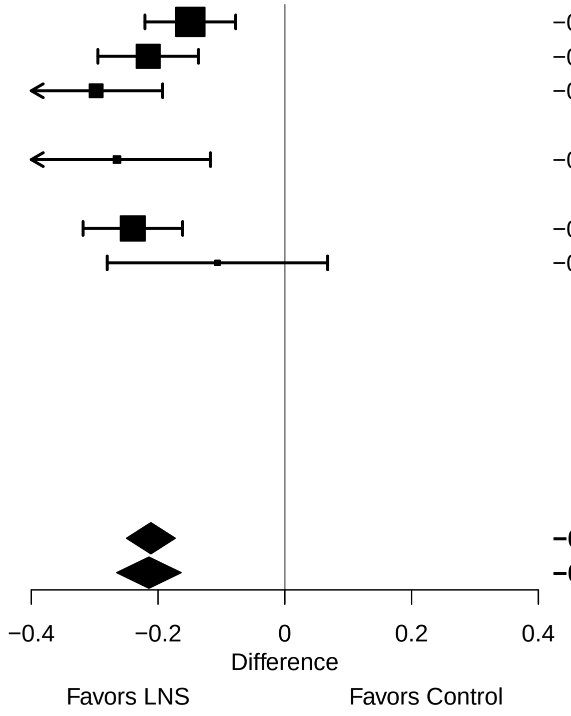

Supplemental figure 3N: Geometric mean ratio of zinc protoporphyrin concentration

Country	Trial	LNS N	Control N	GMR (95% CI)	Fixed W	Random W
Bangladesh	JiVitA−4 (35)					
Bangladesh	RDNS (36)					
Bangladesh	WASH−B (37)					
Burkina Faso	iLiNS−Zinc (38)	312	95	0.64 (0.57, 0.74)	0.21	0.24
Burkina Faso	PROMIS CS (39)					
Ghana	GHANA (40)					
Ghana	iLiNS−DYAD−G (41)	100	202	0.86 (0.76, 0.98)	0.23	0.25
Kenya	WASH−B (42)					
Madagascar	MAHAY (43)					
Malawi	iLiNS−DYAD−M (44)	190	375	0.87 (0.78, 0.96)	0.37	0.27
Malawi	iLiNS−DOSE (45)	181	87	0.78 (0.68, 0.89)	0.19	0.24
Mali	PROMIS CS (46)					
Zimbabwe	SHINE (HIV−) (47)					
Zimbabwe	SHINE (HIV+) (48)					
	**I^2 = 0.79, Tau^2 = 0.02**	**783**	**759**			
	**Fixed**			**0.80 (0.75, 0.85)**		
	**Random**			**0.79 (0.69, 0.90)**		

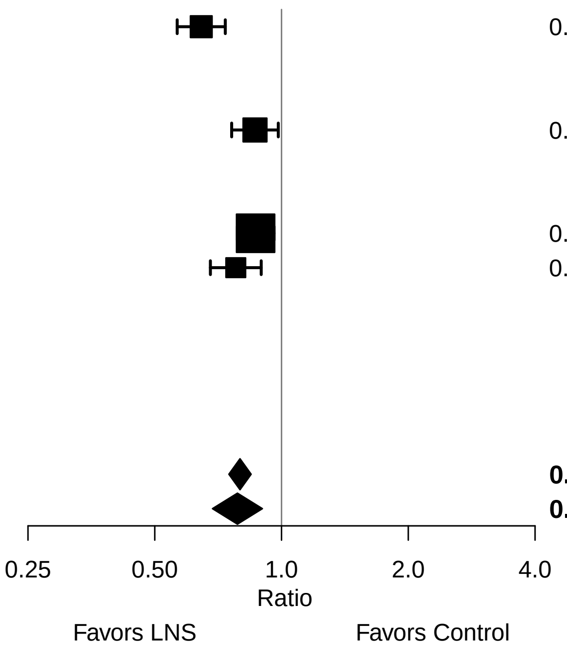

Supplemental figure 3O: Elevated zinc protoporphyrin prevalence ratio

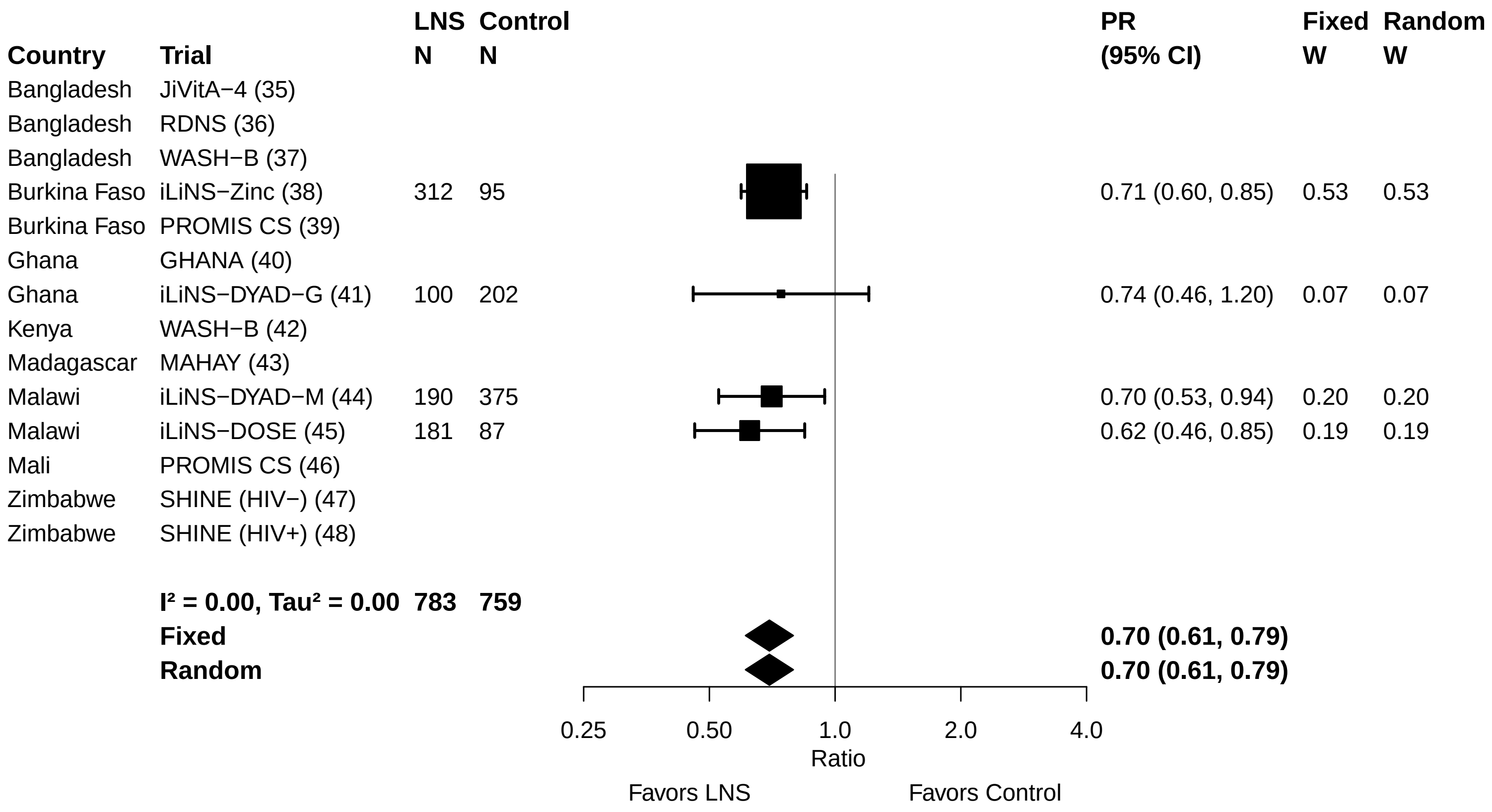

Supplemental figure 3P: Elevated zinc protoporphyrin prevalence difference

Country	Trial	LNS N	Control N	PD (95% CI)	Fixed W	Random W
Bangladesh	JiVitA−4 (35)					
Bangladesh	RDNS (36)					
Bangladesh	WASH−B (37)					
Burkina Faso	iLiNS−Zinc (38)	312	95	−0.21 (−0.31, −0.11)	0.22	0.24
Burkina Faso	PROMIS CS (39)					
Ghana	GHANA (40)					
Ghana	iLiNS−DYAD−G (41)	100	202	−0.06 (−0.16, 0.03)	0.24	0.25
Kenya	WASH−B (42)					
Madagascar	MAHAY (43)					
Malawi	iLiNS−DYAD−M (44)	190	375	−0.10 (−0.18, −0.02)	0.37	0.31
Malawi	iLiNS−DOSE (45)	181	87	−0.17 (−0.28, −0.05)	0.18	0.21
Mali	PROMIS CS (46)					
Zimbabwe	SHINE (HIV−) (47)					
Zimbabwe	SHINE (HIV+) (48)					
	**I² = 0.46, Tau² = 0.00**	**783**	**759**			
	**Fixed**			**−0.13 (−0.17, −0.08)**		
	**Random**			**−0.13 (−0.20, −0.07)**		

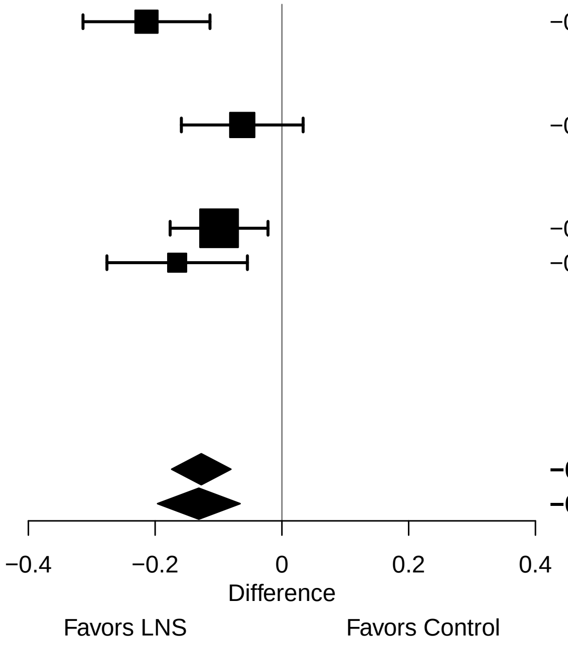

Supplemental figure 3Q: Geometric mean ratio of plasma zinc concentration

Country	Trial	LNS N	Control N	GMR (95% CI)	Fixed W	Random W
Bangladesh	JiVitA−4 (35)	452	144	0.96 (0.91, 1.02)	0.23	0.23
Bangladesh	RDNS (36)					
Bangladesh	WASH−B (37)					
Burkina Faso	iLiNS−Zinc (38)	314	96	1.01 (0.97, 1.04)	0.62	0.62
Burkina Faso	PROMIS CS (39)					
Ghana	GHANA (40)	58	69	1.00 (0.94, 1.08)	0.15	0.15
Ghana	iLiNS−DYAD−G (41)					
Kenya	WASH−B (42)					
Madagascar	MAHAY (43)					
Malawi	iLiNS−DYAD−M (44)					
Malawi	iLiNS−DOSE (45)					
Mali	PROMIS CS (46)					
Zimbabwe	SHINE (HIV−) (47)					
Zimbabwe	SHINE (HIV+) (48)					
	**I² = 0.00, Tau² = 0.00**	**824**	**309**			
	**Fixed**			**1.00 (0.97, 1.02)**		
	**Random**			**1.00 (0.97, 1.02)**		

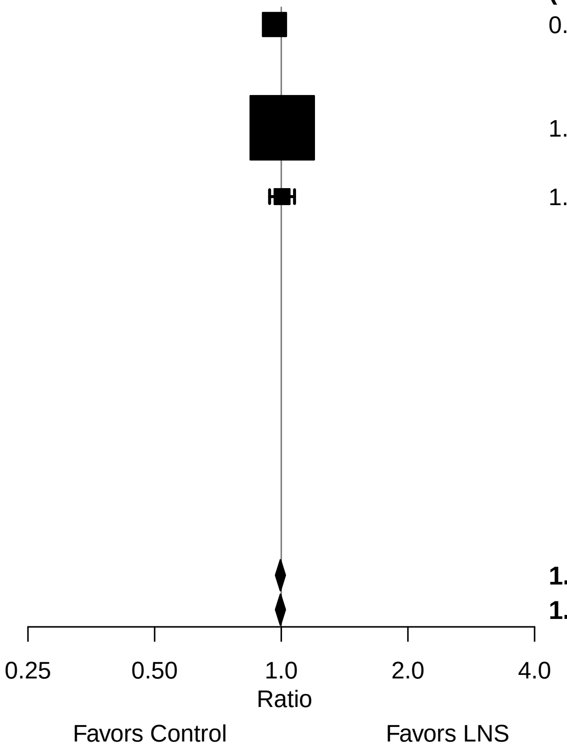

Supplemental figure 3R: Geometric mean ratio of retinol concentration

Country	Trial	LNS N	Control N	GMR (95% CI)	Fixed W	Random W
Bangladesh	JiVitA−4 (35)	434	139	1.08 (1.02, 1.16)	0.35	0.35
Bangladesh	RDNS (36)					
Bangladesh	WASH−B (37)					
Burkina Faso	iLiNS−Zinc (38)					
Burkina Faso	PROMIS CS (39)					
Ghana	GHANA (40)	63	74	1.07 (0.93, 1.23)	0.08	0.08
Ghana	iLiNS−DYAD−G (41)	96	196	1.01 (0.95, 1.07)	0.36	0.35
Kenya	WASH−B (42)					
Madagascar	MAHAY (43)					
Malawi	iLiNS−DYAD−M (44)	71	163	1.00 (0.92, 1.09)	0.21	0.21
Malawi	iLiNS−DOSE (45)					
Mali	PROMIS CS (46)					
Zimbabwe	SHINE (HIV−) (47)					
Zimbabwe	SHINE (HIV+) (48)					
	**I² = 0.08, Tau² = 0.00**	**664**	**572**			
	**Fixed**			**1.04 (1.00, 1.08)**		
	**Random**			**1.04 (1.00, 1.08)**		

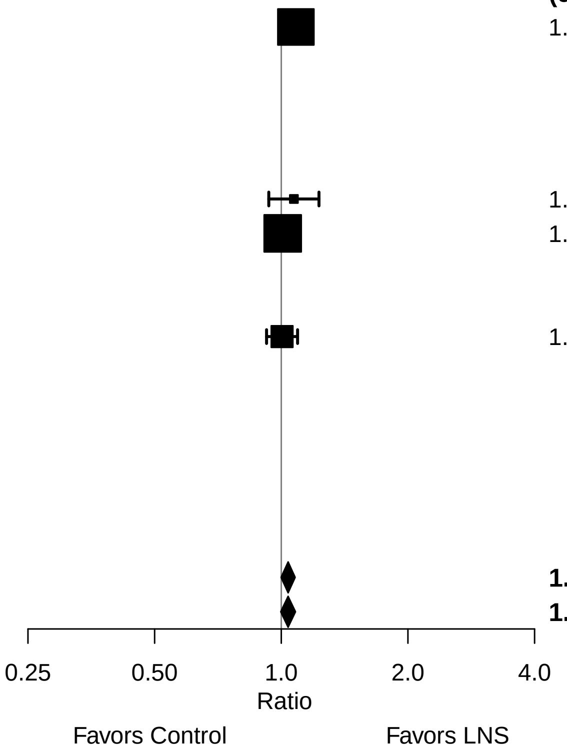

Supplemental figure 3S: Low vitamin A (retinol < 0.70 µmol/L) prevalence ratio

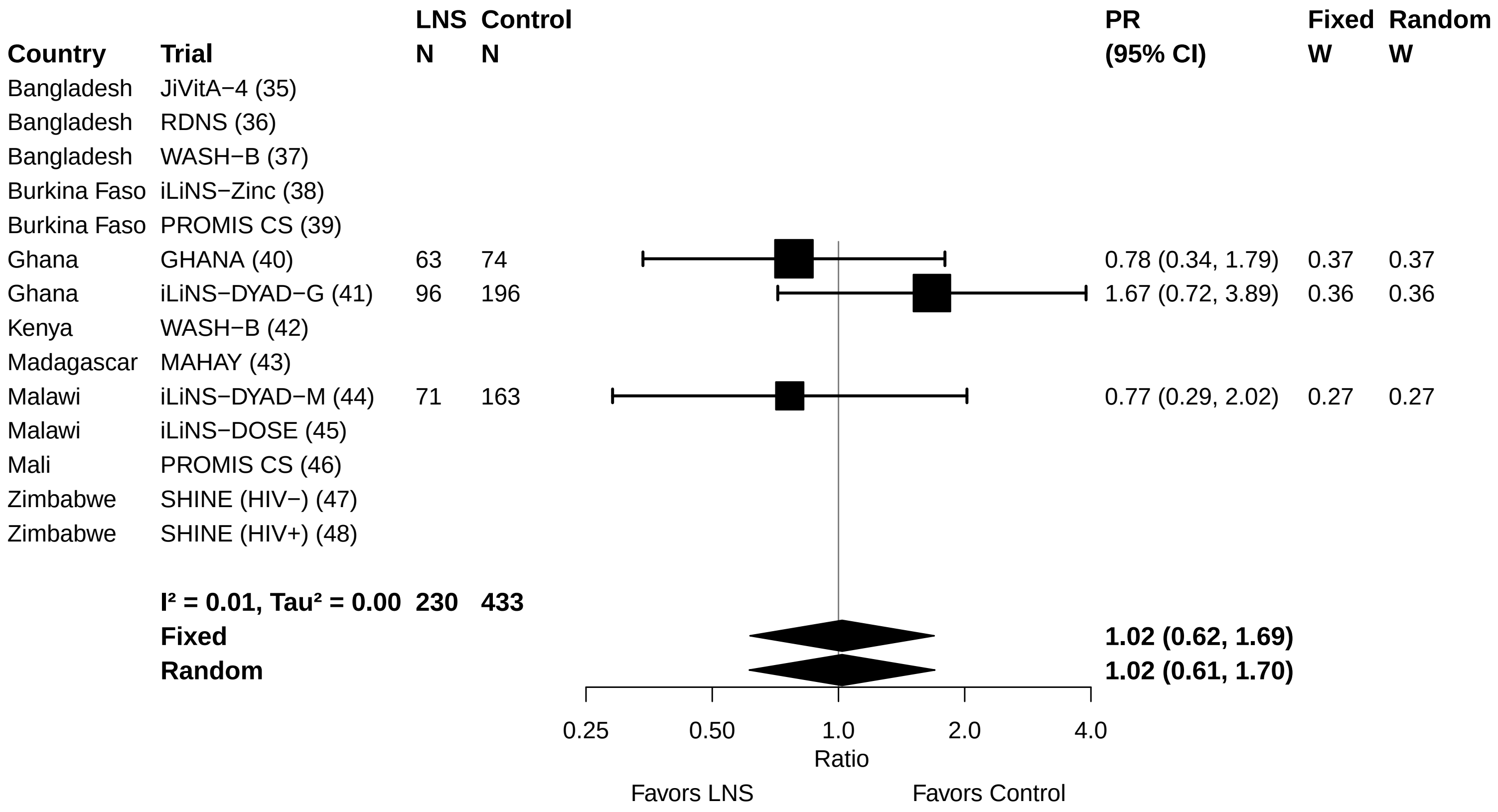

Supplemental figure 3T: Low vitamin A (retinol < 0.70 µmol/L) prevalence difference

Country	Trial	LNS N	Control N	PD (95% CI)	Fixed W	Random W
Bangladesh	JiVitA−4 (35)					
Bangladesh	RDNS (36)					
Bangladesh	WASH−B (37)					
Burkina Faso	iLiNS−Zinc (38)					
Burkina Faso	PROMIS CS (39)					
Ghana	GHANA (40)	63	74	−0.04 (−0.15, 0.08)	0.15	0.15
Ghana	iLiNS−DYAD−G (41)	96	196	0.04 (−0.03, 0.10)	0.47	0.47
Kenya	WASH−B (42)					
Madagascar	MAHAY (43)					
Malawi	iLiNS−DYAD−M (44)	71	163	−0.02 (−0.10, 0.05)	0.38	0.38
Malawi	iLiNS−DOSE (45)					
Mali	PROMIS CS (46)					
Zimbabwe	SHINE (HIV−) (47)					
Zimbabwe	SHINE (HIV+) (48)					
	**I² = 0.00, Tau² = 0.00**	**230**	**433**			
	**Fixed**			**0.00 (−0.04, 0.05)**		
	**Random**			**0.00 (−0.04, 0.05)**		

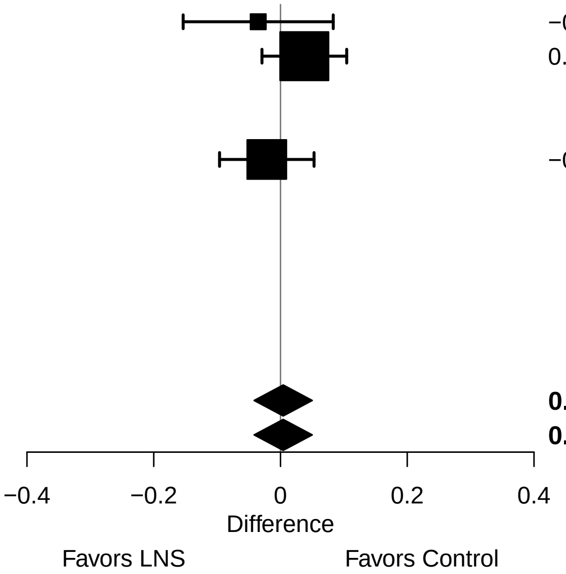

Supplemental figure 3U: Marginal vitamin A (retinol < 1.05 µmol/L) prevalence ratio

Country	Trial	LNS N	Control N	PR (95% CI)	Fixed W	Random W
Bangladesh	JiVitA−4 (35)	434	139	0.74 (0.50, 1.09)	0.13	0.16
Bangladesh	RDNS (36)					
Bangladesh	WASH−B (37)					
Burkina Faso	iLiNS−Zinc (38)					
Burkina Faso	PROMIS CS (39)					
Ghana	GHANA (40)	63	74	1.01 (0.72, 1.43)	0.17	0.20
Ghana	iLiNS−DYAD−G (41)	96	196	0.92 (0.73, 1.15)	0.39	0.33
Kenya	WASH−B (42)					
Madagascar	MAHAY (43)					
Malawi	iLiNS−DYAD−M (44)	71	163	1.19 (0.92, 1.54)	0.31	0.30
Malawi	iLiNS−DOSE (45)					
Mali	PROMIS CS (46)					
Zimbabwe	SHINE (HIV−) (47)					
Zimbabwe	SHINE (HIV+) (48)					
	**I² = 0.35, Tau² = 0.01**	**664**	**572**			
	**Fixed**			**0.98 (0.85, 1.13)**		
	**Random**			**0.98 (0.81, 1.17)**		

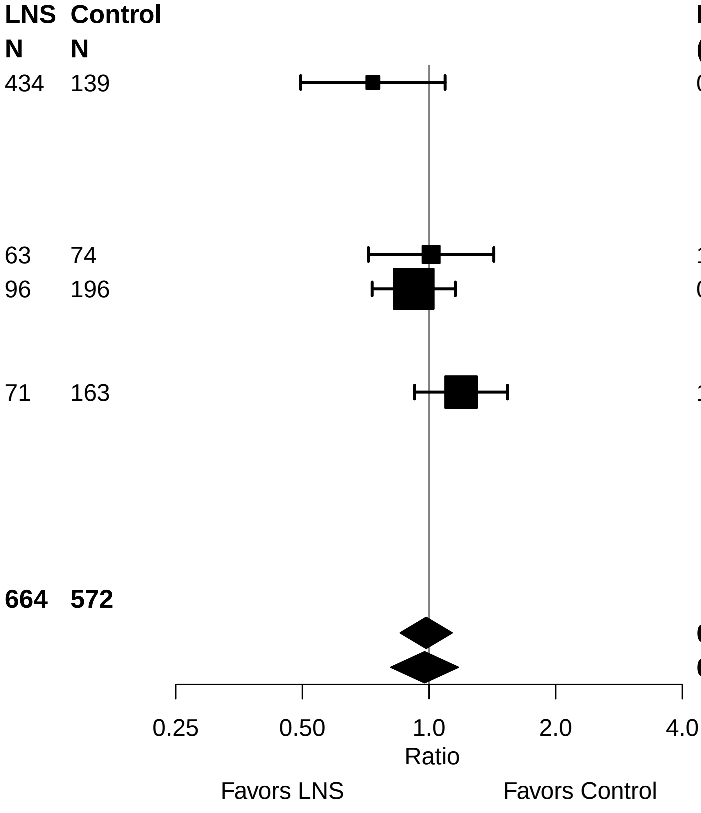

Supplemental figure 3V: Marginal vitamin A (retinol < 1.05 μmol/L) prevalence difference

Country	Trial	LNS N	Control N	PD (95% CI)	Fixed W	Random W
Bangladesh	JiVitA−4 (35)	434	139	−0.05 (−0.13, 0.02)	0.54	0.49
Bangladesh	RDNS (36)					
Bangladesh	WASH−B (37)					
Burkina Faso	iLiNS−Zinc (38)					
Burkina Faso	PROMIS CS (39)					
Ghana	GHANA (40)	63	74	0.01 (−0.16, 0.17)	0.10	0.12
Ghana	iLiNS−DYAD−G (41)	96	196	−0.05 (−0.17, 0.08)	0.20	0.22
Kenya	WASH−B (42)					
Madagascar	MAHAY (43)					
Malawi	iLiNS−DYAD−M (44)	71	163	0.09 (−0.05, 0.23)	0.15	0.17
Malawi	iLiNS−DOSE (45)					
Mali	PROMIS CS (46)					
Zimbabwe	SHINE (HIV−) (47)					
Zimbabwe	SHINE (HIV+) (48)					
	**I² = 0.14, Tau² = 0.00**	**664**	**572**			
	**Fixed**			**−0.02 (−0.08, 0.03)**		
	**Random**			**−0.02 (−0.08, 0.04)**		

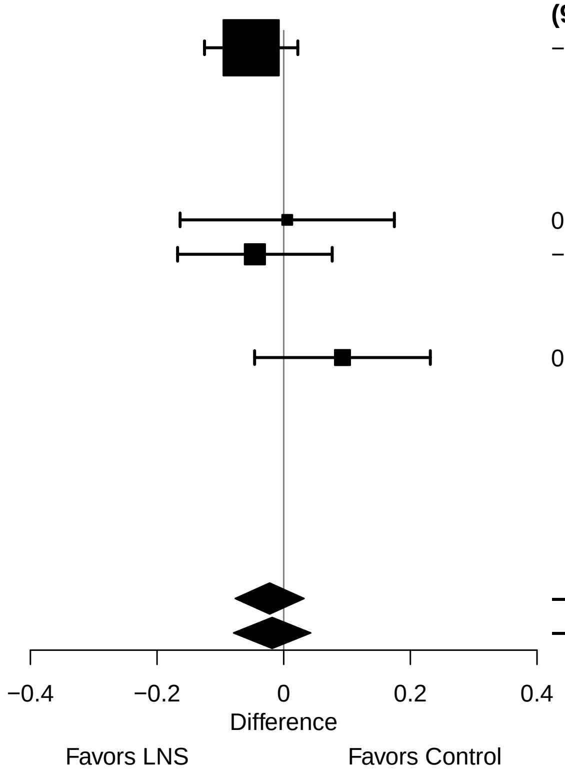

Supplemental figure 3W: Geometric mean ratio of retinol binding protein concentration

Country	Trial	LNS N	Control N	GMR (95% CI)	Fixed W	Random W
Bangladesh	JiVitA−4 (35)					
Bangladesh	RDNS (36)	550	272	1.02 (0.98, 1.07)	0.28	0.24
Bangladesh	WASH−B (37)	212	178	1.04 (0.99, 1.10)	0.22	0.22
Burkina Faso	iLiNS−Zinc (38)	315	96	1.09 (1.03, 1.15)	0.20	0.21
Burkina Faso	PROMIS CS (39)					
Ghana	GHANA (40)					
Ghana	iLiNS−DYAD−G (41)					
Kenya	WASH−B (42)	298	259	1.14 (1.08, 1.19)	0.24	0.22
Madagascar	MAHAY (43)	83	51	1.05 (0.95, 1.15)	0.06	0.11
Malawi	iLiNS−DYAD−M (44)					
Malawi	iLiNS−DOSE (45)					
Mali	PROMIS CS (46)					
Zimbabwe	SHINE (HIV−) (47)					
Zimbabwe	SHINE (HIV+) (48)					
	**I^2 = 0.68, Tau^2 = 0.00**	**1458**	**856**			
	**Fixed**			**1.07 (1.04, 1.09)**		
	**Random**			**1.07 (1.03, 1.11)**		

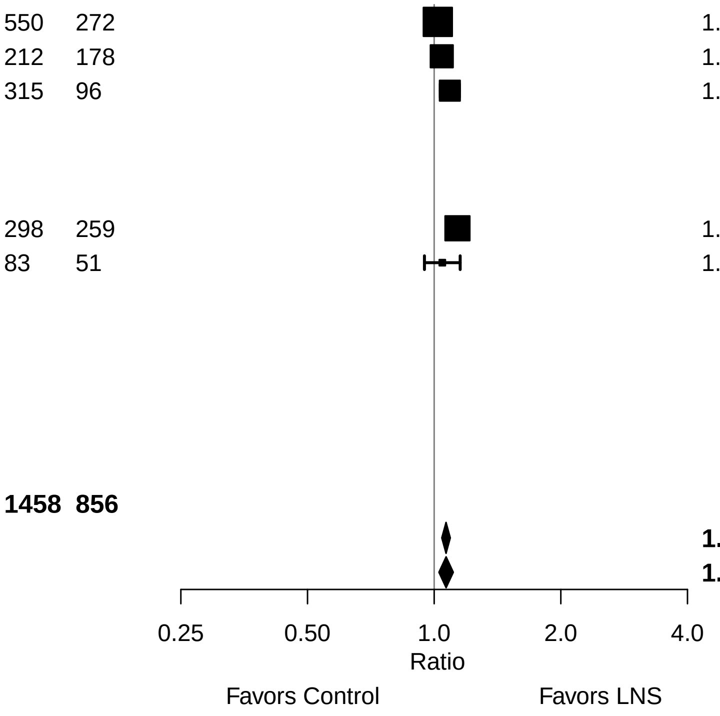

Supplemental figure 3X: Low vitamin A status (RBP < 0.70 µmol/L) prevalence ratio

Country	Trial	LNS N	Control N	PR (95% CI)	Fixed W	Random W
Bangladesh	JiVitA−4 (35)					
Bangladesh	RDNS (36)	550	272	0.55 (0.23, 1.29)	0.30	0.30
Bangladesh	WASH−B (37)					
Burkina Faso	iLiNS−Zinc (38)	315	96	0.47 (0.22, 1.04)	0.36	0.36
Burkina Faso	PROMIS CS (39)					
Ghana	GHANA (40)					
Ghana	iLiNS−DYAD−G (41)					
Kenya	WASH−B (42)	298	259	0.33 (0.15, 0.73)	0.34	0.34
Madagascar	MAHAY (43)					
Malawi	iLiNS−DYAD−M (44)					
Malawi	iLiNS−DOSE (45)					
Mali	PROMIS CS (46)					
Zimbabwe	SHINE (HIV−) (47)					
Zimbabwe	SHINE (HIV+) (48)					
	**I² = 0.00, Tau² = 0.00**	**1163**	**627**			
	**Fixed**			**0.44 (0.27, 0.70)**		
	**Random**			**0.44 (0.27, 0.70)**		

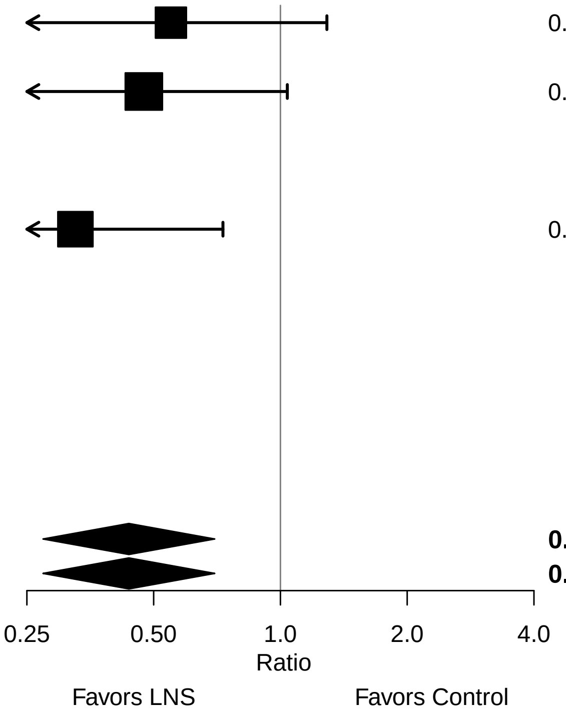

Supplemental figure 3Y: Low vitamin A status (RBP < 0.70 µmol/L) prevalence difference

Country	Trial	LNS N	Control N	PD (95% CI)	Fixed W	Random W
Bangladesh	JiVitA−4 (35)					
Bangladesh	RDNS (36)	550	272	−0.01 (−0.04, 0.01)	0.65	0.50
Bangladesh	WASH−B (37)					
Burkina Faso	iLiNS−Zinc (38)	315	96	−0.04 (−0.10, 0.02)	0.11	0.19
Burkina Faso	PROMIS CS (39)					
Ghana	GHANA (40)					
Ghana	iLiNS−DYAD−G (41)					
Kenya	WASH−B (42)	298	259	−0.06 (−0.10, −0.02)	0.23	0.31
Madagascar	MAHAY (43)					
Malawi	iLiNS−DYAD−M (44)					
Malawi	iLiNS−DOSE (45)					
Mali	PROMIS CS (46)					
Zimbabwe	SHINE (HIV−) (47)					
Zimbabwe	SHINE (HIV+) (48)					
	**I² = 0.52, Tau² = 0.00**	**1163**	**627**			
	**Fixed**			**−0.03 (−0.05, −0.01)**		
	**Random**			**−0.04 (−0.06, −0.01)**		

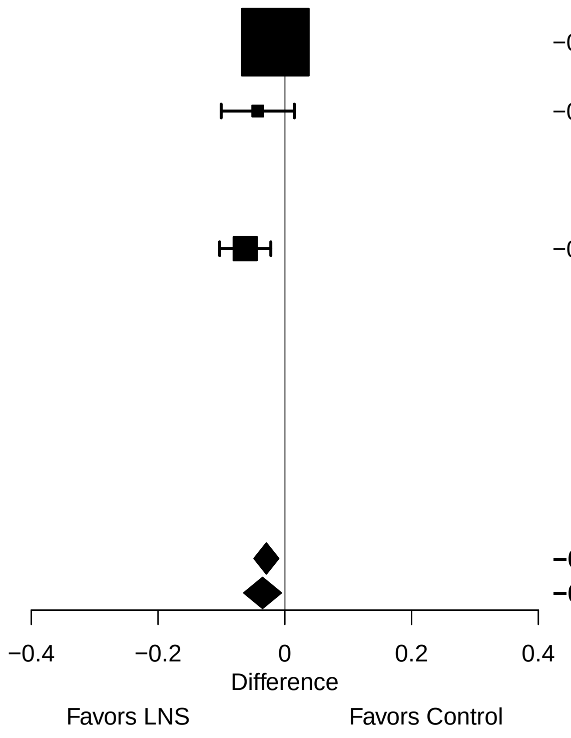

Supplemental figure 3Z: Marginal vitamin A status (RBP < 1.05 µmol/L) prevalence ratio

Country	Trial	LNS N	Control N	PR (95% CI)	Fixed W	Random W
Bangladesh	JiVitA−4 (35)					
Bangladesh	RDNS (36)	550	272	0.87 (0.68, 1.12)	0.17	0.17
Bangladesh	WASH−B (37)	212	178	0.85 (0.65, 1.12)	0.14	0.14
Burkina Faso	iLiNS−Zinc (38)	315	96	0.76 (0.63, 0.93)	0.29	0.29
Burkina Faso	PROMIS CS (39)					
Ghana	GHANA (40)					
Ghana	iLiNS−DYAD−G (41)					
Kenya	WASH−B (42)	298	259	0.72 (0.61, 0.86)	0.35	0.35
Madagascar	MAHAY (43)	83	51	0.83 (0.48, 1.42)	0.04	0.04
Malawi	iLiNS−DYAD−M (44)					
Malawi	iLiNS−DOSE (45)					
Mali	PROMIS CS (46)					
Zimbabwe	SHINE (HIV−) (47)					
Zimbabwe	SHINE (HIV+) (48)					
	**I^2 = 0.00, Tau^2 = 0.00**	**1458**	**856**			
	**Fixed**			**0.78 (0.70, 0.87)**		
	**Random**			**0.78 (0.70, 0.87)**		

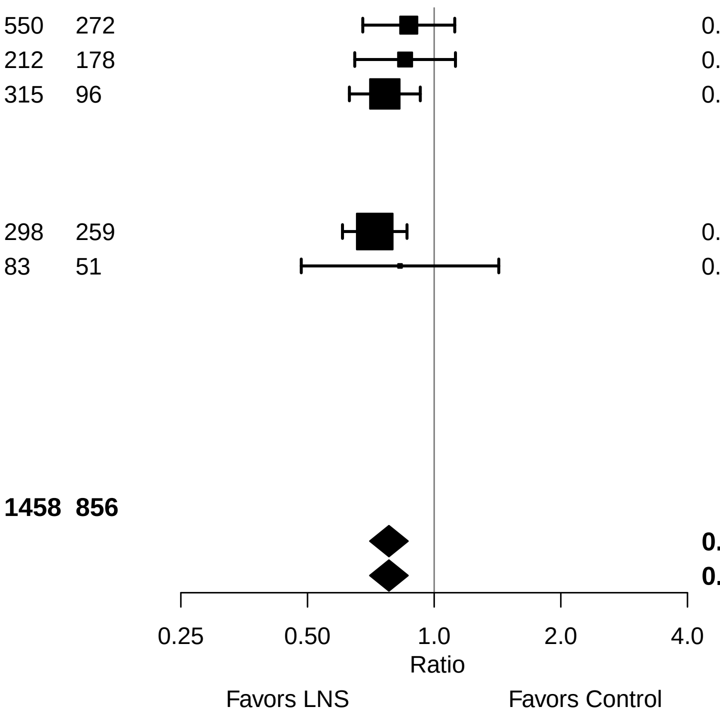

Supplemental figure 3AA: Marginal vitamin A status (RBP < 1.05 µmol/L) prevalence difference

Country	Trial	LNS N	Control N	PD (95% CI)	Fixed W	Random W
Bangladesh	JiVitA−4 (35)					
Bangladesh	RDNS (36)	550	272	−0.04 (−0.10, 0.03)	0.38	0.32
Bangladesh	WASH−B (37)	212	178	−0.05 (−0.15, 0.04)	0.18	0.20
Burkina Faso	iLiNS−Zinc (38)	315	96	−0.11 (−0.22, −0.01)	0.14	0.16
Burkina Faso	PROMIS CS (39)					
Ghana	GHANA (40)					
Ghana	iLiNS−DYAD−G (41)					
Kenya	WASH−B (42)	298	259	−0.17 (−0.25, −0.08)	0.24	0.24
Madagascar	MAHAY (43)	83	51	−0.07 (−0.24, 0.10)	0.06	0.08
Malawi	iLiNS−DYAD−M (44)					
Malawi	iLiNS−DOSE (45)					
Mali	PROMIS CS (46)					
Zimbabwe	SHINE (HIV−) (47)					
Zimbabwe	SHINE (HIV+) (48)					
	**I² = 0.38, Tau² = 0.00**	**1458**	**856**			
	**Fixed**			**−0.08 (−0.12, −0.04)**		
	**Random**			**−0.09 (−0.14, −0.04)**		

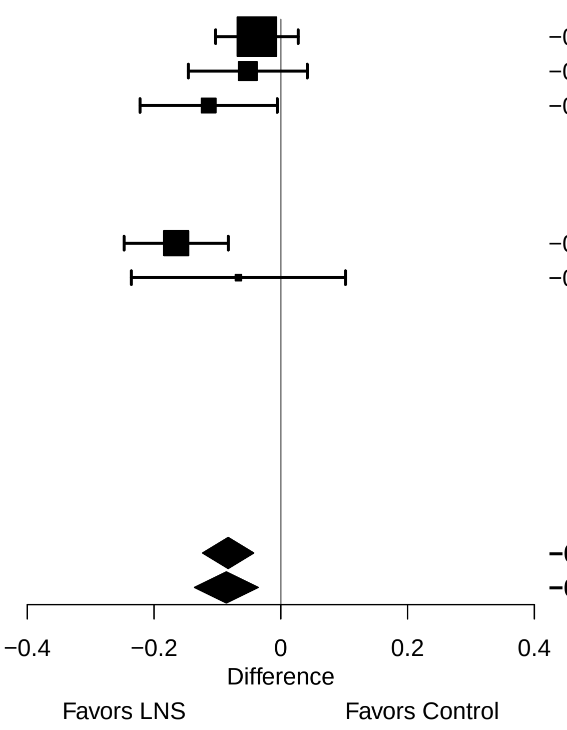

Supplement: nqab276_Supplemental_Files [file nqab276_supplemental_files.zip › 7_ipdb_suppfig3_20210331.pdf]
