# Supplementary material for: Characteristics that modify the effect of small-quantity lipid-based nutrient supplementation on child anemia and micronutrient status: an individual participant data meta-analysis of randomized controlled trials
Source: Am J Clin Nutr. 2021 Sep 29;114(Suppl 1):68S–94S. doi: 10.1093/ajcn/nqab276 (PMC8560313; doi:10.1093/ajcn/nqab276)

Supplemental figure 4: Forest plots for effects of SQ-LNS on biochemical outcomes stratified by study implementation within an existing program vs not program-based

Contents

Supplemental figure 4: Anemia prevalence ratio stratified by Study implementation **2**

This figure shows intervention effects stratified by study implementation. The figure shows the study-level estimates along with the corresponding pooled estimate grouped by category. For dichotomous outcomes analyzed via prevalence ratios, the effect estimate is the prevalence in the LNS group divided by the prevalence in the control group.
The labels on the left y-axis correspond to trial level information. The values on the right indicate the study level effect estimate, confidence interval, and weighting for deriving the pooled estimates.

Supplemental figure 4: Anemia prevalence ratio stratified by Study implementation

Country	Trial	N	N	PR (95% CI)	W
**Study implementation − Program−based**					
Bangladesh	RDNS (36)	549	272	0.66 (0.51, 0.84)	0.12
Burkina Faso	PROMIS CS (39)	574	581	0.94 (0.85, 1.03)	0.19
Madagascar	MAHAY (43)	600	588	0.88 (0.78, 1.01)	0.18
Mali	PROMIS CS (46)	953	970	0.82 (0.76, 0.88)	0.20
Zimbabwe	SHINE (HIV−) (47)	1682	1594	0.78 (0.70, 0.86)	0.19
Zimbabwe	SHINE (HIV+) (48)	306	285	0.65 (0.50, 0.83)	0.12
**$I^2 = 0.68$, $Tau^2 = 0.01$**		**4664**	**4290**	**0.80 (0.72, 0.90)**	
**Study implementation − Not program−based**					
Bangladesh	JiVitA−4 (35)	457	146	0.83 (0.54, 1.28)	0.07
Bangladesh	WASH−B (37)	234	186	0.42 (0.24, 0.74)	0.04
Burkina Faso	iLiNS−Zinc (38)	1957	664	0.87 (0.83, 0.91)	0.20
Ghana	GHANA (40)	98	96	0.51 (0.36, 0.72)	0.08
Ghana	iLiNS−DYAD−G (41)	328	661	0.86 (0.73, 1.01)	0.16
Kenya	WASH−B (42)	350	300	0.68 (0.57, 0.81)	0.15
Malawi	iLiNS−DYAD−M (44)	210	432	0.90 (0.76, 1.07)	0.15
Malawi	iLiNS−DOSE (45)	243	82	0.87 (0.74, 1.02)	0.15
**$I^2 = 0.68$, $Tau^2 = 0.04$**		**3877**	**2567**	**0.76 (0.65, 0.90)**	

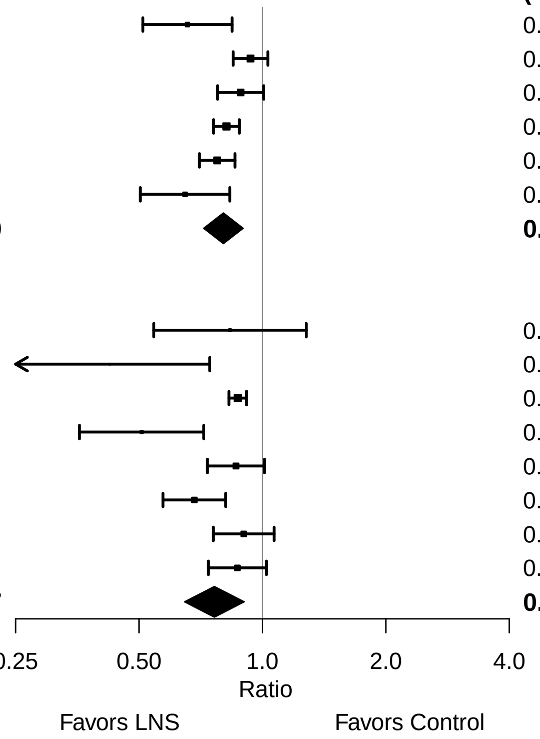

Supplement: nqab276_Supplemental_Files [file nqab276_supplemental_files.zip › 8_ipdb_suppfig4_20210707.pdf]
