# Supplementary material for: Characteristics that modify the effect of small-quantity lipid-based nutrient supplementation on child anemia and micronutrient status: an individual participant data meta-analysis of randomized controlled trials
Source: Am J Clin Nutr. 2021 Sep 29;114(Suppl 1):68S–94S. doi: 10.1093/ajcn/nqab276 (PMC8560313; doi:10.1093/ajcn/nqab276)
Supplement: nqab276_Supplemental_Files [file nqab276_supplemental_files.zip › 10_ipdb_suppfig6_20210401.pdf]

Supplemental figure 6: Forest plots for effects of SQ-LNS on biochemical outcomes stratified by study-level effect modifiers

Contents

Supplemental figure 6A: Mean difference in hemoglobin concentration 9
6A1: Stratified by Geographic region . . . 9
6A2: Stratified by Anemia burden . . . 10
6A3: Stratified by Malaria prevalence . . . 11
6A4: Stratified by Inflammation burden . . . 12
6A5: Stratified by Source water quality . . . 13
6A6: Stratified by Sanitation . . . 14
6A7: Stratified by Supplement duration . . . 15
6A8: Stratified by Iron dose . . . 16
6A9: Stratified by Frequency of contact . . . 17
6A10: Stratified by Average SQ-LNS compliance . . . 18

Supplemental figure 6B: Anemia prevalence ratio 19
6B1: Stratified by Geographic region . . . 19
6B2: Stratified by Anemia burden . . . 20
6B3: Stratified by Malaria prevalence . . . 21
6B4: Stratified by Inflammation burden . . . 22
6B5: Stratified by Source water quality . . . 23
6B6: Stratified by Sanitation . . . 24
6B7: Stratified by Supplement duration . . . 25
6B8: Stratified by Iron dose . . . 26
6B9: Stratified by Frequency of contact . . . 27
6B10: Stratified by Average SQ-LNS compliance . . . 28

Supplemental figure 6C: Anemia prevalence difference 29
6C1: Stratified by Geographic region . . . 29
6C2: Stratified by Anemia burden . . . 30
6C3: Stratified by Malaria prevalence . . . 31
6C4: Stratified by Inflammation burden . . . 32
6C5: Stratified by Source water quality . . . 33

6C6: Stratified by Sanitation . . . 34
6C7: Stratified by Supplement duration . . . 35
6C8: Stratified by Iron dose . . . 36
6C9: Stratified by Frequency of contact . . . 37
6C10: Stratified by Average SQ-LNS compliance . . . 38

Supplemental figure 6D: Moderate-to-severe anemia prevalence ratio 39
6D1: Stratified by Geographic region (insufficient comparisons) . . . 39
6D2: Stratified by Anemia burden . . . 40
6D3: Stratified by Malaria prevalence . . . 41
6D4: Stratified by Inflammation burden . . . 42
6D5: Stratified by Source water quality . . . 43
6D6: Stratified by Sanitation . . . 44
6D7: Stratified by Supplement duration . . . 45
6D8: Stratified by Iron dose . . . 46
6D9: Stratified by Frequency of contact . . . 47
6D10: Stratified by Average SQ-LNS compliance . . . 48

Supplemental figure 6E: Moderate-to-severe anemia prevalence difference 49
6E1: Stratified by Geographic region (insufficient comparisons) . . . 49
6E2: Stratified by Anemia burden . . . 50
6E3: Stratified by Malaria prevalence . . . 51
6E4: Stratified by Inflammation burden . . . 52
6E5: Stratified by Source water quality . . . 53
6E6: Stratified by Sanitation . . . 54
6E7: Stratified by Supplement duration . . . 55
6E8: Stratified by Iron dose . . . 56
6E9: Stratified by Frequency of contact . . . 57
6E10: Stratified by Average SQ-LNS compliance . . . 58

Supplemental figure 6F: Geometric mean ratio of ferritin concentration 59
6F1: Stratified by Geographic region . . . 59
6F2: Stratified by Anemia burden (insufficient comparisons) . . . 60
6F3: Stratified by Malaria prevalence (insufficient comparisons) . . . 61
6F4: Stratified by Inflammation burden (insufficient comparisons) . . . 62
6F5: Stratified by Source water quality . . . 63
6F6: Stratified by Sanitation . . . 64
6F7: Stratified by Supplement duration . . . 65
6F8: Stratified by Iron dose . . . 66
6F9: Stratified by Frequency of contact . . . 67
6F10: Stratified by Average SQ-LNS compliance (insufficient comparisons) . . . 68

Supplemental figure 6G: Iron deficiency (ferritin < 12 µg/L) prevalence ratio **69**
6G1: Stratified by Geographic region . . . 69
6G2: Stratified by Anemia burden (insufficient comparisons) . . . 70
6G3: Stratified by Malaria prevalence (insufficient comparisons) . . . 71
6G4: Stratified by Inflammation burden (insufficient comparisons) . . . 72
6G5: Stratified by Source water quality . . . 73
6G6: Stratified by Sanitation . . . 74
6G7: Stratified by Supplement duration . . . 75
6G8: Stratified by Iron dose . . . 76
6G9: Stratified by Frequency of contact . . . 77
6G10: Stratified by Average SQ-LNS compliance (insufficient comparisons) . . . 78

Supplemental figure 6H: Iron deficiency (ferritin < 12 µg/L) prevalence difference **79**
6H1: Stratified by Geographic region . . . 79
6H2: Stratified by Anemia burden (insufficient comparisons) . . . 80
6H3: Stratified by Malaria prevalence (insufficient comparisons) . . . 81
6H4: Stratified by Inflammation burden (insufficient comparisons) . . . 82
6H5: Stratified by Source water quality . . . 83
6H6: Stratified by Sanitation . . . 84
6H7: Stratified by Supplement duration . . . 85
6H8: Stratified by Iron dose . . . 86
6H9: Stratified by Frequency of contact . . . 87
6H10: Stratified by Average SQ-LNS compliance (insufficient comparisons) . . . 88

Supplemental figure 6I: Iron deficiency anemia prevalence ratio **89**
6I1: Stratified by Geographic region (insufficient comparisons) . . . 89
6I2: Stratified by Anemia burden (insufficient comparisons) . . . 90
6I3: Stratified by Malaria prevalence (insufficient comparisons) . . . 91
6I4: Stratified by Inflammation burden (insufficient comparisons) . . . 92
6I5: Stratified by Source water quality . . . 93
6I6: Stratified by Sanitation . . . 94
6I7: Stratified by Supplement duration (insufficient comparisons) . . . 95
6I8: Stratified by Iron dose . . . 96
6I9: Stratified by Frequency of contact . . . 97
6I10: Stratified by Average SQ-LNS compliance (insufficient comparisons) . . . 98

Supplemental figure 6J: Iron deficiency anemia prevalence difference **99**
6J1: Stratified by Geographic region (insufficient comparisons) . . . 99
6J2: Stratified by Anemia burden (insufficient comparisons) . . . 100
6J3: Stratified by Malaria prevalence (insufficient comparisons) . . . 101
6J4: Stratified by Inflammation burden (insufficient comparisons) . . . 102
6J5: Stratified by Source water quality . . . 103

6J6: Stratified by Sanitation . . . 104
6J7: Stratified by Supplement duration (insufficient comparisons) . . . 105
6J8: Stratified by Iron dose . . . 106
6J9: Stratified by Frequency of contact . . . 107
6J10: Stratified by Average SQ-LNS compliance (insufficient comparisons) . . . 108

Supplemental figure 6K: Geometric mean ratio of soluble transferrin receptor concentration **109**
6K1: Stratified by Geographic region (insufficient comparisons) . . . 109
6K2: Stratified by Anemia burden (insufficient comparisons) . . . 110
6K3: Stratified by Malaria prevalence (insufficient comparisons) . . . 111
6K4: Stratified by Inflammation burden (insufficient comparisons) . . . 112
6K5: Stratified by Source water quality . . . 113
6K6: Stratified by Sanitation . . . 114
6K7: Stratified by Supplement duration . . . 115
6K8: Stratified by Iron dose (insufficient comparisons) . . . 116
6K9: Stratified by Frequency of contact . . . 117
6K10: Stratified by Average SQ-LNS compliance (insufficient comparisons) . . . 118

Supplemental figure 6L: Elevated soluble transferrin receptor prevalence ratio **119**
6L1: Stratified by Geographic region (insufficient comparisons) . . . 119
6L2: Stratified by Anemia burden (insufficient comparisons) . . . 120
6L3: Stratified by Malaria prevalence (insufficient comparisons) . . . 121
6L4: Stratified by Inflammation burden (insufficient comparisons) . . . 122
6L5: Stratified by Source water quality . . . 123
6L6: Stratified by Sanitation . . . 124
6L7: Stratified by Supplement duration . . . 125
6L8: Stratified by Iron dose (insufficient comparisons) . . . 126
6L9: Stratified by Frequency of contact . . . 127
6L10: Stratified by Average SQ-LNS compliance (insufficient comparisons) . . . 128

Supplemental figure 6M: Elevated soluble transferrin receptor prevalence difference **129**
6M1: Stratified by Geographic region (insufficient comparisons) . . . 129
6M2: Stratified by Anemia burden (insufficient comparisons) . . . 130
6M3: Stratified by Malaria prevalence (insufficient comparisons) . . . 131
6M4: Stratified by Inflammation burden (insufficient comparisons) . . . 132
6M5: Stratified by Source water quality . . . 133
6M6: Stratified by Sanitation . . . 134
6M7: Stratified by Supplement duration . . . 135
6M8: Stratified by Iron dose (insufficient comparisons) . . . 136
6M9: Stratified by Frequency of contact . . . 137
6M10: Stratified by Average SQ-LNS compliance (insufficient comparisons) . . . 138

Supplemental figure 6N: Geometric mean ratio of zinc protoporphyrin concentration 139
6N1: Stratified by Geographic region (insufficient comparisons) . . . 139
6N2: Stratified by Anemia burden (insufficient comparisons) . . . 140
6N3: Stratified by Malaria prevalence (insufficient comparisons) . . . 141
6N4: Stratified by Inflammation burden (insufficient comparisons) . . . 142
6N5: Stratified by Source water quality (insufficient comparisons) . . . 143
6N6: Stratified by Sanitation (insufficient comparisons) . . . 144
6N7: Stratified by Supplement duration (insufficient comparisons) . . . 145
6N8: Stratified by Iron dose (insufficient comparisons) . . . 146
6N9: Stratified by Frequency of contact (insufficient comparisons) . . . 147
6N10: Stratified by Average SQ-LNS compliance (insufficient comparisons) . . . 148

Supplemental figure 6O: Elevated zinc protoporphyrin prevalence ratio 149
6O1: Stratified by Geographic region (insufficient comparisons) . . . 149
6O2: Stratified by Anemia burden (insufficient comparisons) . . . 150
6O3: Stratified by Malaria prevalence (insufficient comparisons) . . . 151
6O4: Stratified by Inflammation burden (insufficient comparisons) . . . 152
6O5: Stratified by Source water quality (insufficient comparisons) . . . 153
6O6: Stratified by Sanitation (insufficient comparisons) . . . 154
6O7: Stratified by Supplement duration (insufficient comparisons) . . . 155
6O8: Stratified by Iron dose (insufficient comparisons) . . . 156
6O9: Stratified by Frequency of contact (insufficient comparisons) . . . 157
6O10: Stratified by Average SQ-LNS compliance (insufficient comparisons) . . . 158

Supplemental figure 6P: Elevated zinc protoporphyrin prevalence difference 159
6P1: Stratified by Geographic region (insufficient comparisons) . . . 159
6P2: Stratified by Anemia burden (insufficient comparisons) . . . 160
6P3: Stratified by Malaria prevalence (insufficient comparisons) . . . 161
6P4: Stratified by Inflammation burden (insufficient comparisons) . . . 162
6P5: Stratified by Source water quality (insufficient comparisons) . . . 163
6P6: Stratified by Sanitation (insufficient comparisons) . . . 164
6P7: Stratified by Supplement duration (insufficient comparisons) . . . 165
6P8: Stratified by Iron dose (insufficient comparisons) . . . 166
6P9: Stratified by Frequency of contact (insufficient comparisons) . . . 167
6P10: Stratified by Average SQ-LNS compliance (insufficient comparisons) . . . 168

Supplemental figure 6Q: Geometric mean ratio of plasma zinc concentration 169
6Q1: Stratified by Geographic region (insufficient comparisons) . . . 169
6Q2: Stratified by Anemia burden (insufficient comparisons) . . . 170
6Q3: Stratified by Malaria prevalence (insufficient comparisons) . . . 171
6Q4: Stratified by Inflammation burden (insufficient comparisons) . . . 172
6Q5: Stratified by Source water quality (insufficient comparisons) . . . 173

6Q6: Stratified by Sanitation (insufficient comparisons) . . . 174
6Q7: Stratified by Supplement duration (insufficient comparisons) . . . 175
6Q8: Stratified by Iron dose (insufficient comparisons) . . . 176
6Q9: Stratified by Frequency of contact (insufficient comparisons) . . . 177
6Q10: Stratified by Average SQ-LNS compliance (insufficient comparisons) . . . 178

Supplemental figure 6R: Geometric mean ratio of retinol concentration **179**
6R1: Stratified by Geographic region (insufficient comparisons) . . . 179
6R2: Stratified by Anemia burden (insufficient comparisons) . . . 180
6R3: Stratified by Malaria prevalence (insufficient comparisons) . . . 181
6R4: Stratified by Inflammation burden (insufficient comparisons) . . . 182
6R5: Stratified by Source water quality (insufficient comparisons) . . . 183
6R6: Stratified by Sanitation (insufficient comparisons) . . . 184
6R7: Stratified by Supplement duration (insufficient comparisons) . . . 185
6R8: Stratified by Iron dose (insufficient comparisons) . . . 186
6R9: Stratified by Frequency of contact (insufficient comparisons) . . . 187
6R10: Stratified by Average SQ-LNS compliance (insufficient comparisons) . . . 188

Supplemental figure 6S: Low vitamin A (retinol < 0.70 µmol/L) prevalence ratio **189**
6S1: Stratified by Geographic region (insufficient comparisons) . . . 189
6S2: Stratified by Anemia burden (insufficient comparisons) . . . 190
6S3: Stratified by Malaria prevalence (insufficient comparisons) . . . 191
6S4: Stratified by Inflammation burden (insufficient comparisons) . . . 192
6S5: Stratified by Source water quality (insufficient comparisons) . . . 193
6S6: Stratified by Sanitation (insufficient comparisons) . . . 194
6S7: Stratified by Supplement duration (insufficient comparisons) . . . 195
6S8: Stratified by Iron dose (insufficient comparisons) . . . 196
6S9: Stratified by Frequency of contact (insufficient comparisons) . . . 197
6S10: Stratified by Average SQ-LNS compliance (insufficient comparisons) . . . 198

Supplemental figure 6T: Low vitamin A (retinol < 0.70 µmol/L) prevalence difference **199**
6T1: Stratified by Geographic region (insufficient comparisons) . . . 199
6T2: Stratified by Anemia burden (insufficient comparisons) . . . 200
6T3: Stratified by Malaria prevalence (insufficient comparisons) . . . 201
6T4: Stratified by Inflammation burden (insufficient comparisons) . . . 202
6T5: Stratified by Source water quality (insufficient comparisons) . . . 203
6T6: Stratified by Sanitation (insufficient comparisons) . . . 204
6T7: Stratified by Supplement duration (insufficient comparisons) . . . 205
6T8: Stratified by Iron dose (insufficient comparisons) . . . 206
6T9: Stratified by Frequency of contact (insufficient comparisons) . . . 207
6T10: Stratified by Average SQ-LNS compliance (insufficient comparisons) . . . 208

Supplemental figure 6U: Marginal vitamin A (retinol < 1.05 µmol/L) prevalence ratio **209**
6U1: Stratified by Geographic region (insufficient comparisons) . . . 209
6U2: Stratified by Anemia burden (insufficient comparisons) . . . 210
6U3: Stratified by Malaria prevalence (insufficient comparisons) . . . 211
6U4: Stratified by Inflammation burden (insufficient comparisons) . . . 212
6U5: Stratified by Source water quality (insufficient comparisons) . . . 213
6U6: Stratified by Sanitation (insufficient comparisons) . . . 214
6U7: Stratified by Supplement duration (insufficient comparisons) . . . 215
6U8: Stratified by Iron dose (insufficient comparisons) . . . 216
6U9: Stratified by Frequency of contact (insufficient comparisons) . . . 217
6U10: Stratified by Average SQ-LNS compliance (insufficient comparisons) . . . 218

Supplemental figure 6V: Marginal vitamin A (retinol < 1.05 µmol/L) prevalence difference **219**
6V1: Stratified by Geographic region (insufficient comparisons) . . . 219
6V2: Stratified by Anemia burden (insufficient comparisons) . . . 220
6V3: Stratified by Malaria prevalence (insufficient comparisons) . . . 221
6V4: Stratified by Inflammation burden (insufficient comparisons) . . . 222
6V5: Stratified by Source water quality (insufficient comparisons) . . . 223
6V6: Stratified by Sanitation (insufficient comparisons) . . . 224
6V7: Stratified by Supplement duration (insufficient comparisons) . . . 225
6V8: Stratified by Iron dose (insufficient comparisons) . . . 226
6V9: Stratified by Frequency of contact (insufficient comparisons) . . . 227
6V10: Stratified by Average SQ-LNS compliance (insufficient comparisons) . . . 228

Supplemental figure 6W: Geometric mean ratio of retinol binding protein concentration **229**
6W1: Stratified by Geographic region (insufficient comparisons) . . . 229
6W2: Stratified by Anemia burden (insufficient comparisons) . . . 230
6W3: Stratified by Malaria prevalence (insufficient comparisons) . . . 231
6W4: Stratified by Inflammation burden (insufficient comparisons) . . . 232
6W5: Stratified by Source water quality (insufficient comparisons) . . . 233
6W6: Stratified by Sanitation (insufficient comparisons) . . . 234
6W7: Stratified by Supplement duration (insufficient comparisons) . . . 235
6W8: Stratified by Iron dose (insufficient comparisons) . . . 236
6W9: Stratified by Frequency of contact (insufficient comparisons) . . . 237
6W10: Stratified by Average SQ-LNS compliance (insufficient comparisons) . . . 238

Supplemental figure 6X: Low vitamin A status (RBP < 0.70 µmol/L) prevalence ratio **239**
6X1: Stratified by Geographic region (insufficient comparisons) . . . 239
6X2: Stratified by Anemia burden (insufficient comparisons) . . . 240
6X3: Stratified by Malaria prevalence (insufficient comparisons) . . . 241
6X4: Stratified by Inflammation burden (insufficient comparisons) . . . 242
6X5: Stratified by Source water quality (insufficient comparisons) . . . 243

6X6: Stratified by Sanitation (insufficient comparisons) . . . 244
6X7: Stratified by Supplement duration (insufficient comparisons) . . . 245
6X8: Stratified by Iron dose (insufficient comparisons) . . . 246
6X9: Stratified by Frequency of contact (insufficient comparisons) . . . 247
6X10: Stratified by Average SQ-LNS compliance (insufficient comparisons) . . . 248

Supplemental figure 6Y: Low vitamin A status (RBP < 0.70 µmol/L) prevalence difference **249**
6Y1: Stratified by Geographic region (insufficient comparisons) . . . 249
6Y2: Stratified by Anemia burden (insufficient comparisons) . . . 250
6Y3: Stratified by Malaria prevalence (insufficient comparisons) . . . 251
6Y4: Stratified by Inflammation burden (insufficient comparisons) . . . 252
6Y5: Stratified by Source water quality (insufficient comparisons) . . . 253
6Y6: Stratified by Sanitation (insufficient comparisons) . . . 254
6Y7: Stratified by Supplement duration (insufficient comparisons) . . . 255
6Y8: Stratified by Iron dose (insufficient comparisons) . . . 256
6Y9: Stratified by Frequency of contact (insufficient comparisons) . . . 257
6Y10: Stratified by Average SQ-LNS compliance (insufficient comparisons) . . . 258

Supplemental figure 6Z: Marginal vitamin A status (RBP < 1.05 µmol/L) prevalence ratio **259**
6Z1: Stratified by Geographic region (insufficient comparisons) . . . 259
6Z2: Stratified by Anemia burden (insufficient comparisons) . . . 260
6Z3: Stratified by Malaria prevalence (insufficient comparisons) . . . 261
6Z4: Stratified by Inflammation burden (insufficient comparisons) . . . 262
6Z5: Stratified by Source water quality (insufficient comparisons) . . . 263
6Z6: Stratified by Sanitation (insufficient comparisons) . . . 264
6Z7: Stratified by Supplement duration (insufficient comparisons) . . . 265
6Z8: Stratified by Iron dose (insufficient comparisons) . . . 266
6Z9: Stratified by Frequency of contact (insufficient comparisons) . . . 267
6Z10: Stratified by Average SQ-LNS compliance (insufficient comparisons) . . . 268

Supplemental figure 6AA: Marginal vitamin A status (RBP < 1.05 µmol/L) prevalence difference **269**
6AA1: Stratified by Geographic region (insufficient comparisons) . . . 269
6AA2: Stratified by Anemia burden (insufficient comparisons) . . . 270
6AA3: Stratified by Malaria prevalence (insufficient comparisons) . . . 271
6AA4: Stratified by Inflammation burden (insufficient comparisons) . . . 272
6AA5: Stratified by Source water quality (insufficient comparisons) . . . 273
6AA6: Stratified by Sanitation (insufficient comparisons) . . . 274
6AA7: Stratified by Supplement duration (insufficient comparisons) . . . 275
6AA8: Stratified by Iron dose (insufficient comparisons) . . . 276
6AA9: Stratified by Frequency of contact (insufficient comparisons) . . . 277
6AA10: Stratified by Average SQ-LNS compliance (insufficient comparisons) . . . 278

These figures are forest plots showing the study-level effect modification of intervention effects. Each figure shows the study-level estimates along with the corresponding pooled estimate grouped by study-level effect modifier category. For definitions of effect modifiers, see Box 1 in the main paper.
Individual study estimates were generated from log-binomial regression for dichotomous outcomes and simple linear regression for continuous outcomes; controlling for baseline measure when available and with clustered observations using robust standard errors for cluster-randomized trials. Pooled sub-group estimates were generated using inverse-variance weighting random effects. P-value for the difference was estimated using random effects meta-regression with the indicated effect modifier as the predictor of intervention effect size; stratified pooled estimates are presented for each strata. For continuous outcomes the intervention effect is measured by the difference in mean of the LNS group minus control. For log transformed continuous outcomes, the intervention effect is measured by the ratio of geometric means, the effect estimate is the geometric mean in the LNS group divided by the geometric mean in the control group. For dichotomous outcomes analyzed via prevalence ratios, the effect estimate is the prevalence in the LNS group divided by the prevalence in the control group. For dichotomous outcomes analyzed via prevalence differences, the effect estimate is the prevalence in the LNS group minus the prevalence in the control group. The labels on the left y-axis correspond to trial level information. The values on the right indicate the study level effect estimate, confidence interval, and weighting for deriving the pooled estimates.
Ferritin, sTfR, ZPP, zinc, retinol and RBP concentrations were adjusted for inflammation (i.e., C-reactive protein (CRP) and/or α-1-acid glycoprotein (AGP) concentrations, as available), using a regression correction approach adapted from the Biomarkers Reflecting Inflammation and Nutritional Determinants of Anemia (BRINDA) project (28)
If fewer than three studies contribute to a pooled estimate then the pooled estimate was not generated (e.g. if fewer than 3 studies are categorized into a study level effect modification category), and this is labeled as “insufficient comparisons”. Due to the limited number of studies, we were able to examine only a few, if any, study-level effect modifiers for sTfR, ZPP, plasma zinc, retinol and RBP, and could not examine differences in the effect of SQ-LNS on ferritin by study-level anemia, malaria or inflammation prevalence.
RBP, retinol binding protein.

Supplemental figure 6A: Mean difference in hemoglobin concentration

6A1: Stratified by Geographic region

Geographic region
(p−diff = 0.725)

**Country**	**Trial**	**N**	**N**	**MD (95% CI)**	**W**
**Geographic region − SEAR**					
Bangladesh	JiVitA−4 (35)	457	146	2.21 (0.34, 4.07)	0.34
Bangladesh	RDNS (36)	549	272	4.10 (1.86, 6.34)	0.32
Bangladesh	WASH−B (37)	234	186	3.09 (1.22, 4.97)	0.34
**I² = 0.00, Tau² = 0.00**		**1240**	**604**	**3.03 (1.89, 4.16)**	
**Geographic region − AFR**					
Burkina Faso	iLiNS−Zinc (38)	1957	664	8.47 (5.47, 11.46)	0.08
Burkina Faso	PROMIS CS (39)	574	581	1.77 (−0.11, 3.64)	0.10
Ghana	GHANA (40)	98	96	8.84 (4.87, 12.81)	0.06
Ghana	iLiNS−DYAD−G (41)	328	661	1.24 (−0.14, 2.61)	0.11
Kenya	WASH−B (42)	350	300	3.94 (2.05, 5.84)	0.10
Madagascar	MAHAY (43)	600	588	2.77 (−0.12, 5.67)	0.08
Malawi	iLiNS−DYAD−M (44)	210	432	1.12 (−1.45, 3.69)	0.09
Malawi	iLiNS−DOSE (45)	243	82	3.76 (0.02, 7.51)	0.07
Mali	PROMIS CS (46)	953	970	6.25 (4.16, 8.34)	0.10
Zimbabwe	SHINE (HIV−) (47)	1682	1594	2.13 (1.36, 2.91)	0.12
Zimbabwe	SHINE (HIV+) (48)	306	285	2.78 (0.38, 5.19)	0.09
**I² = 0.78, Tau² = 5.36**		**7301**	**6253**	**3.68 (2.12, 5.23)**	

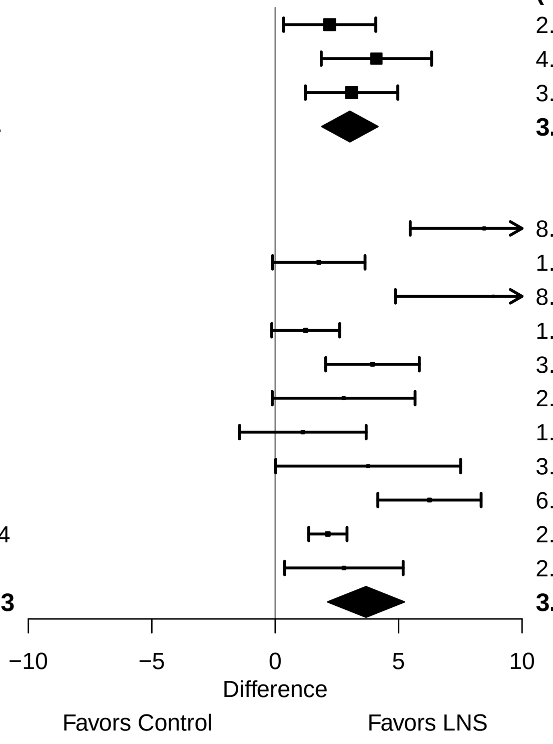

Supplemental figure 6A: Mean difference in hemoglobin concentration

6A2: Stratified by Anemia burden

Anemia burden
(p−diff = 0.077)

Anemia burden − Moderate

**Country**	**Trial**	**N**	**N**	**MD (95% CI)**	**W**
Bangladesh	JiVitA−4 (35)	457	146	2.21 (0.34, 4.07)	0.13
Bangladesh	RDNS (36)	549	272	4.10 (1.86, 6.34)	0.12
Bangladesh	WASH−B (37)	234	186	3.09 (1.22, 4.97)	0.13
Ghana	iLiNS−DYAD−G (41)	328	661	1.24 (−0.14, 2.61)	0.14
Kenya	WASH−B (42)	350	300	3.94 (2.05, 5.84)	0.13
Madagascar	MAHAY (43)	600	588	2.77 (−0.12, 5.67)	0.10
Zimbabwe	SHINE (HIV−) (47)	1682	1594	2.13 (1.36, 2.91)	0.15
Zimbabwe	SHINE (HIV+) (48)	306	285	2.78 (0.38, 5.19)	0.11
**I² = 0.20, Tau² = 0.16**		**4506**	**4032**	**2.49 (1.84, 3.14)**	

Anemia burden − High

**Country**	**Trial**	**N**	**N**	**MD (95% CI)**	**W**
Burkina Faso	iLiNS−Zinc (38)	1957	664	8.47 (5.47, 11.46)	0.16
Burkina Faso	PROMIS CS (39)	574	581	1.77 (−0.11, 3.64)	0.21
Ghana	GHANA (40)	98	96	8.84 (4.87, 12.81)	0.12
Malawi	iLiNS−DYAD−M (44)	210	432	1.12 (−1.45, 3.69)	0.18
Malawi	iLiNS−DOSE (45)	243	82	3.76 (0.02, 7.51)	0.13
Mali	PROMIS CS (46)	953	970	6.25 (4.16, 8.34)	0.20
**I² = 0.83, Tau² = 8.80**		**4035**	**2825**	**4.88 (2.23, 7.53)**	

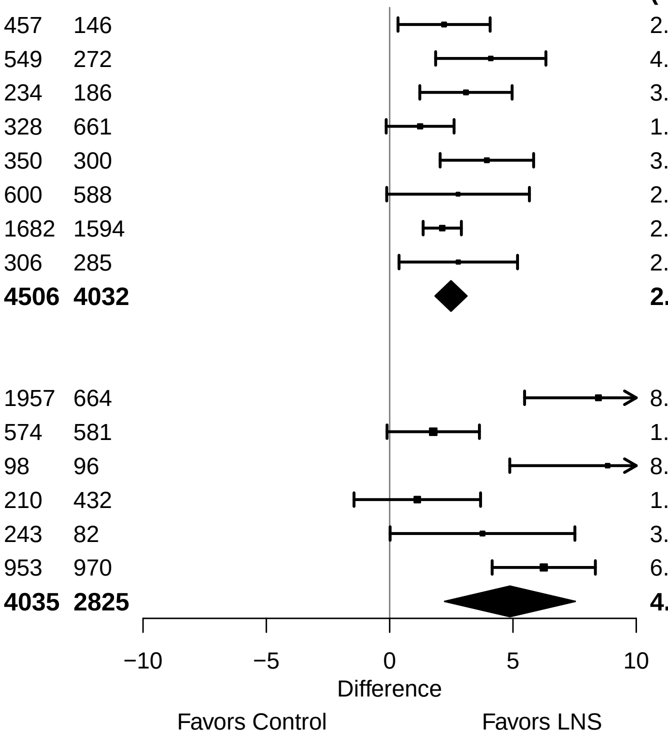

Supplemental figure 6A: Mean difference in hemoglobin concentration

6A3: Stratified by Malaria prevalence

Malaria prevalence
(p−diff = 0.354)

Country	Trial	N	N	MD (95% CI)	W
**Malaria prevalence − Less than 10%**					
Bangladesh	JiVitA−4 (35)	457	146	2.21 (0.34, 4.07)	0.15
Bangladesh	RDNS (36)	549	272	4.10 (1.86, 6.34)	0.14
Bangladesh	WASH−B (37)	234	186	3.09 (1.22, 4.97)	0.15
Kenya	WASH−B (42)	350	300	3.94 (2.05, 5.84)	0.15
Madagascar	MAHAY (43)	600	588	2.77 (−0.12, 5.67)	0.12
Zimbabwe	SHINE (HIV−) (47)	1682	1594	2.13 (1.36, 2.91)	0.17
Zimbabwe	SHINE (HIV+) (48)	306	285	2.78 (0.38, 5.19)	0.13
**I² = 0.00, Tau² = 0.00**		**4178**	**3371**	**2.59 (2.02, 3.17)**	
**Malaria prevalence − At least 10%**					
Burkina Faso	iLiNS−Zinc (38)	1957	664	8.47 (5.47, 11.46)	0.13
Burkina Faso	PROMIS CS (39)	574	581	1.77 (−0.11, 3.64)	0.17
Ghana	GHANA (40)	98	96	8.84 (4.87, 12.81)	0.10
Ghana	iLiNS−DYAD−G (41)	328	661	1.24 (−0.14, 2.61)	0.19
Malawi	iLiNS−DYAD−M (44)	210	432	1.12 (−1.45, 3.69)	0.14
Malawi	iLiNS−DOSE (45)	243	82	3.76 (0.02, 7.51)	0.11
Mali	PROMIS CS (46)	953	970	6.25 (4.16, 8.34)	0.16
**I² = 0.85, Tau² = 9.15**		**4363**	**3486**	**4.29 (1.82, 6.75)**	

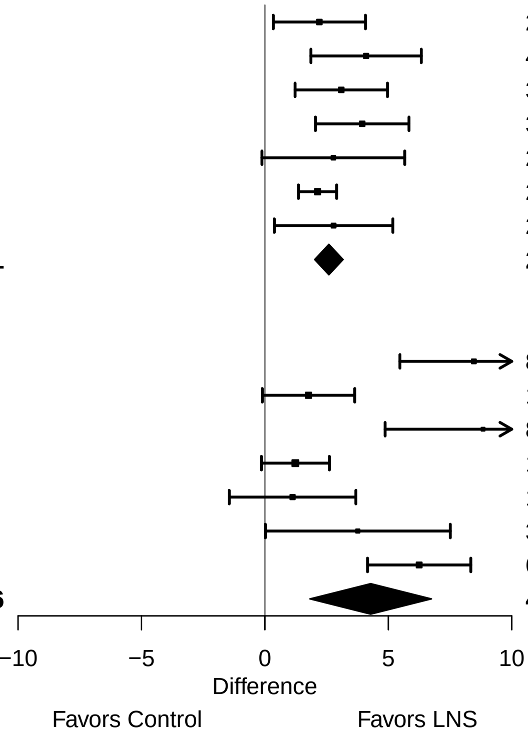

Supplemental figure 6A: Mean difference in hemoglobin concentration

6A4: Stratified by Inflammation burden

Inflammation burden
(p−diff = 0.676)

Country	Trial	N	N	MD (95% CI)	W
**Inflammation burden − Low**					
Bangladesh	RDNS (36)	549	272	4.10 (1.86, 6.34)	0.12
Bangladesh	WASH−B (37)	234	186	3.09 (1.22, 4.97)	0.13
Ghana	GHANA (40)	98	96	8.84 (4.87, 12.81)	0.08
Ghana	iLiNS−DYAD−G (41)	328	661	1.24 (−0.14, 2.61)	0.15
Kenya	WASH−B (42)	350	300	3.94 (2.05, 5.84)	0.13
Madagascar	MAHAY (43)	600	588	2.77 (−0.12, 5.67)	0.11
Zimbabwe	SHINE (HIV−) (47)	1682	1594	2.13 (1.36, 2.91)	0.16
Zimbabwe	SHINE (HIV+) (48)	306	285	2.78 (0.38, 5.19)	0.12
**I² = 0.62, Tau² = 2.50**		**4147**	**3982**	**3.21 (1.88, 4.54)**	
**Inflammation burden − High**					
Bangladesh	JiVitA−4 (35)	457	146	2.21 (0.34, 4.07)	0.19
Burkina Faso	iLiNS−Zinc (38)	1957	664	8.47 (5.47, 11.46)	0.15
Burkina Faso	PROMIS CS (39)	574	581	1.77 (−0.11, 3.64)	0.19
Malawi	iLiNS−DYAD−M (44)	210	432	1.12 (−1.45, 3.69)	0.16
Malawi	iLiNS−DOSE (45)	243	82	3.76 (0.02, 7.51)	0.12
Mali	PROMIS CS (46)	953	970	6.25 (4.16, 8.34)	0.18
**I² = 0.81, Tau² = 6.54**		**4394**	**2875**	**3.84 (1.55, 6.13)**	

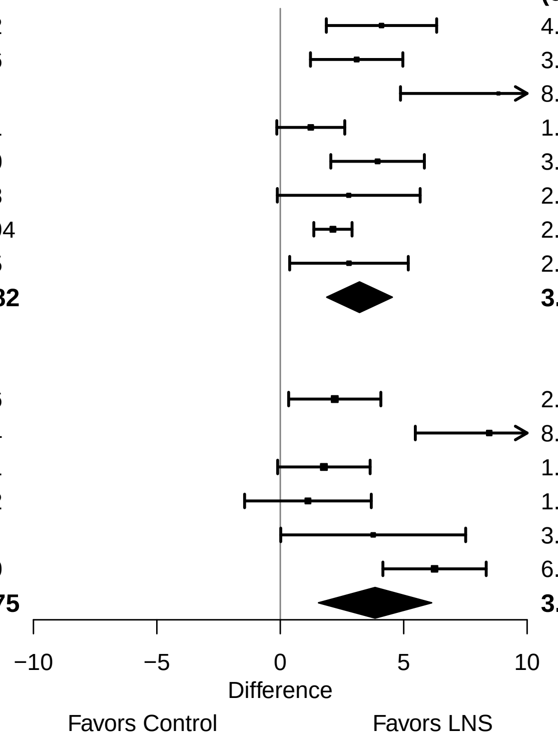

Supplemental figure 6A: Mean difference in hemoglobin concentration

6A5: Stratified by Source water quality

Source water quality
(p−diff = 0.711)

Country	Trial	N	N	MD (95% CI)	W
**Source water quality − Improved**					
Bangladesh	JiVitA−4 (35)	457	146	2.21 (0.34, 4.07)	0.17
Bangladesh	RDNS (36)	549	272	4.10 (1.86, 6.34)	0.16
Bangladesh	WASH−B (37)	105	60	3.25 (−0.27, 6.78)	0.12
Ghana	GHANA (40)	98	96	8.84 (4.87, 12.81)	0.10
Ghana	iLiNS−DYAD−G (41)	328	661	1.24 (−0.14, 2.61)	0.19
Malawi	iLiNS−DYAD−M (44)	210	432	1.12 (−1.45, 3.69)	0.15
Malawi	iLiNS−DOSE (45)	243	82	3.76 (0.02, 7.51)	0.11
**I² = 0.64, Tau² = 3.93**		**1990**	**1749**	**3.15 (1.36, 4.94)**	
**Source water quality − Unimproved**					
Burkina Faso	iLiNS−Zinc (38)	1957	664	8.47 (5.47, 11.46)	0.12
Burkina Faso	PROMIS CS (39)	574	581	1.77 (−0.11, 3.64)	0.16
Kenya	WASH−B (42)	196	162	3.20 (0.41, 6.00)	0.13
Madagascar	MAHAY (43)	600	588	2.77 (−0.12, 5.67)	0.13
Mali	PROMIS CS (46)	953	970	6.25 (4.16, 8.34)	0.15
Zimbabwe	SHINE (HIV−) (47)	819	795	2.12 (0.93, 3.31)	0.18
Zimbabwe	SHINE (HIV+) (48)	133	126	1.52 (−1.46, 4.49)	0.13
**I² = 0.78, Tau² = 5.06**		**5232**	**3886**	**3.65 (1.75, 5.55)**	

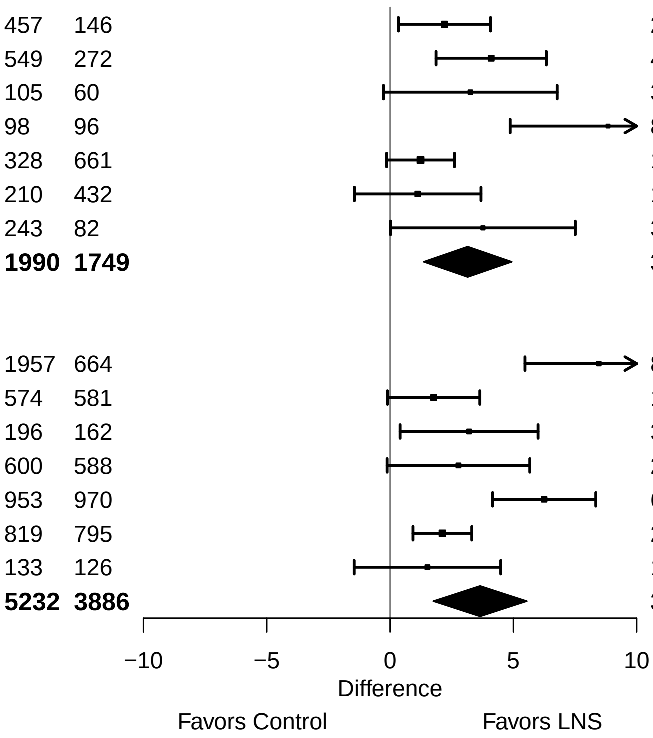

Supplemental figure 6A: Mean difference in hemoglobin concentration

6A6: Stratified by Sanitation

Sanitation
(p−diff = 0.723)

Country	Trial	N	N	MD (95% CI)	W
**Sanitation − Improved**					
Bangladesh	JiVitA−4 (35)	457	146	2.21 (0.34, 4.07)	0.16
Bangladesh	RDNS (36)	549	272	4.10 (1.86, 6.34)	0.15
Bangladesh	WASH−B (37)	105	60	3.25 (−0.27, 6.78)	0.11
Burkina Faso	PROMIS CS (39)	574	581	1.77 (−0.11, 3.64)	0.16
Ghana	GHANA (40)	98	96	8.84 (4.87, 12.81)	0.10
Ghana	iLiNS−DYAD−G (41)	328	661	1.24 (−0.14, 2.61)	0.17
Mali	PROMIS CS (46)	953	970	6.25 (4.16, 8.34)	0.15
**I² = 0.78, Tau² = 5.14**		**3064**	**2786**	**3.67 (1.76, 5.58)**	
**Sanitation − Unimproved**					
Burkina Faso	iLiNS−Zinc (38)	1957	664	8.47 (5.47, 11.46)	0.13
Kenya	WASH−B (42)	196	162	3.20 (0.41, 6.00)	0.14
Madagascar	MAHAY (43)	600	588	2.77 (−0.12, 5.67)	0.14
Malawi	iLiNS−DYAD−M (44)	210	432	1.12 (−1.45, 3.69)	0.15
Malawi	iLiNS−DOSE (45)	243	82	3.76 (0.02, 7.51)	0.11
Zimbabwe	SHINE (HIV−) (47)	819	795	2.12 (0.93, 3.31)	0.19
Zimbabwe	SHINE (HIV+) (48)	133	126	1.52 (−1.46, 4.49)	0.13
**I² = 0.66, Tau² = 3.92**		**4158**	**2849**	**3.16 (1.37, 4.96)**	

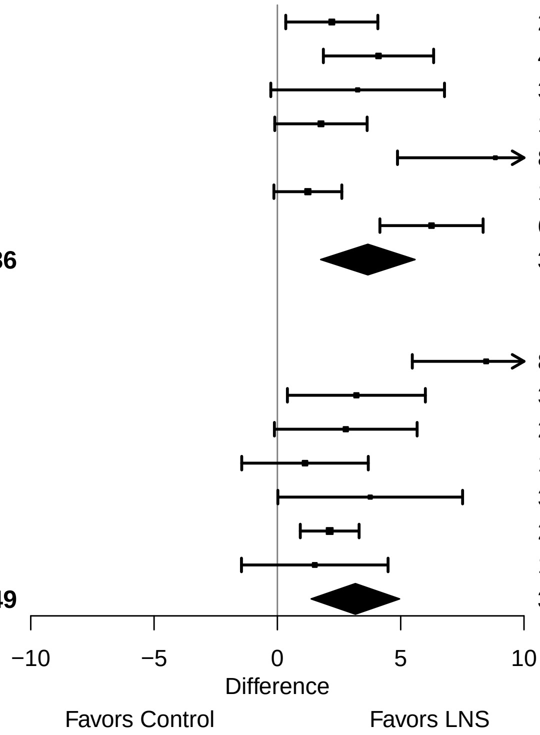

Supplemental figure 6A: Mean difference in hemoglobin concentration

6A7: Stratified by Supplement duration

Supplement duration
(p−diff = 0.304)

Country	Trial	N	N	MD (95% CI)	W
**Supplement duration − 12m or less**					
Bangladesh	JiVitA−4 (35)	457	146	2.21 (0.34, 4.07)	0.11
Burkina Faso	iLiNS−Zinc (38)	1957	664	8.47 (5.47, 11.46)	0.09
Burkina Faso	PROMIS CS (39)	574	581	1.77 (−0.11, 3.64)	0.11
Ghana	GHANA (40)	98	96	8.84 (4.87, 12.81)	0.07
Ghana	iLiNS−DYAD−G (41)	328	661	1.24 (−0.14, 2.61)	0.12
Madagascar	MAHAY (43)	600	588	2.77 (−0.12, 5.67)	0.09
Malawi	iLiNS−DYAD−M (44)	210	432	1.12 (−1.45, 3.69)	0.10
Malawi	iLiNS−DOSE (45)	243	82	3.76 (0.02, 7.51)	0.07
Zimbabwe	SHINE (HIV−) (47)	1682	1594	2.13 (1.36, 2.91)	0.13
Zimbabwe	SHINE (HIV+) (48)	306	285	2.78 (0.38, 5.19)	0.10
**$I^2 = 0.71$, $Tau^2 = 5.37$**		**6455**	**5129**	**3.21 (1.58, 4.85)**	
**Supplement duration − > 12m**					
Bangladesh	RDNS (36)	549	272	4.10 (1.86, 6.34)	0.24
Bangladesh	WASH−B (37)	234	186	3.09 (1.22, 4.97)	0.26
Kenya	WASH−B (42)	350	300	3.94 (2.05, 5.84)	0.26
Mali	PROMIS CS (46)	953	970	6.25 (4.16, 8.34)	0.25
**$I^2 = 0.41$, $Tau^2 = 0.74$**		**2086**	**1728**	**4.30 (2.98, 5.61)**	

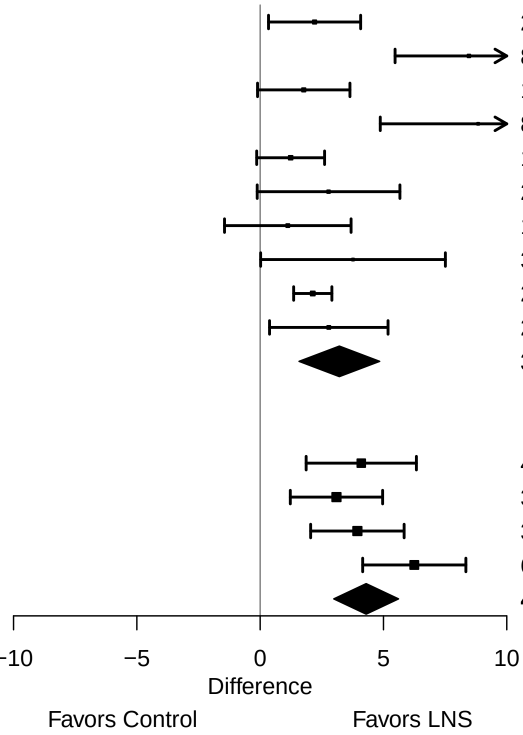

Supplemental figure 6A: Mean difference in hemoglobin concentration

6A8: Stratified by Iron dose

Iron dose
(p−diff = 0.253)

Country	Trial	N	N	MD (95% CI)	W
**Iron dose − Less than 9 mg**					
Bangladesh	JiVitA−4 (35)	457	146	2.21 (0.34, 4.07)	0.11
Burkina Faso	iLiNS−Zinc (38)	1957	664	8.47 (5.47, 11.46)	0.08
Burkina Faso	PROMIS CS (39)	574	581	1.77 (−0.11, 3.64)	0.11
Ghana	iLiNS−DYAD−G (41)	328	661	1.24 (−0.14, 2.61)	0.12
Madagascar	MAHAY (43)	600	588	2.77 (−0.12, 5.67)	0.09
Malawi	iLiNS−DYAD−M (44)	210	432	1.12 (−1.45, 3.69)	0.09
Malawi	iLiNS−DOSE (45)	243	82	3.76 (0.02, 7.51)	0.07
Mali	PROMIS CS (46)	953	970	6.25 (4.16, 8.34)	0.10
Zimbabwe	SHINE (HIV−) (47)	1682	1594	2.13 (1.36, 2.91)	0.13
Zimbabwe	SHINE (HIV+) (48)	306	285	2.78 (0.38, 5.19)	0.10
**I^2 = 0.74, Tau^2 = 3.86**		**7310**	**6003**	**3.09 (1.68, 4.50)**	
**Iron dose − 9 mg**					
Bangladesh	RDNS (36)	549	272	4.10 (1.86, 6.34)	0.26
Bangladesh	WASH−B (37)	234	186	3.09 (1.22, 4.97)	0.28
Ghana	GHANA (40)	98	96	8.84 (4.87, 12.81)	0.17
Kenya	WASH−B (42)	350	300	3.94 (2.05, 5.84)	0.28
**I^2 = 0.55, Tau^2 = 3.56**		**1231**	**854**	**4.56 (2.34, 6.77)**	

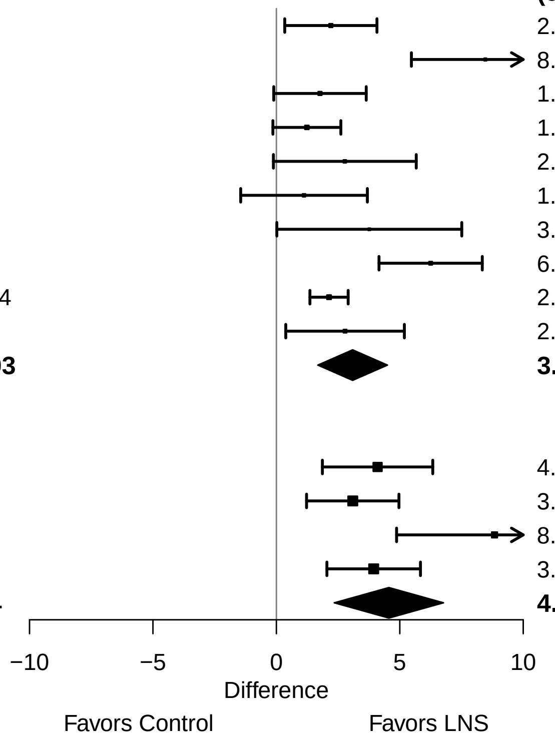

Supplemental figure 6A: Mean difference in hemoglobin concentration

6A9: Stratified by Frequency of contact

Frequency of contact
(p−diff = 0.806)

Country	Trial	N	N	MD (95% CI)	W
**Frequency of contact − Monthly**					
Bangladesh	RDNS (36)	549	272	4.10 (1.86, 6.34)	0.14
Burkina Faso	PROMIS CS (39)	574	581	1.77 (−0.11, 3.64)	0.15
Kenya	WASH−B (42)	350	300	3.94 (2.05, 5.84)	0.15
Madagascar	MAHAY (43)	600	588	2.77 (−0.12, 5.67)	0.12
Mali	PROMIS CS (46)	953	970	6.25 (4.16, 8.34)	0.14
Zimbabwe	SHINE (HIV−) (47)	1682	1594	2.13 (1.36, 2.91)	0.18
Zimbabwe	SHINE (HIV+) (48)	306	285	2.78 (0.38, 5.19)	0.13
**I² = 0.65, Tau² = 1.36**		**5014**	**4590**	**3.30 (2.15, 4.44)**	
**Frequency of contact − Weekly**					
Bangladesh	JiVitA−4 (35)	457	146	2.21 (0.34, 4.07)	0.17
Bangladesh	WASH−B (37)	234	186	3.09 (1.22, 4.97)	0.17
Burkina Faso	iLiNS−Zinc (38)	1957	664	8.47 (5.47, 11.46)	0.13
Ghana	GHANA (40)	98	96	8.84 (4.87, 12.81)	0.10
Ghana	iLiNS−DYAD−G (41)	328	661	1.24 (−0.14, 2.61)	0.18
Malawi	iLiNS−DYAD−M (44)	210	432	1.12 (−1.45, 3.69)	0.14
Malawi	iLiNS−DOSE (45)	243	82	3.76 (0.02, 7.51)	0.11
**I² = 0.80, Tau² = 8.10**		**3527**	**2267**	**3.84 (1.51, 6.18)**	

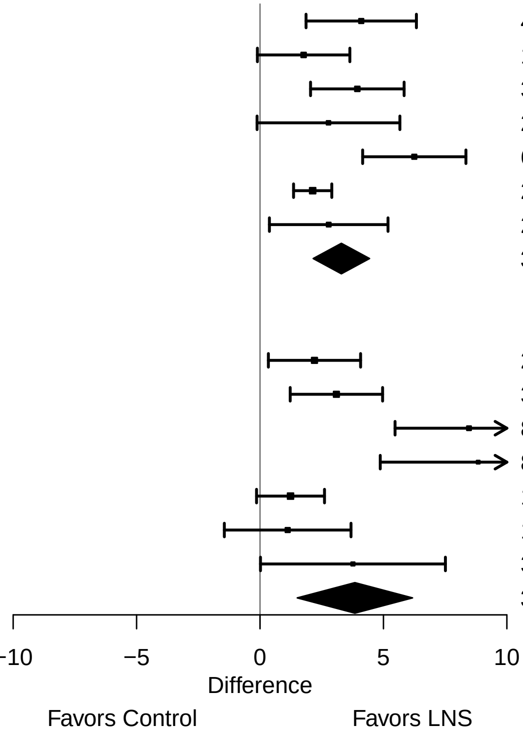

Supplemental figure 6A: Mean difference in hemoglobin concentration

6A10: Stratified by Average SQ-LNS compliance

Average SQ−LNS compliance
(p−diff = 0.086)

Country	Trial	N	N	MD (95% CI)	W
**Average SQ−LNS compliance − Low**					
Burkina Faso	PROMIS CS (39)	574	581	1.77 (−0.11, 3.64)	0.15
Ghana	iLiNS−DYAD−G (41)	328	661	1.24 (−0.14, 2.61)	0.16
Malawi	iLiNS−DYAD−M (44)	210	432	1.12 (−1.45, 3.69)	0.13
Malawi	iLiNS−DOSE (45)	243	82	3.76 (0.02, 7.51)	0.10
Mali	PROMIS CS (46)	953	970	6.25 (4.16, 8.34)	0.14
Zimbabwe	SHINE (HIV−) (47)	1682	1594	2.13 (1.36, 2.91)	0.18
Zimbabwe	SHINE (HIV+) (48)	306	285	2.78 (0.38, 5.19)	0.14
**I² = 0.67, Tau² = 2.09**		**4296**	**4605**	**2.59 (1.27, 3.91)**	
**Average SQ−LNS compliance − High**					
Bangladesh	JiVitA−4 (35)	457	146	2.21 (0.34, 4.07)	0.19
Bangladesh	RDNS (36)	549	272	4.10 (1.86, 6.34)	0.17
Bangladesh	WASH−B (37)	234	186	3.09 (1.22, 4.97)	0.19
Burkina Faso	iLiNS−Zinc (38)	1957	664	8.47 (5.47, 11.46)	0.15
Ghana	GHANA (40)	98	96	8.84 (4.87, 12.81)	0.12
Kenya	WASH−B (42)	350	300	3.94 (2.05, 5.84)	0.19
**I² = 0.73, Tau² = 5.66**		**3645**	**1664**	**4.78 (2.63, 6.94)**	

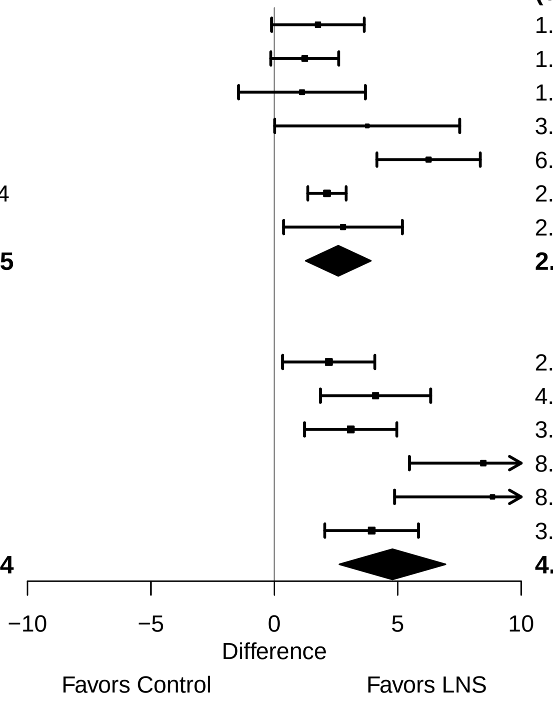

Supplemental figure 6B: Anemia prevalence ratio

6B1: Stratified by Geographic region

Geographic region
(p−diff = 0.055)

Country	Trial	N	N	PR (95% CI)	W
**Geographic region − SEAR**					
Bangladesh	JiVitA−4 (35)	457	146	0.83 (0.54, 1.28)	0.29
Bangladesh	RDNS (36)	549	272	0.66 (0.51, 0.84)	0.52
Bangladesh	WASH−B (37)	234	186	0.42 (0.24, 0.74)	0.19
**I^2 = 0.43, Tau^2 = 0.05**		**1240**	**604**	**0.64 (0.45, 0.90)**	
**Geographic region − AFR**					
Burkina Faso	iLiNS−Zinc (38)	1957	664	0.87 (0.83, 0.91)	0.11
Burkina Faso	PROMIS CS (39)	574	581	0.94 (0.85, 1.03)	0.11
Ghana	GHANA (40)	98	96	0.51 (0.36, 0.72)	0.05
Ghana	iLiNS−DYAD−G (41)	328	661	0.86 (0.73, 1.01)	0.09
Kenya	WASH−B (42)	350	300	0.68 (0.57, 0.81)	0.09
Madagascar	MAHAY (43)	600	588	0.88 (0.78, 1.01)	0.10
Malawi	iLiNS−DYAD−M (44)	210	432	0.90 (0.76, 1.07)	0.09
Malawi	iLiNS−DOSE (45)	243	82	0.87 (0.74, 1.02)	0.09
Mali	PROMIS CS (46)	953	970	0.82 (0.76, 0.88)	0.11
Zimbabwe	SHINE (HIV−) (47)	1682	1594	0.78 (0.70, 0.86)	0.10
Zimbabwe	SHINE (HIV+) (48)	306	285	0.65 (0.50, 0.85)	0.06
**I^2 = 0.64, Tau^2 = 0.02**		**7301**	**6253**	**0.81 (0.74, 0.89)**	

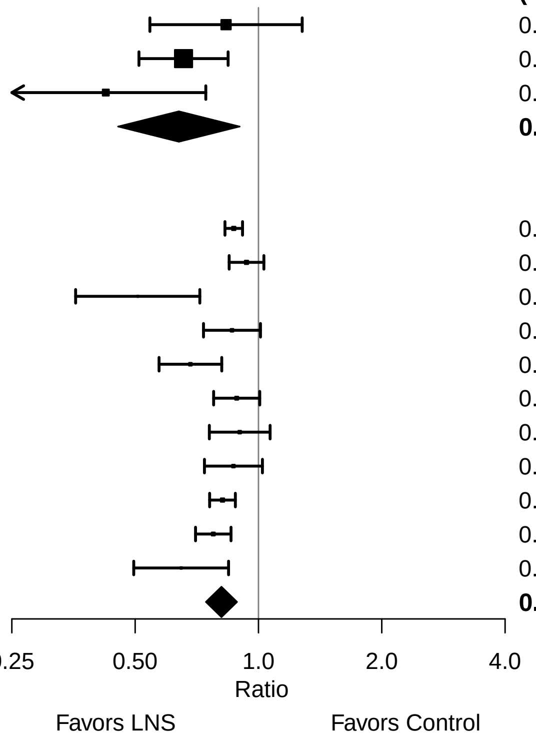

Supplemental figure 6B: Anemia prevalence ratio

6B2: Stratified by Anemia burden

Anemia burden
(p−diff = 0.073)

Country	Trial	N	N	PR (95% CI)	W
**Anemia burden − Moderate**					
Bangladesh	JiVitA−4 (35)	457	146	0.83 (0.54, 1.28)	0.07
Bangladesh	RDNS (36)	549	272	0.66 (0.51, 0.84)	0.12
Bangladesh	WASH−B (37)	234	186	0.42 (0.24, 0.74)	0.04
Ghana	iLiNS−DYAD−G (41)	328	661	0.86 (0.73, 1.01)	0.16
Kenya	WASH−B (42)	350	300	0.68 (0.57, 0.81)	0.15
Madagascar	MAHAY (43)	600	588	0.88 (0.78, 1.01)	0.17
Zimbabwe	SHINE (HIV−) (47)	1682	1594	0.78 (0.70, 0.86)	0.18
Zimbabwe	SHINE (HIV+) (48)	306	285	0.65 (0.50, 0.85)	0.11
**I² = 0.55, Tau² = 0.02**		**4506**	**4032**	**0.75 (0.66, 0.84)**	
**Anemia burden − High**					
Burkina Faso	iLiNS−Zinc (38)	1957	664	0.87 (0.83, 0.91)	0.20
Burkina Faso	PROMIS CS (39)	574	581	0.94 (0.85, 1.03)	0.19
Ghana	GHANA (40)	98	96	0.51 (0.36, 0.72)	0.09
Malawi	iLiNS−DYAD−M (44)	210	432	0.90 (0.76, 1.07)	0.16
Malawi	iLiNS−DOSE (45)	243	82	0.87 (0.74, 1.02)	0.16
Mali	PROMIS CS (46)	953	970	0.82 (0.76, 0.88)	0.20
**I² = 0.64, Tau² = 0.03**		**4035**	**2825**	**0.83 (0.72, 0.96)**	

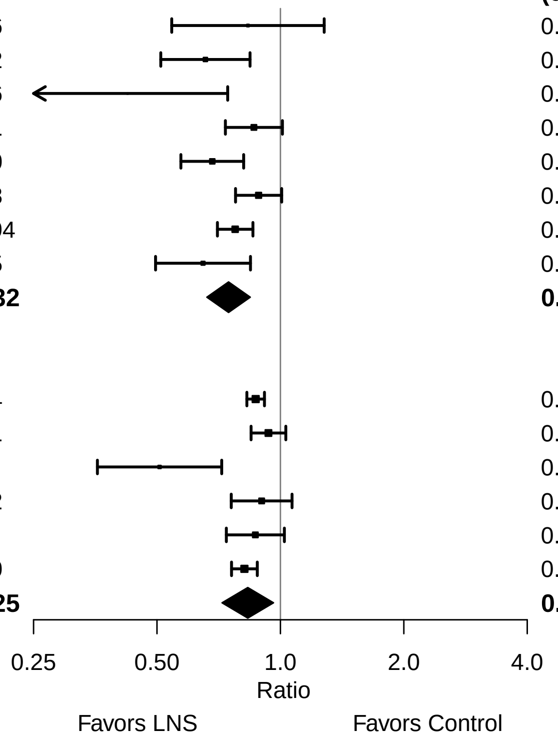

Supplemental figure 6B: Anemia prevalence ratio

6B3: Stratified by Malaria prevalence

Malaria prevalence
(p−diff = 0.023)

Country	Trial	N	N	PR (95% CI)	W
**Malaria prevalence − Less than 10%**					
Bangladesh	JiVitA−4 (35)	457	146	0.83 (0.54, 1.28)	0.08
Bangladesh	RDNS (36)	549	272	0.66 (0.51, 0.84)	0.14
Bangladesh	WASH−B (37)	234	186	0.42 (0.24, 0.74)	0.05
Kenya	WASH−B (42)	350	300	0.68 (0.57, 0.81)	0.18
Madagascar	MAHAY (43)	600	588	0.88 (0.78, 1.01)	0.20
Zimbabwe	SHINE (HIV−) (47)	1682	1594	0.78 (0.70, 0.86)	0.22
Zimbabwe	SHINE (HIV+) (48)	306	285	0.65 (0.50, 0.85)	0.13
**I² = 0.57, Tau² = 0.02**		**4178**	**3371**	**0.73 (0.64, 0.83)**	
**Malaria prevalence − At least 10%**					
Burkina Faso	iLiNS−Zinc (38)	1957	664	0.87 (0.83, 0.91)	0.18
Burkina Faso	PROMIS CS (39)	574	581	0.94 (0.85, 1.03)	0.16
Ghana	GHANA (40)	98	96	0.51 (0.36, 0.72)	0.08
Ghana	iLiNS−DYAD−G (41)	328	661	0.86 (0.73, 1.01)	0.14
Malawi	iLiNS−DYAD−M (44)	210	432	0.90 (0.76, 1.07)	0.14
Malawi	iLiNS−DOSE (45)	243	82	0.87 (0.74, 1.02)	0.14
Mali	PROMIS CS (46)	953	970	0.82 (0.76, 0.88)	0.17
**I² = 0.57, Tau² = 0.02**		**4363**	**3486**	**0.84 (0.75, 0.94)**	

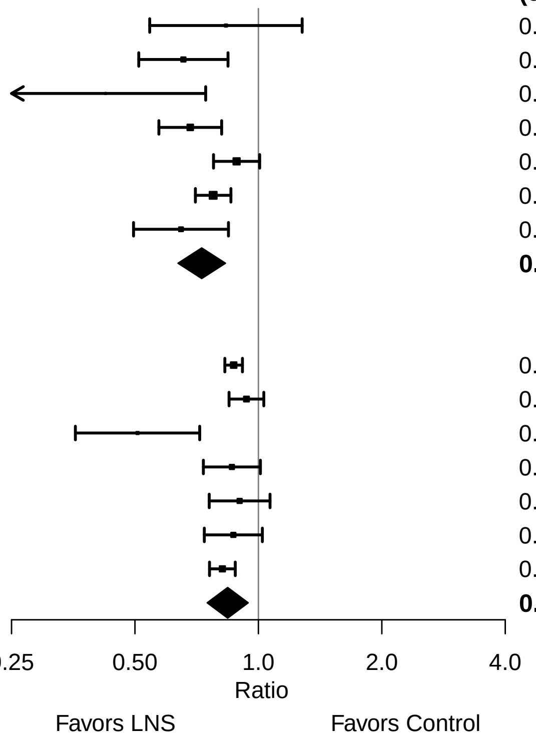

Supplemental figure 6B: Anemia prevalence ratio

6B4: Stratified by Inflammation burden

Inflammation burden
(p−diff = 0.008)

Country	Trial	N	N	PR (95% CI)	W
**Inflammation burden − Low**					
Bangladesh	RDNS (36)	549	272	0.66 (0.51, 0.84)	0.12
Bangladesh	WASH−B (37)	234	186	0.42 (0.24, 0.74)	0.04
Ghana	GHANA (40)	98	96	0.51 (0.36, 0.72)	0.08
Ghana	iLiNS−DYAD−G (41)	328	661	0.86 (0.73, 1.01)	0.15
Kenya	WASH−B (42)	350	300	0.68 (0.57, 0.81)	0.15
Madagascar	MAHAY (43)	600	588	0.88 (0.78, 1.01)	0.17
Zimbabwe	SHINE (HIV−) (47)	1682	1594	0.78 (0.70, 0.86)	0.18
Zimbabwe	SHINE (HIV+) (48)	306	285	0.65 (0.50, 0.85)	0.11
**I^2 = 0.67, Tau^2 = 0.03**		**4147**	**3982**	**0.71 (0.61, 0.82)**	
**Inflammation burden − High**					
Bangladesh	JiVitA−4 (35)	457	146	0.83 (0.54, 1.28)	0.07
Burkina Faso	iLiNS−Zinc (38)	1957	664	0.87 (0.83, 0.91)	0.21
Burkina Faso	PROMIS CS (39)	574	581	0.94 (0.85, 1.03)	0.19
Malawi	iLiNS−DYAD−M (44)	210	432	0.90 (0.76, 1.07)	0.16
Malawi	iLiNS−DOSE (45)	243	82	0.87 (0.74, 1.02)	0.16
Mali	PROMIS CS (46)	953	970	0.82 (0.76, 0.88)	0.20
**I^2 = 0.03, Tau^2 = 0.00**		**4394**	**2875**	**0.87 (0.84, 0.90)**	

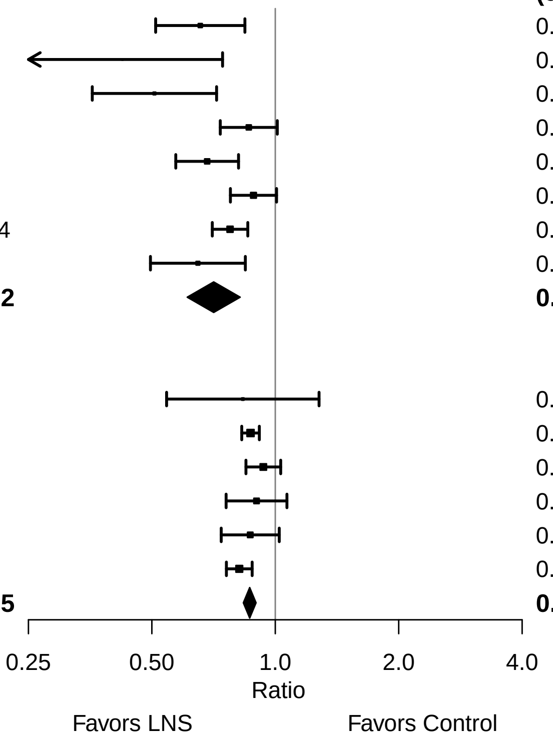

Supplemental figure 6B: Anemia prevalence ratio

6B5: Stratified by Source water quality

Source water quality
(p−diff = 0.372)

Source water quality − Improved

Country	Trial	N	N	PR (95% CI)	W
Bangladesh	JiVitA−4 (35)	457	146	0.83 (0.54, 1.28)	0.07
Bangladesh	RDNS (36)	549	272	0.66 (0.51, 0.84)	0.15
Bangladesh	WASH−B (37)	105	60	0.31 (0.10, 1.02)	0.01
Ghana	GHANA (40)	98	96	0.51 (0.36, 0.72)	0.10
Ghana	iLiNS−DYAD−G (41)	328	661	0.86 (0.73, 1.01)	0.23
Malawi	iLiNS−DYAD−M (44)	210	432	0.90 (0.76, 1.07)	0.22
Malawi	iLiNS−DOSE (45)	243	82	0.87 (0.74, 1.02)	0.23
**I² = 0.60, Tau² = 0.04**		**1990**	**1749**	**0.76 (0.63, 0.91)**	
**Source water quality − Unimproved**					
Burkina Faso	iLiNS−Zinc (38)	1957	664	0.87 (0.83, 0.91)	0.20
Burkina Faso	PROMIS CS (39)	574	581	0.94 (0.85, 1.03)	0.18
Kenya	WASH−B (42)	196	162	0.74 (0.58, 0.94)	0.09
Madagascar	MAHAY (43)	600	588	0.88 (0.78, 1.01)	0.15
Mali	PROMIS CS (46)	953	970	0.82 (0.76, 0.88)	0.19
Zimbabwe	SHINE (HIV−) (47)	819	795	0.76 (0.65, 0.88)	0.14
Zimbabwe	SHINE (HIV+) (48)	133	126	0.79 (0.53, 1.18)	0.05
**I² = 0.37, Tau² = 0.00**		**5232**	**3886**	**0.85 (0.81, 0.90)**	

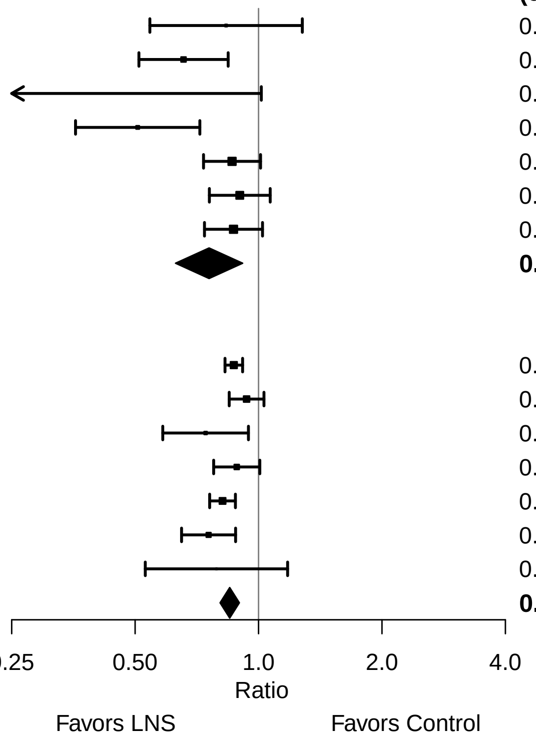

Supplemental figure 6B: Anemia prevalence ratio

6B6: Stratified by Sanitation

Sanitation
(p−diff = 0.518)

Country	Trial	N	N	PR (95% CI)	W
**Sanitation − Improved**					
Bangladesh	JiVitA−4 (35)	457	146	0.83 (0.54, 1.28)	0.06
Bangladesh	RDNS (36)	549	272	0.66 (0.51, 0.84)	0.13
Bangladesh	WASH−B (37)	105	60	0.31 (0.10, 1.02)	0.01
Burkina Faso	PROMIS CS (39)	574	581	0.94 (0.85, 1.03)	0.25
Ghana	GHANA (40)	98	96	0.51 (0.36, 0.72)	0.08
Ghana	iLiNS−DYAD−G (41)	328	661	0.86 (0.73, 1.01)	0.19
Mali	PROMIS CS (46)	953	970	0.82 (0.76, 0.88)	0.27
**I^2 = 0.70, Tau^2 = 0.04**		**3064**	**2786**	**0.76 (0.63, 0.91)**	
**Sanitation − Unimproved**					
Burkina Faso	iLiNS−Zinc (38)	1957	664	0.87 (0.83, 0.91)	0.22
Kenya	WASH−B (42)	196	162	0.74 (0.58, 0.94)	0.10
Madagascar	MAHAY (43)	600	588	0.88 (0.78, 1.01)	0.17
Malawi	iLiNS−DYAD−M (44)	210	432	0.90 (0.76, 1.07)	0.14
Malawi	iLiNS−DOSE (45)	243	82	0.87 (0.74, 1.02)	0.15
Zimbabwe	SHINE (HIV−) (47)	819	795	0.76 (0.65, 0.88)	0.16
Zimbabwe	SHINE (HIV+) (48)	133	126	0.79 (0.53, 1.18)	0.05
**I^2 = 0.00, Tau^2 = 0.00**		**4158**	**2849**	**0.86 (0.83, 0.90)**	

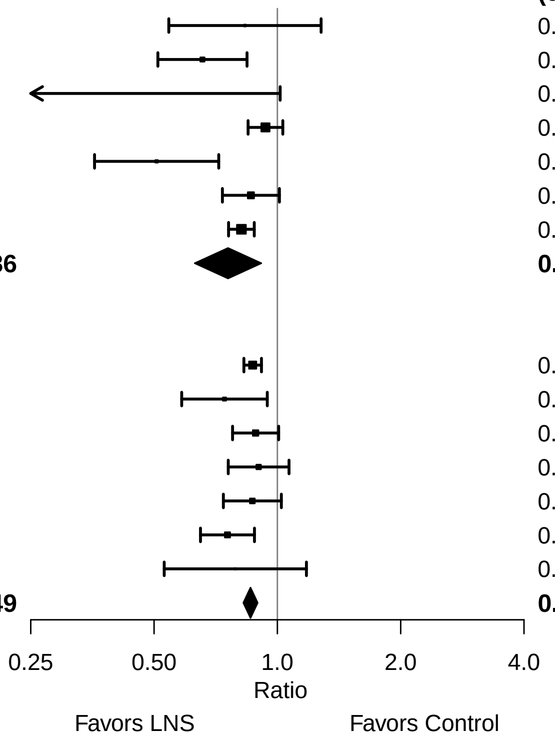

Supplemental figure 6B: Anemia prevalence ratio

6B7: Stratified by Supplement duration

Supplement duration
(p−diff = 0.058)

Country	Trial	N	N	PR (95% CI)	W
**Supplement duration − 12m or less**					
Bangladesh	JiVitA−4 (35)	457	146	0.83 (0.54, 1.28)	0.05
Burkina Faso	iLiNS−Zinc (38)	1957	664	0.87 (0.83, 0.91)	0.14
Burkina Faso	PROMIS CS (39)	574	581	0.94 (0.85, 1.03)	0.13
Ghana	GHANA (40)	98	96	0.51 (0.36, 0.72)	0.06
Ghana	iLiNS−DYAD−G (41)	328	661	0.86 (0.73, 1.01)	0.11
Madagascar	MAHAY (43)	600	588	0.88 (0.78, 1.01)	0.12
Malawi	iLiNS−DYAD−M (44)	210	432	0.90 (0.76, 1.07)	0.10
Malawi	iLiNS−DOSE (45)	243	82	0.87 (0.74, 1.02)	0.11
Zimbabwe	SHINE (HIV−) (47)	1682	1594	0.78 (0.70, 0.86)	0.12
Zimbabwe	SHINE (HIV+) (48)	306	285	0.65 (0.50, 0.85)	0.08
**I^2 = 0.57, Tau^2 = 0.01**		**6455**	**5129**	**0.83 (0.75, 0.91)**	
**Supplement duration − > 12m**					
Bangladesh	RDNS (36)	549	272	0.66 (0.51, 0.84)	0.23
Bangladesh	WASH−B (37)	234	186	0.42 (0.24, 0.74)	0.09
Kenya	WASH−B (42)	350	300	0.68 (0.57, 0.81)	0.30
Mali	PROMIS CS (46)	953	970	0.82 (0.76, 0.88)	0.38
**I^2 = 0.70, Tau^2 = 0.03**		**2086**	**1728**	**0.69 (0.55, 0.85)**	

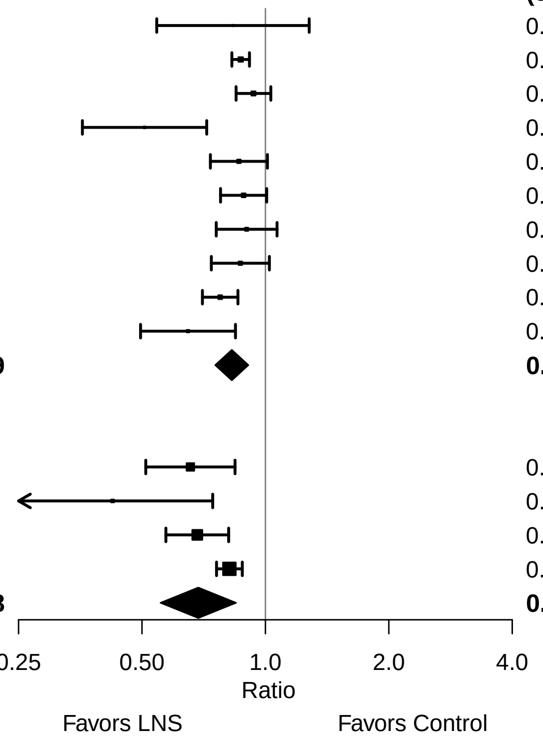

Supplemental figure 6B: Anemia prevalence ratio

6B8: Stratified by Iron dose

Iron dose
(p−diff = 0.000)

Country	Trial	N	N	PR (95% CI)	W
**Iron dose − Less than 9 mg**					
Bangladesh	JiVitA−4 (35)	457	146	0.83 (0.54, 1.28)	0.04
Burkina Faso	iLiNS−Zinc (38)	1957	664	0.87 (0.83, 0.91)	0.13
Burkina Faso	PROMIS CS (39)	574	581	0.94 (0.85, 1.03)	0.12
Ghana	iLiNS−DYAD−G (41)	328	661	0.86 (0.73, 1.01)	0.10
Madagascar	MAHAY (43)	600	588	0.88 (0.78, 1.01)	0.11
Malawi	iLiNS−DYAD−M (44)	210	432	0.90 (0.76, 1.07)	0.10
Malawi	iLiNS−DOSE (45)	243	82	0.87 (0.74, 1.02)	0.10
Mali	PROMIS CS (46)	953	970	0.82 (0.76, 0.88)	0.12
Zimbabwe	SHINE (HIV−) (47)	1682	1594	0.78 (0.70, 0.86)	0.12
Zimbabwe	SHINE (HIV+) (48)	306	285	0.65 (0.50, 0.85)	0.07
**I² = 0.35, Tau² = 0.00**		**7310**	**6003**	**0.85 (0.81, 0.89)**	
**Iron dose − 9 mg**					
Bangladesh	RDNS (36)	549	272	0.66 (0.51, 0.84)	0.30
Bangladesh	WASH−B (37)	234	186	0.42 (0.24, 0.74)	0.11
Ghana	GHANA (40)	98	96	0.51 (0.36, 0.72)	0.21
Kenya	WASH−B (42)	350	300	0.68 (0.57, 0.81)	0.38
**I² = 0.30, Tau² = 0.01**		**1231**	**854**	**0.61 (0.51, 0.73)**	

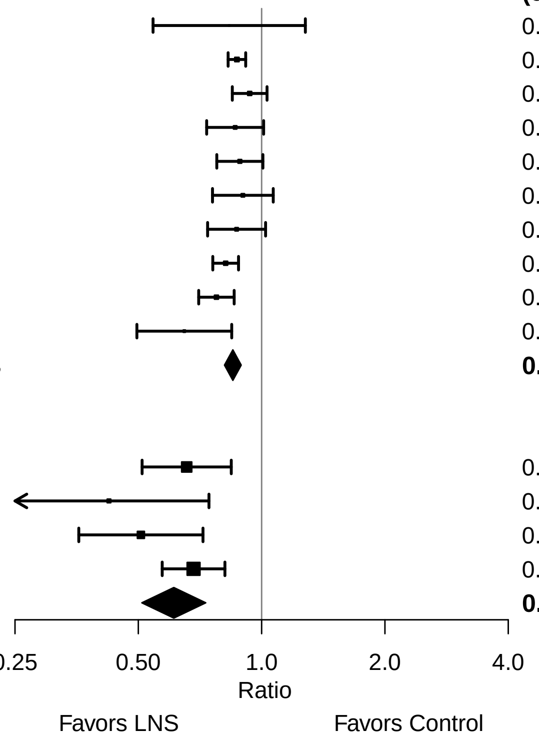

Supplemental figure 6B: Anemia prevalence ratio

6B9: Stratified by Frequency of contact

Frequency of contact
(p−diff = 0.714)

Country	Trial	N	N	PR (95% CI)	W
**Frequency of contact − Monthly**					
Bangladesh	RDNS (36)	549	272	0.66 (0.51, 0.84)	0.11
Burkina Faso	PROMIS CS (39)	574	581	0.94 (0.85, 1.03)	0.17
Kenya	WASH−B (42)	350	300	0.68 (0.57, 0.81)	0.14
Madagascar	MAHAY (43)	600	588	0.88 (0.78, 1.01)	0.15
Mali	PROMIS CS (46)	953	970	0.82 (0.76, 0.88)	0.17
Zimbabwe	SHINE (HIV−) (47)	1682	1594	0.78 (0.70, 0.86)	0.16
Zimbabwe	SHINE (HIV+) (48)	306	285	0.65 (0.50, 0.85)	0.10
**I² = 0.70, Tau² = 0.01**		**5014**	**4590**	**0.79 (0.71, 0.87)**	
**Frequency of contact − Weekly**					
Bangladesh	JiVitA−4 (35)	457	146	0.83 (0.54, 1.28)	0.08
Bangladesh	WASH−B (37)	234	186	0.42 (0.24, 0.74)	0.05
Burkina Faso	iLiNS−Zinc (38)	1957	664	0.87 (0.83, 0.91)	0.23
Ghana	GHANA (40)	98	96	0.51 (0.36, 0.72)	0.10
Ghana	iLiNS−DYAD−G (41)	328	661	0.86 (0.73, 1.01)	0.18
Malawi	iLiNS−DYAD−M (44)	210	432	0.90 (0.76, 1.07)	0.18
Malawi	iLiNS−DOSE (45)	243	82	0.87 (0.74, 1.02)	0.18
**I² = 0.61, Tau² = 0.05**		**3527**	**2267**	**0.77 (0.64, 0.94)**	

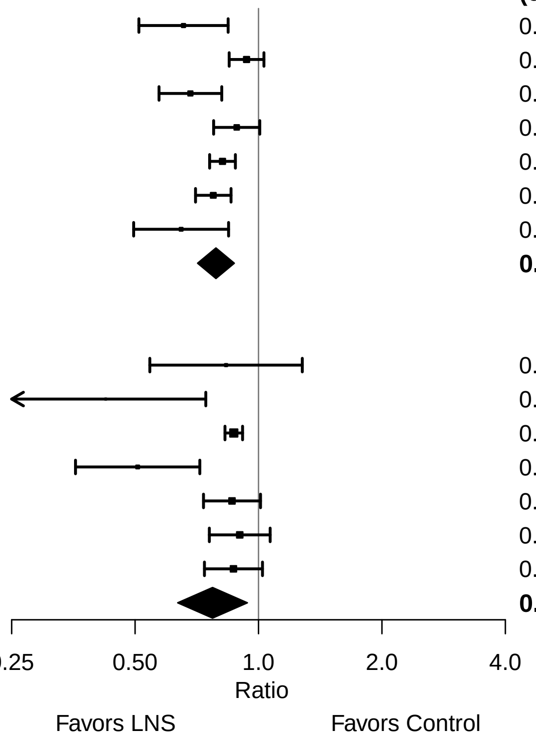

Supplemental figure 6B: Anemia prevalence ratio

6B10: Stratified by Average SQ-LNS compliance

Average SQ−LNS compliance
(p−diff = 0.062)

Country	Trial	N	N	PR (95% CI)	W
**Average SQ−LNS compliance − Low**					
Burkina Faso	PROMIS CS (39)	574	581	0.94 (0.85, 1.03)	0.16
Ghana	iLiNS−DYAD−G (41)	328	661	0.86 (0.73, 1.01)	0.14
Malawi	iLiNS−DYAD−M (44)	210	432	0.90 (0.76, 1.07)	0.13
Malawi	iLiNS−DOSE (45)	243	82	0.87 (0.74, 1.02)	0.14
Mali	PROMIS CS (46)	953	970	0.82 (0.76, 0.88)	0.17
Zimbabwe	SHINE (HIV−) (47)	1682	1594	0.78 (0.70, 0.86)	0.16
Zimbabwe	SHINE (HIV+) (48)	306	285	0.65 (0.50, 0.85)	0.10
**I² = 0.51, Tau² = 0.00**		**4296**	**4605**	**0.84 (0.78, 0.90)**	
**Average SQ−LNS compliance − High**					
Bangladesh	JiVitA−4 (35)	457	146	0.83 (0.54, 1.28)	0.10
Bangladesh	RDNS (36)	549	272	0.66 (0.51, 0.84)	0.18
Bangladesh	WASH−B (37)	234	186	0.42 (0.24, 0.74)	0.07
Burkina Faso	iLiNS−Zinc (38)	1957	664	0.87 (0.83, 0.91)	0.29
Ghana	GHANA (40)	98	96	0.51 (0.36, 0.72)	0.13
Kenya	WASH−B (42)	350	300	0.68 (0.57, 0.81)	0.23
**I² = 0.80, Tau² = 0.04**		**3645**	**1664**	**0.68 (0.56, 0.83)**	

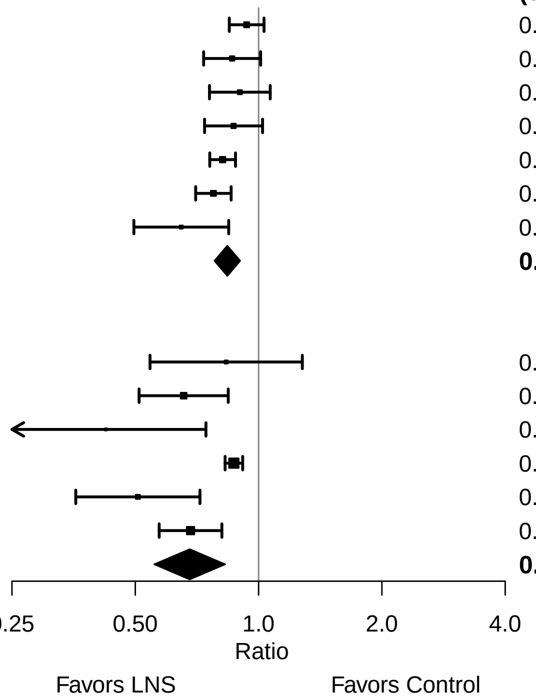

Supplemental figure 6C: Anemia prevalence difference

6C1: Stratified by Geographic region

Geographic region
(p−diff = 0.590)

Country	Trial	N	N	PD (95% CI)	W
**Geographic region − SEAR**					
Bangladesh	JiVitA−4 (35)	457	146	−0.03 (−0.09, 0.04)	0.33
Bangladesh	RDNS (36)	549	272	−0.14 (−0.21, −0.07)	0.32
Bangladesh	WASH−B (37)	234	186	−0.09 (−0.16, −0.03)	0.35
**I² = 0.65, Tau² = 0.00**		**1240**	**604**	**−0.09 (−0.15, −0.02)**	
**Geographic region − AFR**					
Burkina Faso	iLiNS−Zinc (38)	1957	664	−0.12 (−0.15, −0.09)	0.13
Burkina Faso	PROMIS CS (39)	574	581	−0.05 (−0.10, 0.01)	0.10
Ghana	GHANA (40)	98	96	−0.29 (−0.42, −0.15)	0.04
Ghana	iLiNS−DYAD−G (41)	328	661	−0.06 (−0.13, 0.00)	0.09
Kenya	WASH−B (42)	350	300	−0.15 (−0.23, −0.08)	0.08
Madagascar	MAHAY (43)	600	588	−0.07 (−0.13, −0.02)	0.10
Malawi	iLiNS−DYAD−M (44)	210	432	−0.05 (−0.13, 0.03)	0.07
Malawi	iLiNS−DOSE (45)	243	82	−0.10 (−0.21, 0.02)	0.05
Mali	PROMIS CS (46)	953	970	−0.16 (−0.19, −0.12)	0.12
Zimbabwe	SHINE (HIV−) (47)	1682	1594	−0.08 (−0.11, −0.05)	0.13
Zimbabwe	SHINE (HIV+) (48)	306	285	−0.13 (−0.20, −0.06)	0.08
**I² = 0.66, Tau² = 0.00**		**7301**	**6253**	**−0.11 (−0.14, −0.07)**	

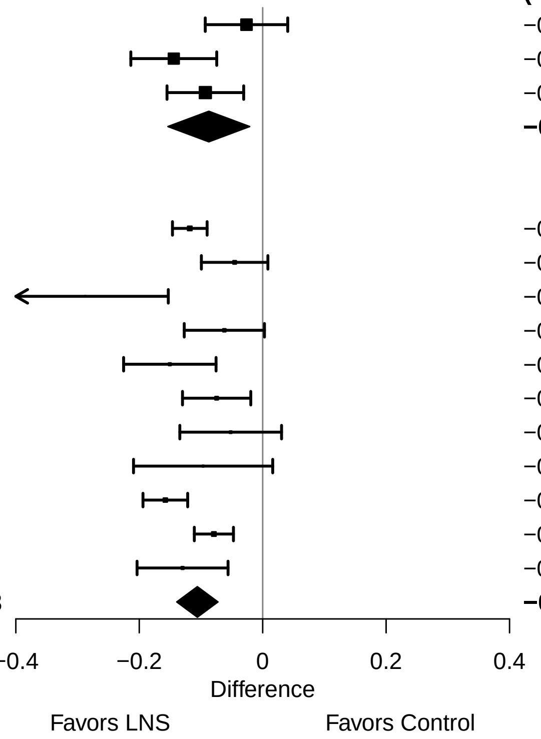

Supplemental figure 6C: Anemia prevalence difference

6C2: Stratified by Anemia burden

Anemia burden
(p−diff = 0.407)

Country	Trial	N	N	PD (95% CI)	W
**Anemia burden − Moderate**					
Bangladesh	JiVitA−4 (35)	457	146	−0.03 (−0.09, 0.04)	0.12
Bangladesh	RDNS (36)	549	272	−0.14 (−0.21, −0.07)	0.11
Bangladesh	WASH−B (37)	234	186	−0.09 (−0.16, −0.03)	0.13
Ghana	iLiNS−DYAD−G (41)	328	661	−0.06 (−0.13, 0.00)	0.12
Kenya	WASH−B (42)	350	300	−0.15 (−0.23, −0.08)	0.11
Madagascar	MAHAY (43)	600	588	−0.07 (−0.13, −0.02)	0.13
Zimbabwe	SHINE (HIV−) (47)	1682	1594	−0.08 (−0.11, −0.05)	0.17
Zimbabwe	SHINE (HIV+) (48)	306	285	−0.13 (−0.20, −0.06)	0.11
**I^2 = 0.35, Tau^2 = 0.00**		**4506**	**4032**	**−0.09 (−0.12, −0.06)**	
**Anemia burden − High**					
Burkina Faso	iLiNS−Zinc (38)	1957	664	−0.12 (−0.15, −0.09)	0.25
Burkina Faso	PROMIS CS (39)	574	581	−0.05 (−0.10, 0.01)	0.20
Ghana	GHANA (40)	98	96	−0.29 (−0.42, −0.15)	0.08
Malawi	iLiNS−DYAD−M (44)	210	432	−0.05 (−0.13, 0.03)	0.14
Malawi	iLiNS−DOSE (45)	243	82	−0.10 (−0.21, 0.02)	0.10
Mali	PROMIS CS (46)	953	970	−0.16 (−0.19, −0.12)	0.23
**I^2 = 0.75, Tau^2 = 0.00**		**4035**	**2825**	**−0.12 (−0.18, −0.05)**	

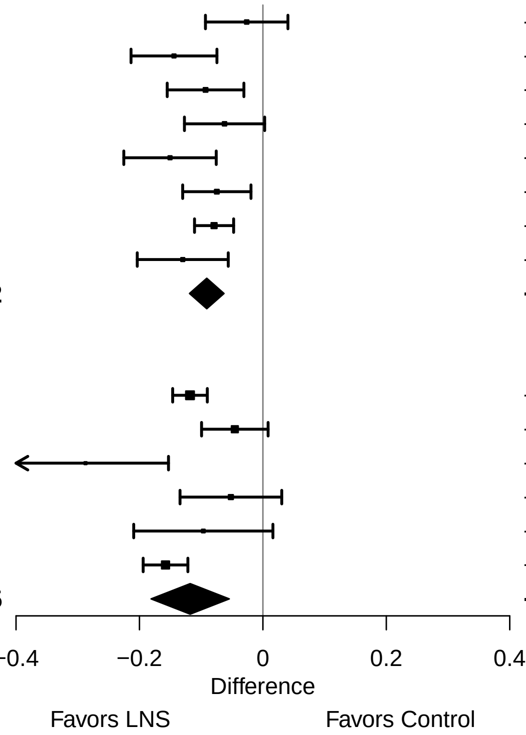

Supplemental figure 6C: Anemia prevalence difference

6C3: Stratified by Malaria prevalence

Malaria prevalence
(p−diff = 0.702)

Country	Trial	N	N	PD (95% CI)	W
**Malaria prevalence − Less than 10%**					
Bangladesh	JiVitA−4 (35)	457	146	−0.03 (−0.09, 0.04)	0.13
Bangladesh	RDNS (36)	549	272	−0.14 (−0.21, −0.07)	0.13
Bangladesh	WASH−B (37)	234	186	−0.09 (−0.16, −0.03)	0.14
Kenya	WASH−B (42)	350	300	−0.15 (−0.23, −0.08)	0.12
Madagascar	MAHAY (43)	600	588	−0.07 (−0.13, −0.02)	0.15
Zimbabwe	SHINE (HIV−) (47)	1682	1594	−0.08 (−0.11, −0.05)	0.19
Zimbabwe	SHINE (HIV+) (48)	306	285	−0.13 (−0.20, −0.06)	0.12
**I² = 0.41, Tau² = 0.00**		**4178**	**3371**	**−0.09 (−0.13, −0.06)**	
**Malaria prevalence − At least 10%**					
Burkina Faso	iLiNS−Zinc (38)	1957	664	−0.12 (−0.15, −0.09)	0.21
Burkina Faso	PROMIS CS (39)	574	581	−0.05 (−0.10, 0.01)	0.17
Ghana	GHANA (40)	98	96	−0.29 (−0.42, −0.15)	0.07
Ghana	iLiNS−DYAD−G (41)	328	661	−0.06 (−0.13, 0.00)	0.15
Malawi	iLiNS−DYAD−M (44)	210	432	−0.05 (−0.13, 0.03)	0.12
Malawi	iLiNS−DOSE (45)	243	82	−0.10 (−0.21, 0.02)	0.09
Mali	PROMIS CS (46)	953	970	−0.16 (−0.19, −0.12)	0.20
**I² = 0.74, Tau² = 0.00**		**4363**	**3486**	**−0.11 (−0.16, −0.05)**	

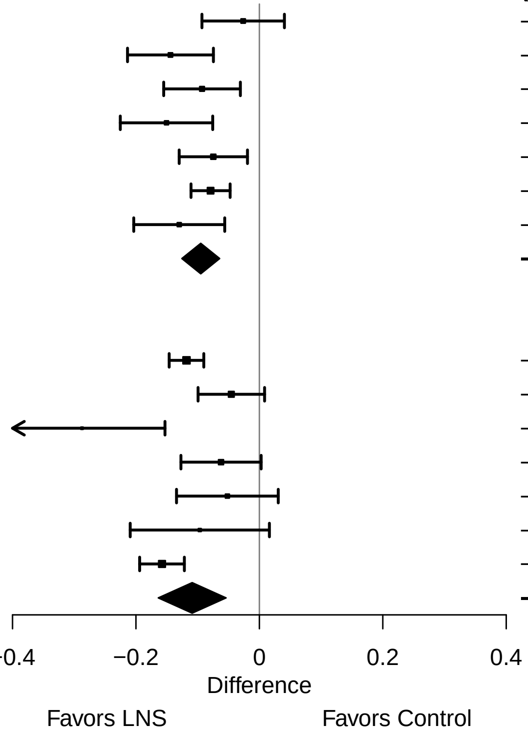

Supplemental figure 6C: Anemia prevalence difference

6C4: Stratified by Inflammation burden

Inflammation burden
(p−diff = 0.423)

Country	Trial	N	N	PD (95% CI)	W
**Inflammation burden − Low**					
Bangladesh	RDNS (36)	549	272	−0.14 (−0.21, −0.07)	0.12
Bangladesh	WASH−B (37)	234	186	−0.09 (−0.16, −0.03)	0.13
Ghana	GHANA (40)	98	96	−0.29 (−0.42, −0.15)	0.06
Ghana	iLiNS−DYAD−G (41)	328	661	−0.06 (−0.13, 0.00)	0.13
Kenya	WASH−B (42)	350	300	−0.15 (−0.23, −0.08)	0.12
Madagascar	MAHAY (43)	600	588	−0.07 (−0.13, −0.02)	0.14
Zimbabwe	SHINE (HIV−) (47)	1682	1594	−0.08 (−0.11, −0.05)	0.18
Zimbabwe	SHINE (HIV+) (48)	306	285	−0.13 (−0.20, −0.06)	0.12
**I^2 = 0.54, Tau^2 = 0.00**		**4147**	**3982**	**−0.11 (−0.15, −0.07)**	
**Inflammation burden − High**					
Bangladesh	JiVitA−4 (35)	457	146	−0.03 (−0.09, 0.04)	0.16
Burkina Faso	iLiNS−Zinc (38)	1957	664	−0.12 (−0.15, −0.09)	0.23
Burkina Faso	PROMIS CS (39)	574	581	−0.05 (−0.10, 0.01)	0.18
Malawi	iLiNS−DYAD−M (44)	210	432	−0.05 (−0.13, 0.03)	0.13
Malawi	iLiNS−DOSE (45)	243	82	−0.10 (−0.21, 0.02)	0.09
Mali	PROMIS CS (46)	953	970	−0.16 (−0.19, −0.12)	0.21
**I^2 = 0.76, Tau^2 = 0.00**		**4394**	**2875**	**−0.09 (−0.13, −0.05)**	

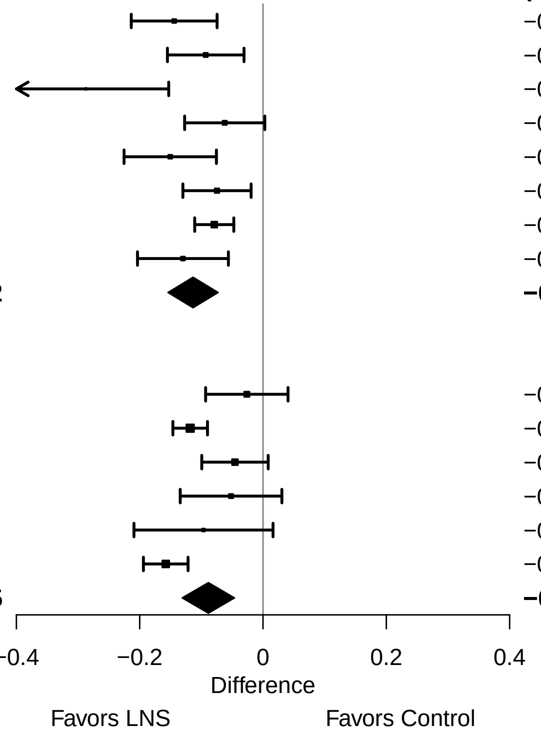

Supplemental figure 6C: Anemia prevalence difference

6C5: Stratified by Source water quality

Source water quality
(p−diff = 0.943)

Country	Trial	N	N	PD (95% CI)	W
**Source water quality − Improved**					
Bangladesh	JiVitA−4 (35)	457	146	−0.03 (−0.09, 0.04)	0.18
Bangladesh	RDNS (36)	549	272	−0.14 (−0.21, −0.07)	0.18
Bangladesh	WASH−B (37)	105	60	−0.13 (−0.23, −0.02)	0.11
Ghana	GHANA (40)	98	96	−0.29 (−0.42, −0.15)	0.08
Ghana	iLiNS−DYAD−G (41)	328	661	−0.06 (−0.13, 0.00)	0.19
Malawi	iLiNS−DYAD−M (44)	210	432	−0.05 (−0.13, 0.03)	0.15
Malawi	iLiNS−DOSE (45)	243	82	−0.10 (−0.21, 0.02)	0.11
**I^2 = 0.63, Tau^2 = 0.00**		**1990**	**1749**	**−0.10 (−0.16, −0.04)**	
**Source water quality − Unimproved**					
Burkina Faso	iLiNS−Zinc (38)	1957	664	−0.12 (−0.15, −0.09)	0.19
Burkina Faso	PROMIS CS (39)	574	581	−0.05 (−0.10, 0.01)	0.15
Kenya	WASH−B (42)	196	162	−0.13 (−0.23, −0.02)	0.08
Madagascar	MAHAY (43)	600	588	−0.07 (−0.13, −0.02)	0.15
Mali	PROMIS CS (46)	953	970	−0.16 (−0.19, −0.12)	0.18
Zimbabwe	SHINE (HIV−) (47)	819	795	−0.08 (−0.13, −0.04)	0.17
Zimbabwe	SHINE (HIV+) (48)	133	126	−0.07 (−0.18, 0.04)	0.08
**I^2 = 0.62, Tau^2 = 0.00**		**5232**	**3886**	**−0.10 (−0.13, −0.07)**	

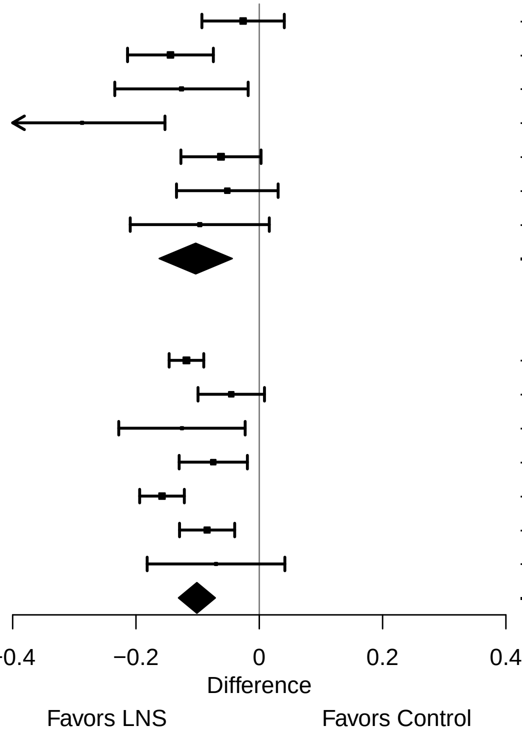

Supplemental figure 6C: Anemia prevalence difference

6C6: Stratified by Sanitation

Sanitation
(p−diff = 0.565)

Country	Trial	N	N	PD (95% CI)	W
**Sanitation − Improved**					
Bangladesh	JiVitA−4 (35)	457	146	−0.03 (−0.09, 0.04)	0.15
Bangladesh	RDNS (36)	549	272	−0.14 (−0.21, −0.07)	0.15
Bangladesh	WASH−B (37)	105	60	−0.13 (−0.23, −0.02)	0.09
Burkina Faso	PROMIS CS (39)	574	581	−0.05 (−0.10, 0.01)	0.18
Ghana	GHANA (40)	98	96	−0.29 (−0.42, −0.15)	0.07
Ghana	iLiNS−DYAD−G (41)	328	661	−0.06 (−0.13, 0.00)	0.15
Mali	PROMIS CS (46)	953	970	−0.16 (−0.19, −0.12)	0.21
**I² = 0.79, Tau² = 0.01**		**3064**	**2786**	**−0.11 (−0.17, −0.05)**	
**Sanitation − Unimproved**					
Burkina Faso	iLiNS−Zinc (38)	1957	664	−0.12 (−0.15, −0.09)	0.23
Kenya	WASH−B (42)	196	162	−0.13 (−0.23, −0.02)	0.10
Madagascar	MAHAY (43)	600	588	−0.07 (−0.13, −0.02)	0.17
Malawi	iLiNS−DYAD−M (44)	210	432	−0.05 (−0.13, 0.03)	0.13
Malawi	iLiNS−DOSE (45)	243	82	−0.10 (−0.21, 0.02)	0.09
Zimbabwe	SHINE (HIV−) (47)	819	795	−0.08 (−0.13, −0.04)	0.20
Zimbabwe	SHINE (HIV+) (48)	133	126	−0.07 (−0.18, 0.04)	0.09
**I² = 0.00, Tau² = 0.00**		**4158**	**2849**	**−0.10 (−0.12, −0.08)**	

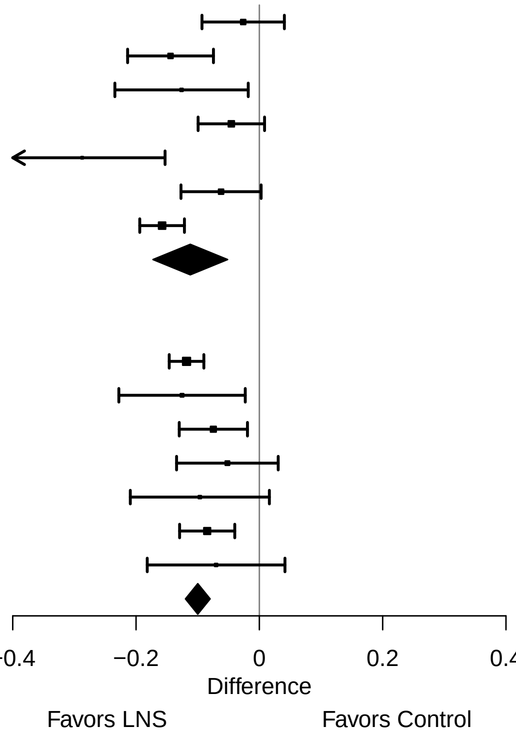

Supplemental figure 6C: Anemia prevalence difference

6C7: Stratified by Supplement duration

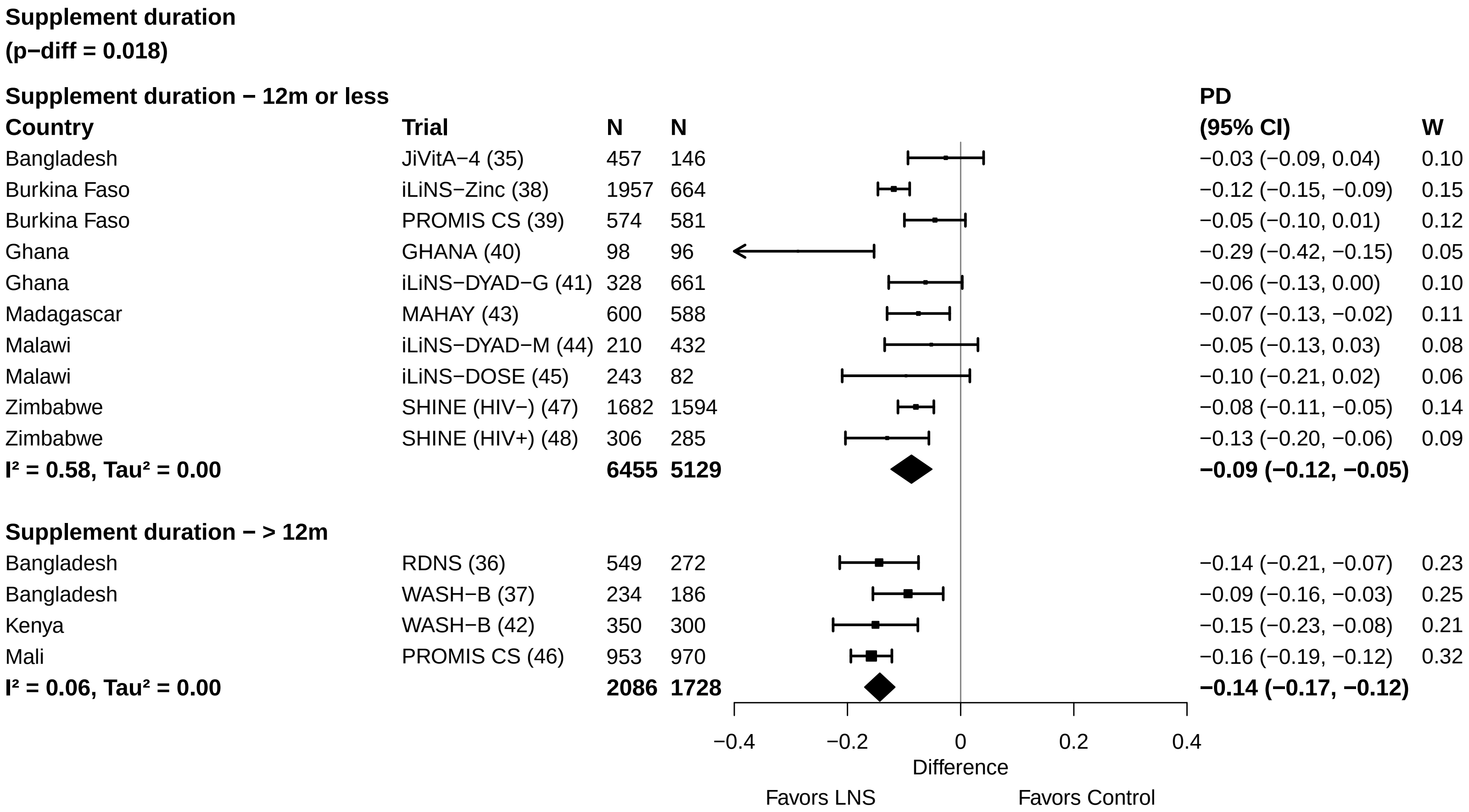

Supplemental figure 6C: Anemia prevalence difference

6C8: Stratified by Iron dose

Iron dose
(p−diff = 0.055)

Iron dose − Less than 9 mg

**Country**	**Trial**	**N**	**N**	**PD (95% CI)**	**W**
Bangladesh	JiVitA−4 (35)	457	146	−0.03 (−0.09, 0.04)	0.09
Burkina Faso	iLiNS−Zinc (38)	1957	664	−0.12 (−0.15, −0.09)	0.13
Burkina Faso	PROMIS CS (39)	574	581	−0.05 (−0.10, 0.01)	0.11
Ghana	iLiNS−DYAD−G (41)	328	661	−0.06 (−0.13, 0.00)	0.09
Madagascar	MAHAY (43)	600	588	−0.07 (−0.13, −0.02)	0.10
Malawi	iLiNS−DYAD−M (44)	210	432	−0.05 (−0.13, 0.03)	0.08
Malawi	iLiNS−DOSE (45)	243	82	−0.10 (−0.21, 0.02)	0.05
Mali	PROMIS CS (46)	953	970	−0.16 (−0.19, −0.12)	0.13
Zimbabwe	SHINE (HIV−) (47)	1682	1594	−0.08 (−0.11, −0.05)	0.13
Zimbabwe	SHINE (HIV+) (48)	306	285	−0.13 (−0.20, −0.06)	0.08
**I² = 0.65, Tau² = 0.00**		**7310**	**6003**	**−0.09 (−0.12, −0.06)**	

Iron dose − 9 mg

Bangladesh	RDNS (36)	549	272	−0.14 (−0.21, −0.07)	0.29
Bangladesh	WASH−B (37)	234	186	−0.09 (−0.16, −0.03)	0.31
Ghana	GHANA (40)	98	96	−0.29 (−0.42, −0.15)	0.14
Kenya	WASH−B (42)	350	300	−0.15 (−0.23, −0.08)	0.27
**I² = 0.57, Tau² = 0.00**		**1231**	**854**	**−0.15 (−0.23, −0.08)**	

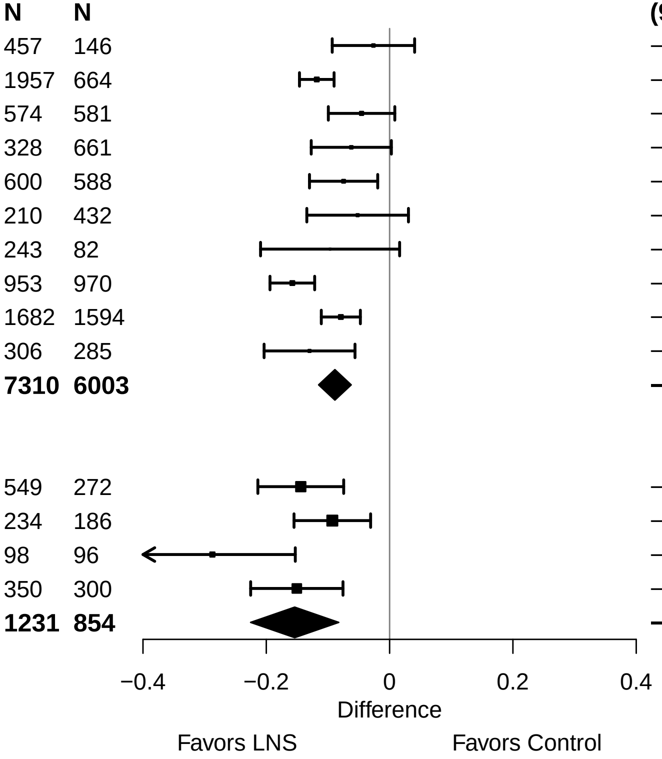

Supplemental figure 6C: Anemia prevalence difference

6C9: Stratified by Frequency of contact

Frequency of contact
(p−diff = 0.524)

Frequency of contact − Monthly

Country	Trial	N	N	PD (95% CI)	W
Bangladesh	RDNS (36)	549	272	−0.14 (−0.21, −0.07)	0.12
Burkina Faso	PROMIS CS (39)	574	581	−0.05 (−0.10, 0.01)	0.15
Kenya	WASH−B (42)	350	300	−0.15 (−0.23, −0.08)	0.12
Madagascar	MAHAY (43)	600	588	−0.07 (−0.13, −0.02)	0.14
Mali	PROMIS CS (46)	953	970	−0.16 (−0.19, −0.12)	0.17
Zimbabwe	SHINE (HIV−) (47)	1682	1594	−0.08 (−0.11, −0.05)	0.18
Zimbabwe	SHINE (HIV+) (48)	306	285	−0.13 (−0.20, −0.06)	0.12
**I^2 = 0.69, Tau^2 = 0.00**		**5014**	**4590**	**−0.11 (−0.14, −0.08)**	

Frequency of contact − Weekly

Country	Trial	N	N	PD (95% CI)	W
Bangladesh	JiVitA−4 (35)	457	146	−0.03 (−0.09, 0.04)	0.16
Bangladesh	WASH−B (37)	234	186	−0.09 (−0.16, −0.03)	0.16
Burkina Faso	iLiNS−Zinc (38)	1957	664	−0.12 (−0.15, −0.09)	0.23
Ghana	GHANA (40)	98	96	−0.29 (−0.42, −0.15)	0.07
Ghana	iLiNS−DYAD−G (41)	328	661	−0.06 (−0.13, 0.00)	0.16
Malawi	iLiNS−DYAD−M (44)	210	432	−0.05 (−0.13, 0.03)	0.13
Malawi	iLiNS−DOSE (45)	243	82	−0.10 (−0.21, 0.02)	0.09
**I^2 = 0.63, Tau^2 = 0.00**		**3527**	**2267**	**−0.09 (−0.15, −0.04)**	

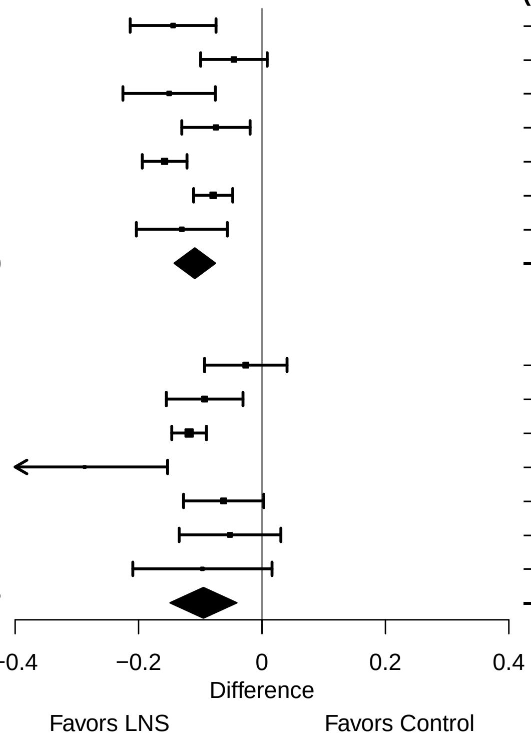

Supplemental figure 6C: Anemia prevalence difference

6C10: Stratified by Average SQ-LNS compliance

Average SQ−LNS compliance
(p−diff = 0.327)

Country	Trial	N	N	PD (95% CI)	W
**Average SQ−LNS compliance − Low**					
Burkina Faso	PROMIS CS (39)	574	581	−0.05 (−0.10, 0.01)	0.16
Ghana	iLiNS−DYAD−G (41)	328	661	−0.06 (−0.13, 0.00)	0.14
Malawi	iLiNS−DYAD−M (44)	210	432	−0.05 (−0.13, 0.03)	0.12
Malawi	iLiNS−DOSE (45)	243	82	−0.10 (−0.21, 0.02)	0.08
Mali	PROMIS CS (46)	953	970	−0.16 (−0.19, −0.12)	0.18
Zimbabwe	SHINE (HIV−) (47)	1682	1594	−0.08 (−0.11, −0.05)	0.19
Zimbabwe	SHINE (HIV+) (48)	306	285	−0.13 (−0.20, −0.06)	0.13
**I² = 0.68, Tau² = 0.00**		**4296**	**4605**	**−0.09 (−0.13, −0.06)**	
**Average SQ−LNS compliance − High**					
Bangladesh	JiVitA−4 (35)	457	146	−0.03 (−0.09, 0.04)	0.17
Bangladesh	RDNS (36)	549	272	−0.14 (−0.21, −0.07)	0.17
Bangladesh	WASH−B (37)	234	186	−0.09 (−0.16, −0.03)	0.18
Burkina Faso	iLiNS−Zinc (38)	1957	664	−0.12 (−0.15, −0.09)	0.24
Ghana	GHANA (40)	98	96	−0.29 (−0.42, −0.15)	0.08
Kenya	WASH−B (42)	350	300	−0.15 (−0.23, −0.08)	0.16
**I² = 0.67, Tau² = 0.00**		**3645**	**1664**	**−0.13 (−0.19, −0.06)**	

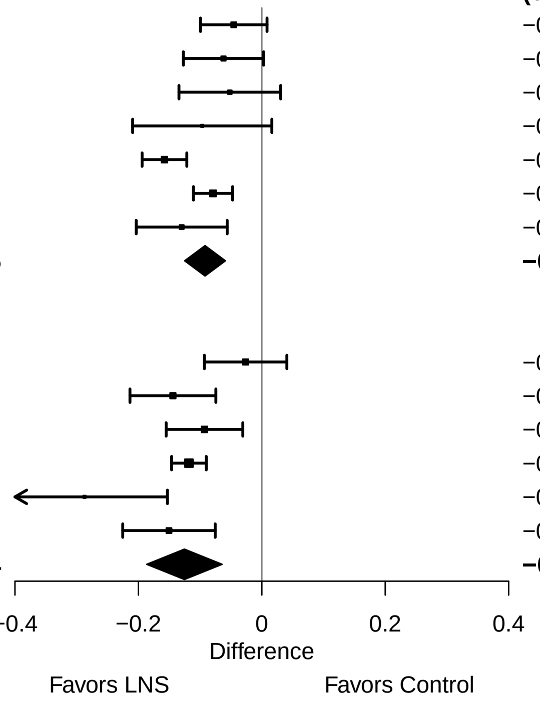

Supplemental figure 6D: Moderate-to-severe anemia prevalence ratio

6D1: Stratified by Geographic region (insufficient comparisons)

Supplemental figure 6D: Moderate-to-severe anemia prevalence ratio

6D2: Stratified by Anemia burden

Anemia burden
(p−diff = 0.388)

Country	Trial	N	N	PR (95% CI)	W
**Anemia burden − Moderate**					
Bangladesh	JiVitA−4 (35)				
Bangladesh	RDNS (36)	549	272	0.54 (0.38, 0.76)	0.17
Bangladesh	WASH−B (37)				
Ghana	iLiNS−DYAD−G (41)	328	661	0.84 (0.47, 1.51)	0.10
Kenya	WASH−B (42)	350	300	0.63 (0.45, 0.88)	0.18
Madagascar	MAHAY (43)	600	588	0.81 (0.64, 1.03)	0.22
Zimbabwe	SHINE (HIV−) (47)	1682	1594	0.67 (0.53, 0.84)	0.22
Zimbabwe	SHINE (HIV+) (48)	306	285	0.51 (0.30, 0.89)	0.11
**I² = 0.10, Tau² = 0.00**		**3815**	**3700**	**0.68 (0.59, 0.77)**	
**Anemia burden − High**					
Burkina Faso	iLiNS−Zinc (38)	1957	664	0.72 (0.65, 0.78)	0.22
Burkina Faso	PROMIS CS (39)	574	581	0.88 (0.73, 1.07)	0.19
Ghana	GHANA (40)	98	96	0.32 (0.16, 0.61)	0.07
Malawi	iLiNS−DYAD−M (44)	210	432	1.15 (0.87, 1.52)	0.16
Malawi	iLiNS−DOSE (45)	243	82	0.72 (0.54, 0.96)	0.16
Mali	PROMIS CS (46)	953	970	0.63 (0.54, 0.74)	0.20
**I² = 0.79, Tau² = 0.12**		**4035**	**2825**	**0.73 (0.54, 0.98)**	

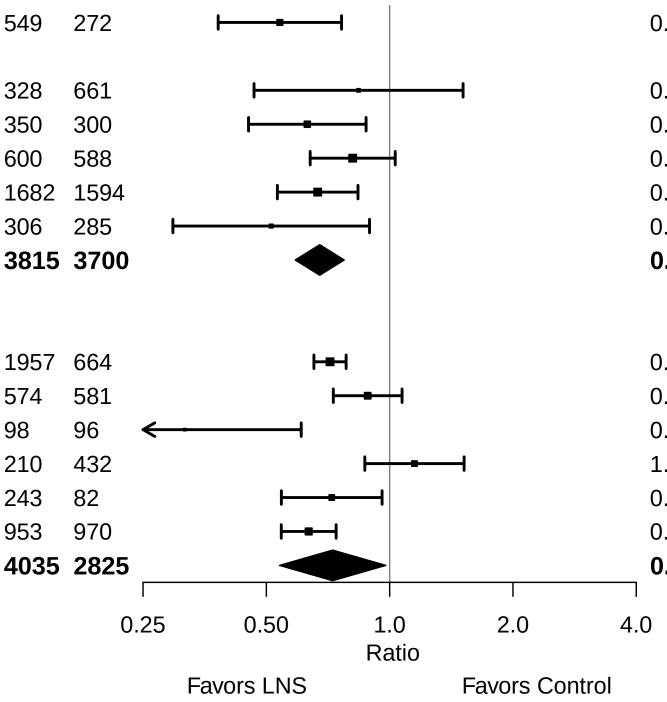

Supplemental figure 6D: Moderate-to-severe anemia prevalence ratio

6D3: Stratified by Malaria prevalence

Malaria prevalence
(p−diff = 0.282)

Country	Trial	N	N	PR (95% CI)	W
**Malaria prevalence − Less than 10%**					
Bangladesh	JiVitA−4 (35)				
Bangladesh	RDNS (36)	549	272	0.54 (0.38, 0.76)	0.19
Bangladesh	WASH−B (37)				
Kenya	WASH−B (42)	350	300	0.63 (0.45, 0.88)	0.20
Madagascar	MAHAY (43)	600	588	0.81 (0.64, 1.03)	0.24
Zimbabwe	SHINE (HIV−) (47)	1682	1594	0.67 (0.53, 0.84)	0.25
Zimbabwe	SHINE (HIV+) (48)	306	285	0.51 (0.30, 0.89)	0.12
**I² = 0.20, Tau² = 0.01**		**3487**	**3039**	**0.66 (0.57, 0.77)**	
**Malaria prevalence − At least 10%**					
Burkina Faso	iLiNS−Zinc (38)	1957	664	0.72 (0.65, 0.78)	0.20
Burkina Faso	PROMIS CS (39)	574	581	0.88 (0.73, 1.07)	0.18
Ghana	GHANA (40)	98	96	0.32 (0.16, 0.61)	0.06
Ghana	iLiNS−DYAD−G (41)	328	661	0.84 (0.47, 1.51)	0.07
Malawi	iLiNS−DYAD−M (44)	210	432	1.15 (0.87, 1.52)	0.15
Malawi	iLiNS−DOSE (45)	243	82	0.72 (0.54, 0.96)	0.15
Mali	PROMIS CS (46)	953	970	0.63 (0.54, 0.74)	0.19
**I² = 0.75, Tau² = 0.09**		**4363**	**3486**	**0.74 (0.57, 0.95)**	

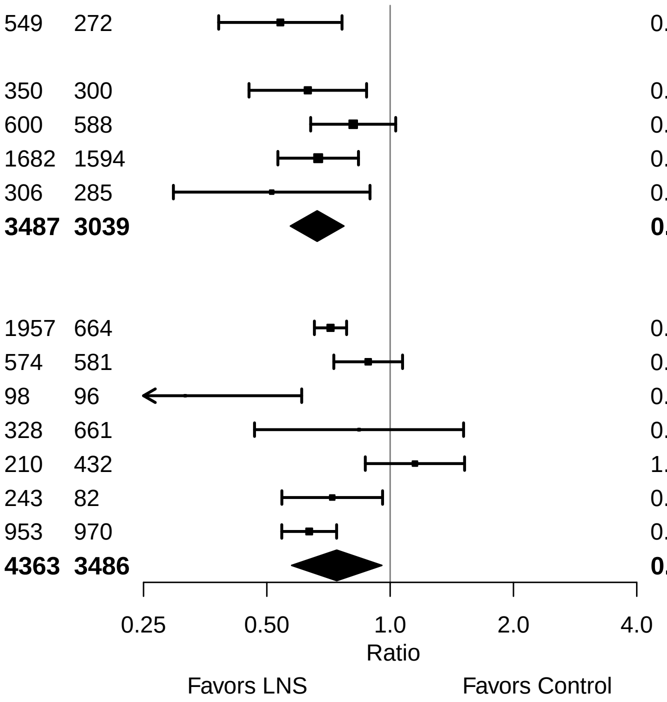

Supplemental figure 6D: Moderate-to-severe anemia prevalence ratio

6D4: Stratified by Inflammation burden

Inflammation burden
(p−diff = 0.100)

Country	Trial	N	N	PR (95% CI)	W
**Inflammation burden − Low**					
Bangladesh	RDNS (36)	549	272	0.54 (0.38, 0.76)	0.16
Bangladesh	WASH−B (37)				
Ghana	GHANA (40)	98	96	0.32 (0.16, 0.61)	0.08
Ghana	iLiNS−DYAD−G (41)	328	661	0.84 (0.47, 1.51)	0.09
Kenya	WASH−B (42)	350	300	0.63 (0.45, 0.88)	0.17
Madagascar	MAHAY (43)	600	588	0.81 (0.64, 1.03)	0.20
Zimbabwe	SHINE (HIV−) (47)	1682	1594	0.67 (0.53, 0.84)	0.21
Zimbabwe	SHINE (HIV+) (48)	306	285	0.51 (0.30, 0.89)	0.10
**I² = 0.43, Tau² = 0.03**		**3913**	**3796**	**0.63 (0.51, 0.77)**	
**Inflammation burden − High**					
Bangladesh	JiVitA−4 (35)				
Burkina Faso	iLiNS−Zinc (38)	1957	664	0.72 (0.65, 0.78)	0.23
Burkina Faso	PROMIS CS (39)	574	581	0.88 (0.73, 1.07)	0.20
Malawi	iLiNS−DYAD−M (44)	210	432	1.15 (0.87, 1.52)	0.17
Malawi	iLiNS−DOSE (45)	243	82	0.72 (0.54, 0.96)	0.17
Mali	PROMIS CS (46)	953	970	0.63 (0.54, 0.74)	0.22
**I² = 0.77, Tau² = 0.04**		**3937**	**2729**	**0.79 (0.65, 0.96)**	

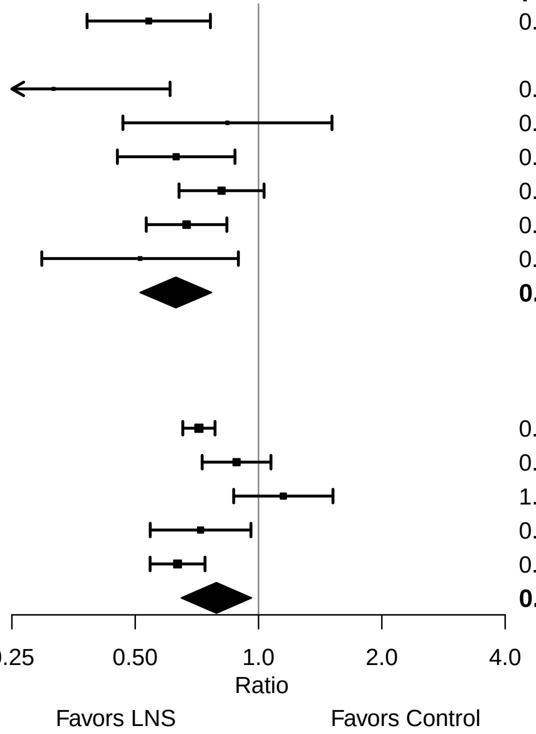

Supplemental figure 6D: Moderate-to-severe anemia prevalence ratio

6D5: Stratified by Source water quality

Source water quality
(p−diff = 0.934)

Country	Trial	N	N	PR (95% CI)	W
**Source water quality − Improved**					
Bangladesh	JiVitA−4 (35)				
Bangladesh	RDNS (36)	549	272	0.54 (0.38, 0.76)	0.23
Bangladesh	WASH−B (37)				
Ghana	GHANA (40)	98	96	0.32 (0.16, 0.61)	0.11
Ghana	iLiNS−DYAD−G (41)	328	661	0.84 (0.47, 1.51)	0.13
Malawi	iLiNS−DYAD−M (44)	210	432	1.15 (0.87, 1.52)	0.26
Malawi	iLiNS−DOSE (45)	243	82	0.72 (0.54, 0.96)	0.26
**I² = 0.79, Tau² = 0.17**		**1428**	**1543**	**0.68 (0.45, 1.02)**	
**Source water quality − Unimproved**					
Burkina Faso	iLiNS−Zinc (38)	1957	664	0.72 (0.65, 0.78)	0.20
Burkina Faso	PROMIS CS (39)	574	581	0.88 (0.73, 1.07)	0.18
Kenya	WASH−B (42)	196	162	0.80 (0.50, 1.27)	0.10
Madagascar	MAHAY (43)	600	588	0.81 (0.64, 1.03)	0.16
Mali	PROMIS CS (46)	953	970	0.63 (0.54, 0.74)	0.19
Zimbabwe	SHINE (HIV−) (47)	819	795	0.59 (0.43, 0.81)	0.14
Zimbabwe	SHINE (HIV+) (48)	133	126	0.52 (0.20, 1.30)	0.04
**I² = 0.41, Tau² = 0.01**		**5232**	**3886**	**0.72 (0.65, 0.81)**	

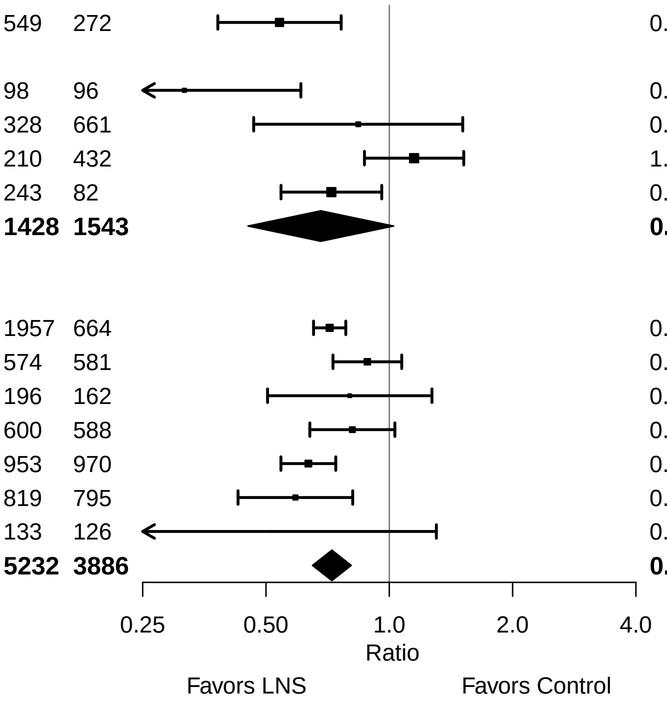

Supplemental figure 6D: Moderate-to-severe anemia prevalence ratio

6D6: Stratified by Sanitation

Sanitation
(p−diff = 0.290)

Sanitation − Improved

Country	Trial	N	N	PR (95% CI)	W
Bangladesh	JiVitA−4 (35)				
Bangladesh	RDNS (36)	549	272	0.54 (0.38, 0.76)	0.20
Bangladesh	WASH−B (37)				
Burkina Faso	PROMIS CS (39)	574	581	0.88 (0.73, 1.07)	0.28
Ghana	GHANA (40)	98	96	0.32 (0.16, 0.61)	0.10
Ghana	iLiNS−DYAD−G (41)	328	661	0.84 (0.47, 1.51)	0.12
Mali	PROMIS CS (46)	953	970	0.63 (0.54, 0.74)	0.30
**I² = 0.74, Tau² = 0.10**		**2502**	**2580**	**0.63 (0.46, 0.88)**	

Sanitation − Unimproved

Country	Trial	N	N	PR (95% CI)	W
Burkina Faso	iLiNS−Zinc (38)	1957	664	0.72 (0.65, 0.78)	0.22
Kenya	WASH−B (42)	196	162	0.80 (0.50, 1.27)	0.11
Madagascar	MAHAY (43)	600	588	0.81 (0.64, 1.03)	0.17
Malawi	iLiNS−DYAD−M (44)	210	432	1.15 (0.87, 1.52)	0.16
Malawi	iLiNS−DOSE (45)	243	82	0.72 (0.54, 0.96)	0.16
Zimbabwe	SHINE (HIV−) (47)	819	795	0.59 (0.43, 0.81)	0.15
Zimbabwe	SHINE (HIV+) (48)	133	126	0.52 (0.20, 1.30)	0.04
**I² = 0.55, Tau² = 0.02**		**4158**	**2849**	**0.77 (0.65, 0.91)**	

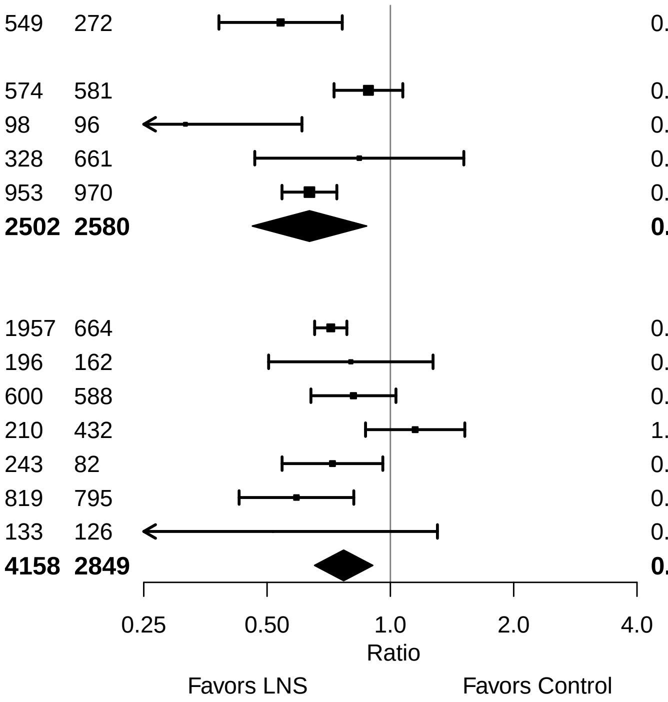

Supplemental figure 6D: Moderate-to-severe anemia prevalence ratio

6D7: Stratified by Supplement duration

Supplement duration
(p−diff = 0.088)

Country	Trial	N	N	PR (95% CI)	W
**Supplement duration − 12m or less**					
Bangladesh	JiVitA−4 (35)				
Burkina Faso	iLiNS−Zinc (38)	1957	664	0.72 (0.65, 0.78)	0.17
Burkina Faso	PROMIS CS (39)	574	581	0.88 (0.73, 1.07)	0.14
Ghana	GHANA (40)	98	96	0.32 (0.16, 0.61)	0.05
Ghana	iLiNS−DYAD−G (41)	328	661	0.84 (0.47, 1.51)	0.06
Madagascar	MAHAY (43)	600	588	0.81 (0.64, 1.03)	0.13
Malawi	iLiNS−DYAD−M (44)	210	432	1.15 (0.87, 1.52)	0.12
Malawi	iLiNS−DOSE (45)	243	82	0.72 (0.54, 0.96)	0.12
Zimbabwe	SHINE (HIV−) (47)	1682	1594	0.67 (0.53, 0.84)	0.14
Zimbabwe	SHINE (HIV+) (48)	306	285	0.51 (0.30, 0.89)	0.07
**I² = 0.65, Tau² = 0.07**		**5998**	**4983**	**0.74 (0.61, 0.91)**	
**Supplement duration − > 12m**					
Bangladesh	RDNS (36)	549	272	0.54 (0.38, 0.76)	0.29
Bangladesh	WASH−B (37)				
Kenya	WASH−B (42)	350	300	0.63 (0.45, 0.88)	0.30
Mali	PROMIS CS (46)	953	970	0.63 (0.54, 0.74)	0.42
**I² = 0.00, Tau² = 0.00**		**1852**	**1542**	**0.62 (0.54, 0.71)**	

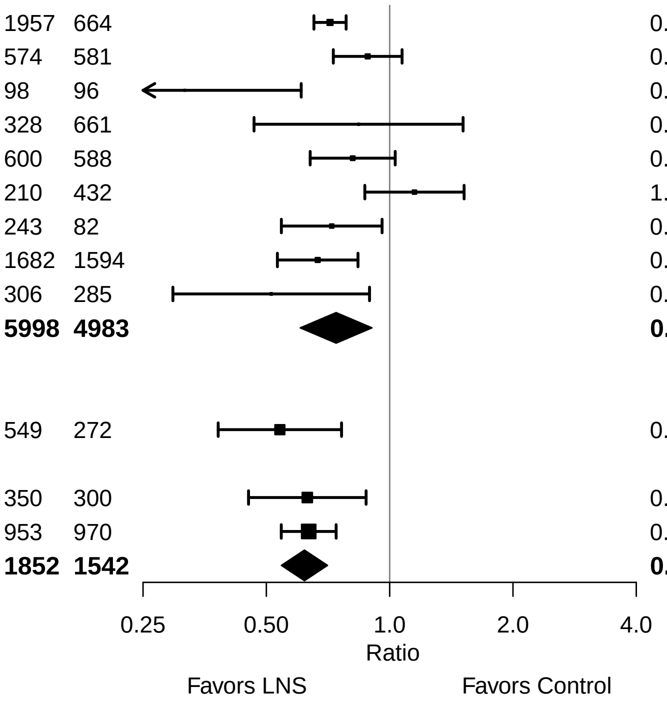

Supplemental figure 6D: Moderate-to-severe anemia prevalence ratio

6D8: Stratified by Iron dose

Iron dose
(p−diff = 0.025)

Country	Trial	N	N	PR (95% CI)	W
**Iron dose − Less than 9 mg**					
Bangladesh	JiVitA−4 (35)				
Burkina Faso	iLiNS−Zinc (38)	1957	664	0.72 (0.65, 0.78)	0.15
Burkina Faso	PROMIS CS (39)	574	581	0.88 (0.73, 1.07)	0.13
Ghana	iLiNS−DYAD−G (41)	328	661	0.84 (0.47, 1.51)	0.06
Madagascar	MAHAY (43)	600	588	0.81 (0.64, 1.03)	0.12
Malawi	iLiNS−DYAD−M (44)	210	432	1.15 (0.87, 1.52)	0.11
Malawi	iLiNS−DOSE (45)	243	82	0.72 (0.54, 0.96)	0.11
Mali	PROMIS CS (46)	953	970	0.63 (0.54, 0.74)	0.14
Zimbabwe	SHINE (HIV−) (47)	1682	1594	0.67 (0.53, 0.84)	0.12
Zimbabwe	SHINE (HIV+) (48)	306	285	0.51 (0.30, 0.89)	0.06
**I² = 0.61, Tau² = 0.02**		**6853**	**5857**	**0.76 (0.66, 0.87)**	
**Iron dose − 9 mg**					
Bangladesh	RDNS (36)	549	272	0.54 (0.38, 0.76)	0.39
Bangladesh	WASH−B (37)				
Ghana	GHANA (40)	98	96	0.32 (0.16, 0.61)	0.20
Kenya	WASH−B (42)	350	300	0.63 (0.45, 0.88)	0.41
**I² = 0.41, Tau² = 0.05**		**997**	**668**	**0.52 (0.36, 0.73)**	

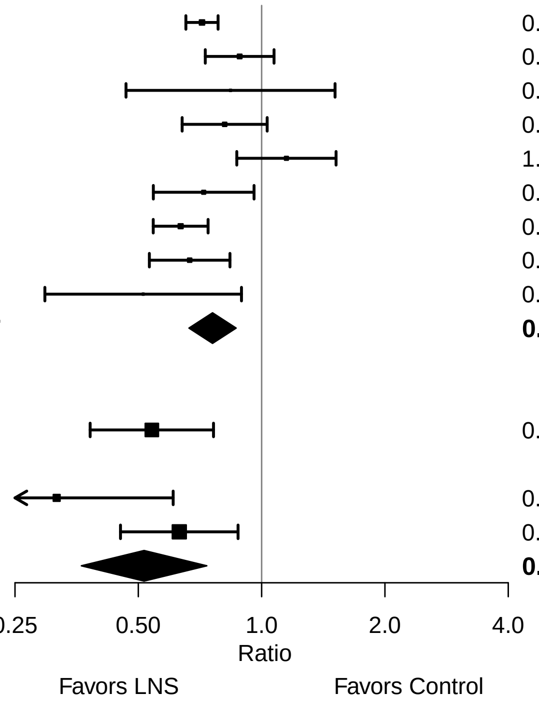

Supplemental figure 6D: Moderate-to-severe anemia prevalence ratio

6D9: Stratified by Frequency of contact

Frequency of contact
(p−diff = 0.459)

Country	Trial	N	N	PR (95% CI)	W
**Frequency of contact − Monthly**					
Bangladesh	RDNS (36)	549	272	0.54 (0.38, 0.76)	0.12
Burkina Faso	PROMIS CS (39)	574	581	0.88 (0.73, 1.07)	0.17
Kenya	WASH−B (42)	350	300	0.63 (0.45, 0.88)	0.13
Madagascar	MAHAY (43)	600	588	0.81 (0.64, 1.03)	0.16
Mali	PROMIS CS (46)	953	970	0.63 (0.54, 0.74)	0.18
Zimbabwe	SHINE (HIV−) (47)	1682	1594	0.67 (0.53, 0.84)	0.16
Zimbabwe	SHINE (HIV+) (48)	306	285	0.51 (0.30, 0.89)	0.08
**I² = 0.52, Tau² = 0.02**		**5014**	**4590**	**0.69 (0.60, 0.79)**	
**Frequency of contact − Weekly**					
Bangladesh	JiVitA−4 (35)				
Bangladesh	WASH−B (37)				
Burkina Faso	iLiNS−Zinc (38)	1957	664	0.72 (0.65, 0.78)	0.32
Ghana	GHANA (40)	98	96	0.32 (0.16, 0.61)	0.10
Ghana	iLiNS−DYAD−G (41)	328	661	0.84 (0.47, 1.51)	0.12
Malawi	iLiNS−DYAD−M (44)	210	432	1.15 (0.87, 1.52)	0.23
Malawi	iLiNS−DOSE (45)	243	82	0.72 (0.54, 0.96)	0.23
**I² = 0.76, Tau² = 0.15**		**2836**	**1935**	**0.73 (0.50, 1.06)**	

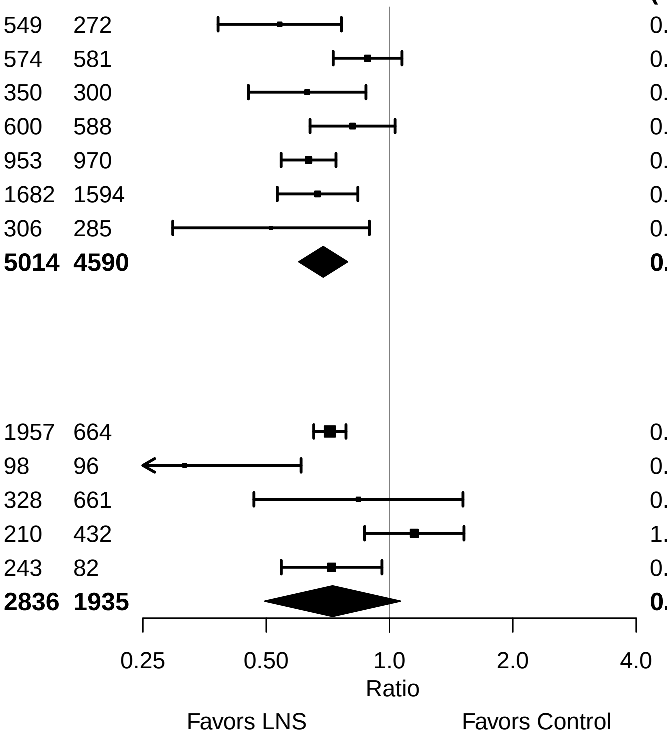

Supplemental figure 6D: Moderate-to-severe anemia prevalence ratio

6D10: Stratified by Average SQ-LNS compliance

Average SQ−LNS compliance
(p−diff = 0.115)

Country	Trial	N	N	PR (95% CI)	W
**Average SQ−LNS compliance − Low**					
Burkina Faso	PROMIS CS (39)	574	581	0.88 (0.73, 1.07)	0.18
Ghana	iLiNS−DYAD−G (41)	328	661	0.84 (0.47, 1.51)	0.08
Malawi	iLiNS−DYAD−M (44)	210	432	1.15 (0.87, 1.52)	0.15
Malawi	iLiNS−DOSE (45)	243	82	0.72 (0.54, 0.96)	0.15
Mali	PROMIS CS (46)	953	970	0.63 (0.54, 0.74)	0.19
Zimbabwe	SHINE (HIV−) (47)	1682	1594	0.67 (0.53, 0.84)	0.17
Zimbabwe	SHINE (HIV+) (48)	306	285	0.51 (0.30, 0.89)	0.09
**I² = 0.69, Tau² = 0.04**		**4296**	**4605**	**0.76 (0.63, 0.91)**	
**Average SQ−LNS compliance − High**					
Bangladesh	JiVitA−4 (35)				
Bangladesh	RDNS (36)	549	272	0.54 (0.38, 0.76)	0.25
Bangladesh	WASH−B (37)				
Burkina Faso	iLiNS−Zinc (38)	1957	664	0.72 (0.65, 0.78)	0.37
Ghana	GHANA (40)	98	96	0.32 (0.16, 0.61)	0.13
Kenya	WASH−B (42)	350	300	0.63 (0.45, 0.88)	0.25
**I² = 0.64, Tau² = 0.06**		**2954**	**1332**	**0.58 (0.43, 0.78)**	

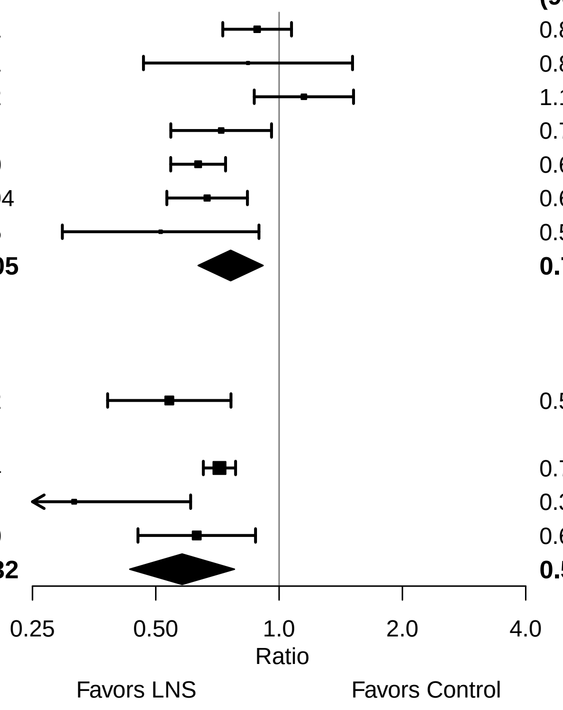

Supplemental figure 6E: Moderate-to-severe anemia prevalence difference

6E1: Stratified by Geographic region (insufficient comparisons)

Supplemental figure 6E: Moderate-to-severe anemia prevalence difference

6E2: Stratified by Anemia burden

Anemia burden
(p−diff = 0.065)

Country	Trial	N	N	PD (95% CI)	W
**Anemia burden − Moderate**					
Bangladesh	JiVitA−4 (35)				
Bangladesh	RDNS (36)	549	272	−0.08 (−0.13, −0.03)	0.16
Bangladesh	WASH−B (37)				
Ghana	iLiNS−DYAD−G (41)	328	661	−0.01 (−0.04, 0.02)	0.17
Kenya	WASH−B (42)	350	300	−0.08 (−0.14, −0.02)	0.16
Madagascar	MAHAY (43)	600	588	−0.06 (−0.11, −0.01)	0.16
Zimbabwe	SHINE (HIV−) (47)	1682	1594	−0.04 (−0.05, −0.02)	0.18
Zimbabwe	SHINE (HIV+) (48)	306	285	−0.05 (−0.09, −0.01)	0.17
**I^2 = 0.46, Tau^2 = 0.00**		**3815**	**3700**	**−0.04 (−0.07, −0.02)**	
**Anemia burden − High**					
Burkina Faso	iLiNS−Zinc (38)	1957	664	−0.21 (−0.25, −0.17)	0.19
Burkina Faso	PROMIS CS (39)	574	581	−0.04 (−0.10, 0.01)	0.18
Ghana	GHANA (40)	98	96	−0.22 (−0.33, −0.11)	0.14
Malawi	iLiNS−DYAD−M (44)	210	432	0.04 (−0.04, 0.11)	0.17
Malawi	iLiNS−DOSE (45)	243	82	−0.13 (−0.25, −0.01)	0.13
Mali	PROMIS CS (46)	953	970	−0.22 (−0.26, −0.18)	0.19
**I^2 = 0.92, Tau^2 = 0.01**		**4035**	**2825**	**−0.13 (−0.22, −0.04)**	

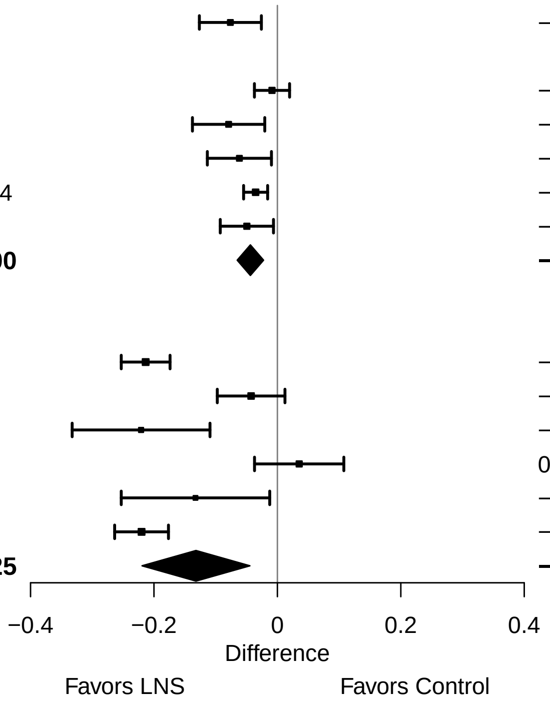

Supplemental figure 6E: Moderate-to-severe anemia prevalence difference

6E3: Stratified by Malaria prevalence

Malaria prevalence
(p−diff = 0.293)

Country	Trial	N	N	PD (95% CI)	W
**Malaria prevalence − Less than 10%**					
Bangladesh	JiVitA−4 (35)				
Bangladesh	RDNS (36)	549	272	−0.08 (−0.13, −0.03)	0.20
Bangladesh	WASH−B (37)				
Kenya	WASH−B (42)	350	300	−0.08 (−0.14, −0.02)	0.19
Madagascar	MAHAY (43)	600	588	−0.06 (−0.11, −0.01)	0.20
Zimbabwe	SHINE (HIV−) (47)	1682	1594	−0.04 (−0.05, −0.02)	0.21
Zimbabwe	SHINE (HIV+) (48)	306	285	−0.05 (−0.09, −0.01)	0.20
**I² = 0.03, Tau² = 0.00**		**3487**	**3039**	**−0.05 (−0.06, −0.03)**	
**Malaria prevalence − At least 10%**					
Burkina Faso	iLiNS−Zinc (38)	1957	664	−0.21 (−0.25, −0.17)	0.16
Burkina Faso	PROMIS CS (39)	574	581	−0.04 (−0.10, 0.01)	0.15
Ghana	GHANA (40)	98	96	−0.22 (−0.33, −0.11)	0.11
Ghana	iLiNS−DYAD−G (41)	328	661	−0.01 (−0.04, 0.02)	0.17
Malawi	iLiNS−DYAD−M (44)	210	432	0.04 (−0.04, 0.11)	0.14
Malawi	iLiNS−DOSE (45)	243	82	−0.13 (−0.25, −0.01)	0.11
Mali	PROMIS CS (46)	953	970	−0.22 (−0.26, −0.18)	0.16
**I² = 0.95, Tau² = 0.01**		**4363**	**3486**	**−0.11 (−0.19, −0.03)**	

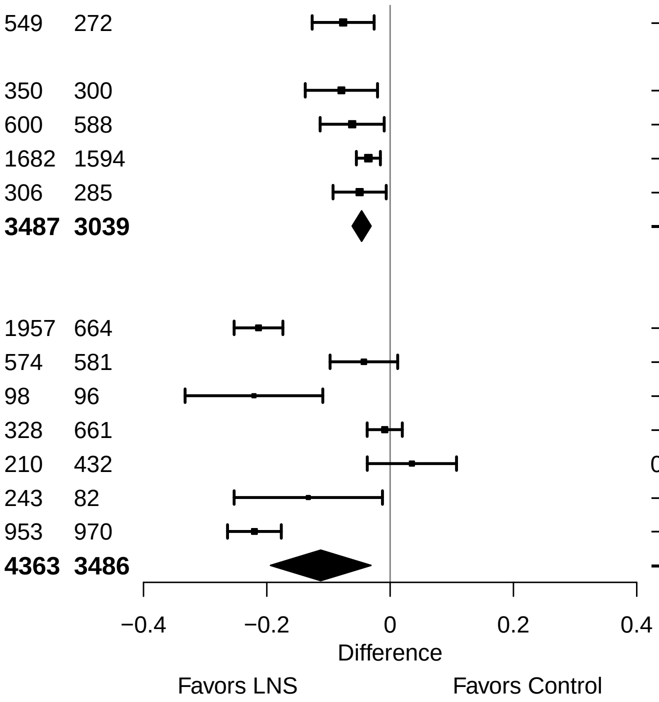

Supplemental figure 6E: Moderate-to-severe anemia prevalence difference

6E4: Stratified by Inflammation burden

Inflammation burden
(p−diff = 0.312)

Country	Trial	N	N	PD (95% CI)	W
**Inflammation burden − Low**					
Bangladesh	RDNS (36)	549	272	−0.08 (−0.13, −0.03)	0.15
Bangladesh	WASH−B (37)				
Ghana	GHANA (40)	98	96	−0.22 (−0.33, −0.11)	0.11
Ghana	iLiNS−DYAD−G (41)	328	661	−0.01 (−0.04, 0.02)	0.16
Kenya	WASH−B (42)	350	300	−0.08 (−0.14, −0.02)	0.14
Madagascar	MAHAY (43)	600	588	−0.06 (−0.11, −0.01)	0.14
Zimbabwe	SHINE (HIV−) (47)	1682	1594	−0.04 (−0.05, −0.02)	0.16
Zimbabwe	SHINE (HIV+) (48)	306	285	−0.05 (−0.09, −0.01)	0.15
**I^2 = 0.69, Tau^2 = 0.00**		**3913**	**3796**	**−0.06 (−0.10, −0.02)**	
**Inflammation burden − High**					
Bangladesh	JiVitA−4 (35)				
Burkina Faso	iLiNS−Zinc (38)	1957	664	−0.21 (−0.25, −0.17)	0.22
Burkina Faso	PROMIS CS (39)	574	581	−0.04 (−0.10, 0.01)	0.21
Malawi	iLiNS−DYAD−M (44)	210	432	0.04 (−0.04, 0.11)	0.20
Malawi	iLiNS−DOSE (45)	243	82	−0.13 (−0.25, −0.01)	0.15
Mali	PROMIS CS (46)	953	970	−0.22 (−0.26, −0.18)	0.22
**I^2 = 0.93, Tau^2 = 0.01**		**3937**	**2729**	**−0.12 (−0.21, −0.02)**	

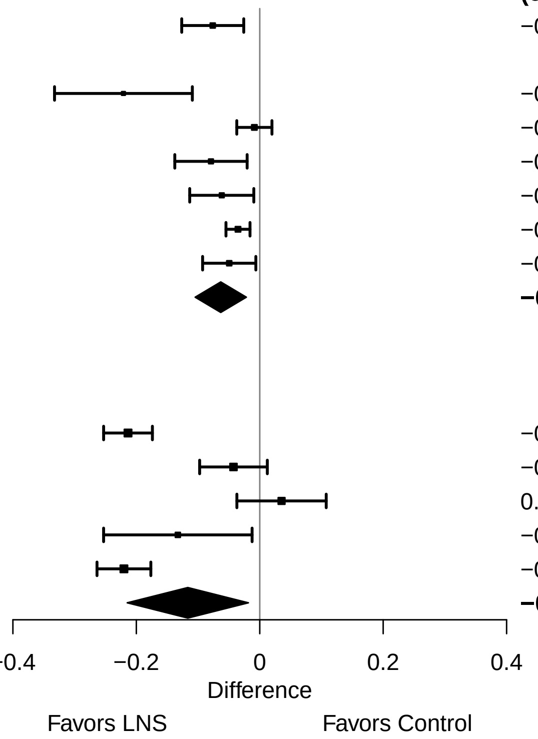

Supplemental figure 6E: Moderate-to-severe anemia prevalence difference

6E5: Stratified by Source water quality

Source water quality
(p−diff = 0.626)

Country	Trial	N	N	PD (95% CI)	W
**Source water quality − Improved**					
Bangladesh	JiVitA−4 (35)				
Bangladesh	RDNS (36)	549	272	−0.08 (−0.13, −0.03)	0.23
Bangladesh	WASH−B (37)				
Ghana	GHANA (40)	98	96	−0.22 (−0.33, −0.11)	0.17
Ghana	iLiNS−DYAD−G (41)	328	661	−0.01 (−0.04, 0.02)	0.24
Malawi	iLiNS−DYAD−M (44)	210	432	0.04 (−0.04, 0.11)	0.21
Malawi	iLiNS−DOSE (45)	243	82	−0.13 (−0.25, −0.01)	0.16
**I² = 0.82, Tau² = 0.01**		**1428**	**1543**	**−0.07 (−0.16, 0.01)**	
**Source water quality − Unimproved**					
Burkina Faso	iLiNS−Zinc (38)	1957	664	−0.21 (−0.25, −0.17)	0.15
Burkina Faso	PROMIS CS (39)	574	581	−0.04 (−0.10, 0.01)	0.14
Kenya	WASH−B (42)	196	162	−0.04 (−0.12, 0.04)	0.13
Madagascar	MAHAY (43)	600	588	−0.06 (−0.11, −0.01)	0.14
Mali	PROMIS CS (46)	953	970	−0.22 (−0.26, −0.18)	0.15
Zimbabwe	SHINE (HIV−) (47)	819	795	−0.04 (−0.07, −0.01)	0.15
Zimbabwe	SHINE (HIV+) (48)	133	126	−0.04 (−0.10, 0.02)	0.14
**I² = 0.93, Tau² = 0.01**		**5232**	**3886**	**−0.10 (−0.16, −0.03)**	

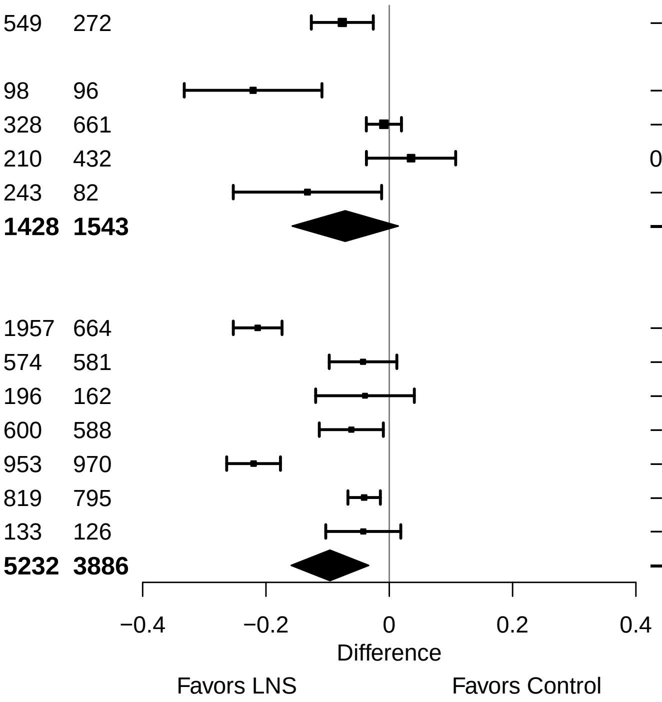

Supplemental figure 6E: Moderate-to-severe anemia prevalence difference

6E6: Stratified by Sanitation

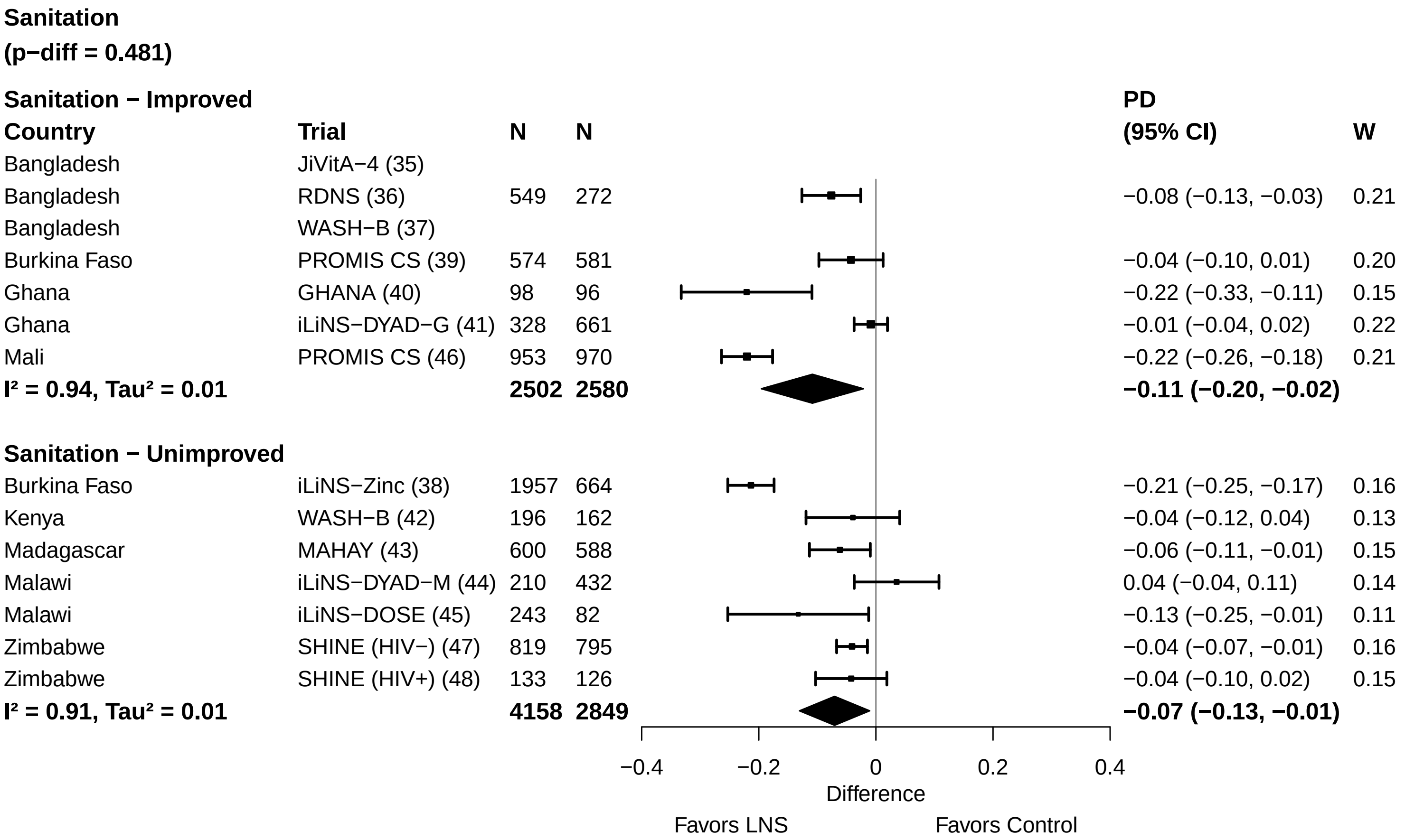

Supplemental figure 6E: Moderate-to-severe anemia prevalence difference

6E7: Stratified by Supplement duration

Supplement duration
(p−diff = 0.363)

Country	Trial	N	N	PD (95% CI)	W
**Supplement duration − 12m or less**					
Bangladesh	JiVitA−4 (35)				
Burkina Faso	iLiNS−Zinc (38)	1957	664	−0.21 (−0.25, −0.17)	0.12
Burkina Faso	PROMIS CS (39)	574	581	−0.04 (−0.10, 0.01)	0.12
Ghana	GHANA (40)	98	96	−0.22 (−0.33, −0.11)	0.09
Ghana	iLiNS−DYAD−G (41)	328	661	−0.01 (−0.04, 0.02)	0.13
Madagascar	MAHAY (43)	600	588	−0.06 (−0.11, −0.01)	0.12
Malawi	iLiNS−DYAD−M (44)	210	432	0.04 (−0.04, 0.11)	0.11
Malawi	iLiNS−DOSE (45)	243	82	−0.13 (−0.25, −0.01)	0.08
Zimbabwe	SHINE (HIV−) (47)	1682	1594	−0.04 (−0.05, −0.02)	0.13
Zimbabwe	SHINE (HIV+) (48)	306	285	−0.05 (−0.09, −0.01)	0.12
**I^2 = 0.91, Tau^2 = 0.01**		**5998**	**4983**	**−0.08 (−0.13, −0.02)**	
**Supplement duration − > 12m**					
Bangladesh	RDNS (36)	549	272	−0.08 (−0.13, −0.03)	0.33
Bangladesh	WASH−B (37)				
Kenya	WASH−B (42)	350	300	−0.08 (−0.14, −0.02)	0.32
Mali	PROMIS CS (46)	953	970	−0.22 (−0.26, −0.18)	0.34
**I^2 = 0.91, Tau^2 = 0.01**		**1852**	**1542**	**−0.13 (−0.22, −0.03)**	

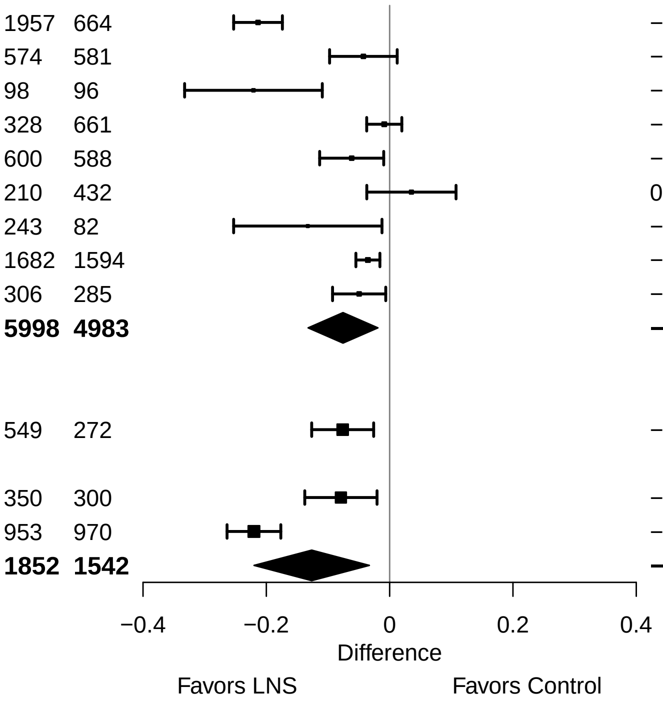

Supplemental figure 6E: Moderate-to-severe anemia prevalence difference

6E8: Stratified by Iron dose

Iron dose
(p−diff = 0.542)

Country	Trial	N	N	PD (95% CI)	W
**Iron dose − Less than 9 mg**					
Bangladesh	JiVitA−4 (35)				
Burkina Faso	iLiNS−Zinc (38)	1957	664	−0.21 (−0.25, −0.17)	0.12
Burkina Faso	PROMIS CS (39)	574	581	−0.04 (−0.10, 0.01)	0.11
Ghana	iLiNS−DYAD−G (41)	328	661	−0.01 (−0.04, 0.02)	0.12
Madagascar	MAHAY (43)	600	588	−0.06 (−0.11, −0.01)	0.11
Malawi	iLiNS−DYAD−M (44)	210	432	0.04 (−0.04, 0.11)	0.10
Malawi	iLiNS−DOSE (45)	243	82	−0.13 (−0.25, −0.01)	0.08
Mali	PROMIS CS (46)	953	970	−0.22 (−0.26, −0.18)	0.12
Zimbabwe	SHINE (HIV−) (47)	1682	1594	−0.04 (−0.05, −0.02)	0.12
Zimbabwe	SHINE (HIV+) (48)	306	285	−0.05 (−0.09, −0.01)	0.12
**I² = 0.94, Tau² = 0.01**		**6853**	**5857**	**−0.08 (−0.14, −0.02)**	
**Iron dose − 9 mg**					
Bangladesh	RDNS (36)	549	272	−0.08 (−0.13, −0.03)	0.37
Bangladesh	WASH−B (37)				
Ghana	GHANA (40)	98	96	−0.22 (−0.33, −0.11)	0.27
Kenya	WASH−B (42)	350	300	−0.08 (−0.14, −0.02)	0.36
**I² = 0.65, Tau² = 0.00**		**997**	**668**	**−0.11 (−0.20, −0.03)**	

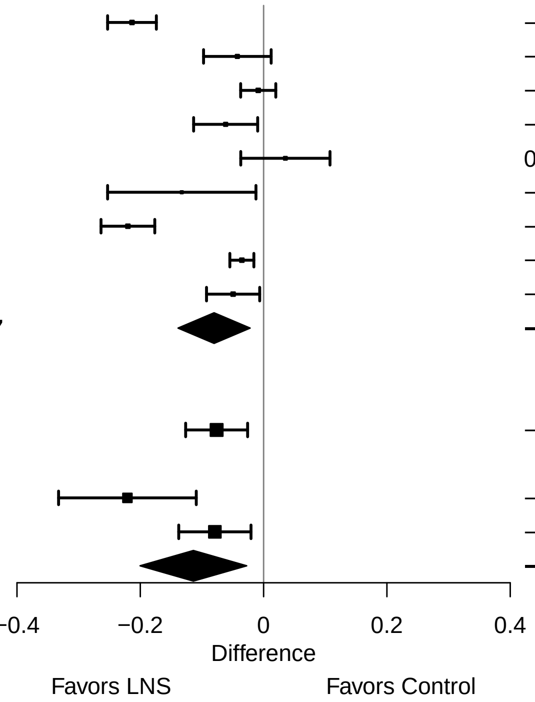

Supplemental figure 6E: Moderate-to-severe anemia prevalence difference

6E9: Stratified by Frequency of contact

Frequency of contact
(p−diff = 0.680)

Country	Trial	N	N	PD (95% CI)	W
**Frequency of contact − Monthly**					
Bangladesh	RDNS (36)	549	272	−0.08 (−0.13, −0.03)	0.14
Burkina Faso	PROMIS CS (39)	574	581	−0.04 (−0.10, 0.01)	0.14
Kenya	WASH−B (42)	350	300	−0.08 (−0.14, −0.02)	0.14
Madagascar	MAHAY (43)	600	588	−0.06 (−0.11, −0.01)	0.14
Mali	PROMIS CS (46)	953	970	−0.22 (−0.26, −0.18)	0.14
Zimbabwe	SHINE (HIV−) (47)	1682	1594	−0.04 (−0.05, −0.02)	0.15
Zimbabwe	SHINE (HIV+) (48)	306	285	−0.05 (−0.09, −0.01)	0.14
**I² = 0.90, Tau² = 0.00**		**5014**	**4590**	**−0.08 (−0.13, −0.03)**	
**Frequency of contact − Weekly**					
Bangladesh	JiVitA−4 (35)				
Bangladesh	WASH−B (37)				
Burkina Faso	iLiNS−Zinc (38)	1957	664	−0.21 (−0.25, −0.17)	0.23
Ghana	GHANA (40)	98	96	−0.22 (−0.33, −0.11)	0.16
Ghana	iLiNS−DYAD−G (41)	328	661	−0.01 (−0.04, 0.02)	0.24
Malawi	iLiNS−DYAD−M (44)	210	432	0.04 (−0.04, 0.11)	0.20
Malawi	iLiNS−DOSE (45)	243	82	−0.13 (−0.25, −0.01)	0.16
**I² = 0.95, Tau² = 0.01**		**2836**	**1935**	**−0.10 (−0.21, 0.00)**	

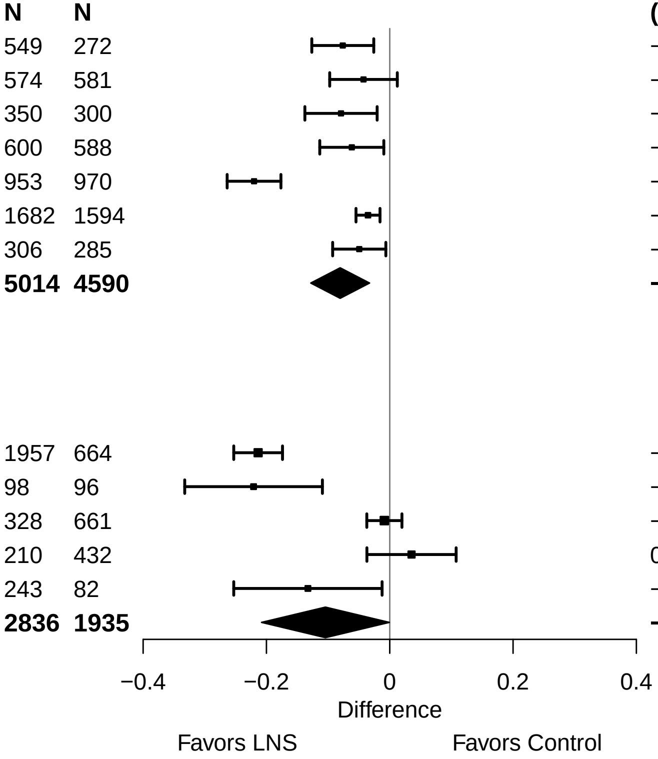

Supplemental figure 6E: Moderate-to-severe anemia prevalence difference

6E10: Stratified by Average SQ-LNS compliance

Average SQ−LNS compliance
(p−diff = 0.128)

Average SQ−LNS compliance − Low

**Country**	**Trial**	**N**	**N**	**PD (95% CI)**	**W**
Burkina Faso	PROMIS CS (39)	574	581	−0.04 (−0.10, 0.01)	0.14
Ghana	iLiNS−DYAD−G (41)	328	661	−0.01 (−0.04, 0.02)	0.16
Malawi	iLiNS−DYAD−M (44)	210	432	0.04 (−0.04, 0.11)	0.13
Malawi	iLiNS−DOSE (45)	243	82	−0.13 (−0.25, −0.01)	0.10
Mali	PROMIS CS (46)	953	970	−0.22 (−0.26, −0.18)	0.15
Zimbabwe	SHINE (HIV−) (47)	1682	1594	−0.04 (−0.05, −0.02)	0.16
Zimbabwe	SHINE (HIV+) (48)	306	285	−0.05 (−0.09, −0.01)	0.15
**I² = 0.92, Tau² = 0.01**		**4296**	**4605**	**−0.06 (−0.13, 0.00)**	

Average SQ−LNS compliance − High

**Country**	**Trial**	**N**	**N**	**PD (95% CI)**	**W**
Bangladesh	JiVitA−4 (35)				
Bangladesh	RDNS (36)	549	272	−0.08 (−0.13, −0.03)	0.27
Bangladesh	WASH−B (37)				
Burkina Faso	iLiNS−Zinc (38)	1957	664	−0.21 (−0.25, −0.17)	0.28
Ghana	GHANA (40)	98	96	−0.22 (−0.33, −0.11)	0.20
Kenya	WASH−B (42)	350	300	−0.08 (−0.14, −0.02)	0.26
**I² = 0.88, Tau² = 0.01**		**2954**	**1332**	**−0.14 (−0.22, −0.06)**	

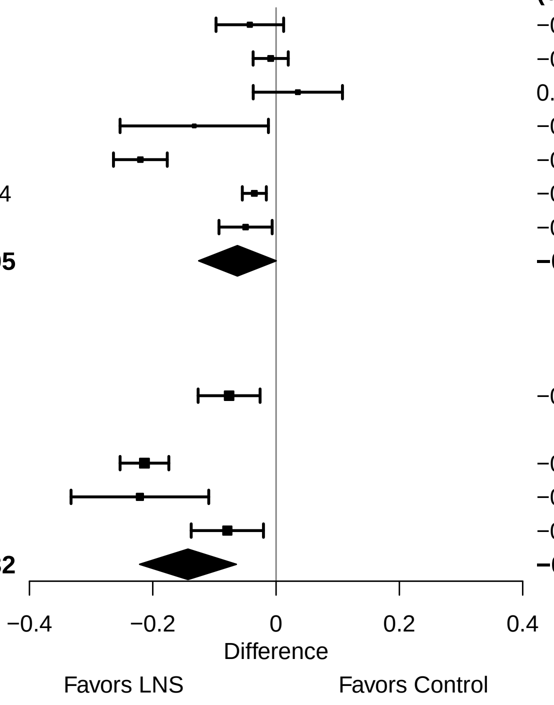

Supplemental figure 6F: Geometric mean ratio of ferritin concentration

6F1: Stratified by Geographic region

Geographic region
(p−diff = 0.800)

Country	Trial	N	N	GMR (95% CI)	W
**Geographic region − SEAR**					
Bangladesh	JiVitA−4 (35)	455	144	1.39 (1.17, 1.64)	0.29
Bangladesh	RDNS (36)	550	272	1.52 (1.40, 1.64)	0.40
Bangladesh	WASH−B (37)	212	178	1.74 (1.50, 2.01)	0.31
**I² = 0.53, Tau² = 0.01**		**1217**	**594**	**1.54 (1.37, 1.74)**	
**Geographic region − AFR**					
Burkina Faso	iLiNS−Zinc (38)	315	96	1.55 (1.31, 1.84)	0.30
Burkina Faso	PROMIS CS (39)				
Ghana	GHANA (40)	83	82	2.52 (1.49, 4.25)	0.07
Ghana	iLiNS−DYAD−G (41)				
Kenya	WASH−B (42)	298	259	1.69 (1.50, 1.91)	0.37
Madagascar	MAHAY (43)	83	51	1.29 (1.05, 1.59)	0.26
Malawi	iLiNS−DYAD−M (44)				
Malawi	iLiNS−DOSE (45)				
Mali	PROMIS CS (46)				
Zimbabwe	SHINE (HIV−) (47)				
Zimbabwe	SHINE (HIV+) (48)				
**I² = 0.63, Tau² = 0.04**		**779**	**488**	**1.61 (1.29, 2.00)**	

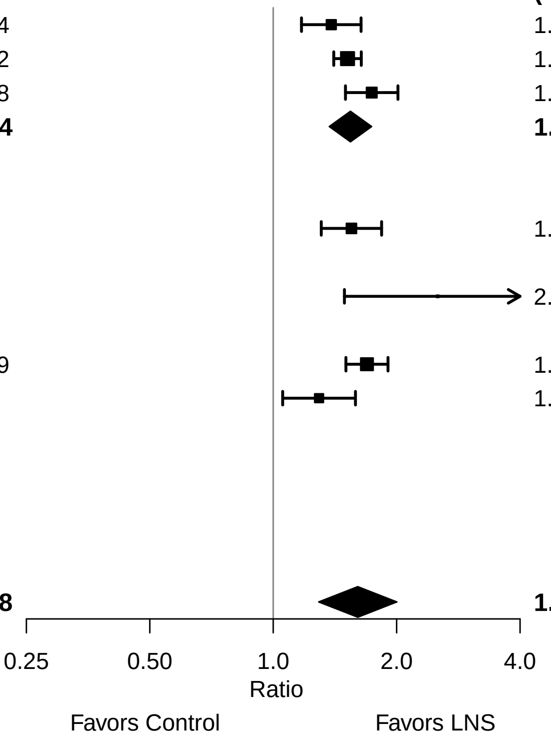

Supplemental figure 6F: Geometric mean ratio of ferritin concentration

6F2: Stratified by Anemia burden (insufficient comparisons)

Supplemental figure 6F: Geometric mean ratio of ferritin concentration

6F3: Stratified by Malaria prevalence (insufficient comparisons)

Supplemental figure 6F: Geometric mean ratio of ferritin concentration

6F4: Stratified by Inflammation burden (insufficient comparisons)

Supplemental figure 6F: Geometric mean ratio of ferritin concentration

6F5: Stratified by Source water quality

Source water quality
(p−diff = 0.862)

Country	Trial	N	N	GMR (95% CI)	W
**Source water quality − Improved**					
Bangladesh	JiVitA−4 (35)	455	144	1.39 (1.17, 1.64)	0.30
Bangladesh	RDNS (36)	550	272	1.52 (1.40, 1.64)	0.45
Bangladesh	WASH−B (37)	94	58	1.75 (1.33, 2.31)	0.18
Ghana	GHANA (40)	83	82	2.52 (1.49, 4.25)	0.07
Ghana	iLiNS−DYAD−G (41)				
Malawi	iLiNS−DYAD−M (44)				
Malawi	iLiNS−DOSE (45)				
**I² = 0.48, Tau² = 0.03**		**1182**	**556**	**1.61 (1.32, 1.96)**	
**Source water quality − Unimproved**					
Burkina Faso	iLiNS−Zinc (38)	315	96	1.55 (1.31, 1.84)	0.34
Burkina Faso	PROMIS CS (39)				
Kenya	WASH−B (42)	166	144	1.67 (1.44, 1.94)	0.38
Madagascar	MAHAY (43)	83	51	1.29 (1.05, 1.59)	0.28
Mali	PROMIS CS (46)				
Zimbabwe	SHINE (HIV−) (47)				
Zimbabwe	SHINE (HIV+) (48)				
**I² = 0.49, Tau² = 0.01**		**564**	**291**	**1.52 (1.31, 1.75)**	

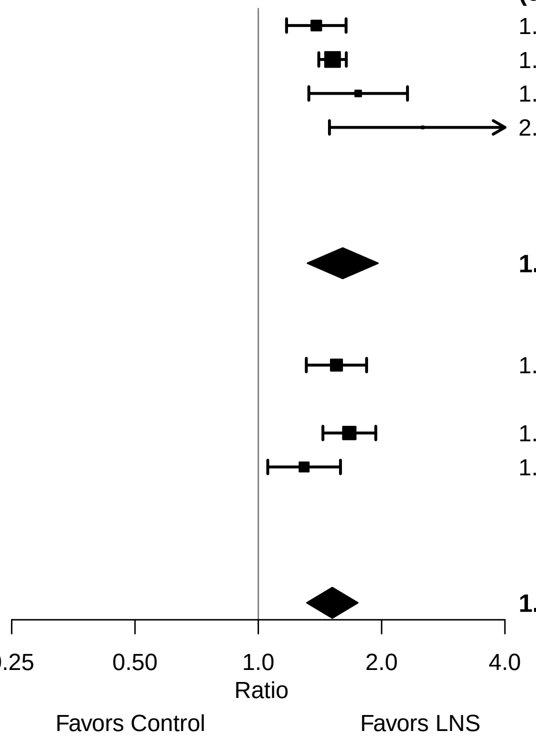

Supplemental figure 6F: Geometric mean ratio of ferritin concentration

6F6: Stratified by Sanitation

Sanitation
(p−diff = 0.862)

Country	Trial	N	N	GMR (95% CI)	W
**Sanitation − Improved**					
Bangladesh	JiVitA−4 (35)	455	144	1.39 (1.17, 1.64)	0.30
Bangladesh	RDNS (36)	550	272	1.52 (1.40, 1.64)	0.45
Bangladesh	WASH−B (37)	94	58	1.75 (1.33, 2.31)	0.18
Burkina Faso	PROMIS CS (39)				
Ghana	GHANA (40)	83	82	2.52 (1.49, 4.25)	0.07
Ghana	iLiNS−DYAD−G (41)				
Mali	PROMIS CS (46)				
**I² = 0.48, Tau² = 0.03**		**1182**	**556**	**1.61 (1.32, 1.96)**	
**Sanitation − Unimproved**					
Burkina Faso	iLiNS−Zinc (38)	315	96	1.55 (1.31, 1.84)	0.34
Kenya	WASH−B (42)	166	144	1.67 (1.44, 1.94)	0.38
Madagascar	MAHAY (43)	83	51	1.29 (1.05, 1.59)	0.28
Malawi	iLiNS−DYAD−M (44)				
Malawi	iLiNS−DOSE (45)				
Zimbabwe	SHINE (HIV−) (47)				
Zimbabwe	SHINE (HIV+) (48)				
**I² = 0.49, Tau² = 0.01**		**564**	**291**	**1.52 (1.31, 1.75)**	

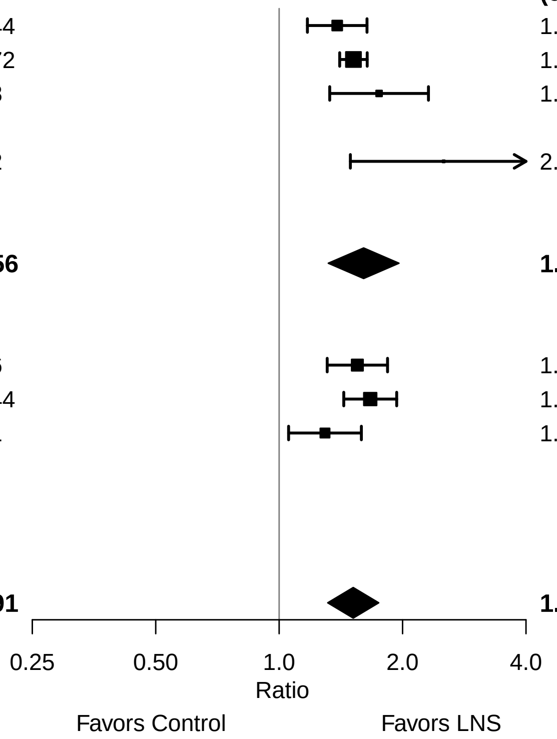

Supplemental figure 6F: Geometric mean ratio of ferritin concentration

6F7: Stratified by Supplement duration

Supplement duration
(p−diff = 0.153)

Country	Trial	N	N	GMR (95% CI)	W
**Supplement duration − 12m or less**					
Bangladesh	JiVitA−4 (35)	455	144	1.39 (1.17, 1.64)	0.32
Burkina Faso	iLiNS−Zinc (38)	315	96	1.55 (1.31, 1.84)	0.32
Burkina Faso	PROMIS CS (39)				
Ghana	GHANA (40)	83	82	2.52 (1.49, 4.25)	0.08
Ghana	iLiNS−DYAD−G (41)				
Madagascar	MAHAY (43)	83	51	1.29 (1.05, 1.59)	0.28
Malawi	iLiNS−DYAD−M (44)				
Malawi	iLiNS−DOSE (45)				
Zimbabwe	SHINE (HIV−) (47)				
Zimbabwe	SHINE (HIV+) (48)				
**I² = 0.53, Tau² = 0.04**		**936**	**373**	**1.52 (1.20, 1.91)**	
**Supplement duration − > 12m**					
Bangladesh	RDNS (36)	550	272	1.52 (1.40, 1.64)	0.38
Bangladesh	WASH−B (37)	212	178	1.74 (1.50, 2.01)	0.29
Kenya	WASH−B (42)	298	259	1.69 (1.50, 1.91)	0.33
Mali	PROMIS CS (46)				
**I² = 0.48, Tau² = 0.00**		**1060**	**709**	**1.62 (1.49, 1.76)**	

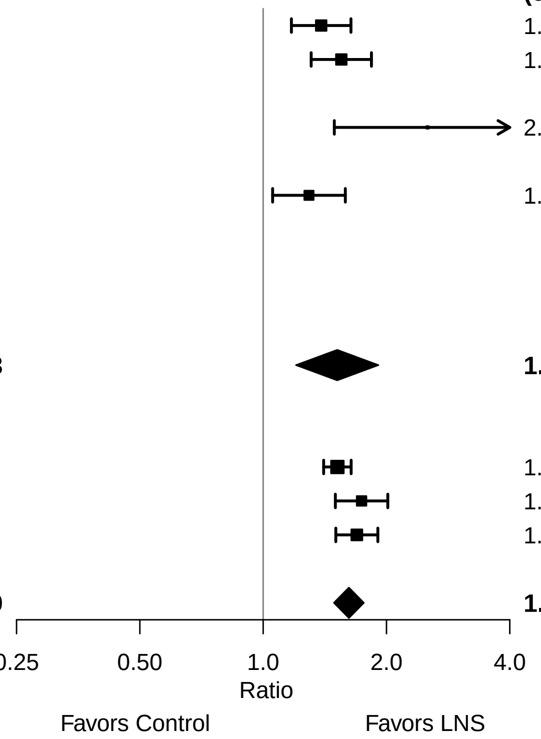

Supplemental figure 6F: Geometric mean ratio of ferritin concentration

6F8: Stratified by Iron dose

Iron dose
(p−diff = 0.054)

Country	Trial	N	N	GMR (95% CI)	W
**Iron dose − Less than 9 mg**					
Bangladesh	JiVitA−4 (35)	455	144	1.39 (1.17, 1.64)	0.35
Burkina Faso	iLiNS−Zinc (38)	315	96	1.55 (1.31, 1.84)	0.35
Burkina Faso	PROMIS CS (39)				
Ghana	iLiNS−DYAD−G (41)				
Madagascar	MAHAY (43)	83	51	1.29 (1.05, 1.59)	0.30
Malawi	iLiNS−DYAD−M (44)				
Malawi	iLiNS−DOSE (45)				
Mali	PROMIS CS (46)				
Zimbabwe	SHINE (HIV−) (47)				
Zimbabwe	SHINE (HIV+) (48)				
**I² = 0.00, Tau² = 0.00**		**853**	**291**	**1.42 (1.28, 1.57)**	
**Iron dose − 9 mg**					
Bangladesh	RDNS (36)	550	272	1.52 (1.40, 1.64)	0.35
Bangladesh	WASH−B (37)	212	178	1.74 (1.50, 2.01)	0.28
Ghana	GHANA (40)	83	82	2.52 (1.49, 4.25)	0.06
Kenya	WASH−B (42)	298	259	1.69 (1.50, 1.91)	0.31
**I² = 0.56, Tau² = 0.01**		**1143**	**791**	**1.67 (1.47, 1.90)**	

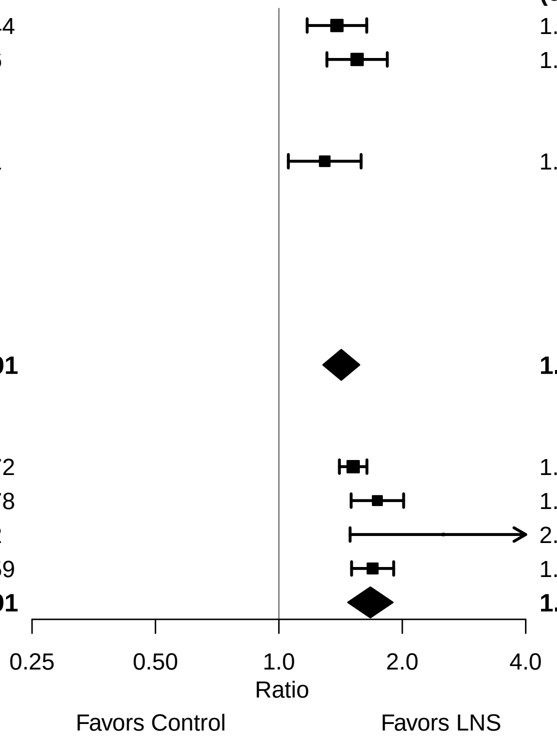

Supplemental figure 6F: Geometric mean ratio of ferritin concentration

6F9: Stratified by Frequency of contact

Frequency of contact
(p−diff = 0.584)

Country	Trial	N	N	GMR (95% CI)	W
**Frequency of contact − Monthly**					
Bangladesh	RDNS (36)	550	272	1.52 (1.40, 1.64)	0.40
Burkina Faso	PROMIS CS (39)				
Kenya	WASH−B (42)	298	259	1.69 (1.50, 1.91)	0.35
Madagascar	MAHAY (43)	83	51	1.29 (1.05, 1.59)	0.24
Mali	PROMIS CS (46)				
Zimbabwe	SHINE (HIV−) (47)				
Zimbabwe	SHINE (HIV+) (48)				
**I^2 = 0.63, Tau^2 = 0.01**		**931**	**582**	**1.52 (1.32, 1.75)**	
**Frequency of contact − Weekly**					
Bangladesh	JiVitA−4 (35)	455	144	1.39 (1.17, 1.64)	0.30
Bangladesh	WASH−B (37)	212	178	1.74 (1.50, 2.01)	0.33
Burkina Faso	iLiNS−Zinc (38)	315	96	1.55 (1.31, 1.84)	0.30
Ghana	GHANA (40)	83	82	2.52 (1.49, 4.25)	0.07
Ghana	iLiNS−DYAD−G (41)				
Malawi	iLiNS−DYAD−M (44)				
Malawi	iLiNS−DOSE (45)				
**I^2 = 0.57, Tau^2 = 0.02**		**1065**	**500**	**1.63 (1.35, 1.97)**	

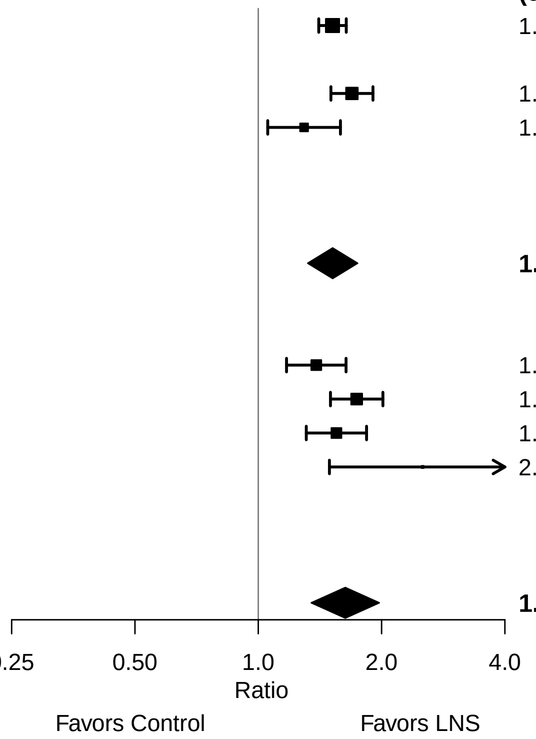

Supplemental figure 6F: Geometric mean ratio of ferritin concentration

6F10: Stratified by Average SQ-LNS compliance (insufficient comparisons)

Supplemental figure 6G: Iron deficiency (ferritin < 12 µg/L) prevalence ratio

6G1: Stratified by Geographic region

Geographic region
(p−diff = 0.016)

Country	Trial	N	N	PR (95% CI)	W
**Geographic region − SEAR**					
Bangladesh	JiVitA−4 (35)	455	144	0.36 (0.21, 0.59)	0.29
Bangladesh	RDNS (36)	550	272	0.34 (0.24, 0.48)	0.41
Bangladesh	WASH−B (37)	212	178	0.24 (0.15, 0.39)	0.30
**I² = 0.00, Tau² = 0.00**		**1217**	**594**	**0.31 (0.24, 0.40)**	
**Geographic region − AFR**					
Burkina Faso	iLiNS−Zinc (38)	315	96	0.61 (0.49, 0.78)	0.32
Burkina Faso	PROMIS CS (39)				
Ghana	GHANA (40)	83	82	0.49 (0.33, 0.73)	0.23
Ghana	iLiNS−DYAD−G (41)				
Kenya	WASH−B (42)	298	259	0.41 (0.32, 0.53)	0.31
Madagascar	MAHAY (43)	83	51	0.38 (0.21, 0.67)	0.15
Malawi	iLiNS−DYAD−M (44)				
Malawi	iLiNS−DOSE (45)				
Mali	PROMIS CS (46)				
Zimbabwe	SHINE (HIV−) (47)				
Zimbabwe	SHINE (HIV+) (48)				
**I² = 0.51, Tau² = 0.02**		**779**	**488**	**0.49 (0.40, 0.60)**	

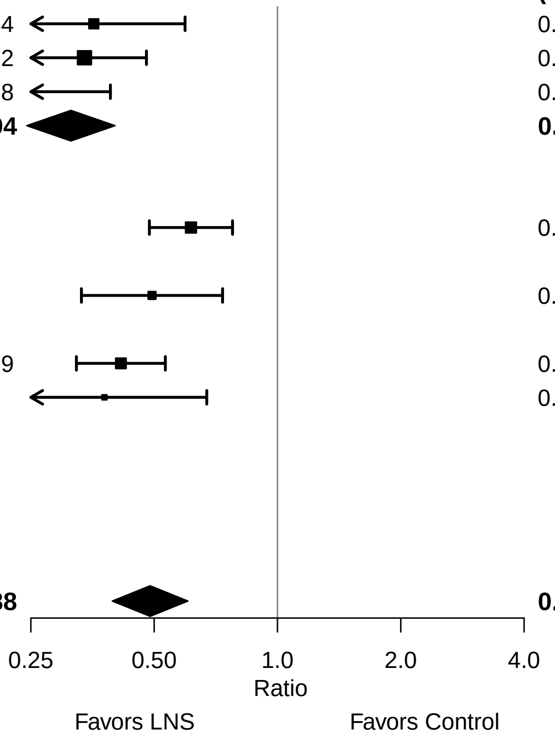

Supplemental figure 6G: Iron deficiency (ferritin < 12 µg/L) prevalence ratio

6G2: Stratified by Anemia burden (insufficient comparisons)

Supplemental figure 6G: Iron deficiency (ferritin < 12 µg/L) prevalence ratio

6G3: Stratified by Malaria prevalence (insufficient comparisons)

Supplemental figure 6G: Iron deficiency (ferritin < 12 µg/L) prevalence ratio

6G4: Stratified by Inflammation burden (insufficient comparisons)

Supplemental figure 6G: Iron deficiency (ferritin < 12 µg/L) prevalence ratio

6G5: Stratified by Source water quality

Source water quality
(p−diff = 0.105)

Country	Trial	N	N	PR (95% CI)	W
**Source water quality − Improved**					
Bangladesh	JiVitA−4 (35)	455	144	0.36 (0.21, 0.59)	0.21
Bangladesh	RDNS (36)	550	272	0.34 (0.24, 0.48)	0.36
Bangladesh	WASH−B (37)	94	58	0.31 (0.15, 0.64)	0.12
Ghana	GHANA (40)	83	82	0.49 (0.33, 0.73)	0.31
Ghana	iLiNS−DYAD−G (41)				
Malawi	iLiNS−DYAD−M (44)				
Malawi	iLiNS−DOSE (45)				
**I² = 0.00, Tau² = 0.00**		**1182**	**556**	**0.38 (0.31, 0.48)**	
**Source water quality − Unimproved**					
Burkina Faso	iLiNS−Zinc (38)	315	96	0.61 (0.49, 0.78)	0.49
Burkina Faso	PROMIS CS (39)				
Kenya	WASH−B (42)	166	144	0.45 (0.32, 0.63)	0.34
Madagascar	MAHAY (43)	83	51	0.38 (0.21, 0.67)	0.17
Mali	PROMIS CS (46)				
Zimbabwe	SHINE (HIV−) (47)				
Zimbabwe	SHINE (HIV+) (48)				
**I² = 0.47, Tau² = 0.02**		**564**	**291**	**0.51 (0.39, 0.66)**	

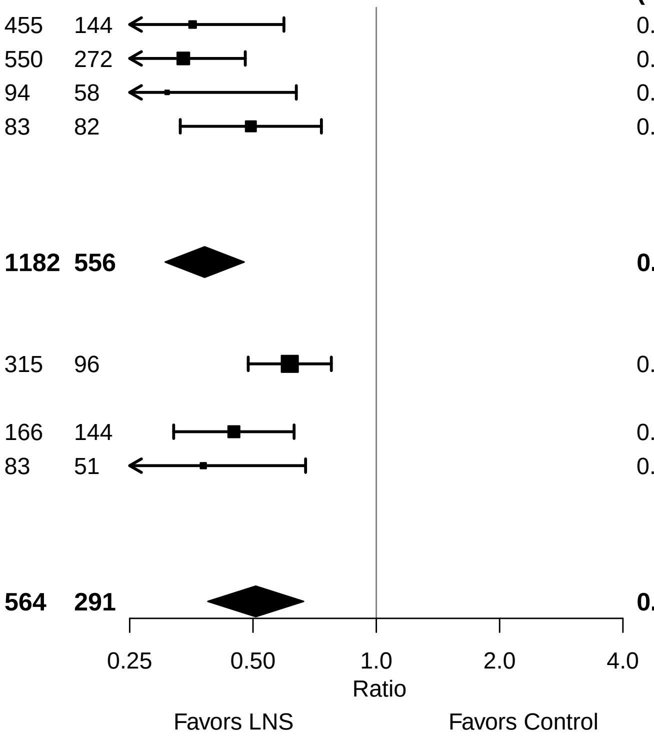

Supplemental figure 6G: Iron deficiency (ferritin < 12 µg/L) prevalence ratio

6G6: Stratified by Sanitation

Sanitation
(p−diff = 0.105)

Country	Trial	N	N	PR (95% CI)	W
**Sanitation − Improved**					
Bangladesh	JiVitA−4 (35)	455	144	0.36 (0.21, 0.59)	0.21
Bangladesh	RDNS (36)	550	272	0.34 (0.24, 0.48)	0.36
Bangladesh	WASH−B (37)	94	58	0.31 (0.15, 0.64)	0.12
Burkina Faso	PROMIS CS (39)				
Ghana	GHANA (40)	83	82	0.49 (0.33, 0.73)	0.31
Ghana	iLiNS−DYAD−G (41)				
Mali	PROMIS CS (46)				
**I² = 0.00, Tau² = 0.00**		**1182**	**556**	**0.38 (0.31, 0.48)**	
**Sanitation − Unimproved**					
Burkina Faso	iLiNS−Zinc (38)	315	96	0.61 (0.49, 0.78)	0.49
Kenya	WASH−B (42)	166	144	0.45 (0.32, 0.63)	0.34
Madagascar	MAHAY (43)	83	51	0.38 (0.21, 0.67)	0.17
Malawi	iLiNS−DYAD−M (44)				
Malawi	iLiNS−DOSE (45)				
Zimbabwe	SHINE (HIV−) (47)				
Zimbabwe	SHINE (HIV+) (48)				
**I² = 0.47, Tau² = 0.02**		**564**	**291**	**0.51 (0.39, 0.66)**	

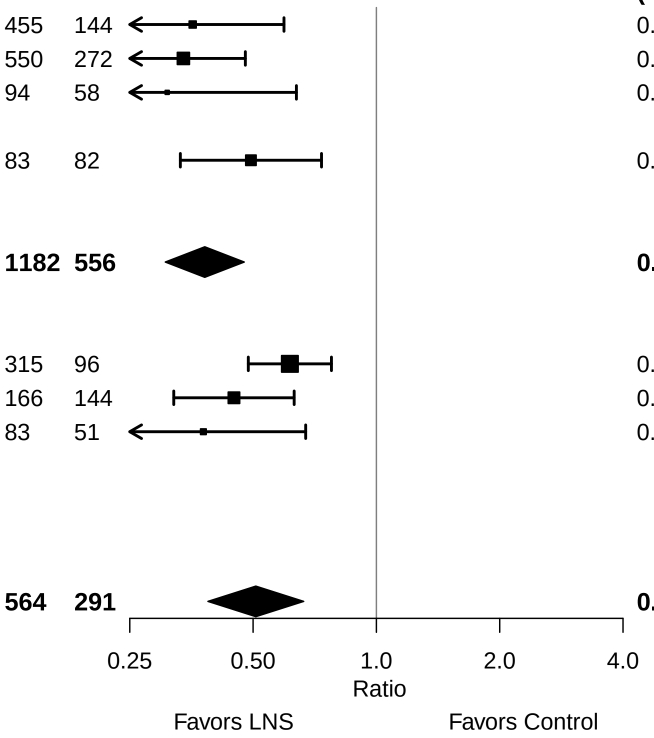

Supplemental figure 6G: Iron deficiency (ferritin < 12 µg/L) prevalence ratio

6G7: Stratified by Supplement duration

Supplement duration
(p−diff = 0.081)

Country	Trial	N	N	PR (95% CI)	W
**Supplement duration − 12m or less**					
Bangladesh	JiVitA−4 (35)	455	144	0.36 (0.21, 0.59)	0.20
Burkina Faso	iLiNS−Zinc (38)	315	96	0.61 (0.49, 0.78)	0.36
Burkina Faso	PROMIS CS (39)				
Ghana	GHANA (40)	83	82	0.49 (0.33, 0.73)	0.26
Ghana	iLiNS−DYAD−G (41)				
Madagascar	MAHAY (43)	83	51	0.38 (0.21, 0.67)	0.18
Malawi	iLiNS−DYAD−M (44)				
Malawi	iLiNS−DOSE (45)				
Zimbabwe	SHINE (HIV−) (47)				
Zimbabwe	SHINE (HIV+) (48)				
**I² = 0.43, Tau² = 0.03**		**936**	**373**	**0.49 (0.38, 0.63)**	
**Supplement duration − > 12m**					
Bangladesh	RDNS (36)	550	272	0.34 (0.24, 0.48)	0.34
Bangladesh	WASH−B (37)	212	178	0.24 (0.15, 0.39)	0.25
Kenya	WASH−B (42)	298	259	0.41 (0.32, 0.53)	0.41
Mali	PROMIS CS (46)				
**I² = 0.50, Tau² = 0.04**		**1060**	**709**	**0.34 (0.25, 0.46)**	

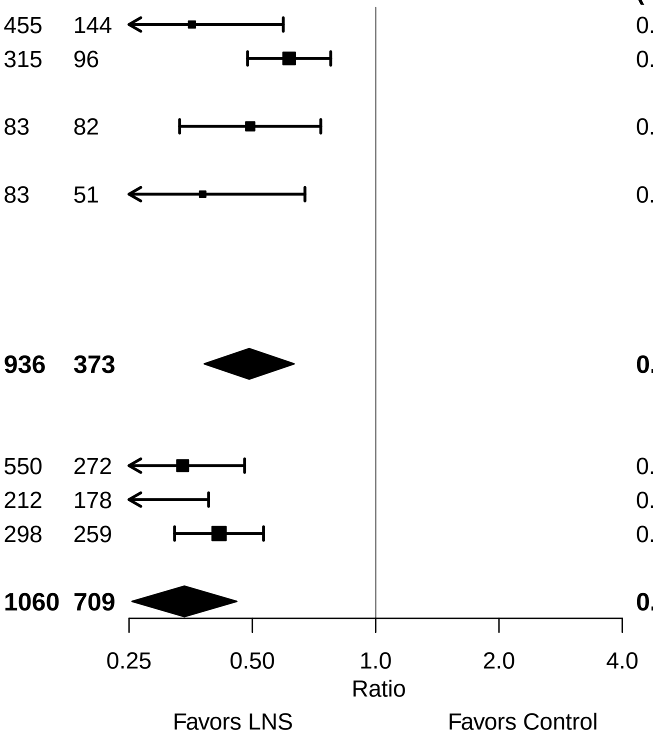

Supplemental figure 6G: Iron deficiency (ferritin < 12 µg/L) prevalence ratio

6G8: Stratified by Iron dose

Iron dose
(p−diff = 0.278)

Country	Trial	N	N	PR (95% CI)	W
**Iron dose − Less than 9 mg**					
Bangladesh	JiVitA−4 (35)	455	144	0.36 (0.21, 0.59)	0.27
Burkina Faso	iLiNS−Zinc (38)	315	96	0.61 (0.49, 0.78)	0.49
Burkina Faso	PROMIS CS (39)				
Ghana	iLiNS−DYAD−G (41)				
Madagascar	MAHAY (43)	83	51	0.38 (0.21, 0.67)	0.24
Malawi	iLiNS−DYAD−M (44)				
Malawi	iLiNS−DOSE (45)				
Mali	PROMIS CS (46)				
Zimbabwe	SHINE (HIV−) (47)				
Zimbabwe	SHINE (HIV+) (48)				
**I^2 = 0.61, Tau^2 = 0.06**		**853**	**291**	**0.47 (0.33, 0.68)**	
**Iron dose − 9 mg**					
Bangladesh	RDNS (36)	550	272	0.34 (0.24, 0.48)	0.26
Bangladesh	WASH−B (37)	212	178	0.24 (0.15, 0.39)	0.19
Ghana	GHANA (40)	83	82	0.49 (0.33, 0.73)	0.23
Kenya	WASH−B (42)	298	259	0.41 (0.32, 0.53)	0.32
**I^2 = 0.50, Tau^2 = 0.05**		**1143**	**791**	**0.37 (0.28, 0.49)**	

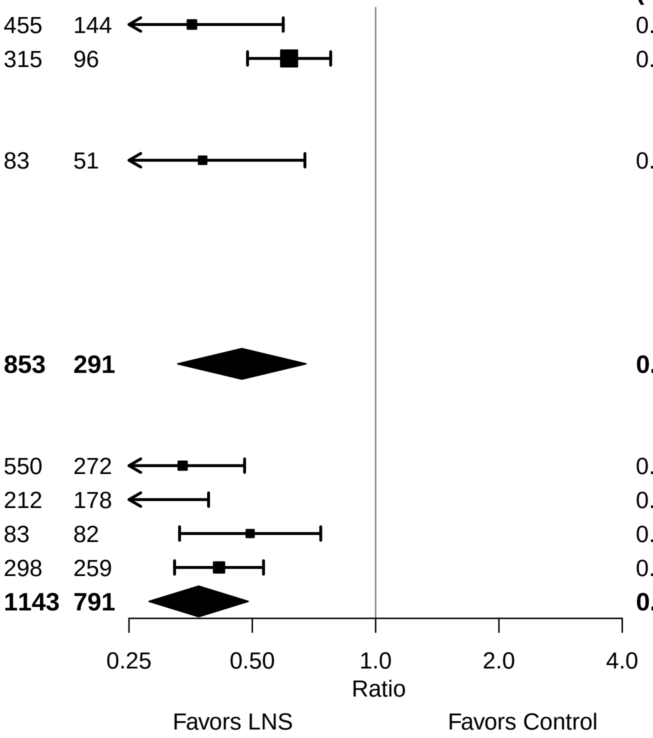

Supplemental figure 6G: Iron deficiency (ferritin < 12 µg/L) prevalence ratio

6G9: Stratified by Frequency of contact

Frequency of contact
(p−diff = 0.624)

Country	Trial	N	N	PR (95% CI)	W
**Frequency of contact − Monthly**					
Bangladesh	RDNS (36)	550	272	0.34 (0.24, 0.48)	0.35
Burkina Faso	PROMIS CS (39)				
Kenya	WASH−B (42)	298	259	0.41 (0.32, 0.53)	0.43
Madagascar	MAHAY (43)	83	51	0.38 (0.21, 0.67)	0.21
Mali	PROMIS CS (46)				
Zimbabwe	SHINE (HIV−) (47)				
Zimbabwe	SHINE (HIV+) (48)				
**I² = 0.00, Tau² = 0.00**		**931**	**582**	**0.39 (0.32, 0.47)**	
**Frequency of contact − Weekly**					
Bangladesh	JiVitA−4 (35)	455	144	0.36 (0.21, 0.59)	0.19
Bangladesh	WASH−B (37)	212	178	0.24 (0.15, 0.39)	0.20
Burkina Faso	iLiNS−Zinc (38)	315	96	0.61 (0.49, 0.78)	0.35
Ghana	GHANA (40)	83	82	0.49 (0.33, 0.73)	0.25
Ghana	iLiNS−DYAD−G (41)				
Malawi	iLiNS−DYAD−M (44)				
Malawi	iLiNS−DOSE (45)				
**I² = 0.77, Tau² = 0.13**		**1065**	**500**	**0.42 (0.28, 0.63)**	

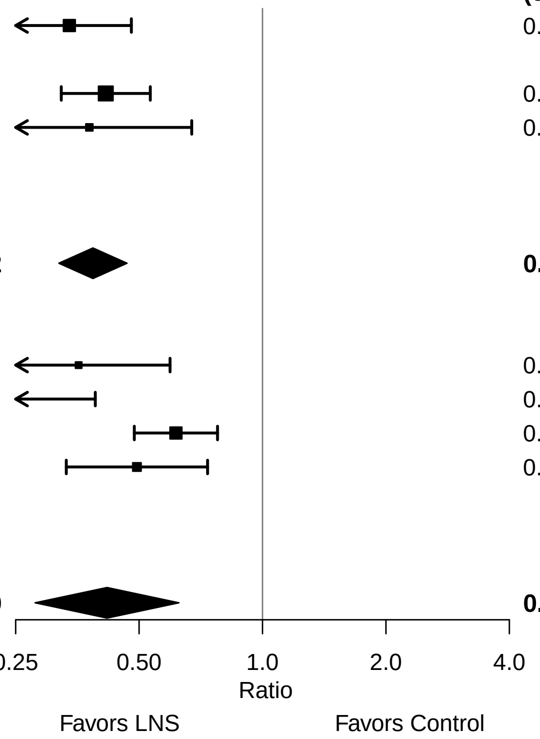

Supplemental figure 6G: Iron deficiency (ferritin < 12 μg/L) prevalence ratio

6G10: Stratified by Average SQ-LNS compliance (insufficient comparisons)

Supplemental figure 6H: Iron deficiency (ferritin < 12 µg/L) prevalence difference

6H1: Stratified by Geographic region

Geographic region
(p−diff = 0.026)

Country	Trial	N	N	PD (95% CI)	W
**Geographic region − SEAR**					
Bangladesh	JiVitA−4 (35)	455	144	−0.14 (−0.22, −0.07)	0.33
Bangladesh	RDNS (36)	550	272	−0.15 (−0.20, −0.09)	0.36
Bangladesh	WASH−B (37)	212	178	−0.27 (−0.35, −0.19)	0.31
**I² = 0.72, Tau² = 0.00**		**1217**	**594**	**−0.18 (−0.26, −0.10)**	
**Geographic region − AFR**					
Burkina Faso	iLiNS−Zinc (38)	315	96	−0.23 (−0.33, −0.12)	0.27
Burkina Faso	PROMIS CS (39)				
Ghana	GHANA (40)	83	82	−0.28 (−0.43, −0.14)	0.21
Ghana	iLiNS−DYAD−G (41)				
Kenya	WASH−B (42)	298	259	−0.35 (−0.42, −0.27)	0.34
Madagascar	MAHAY (43)	83	51	−0.32 (−0.48, −0.15)	0.18
Malawi	iLiNS−DYAD−M (44)				
Malawi	iLiNS−DOSE (45)				
Mali	PROMIS CS (46)				
Zimbabwe	SHINE (HIV−) (47)				
Zimbabwe	SHINE (HIV+) (48)				
**I² = 0.06, Tau² = 0.00**		**779**	**488**	**−0.30 (−0.36, −0.25)**	

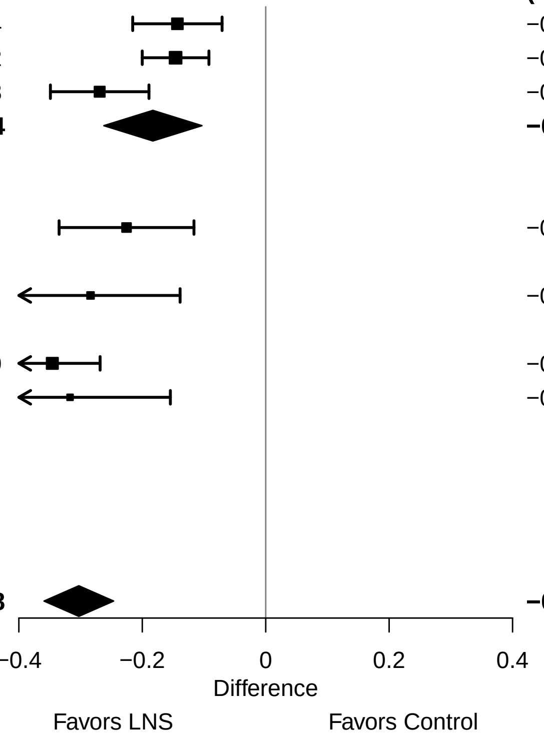

Supplemental figure 6H: Iron deficiency (ferritin < 12 µg/L) prevalence difference

6H2: Stratified by Anemia burden (insufficient comparisons)

Supplemental figure 6H: Iron deficiency (ferritin $<$ 12 µg/L) prevalence difference

6H3: Stratified by Malaria prevalence (insufficient comparisons)

Supplemental figure 6H: Iron deficiency (ferritin < 12 µg/L) prevalence difference

6H4: Stratified by Inflammation burden (insufficient comparisons)

Supplemental figure 6H: Iron deficiency (ferritin < 12 μg/L) prevalence difference

6H5: Stratified by Source water quality

Source water quality
(p−diff = 0.006)

Country	Trial	N	N	PD (95% CI)	W
**Source water quality − Improved**					
Bangladesh	JiVitA−4 (35)	455	144	−0.14 (−0.22, −0.07)	0.31
Bangladesh	RDNS (36)	550	272	−0.15 (−0.20, −0.09)	0.36
Bangladesh	WASH−B (37)	94	58	−0.24 (−0.38, −0.10)	0.17
Ghana	GHANA (40)	83	82	−0.28 (−0.43, −0.14)	0.16
Ghana	iLiNS−DYAD−G (41)				
Malawi	iLiNS−DYAD−M (44)				
Malawi	iLiNS−DOSE (45)				
**I² = 0.33, Tau² = 0.00**		**1182**	**556**	**−0.18 (−0.24, −0.12)**	
**Source water quality − Unimproved**					
Burkina Faso	iLiNS−Zinc (38)	315	96	−0.23 (−0.33, −0.12)	0.38
Burkina Faso	PROMIS CS (39)				
Kenya	WASH−B (42)	166	144	−0.33 (−0.43, −0.22)	0.39
Madagascar	MAHAY (43)	83	51	−0.32 (−0.48, −0.15)	0.23
Mali	PROMIS CS (46)				
Zimbabwe	SHINE (HIV−) (47)				
Zimbabwe	SHINE (HIV+) (48)				
**I² = 0.00, Tau² = 0.00**		**564**	**291**	**−0.28 (−0.35, −0.22)**	

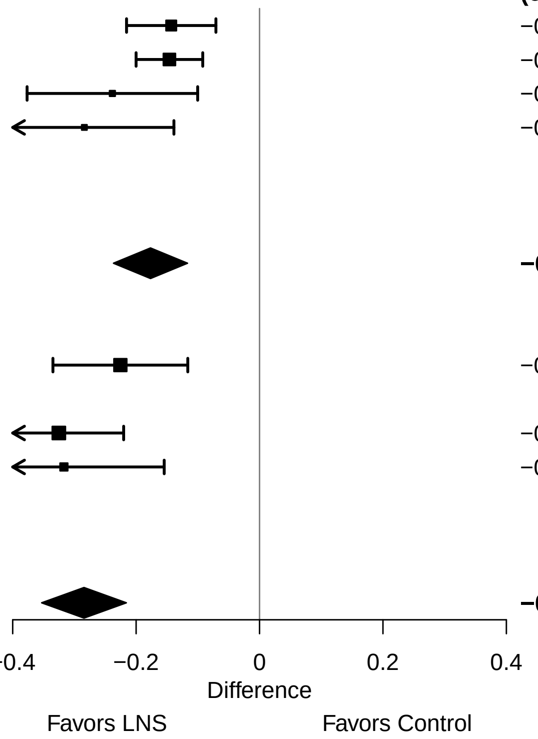

Supplemental figure 6H: Iron deficiency (ferritin < 12 µg/L) prevalence difference

6H6: Stratified by Sanitation

Sanitation
(p−diff = 0.006)

Country	Trial	N	N	PD (95% CI)	W
**Sanitation − Improved**					
Bangladesh	JiVitA−4 (35)	455	144	−0.14 (−0.22, −0.07)	0.31
Bangladesh	RDNS (36)	550	272	−0.15 (−0.20, −0.09)	0.36
Bangladesh	WASH−B (37)	94	58	−0.24 (−0.38, −0.10)	0.17
Burkina Faso	PROMIS CS (39)				
Ghana	GHANA (40)	83	82	−0.28 (−0.43, −0.14)	0.16
Ghana	iLiNS−DYAD−G (41)				
Mali	PROMIS CS (46)				
**I² = 0.33, Tau² = 0.00**		**1182**	**556**	**−0.18 (−0.24, −0.12)**	
**Sanitation − Unimproved**					
Burkina Faso	iLiNS−Zinc (38)	315	96	−0.23 (−0.33, −0.12)	0.38
Kenya	WASH−B (42)	166	144	−0.33 (−0.43, −0.22)	0.39
Madagascar	MAHAY (43)	83	51	−0.32 (−0.48, −0.15)	0.23
Malawi	iLiNS−DYAD−M (44)				
Malawi	iLiNS−DOSE (45)				
Zimbabwe	SHINE (HIV−) (47)				
Zimbabwe	SHINE (HIV+) (48)				
**I² = 0.00, Tau² = 0.00**		**564**	**291**	**−0.28 (−0.35, −0.22)**	

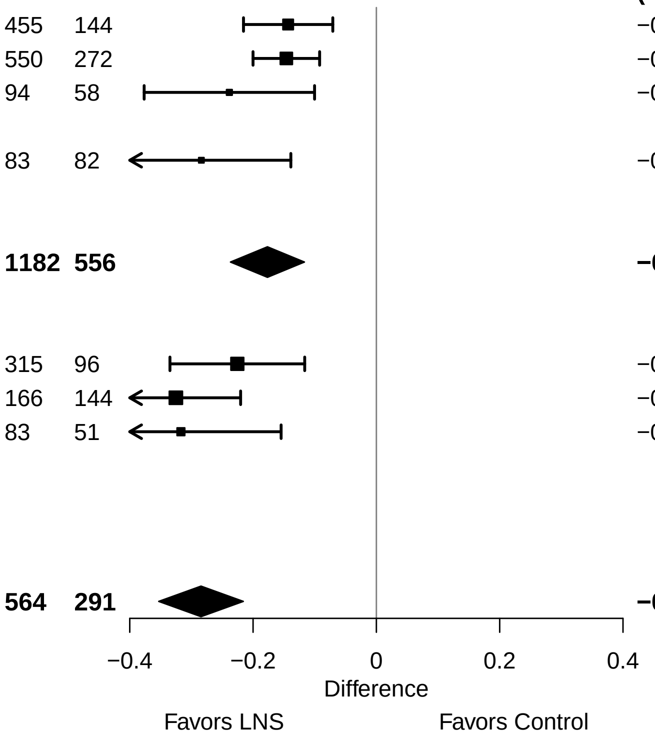

Supplemental figure 6H: Iron deficiency (ferritin < 12 µg/L) prevalence difference

6H7: Stratified by Supplement duration

Supplement duration
(p−diff = 0.767)

Country	Trial	N	N	PD (95% CI)	W
**Supplement duration − 12m or less**					
Bangladesh	JiVitA−4 (35)	455	144	−0.14 (−0.22, −0.07)	0.35
Burkina Faso	iLiNS−Zinc (38)	315	96	−0.23 (−0.33, −0.12)	0.27
Burkina Faso	PROMIS CS (39)				
Ghana	GHANA (40)	83	82	−0.28 (−0.43, −0.14)	0.20
Ghana	iLiNS−DYAD−G (41)				
Madagascar	MAHAY (43)	83	51	−0.32 (−0.48, −0.15)	0.18
Malawi	iLiNS−DYAD−M (44)				
Malawi	iLiNS−DOSE (45)				
Zimbabwe	SHINE (HIV−) (47)				
Zimbabwe	SHINE (HIV+) (48)				
**I^2 = 0.49, Tau^2 = 0.00**		**936**	**373**	**−0.22 (−0.30, −0.14)**	
**Supplement duration − > 12m**					
Bangladesh	RDNS (36)	550	272	−0.15 (−0.20, −0.09)	0.37
Bangladesh	WASH−B (37)	212	178	−0.27 (−0.35, −0.19)	0.31
Kenya	WASH−B (42)	298	259	−0.35 (−0.42, −0.27)	0.32
Mali	PROMIS CS (46)				
**I^2 = 0.89, Tau^2 = 0.01**		**1060**	**709**	**−0.25 (−0.37, −0.14)**	

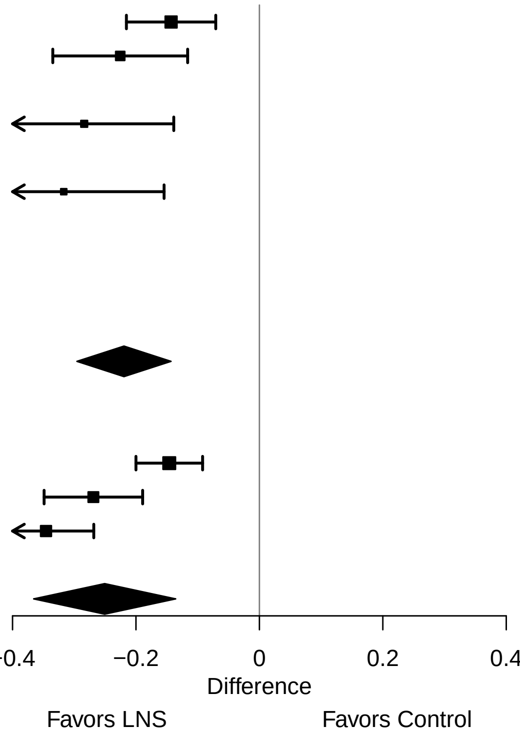

Supplemental figure 6H: Iron deficiency (ferritin < 12 µg/L) prevalence difference

6H8: Stratified by Iron dose

Iron dose
(p−diff = 0.548)

Country	Trial	N	N	PD (95% CI)	W
**Iron dose − Less than 9 mg**					
Bangladesh	JiVitA−4 (35)	455	144	−0.14 (−0.22, −0.07)	0.44
Burkina Faso	iLiNS−Zinc (38)	315	96	−0.23 (−0.33, −0.12)	0.34
Burkina Faso	PROMIS CS (39)				
Ghana	iLiNS−DYAD−G (41)				
Madagascar	MAHAY (43)	83	51	−0.32 (−0.48, −0.15)	0.23
Malawi	iLiNS−DYAD−M (44)				
Malawi	iLiNS−DOSE (45)				
Mali	PROMIS CS (46)				
Zimbabwe	SHINE (HIV−) (47)				
Zimbabwe	SHINE (HIV+) (48)				
**I² = 0.54, Tau² = 0.00**		**853**	**291**	**−0.21 (−0.30, −0.11)**	
**Iron dose − 9 mg**					
Bangladesh	RDNS (36)	550	272	−0.15 (−0.20, −0.09)	0.31
Bangladesh	WASH−B (37)	212	178	−0.27 (−0.35, −0.19)	0.26
Ghana	GHANA (40)	83	82	−0.28 (−0.43, −0.14)	0.16
Kenya	WASH−B (42)	298	259	−0.35 (−0.42, −0.27)	0.27
**I² = 0.84, Tau² = 0.01**		**1143**	**791**	**−0.26 (−0.34, −0.17)**	

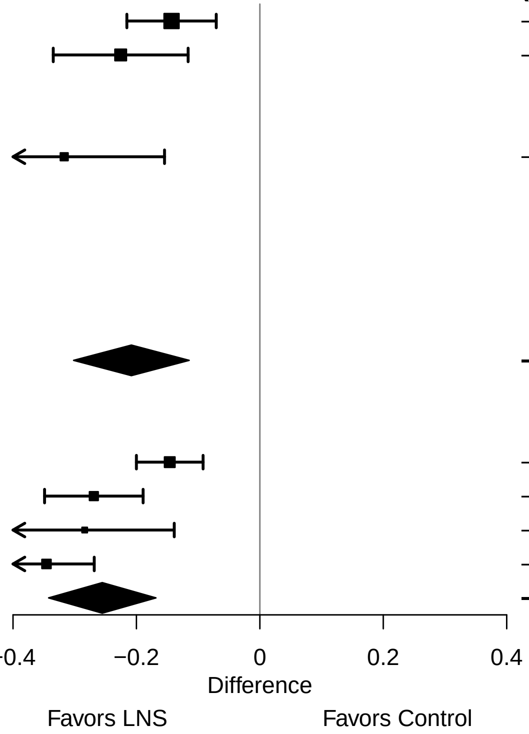

Supplemental figure 6H: Iron deficiency (ferritin < 12 µg/L) prevalence difference

6H9: Stratified by Frequency of contact

Frequency of contact
(p−diff = 0.650)

Country	Trial	N	N	PD (95% CI)	W
**Frequency of contact − Monthly**					
Bangladesh	RDNS (36)	550	272	−0.15 (−0.20, −0.09)	0.43
Burkina Faso	PROMIS CS (39)				
Kenya	WASH−B (42)	298	259	−0.35 (−0.42, −0.27)	0.37
Madagascar	MAHAY (43)	83	51	−0.32 (−0.48, −0.15)	0.20
Mali	PROMIS CS (46)				
Zimbabwe	SHINE (HIV−) (47)				
Zimbabwe	SHINE (HIV+) (48)				
**I² = 0.89, Tau² = 0.01**		**931**	**582**	**−0.26 (−0.39, −0.13)**	
**Frequency of contact − Weekly**					
Bangladesh	JiVitA−4 (35)	455	144	−0.14 (−0.22, −0.07)	0.30
Bangladesh	WASH−B (37)	212	178	−0.27 (−0.35, −0.19)	0.29
Burkina Faso	iLiNS−Zinc (38)	315	96	−0.23 (−0.33, −0.12)	0.23
Ghana	GHANA (40)	83	82	−0.28 (−0.43, −0.14)	0.18
Ghana	iLiNS−DYAD−G (41)				
Malawi	iLiNS−DYAD−M (44)				
Malawi	iLiNS−DOSE (45)				
**I² = 0.53, Tau² = 0.00**		**1065**	**500**	**−0.22 (−0.29, −0.16)**	

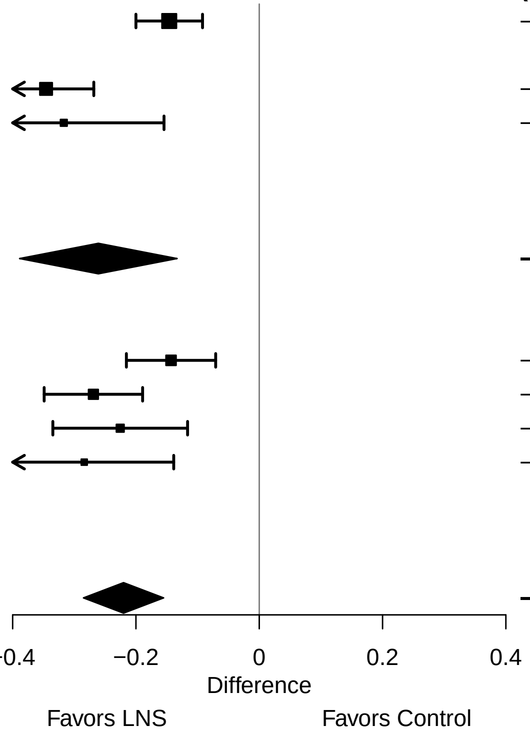

Supplemental figure 6H: Iron deficiency (ferritin < 12 µg/L) prevalence difference

6H10: Stratified by Average SQ-LNS compliance (insufficient comparisons)

Supplemental figure 6I: Iron deficiency anemia prevalence ratio

6I1: Stratified by Geographic region (insufficient comparisons)

Supplemental figure 6I: Iron deficiency anemia prevalence ratio

6I2: Stratified by Anemia burden (insufficient comparisons)

Supplemental figure 6I: Iron deficiency anemia prevalence ratio

6I3: Stratified by Malaria prevalence (insufficient comparisons)

Supplemental figure 6I: Iron deficiency anemia prevalence ratio

6I4: Stratified by Inflammation burden (insufficient comparisons)

Supplemental figure 6I: Iron deficiency anemia prevalence ratio

6I5: Stratified by Source water quality

Source water quality
(p−diff = 0.012)

Country	Trial	N	N	PR (95% CI)	W
**Source water quality − Improved**					
Bangladesh	JiVitA−4 (35)	454	144	0.12 (0.04, 0.34)	0.24
Bangladesh	RDNS (36)	549	272	0.25 (0.15, 0.41)	0.48
Bangladesh	WASH−B (37)				
Ghana	GHANA (40)	83	82	0.17 (0.07, 0.42)	0.28
Ghana	iLiNS−DYAD−G (41)				
Malawi	iLiNS−DYAD−M (44)				
Malawi	iLiNS−DOSE (45)				
**I² = 0.00, Tau² = 0.00**		**1086**	**498**	**0.20 (0.14, 0.31)**	
**Source water quality − Unimproved**					
Burkina Faso	iLiNS−Zinc (38)	327	101	0.51 (0.39, 0.67)	0.45
Burkina Faso	PROMIS CS (39)				
Kenya	WASH−B (42)	166	143	0.33 (0.19, 0.56)	0.33
Madagascar	MAHAY (43)	83	51	0.31 (0.13, 0.71)	0.22
Mali	PROMIS CS (46)				
Zimbabwe	SHINE (HIV−) (47)				
Zimbabwe	SHINE (HIV+) (48)				
**I² = 0.35, Tau² = 0.03**		**576**	**295**	**0.43 (0.31, 0.59)**	

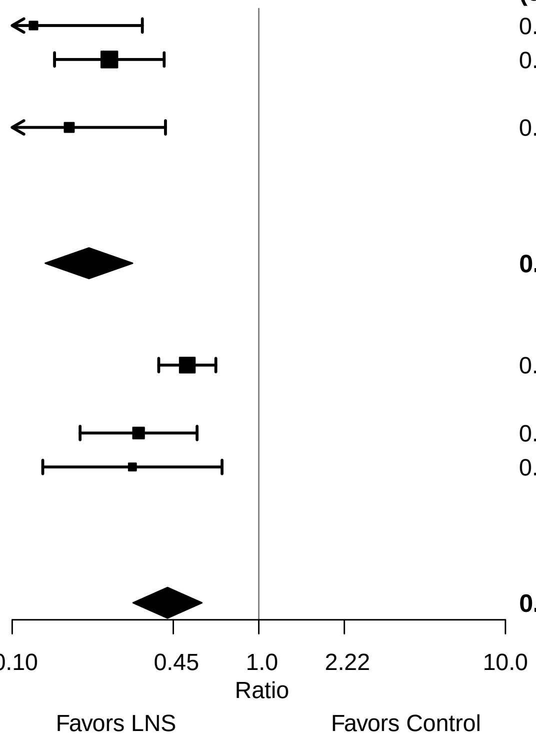

Supplemental figure 6I: Iron deficiency anemia prevalence ratio

6I6: Stratified by Sanitation

Sanitation
(p−diff = 0.012)

Country	Trial	N	N	PR (95% CI)	W
**Sanitation − Improved**					
Bangladesh	JiVitA−4 (35)	454	144	0.12 (0.04, 0.34)	0.24
Bangladesh	RDNS (36)	549	272	0.25 (0.15, 0.41)	0.48
Bangladesh	WASH−B (37)				
Burkina Faso	PROMIS CS (39)				
Ghana	GHANA (40)	83	82	0.17 (0.07, 0.42)	0.28
Ghana	iLiNS−DYAD−G (41)				
Mali	PROMIS CS (46)				
**I² = 0.00, Tau² = 0.00**		**1086**	**498**	**0.20 (0.14, 0.31)**	
**Sanitation − Unimproved**					
Burkina Faso	iLiNS−Zinc (38)	327	101	0.51 (0.39, 0.67)	0.45
Kenya	WASH−B (42)	166	143	0.33 (0.19, 0.56)	0.33
Madagascar	MAHAY (43)	83	51	0.31 (0.13, 0.71)	0.22
Malawi	iLiNS−DYAD−M (44)				
Malawi	iLiNS−DOSE (45)				
Zimbabwe	SHINE (HIV−) (47)				
Zimbabwe	SHINE (HIV+) (48)				
**I² = 0.35, Tau² = 0.03**		**576**	**295**	**0.43 (0.31, 0.59)**	

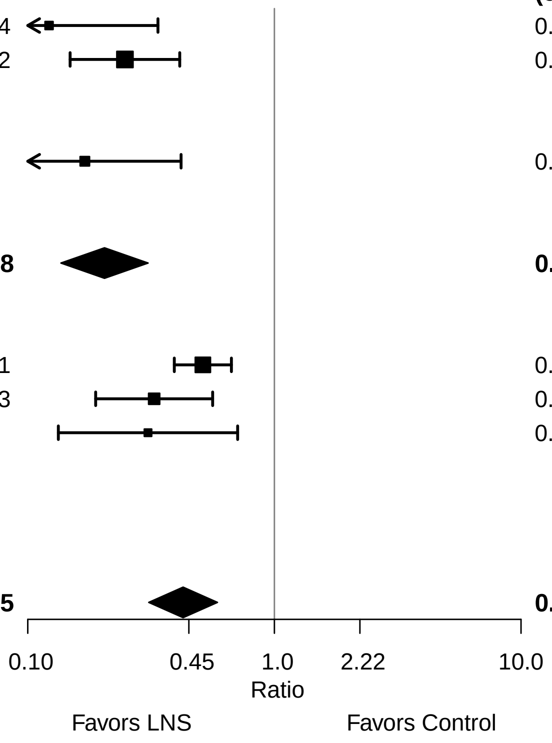

Supplemental figure 6I: Iron deficiency anemia prevalence ratio

6I7: Stratified by Supplement duration (insufficient comparisons)

Supplemental figure 6I: Iron deficiency anemia prevalence ratio

6I8: Stratified by Iron dose

Iron dose
(p−diff = 0.402)

Country	Trial	N	N	PR (95% CI)	W
**Iron dose − Less than 9 mg**					
Bangladesh	JiVitA−4 (35)	454	144	0.12 (0.04, 0.34)	0.20
Burkina Faso	iLiNS−Zinc (38)	327	101	0.51 (0.39, 0.67)	0.54
Burkina Faso	PROMIS CS (39)				
Ghana	iLiNS−DYAD−G (41)				
Madagascar	MAHAY (43)	83	51	0.31 (0.13, 0.71)	0.26
Malawi	iLiNS−DYAD−M (44)				
Malawi	iLiNS−DOSE (45)				
Mali	PROMIS CS (46)				
Zimbabwe	SHINE (HIV−) (47)				
Zimbabwe	SHINE (HIV+) (48)				
**I^2 = 0.75, Tau^2 = 0.37**		**864**	**296**	**0.30 (0.13, 0.67)**	
**Iron dose − 9 mg**					
Bangladesh	RDNS (36)	549	272	0.25 (0.15, 0.41)	0.38
Bangladesh	WASH−B (37)				
Ghana	GHANA (40)	83	82	0.17 (0.07, 0.42)	0.22
Kenya	WASH−B (42)	298	258	0.28 (0.18, 0.45)	0.40
**I^2 = 0.00, Tau^2 = 0.00**		**930**	**612**	**0.25 (0.18, 0.35)**	

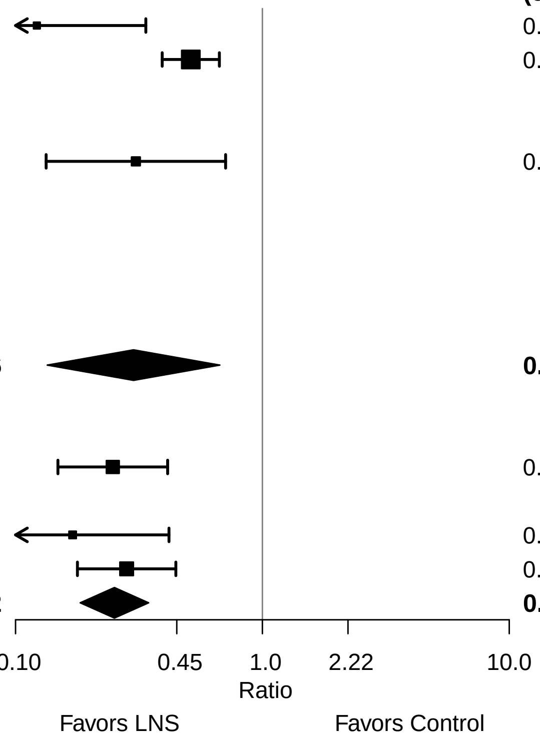

Supplemental figure 6I: Iron deficiency anemia prevalence ratio

6I9: Stratified by Frequency of contact

Frequency of contact
(p−diff = 0.981)

Country	Trial	N	N	PR (95% CI)	W
**Frequency of contact − Monthly**					
Bangladesh	RDNS (36)	549	272	0.25 (0.15, 0.41)	0.37
Burkina Faso	PROMIS CS (39)				
Kenya	WASH−B (42)	298	258	0.28 (0.18, 0.45)	0.40
Madagascar	MAHAY (43)	83	51	0.31 (0.13, 0.71)	0.23
Mali	PROMIS CS (46)				
Zimbabwe	SHINE (HIV−) (47)				
Zimbabwe	SHINE (HIV+) (48)				
**I² = 0.00, Tau² = 0.00**		**930**	**581**	**0.27 (0.20, 0.37)**	
**Frequency of contact − Weekly**					
Bangladesh	JiVitA−4 (35)	454	144	0.12 (0.04, 0.34)	0.21
Bangladesh	WASH−B (37)				
Burkina Faso	iLiNS−Zinc (38)	327	101	0.51 (0.39, 0.67)	0.55
Ghana	GHANA (40)	83	82	0.17 (0.07, 0.42)	0.24
Ghana	iLiNS−DYAD−G (41)				
Malawi	iLiNS−DYAD−M (44)				
Malawi	iLiNS−DOSE (45)				
**I² = 0.83, Tau² = 0.46**		**864**	**327**	**0.25 (0.10, 0.60)**	

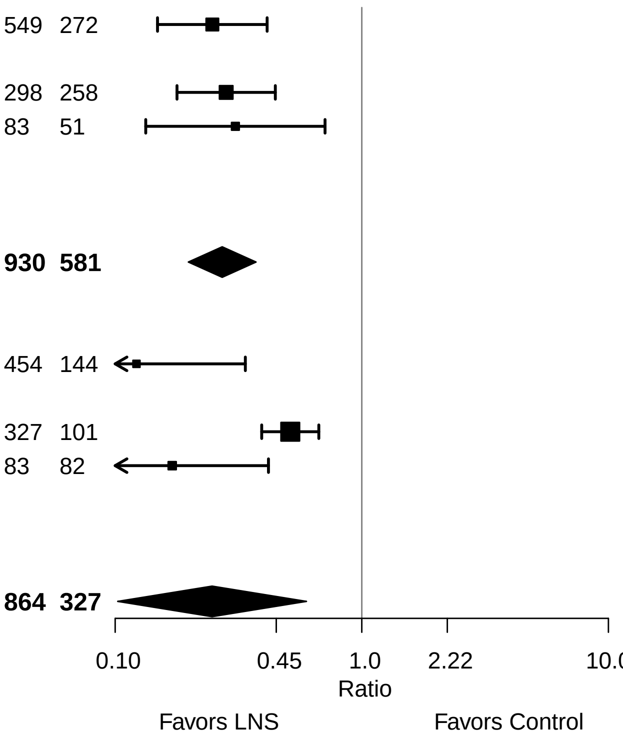

Supplemental figure 6I: Iron deficiency anemia prevalence ratio

6I10: Stratified by Average SQ-LNS compliance (insufficient comparisons)

Supplemental figure 6J: Iron deficiency anemia prevalence difference

6J1: Stratified by Geographic region (insufficient comparisons)

Supplemental figure 6J: Iron deficiency anemia prevalence difference

6J2: Stratified by Anemia burden (insufficient comparisons)

Supplemental figure 6J: Iron deficiency anemia prevalence difference

6J3: Stratified by Malaria prevalence (insufficient comparisons)

Supplemental figure 6J: Iron deficiency anemia prevalence difference

6J4: Stratified by Inflammation burden (insufficient comparisons)

Supplemental figure 6J: Iron deficiency anemia prevalence difference

6J5: Stratified by Source water quality

Source water quality
(p−diff = 0.222)

Country	Trial	N	N	PD (95% CI)	W
**Source water quality − Improved**					
Bangladesh	JiVitA−4 (35)	454	144	−0.08 (−0.13, −0.03)	0.37
Bangladesh	RDNS (36)	549	272	−0.11 (−0.16, −0.07)	0.38
Bangladesh	WASH−B (37)				
Ghana	GHANA (40)	83	82	−0.29 (−0.41, −0.18)	0.25
Ghana	iLiNS−DYAD−G (41)				
Malawi	iLiNS−DYAD−M (44)				
Malawi	iLiNS−DOSE (45)				
**I² = 0.82, Tau² = 0.01**		**1086**	**498**	**−0.15 (−0.28, −0.03)**	
**Source water quality − Unimproved**					
Burkina Faso	iLiNS−Zinc (38)	327	101	−0.27 (−0.37, −0.16)	0.34
Burkina Faso	PROMIS CS (39)				
Kenya	WASH−B (42)	166	143	−0.21 (−0.30, −0.12)	0.38
Madagascar	MAHAY (43)	83	51	−0.19 (−0.33, −0.05)	0.28
Mali	PROMIS CS (46)				
Zimbabwe	SHINE (HIV−) (47)				
Zimbabwe	SHINE (HIV+) (48)				
**I² = 0.00, Tau² = 0.00**		**576**	**295**	**−0.23 (−0.29, −0.16)**	

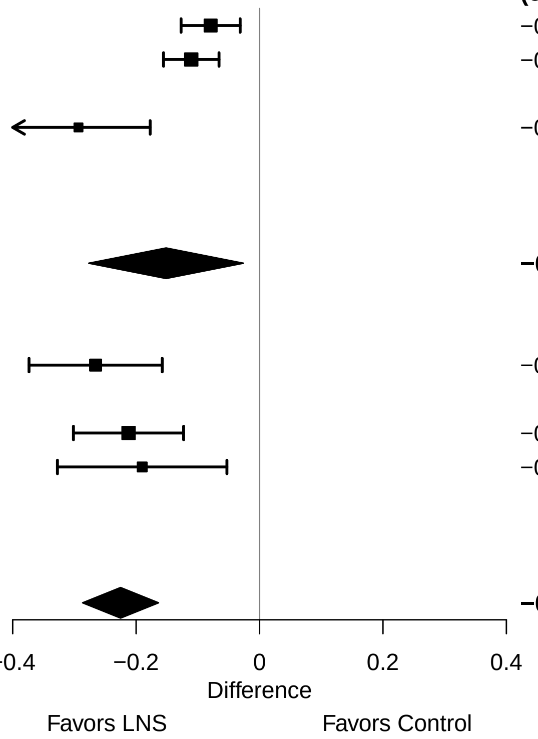

Supplemental figure 6J: Iron deficiency anemia prevalence difference

6J6: Stratified by Sanitation

Sanitation
(p−diff = 0.222)

Country	Trial	N	N	PD (95% CI)	W
**Sanitation − Improved**					
Bangladesh	JiVitA−4 (35)	454	144	−0.08 (−0.13, −0.03)	0.37
Bangladesh	RDNS (36)	549	272	−0.11 (−0.16, −0.07)	0.38
Bangladesh	WASH−B (37)				
Burkina Faso	PROMIS CS (39)				
Ghana	GHANA (40)	83	82	−0.29 (−0.41, −0.18)	0.25
Ghana	iLiNS−DYAD−G (41)				
Mali	PROMIS CS (46)				
**I² = 0.82, Tau² = 0.01**		**1086**	**498**	**−0.15 (−0.28, −0.03)**	
**Sanitation − Unimproved**					
Burkina Faso	iLiNS−Zinc (38)	327	101	−0.27 (−0.37, −0.16)	0.34
Kenya	WASH−B (42)	166	143	−0.21 (−0.30, −0.12)	0.38
Madagascar	MAHAY (43)	83	51	−0.19 (−0.33, −0.05)	0.28
Malawi	iLiNS−DYAD−M (44)				
Malawi	iLiNS−DOSE (45)				
Zimbabwe	SHINE (HIV−) (47)				
Zimbabwe	SHINE (HIV+) (48)				
**I² = 0.00, Tau² = 0.00**		**576**	**295**	**−0.23 (−0.29, −0.16)**	

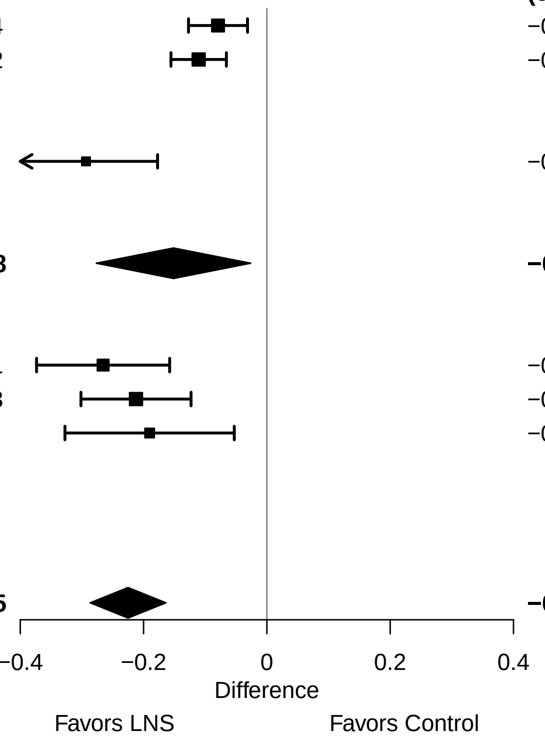

Supplemental figure 6J: Iron deficiency anemia prevalence difference

6J7: Stratified by Supplement duration (insufficient comparisons)

Supplemental figure 6J: Iron deficiency anemia prevalence difference

6J8: Stratified by Iron dose

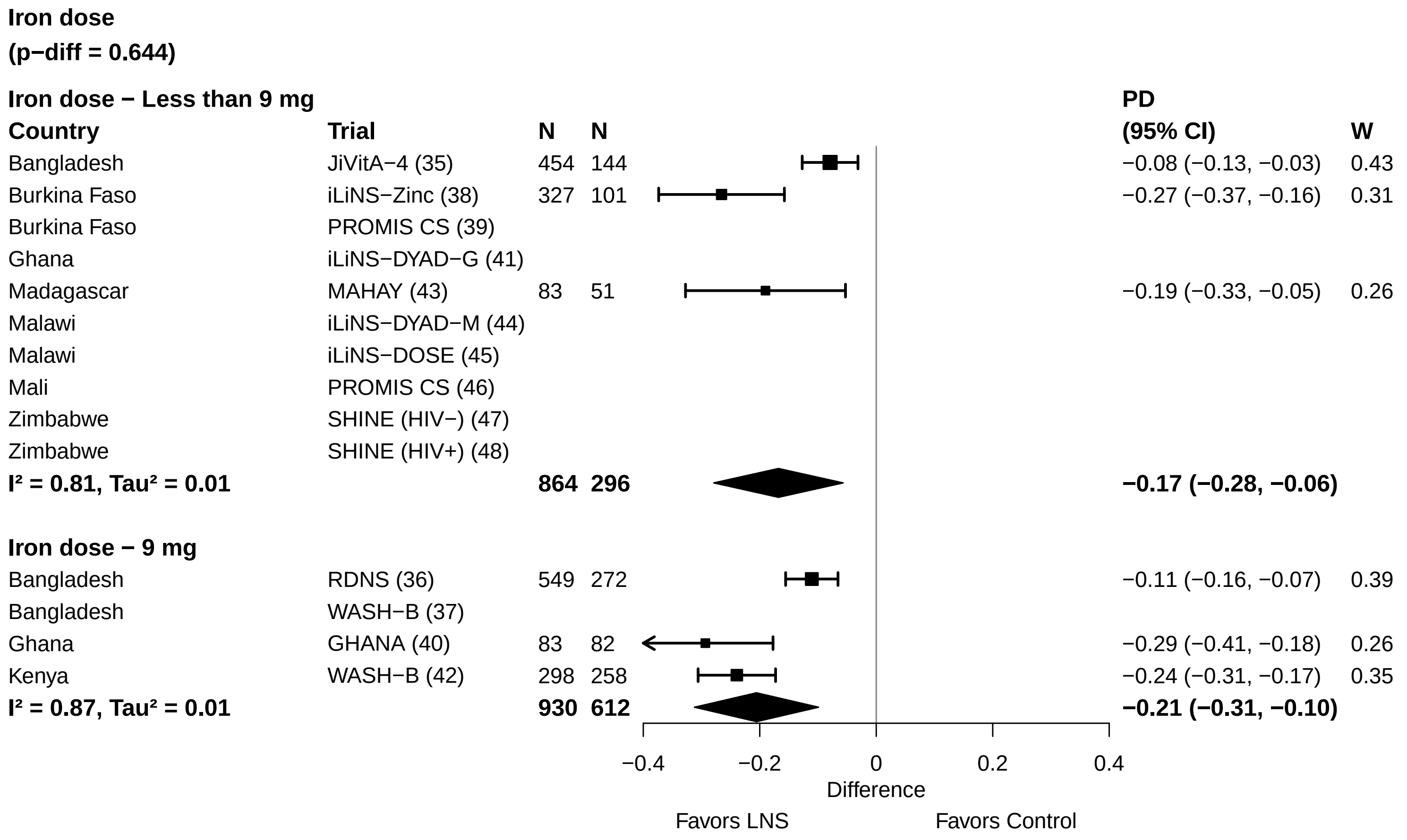

Supplemental figure 6J: Iron deficiency anemia prevalence difference

6J9: Stratified by Frequency of contact

Frequency of contact
(p−diff = 0.780)

Country	Trial	N	N	PD (95% CI)	W
**Frequency of contact − Monthly**					
Bangladesh	RDNS (36)	549	272	−0.11 (−0.16, −0.07)	0.40
Burkina Faso	PROMIS CS (39)				
Kenya	WASH−B (42)	298	258	−0.24 (−0.31, −0.17)	0.36
Madagascar	MAHAY (43)	83	51	−0.19 (−0.33, −0.05)	0.24
Mali	PROMIS CS (46)				
Zimbabwe	SHINE (HIV−) (47)				
Zimbabwe	SHINE (HIV+) (48)				
**I² = 0.80, Tau² = 0.00**		**930**	**581**	**−0.17 (−0.25, −0.09)**	
**Frequency of contact − Weekly**					
Bangladesh	JiVitA−4 (35)	454	144	−0.08 (−0.13, −0.03)	0.41
Bangladesh	WASH−B (37)				
Burkina Faso	iLiNS−Zinc (38)	327	101	−0.27 (−0.37, −0.16)	0.30
Ghana	GHANA (40)	83	82	−0.29 (−0.41, −0.18)	0.29
Ghana	iLiNS−DYAD−G (41)				
Malawi	iLiNS−DYAD−M (44)				
Malawi	iLiNS−DOSE (45)				
**I² = 0.89, Tau² = 0.01**		**864**	**327**	**−0.20 (−0.34, −0.07)**	

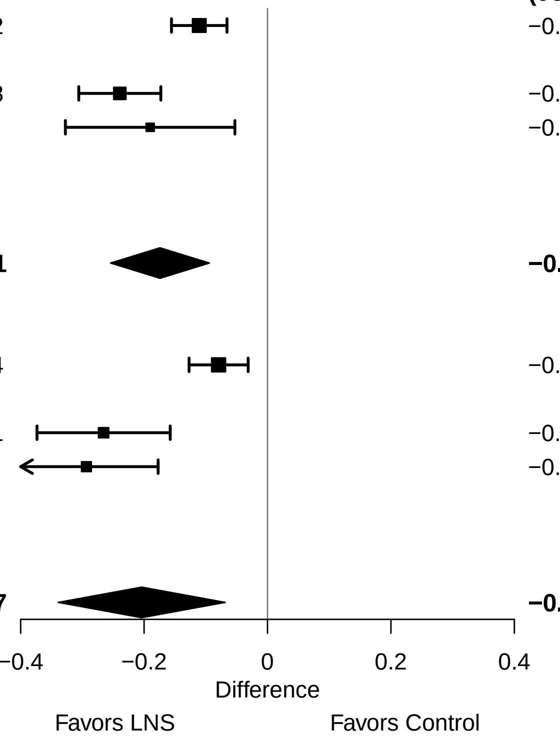

Supplemental figure 6J: Iron deficiency anemia prevalence difference

6J10: Stratified by Average SQ-LNS compliance (insufficient comparisons)

Supplemental figure 6K: Geometric mean ratio of soluble transferrin receptor concentration

6K1: Stratified by Geographic region (insufficient comparisons)

Supplemental figure 6K: Geometric mean ratio of soluble transferrin receptor concentration

6K2: Stratified by Anemia burden (insufficient comparisons)

Supplemental figure 6K: Geometric mean ratio of soluble transferrin receptor concentration

6K3: Stratified by Malaria prevalence (insufficient comparisons)

Supplemental figure 6K: Geometric mean ratio of soluble transferrin receptor concentration

6K4: Stratified by Inflammation burden (insufficient comparisons)

Supplemental figure 6K: Geometric mean ratio of soluble transferrin receptor concentration

6K5: Stratified by Source water quality

Source water quality
(p−diff = 0.779)

Country	Trial	N	N	GMR (95% CI)	W
**Source water quality − Improved**					
Bangladesh	JiVitA−4 (35)				
Bangladesh	RDNS (36)	550	272	0.87 (0.83, 0.92)	0.48
Bangladesh	WASH−B (37)	94	58	0.73 (0.63, 0.84)	0.22
Ghana	GHANA (40)	84	82	0.80 (0.72, 0.89)	0.30
Ghana	iLiNS−DYAD−G (41)				
Malawi	iLiNS−DYAD−M (44)				
Malawi	iLiNS−DOSE (45)				
**I² = 0.69, Tau² = 0.01**		**728**	**412**	**0.81 (0.73, 0.89)**	
**Source water quality − Unimproved**					
Burkina Faso	iLiNS−Zinc (38)	315	96	0.76 (0.68, 0.84)	0.35
Burkina Faso	PROMIS CS (39)				
Kenya	WASH−B (42)	166	144	0.78 (0.72, 0.85)	0.43
Madagascar	MAHAY (43)	83	51	0.90 (0.77, 1.05)	0.21
Mali	PROMIS CS (46)				
Zimbabwe	SHINE (HIV−) (47)				
Zimbabwe	SHINE (HIV+) (48)				
**I² = 0.37, Tau² = 0.00**		**564**	**291**	**0.80 (0.73, 0.87)**	

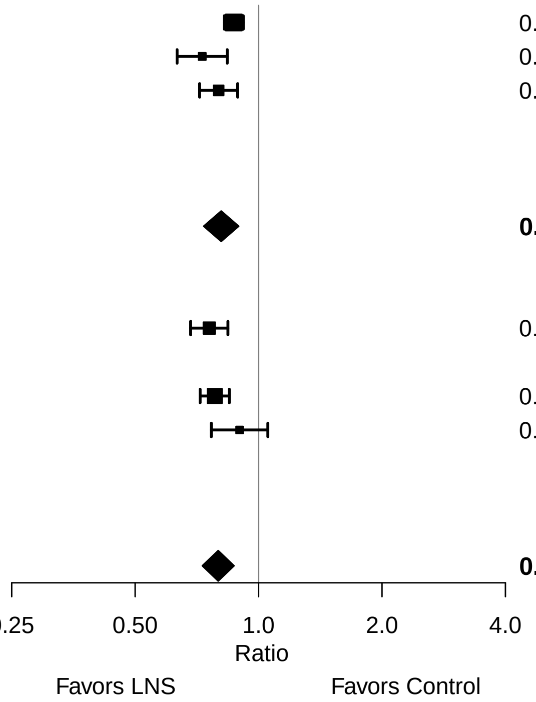

Supplemental figure 6K: Geometric mean ratio of soluble transferrin receptor concentration

6K6: Stratified by Sanitation

Sanitation
(p−diff = 0.779)

Country	Trial	N	N	GMR (95% CI)	W
**Sanitation − Improved**					
Bangladesh	JiVitA−4 (35)				
Bangladesh	RDNS (36)	550	272	0.87 (0.83, 0.92)	0.48
Bangladesh	WASH−B (37)	94	58	0.73 (0.63, 0.84)	0.22
Burkina Faso	PROMIS CS (39)				
Ghana	GHANA (40)	84	82	0.80 (0.72, 0.89)	0.30
Ghana	iLiNS−DYAD−G (41)				
Mali	PROMIS CS (46)				
**I² = 0.69, Tau² = 0.01**		**728**	**412**	**0.81 (0.73, 0.89)**	
**Sanitation − Unimproved**					
Burkina Faso	iLiNS−Zinc (38)	315	96	0.76 (0.68, 0.84)	0.35
Kenya	WASH−B (42)	166	144	0.78 (0.72, 0.85)	0.43
Madagascar	MAHAY (43)	83	51	0.90 (0.77, 1.05)	0.21
Malawi	iLiNS−DYAD−M (44)				
Malawi	iLiNS−DOSE (45)				
Zimbabwe	SHINE (HIV−) (47)				
Zimbabwe	SHINE (HIV+) (48)				
**I² = 0.37, Tau² = 0.00**		**564**	**291**	**0.80 (0.73, 0.87)**	

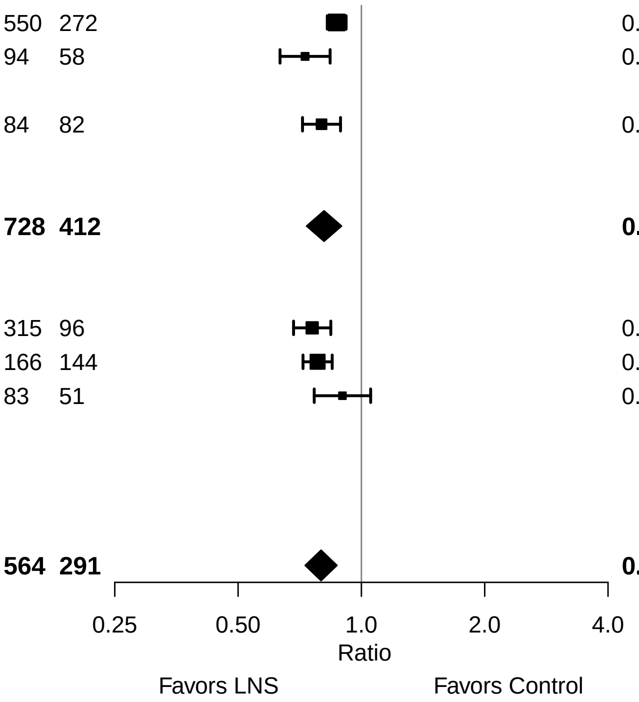

Supplemental figure 6K: Geometric mean ratio of soluble transferrin receptor concentration

6K7: Stratified by Supplement duration

Supplement duration
(p−diff = 0.539)

Supplement duration − 12m or less

**Country**	**Trial**	**N**	**N**	**GMR (95% CI)**	**W**
Bangladesh	JiVitA−4 (35)				
Burkina Faso	iLiNS−Zinc (38)	315	96	0.76 (0.68, 0.84)	0.40
Burkina Faso	PROMIS CS (39)				
Ghana	GHANA (40)	84	82	0.80 (0.72, 0.89)	0.39
Ghana	iLiNS−DYAD−G (41)				
Madagascar	MAHAY (43)	83	51	0.90 (0.77, 1.05)	0.21
Malawi	iLiNS−DYAD−M (44)				
Malawi	iLiNS−DOSE (45)				
Zimbabwe	SHINE (HIV−) (47)				
Zimbabwe	SHINE (HIV+) (48)				
**I² = 0.35, Tau² = 0.00**		**482**	**229**	**0.80 (0.74, 0.88)**	

Supplement duration − > 12m

Bangladesh	RDNS (36)	550	272	0.87 (0.83, 0.92)	0.38
Bangladesh	WASH−B (37)	212	178	0.82 (0.76, 0.87)	0.32
Kenya	WASH−B (42)	298	259	0.79 (0.74, 0.85)	0.30
Mali	PROMIS CS (46)				
**I² = 0.61, Tau² = 0.00**		**1060**	**709**	**0.83 (0.78, 0.88)**	

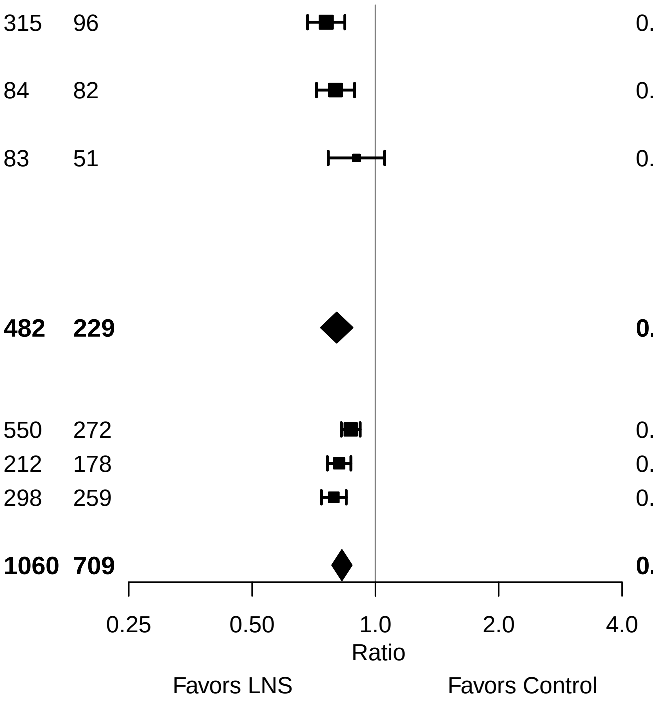

Supplemental figure 6K: Geometric mean ratio of soluble transferrin receptor concentration

6K8: Stratified by Iron dose (insufficient comparisons)

Supplemental figure 6K: Geometric mean ratio of soluble transferrin receptor concentration

6K9: Stratified by Frequency of contact

Frequency of contact
(p−diff = 0.208)

Country	Trial	N	N	GMR (95% CI)	W
**Frequency of contact − Monthly**					
Bangladesh	RDNS (36)	550	272	0.87 (0.83, 0.92)	0.49
Burkina Faso	PROMIS CS (39)				
Kenya	WASH−B (42)	298	259	0.79 (0.74, 0.85)	0.39
Madagascar	MAHAY (43)	83	51	0.90 (0.77, 1.05)	0.13
Mali	PROMIS CS (46)				
Zimbabwe	SHINE (HIV−) (47)				
Zimbabwe	SHINE (HIV+) (48)				
**I² = 0.61, Tau² = 0.00**		**931**	**582**	**0.84 (0.78, 0.91)**	
**Frequency of contact − Weekly**					
Bangladesh	JiVitA−4 (35)				
Bangladesh	WASH−B (37)	212	178	0.82 (0.76, 0.87)	0.46
Burkina Faso	iLiNS−Zinc (38)	315	96	0.76 (0.68, 0.84)	0.27
Ghana	GHANA (40)	84	82	0.80 (0.72, 0.89)	0.26
Ghana	iLiNS−DYAD−G (41)				
Malawi	iLiNS−DYAD−M (44)				
Malawi	iLiNS−DOSE (45)				
**I² = 0.00, Tau² = 0.00**		**611**	**356**	**0.80 (0.76, 0.84)**	

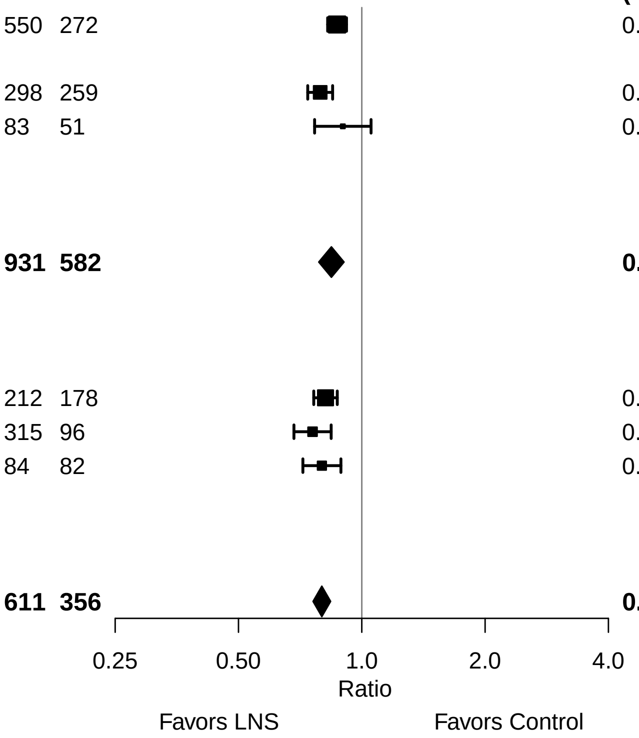

Supplemental figure 6K: Geometric mean ratio of soluble transferrin receptor concentration

6K10: Stratified by Average SQ-LNS compliance (insufficient comparisons)

Supplemental figure 6L: Elevated soluble transferrin receptor prevalence ratio

6L1: Stratified by Geographic region (insufficient comparisons)

Supplemental figure 6L: Elevated soluble transferrin receptor prevalence ratio

6L2: Stratified by Anemia burden (insufficient comparisons)

Supplemental figure 6L: Elevated soluble transferrin receptor prevalence ratio

6L3: Stratified by Malaria prevalence (insufficient comparisons)

Supplemental figure 6L: Elevated soluble transferrin receptor prevalence ratio

6L4: Stratified by Inflammation burden (insufficient comparisons)

Supplemental figure 6L: Elevated soluble transferrin receptor prevalence ratio

6L5: Stratified by Source water quality

Source water quality
(p−diff = 0.496)

Country	Trial	N	N	PR (95% CI)	W
**Source water quality − Improved**					
Bangladesh	JiVitA−4 (35)				
Bangladesh	RDNS (36)	550	272	0.70 (0.59, 0.84)	0.44
Bangladesh	WASH−B (37)	94	58	0.23 (0.11, 0.48)	0.20
Ghana	GHANA (40)	84	82	0.57 (0.41, 0.80)	0.37
Ghana	iLiNS−DYAD−G (41)				
Malawi	iLiNS−DYAD−M (44)				
Malawi	iLiNS−DOSE (45)				
**I^2 = 0.77, Tau^2 = 0.25**		**728**	**412**	**0.49 (0.26, 0.92)**	
**Source water quality − Unimproved**					
Burkina Faso	iLiNS−Zinc (38)	315	96	0.51 (0.38, 0.68)	0.32
Burkina Faso	PROMIS CS (39)				
Kenya	WASH−B (42)	166	144	0.64 (0.54, 0.75)	0.37
Madagascar	MAHAY (43)	83	51	0.82 (0.60, 1.12)	0.31
Mali	PROMIS CS (46)				
Zimbabwe	SHINE (HIV−) (47)				
Zimbabwe	SHINE (HIV+) (48)				
**I^2 = 0.57, Tau^2 = 0.03**		**564**	**291**	**0.64 (0.50, 0.82)**	

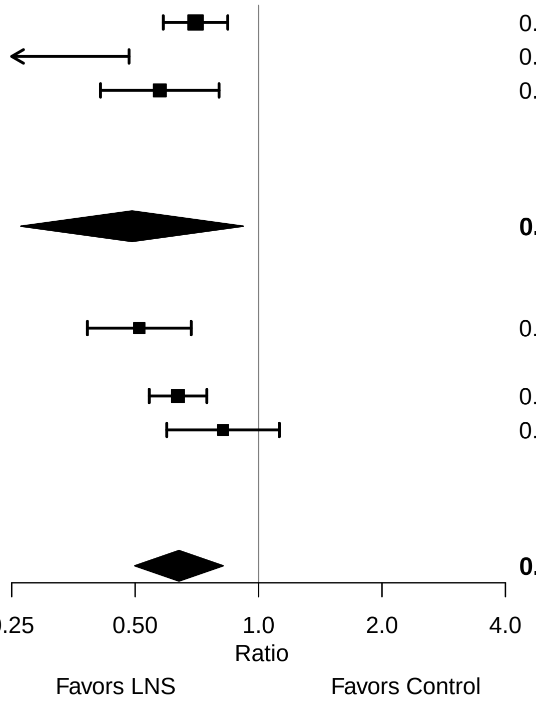

Supplemental figure 6L: Elevated soluble transferrin receptor prevalence ratio

6L6: Stratified by Sanitation

Sanitation
(p−diff = 0.496)

Country	Trial	N	N	PR (95% CI)	W
**Sanitation − Improved**					
Bangladesh	JiVitA−4 (35)				
Bangladesh	RDNS (36)	550	272	0.70 (0.59, 0.84)	0.44
Bangladesh	WASH−B (37)	94	58	0.23 (0.11, 0.48)	0.20
Burkina Faso	PROMIS CS (39)				
Ghana	GHANA (40)	84	82	0.57 (0.41, 0.80)	0.37
Ghana	iLiNS−DYAD−G (41)				
Mali	PROMIS CS (46)				
**I² = 0.77, Tau² = 0.25**		**728**	**412**	**0.49 (0.26, 0.92)**	
**Sanitation − Unimproved**					
Burkina Faso	iLiNS−Zinc (38)	315	96	0.51 (0.38, 0.68)	0.32
Kenya	WASH−B (42)	166	144	0.64 (0.54, 0.75)	0.37
Madagascar	MAHAY (43)	83	51	0.82 (0.60, 1.12)	0.31
Malawi	iLiNS−DYAD−M (44)				
Malawi	iLiNS−DOSE (45)				
Zimbabwe	SHINE (HIV−) (47)				
Zimbabwe	SHINE (HIV+) (48)				
**I² = 0.57, Tau² = 0.03**		**564**	**291**	**0.64 (0.50, 0.82)**	

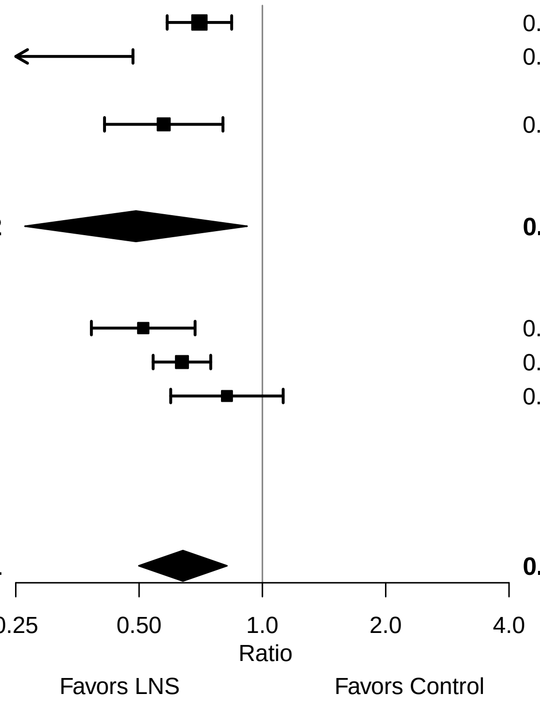

Supplemental figure 6L: Elevated soluble transferrin receptor prevalence ratio

6L7: Stratified by Supplement duration

Supplement duration
(p−diff = 0.692)

Country	Trial	N	N	PR (95% CI)	W
**Supplement duration − 12m or less**					
Bangladesh	JiVitA−4 (35)				
Burkina Faso	iLiNS−Zinc (38)	315	96	0.51 (0.38, 0.68)	0.34
Burkina Faso	PROMIS CS (39)				
Ghana	GHANA (40)	84	82	0.57 (0.41, 0.80)	0.32
Ghana	iLiNS−DYAD−G (41)				
Madagascar	MAHAY (43)	83	51	0.82 (0.60, 1.12)	0.33
Malawi	iLiNS−DYAD−M (44)				
Malawi	iLiNS−DOSE (45)				
Zimbabwe	SHINE (HIV−) (47)				
Zimbabwe	SHINE (HIV+) (48)				
**I² = 0.59, Tau² = 0.04**		**482**	**229**	**0.62 (0.47, 0.82)**	
**Supplement duration − > 12m**					
Bangladesh	RDNS (36)	550	272	0.70 (0.59, 0.84)	0.36
Bangladesh	WASH−B (37)	212	178	0.31 (0.21, 0.47)	0.26
Kenya	WASH−B (42)	298	259	0.67 (0.59, 0.77)	0.38
Mali	PROMIS CS (46)				
**I² = 0.85, Tau² = 0.17**		**1060**	**709**	**0.55 (0.33, 0.89)**	

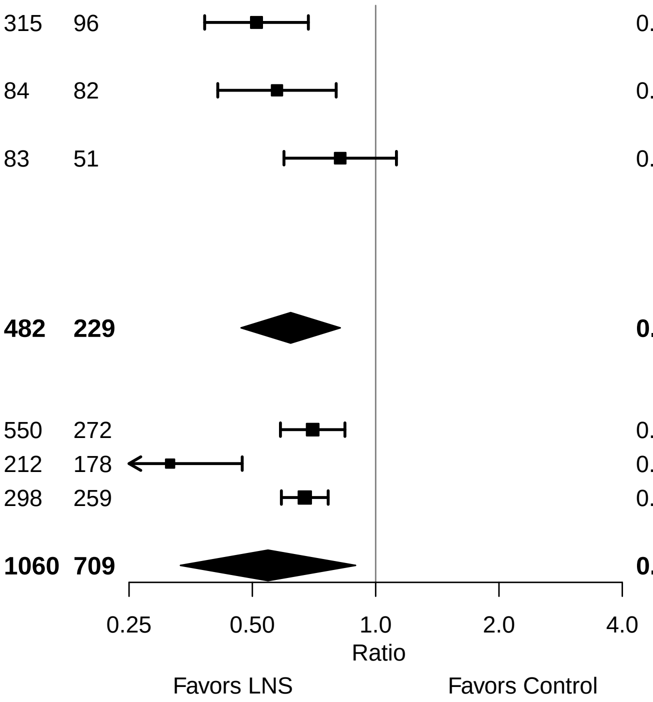

Supplemental figure 6L: Elevated soluble transferrin receptor prevalence ratio

6L8: Stratified by Iron dose (insufficient comparisons)

Supplemental figure 6L: Elevated soluble transferrin receptor prevalence ratio

6L9: Stratified by Frequency of contact

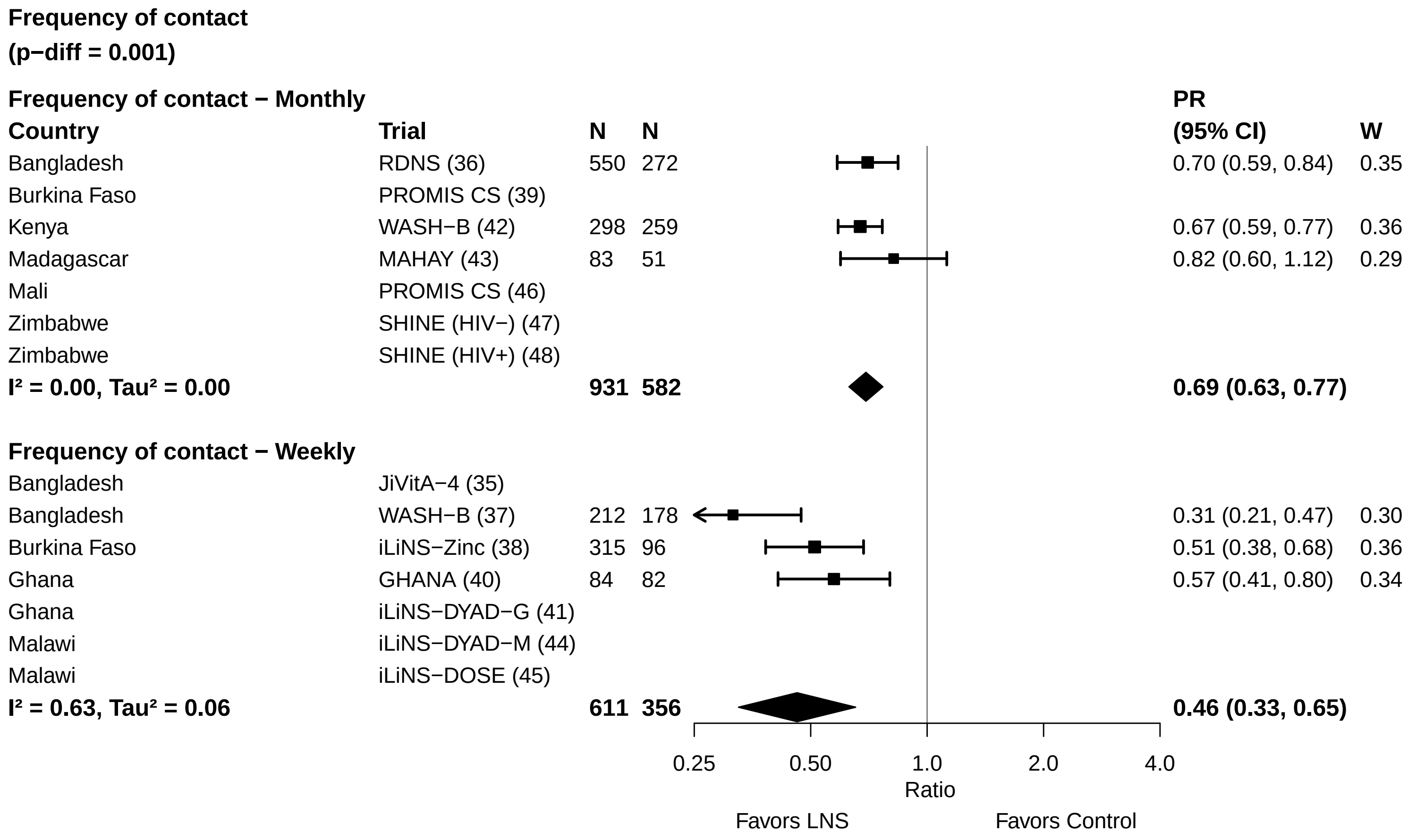

Supplemental figure 6L: Elevated soluble transferrin receptor prevalence ratio

6L10: Stratified by Average SQ-LNS compliance (insufficient comparisons)

Supplemental figure 6M: Elevated soluble transferrin receptor prevalence difference

6M1: Stratified by Geographic region (insufficient comparisons)

Supplemental figure 6M: Elevated soluble transferrin receptor prevalence difference

6M2: Stratified by Anemia burden (insufficient comparisons)

Supplemental figure 6M: Elevated soluble transferrin receptor prevalence difference

6M3: Stratified by Malaria prevalence (insufficient comparisons)

Supplemental figure 6M: Elevated soluble transferrin receptor prevalence difference

6M4: Stratified by Inflammation burden (insufficient comparisons)

Supplemental figure 6M: Elevated soluble transferrin receptor prevalence difference

6M5: Stratified by Source water quality

Source water quality
(p−diff = 0.841)

Country	Trial	N	N	PD (95% CI)	W
**Source water quality − Improved**					
Bangladesh	JiVitA−4 (35)				
Bangladesh	RDNS (36)	550	272	−0.15 (−0.22, −0.08)	0.49
Bangladesh	WASH−B (37)	94	58	−0.32 (−0.46, −0.18)	0.26
Ghana	GHANA (40)	84	82	−0.26 (−0.41, −0.12)	0.25
Ghana	iLiNS−DYAD−G (41)				
Malawi	iLiNS−DYAD−M (44)				
Malawi	iLiNS−DOSE (45)				
**I² = 0.63, Tau² = 0.00**		**728**	**412**	**−0.23 (−0.33, −0.12)**	
**Source water quality − Unimproved**					
Burkina Faso	iLiNS−Zinc (38)	315	96	−0.30 (−0.40, −0.19)	0.39
Burkina Faso	PROMIS CS (39)				
Kenya	WASH−B (42)	166	144	−0.27 (−0.37, −0.16)	0.39
Madagascar	MAHAY (43)	83	51	−0.11 (−0.28, 0.07)	0.21
Mali	PROMIS CS (46)				
Zimbabwe	SHINE (HIV−) (47)				
Zimbabwe	SHINE (HIV+) (48)				
**I² = 0.43, Tau² = 0.00**		**564**	**291**	**−0.24 (−0.35, −0.14)**	

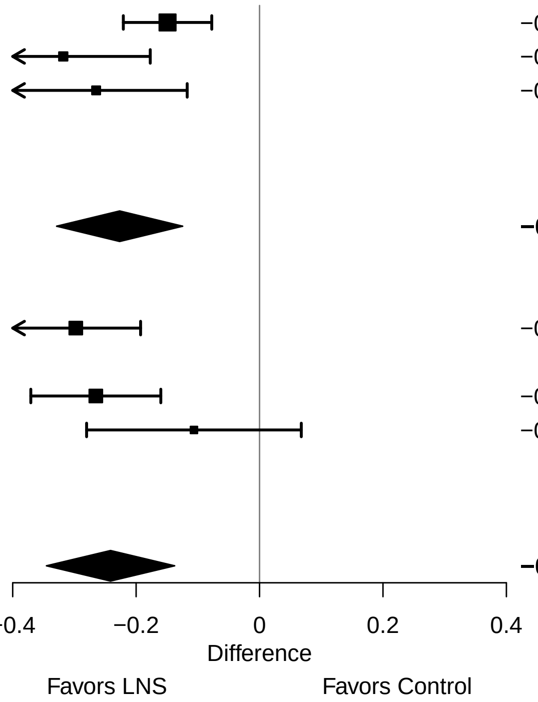

Supplemental figure 6M: Elevated soluble transferrin receptor prevalence difference

6M6: Stratified by Sanitation

Sanitation
(p−diff = 0.841)

Country	Trial	N	N	PD (95% CI)	W
**Sanitation − Improved**					
Bangladesh	JiVitA−4 (35)				
Bangladesh	RDNS (36)	550	272	−0.15 (−0.22, −0.08)	0.49
Bangladesh	WASH−B (37)	94	58	−0.32 (−0.46, −0.18)	0.26
Burkina Faso	PROMIS CS (39)				
Ghana	GHANA (40)	84	82	−0.26 (−0.41, −0.12)	0.25
Ghana	iLiNS−DYAD−G (41)				
Mali	PROMIS CS (46)				
**I² = 0.63, Tau² = 0.00**		**728**	**412**	**−0.23 (−0.33, −0.12)**	
**Sanitation − Unimproved**					
Burkina Faso	iLiNS−Zinc (38)	315	96	−0.30 (−0.40, −0.19)	0.39
Kenya	WASH−B (42)	166	144	−0.27 (−0.37, −0.16)	0.39
Madagascar	MAHAY (43)	83	51	−0.11 (−0.28, 0.07)	0.21
Malawi	iLiNS−DYAD−M (44)				
Malawi	iLiNS−DOSE (45)				
Zimbabwe	SHINE (HIV−) (47)				
Zimbabwe	SHINE (HIV+) (48)				
**I² = 0.43, Tau² = 0.00**		**564**	**291**	**−0.24 (−0.35, −0.14)**	

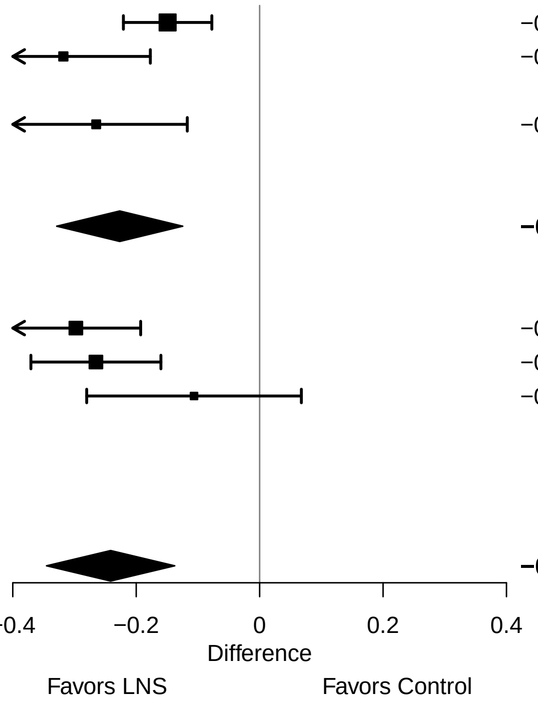

Supplemental figure 6M: Elevated soluble transferrin receptor prevalence difference

6M7: Stratified by Supplement duration

Supplement duration
(p−diff = 0.385)

Country	Trial	N	N	PD (95% CI)	W
**Supplement duration − 12m or less**					
Bangladesh	JiVitA−4 (35)				
Burkina Faso	iLiNS−Zinc (38)	315	96	−0.30 (−0.40, −0.19)	0.48
Burkina Faso	PROMIS CS (39)				
Ghana	GHANA (40)	84	82	−0.26 (−0.41, −0.12)	0.29
Ghana	iLiNS−DYAD−G (41)				
Madagascar	MAHAY (43)	83	51	−0.11 (−0.28, 0.07)	0.22
Malawi	iLiNS−DYAD−M (44)				
Malawi	iLiNS−DOSE (45)				
Zimbabwe	SHINE (HIV−) (47)				
Zimbabwe	SHINE (HIV+) (48)				
**I² = 0.42, Tau² = 0.00**		**482**	**229**	**−0.24 (−0.35, −0.13)**	
**Supplement duration − > 12m**					
Bangladesh	RDNS (36)	550	272	−0.15 (−0.22, −0.08)	0.36
Bangladesh	WASH−B (37)	212	178	−0.22 (−0.29, −0.14)	0.32
Kenya	WASH−B (42)	298	259	−0.24 (−0.32, −0.16)	0.32
Mali	PROMIS CS (46)				
**I² = 0.35, Tau² = 0.00**		**1060**	**709**	**−0.20 (−0.25, −0.15)**	

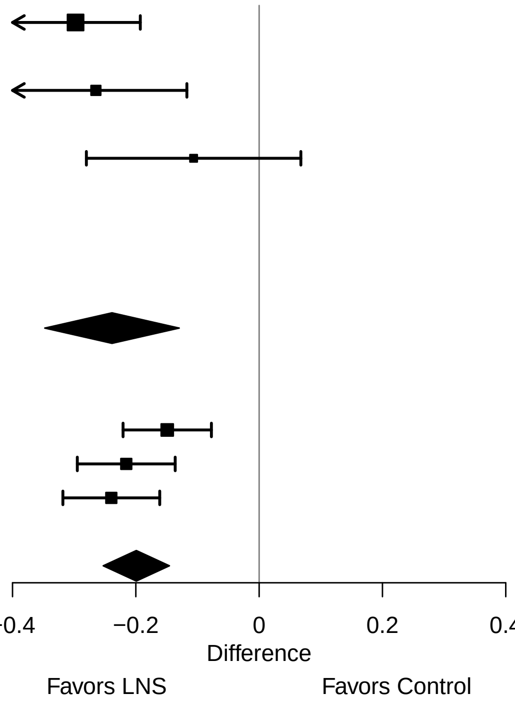

Supplemental figure 6M: Elevated soluble transferrin receptor prevalence difference

6M8: Stratified by Iron dose (insufficient comparisons)

Supplemental figure 6M: Elevated soluble transferrin receptor prevalence difference

6M9: Stratified by Frequency of contact

Frequency of contact
(p−diff = 0.152)

Country	Trial	N	N	PD (95% CI)	W
**Frequency of contact − Monthly**					
Bangladesh	RDNS (36)	550	272	−0.15 (−0.22, −0.08)	0.45
Burkina Faso	PROMIS CS (39)				
Kenya	WASH−B (42)	298	259	−0.24 (−0.32, −0.16)	0.41
Madagascar	MAHAY (43)	83	51	−0.11 (−0.28, 0.07)	0.13
Mali	PROMIS CS (46)				
Zimbabwe	SHINE (HIV−) (47)				
Zimbabwe	SHINE (HIV+) (48)				
**I² = 0.45, Tau² = 0.00**		**931**	**582**	**−0.18 (−0.25, −0.11)**	
**Frequency of contact − Weekly**					
Bangladesh	JiVitA−4 (35)				
Bangladesh	WASH−B (37)	212	178	−0.22 (−0.29, −0.14)	0.47
Burkina Faso	iLiNS−Zinc (38)	315	96	−0.30 (−0.40, −0.19)	0.33
Ghana	GHANA (40)	84	82	−0.26 (−0.41, −0.12)	0.20
Ghana	iLiNS−DYAD−G (41)				
Malawi	iLiNS−DYAD−M (44)				
Malawi	iLiNS−DOSE (45)				
**I² = 0.00, Tau² = 0.00**		**611**	**356**	**−0.25 (−0.31, −0.19)**	

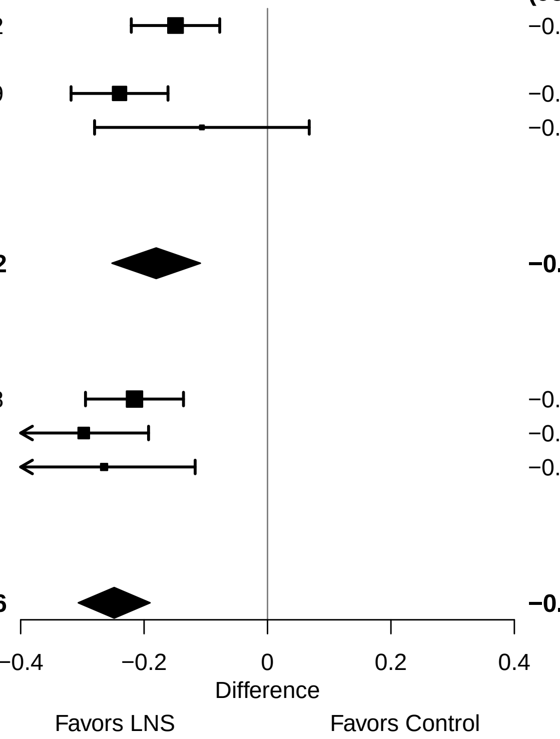

Supplemental figure 6M: Elevated soluble transferrin receptor prevalence difference

6M10: Stratified by Average SQ-LNS compliance (insufficient comparisons)

Supplemental figure 6N: Geometric mean ratio of zinc protoporphyrin concentration

6N1: Stratified by Geographic region (insufficient comparisons)

Supplemental figure 6N: Geometric mean ratio of zinc protoporphyrin concentration

6N2: Stratified by Anemia burden (insufficient comparisons)

Supplemental figure 6N: Geometric mean ratio of zinc protoporphyrin concentration

6N3: Stratified by Malaria prevalence (insufficient comparisons)

Supplemental figure 6N: Geometric mean ratio of zinc protoporphyrin concentration

6N4: Stratified by Inflammation burden (insufficient comparisons)

Supplemental figure 6N: Geometric mean ratio of zinc protoporphyrin concentration

6N5: Stratified by Source water quality (insufficient comparisons)

Supplemental figure 6N: Geometric mean ratio of zinc protoporphyrin concentration

6N6: Stratified by Sanitation (insufficient comparisons)

Supplemental figure 6N: Geometric mean ratio of zinc protoporphyrin concentration

6N7: Stratified by Supplement duration (insufficient comparisons)

Supplemental figure 6N: Geometric mean ratio of zinc protoporphyrin concentration

6N8: Stratified by Iron dose (insufficient comparisons)

Supplemental figure 6N: Geometric mean ratio of zinc protoporphyrin concentration

6N9: Stratified by Frequency of contact (insufficient comparisons)

Supplemental figure 6N: Geometric mean ratio of zinc protoporphyrin concentration

6N10: Stratified by Average SQ-LNS compliance (insufficient comparisons)

Supplemental figure 6O: Elevated zinc protoporphyrin prevalence ratio

6O1: Stratified by Geographic region (insufficient comparisons)

Supplemental figure 6O: Elevated zinc protoporphyrin prevalence ratio

6O2: Stratified by Anemia burden (insufficient comparisons)

Supplemental figure 6O: Elevated zinc protoporphyrin prevalence ratio

6O3: Stratified by Malaria prevalence (insufficient comparisons)

Supplemental figure 6O: Elevated zinc protoporphyrin prevalence ratio

6O4: Stratified by Inflammation burden (insufficient comparisons)

Supplemental figure 6O: Elevated zinc protoporphyrin prevalence ratio

6O5: Stratified by Source water quality (insufficient comparisons)

Supplemental figure 6O: Elevated zinc protoporphyrin prevalence ratio

6O6: Stratified by Sanitation (insufficient comparisons)

Supplemental figure 6O: Elevated zinc protoporphyrin prevalence ratio

6O7: Stratified by Supplement duration (insufficient comparisons)

Supplemental figure 6O: Elevated zinc protoporphyrin prevalence ratio

6O8: Stratified by Iron dose (insufficient comparisons)

Supplemental figure 6O: Elevated zinc protoporphyrin prevalence ratio

6O9: Stratified by Frequency of contact (insufficient comparisons)

Supplemental figure 6O: Elevated zinc protoporphyrin prevalence ratio

6O10: Stratified by Average SQ-LNS compliance (insufficient comparisons)

Supplemental figure 6P: Elevated zinc protoporphyrin prevalence difference

6P1: Stratified by Geographic region (insufficient comparisons)

Supplemental figure 6P: Elevated zinc protoporphyrin prevalence difference

6P2: Stratified by Anemia burden (insufficient comparisons)

Supplemental figure 6P: Elevated zinc protoporphyrin prevalence difference

6P3: Stratified by Malaria prevalence (insufficient comparisons)

Supplemental figure 6P: Elevated zinc protoporphyrin prevalence difference

6P4: Stratified by Inflammation burden (insufficient comparisons)

Supplemental figure 6P: Elevated zinc protoporphyrin prevalence difference

6P5: Stratified by Source water quality (insufficient comparisons)

Supplemental figure 6P: Elevated zinc protoporphyrin prevalence difference

6P6: Stratified by Sanitation (insufficient comparisons)

Supplemental figure 6P: Elevated zinc protoporphyrin prevalence difference

6P7: Stratified by Supplement duration (insufficient comparisons)

Supplemental figure 6P: Elevated zinc protoporphyrin prevalence difference

6P8: Stratified by Iron dose (insufficient comparisons)

Supplemental figure 6P: Elevated zinc protoporphyrin prevalence difference

6P9: Stratified by Frequency of contact (insufficient comparisons)

Supplemental figure 6P: Elevated zinc protoporphyrin prevalence difference

6P10: Stratified by Average SQ-LNS compliance (insufficient comparisons)

Supplemental figure 6Q: Geometric mean ratio of plasma zinc concentration

6Q1: Stratified by Geographic region (insufficient comparisons)

Supplemental figure 6Q: Geometric mean ratio of plasma zinc concentration

6Q2: Stratified by Anemia burden (insufficient comparisons)

Supplemental figure 6Q: Geometric mean ratio of plasma zinc concentration

6Q3: Stratified by Malaria prevalence (insufficient comparisons)

Supplemental figure 6Q: Geometric mean ratio of plasma zinc concentration

6Q4: Stratified by Inflammation burden (insufficient comparisons)

Supplemental figure 6Q: Geometric mean ratio of plasma zinc concentration

6Q5: Stratified by Source water quality (insufficient comparisons)

Supplemental figure 6Q: Geometric mean ratio of plasma zinc concentration

6Q6: Stratified by Sanitation (insufficient comparisons)

Supplemental figure 6Q: Geometric mean ratio of plasma zinc concentration

6Q7: Stratified by Supplement duration (insufficient comparisons)

Supplemental figure 6Q: Geometric mean ratio of plasma zinc concentration

6Q8: Stratified by Iron dose (insufficient comparisons)

Supplemental figure 6Q: Geometric mean ratio of plasma zinc concentration

6Q9: Stratified by Frequency of contact (insufficient comparisons)

Supplemental figure 6Q: Geometric mean ratio of plasma zinc concentration

6Q10: Stratified by Average SQ-LNS compliance (insufficient comparisons)

Supplemental figure 6R: Geometric mean ratio of retinol concentration

6R1: Stratified by Geographic region (insufficient comparisons)

Supplemental figure 6R: Geometric mean ratio of retinol concentration

6R2: Stratified by Anemia burden (insufficient comparisons)

Supplemental figure 6R: Geometric mean ratio of retinol concentration

6R3: Stratified by Malaria prevalence (insufficient comparisons)

Supplemental figure 6R: Geometric mean ratio of retinol concentration

6R4: Stratified by Inflammation burden (insufficient comparisons)

Supplemental figure 6R: Geometric mean ratio of retinol concentration

6R5: Stratified by Source water quality (insufficient comparisons)

Supplemental figure 6R: Geometric mean ratio of retinol concentration

6R6: Stratified by Sanitation (insufficient comparisons)

Supplemental figure 6R: Geometric mean ratio of retinol concentration

6R7: Stratified by Supplement duration (insufficient comparisons)

Supplemental figure 6R: Geometric mean ratio of retinol concentration

6R8: Stratified by Iron dose (insufficient comparisons)

Supplemental figure 6R: Geometric mean ratio of retinol concentration

6R9: Stratified by Frequency of contact (insufficient comparisons)

Supplemental figure 6R: Geometric mean ratio of retinol concentration

6R10: Stratified by Average SQ-LNS compliance (insufficient comparisons)

Supplemental figure 6S: Low vitamin A (retinol < 0.70 µmol/L) prevalence ratio

6S1: Stratified by Geographic region (insufficient comparisons)

Supplemental figure 6S: Low vitamin A (retinol < 0.70 µmol/L) prevalence ratio

6S2: Stratified by Anemia burden (insufficient comparisons)

Supplemental figure 6S: Low vitamin A (retinol < 0.70 µmol/L) prevalence ratio

6S3: Stratified by Malaria prevalence (insufficient comparisons)

Supplemental figure 6S: Low vitamin A (retinol < 0.70 µmol/L) prevalence ratio

6S4: Stratified by Inflammation burden (insufficient comparisons)

Supplemental figure 6S: Low vitamin A (retinol < 0.70 μmol/L) prevalence ratio

6S5: Stratified by Source water quality (insufficient comparisons)

Supplemental figure 6S: Low vitamin A (retinol < 0.70 μmol/L) prevalence ratio

6S6: Stratified by Sanitation (insufficient comparisons)

Supplemental figure 6S: Low vitamin A (retinol < 0.70 µmol/L) prevalence ratio

6S7: Stratified by Supplement duration (insufficient comparisons)

Supplemental figure 6S: Low vitamin A (retinol < 0.70 µmol/L) prevalence ratio

6S8: Stratified by Iron dose (insufficient comparisons)

Supplemental figure 6S: Low vitamin A (retinol < 0.70 µmol/L) prevalence ratio

6S9: Stratified by Frequency of contact (insufficient comparisons)

Supplemental figure 6S: Low vitamin A (retinol < 0.70 µmol/L) prevalence ratio

6S10: Stratified by Average SQ-LNS compliance (insufficient comparisons)

Supplemental figure 6T: Low vitamin A (retinol < 0.70 µmol/L) prevalence difference

6T1: Stratified by Geographic region (insufficient comparisons)

Supplemental figure 6T: Low vitamin A (retinol < 0.70 µmol/L) prevalence difference

6T2: Stratified by Anemia burden (insufficient comparisons)

Supplemental figure 6T: Low vitamin A (retinol < 0.70 μmol/L) prevalence difference

6T3: Stratified by Malaria prevalence (insufficient comparisons)

Supplemental figure 6T: Low vitamin A (retinol < 0.70 µmol/L) prevalence difference

6T4: Stratified by Inflammation burden (insufficient comparisons)

Supplemental figure 6T: Low vitamin A (retinol < 0.70 µmol/L) prevalence difference

6T5: Stratified by Source water quality (insufficient comparisons)

Supplemental figure 6T: Low vitamin A (retinol $<$ 0.70 µmol/L) prevalence difference

6T6: Stratified by Sanitation (insufficient comparisons)

Supplemental figure 6T: Low vitamin A (retinol < 0.70 µmol/L) prevalence difference

6T7: Stratified by Supplement duration (insufficient comparisons)

Supplemental figure 6T: Low vitamin A (retinol < 0.70 µmol/L) prevalence difference

6T8: Stratified by Iron dose (insufficient comparisons)

Supplemental figure 6T: Low vitamin A (retinol < 0.70 µmol/L) prevalence difference

6T9: Stratified by Frequency of contact (insufficient comparisons)

Supplemental figure 6T: Low vitamin A (retinol < 0.70 µmol/L) prevalence difference

6T10: Stratified by Average SQ-LNS compliance (insufficient comparisons)

Supplemental figure 6U: Marginal vitamin A (retinol < 1.05 µmol/L) prevalence ratio

6U1: Stratified by Geographic region (insufficient comparisons)

Supplemental figure 6U: Marginal vitamin A (retinol < 1.05 µmol/L) prevalence ratio

6U2: Stratified by Anemia burden (insufficient comparisons)

Supplemental figure 6U: Marginal vitamin A (retinol < 1.05 µmol/L) prevalence ratio

6U3: Stratified by Malaria prevalence (insufficient comparisons)

Supplemental figure 6U: Marginal vitamin A (retinol < 1.05 µmol/L) prevalence ratio

6U4: Stratified by Inflammation burden (insufficient comparisons)

Supplemental figure 6U: Marginal vitamin A (retinol < 1.05 µmol/L) prevalence ratio

6U5: Stratified by Source water quality (insufficient comparisons)

Supplemental figure 6U: Marginal vitamin A (retinol < 1.05 µmol/L) prevalence ratio

6U6: Stratified by Sanitation (insufficient comparisons)

Supplemental figure 6U: Marginal vitamin A (retinol < 1.05 µmol/L) prevalence ratio

6U7: Stratified by Supplement duration (insufficient comparisons)

Supplemental figure 6U: Marginal vitamin A (retinol < 1.05 µmol/L) prevalence ratio

6U8: Stratified by Iron dose (insufficient comparisons)

Supplemental figure 6U: Marginal vitamin A (retinol < 1.05 µmol/L) prevalence ratio

6U9: Stratified by Frequency of contact (insufficient comparisons)

Supplemental figure 6U: Marginal vitamin A (retinol < 1.05 µmol/L) prevalence ratio

6U10: Stratified by Average SQ-LNS compliance (insufficient comparisons)

Supplemental figure 6V: Marginal vitamin A (retinol < 1.05 µmol/L) prevalence difference

6V1: Stratified by Geographic region (insufficient comparisons)

Supplemental figure 6V: Marginal vitamin A (retinol < 1.05 µmol/L) prevalence difference

6V2: Stratified by Anemia burden (insufficient comparisons)

Supplemental figure 6V: Marginal vitamin A (retinol < 1.05 µmol/L) prevalence difference

6V3: Stratified by Malaria prevalence (insufficient comparisons)

Supplemental figure 6V: Marginal vitamin A (retinol < 1.05 µmol/L) prevalence difference

6V4: Stratified by Inflammation burden (insufficient comparisons)

Supplemental figure 6V: Marginal vitamin A (retinol < 1.05 μmol/L) prevalence difference

6V5: Stratified by Source water quality (insufficient comparisons)

Supplemental figure 6V: Marginal vitamin A (retinol < 1.05 µmol/L) prevalence difference

6V6: Stratified by Sanitation (insufficient comparisons)

Supplemental figure 6V: Marginal vitamin A (retinol < 1.05 µmol/L) prevalence difference

6V7: Stratified by Supplement duration (insufficient comparisons)

Supplemental figure 6V: Marginal vitamin A (retinol < 1.05 µmol/L) prevalence difference

6V8: Stratified by Iron dose (insufficient comparisons)

Supplemental figure 6V: Marginal vitamin A (retinol < 1.05 µmol/L) prevalence difference

6V9: Stratified by Frequency of contact (insufficient comparisons)

Supplemental figure 6V: Marginal vitamin A (retinol $<$ 1.05 µmol/L) prevalence difference

6V10: Stratified by Average SQ-LNS compliance (insufficient comparisons)

Supplemental figure 6W: Geometric mean ratio of retinol binding protein concentration

6W1: Stratified by Geographic region (insufficient comparisons)

Supplemental figure 6W: Geometric mean ratio of retinol binding protein concentration

6W2: Stratified by Anemia burden (insufficient comparisons)

Supplemental figure 6W: Geometric mean ratio of retinol binding protein concentration

6W3: Stratified by Malaria prevalence (insufficient comparisons)

Supplemental figure 6W: Geometric mean ratio of retinol binding protein concentration

6W4: Stratified by Inflammation burden (insufficient comparisons)

Supplemental figure 6W: Geometric mean ratio of retinol binding protein concentration

6W5: Stratified by Source water quality (insufficient comparisons)

Supplemental figure 6W: Geometric mean ratio of retinol binding protein concentration

6W6: Stratified by Sanitation (insufficient comparisons)

Supplemental figure 6W: Geometric mean ratio of retinol binding protein concentration

6W7: Stratified by Supplement duration (insufficient comparisons)

Supplemental figure 6W: Geometric mean ratio of retinol binding protein concentration

6W8: Stratified by Iron dose (insufficient comparisons)

Supplemental figure 6W: Geometric mean ratio of retinol binding protein concentration

6W9: Stratified by Frequency of contact (insufficient comparisons)

Supplemental figure 6W: Geometric mean ratio of retinol binding protein concentration

6W10: Stratified by Average SQ-LNS compliance (insufficient comparisons)

Supplemental figure 6X: Low vitamin A status (RBP < 0.70 µmol/L) prevalence ratio

6X1: Stratified by Geographic region (insufficient comparisons)

Supplemental figure 6X: Low vitamin A status (RBP < 0.70 μmol/L) prevalence ratio

6X2: Stratified by Anemia burden (insufficient comparisons)

Supplemental figure 6X: Low vitamin A status (RBP < 0.70 µmol/L) prevalence ratio

6X3: Stratified by Malaria prevalence (insufficient comparisons)

Supplemental figure 6X: Low vitamin A status (RBP < 0.70 µmol/L) prevalence ratio

6X4: Stratified by Inflammation burden (insufficient comparisons)

Supplemental figure 6X: Low vitamin A status (RBP < 0.70 µmol/L) prevalence ratio

6X5: Stratified by Source water quality (insufficient comparisons)

Supplemental figure 6X: Low vitamin A status (RBP < 0.70 µmol/L) prevalence ratio

6X6: Stratified by Sanitation (insufficient comparisons)

Supplemental figure 6X: Low vitamin A status (RBP < 0.70 µmol/L) prevalence ratio

6X7: Stratified by Supplement duration (insufficient comparisons)

Supplemental figure 6X: Low vitamin A status (RBP < 0.70 µmol/L) prevalence ratio

6X8: Stratified by Iron dose (insufficient comparisons)

Supplemental figure 6X: Low vitamin A status (RBP < 0.70 µmol/L) prevalence ratio

6X9: Stratified by Frequency of contact (insufficient comparisons)

Supplemental figure 6X: Low vitamin A status (RBP < 0.70 µmol/L) prevalence ratio

6X10: Stratified by Average SQ-LNS compliance (insufficient comparisons)

Supplemental figure 6Y: Low vitamin A status (RBP < 0.70 µmol/L) prevalence difference

6Y1: Stratified by Geographic region (insufficient comparisons)

Supplemental figure 6Y: Low vitamin A status (RBP < 0.70 µmol/L) prevalence difference

6Y2: Stratified by Anemia burden (insufficient comparisons)

Supplemental figure 6Y: Low vitamin A status (RBP < 0.70 µmol/L) prevalence difference

6Y3: Stratified by Malaria prevalence (insufficient comparisons)

Supplemental figure 6Y: Low vitamin A status (RBP < 0.70 µmol/L) prevalence difference

6Y4: Stratified by Inflammation burden (insufficient comparisons)

Supplemental figure 6Y: Low vitamin A status (RBP < 0.70 µmol/L) prevalence difference

6Y5: Stratified by Source water quality (insufficient comparisons)

Supplemental figure 6Y: Low vitamin A status (RBP < 0.70 µmol/L) prevalence difference

6Y6: Stratified by Sanitation (insufficient comparisons)

Supplemental figure 6Y: Low vitamin A status (RBP < 0.70 µmol/L) prevalence difference

6Y7: Stratified by Supplement duration (insufficient comparisons)

Supplemental figure 6Y: Low vitamin A status (RBP < 0.70 µmol/L) prevalence difference

6Y8: Stratified by Iron dose (insufficient comparisons)

Supplemental figure 6Y: Low vitamin A status (RBP $<$ 0.70 µmol/L) prevalence difference

6Y9: Stratified by Frequency of contact (insufficient comparisons)

Supplemental figure 6Y: Low vitamin A status (RBP < 0.70 µmol/L) prevalence difference

6Y10: Stratified by Average SQ-LNS compliance (insufficient comparisons)

Supplemental figure 6Z: Marginal vitamin A status (RBP < 1.05 µmol/L) prevalence ratio

6Z1: Stratified by Geographic region (insufficient comparisons)

Supplemental figure 6Z: Marginal vitamin A status (RBP < 1.05 µmol/L) prevalence ratio

6Z2: Stratified by Anemia burden (insufficient comparisons)

Supplemental figure 6Z: Marginal vitamin A status (RBP < 1.05 µmol/L) prevalence ratio

6Z3: Stratified by Malaria prevalence (insufficient comparisons)

Supplemental figure 6Z: Marginal vitamin A status (RBP < 1.05 µmol/L) prevalence ratio

6Z4: Stratified by Inflammation burden (insufficient comparisons)

Supplemental figure 6Z: Marginal vitamin A status (RBP < 1.05 µmol/L) prevalence ratio

6Z5: Stratified by Source water quality (insufficient comparisons)

Supplemental figure 6Z: Marginal vitamin A status (RBP < 1.05 µmol/L) prevalence ratio

6Z6: Stratified by Sanitation (insufficient comparisons)

Supplemental figure 6Z: Marginal vitamin A status (RBP < 1.05 µmol/L) prevalence ratio

6Z7: Stratified by Supplement duration (insufficient comparisons)

Supplemental figure 6Z: Marginal vitamin A status (RBP < 1.05 µmol/L) prevalence ratio

6Z8: Stratified by Iron dose (insufficient comparisons)

Supplemental figure 6Z: Marginal vitamin A status (RBP < 1.05 µmol/L) prevalence ratio

6Z9: Stratified by Frequency of contact (insufficient comparisons)

Supplemental figure 6Z: Marginal vitamin A status (RBP < 1.05 µmol/L) prevalence ratio

6Z10: Stratified by Average SQ-LNS compliance (insufficient comparisons)

Supplemental figure 6AA: Marginal vitamin A status (RBP < 1.05 µmol/L) prevalence difference

6AA1: Stratified by Geographic region (insufficient comparisons)

Supplemental figure 6AA: Marginal vitamin A status (RBP < 1.05 µmol/L) prevalence difference

6AA2: Stratified by Anemia burden (insufficient comparisons)

Supplemental figure 6AA: Marginal vitamin A status (RBP < 1.05 µmol/L) prevalence difference

6AA3: Stratified by Malaria prevalence (insufficient comparisons)

Supplemental figure 6AA: Marginal vitamin A status (RBP < 1.05 µmol/L) prevalence difference

6AA4: Stratified by Inflammation burden (insufficient comparisons)

Supplemental figure 6AA: Marginal vitamin A status (RBP < 1.05 µmol/L) prevalence difference

6AA5: Stratified by Source water quality (insufficient comparisons)

Supplemental figure 6AA: Marginal vitamin A status (RBP < 1.05 µmol/L) prevalence difference

6AA6: Stratified by Sanitation (insufficient comparisons)

Supplemental figure 6AA: Marginal vitamin A status (RBP < 1.05 µmol/L) prevalence difference

6AA7: Stratified by Supplement duration (insufficient comparisons)

Supplemental figure 6AA: Marginal vitamin A status (RBP < 1.05 µmol/L) prevalence difference

6AA8: Stratified by Iron dose (insufficient comparisons)

Supplemental figure 6AA: Marginal vitamin A status (RBP < 1.05 µmol/L) prevalence difference

6AA9: Stratified by Frequency of contact (insufficient comparisons)

Supplemental figure 6AA: Marginal vitamin A status (RBP < 1.05 µmol/L) prevalence difference

6AA10: Stratified by Average SQ-LNS compliance (insufficient comparisons)
