# Supplementary material for: Characteristics that modify the effect of small-quantity lipid-based nutrient supplementation on child anemia and micronutrient status: an individual participant data meta-analysis of randomized controlled trials
Source: Am J Clin Nutr. 2021 Sep 29;114(Suppl 1):68S–94S. doi: 10.1093/ajcn/nqab276 (PMC8560313; doi:10.1093/ajcn/nqab276)
Supplement: nqab276_Supplemental_Files [file nqab276_supplemental_files.zip › 11_ipdb_suppfig7_20210401.pdf]

Supplemental figure 7: Sensitivity analyses of effect modification of SQ-LNS on biochemical outcomes by study-level effect modifiers

Contents

Supplemental figure 7A: Difference in mean differences in hemoglobin concentration **4**
7A1: By study context effect modifiers 4
7A2: By study design effect modifiers 5

Supplemental figure 7B: Ratio of anemia prevalence ratios **6**
7B1: By study context effect modifiers 6
7B2: By study design effect modifiers 7

Supplemental figure 7C: Difference in anemia prevalence differences **8**
7C1: By study context effect modifiers 8
7C2: By study design effect modifiers 9

Supplemental figure 7D: Ratio of moderate-to-severe anemia prevalence ratios **10**
7D1: By study context effect modifiers 10
7D2: By study design effect modifiers 11

Supplemental figure 7E: Difference in moderate-to-severe anemia prevalence differences **12**
7E1: By study context effect modifiers 12
7E2: By study design effect modifiers 13

Supplemental figure 7F: Ratio of geometric mean ratios of ferritin concentration **14**
7F1: By study context effect modifiers 14
7F2: By study design effect modifiers 15

Supplemental figure 7G: Ratio of iron deficiency (ferritin < 12 µg/L) prevalence ratios **16**
7G1: By study context effect modifiers 16
7G2: By study design effect modifiers 17

Supplemental figure 7H: Difference in iron deficiency (ferritin < 12 µg/L) prevalence differences **18**
7H1: By study context effect modifiers 18
7H2: By study design effect modifiers 19

Supplemental figure 7I: Ratio of iron deficiency anemia prevalence ratios **20**
7I1: By study context effect modifiers . . . 20
7I2: By study design effect modifiers . . . 21

Supplemental figure 7J: Difference in iron deficiency anemia prevalence differences **22**
7J1: By study context effect modifiers . . . 22
7J2: By study design effect modifiers . . . 23

Supplemental figure 7K: Ratio of geometric mean ratios of soluble transferrin receptor concentration **24**
7K1: By study context effect modifiers . . . 24
7K2: By study design effect modifiers . . . 25

Supplemental figure 7L: Ratio of elevated soluble transferrin receptor prevalence ratios **26**
7L1: By study context effect modifiers . . . 26
7L2: By study design effect modifiers . . . 27

Supplemental figure 7M: Difference in elevated soluble transferrin receptor prevalence differences **28**
7M1: By study context effect modifiers . . . 28
7M2: By study design effect modifiers . . . 29

Supplemental figure 7N: Ratio of geometric mean ratios of zinc protoporphyrin concentration **30**
7N1: By study context effect modifiers (insufficient comparisons) . . . 30
7N2: By study design effect modifiers (insufficient comparisons) . . . 31

Supplemental figure 7O: Ratio of elevated zinc protoporphyrin prevalence ratios **32**
7O1: By study context effect modifiers (insufficient comparisons) . . . 32
7O2: By study design effect modifiers (insufficient comparisons) . . . 33

Supplemental figure 7P: Difference in elevated zinc protoporphyrin prevalence differences **34**
7P1: By study context effect modifiers (insufficient comparisons) . . . 34
7P2: By study design effect modifiers (insufficient comparisons) . . . 35

Supplemental figure 7Q: Ratio of geometric mean ratios of plasma zinc concentration **36**
7Q1: By study context effect modifiers (insufficient comparisons) . . . 36
7Q2: By study design effect modifiers (insufficient comparisons) . . . 37

Supplemental figure 7R: Ratio of geometric mean ratios of retinol concentration **38**
7R1: By study context effect modifiers (insufficient comparisons) . . . 38
7R2: By study design effect modifiers (insufficient comparisons) . . . 39

Supplemental figure 7S: Ratio of low vitamin A (retinol $<$ 0.70 µmol/L) prevalence ratios **40**
7S1: By study context effect modifiers (insufficient comparisons) . . . 40
7S2: By study design effect modifiers (insufficient comparisons) . . . 41

Supplemental figure 7T: Difference in low vitamin A (retinol < 0.70 µmol/L) prevalence differences **42**
7T1: By study context effect modifiers (insufficient comparisons) 42
7T2: By study design effect modifiers (insufficient comparisons) 43

Supplemental figure 7U: Ratio of marginal vitamin A (retinol < 1.05 µmol/L) prevalence ratios **44**
7U1: By study context effect modifiers (insufficient comparisons) 44
7U2: By study design effect modifiers (insufficient comparisons) 45

Supplemental figure 7V: Difference in marginal vitamin A (retinol < 1.05 µmol/L) prevalence differences **46**
7V1: By study context effect modifiers (insufficient comparisons) 46
7V2: By study design effect modifiers (insufficient comparisons) 47

Supplemental figure 7W: Ratio of geometric mean ratio of retinol binding protein concentrations **48**
7W1: By study context effect modifiers (insufficient comparisons) 48
7W2: By study design effect modifiers (insufficient comparisons) 49

Supplemental figure 7X: Ratio of Low vitamin A status (RBP < 0.70 µmol/L) prevalence ratios **50**
7X1: By study context effect modifiers (insufficient comparisons) 50
7X2: By study design effect modifiers (insufficient comparisons) 51

Supplemental figure 7Y: Difference in low vitamin A status (RBP < 0.70 µmol/L) prevalence differences **52**
7Y1: By study context effect modifiers (insufficient comparisons) 52
7Y2: By study design effect modifiers (insufficient comparisons) 53

Supplemental figure 7Z: Ratio of marginal vitamin A status (RBP < 1.05 µmol/L) prevalence ratios **54**
7Z1: By study context effect modifiers (insufficient comparisons) 54
7Z2: By study design effect modifiers (insufficient comparisons) 55

Supplemental figure 7AA: Difference in marginal vitamin A status (RBP < 1.05 µmol/L) prevalence differences **56**
7AA1: By study context effect modifiers (insufficient comparisons) 56
7AA2: By study design effect modifiers (insufficient comparisons) 57

These figures show the pooled estimates of effect modification by different sensitivity analyses. For continuous outcomes, the intervention effect is measured by the difference in mean of the LNS group minus control. For log transformed continuous outcomes, the intervention effect is measured by the ratio of geometric means, the effect estimate is the geometric mean in the LNS group divided by the geometric mean in the control group. For dichotomous outcomes analyzed via prevalence ratios, the effect estimate is the prevalence in the LNS group divided by the prevalence in the control group. For dichotomous outcomes analyzed via prevalence differences, the effect estimate is the prevalence in the LNS group minus the prevalence in the control group. If fewer than three studies contribute to a pooled estimate then the pooled estimate was not generated (e.g. if fewer than 3 studies are categorized into a study level effect modification category), and this is labeled as "insufficient comparisons".

The labels on the left y-axis indicate which outcome is assessed. The different columns correspond to sensitivity analyses in which intervention group categorization differs. All-trial analysis includes all trials; Child-LNS-only excludes trial arms that provided both maternal and child LNS; Multi-component analysis separates comparisons within trials that included multi-component interventions, so that the SQ-LNS vs. no SQ-LNS comparisons were conducted separately between pairs of arms that included the same non-nutrition components (e.g. SQ-LNS+WASH vs. WASH; SQ-LNS vs. Control); Passive arms excluded analysis excludes passive control arms. Depending on the sensitivity analysis, there may not have been enough comparisons available to generate a pooled estimate.

Ferritin, sTfR, ZPP, zinc, retinol and RBP concentrations were adjusted for inflammation (i.e., C-reactive protein (CRP) and/or α-1-acid glycoprotein (AGP) concentrations, as available), using a regression correction approach adapted from the Biomarkers Reflecting Inflammation and Nutritional Determinants of Anemia (BRINDA) project (28)

sTfR, soluble transferrin receptor; ZPP, zinc protoporphyrin; RBP, retinol binding protein.

Supplemental figure 7A: Difference in mean differences in hemoglobin concentration

7A1: By study context effect modifiers

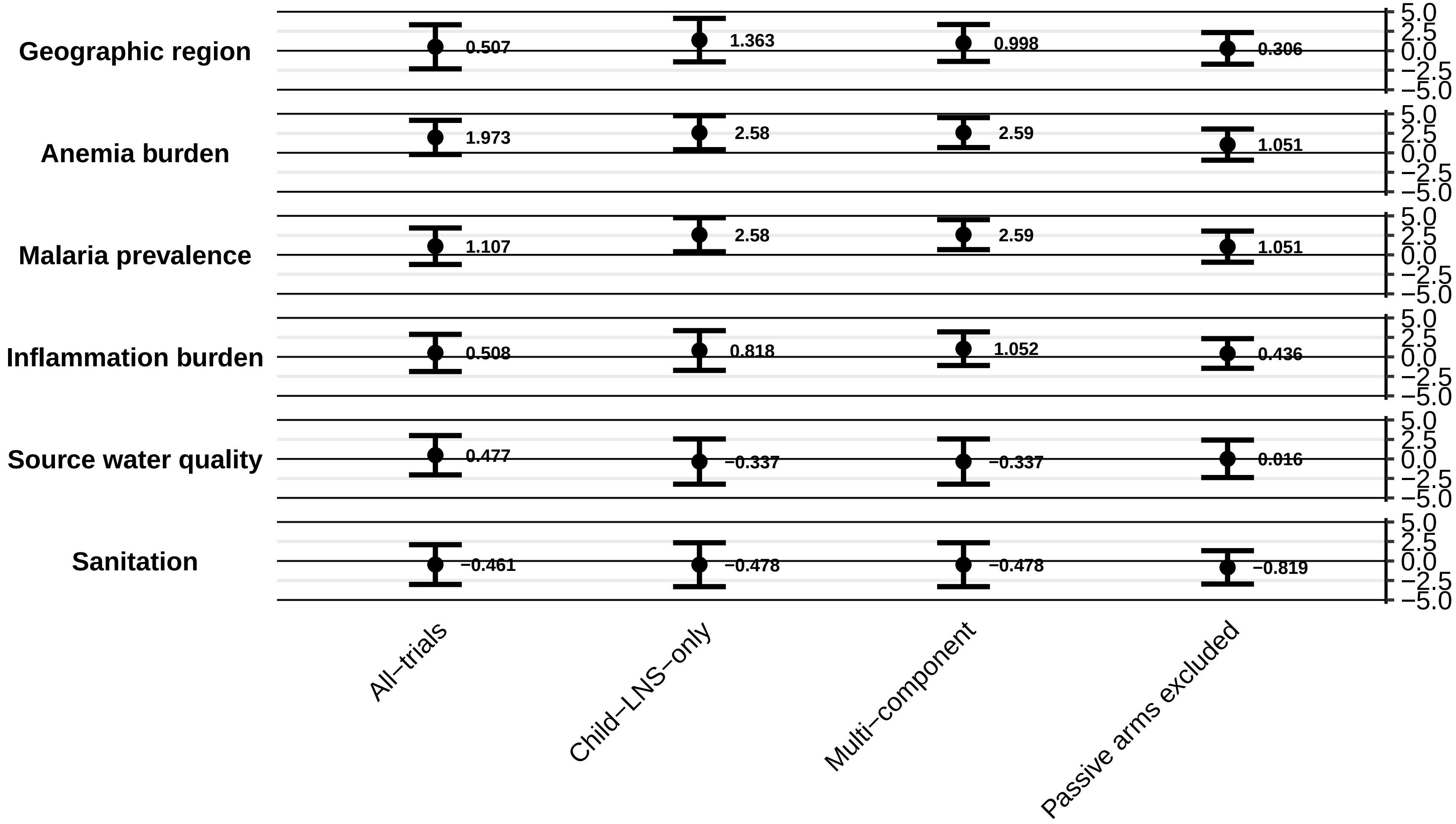

Supplemental figure 7A: Difference in mean differences in hemoglobin concentration

7A2: By study design effect modifiers

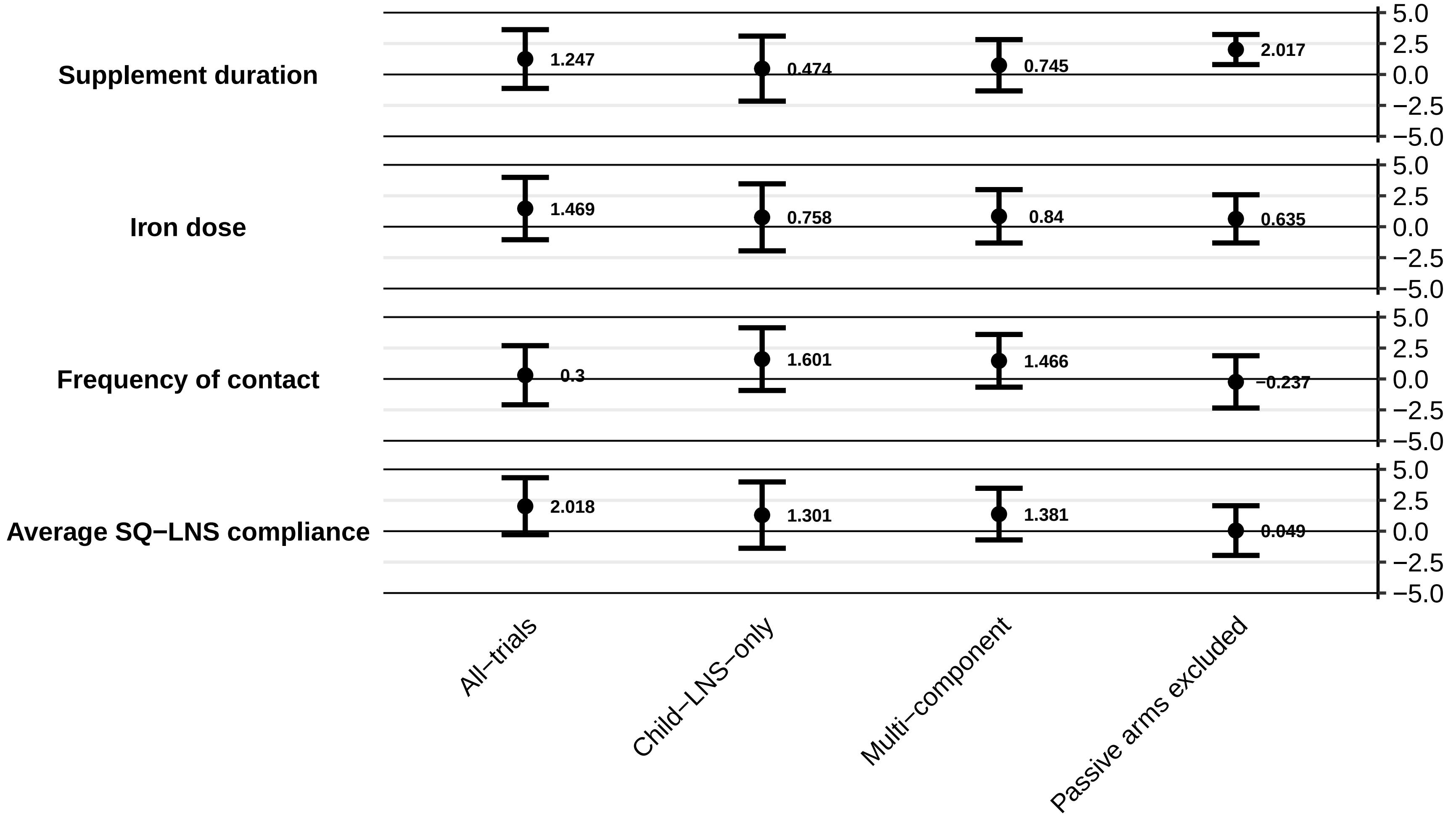

Supplemental figure 7B: Ratio of anemia prevalence ratios

7B1: By study context effect modifiers

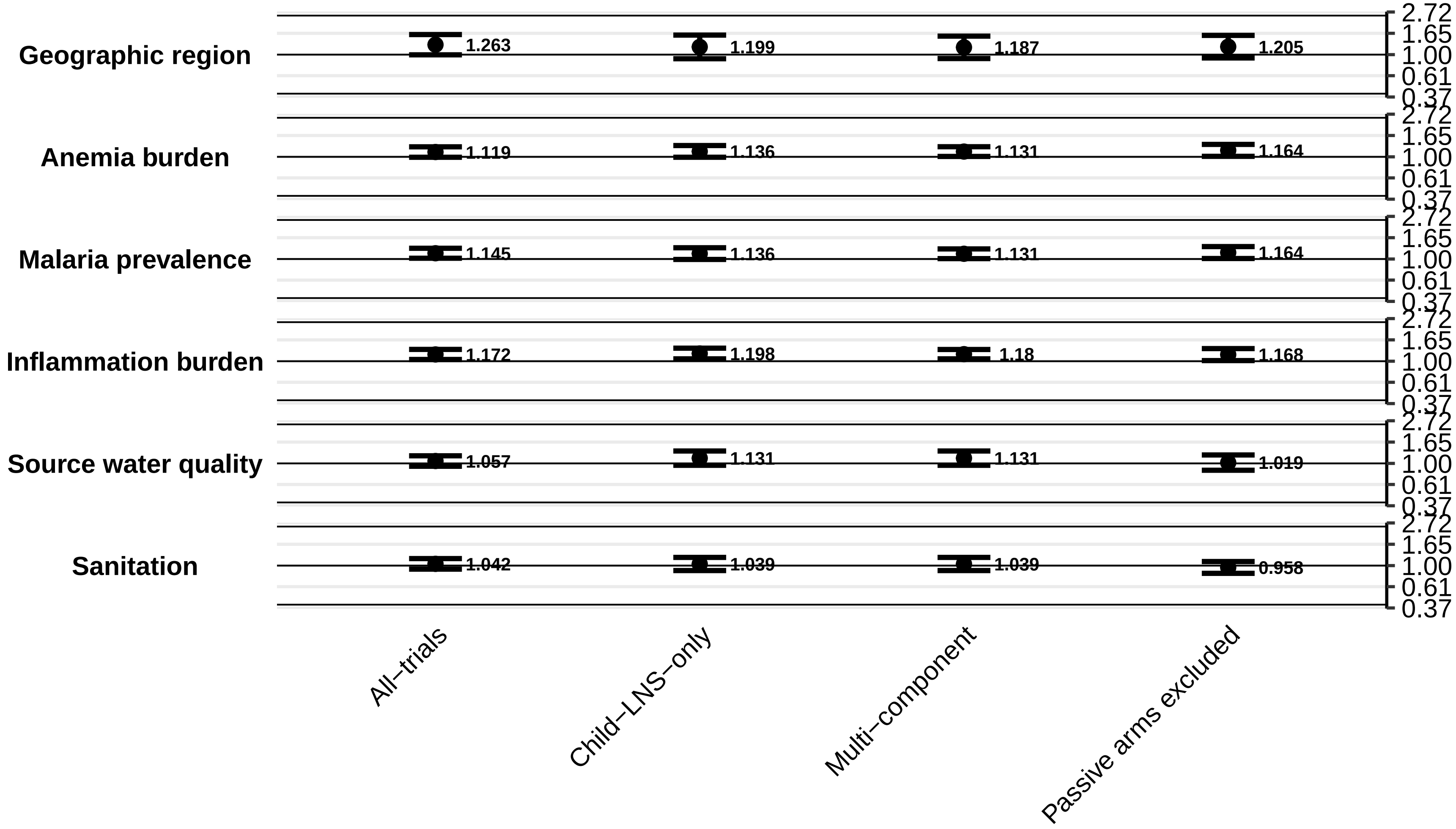

Supplemental figure 7B: Ratio of anemia prevalence ratios

7B2: By study design effect modifiers

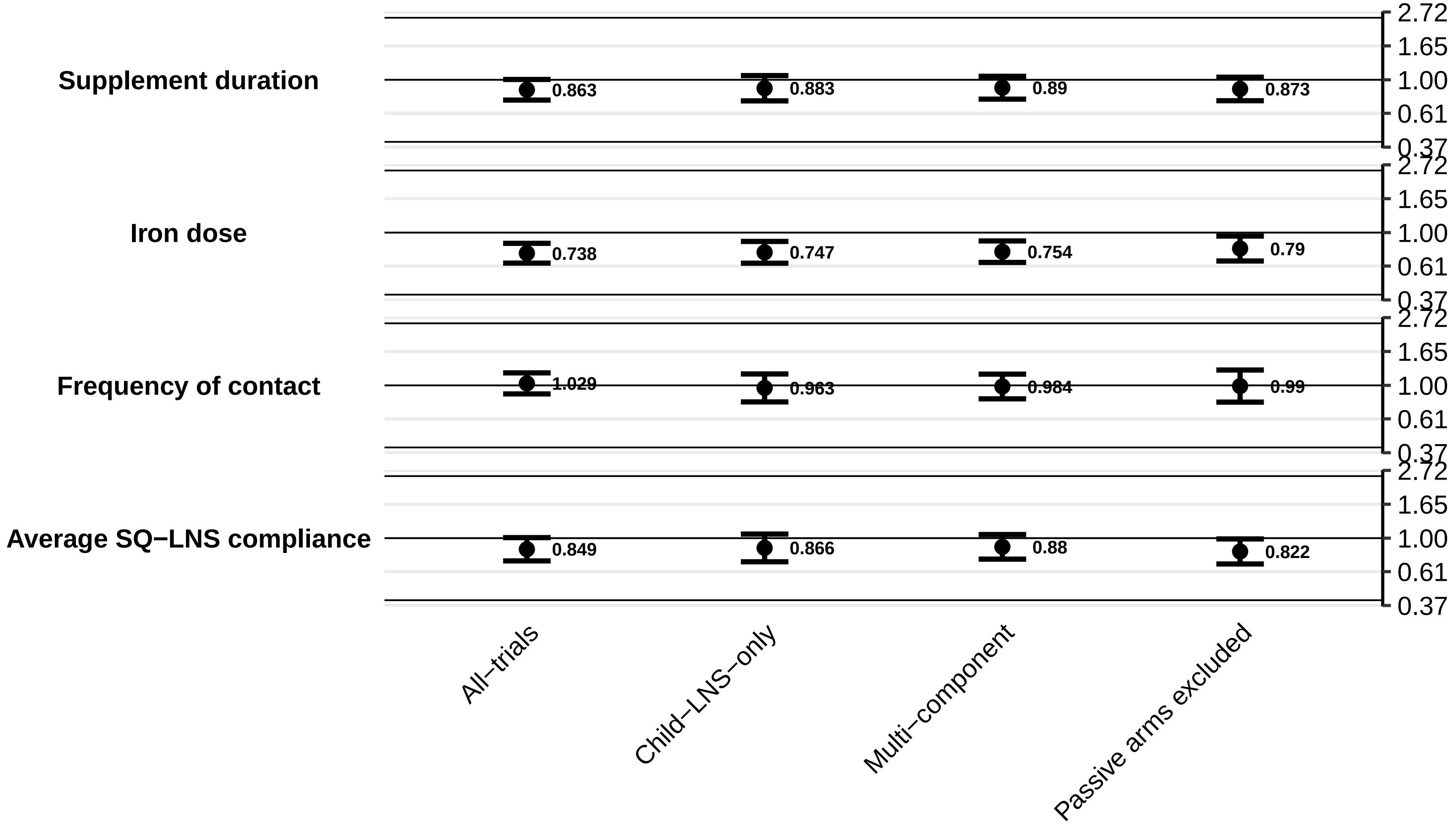

Supplemental figure 7C: Difference in anemia prevalence differences

7C1: By study context effect modifiers

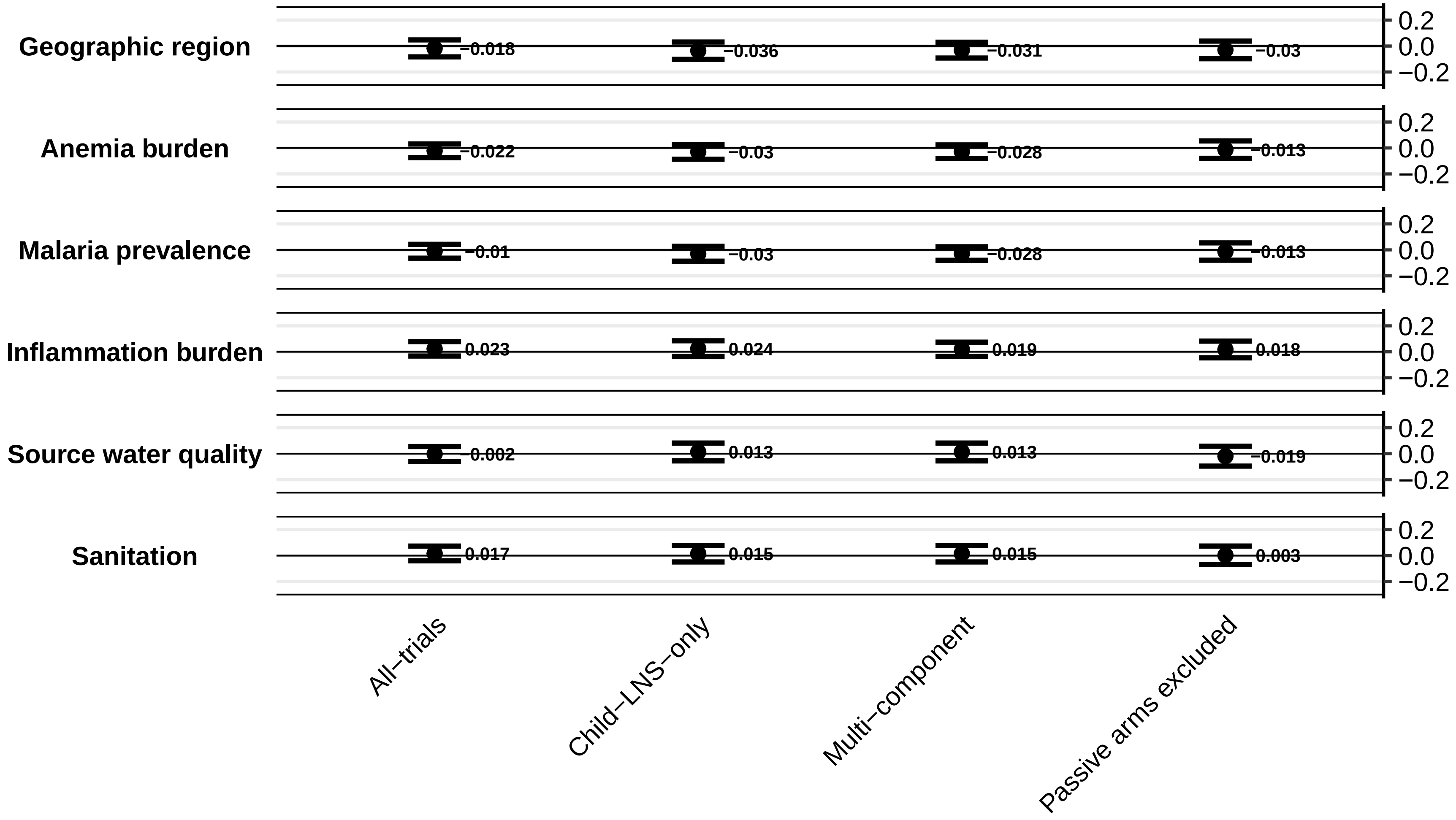

Supplemental figure 7C: Difference in anemia prevalence differences

7C2: By study design effect modifiers

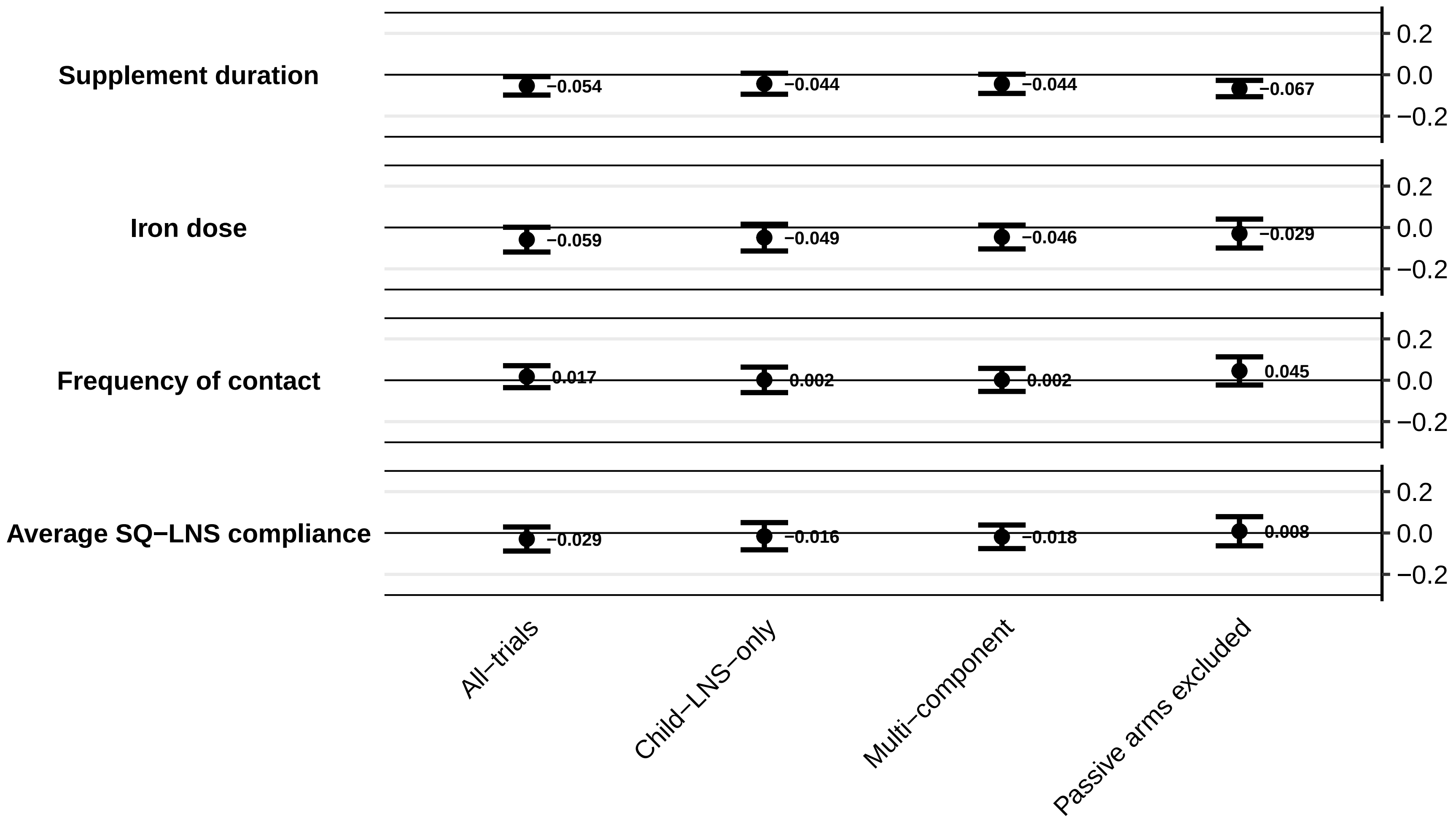

Supplemental figure 7D: Ratio of moderate-to-severe anemia prevalence ratios

7D1: By study context effect modifiers

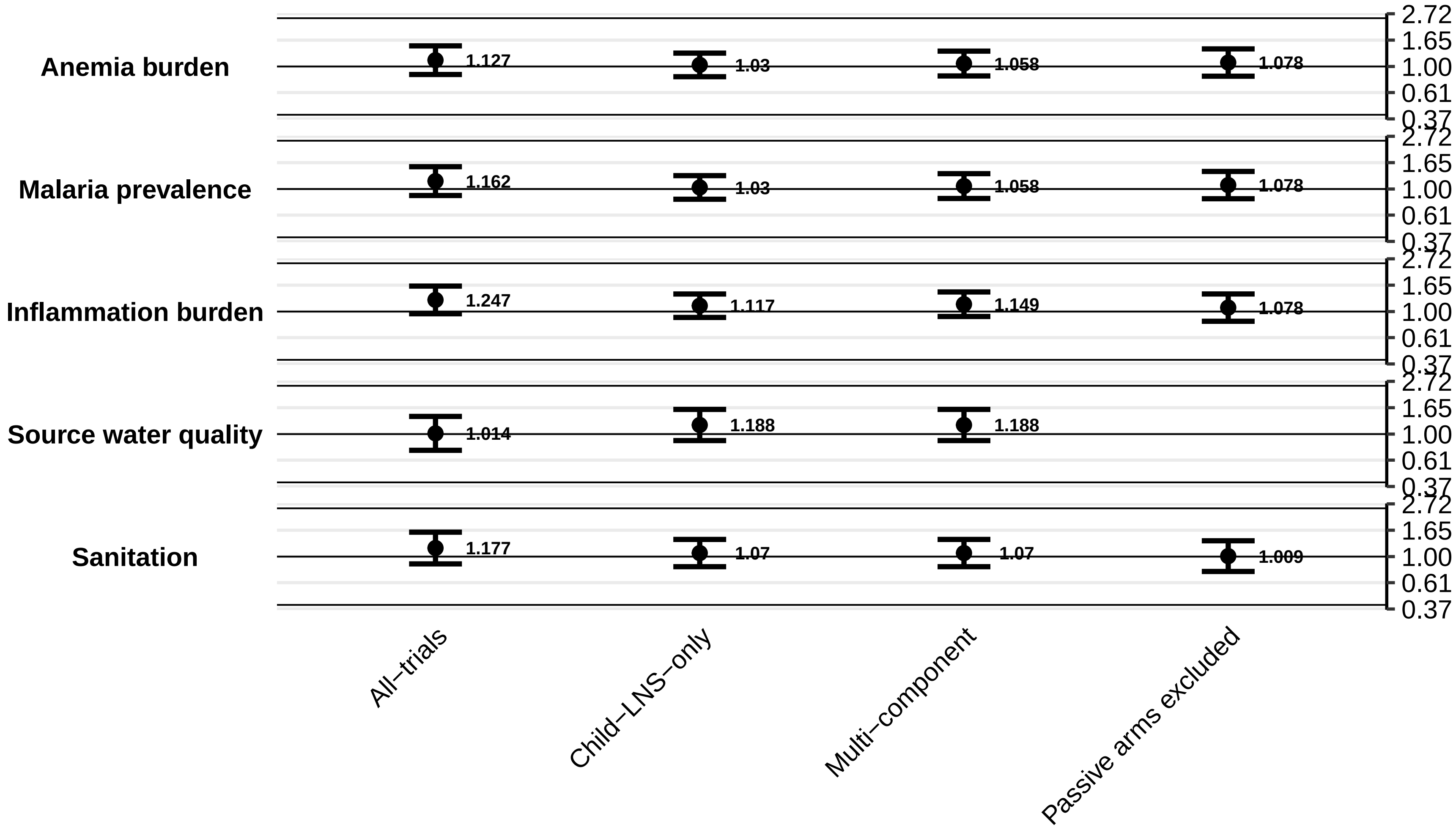

Supplemental figure 7D: Ratio of moderate-to-severe anemia prevalence ratios

7D2: By study design effect modifiers

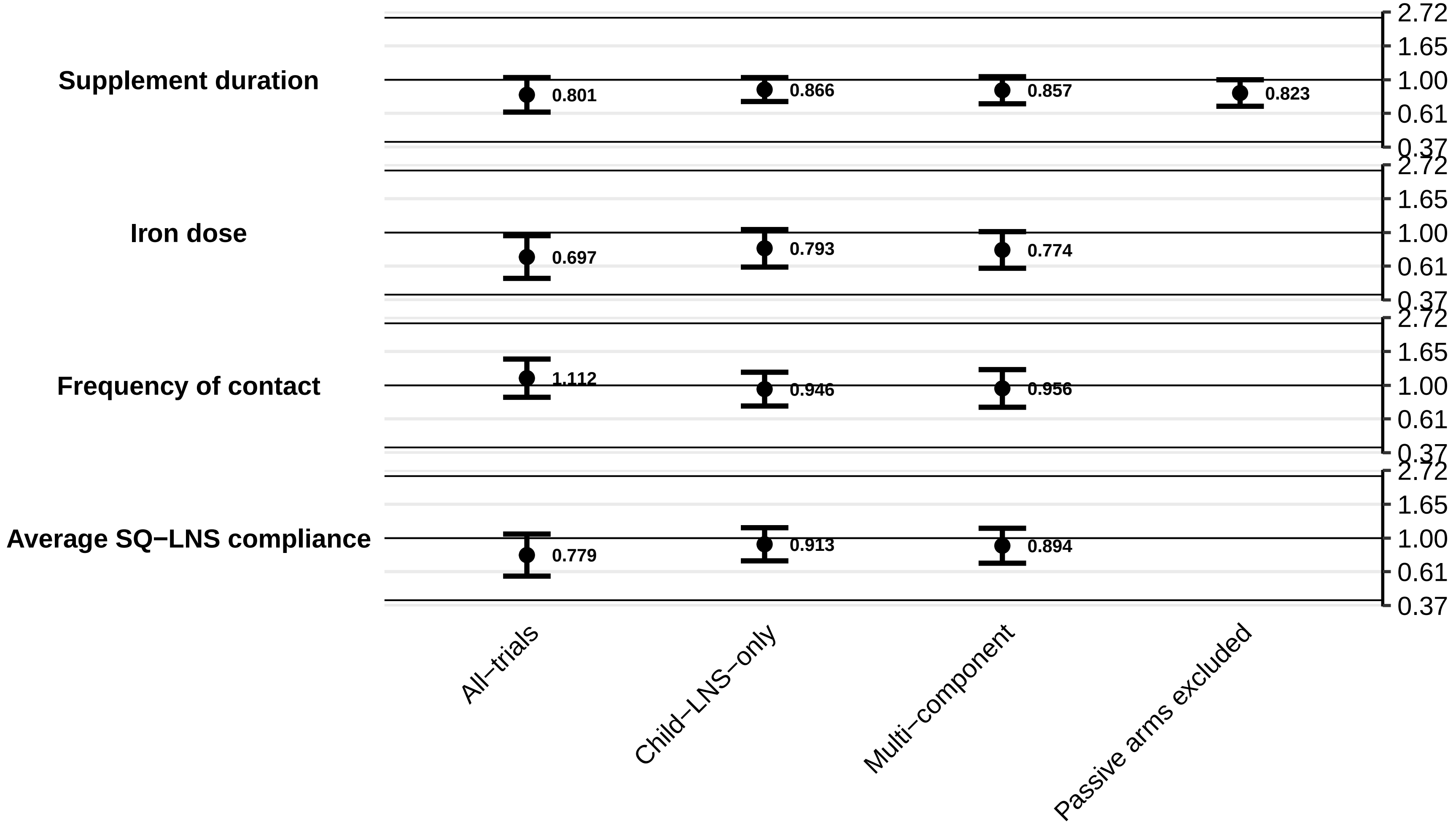

Supplemental figure 7E: Difference in moderate-to-severe anemia prevalence differences

7E1: By study context effect modifiers

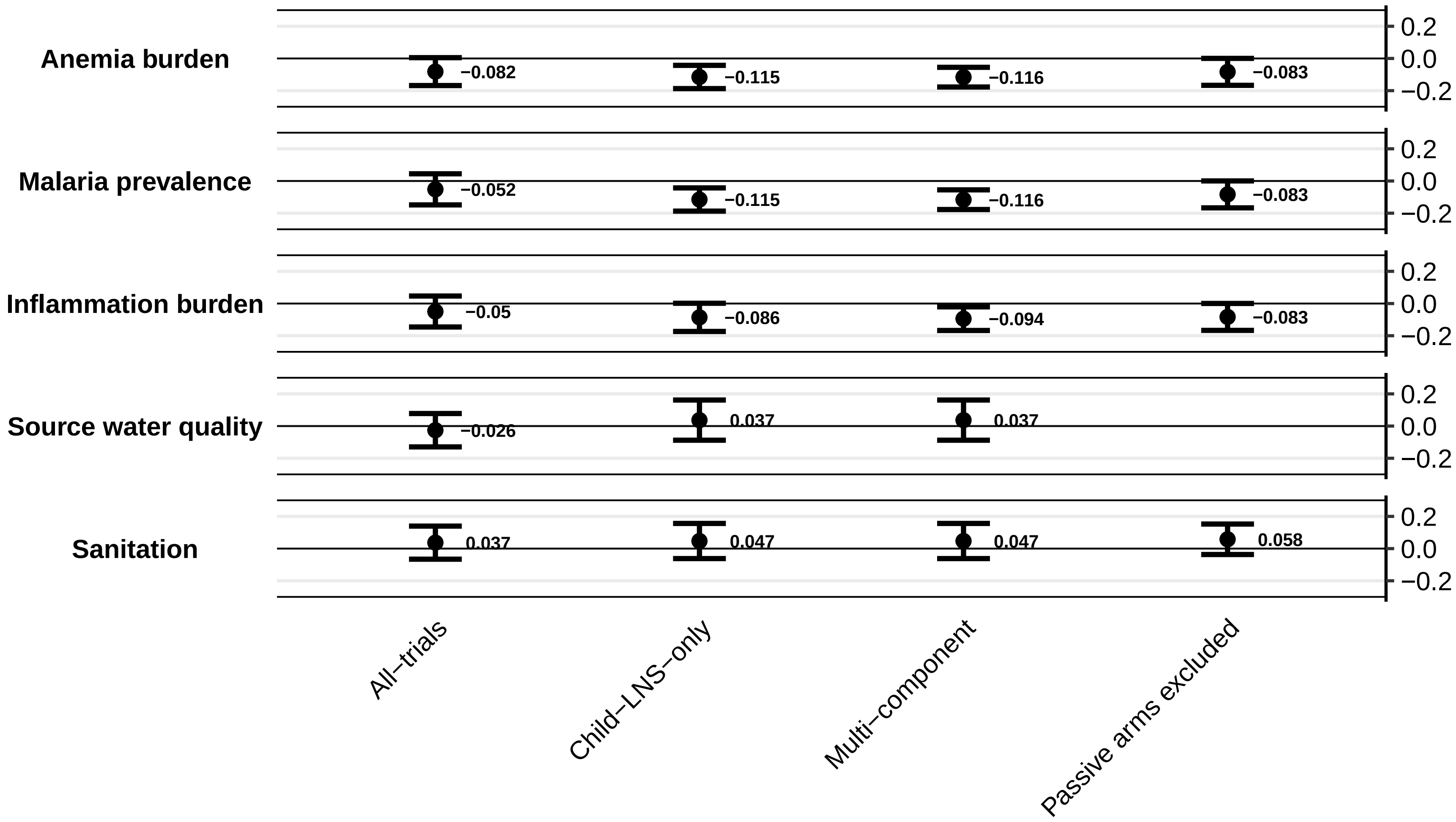

Supplemental figure 7E: Difference in moderate-to-severe anemia prevalence differences

7E2: By study design effect modifiers

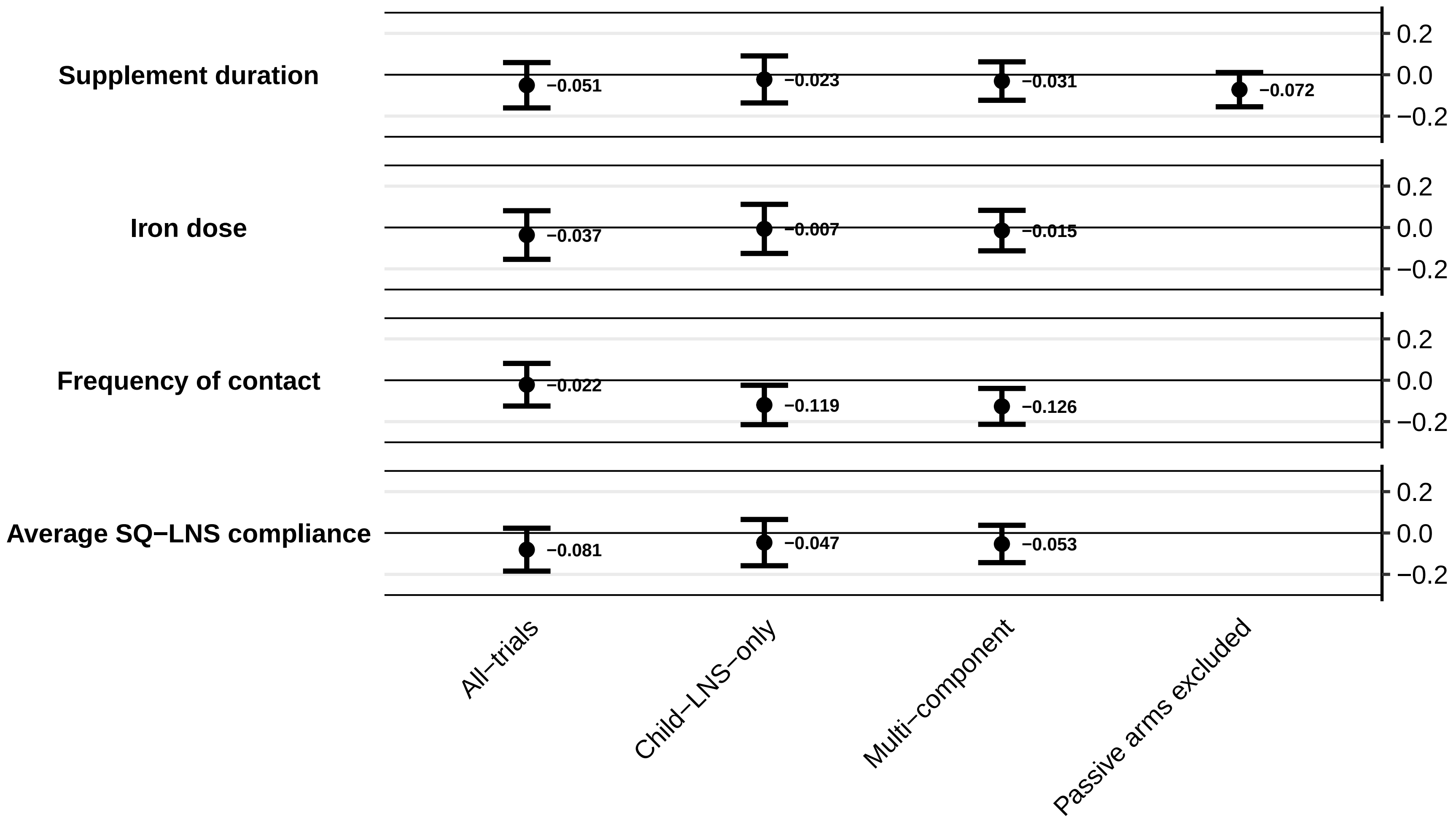

Supplemental figure 7F: Ratio of geometric mean ratios of ferritin concentration

7F1: By study context effect modifiers

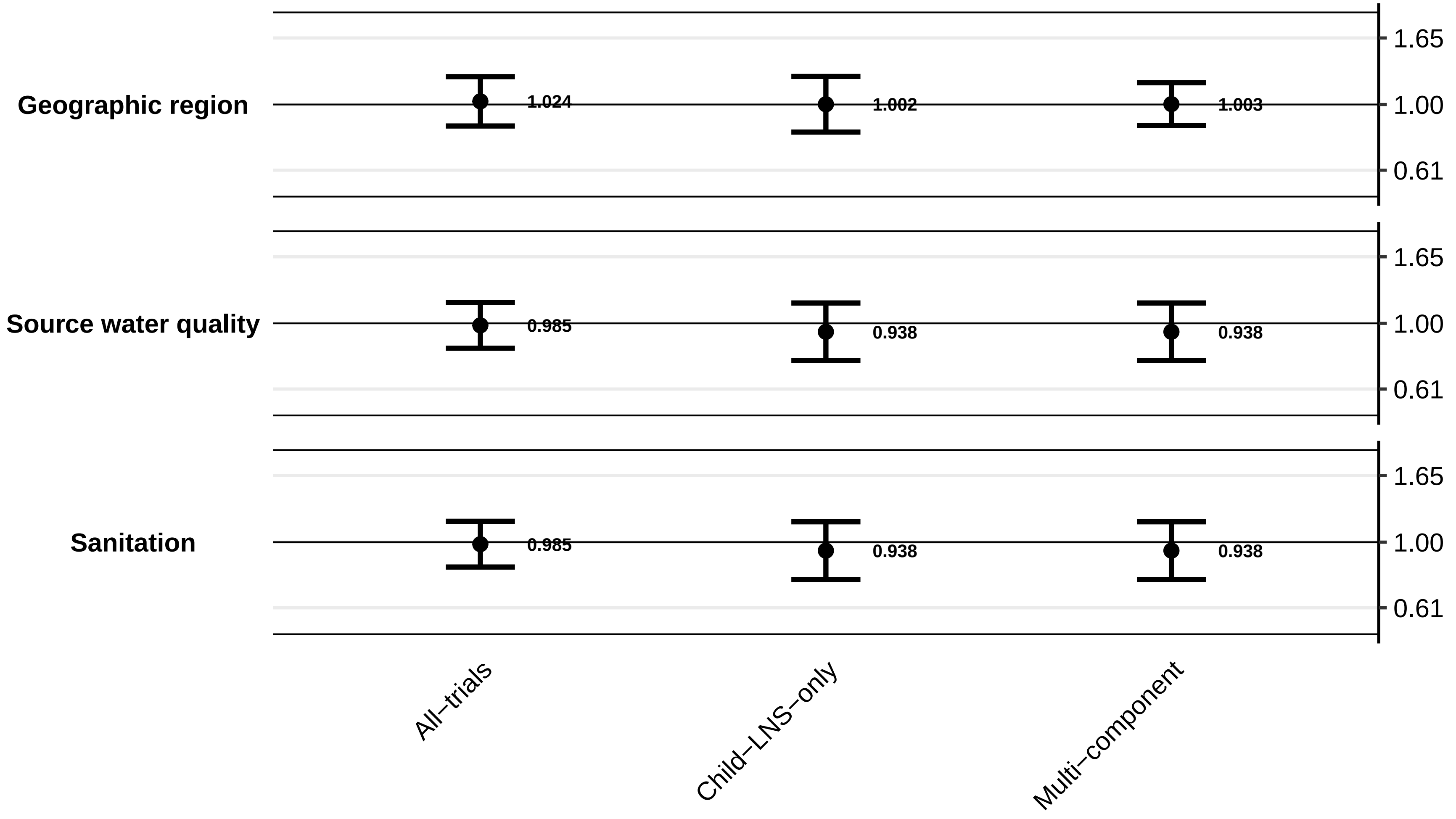

Supplemental figure 7F: Ratio of geometric mean ratios of ferritin concentration

7F2: By study design effect modifiers

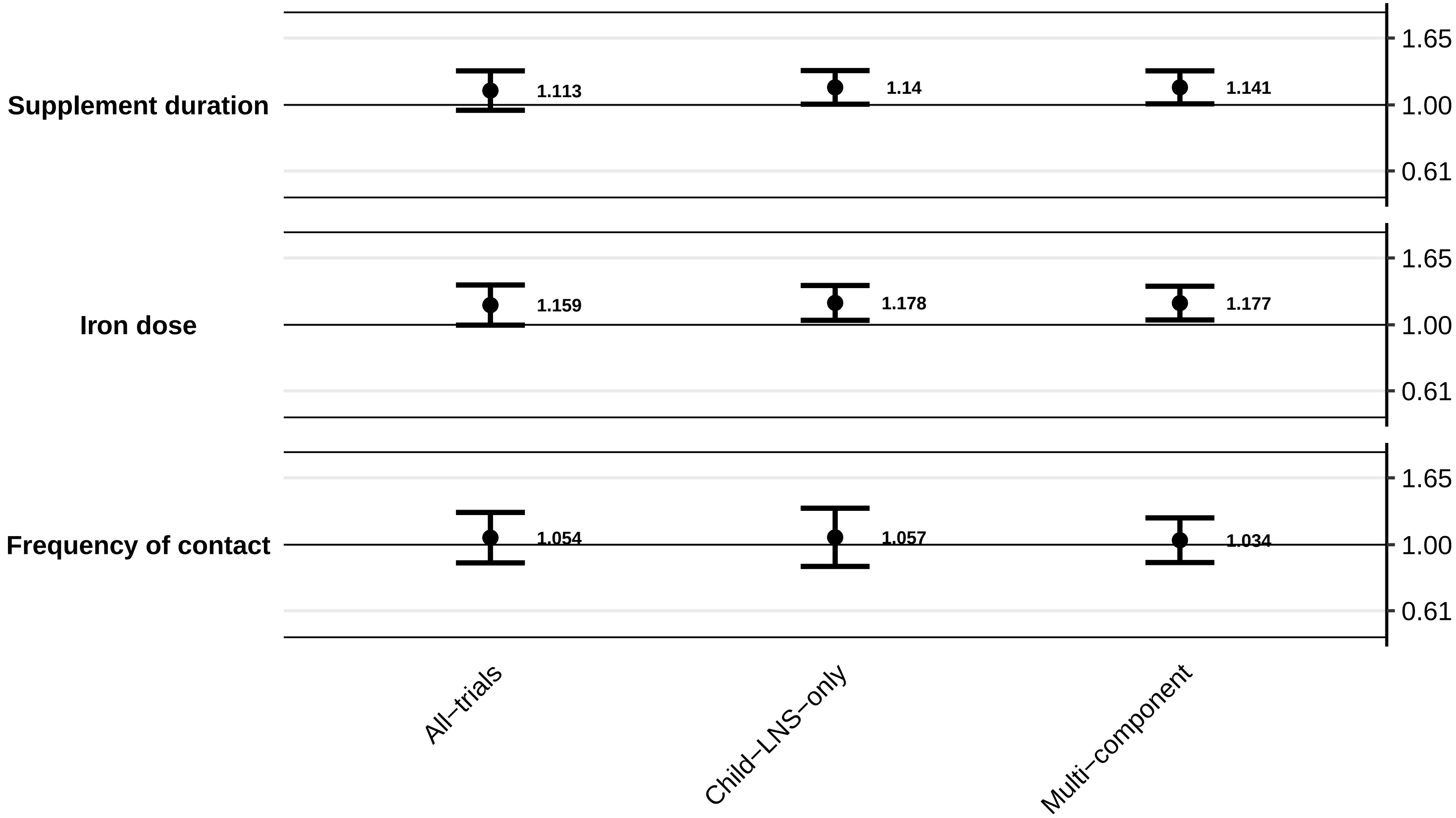

Supplemental figure 7G: Ratio of iron deficiency (ferritin < 12 μg/L) prevalence ratios

7G1: By study context effect modifiers

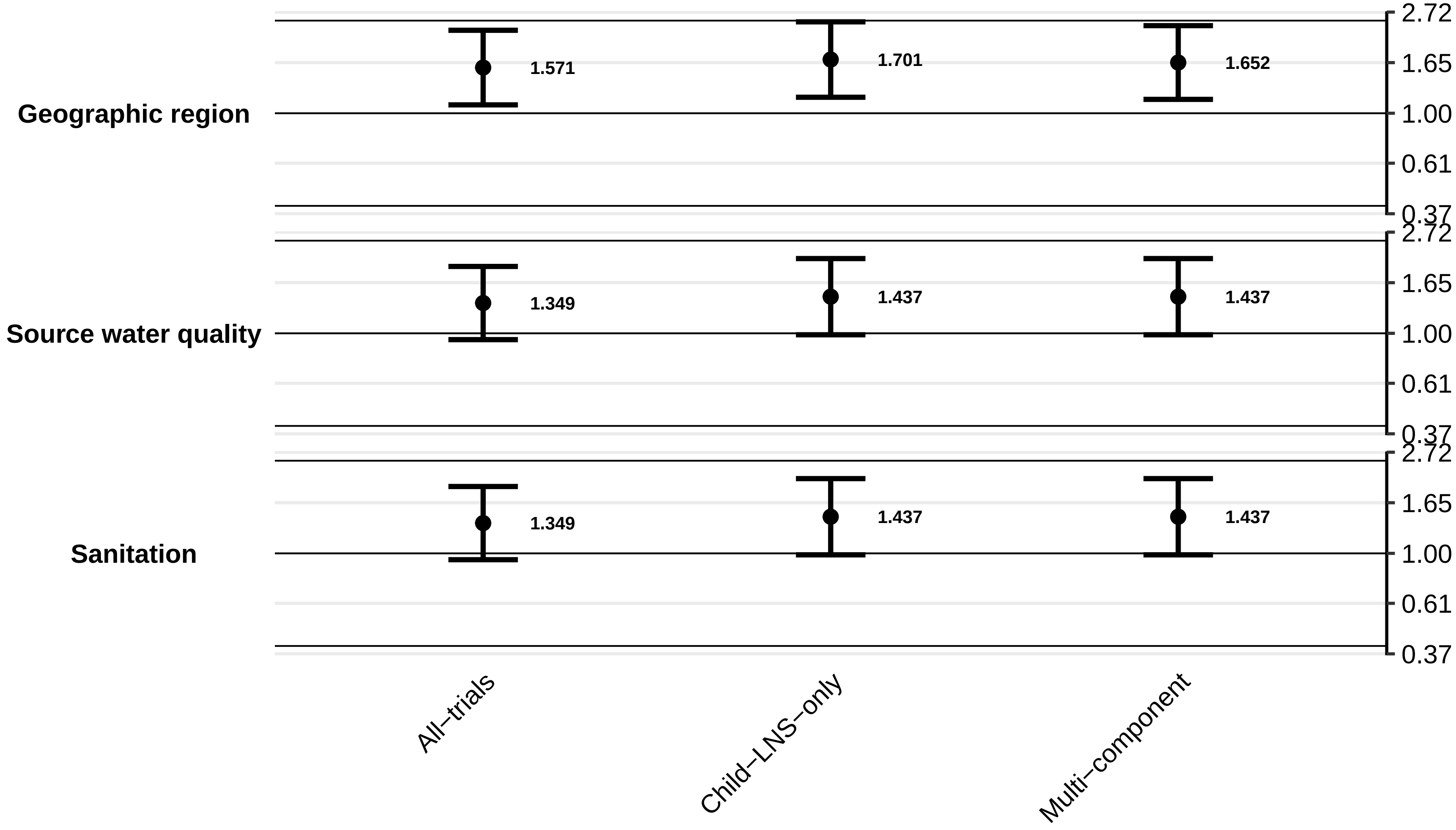

Supplemental figure 7G: Ratio of iron deficiency (ferritin < 12 μg/L) prevalence ratios

7G2: By study design effect modifiers

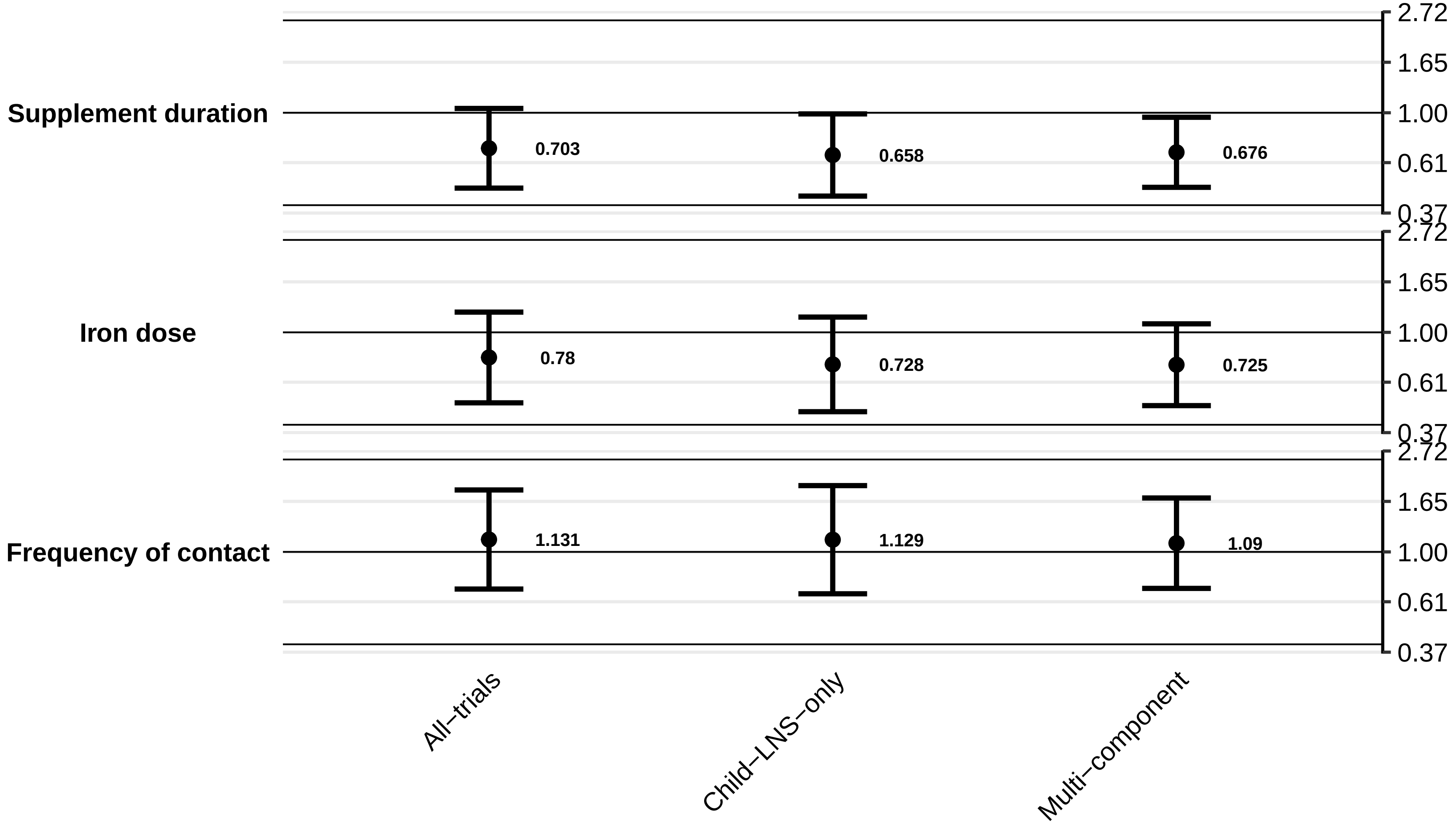

Supplemental figure 7H: Difference in iron deficiency (ferritin < 12 µg/L) prevalence differences

7H1: By study context effect modifiers

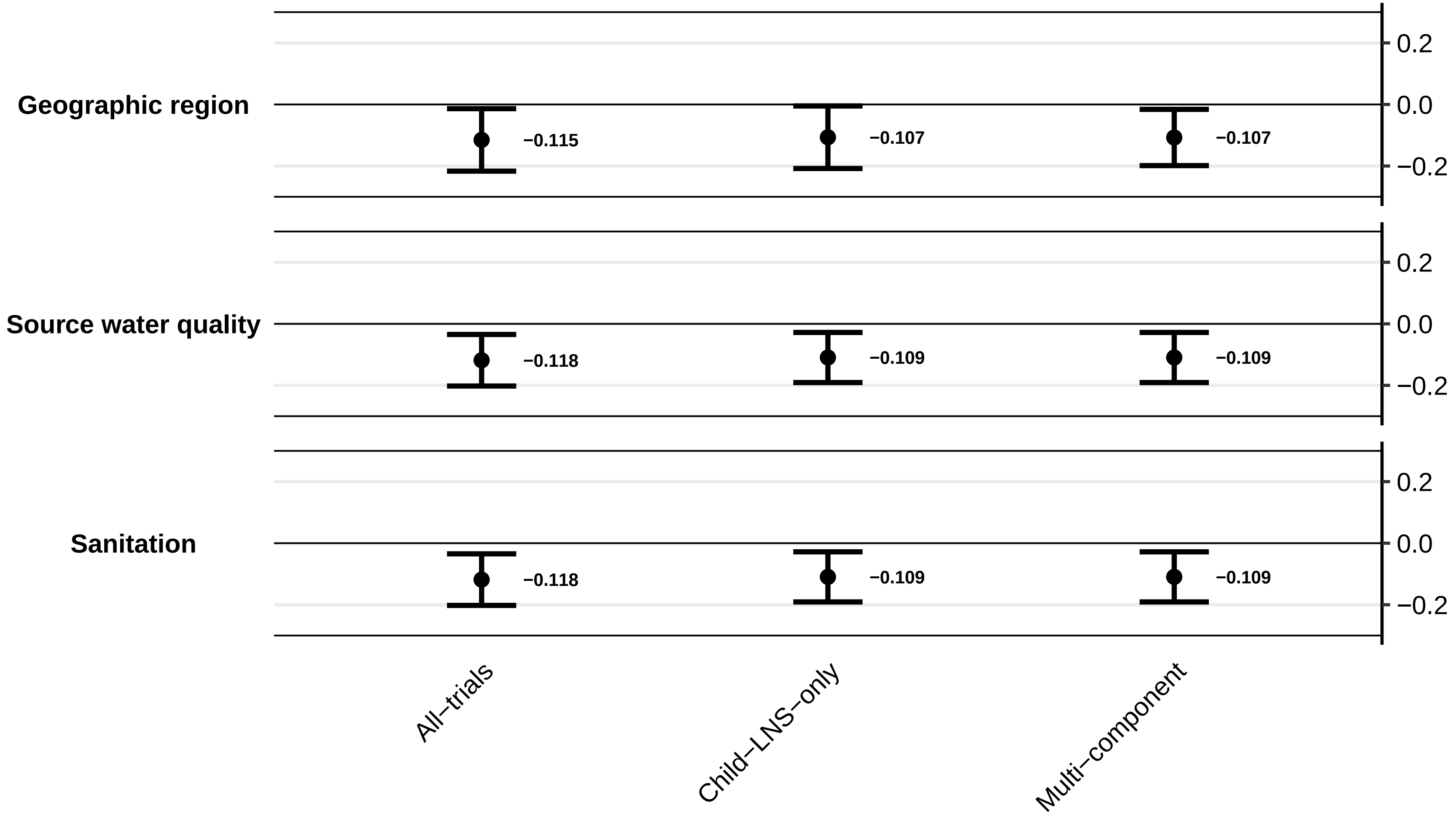

Supplemental figure 7H: Difference in iron deficiency (ferritin < 12 µg/L) prevalence differences

7H2: By study design effect modifiers

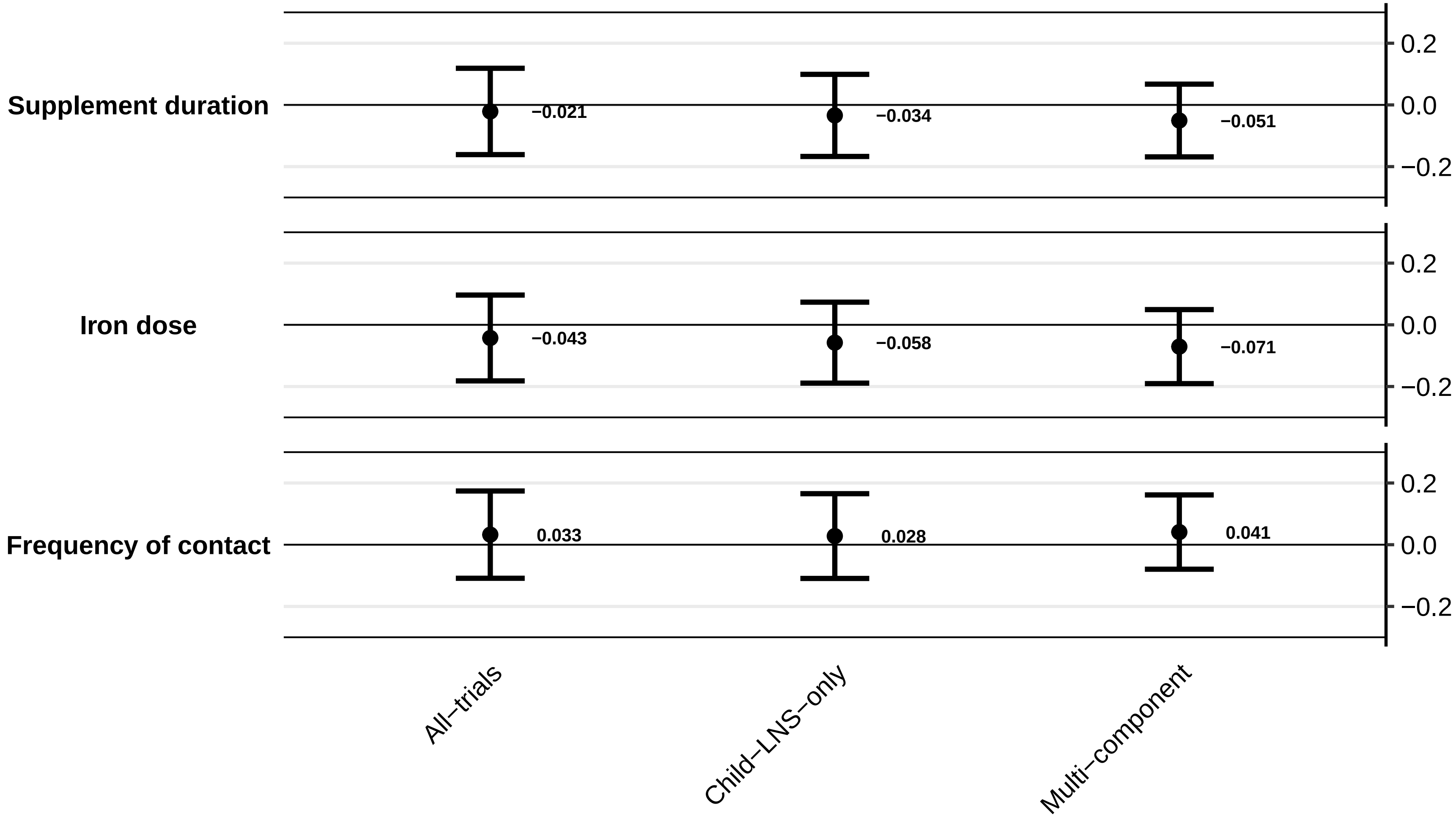

Supplemental figure 7I: Ratio of iron deficiency anemia prevalence ratios

7I1: By study context effect modifiers

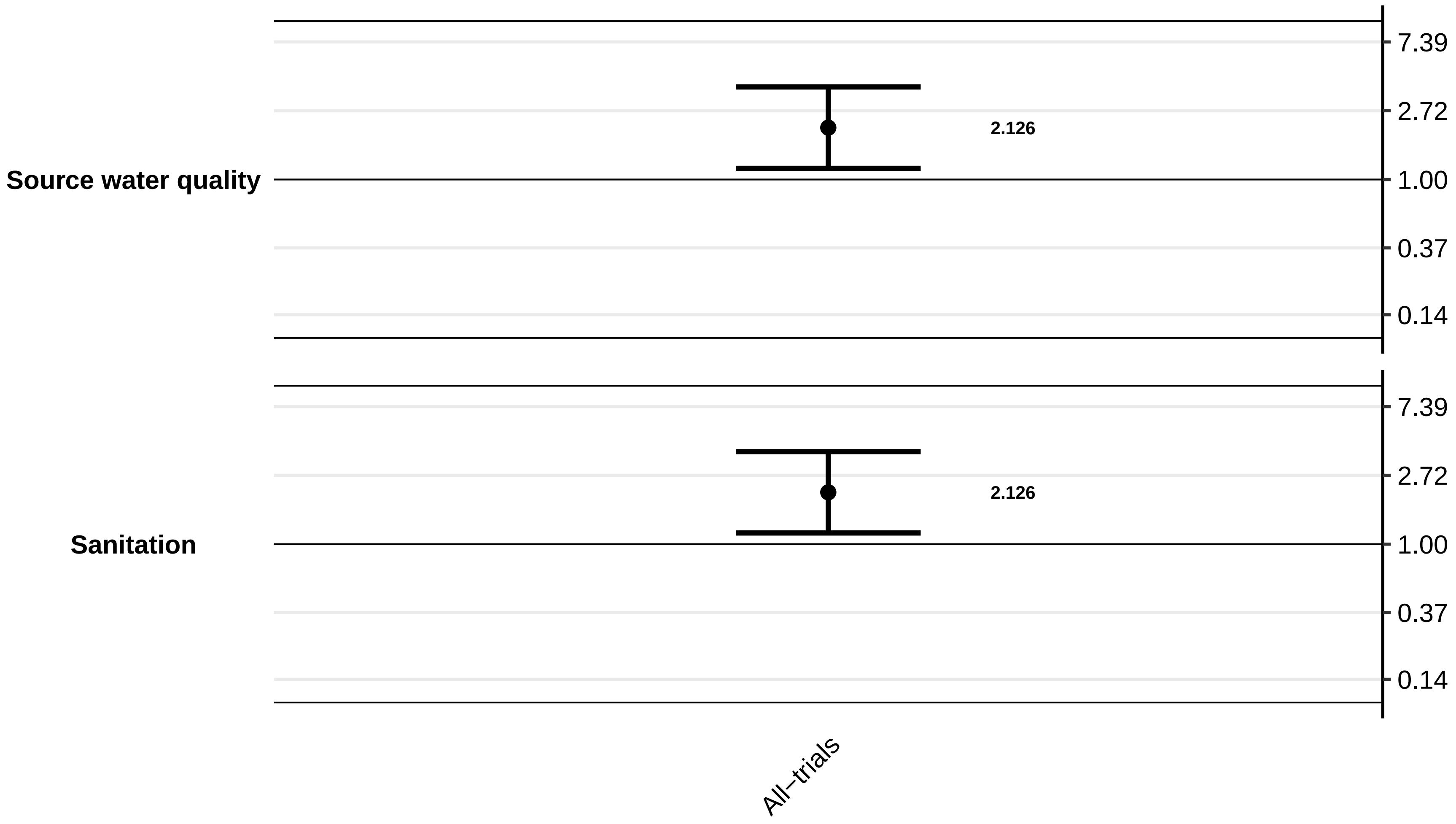

Supplemental figure 7I: Ratio of iron deficiency anemia prevalence ratios

7I2: By study design effect modifiers

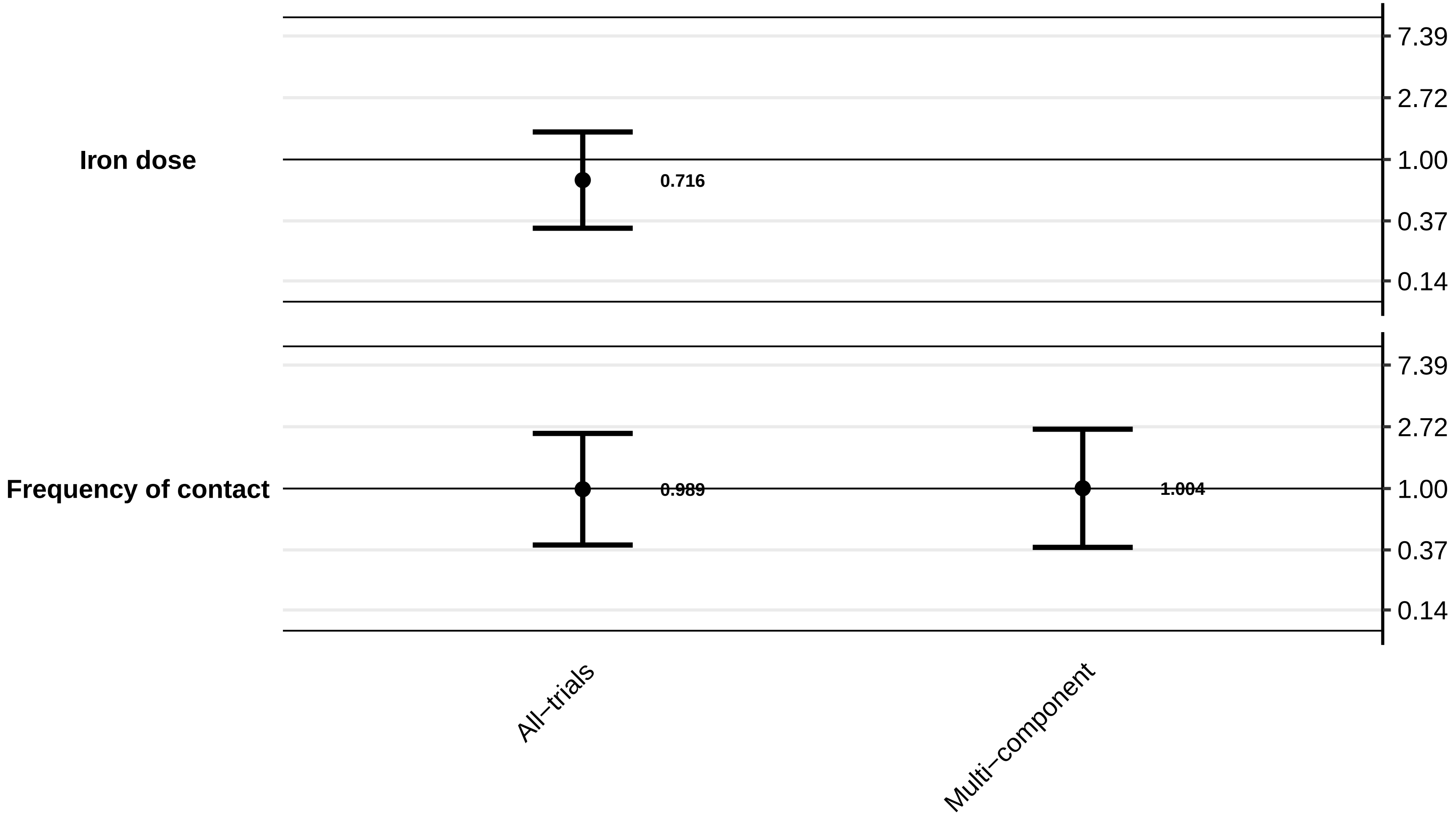

Supplemental figure 7J: Difference in iron deficiency anemia prevalence differences

7J1: By study context effect modifiers

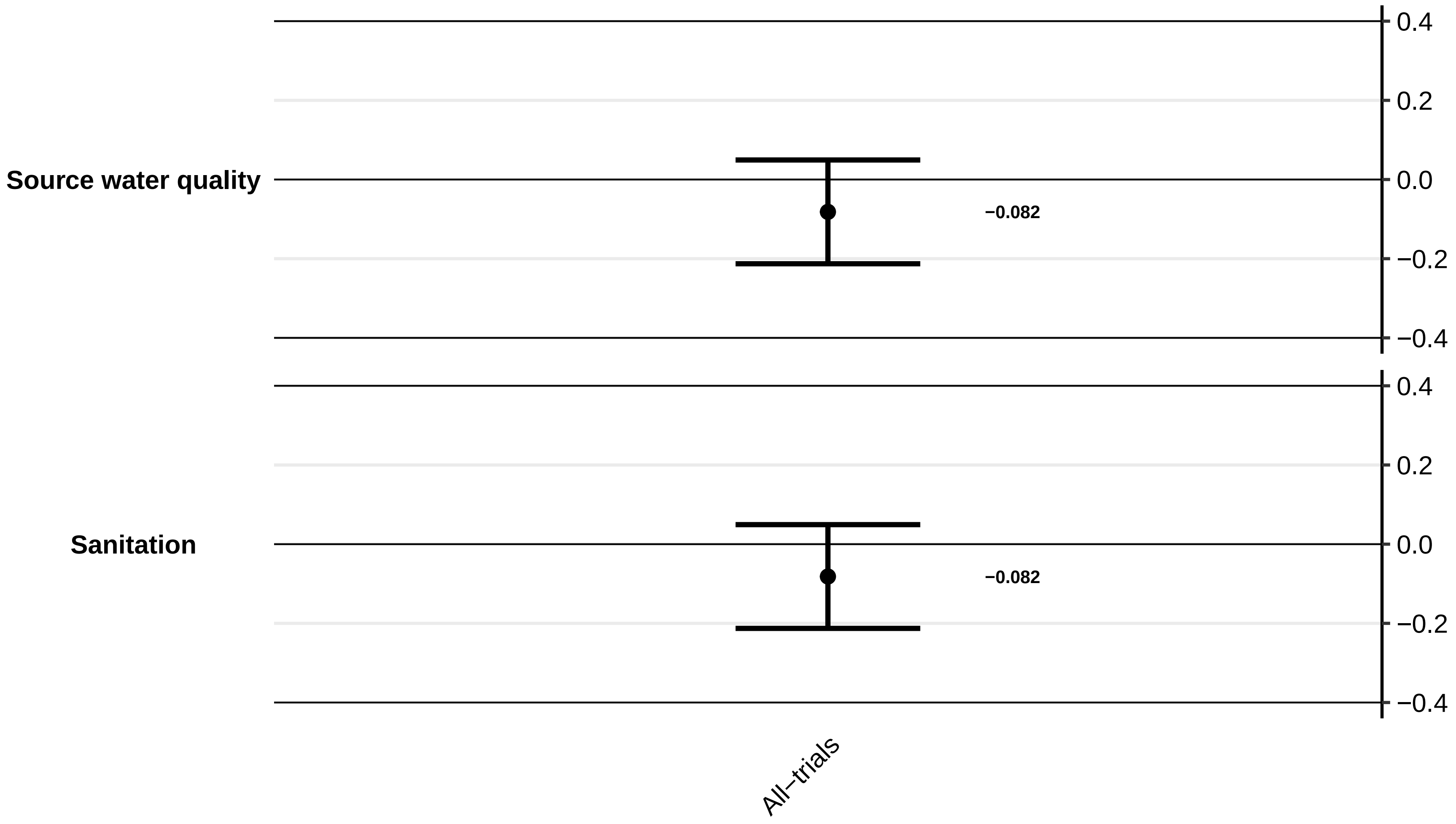

Supplemental figure 7J: Difference in iron deficiency anemia prevalence differences

7J2: By study design effect modifiers

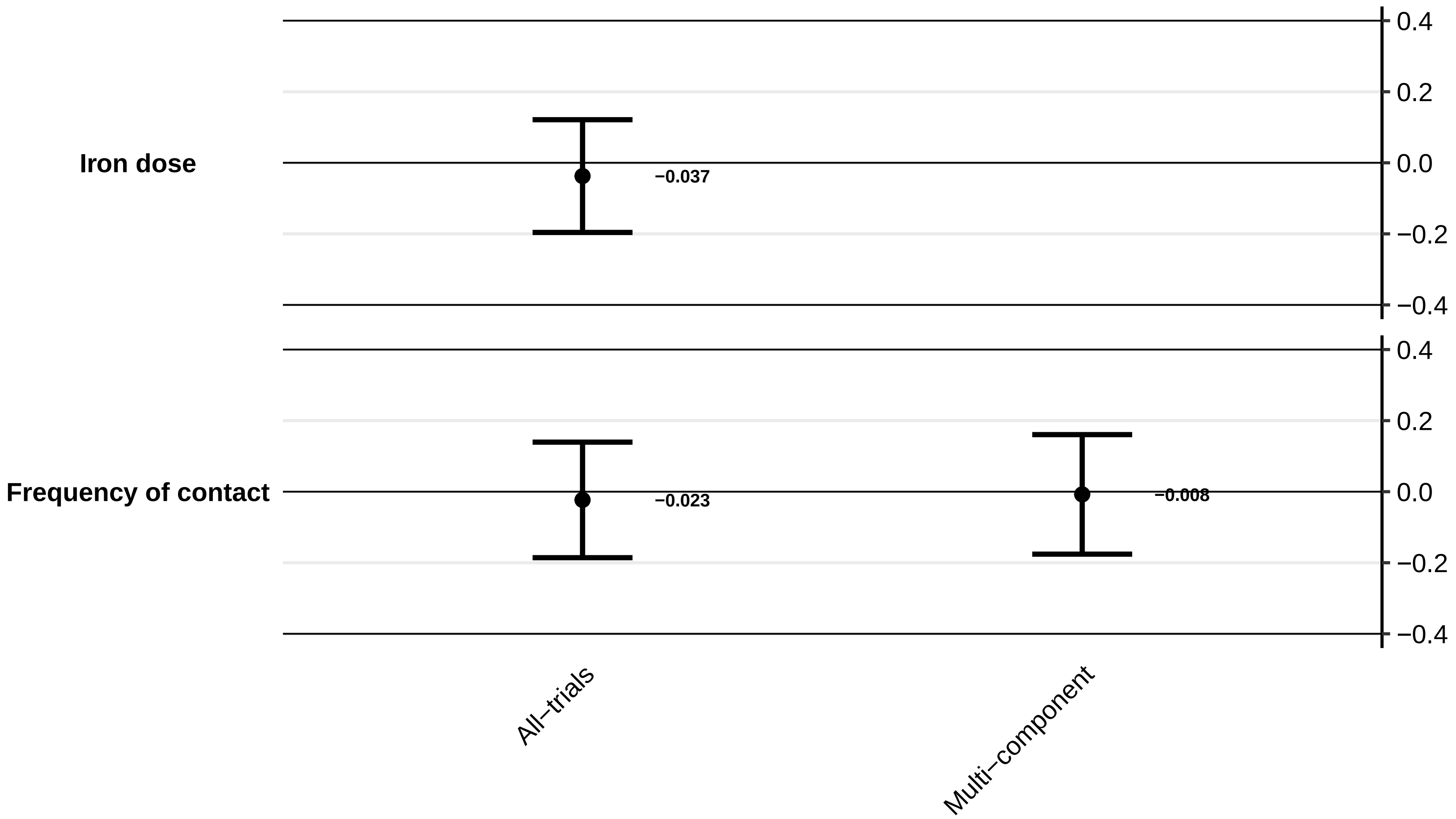

Supplemental figure 7K: Ratio of geometric mean ratios of soluble transferrin receptor concentration

7K1: By study context effect modifiers

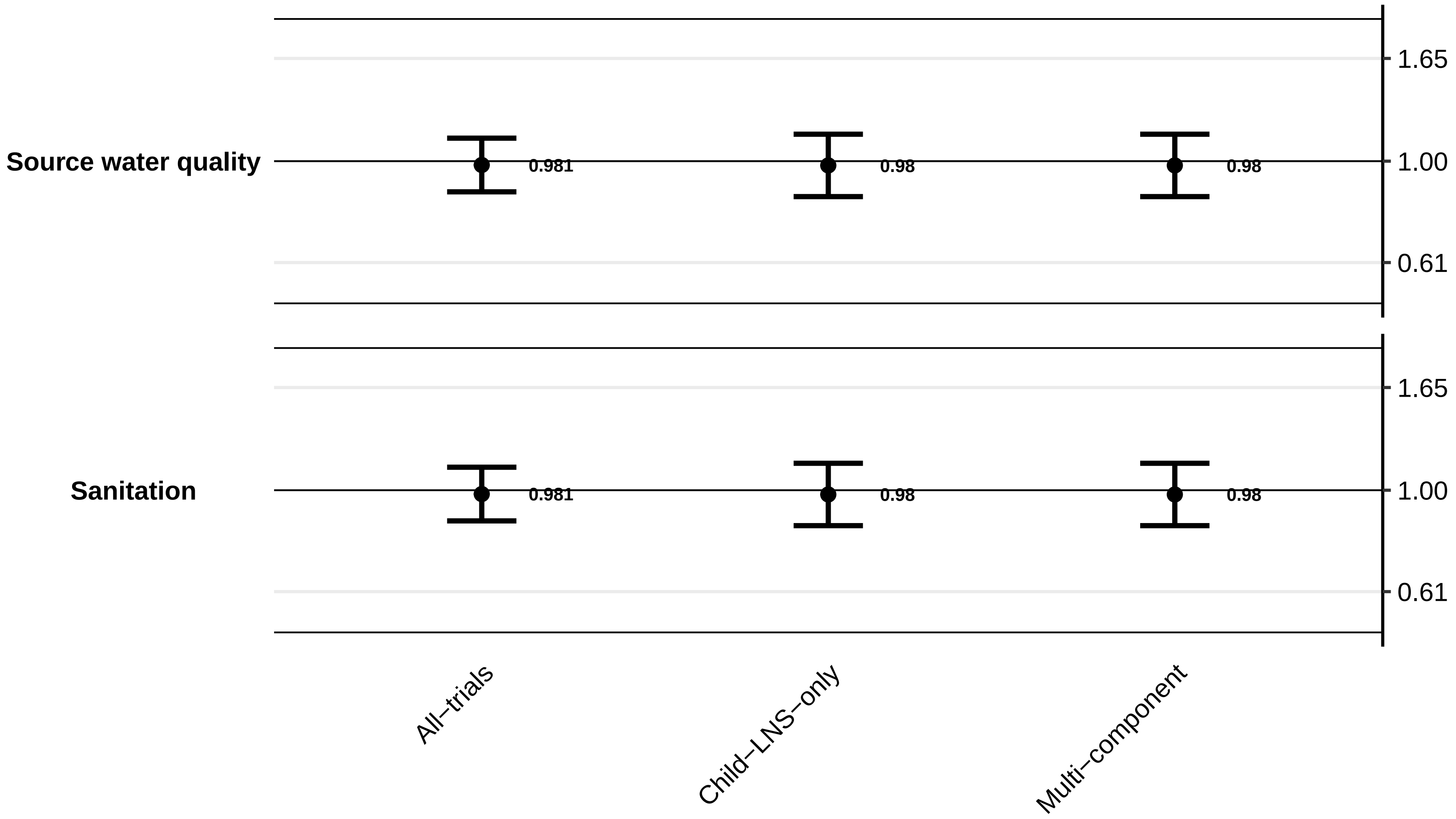

Supplemental figure 7K: Ratio of geometric mean ratios of soluble transferrin receptor concentration

7K2: By study design effect modifiers

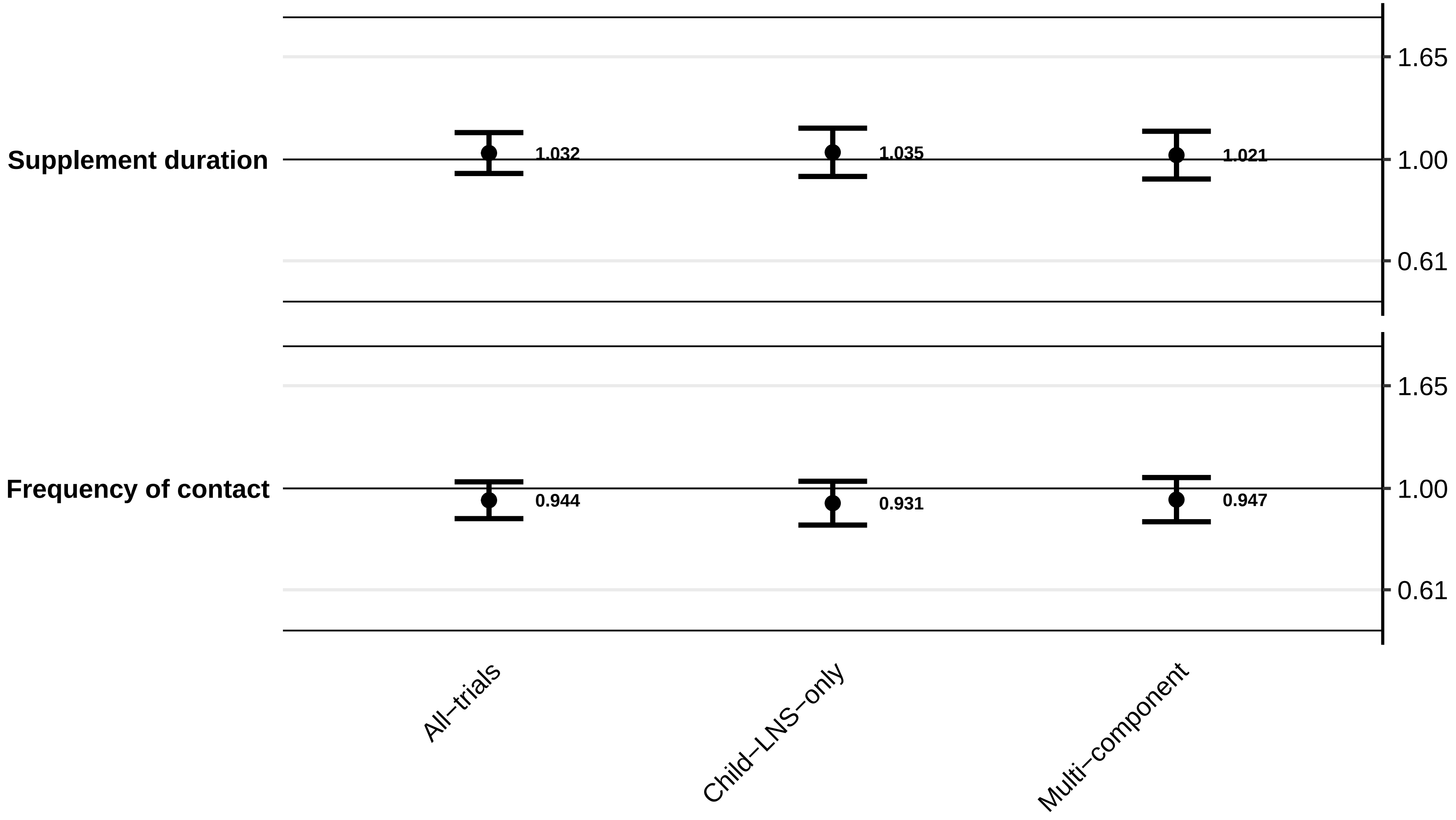

Supplemental figure 7L: Ratio of elevated soluble transferrin receptor prevalence ratios

7L1: By study context effect modifiers

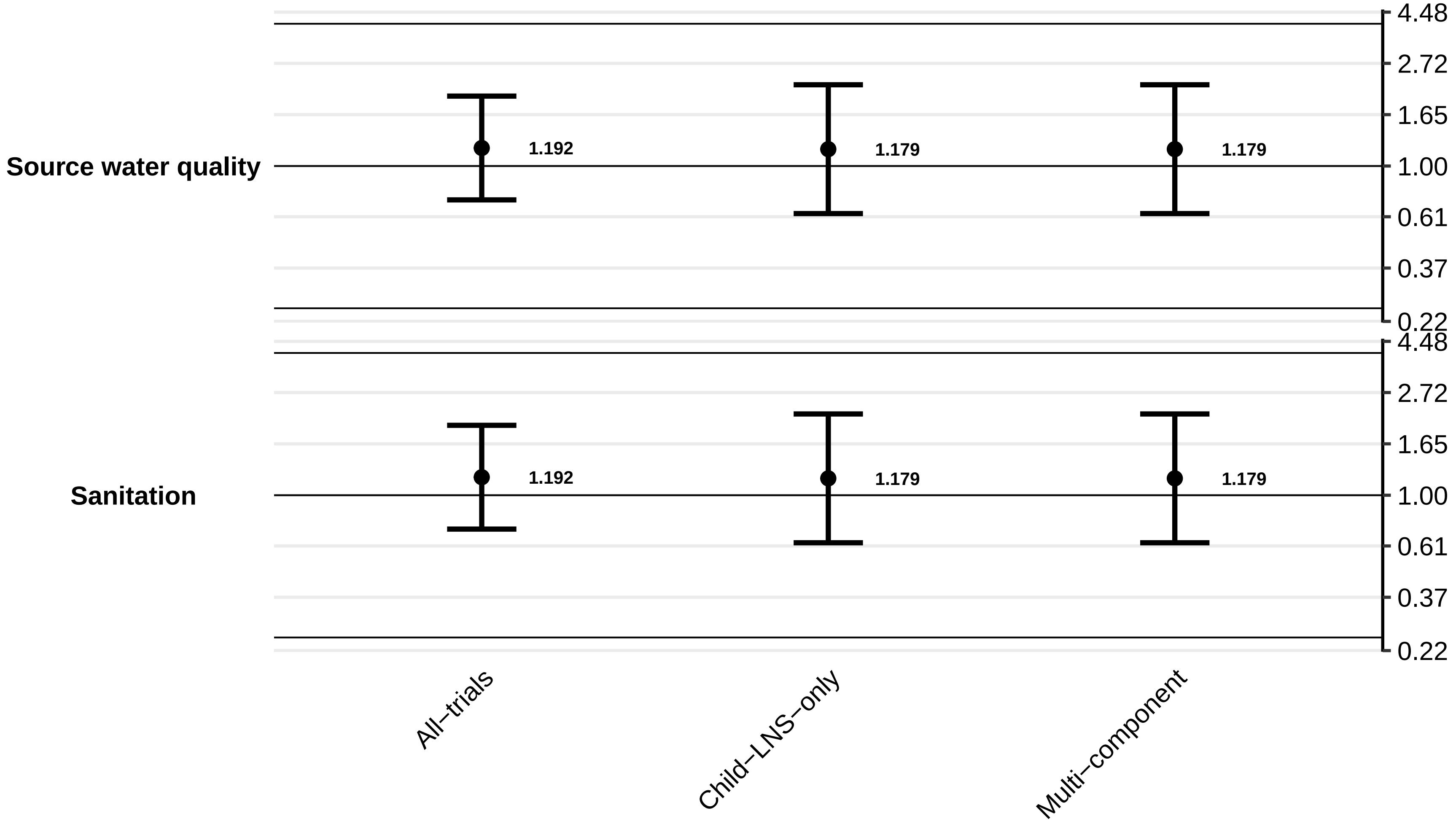

Supplemental figure 7L: Ratio of elevated soluble transferrin receptor prevalence ratios

7L2: By study design effect modifiers

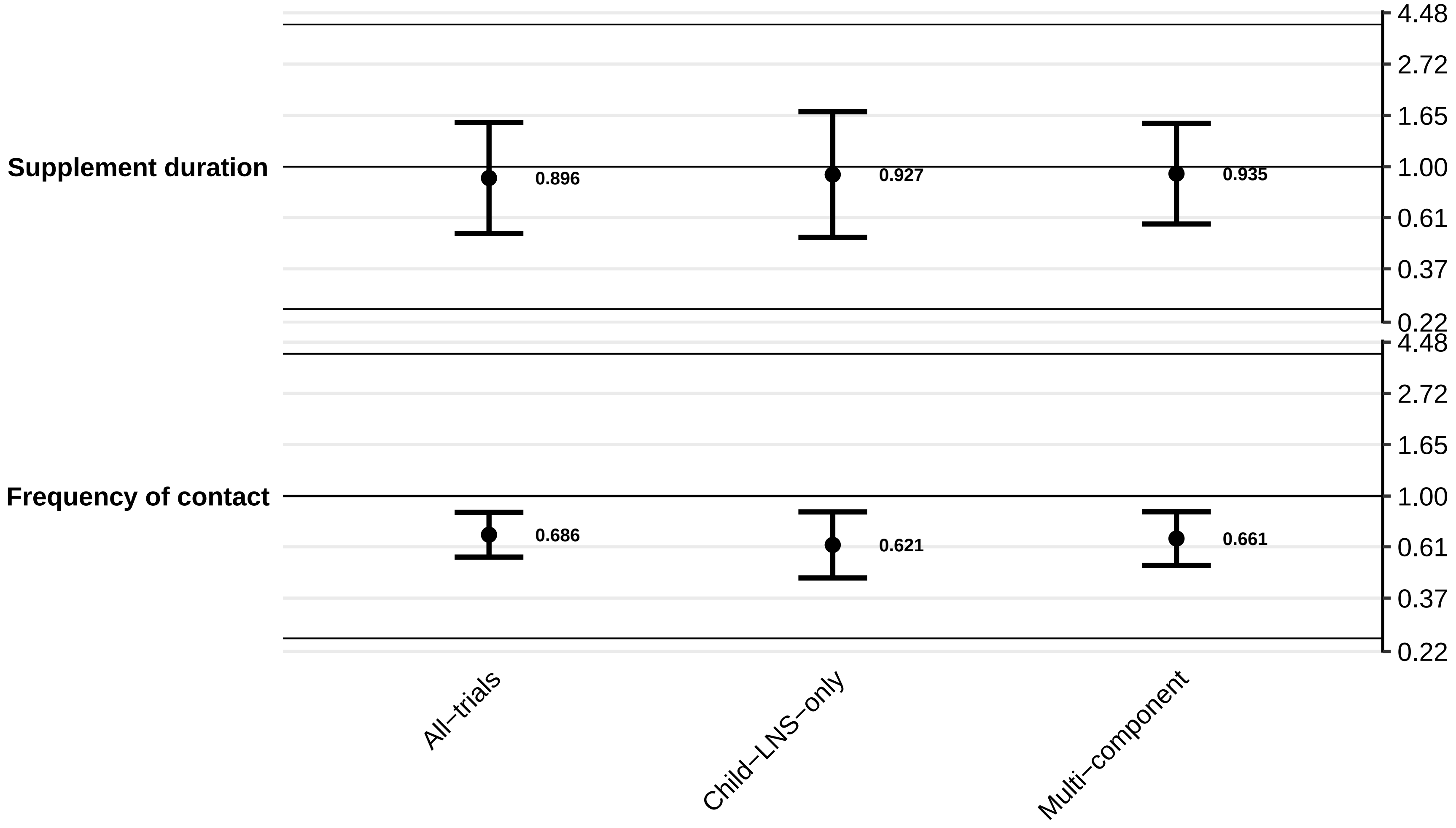

Supplemental figure 7M: Difference in elevated soluble transferrin receptor prevalence differences

7M1: By study context effect modifiers

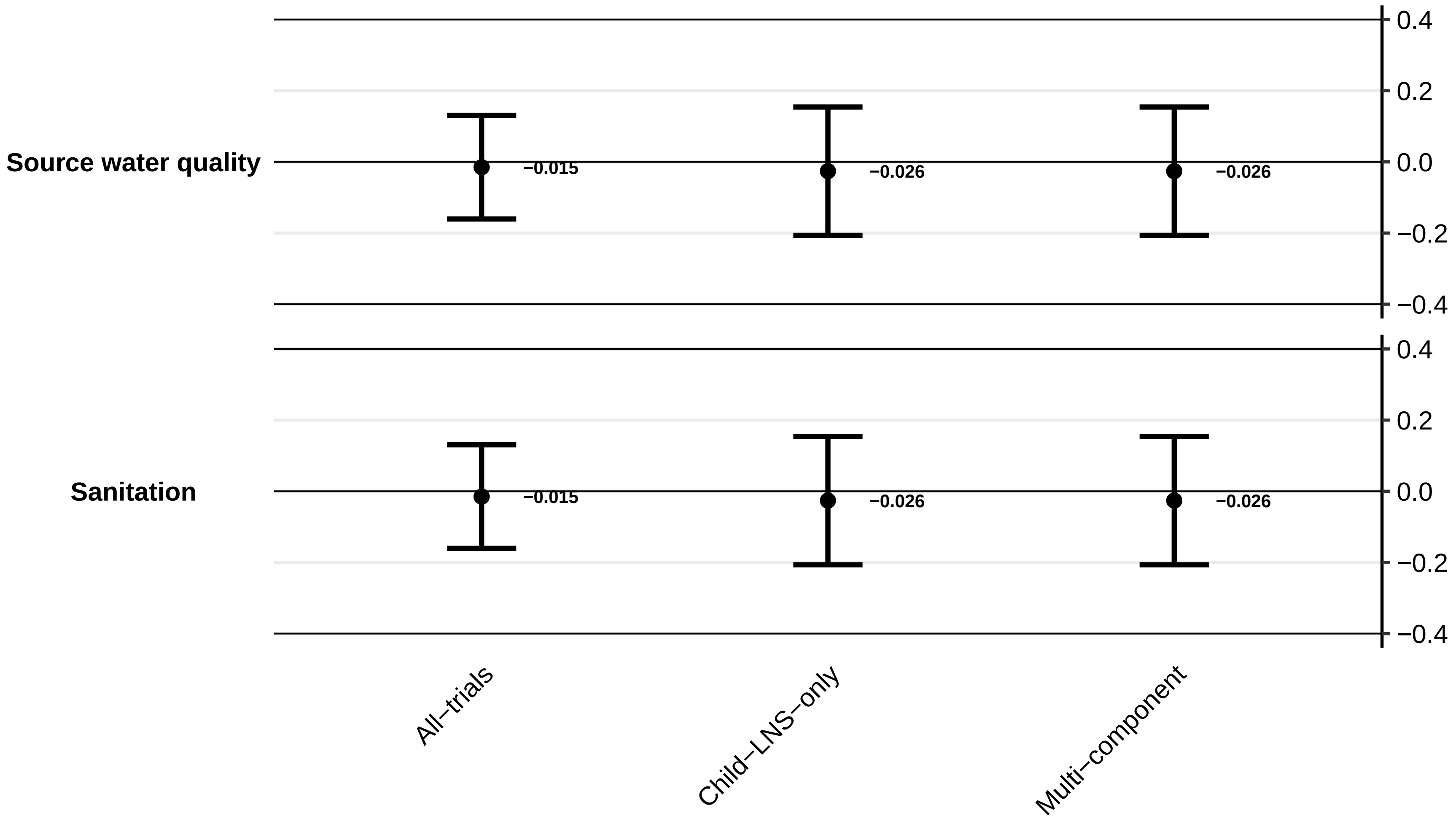

Supplemental figure 7M: Difference in elevated soluble transferrin receptor prevalence differences

7M2: By study design effect modifiers

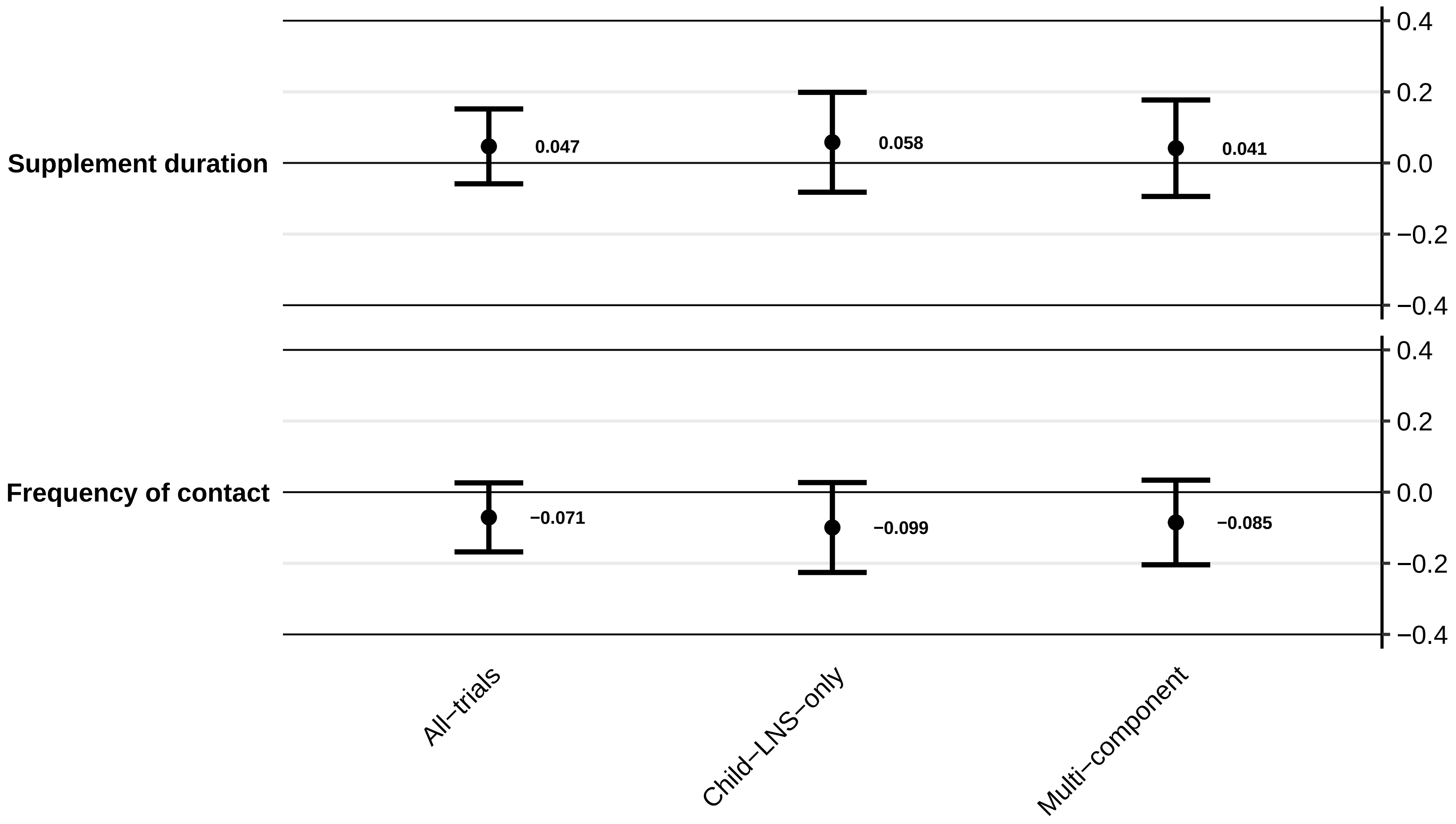

Supplemental figure 7N: Ratio of geometric mean ratios of zinc protoporphyrin concentration

7N1: By study context effect modifiers (insufficient comparisons)

Supplemental figure 7N: Ratio of geometric mean ratios of zinc protoporphyrin concentration

7N2: By study design effect modifiers (insufficient comparisons)

Supplemental figure 7O: Ratio of elevated zinc protoporphyrin prevalence ratios

7O1: By study context effect modifiers (insufficient comparisons)

Supplemental figure 7O: Ratio of elevated zinc protoporphyrin prevalence ratios

7O2: By study design effect modifiers (insufficient comparisons)

Supplemental figure 7P: Difference in elevated zinc protoporphyrin prevalence differences

7P1: By study context effect modifiers (insufficient comparisons)

Supplemental figure 7P: Difference in elevated zinc protoporphyrin prevalence differences

7P2: By study design effect modifiers (insufficient comparisons)

Supplemental figure 7Q: Ratio of geometric mean ratios of plasma zinc concentration

7Q1: By study context effect modifiers (insufficient comparisons)

Supplemental figure 7Q: Ratio of geometric mean ratios of plasma zinc concentration

7Q2: By study design effect modifiers (insufficient comparisons)

Supplemental figure 7R: Ratio of geometric mean ratios of retinol concentration

7R1: By study context effect modifiers (insufficient comparisons)

Supplemental figure 7R: Ratio of geometric mean ratios of retinol concentration

7R2: By study design effect modifiers (insufficient comparisons)

Supplemental figure 7S: Ratio of low vitamin A (retinol < 0.70 µmol/L) prevalence ratios

7S1: By study context effect modifiers (insufficient comparisons)

Supplemental figure 7S: Ratio of low vitamin A (retinol < 0.70 µmol/L) prevalence ratios

7S2: By study design effect modifiers (insufficient comparisons)

Supplemental figure 7T: Difference in low vitamin A (retinol < 0.70 µmol/L) prevalence differences

7T1: By study context effect modifiers (insufficient comparisons)

Supplemental figure 7T: Difference in low vitamin A (retinol < 0.70 µmol/L) prevalence differences

7T2: By study design effect modifiers (insufficient comparisons)

Supplemental figure 7U: Ratio of marginal vitamin A (retinol < 1.05 µmol/L) prevalence ratios

7U1: By study context effect modifiers (insufficient comparisons)

Supplemental figure 7U: Ratio of marginal vitamin A (retinol < 1.05 µmol/L) prevalence ratios

7U2: By study design effect modifiers (insufficient comparisons)

Supplemental figure 7V: Difference in marginal vitamin A (retinol < 1.05 µmol/L) prevalence differences

7V1: By study context effect modifiers (insufficient comparisons)

Supplemental figure 7V: Difference in marginal vitamin A (retinol < 1.05 μmol/L) prevalence differences

7V2: By study design effect modifiers (insufficient comparisons)

Supplemental figure 7W: Ratio of geometric mean ratio of retinol binding protein concentrations

7W1: By study context effect modifiers (insufficient comparisons)

Supplemental figure 7W: Ratio of geometric mean ratio of retinol binding protein concentrations

7W2: By study design effect modifiers (insufficient comparisons)

Supplemental figure 7X: Ratio of Low vitamin A status (RBP < 0.70 μmol/L) prevalence ratios

7X1: By study context effect modifiers (insufficient comparisons)

Supplemental figure 7X: Ratio of Low vitamin A status (RBP < 0.70 µmol/L) prevalence ratios

7X2: By study design effect modifiers (insufficient comparisons)

Supplemental figure 7Y: Difference in low vitamin A status (RBP < 0.70 µmol/L) prevalence differences

7Y1: By study context effect modifiers (insufficient comparisons)

Supplemental figure 7Y: Difference in low vitamin A status (RBP < 0.70 µmol/L) prevalence differences

7Y2: By study design effect modifiers (insufficient comparisons)

Supplemental figure 7Z: Ratio of marginal vitamin A status (RBP < 1.05 µmol/L) prevalence ratios

7Z1: By study context effect modifiers (insufficient comparisons)

Supplemental figure 7Z: Ratio of marginal vitamin A status (RBP < 1.05 µmol/L) prevalence ratios

7Z2: By study design effect modifiers (insufficient comparisons)

Supplemental figure 7AA: Difference in marginal vitamin A status (RBP < 1.05 µmol/L) prevalence differences

7AA1: By study context effect modifiers (insufficient comparisons)

Supplemental figure 7AA: Difference in marginal vitamin A status (RBP < 1.05 µmol/L) prevalence differences

7AA2: By study design effect modifiers (insufficient comparisons)
