# Supplementary material for: Characteristics that modify the effect of small-quantity lipid-based nutrient supplementation on child anemia and micronutrient status: an individual participant data meta-analysis of randomized controlled trials
Source: Am J Clin Nutr. 2021 Sep 29;114(Suppl 1):68S–94S. doi: 10.1093/ajcn/nqab276 (PMC8560313; doi:10.1093/ajcn/nqab276)

Supplemental figure 8: Forest plots for effects of SQ-LNS on biochemical outcomes stratified by individual-level maternal and child effect modifiers

Contents

Supplemental figure 8A: Mean difference in hemoglobin concentration **7**
8A1: Stratified by Maternal BMI . . . 7
8A2: Stratified by Maternal age . . . 8
8A3: Stratified by Maternal education . . . 9
8A4: Stratified by Child sex . . . 10
8A5: Stratified by Child birth order . . . 11
8A6: Stratified by Child baseline acute malnutrition . . . 12
8A7: Stratified by Child baseline anemia . . . 13
8A8: Stratified by Child high-dose vitamin A supplementation . . . 14
8A9: Stratified by Child inflammation . . . 15

Supplemental figure 8B: Anemia prevalence ratio **16**
8B1: Stratified by Maternal BMI . . . 16
8B2: Stratified by Maternal age . . . 17
8B3: Stratified by Maternal education . . . 18
8B4: Stratified by Child sex . . . 19
8B5: Stratified by Child birth order . . . 20
8B6: Stratified by Child baseline acute malnutrition . . . 21
8B7: Stratified by Child baseline anemia . . . 22
8B8: Stratified by Child high-dose vitamin A supplementation . . . 23
8B9: Stratified by Child inflammation . . . 24

Supplemental figure 8C: Anemia prevalence difference **25**
8C1: Stratified by Maternal BMI . . . 25
8C2: Stratified by Maternal age . . . 26
8C3: Stratified by Maternal education . . . 27
8C4: Stratified by Child sex . . . 28
8C5: Stratified by Child birth order . . . 29
8C6: Stratified by Child baseline acute malnutrition . . . 30
8C7: Stratified by Child baseline anemia . . . 31
8C8: Stratified by Child high-dose vitamin A supplementation . . . 32
8C9: Stratified by Child inflammation . . . 33

Supplemental figure 8D: Moderate-to-severe anemia prevalence ratio **34**
8D1: Stratified by Maternal BMI . . . 34
8D2: Stratified by Maternal age . . . 35
8D3: Stratified by Maternal education . . . 36
8D4: Stratified by Child sex . . . 37
8D5: Stratified by Child birth order . . . 38
8D6: Stratified by Child baseline acute malnutrition (insufficient comparisons) . . . 39
8b7: Stratified by Child baseline anemia . . . 40

8D8: Stratified by Child high-dose vitamin A supplementation . . . 41
8D9: Stratified by Child inflammation . . . 42

Supplemental figure 8E: Moderate-to-severe anemia prevalence difference **43**
8E1: Stratified by Maternal BMI . . . 43
8E2: Stratified by Maternal age . . . 44
8E3: Stratified by Maternal education . . . 45
8E4: Stratified by Child sex . . . 46
8E5: Stratified by Child birth order . . . 47
8E6: Stratified by Child baseline acute malnutrition (insufficient comparisons) . . . 48
8E7: Stratified by Child baseline anemia . . . 49
8E8: Stratified by Child high-dose vitamin A supplementation . . . 50
8E9: Stratified by Child inflammation . . . 51

Supplemental figure 8F: Geometric mean ratio of ferritin concentration **52**
8F1: Stratified by Maternal BMI . . . 52
8F2: Stratified by Maternal age . . . 53
8F3: Stratified by Maternal education . . . 54
8F4: Stratified by Child sex . . . 55
8F5: Stratified by Child birth order . . . 56
8F6: Stratified by Child baseline acute malnutrition . . . 57
8F7: Stratified by Child baseline anemia (insufficient comparisons) . . . 58
8F8: Stratified by Child high-dose vitamin A supplementation . . . 59
8F9: Stratified by Child inflammation . . . 60

Supplemental figure 8G: Iron deficiency (ferritin $<$ 12 µg/L) prevalence ratio **61**
8G1: Stratified by Maternal BMI . . . 61
8G2: Stratified by Maternal age . . . 62
8G3: Stratified by Maternal education . . . 63
8G4: Stratified by Child sex . . . 64
8G5: Stratified by Child birth order . . . 65
8G6: Stratified by Child baseline acute malnutrition (insufficient comparisons) . . . 66
8G7: Stratified by Child baseline anemia (insufficient comparisons) . . . 67
8G8: Stratified by Child high-dose vitamin A supplementation . . . 68
8G9: Stratified by Child inflammation . . . 69

Supplemental figure 8H: Iron deficiency (ferritin $<$ 12 µg/L) prevalence difference **70**
8H1: Stratified by Maternal BMI . . . 70
8H2: Stratified by Maternal age . . . 71
8H3: Stratified by Maternal education . . . 72
8H4: Stratified by Child sex . . . 73
8H5: Stratified by Child birth order . . . 74
8H6: Stratified by Child baseline acute malnutrition (insufficient comparisons) . . . 75
8H7: Stratified by Child baseline anemia (insufficient comparisons) . . . 76
8H8: Stratified by Child high-dose vitamin A supplementation . . . 77
8H9: Stratified by Child inflammation . . . 78

Supplemental figure 8I: Iron deficiency anemia prevalence ratio **79**
8I1: Stratified by Maternal BMI . . . 79
8I2: Stratified by Maternal age (insufficient comparisons) . . . 80
8I3: Stratified by Maternal education (insufficient comparisons) . . . 81

8I4: Stratified by Child sex . . . 82
8I5: Stratified by Child birth order . . . 83
8I6: Stratified by Child baseline acute malnutrition (insufficient comparisons) . . . 84
8I7: Stratified by Child baseline anemia (insufficient comparisons) . . . 85
8I8: Stratified by Child high-dose vitamin A supplementation (insufficient comparisons) . . . 86
8I9: Stratified by Child inflammation . . . 87

Supplemental figure 8J: Iron deficiency anemia prevalence difference **88**
8J1: Stratified by Maternal BMI . . . 88
8J2: Stratified by Maternal age (insufficient comparisons) . . . 89
8J3: Stratified by Maternal education (insufficient comparisons) . . . 90
8J4: Stratified by Child sex . . . 91
8J5: Stratified by Child birth order . . . 92
8J6: Stratified by Child baseline acute malnutrition (insufficient comparisons) . . . 93
8J7: Stratified by Child baseline anemia (insufficient comparisons) . . . 94
8J8: Stratified by Child high-dose vitamin A supplementation (insufficient comparisons) . . . 95
8J9: Stratified by Child inflammation . . . 96

Supplemental figure 8K: Geometric mean ratio of soluble transferrin receptor concentration **97**
8K1: Stratified by Maternal BMI . . . 97
8K2: Stratified by Maternal age . . . 98
8K3: Stratified by Maternal education . . . 99
8K4: Stratified by Child sex . . . 100
8K5: Stratified by Child birth order . . . 101
8K6: Stratified by Child baseline acute malnutrition (insufficient comparisons) . . . 102
8K7: Stratified by Child baseline anemia (insufficient comparisons) . . . 103
8K8: Stratified by Child high-dose vitamin A supplementation . . . 104
8K9: Stratified by Child inflammation . . . 105

Supplemental figure 8L: Elevated soluble transferrin receptor prevalence ratio **106**
8L1: Stratified by Maternal BMI . . . 106
8L2: Stratified by Maternal age . . . 107
8L3: Stratified by Maternal education . . . 108
8L4: Stratified by Child sex . . . 109
8L5: Stratified by Child birth order . . . 110
8L6: Stratified by Child baseline acute malnutrition (insufficient comparisons) . . . 111
8L7: Stratified by Child baseline anemia (insufficient comparisons) . . . 112
8L8: Stratified by Child high-dose vitamin A supplementation . . . 113
8L9: Stratified by Child inflammation . . . 114

Supplemental figure 8M: Elevated soluble transferrin receptor prevalence difference **115**
8M1: Stratified by Maternal BMI . . . 115
8M2: Stratified by Maternal age . . . 116
8M3: Stratified by Maternal education . . . 117
8M4: Stratified by Child sex . . . 118
8M5: Stratified by Child birth order . . . 119
8M6: Stratified by Child baseline acute malnutrition (insufficient comparisons) . . . 120
8M7: Stratified by Child baseline anemia (insufficient comparisons) . . . 121
8M8: Stratified by Child high-dose vitamin A supplementation . . . 122
8M9: Stratified by Child inflammation . . . 123

Supplemental figure 8N: Geometric mean ratio of zinc protoporphyrin concentration **124**
8N1: Stratified by Maternal BMI . . . 124
8N2: Stratified by Maternal age . . . 125
8N3: Stratified by Maternal education . . . 126
8N4: Stratified by Child sex . . . 127
8N5: Stratified by Child birth order . . . 128
8N6: Stratified by Child baseline acute malnutrition . . . 129
8N7: Stratified by Child baseline anemia . . . 130
8N8: Stratified by Child high-dose vitamin A supplementation (insufficient comparisons) . . . 131
8N9: Stratified by Child inflammation . . . 132

Supplemental figure 8O: Elevated zinc protoporphyrin prevalence ratio **133**
8O1: Stratified by Maternal BMI . . . 133
8O2: Stratified by Maternal age . . . 134
8O3: Stratified by Maternal education . . . 135
8O4: Stratified by Child sex . . . 136
8O5: Stratified by Child birth order . . . 137
8O6: Stratified by Child baseline acute malnutrition (insufficient comparisons) . . . 138
8O7: Stratified by Child baseline anemia . . . 139
8O8: Stratified by Child high-dose vitamin A supplementation (insufficient comparisons) . . . 140
8O9: Stratified by Child inflammation . . . 141

Supplemental figure 8P: Elevated zinc protoporphyrin prevalence difference **142**
8P1: Stratified by Maternal BMI . . . 142
8P2: Stratified by Maternal age . . . 143
8P3: Stratified by Maternal education . . . 144
8P4: Stratified by Child sex . . . 145
8P5: Stratified by Child birth order . . . 146
8P6: Stratified by Child baseline acute malnutrition (insufficient comparisons) . . . 147
8P7: Stratified by Child baseline anemia . . . 148
8P8: Stratified by Child high-dose vitamin A supplementation (insufficient comparisons) . . . 149
8P9: Stratified by Child inflammation . . . 150

Supplemental figure 8Q: Geometric mean ratio of plasma zinc concentration **151**
8Q1: Stratified by Maternal BMI (insufficient comparisons) . . . 151
8Q2: Stratified by Maternal age . . . 152
8Q3: Stratified by Maternal education (insufficient comparisons) . . . 153
8Q4: Stratified by Child sex . . . 154
8Q5: Stratified by Child birth order . . . 155
8Q6: Stratified by Child baseline acute malnutrition (insufficient comparisons) . . . 156
8Q7: Stratified by Child baseline anemia (insufficient comparisons) . . . 157
8Q8: Stratified by Child high-dose vitamin A supplementation (insufficient comparisons) . . . 158
8Q9: Stratified by Child inflammation . . . 159

Supplemental figure 8R: Geometric mean ratio of retinol concentration **160**
8R1: Stratified by Maternal BMI (insufficient comparisons) . . . 160
8R2: Stratified by Maternal age . . . 161
8R3: Stratified by Maternal education . . . 162
8R4: Stratified by Child sex . . . 163
8R5: Stratified by Child birth order . . . 164
8R6: Stratified by Child baseline acute malnutrition . . . 165

8R7: Stratified by Child baseline anemia (insufficient comparisons) . . . 166
8R8: Stratified by Child high-dose vitamin A supplementation . . . 167
8R9: Stratified by Child inflammation . . . 168

Supplemental figure 8S: Low vitamin A (retinol < 0.70 µmol/L) prevalence ratio **169**
8S1: Stratified by Maternal BMI (insufficient comparisons) . . . 169
8S2: Stratified by Maternal age (insufficient comparisons) . . . 170
8S3: Stratified by Maternal education (insufficient comparisons) . . . 171
8S4: Stratified by Child sex (insufficient comparisons) . . . 172
8S5: Stratified by Child birth order (insufficient comparisons) . . . 173
8S6: Stratified by Child baseline acute malnutrition (insufficient comparisons) . . . 174
8S7: Stratified by Child baseline anemia (insufficient comparisons) . . . 175
8S8: Stratified by Child high-dose vitamin A supplementation (insufficient comparisons) . . . 176
8S9: Stratified by Child inflammation (insufficient comparisons) . . . 177

Supplemental figure 8T: Low vitamin A (retinol < 0.70 µmol/L) prevalence difference **178**
8T1: Stratified by Maternal BMI (insufficient comparisons) . . . 178
8T2: Stratified by Maternal age (insufficient comparisons) . . . 179
8T3: Stratified by Maternal education (insufficient comparisons) . . . 180
8T4: Stratified by Child sex (insufficient comparisons) . . . 181
8T5: Stratified by Child birth order (insufficient comparisons) . . . 182
8T6: Stratified by Child baseline acute malnutrition (insufficient comparisons) . . . 183
8T7: Stratified by Child baseline anemia (insufficient comparisons) . . . 184
8T8: Stratified by Child high-dose vitamin A supplementation (insufficient comparisons) . . . 185
8T9: Stratified by Child inflammation (insufficient comparisons) . . . 186

Supplemental figure 8U: Marginal vitamin A (retinol < 1.05 µmol/L) prevalence ratio **187**
8U1: Stratified by Maternal BMI (insufficient comparisons) . . . 187
8U2: Stratified by Maternal age . . . 188
8U3: Stratified by Maternal education (insufficient comparisons) . . . 189
8U4: Stratified by Child sex . . . 190
8U5: Stratified by Child birth order . . . 191
8U6: Stratified by Child baseline acute malnutrition (insufficient comparisons) . . . 192
8U7: Stratified by Child baseline anemia (insufficient comparisons) . . . 193
8U8: Stratified by Child high-dose vitamin A supplementation . . . 194
8U9: Stratified by Child inflammation . . . 195

Supplemental figure 8V: Marginal vitamin A (retinol < 1.05 µmol/L) prevalence difference **196**
8V1: Stratified by Maternal BMI (insufficient comparisons) . . . 196
8V2: Stratified by Maternal age . . . 197
8V3: Stratified by Maternal education (insufficient comparisons) . . . 198
8V4: Stratified by Child sex . . . 199
8V5: Stratified by Child birth order . . . 200
8V6: Stratified by Child baseline acute malnutrition (insufficient comparisons) . . . 201
8V7: Stratified by Child baseline anemia (insufficient comparisons) . . . 202
8V8: Stratified by Child high-dose vitamin A supplementation . . . 203
8V9: Stratified by Child inflammation . . . 204

Supplemental figure 8W: Geometric mean ratio of retinol binding protein concentration **205**
8W1: Stratified by Maternal BMI . . . 205
8W2: Stratified by Maternal age . . . 206

8W3: Stratified by Maternal education . . . 207
8W4: Stratified by Child sex . . . 208
8W5: Stratified by Child birth order . . . 209
8W6: Stratified by Child baseline acute malnutrition (insufficient comparisons) . . . 210
8W7: Stratified by Child baseline anemia (insufficient comparisons) . . . 211
8W8: Stratified by Child high-dose vitamin A supplementation . . . 212
8W9: Stratified by Child inflammation . . . 213

Supplemental figure 8X: Low vitamin A status (RBP < 0.70 µmol/L) prevalence ratio 214
8X1: Stratified by Maternal BMI (insufficient comparisons) . . . 214
8X2: Stratified by Maternal age (insufficient comparisons) . . . 215
8X3: Stratified by Maternal education (insufficient comparisons) . . . 216
8X4: Stratified by Child sex (insufficient comparisons) . . . 217
8X5: Stratified by Child birth order (insufficient comparisons) . . . 218
8X6: Stratified by Child baseline acute malnutrition (insufficient comparisons) . . . 219
8X7: Stratified by Child baseline anemia (insufficient comparisons) . . . 220
8X8: Stratified by Child high-dose vitamin A supplementation (insufficient comparisons) . . . 221
8X9: Stratified by Child inflammation (insufficient comparisons) . . . 222

Supplemental figure 8Y: Low vitamin A status (RBP < 0.70 µmol/L) prevalence difference 223
8Y1: Stratified by Maternal BMI (insufficient comparisons) . . . 223
8Y2: Stratified by Maternal age (insufficient comparisons) . . . 224
8Y3: Stratified by Maternal education (insufficient comparisons) . . . 225
8Y4: Stratified by Child sex (insufficient comparisons) . . . 226
8Y5: Stratified by Child birth order (insufficient comparisons) . . . 227
8Y6: Stratified by Child baseline acute malnutrition (insufficient comparisons) . . . 228
8Y7: Stratified by Child baseline anemia (insufficient comparisons) . . . 229
8Y8: Stratified by Child high-dose vitamin A supplementation (insufficient comparisons) . . . 230
8Y9: Stratified by Child inflammation (insufficient comparisons) . . . 231

Supplemental figure 8Z: Marginal vitamin A status (RBP < 1.05 µmol/L) prevalence ratio 232
8Z1: Stratified by Maternal BMI . . . 232
8Z2: Stratified by Maternal age . . . 233
8Z3: Stratified by Maternal education . . . 234
8Z4: Stratified by Child sex . . . 235
8Z5: Stratified by Child birth order . . . 236
8Z6: Stratified by Child baseline acute malnutrition (insufficient comparisons) . . . 237
8Z7: Stratified by Child baseline anemia (insufficient comparisons) . . . 238
8Z8: Stratified by Child high-dose vitamin A supplementation (insufficient comparisons) . . . 239
8Z9: Stratified by Child inflammation . . . 240

Supplemental figure 8AA: Marginal vitamin A status (RBP < 1.05 µmol/L) prevalence difference 241
8AA1: Stratified by Maternal BMI . . . 241
8AA2: Stratified by Maternal age . . . 242
8AA3: Stratified by Maternal education . . . 243
8AA4: Stratified by Child sex . . . 244
8AA5: Stratified by Child birth order . . . 245
8AA6: Stratified by Child baseline acute malnutrition (insufficient comparisons) . . . 246
8AA7: Stratified by Child baseline anemia (insufficient comparisons) . . . 247
8AA8: Stratified by Child high-dose vitamin A supplementation (insufficient comparisons) . . . 248
8AA9: Stratified by Child inflammation . . . 249

Supplemental figure 8: Forest plots for effects of SQ-LNS on biochemical outcomes stratified by individual-level maternal and child effect modifiers

These figures are forest plots showing the individual-level effect modification of intervention effects. Each figure has the estimates of intervention effect stratified within study by individual-level effect modifier category. For definitions of effect modifiers, see Box 1 in the main paper. Individual study estimates were generated from log-binomial regression for dichotomous outcomes and simple linear regression for continuous outcomes; controlling for baseline measure when available and with clustered observations using robust standard errors for cluster-randomized trials. Pooled interaction term and sub-group estimates were generated using inverse-variance weighting fixed and random effects. For continuous outcomes the intervention effect is measured by the difference in mean of the LNS group minus control. For log transformed continuous outcomes, the intervention effect is measured by the ratio of geometric means, the effect estimate is the geometric mean in the LNS group divided by the geometric mean in the control group. For dichotomous outcomes analyzed via prevalence ratios, the effect estimate is the prevalence in the LNS group divided by the prevalence in the control group. For dichotomous outcomes analyzed via prevalence differences, the effect estimate is the prevalence in the LNS group minus the prevalence in the control group.

The labels on the far left correspond to trial level information. In the middle left and on the right the values indicate the study level effect estimate, confidence interval, and weighting for deriving the pooled estimates is shown by subgroup.

Not all trials were included in all individual-level effect modification analyses, either because they did not measure the biomarker outcome or the effect modifier of interest (e.g., baseline anemia or acute malnutrition, receipt of high-dose vitamin A supplement), or because the prevalence of the binary outcome or proportion of children within one of the effect modifier subgroups was too low to allow us to generate effect estimates. If fewer than three studies contribute to a pooled estimate then the pooled estimate was not generated (e.g. if fewer than 3 studies are categorized into a study level effect modification category), and this is labeled as “insufficient comparisons”.

Ferritin, sTfR, ZPP, zinc, retinol and RBP concentrations were adjusted for inflammation (i.e., C-reactive protein (CRP) and/or α-1-acid glycoprotein (AGP) concentrations, as available), using a regression correction approach adapted from the Biomarkers Reflecting Inflammation and Nutritional Determinants of Anemia (BRINDA) project (28)

RBP, retinol binding protein.

Supplemental figure 8A: Mean difference in hemoglobin concentration

8A1: Stratified by Maternal BMI

P−for−interaction = 0.320

Difference in MDs = −0.49 (−1.46, 0.48)

		At least 20 kg/m²						Less than 20 kg/m²					
Country	Trial	LNS N	Control N	Control Mean	MD (95% CI)	Fixed W	Random W	LNS N	Control N	Control Mean	MD (95% CI)	Fixed W	Random W
Bangladesh	JiVitA−4 (35)												
Bangladesh	RDNS (36)	227	102	111.1	5.00 (1.85, 8.15)	0.03	0.08	300	160	113.1	3.69 (0.90, 6.49)	0.11	0.11
Bangladesh	WASH−B (37)	102	102	118.0	4.54 (2.27, 6.81)	0.07	0.10	132	84	118.3	1.91 (−0.70, 4.51)	0.13	0.11
Burkina Faso	iLiNS−Zinc (38)	1217	390	88.5	8.29 (5.51, 11.07)	0.04	0.09	738	274	88.7	8.73 (5.13, 12.33)	0.07	0.09
Burkina Faso	PROMIS CS (39)	335	349	102.4	1.13 (−0.92, 3.19)	0.08	0.10	239	227	102.7	2.79 (−0.12, 5.69)	0.11	0.10
Ghana	GHANA (40)												
Ghana	iLiNS−DYAD−G (41)	288	536	112.4	0.99 (−0.49, 2.48)	0.15	0.11	35	114	110.2	2.33 (−1.69, 6.35)	0.05	0.08
Kenya	WASH−B (42)	266	227	109.3	4.70 (2.53, 6.88)	0.07	0.10	72	60	113.0	0.17 (−3.87, 4.21)	0.05	0.08
Madagascar	MAHAY (43)												
Malawi	iLiNS−DYAD−M (44)	124	258	107.8	1.99 (−1.35, 5.33)	0.03	0.07	85	171	108.0	0.42 (−3.59, 4.42)	0.06	0.08
Malawi	iLiNS−DOSE (45)	179	52	100.3	6.04 (1.31, 10.78)	0.02	0.05	61	29	100.6	−0.01 (−6.19, 6.16)	0.02	0.05
Mali	PROMIS CS (46)	702	685	95.9	6.19 (3.84, 8.54)	0.06	0.09	246	279	95.4	6.43 (4.14, 8.73)	0.17	0.12
Zimbabwe	SHINE (HIV−) (47)	1222	1161	114.3	2.26 (1.34, 3.18)	0.40	0.12	213	220	114.8	1.10 (−1.04, 3.23)	0.20	0.12
Zimbabwe	SHINE (HIV+) (48)	221	222	114.7	2.78 (−0.19, 5.76)	0.04	0.08	58	40	114.4	3.48 (−1.98, 8.94)	0.03	0.06
		**4883**	**4084**		**I² = 0.76, Tau² = 3.84**			**2179**	**1658**		**I² = 0.63, Tau² = 4.11**		
**Fixed**					**2.97 (2.39, 3.55)**						**3.13 (2.18, 4.07)**		
**Random**					**3.79 (2.40, 5.17)**						**3.01 (1.42, 4.61)**		

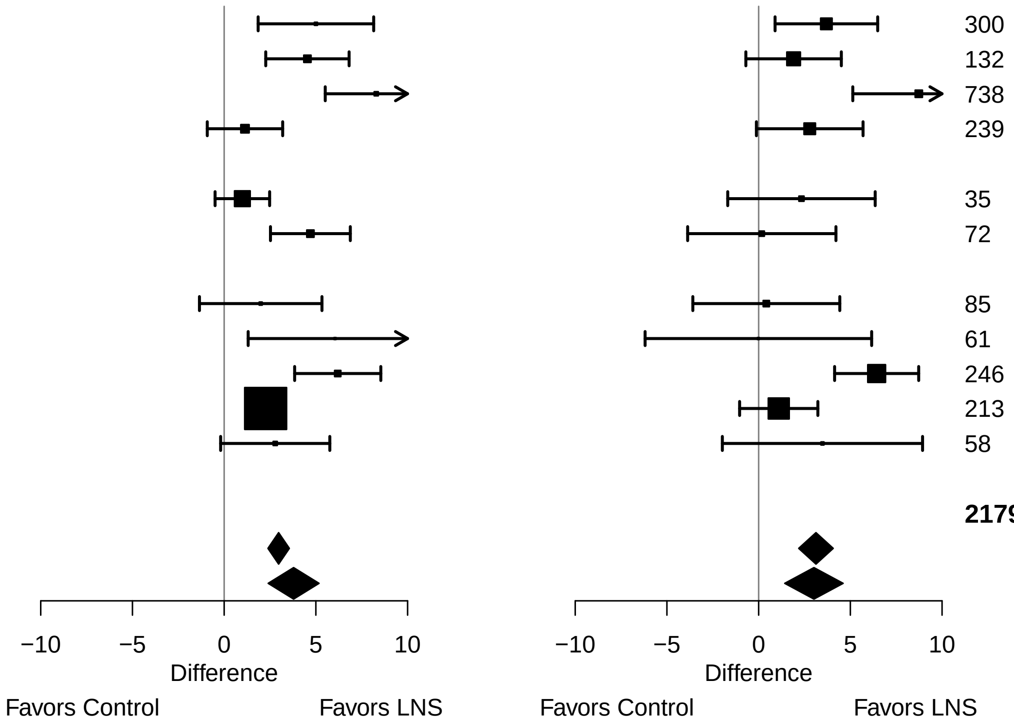

Supplemental figure 8A: Mean difference in hemoglobin concentration

8A2: Stratified by Maternal age

P−for−interaction = 0.015
Difference in MDs = −1.03 (−1.86, −0.20)

		At least 25 y						Less than 25 y					
Country	Trial	LNS N	Control N	Control Mean	MD (95% CI)	Fixed W	Random W	LNS N	Control N	Control Mean	MD (95% CI)	Fixed W	Random W
Bangladesh	JiVitA−4 (35)	203	72	117.4	2.75 (0.13, 5.37)	0.06	0.08	251	73	118.5	1.59 (−1.10, 4.28)	0.07	0.08
Bangladesh	RDNS (36)	152	81	113.2	4.42 (0.28, 8.55)	0.02	0.05	397	191	112.1	4.01 (1.98, 6.05)	0.11	0.10
Bangladesh	WASH−B (37)	89	83	117.9	3.48 (1.50, 5.46)	0.10	0.09	144	103	118.3	2.90 (0.33, 5.46)	0.07	0.08
Burkina Faso	iLiNS−Zinc (38)	1132	399	88.4	9.30 (5.99, 12.60)	0.03	0.07	812	262	88.9	7.22 (3.66, 10.78)	0.04	0.06
Burkina Faso	PROMIS CS (39)	271	263	103.5	2.24 (−0.19, 4.67)	0.06	0.08	303	318	101.9	1.28 (−1.17, 3.74)	0.08	0.09
Ghana	GHANA (40)	69	63	106.8	9.03 (4.63, 13.44)	0.02	0.05	29	25	101.0	10.43 (2.08, 18.78)	0.01	0.01
Ghana	iLiNS−DYAD−G (41)	206	397	112.7	1.46 (−0.23, 3.16)	0.13	0.09	122	264	110.8	0.71 (−1.61, 3.03)	0.09	0.09
Kenya	WASH−B (42)	213	168	110.7	3.54 (1.20, 5.87)	0.07	0.08	133	131	108.7	4.21 (1.02, 7.39)	0.05	0.07
Madagascar	MAHAY (43)	301	329	104.6	2.38 (−1.14, 5.90)	0.03	0.06	299	259	103.2	3.34 (0.16, 6.52)	0.05	0.07
Malawi	iLiNS−DYAD−M (44)	99	220	109.5	0.07 (−3.51, 3.64)	0.03	0.06	111	212	106.6	2.27 (−1.40, 5.94)	0.04	0.06
Malawi	iLiNS−DOSE (45)	135	50	100.8	3.58 (−1.36, 8.52)	0.02	0.04	104	28	100.1	4.62 (−1.55, 10.78)	0.01	0.02
Mali	PROMIS CS (46)	502	508	95.0	7.55 (5.33, 9.78)	0.08	0.08	451	462	96.5	4.81 (2.39, 7.22)	0.08	0.09
Zimbabwe	SHINE (HIV−) (47)	808	726	114.8	2.60 (1.50, 3.70)	0.31	0.10	697	694	113.7	1.45 (0.18, 2.73)	0.29	0.13
Zimbabwe	SHINE (HIV+) (48)	221	204	115.0	3.33 (0.25, 6.41)	0.04	0.07	66	60	114.2	1.29 (−3.02, 5.60)	0.03	0.04
		**4401**	**3563**		**I² = 0.70, Tau² = 4.76**			**3919**	**3082**		**I² = 0.46, Tau² = 1.75**		
**Fixed**					**3.33 (2.71, 3.94)**						**2.61 (1.92, 3.29)**		
**Random**					**3.83 (2.44, 5.21)**						**2.95 (1.89, 4.01)**		

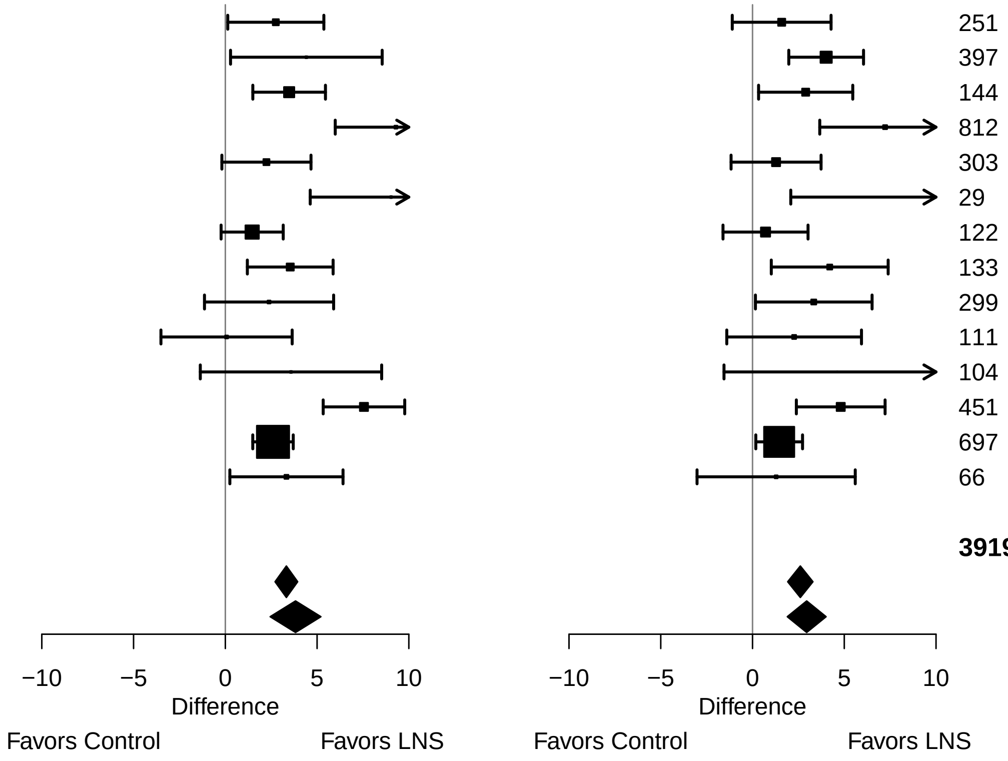

Supplemental figure 8A: Mean difference in hemoglobin concentration

8A3: Stratified by Maternal education

P−for−interaction = 0.212
Difference in MDs = −0.80 (−2.05, 0.45)

Primary or greater

Country	Trial	LNS N	Control N	Control Mean	MD (95% CI)	Fixed W	Random W
Bangladesh	JiVitA−4 (35)	299	93	117.4	3.22 (0.81, 5.62)	0.06	0.09
Bangladesh	RDNS (36)	405	195	112.8	4.17 (1.54, 6.81)	0.05	0.09
Bangladesh	WASH−B (37)	163	145	118.5	2.92 (0.80, 5.03)	0.08	0.10
Burkina Faso	iLiNS−Zinc (38)	82	16	86.0	11.27 (3.53, 19.01)	0.01	0.03
Burkina Faso	PROMIS CS (39)	46	47	103.1	0.39 (−4.37, 5.16)	0.02	0.05
Ghana	GHANA (40)	91	75	105.8	8.93 (4.80, 13.06)	0.02	0.06
Ghana	iLiNS−DYAD−G (41)	253	526	111.7	1.38 (−0.16, 2.92)	0.14	0.11
Kenya	WASH−B (42)	168	136	108.4	7.46 (4.92, 10.01)	0.05	0.09
Madagascar	MAHAY (43)	128	143	103.5	3.17 (−1.42, 7.75)	0.02	0.06
Malawi	iLiNS−DYAD−M (44)	34	66	109.6	4.51 (−1.73, 10.75)	0.01	0.04
Malawi	iLiNS−DOSE (45)	49	17	97.4	11.72 (2.32, 21.13)	0.00	0.02
Mali	PROMIS CS (46)	107	96	97.5	4.27 (0.60, 7.94)	0.03	0.07
Zimbabwe	SHINE (HIV−) (47)	1536	1462	114.4	1.88 (1.04, 2.73)	0.48	0.11
Zimbabwe	SHINE (HIV+) (48)	274	252	114.8	2.81 (0.13, 5.49)	0.05	0.09
		**3635**	**3269**		**I² = 0.68, Tau² = 4.95**		
**Fixed**					**2.73 (2.15, 3.32)**		
**Random**					**4.00 (2.50, 5.50)**		

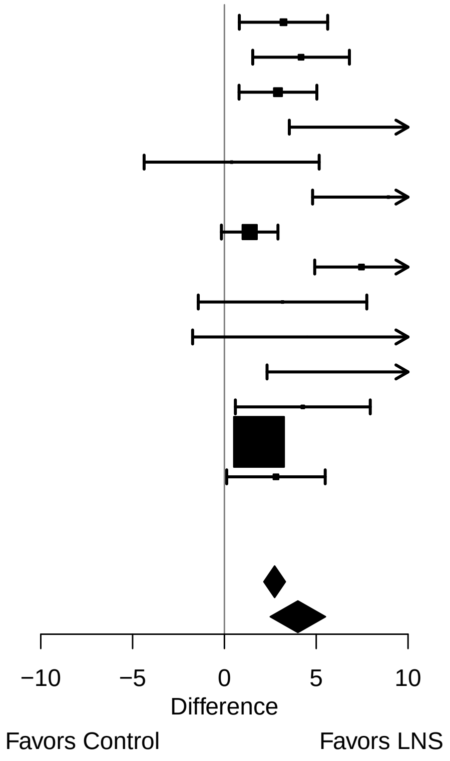

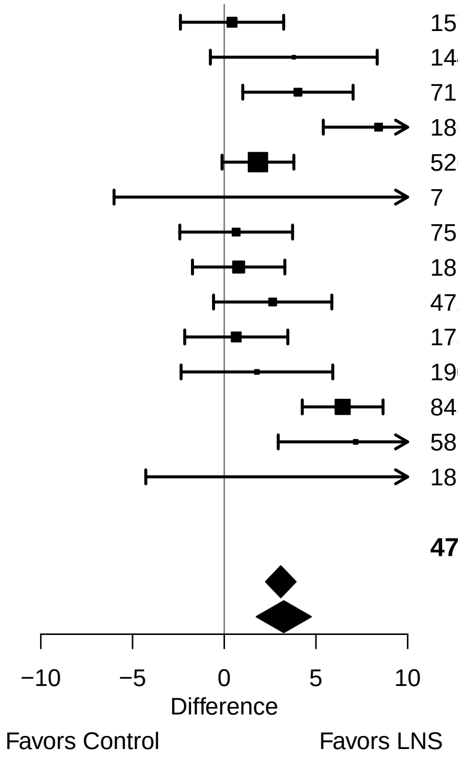

Incomplete or no formal

Country	Trial	LNS N	Control N	Control Mean	MD (95% CI)	Fixed W	Random W
Bangladesh	JiVitA−4 (35)	157	53	118.9	0.43 (−2.39, 3.24)	0.09	0.09
Bangladesh	RDNS (36)	144	77	111.5	3.79 (−0.76, 8.34)	0.03	0.06
Bangladesh	WASH−B (37)	71	41	116.7	4.02 (1.01, 7.03)	0.07	0.08
Burkina Faso	iLiNS−Zinc (38)	1862	645	88.6	8.41 (5.40, 11.43)	0.07	0.08
Burkina Faso	PROMIS CS (39)	526	532	102.6	1.84 (−0.12, 3.80)	0.18	0.10
Ghana	GHANA (40)	7	15	102.0	9.57 (−6.01, 25.15)	0.00	0.01
Ghana	iLiNS−DYAD−G (41)	75	135	112.8	0.65 (−2.42, 3.72)	0.07	0.08
Kenya	WASH−B (42)	181	164	110.9	0.79 (−1.73, 3.30)	0.11	0.09
Madagascar	MAHAY (43)	472	445	104.2	2.64 (−0.58, 5.87)	0.07	0.08
Malawi	iLiNS−DYAD−M (44)	175	363	107.6	0.65 (−2.16, 3.47)	0.09	0.09
Malawi	iLiNS−DOSE (45)	190	61	101.4	1.78 (−2.35, 5.92)	0.04	0.06
Mali	PROMIS CS (46)	845	874	95.6	6.46 (4.25, 8.66)	0.14	0.10
Zimbabwe	SHINE (HIV−) (47)	58	53	112.3	7.17 (2.94, 11.39)	0.04	0.06
Zimbabwe	SHINE (HIV+) (48)	18	14	112.6	6.65 (−4.28, 17.58)	0.01	0.02
		**4781**	**3472**		**I² = 0.68, Tau² = 4.63**		
**Fixed**					**3.08 (2.26, 3.91)**		
**Random**					**3.25 (1.76, 4.74)**		

Supplemental figure 8A: Mean difference in hemoglobin concentration

8A4: Stratified by Child sex

P−for−interaction = 0.449
Difference in MDs = −0.30 (−1.08, 0.48)

Male

Country	Trial	LNS N	Control N	Control Mean	MD (95% CI)	Fixed W	Random W
Bangladesh	JiVitA−4 (35)	235	71	116.9	4.05 (1.55, 6.55)	0.07	0.08
Bangladesh	RDNS (36)	266	131	111.0	5.50 (2.06, 8.93)	0.04	0.07
Bangladesh	WASH−B (37)	116	89	118.2	3.70 (1.12, 6.27)	0.06	0.08
Burkina Faso	iLiNS−Zinc (38)	993	334	87.3	9.05 (6.04, 12.05)	0.05	0.07
Burkina Faso	PROMIS CS (39)	317	295	101.1	1.63 (−0.56, 3.82)	0.09	0.08
Ghana	GHANA (40)	60	41	104.5	11.05 (5.41, 16.68)	0.01	0.04
Ghana	iLiNS−DYAD−G (41)	164	310	111.7	0.33 (−1.67, 2.32)	0.10	0.09
Kenya	WASH−B (42)	168	145	110.3	3.13 (0.34, 5.92)	0.05	0.07
Madagascar	MAHAY (43)	277	288	102.4	3.37 (−0.23, 6.96)	0.03	0.06
Malawi	iLiNS−DYAD−M (44)	103	202	106.6	0.87 (−2.84, 4.59)	0.03	0.06
Malawi	iLiNS−DOSE (45)	123	43	98.0	4.62 (−0.50, 9.74)	0.02	0.05
Mali	PROMIS CS (46)	479	526	94.6	6.32 (4.06, 8.58)	0.08	0.08
Zimbabwe	SHINE (HIV−) (47)	830	814	113.9	1.77 (0.65, 2.89)	0.33	0.10
Zimbabwe	SHINE (HIV+) (48)	146	149	112.8	3.83 (0.70, 6.97)	0.04	0.07
		**4277**	**3438**		**I² = 0.74, Tau² = 5.44**		
**Fixed**					**3.07 (2.42, 3.71)**		
**Random**					**3.91 (2.44, 5.37)**		

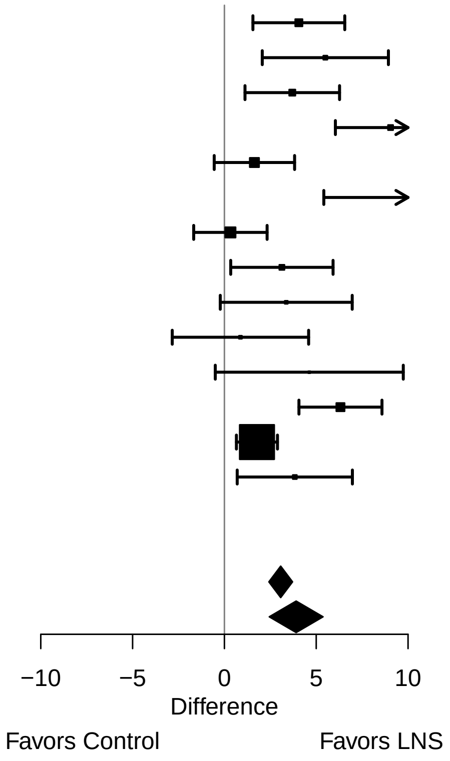

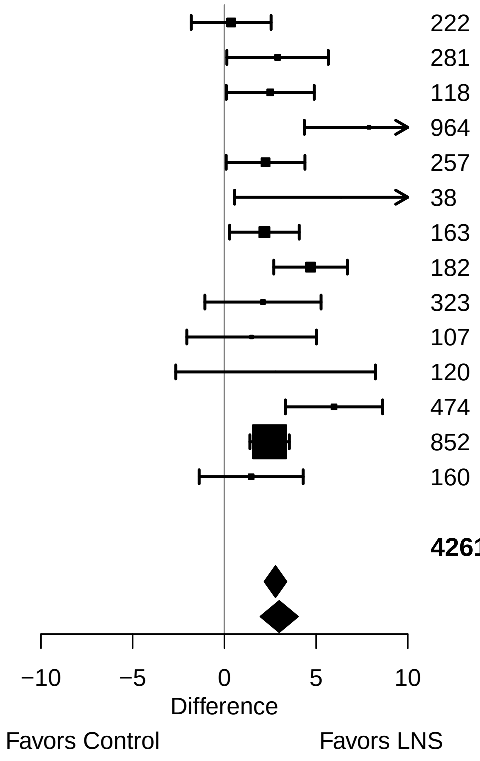

Female

Country	Trial	LNS N	Control N	Control Mean	MD (95% CI)	Fixed W	Random W
Bangladesh	JiVitA−4 (35)	222	75	119.0	0.37 (−1.81, 2.55)	0.08	0.09
Bangladesh	RDNS (36)	281	141	113.7	2.90 (0.13, 5.66)	0.05	0.07
Bangladesh	WASH−B (37)	118	97	118.1	2.50 (0.10, 4.90)	0.06	0.08
Burkina Faso	iLiNS−Zinc (38)	964	330	89.8	7.89 (4.36, 11.41)	0.03	0.05
Burkina Faso	PROMIS CS (39)	257	286	104.2	2.24 (0.10, 4.39)	0.08	0.09
Ghana	GHANA (40)	38	55	106.6	6.36 (0.56, 12.17)	0.01	0.03
Ghana	iLiNS−DYAD−G (41)	163	348	112.2	2.18 (0.29, 4.08)	0.10	0.10
Kenya	WASH−B (42)	182	155	109.4	4.70 (2.69, 6.70)	0.09	0.09
Madagascar	MAHAY (43)	323	300	105.5	2.10 (−1.06, 5.27)	0.04	0.06
Malawi	iLiNS−DYAD−M (44)	107	230	109.3	1.48 (−2.05, 5.01)	0.03	0.05
Malawi	iLiNS−DOSE (45)	120	39	103.6	2.79 (−2.65, 8.23)	0.01	0.03
Mali	PROMIS CS (46)	474	444	97.1	5.98 (3.33, 8.63)	0.05	0.07
Zimbabwe	SHINE (HIV−) (47)	852	780	114.6	2.46 (1.39, 3.53)	0.32	0.12
Zimbabwe	SHINE (HIV+) (48)	160	136	116.9	1.46 (−1.38, 4.29)	0.05	0.07
		**4261**	**3416**		**I² = 0.50, Tau² = 1.94**		
**Fixed**					**2.79 (2.18, 3.39)**		
**Random**					**2.99 (1.96, 4.02)**		

Supplemental figure 8A: Mean difference in hemoglobin concentration

8A5: Stratified by Child birth order

P−for−interaction = 0.003
Difference in MDs = −1.46 (−2.42, −0.50)

Later born

Country	Trial	LNS N	Control N	Control Mean	MD (95% CI)	Fixed W	Random W
Bangladesh	JiVitA−4 (35)	115	33	115.1	4.41 (0.47, 8.34)	0.02	0.05
Bangladesh	RDNS (36)	339	171	112.6	3.88 (1.15, 6.62)	0.04	0.07
Bangladesh	WASH−B (37)	137	123	117.7	3.01 (1.07, 4.94)	0.08	0.09
Burkina Faso	iLiNS−Zinc (38)	1522	520	88.7	8.30 (4.85, 11.75)	0.02	0.05
Burkina Faso	PROMIS CS (39)	498	477	102.4	2.26 (0.28, 4.25)	0.07	0.09
Ghana	GHANA (40)	60	54	106.3	9.73 (4.70, 14.77)	0.01	0.03
Ghana	iLiNS−DYAD−G (41)	220	435	111.9	2.35 (0.69, 4.01)	0.11	0.10
Kenya	WASH−B (42)	282	244	110.1	3.73 (1.59, 5.87)	0.06	0.08
Madagascar	MAHAY (43)	458	454	103.7	2.84 (−0.35, 6.02)	0.03	0.06
Malawi	iLiNS−DYAD−M (44)	165	342	108.5	1.61 (−1.21, 4.44)	0.04	0.07
Malawi	iLiNS−DOSE (45)	148	61	99.7	3.54 (−0.95, 8.02)	0.01	0.04
Mali	PROMIS CS (46)	790	832	95.7	6.39 (4.23, 8.56)	0.06	0.08
Zimbabwe	SHINE (HIV−) (47)	1188	1100	114.2	2.70 (1.82, 3.57)	0.39	0.12
Zimbabwe	SHINE (HIV+) (48)	252	216	114.7	3.15 (0.63, 5.67)	0.05	0.07
		**6174**	**5062**		**I² = 0.54, Tau² = 2.36**		
**Fixed**					**3.25 (2.71, 3.79)**		
**Random**					**3.75 (2.68, 4.82)**		

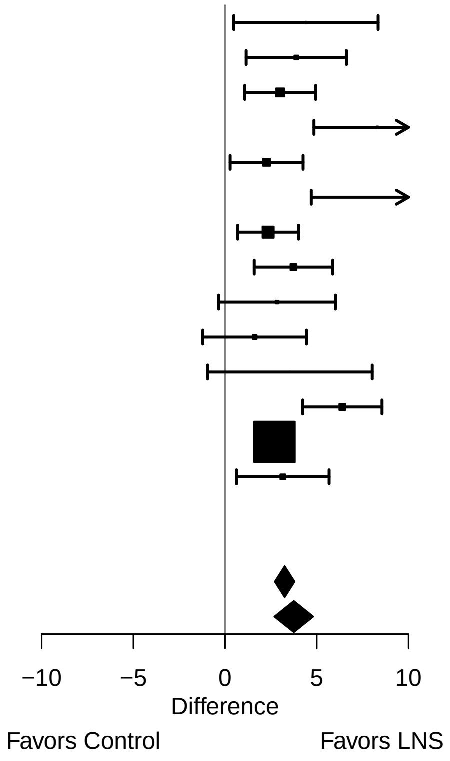

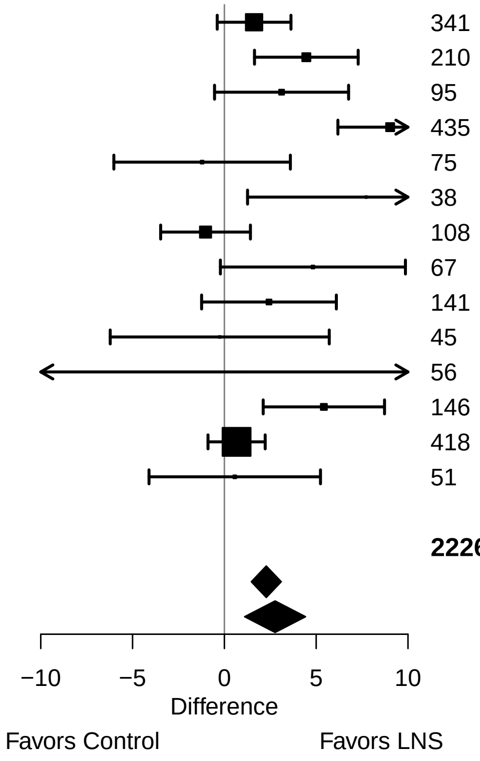

Firstborn

LNS N	Control N	Control Mean	MD (95% CI)	Fixed W	Random W
341	112	118.8	1.62 (−0.39, 3.63)	0.16	0.10
210	101	112.1	4.46 (1.64, 7.29)	0.08	0.09
95	61	118.7	3.11 (−0.54, 6.77)	0.05	0.08
435	144	88.0	9.03 (6.18, 11.87)	0.08	0.09
75	103	103.6	−1.21 (−6.02, 3.59)	0.03	0.06
38	38	104.4	7.72 (1.26, 14.19)	0.02	0.04
108	226	112.1	−1.03 (−3.47, 1.42)	0.11	0.09
67	56	108.4	4.82 (−0.22, 9.86)	0.03	0.06
141	131	105.0	2.43 (−1.24, 6.10)	0.05	0.08
45	88	105.9	−0.25 (−6.22, 5.71)	0.02	0.05
56	10	108.2	0.99 (−10.07, 12.04)	0.01	0.02
146	126	95.7	5.42 (2.11, 8.72)	0.06	0.08
418	416	114.4	0.67 (−0.89, 2.22)	0.27	0.11
51	64	115.1	0.56 (−4.11, 5.24)	0.03	0.06
**2226**	**1676**		**I² = 0.72, Tau² = 5.98**		
			**2.28 (1.47, 3.10)**		
			**2.76 (1.10, 4.42)**		

Supplemental figure 8A: Mean difference in hemoglobin concentration

8A6: Stratified by Child baseline acute malnutrition

P−for−interaction = 0.859
Difference in MDs = 0.15 (−1.54, 1.84)

Country	Trial	No: LNS N	No: Control N	No: Control Mean	No: MD (95% CI)	No: Fixed W	No: Random W	Yes: LNS N	Yes: Control N	Yes: Control Mean	Yes: MD (95% CI)	Yes: Fixed W	Yes: Random W
Bangladesh	JiVitA−4 (35)	365	114	117.5	2.77 (0.93, 4.60)	0.11	0.14	90	32	119.5	0.10 (−3.25, 3.46)	0.31	0.18
Bangladesh	RDNS (36)	493	243	113.0	3.77 (1.44, 6.11)	0.07	0.12	35	12	108.8	6.00 (−1.95, 13.94)	0.05	0.10
Bangladesh	WASH−B (37)												
Burkina Faso	iLiNS−Zinc (38)	1457	487	89.2	8.10 (5.54, 10.65)	0.06	0.12	500	177	86.7	9.42 (4.35, 14.49)	0.13	0.15
Burkina Faso	PROMIS CS (39)												
Ghana	GHANA (40)												
Ghana	iLiNS−DYAD−G (41)	285	556	111.5	1.51 (0.05, 2.98)	0.18	0.15	21	54	115.4	−1.97 (−7.31, 3.36)	0.12	0.14
Kenya	WASH−B (42)												
Madagascar	MAHAY (43)												
Malawi	iLiNS−DYAD−M (44)	183	379	108.2	0.85 (−1.91, 3.61)	0.05	0.11	14	37	106.4	2.31 (−6.74, 11.36)	0.04	0.08
Malawi	iLiNS−DOSE (45)	230	75	100.5	4.29 (0.45, 8.14)	0.03	0.08	13	7	101.9	−3.34 (−20.72, 14.03)	0.01	0.03
Mali	PROMIS CS (46)												
Zimbabwe	SHINE (HIV−) (47)	1185	1069	114.9	1.60 (0.70, 2.51)	0.46	0.16	66	67	114.7	3.54 (−0.63, 7.71)	0.20	0.17
Zimbabwe	SHINE (HIV+) (48)	226	202	115.3	3.01 (0.05, 5.96)	0.04	0.11	26	13	108.5	10.08 (5.03, 15.12)	0.14	0.15
		**4424**	**3125**		**I² = 0.74, Tau² = 3.69**			**765**	**399**		**I² = 0.66, Tau² = 12.68**		
**Fixed**					**2.34 (1.73, 2.96)**						**3.50 (1.64, 5.35)**		
**Random**					**3.08 (1.52, 4.65)**						**3.88 (0.57, 7.20)**		

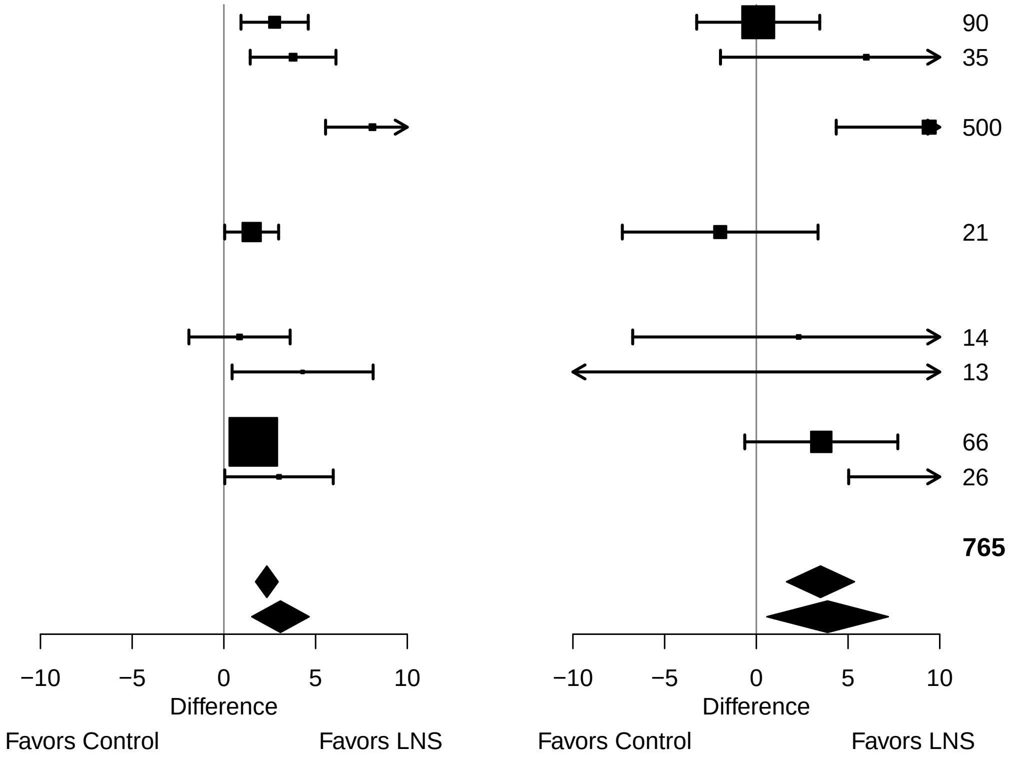

Supplemental figure 8A: Mean difference in hemoglobin concentration

8A7: Stratified by Child baseline anemia

P−for−interaction = 0.063
Difference in MDs = 1.84 (−0.10, 3.79)

		Not anemic						Anemic					
Country	Trial	LNS N	Control N	Control Mean	MD (95% CI)	Fixed W	Random W	LNS N	Control N	Control Mean	MD (95% CI)	Fixed W	Random W
Bangladesh	JiVitA−4 (35)												
Bangladesh	RDNS (36)	213	93	118.7	2.31 (−1.04, 5.66)	0.15	0.21	314	160	109.4	4.25 (1.53, 6.97)	0.25	0.23
Bangladesh	WASH−B (37)												
Burkina Faso	iLiNS−Zinc (38)	177	61	93.9	8.12 (4.37, 11.86)	0.12	0.19	1780	603	88.0	8.53 (5.27, 11.79)	0.18	0.20
Burkina Faso	PROMIS CS (39)												
Ghana	GHANA (40)												
Ghana	iLiNS−DYAD−G (41)	189	355	114.7	0.01 (−1.76, 1.78)	0.55	0.26	90	207	107.1	2.85 (0.45, 5.25)	0.33	0.24
Kenya	WASH−B (42)												
Madagascar	MAHAY (43)												
Malawi	iLiNS−DYAD−M (44)	66	145	112.9	0.22 (−4.11, 4.55)	0.09	0.17	134	269	106.0	0.95 (−2.20, 4.10)	0.19	0.21
Malawi	iLiNS−DOSE (45)	101	45	103.7	3.17 (−1.42, 7.76)	0.08	0.17	142	37	96.9	3.98 (−1.99, 9.94)	0.05	0.11
Mali	PROMIS CS (46)												
Zimbabwe	SHINE (HIV−) (47)												
Zimbabwe	SHINE (HIV+) (48)												
		**746**	**699**		**I² = 0.75, Tau² = 7.57**			**2460**	**1276**		**I² = 0.66, Tau² = 5.13**		
**Fixed**					**1.63 (0.32, 2.95)**						**3.91 (2.54, 5.28)**		
**Random**					**2.62 (−0.27, 5.50)**						**4.06 (1.57, 6.56)**		

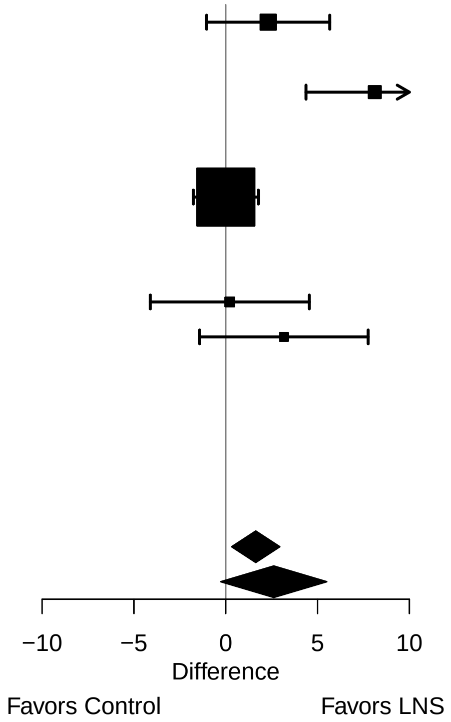

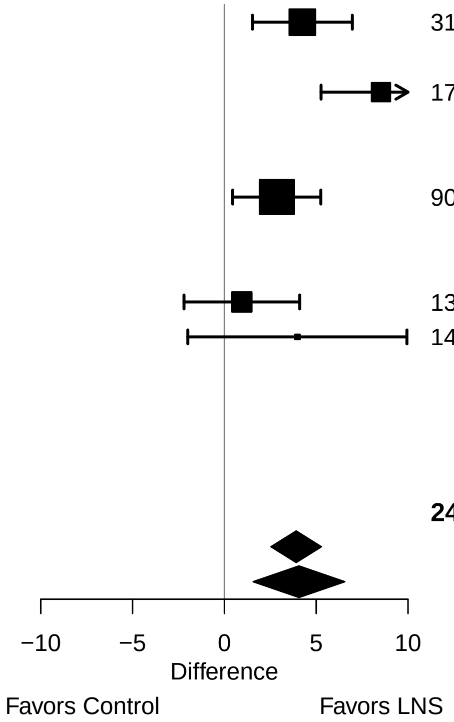

Supplemental figure 8A: Mean difference in hemoglobin concentration

8A8: Stratified by Child high-dose vitamin A supplementation

P−for−interaction = 0.808
Difference in MDs = 0.15 (−1.03, 1.33)

		Received VitA						Not treated					
Country	Trial	LNS N	Control N	Control Mean	MD (95% CI)	Fixed W	Random W	LNS N	Control N	Control Mean	MD (95% CI)	Fixed W	Random W
Bangladesh	JiVitA−4 (35)	141	37	118.5	1.63 (−2.54, 5.81)	0.03	0.09	133	41	118.8	1.17 (−1.56, 3.91)	0.09	0.13
Bangladesh	RDNS (36)	444	223	112.8	3.91 (1.28, 6.54)	0.09	0.14	105	49	110.5	5.04 (1.41, 8.68)	0.05	0.10
Bangladesh	WASH−B (37)	20	18	117.3	3.57 (−1.97, 9.11)	0.02	0.06	16	9	119.6	−2.43 (−9.10, 4.23)	0.02	0.04
Burkina Faso	iLiNS−Zinc (38)												
Burkina Faso	PROMIS CS (39)												
Ghana	GHANA (40)	50	62	105.6	10.67 (5.60, 15.74)	0.02	0.07	48	29	104.7	8.05 (1.40, 14.70)	0.02	0.04
Ghana	iLiNS−DYAD−G (41)	215	412	111.7	1.71 (−0.02, 3.44)	0.20	0.19	113	249	112.4	0.40 (−1.88, 2.67)	0.13	0.14
Kenya	WASH−B (42)	29	34	109.9	1.75 (−5.04, 8.54)	0.01	0.04	263	212	109.7	4.24 (1.90, 6.58)	0.12	0.14
Madagascar	MAHAY (43)												
Malawi	iLiNS−DYAD−M (44)	75	136	106.6	2.65 (−1.96, 7.27)	0.03	0.08	133	289	108.6	0.43 (−2.70, 3.56)	0.07	0.11
Malawi	iLiNS−DOSE (45)												
Mali	PROMIS CS (46)												
Zimbabwe	SHINE (HIV−) (47)	874	833	114.7	1.71 (0.67, 2.74)	0.55	0.22	665	622	114.0	2.57 (1.31, 3.83)	0.43	0.19
Zimbabwe	SHINE (HIV+) (48)	165	127	115.8	2.35 (−1.09, 5.78)	0.05	0.11	112	134	113.9	3.09 (−0.12, 6.29)	0.07	0.11
		**2013**	**1882**		**I² = 0.43, Tau² = 2.66**			**1588**	**1634**		**I² = 0.46, Tau² = 2.70**		
**Fixed**					**2.19 (1.42, 2.96)**						**2.39 (1.56, 3.21)**		
**Random**					**2.89 (1.32, 4.46)**						**2.39 (0.89, 3.89)**		

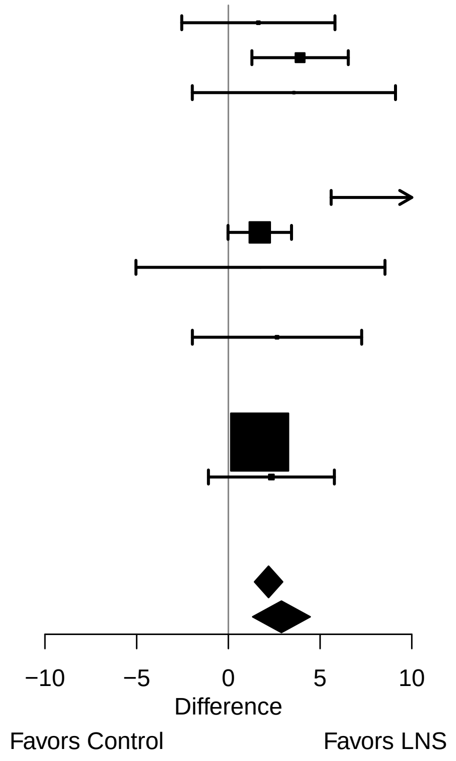

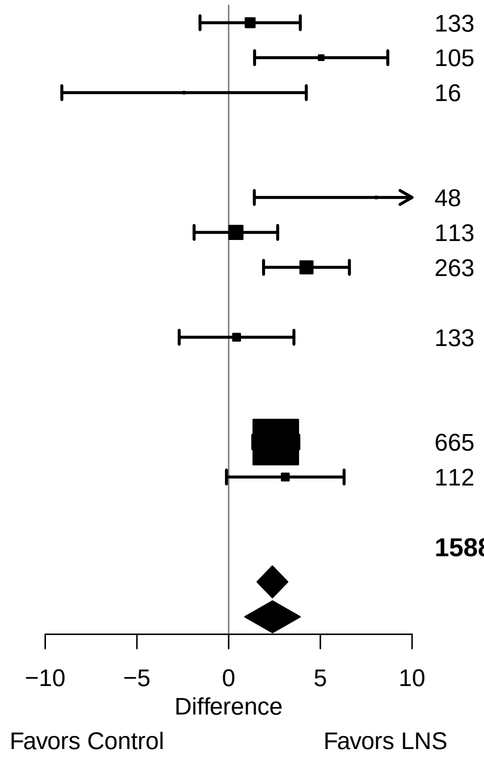

Supplemental figure 8A: Mean difference in hemoglobin concentration

8A9: Stratified by Child inflammation

P−for−interaction = 0.712
Difference in MDs = −0.25 (−1.59, 1.09)

Not inflamed

Country	Trial	LNS N	Control N	Control Mean	MD (95% CI)	Fixed W	Random W
Bangladesh	JiVitA−4 (35)	185	47	118.2	2.82 (0.23, 5.41)	0.10	0.11
Bangladesh	RDNS (36)	369	168	112.6	4.59 (1.81, 7.37)	0.09	0.10
Bangladesh	WASH−B (37)	165	136	118.7	2.74 (0.53, 4.95)	0.14	0.12
Burkina Faso	iLiNS−Zinc (38)	131	33	91.7	8.95 (1.27, 16.62)	0.01	0.03
Burkina Faso	PROMIS CS (39)						
Ghana	GHANA (40)	70	66	106.6	9.72 (5.38, 14.07)	0.04	0.06
Ghana	iLiNS−DYAD−G (41)	45	120	111.0	1.78 (−1.79, 5.36)	0.05	0.08
Kenya	WASH−B (42)	157	135	112.2	4.72 (2.12, 7.31)	0.10	0.11
Madagascar	MAHAY (43)	44	32	111.2	6.73 (1.94, 11.51)	0.03	0.06
Malawi	iLiNS−DYAD−M (44)	59	123	112.6	2.45 (−1.78, 6.68)	0.04	0.07
Malawi	iLiNS−DOSE (45)	20	6	105.2	7.34 (−2.36, 17.05)	0.01	0.02
Mali	PROMIS CS (46)						
Zimbabwe	SHINE (HIV−) (47)	414	354	116.1	0.98 (−0.61, 2.56)	0.27	0.14
Zimbabwe	SHINE (HIV+) (48)	186	158	114.8	3.76 (1.39, 6.13)	0.12	0.11
		**1845**	**1378**		**I² = 0.55, Tau² = 3.07**		
**Fixed**					**3.17 (2.34, 3.99)**		
**Random**					**3.86 (2.45, 5.27)**		

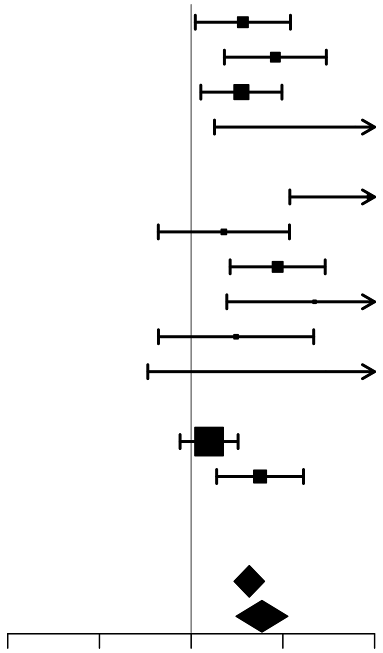

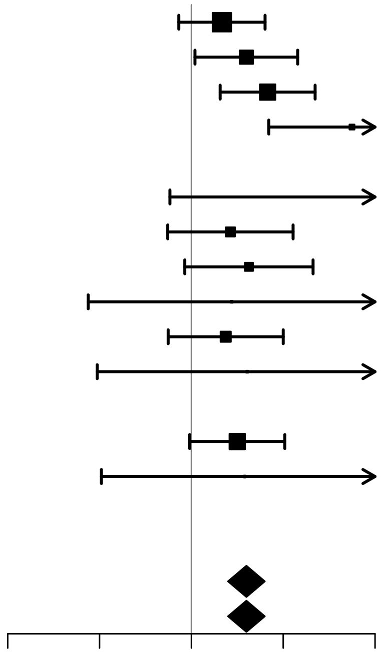

High AGP or CRP

LNS N	Control N	Control Mean	MD (95% CI)	Fixed W	Random W
269	98	118.0	1.66 (−0.68, 4.01)	0.18	0.18
180	104	112.0	2.99 (0.20, 5.79)	0.13	0.13
47	42	117.0	4.15 (1.57, 6.74)	0.15	0.15
197	69	87.3	8.74 (4.22, 13.27)	0.05	0.05
14	16	94.1	9.91 (−1.17, 20.99)	0.01	0.01
55	82	109.4	2.13 (−1.29, 5.54)	0.09	0.09
141	123	106.8	3.13 (−0.36, 6.63)	0.08	0.08
39	19	110.4	2.20 (−5.62, 10.01)	0.02	0.02
138	269	105.8	1.87 (−1.27, 5.00)	0.10	0.10
49	24	100.7	3.04 (−5.13, 11.21)	0.02	0.02
105	99	112.0	2.50 (−0.09, 5.09)	0.15	0.15
32	31	114.2	2.90 (−4.90, 10.70)	0.02	0.02
**1266**	**976**		**I² = 0.00, Tau² = 0.00**		
			**3.00 (1.99, 4.00)**		
			**3.00 (1.99, 4.00)**		

Supplemental figure 8B: Anemia prevalence ratio

8B1: Stratified by Maternal BMI

P−for−interaction = 0.637
Ratio of PRs = 1.01 (0.97, 1.06)

		**At least 20 kg/m²**						**Less than 20 kg/m²**					
Country	Trial	**LNS** N	**Control** N	**Control** Prevalence	**PR** (95% CI)	**Fixed** W	**Random** W	**LNS** N	**Control** N	**Control** Prevalence	**PR** (95% CI)	**Fixed** W	**Random** W
Bangladesh	JiVitA−4 (35)												
Bangladesh	RDNS (36)	227	102	42.2	0.70 (0.50, 0.98)	0.01	0.05	300	160	41.9	0.62 (0.46, 0.84)	0.02	0.08
Bangladesh	WASH−B (37)	102	102	17.6	0.33 (0.14, 0.80)	0.00	0.01	132	84	14.3	0.53 (0.26, 1.07)	0.00	0.02
Burkina Faso	iLiNS−Zinc (38)	1217	390	91.5	0.87 (0.83, 0.91)	0.49	0.15	738	274	90.5	0.87 (0.82, 0.93)	0.50	0.18
Burkina Faso	PROMIS CS (39)	335	349	70.2	0.97 (0.87, 1.08)	0.10	0.13	239	227	70.0	0.88 (0.75, 1.04)	0.09	0.14
Ghana	GHANA (40)												
Ghana	iLiNS−DYAD−G (41)	288	536	42.9	0.86 (0.72, 1.03)	0.04	0.10	35	114	51.8	0.94 (0.64, 1.38)	0.01	0.06
Kenya	WASH−B (42)	266	227	48.9	0.65 (0.52, 0.80)	0.03	0.09	72	60	36.7	0.91 (0.62, 1.34)	0.01	0.06
Madagascar	MAHAY (43)												
Malawi	iLiNS−DYAD−M (44)	124	258	51.2	0.87 (0.69, 1.09)	0.02	0.08	85	171	53.8	0.92 (0.71, 1.19)	0.03	0.09
Malawi	iLiNS−DOSE (45)	179	52	75.0	0.77 (0.63, 0.93)	0.03	0.09	61	29	69.0	1.06 (0.80, 1.40)	0.03	0.08
Mali	PROMIS CS (46)	702	685	86.6	0.82 (0.76, 0.88)	0.20	0.14	246	279	85.7	0.82 (0.74, 0.90)	0.25	0.17
Zimbabwe	SHINE (HIV−) (47)	1222	1161	34.7	0.76 (0.67, 0.86)	0.07	0.12	213	220	33.2	0.93 (0.72, 1.22)	0.03	0.09
Zimbabwe	SHINE (HIV+) (48)	221	222	36.9	0.67 (0.48, 0.95)	0.01	0.05	58	40	40.0	0.43 (0.22, 0.83)	0.01	0.02
		**4883**	**4084**		**I² = 0.61, Tau² = 0.01**			**2179**	**1658**		**I² = 0.34, Tau² = 0.02**		
**Fixed**					**0.84 (0.81, 0.87)**						**0.86 (0.82, 0.90)**		
**Random**					**0.80 (0.73, 0.88)**						**0.85 (0.76, 0.94)**		

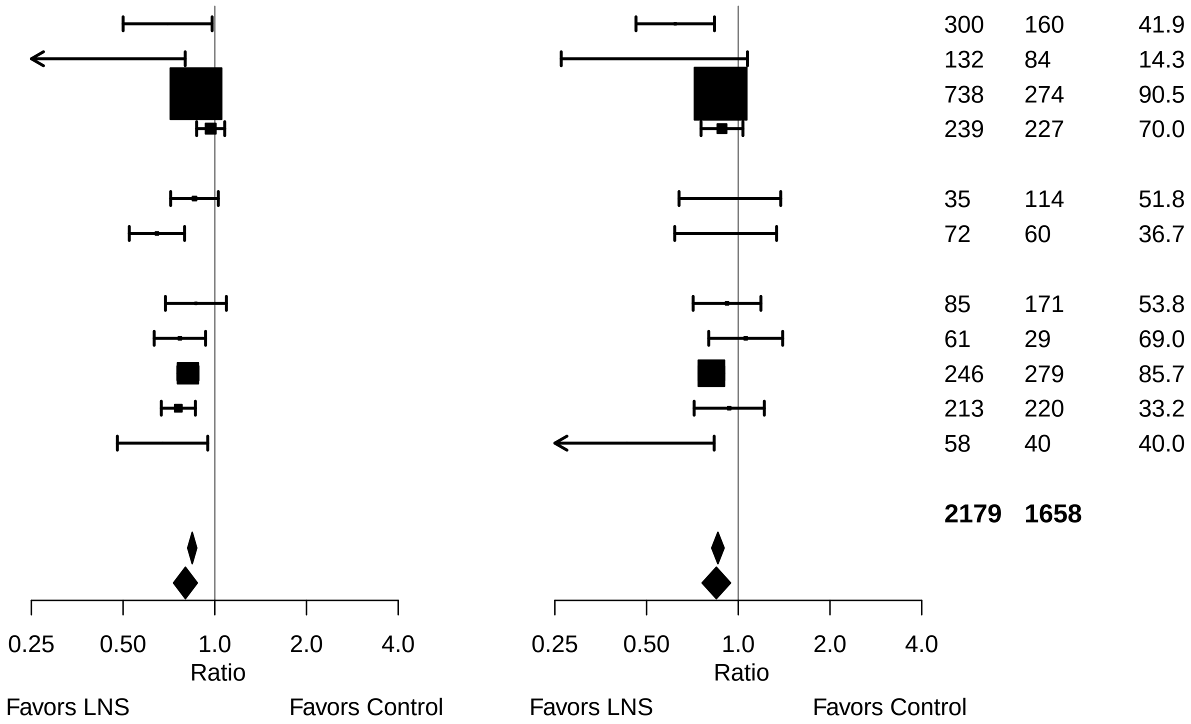

Supplemental figure 8B: Anemia prevalence ratio

8B2: Stratified by Maternal age

P−for−interaction = 0.041

Ratio of PRs = 1.05 (1.00, 1.10)

Country	Trial	At least 25 y: LNS N	Control N	Control Prevalence	PR (95% CI)	Fixed W	Random W	Less than 25 y: LNS N	Control N	Control Prevalence	PR (95% CI)	Fixed W	Random W
Bangladesh	JiVitA−4 (35)	203	72	19.4	0.63 (0.35, 1.15)	0.00	0.03	251	73	12.3	1.10 (0.54, 2.22)	0.00	0.01
Bangladesh	RDNS (36)	152	81	40.7	0.60 (0.39, 0.92)	0.01	0.04	397	191	42.4	0.68 (0.53, 0.86)	0.03	0.06
Bangladesh	WASH−B (37)	89	83	14.5	0.39 (0.14, 1.06)	0.00	0.01	144	103	17.5	0.44 (0.24, 0.81)	0.00	0.01
Burkina Faso	iLiNS−Zinc (38)	1132	399	91.7	0.86 (0.82, 0.90)	0.52	0.12	812	262	90.1	0.89 (0.82, 0.96)	0.28	0.15
Burkina Faso	PROMIS CS (39)	271	263	67.3	0.89 (0.78, 1.03)	0.07	0.10	303	318	72.3	0.97 (0.87, 1.09)	0.14	0.13
Ghana	GHANA (40)	69	63	57.1	0.43 (0.27, 0.69)	0.01	0.04	29	25	64.0	0.65 (0.38, 1.09)	0.01	0.02
Ghana	iLiNS−DYAD−G (41)	206	397	42.3	0.80 (0.64, 1.00)	0.03	0.08	122	264	48.9	0.96 (0.76, 1.20)	0.03	0.07
Kenya	WASH−B (42)	213	168	45.2	0.71 (0.57, 0.87)	0.03	0.08	133	131	49.6	0.65 (0.48, 0.88)	0.02	0.04
Madagascar	MAHAY (43)	301	329	62.9	0.92 (0.78, 1.08)	0.05	0.09	299	259	67.2	0.85 (0.73, 0.98)	0.09	0.11
Malawi	iLiNS−DYAD−M (44)	99	220	47.3	0.96 (0.74, 1.24)	0.02	0.07	111	212	56.6	0.84 (0.67, 1.06)	0.03	0.07
Malawi	iLiNS−DOSE (45)	135	50	72.0	0.84 (0.69, 1.04)	0.03	0.08	104	28	71.4	0.89 (0.68, 1.16)	0.02	0.05
Mali	PROMIS CS (46)	502	508	87.8	0.79 (0.72, 0.86)	0.18	0.11	451	462	84.4	0.85 (0.78, 0.92)	0.25	0.15
Zimbabwe	SHINE (HIV−) (47)	808	726	33.7	0.71 (0.61, 0.83)	0.05	0.10	697	694	36.3	0.85 (0.73, 0.98)	0.08	0.11
Zimbabwe	SHINE (HIV+) (48)	221	204	37.7	0.56 (0.39, 0.80)	0.01	0.05	66	60	33.3	0.91 (0.57, 1.45)	0.01	0.02
		**4401**	**3563**		**I² = 0.57, Tau² = 0.02**			**3919**	**3082**		**I² = 0.33, Tau² = 0.01**		
**Fixed**					**0.83 (0.80, 0.86)**						**0.87 (0.83, 0.90)**		
**Random**					**0.77 (0.69, 0.86)**						**0.85 (0.79, 0.91)**		

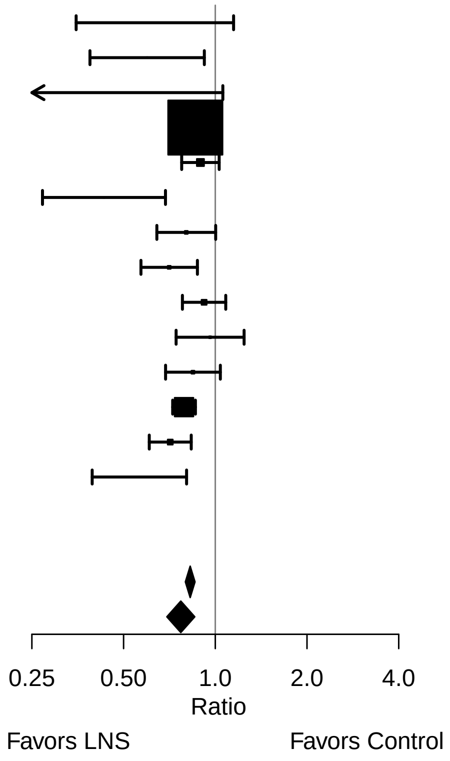

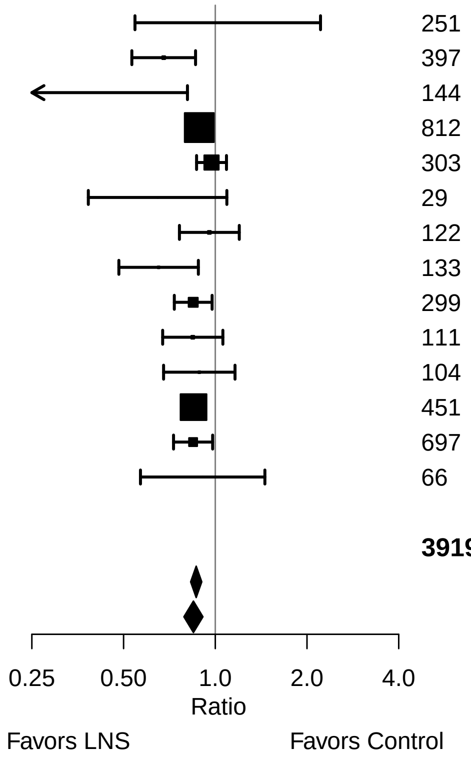

Supplemental figure 8B: Anemia prevalence ratio

8B3: Stratified by Maternal education

P−for−interaction = 0.621
Ratio of PRs = 1.03 (0.93, 1.14)

		Primary or greater						Incomplete or no formal					
Country	Trial	LNS N	Control N	Control Prevalence	PR (95% CI)	Fixed W	Random W	LNS N	Control N	Control Prevalence	PR (95% CI)	Fixed W	Random W
Bangladesh	JiVitA−4 (35)	299	93	17.2	0.64 (0.35, 1.19)	0.01	0.05	157	53	13.2	1.30 (0.69, 2.47)	0.01	0.03
Bangladesh	RDNS (36)	405	195	41.0	0.62 (0.47, 0.82)	0.05	0.11	144	77	44.2	0.75 (0.50, 1.13)	0.02	0.06
Bangladesh	WASH−B (37)	163	145	15.9	0.39 (0.20, 0.75)	0.01	0.05	71	41	17.1	0.49 (0.18, 1.36)	0.00	0.01
Burkina Faso	iLiNS−Zinc (38)												
Burkina Faso	PROMIS CS (39)	46	47	70.2	1.05 (0.83, 1.34)	0.07	0.11	526	532	69.9	0.93 (0.84, 1.03)	0.26	0.19
Ghana	GHANA (40)												
Ghana	iLiNS−DYAD−G (41)	253	526	45.4	0.83 (0.69, 0.99)	0.12	0.13	75	135	43.0	0.99 (0.72, 1.38)	0.03	0.08
Kenya	WASH−B (42)	168	136	50.0	0.46 (0.34, 0.63)	0.04	0.10	181	164	45.1	0.91 (0.72, 1.14)	0.05	0.12
Madagascar	MAHAY (43)	128	143	64.3	0.79 (0.62, 1.00)	0.07	0.12	472	445	64.9	0.91 (0.79, 1.05)	0.13	0.16
Malawi	iLiNS−DYAD−M (44)	34	66	48.5	0.79 (0.48, 1.29)	0.02	0.07	175	363	52.9	0.91 (0.76, 1.09)	0.08	0.14
Malawi	iLiNS−DOSE (45)												
Mali	PROMIS CS (46)	107	96	84.4	0.90 (0.78, 1.03)	0.22	0.14	845	874	86.4	0.81 (0.75, 0.88)	0.42	0.20
Zimbabwe	SHINE (HIV−) (47)	1536	1462	34.9	0.80 (0.72, 0.89)	0.37	0.14	58	53	39.6	0.35 (0.16, 0.75)	0.00	0.02
Zimbabwe	SHINE (HIV+) (48)												
		**3139**	**2909**		**I² = 0.68, Tau² = 0.05**			**2704**	**2737**		**I² = 0.39, Tau² = 0.02**		
**Fixed**					**0.80 (0.75, 0.86)**						**0.87 (0.82, 0.91)**		
**Random**					**0.74 (0.62, 0.88)**						**0.87 (0.78, 0.98)**		

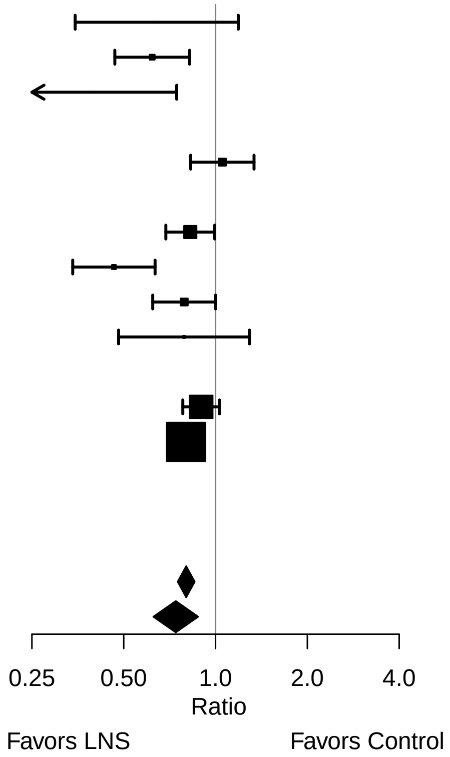

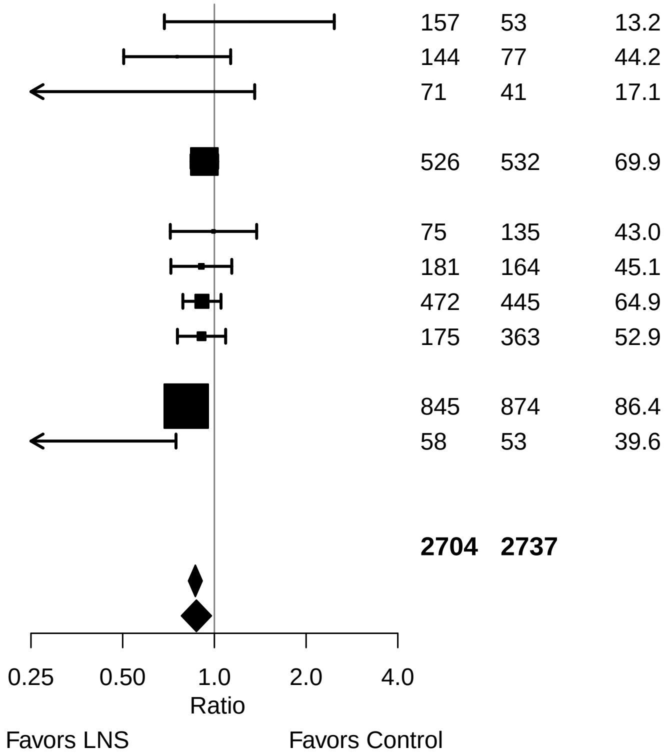

Supplemental figure 8B: Anemia prevalence ratio

8B4: Stratified by Child sex

P−for−interaction = 0.039
Ratio of PRs = 0.96 (0.92, 1.00)

		**Male**						**Female**					
Country	Trial	**LNS** N	**Control** N	**Control** Prevalence	**PR** (95% CI)	**Fixed** W	**Random** W	**LNS** N	**Control** N	**Control** Prevalence	**PR** (95% CI)	**Fixed** W	**Random** W
Bangladesh	JiVitA−4 (35)	235	71	22.5	0.47 (0.28, 0.78)	0.00	0.04	222	75	9.3	1.69 (0.91, 3.13)	0.00	0.02
Bangladesh	RDNS (36)	266	131	41.2	0.62 (0.42, 0.91)	0.01	0.05	281	141	42.6	0.68 (0.51, 0.91)	0.02	0.06
Bangladesh	WASH−B (37)	116	89	16.9	0.31 (0.12, 0.82)	0.00	0.01	118	97	15.5	0.55 (0.29, 1.04)	0.00	0.02
Burkina Faso	iLiNS−Zinc (38)	993	334	91.6	0.88 (0.85, 0.92)	0.56	0.10	964	330	90.6	0.86 (0.80, 0.92)	0.38	0.12
Burkina Faso	PROMIS CS (39)	317	295	77.3	0.93 (0.83, 1.05)	0.07	0.09	257	286	62.6	0.91 (0.80, 1.04)	0.11	0.11
Ghana	GHANA (40)	60	41	61.0	0.46 (0.29, 0.74)	0.00	0.04	38	55	56.4	0.56 (0.33, 0.94)	0.01	0.03
Ghana	iLiNS−DYAD−G (41)	164	310	44.8	0.97 (0.78, 1.20)	0.02	0.08	163	348	44.8	0.75 (0.59, 0.96)	0.03	0.07
Kenya	WASH−B (42)	168	145	44.8	0.81 (0.63, 1.04)	0.02	0.07	182	155	49.7	0.58 (0.45, 0.73)	0.03	0.08
Madagascar	MAHAY (43)	277	288	65.6	0.95 (0.83, 1.09)	0.05	0.09	323	300	64.0	0.83 (0.70, 0.98)	0.07	0.09
Malawi	iLiNS−DYAD−M (44)	103	202	56.4	0.95 (0.76, 1.18)	0.02	0.08	107	230	47.8	0.84 (0.64, 1.10)	0.03	0.07
Malawi	iLiNS−DOSE (45)	123	43	76.7	0.84 (0.68, 1.04)	0.02	0.08	120	39	66.7	0.90 (0.70, 1.17)	0.03	0.07
Mali	PROMIS CS (46)	479	526	89.0	0.84 (0.78, 0.91)	0.17	0.10	474	444	82.9	0.79 (0.71, 0.88)	0.18	0.11
Zimbabwe	SHINE (HIV−) (47)	830	814	36.2	0.81 (0.71, 0.94)	0.05	0.09	852	780	34.2	0.74 (0.64, 0.85)	0.09	0.10
Zimbabwe	SHINE (HIV+) (48)	146	149	40.9	0.62 (0.46, 0.83)	0.01	0.07	160	136	32.4	0.70 (0.46, 1.04)	0.01	0.04
		**4277**	**3438**		**I² = 0.59, Tau² = 0.04**			**4261**	**3416**		**I² = 0.53, Tau² = 0.02**		
**Fixed**					**0.87 (0.84, 0.90)**						**0.82 (0.78, 0.85)**		
**Random**					**0.79 (0.70, 0.90)**						**0.78 (0.71, 0.87)**		

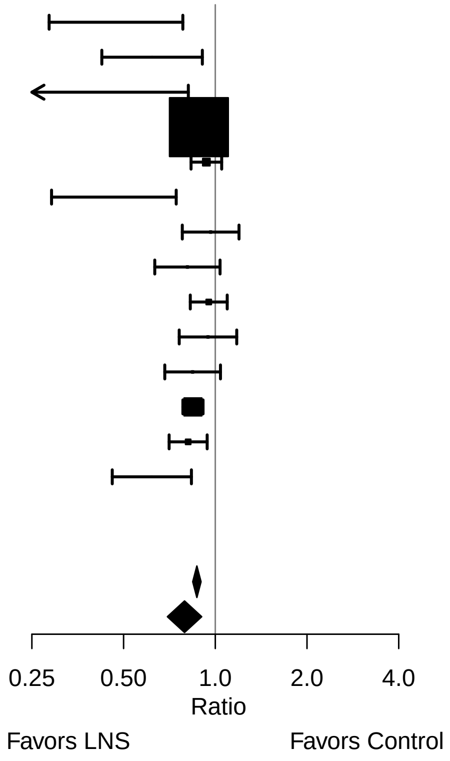

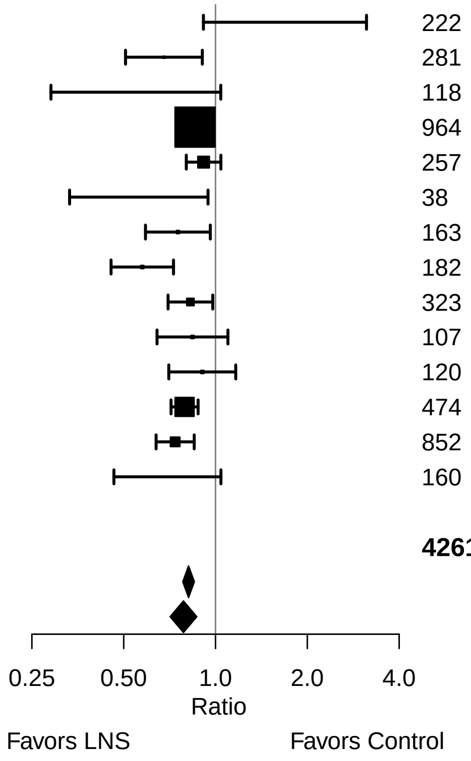

Supplemental figure 8B: Anemia prevalence ratio

8B5: Stratified by Child birth order

P−for−interaction = 0.156
Ratio of PRs = 1.04 (0.99, 1.09)

		Later born						Firstborn					
Country	Trial	LNS N	Control N	Control Prevalence	PR (95% CI)	Fixed W	Random W	LNS N	Control N	Control Prevalence	PR (95% CI)	Fixed W	Random W
Bangladesh	JiVitA−4 (35)	115	33	27.3	0.54 (0.27, 1.09)	0.00	0.03	341	112	12.5	1.01 (0.63, 1.61)	0.01	0.02
Bangladesh	RDNS (36)	339	171	40.9	0.66 (0.49, 0.88)	0.01	0.07	210	101	43.6	0.66 (0.47, 0.92)	0.02	0.04
Bangladesh	WASH−B (37)	137	123	16.3	0.31 (0.14, 0.69)	0.00	0.02	95	61	16.4	0.51 (0.20, 1.31)	0.00	0.01
Burkina Faso	iLiNS−Zinc (38)	1522	520	90.4	0.87 (0.82, 0.92)	0.38	0.11	435	144	93.8	0.87 (0.82, 0.92)	0.64	0.29
Burkina Faso	PROMIS CS (39)	498	477	70.0	0.93 (0.84, 1.03)	0.11	0.10	75	103	69.9	0.99 (0.80, 1.23)	0.04	0.09
Ghana	GHANA (40)	60	54	61.1	0.38 (0.23, 0.63)	0.00	0.04	38	38	55.3	0.71 (0.44, 1.16)	0.01	0.02
Ghana	iLiNS−DYAD−G (41)	220	435	45.3	0.76 (0.62, 0.94)	0.03	0.09	108	226	44.2	1.07 (0.83, 1.37)	0.03	0.07
Kenya	WASH−B (42)	282	244	47.1	0.67 (0.54, 0.83)	0.03	0.08	67	56	48.2	0.74 (0.46, 1.20)	0.01	0.02
Madagascar	MAHAY (43)	458	454	66.1	0.90 (0.78, 1.03)	0.06	0.10	141	131	61.1	0.84 (0.66, 1.05)	0.04	0.08
Malawi	iLiNS−DYAD−M (44)	165	342	50.9	0.82 (0.67, 1.01)	0.03	0.09	45	88	55.7	1.16 (0.87, 1.54)	0.03	0.06
Malawi	iLiNS−DOSE (45)												
Mali	PROMIS CS (46)	790	832	86.1	0.81 (0.76, 0.88)	0.22	0.10	146	126	88.1	0.82 (0.71, 0.93)	0.11	0.17
Zimbabwe	SHINE (HIV−) (47)	1188	1100	35.5	0.71 (0.63, 0.80)	0.10	0.10	418	416	33.2	0.94 (0.77, 1.15)	0.05	0.10
Zimbabwe	SHINE (HIV+) (48)	252	216	36.6	0.63 (0.47, 0.84)	0.02	0.07	51	64	37.5	0.78 (0.46, 1.35)	0.01	0.02
		**6026**	**5001**		**I² = 0.72, Tau² = 0.04**			**2170**	**1666**		**I² = 0.19, Tau² = 0.00**		
**Fixed**					**0.83 (0.80, 0.86)**						**0.87 (0.83, 0.91)**		
**Random**					**0.73 (0.64, 0.84)**						**0.88 (0.82, 0.95)**		

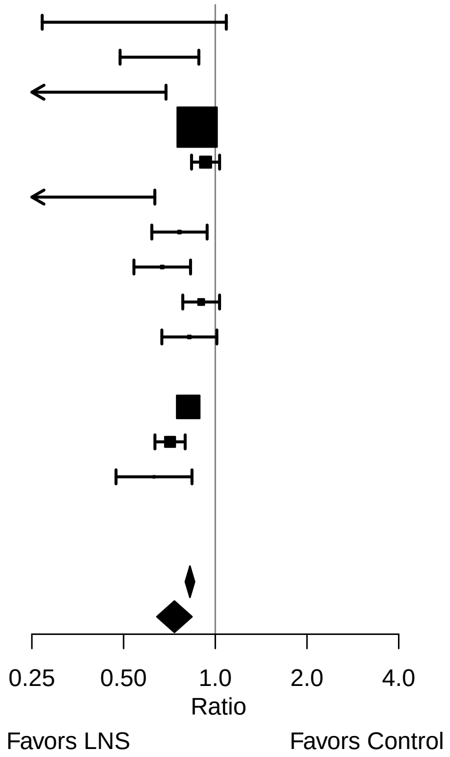

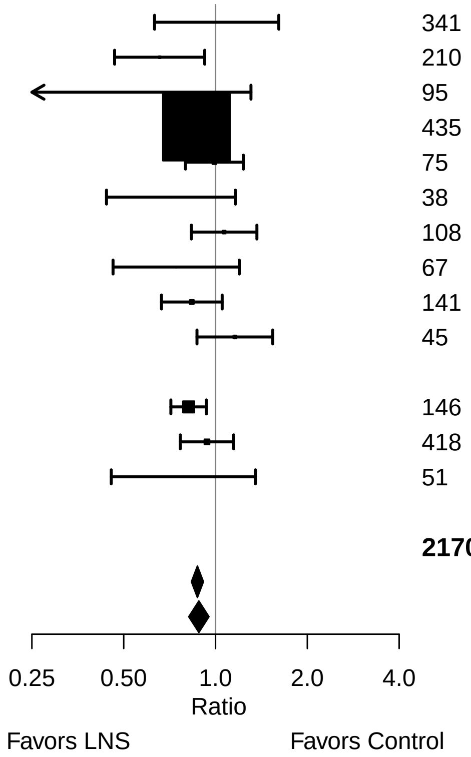

Supplemental figure 8B: Anemia prevalence ratio

8B6: Stratified by Child baseline acute malnutrition

P−for−interaction = 0.393
Ratio of PRs = 0.98 (0.93, 1.03)

Country	Trial	No: LNS N	No: Control N	No: Control Prevalence	No: PR (95% CI)	No: Fixed W	No: Random W	Yes: LNS N	Yes: Control N	Yes: Control Prevalence	Yes: PR (95% CI)	Yes: Fixed W	Yes: Random W
Bangladesh	JiVitA−4 (35)	365	114	15.8	0.80 (0.53, 1.21)	0.01	0.04	90	32	15.6	1.00 (0.40, 2.49)	0.00	0.00
Bangladesh	RDNS (36)	493	243	40.3	0.65 (0.51, 0.85)	0.03	0.09	35	12	41.7	0.82 (0.31, 2.21)	0.00	0.00
Bangladesh	WASH−B (37)												
Burkina Faso	iLiNS−Zinc (38)	1457	487	90.3	0.88 (0.83, 0.92)	0.71	0.31	500	177	93.2	0.85 (0.80, 0.91)	0.95	0.95
Burkina Faso	PROMIS CS (39)												
Ghana	GHANA (40)												
Ghana	iLiNS−DYAD−G (41)	285	556	46.4	0.84 (0.71, 1.00)	0.06	0.15	21	54	31.5	1.06 (0.51, 2.18)	0.01	0.01
Kenya	WASH−B (42)												
Madagascar	MAHAY (43)												
Malawi	iLiNS−DYAD−M (44)	183	379	51.5	0.90 (0.75, 1.08)	0.06	0.14	14	37	51.4	1.11 (0.64, 1.93)	0.01	0.01
Malawi	iLiNS−DOSE (45)												
Mali	PROMIS CS (46)												
Zimbabwe	SHINE (HIV−) (47)	1185	1069	32.8	0.80 (0.70, 0.91)	0.11	0.20	66	67	32.8	0.74 (0.42, 1.31)	0.01	0.01
Zimbabwe	SHINE (HIV+) (48)	226	202	34.7	0.63 (0.46, 0.85)	0.02	0.07	26	13	61.5	0.38 (0.16, 0.86)	0.01	0.01
		**4194**	**3050**		**I^2 = 0.42, Tau^2 = 0.01**			**752**	**392**		**I^2 = 0.00, Tau^2 = 0.00**		
**Fixed**					**0.85 (0.82, 0.89)**						**0.85 (0.80, 0.91)**		
**Random**					**0.82 (0.75, 0.89)**						**0.85 (0.80, 0.91)**		

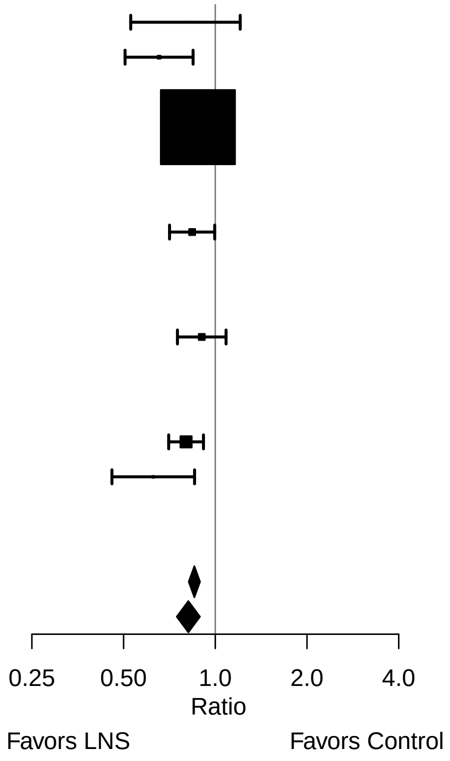

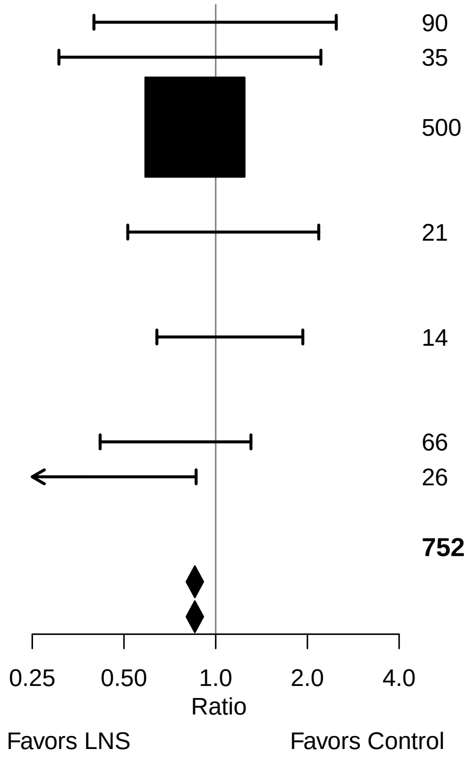

Supplemental figure 8B: Anemia prevalence ratio

8B7: Stratified by Child baseline anemia

P−for−interaction = 0.683
Ratio of PRs = 1.03 (0.91, 1.16)

		Not anemic						Anemic					
Country	Trial	LNS N	Control N	Control Prevalence	PR (95% CI)	Fixed W	Random W	LNS N	Control N	Control Prevalence	PR (95% CI)	Fixed W	Random W
Bangladesh	JiVitA−4 (35)												
Bangladesh	RDNS (36)	213	93	23.7	0.62 (0.34, 1.10)	0.04	0.05	314	160	50.0	0.71 (0.55, 0.90)	0.04	0.04
Bangladesh	WASH−B (37)												
Burkina Faso	iLiNS−Zinc (38)	177	61	82.0	0.79 (0.68, 0.93)	0.50	0.44	1780	603	92.0	0.88 (0.83, 0.92)	0.79	0.79
Burkina Faso	PROMIS CS (39)												
Ghana	GHANA (40)												
Ghana	iLiNS−DYAD−G (41)	189	355	32.7	1.00 (0.78, 1.29)	0.20	0.22	90	207	64.7	0.81 (0.65, 1.01)	0.05	0.05
Kenya	WASH−B (42)												
Madagascar	MAHAY (43)												
Malawi	iLiNS−DYAD−M (44)	66	145	36.6	1.04 (0.71, 1.51)	0.09	0.11	134	269	59.1	0.88 (0.73, 1.07)	0.06	0.06
Malawi	iLiNS−DOSE (45)	101	45	64.4	0.85 (0.64, 1.12)	0.17	0.19	142	37	81.1	0.88 (0.73, 1.06)	0.06	0.06
Mali	PROMIS CS (46)												
Zimbabwe	SHINE (HIV−) (47)												
Zimbabwe	SHINE (HIV+) (48)												
		**746**	**699**		**I² = 0.13, Tau² = 0.00**			**2460**	**1276**		**I² = 0.00, Tau² = 0.00**		
**Fixed**					**0.85 (0.76, 0.96)**						**0.87 (0.83, 0.91)**		
**Random**					**0.86 (0.76, 0.98)**						**0.87 (0.83, 0.91)**		

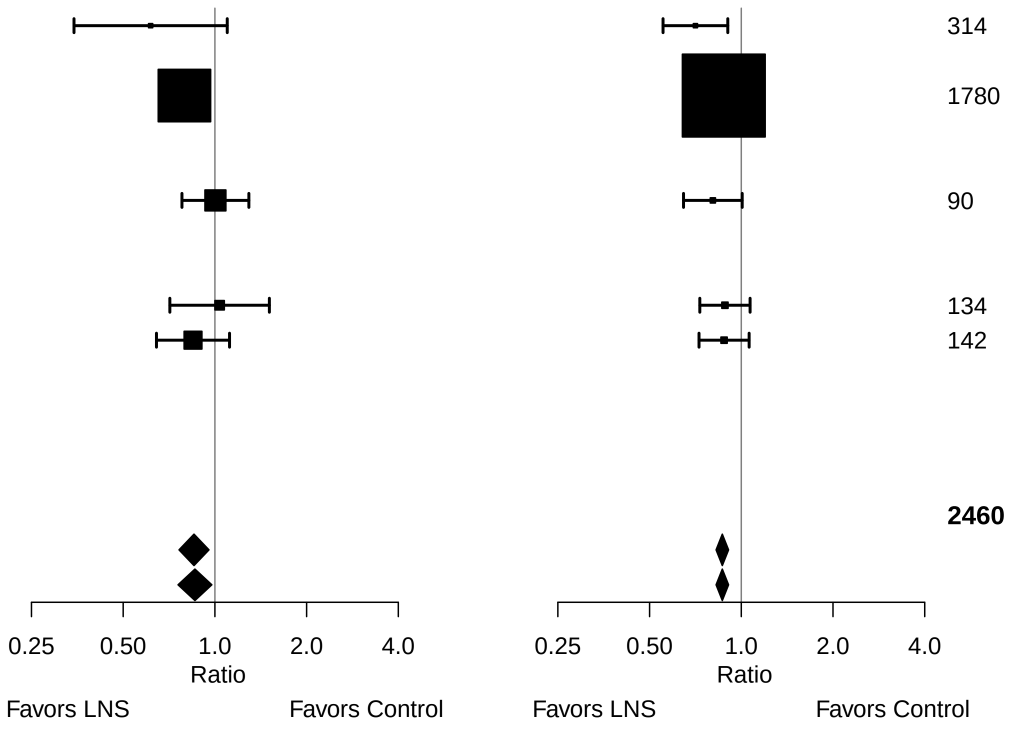

Supplemental figure 8B: Anemia prevalence ratio

8B8: Stratified by Child high-dose vitamin A supplementation

P−for−interaction = 0.760
Ratio of PRs = 0.98 (0.85, 1.12)

		Received VitA						Not treated					
Country	Trial	LNS N	Control N	Control Prevalence	PR (95% CI)	Fixed W	Random W	LNS N	Control N	Control Prevalence	PR (95% CI)	Fixed W	Random W
Bangladesh	JiVitA−4 (35)	141	37	16.2	0.96 (0.38, 2.44)	0.01	0.03	133	41	14.6	0.87 (0.36, 2.14)	0.01	0.02
Bangladesh	RDNS (36)	444	223	40.8	0.67 (0.50, 0.89)	0.11	0.15	105	49	46.9	0.61 (0.43, 0.87)	0.07	0.09
Bangladesh	WASH−B (37)												
Burkina Faso	iLiNS−Zinc (38)												
Burkina Faso	PROMIS CS (39)												
Ghana	GHANA (40)	50	62	56.5	0.39 (0.22, 0.69)	0.03	0.06	48	29	65.5	0.57 (0.36, 0.90)	0.04	0.06
Ghana	iLiNS−DYAD−G (41)	215	412	45.6	0.87 (0.71, 1.05)	0.23	0.21	113	249	43.8	0.85 (0.64, 1.12)	0.11	0.13
Kenya	WASH−B (42)	29	34	52.9	0.65 (0.36, 1.19)	0.02	0.05	263	212	46.7	0.68 (0.55, 0.84)	0.19	0.19
Madagascar	MAHAY (43)												
Malawi	iLiNS−DYAD−M (44)	75	136	58.8	0.77 (0.58, 1.03)	0.11	0.15	133	289	48.4	0.98 (0.79, 1.21)	0.18	0.18
Malawi	iLiNS−DOSE (45)												
Mali	PROMIS CS (46)												
Zimbabwe	SHINE (HIV−) (47)	874	833	33.7	0.83 (0.72, 0.97)	0.42	0.24	665	622	35.9	0.71 (0.61, 0.83)	0.34	0.24
Zimbabwe	SHINE (HIV+) (48)	165	127	37.0	0.59 (0.41, 0.85)	0.07	0.11	112	134	36.6	0.63 (0.43, 0.94)	0.06	0.08
		**1993**	**1864**		**I² = 0.39, Tau² = 0.02**			**1572**	**1625**		**I² = 0.38, Tau² = 0.01**		
**Fixed**					**0.78 (0.71, 0.85)**						**0.75 (0.68, 0.82)**		
**Random**					**0.73 (0.63, 0.85)**						**0.74 (0.65, 0.84)**		

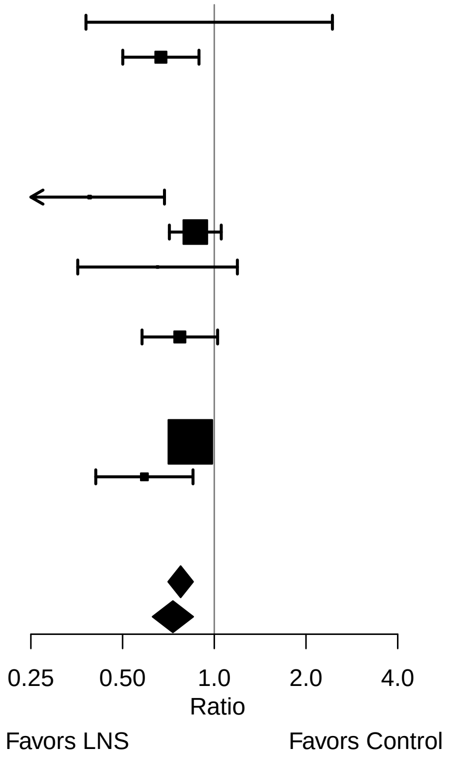

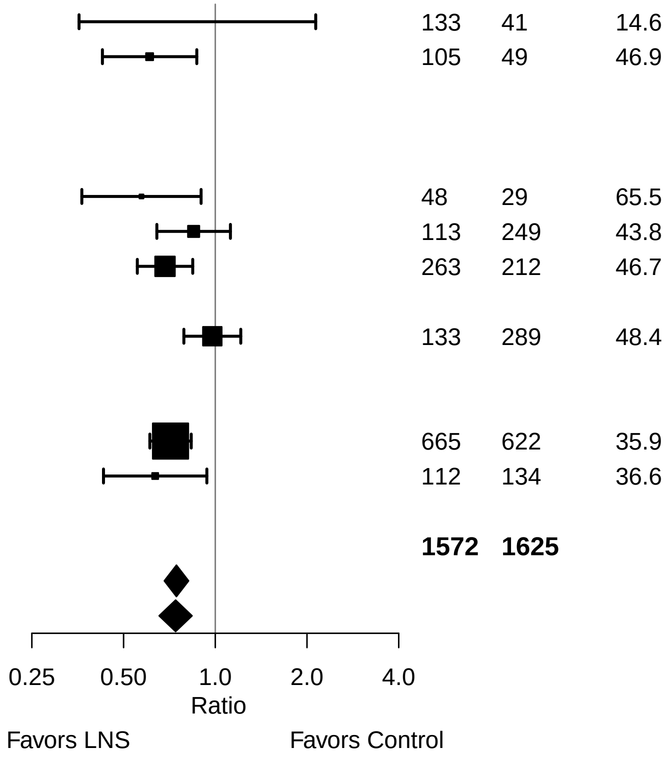

Supplemental figure 8B: Anemia prevalence ratio

8B9: Stratified by Child inflammation

P−for−interaction = 0.006
Ratio of PRs = 1.30 (1.08, 1.57)

		**Not inflamed**						**High AGP or CRP**					
Country	Trial	**LNS** N	**Control** N	**Control** Prevalence	**PR** (95% CI)	**Fixed** W	**Random** W	**LNS** N	**Control** N	**Control** Prevalence	**PR** (95% CI)	**Fixed** W	**Random** W
Bangladesh	JiVitA−4 (35)	185	47	17.0	0.67 (0.27, 1.63)	0.02	0.04	269	98	14.3	0.99 (0.60, 1.62)	0.05	0.05
Bangladesh	RDNS (36)	369	168	42.3	0.57 (0.42, 0.77)	0.19	0.18	180	104	41.3	0.83 (0.61, 1.13)	0.13	0.13
Bangladesh	WASH−B (37)												
Burkina Faso	iLiNS−Zinc (38)												
Burkina Faso	PROMIS CS (39)												
Ghana	GHANA (40)												
Ghana	iLiNS−DYAD−G (41)	45	120	46.7	0.90 (0.61, 1.34)	0.11	0.13	55	82	53.7	0.81 (0.57, 1.17)	0.09	0.09
Kenya	WASH−B (42)	157	135	43.0	0.47 (0.33, 0.68)	0.13	0.15	141	123	52.8	0.87 (0.69, 1.10)	0.22	0.22
Madagascar	MAHAY (43)	44	32	43.8	0.42 (0.20, 0.87)	0.03	0.05	39	19	47.4	0.60 (0.31, 1.13)	0.03	0.03
Malawi	iLiNS−DYAD−M (44)	59	123	40.7	0.58 (0.35, 0.97)	0.07	0.09	138	269	57.6	0.93 (0.77, 1.12)	0.35	0.35
Malawi	iLiNS−DOSE (45)												
Mali	PROMIS CS (46)												
Zimbabwe	SHINE (HIV−) (47)	414	354	28.8	0.84 (0.66, 1.06)	0.31	0.22	105	99	43.4	0.72 (0.52, 1.01)	0.11	0.11
Zimbabwe	SHINE (HIV+) (48)	186	158	35.4	0.56 (0.39, 0.81)	0.13	0.14	32	31	41.9	0.67 (0.32, 1.39)	0.02	0.02
		**1459**	**1137**		**I² = 0.46, Tau² = 0.02**			**959**	**825**		**I² = 0.00, Tau² = 0.00**		
**Fixed**					**0.66 (0.58, 0.75)**						**0.85 (0.77, 0.95)**		
**Random**					**0.64 (0.53, 0.76)**						**0.85 (0.77, 0.95)**		

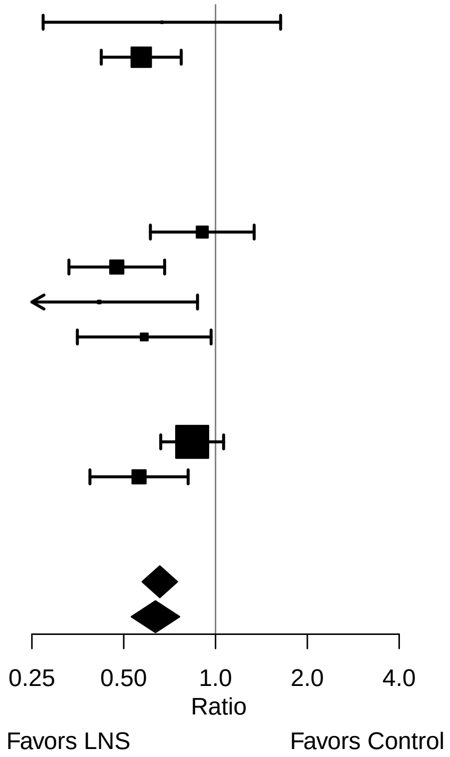

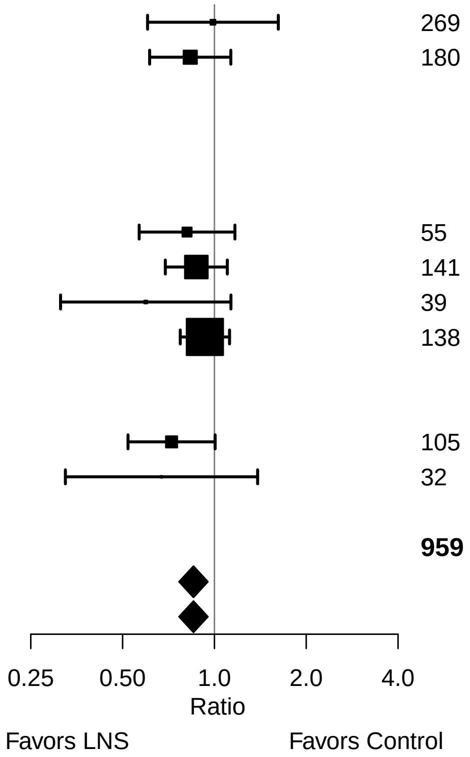

Supplemental figure 8C: Anemia prevalence difference

8C1: Stratified by Maternal BMI

P−for−interaction = 0.397
Difference in PDs = 0.01 (−0.02, 0.05)

		At least 20 kg/m²						Less than 20 kg/m²					
Country	Trial	LNS N	Control N	Control Prevalence	PD (95% CI)	Fixed W	Random W	LNS N	Control N	Control Prevalence	PD (95% CI)	Fixed W	Random W
Bangladesh	JiVitA−4 (35)												
Bangladesh	RDNS (36)	227	102	42.2	−0.13 (−0.25, 0.00)	0.03	0.04	300	160	41.9	−0.16 (−0.27, −0.05)	0.07	0.09
Bangladesh	WASH−B (37)	102	102	17.6	−0.12 (−0.21, −0.03)	0.05	0.07	132	84	14.3	−0.07 (−0.15, 0.02)	0.12	0.13
Burkina Faso	iLiNS−Zinc (38)	1217	390	91.5	−0.12 (−0.16, −0.08)	0.23	0.17	738	274	90.5	−0.11 (−0.17, −0.06)	0.26	0.19
Burkina Faso	PROMIS CS (39)	335	349	70.2	−0.02 (−0.09, 0.05)	0.07	0.09	239	227	70.0	−0.08 (−0.19, 0.02)	0.08	0.09
Ghana	GHANA (40)												
Ghana	iLiNS−DYAD−G (41)	288	536	42.9	−0.06 (−0.13, 0.01)	0.08	0.10	35	114	51.8	−0.03 (−0.22, 0.16)	0.02	0.03
Kenya	WASH−B (42)	266	227	48.9	−0.17 (−0.26, −0.09)	0.06	0.08	72	60	36.7	−0.03 (−0.17, 0.10)	0.05	0.06
Madagascar	MAHAY (43)												
Malawi	iLiNS−DYAD−M (44)	124	258	51.2	−0.07 (−0.18, 0.04)	0.03	0.05	85	171	53.8	−0.04 (−0.17, 0.09)	0.05	0.06
Malawi	iLiNS−DOSE (45)	179	52	75.0	−0.18 (−0.32, −0.03)	0.02	0.03	61	29	69.0	0.04 (−0.16, 0.23)	0.02	0.03
Mali	PROMIS CS (46)	702	685	86.6	−0.16 (−0.22, −0.10)	0.12	0.13	246	279	85.7	−0.16 (−0.23, −0.09)	0.18	0.16
Zimbabwe	SHINE (HIV−) (47)	1222	1161	34.7	−0.08 (−0.12, −0.04)	0.26	0.18	213	220	33.2	−0.02 (−0.11, 0.06)	0.12	0.12
Zimbabwe	SHINE (HIV+) (48)	221	222	36.9	−0.12 (−0.22, −0.02)	0.04	0.06	58	40	40.0	−0.23 (−0.40, −0.05)	0.03	0.04
		**4883**	**4084**		**I² = 0.38, Tau² = 0.00**			**2179**	**1658**		**I² = 0.28, Tau² = 0.00**		
**Fixed**					**−0.11 (−0.13, −0.09)**						**−0.10 (−0.13, −0.07)**		
**Random**					**−0.11 (−0.13, −0.08)**						**−0.09 (−0.13, −0.05)**		

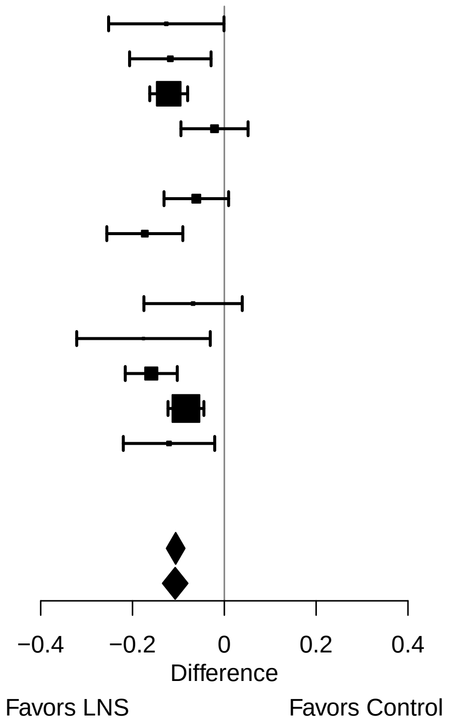

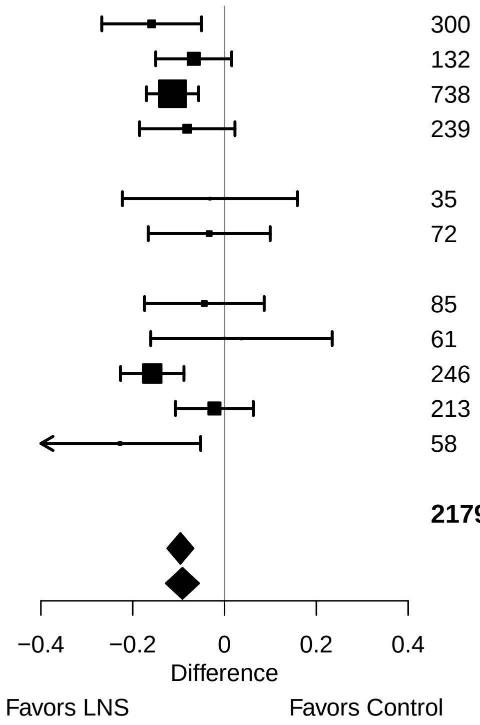

Supplemental figure 8C: Anemia prevalence difference

8C2: Stratified by Maternal age

P−for−interaction = 0.035

Difference in PDs = 0.03 (0.00, 0.06)

At least 25 y

Country	Trial	LNS N	Control N	Control Prevalence	PD (95% CI)	Fixed W	Random W
Bangladesh	JiVitA−4 (35)	203	72	19.4	−0.07 (−0.17, 0.03)	0.04	0.06
Bangladesh	RDNS (36)	152	81	40.7	−0.16 (−0.31, −0.02)	0.02	0.04
Bangladesh	WASH−B (37)	89	83	14.5	−0.09 (−0.19, 0.01)	0.04	0.06
Burkina Faso	iLiNS−Zinc (38)	1132	399	91.7	−0.13 (−0.17, −0.09)	0.24	0.13
Burkina Faso	PROMIS CS (39)	271	263	67.3	−0.07 (−0.16, 0.02)	0.05	0.07
Ghana	GHANA (40)	69	63	57.1	−0.33 (−0.48, −0.17)	0.02	0.03
Ghana	iLiNS−DYAD−G (41)	206	397	42.3	−0.08 (−0.17, 0.00)	0.06	0.08
Kenya	WASH−B (42)	213	168	45.2	−0.13 (−0.21, −0.05)	0.07	0.08
Madagascar	MAHAY (43)	301	329	62.9	−0.05 (−0.15, 0.05)	0.04	0.06
Malawi	iLiNS−DYAD−M (44)	99	220	47.3	−0.02 (−0.14, 0.10)	0.03	0.05
Malawi	iLiNS−DOSE (45)	135	50	72.0	−0.11 (−0.26, 0.04)	0.02	0.04
Mali	PROMIS CS (46)	502	508	87.8	−0.18 (−0.25, −0.12)	0.11	0.10
Zimbabwe	SHINE (HIV−) (47)	808	726	33.7	−0.10 (−0.14, −0.05)	0.21	0.13
Zimbabwe	SHINE (HIV+) (48)	221	204	37.7	−0.16 (−0.26, −0.07)	0.04	0.06
		**4401**	**3563**		**I^2 = 0.37, Tau^2 = 0.00**		
**Fixed**					**−0.12 (−0.14, −0.10)**		
**Random**					**−0.12 (−0.15, −0.08)**		

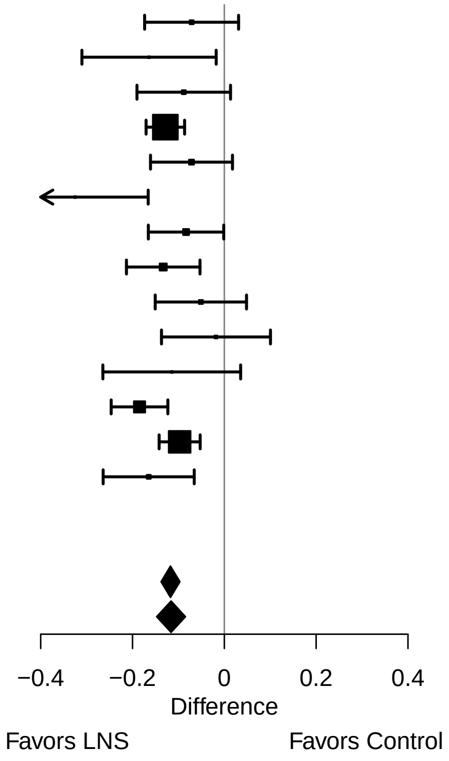

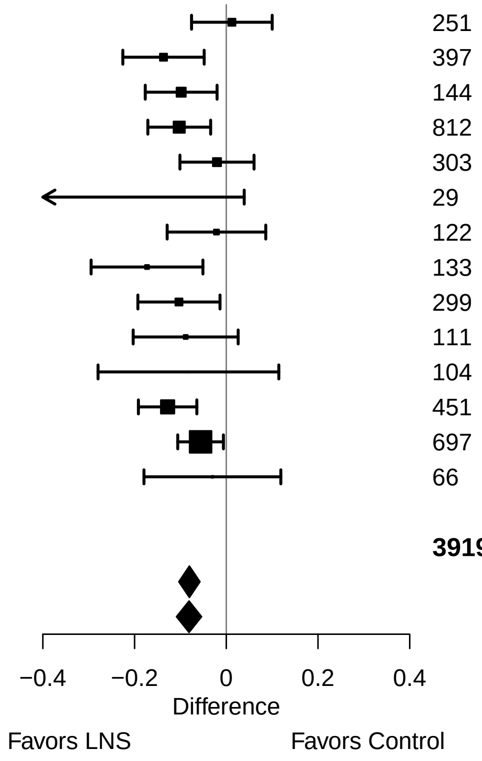

Less than 25 y

LNS N	Control N	Control Prevalence	PD (95% CI)	Fixed W	Random W
251	73	12.3	0.01 (−0.08, 0.10)	0.07	0.08
397	191	42.4	−0.14 (−0.23, −0.05)	0.07	0.07
144	103	17.5	−0.10 (−0.18, −0.02)	0.09	0.09
812	262	90.1	−0.10 (−0.17, −0.03)	0.11	0.11
303	318	72.3	−0.02 (−0.10, 0.06)	0.08	0.09
29	25	64.0	−0.23 (−0.49, 0.04)	0.01	0.01
122	264	48.9	−0.02 (−0.13, 0.09)	0.05	0.05
133	131	49.6	−0.17 (−0.29, −0.05)	0.04	0.04
299	259	67.2	−0.10 (−0.19, −0.01)	0.07	0.07
111	212	56.6	−0.09 (−0.20, 0.03)	0.04	0.05
104	28	71.4	−0.08 (−0.28, 0.11)	0.01	0.02
451	462	84.4	−0.13 (−0.19, −0.06)	0.13	0.12
697	694	36.3	−0.06 (−0.11, −0.01)	0.21	0.16
66	60	33.3	−0.03 (−0.18, 0.12)	0.02	0.03
**3919**	**3082**		**I^2 = 0.23, Tau^2 = 0.00**		
			**−0.08 (−0.10, −0.06)**		
			**−0.08 (−0.11, −0.05)**		

Supplemental figure 8C: Anemia prevalence difference

8C3: Stratified by Maternal education

P−for−interaction = 0.307
Difference in PDs = 0.02 (−0.02, 0.07)

		Primary or greater						Incomplete or no formal					
Country	Trial	LNS N	Control N	Control Prevalence	PD (95% CI)	Fixed W	Random W	LNS N	Control N	Control Prevalence	PD (95% CI)	Fixed W	Random W
Bangladesh	JiVitA−4 (35)	299	93	17.2	−0.06 (−0.16, 0.04)	0.06	0.10	157	53	13.2	0.04 (−0.05, 0.13)	0.10	0.11
Bangladesh	RDNS (36)	405	195	41.0	−0.16 (−0.26, −0.05)	0.05	0.09	144	77	44.2	−0.11 (−0.27, 0.05)	0.03	0.06
Bangladesh	WASH−B (37)	163	145	15.9	−0.10 (−0.17, −0.03)	0.12	0.14	71	41	17.1	−0.09 (−0.21, 0.04)	0.05	0.08
Burkina Faso	iLiNS−Zinc (38)												
Burkina Faso	PROMIS CS (39)	46	47	70.2	0.04 (−0.14, 0.21)	0.02	0.05	526	532	69.9	−0.05 (−0.12, 0.02)	0.19	0.14
Ghana	GHANA (40)												
Ghana	iLiNS−DYAD−G (41)	253	526	45.4	−0.08 (−0.15, 0.00)	0.10	0.13	75	135	43.0	0.00 (−0.14, 0.14)	0.04	0.07
Kenya	WASH−B (42)	168	136	50.0	−0.27 (−0.37, −0.17)	0.06	0.10	181	164	45.1	−0.04 (−0.14, 0.06)	0.09	0.10
Madagascar	MAHAY (43)	128	143	64.3	−0.14 (−0.27, 0.00)	0.03	0.07	472	445	64.9	−0.06 (−0.15, 0.03)	0.11	0.11
Malawi	iLiNS−DYAD−M (44)	34	66	48.5	−0.10 (−0.31, 0.10)	0.01	0.04	175	363	52.9	−0.05 (−0.14, 0.04)	0.11	0.11
Malawi	iLiNS−DOSE (45)												
Mali	PROMIS CS (46)	107	96	84.4	−0.09 (−0.20, 0.02)	0.05	0.09	845	874	86.4	−0.17 (−0.22, −0.11)	0.26	0.15
Zimbabwe	SHINE (HIV−) (47)	1536	1462	34.9	−0.07 (−0.10, −0.04)	0.50	0.19	58	53	39.6	−0.26 (−0.42, −0.10)	0.03	0.06
Zimbabwe	SHINE (HIV+) (48)												
		**3139**	**2909**		**I² = 0.49, Tau² = 0.00**			**2704**	**2737**		**I² = 0.60, Tau² = 0.00**		
**Fixed**					**−0.09 (−0.11, −0.07)**						**−0.08 (−0.11, −0.05)**		
**Random**					**−0.10 (−0.15, −0.06)**						**−0.07 (−0.12, −0.02)**		

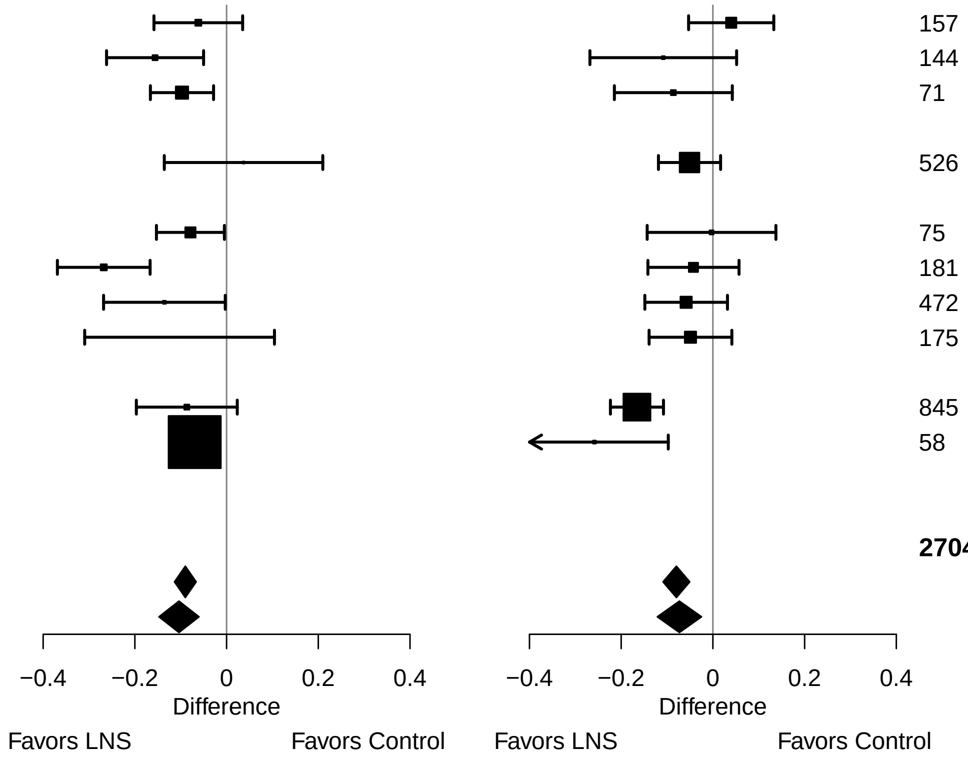

Supplemental figure 8C: Anemia prevalence difference

8C4: Stratified by Child sex

P−for−interaction = 0.283
Difference in PDs = −0.02 (−0.04, 0.01)

Country	Trial	Male LNS N	Male Control N	Male Control Prevalence	Male PD (95% CI)	Male Fixed W	Male Random W	Female LNS N	Female Control N	Female Control Prevalence	Female PD (95% CI)	Female Fixed W	Female Random W
Bangladesh	JiVitA−4 (35)	235	71	22.5	−0.12 (−0.22, −0.02)	0.04	0.06	222	75	9.3	0.06 (−0.01, 0.13)	0.09	0.09
Bangladesh	RDNS (36)	266	131	41.2	−0.16 (−0.29, −0.02)	0.02	0.04	281	141	42.6	−0.14 (−0.24, −0.03)	0.04	0.06
Bangladesh	WASH−B (37)	116	89	16.9	−0.12 (−0.22, −0.02)	0.04	0.06	118	97	15.5	−0.07 (−0.16, 0.02)	0.06	0.08
Burkina Faso	iLiNS−Zinc (38)	993	334	91.6	−0.11 (−0.14, −0.07)	0.32	0.15	964	330	90.6	−0.13 (−0.19, −0.07)	0.13	0.09
Burkina Faso	PROMIS CS (39)	317	295	77.3	−0.05 (−0.14, 0.04)	0.05	0.08	257	286	62.6	−0.05 (−0.13, 0.03)	0.07	0.08
Ghana	GHANA (40)	60	41	61.0	−0.33 (−0.51, −0.14)	0.01	0.02	38	55	56.4	−0.25 (−0.45, −0.05)	0.01	0.03
Ghana	iLiNS−DYAD−G (41)	164	310	44.8	−0.02 (−0.11, 0.08)	0.05	0.07	163	348	44.8	−0.11 (−0.20, −0.02)	0.06	0.07
Kenya	WASH−B (42)	168	145	44.8	−0.09 (−0.19, 0.02)	0.04	0.06	182	155	49.7	−0.21 (−0.30, −0.12)	0.06	0.08
Madagascar	MAHAY (43)	277	288	65.6	−0.03 (−0.12, 0.06)	0.05	0.07	323	300	64.0	−0.11 (−0.21, −0.01)	0.05	0.07
Malawi	iLiNS−DYAD−M (44)	103	202	56.4	−0.03 (−0.15, 0.09)	0.03	0.05	107	230	47.8	−0.08 (−0.19, 0.04)	0.04	0.06
Malawi	iLiNS−DOSE (45)	123	43	76.7	−0.12 (−0.28, 0.04)	0.02	0.03	120	39	66.7	−0.07 (−0.24, 0.10)	0.02	0.04
Mali	PROMIS CS (46)	479	526	89.0	−0.14 (−0.20, −0.08)	0.11	0.11	474	444	82.9	−0.17 (−0.24, −0.10)	0.09	0.09
Zimbabwe	SHINE (HIV−) (47)	830	814	36.2	−0.07 (−0.12, −0.02)	0.17	0.13	852	780	34.2	−0.09 (−0.13, −0.05)	0.24	0.11
Zimbabwe	SHINE (HIV+) (48)	146	149	40.9	−0.16 (−0.25, −0.06)	0.04	0.07	160	136	32.4	−0.10 (−0.20, 0.01)	0.04	0.06
		**4277**	**3438**		**I² = 0.32, Tau² = 0.00**			**4261**	**3416**		**I² = 0.65, Tau² = 0.00**		
**Fixed**					**−0.10 (−0.12, −0.08)**						**−0.10 (−0.12, −0.08)**		
**Random**					**−0.10 (−0.13, −0.07)**						**−0.10 (−0.14, −0.06)**		

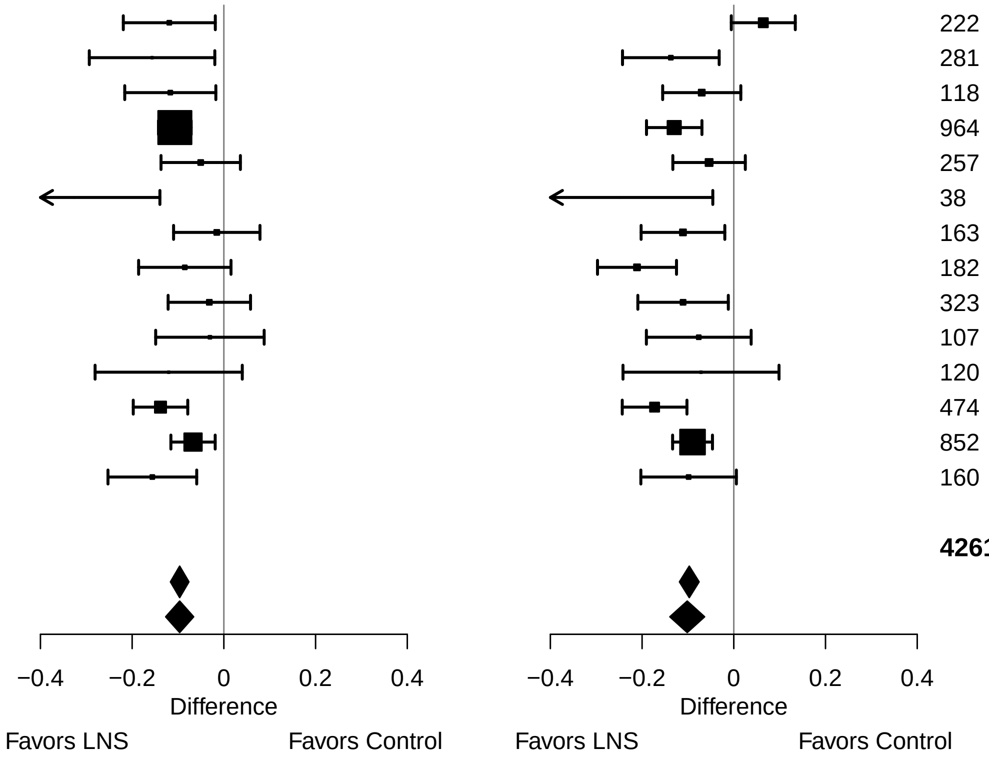

Supplemental figure 8C: Anemia prevalence difference

8C5: Stratified by Child birth order

P−for−interaction = 0.024
Difference in PDs = 0.04 (0.01, 0.07)

		Later born						Firstborn					
Country	Trial	LNS N	Control N	Control Prevalence	PD (95% CI)	Fixed W	Random W	LNS N	Control N	Control Prevalence	PD (95% CI)	Fixed W	Random W
Bangladesh	JiVitA−4 (35)	115	33	27.3	−0.12 (−0.29, 0.04)	0.01	0.03	341	112	12.5	0.00 (−0.06, 0.06)	0.20	0.14
Bangladesh	RDNS (36)	339	171	40.9	−0.14 (−0.24, −0.04)	0.03	0.06	210	101	43.6	−0.15 (−0.28, −0.02)	0.04	0.07
Bangladesh	WASH−B (37)	137	123	16.3	−0.11 (−0.20, −0.03)	0.05	0.07	95	61	16.4	−0.08 (−0.20, 0.04)	0.05	0.07
Burkina Faso	iLiNS−Zinc (38)	1522	520	90.4	−0.12 (−0.16, −0.07)	0.15	0.12	435	144	93.8	−0.13 (−0.18, −0.08)	0.29	0.16
Burkina Faso	PROMIS CS (39)	498	477	70.0	−0.05 (−0.12, 0.02)	0.07	0.09	75	103	69.9	−0.01 (−0.16, 0.15)	0.03	0.05
Ghana	GHANA (40)	60	54	61.1	−0.38 (−0.55, −0.21)	0.01	0.03	38	38	55.3	−0.16 (−0.38, 0.07)	0.01	0.03
Ghana	iLiNS−DYAD−G (41)	220	435	45.3	−0.11 (−0.19, −0.03)	0.06	0.08	108	226	44.2	0.03 (−0.08, 0.14)	0.05	0.08
Kenya	WASH−B (42)	282	244	47.1	−0.16 (−0.24, −0.08)	0.05	0.08	67	56	48.2	−0.12 (−0.32, 0.08)	0.02	0.03
Madagascar	MAHAY (43)	458	454	66.1	−0.07 (−0.15, 0.02)	0.05	0.07	141	131	61.1	−0.10 (−0.23, 0.03)	0.04	0.06
Malawi	iLiNS−DYAD−M (44)	165	342	50.9	−0.09 (−0.18, 0.00)	0.04	0.07	45	88	55.7	0.09 (−0.09, 0.27)	0.02	0.04
Malawi	iLiNS−DOSE (45)												
Mali	PROMIS CS (46)	790	832	86.1	−0.16 (−0.21, −0.10)	0.12	0.11	146	126	88.1	−0.16 (−0.26, −0.06)	0.07	0.09
Zimbabwe	SHINE (HIV−) (47)	1188	1100	35.5	−0.10 (−0.14, −0.07)	0.31	0.14	418	416	33.2	−0.02 (−0.09, 0.04)	0.16	0.13
Zimbabwe	SHINE (HIV+) (48)	252	216	36.6	−0.14 (−0.22, −0.05)	0.05	0.07	51	64	37.5	−0.08 (−0.26, 0.09)	0.02	0.04
		**6026**	**5001**		**I² = 0.35, Tau² = 0.00**			**2170**	**1666**		**I² = 0.52, Tau² = 0.00**		
**Fixed**					**−0.12 (−0.13, −0.10)**						**−0.07 (−0.09, −0.04)**		
**Random**					**−0.12 (−0.15, −0.09)**						**−0.07 (−0.11, −0.03)**		

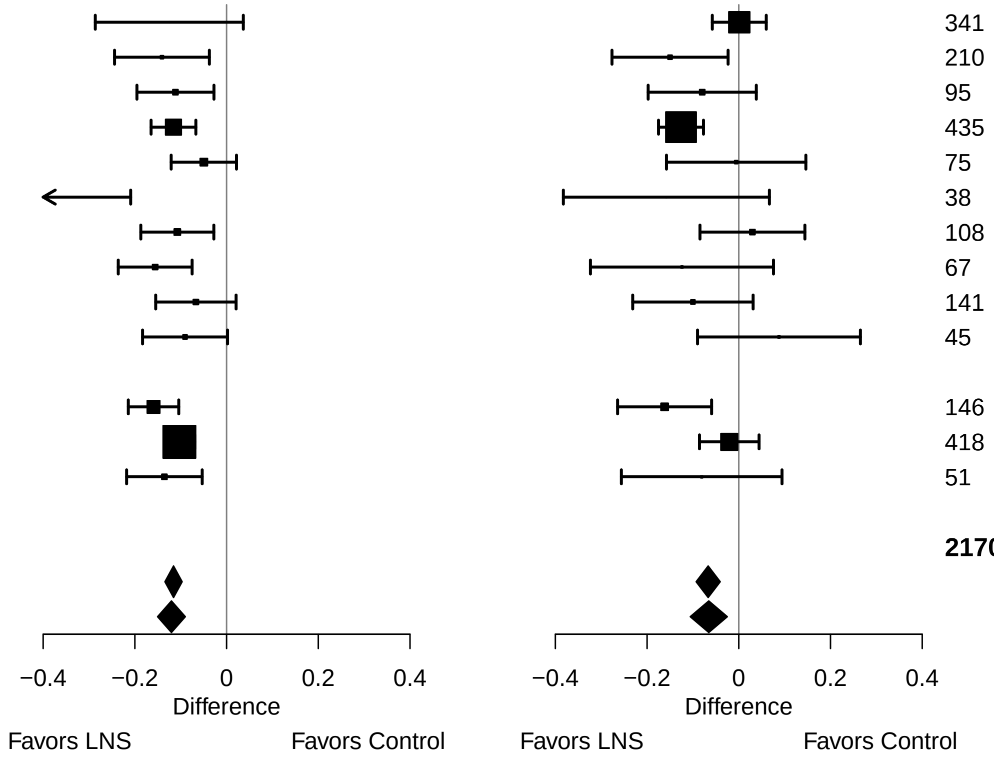

Supplemental figure 8C: Anemia prevalence difference

8C6: Stratified by Child baseline acute malnutrition

P−for−interaction = 0.595
Difference in PDs = −0.01 (−0.06, 0.04)

		No						Yes					
		**LNS**	**Control**	**Control**	**PD**	**Fixed**	**Random**	**LNS**	**Control**	**Control**	**PD**	**Fixed**	**Random**
Country	Trial	N	N	Prevalence	(95% CI)	W	W	N	N	Prevalence	(95% CI)	W	W
Bangladesh	JiVitA−4 (35)	365	114	15.8	−0.03 (−0.09, 0.03)	0.13	0.15	90	32	15.6	0.00 (−0.14, 0.14)	0.11	0.18
Bangladesh	RDNS (36)	493	243	40.3	−0.14 (−0.23, −0.05)	0.06	0.08	35	12	41.7	−0.07 (−0.45, 0.31)	0.01	0.03
Bangladesh	WASH−B (37)												
Burkina Faso	iLiNS−Zinc (38)	1457	487	90.3	−0.11 (−0.16, −0.07)	0.24	0.22	500	177	93.2	−0.14 (−0.19, −0.08)	0.72	0.47
Burkina Faso	PROMIS CS (39)												
Ghana	GHANA (40)												
Ghana	iLiNS−DYAD−G (41)	285	556	46.4	−0.07 (−0.15, 0.00)	0.10	0.12	21	54	31.5	0.02 (−0.22, 0.26)	0.04	0.08
Kenya	WASH−B (42)												
Madagascar	MAHAY (43)												
Malawi	iLiNS−DYAD−M (44)	183	379	51.5	−0.05 (−0.14, 0.04)	0.06	0.08	14	37	51.4	0.06 (−0.25, 0.37)	0.02	0.05
Malawi	iLiNS−DOSE (45)												
Mali	PROMIS CS (46)												
Zimbabwe	SHINE (HIV−) (47)	1185	1069	32.8	−0.07 (−0.10, −0.03)	0.34	0.26	66	67	32.8	−0.09 (−0.25, 0.07)	0.08	0.15
Zimbabwe	SHINE (HIV+) (48)	226	202	34.7	−0.13 (−0.21, −0.05)	0.07	0.09	26	13	61.5	−0.38 (−0.73, −0.04)	0.02	0.04
		**4194**	**3050**		**I² = 0.28, Tau² = 0.00**			**752**	**392**		**I² = 0.24, Tau² = 0.00**		
**Fixed**					**−0.08 (−0.10, −0.06)**						**−0.11 (−0.16, −0.06)**		
**Random**					**−0.08 (−0.11, −0.05)**						**−0.09 (−0.16, −0.02)**		

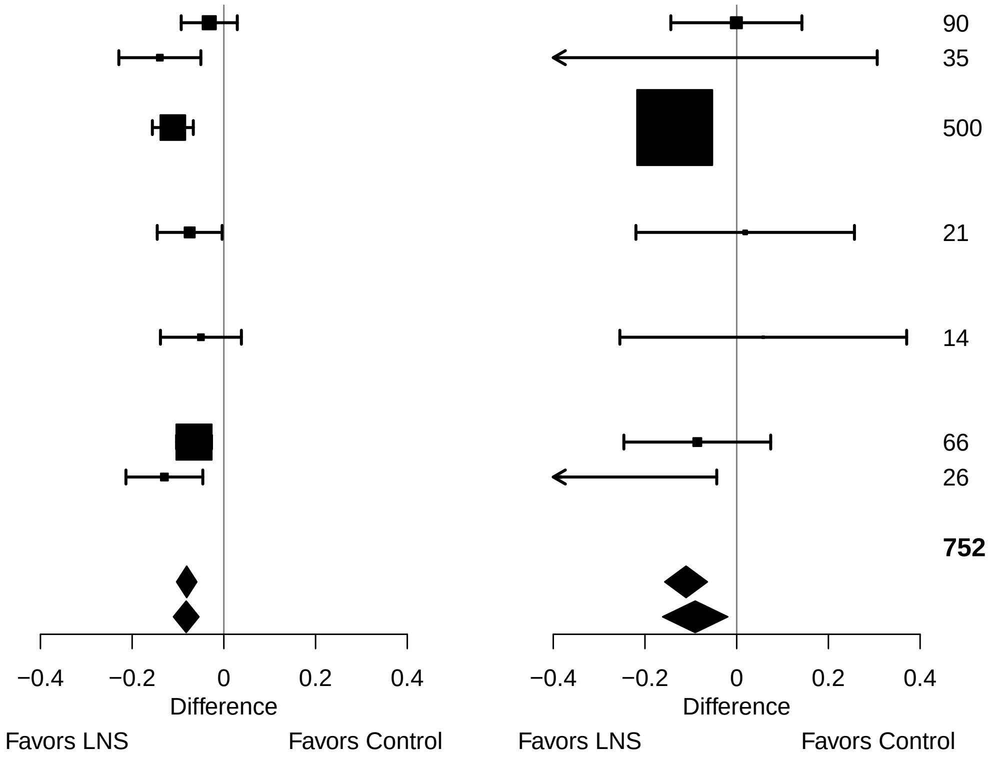

Supplemental figure 8C: Anemia prevalence difference

8C7: Stratified by Child baseline anemia

P−for−interaction = 0.308
Difference in PDs = −0.04 (−0.10, 0.03)

Country	Trial	Not anemic LNS N	Not anemic Control N	Not anemic Control Prevalence	Not anemic PD (95% CI)	Not anemic Fixed W	Not anemic Random W	Anemic LNS N	Anemic Control N	Anemic Control Prevalence	Anemic PD (95% CI)	Anemic Fixed W	Anemic Random W
Bangladesh	JiVitA−4 (35)												
Bangladesh	RDNS (36)	213	93	23.7	−0.09 (−0.21, 0.02)	0.20	0.21	314	160	50.0	−0.15 (−0.25, −0.04)	0.11	0.11
Bangladesh	WASH−B (37)												
Burkina Faso	iLiNS−Zinc (38)	177	61	82.0	−0.17 (−0.28, −0.06)	0.21	0.22	1780	603	92.0	−0.11 (−0.16, −0.07)	0.62	0.62
Burkina Faso	PROMIS CS (39)												
Ghana	GHANA (40)												
Ghana	iLiNS−DYAD−G (41)	189	355	32.7	0.00 (−0.08, 0.08)	0.38	0.29	90	207	64.7	−0.13 (−0.25, 0.00)	0.09	0.09
Kenya	WASH−B (42)												
Madagascar	MAHAY (43)												
Malawi	iLiNS−DYAD−M (44)	66	145	36.6	0.01 (−0.13, 0.15)	0.13	0.16	134	269	59.1	−0.07 (−0.17, 0.03)	0.13	0.13
Malawi	iLiNS−DOSE (45)	101	45	64.4	−0.10 (−0.27, 0.07)	0.09	0.12	142	37	81.1	−0.10 (−0.26, 0.06)	0.05	0.05
Mali	PROMIS CS (46)												
Zimbabwe	SHINE (HIV−) (47)												
Zimbabwe	SHINE (HIV+) (48)												
		**746**	**699**		**I² = 0.44, Tau² = 0.00**			**2460**	**1276**		**I² = 0.00, Tau² = 0.00**		
**Fixed**					**−0.06 (−0.11, −0.01)**						**−0.11 (−0.15, −0.08)**		
**Random**					**−0.06 (−0.13, 0.00)**						**−0.11 (−0.15, −0.08)**		

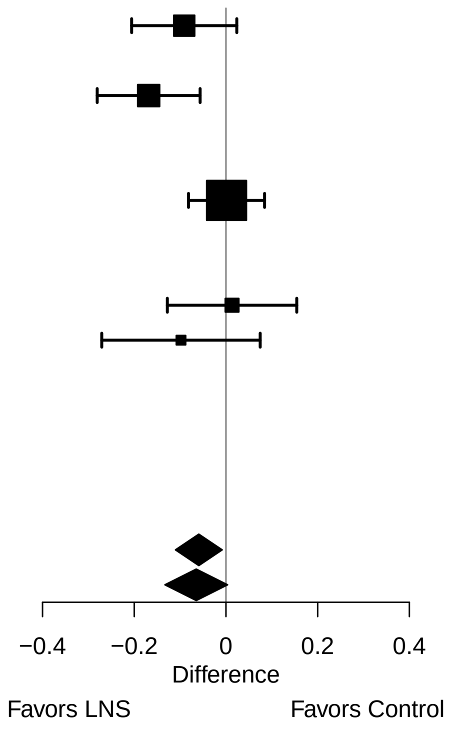

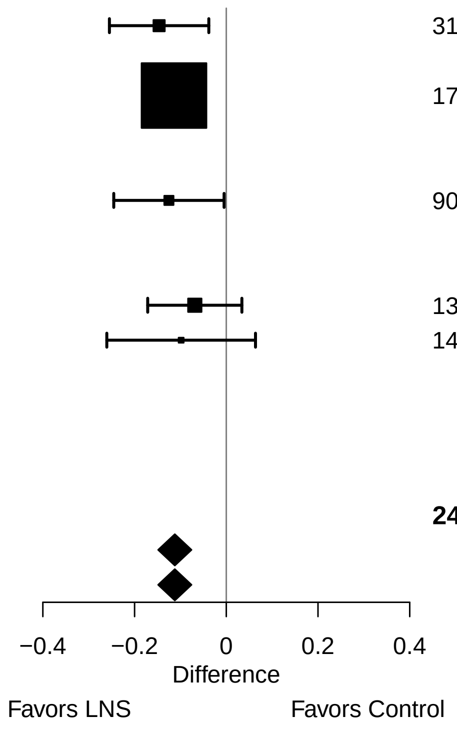

Supplemental figure 8C: Anemia prevalence difference

8C8: Stratified by Child high-dose vitamin A supplementation

P−for−interaction = 0.549
Difference in PDs = −0.01 (−0.06, 0.03)

Received VitA

Country	Trial	**LNS** N	**Control** N	**Control** Prevalence	**PD** (95% CI)	**Fixed** W	**Random** W
Bangladesh	JiVitA−4 (35)	141	37	16.2	−0.01 (−0.16, 0.14)	0.05	0.10
Bangladesh	RDNS (36)	444	223	40.8	−0.14 (−0.24, −0.03)	0.10	0.14
Bangladesh	WASH−B (37)						
Burkina Faso	iLiNS−Zinc (38)						
Burkina Faso	PROMIS CS (39)						
Ghana	GHANA (40)	50	62	56.5	−0.34 (−0.52, −0.17)	0.03	0.08
Ghana	iLiNS−DYAD−G (41)	215	412	45.6	−0.06 (−0.14, 0.02)	0.15	0.17
Kenya	WASH−B (42)	29	34	52.9	−0.18 (−0.43, 0.06)	0.02	0.05
Madagascar	MAHAY (43)						
Malawi	iLiNS−DYAD−M (44)	75	136	58.8	−0.13 (−0.27, 0.01)	0.05	0.11
Malawi	iLiNS−DOSE (45)						
Mali	PROMIS CS (46)						
Zimbabwe	SHINE (HIV−) (47)	874	833	33.7	−0.06 (−0.10, −0.01)	0.51	0.20
Zimbabwe	SHINE (HIV+) (48)	165	127	37.0	−0.15 (−0.26, −0.05)	0.09	0.14
		**1993**	**1864**		**I² = 0.54, Tau² = 0.00**		
**Fixed**					**−0.09 (−0.12, −0.06)**		
**Random**					**−0.12 (−0.18, −0.05)**		

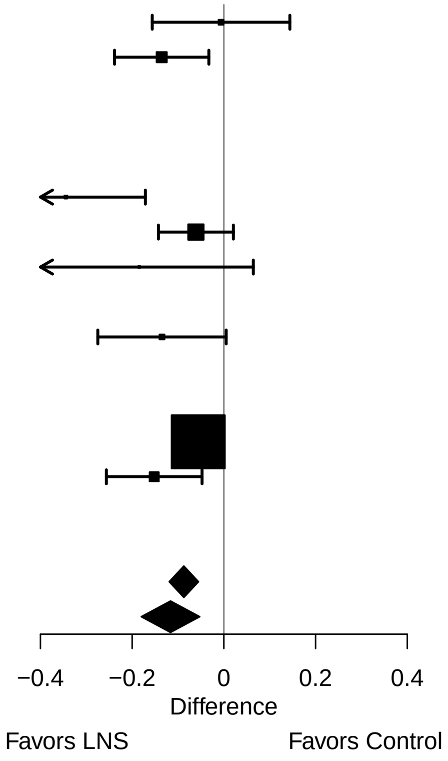

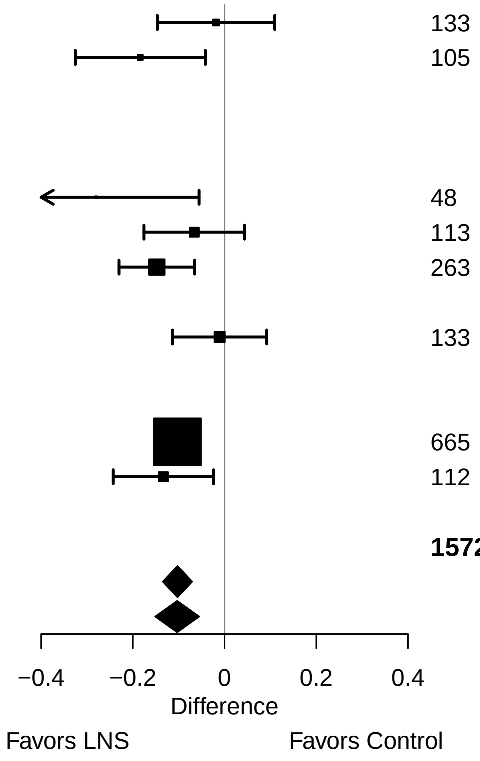

Not treated

Country	Trial	**LNS** N	**Control** N	**Control** Prevalence	**PD** (95% CI)	**Fixed** W	**Random** W
Bangladesh	JiVitA−4 (35)	133	41	14.6	−0.02 (−0.15, 0.11)	0.06	0.10
Bangladesh	RDNS (36)	105	49	46.9	−0.18 (−0.33, −0.04)	0.05	0.08
Bangladesh	WASH−B (37)						
Burkina Faso	iLiNS−Zinc (38)						
Burkina Faso	PROMIS CS (39)						
Ghana	GHANA (40)	48	29	65.5	−0.28 (−0.50, −0.06)	0.02	0.04
Ghana	iLiNS−DYAD−G (41)	113	249	43.8	−0.07 (−0.18, 0.04)	0.09	0.12
Kenya	WASH−B (42)	263	212	46.7	−0.15 (−0.23, −0.07)	0.15	0.16
Madagascar	MAHAY (43)						
Malawi	iLiNS−DYAD−M (44)	133	289	48.4	−0.01 (−0.11, 0.09)	0.10	0.13
Malawi	iLiNS−DOSE (45)						
Mali	PROMIS CS (46)						
Zimbabwe	SHINE (HIV−) (47)	665	622	35.9	−0.10 (−0.15, −0.05)	0.45	0.24
Zimbabwe	SHINE (HIV+) (48)	112	134	36.6	−0.13 (−0.24, −0.02)	0.09	0.12
		**1572**	**1625**		**I² = 0.32, Tau² = 0.00**		
**Fixed**					**−0.10 (−0.13, −0.07)**		
**Random**					**−0.10 (−0.15, −0.06)**		

Supplemental figure 8C: Anemia prevalence difference

8C9: Stratified by Child inflammation

P−for−interaction = 0.068
Difference in PDs = 0.06 (0.00, 0.12)

		Not inflamed						High AGP or CRP					
Country	Trial	LNS N	Control N	Control Prevalence	PD (95% CI)	Fixed W	Random W	LNS N	Control N	Control Prevalence	PD (95% CI)	Fixed W	Random W
Bangladesh	JiVitA−4 (35)	185	47	17.0	−0.06 (−0.20, 0.09)	0.07	0.10	269	98	14.3	0.00 (−0.07, 0.07)	0.35	0.35
Bangladesh	RDNS (36)	369	168	42.3	−0.18 (−0.29, −0.07)	0.12	0.14	180	104	41.3	−0.07 (−0.19, 0.05)	0.12	0.12
Bangladesh	WASH−B (37)												
Burkina Faso	iLiNS−Zinc (38)												
Burkina Faso	PROMIS CS (39)												
Ghana	GHANA (40)												
Ghana	iLiNS−DYAD−G (41)	45	120	46.7	−0.04 (−0.22, 0.13)	0.05	0.08	55	82	53.7	−0.10 (−0.27, 0.07)	0.06	0.06
Kenya	WASH−B (42)	157	135	43.0	−0.23 (−0.33, −0.12)	0.13	0.15	141	123	52.8	−0.07 (−0.19, 0.05)	0.12	0.12
Madagascar	MAHAY (43)	44	32	43.8	−0.26 (−0.44, −0.07)	0.04	0.07	39	19	47.4	−0.19 (−0.44, 0.06)	0.03	0.03
Malawi	iLiNS−DYAD−M (44)	59	123	40.7	−0.17 (−0.32, −0.02)	0.07	0.10	138	269	57.6	−0.04 (−0.14, 0.06)	0.17	0.17
Malawi	iLiNS−DOSE (45)												
Mali	PROMIS CS (46)												
Zimbabwe	SHINE (HIV−) (47)	414	354	28.8	−0.05 (−0.11, 0.02)	0.35	0.21	105	99	43.4	−0.12 (−0.24, 0.00)	0.12	0.12
Zimbabwe	SHINE (HIV+) (48)	186	158	35.4	−0.16 (−0.25, −0.06)	0.17	0.16	32	31	41.9	−0.14 (−0.38, 0.11)	0.03	0.03
		**1459**	**1137**		**I² = 0.53, Tau² = 0.00**			**959**	**825**		**I² = 0.00, Tau² = 0.00**		
**Fixed**					**−0.12 (−0.16, −0.08)**						**−0.05 (−0.10, −0.01)**		
**Random**					**−0.14 (−0.19, −0.08)**						**−0.05 (−0.10, −0.01)**		

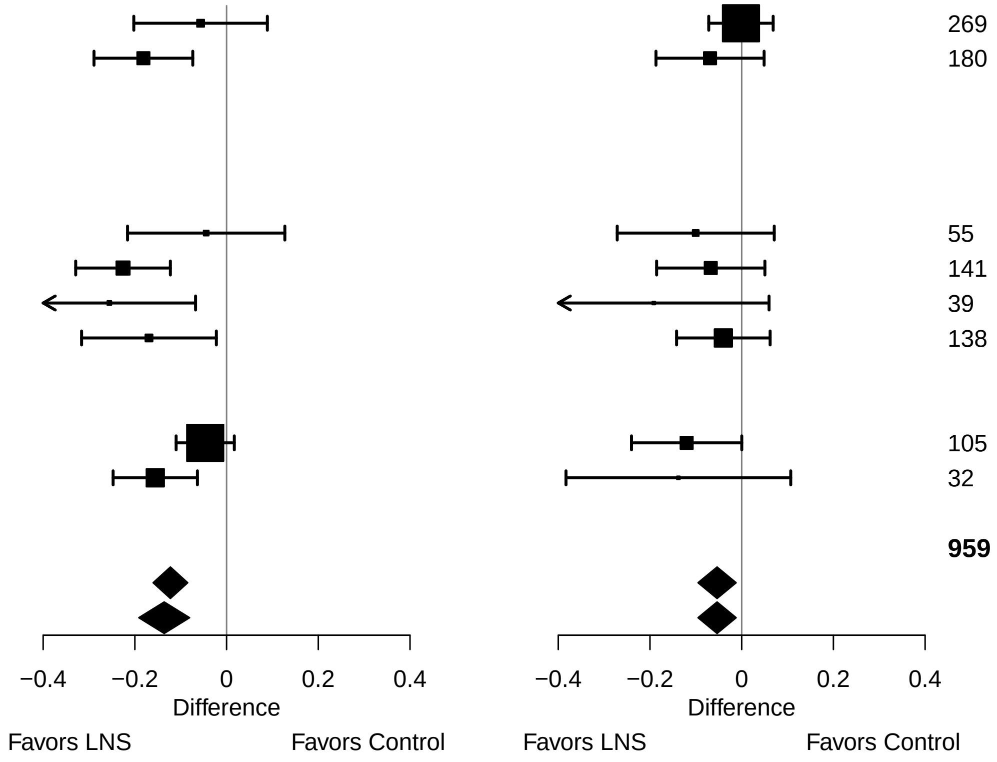

Supplemental figure 8D: Moderate-to-severe anemia prevalence ratio

8D1: Stratified by Maternal BMI

P−for−interaction = 0.459
Ratio of PRs = 1.03 (0.96, 1.10)

		At least 20 kg/m²						Less than 20 kg/m²					
Country	Trial	LNS N	Control N	Control Prevalence	PR (95% CI)	Fixed W	Random W	LNS N	Control N	Control Prevalence	PR (95% CI)	Fixed W	Random W
Bangladesh	JiVitA−4 (35)												
Bangladesh	RDNS (36)	227	102	20.6	0.47 (0.28, 0.80)	0.02	0.07	300	160	13.8	0.61 (0.37, 0.99)	0.03	0.09
Bangladesh	WASH−B (37)												
Burkina Faso	iLiNS−Zinc (38)	1217	390	76.2	0.71 (0.65, 0.77)	0.59	0.17	738	274	73.4	0.72 (0.65, 0.81)	0.51	0.22
Burkina Faso	PROMIS CS (39)	335	349	36.7	0.96 (0.77, 1.20)	0.09	0.14	239	227	37.0	0.77 (0.57, 1.03)	0.07	0.15
Ghana	GHANA (40)												
Ghana	iLiNS−DYAD−G (41)												
Kenya	WASH−B (42)	266	227	22.9	0.54 (0.36, 0.81)	0.03	0.10	72	60	11.7	1.43 (0.67, 3.05)	0.01	0.05
Madagascar	MAHAY (43)												
Malawi	iLiNS−DYAD−M (44)	124	258	24.0	1.14 (0.80, 1.63)	0.03	0.11	85	171	23.4	1.11 (0.71, 1.74)	0.03	0.10
Malawi	iLiNS−DOSE (45)	179	52	46.2	0.60 (0.42, 0.85)	0.04	0.11	61	29	48.3	0.97 (0.62, 1.52)	0.03	0.10
Mali	PROMIS CS (46)	702	685	60.0	0.63 (0.53, 0.76)	0.14	0.16	246	279	61.3	0.63 (0.54, 0.73)	0.30	0.21
Zimbabwe	SHINE (HIV−) (47)	1222	1161	10.9	0.64 (0.50, 0.83)	0.07	0.14	213	220	6.8	1.24 (0.66, 2.31)	0.02	0.07
Zimbabwe	SHINE (HIV+) (48)												
		**4272**	**3224**		**I² = 0.66, Tau² = 0.05**			**1954**	**1420**		**I² = 0.53, Tau² = 0.04**		
**Fixed**					**0.71 (0.66, 0.76)**						**0.72 (0.67, 0.78)**		
**Random**					**0.70 (0.58, 0.85)**						**0.80 (0.67, 0.97)**		

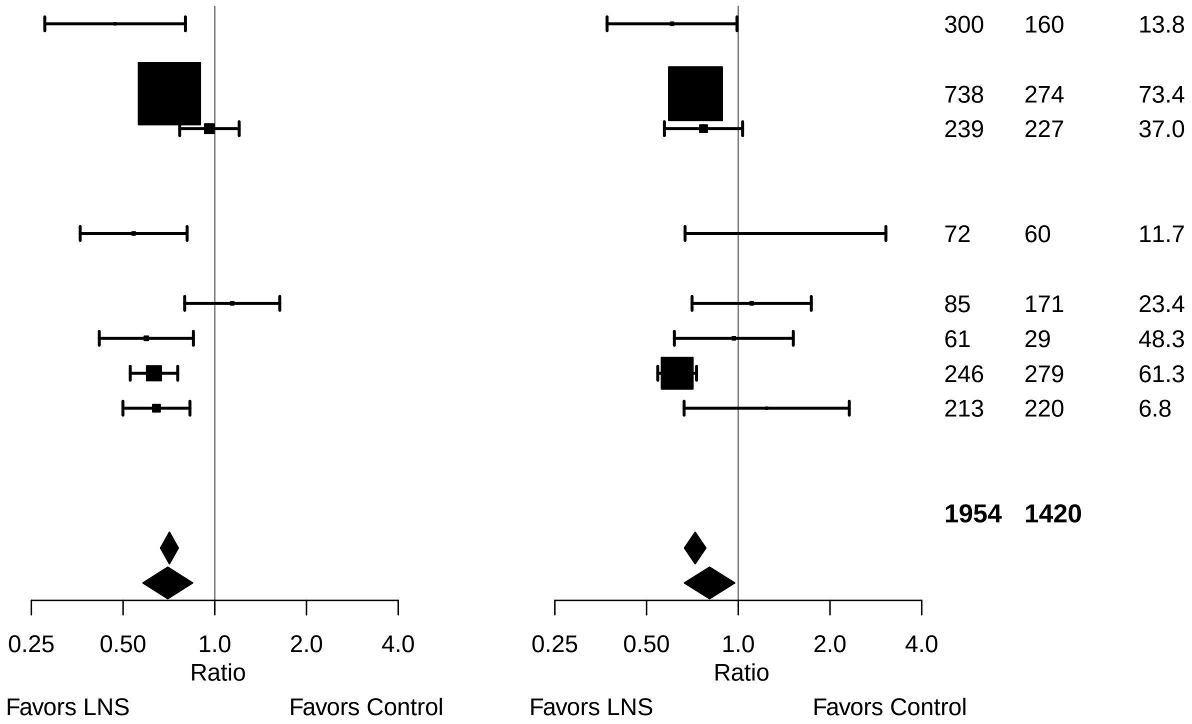

Supplemental figure 8D: Moderate-to-severe anemia prevalence ratio

8D2: Stratified by Maternal age

P−for−interaction = 0.260
Ratio of PRs = 1.06 (0.96, 1.18)

		At least 25 y						Less than 25 y					
Country	Trial	LNS N	Control N	Control Prevalence	PR (95% CI)	Fixed W	Random W	LNS N	Control N	Control Prevalence	PR (95% CI)	Fixed W	Random W
Bangladesh	JiVitA−4 (35)												
Bangladesh	RDNS (36)	152	81	17.3	0.46 (0.20, 1.04)	0.01	0.03	397	191	16.2	0.57 (0.39, 0.85)	0.04	0.04
Bangladesh	WASH−B (37)												
Burkina Faso	iLiNS−Zinc (38)	1132	399	74.2	0.69 (0.61, 0.79)	0.41	0.17	812	262	76.0	0.74 (0.67, 0.83)	0.48	0.48
Burkina Faso	PROMIS CS (39)	271	263	32.7	0.89 (0.68, 1.17)	0.09	0.12	303	318	39.9	0.88 (0.68, 1.15)	0.08	0.08
Ghana	GHANA (40)												
Ghana	iLiNS−DYAD−G (41)	206	397	5.0	0.77 (0.35, 1.72)	0.01	0.03	122	264	6.1	0.95 (0.40, 2.24)	0.01	0.01
Kenya	WASH−B (42)	213	168	20.8	0.59 (0.37, 0.93)	0.03	0.07	133	131	22.1	0.68 (0.42, 1.09)	0.02	0.02
Madagascar	MAHAY (43)	301	329	30.7	0.82 (0.59, 1.14)	0.07	0.11	299	259	35.5	0.79 (0.61, 1.02)	0.08	0.08
Malawi	iLiNS−DYAD−M (44)	99	220	21.8	1.34 (0.90, 1.99)	0.05	0.09	111	212	25.5	0.99 (0.67, 1.47)	0.04	0.04
Malawi	iLiNS−DOSE (45)	135	50	46.0	0.70 (0.49, 1.01)	0.05	0.10	104	28	46.4	0.74 (0.46, 1.16)	0.03	0.03
Mali	PROMIS CS (46)	502	508	61.4	0.61 (0.50, 0.73)	0.21	0.15	451	462	58.9	0.67 (0.56, 0.80)	0.17	0.17
Zimbabwe	SHINE (HIV−) (47)	808	726	9.4	0.65 (0.45, 0.92)	0.06	0.10	697	694	12.1	0.72 (0.53, 0.98)	0.06	0.06
Zimbabwe	SHINE (HIV+) (48)	221	204	9.8	0.42 (0.19, 0.93)	0.01	0.03	66	60	10.0	0.91 (0.32, 2.57)	0.01	0.01
		**4040**	**3345**		**I² = 0.50, Tau² = 0.04**			**3495**	**2881**		**I² = 0.00, Tau² = 0.00**		
**Fixed**					**0.71 (0.65, 0.77)**						**0.74 (0.69, 0.80)**		
**Random**					**0.72 (0.62, 0.85)**						**0.74 (0.69, 0.80)**		

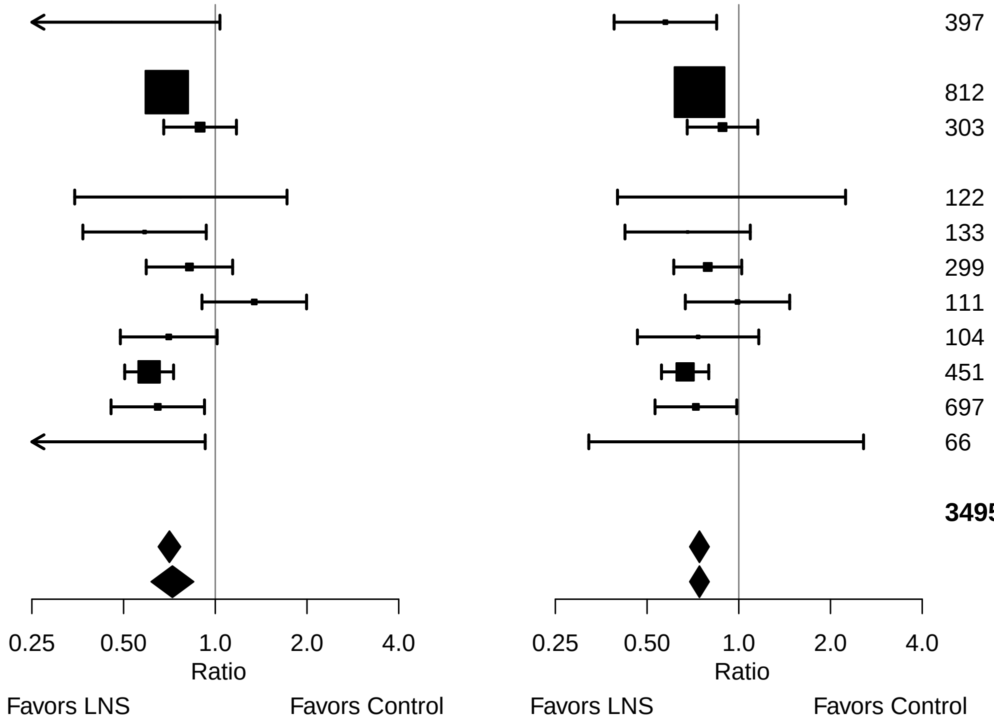

Supplemental figure 8D: Moderate-to-severe anemia prevalence ratio

8D3: Stratified by Maternal education

P−for−interaction = 0.437
Ratio of PRs = 1.07 (0.90, 1.28)

		Primary or greater						Incomplete or no formal					
Country	Trial	LNS N	Control N	Control Prevalence	PR (95% CI)	Fixed W	Random W	LNS N	Control N	Control Prevalence	PR (95% CI)	Fixed W	Random W
Bangladesh	JiVitA−4 (35)												
Bangladesh	RDNS (36)	405	195	13.3	0.57 (0.34, 0.98)	0.09	0.12	144	77	24.7	0.51 (0.30, 0.87)	0.01	0.06
Bangladesh	WASH−B (37)												
Burkina Faso	iLiNS−Zinc (38)	82	16	68.8	0.79 (0.51, 1.23)	0.14	0.14	1862	645	75.0	0.71 (0.65, 0.78)	0.55	0.18
Burkina Faso	PROMIS CS (39)	46	47	36.2	1.02 (0.57, 1.85)	0.08	0.11	526	532	36.5	0.88 (0.72, 1.08)	0.10	0.15
Ghana	GHANA (40)												
Ghana	iLiNS−DYAD−G (41)												
Kenya	WASH−B (42)	168	136	23.5	0.33 (0.21, 0.52)	0.13	0.14	181	164	19.5	0.96 (0.64, 1.46)	0.02	0.09
Madagascar	MAHAY (43)	128	143	33.6	0.84 (0.57, 1.23)	0.18	0.16	472	445	32.6	0.81 (0.62, 1.05)	0.06	0.13
Malawi	iLiNS−DYAD−M (44)	34	66	22.7	0.78 (0.33, 1.82)	0.04	0.07	175	363	24.0	1.19 (0.89, 1.61)	0.05	0.12
Malawi	iLiNS−DOSE (45)	49	17	52.9	0.42 (0.23, 0.78)	0.07	0.10	190	61	44.3	0.81 (0.59, 1.12)	0.04	0.11
Mali	PROMIS CS (46)	107	96	53.1	0.70 (0.52, 0.96)	0.28	0.18	845	874	61.0	0.63 (0.53, 0.74)	0.16	0.16
Zimbabwe	SHINE (HIV−) (47)												
Zimbabwe	SHINE (HIV+) (48)												
		**1019**	**716**		**I² = 0.55, Tau² = 0.08**			**4395**	**3161**		**I² = 0.67, Tau² = 0.03**		
**Fixed**					**0.66 (0.56, 0.77)**						**0.74 (0.70, 0.79)**		
**Random**					**0.64 (0.50, 0.84)**						**0.79 (0.67, 0.93)**		

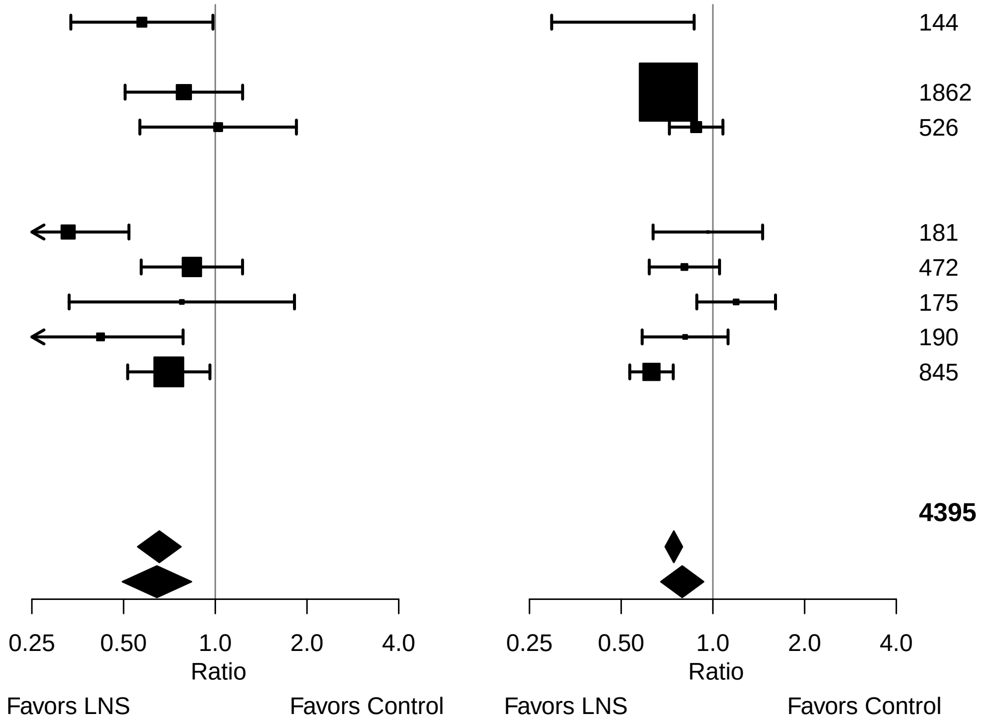

Supplemental figure 8D: Moderate-to-severe anemia prevalence ratio

8D4: Stratified by Child sex

P−for−interaction = 0.692
Ratio of PRs = 1.02 (0.93, 1.12)

Country	Trial	Male LNS N	Male Control N	Male Control Prevalence	Male PR (95% CI)	Male Fixed W	Male Random W	Female LNS N	Female Control N	Female Control Prevalence	Female PR (95% CI)	Female Fixed W	Female Random W
Bangladesh	JiVitA−4 (35)												
Bangladesh	RDNS (36)	266	131	20.6	0.44 (0.25, 0.78)	0.01	0.06	281	141	12.8	0.67 (0.40, 1.11)	0.03	0.03
Bangladesh	WASH−B (37)												
Burkina Faso	iLiNS−Zinc (38)	993	334	79.0	0.70 (0.65, 0.75)	0.65	0.14	964	330	70.9	0.74 (0.64, 0.85)	0.38	0.38
Burkina Faso	PROMIS CS (39)	317	295	42.0	0.87 (0.69, 1.09)	0.07	0.12	257	286	31.1	0.88 (0.67, 1.15)	0.10	0.10
Ghana	GHANA (40)												
Ghana	iLiNS−DYAD−G (41)	164	310	6.5	0.95 (0.45, 1.97)	0.01	0.05	163	348	4.3	0.71 (0.26, 1.92)	0.01	0.01
Kenya	WASH−B (42)	168	145	21.4	0.64 (0.41, 0.99)	0.02	0.08	182	155	21.3	0.62 (0.40, 0.96)	0.04	0.04
Madagascar	MAHAY (43)	277	288	33.0	0.86 (0.64, 1.16)	0.04	0.10	323	300	32.7	0.77 (0.57, 1.04)	0.08	0.08
Malawi	iLiNS−DYAD−M (44)	103	202	25.7	1.32 (0.92, 1.89)	0.03	0.09	107	230	21.7	0.95 (0.61, 1.48)	0.04	0.04
Malawi	iLiNS−DOSE (45)	123	43	51.2	0.74 (0.52, 1.06)	0.03	0.09	120	39	41.0	0.71 (0.45, 1.13)	0.04	0.04
Mali	PROMIS CS (46)	479	526	64.6	0.63 (0.53, 0.75)	0.12	0.13	474	444	55.0	0.65 (0.53, 0.79)	0.20	0.20
Zimbabwe	SHINE (HIV−) (47)	830	814	10.8	0.71 (0.52, 0.98)	0.03	0.10	852	780	10.4	0.62 (0.45, 0.85)	0.08	0.08
Zimbabwe	SHINE (HIV+) (48)	146	149	14.1	0.29 (0.13, 0.67)	0.01	0.04	160	136	5.9	1.06 (0.45, 2.51)	0.01	0.01
		**3866**	**3237**		**I^2 = 0.62, Tau^2 = 0.07**			**3883**	**3189**		**I^2 = 0.00, Tau^2 = 0.00**		
**Fixed**					**0.71 (0.67, 0.76)**						**0.73 (0.66, 0.79)**		
**Random**					**0.73 (0.60, 0.89)**						**0.73 (0.66, 0.79)**		

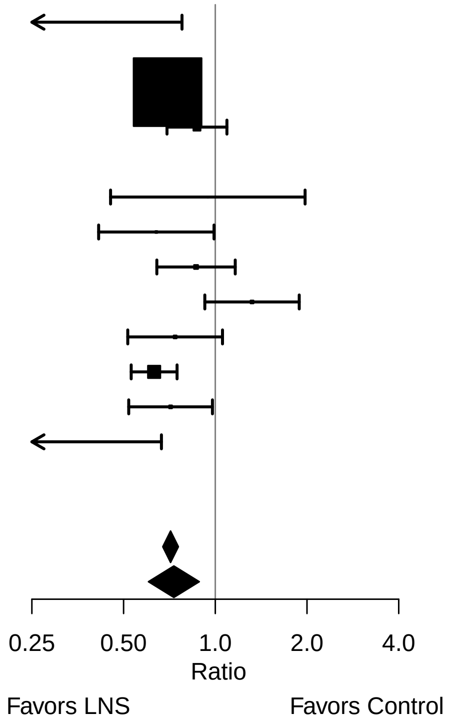

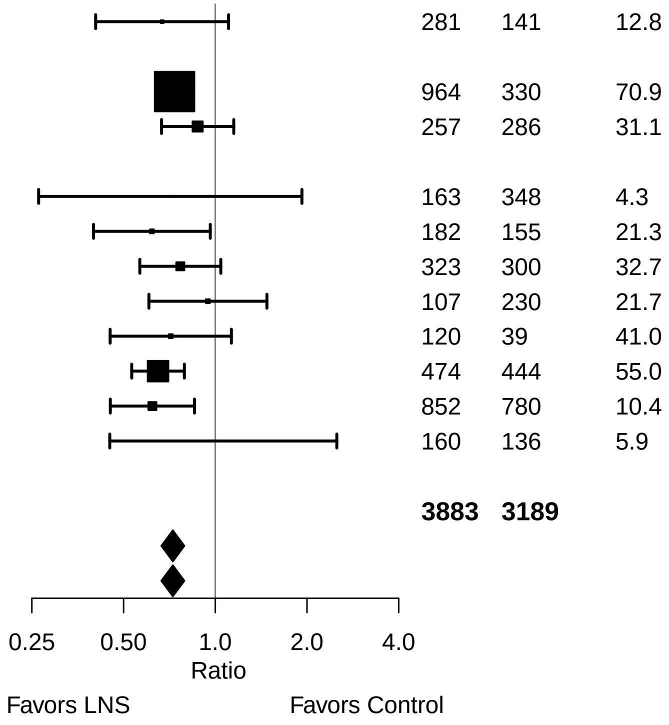

Supplemental figure 8D: Moderate-to-severe anemia prevalence ratio

8D5: Stratified by Child birth order

P−for−interaction = 0.416
Ratio of PRs = 1.05 (0.94, 1.17)

Country	Trial	Later born LNS N	Later born Control N	Later born Control Prevalence	Later born PR (95% CI)	Later born Fixed W	Later born Random W	Firstborn LNS N	Firstborn Control N	Firstborn Control Prevalence	Firstborn PR (95% CI)	Firstborn Fixed W	Firstborn Random W
Bangladesh	JiVitA−4 (35)												
Bangladesh	RDNS (36)	339	171	17.5	0.47 (0.29, 0.76)	0.02	0.08	210	101	14.9	0.67 (0.41, 1.10)	0.03	0.09
Bangladesh	WASH−B (37)												
Burkina Faso	iLiNS−Zinc (38)	1522	520	74.0	0.72 (0.64, 0.81)	0.40	0.13	435	144	78.5	0.70 (0.63, 0.78)	0.70	0.23
Burkina Faso	PROMIS CS (39)	498	477	37.1	0.84 (0.68, 1.04)	0.11	0.12	75	103	35.0	1.18 (0.74, 1.88)	0.03	0.10
Ghana	GHANA (40)	60	54	33.3	0.25 (0.10, 0.63)	0.01	0.03	38	38	31.6	0.42 (0.16, 1.07)	0.01	0.03
Ghana	iLiNS−DYAD−G (41)	220	435	6.0	0.61 (0.28, 1.32)	0.01	0.04	108	226	4.4	1.46 (0.57, 3.74)	0.01	0.03
Kenya	WASH−B (42)	282	244	20.9	0.68 (0.46, 1.00)	0.03	0.09	67	56	23.2	0.45 (0.20, 1.03)	0.01	0.04
Madagascar	MAHAY (43)	458	454	33.3	0.81 (0.63, 1.04)	0.08	0.11	141	131	31.3	0.84 (0.56, 1.27)	0.04	0.11
Malawi	iLiNS−DYAD−M (44)	165	342	23.1	1.13 (0.82, 1.56)	0.05	0.10	45	88	26.1	1.19 (0.68, 2.08)	0.02	0.08
Malawi	iLiNS−DOSE (45)												
Mali	PROMIS CS (46)	790	832	60.0	0.64 (0.55, 0.75)	0.21	0.13	146	126	60.3	0.62 (0.47, 0.83)	0.09	0.15
Zimbabwe	SHINE (HIV−) (47)	1188	1100	10.4	0.60 (0.45, 0.80)	0.06	0.11	418	416	10.8	0.86 (0.58, 1.29)	0.05	0.11
Zimbabwe	SHINE (HIV+) (48)	252	216	10.2	0.39 (0.20, 0.76)	0.01	0.05	51	64	9.4	1.25 (0.45, 3.50)	0.01	0.03
		**5774**	**4845**		**I² = 0.61, Tau² = 0.07**			**1734**	**1493**		**I² = 0.37, Tau² = 0.04**		
**Fixed**					**0.71 (0.66, 0.76)**						**0.73 (0.67, 0.79)**		
**Random**					**0.67 (0.55, 0.81)**						**0.79 (0.66, 0.95)**		

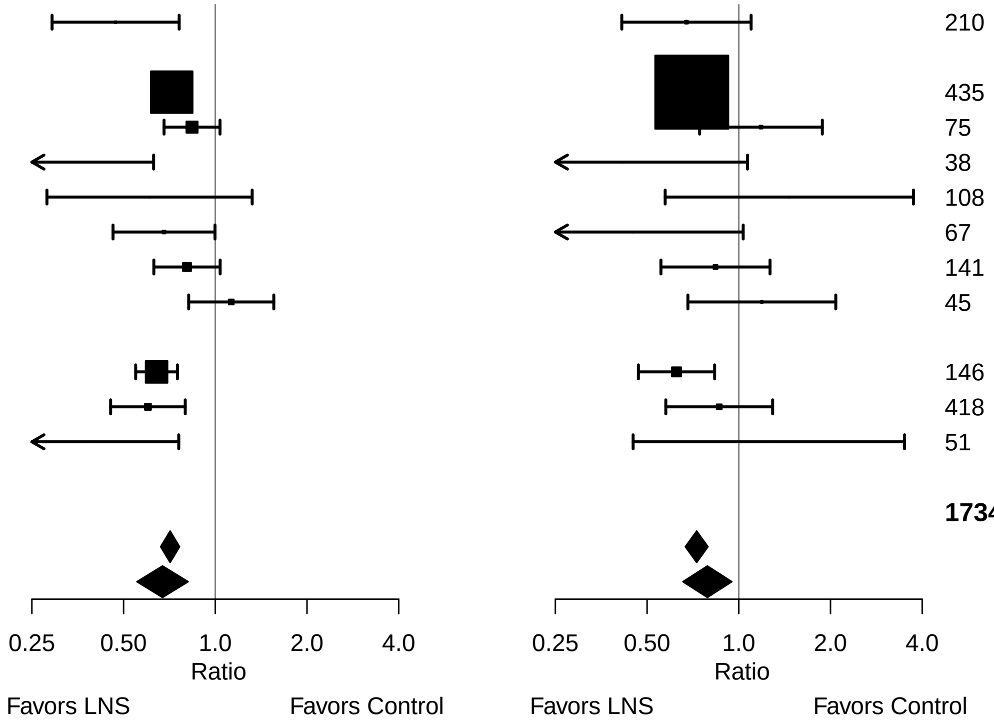

Supplemental figure 8D: Moderate-to-severe anemia prevalence ratio

8D6: Stratified by Child baseline acute malnutrition (insufficient comparisons)

Supplemental figure 8D: Moderate-to-severe anemia prevalence ratio

8b7: Stratified by Child baseline anemia

P−for−interaction = 0.686
Ratio of PRs = 1.05 (0.82, 1.35)

		**Not anemic**						**Anemic**					
Country	Trial	**LNS** N	**Control** N	**Control** Prevalence	**PR** (95% CI)	**Fixed** W	**Random** W	**LNS** N	**Control** N	**Control** Prevalence	**PR** (95% CI)	**Fixed** W	**Random** W
Bangladesh	JiVitA−4 (35)												
Bangladesh	RDNS (36)	213	93	6.5	0.51 (0.15, 1.78)	0.04	0.08	314	160	21.9	0.57 (0.41, 0.78)	0.07	0.23
Bangladesh	WASH−B (37)												
Burkina Faso	iLiNS−Zinc (38)	177	61	60.7	0.64 (0.48, 0.87)	0.61	0.42	1780	603	76.5	0.72 (0.66, 0.79)	0.80	0.31
Burkina Faso	PROMIS CS (39)												
Ghana	GHANA (40)												
Ghana	iLiNS−DYAD−G (41)												
Kenya	WASH−B (42)												
Madagascar	MAHAY (43)												
Malawi	iLiNS−DYAD−M (44)	66	145	12.4	1.46 (0.75, 2.86)	0.12	0.21	134	269	27.5	1.17 (0.85, 1.60)	0.07	0.23
Malawi	iLiNS−DOSE (45)	101	45	37.8	0.74 (0.45, 1.20)	0.23	0.29	142	37	56.8	0.72 (0.51, 1.02)	0.06	0.22
Mali	PROMIS CS (46)												
Zimbabwe	SHINE (HIV−) (47)												
Zimbabwe	SHINE (HIV+) (48)												
		**557**	**344**		**I² = 0.41, Tau² = 0.07**			**2370**	**1069**		**I² = 0.73, Tau² = 0.07**		
**Fixed**					**0.73 (0.58, 0.92)**						**0.73 (0.67, 0.80)**		
**Random**					**0.78 (0.53, 1.14)**						**0.76 (0.57, 1.02)**		

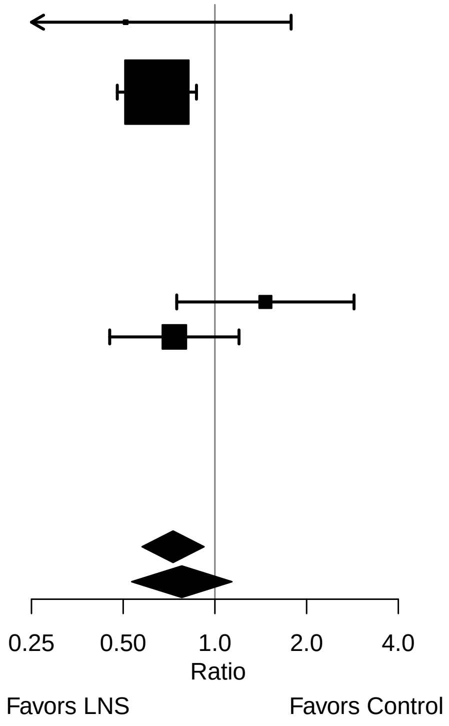

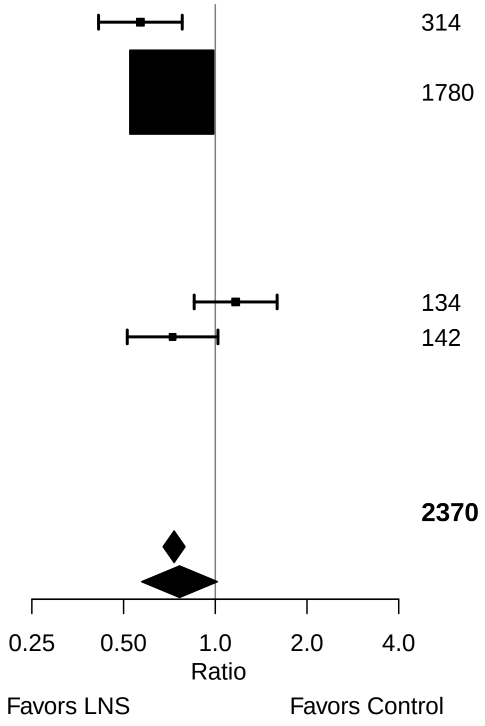

Supplemental figure 8D: Moderate-to-severe anemia prevalence ratio

8D8: Stratified by Child high-dose vitamin A supplementation

P−for−interaction = 0.874
Ratio of PRs = 1.03 (0.75, 1.40)

Country	Trial	Received VitA LNS N	Received VitA Control N	Received VitA Control Prevalence	Received VitA PR (95% CI)	Received VitA Fixed W	Received VitA Random W	Not treated LNS N	Not treated Control N	Not treated Control Prevalence	Not treated PR (95% CI)	Not treated Fixed W	Not treated Random W
Bangladesh	JiVitA−4 (35)												
Bangladesh	RDNS (36)	444	223	16.6	0.52 (0.35, 0.75)	0.32	0.30	105	49	16.3	0.64 (0.28, 1.47)	0.06	0.11
Bangladesh	WASH−B (37)												
Burkina Faso	iLiNS−Zinc (38)												
Burkina Faso	PROMIS CS (39)												
Ghana	GHANA (40)												
Ghana	iLiNS−DYAD−G (41)												
Kenya	WASH−B (42)	29	34	26.5	0.78 (0.30, 2.02)	0.05	0.08	263	212	19.8	0.63 (0.42, 0.95)	0.24	0.24
Madagascar	MAHAY (43)												
Malawi	iLiNS−DYAD−M (44)	75	136	25.7	0.98 (0.61, 1.59)	0.19	0.22	133	289	22.5	1.27 (0.90, 1.79)	0.32	0.27
Malawi	iLiNS−DOSE (45)												
Mali	PROMIS CS (46)												
Zimbabwe	SHINE (HIV−) (47)	874	833	9.6	0.70 (0.50, 0.99)	0.40	0.33	665	622	11.6	0.60 (0.42, 0.84)	0.33	0.27
Zimbabwe	SHINE (HIV+) (48)	165	127	8.7	0.35 (0.13, 0.98)	0.04	0.07	112	134	12.7	0.49 (0.21, 1.13)	0.06	0.11
		**1587**	**1353**		**I² = 0.33, Tau² = 0.03**			**1278**	**1306**		**I² = 0.68, Tau² = 0.08**		
**Fixed**					**0.66 (0.54, 0.82)**						**0.77 (0.63, 0.94)**		
**Random**					**0.66 (0.50, 0.88)**						**0.73 (0.52, 1.02)**		

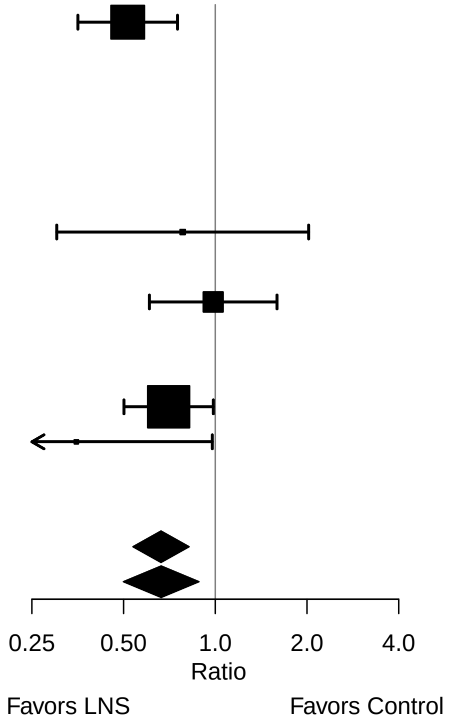

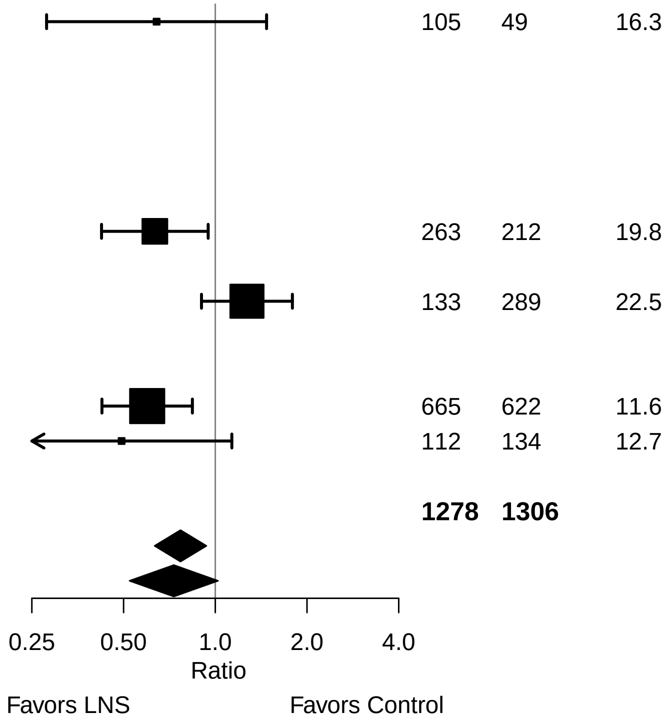

Supplemental figure 8D: Moderate-to-severe anemia prevalence ratio

8D9: Stratified by Child inflammation

P−for−interaction = 0.197
Ratio of PRs = 1.17 (0.92, 1.47)

		Not inflamed						High AGP or CRP					
Country	Trial	LNS N	Control N	Control Prevalence	PR (95% CI)	Fixed W	Random W	LNS N	Control N	Control Prevalence	PR (95% CI)	Fixed W	Random W
Bangladesh	JiVitA−4 (35)												
Bangladesh	RDNS (36)	369	168	17.3	0.50 (0.33, 0.76)	0.26	0.26	180	104	15.4	0.61 (0.36, 1.03)	0.07	0.10
Bangladesh	WASH−B (37)												
Burkina Faso	iLiNS−Zinc (38)	131	33	69.7	0.61 (0.44, 0.84)	0.43	0.32	197	69	81.2	0.73 (0.61, 0.86)	0.62	0.49
Burkina Faso	PROMIS CS (39)												
Ghana	GHANA (40)												
Ghana	iLiNS−DYAD−G (41)												
Kenya	WASH−B (42)	157	135	14.1	0.45 (0.23, 0.88)	0.10	0.14	141	123	30.9	0.71 (0.47, 1.09)	0.10	0.14
Madagascar	MAHAY (43)												
Malawi	iLiNS−DYAD−M (44)	59	123	13.0	0.91 (0.40, 2.10)	0.06	0.10	138	269	29.4	1.04 (0.76, 1.42)	0.18	0.23
Malawi	iLiNS−DOSE (45)												
Mali	PROMIS CS (46)												
Zimbabwe	SHINE (HIV−) (47)	414	354	5.6	1.03 (0.60, 1.76)	0.15	0.19	105	99	14.1	0.61 (0.29, 1.29)	0.03	0.05
Zimbabwe	SHINE (HIV+) (48)												
		**1130**	**813**		**I^2 = 0.34, Tau^2 = 0.04**			**761**	**664**		**I^2 = 0.22, Tau^2 = 0.01**		
**Fixed**					**0.63 (0.51, 0.77)**						**0.76 (0.66, 0.87)**		
**Random**					**0.64 (0.48, 0.85)**						**0.76 (0.65, 0.91)**		

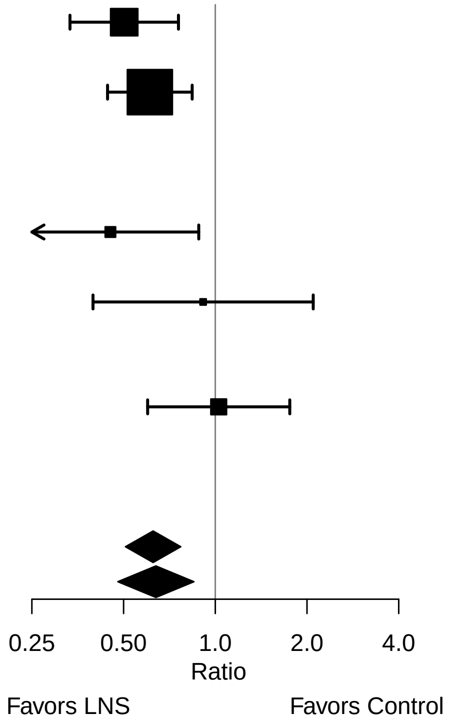

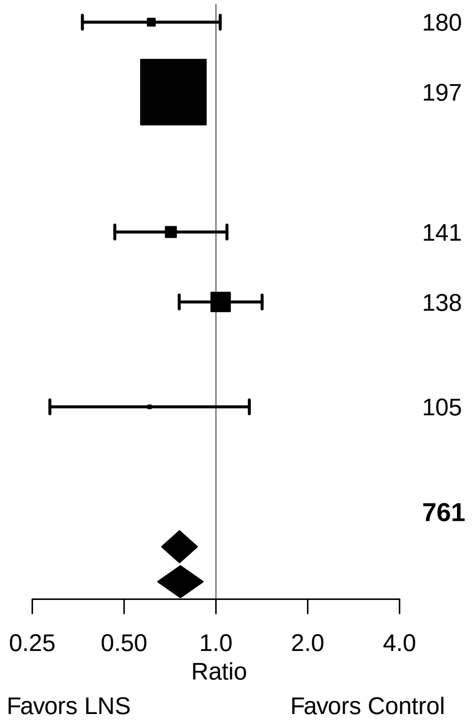

Supplemental figure 8E: Moderate-to-severe anemia prevalence difference

8E1: Stratified by Maternal BMI

P−for−interaction = 0.047
Difference in PDs = 0.03 (0.00, 0.07)

Country	Trial	At least 20 kg/m² LNS N	Control N	Control Prevalence	PD (95% CI)	Fixed W	Random W	Less than 20 kg/m² LNS N	Control N	Control Prevalence	PD (95% CI)	Fixed W	Random W
Bangladesh	JiVitA−4 (35)												
Bangladesh	RDNS (36)	227	102	20.6	−0.11 (−0.20, −0.02)	0.04	0.12	300	160	13.8	−0.05 (−0.11, 0.00)	0.22	0.15
Bangladesh	WASH−B (37)												
Burkina Faso	iLiNS−Zinc (38)	1217	390	76.2	−0.22 (−0.28, −0.17)	0.10	0.14	738	274	73.4	−0.20 (−0.27, −0.13)	0.14	0.14
Burkina Faso	PROMIS CS (39)	335	349	36.7	−0.01 (−0.09, 0.07)	0.05	0.13	239	227	37.0	−0.09 (−0.19, 0.01)	0.07	0.12
Ghana	GHANA (40)												
Ghana	iLiNS−DYAD−G (41)												
Kenya	WASH−B (42)	266	227	22.9	−0.11 (−0.18, −0.03)	0.06	0.13	72	60	11.7	0.05 (−0.05, 0.15)	0.07	0.12
Madagascar	MAHAY (43)												
Malawi	iLiNS−DYAD−M (44)	124	258	24.0	0.03 (−0.06, 0.13)	0.04	0.12	85	171	23.4	0.02 (−0.09, 0.14)	0.05	0.12
Malawi	iLiNS−DOSE (45)	179	52	46.2	−0.19 (−0.33, −0.05)	0.02	0.09	61	29	48.3	−0.02 (−0.24, 0.20)	0.01	0.07
Mali	PROMIS CS (46)	702	685	60.0	−0.22 (−0.30, −0.14)	0.05	0.13	246	279	61.3	−0.23 (−0.30, −0.16)	0.14	0.14
Zimbabwe	SHINE (HIV−) (47)	1222	1161	10.9	−0.04 (−0.06, −0.02)	0.65	0.15	213	220	6.8	0.02 (−0.03, 0.06)	0.30	0.15
Zimbabwe	SHINE (HIV+) (48)												
		**4272**	**3224**		**I² = 0.88, Tau² = 0.01**			**1954**	**1420**		**I² = 0.87, Tau² = 0.01**		
**Fixed**					**−0.07 (−0.09, −0.05)**						**−0.07 (−0.09, −0.04)**		
**Random**					**−0.11 (−0.17, −0.04)**						**−0.07 (−0.14, 0.01)**		

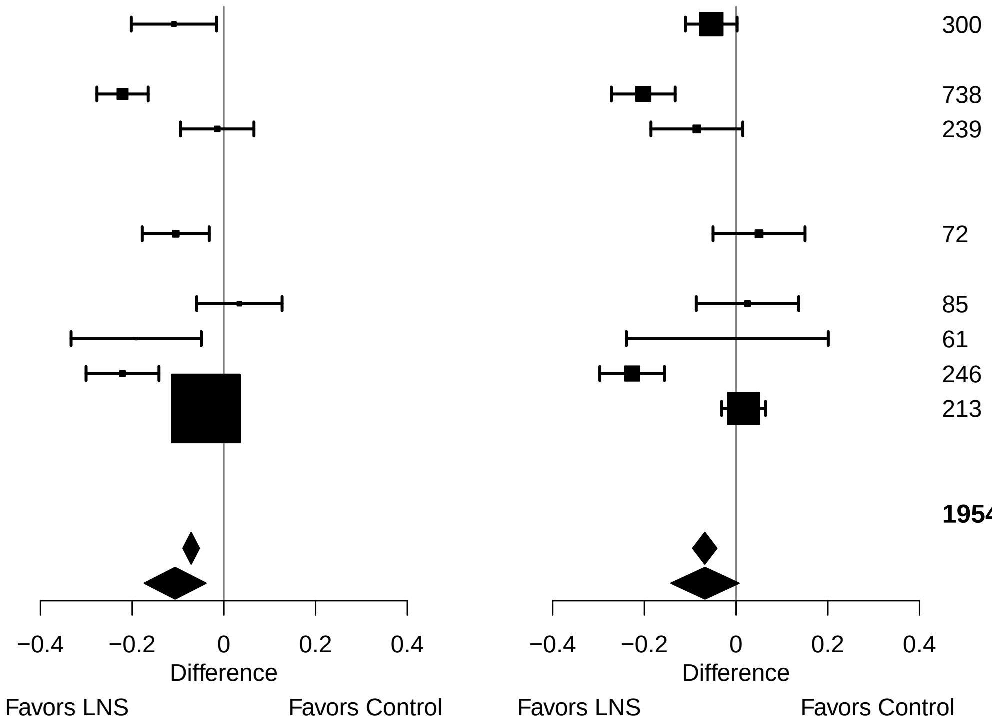

Supplemental figure 8E: Moderate-to-severe anemia prevalence difference

8E2: Stratified by Maternal age

P−for−interaction = 0.502
Difference in PDs = 0.01 (−0.02, 0.03)

		At least 25 y						Less than 25 y					
Country	Trial	LNS N	Control N	Control Prevalence	PD (95% CI)	Fixed W	Random W	LNS N	Control N	Control Prevalence	PD (95% CI)	Fixed W	Random W
Bangladesh	JiVitA−4 (35)												
Bangladesh	RDNS (36)	152	81	17.3	−0.09 (−0.21, 0.02)	0.02	0.07	397	191	16.2	−0.07 (−0.12, −0.02)	0.14	0.12
Bangladesh	WASH−B (37)												
Burkina Faso	iLiNS−Zinc (38)	1132	399	74.2	−0.23 (−0.31, −0.15)	0.04	0.09	812	262	76.0	−0.19 (−0.26, −0.13)	0.08	0.10
Burkina Faso	PROMIS CS (39)	271	263	32.7	−0.04 (−0.12, 0.05)	0.04	0.09	303	318	39.9	−0.05 (−0.15, 0.05)	0.04	0.08
Ghana	GHANA (40)												
Ghana	iLiNS−DYAD−G (41)	206	397	5.0	−0.01 (−0.05, 0.02)	0.23	0.11	122	264	6.1	0.00 (−0.05, 0.05)	0.14	0.12
Kenya	WASH−B (42)	213	168	20.8	−0.09 (−0.17, −0.01)	0.05	0.09	133	131	22.1	−0.07 (−0.16, 0.02)	0.05	0.09
Madagascar	MAHAY (43)	301	329	30.7	−0.05 (−0.15, 0.04)	0.03	0.09	299	259	35.5	−0.07 (−0.16, 0.01)	0.05	0.09
Malawi	iLiNS−DYAD−M (44)	99	220	21.8	0.07 (−0.03, 0.18)	0.03	0.08	111	212	25.5	0.00 (−0.10, 0.10)	0.04	0.08
Malawi	iLiNS−DOSE (45)	135	50	46.0	−0.14 (−0.29, 0.01)	0.01	0.06	104	28	46.4	−0.13 (−0.32, 0.07)	0.01	0.03
Mali	PROMIS CS (46)	502	508	61.4	−0.24 (−0.32, −0.16)	0.04	0.09	451	462	58.9	−0.20 (−0.27, −0.12)	0.06	0.09
Zimbabwe	SHINE (HIV−) (47)	808	726	9.4	−0.03 (−0.06, −0.01)	0.41	0.11	697	694	12.1	−0.03 (−0.06, 0.00)	0.37	0.13
Zimbabwe	SHINE (HIV+) (48)	221	204	9.8	−0.06 (−0.11, 0.00)	0.10	0.10	66	60	10.0	−0.01 (−0.11, 0.09)	0.04	0.08
		**4040**	**3345**		**I² = 0.81, Tau² = 0.01**			**3495**	**2881**		**I² = 0.73, Tau² = 0.00**		
**Fixed**					**−0.05 (−0.07, −0.03)**						**−0.06 (−0.08, −0.04)**		
**Random**					**−0.08 (−0.13, −0.03)**						**−0.07 (−0.11, −0.03)**		

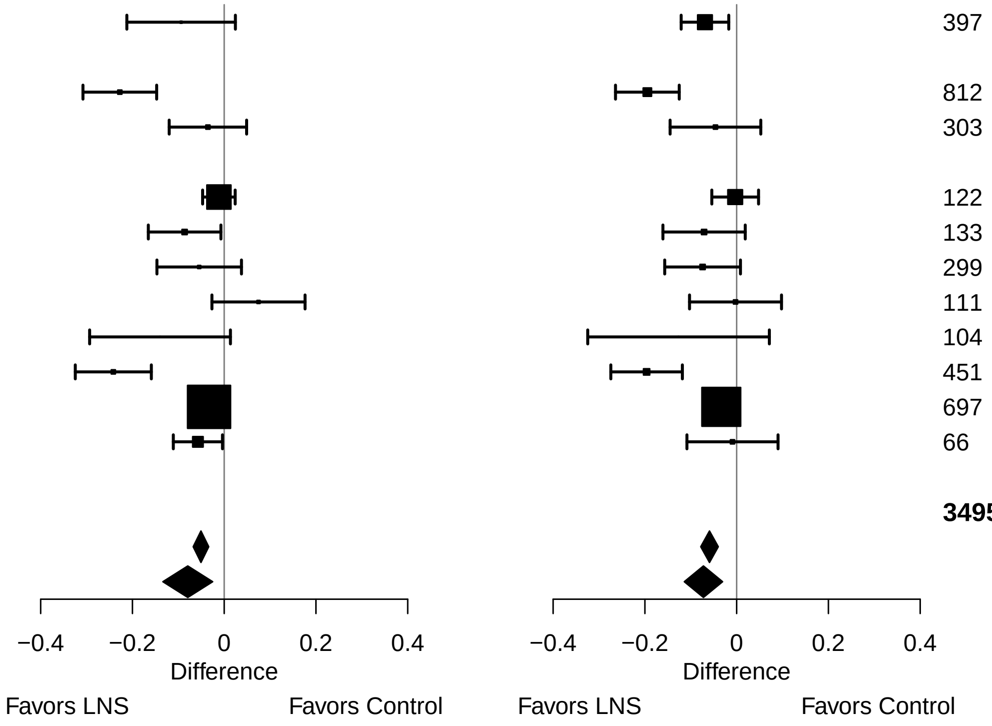

Supplemental figure 8E: Moderate-to-severe anemia prevalence difference

8E3: Stratified by Maternal education

P−for−interaction = 0.526
Difference in PDs = 0.02 (−0.04, 0.07)

		Primary or greater						Incomplete or no formal					
Country	Trial	LNS N	Control N	Control Prevalence	PD (95% CI)	Fixed W	Random W	LNS N	Control N	Control Prevalence	PD (95% CI)	Fixed W	Random W
Bangladesh	JiVitA−4 (35)												
Bangladesh	RDNS (36)	405	195	13.3	−0.06 (−0.12, 0.01)	0.37	0.29	144	77	24.7	−0.12 (−0.23, −0.01)	0.06	0.11
Bangladesh	WASH−B (37)												
Burkina Faso	iLiNS−Zinc (38)	82	16	68.8	−0.15 (−0.42, 0.13)	0.02	0.03	1862	645	75.0	−0.21 (−0.27, −0.16)	0.23	0.14
Burkina Faso	PROMIS CS (39)	46	47	36.2	0.01 (−0.21, 0.22)	0.03	0.05	526	532	36.5	−0.04 (−0.11, 0.03)	0.16	0.13
Ghana	GHANA (40)												
Ghana	iLiNS−DYAD−G (41)												
Kenya	WASH−B (42)	168	136	23.5	−0.16 (−0.23, −0.09)	0.31	0.27	181	164	19.5	−0.01 (−0.09, 0.07)	0.12	0.13
Madagascar	MAHAY (43)	128	143	33.6	−0.05 (−0.17, 0.06)	0.11	0.14	472	445	32.6	−0.06 (−0.14, 0.02)	0.12	0.13
Malawi	iLiNS−DYAD−M (44)	34	66	22.7	−0.05 (−0.22, 0.12)	0.05	0.08	175	363	24.0	0.05 (−0.03, 0.12)	0.12	0.13
Malawi	iLiNS−DOSE (45)	49	17	52.9	−0.32 (−0.57, −0.07)	0.02	0.04	190	61	44.3	−0.09 (−0.22, 0.05)	0.04	0.10
Mali	PROMIS CS (46)	107	96	53.1	−0.16 (−0.30, −0.02)	0.08	0.11	845	874	61.0	−0.23 (−0.30, −0.16)	0.15	0.13
Zimbabwe	SHINE (HIV−) (47)												
Zimbabwe	SHINE (HIV+) (48)												
		**1019**	**716**		**I² = 0.31, Tau² = 0.00**			**4395**	**3161**		**I² = 0.86, Tau² = 0.01**		
**Fixed**					**−0.10 (−0.14, −0.06)**						**−0.10 (−0.13, −0.08)**		
**Random**					**−0.10 (−0.15, −0.05)**						**−0.09 (−0.16, −0.02)**		

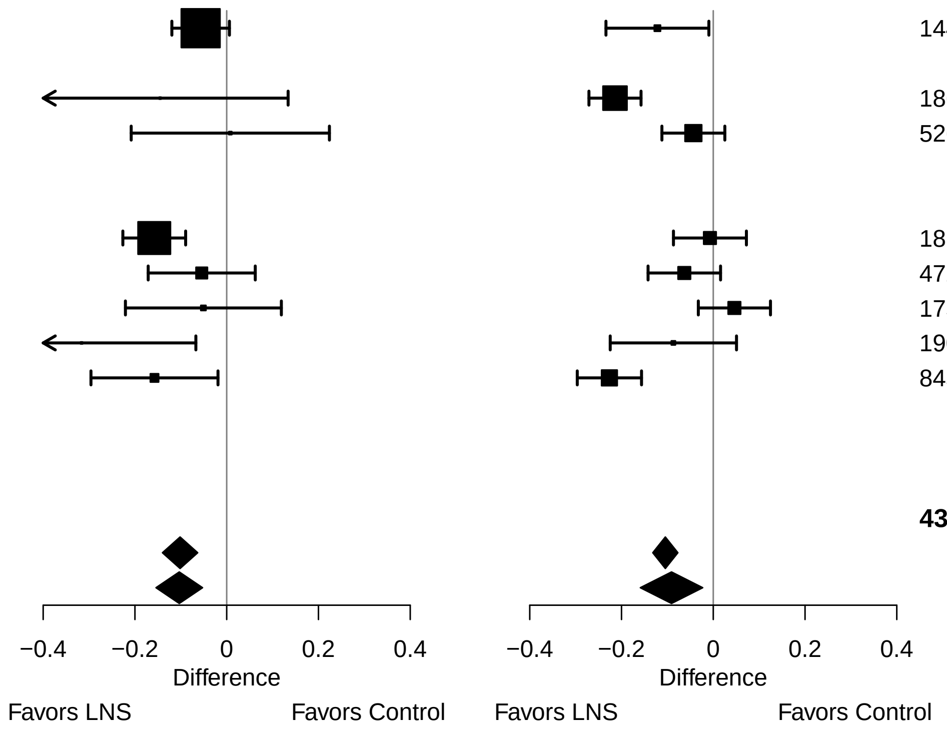

Supplemental figure 8E: Moderate-to-severe anemia prevalence difference

8E4: Stratified by Child sex

P−for−interaction = 0.323
Difference in PDs = 0.01 (−0.01, 0.04)

		Male						Female					
Country	Trial	LNS N	Control N	Control Prevalence	PD (95% CI)	Fixed W	Random W	LNS N	Control N	Control Prevalence	PD (95% CI)	Fixed W	Random W
Bangladesh	JiVitA−4 (35)												
Bangladesh	RDNS (36)	266	131	20.6	−0.12 (−0.21, −0.02)	0.03	0.08	281	141	12.8	−0.04 (−0.10, 0.01)	0.08	0.11
Bangladesh	WASH−B (37)												
Burkina Faso	iLiNS−Zinc (38)	993	334	79.0	−0.24 (−0.28, −0.19)	0.15	0.11	964	330	70.9	−0.19 (−0.28, −0.10)	0.03	0.08
Burkina Faso	PROMIS CS (39)	317	295	42.0	−0.05 (−0.14, 0.03)	0.04	0.09	257	286	31.1	−0.04 (−0.12, 0.04)	0.04	0.09
Ghana	GHANA (40)												
Ghana	iLiNS−DYAD−G (41)	164	310	6.5	0.00 (−0.05, 0.04)	0.15	0.11	163	348	4.3	−0.01 (−0.05, 0.02)	0.20	0.12
Kenya	WASH−B (42)	168	145	21.4	−0.08 (−0.16, 0.00)	0.05	0.09	182	155	21.3	−0.08 (−0.15, −0.01)	0.05	0.09
Madagascar	MAHAY (43)	277	288	33.0	−0.04 (−0.14, 0.05)	0.04	0.09	323	300	32.7	−0.08 (−0.16, 0.01)	0.03	0.08
Malawi	iLiNS−DYAD−M (44)	103	202	25.7	0.08 (−0.02, 0.19)	0.03	0.08	107	230	21.7	−0.01 (−0.11, 0.08)	0.03	0.08
Malawi	iLiNS−DOSE (45)	123	43	51.2	−0.13 (−0.30, 0.04)	0.01	0.06	120	39	41.0	−0.13 (−0.30, 0.03)	0.01	0.04
Mali	PROMIS CS (46)	479	526	64.6	−0.24 (−0.32, −0.16)	0.04	0.09	474	444	55.0	−0.19 (−0.27, −0.11)	0.04	0.09
Zimbabwe	SHINE (HIV−) (47)	830	814	10.8	−0.03 (−0.06, 0.00)	0.39	0.11	852	780	10.4	−0.04 (−0.07, −0.01)	0.37	0.13
Zimbabwe	SHINE (HIV+) (48)	146	149	14.1	−0.10 (−0.17, −0.03)	0.07	0.10	160	136	5.9	0.00 (−0.05, 0.06)	0.10	0.11
		**3866**	**3237**		**I^2 = 0.89, Tau^2 = 0.01**			**3883**	**3189**		**I^2 = 0.68, Tau^2 = 0.00**		
**Fixed**					**−0.08 (−0.09, −0.06)**						**−0.04 (−0.06, −0.03)**		
**Random**					**−0.09 (−0.14, −0.03)**						**−0.06 (−0.10, −0.03)**		

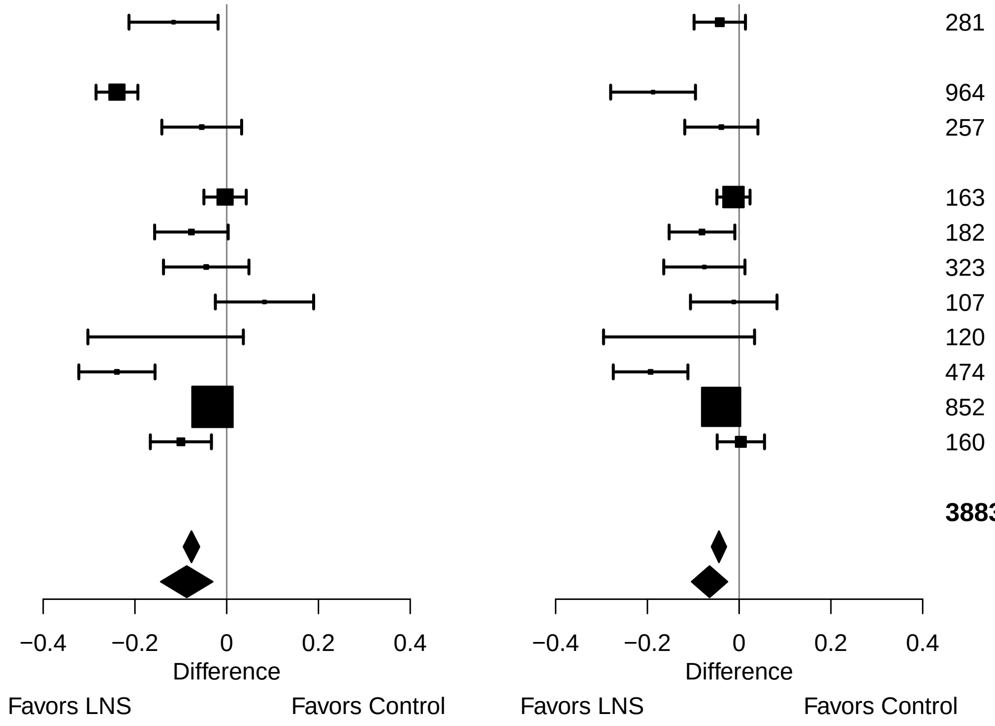

Supplemental figure 8E: Moderate-to-severe anemia prevalence difference

8E5: Stratified by Child birth order

P−for−interaction = 0.058
Difference in PDs = 0.03 (0.00, 0.06)

Later born

Country	Trial	LNS N	Control N	Control Prevalence	PD (95% CI)	Fixed W	Random W
Bangladesh	JiVitA−4 (35)						
Bangladesh	RDNS (36)	339	171	17.5	−0.09 (−0.16, −0.02)	0.04	0.09
Bangladesh	WASH−B (37)						
Burkina Faso	iLiNS−Zinc (38)	1522	520	74.0	−0.21 (−0.28, −0.14)	0.04	0.09
Burkina Faso	PROMIS CS (39)	498	477	37.1	−0.06 (−0.13, 0.01)	0.04	0.09
Ghana	GHANA (40)	60	54	33.3	−0.25 (−0.39, −0.11)	0.01	0.06
Ghana	iLiNS−DYAD−G (41)	220	435	6.0	−0.02 (−0.06, 0.01)	0.16	0.10
Kenya	WASH−B (42)	282	244	20.9	−0.07 (−0.13, 0.00)	0.05	0.09
Madagascar	MAHAY (43)	458	454	33.3	−0.06 (−0.14, 0.01)	0.04	0.09
Malawi	iLiNS−DYAD−M (44)	165	342	23.1	0.03 (−0.05, 0.11)	0.03	0.09
Malawi	iLiNS−DOSE (45)						
Mali	PROMIS CS (46)	790	832	60.0	−0.21 (−0.29, −0.14)	0.04	0.09
Zimbabwe	SHINE (HIV−) (47)	1188	1100	10.4	−0.04 (−0.06, −0.02)	0.44	0.11
Zimbabwe	SHINE (HIV+) (48)	252	216	10.2	−0.06 (−0.11, −0.02)	0.10	0.10
		**5774**	**4845**		**I² = 0.82, Tau² = 0.01**		
**Fixed**					**−0.06 (−0.07, −0.05)**		
**Random**					**−0.09 (−0.14, −0.04)**		

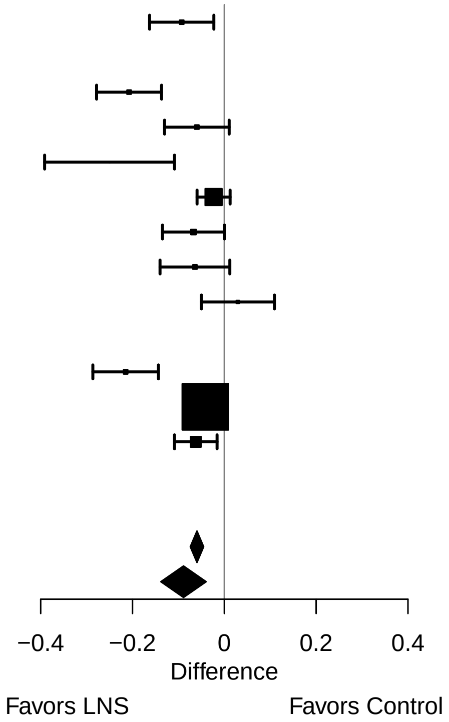

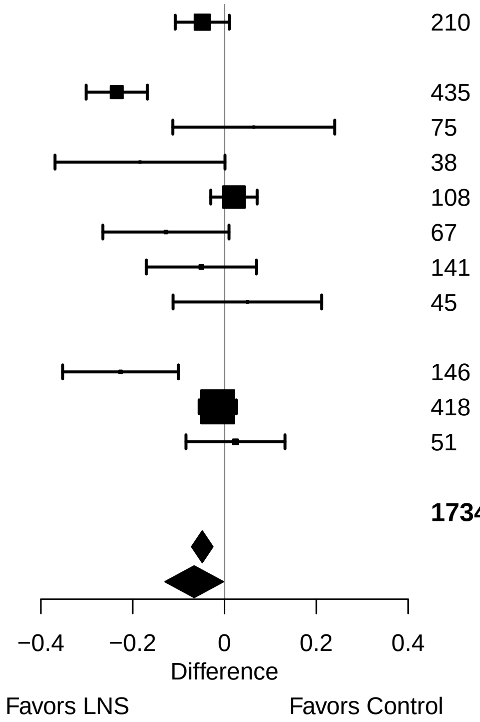

Firstborn

Country	Trial	LNS N	Control N	Control Prevalence	PD (95% CI)	Fixed W	Random W
Bangladesh	JiVitA−4 (35)						
Bangladesh	RDNS (36)	210	101	14.9	−0.05 (−0.11, 0.01)	0.15	0.11
Bangladesh	WASH−B (37)						
Burkina Faso	iLiNS−Zinc (38)	435	144	78.5	−0.23 (−0.30, −0.17)	0.12	0.11
Burkina Faso	PROMIS CS (39)	75	103	35.0	0.06 (−0.11, 0.24)	0.02	0.06
Ghana	GHANA (40)	38	38	31.6	−0.18 (−0.37, 0.00)	0.02	0.06
Ghana	iLiNS−DYAD−G (41)	108	226	4.4	0.02 (−0.03, 0.07)	0.21	0.12
Kenya	WASH−B (42)	67	56	23.2	−0.13 (−0.27, 0.01)	0.03	0.08
Madagascar	MAHAY (43)	141	131	31.3	−0.05 (−0.17, 0.07)	0.04	0.09
Malawi	iLiNS−DYAD−M (44)	45	88	26.1	0.05 (−0.11, 0.21)	0.02	0.07
Malawi	iLiNS−DOSE (45)						
Mali	PROMIS CS (46)	146	126	60.3	−0.23 (−0.35, −0.10)	0.03	0.08
Zimbabwe	SHINE (HIV−) (47)	418	416	10.8	−0.01 (−0.06, 0.03)	0.32	0.12
Zimbabwe	SHINE (HIV+) (48)	51	64	9.4	0.02 (−0.08, 0.13)	0.05	0.09
		**1734**	**1493**		**I² = 0.82, Tau² = 0.01**		
**Fixed**					**−0.05 (−0.07, −0.03)**		
**Random**					**−0.07 (−0.13, 0.00)**		

Supplemental figure 8E: Moderate-to-severe anemia prevalence difference

8E6: Stratified by Child baseline acute malnutrition (insufficient comparisons)

Supplemental figure 8E: Moderate-to-severe anemia prevalence difference

8E7: Stratified by Child baseline anemia

P−for−interaction = 0.251
Difference in PDs = −0.04 (−0.10, 0.03)

		Not anemic						Anemic					
Country	Trial	LNS N	Control N	Control Prevalence	PD (95% CI)	Fixed W	Random W	LNS N	Control N	Control Prevalence	PD (95% CI)	Fixed W	Random W
Bangladesh	JiVitA−4 (35)												
Bangladesh	RDNS (36)	213	93	6.5	−0.03 (−0.10, 0.04)	0.53	0.31	314	160	21.9	−0.09 (−0.15, −0.04)	0.41	0.29
Bangladesh	WASH−B (37)												
Burkina Faso	iLiNS−Zinc (38)	177	61	60.7	−0.22 (−0.37, −0.06)	0.11	0.21	1780	603	76.5	−0.21 (−0.28, −0.15)	0.39	0.28
Burkina Faso	PROMIS CS (39)												
Ghana	GHANA (40)												
Ghana	iLiNS−DYAD−G (41)												
Kenya	WASH−B (42)												
Madagascar	MAHAY (43)												
Malawi	iLiNS−DYAD−M (44)	66	145	12.4	0.06 (−0.04, 0.16)	0.26	0.28	134	269	27.5	0.05 (−0.05, 0.14)	0.16	0.25
Malawi	iLiNS−DOSE (45)	101	45	37.8	−0.10 (−0.26, 0.06)	0.10	0.20	142	37	56.8	−0.15 (−0.33, 0.02)	0.05	0.18
Mali	PROMIS CS (46)												
Zimbabwe	SHINE (HIV−) (47)												
Zimbabwe	SHINE (HIV+) (48)												
		**557**	**344**		**I^2 = 0.66, Tau^2 = 0.01**			**2370**	**1069**		**I^2 = 0.86, Tau^2 = 0.01**		
**Fixed**					**−0.04 (−0.09, 0.02)**						**−0.12 (−0.16, −0.08)**		
**Random**					**−0.06 (−0.17, 0.05)**						**−0.10 (−0.21, 0.01)**		

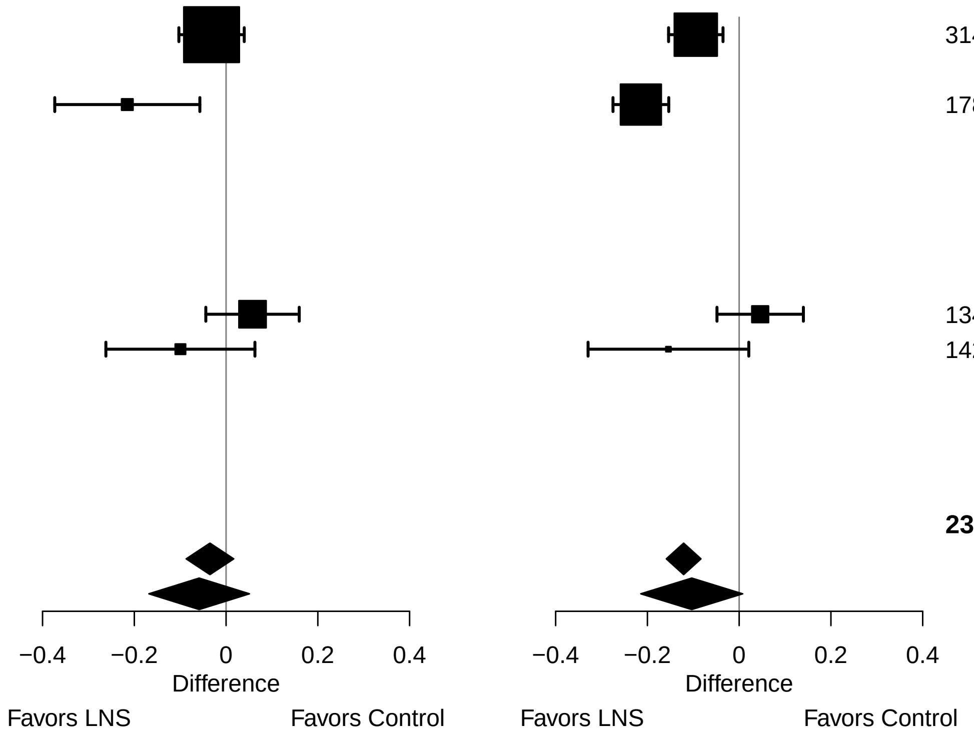

Supplemental figure 8E: Moderate-to-severe anemia prevalence difference

8E8: Stratified by Child high-dose vitamin A supplementation

P−for−interaction = 0.589
Difference in PDs = −0.01 (−0.04, 0.03)

		Received VitA						Not treated					
Country	Trial	LNS N	Control N	Control Prevalence	PD (95% CI)	Fixed W	Random W	LNS N	Control N	Control Prevalence	PD (95% CI)	Fixed W	Random W
Bangladesh	JiVitA−4 (35)												
Bangladesh	RDNS (36)	444	223	16.6	−0.08 (−0.13, −0.03)	0.17	0.17	105	49	16.3	−0.06 (−0.18, 0.06)	0.04	0.10
Bangladesh	WASH−B (37)												
Burkina Faso	iLiNS−Zinc (38)												
Burkina Faso	PROMIS CS (39)												
Ghana	GHANA (40)												
Ghana	iLiNS−DYAD−G (41)												
Kenya	WASH−B (42)	29	34	26.5	−0.06 (−0.27, 0.16)	0.01	0.01	263	212	19.8	−0.07 (−0.14, 0.00)	0.13	0.21
Madagascar	MAHAY (43)												
Malawi	iLiNS−DYAD−M (44)	75	136	25.7	0.00 (−0.13, 0.12)	0.03	0.03	133	289	22.5	0.06 (−0.03, 0.15)	0.08	0.15
Malawi	iLiNS−DOSE (45)												
Mali	PROMIS CS (46)												
Zimbabwe	SHINE (HIV−) (47)	874	833	9.6	−0.03 (−0.05, 0.00)	0.67	0.67	665	622	11.6	−0.05 (−0.08, −0.02)	0.65	0.36
Zimbabwe	SHINE (HIV+) (48)	165	127	8.7	−0.06 (−0.12, 0.00)	0.12	0.12	112	134	12.7	−0.06 (−0.14, 0.01)	0.11	0.19
		**1587**	**1353**		**I² = 0.00, Tau² = 0.00**			**1278**	**1306**		**I² = 0.38, Tau² = 0.00**		
**Fixed**					**−0.04 (−0.06, −0.02)**						**−0.04 (−0.07, −0.02)**		
**Random**					**−0.04 (−0.06, −0.02)**						**−0.04 (−0.08, 0.00)**		

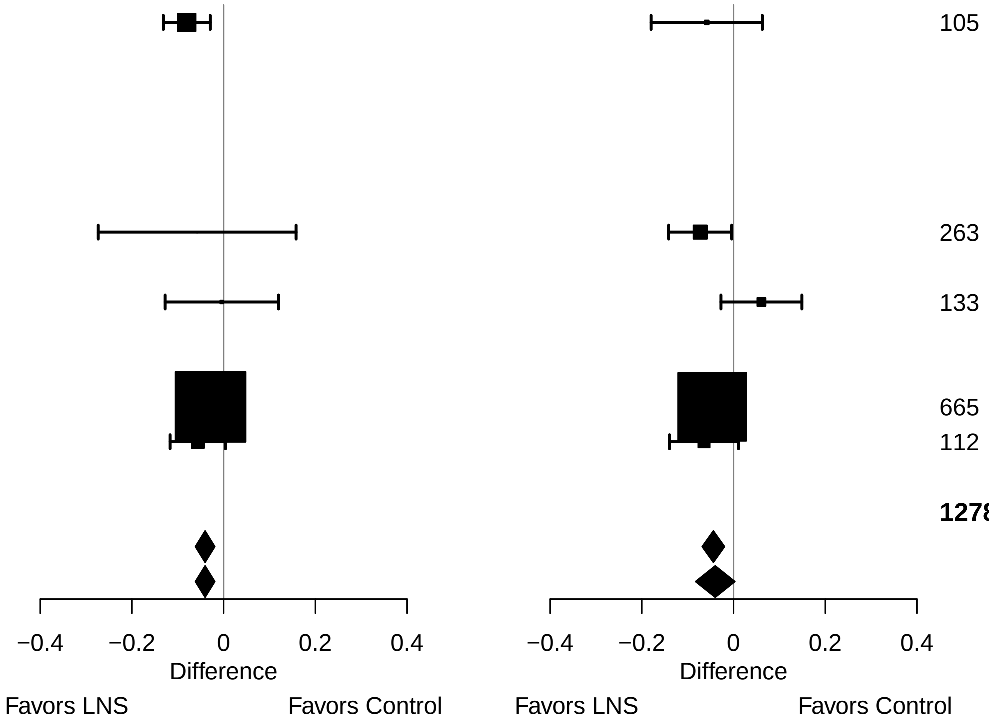

Supplemental figure 8E: Moderate-to-severe anemia prevalence difference

8E9: Stratified by Child inflammation

P−for−interaction = 0.777
Difference in PDs = −0.01 (−0.06, 0.05)

		Not inflamed						High AGP or CRP					
Country	Trial	LNS N	Control N	Control Prevalence	PD (95% CI)	Fixed W	Random W	LNS N	Control N	Control Prevalence	PD (95% CI)	Fixed W	Random W
Bangladesh	JiVitA−4 (35)												
Bangladesh	RDNS (36)	369	168	17.3	−0.09 (−0.15, −0.02)	0.16	0.23	180	104	15.4	−0.06 (−0.12, 0.00)	0.38	0.24
Bangladesh	WASH−B (37)												
Burkina Faso	iLiNS−Zinc (38)	131	33	69.7	−0.27 (−0.45, −0.09)	0.02	0.11	197	69	81.2	−0.22 (−0.33, −0.11)	0.12	0.18
Burkina Faso	PROMIS CS (39)												
Ghana	GHANA (40)												
Ghana	iLiNS−DYAD−G (41)												
Kenya	WASH−B (42)	157	135	14.1	−0.08 (−0.14, −0.01)	0.13	0.22	141	123	30.9	−0.09 (−0.20, 0.02)	0.12	0.17
Madagascar	MAHAY (43)												
Malawi	iLiNS−DYAD−M (44)	59	123	13.0	−0.01 (−0.12, 0.09)	0.06	0.18	138	269	29.4	0.01 (−0.08, 0.10)	0.17	0.20
Malawi	iLiNS−DOSE (45)												
Mali	PROMIS CS (46)												
Zimbabwe	SHINE (HIV−) (47)	414	354	5.6	0.00 (−0.03, 0.03)	0.64	0.26	105	99	14.1	−0.06 (−0.14, 0.03)	0.21	0.21
Zimbabwe	SHINE (HIV+) (48)												
		**1130**	**813**		**I^2 = 0.75, Tau^2 = 0.01**			**761**	**664**		**I^2 = 0.63, Tau^2 = 0.00**		
**Fixed**					**−0.03 (−0.05, 0.00)**						**−0.07 (−0.11, −0.03)**		
**Random**					**−0.07 (−0.15, 0.01)**						**−0.08 (−0.15, −0.01)**		

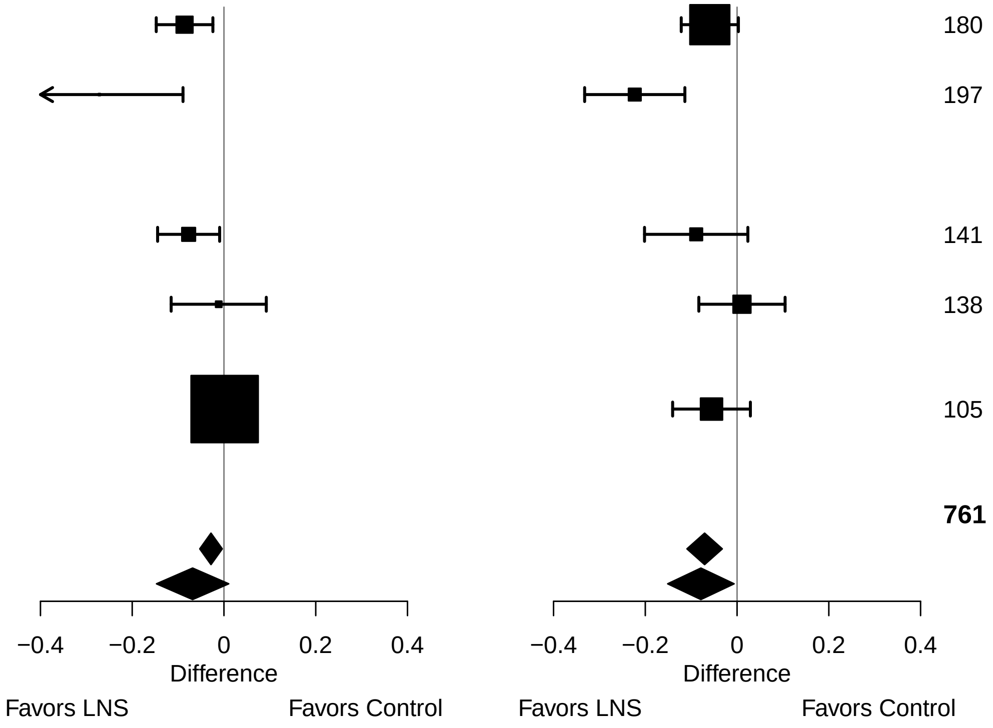

Supplemental figure 8F: Geometric mean ratio of ferritin concentration

8F1: Stratified by Maternal BMI

P−for−interaction = 0.221
Ratio of GMRs = 0.91 (0.79, 1.06)

		At least 20 kg/m²						Less than 20 kg/m²					
Country	Trial	LNS N	Control N	Control Median	GMR (95% CI)	Fixed W	Random W	LNS N	Control N	Control Median	GMR (95% CI)	Fixed W	Random W
Bangladesh	JiVitA−4 (35)												
Bangladesh	RDNS (36)	228	102	19.8	1.63 (1.40, 1.90)	0.33	0.33	300	160	21.6	1.43 (1.24, 1.64)	0.45	0.44
Bangladesh	WASH−B (37)	93	97	15.1	1.74 (1.37, 2.20)	0.14	0.14	119	81	16.7	1.74 (1.45, 2.09)	0.25	0.26
Burkina Faso	iLiNS−Zinc (38)	209	55	10.6	1.56 (1.23, 1.96)	0.14	0.14	106	41	8.9	1.56 (1.26, 1.94)	0.18	0.19
Burkina Faso	PROMIS CS (39)												
Ghana	GHANA (40)												
Ghana	iLiNS−DYAD−G (41)												
Kenya	WASH−B (42)	223	200	9.8	1.73 (1.50, 1.99)	0.39	0.39	64	51	13.1	1.41 (1.07, 1.86)	0.11	0.12
Madagascar	MAHAY (43)												
Malawi	iLiNS−DYAD−M (44)												
Malawi	iLiNS−DOSE (45)												
Mali	PROMIS CS (46)												
Zimbabwe	SHINE (HIV−) (47)												
Zimbabwe	SHINE (HIV+) (48)												
		**753**	**454**		**I² = 0.00, Tau² = 0.00**			**589**	**333**		**I² = 0.07, Tau² = 0.00**		
**Fixed**					**1.67 (1.53, 1.83)**						**1.52 (1.39, 1.67)**		
**Random**					**1.67 (1.53, 1.83)**						**1.52 (1.38, 1.68)**		

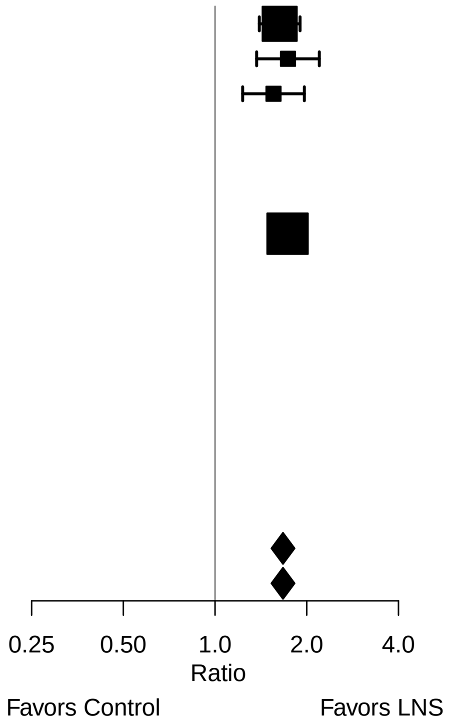

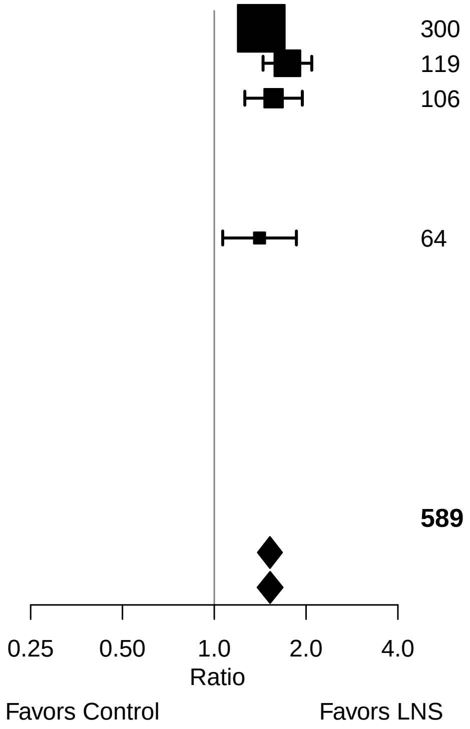

Supplemental figure 8F: Geometric mean ratio of ferritin concentration

8F2: Stratified by Maternal age

P−for−interaction = 0.112
Ratio of GMRs = 0.92 (0.83, 1.02)

		At least 25 y						Less than 25 y					
Country	Trial	LNS N	Control N	Control Median	GMR (95% CI)	Fixed W	Random W	LNS N	Control N	Control Median	GMR (95% CI)	Fixed W	Random W
Bangladesh	JiVitA−4 (35)	202	70	23.2	1.47 (1.17, 1.85)	0.11	0.11	250	73	25.3	1.33 (1.08, 1.63)	0.10	0.16
Bangladesh	RDNS (36)	152	81	21.0	1.71 (1.47, 1.98)	0.26	0.26	398	191	21.1	1.45 (1.32, 1.59)	0.48	0.21
Bangladesh	WASH−B (37)	79	80	17.7	1.50 (1.20, 1.88)	0.12	0.12	132	98	14.0	1.94 (1.64, 2.31)	0.15	0.17
Burkina Faso	iLiNS−Zinc (38)	178	50	9.7	1.56 (1.30, 1.89)	0.17	0.17	137	46	10.0	1.50 (1.19, 1.91)	0.08	0.14
Burkina Faso	PROMIS CS (39)												
Ghana	GHANA (40)	61	55	11.2	2.40 (1.32, 4.38)	0.02	0.02	22	21	7.6	2.64 (0.81, 8.64)	0.00	0.02
Ghana	iLiNS−DYAD−G (41)												
Kenya	WASH−B (42)	178	149	9.5	1.79 (1.55, 2.08)	0.26	0.26	116	109	10.5	1.53 (1.28, 1.82)	0.14	0.17
Madagascar	MAHAY (43)	47	25	10.1	1.58 (1.17, 2.14)	0.06	0.06	36	26	15.4	1.04 (0.79, 1.37)	0.06	0.13
Malawi	iLiNS−DYAD−M (44)												
Malawi	iLiNS−DOSE (45)												
Mali	PROMIS CS (46)												
Zimbabwe	SHINE (HIV−) (47)												
Zimbabwe	SHINE (HIV+) (48)												
		**897**	**510**		**I² = 0.00, Tau² = 0.00**			**1091**	**564**		**I² = 0.67, Tau² = 0.03**		
**Fixed**					**1.65 (1.53, 1.78)**						**1.49 (1.39, 1.59)**		
**Random**					**1.65 (1.53, 1.78)**						**1.48 (1.27, 1.72)**		

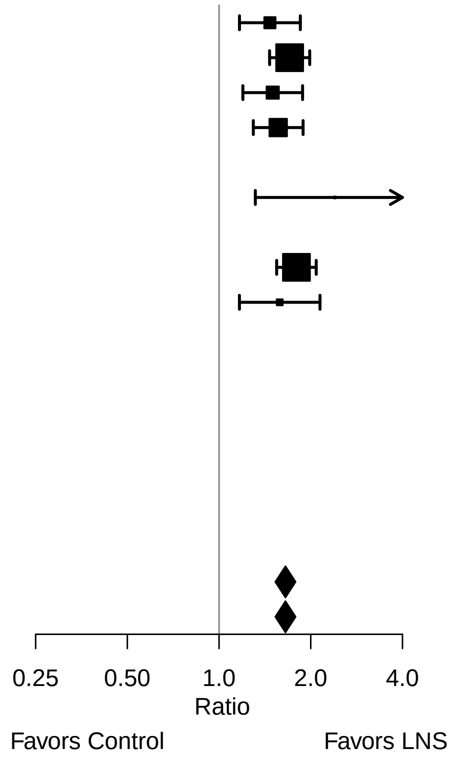

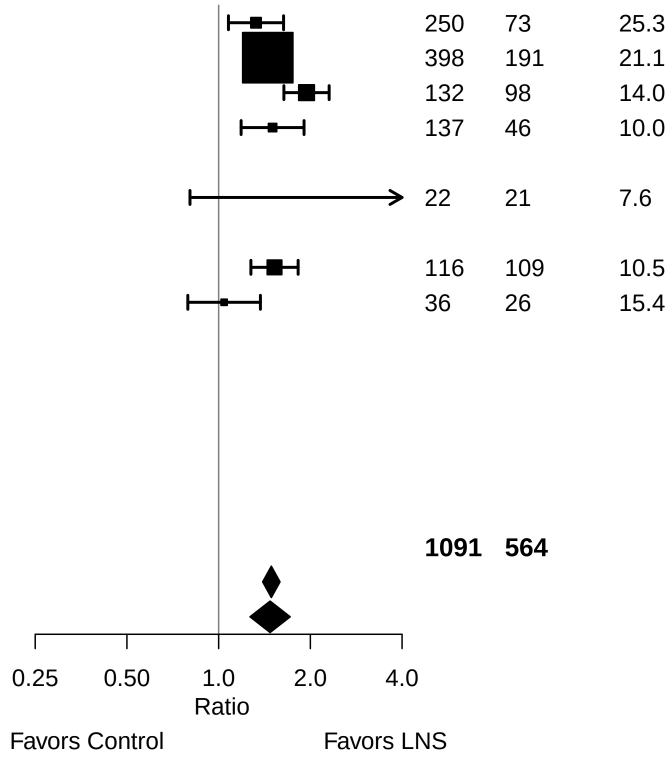

Supplemental figure 8F: Geometric mean ratio of ferritin concentration

8F3: Stratified by Maternal education

P−for−interaction = 0.928
Ratio of GMRs = 0.99 (0.89, 1.12)

		Primary or greater						Incomplete or no formal					
		**LNS**	**Control**	**Control**	**GMR**	**Fixed**	**Random**	**LNS**	**Control**	**Control**	**GMR**	**Fixed**	**Random**
Country	Trial	N	N	Median	(95% CI)	W	W	N	N	Median	(95% CI)	W	W
Bangladesh	JiVitA−4 (35)	298	93	21.0	1.57 (1.28, 1.92)	0.10	0.17	156	51	29.8	1.10 (0.88, 1.38)	0.16	0.19
Bangladesh	RDNS (36)	406	195	21.2	1.46 (1.34, 1.60)	0.53	0.22	144	77	18.7	1.66 (1.42, 1.94)	0.34	0.26
Bangladesh	WASH−B (37)	149	139	15.0	1.84 (1.56, 2.17)	0.15	0.19	63	39	16.7	1.45 (1.05, 1.99)	0.08	0.13
Burkina Faso	iLiNS−Zinc (38)												
Burkina Faso	PROMIS CS (39)												
Ghana	GHANA (40)	76	65	9.4	2.37 (1.35, 4.15)	0.01	0.06	7	13	11.2	1.70 (0.28, 10.25)	0.00	0.01
Ghana	iLiNS−DYAD−G (41)												
Kenya	WASH−B (42)	143	117	10.5	1.71 (1.39, 2.11)	0.09	0.17	154	142	10.0	1.69 (1.43, 1.99)	0.30	0.25
Madagascar	MAHAY (43)	16	16	15.4	1.17 (0.98, 1.41)	0.12	0.18	67	35	11.3	1.35 (1.04, 1.76)	0.12	0.16
Malawi	iLiNS−DYAD−M (44)												
Malawi	iLiNS−DOSE (45)												
Mali	PROMIS CS (46)												
Zimbabwe	SHINE (HIV−) (47)												
Zimbabwe	SHINE (HIV+) (48)												
		**1088**	**625**		**I^2 = 0.71, Tau^2 = 0.03**			**591**	**357**		**I^2 = 0.55, Tau^2 = 0.01**		
**Fixed**					**1.52 (1.42, 1.62)**						**1.51 (1.38, 1.65)**		
**Random**					**1.57 (1.34, 1.84)**						**1.46 (1.27, 1.69)**		

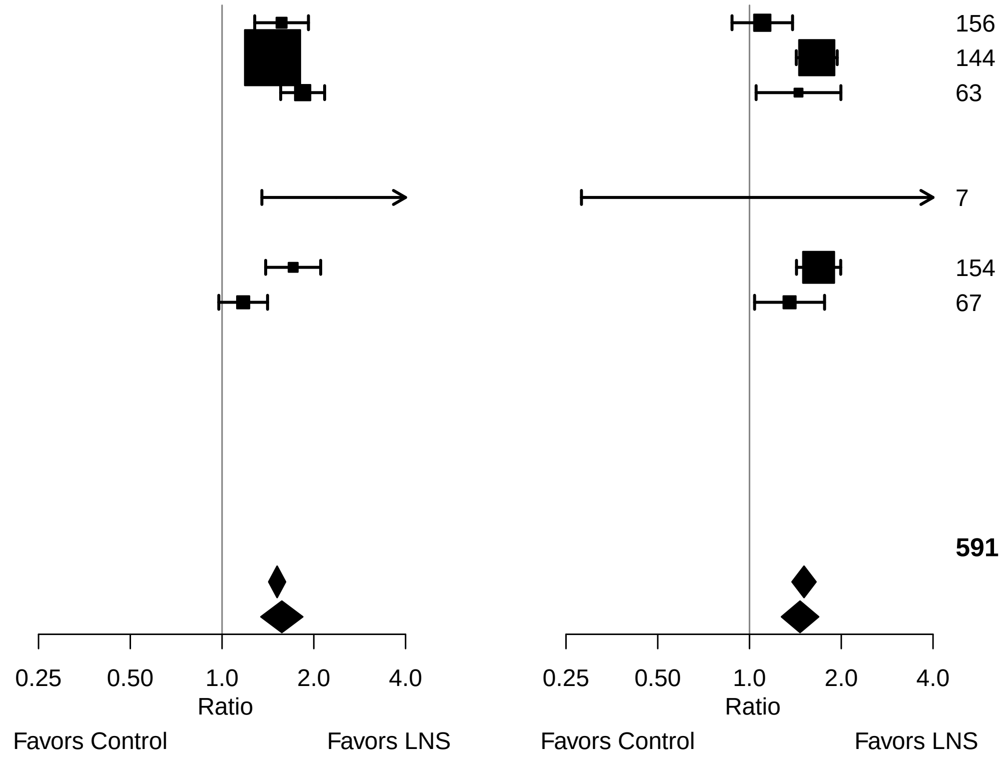

Supplemental figure 8F: Geometric mean ratio of ferritin concentration

8F4: Stratified by Child sex

P−for−interaction = 0.649
Ratio of GMRs = 0.98 (0.88, 1.08)

Country	Trial	Male LNS N	Male Control N	Male Control Median	Male GMR (95% CI)	Male Fixed W	Male Random W	Female LNS N	Female Control N	Female Control Median	Female GMR (95% CI)	Female Fixed W	Female Random W
Bangladesh	JiVitA−4 (35)	235	70	21.1	1.45 (1.15, 1.84)	0.10	0.14	220	74	27.7	1.33 (1.09, 1.64)	0.12	0.15
Bangladesh	RDNS (36)	266	131	18.1	1.53 (1.38, 1.71)	0.47	0.25	281	141	22.5	1.51 (1.33, 1.71)	0.31	0.22
Bangladesh	WASH−B (37)	105	85	16.2	1.67 (1.32, 2.10)	0.10	0.15	107	93	15.7	1.81 (1.52, 2.17)	0.16	0.17
Burkina Faso	iLiNS−Zinc (38)	150	49	10.5	1.62 (1.23, 2.14)	0.07	0.12	165	47	9.5	1.51 (1.21, 1.89)	0.11	0.14
Burkina Faso	PROMIS CS (39)												
Ghana	GHANA (40)	51	37	6.9	3.87 (1.83, 8.18)	0.01	0.02	32	45	16.5	1.93 (0.94, 3.98)	0.01	0.02
Ghana	iLiNS−DYAD−G (41)												
Kenya	WASH−B (42)	146	125	10.8	1.65 (1.38, 1.97)	0.17	0.19	152	134	9.9	1.74 (1.49, 2.03)	0.21	0.19
Madagascar	MAHAY (43)	34	22	10.6	1.42 (1.09, 1.85)	0.08	0.13	49	29	14.2	1.21 (0.93, 1.57)	0.07	0.11
Malawi	iLiNS−DYAD−M (44)												
Malawi	iLiNS−DOSE (45)												
Mali	PROMIS CS (46)												
Zimbabwe	SHINE (HIV−) (47)												
Zimbabwe	SHINE (HIV+) (48)												
		**987**	**519**		**I² = 0.18, Tau² = 0.01**			**1006**	**563**		**I² = 0.46, Tau² = 0.01**		
**Fixed**					**1.57 (1.46, 1.69)**						**1.56 (1.45, 1.67)**		
**Random**					**1.59 (1.41, 1.79)**						**1.54 (1.38, 1.72)**		

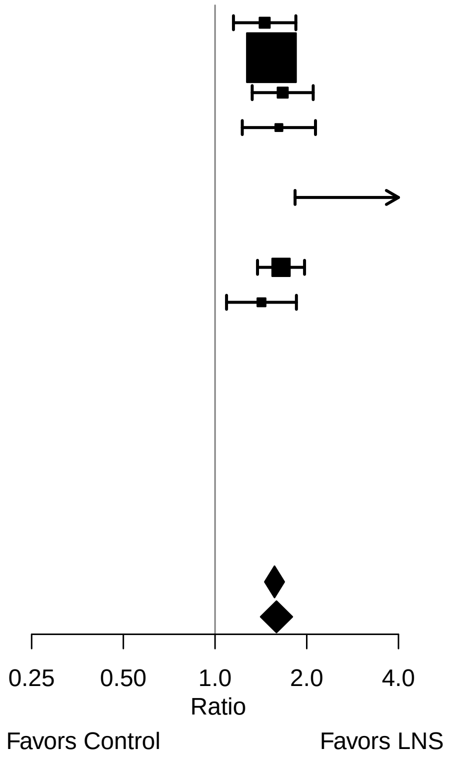

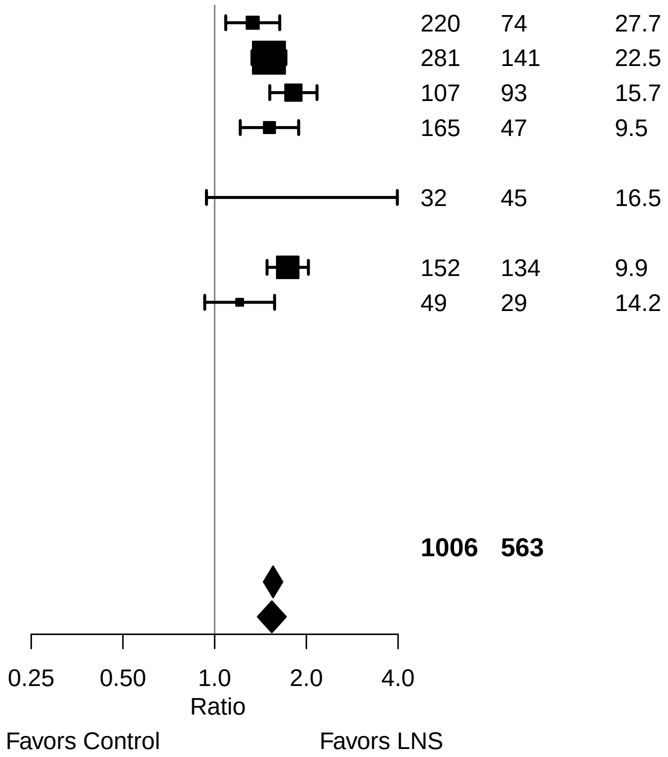

Supplemental figure 8F: Geometric mean ratio of ferritin concentration

8F5: Stratified by Child birth order

P−for−interaction = 0.040
Ratio of GMRs = 0.88 (0.78, 0.99)

		Later born						Firstborn					
Country	Trial	LNS N	Control N	Control Median	GMR (95% CI)	Fixed W	Random W	LNS N	Control N	Control Median	GMR (95% CI)	Fixed W	Random W
Bangladesh	JiVitA−4 (35)	114	32	28.6	1.26 (0.88, 1.80)	0.03	0.03	340	111	23.2	1.42 (1.20, 1.68)	0.27	0.19
Bangladesh	RDNS (36)	340	171	19.8	1.64 (1.50, 1.80)	0.47	0.47	210	101	22.5	1.33 (1.14, 1.56)	0.31	0.19
Bangladesh	WASH−B (37)	124	119	16.7	1.75 (1.44, 2.12)	0.11	0.11	86	57	15.1	1.74 (1.36, 2.22)	0.13	0.16
Burkina Faso	iLiNS−Zinc (38)	249	71	9.2	1.59 (1.30, 1.94)	0.10	0.10	66	25	11.4	1.42 (1.11, 1.83)	0.12	0.16
Burkina Faso	PROMIS CS (39)												
Ghana	GHANA (40)	54	50	11.6	2.27 (1.25, 4.11)	0.01	0.01	29	29	7.6	2.68 (0.96, 7.46)	0.01	0.03
Ghana	iLiNS−DYAD−G (41)												
Kenya	WASH−B (42)	237	213	10.1	1.71 (1.49, 1.97)	0.21	0.21	60	46	9.3	1.63 (1.23, 2.17)	0.09	0.14
Madagascar	MAHAY (43)	61	37	11.3	1.43 (1.12, 1.82)	0.07	0.07	21	11	16.8	0.91 (0.67, 1.24)	0.08	0.13
Malawi	iLiNS−DYAD−M (44)												
Malawi	iLiNS−DOSE (45)												
Mali	PROMIS CS (46)												
Zimbabwe	SHINE (HIV−) (47)												
Zimbabwe	SHINE (HIV+) (48)												
		**1179**	**693**		**I² = 0.00, Tau² = 0.00**			**812**	**380**		**I² = 0.55, Tau² = 0.03**		
**Fixed**					**1.64 (1.54, 1.75)**						**1.40 (1.29, 1.53)**		
**Random**					**1.64 (1.54, 1.75)**						**1.41 (1.19, 1.68)**		

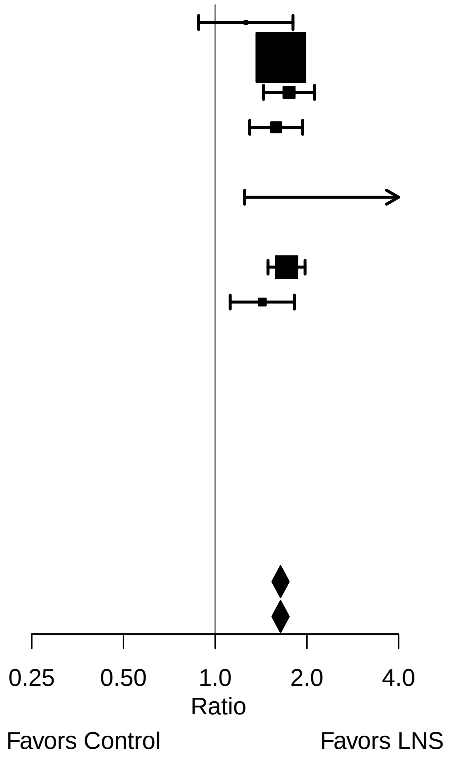

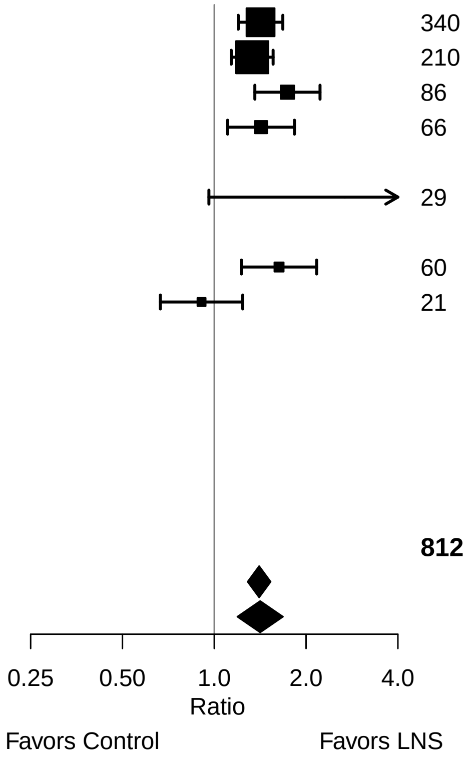

Supplemental figure 8F: Geometric mean ratio of ferritin concentration

8F6: Stratified by Child baseline acute malnutrition

P−for−interaction = 0.023
Ratio of GMRs = 1.27 (1.03, 1.56)

		No						Yes					
		**LNS**	**Control**	**Control**	**GMR**	**Fixed**	**Random**	**LNS**	**Control**	**Control**	**GMR**	**Fixed**	**Random**
Country	Trial	N	N	Median	(95% CI)	W	W	N	N	Median	(95% CI)	W	W
Bangladesh	JiVitA−4 (35)	363	112	23.1	1.37 (1.17, 1.61)	0.17	0.17	90	32	26.4	1.43 (0.92, 2.20)	0.19	0.22
Bangladesh	RDNS (36)	493	243	21.0	1.51 (1.40, 1.64)	0.72	0.72	35	12	17.0	1.76 (1.28, 2.41)	0.36	0.36
Bangladesh	WASH−B (37)												
Burkina Faso	iLiNS−Zinc (38)	229	72	10.7	1.38 (1.12, 1.69)	0.10	0.10	86	24	8.1	2.16 (1.63, 2.87)	0.45	0.42
Burkina Faso	PROMIS CS (39)												
Ghana	GHANA (40)												
Ghana	iLiNS−DYAD−G (41)												
Kenya	WASH−B (42)												
Madagascar	MAHAY (43)												
Malawi	iLiNS−DYAD−M (44)												
Malawi	iLiNS−DOSE (45)												
Mali	PROMIS CS (46)												
Zimbabwe	SHINE (HIV−) (47)												
Zimbabwe	SHINE (HIV+) (48)												
		**1085**	**427**		**I² = 0.00, Tau² = 0.00**			**211**	**68**		**I² = 0.25, Tau² = 0.01**		
**Fixed**					**1.47 (1.38, 1.57)**						**1.85 (1.53, 2.24)**		
**Random**					**1.47 (1.38, 1.57)**						**1.83 (1.46, 2.29)**		

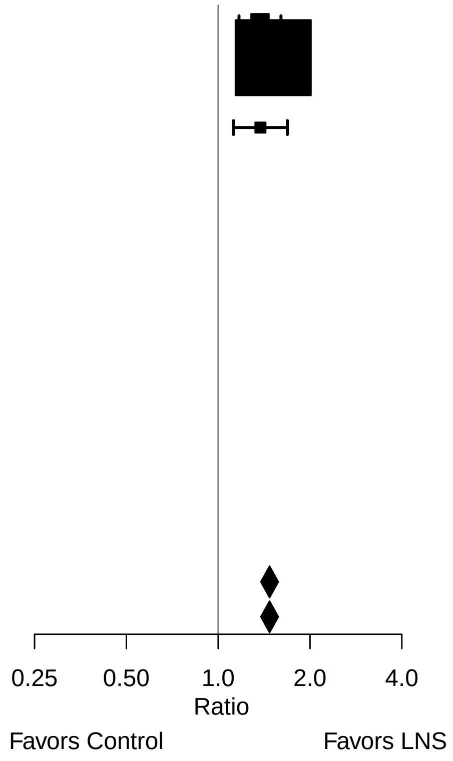

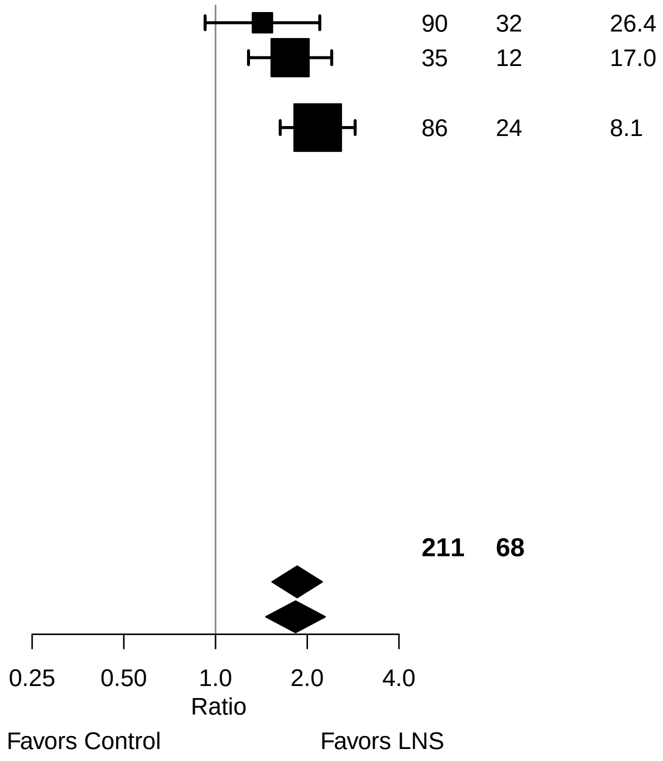

Supplemental figure 8F: Geometric mean ratio of ferritin concentration

8F7: Stratified by Child baseline anemia (insufficient comparisons)

Supplemental figure 8F: Geometric mean ratio of ferritin concentration

8F8: Stratified by Child high-dose vitamin A supplementation

P−for−interaction = 0.348
Ratio of GMRs = 1.08 (0.92, 1.27)

		Received VitA						Not treated					
		**LNS**	**Control**	**Control**	**GMR**	**Fixed**	**Random**	**LNS**	**Control**	**Control**	**GMR**	**Fixed**	**Random**
Country	Trial	N	N	Median	(95% CI)	W	W	N	N	Median	(95% CI)	W	W
Bangladesh	JiVitA−4 (35)	140	37	24.2	1.42 (1.11, 1.82)	0.08	0.23	133	41	22.6	1.27 (0.98, 1.65)	0.18	0.21
Bangladesh	RDNS (36)	444	223	21.3	1.47 (1.36, 1.59)	0.84	0.50	105	49	17.6	1.74 (1.40, 2.17)	0.24	0.27
Bangladesh	WASH−B (37)	16	16	13.1	1.65 (0.99, 2.73)	0.02	0.08	15	9	17.5	1.51 (0.89, 2.58)	0.04	0.06
Burkina Faso	iLiNS−Zinc (38)												
Burkina Faso	PROMIS CS (39)												
Ghana	GHANA (40)	39	53	8.5	3.37 (1.53, 7.45)	0.01	0.03	44	26	14.0	1.26 (0.64, 2.45)	0.03	0.04
Ghana	iLiNS−DYAD−G (41)												
Kenya	WASH−B (42)	26	31	12.5	1.49 (1.08, 2.05)	0.05	0.16	223	180	9.5	1.74 (1.49, 2.03)	0.51	0.43
Madagascar	MAHAY (43)												
Malawi	iLiNS−DYAD−M (44)												
Malawi	iLiNS−DOSE (45)												
Mali	PROMIS CS (46)												
Zimbabwe	SHINE (HIV−) (47)												
Zimbabwe	SHINE (HIV+) (48)												
		**665**	**360**		**I^2 = 0.10, Tau^2 = 0.01**			**520**	**305**		**I^2 = 0.23, Tau^2 = 0.00**		
**Fixed**					**1.48 (1.38, 1.59)**						**1.62 (1.45, 1.81)**		
**Random**					**1.52 (1.31, 1.77)**						**1.60 (1.40, 1.82)**		

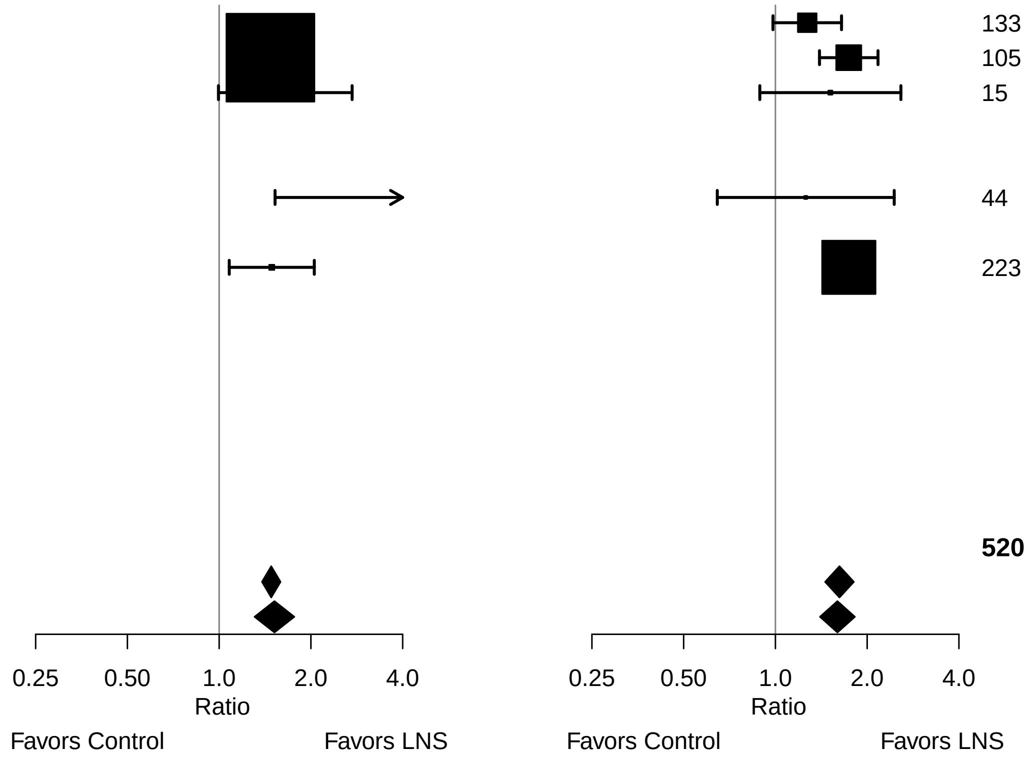

Supplemental figure 8F: Geometric mean ratio of ferritin concentration

8F9: Stratified by Child inflammation

P−for−interaction = 0.089
Ratio of GMRs = 0.90 (0.81, 1.02)

		Not inflamed						High AGP or CRP					
Country	Trial	LNS N	Control N	Control Median	GMR (95% CI)	Fixed W	Random W	LNS N	Control N	Control Median	GMR (95% CI)	Fixed W	Random W
Bangladesh	JiVitA−4 (35)	185	46	23.4	1.53 (1.09, 2.14)	0.04	0.12	270	98	24.4	1.31 (1.08, 1.60)	0.16	0.18
Bangladesh	RDNS (36)	370	168	19.3	1.58 (1.42, 1.77)	0.39	0.18	180	104	21.9	1.41 (1.23, 1.61)	0.34	0.25
Bangladesh	WASH−B (37)	165	136	13.3	1.85 (1.61, 2.14)	0.25	0.18	47	42	20.0	1.42 (1.04, 1.93)	0.07	0.10
Burkina Faso	iLiNS−Zinc (38)	124	31	9.4	1.86 (1.47, 2.35)	0.09	0.15	191	65	9.7	1.40 (1.12, 1.76)	0.12	0.15
Burkina Faso	PROMIS CS (39)												
Ghana	GHANA (40)	69	66	7.6	3.35 (1.87, 5.97)	0.01	0.07	14	16	33.0	0.79 (0.28, 2.22)	0.01	0.01
Ghana	iLiNS−DYAD−G (41)												
Kenya	WASH−B (42)	157	135	10.1	1.56 (1.28, 1.89)	0.13	0.16	141	124	10.0	1.86 (1.58, 2.18)	0.24	0.22
Madagascar	MAHAY (43)	44	32	11.4	1.23 (0.97, 1.57)	0.08	0.15	39	19	16.1	1.36 (0.98, 1.89)	0.06	0.09
Malawi	iLiNS−DYAD−M (44)												
Malawi	iLiNS−DOSE (45)												
Mali	PROMIS CS (46)												
Zimbabwe	SHINE (HIV−) (47)												
Zimbabwe	SHINE (HIV+) (48)												
		**1114**	**614**		**I^2 = 0.63, Tau^2 = 0.04**			**882**	**468**		**I^2 = 0.48, Tau^2 = 0.01**		
**Fixed**					**1.65 (1.53, 1.76)**						**1.48 (1.37, 1.60)**		
**Random**					**1.68 (1.40, 2.01)**						**1.46 (1.31, 1.64)**		

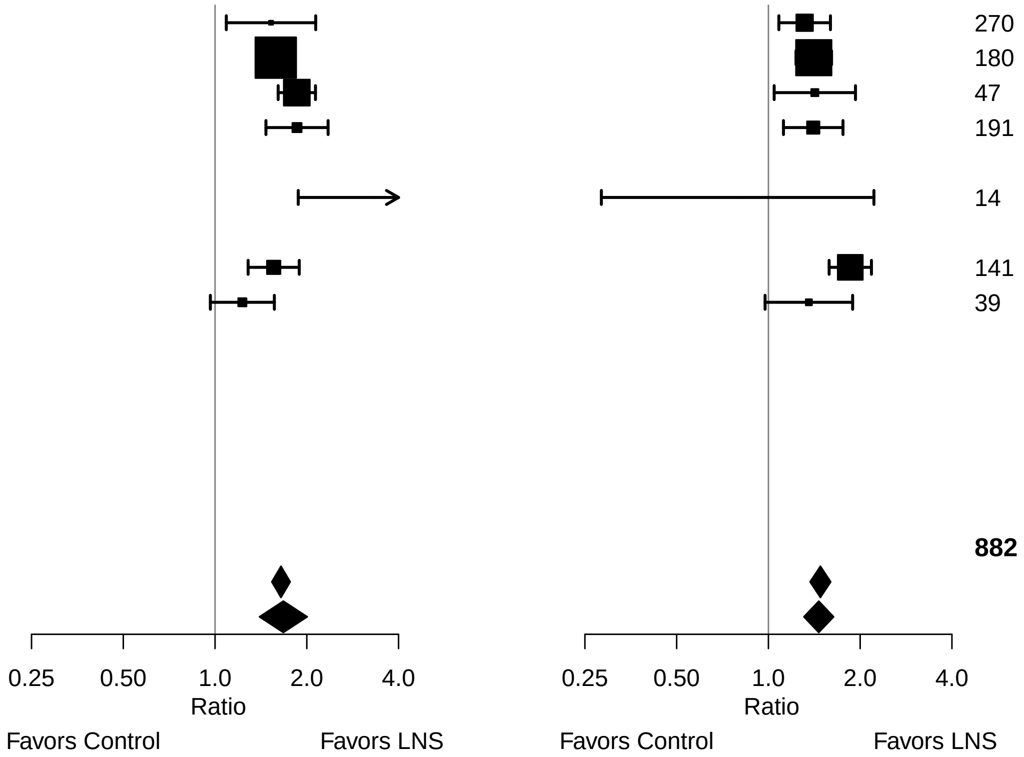

Supplemental figure 8G: Iron deficiency (ferritin < 12 µg/L) prevalence ratio

8G1: Stratified by Maternal BMI

P−for−interaction = 0.786
Ratio of PRs = 0.96 (0.71, 1.30)

		At least 20 kg/m²						Less than 20 kg/m²					
Country	Trial	LNS N	Control N	Control Prevalence	PR (95% CI)	Fixed W	Random W	LNS N	Control N	Control Prevalence	PR (95% CI)	Fixed W	Random W
Bangladesh	JiVitA−4 (35)												
Bangladesh	RDNS (36)	228	102	22.5	0.23 (0.12, 0.44)	0.10	0.21	300	160	21.2	0.44 (0.28, 0.70)	0.22	0.26
Bangladesh	WASH−B (37)	93	97	36.1	0.30 (0.15, 0.61)	0.08	0.19	119	81	34.6	0.19 (0.10, 0.38)	0.10	0.19
Burkina Faso	iLiNS−Zinc (38)	209	55	54.1	0.69 (0.50, 0.94)	0.40	0.30	106	41	64.4	0.50 (0.37, 0.69)	0.49	0.31
Burkina Faso	PROMIS CS (39)												
Ghana	GHANA (40)												
Ghana	iLiNS−DYAD−G (41)												
Kenya	WASH−B (42)	223	200	61.5	0.41 (0.30, 0.56)	0.42	0.30	64	51	45.1	0.52 (0.31, 0.86)	0.19	0.24
Madagascar	MAHAY (43)												
Malawi	iLiNS−DYAD−M (44)												
Malawi	iLiNS−DOSE (45)												
Mali	PROMIS CS (46)												
Zimbabwe	SHINE (HIV−) (47)												
Zimbabwe	SHINE (HIV+) (48)												
		**753**	**454**		**I² = 0.77, Tau² = 0.16**			**589**	**333**		**I² = 0.56, Tau² = 0.12**		
**Fixed**					**0.47 (0.38, 0.57)**						**0.45 (0.36, 0.55)**		
**Random**					**0.40 (0.25, 0.63)**						**0.41 (0.27, 0.62)**		

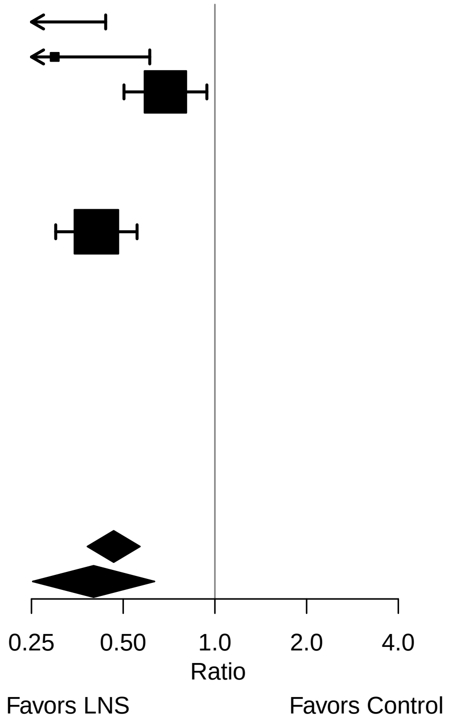

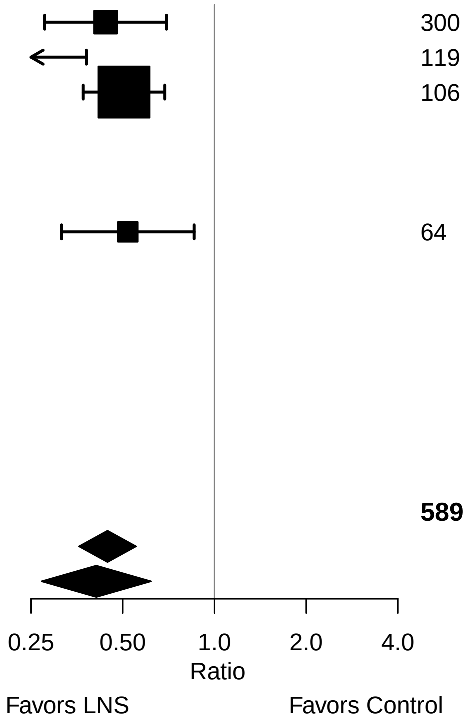

Supplemental figure 8G: Iron deficiency (ferritin < 12 µg/L) prevalence ratio

8G2: Stratified by Maternal age

P−for−interaction = 0.131
Ratio of PRs = 1.19 (0.95, 1.48)

		At least 25 y						Less than 25 y					
Country	Trial	LNS N	Control N	Control Prevalence	PR (95% CI)	Fixed W	Random W	LNS N	Control N	Control Prevalence	PR (95% CI)	Fixed W	Random W
Bangladesh	JiVitA−4 (35)	202	70	22.9	0.26 (0.13, 0.52)	0.06	0.07	250	73	21.9	0.44 (0.23, 0.85)	0.07	0.12
Bangladesh	RDNS (36)	152	81	22.2	0.27 (0.12, 0.60)	0.04	0.05	398	191	22.0	0.37 (0.25, 0.54)	0.20	0.17
Bangladesh	WASH−B (37)	79	80	27.5	0.41 (0.20, 0.87)	0.05	0.07	132	98	41.8	0.16 (0.08, 0.31)	0.07	0.12
Burkina Faso	iLiNS−Zinc (38)	178	50	59.3	0.55 (0.43, 0.72)	0.41	0.35	137	46	57.7	0.71 (0.53, 0.95)	0.34	0.19
Burkina Faso	PROMIS CS (39)												
Ghana	GHANA (40)	61	55	52.7	0.47 (0.28, 0.77)	0.11	0.13	22	21	61.9	0.59 (0.31, 1.12)	0.07	0.12
Ghana	iLiNS−DYAD−G (41)												
Kenya	WASH−B (42)	178	149	60.4	0.40 (0.29, 0.55)	0.29	0.27	116	109	56.9	0.44 (0.30, 0.65)	0.19	0.17
Madagascar	MAHAY (43)	47	25	60.0	0.32 (0.14, 0.71)	0.04	0.06	36	26	42.3	0.46 (0.22, 0.95)	0.06	0.11
Malawi	iLiNS−DYAD−M (44)												
Malawi	iLiNS−DOSE (45)												
Mali	PROMIS CS (46)												
Zimbabwe	SHINE (HIV−) (47)												
Zimbabwe	SHINE (HIV+) (48)												
		**897**	**510**		**I² = 0.21, Tau² = 0.01**			**1091**	**564**		**I² = 0.69, Tau² = 0.13**		
**Fixed**					**0.44 (0.37, 0.52)**						**0.48 (0.40, 0.57)**		
**Random**					**0.43 (0.35, 0.52)**						**0.43 (0.31, 0.60)**		

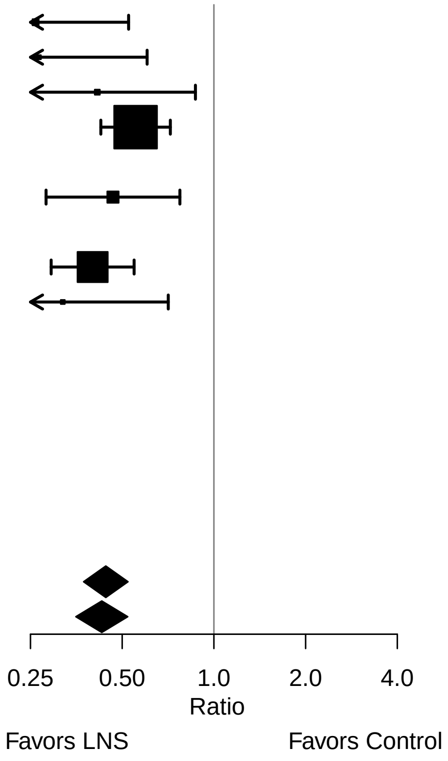

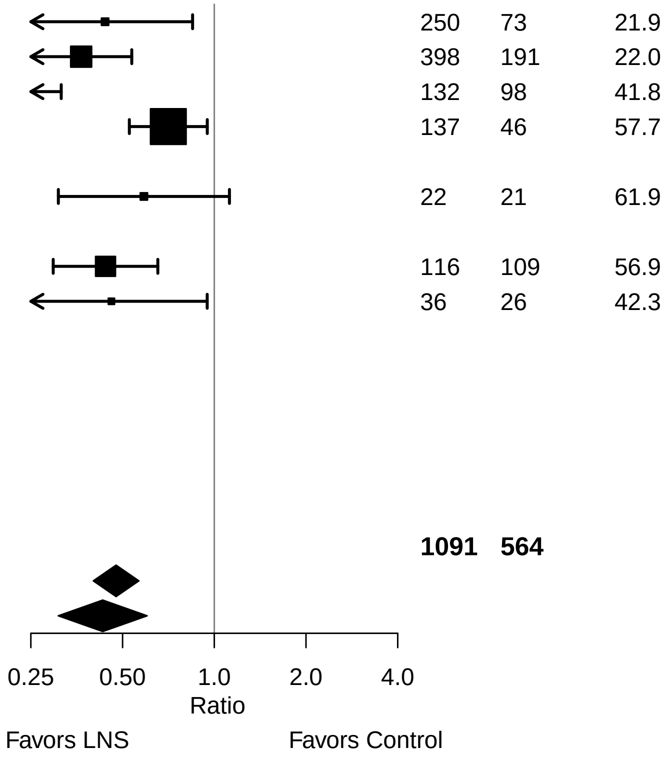

Supplemental figure 8G: Iron deficiency (ferritin < 12 µg/L) prevalence ratio

8G3: Stratified by Maternal education

P−for−interaction = 0.783
Ratio of PRs = 0.95 (0.66, 1.37)

					**Primary or greater**						**Incomplete or no formal**		
		**LNS**	**Control**	**Control**	**PR**	**Fixed**	**Random**	**LNS**	**Control**	**Control**	**PR**	**Fixed**	**Random**
Country	Trial	N	N	Prevalence	(95% CI)	W	W	N	N	Prevalence	(95% CI)	W	W
Bangladesh	JiVitA−4 (35)	298	93	26.9	0.31 (0.18, 0.55)	0.17	0.18	156	51	13.7	0.51 (0.20, 1.32)	0.08	0.08
Bangladesh	RDNS (36)	406	195	18.5	0.40 (0.26, 0.62)	0.28	0.28	144	77	31.2	0.25 (0.13, 0.45)	0.20	0.20
Bangladesh	WASH−B (37)	149	139	38.1	0.23 (0.12, 0.42)	0.14	0.16	63	39	25.6	0.31 (0.14, 0.70)	0.11	0.11
Burkina Faso	iLiNS−Zinc (38)												
Burkina Faso	PROMIS CS (39)												
Ghana	GHANA (40)												
Ghana	iLiNS−DYAD−G (41)												
Kenya	WASH−B (42)	143	117	55.6	0.43 (0.30, 0.62)	0.41	0.38	154	142	62.0	0.40 (0.28, 0.56)	0.61	0.61
Madagascar	MAHAY (43)												
Malawi	iLiNS−DYAD−M (44)												
Malawi	iLiNS−DOSE (45)												
Mali	PROMIS CS (46)												
Zimbabwe	SHINE (HIV−) (47)												
Zimbabwe	SHINE (HIV+) (48)												
		**996**	**544**		**I² = 0.13, Tau² = 0.01**			**517**	**309**		**I² = 0.00, Tau² = 0.00**		
**Fixed**					**0.36 (0.29, 0.46)**						**0.36 (0.27, 0.47)**		
**Random**					**0.36 (0.28, 0.46)**						**0.36 (0.27, 0.47)**		

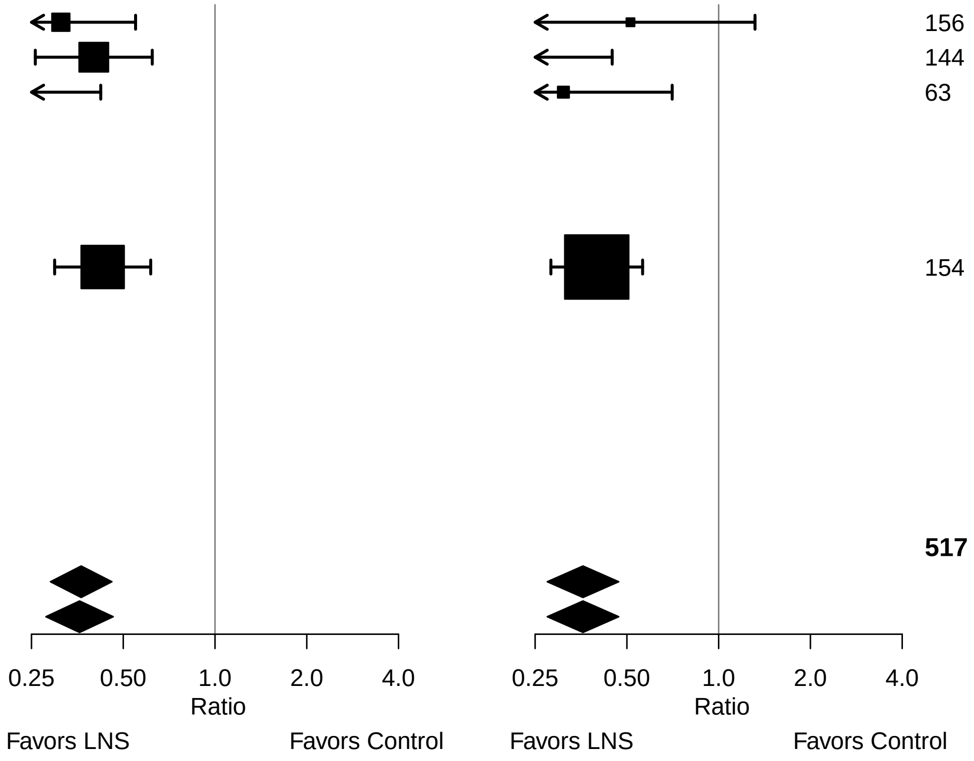

Supplemental figure 8G: Iron deficiency (ferritin < 12 µg/L) prevalence ratio

8G4: Stratified by Child sex

P−for−interaction = 0.771
Ratio of PRs = 1.04 (0.80, 1.36)

		Male						Female					
Country	Trial	LNS N	Control N	Control Prevalence	PR (95% CI)	Fixed W	Random W	LNS N	Control N	Control Prevalence	PR (95% CI)	Fixed W	Random W
Bangladesh	JiVitA−4 (35)	235	70	27.1	0.31 (0.17, 0.56)	0.10	0.11	220	74	17.6	0.41 (0.19, 0.89)	0.06	0.08
Bangladesh	RDNS (36)	266	131	25.2	0.36 (0.23, 0.55)	0.18	0.18	281	141	19.1	0.32 (0.18, 0.55)	0.11	0.14
Bangladesh	WASH−B (37)	105	85	37.6	0.25 (0.12, 0.54)	0.06	0.07	107	93	33.3	0.22 (0.11, 0.46)	0.07	0.09
Burkina Faso	iLiNS−Zinc (38)	150	49	55.6	0.62 (0.42, 0.92)	0.21	0.20	165	47	61.5	0.61 (0.45, 0.82)	0.37	0.27
Burkina Faso	PROMIS CS (39)												
Ghana	GHANA (40)	51	37	75.7	0.41 (0.27, 0.65)	0.17	0.17	32	45	40.0	0.55 (0.26, 1.15)	0.06	0.09
Ghana	iLiNS−DYAD−G (41)												
Kenya	WASH−B (42)	146	125	58.4	0.43 (0.30, 0.62)	0.26	0.23	152	134	59.7	0.40 (0.28, 0.57)	0.25	0.23
Madagascar	MAHAY (43)	34	22	59.1	0.25 (0.09, 0.70)	0.03	0.04	49	29	44.8	0.50 (0.26, 0.96)	0.08	0.11
Malawi	iLiNS−DYAD−M (44)												
Malawi	iLiNS−DOSE (45)												
Mali	PROMIS CS (46)												
Zimbabwe	SHINE (HIV−) (47)												
Zimbabwe	SHINE (HIV+) (48)												
		**987**	**519**		**I² = 0.24, Tau² = 0.02**			**1006**	**563**		**I² = 0.39, Tau² = 0.03**		
**Fixed**					**0.41 (0.35, 0.50)**						**0.45 (0.38, 0.55)**		
**Random**					**0.41 (0.33, 0.50)**						**0.43 (0.34, 0.55)**		

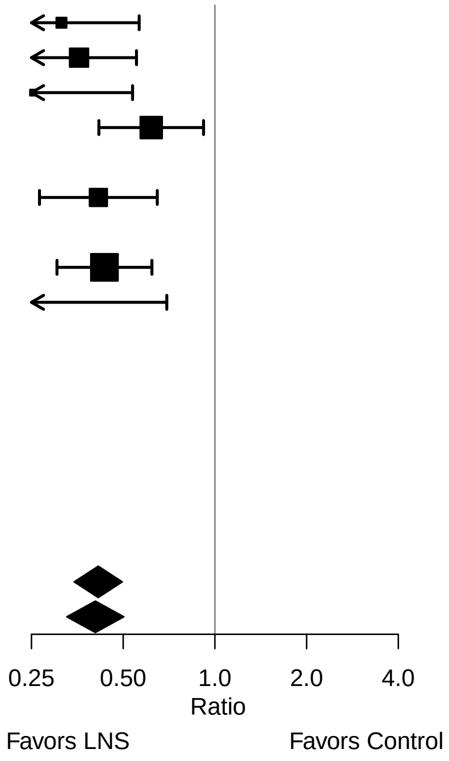

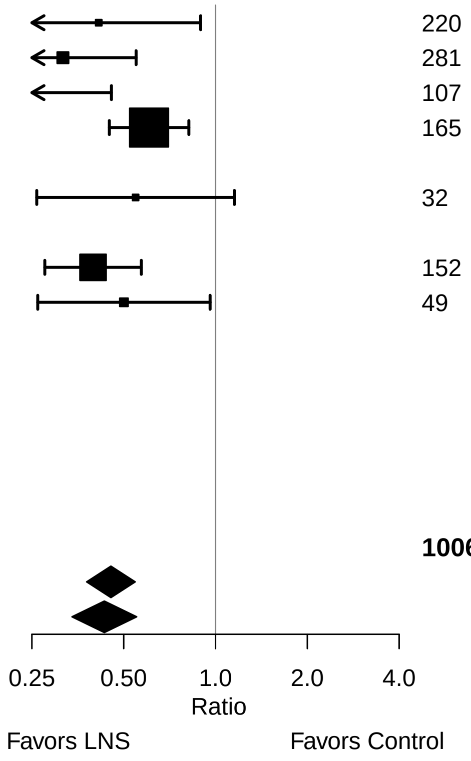

Supplemental figure 8G: Iron deficiency (ferritin < 12 µg/L) prevalence ratio

8G5: Stratified by Child birth order

P−for−interaction = 0.101
Ratio of PRs = 1.22 (0.96, 1.56)

		Later born						Firstborn					
Country	Trial	LNS N	Control N	Control Prevalence	PR (95% CI)	Fixed W	Random W	LNS N	Control N	Control Prevalence	PR (95% CI)	Fixed W	Random W
Bangladesh	JiVitA−4 (35)	114	32	25.0	0.32 (0.11, 0.92)	0.02	0.04	340	111	21.6	0.37 (0.21, 0.65)	0.11	0.17
Bangladesh	RDNS (36)	340	171	22.8	0.32 (0.21, 0.49)	0.15	0.18	210	101	20.8	0.37 (0.20, 0.66)	0.11	0.16
Bangladesh	WASH−B (37)	124	119	35.3	0.27 (0.14, 0.53)	0.06	0.09	86	57	36.8	0.16 (0.07, 0.37)	0.05	0.12
Burkina Faso	iLiNS−Zinc (38)	249	71	61.3	0.57 (0.43, 0.74)	0.36	0.30	66	25	51.6	0.82 (0.62, 1.08)	0.48	0.21
Burkina Faso	PROMIS CS (39)												
Ghana	GHANA (40)	54	50	52.0	0.43 (0.24, 0.75)	0.08	0.11	29	29	58.6	0.65 (0.37, 1.13)	0.11	0.17
Ghana	iLiNS−DYAD−G (41)												
Kenya	WASH−B (42)	237	213	58.2	0.41 (0.31, 0.55)	0.32	0.28	60	46	63.0	0.40 (0.24, 0.66)	0.14	0.17
Madagascar	MAHAY (43)												
Malawi	iLiNS−DYAD−M (44)												
Malawi	iLiNS−DOSE (45)												
Mali	PROMIS CS (46)												
Zimbabwe	SHINE (HIV−) (47)												
Zimbabwe	SHINE (HIV+) (48)												
		**1118**	**656**		**I² = 0.37, Tau² = 0.02**			**791**	**369**		**I² = 0.77, Tau² = 0.21**		
**Fixed**					**0.43 (0.37, 0.51)**						**0.56 (0.46, 0.68)**		
**Random**					**0.42 (0.34, 0.52)**						**0.44 (0.28, 0.68)**		

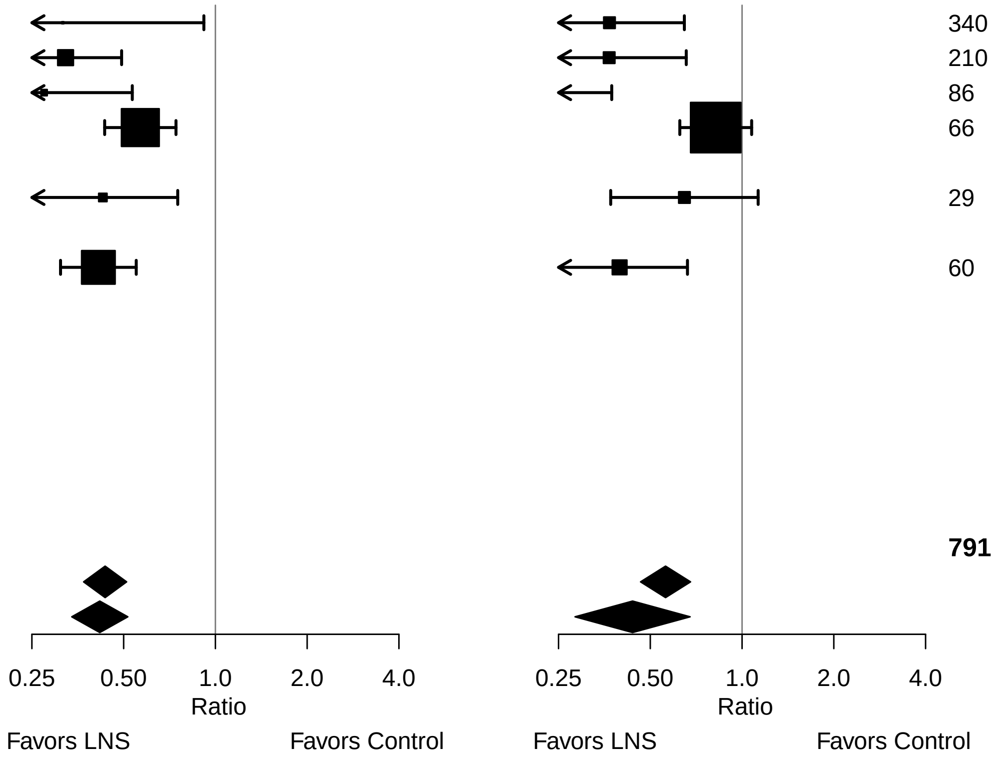

Supplemental figure 8G: Iron deficiency (ferritin < 12 µg/L) prevalence ratio

8G6: Stratified by Child baseline acute malnutrition (insufficient comparisons)

Supplemental figure 8G: Iron deficiency (ferritin < 12 μg/L) prevalence ratio

8G7: Stratified by Child baseline anemia (insufficient comparisons)

Supplemental figure 8G: Iron deficiency (ferritin < 12 µg/L) prevalence ratio

8G8: Stratified by Child high-dose vitamin A supplementation

P−for−interaction = 0.528
Ratio of PRs = 1.16 (0.73, 1.85)

		**Received VitA**						**Not treated**					
		**LNS**	**Control**	**Control**	**PR**	**Fixed**	**Random**	**LNS**	**Control**	**Control**	**PR**	**Fixed**	**Random**
Country	Trial	N	N	Prevalence	(95% CI)	W	W	N	N	Prevalence	(95% CI)	W	W
Bangladesh	JiVitA−4 (35)	140	37	29.7	0.17 (0.06, 0.44)	0.08	0.10	133	41	19.5	0.58 (0.27, 1.25)	0.09	0.16
Bangladesh	RDNS (36)	444	223	20.2	0.37 (0.25, 0.54)	0.56	0.51	105	49	30.6	0.25 (0.11, 0.55)	0.09	0.15
Bangladesh	WASH−B (37)												
Burkina Faso	iLiNS−Zinc (38)												
Burkina Faso	PROMIS CS (39)												
Ghana	GHANA (40)	39	53	60.4	0.47 (0.27, 0.81)	0.26	0.28	44	26	42.3	0.64 (0.33, 1.25)	0.13	0.20
Ghana	iLiNS−DYAD−G (41)												
Kenya	WASH−B (42)	26	31	48.4	0.40 (0.16, 0.99)	0.09	0.11	223	180	62.2	0.40 (0.30, 0.53)	0.69	0.48
Madagascar	MAHAY (43)												
Malawi	iLiNS−DYAD−M (44)												
Malawi	iLiNS−DOSE (45)												
Mali	PROMIS CS (46)												
Zimbabwe	SHINE (HIV−) (47)												
Zimbabwe	SHINE (HIV+) (48)												
		**649**	**344**		**I² = 0.08, Tau² = 0.01**			**505**	**296**		**I² = 0.27, Tau² = 0.05**		
**Fixed**					**0.37 (0.28, 0.49)**						**0.42 (0.33, 0.53)**		
**Random**					**0.37 (0.27, 0.50)**						**0.43 (0.30, 0.61)**		

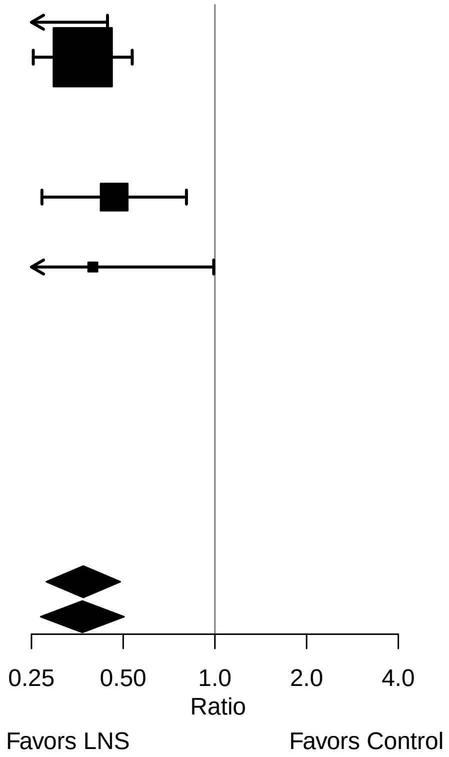

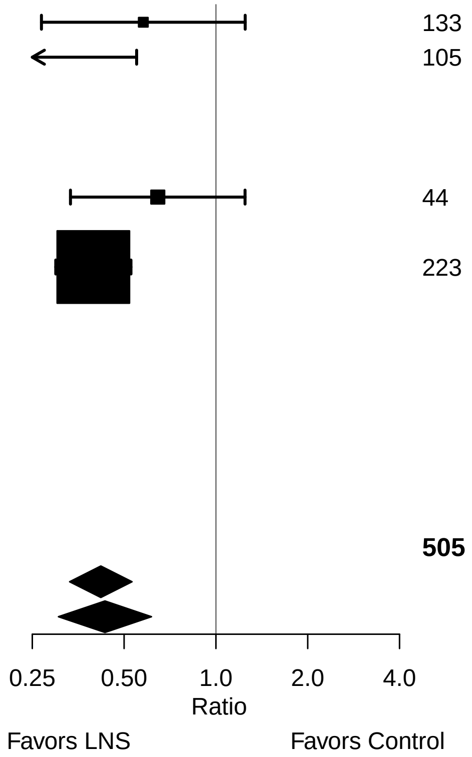

Supplemental figure 8G: Iron deficiency (ferritin < 12 µg/L) prevalence ratio

8G9: Stratified by Child inflammation

P−for−interaction = 0.951
Ratio of PRs = 0.99 (0.74, 1.33)

Country	Trial	Not inflamed: LNS N	Control N	Control Prevalence	PR (95% CI)	Fixed W	Random W	High AGP or CRP: LNS N	Control N	Control Prevalence	PR (95% CI)	Fixed W	Random W
Bangladesh	JiVitA−4 (35)	185	46	26.1	0.27 (0.11, 0.66)	0.05	0.08	270	98	20.4	0.42 (0.23, 0.75)	0.11	0.15
Bangladesh	RDNS (36)	370	168	22.6	0.29 (0.18, 0.46)	0.19	0.22	180	104	21.2	0.45 (0.26, 0.77)	0.12	0.17
Bangladesh	WASH−B (37)												
Burkina Faso	iLiNS−Zinc (38)	124	31	55.9	0.55 (0.35, 0.86)	0.22	0.23	191	65	59.7	0.66 (0.50, 0.87)	0.48	0.35
Burkina Faso	PROMIS CS (39)												
Ghana	GHANA (40)												
Ghana	iLiNS−DYAD−G (41)												
Kenya	WASH−B (42)	157	135	57.8	0.50 (0.37, 0.67)	0.46	0.35	141	124	60.5	0.33 (0.22, 0.49)	0.24	0.26
Madagascar	MAHAY (43)	44	32	53.1	0.39 (0.19, 0.78)	0.09	0.12	39	19	47.4	0.38 (0.15, 0.94)	0.05	0.08
Malawi	iLiNS−DYAD−M (44)												
Malawi	iLiNS−DOSE (45)												
Mali	PROMIS CS (46)												
Zimbabwe	SHINE (HIV−) (47)												
Zimbabwe	SHINE (HIV+) (48)												
		**880**	**412**		**I² = 0.33, Tau² = 0.03**			**821**	**410**		**I² = 0.55, Tau² = 0.04**		
**Fixed**					**0.43 (0.35, 0.53)**						**0.49 (0.40, 0.60)**		
**Random**					**0.42 (0.32, 0.55)**						**0.46 (0.35, 0.60)**		

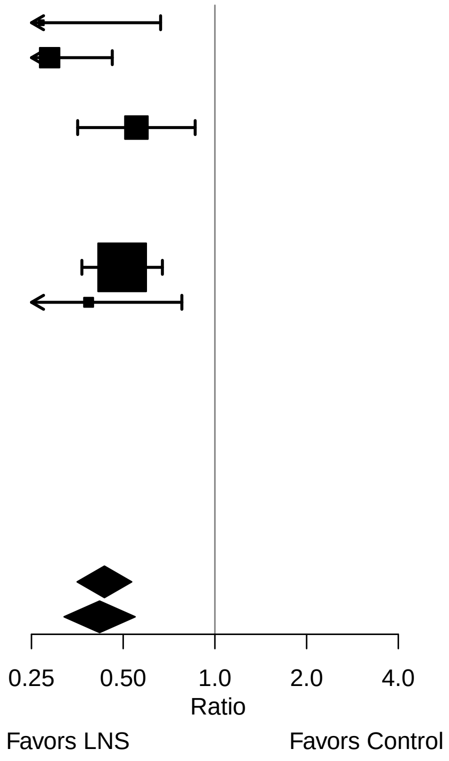

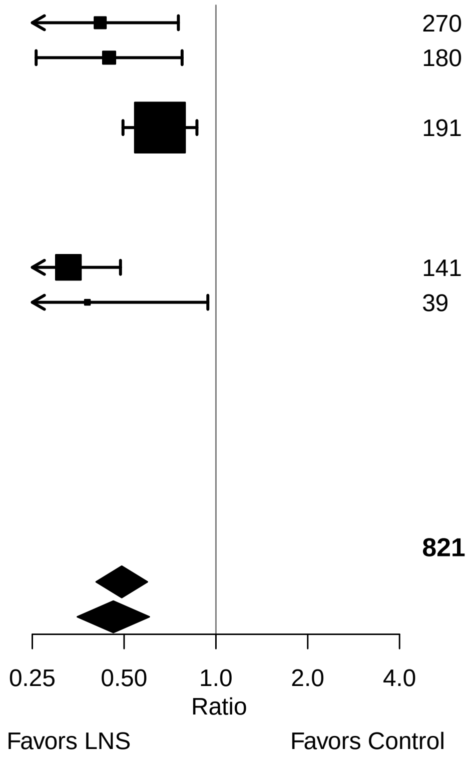

Supplemental figure 8H: Iron deficiency (ferritin < 12 µg/L) prevalence difference

8H1: Stratified by Maternal BMI

P−for−interaction = 0.473
Difference in PDs = 0.03 (−0.05, 0.11)

		At least 20 kg/m²						Less than 20 kg/m²					
Country	Trial	LNS N	Control N	Control Prevalence	PD (95% CI)	Fixed W	Random W	LNS N	Control N	Control Prevalence	PD (95% CI)	Fixed W	Random W
Bangladesh	JiVitA−4 (35)												
Bangladesh	RDNS (36)	228	102	22.5	−0.17 (−0.26, −0.09)	0.41	0.30	300	160	21.2	−0.12 (−0.19, −0.05)	0.51	0.33
Bangladesh	WASH−B (37)	93	97	36.1	−0.25 (−0.39, −0.12)	0.16	0.22	119	81	34.6	−0.28 (−0.38, −0.17)	0.22	0.26
Burkina Faso	iLiNS−Zinc (38)	209	55	54.1	−0.17 (−0.32, −0.03)	0.14	0.20	106	41	64.4	−0.31 (−0.43, −0.19)	0.17	0.23
Burkina Faso	PROMIS CS (39)												
Ghana	GHANA (40)												
Ghana	iLiNS−DYAD−G (41)												
Kenya	WASH−B (42)	223	200	61.5	−0.36 (−0.46, −0.26)	0.29	0.28	64	51	45.1	−0.22 (−0.38, −0.05)	0.09	0.17
Madagascar	MAHAY (43)												
Malawi	iLiNS−DYAD−M (44)												
Malawi	iLiNS−DOSE (45)												
Mali	PROMIS CS (46)												
Zimbabwe	SHINE (HIV−) (47)												
Zimbabwe	SHINE (HIV+) (48)												
		**753**	**454**		**I² = 0.67, Tau² = 0.01**			**589**	**333**		**I² = 0.72, Tau² = 0.01**		
**Fixed**					**−0.24 (−0.30, −0.19)**						**−0.20 (−0.25, −0.15)**		
**Random**					**−0.24 (−0.33, −0.15)**						**−0.22 (−0.31, −0.13)**		

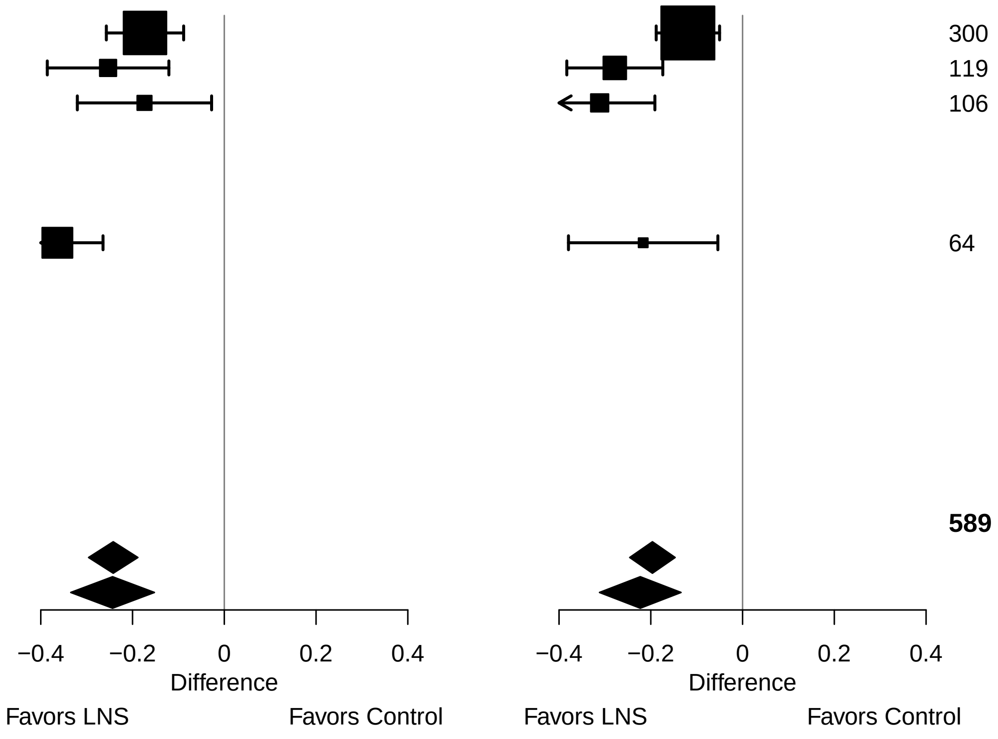

Supplemental figure 8H: Iron deficiency (ferritin < 12 µg/L) prevalence difference

8H2: Stratified by Maternal age

P−for−interaction = 0.779
Difference in PDs = 0.01 (−0.06, 0.07)

		At least 25 y						Less than 25 y					
Country	Trial	LNS N	Control N	Control Prevalence	PD (95% CI)	Fixed W	Random W	LNS N	Control N	Control Prevalence	PD (95% CI)	Fixed W	Random W
Bangladesh	JiVitA−4 (35)	202	70	22.9	−0.17 (−0.29, −0.05)	0.14	0.15	250	73	21.9	−0.12 (−0.24, 0.00)	0.10	0.16
Bangladesh	RDNS (36)	152	81	22.2	−0.16 (−0.26, −0.07)	0.24	0.18	398	191	22.0	−0.14 (−0.19, −0.09)	0.60	0.26
Bangladesh	WASH−B (37)	79	80	27.5	−0.16 (−0.29, −0.03)	0.13	0.14	132	98	41.8	−0.35 (−0.46, −0.24)	0.12	0.18
Burkina Faso	iLiNS−Zinc (38)	178	50	59.3	−0.27 (−0.38, −0.15)	0.16	0.16	137	46	57.7	−0.17 (−0.31, −0.02)	0.07	0.13
Burkina Faso	PROMIS CS (39)												
Ghana	GHANA (40)	61	55	52.7	−0.28 (−0.45, −0.11)	0.07	0.11	22	21	61.9	−0.26 (−0.55, 0.04)	0.02	0.05
Ghana	iLiNS−DYAD−G (41)												
Kenya	WASH−B (42)	178	149	60.4	−0.36 (−0.46, −0.26)	0.21	0.18	116	109	56.9	−0.32 (−0.46, −0.18)	0.07	0.14
Madagascar	MAHAY (43)	47	25	60.0	−0.41 (−0.62, −0.20)	0.05	0.08	36	26	42.3	−0.23 (−0.45, −0.01)	0.03	0.08
Malawi	iLiNS−DYAD−M (44)												
Malawi	iLiNS−DOSE (45)												
Mali	PROMIS CS (46)												
Zimbabwe	SHINE (HIV−) (47)												
Zimbabwe	SHINE (HIV+) (48)												
		**897**	**510**		**I² = 0.57, Tau² = 0.00**			**1091**	**564**		**I² = 0.67, Tau² = 0.00**		
**Fixed**					**−0.24 (−0.29, −0.20)**						**−0.18 (−0.22, −0.15)**		
**Random**					**−0.25 (−0.32, −0.18)**						**−0.22 (−0.29, −0.14)**		

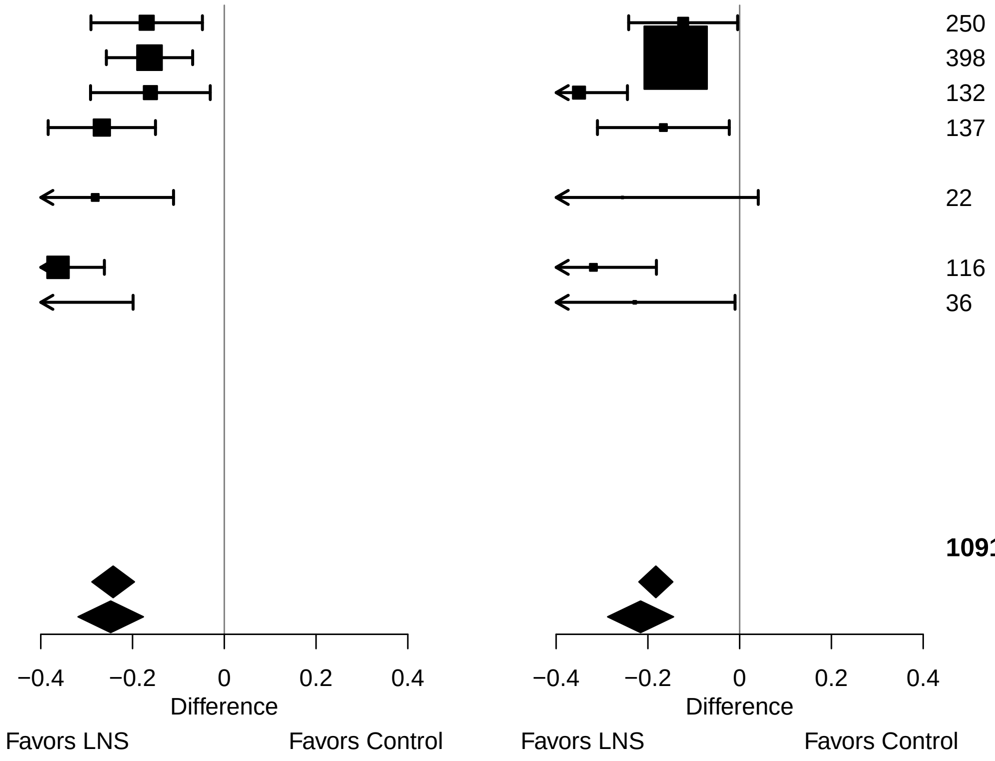

Supplemental figure 8H: Iron deficiency (ferritin < 12 µg/L) prevalence difference

8H3: Stratified by Maternal education

P−for−interaction = 0.963
Difference in PDs = 0.00 (−0.08, 0.07)

		Primary or greater						Incomplete or no formal					
Country	Trial	LNS N	Control N	Control Prevalence	PD (95% CI)	Fixed W	Random W	LNS N	Control N	Control Prevalence	PD (95% CI)	Fixed W	Random W
Bangladesh	JiVitA−4 (35)	298	93	26.9	−0.18 (−0.30, −0.07)	0.12	0.23	156	51	13.7	−0.07 (−0.17, 0.04)	0.29	0.26
Bangladesh	RDNS (36)	406	195	18.5	−0.11 (−0.16, −0.06)	0.63	0.31	144	77	31.2	−0.24 (−0.34, −0.14)	0.32	0.27
Bangladesh	WASH−B (37)	149	139	38.1	−0.29 (−0.40, −0.19)	0.15	0.25	63	39	25.6	−0.18 (−0.32, −0.03)	0.15	0.22
Burkina Faso	iLiNS−Zinc (38)												
Burkina Faso	PROMIS CS (39)												
Ghana	GHANA (40)												
Ghana	iLiNS−DYAD−G (41)												
Kenya	WASH−B (42)	143	117	55.6	−0.32 (−0.45, −0.19)	0.10	0.21	154	142	62.0	−0.37 (−0.49, −0.26)	0.24	0.25
Madagascar	MAHAY (43)												
Malawi	iLiNS−DYAD−M (44)												
Malawi	iLiNS−DOSE (45)												
Mali	PROMIS CS (46)												
Zimbabwe	SHINE (HIV−) (47)												
Zimbabwe	SHINE (HIV+) (48)												
		**996**	**544**		**I² = 0.81, Tau² = 0.01**			**517**	**309**		**I² = 0.80, Tau² = 0.01**		
**Fixed**					**−0.17 (−0.21, −0.13)**						**−0.21 (−0.27, −0.15)**		
**Random**					**−0.22 (−0.31, −0.12)**						**−0.21 (−0.34, −0.09)**		

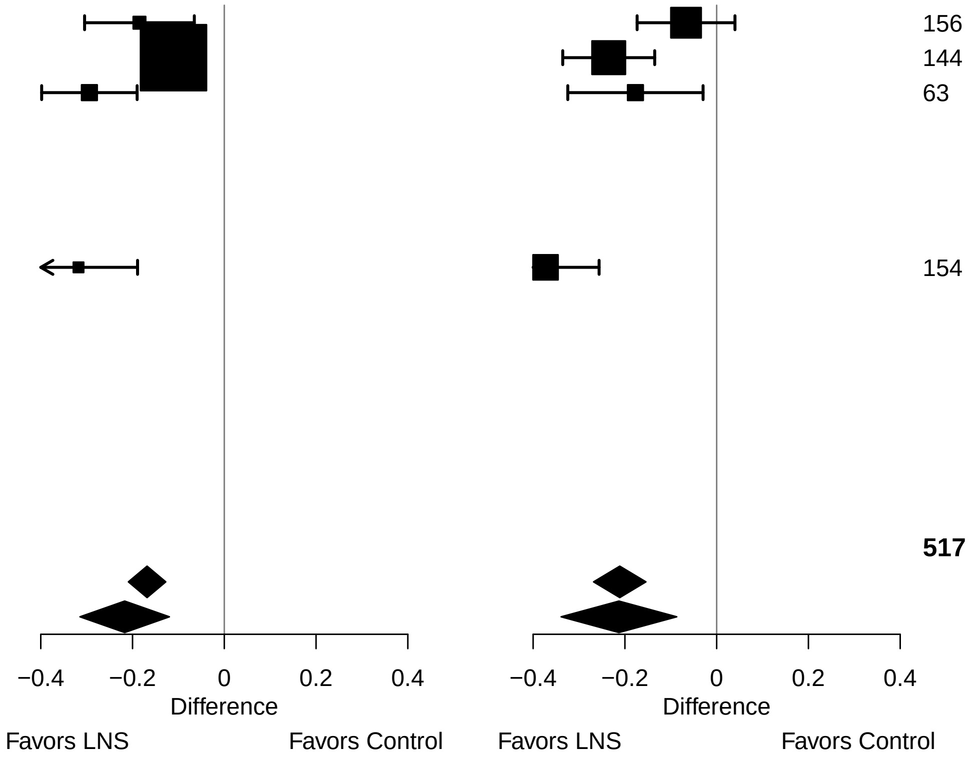

Supplemental figure 8H: Iron deficiency (ferritin < 12 µg/L) prevalence difference

8H4: Stratified by Child sex

P−for−interaction = 0.179
Difference in PDs = 0.04 (−0.02, 0.11)

Country	Trial	Male LNS N	Male Control N	Male Control Prevalence	Male PD (95% CI)	Male Fixed W	Male Random W	Female LNS N	Female Control N	Female Control Prevalence	Female PD (95% CI)	Female Fixed W	Female Random W
Bangladesh	JiVitA−4 (35)	235	70	27.1	−0.19 (−0.31, −0.06)	0.13	0.16	220	74	17.6	−0.10 (−0.21, 0.00)	0.17	0.17
Bangladesh	RDNS (36)	266	131	25.2	−0.16 (−0.23, −0.10)	0.46	0.21	281	141	19.1	−0.13 (−0.21, −0.05)	0.33	0.22
Bangladesh	WASH−B (37)	105	85	37.6	−0.28 (−0.43, −0.14)	0.10	0.14	107	93	33.3	−0.26 (−0.35, −0.17)	0.23	0.19
Burkina Faso	iLiNS−Zinc (38)	150	49	55.6	−0.21 (−0.39, −0.03)	0.06	0.12	165	47	61.5	−0.25 (−0.41, −0.09)	0.08	0.11
Burkina Faso	PROMIS CS (39)												
Ghana	GHANA (40)	51	37	75.7	−0.44 (−0.64, −0.25)	0.05	0.11	32	45	40.0	−0.18 (−0.39, 0.03)	0.04	0.08
Ghana	iLiNS−DYAD−G (41)												
Kenya	WASH−B (42)	146	125	58.4	−0.33 (−0.44, −0.22)	0.15	0.17	152	134	59.7	−0.36 (−0.49, −0.23)	0.11	0.14
Madagascar	MAHAY (43)	34	22	59.1	−0.44 (−0.65, −0.23)	0.04	0.09	49	29	44.8	−0.22 (−0.43, −0.02)	0.05	0.08
Malawi	iLiNS−DYAD−M (44)												
Malawi	iLiNS−DOSE (45)												
Mali	PROMIS CS (46)												
Zimbabwe	SHINE (HIV−) (47)												
Zimbabwe	SHINE (HIV+) (48)												
		**987**	**519**		**I² = 0.64, Tau² = 0.01**			**1006**	**563**		**I² = 0.57, Tau² = 0.00**		
**Fixed**					**−0.23 (−0.28, −0.19)**						**−0.20 (−0.24, −0.15)**		
**Random**					**−0.27 (−0.36, −0.19)**						**−0.21 (−0.28, −0.14)**		

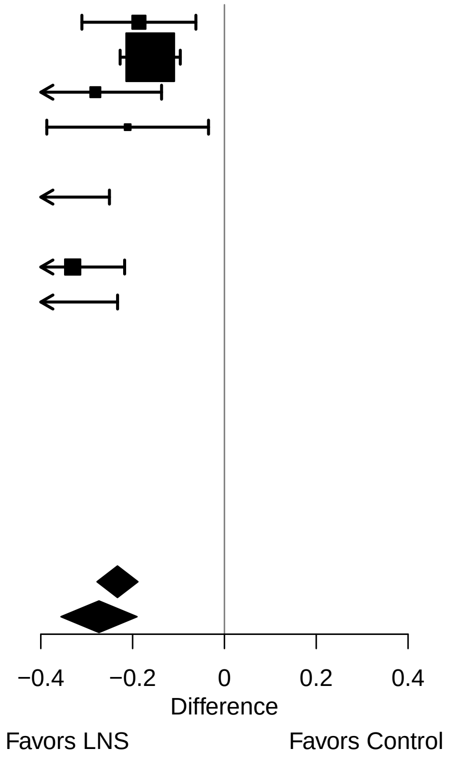

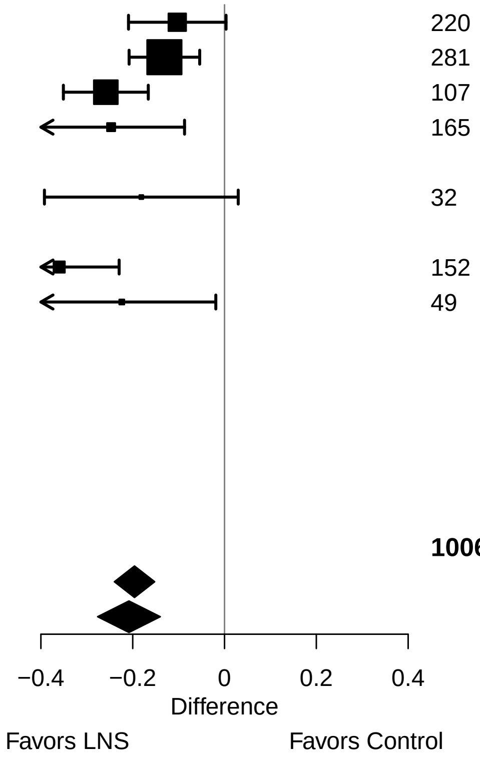

Supplemental figure 8H: Iron deficiency (ferritin < 12 µg/L) prevalence difference

8H5: Stratified by Child birth order

P−for−interaction = 0.638
Difference in PDs = 0.02 (−0.05, 0.09)

Country	Trial	Later born: LNS N	Later born: Control N	Later born: Control Prevalence	Later born: PD (95% CI)	Later born: Fixed W	Later born: Random W	Firstborn: LNS N	Firstborn: Control N	Firstborn: Control Prevalence	Firstborn: PD (95% CI)	Firstborn: Fixed W	Firstborn: Random W
Bangladesh	JiVitA−4 (35)	114	32	25.0	−0.17 (−0.39, 0.05)	0.03	0.07	340	111	21.6	−0.14 (−0.23, −0.04)	0.24	0.21
Bangladesh	RDNS (36)	340	171	22.8	−0.15 (−0.21, −0.10)	0.54	0.31	210	101	20.8	−0.13 (−0.20, −0.06)	0.40	0.24
Bangladesh	WASH−B (37)	124	119	35.3	−0.26 (−0.37, −0.14)	0.11	0.17	86	57	36.8	−0.31 (−0.45, −0.17)	0.11	0.16
Burkina Faso	iLiNS−Zinc (38)	249	71	61.3	−0.27 (−0.40, −0.13)	0.08	0.14	66	25	51.6	−0.10 (−0.21, 0.01)	0.17	0.19
Burkina Faso	PROMIS CS (39)												
Ghana	GHANA (40)	54	50	52.0	−0.30 (−0.48, −0.12)	0.05	0.10	29	29	58.6	−0.21 (−0.46, 0.05)	0.03	0.08
Ghana	iLiNS−DYAD−G (41)												
Kenya	WASH−B (42)	237	213	58.2	−0.34 (−0.43, −0.25)	0.18	0.22	60	46	63.0	−0.38 (−0.58, −0.18)	0.05	0.11
Madagascar	MAHAY (43)												
Malawi	iLiNS−DYAD−M (44)												
Malawi	iLiNS−DOSE (45)												
Mali	PROMIS CS (46)												
Zimbabwe	SHINE (HIV−) (47)												
Zimbabwe	SHINE (HIV+) (48)												
		**1118**	**656**		**I² = 0.66, Tau² = 0.00**			**791**	**369**		**I² = 0.55, Tau² = 0.01**		
**Fixed**					**−0.22 (−0.26, −0.18)**						**−0.16 (−0.21, −0.12)**		
**Random**					**−0.24 (−0.31, −0.18)**						**−0.19 (−0.27, −0.10)**		

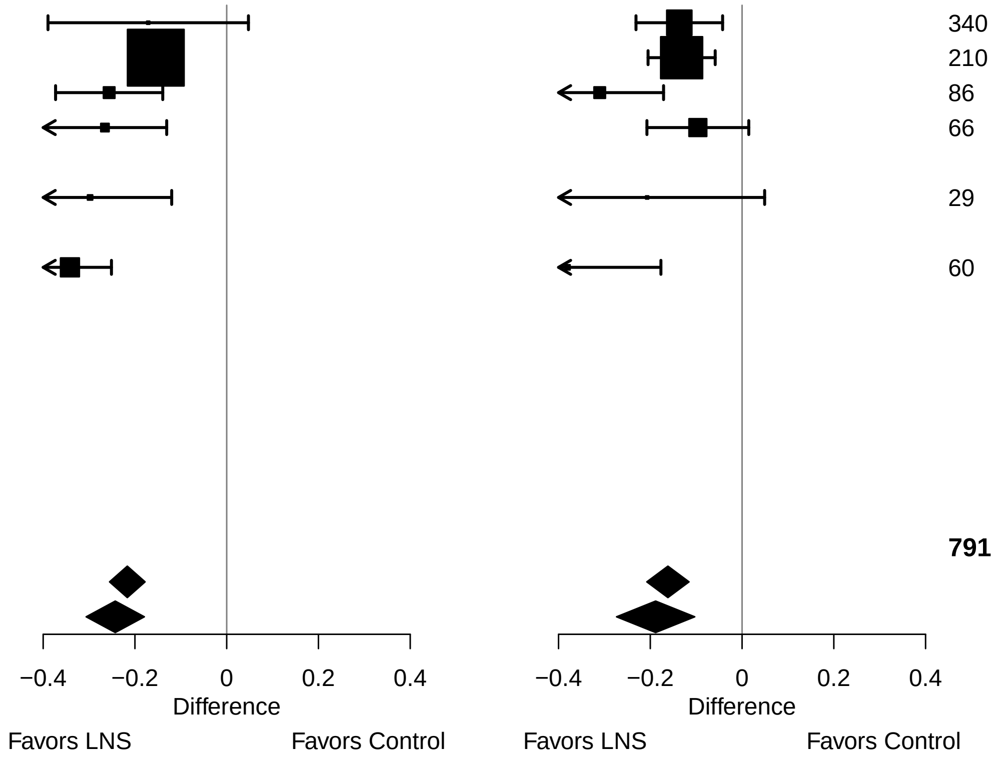

Supplemental figure 8H: Iron deficiency (ferritin < 12 µg/L) prevalence difference

8H6: Stratified by Child baseline acute malnutrition (insufficient comparisons)

Supplemental figure 8H: Iron deficiency (ferritin < 12 µg/L) prevalence difference

8H7: Stratified by Child baseline anemia (insufficient comparisons)

Supplemental figure 8H: Iron deficiency (ferritin < 12 µg/L) prevalence difference

8H8: Stratified by Child high-dose vitamin A supplementation

P−for−interaction = 0.999
Difference in PDs = 0.00 (−0.10, 0.10)

		Received VitA						Not treated					
Country	Trial	LNS N	Control N	Control Prevalence	PD (95% CI)	Fixed W	Random W	LNS N	Control N	Control Prevalence	PD (95% CI)	Fixed W	Random W
Bangladesh	JiVitA−4 (35)	140	37	29.7	−0.25 (−0.42, −0.07)	0.05	0.19	133	41	19.5	−0.08 (−0.22, 0.05)	0.22	0.26
Bangladesh	RDNS (36)	444	223	20.2	−0.13 (−0.17, −0.08)	0.88	0.55	105	49	30.6	−0.23 (−0.35, −0.11)	0.27	0.27
Bangladesh	WASH−B (37)												
Burkina Faso	iLiNS−Zinc (38)												
Burkina Faso	PROMIS CS (39)												
Ghana	GHANA (40)	39	53	60.4	−0.32 (−0.52, −0.12)	0.04	0.16	44	26	42.3	−0.15 (−0.38, 0.08)	0.07	0.17
Ghana	iLiNS−DYAD−G (41)												
Kenya	WASH−B (42)	26	31	48.4	−0.29 (−0.55, −0.03)	0.02	0.10	223	180	62.2	−0.38 (−0.47, −0.28)	0.44	0.30
Madagascar	MAHAY (43)												
Malawi	iLiNS−DYAD−M (44)												
Malawi	iLiNS−DOSE (45)												
Mali	PROMIS CS (46)												
Zimbabwe	SHINE (HIV−) (47)												
Zimbabwe	SHINE (HIV+) (48)												
		**649**	**344**		**I^2 = 0.52, Tau^2 = 0.00**			**505**	**296**		**I^2 = 0.78, Tau^2 = 0.01**		
**Fixed**					**−0.15 (−0.19, −0.11)**						**−0.26 (−0.32, −0.19)**		
**Random**					**−0.20 (−0.29, −0.11)**						**−0.22 (−0.35, −0.09)**		

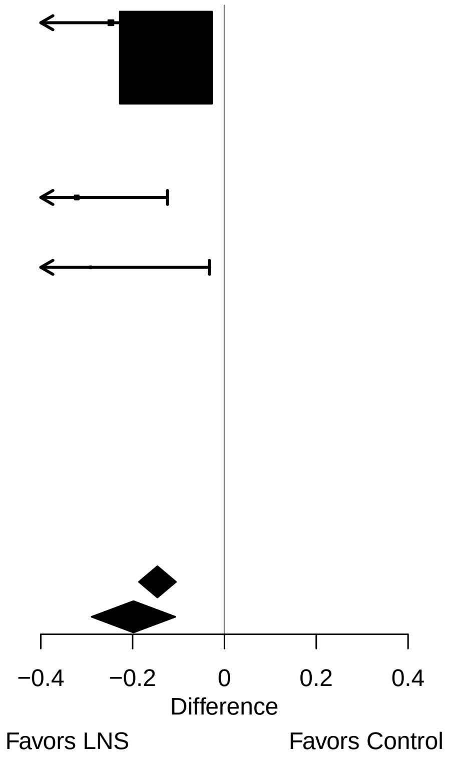

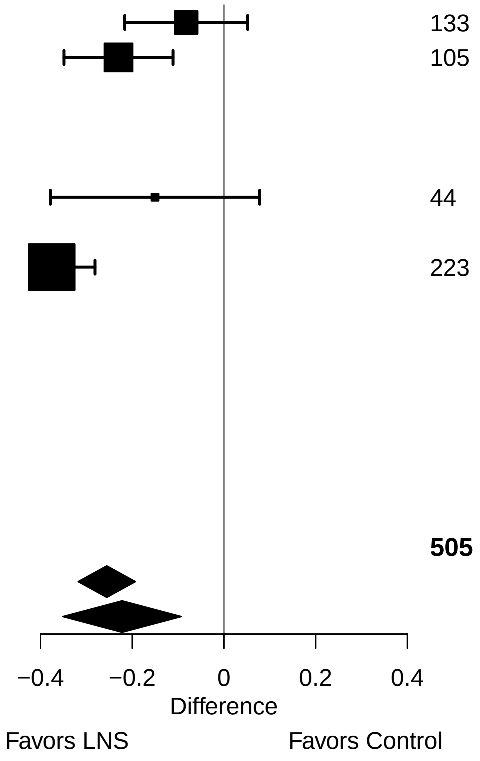

Supplemental figure 8H: Iron deficiency (ferritin < 12 µg/L) prevalence difference

8H9: Stratified by Child inflammation

P−for−interaction = 0.698
Difference in PDs = 0.01 (−0.06, 0.09)

					Not inflamed						High AGP or CRP		
		**LNS**	**Control**	**Control**	**PD**	**Fixed**	**Random**	**LNS**	**Control**	**Control**	**PD**	**Fixed**	**Random**
Country	Trial	N	N	Prevalence	(95% CI)	W	W	N	N	Prevalence	(95% CI)	W	W
Bangladesh	JiVitA−4 (35)	185	46	26.1	−0.19 (−0.38, 0.00)	0.05	0.09	270	98	20.4	−0.12 (−0.22, −0.02)	0.24	0.22
Bangladesh	RDNS (36)	370	168	22.6	−0.16 (−0.21, −0.11)	0.72	0.51	180	104	21.2	−0.12 (−0.20, −0.04)	0.38	0.24
Bangladesh	WASH−B (37)												
Burkina Faso	iLiNS−Zinc (38)	124	31	55.9	−0.25 (−0.43, −0.08)	0.06	0.10	191	65	59.7	−0.21 (−0.35, −0.06)	0.12	0.19
Burkina Faso	PROMIS CS (39)												
Ghana	GHANA (40)												
Ghana	iLiNS−DYAD−G (41)												
Kenya	WASH−B (42)	157	135	57.8	−0.29 (−0.41, −0.18)	0.13	0.21	141	124	60.5	−0.41 (−0.51, −0.30)	0.22	0.22
Madagascar	MAHAY (43)	44	32	53.1	−0.33 (−0.53, −0.13)	0.04	0.09	39	19	47.4	−0.29 (−0.53, −0.05)	0.04	0.12
Malawi	iLiNS−DYAD−M (44)												
Malawi	iLiNS−DOSE (45)												
Mali	PROMIS CS (46)												
Zimbabwe	SHINE (HIV−) (47)												
Zimbabwe	SHINE (HIV+) (48)												
		**880**	**412**		**I² = 0.39, Tau² = 0.00**			**821**	**410**		**I² = 0.82, Tau² = 0.01**		
**Fixed**					**−0.19 (−0.23, −0.15)**						**−0.20 (−0.25, −0.15)**		
**Random**					**−0.21 (−0.28, −0.15)**						**−0.22 (−0.33, −0.11)**		

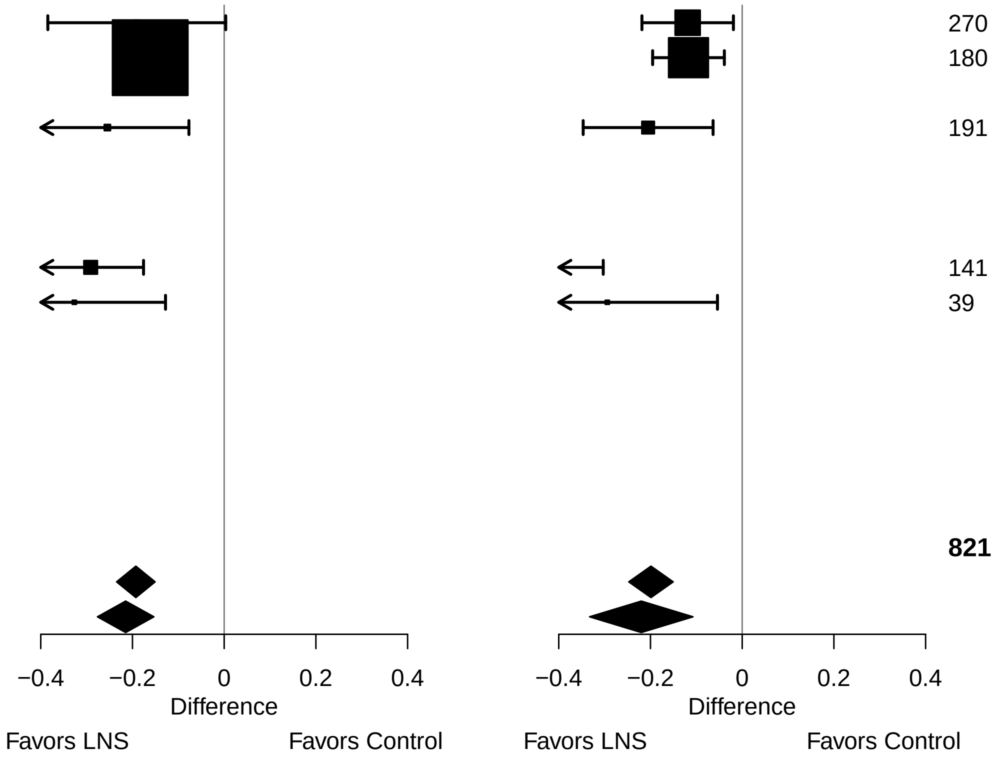

Supplemental figure 8I: Iron deficiency anemia prevalence ratio

8I1: Stratified by Maternal BMI

P−for−interaction = 0.521
Ratio of PRs = 1.16 (0.74, 1.81)

		At least 20 kg/m²						Less than 20 kg/m²					
Country	Trial	LNS N	Control N	Control Prevalence	PR (95% CI)	Fixed W	Random W	LNS N	Control N	Control Prevalence	PR (95% CI)	Fixed W	Random W
Bangladesh	JiVitA−4 (35)												
Bangladesh	RDNS (36)	227	102	16.7	0.19 (0.08, 0.45)	0.11	0.24	300	160	13.1	0.33 (0.16, 0.66)	0.15	0.15
Bangladesh	WASH−B (37)												
Burkina Faso	iLiNS−Zinc (38)	215	59	52.5	0.53 (0.36, 0.78)	0.60	0.41	112	42	57.1	0.47 (0.35, 0.63)	0.78	0.78
Burkina Faso	PROMIS CS (39)												
Ghana	GHANA (40)												
Ghana	iLiNS−DYAD−G (41)												
Kenya	WASH−B (42)	223	199	36.7	0.26 (0.15, 0.45)	0.29	0.35	64	51	13.7	0.68 (0.25, 1.89)	0.07	0.07
Madagascar	MAHAY (43)												
Malawi	iLiNS−DYAD−M (44)												
Malawi	iLiNS−DOSE (45)												
Mali	PROMIS CS (46)												
Zimbabwe	SHINE (HIV−) (47)												
Zimbabwe	SHINE (HIV+) (48)												
		**665**	**360**		**I² = 0.72, Tau² = 0.20**			**476**	**253**		**I² = 0.00, Tau² = 0.00**		
**Fixed**					**0.38 (0.28, 0.52)**						**0.46 (0.35, 0.60)**		
**Random**					**0.32 (0.17, 0.59)**						**0.46 (0.35, 0.60)**		

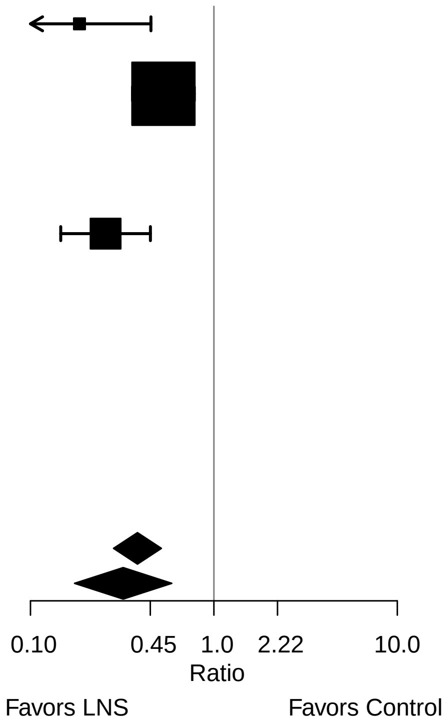

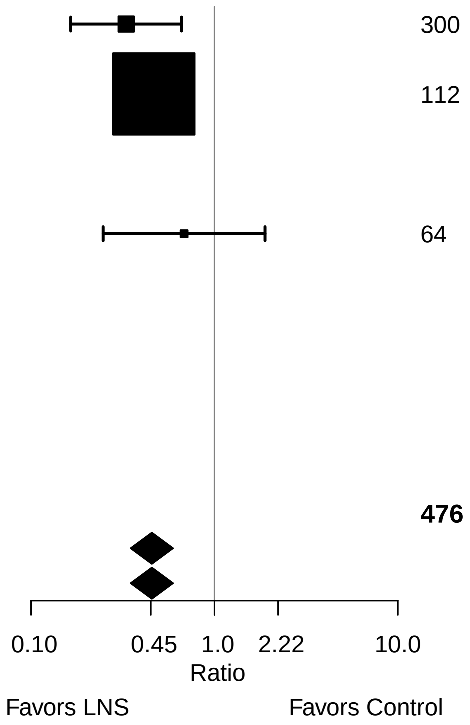

Supplemental figure 8I: Iron deficiency anemia prevalence ratio

8I2: Stratified by Maternal age (insufficient comparisons)

Supplemental figure 8I: Iron deficiency anemia prevalence ratio

8I3: Stratified by Maternal education (insufficient comparisons)

Supplemental figure 8I: Iron deficiency anemia prevalence ratio

8I4: Stratified by Child sex

P−for−interaction = 0.208
Ratio of PRs = 0.78 (0.53, 1.15)

Country	Trial	Male LNS N	Male Control N	Male Control Prevalence	Male PR (95% CI)	Male Fixed W	Male Random W	Female LNS N	Female Control N	Female Control Prevalence	Female PR (95% CI)	Female Fixed W	Female Random W
Bangladesh	JiVitA−4 (35)												
Bangladesh	RDNS (36)	266	131	16.0	0.26 (0.12, 0.57)	0.13	0.23	281	141	13.5	0.24 (0.12, 0.49)	0.15	0.24
Bangladesh	WASH−B (37)												
Burkina Faso	iLiNS−Zinc (38)	157	53	50.9	0.57 (0.40, 0.82)	0.65	0.47	170	48	58.3	0.46 (0.32, 0.66)	0.59	0.44
Burkina Faso	PROMIS CS (39)												
Ghana	GHANA (40)												
Ghana	iLiNS−DYAD−G (41)												
Kenya	WASH−B (42)	146	125	30.4	0.34 (0.18, 0.63)	0.21	0.30	152	133	36.1	0.24 (0.14, 0.41)	0.26	0.32
Madagascar	MAHAY (43)												
Malawi	iLiNS−DYAD−M (44)												
Malawi	iLiNS−DOSE (45)												
Mali	PROMIS CS (46)												
Zimbabwe	SHINE (HIV−) (47)												
Zimbabwe	SHINE (HIV+) (48)												
		**569**	**309**		**I² = 0.55, Tau² = 0.09**			**603**	**322**		**I² = 0.61, Tau² = 0.09**		
**Fixed**					**0.46 (0.34, 0.62)**						**0.35 (0.27, 0.46)**		
**Random**					**0.41 (0.26, 0.65)**						**0.32 (0.20, 0.50)**		

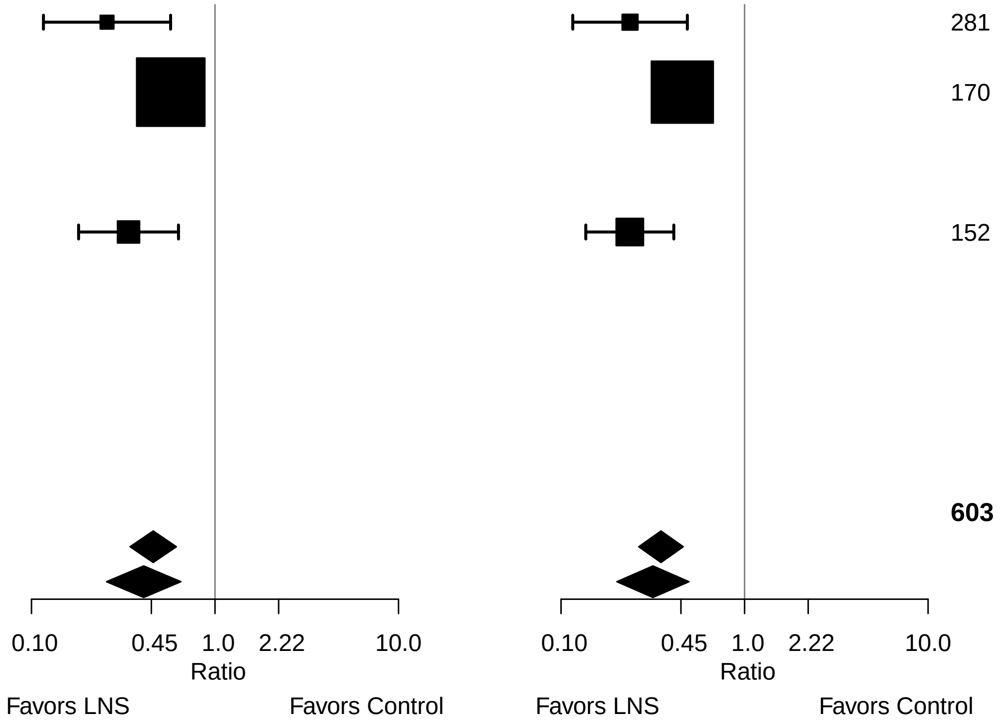

Supplemental figure 8I: Iron deficiency anemia prevalence ratio

8I5: Stratified by Child birth order

P−for−interaction = 0.904
Ratio of PRs = 0.97 (0.62, 1.53)

Country	Trial	Later born LNS N	Later born Control N	Later born Control Prevalence	Later born PR (95% CI)	Later born Fixed W	Later born Random W	Firstborn LNS N	Firstborn Control N	Firstborn Control Prevalence	Firstborn PR (95% CI)	Firstborn Fixed W	Firstborn Random W
Bangladesh	JiVitA−4 (35)												
Bangladesh	RDNS (36)	339	171	14.6	0.26 (0.13, 0.51)	0.11	0.22	210	101	14.9	0.22 (0.10, 0.49)	0.26	0.32
Bangladesh	WASH−B (37)												
Burkina Faso	iLiNS−Zinc (38)	257	71	56.3	0.50 (0.38, 0.65)	0.69	0.48	70	30	50.0	0.55 (0.32, 0.92)	0.57	0.43
Burkina Faso	PROMIS CS (39)												
Ghana	GHANA (40)												
Ghana	iLiNS−DYAD−G (41)												
Kenya	WASH−B (42)	237	213	32.4	0.30 (0.18, 0.50)	0.20	0.30	60	45	37.8	0.22 (0.08, 0.58)	0.17	0.25
Madagascar	MAHAY (43)												
Malawi	iLiNS−DYAD−M (44)												
Malawi	iLiNS−DOSE (45)												
Mali	PROMIS CS (46)												
Zimbabwe	SHINE (HIV−) (47)												
Zimbabwe	SHINE (HIV+) (48)												
		**833**	**455**		**I² = 0.60, Tau² = 0.07**			**340**	**176**		**I² = 0.58, Tau² = 0.16**		
**Fixed**					**0.42 (0.33, 0.53)**						**0.37 (0.25, 0.55)**		
**Random**					**0.37 (0.25, 0.55)**						**0.33 (0.18, 0.61)**		

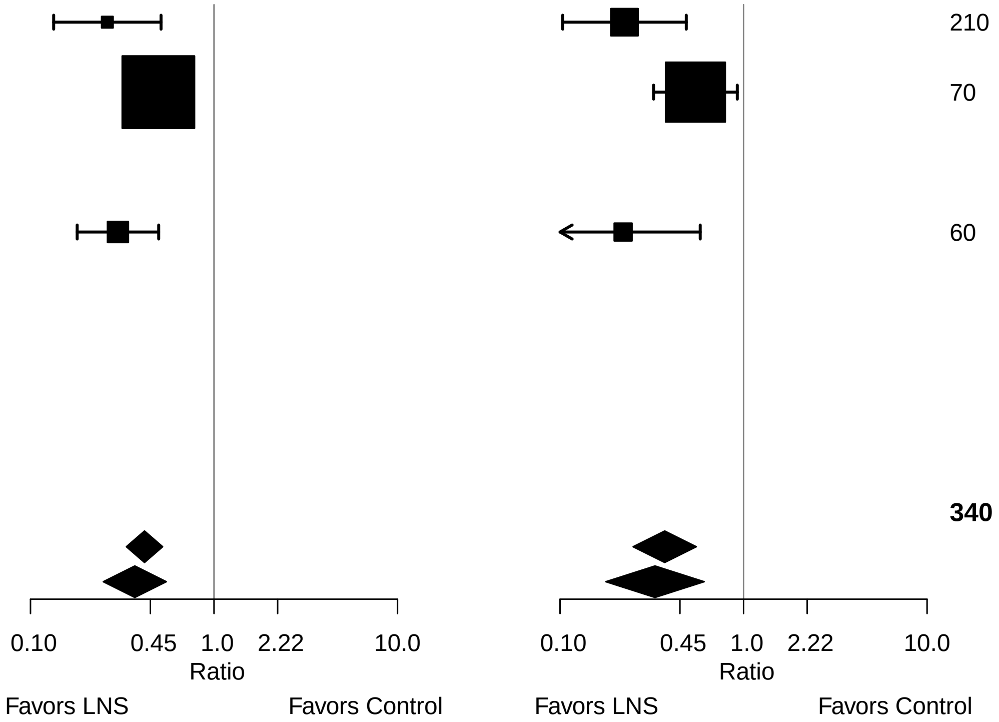

Supplemental figure 8I: Iron deficiency anemia prevalence ratio

8I6: Stratified by Child baseline acute malnutrition (insufficient comparisons)

Supplemental figure 8I: Iron deficiency anemia prevalence ratio

8I7: Stratified by Child baseline anemia (insufficient comparisons)

Supplemental figure 8I: Iron deficiency anemia prevalence ratio

8I8: Stratified by Child high-dose vitamin A supplementation (insufficient comparisons)

Supplemental figure 8I: Iron deficiency anemia prevalence ratio

8I9: Stratified by Child inflammation

P−for−interaction = 0.388
Ratio of PRs = 1.21 (0.79, 1.86)

Country	Trial	LNS N	Control N	Control Prevalence	Not inflamed PR (95% CI)	Fixed W	Random W	LNS N	Control N	Control Prevalence	High AGP or CRP PR (95% CI)	Fixed W	Random W
Bangladesh	JiVitA−4 (35)												
Bangladesh	RDNS (36)	369	168	15.5	0.19 (0.10, 0.37)	0.24	0.28	180	104	13.5	0.37 (0.18, 0.78)	0.12	0.27
Bangladesh	WASH−B (37)												
Burkina Faso	iLiNS−Zinc (38)	130	32	53.1	0.40 (0.24, 0.66)	0.42	0.38	197	69	55.1	0.58 (0.43, 0.79)	0.73	0.43
Burkina Faso	PROMIS CS (39)												
Ghana	GHANA (40)												
Ghana	iLiNS−DYAD−G (41)												
Kenya	WASH−B (42)	157	135	33.3	0.34 (0.20, 0.60)	0.34	0.34	141	123	33.3	0.21 (0.11, 0.42)	0.15	0.29
Madagascar	MAHAY (43)												
Malawi	iLiNS−DYAD−M (44)												
Malawi	iLiNS−DOSE (45)												
Mali	PROMIS CS (46)												
Zimbabwe	SHINE (HIV−) (47)												
Zimbabwe	SHINE (HIV+) (48)												
		**656**	**335**		**I² = 0.36, Tau² = 0.05**			**518**	**296**		**I² = 0.73, Tau² = 0.18**		
**Fixed**					**0.32 (0.23, 0.44)**						**0.47 (0.37, 0.61)**		
**Random**					**0.31 (0.20, 0.47)**						**0.38 (0.21, 0.68)**		

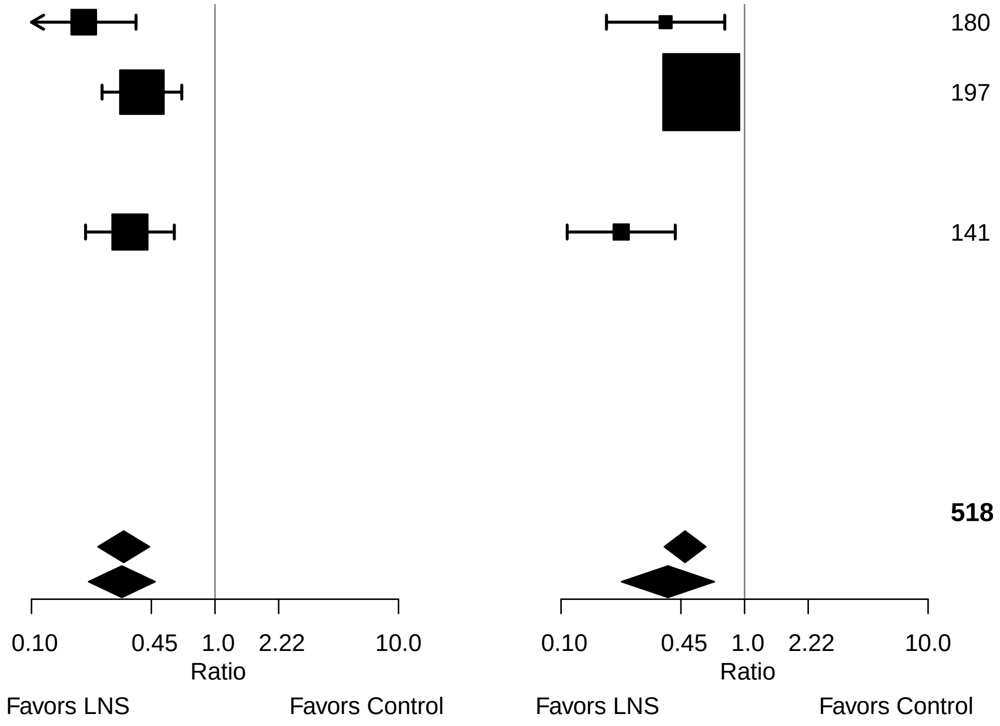

Supplemental figure 8J: Iron deficiency anemia prevalence difference

8J1: Stratified by Maternal BMI

P−for−interaction = 0.023
Difference in PDs = 0.09 (0.01, 0.16)

		At least 20 kg/m²						Less than 20 kg/m²					
Country	Trial	LNS N	Control N	Control Prevalence	PD (95% CI)	Fixed W	Random W	LNS N	Control N	Control Prevalence	PD (95% CI)	Fixed W	Random W
Bangladesh	JiVitA−4 (35)												
Bangladesh	RDNS (36)	227	102	16.7	−0.14 (−0.21, −0.06)	0.44	0.40	300	160	13.1	−0.09 (−0.16, −0.02)	0.57	0.36
Bangladesh	WASH−B (37)												
Burkina Faso	iLiNS−Zinc (38)	215	59	52.5	−0.25 (−0.41, −0.09)	0.11	0.20	112	42	57.1	−0.30 (−0.41, −0.19)	0.23	0.32
Burkina Faso	PROMIS CS (39)												
Ghana	GHANA (40)												
Ghana	iLiNS−DYAD−G (41)												
Kenya	WASH−B (42)	223	199	36.7	−0.27 (−0.35, −0.19)	0.45	0.40	64	51	13.7	−0.04 (−0.16, 0.08)	0.20	0.32
Madagascar	MAHAY (43)												
Malawi	iLiNS−DYAD−M (44)												
Malawi	iLiNS−DOSE (45)												
Mali	PROMIS CS (46)												
Zimbabwe	SHINE (HIV−) (47)												
Zimbabwe	SHINE (HIV+) (48)												
		**665**	**360**		**I² = 0.67, Tau² = 0.00**			**476**	**253**		**I² = 0.84, Tau² = 0.02**		
**Fixed**					**−0.21 (−0.26, −0.16)**						**−0.13 (−0.18, −0.08)**		
**Random**					**−0.21 (−0.30, −0.13)**						**−0.14 (−0.30, 0.01)**		

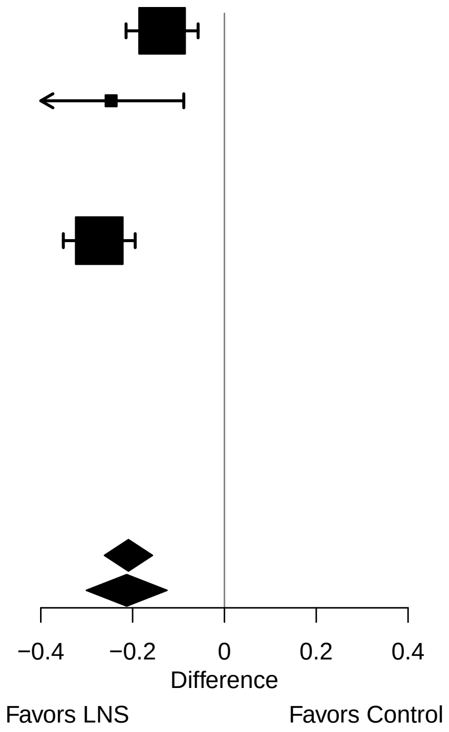

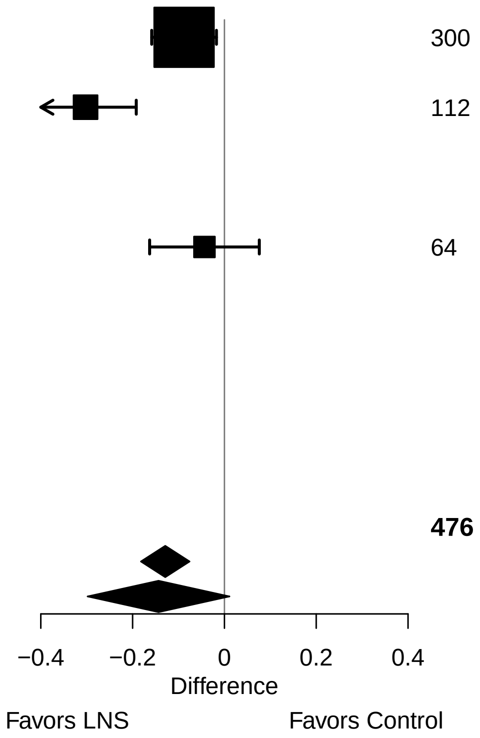

Supplemental figure 8J: Iron deficiency anemia prevalence difference

8J2: Stratified by Maternal age (insufficient comparisons)

Supplemental figure 8J: Iron deficiency anemia prevalence difference

8J3: Stratified by Maternal education (insufficient comparisons)

Supplemental figure 8J: Iron deficiency anemia prevalence difference

8J4: Stratified by Child sex

P−for−interaction = 0.555
Difference in PDs = −0.02 (−0.09, 0.05)

Country	Trial	Male LNS N	Male Control N	Male Control Prevalence	Male PD (95% CI)	Male Fixed W	Male Random W	Female LNS N	Female Control N	Female Control Prevalence	Female PD (95% CI)	Female Fixed W	Female Random W
Bangladesh	JiVitA−4 (35)												
Bangladesh	RDNS (36)	266	131	16.0	−0.12 (−0.19, −0.04)	0.56	0.52	281	141	13.5	−0.10 (−0.16, −0.04)	0.62	0.39
Bangladesh	WASH−B (37)												
Burkina Faso	iLiNS−Zinc (38)	157	53	50.9	−0.22 (−0.36, −0.08)	0.15	0.17	170	48	58.3	−0.32 (−0.47, −0.16)	0.08	0.26
Burkina Faso	PROMIS CS (39)												
Ghana	GHANA (40)												
Ghana	iLiNS−DYAD−G (41)												
Kenya	WASH−B (42)	146	125	30.4	−0.20 (−0.31, −0.10)	0.29	0.30	152	133	36.1	−0.28 (−0.36, −0.19)	0.30	0.36
Madagascar	MAHAY (43)												
Malawi	iLiNS−DYAD−M (44)												
Malawi	iLiNS−DOSE (45)												
Mali	PROMIS CS (46)												
Zimbabwe	SHINE (HIV−) (47)												
Zimbabwe	SHINE (HIV+) (48)												
		**569**	**309**		**I^2 = 0.18, Tau^2 = 0.00**			**603**	**322**		**I^2 = 0.86, Tau^2 = 0.01**		
**Fixed**					**−0.16 (−0.21, −0.10)**						**−0.17 (−0.22, −0.13)**		
**Random**					**−0.16 (−0.22, −0.10)**						**−0.22 (−0.35, −0.09)**		

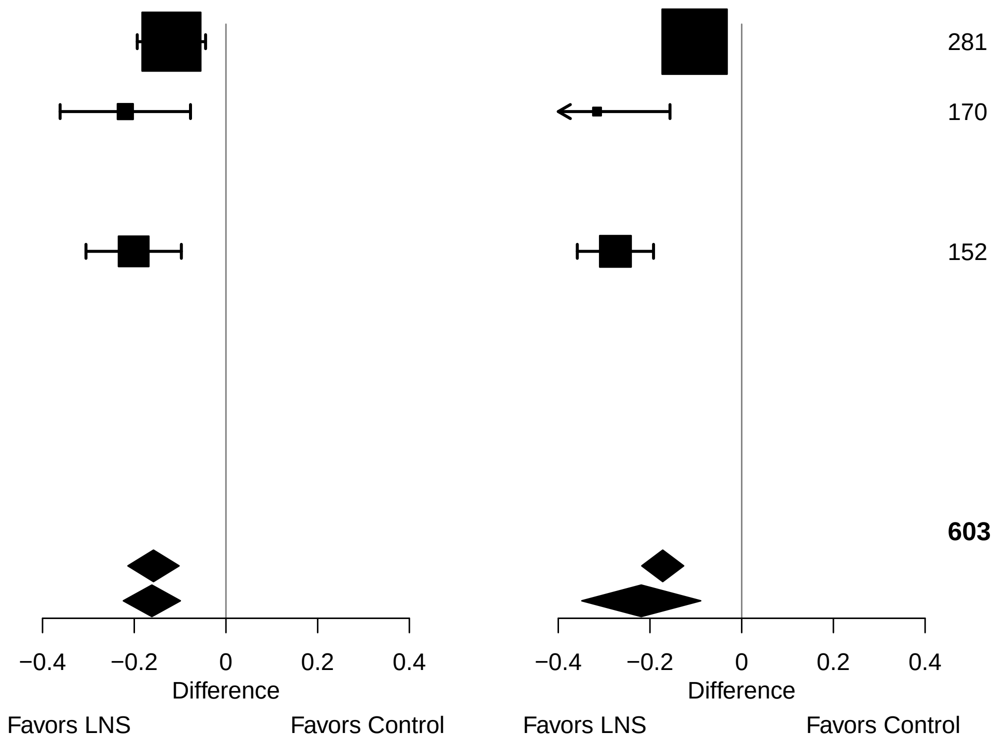

Supplemental figure 8J: Iron deficiency anemia prevalence difference

8J5: Stratified by Child birth order

P−for−interaction = 0.745
Difference in PDs = −0.01 (−0.09, 0.06)

Country	Trial	LNS N	Control N	Control Prevalence	Later born PD (95% CI)	Fixed W	Random W	LNS N	Control N	Control Prevalence	Firstborn PD (95% CI)	Fixed W	Random W
Bangladesh	JiVitA−4 (35)												
Bangladesh	RDNS (36)	339	171	14.6	−0.11 (−0.16, −0.05)	0.54	0.37	210	101	14.9	−0.12 (−0.18, −0.05)	0.82	0.60
Bangladesh	WASH−B (37)												
Burkina Faso	iLiNS−Zinc (38)	257	71	56.3	−0.28 (−0.38, −0.18)	0.18	0.30	70	30	50.0	−0.23 (−0.45, −0.01)	0.08	0.18
Burkina Faso	PROMIS CS (39)												
Ghana	GHANA (40)												
Ghana	iLiNS−DYAD−G (41)												
Kenya	WASH−B (42)	237	213	32.4	−0.23 (−0.31, −0.15)	0.28	0.33	60	45	37.8	−0.29 (−0.49, −0.10)	0.10	0.21
Madagascar	MAHAY (43)												
Malawi	iLiNS−DYAD−M (44)												
Malawi	iLiNS−DOSE (45)												
Mali	PROMIS CS (46)												
Zimbabwe	SHINE (HIV−) (47)												
Zimbabwe	SHINE (HIV+) (48)												
		**833**	**455**		**I^2 = 0.83, Tau^2 = 0.01**			**340**	**176**		**I^2 = 0.44, Tau^2 = 0.00**		
**Fixed**					**−0.17 (−0.21, −0.13)**						**−0.14 (−0.20, −0.08)**		
**Random**					**−0.20 (−0.30, −0.10)**						**−0.17 (−0.28, −0.07)**		

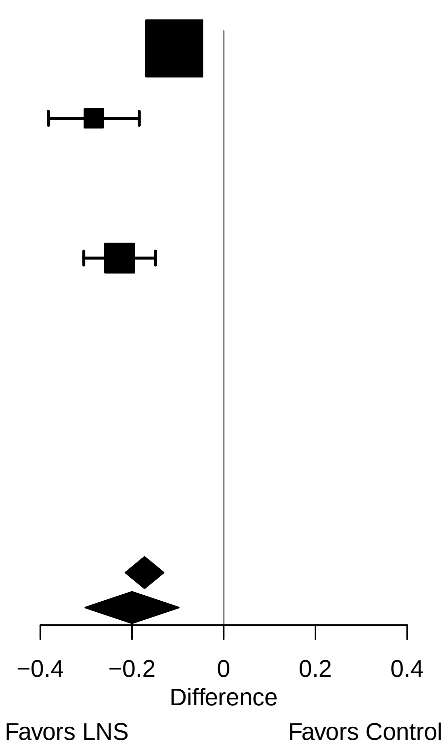

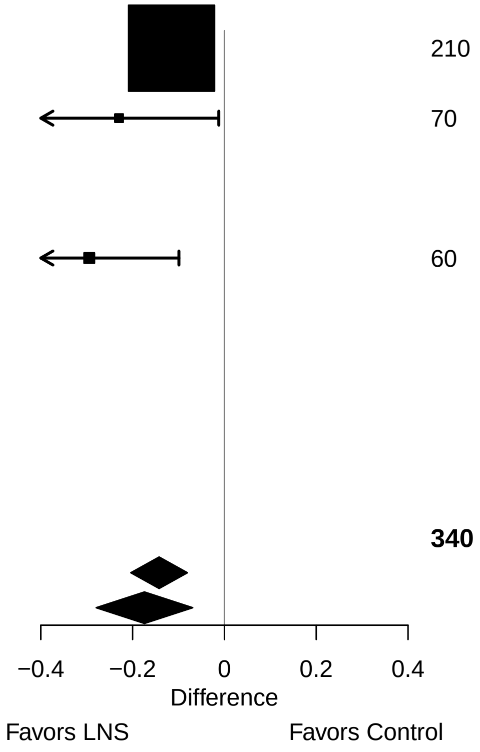

Supplemental figure 8J: Iron deficiency anemia prevalence difference

8J6: Stratified by Child baseline acute malnutrition (insufficient comparisons)

Supplemental figure 8J: Iron deficiency anemia prevalence difference

8J7: Stratified by Child baseline anemia (insufficient comparisons)

Supplemental figure 8J: Iron deficiency anemia prevalence difference

8J8: Stratified by Child high-dose vitamin A supplementation (insufficient comparisons)

Supplemental figure 8J: Iron deficiency anemia prevalence difference

8J9: Stratified by Child inflammation

P−for−interaction = 0.590
Difference in PDs = 0.02 (−0.05, 0.09)

Country	Trial	LNS N	Control N	Control Prevalence	Not inflamed PD (95% CI)	Fixed W	Random W	LNS N	Control N	Control Prevalence	High AGP or CRP PD (95% CI)	Fixed W	Random W
Bangladesh	JiVitA−4 (35)												
Bangladesh	RDNS (36)	369	168	15.5	−0.12 (−0.17, −0.08)	0.76	0.48	180	104	13.5	−0.08 (−0.15, −0.01)	0.53	0.38
Bangladesh	WASH−B (37)												
Burkina Faso	iLiNS−Zinc (38)	130	32	53.1	−0.32 (−0.51, −0.12)	0.05	0.18	197	69	55.1	−0.23 (−0.36, −0.10)	0.15	0.27
Burkina Faso	PROMIS CS (39)												
Ghana	GHANA (40)												
Ghana	iLiNS−DYAD−G (41)												
Kenya	WASH−B (42)	157	135	33.3	−0.22 (−0.32, −0.12)	0.18	0.35	141	123	33.3	−0.26 (−0.35, −0.17)	0.33	0.35
Madagascar	MAHAY (43)												
Malawi	iLiNS−DYAD−M (44)												
Malawi	iLiNS−DOSE (45)												
Mali	PROMIS CS (46)												
Zimbabwe	SHINE (HIV−) (47)												
Zimbabwe	SHINE (HIV+) (48)												
		**656**	**335**		**I² = 0.64, Tau² = 0.00**			**518**	**296**		**I² = 0.81, Tau² = 0.01**		
**Fixed**					**−0.15 (−0.20, −0.11)**						**−0.16 (−0.22, −0.11)**		
**Random**					**−0.19 (−0.29, −0.09)**						**−0.19 (−0.30, −0.07)**		

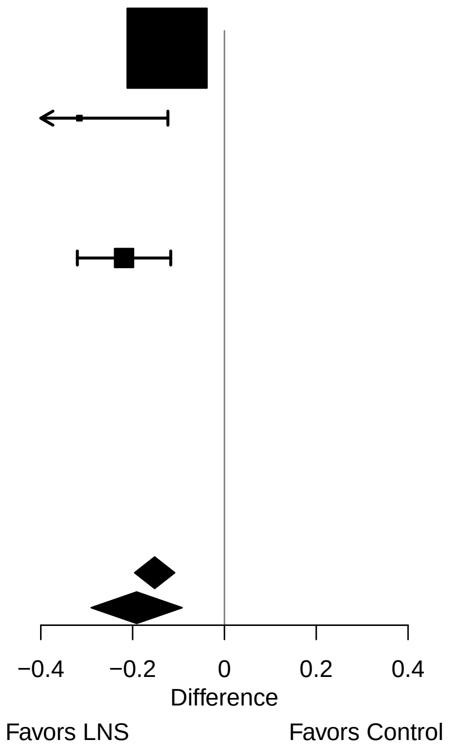

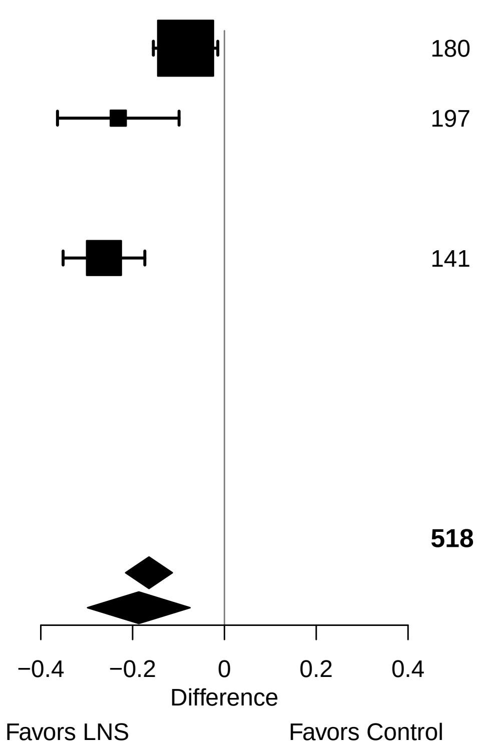

Supplemental figure 8K: Geometric mean ratio of soluble transferrin receptor concentration

8K1: Stratified by Maternal BMI

P−for−interaction = 0.955
Ratio of GMRs = 1.00 (0.94, 1.06)

		At least 20 kg/m²						Less than 20 kg/m²					
Country	Trial	LNS N	Control N	Control Median	GMR (95% CI)	Fixed W	Random W	LNS N	Control N	Control Median	GMR (95% CI)	Fixed W	Random W
Bangladesh	JiVitA−4 (35)												
Bangladesh	RDNS (36)	228	102	8.1	0.88 (0.82, 0.94)	0.37	0.29	300	160	8.4	0.87 (0.81, 0.93)	0.45	0.44
Bangladesh	WASH−B (37)	93	97	7.6	0.81 (0.75, 0.88)	0.28	0.27	119	81	7.5	0.81 (0.74, 0.89)	0.28	0.28
Burkina Faso	iLiNS−Zinc (38)	209	55	10.0	0.74 (0.66, 0.82)	0.15	0.21	106	41	9.9	0.78 (0.65, 0.93)	0.07	0.07
Burkina Faso	PROMIS CS (39)												
Ghana	GHANA (40)												
Ghana	iLiNS−DYAD−G (41)												
Kenya	WASH−B (42)	223	200	11.3	0.80 (0.72, 0.88)	0.19	0.23	64	51	9.9	0.79 (0.71, 0.88)	0.20	0.21
Madagascar	MAHAY (43)												
Malawi	iLiNS−DYAD−M (44)												
Malawi	iLiNS−DOSE (45)												
Mali	PROMIS CS (46)												
Zimbabwe	SHINE (HIV−) (47)												
Zimbabwe	SHINE (HIV+) (48)												
		**753**	**454**		**I² = 0.65, Tau² = 0.00**			**589**	**333**		**I² = 0.03, Tau² = 0.00**		
**Fixed**					**0.82 (0.79, 0.86)**						**0.83 (0.79, 0.87)**		
**Random**					**0.81 (0.75, 0.87)**						**0.83 (0.79, 0.87)**		

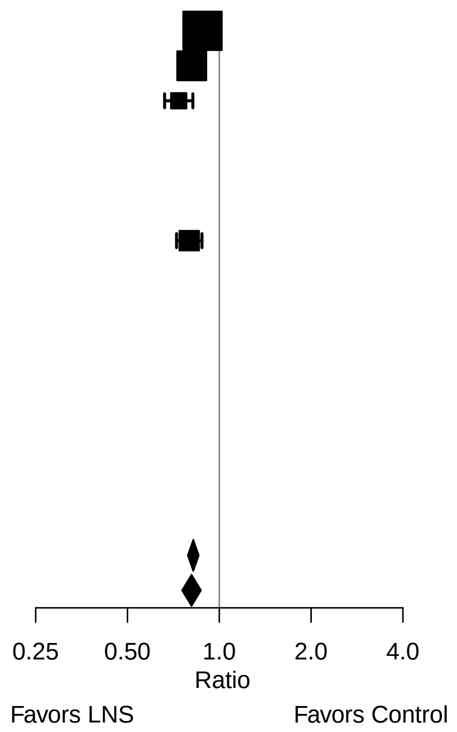

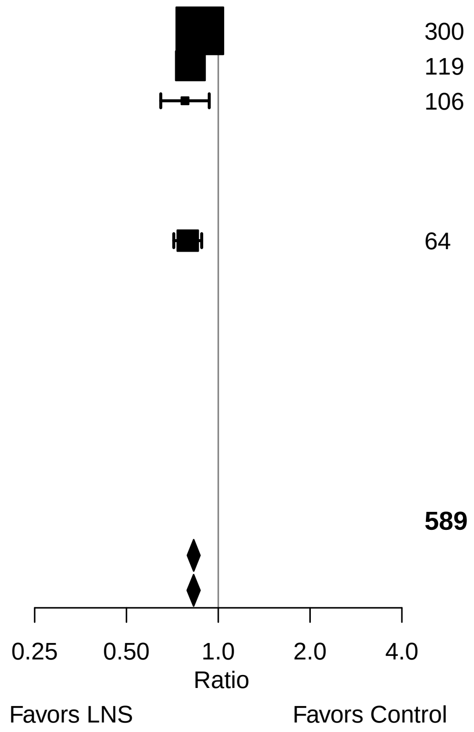

Supplemental figure 8K: Geometric mean ratio of soluble transferrin receptor concentration

8K2: Stratified by Maternal age

P−for−interaction = 0.213
Ratio of GMRs = 0.96 (0.91, 1.02)

		At least 25 y						Less than 25 y					
Country	Trial	LNS N	Control N	Control Median	GMR (95% CI)	Fixed W	Random W	LNS N	Control N	Control Median	GMR (95% CI)	Fixed W	Random W
Bangladesh	JiVitA−4 (35)												
Bangladesh	RDNS (36)	152	81	7.8	0.88 (0.79, 0.97)	0.20	0.20	398	191	8.6	0.87 (0.83, 0.91)	0.58	0.29
Bangladesh	WASH−B (37)	79	80	7.3	0.90 (0.82, 0.98)	0.27	0.25	132	98	7.6	0.76 (0.69, 0.84)	0.12	0.18
Burkina Faso	iLiNS−Zinc (38)	178	50	9.5	0.77 (0.67, 0.89)	0.09	0.11	137	46	10.1	0.75 (0.68, 0.82)	0.13	0.19
Burkina Faso	PROMIS CS (39)												
Ghana	GHANA (40)	62	55	9.6	0.79 (0.70, 0.89)	0.14	0.15	22	21	12.8	0.83 (0.65, 1.05)	0.02	0.06
Ghana	iLiNS−DYAD−G (41)												
Kenya	WASH−B (42)	178	149	10.8	0.80 (0.73, 0.88)	0.26	0.24	116	109	11.3	0.78 (0.70, 0.87)	0.11	0.18
Madagascar	MAHAY (43)	47	25	9.0	0.85 (0.68, 1.06)	0.04	0.05	36	26	9.0	0.94 (0.78, 1.14)	0.04	0.09
Malawi	iLiNS−DYAD−M (44)												
Malawi	iLiNS−DOSE (45)												
Mali	PROMIS CS (46)												
Zimbabwe	SHINE (HIV−) (47)												
Zimbabwe	SHINE (HIV+) (48)												
		**696**	**440**		**I² = 0.23, Tau² = 0.00**			**841**	**491**		**I² = 0.63, Tau² = 0.00**		
**Fixed**					**0.84 (0.80, 0.88)**						**0.83 (0.80, 0.86)**		
**Random**					**0.84 (0.79, 0.88)**						**0.81 (0.76, 0.87)**		

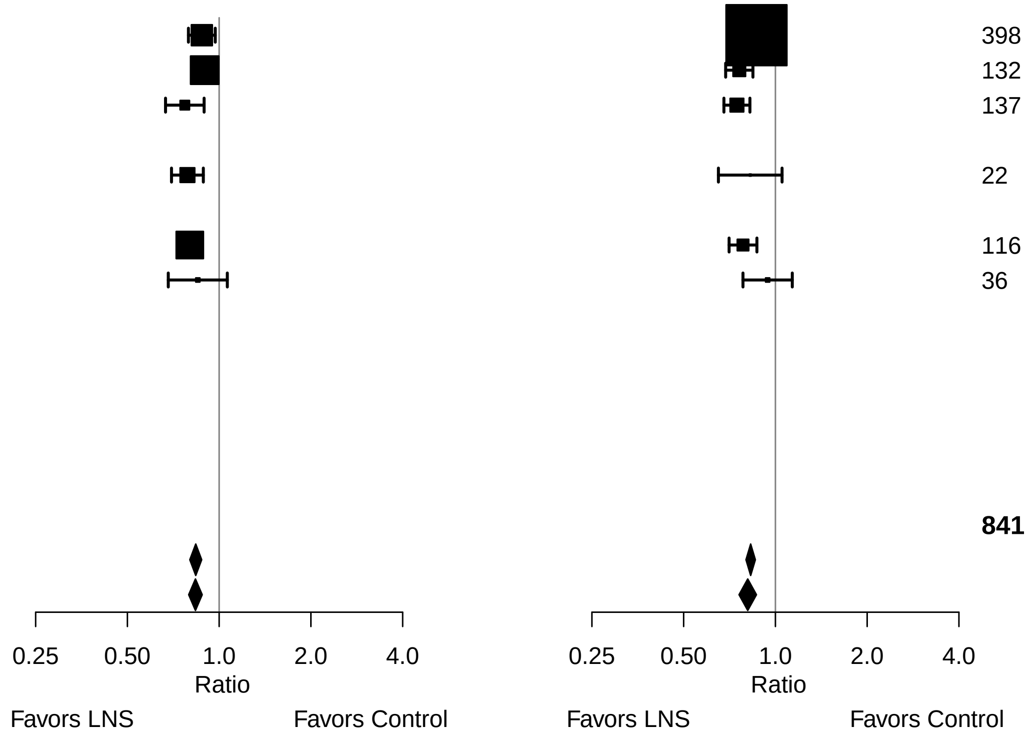

Supplemental figure 8K: Geometric mean ratio of soluble transferrin receptor concentration

8K3: Stratified by Maternal education

P−for−interaction = 0.576
Ratio of GMRs = 0.98 (0.92, 1.05)

Country	Trial	Primary or greater: LNS N	Control N	Control Median	GMR (95% CI)	Fixed W	Random W	Incomplete or no formal: LNS N	Control N	Control Median	GMR (95% CI)	Fixed W	Random W
Bangladesh	JiVitA−4 (35)												
Bangladesh	RDNS (36)	406	195	8.2	0.89 (0.84, 0.94)	0.47	0.33	144	77	8.9	0.82 (0.74, 0.91)	0.31	0.31
Bangladesh	WASH−B (37)	149	139	7.6	0.82 (0.76, 0.88)	0.27	0.27	63	39	7.5	0.81 (0.71, 0.93)	0.18	0.18
Burkina Faso	iLiNS−Zinc (38)												
Burkina Faso	PROMIS CS (39)												
Ghana	GHANA (40)	77	65	9.3	0.83 (0.74, 0.93)	0.11	0.16	7	13	12.9	0.69 (0.52, 0.91)	0.04	0.04
Ghana	iLiNS−DYAD−G (41)												
Kenya	WASH−B (42)	143	117	11.3	0.76 (0.68, 0.84)	0.13	0.19	154	142	10.4	0.82 (0.75, 0.90)	0.38	0.38
Madagascar	MAHAY (43)	16	16	8.8	0.86 (0.67, 1.12)	0.02	0.05	67	35	9.0	0.90 (0.75, 1.08)	0.09	0.09
Malawi	iLiNS−DYAD−M (44)												
Malawi	iLiNS−DOSE (45)												
Mali	PROMIS CS (46)												
Zimbabwe	SHINE (HIV−) (47)												
Zimbabwe	SHINE (HIV+) (48)												
		**791**	**532**		**I² = 0.55, Tau² = 0.00**			**435**	**306**		**I² = 0.00, Tau² = 0.00**		
**Fixed**					**0.84 (0.81, 0.88)**						**0.82 (0.78, 0.87)**		
**Random**					**0.83 (0.79, 0.88)**						**0.82 (0.78, 0.87)**		

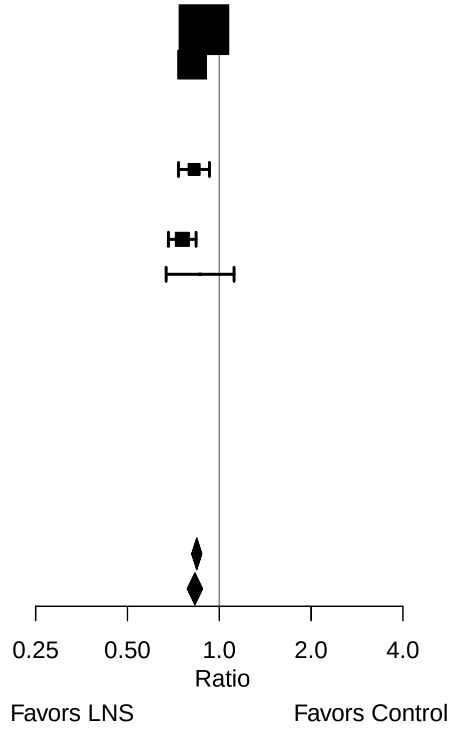

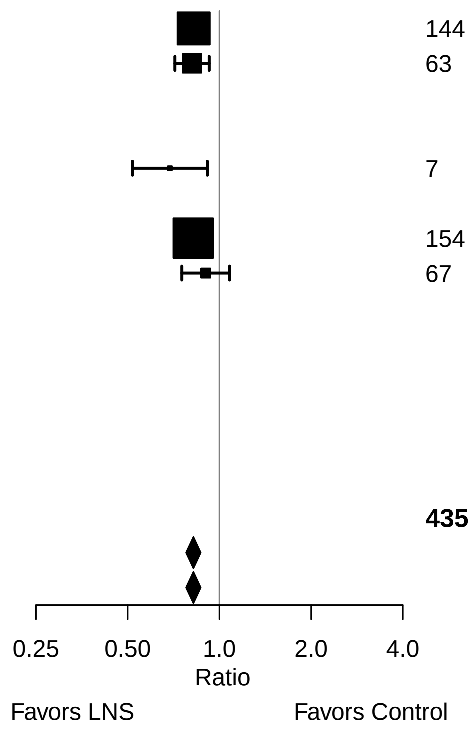

Supplemental figure 8K: Geometric mean ratio of soluble transferrin receptor concentration

8K4: Stratified by Child sex

P−for−interaction = 0.268
Ratio of GMRs = 1.03 (0.98, 1.09)

					**Male**						**Female**		
		**LNS**	**Control**	**Control**	**GMR**	**Fixed**	**Random**	**LNS**	**Control**	**Control**	**GMR**	**Fixed**	**Random**
Country	Trial	N	N	Median	(95% CI)	W	W	N	N	Median	(95% CI)	W	W
Bangladesh	JiVitA−4 (35)												
Bangladesh	RDNS (36)	266	131	8.8	0.84 (0.77, 0.91)	0.33	0.33	281	141	7.7	0.90 (0.86, 0.94)	0.51	0.25
Bangladesh	WASH−B (37)	105	85	7.9	0.80 (0.70, 0.90)	0.15	0.15	107	93	7.4	0.83 (0.76, 0.92)	0.14	0.18
Burkina Faso	iLiNS−Zinc (38)	150	49	10.3	0.74 (0.64, 0.85)	0.12	0.12	165	47	9.5	0.78 (0.70, 0.88)	0.09	0.16
Burkina Faso	PROMIS CS (39)												
Ghana	GHANA (40)	52	37	10.8	0.79 (0.68, 0.92)	0.10	0.10	32	45	9.3	0.78 (0.67, 0.91)	0.05	0.11
Ghana	iLiNS−DYAD−G (41)												
Kenya	WASH−B (42)	146	125	11.1	0.82 (0.74, 0.90)	0.25	0.25	152	134	10.7	0.77 (0.70, 0.83)	0.17	0.20
Madagascar	MAHAY (43)	34	22	9.3	0.83 (0.67, 1.04)	0.05	0.05	49	29	7.7	0.95 (0.80, 1.14)	0.04	0.09
Malawi	iLiNS−DYAD−M (44)												
Malawi	iLiNS−DOSE (45)												
Mali	PROMIS CS (46)												
Zimbabwe	SHINE (HIV−) (47)												
Zimbabwe	SHINE (HIV+) (48)												
		**753**	**449**		**I² = 0.00, Tau² = 0.00**			**786**	**489**		**I² = 0.70, Tau² = 0.00**		
**Fixed**					**0.81 (0.77, 0.85)**						**0.85 (0.82, 0.88)**		
**Random**					**0.81 (0.77, 0.85)**						**0.83 (0.78, 0.89)**		

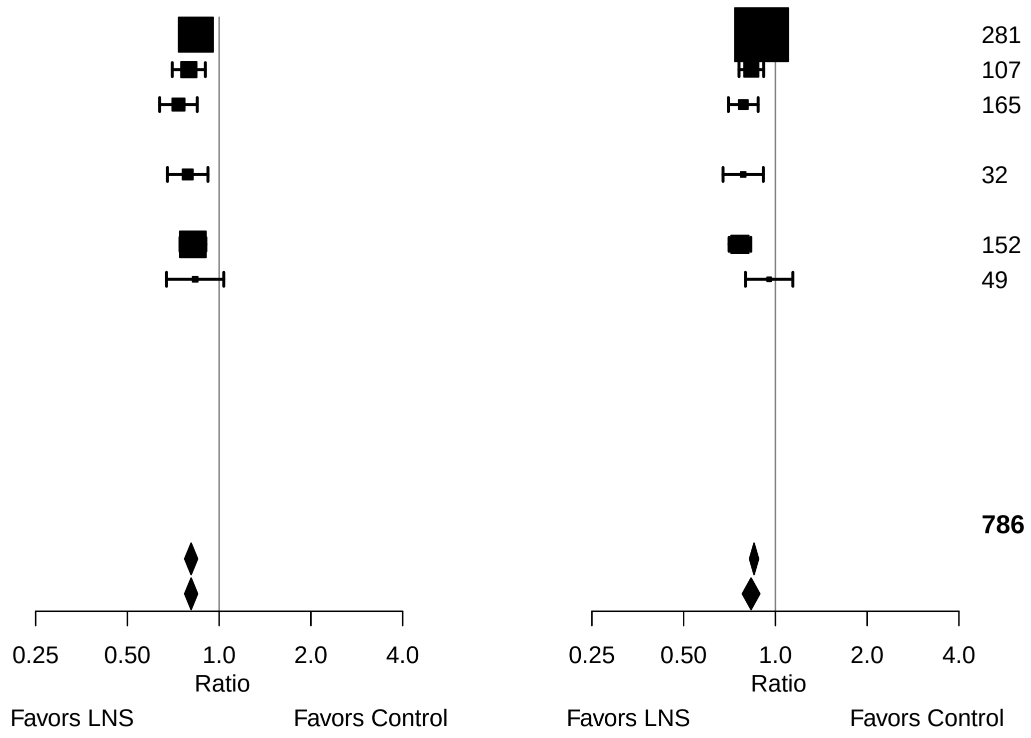

Supplemental figure 8K: Geometric mean ratio of soluble transferrin receptor concentration

8K5: Stratified by Child birth order

P−for−interaction = 0.731
Ratio of GMRs = 0.99 (0.93, 1.05)

		Later born						Firstborn					
Country	Trial	LNS N	Control N	Control Median	GMR (95% CI)	Fixed W	Random W	LNS N	Control N	Control Median	GMR (95% CI)	Fixed W	Random W
Bangladesh	JiVitA−4 (35)												
Bangladesh	RDNS (36)	340	171	8.3	0.87 (0.81, 0.92)	0.29	0.23	210	101	8.1	0.88 (0.81, 0.94)	0.47	0.28
Bangladesh	WASH−B (37)	124	119	7.6	0.83 (0.78, 0.89)	0.28	0.23	86	57	7.4	0.79 (0.68, 0.90)	0.13	0.17
Burkina Faso	iLiNS−Zinc (38)	249	71	9.0	0.78 (0.68, 0.90)	0.06	0.11	66	25	10.3	0.70 (0.63, 0.79)	0.20	0.21
Burkina Faso	PROMIS CS (39)												
Ghana	GHANA (40)	54	50	9.8	0.76 (0.67, 0.86)	0.08	0.12	30	29	9.4	0.88 (0.72, 1.07)	0.07	0.11
Ghana	iLiNS−DYAD−G (41)												
Kenya	WASH−B (42)	237	213	10.8	0.80 (0.74, 0.86)	0.23	0.21	60	46	11.3	0.76 (0.65, 0.90)	0.10	0.15
Madagascar	MAHAY (43)	61	37	8.8	0.95 (0.82, 1.11)	0.05	0.10	21	11	9.4	0.91 (0.69, 1.18)	0.04	0.07
Malawi	iLiNS−DYAD−M (44)												
Malawi	iLiNS−DOSE (45)												
Mali	PROMIS CS (46)												
Zimbabwe	SHINE (HIV−) (47)												
Zimbabwe	SHINE (HIV+) (48)												
		**1065**	**661**		**I² = 0.44, Tau² = 0.00**			**473**	**269**		**I² = 0.60, Tau² = 0.00**		
**Fixed**					**0.83 (0.80, 0.86)**						**0.82 (0.78, 0.86)**		
**Random**					**0.83 (0.78, 0.88)**						**0.81 (0.74, 0.87)**		

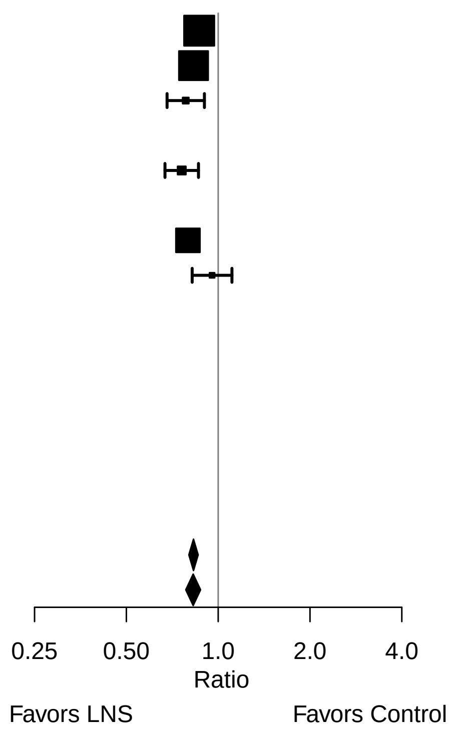

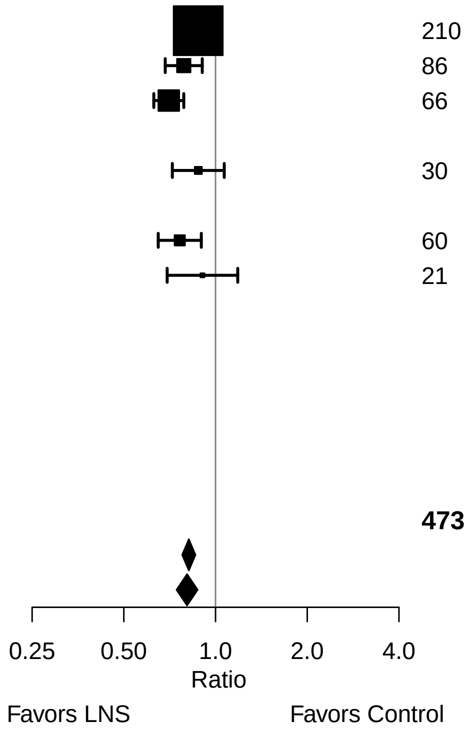

Supplemental figure 8K: Geometric mean ratio of soluble transferrin receptor concentration

8K6: Stratified by Child baseline acute malnutrition (insufficient comparisons)

Supplemental figure 8K: Geometric mean ratio of soluble transferrin receptor concentration

8K7: Stratified by Child baseline anemia (insufficient comparisons)

Supplemental figure 8K: Geometric mean ratio of soluble transferrin receptor concentration

8K8: Stratified by Child high-dose vitamin A supplementation

P−for−interaction = 0.373
Ratio of GMRs = 1.04 (0.96, 1.12)

Country	Trial	LNS N	Control N	Control Median	Received VitA GMR (95% CI)	Fixed W	Random W	LNS N	Control N	Control Median	Not treated GMR (95% CI)	Fixed W	Random W
Bangladesh	JiVitA−4 (35)												
Bangladesh	RDNS (36)	444	223	8.3	0.87 (0.82, 0.92)	0.73	0.51	105	49	7.8	0.89 (0.82, 0.96)	0.48	0.48
Bangladesh	WASH−B (37)	16	16	7.4	0.87 (0.75, 1.02)	0.10	0.19	15	9	8.1	0.84 (0.62, 1.15)	0.03	0.03
Burkina Faso	iLiNS−Zinc (38)												
Burkina Faso	PROMIS CS (39)												
Ghana	GHANA (40)	40	53	10.0	0.79 (0.69, 0.92)	0.12	0.21	44	26	9.0	0.83 (0.70, 0.98)	0.10	0.10
Ghana	iLiNS−DYAD−G (41)												
Kenya	WASH−B (42)	26	31	12.0	0.70 (0.55, 0.88)	0.05	0.10	223	180	11.0	0.81 (0.75, 0.89)	0.39	0.39
Madagascar	MAHAY (43)												
Malawi	iLiNS−DYAD−M (44)												
Malawi	iLiNS−DOSE (45)												
Mali	PROMIS CS (46)												
Zimbabwe	SHINE (HIV−) (47)												
Zimbabwe	SHINE (HIV+) (48)												
		**526**	**323**		**I² = 0.28, Tau² = 0.00**			**387**	**264**		**I² = 0.00, Tau² = 0.00**		
**Fixed**					**0.85 (0.81, 0.89)**						**0.85 (0.81, 0.90)**		
**Random**					**0.83 (0.77, 0.90)**						**0.85 (0.81, 0.90)**		

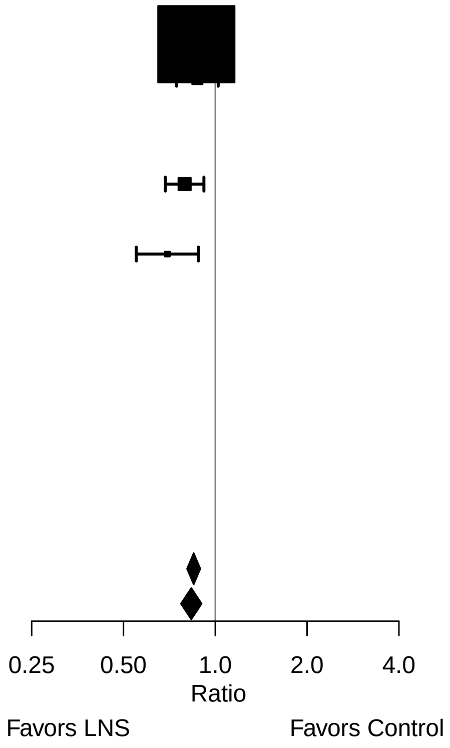

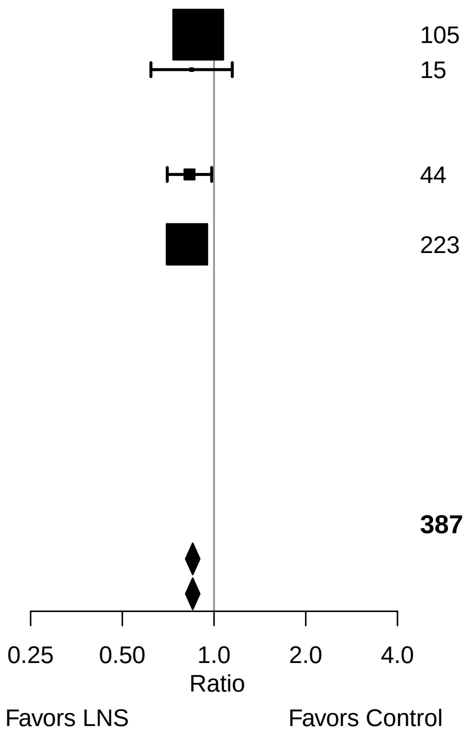

Supplemental figure 8K: Geometric mean ratio of soluble transferrin receptor concentration

8K9: Stratified by Child inflammation

P−for−interaction = 0.478
Ratio of GMRs = 0.98 (0.93, 1.03)

		Not inflamed						High AGP or CRP					
Country	Trial	LNS N	Control N	Control Median	GMR (95% CI)	Fixed W	Random W	LNS N	Control N	Control Median	GMR (95% CI)	Fixed W	Random W
Bangladesh	JiVitA−4 (35)												
Bangladesh	RDNS (36)	370	168	8.0	0.89 (0.83, 0.94)	0.38	0.25	180	104	9.0	0.85 (0.79, 0.91)	0.44	0.36
Bangladesh	WASH−B (37)	165	136	7.7	0.80 (0.74, 0.86)	0.25	0.22	47	42	7.2	0.88 (0.78, 1.00)	0.14	0.16
Burkina Faso	iLiNS−Zinc (38)	124	31	9.6	0.75 (0.66, 0.86)	0.08	0.12	191	65	10.0	0.76 (0.68, 0.86)	0.15	0.18
Burkina Faso	PROMIS CS (39)												
Ghana	GHANA (40)	70	66	9.6	0.79 (0.70, 0.89)	0.10	0.15	14	16	9.8	0.85 (0.65, 1.12)	0.03	0.04
Ghana	iLiNS−DYAD−G (41)												
Kenya	WASH−B (42)	157	135	10.1	0.83 (0.74, 0.93)	0.12	0.16	141	124	11.7	0.75 (0.68, 0.83)	0.21	0.23
Madagascar	MAHAY (43)	44	32	8.8	0.94 (0.81, 1.09)	0.06	0.11	39	19	9.4	0.83 (0.62, 1.10)	0.03	0.04
Malawi	iLiNS−DYAD−M (44)												
Malawi	iLiNS−DOSE (45)												
Mali	PROMIS CS (46)												
Zimbabwe	SHINE (HIV−) (47)												
Zimbabwe	SHINE (HIV+) (48)												
		**930**	**568**		**I² = 0.53, Tau² = 0.00**			**612**	**370**		**I² = 0.28, Tau² = 0.00**		
**Fixed**					**0.84 (0.81, 0.87)**						**0.82 (0.78, 0.86)**		
**Random**					**0.83 (0.78, 0.88)**						**0.81 (0.77, 0.86)**		

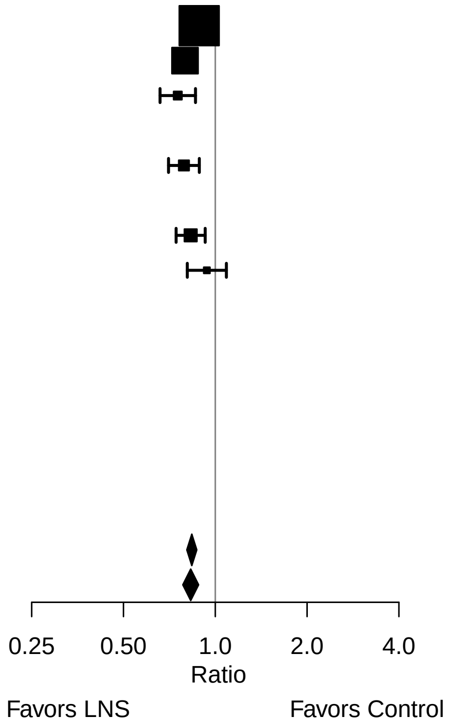

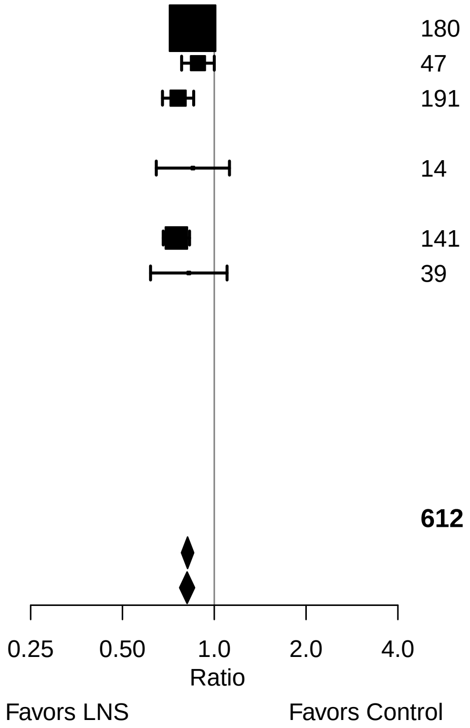

Supplemental figure 8L: Elevated soluble transferrin receptor prevalence ratio

8L1: Stratified by Maternal BMI

P−for−interaction = 0.126
Ratio of PRs = 0.85 (0.69, 1.05)

		At least 20 kg/m²						Less than 20 kg/m²					
Country	Trial	LNS N	Control N	Control Prevalence	PR (95% CI)	Fixed W	Random W	LNS N	Control N	Control Prevalence	PR (95% CI)	Fixed W	Random W
Bangladesh	JiVitA−4 (35)												
Bangladesh	RDNS (36)	228	102	44.1	0.81 (0.61, 1.06)	0.24	0.29	300	160	51.2	0.68 (0.55, 0.85)	0.52	0.39
Bangladesh	WASH−B (37)												
Burkina Faso	iLiNS−Zinc (38)	209	55	60.7	0.56 (0.40, 0.78)	0.16	0.22	106	41	62.2	0.43 (0.30, 0.62)	0.18	0.27
Burkina Faso	PROMIS CS (39)												
Ghana	GHANA (40)												
Ghana	iLiNS−DYAD−G (41)												
Kenya	WASH−B (42)	223	200	72.0	0.70 (0.59, 0.83)	0.60	0.49	64	51	76.5	0.65 (0.49, 0.87)	0.30	0.34
Madagascar	MAHAY (43)												
Malawi	iLiNS−DYAD−M (44)												
Malawi	iLiNS−DOSE (45)												
Mali	PROMIS CS (46)												
Zimbabwe	SHINE (HIV−) (47)												
Zimbabwe	SHINE (HIV+) (48)												
		**660**	**357**		**I² = 0.27, Tau² = 0.01**			**470**	**252**		**I² = 0.59, Tau² = 0.04**		
**Fixed**					**0.70 (0.61, 0.80)**						**0.62 (0.53, 0.72)**		
**Random**					**0.69 (0.58, 0.83)**						**0.59 (0.45, 0.78)**		

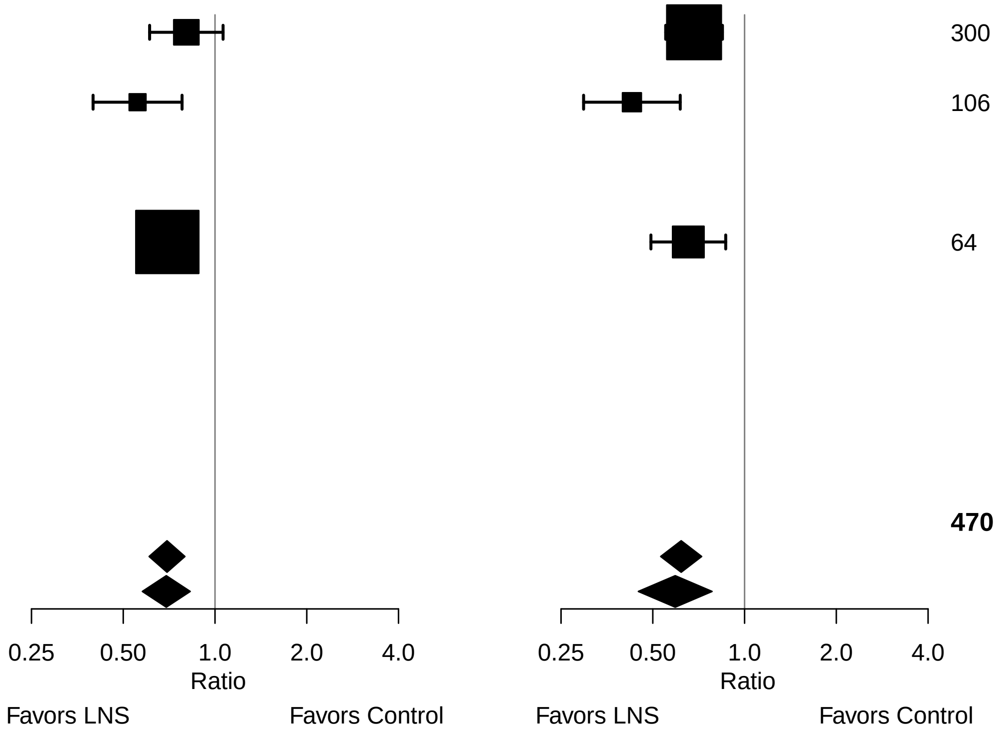

Supplemental figure 8L: Elevated soluble transferrin receptor prevalence ratio

8L2: Stratified by Maternal age

P−for−interaction = 0.585
Ratio of PRs = 0.96 (0.81, 1.13)

		At least 25 y						Less than 25 y					
Country	Trial	LNS N	Control N	Control Prevalence	PR (95% CI)	Fixed W	Random W	LNS N	Control N	Control Prevalence	PR (95% CI)	Fixed W	Random W
Bangladesh	JiVitA−4 (35)												
Bangladesh	RDNS (36)	152	81	40.7	0.78 (0.55, 1.09)	0.12	0.13	398	191	53.9	0.68 (0.56, 0.81)	0.41	0.19
Bangladesh	WASH−B (37)	79	80	26.2	0.58 (0.33, 1.02)	0.05	0.05	132	98	35.7	0.17 (0.08, 0.34)	0.03	0.13
Burkina Faso	iLiNS−Zinc (38)	178	50	61.1	0.49 (0.34, 0.71)	0.10	0.10	137	46	61.5	0.54 (0.40, 0.75)	0.14	0.18
Burkina Faso	PROMIS CS (39)												
Ghana	GHANA (40)	62	55	63.6	0.53 (0.36, 0.79)	0.09	0.09	22	21	61.9	0.66 (0.36, 1.21)	0.04	0.14
Ghana	iLiNS−DYAD−G (41)												
Kenya	WASH−B (42)	178	149	72.5	0.68 (0.58, 0.80)	0.58	0.57	116	109	73.4	0.66 (0.53, 0.81)	0.32	0.19
Madagascar	MAHAY (43)	47	25	56.0	0.76 (0.46, 1.26)	0.06	0.06	36	26	61.5	0.90 (0.58, 1.40)	0.07	0.16
Malawi	iLiNS−DYAD−M (44)												
Malawi	iLiNS−DOSE (45)												
Mali	PROMIS CS (46)												
Zimbabwe	SHINE (HIV−) (47)												
Zimbabwe	SHINE (HIV+) (48)												
		**696**	**440**		**I² = 0.01, Tau² = 0.00**			**841**	**491**		**I² = 0.72, Tau² = 0.24**		
**Fixed**					**0.65 (0.58, 0.74)**						**0.64 (0.57, 0.72)**		
**Random**					**0.65 (0.58, 0.74)**						**0.56 (0.37, 0.87)**		

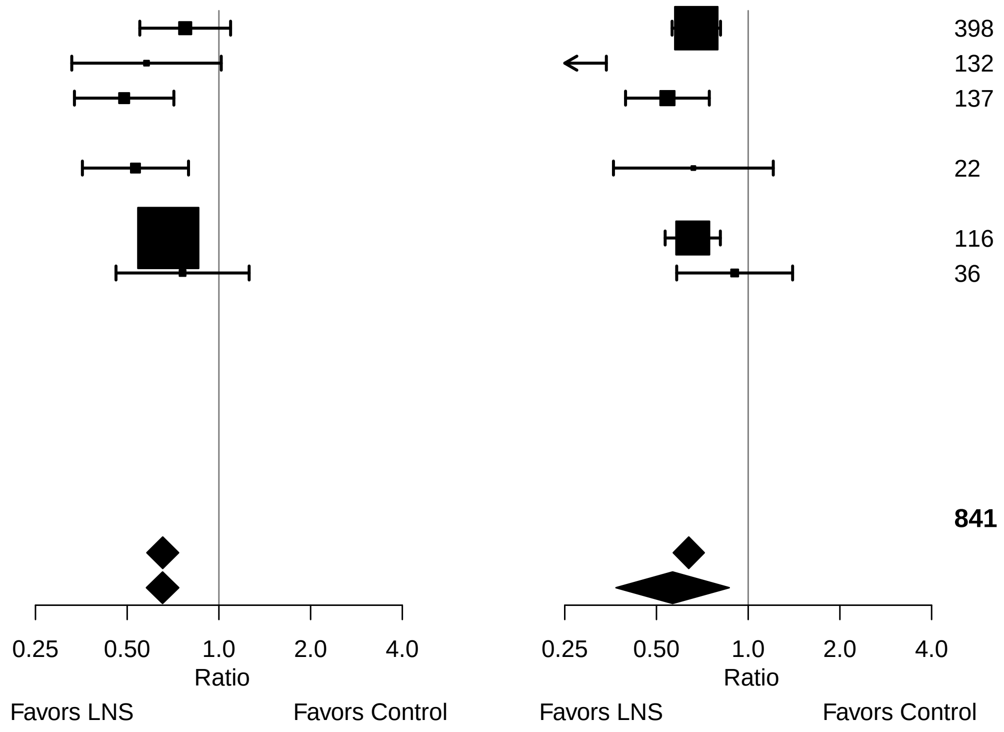

Supplemental figure 8L: Elevated soluble transferrin receptor prevalence ratio

8L3: Stratified by Maternal education

P−for−interaction = 0.227
Ratio of PRs = 1.12 (0.93, 1.35)

		Primary or greater						Incomplete or no formal					
Country	Trial	LNS N	Control N	Control Prevalence	PR (95% CI)	Fixed W	Random W	LNS N	Control N	Control Prevalence	PR (95% CI)	Fixed W	Random W
Bangladesh	JiVitA−4 (35)												
Bangladesh	RDNS (36)	406	195	47.7	0.71 (0.58, 0.86)	0.45	0.33	144	77	55.8	0.70 (0.53, 0.91)	0.28	0.28
Bangladesh	WASH−B (37)	149	139	33.1	0.28 (0.16, 0.50)	0.05	0.20	63	39	25.6	0.43 (0.19, 1.00)	0.03	0.03
Burkina Faso	iLiNS−Zinc (38)												
Burkina Faso	PROMIS CS (39)												
Ghana	GHANA (40)												
Ghana	iLiNS−DYAD−G (41)												
Kenya	WASH−B (42)	143	117	77.8	0.59 (0.49, 0.72)	0.47	0.33	154	142	69.0	0.74 (0.61, 0.90)	0.53	0.53
Madagascar	MAHAY (43)	16	16	50.0	0.63 (0.28, 1.38)	0.03	0.14	67	35	62.9	0.83 (0.59, 1.17)	0.17	0.17
Malawi	iLiNS−DYAD−M (44)												
Malawi	iLiNS−DOSE (45)												
Mali	PROMIS CS (46)												
Zimbabwe	SHINE (HIV−) (47)												
Zimbabwe	SHINE (HIV+) (48)												
		**714**	**467**		**I² = 0.67, Tau² = 0.10**			**428**	**293**		**I² = 0.00, Tau² = 0.00**		
**Fixed**					**0.62 (0.54, 0.70)**						**0.73 (0.64, 0.84)**		
**Random**					**0.55 (0.37, 0.80)**						**0.73 (0.64, 0.84)**		

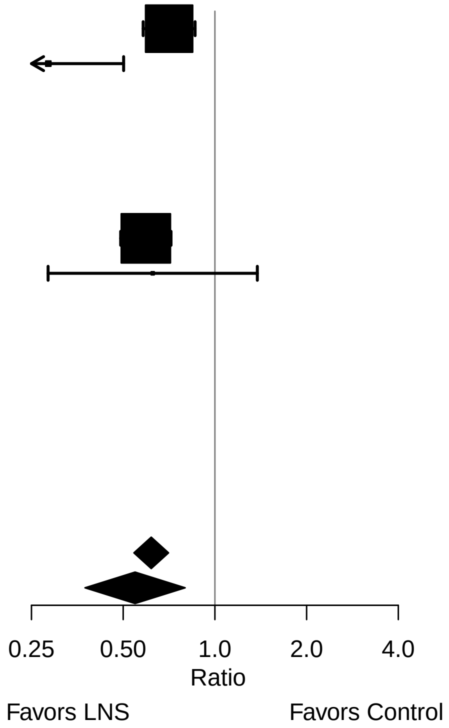

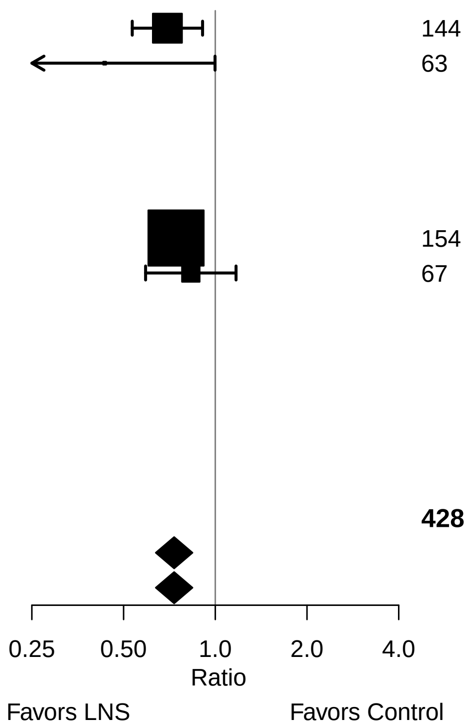

Supplemental figure 8L: Elevated soluble transferrin receptor prevalence ratio

8L4: Stratified by Child sex

P−for−interaction = 0.787
Ratio of PRs = 0.98 (0.83, 1.16)

		Male						Female					
		**LNS**	**Control**	**Control**	**PR**	**Fixed**	**Random**	**LNS**	**Control**	**Control**	**PR**	**Fixed**	**Random**
Country	Trial	N	N	Prevalence	(95% CI)	W	W	N	N	Prevalence	(95% CI)	W	W
Bangladesh	JiVitA−4 (35)												
Bangladesh	RDNS (36)	266	131	58.0	0.70 (0.56, 0.87)	0.28	0.21	281	141	42.6	0.70 (0.54, 0.91)	0.23	0.22
Bangladesh	WASH−B (37)	105	85	37.6	0.30 (0.17, 0.54)	0.04	0.10	107	93	25.8	0.33 (0.17, 0.61)	0.04	0.10
Burkina Faso	iLiNS−Zinc (38)	150	49	66.7	0.51 (0.33, 0.78)	0.07	0.14	165	47	55.8	0.53 (0.40, 0.70)	0.19	0.21
Burkina Faso	PROMIS CS (39)												
Ghana	GHANA (40)	52	37	73.0	0.58 (0.40, 0.84)	0.10	0.16	32	45	53.3	0.47 (0.24, 0.91)	0.04	0.09
Ghana	iLiNS−DYAD−G (41)												
Kenya	WASH−B (42)	146	125	73.6	0.70 (0.58, 0.84)	0.41	0.23	152	134	72.4	0.65 (0.53, 0.78)	0.43	0.25
Madagascar	MAHAY (43)	34	22	72.7	0.77 (0.53, 1.12)	0.09	0.16	49	29	48.3	0.89 (0.56, 1.41)	0.07	0.14
Malawi	iLiNS−DYAD−M (44)												
Malawi	iLiNS−DOSE (45)												
Mali	PROMIS CS (46)												
Zimbabwe	SHINE (HIV−) (47)												
Zimbabwe	SHINE (HIV+) (48)												
		**753**	**449**		**I^2 = 0.51, Tau^2 = 0.05**			**786**	**489**		**I^2 = 0.47, Tau^2 = 0.05**		
**Fixed**					**0.65 (0.58, 0.73)**						**0.62 (0.55, 0.71)**		
**Random**					**0.60 (0.48, 0.76)**						**0.60 (0.47, 0.76)**		

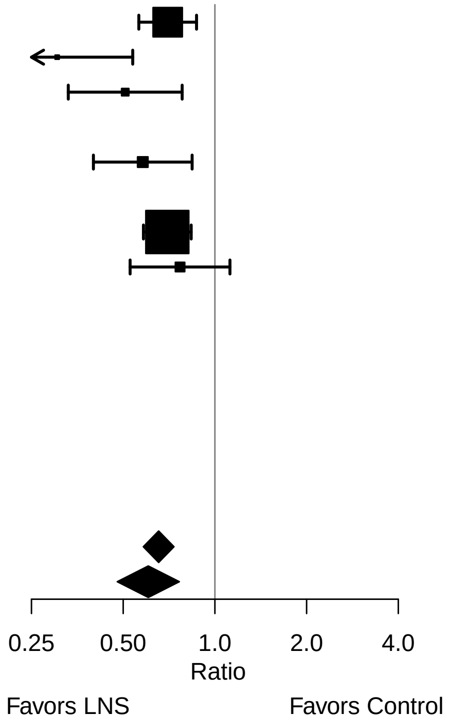

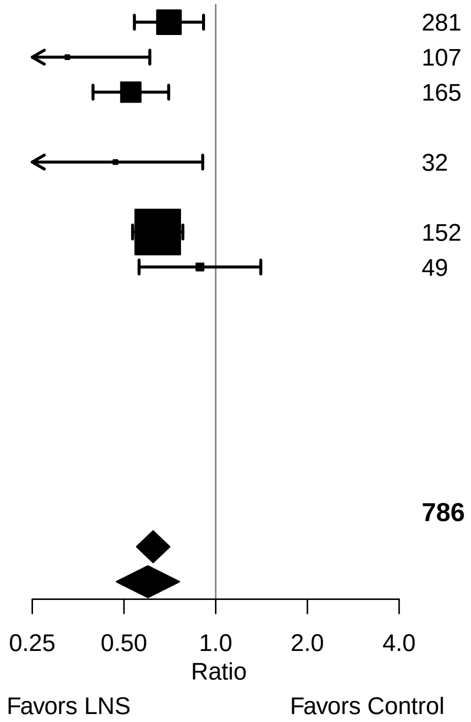

Supplemental figure 8L: Elevated soluble transferrin receptor prevalence ratio

8L5: Stratified by Child birth order

P−for−interaction = 0.638
Ratio of PRs = 0.96 (0.80, 1.15)

Country	Trial	Later born LNS N	Later born Control N	Later born Control Prevalence	Later born PR (95% CI)	Later born Fixed W	Later born Random W	Firstborn LNS N	Firstborn Control N	Firstborn Control Prevalence	Firstborn PR (95% CI)	Firstborn Fixed W	Firstborn Random W
Bangladesh	JiVitA−4 (35)												
Bangladesh	RDNS (36)	340	171	50.9	0.67 (0.55, 0.82)	0.28	0.28	210	101	48.5	0.76 (0.59, 0.97)	0.35	0.23
Bangladesh	WASH−B (37)	124	119	30.3	0.40 (0.25, 0.65)	0.05	0.11	86	57	33.3	0.17 (0.07, 0.43)	0.03	0.13
Burkina Faso	iLiNS−Zinc (38)	249	71	54.7	0.56 (0.38, 0.82)	0.08	0.15	66	25	77.4	0.43 (0.32, 0.57)	0.27	0.23
Burkina Faso	PROMIS CS (39)												
Ghana	GHANA (40)	54	50	62.0	0.48 (0.30, 0.76)	0.05	0.12	30	29	62.1	0.75 (0.47, 1.21)	0.10	0.19
Ghana	iLiNS−DYAD−G (41)												
Kenya	WASH−B (42)	237	213	72.8	0.70 (0.60, 0.81)	0.55	0.34	60	46	73.9	0.56 (0.42, 0.75)	0.26	0.22
Madagascar	MAHAY (43)												
Malawi	iLiNS−DYAD−M (44)												
Malawi	iLiNS−DOSE (45)												
Mali	PROMIS CS (46)												
Zimbabwe	SHINE (HIV−) (47)												
Zimbabwe	SHINE (HIV+) (48)												
		**1004**	**624**		**I² = 0.43, Tau² = 0.02**			**452**	**258**		**I² = 0.76, Tau² = 0.22**		
**Fixed**					**0.65 (0.58, 0.72)**						**0.58 (0.50, 0.67)**		
**Random**					**0.60 (0.50, 0.72)**						**0.52 (0.33, 0.82)**		

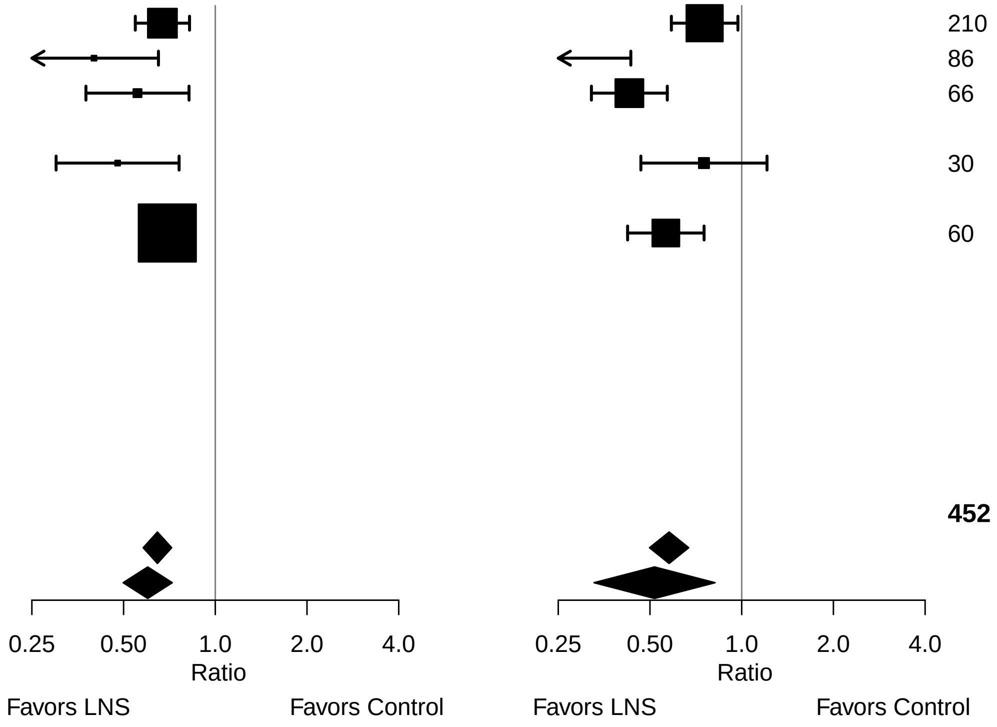

Supplemental figure 8L: Elevated soluble transferrin receptor prevalence ratio

8L6: Stratified by Child baseline acute malnutrition (insufficient comparisons)

Supplemental figure 8L: Elevated soluble transferrin receptor prevalence ratio

8L7: Stratified by Child baseline anemia (insufficient comparisons)

Supplemental figure 8L: Elevated soluble transferrin receptor prevalence ratio

8L8: Stratified by Child high-dose vitamin A supplementation

P−for−interaction = 0.344
Ratio of PRs = 1.14 (0.87, 1.51)

		Received VitA						Not treated					
		**LNS**	**Control**	**Control**	**PR**	**Fixed**	**Random**	**LNS**	**Control**	**Control**	**PR**	**Fixed**	**Random**
Country	Trial	N	N	Prevalence	(95% CI)	W	W	N	N	Prevalence	(95% CI)	W	W
Bangladesh	JiVitA−4 (35)												
Bangladesh	RDNS (36)	444	223	50.7	0.69 (0.57, 0.84)	0.76	0.69	105	49	46.9	0.73 (0.52, 1.02)	0.14	0.14
Bangladesh	WASH−B (37)												
Burkina Faso	iLiNS−Zinc (38)												
Burkina Faso	PROMIS CS (39)												
Ghana	GHANA (40)	40	53	66.0	0.61 (0.40, 0.93)	0.16	0.20	44	26	53.8	0.59 (0.34, 1.03)	0.05	0.05
Ghana	iLiNS−DYAD−G (41)												
Kenya	WASH−B (42)	26	31	71.0	0.43 (0.24, 0.79)	0.08	0.11	223	180	75.0	0.72 (0.63, 0.82)	0.81	0.81
Madagascar	MAHAY (43)												
Malawi	iLiNS−DYAD−M (44)												
Malawi	iLiNS−DOSE (45)												
Mali	PROMIS CS (46)												
Zimbabwe	SHINE (HIV−) (47)												
Zimbabwe	SHINE (HIV+) (48)												
		**510**	**307**		**I² = 0.12, Tau² = 0.01**			**372**	**255**		**I² = 0.00, Tau² = 0.00**		
**Fixed**					**0.65 (0.55, 0.77)**						**0.71 (0.63, 0.81)**		
**Random**					**0.64 (0.53, 0.79)**						**0.71 (0.63, 0.81)**		

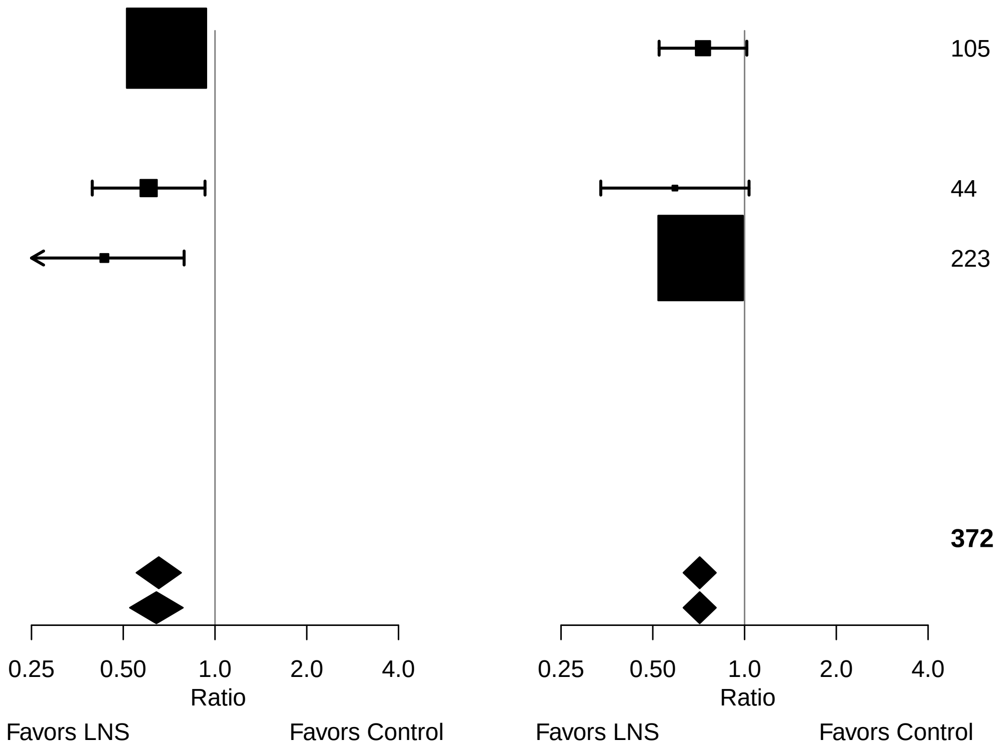

Supplemental figure 8L: Elevated soluble transferrin receptor prevalence ratio

8L9: Stratified by Child inflammation

P−for−interaction = 0.761
Ratio of PRs = 0.97 (0.82, 1.16)

Country	Trial	LNS N	Control N	Control Prevalence	Not inflamed PR (95% CI)	Fixed W	Random W	LNS N	Control N	Control Prevalence	High AGP or CRP PR (95% CI)	Fixed W	Random W
Bangladesh	JiVitA−4 (35)												
Bangladesh	RDNS (36)	370	168	43.5	0.77 (0.61, 0.97)	0.28	0.19	180	104	60.6	0.64 (0.51, 0.82)	0.29	0.29
Bangladesh	WASH−B (37)	165	136	34.6	0.28 (0.18, 0.45)	0.07	0.15	47	42	21.4	0.50 (0.21, 1.17)	0.02	0.02
Burkina Faso	iLiNS−Zinc (38)	124	31	58.8	0.42 (0.25, 0.68)	0.06	0.14	191	65	62.5	0.58 (0.43, 0.77)	0.19	0.19
Burkina Faso	PROMIS CS (39)												
Ghana	GHANA (40)	70	66	62.1	0.53 (0.36, 0.78)	0.10	0.16	14	16	62.5	0.80 (0.42, 1.53)	0.04	0.04
Ghana	iLiNS−DYAD−G (41)												
Kenya	WASH−B (42)	157	135	68.9	0.70 (0.57, 0.86)	0.36	0.19	141	124	77.4	0.64 (0.52, 0.79)	0.39	0.39
Madagascar	MAHAY (43)	44	32	53.1	0.98 (0.70, 1.39)	0.13	0.17	39	19	68.4	0.64 (0.38, 1.06)	0.06	0.06
Malawi	iLiNS−DYAD−M (44)												
Malawi	iLiNS−DOSE (45)												
Mali	PROMIS CS (46)												
Zimbabwe	SHINE (HIV−) (47)												
Zimbabwe	SHINE (HIV+) (48)												
		**930**	**568**		**I² = 0.79, Tau² = 0.16**			**612**	**370**		**I² = 0.00, Tau² = 0.00**		
**Fixed**					**0.66 (0.59, 0.75)**						**0.63 (0.55, 0.72)**		
**Random**					**0.58 (0.41, 0.83)**						**0.63 (0.55, 0.72)**		

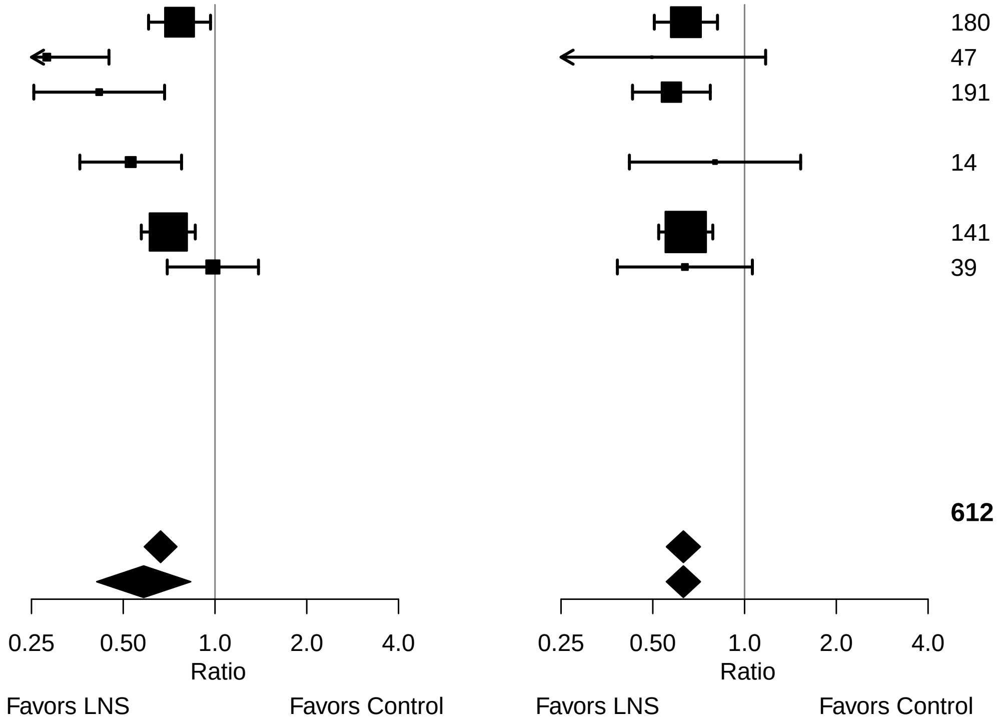

Supplemental figure 8M: Elevated soluble transferrin receptor prevalence difference

8M1: Stratified by Maternal BMI

P−for−interaction = 0.187
Difference in PDs = −0.07 (−0.17, 0.03)

Country	Trial	At least 20 kg/m² LNS N	Control N	Control Prevalence	PD (95% CI)	Fixed W	Random W	Less than 20 kg/m² LNS N	Control N	Control Prevalence	PD (95% CI)	Fixed W	Random W
Bangladesh	JiVitA−4 (35)												
Bangladesh	RDNS (36)	228	102	44.1	−0.09 (−0.20, 0.03)	0.35	0.35	300	160	51.2	−0.16 (−0.25, −0.07)	0.64	0.50
Bangladesh	WASH−B (37)												
Burkina Faso	iLiNS−Zinc (38)	209	55	60.7	−0.27 (−0.42, −0.12)	0.20	0.27	106	41	62.2	−0.35 (−0.54, −0.16)	0.15	0.23
Burkina Faso	PROMIS CS (39)												
Ghana	GHANA (40)												
Ghana	iLiNS−DYAD−G (41)												
Kenya	WASH−B (42)	223	200	72.0	−0.22 (−0.32, −0.12)	0.45	0.38	64	51	76.5	−0.26 (−0.43, −0.10)	0.20	0.28
Madagascar	MAHAY (43)												
Malawi	iLiNS−DYAD−M (44)												
Malawi	iLiNS−DOSE (45)												
Mali	PROMIS CS (46)												
Zimbabwe	SHINE (HIV−) (47)												
Zimbabwe	SHINE (HIV+) (48)												
		**660**	**357**		**I² = 0.55, Tau² = 0.00**			**470**	**252**		**I² = 0.44, Tau² = 0.00**		
**Fixed**					**−0.18 (−0.25, −0.11)**						**−0.21 (−0.29, −0.14)**		
**Random**					**−0.19 (−0.29, −0.08)**						**−0.23 (−0.34, −0.13)**		

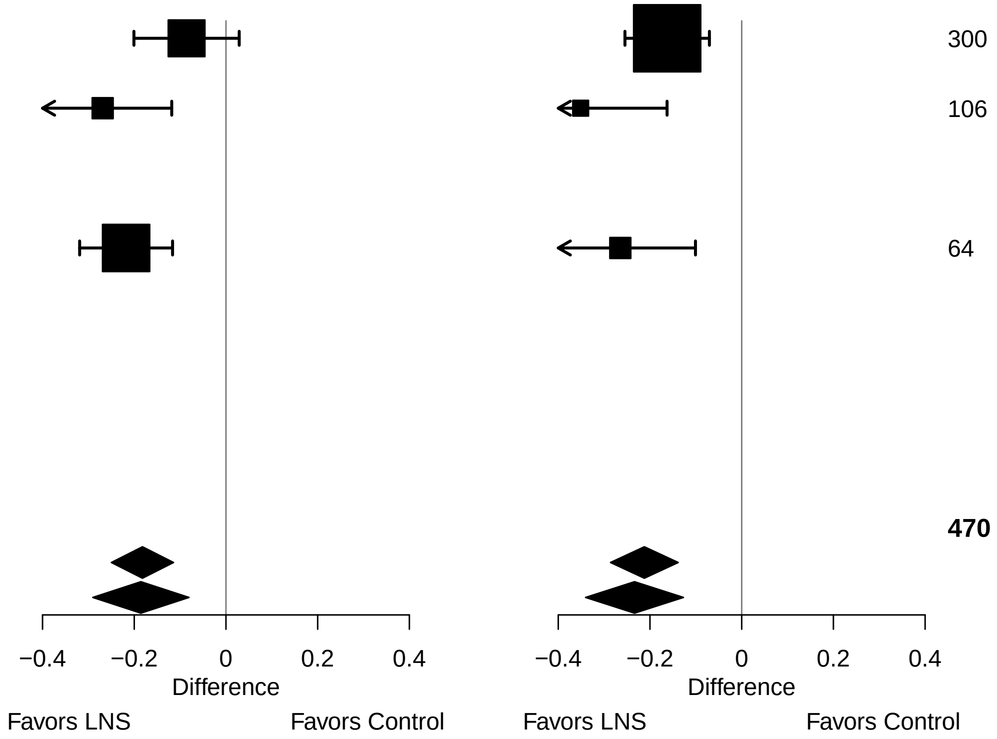

Supplemental figure 8M: Elevated soluble transferrin receptor prevalence difference

8M2: Stratified by Maternal age

P−for−interaction = 0.142
Difference in PDs = −0.06 (−0.14, 0.02)

		At least 25 y						Less than 25 y					
		**LNS**	**Control**	**Control**	**PD**	**Fixed**	**Random**	**LNS**	**Control**	**Control**	**PD**	**Fixed**	**Random**
Country	Trial	N	N	Prevalence	(95% CI)	W	W	N	N	Prevalence	(95% CI)	W	W
Bangladesh	JiVitA−4 (35)												
Bangladesh	RDNS (36)	152	81	40.7	−0.09 (−0.22, 0.04)	0.17	0.18	398	191	53.9	−0.17 (−0.26, −0.09)	0.42	0.39
Bangladesh	WASH−B (37)	79	80	26.2	−0.11 (−0.22, 0.00)	0.23	0.21	132	98	35.7	−0.30 (−0.41, −0.19)	0.23	0.23
Burkina Faso	iLiNS−Zinc (38)	178	50	61.1	−0.31 (−0.45, −0.16)	0.14	0.16	137	46	61.5	−0.28 (−0.46, −0.11)	0.09	0.10
Burkina Faso	PROMIS CS (39)												
Ghana	GHANA (40)	62	55	63.6	−0.30 (−0.47, −0.12)	0.09	0.13	22	21	61.9	−0.21 (−0.51, 0.09)	0.03	0.03
Ghana	iLiNS−DYAD−G (41)												
Kenya	WASH−B (42)	178	149	72.5	−0.23 (−0.32, −0.14)	0.33	0.25	116	109	73.4	−0.25 (−0.37, −0.13)	0.19	0.20
Madagascar	MAHAY (43)	47	25	56.0	−0.13 (−0.38, 0.11)	0.05	0.08	36	26	61.5	−0.06 (−0.32, 0.20)	0.04	0.05
Malawi	iLiNS−DYAD−M (44)												
Malawi	iLiNS−DOSE (45)												
Mali	PROMIS CS (46)												
Zimbabwe	SHINE (HIV−) (47)												
Zimbabwe	SHINE (HIV+) (48)												
		**696**	**440**		**I² = 0.45, Tau² = 0.00**			**841**	**491**		**I² = 0.07, Tau² = 0.00**		
**Fixed**					**−0.19 (−0.25, −0.14)**						**−0.22 (−0.28, −0.17)**		
**Random**					**−0.19 (−0.27, −0.12)**						**−0.22 (−0.28, −0.17)**		

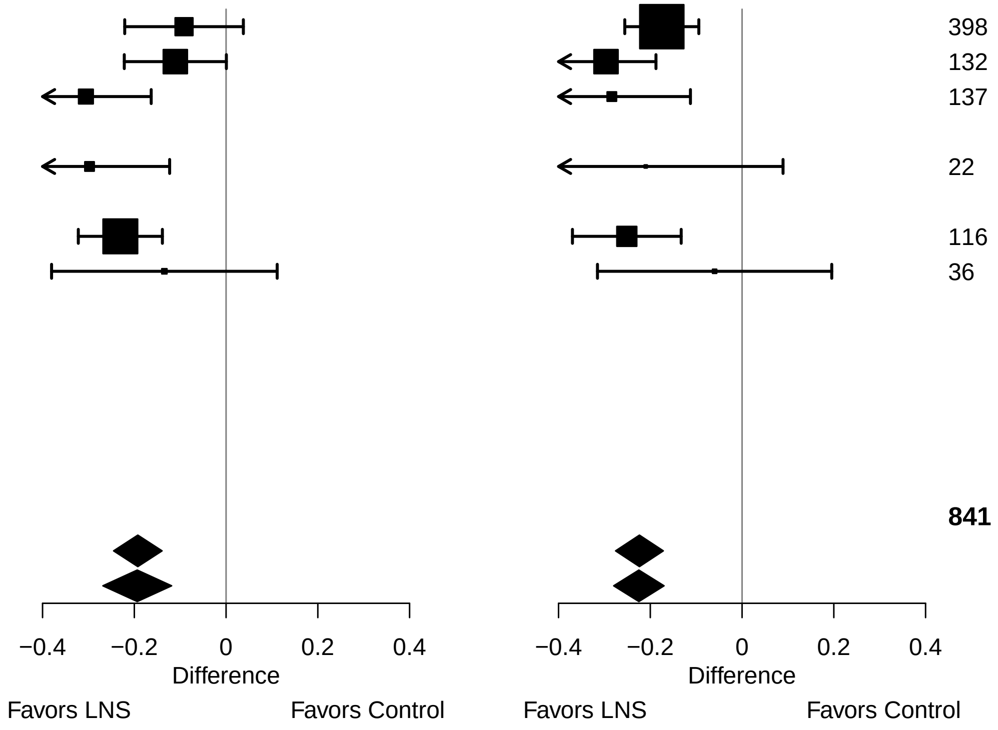

Supplemental figure 8M: Elevated soluble transferrin receptor prevalence difference

8M3: Stratified by Maternal education

P−for−interaction = 0.157
Difference in PDs = 0.07 (−0.03, 0.16)

Country	Trial	Primary or greater: LNS N	Control N	Control Prevalence	PD (95% CI)	Fixed W	Random W	Incomplete or no formal: LNS N	Control N	Control Prevalence	PD (95% CI)	Fixed W	Random W
Bangladesh	JiVitA−4 (35)												
Bangladesh	RDNS (36)	406	195	47.7	−0.14 (−0.22, −0.06)	0.43	0.36	144	77	55.8	−0.17 (−0.29, −0.05)	0.31	0.31
Bangladesh	WASH−B (37)	149	139	33.1	−0.24 (−0.33, −0.14)	0.32	0.32	63	39	25.6	−0.15 (−0.30, 0.01)	0.18	0.18
Burkina Faso	iLiNS−Zinc (38)												
Burkina Faso	PROMIS CS (39)												
Ghana	GHANA (40)												
Ghana	iLiNS−DYAD−G (41)												
Kenya	WASH−B (42)	143	117	77.8	−0.32 (−0.43, −0.20)	0.22	0.26	154	142	69.0	−0.18 (−0.29, −0.07)	0.39	0.39
Madagascar	MAHAY (43)	16	16	50.0	−0.19 (−0.48, 0.11)	0.03	0.06	67	35	62.9	−0.11 (−0.31, 0.09)	0.12	0.12
Malawi	iLiNS−DYAD−M (44)												
Malawi	iLiNS−DOSE (45)												
Mali	PROMIS CS (46)												
Zimbabwe	SHINE (HIV−) (47)												
Zimbabwe	SHINE (HIV+) (48)												
		**714**	**467**		**I² = 0.54, Tau² = 0.00**			**428**	**293**		**I² = 0.00, Tau² = 0.00**		
**Fixed**					**−0.21 (−0.26, −0.16)**						**−0.16 (−0.23, −0.09)**		
**Random**					**−0.22 (−0.30, −0.14)**						**−0.16 (−0.23, −0.09)**		

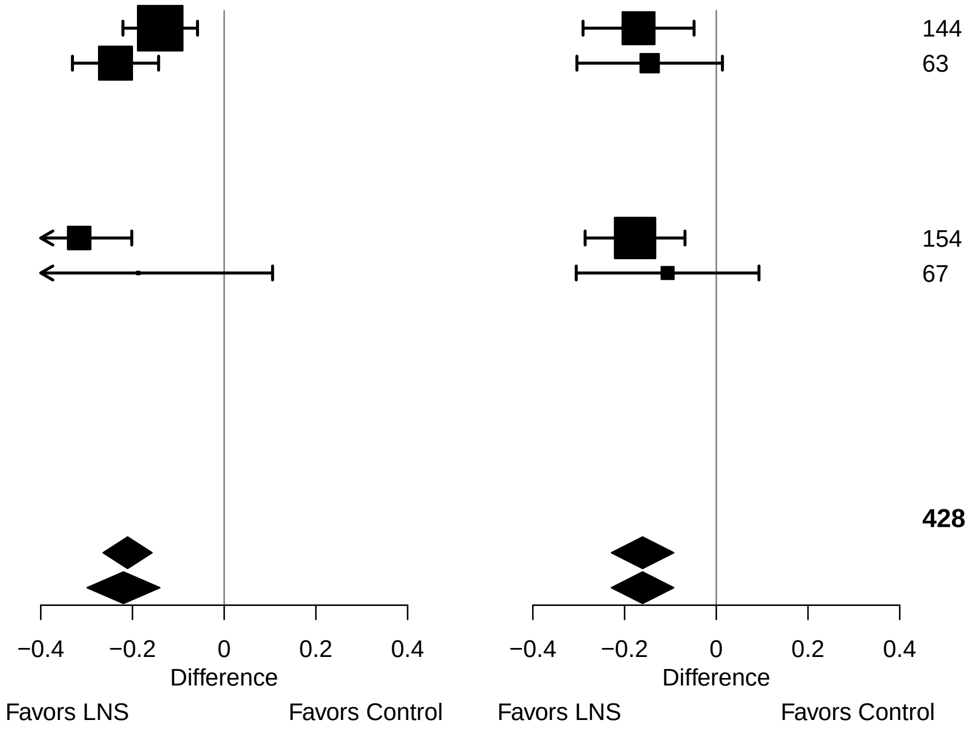

Supplemental figure 8M: Elevated soluble transferrin receptor prevalence difference

8M4: Stratified by Child sex

P−for−interaction = 0.322
Difference in PDs = 0.04 (−0.04, 0.11)

Country	Trial	Male: LNS N	Male: Control N	Male: Control Prevalence	Male: PD (95% CI)	Male: Fixed W	Male: Random W	Female: LNS N	Female: Control N	Female: Control Prevalence	Female: PD (95% CI)	Female: Fixed W	Female: Random W
Bangladesh	JiVitA−4 (35)												
Bangladesh	RDNS (36)	266	131	58.0	−0.17 (−0.28, −0.07)	0.29	0.29	281	141	42.6	−0.13 (−0.22, −0.03)	0.26	0.23
Bangladesh	WASH−B (37)	105	85	37.6	−0.26 (−0.39, −0.14)	0.22	0.22	107	93	25.8	−0.17 (−0.28, −0.07)	0.22	0.21
Burkina Faso	iLiNS−Zinc (38)	150	49	66.7	−0.32 (−0.53, −0.10)	0.07	0.07	165	47	55.8	−0.28 (−0.39, −0.16)	0.18	0.19
Burkina Faso	PROMIS CS (39)												
Ghana	GHANA (40)	52	37	73.0	−0.31 (−0.51, −0.10)	0.08	0.08	32	45	53.3	−0.28 (−0.50, −0.07)	0.05	0.07
Ghana	iLiNS−DYAD−G (41)												
Kenya	WASH−B (42)	146	125	73.6	−0.22 (−0.33, −0.11)	0.28	0.28	152	134	72.4	−0.26 (−0.36, −0.16)	0.24	0.22
Madagascar	MAHAY (43)	34	22	72.7	−0.17 (−0.40, 0.06)	0.06	0.06	49	29	48.3	−0.05 (−0.26, 0.15)	0.05	0.08
Malawi	iLiNS−DYAD−M (44)												
Malawi	iLiNS−DOSE (45)												
Mali	PROMIS CS (46)												
Zimbabwe	SHINE (HIV−) (47)												
Zimbabwe	SHINE (HIV+) (48)												
		**753**	**449**		**I^2 = 0.00, Tau^2 = 0.00**			**786**	**489**		**I^2 = 0.37, Tau^2 = 0.00**		
**Fixed**					**−0.23 (−0.28, −0.17)**						**−0.20 (−0.25, −0.15)**		
**Random**					**−0.23 (−0.28, −0.17)**						**−0.20 (−0.26, −0.14)**		

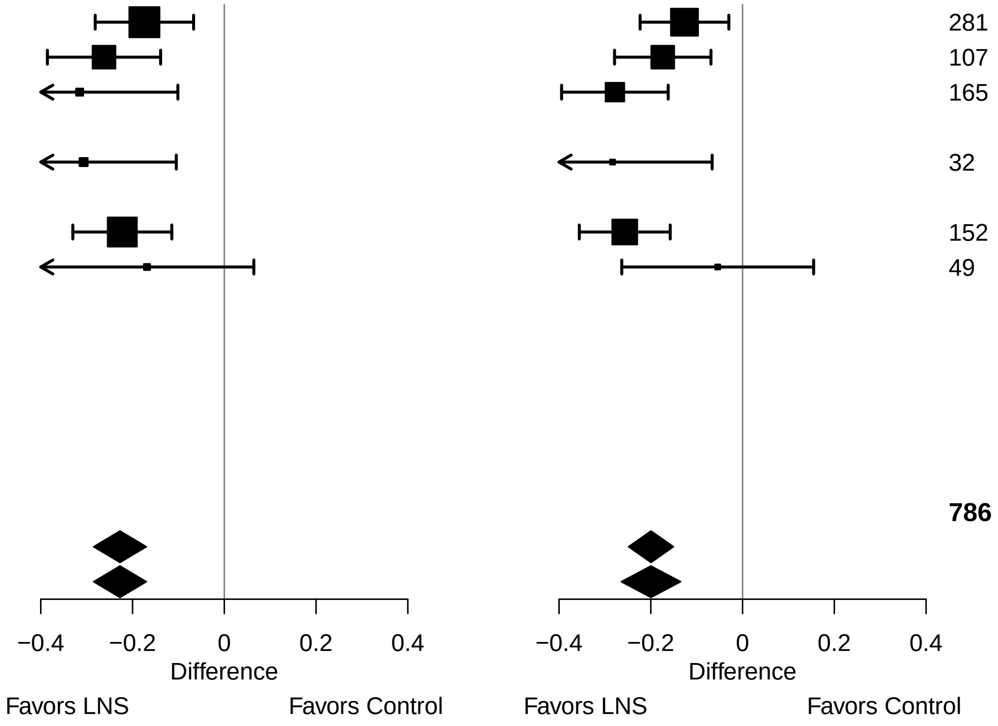

Supplemental figure 8M: Elevated soluble transferrin receptor prevalence difference

8M5: Stratified by Child birth order

P−for−interaction = 0.285
Difference in PDs = −0.05 (−0.13, 0.04)

		Later born						Firstborn					
Country	Trial	LNS N	Control N	Control Prevalence	PD (95% CI)	Fixed W	Random W	LNS N	Control N	Control Prevalence	PD (95% CI)	Fixed W	Random W
Bangladesh	JiVitA−4 (35)												
Bangladesh	RDNS (36)	340	171	50.9	−0.17 (−0.26, −0.08)	0.27	0.27	210	101	48.5	−0.12 (−0.22, −0.02)	0.36	0.24
Bangladesh	WASH−B (37)	124	119	30.3	−0.18 (−0.27, −0.10)	0.29	0.29	86	57	33.3	−0.28 (−0.42, −0.13)	0.18	0.21
Burkina Faso	iLiNS−Zinc (38)	249	71	54.7	−0.24 (−0.41, −0.07)	0.07	0.07	66	25	77.4	−0.46 (−0.58, −0.34)	0.26	0.23
Burkina Faso	PROMIS CS (39)												
Ghana	GHANA (40)	54	50	62.0	−0.32 (−0.51, −0.14)	0.06	0.06	30	29	62.1	−0.15 (−0.41, 0.10)	0.06	0.13
Ghana	iLiNS−DYAD−G (41)												
Kenya	WASH−B (42)	237	213	72.8	−0.22 (−0.31, −0.14)	0.30	0.30	60	46	73.9	−0.32 (−0.48, −0.16)	0.15	0.20
Madagascar	MAHAY (43)												
Malawi	iLiNS−DYAD−M (44)												
Malawi	iLiNS−DOSE (45)												
Mali	PROMIS CS (46)												
Zimbabwe	SHINE (HIV−) (47)												
Zimbabwe	SHINE (HIV+) (48)												
		**1004**	**624**		**I² = 0.00, Tau² = 0.00**			**452**	**258**		**I² = 0.79, Tau² = 0.01**		
**Fixed**					**−0.20 (−0.25, −0.16)**						**−0.27 (−0.33, −0.20)**		
**Random**					**−0.20 (−0.25, −0.16)**						**−0.27 (−0.40, −0.15)**		

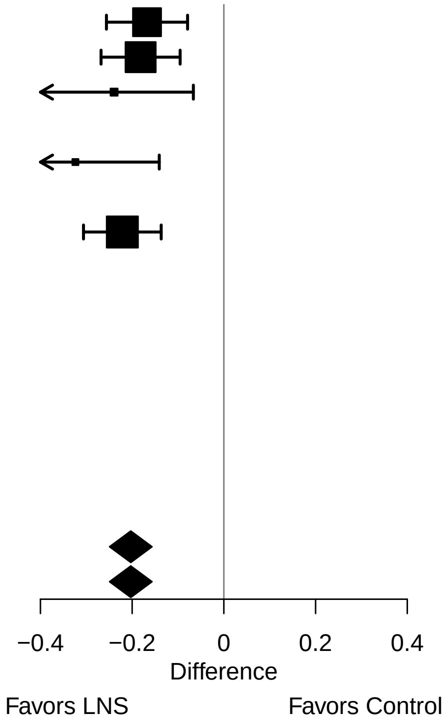

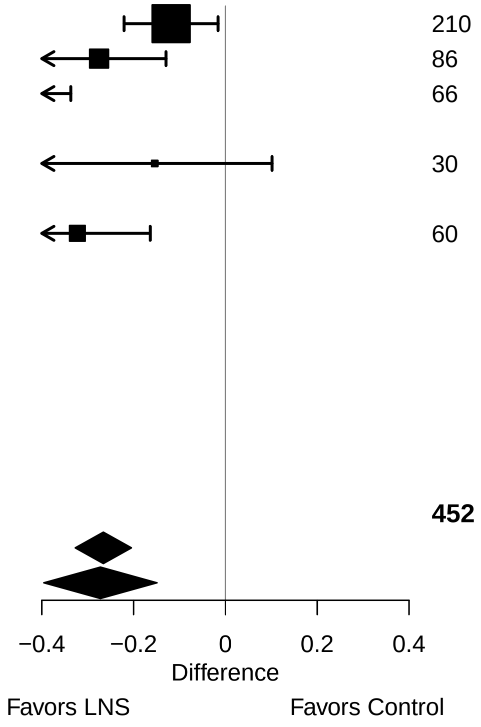

Supplemental figure 8M: Elevated soluble transferrin receptor prevalence difference

8M6: Stratified by Child baseline acute malnutrition (insufficient comparisons)

Supplemental figure 8M: Elevated soluble transferrin receptor prevalence difference

8M7: Stratified by Child baseline anemia (insufficient comparisons)

Supplemental figure 8M: Elevated soluble transferrin receptor prevalence difference

8M8: Stratified by Child high-dose vitamin A supplementation

P−for−interaction = 0.272
Difference in PDs = 0.08 (−0.06, 0.21)

Country	Trial	LNS N	Control N	Control Prevalence	Received VitA PD (95% CI)	Fixed W	Random W	LNS N	Control N	Control Prevalence	Not treated PD (95% CI)	Fixed W	Random W
Bangladesh	JiVitA−4 (35)												
Bangladesh	RDNS (36)	444	223	50.7	−0.16 (−0.24, −0.07)	0.77	0.52	105	49	46.9	−0.13 (−0.26, 0.01)	0.26	0.26
Bangladesh	WASH−B (37)												
Burkina Faso	iLiNS−Zinc (38)												
Burkina Faso	PROMIS CS (39)												
Ghana	GHANA (40)	40	53	66.0	−0.26 (−0.46, −0.06)	0.13	0.27	44	26	53.8	−0.22 (−0.46, 0.01)	0.08	0.08
Ghana	iLiNS−DYAD−G (41)												
Kenya	WASH−B (42)	26	31	71.0	−0.40 (−0.64, −0.17)	0.09	0.22	223	180	75.0	−0.21 (−0.29, −0.13)	0.66	0.66
Madagascar	MAHAY (43)												
Malawi	iLiNS−DYAD−M (44)												
Malawi	iLiNS−DOSE (45)												
Mali	PROMIS CS (46)												
Zimbabwe	SHINE (HIV−) (47)												
Zimbabwe	SHINE (HIV+) (48)												
		**510**	**307**		**I^2 = 0.53, Tau^2 = 0.01**			**372**	**255**		**I^2 = 0.00, Tau^2 = 0.00**		
**Fixed**					**−0.19 (−0.27, −0.12)**						**−0.19 (−0.26, −0.12)**		
**Random**					**−0.24 (−0.37, −0.10)**						**−0.19 (−0.26, −0.12)**		

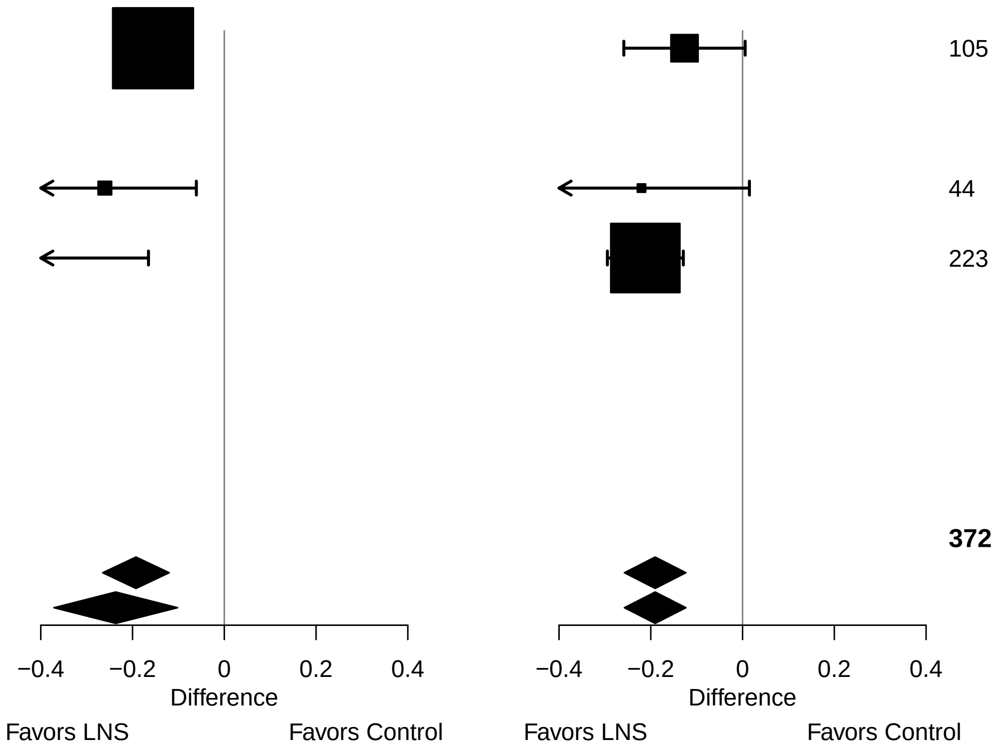

Supplemental figure 8M: Elevated soluble transferrin receptor prevalence difference

8M9: Stratified by Child inflammation

P−for−interaction = 0.599
Difference in PDs = −0.02 (−0.10, 0.06)

		**Not inflamed**						**High AGP or CRP**					
Country	Trial	**LNS** N	**Control** N	**Control** Prevalence	**PD** (95% CI)	**Fixed** W	**Random** W	**LNS** N	**Control** N	**Control** Prevalence	**PD** (95% CI)	**Fixed** W	**Random** W
Bangladesh	JiVitA−4 (35)												
Bangladesh	RDNS (36)	370	168	43.5	−0.10 (−0.20, −0.01)	0.28	0.21	180	104	60.6	−0.22 (−0.32, −0.11)	0.31	0.31
Bangladesh	WASH−B (37)	165	136	34.6	−0.25 (−0.34, −0.16)	0.31	0.21	47	42	21.4	−0.11 (−0.23, 0.01)	0.23	0.23
Burkina Faso	iLiNS−Zinc (38)	124	31	58.8	−0.34 (−0.53, −0.14)	0.07	0.12	191	65	62.5	−0.27 (−0.42, −0.12)	0.15	0.15
Burkina Faso	PROMIS CS (39)												
Ghana	GHANA (40)	70	66	62.1	−0.29 (−0.45, −0.13)	0.09	0.14	14	16	62.5	−0.12 (−0.49, 0.24)	0.03	0.03
Ghana	iLiNS−DYAD−G (41)												
Kenya	WASH−B (42)	157	135	68.9	−0.20 (−0.32, −0.09)	0.18	0.19	141	124	77.4	−0.28 (−0.39, −0.16)	0.25	0.25
Madagascar	MAHAY (43)	44	32	53.1	−0.01 (−0.19, 0.17)	0.07	0.13	39	19	68.4	−0.25 (−0.53, 0.03)	0.04	0.04
Malawi	iLiNS−DYAD−M (44)												
Malawi	iLiNS−DOSE (45)												
Mali	PROMIS CS (46)												
Zimbabwe	SHINE (HIV−) (47)												
Zimbabwe	SHINE (HIV+) (48)												
		**930**	**568**		**I² = 0.61, Tau² = 0.01**			**612**	**370**		**I² = 0.00, Tau² = 0.00**		
**Fixed**					**−0.19 (−0.24, −0.14)**						**−0.21 (−0.27, −0.16)**		
**Random**					**−0.20 (−0.29, −0.11)**						**−0.21 (−0.27, −0.16)**		

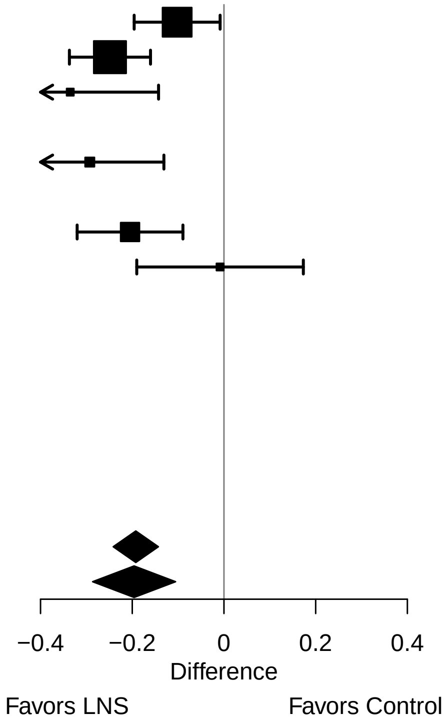

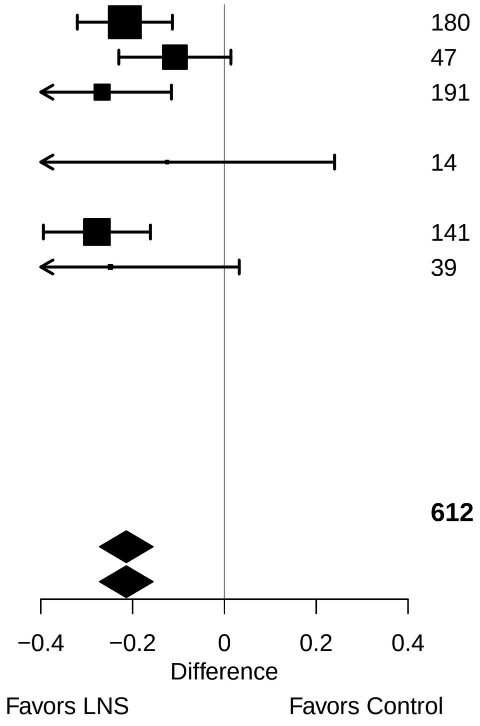

Supplemental figure 8N: Geometric mean ratio of zinc protoporphyrin concentration

8N1: Stratified by Maternal BMI

P−for−interaction = 0.792
Ratio of GMRs = 0.98 (0.85, 1.13)

		At least 20 kg/m²						Less than 20 kg/m²					
Country	Trial	LNS N	Control N	Control Median	GMR (95% CI)	Fixed W	Random W	LNS N	Control N	Control Median	GMR (95% CI)	Fixed W	Random W
Bangladesh	JiVitA−4 (35)												
Bangladesh	RDNS (36)												
Bangladesh	WASH−B (37)												
Burkina Faso	iLiNS−Zinc (38)	208	55	123.3	0.63 (0.51, 0.77)	0.14	0.21	104	40	111.5	0.65 (0.52, 0.82)	0.23	0.26
Burkina Faso	PROMIS CS (39)												
Ghana	GHANA (40)												
Ghana	iLiNS−DYAD−G (41)	85	174	47.0	0.88 (0.76, 1.01)	0.30	0.27	11	25	54.5	0.72 (0.50, 1.05)	0.09	0.12
Kenya	WASH−B (42)												
Madagascar	MAHAY (43)												
Malawi	iLiNS−DYAD−M (44)	111	222	56.0	0.86 (0.75, 0.99)	0.32	0.27	79	151	50.8	0.88 (0.75, 1.02)	0.53	0.43
Malawi	iLiNS−DOSE (45)	127	65	67.0	0.78 (0.67, 0.91)	0.24	0.25	53	22	55.8	0.75 (0.57, 0.99)	0.15	0.19
Mali	PROMIS CS (46)												
Zimbabwe	SHINE (HIV−) (47)												
Zimbabwe	SHINE (HIV+) (48)												
		**531**	**516**		**I² = 0.64, Tau² = 0.02**			**247**	**238**		**I² = 0.38, Tau² = 0.01**		
**Fixed**					**0.81 (0.75, 0.87)**						**0.79 (0.71, 0.88)**		
**Random**					**0.79 (0.68, 0.91)**						**0.77 (0.67, 0.88)**		

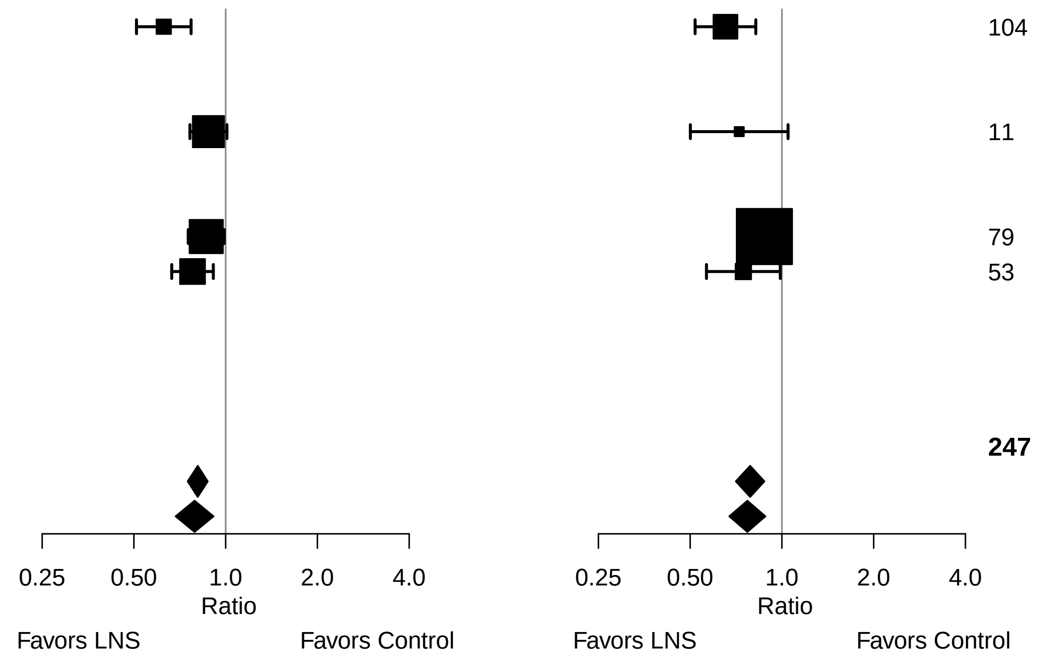

Supplemental figure 8N: Geometric mean ratio of zinc protoporphyrin concentration

8N2: Stratified by Maternal age

P−for−interaction = 0.998
Ratio of GMRs = 1.00 (0.88, 1.14)

		At least 25 y						Less than 25 y					
Country	Trial	LNS N	Control N	Control Median	GMR (95% CI)	Fixed W	Random W	LNS N	Control N	Control Median	GMR (95% CI)	Fixed W	Random W
Bangladesh	JiVitA−4 (35)												
Bangladesh	RDNS (36)												
Bangladesh	WASH−B (37)												
Burkina Faso	iLiNS−Zinc (38)	175	50	121.9	0.65 (0.53, 0.79)	0.17	0.21	137	45	122.6	0.65 (0.53, 0.80)	0.20	0.24
Burkina Faso	PROMIS CS (39)												
Ghana	GHANA (40)												
Ghana	iLiNS−DYAD−G (41)	65	128	45.8	0.88 (0.77, 1.02)	0.32	0.28	35	74	52.1	0.84 (0.65, 1.07)	0.15	0.20
Kenya	WASH−B (42)												
Madagascar	MAHAY (43)												
Malawi	iLiNS−DYAD−M (44)	88	192	50.4	0.84 (0.73, 0.97)	0.32	0.28	102	183	57.4	0.89 (0.77, 1.02)	0.45	0.34
Malawi	iLiNS−DOSE (45)	95	48	65.4	0.79 (0.66, 0.95)	0.19	0.23	83	39	69.1	0.75 (0.60, 0.93)	0.20	0.23
Mali	PROMIS CS (46)												
Zimbabwe	SHINE (HIV−) (47)												
Zimbabwe	SHINE (HIV+) (48)												
		**423**	**418**		**I² = 0.55, Tau² = 0.01**			**357**	**341**		**I² = 0.53, Tau² = 0.01**		
**Fixed**					**0.81 (0.74, 0.88)**						**0.80 (0.73, 0.88)**		
**Random**					**0.79 (0.70, 0.90)**						**0.78 (0.68, 0.90)**		

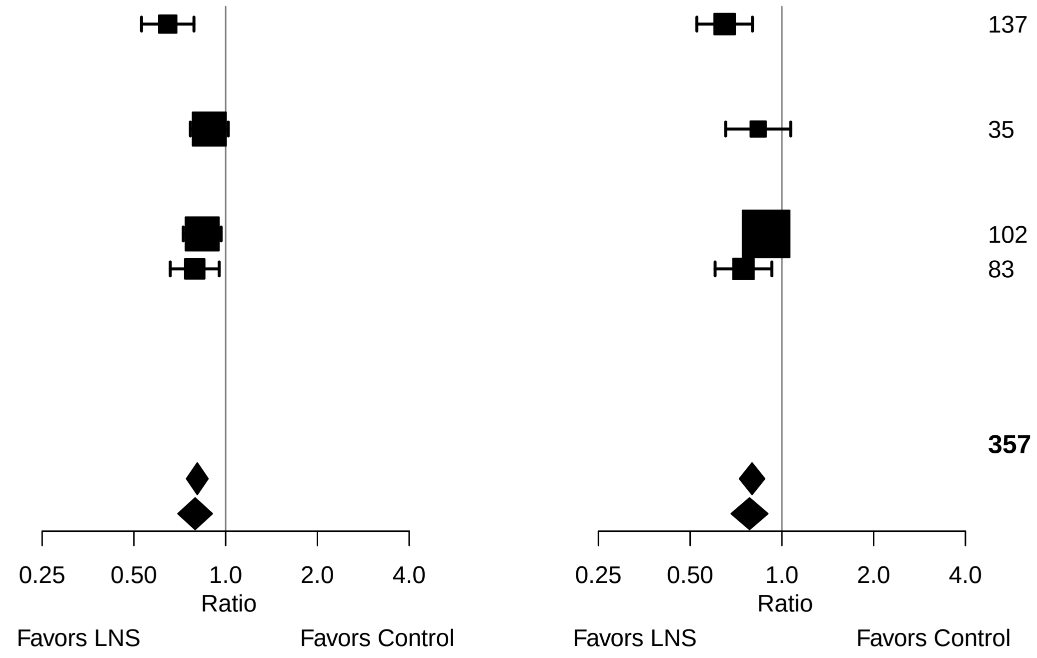

Supplemental figure 8N: Geometric mean ratio of zinc protoporphyrin concentration

8N3: Stratified by Maternal education

P−for−interaction = 0.117
Ratio of GMRs = 1.15 (0.97, 1.37)

		Primary or greater						Incomplete or no formal					
Country	Trial	LNS N	Control N	Control Median	GMR (95% CI)	Fixed W	Random W	LNS N	Control N	Control Median	GMR (95% CI)	Fixed W	Random W
Bangladesh	JiVitA−4 (35)												
Bangladesh	RDNS (36)												
Bangladesh	WASH−B (37)												
Burkina Faso	iLiNS−Zinc (38)												
Burkina Faso	PROMIS CS (39)												
Ghana	GHANA (40)												
Ghana	iLiNS−DYAD−G (41)	83	159	48.4	0.85 (0.74, 0.98)	0.66	0.56	17	43	43.5	0.92 (0.68, 1.25)	0.08	0.08
Kenya	WASH−B (42)												
Madagascar	MAHAY (43)												
Malawi	iLiNS−DYAD−M (44)	31	53	61.6	0.79 (0.58, 1.07)	0.14	0.19	158	320	53.1	0.88 (0.79, 0.98)	0.66	0.66
Malawi	iLiNS−DOSE (45)	48	26	76.1	0.66 (0.51, 0.86)	0.20	0.25	130	61	65.8	0.83 (0.70, 0.98)	0.27	0.27
Mali	PROMIS CS (46)												
Zimbabwe	SHINE (HIV−) (47)												
Zimbabwe	SHINE (HIV+) (48)												
		**162**	**238**		**I² = 0.30, Tau² = 0.00**			**305**	**424**		**I² = 0.00, Tau² = 0.00**		
**Fixed**					**0.80 (0.71, 0.90)**						**0.87 (0.80, 0.95)**		
**Random**					**0.79 (0.68, 0.91)**						**0.87 (0.80, 0.95)**		

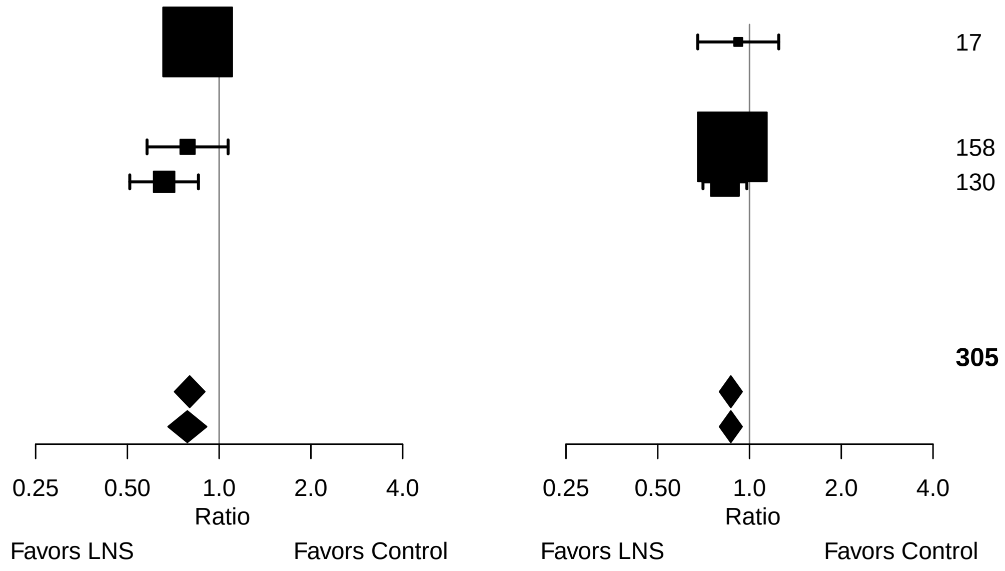

Supplemental figure 8N: Geometric mean ratio of zinc protoporphyrin concentration

8N4: Stratified by Child sex

P−for−interaction = 0.040
Ratio of GMRs = 1.13 (1.01, 1.27)

Country	Trial	Male LNS N	Male Control N	Male Control Median	Male GMR (95% CI)	Male Fixed W	Male Random W	Female LNS N	Female Control N	Female Control Median	Female GMR (95% CI)	Female Fixed W	Female Random W
Bangladesh	JiVitA−4 (35)												
Bangladesh	RDNS (36)												
Bangladesh	WASH−B (37)												
Burkina Faso	iLiNS−Zinc (38)	149	49	131.3	0.58 (0.48, 0.70)	0.19	0.24	163	46	99.3	0.72 (0.63, 0.83)	0.34	0.30
Burkina Faso	PROMIS CS (39)												
Ghana	GHANA (40)												
Ghana	iLiNS−DYAD−G (41)	63	95	49.1	0.89 (0.76, 1.04)	0.27	0.26	36	106	46.1	0.85 (0.69, 1.04)	0.16	0.20
Kenya	WASH−B (42)												
Madagascar	MAHAY (43)												
Malawi	iLiNS−DYAD−M (44)	95	183	57.9	0.81 (0.70, 0.93)	0.35	0.27	95	192	49.0	0.93 (0.81, 1.07)	0.34	0.30
Malawi	iLiNS−DOSE (45)	86	46	66.9	0.74 (0.61, 0.89)	0.19	0.24	95	41	65.2	0.81 (0.66, 1.00)	0.16	0.20
Mali	PROMIS CS (46)												
Zimbabwe	SHINE (HIV−) (47)												
Zimbabwe	SHINE (HIV+) (48)												
		**393**	**373**		**I² = 0.75, Tau² = 0.03**			**389**	**385**		**I² = 0.52, Tau² = 0.01**		
**Fixed**					**0.76 (0.70, 0.83)**						**0.82 (0.76, 0.89)**		
**Random**					**0.75 (0.63, 0.89)**						**0.82 (0.74, 0.92)**		

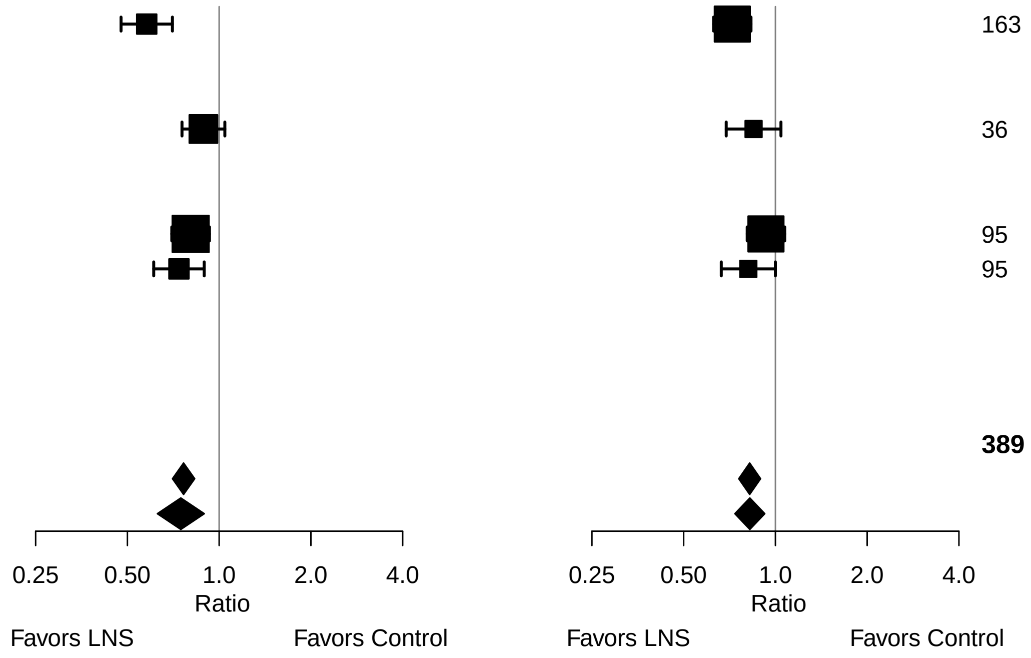

Supplemental figure 8N: Geometric mean ratio of zinc protoporphyrin concentration

8N5: Stratified by Child birth order

P−for−interaction = 0.311
Ratio of GMRs = 0.93 (0.80, 1.07)

Country	Trial	LNS N	Control N	Control Median	Later born GMR (95% CI)	Fixed W	Random W	LNS N	Control N	Control Median	Firstborn GMR (95% CI)	Fixed W	Random W
Bangladesh	JiVitA−4 (35)												
Bangladesh	RDNS (36)												
Bangladesh	WASH−B (37)												
Burkina Faso	iLiNS−Zinc (38)	246	71	116.3	0.68 (0.58, 0.79)	0.22	0.24	66	24	157.4	0.56 (0.43, 0.72)	0.24	0.25
Burkina Faso	PROMIS CS (39)												
Ghana	GHANA (40)												
Ghana	iLiNS−DYAD−G (41)	70	141	45.8	0.92 (0.79, 1.07)	0.22	0.24	30	61	51.1	0.75 (0.59, 0.94)	0.30	0.27
Kenya	WASH−B (42)												
Madagascar	MAHAY (43)												
Malawi	iLiNS−DYAD−M (44)	152	299	52.8	0.86 (0.77, 0.97)	0.39	0.29	38	75	55.9	0.89 (0.72, 1.10)	0.34	0.28
Malawi	iLiNS−DOSE (45)	119	59	65.5	0.77 (0.65, 0.92)	0.18	0.23	33	19	58.4	0.90 (0.63, 1.29)	0.12	0.19
Mali	PROMIS CS (46)												
Zimbabwe	SHINE (HIV−) (47)												
Zimbabwe	SHINE (HIV+) (48)												
		**587**	**570**		**I² = 0.67, Tau² = 0.01**			**167**	**179**		**I² = 0.65, Tau² = 0.03**		
**Fixed**					**0.82 (0.76, 0.88)**						**0.76 (0.67, 0.86)**		
**Random**					**0.81 (0.71, 0.92)**						**0.76 (0.61, 0.94)**		

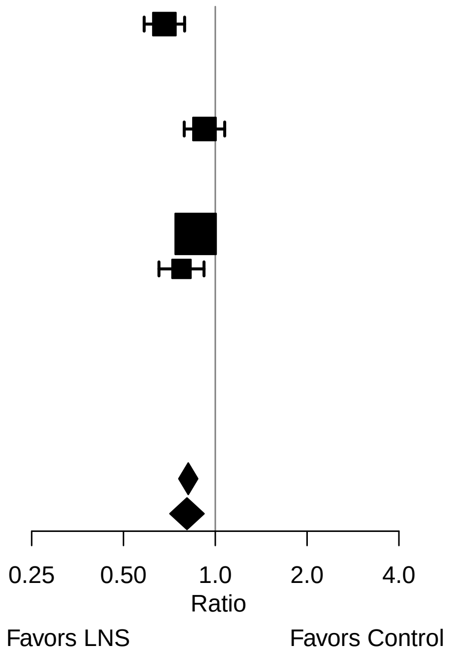

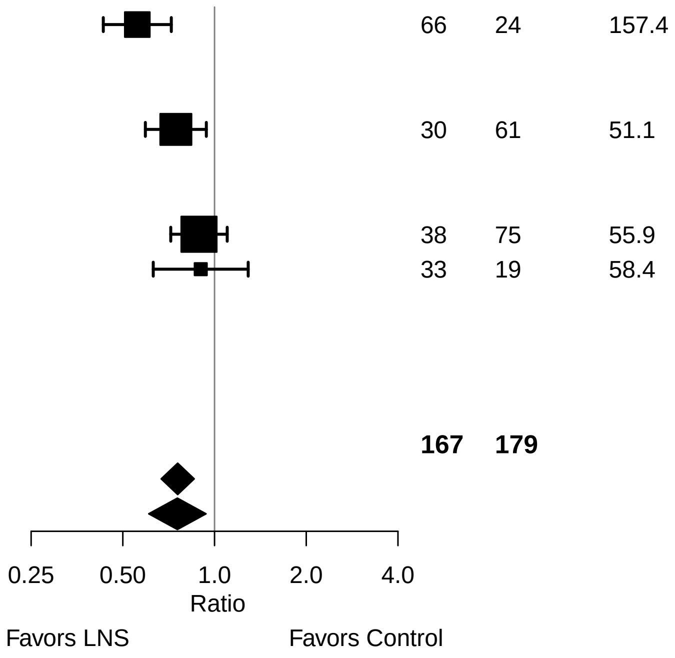

Supplemental figure 8N: Geometric mean ratio of zinc protoporphyrin concentration

8N6: Stratified by Child baseline acute malnutrition

P−for−interaction = 0.817
Ratio of GMRs = 0.98 (0.82, 1.16)

		No						Yes					
Country	Trial	LNS N	Control N	Control Median	GMR (95% CI)	Fixed W	Random W	LNS N	Control N	Control Median	GMR (95% CI)	Fixed W	Random W
Bangladesh	JiVitA−4 (35)												
Bangladesh	RDNS (36)												
Bangladesh	WASH−B (37)												
Burkina Faso	iLiNS−Zinc (38)	227	72	121.6	0.64 (0.55, 0.73)	0.26	0.32	85	23	122.9	0.66 (0.54, 0.81)	0.60	0.60
Burkina Faso	PROMIS CS (39)												
Ghana	GHANA (40)												
Ghana	iLiNS−DYAD−G (41)	94	187	48.1	0.88 (0.77, 1.01)	0.29	0.33	6	15	45.8	0.62 (0.43, 0.89)	0.20	0.20
Kenya	WASH−B (42)												
Madagascar	MAHAY (43)												
Malawi	iLiNS−DYAD−M (44)	165	330	53.2	0.87 (0.79, 0.97)	0.45	0.35	11	33	59.2	0.83 (0.58, 1.20)	0.20	0.20
Malawi	iLiNS−DOSE (45)												
Mali	PROMIS CS (46)												
Zimbabwe	SHINE (HIV−) (47)												
Zimbabwe	SHINE (HIV+) (48)												
		**486**	**589**		**I^2 = 0.86, Tau^2 = 0.03**			**102**	**71**		**I^2 = 0.00, Tau^2 = 0.00**		
**Fixed**					**0.81 (0.75, 0.87)**						**0.68 (0.58, 0.80)**		
**Random**					**0.79 (0.64, 0.97)**						**0.68 (0.58, 0.80)**		

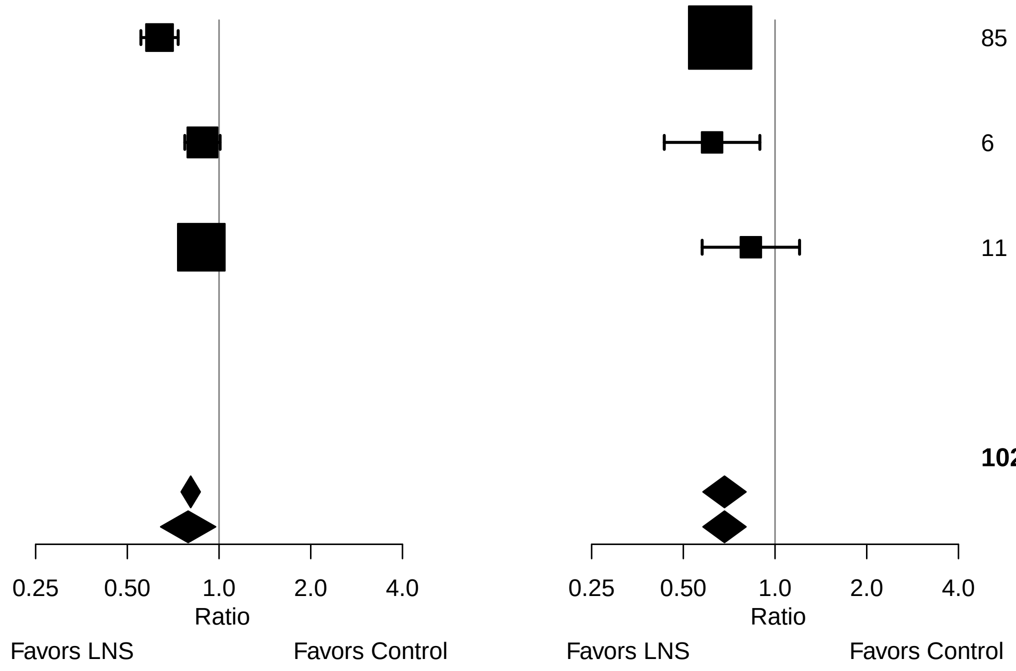

Supplemental figure 8N: Geometric mean ratio of zinc protoporphyrin concentration

8N7: Stratified by Child baseline anemia

P−for−interaction = 0.243
Ratio of GMRs = 1.09 (0.95, 1.25)

		Not anemic						Anemic					
Country	Trial	LNS N	Control N	Control Median	GMR (95% CI)	Fixed W	Random W	LNS N	Control N	Control Median	GMR (95% CI)	Fixed W	Random W
Bangladesh	JiVitA−4 (35)												
Bangladesh	RDNS (36)												
Bangladesh	WASH−B (37)												
Burkina Faso	iLiNS−Zinc (38)	20	7	83.9	0.63 (0.41, 0.98)	0.05	0.06	292	88	122.6	0.65 (0.57, 0.73)	0.35	0.28
Burkina Faso	PROMIS CS (39)												
Ghana	GHANA (40)												
Ghana	iLiNS−DYAD−G (41)	75	132	45.7	0.82 (0.71, 0.95)	0.43	0.41	25	70	52.3	1.03 (0.80, 1.32)	0.09	0.20
Kenya	WASH−B (42)												
Madagascar	MAHAY (43)												
Malawi	iLiNS−DYAD−M (44)	59	121	44.5	0.88 (0.74, 1.04)	0.32	0.32	121	242	58.4	0.87 (0.77, 0.98)	0.39	0.28
Malawi	iLiNS−DOSE (45)	66	36	57.3	0.72 (0.58, 0.89)	0.20	0.22	115	50	70.0	0.82 (0.68, 0.99)	0.17	0.24
Mali	PROMIS CS (46)												
Zimbabwe	SHINE (HIV−) (47)												
Zimbabwe	SHINE (HIV+) (48)												
		**220**	**296**		**I² = 0.10, Tau² = 0.00**			**553**	**450**		**I² = 0.82, Tau² = 0.03**		
**Fixed**					**0.81 (0.73, 0.89)**						**0.79 (0.73, 0.85)**		
**Random**					**0.81 (0.72, 0.89)**						**0.82 (0.68, 0.99)**		

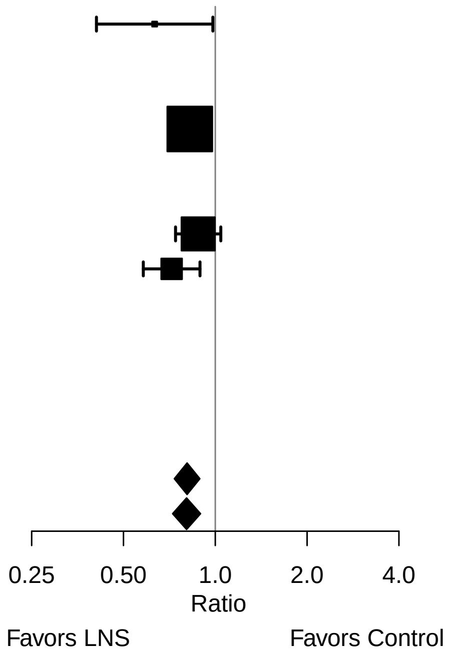

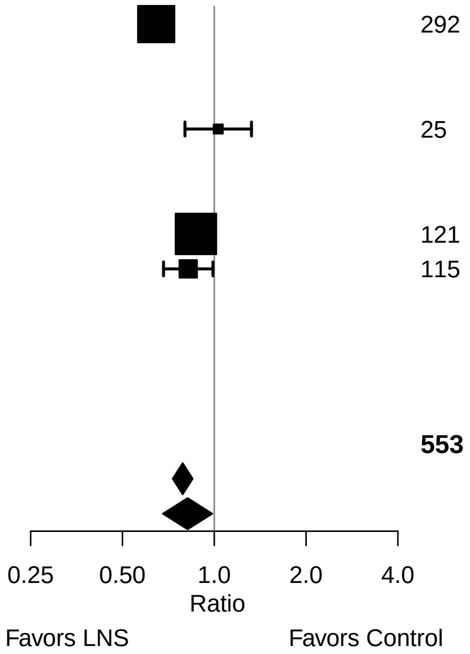

Supplemental figure 8N: Geometric mean ratio of zinc protoporphyrin concentration

8N8: Stratified by Child high-dose vitamin A supplementation (insufficient comparisons)

Supplemental figure 8N: Geometric mean ratio of zinc protoporphyrin concentration

8N9: Stratified by Child inflammation

P−for−interaction = 0.777
Ratio of GMRs = 0.98 (0.86, 1.11)

Country	Trial	Not inflamed: LNS N	Control N	Control Median	GMR (95% CI)	Fixed W	Random W	High AGP or CRP: LNS N	Control N	Control Median	GMR (95% CI)	Fixed W	Random W
Bangladesh	JiVitA−4 (35)												
Bangladesh	RDNS (36)												
Bangladesh	WASH−B (37)												
Burkina Faso	iLiNS−Zinc (38)	123	30	126.7	0.61 (0.48, 0.76)	0.20	0.24	189	65	113.4	0.67 (0.57, 0.78)	0.23	0.24
Burkina Faso	PROMIS CS (39)												
Ghana	GHANA (40)												
Ghana	iLiNS−DYAD−G (41)	45	120	45.3	0.87 (0.72, 1.05)	0.30	0.27	55	82	51.6	0.85 (0.72, 1.02)	0.18	0.22
Kenya	WASH−B (42)												
Madagascar	MAHAY (43)												
Malawi	iLiNS−DYAD−M (44)	53	119	48.8	0.94 (0.78, 1.12)	0.31	0.27	137	256	55.8	0.84 (0.75, 0.95)	0.39	0.31
Malawi	iLiNS−DOSE (45)	62	26	60.3	0.79 (0.62, 1.01)	0.18	0.23	119	61	67.0	0.77 (0.65, 0.91)	0.20	0.23
Mali	PROMIS CS (46)												
Zimbabwe	SHINE (HIV−) (47)												
Zimbabwe	SHINE (HIV+) (48)												
		**283**	**295**		**I² = 0.67, Tau² = 0.02**			**500**	**464**		**I² = 0.51, Tau² = 0.01**		
**Fixed**					**0.81 (0.73, 0.90)**						**0.79 (0.73, 0.85)**		
**Random**					**0.80 (0.66, 0.96)**						**0.78 (0.70, 0.87)**		

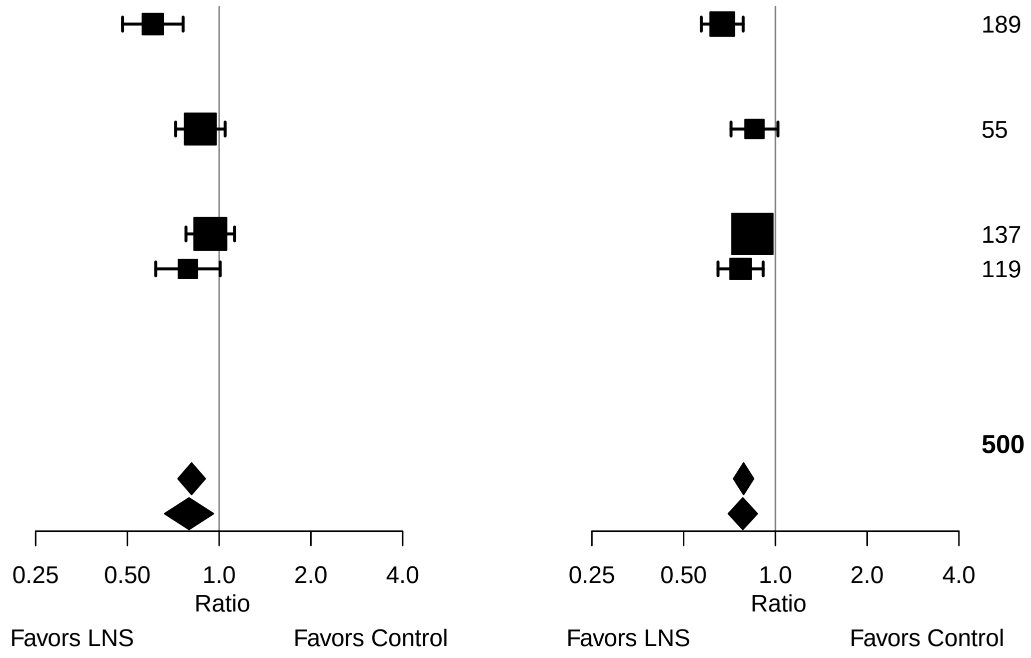

Supplemental figure 8O: Elevated zinc protoporphyrin prevalence ratio

8O1: Stratified by Maternal BMI

P−for−interaction = 0.942
Ratio of PRs = 1.01 (0.78, 1.30)

		At least 20 kg/m²						Less than 20 kg/m²					
Country	Trial	LNS N	Control N	Control Prevalence	PR (95% CI)	Fixed W	Random W	LNS N	Control N	Control Prevalence	PR (95% CI)	Fixed W	Random W
Bangladesh	JiVitA−4 (35)												
Bangladesh	RDNS (36)												
Bangladesh	WASH−B (37)												
Burkina Faso	iLiNS−Zinc (38)	208	55	73.8	0.73 (0.57, 0.94)	0.52	0.52	104	40	77.3	0.69 (0.56, 0.86)	0.74	0.74
Burkina Faso	PROMIS CS (39)												
Ghana	GHANA (40)												
Ghana	iLiNS−DYAD−G (41)												
Kenya	WASH−B (42)												
Madagascar	MAHAY (43)												
Malawi	iLiNS−DYAD−M (44)	111	222	36.5	0.64 (0.44, 0.94)	0.23	0.23	79	151	29.1	0.83 (0.52, 1.31)	0.16	0.16
Malawi	iLiNS−DOSE (45)	127	65	46.2	0.60 (0.41, 0.86)	0.25	0.25	53	22	40.9	0.62 (0.35, 1.10)	0.10	0.10
Mali	PROMIS CS (46)												
Zimbabwe	SHINE (HIV−) (47)												
Zimbabwe	SHINE (HIV+) (48)												
		**446**	**342**		**I² = 0.00, Tau² = 0.00**			**236**	**213**		**I² = 0.00, Tau² = 0.00**		
**Fixed**					**0.67 (0.56, 0.81)**						**0.70 (0.58, 0.85)**		
**Random**					**0.67 (0.56, 0.81)**						**0.70 (0.58, 0.85)**		

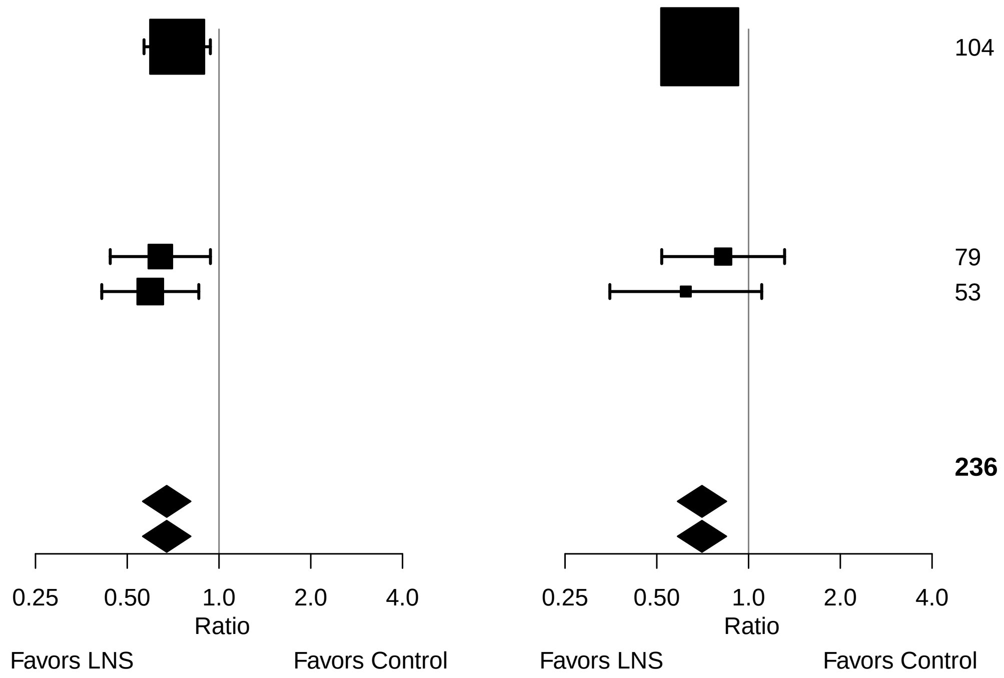

Supplemental figure 8O: Elevated zinc protoporphyrin prevalence ratio

8O2: Stratified by Maternal age

P−for−interaction = 0.660
Ratio of PRs = 1.07 (0.80, 1.43)

		At least 25 y						Less than 25 y					
Country	Trial	LNS N	Control N	Control Prevalence	PR (95% CI)	Fixed W	Random W	LNS N	Control N	Control Prevalence	PR (95% CI)	Fixed W	Random W
Bangladesh	JiVitA−4 (35)												
Bangladesh	RDNS (36)												
Bangladesh	WASH−B (37)												
Burkina Faso	iLiNS−Zinc (38)	175	50	74.1	0.66 (0.49, 0.89)	0.49	0.49	137	45	76.5	0.78 (0.58, 1.03)	0.45	0.45
Burkina Faso	PROMIS CS (39)												
Ghana	GHANA (40)												
Ghana	iLiNS−DYAD−G (41)	65	128	16.4	0.75 (0.35, 1.60)	0.07	0.07	35	74	37.8	0.76 (0.41, 1.38)	0.10	0.10
Kenya	WASH−B (42)												
Madagascar	MAHAY (43)												
Malawi	iLiNS−DYAD−M (44)	88	192	28.1	0.77 (0.49, 1.21)	0.20	0.20	102	183	39.3	0.65 (0.44, 0.94)	0.26	0.26
Malawi	iLiNS−DOSE (45)	95	48	41.7	0.56 (0.37, 0.85)	0.24	0.24	83	39	48.7	0.65 (0.42, 1.01)	0.19	0.19
Mali	PROMIS CS (46)												
Zimbabwe	SHINE (HIV−) (47)												
Zimbabwe	SHINE (HIV+) (48)												
		**423**	**418**		**I² = 0.00, Tau² = 0.00**			**357**	**341**		**I² = 0.00, Tau² = 0.00**		
**Fixed**					**0.66 (0.54, 0.81)**						**0.72 (0.59, 0.87)**		
**Random**					**0.66 (0.54, 0.81)**						**0.72 (0.59, 0.87)**		

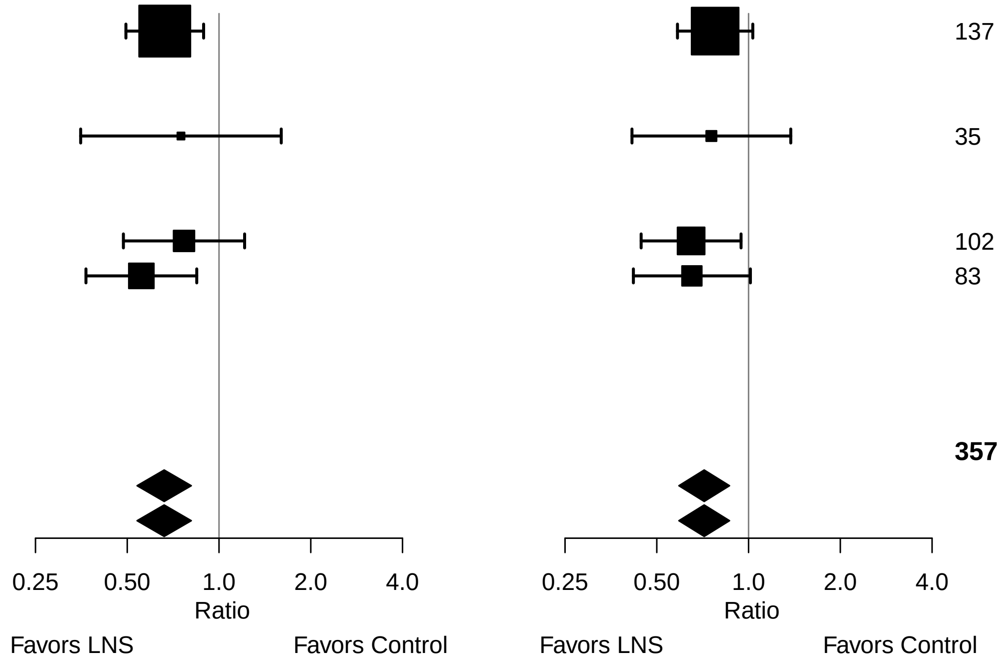

Supplemental figure 8O: Elevated zinc protoporphyrin prevalence ratio

8O3: Stratified by Maternal education

P−for−interaction = 0.114
Ratio of PRs = 1.42 (0.92, 2.20)

Country	Trial	LNS N	Control N	Control Prevalence	Primary or greater PR (95% CI)	Fixed W	Random W	LNS N	Control N	Control Prevalence	Incomplete or no formal PR (95% CI)	Fixed W	Random W
Bangladesh	JiVitA−4 (35)												
Bangladesh	RDNS (36)												
Bangladesh	WASH−B (37)												
Burkina Faso	iLiNS−Zinc (38)												
Burkina Faso	PROMIS CS (39)												
Ghana	GHANA (40)												
Ghana	iLiNS−DYAD−G (41)	83	159	25.2	0.62 (0.35, 1.10)	0.33	0.33	17	43	20.9	1.41 (0.55, 3.59)	0.07	0.07
Kenya	WASH−B (42)												
Madagascar	MAHAY (43)												
Malawi	iLiNS−DYAD−M (44)	31	53	45.3	0.64 (0.34, 1.20)	0.27	0.27	158	320	31.6	0.70 (0.50, 0.98)	0.52	0.52
Malawi	iLiNS−DOSE (45)	48	26	53.8	0.47 (0.28, 0.79)	0.40	0.40	130	61	41.0	0.70 (0.48, 1.02)	0.41	0.41
Mali	PROMIS CS (46)												
Zimbabwe	SHINE (HIV−) (47)												
Zimbabwe	SHINE (HIV+) (48)												
		**162**	**238**		**I² = 0.00, Tau² = 0.00**			**305**	**424**		**I² = 0.00, Tau² = 0.00**		
**Fixed**					**0.56 (0.41, 0.78)**						**0.74 (0.58, 0.94)**		
**Random**					**0.56 (0.41, 0.78)**						**0.74 (0.58, 0.94)**		

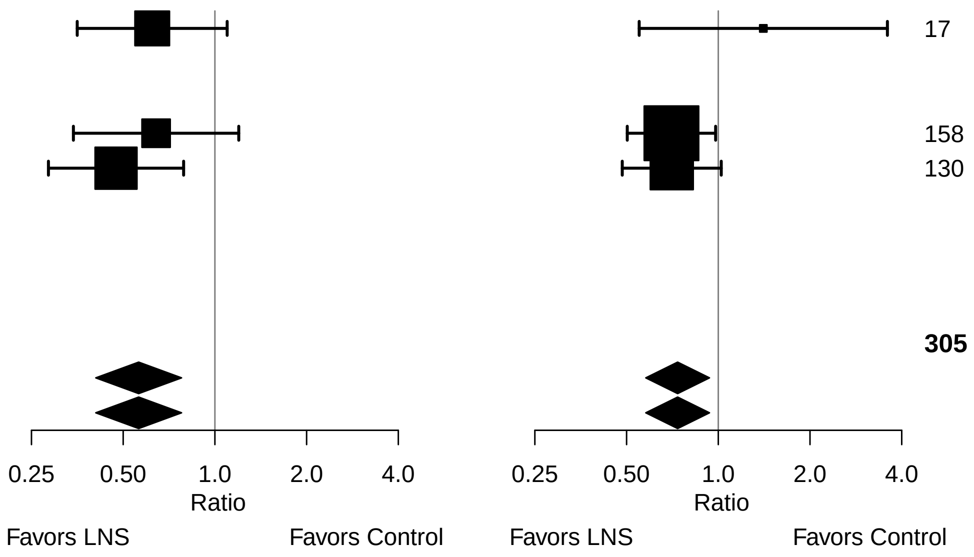

Supplemental figure 8O: Elevated zinc protoporphyrin prevalence ratio

8O4: Stratified by Child sex

P−for−interaction = 0.187
Ratio of PRs = 1.13 (0.94, 1.36)

Country	Trial	Male LNS N	Male Control N	Male Control Prevalence	Male PR (95% CI)	Male Fixed W	Male Random W	Female LNS N	Female Control N	Female Control Prevalence	Female PR (95% CI)	Female Fixed W	Female Random W
Bangladesh	JiVitA−4 (35)												
Bangladesh	RDNS (36)												
Bangladesh	WASH−B (37)												
Burkina Faso	iLiNS−Zinc (38)	149	49	81.5	0.68 (0.53, 0.86)	0.56	0.56	163	46	68.6	0.76 (0.64, 0.91)	0.73	0.73
Burkina Faso	PROMIS CS (39)												
Ghana	GHANA (40)												
Ghana	iLiNS−DYAD−G (41)	63	95	26.3	0.72 (0.39, 1.33)	0.09	0.09	36	106	21.7	0.77 (0.34, 1.74)	0.03	0.03
Kenya	WASH−B (42)												
Madagascar	MAHAY (43)												
Malawi	iLiNS−DYAD−M (44)	95	183	37.2	0.62 (0.41, 0.94)	0.19	0.19	95	192	30.2	0.80 (0.53, 1.21)	0.13	0.13
Malawi	iLiNS−DOSE (45)	86	46	43.5	0.61 (0.40, 0.95)	0.17	0.17	95	41	46.3	0.67 (0.42, 1.05)	0.11	0.11
Mali	PROMIS CS (46)												
Zimbabwe	SHINE (HIV−) (47)												
Zimbabwe	SHINE (HIV+) (48)												
		**393**	**373**		**I² = 0.00, Tau² = 0.00**			**389**	**385**		**I² = 0.00, Tau² = 0.00**		
**Fixed**					**0.66 (0.55, 0.79)**						**0.76 (0.65, 0.88)**		
**Random**					**0.66 (0.55, 0.79)**						**0.76 (0.65, 0.88)**		

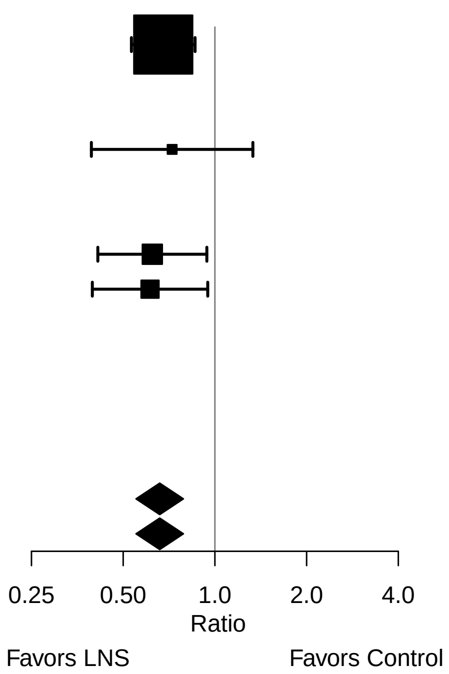

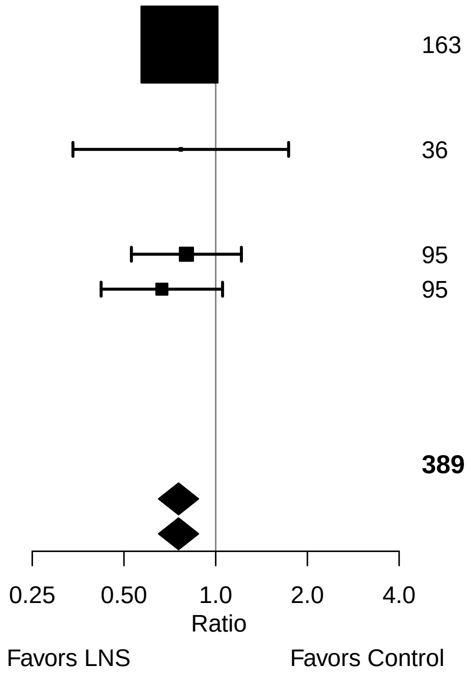

Supplemental figure 8O: Elevated zinc protoporphyrin prevalence ratio

8O5: Stratified by Child birth order

P−for−interaction = 0.123
Ratio of PRs = 1.21 (0.95, 1.53)

Country	Trial	Later born LNS N	Later born Control N	Later born Control Prevalence	Later born PR (95% CI)	Later born Fixed W	Later born Random W	Firstborn LNS N	Firstborn Control N	Firstborn Control Prevalence	Firstborn PR (95% CI)	Firstborn Fixed W	Firstborn Random W
Bangladesh	JiVitA−4 (35)												
Bangladesh	RDNS (36)												
Bangladesh	WASH−B (37)												
Burkina Faso	iLiNS−Zinc (38)	246	71	74.7	0.69 (0.56, 0.84)	0.59	0.59	66	24	76.7	0.86 (0.67, 1.11)	0.79	0.79
Burkina Faso	PROMIS CS (39)												
Ghana	GHANA (40)												
Ghana	iLiNS−DYAD−G (41)												
Kenya	WASH−B (42)												
Madagascar	MAHAY (43)												
Malawi	iLiNS−DYAD−M (44)	152	299	33.4	0.73 (0.53, 1.00)	0.24	0.24	38	75	34.7	0.61 (0.30, 1.21)	0.11	0.11
Malawi	iLiNS−DOSE (45)	119	59	45.8	0.55 (0.37, 0.79)	0.17	0.17	33	19	36.8	0.99 (0.49, 2.02)	0.10	0.10
Mali	PROMIS CS (46)												
Zimbabwe	SHINE (HIV−) (47)												
Zimbabwe	SHINE (HIV+) (48)												
		**517**	**429**		**I² = 0.00, Tau² = 0.00**			**137**	**118**		**I² = 0.00, Tau² = 0.00**		
**Fixed**					**0.67 (0.57, 0.78)**						**0.84 (0.67, 1.06)**		
**Random**					**0.67 (0.57, 0.78)**						**0.84 (0.67, 1.06)**		

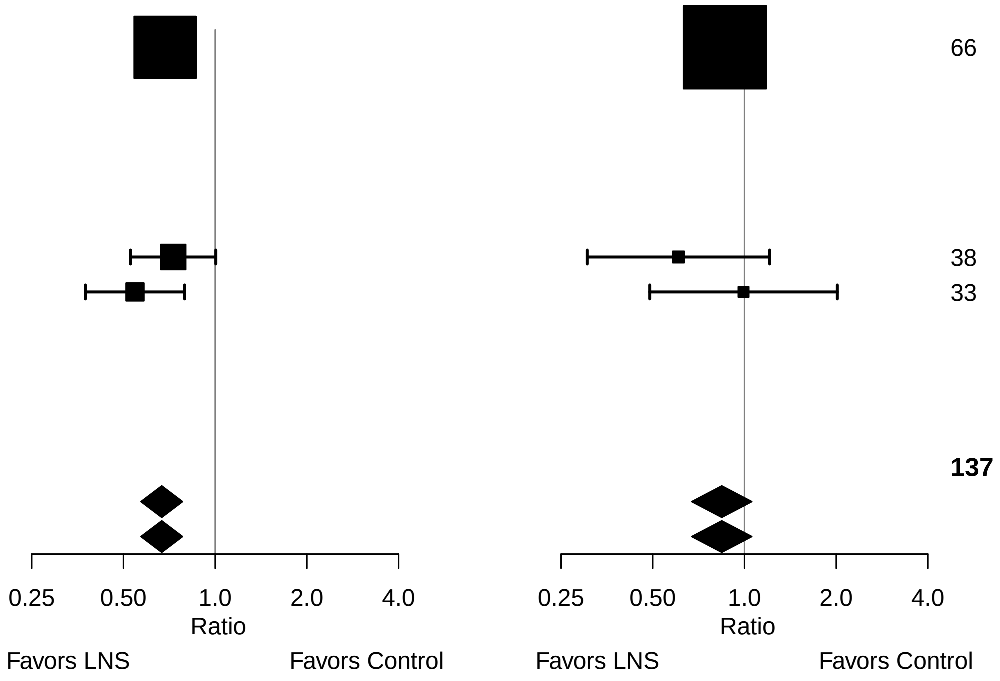

Supplemental figure 8O: Elevated zinc protoporphyrin prevalence ratio

8O6: Stratified by Child baseline acute malnutrition (insufficient comparisons)

Supplemental figure 8O: Elevated zinc protoporphyrin prevalence ratio

8O7: Stratified by Child baseline anemia

P−for−interaction = 0.305
Ratio of PRs = 1.26 (0.81, 1.94)

Country	Trial	Not anemic LNS N	Not anemic Control N	Not anemic Control Prevalence	Not anemic PR (95% CI)	Not anemic Fixed W	Not anemic Random W	Anemic LNS N	Anemic Control N	Anemic Control Prevalence	Anemic PR (95% CI)	Anemic Fixed W	Anemic Random W
Bangladesh	JiVitA−4 (35)												
Bangladesh	RDNS (36)												
Bangladesh	WASH−B (37)												
Burkina Faso	iLiNS−Zinc (38)												
Burkina Faso	PROMIS CS (39)												
Ghana	GHANA (40)												
Ghana	iLiNS−DYAD−G (41)	75	132	23.5	0.51 (0.26, 1.01)	0.27	0.27	25	70	25.7	1.40 (0.73, 2.70)	0.12	0.24
Kenya	WASH−B (42)												
Madagascar	MAHAY (43)												
Malawi	iLiNS−DYAD−M (44)	59	121	23.1	0.66 (0.33, 1.31)	0.27	0.27	121	242	38.0	0.72 (0.51, 1.00)	0.49	0.39
Malawi	iLiNS−DOSE (45)	66	36	36.1	0.62 (0.37, 1.05)	0.46	0.46	115	50	50.0	0.62 (0.43, 0.90)	0.39	0.37
Mali	PROMIS CS (46)												
Zimbabwe	SHINE (HIV−) (47)												
Zimbabwe	SHINE (HIV+) (48)												
		**200**	**289**		**I² = 0.00, Tau² = 0.00**			**261**	**362**		**I² = 0.56, Tau² = 0.10**		
**Fixed**					**0.60 (0.42, 0.86)**						**0.74 (0.58, 0.93)**		
**Random**					**0.60 (0.42, 0.86)**						**0.80 (0.51, 1.25)**		

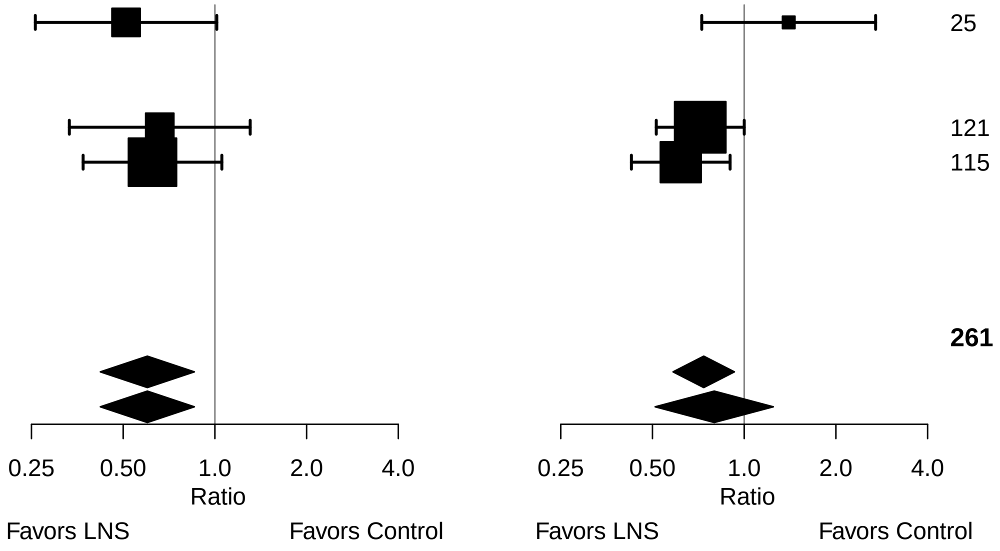

Supplemental figure 8O: Elevated zinc protoporphyrin prevalence ratio

8O8: Stratified by Child high-dose vitamin A supplementation (insufficient comparisons)

Supplemental figure 8O: Elevated zinc protoporphyrin prevalence ratio

8O9: Stratified by Child inflammation

P−for−interaction = 0.980
Ratio of PRs = 1.00 (0.76, 1.30)

		Not inflamed						High AGP or CRP					
Country	Trial	LNS N	Control N	Control Prevalence	PR (95% CI)	Fixed W	Random W	LNS N	Control N	Control Prevalence	PR (95% CI)	Fixed W	Random W
Bangladesh	JiVitA−4 (35)												
Bangladesh	RDNS (36)												
Bangladesh	WASH−B (37)												
Burkina Faso	iLiNS−Zinc (38)	123	30	78.8	0.68 (0.51, 0.92)	0.59	0.59	189	65	73.6	0.73 (0.58, 0.92)	0.50	0.50
Burkina Faso	PROMIS CS (39)												
Ghana	GHANA (40)												
Ghana	iLiNS−DYAD−G (41)	45	120	25.0	0.62 (0.29, 1.31)	0.09	0.09	55	82	23.2	0.86 (0.45, 1.67)	0.06	0.06
Kenya	WASH−B (42)												
Madagascar	MAHAY (43)												
Malawi	iLiNS−DYAD−M (44)	53	119	29.4	0.83 (0.48, 1.44)	0.17	0.17	137	256	35.5	0.66 (0.47, 0.93)	0.22	0.22
Malawi	iLiNS−DOSE (45)	62	26	42.3	0.69 (0.38, 1.26)	0.14	0.14	119	61	45.9	0.58 (0.41, 0.82)	0.22	0.22
Mali	PROMIS CS (46)												
Zimbabwe	SHINE (HIV−) (47)												
Zimbabwe	SHINE (HIV+) (48)												
		**283**	**295**		**I² = 0.00, Tau² = 0.00**			**500**	**464**		**I² = 0.00, Tau² = 0.00**		
**Fixed**					**0.70 (0.56, 0.88)**						**0.69 (0.58, 0.81)**		
**Random**					**0.70 (0.56, 0.88)**						**0.69 (0.58, 0.81)**		

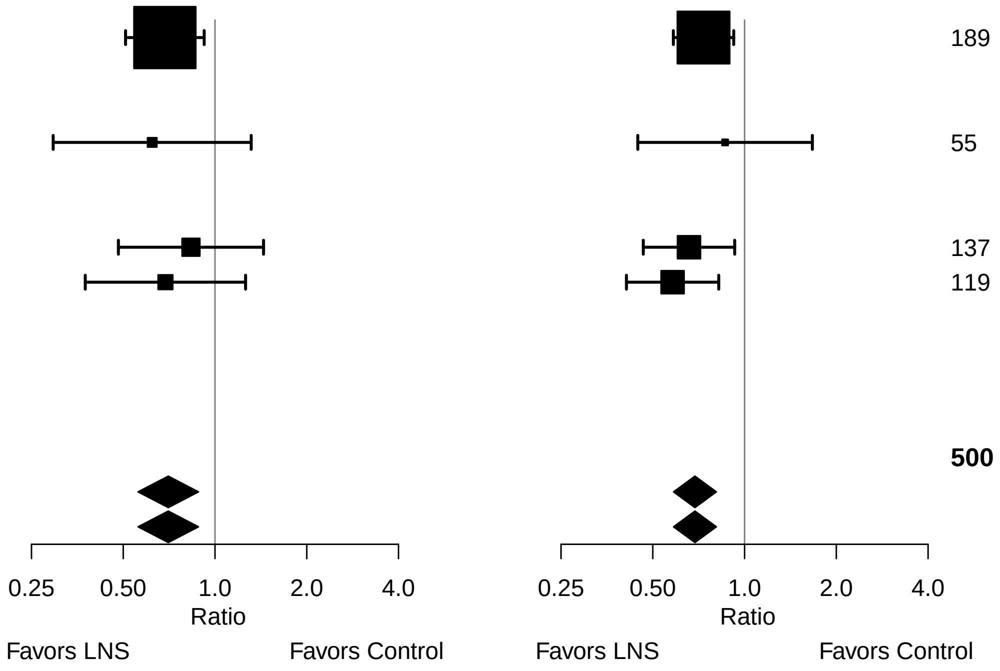

Supplemental figure 8P: Elevated zinc protoporphyrin prevalence difference

8P1: Stratified by Maternal BMI

P−for−interaction = 0.612
Difference in PDs = 0.03 (−0.08, 0.14)

Country	Trial	At least 20 kg/m² LNS N	Control N	Control Prevalence	PD (95% CI)	Fixed W	Random W	Less than 20 kg/m² LNS N	Control N	Control Prevalence	PD (95% CI)	Fixed W	Random W
Bangladesh	JiVitA−4 (35)												
Bangladesh	RDNS (36)												
Bangladesh	WASH−B (37)												
Burkina Faso	iLiNS−Zinc (38)	208	55	73.8	−0.20 (−0.36, −0.04)	0.20	0.20	104	40	77.3	−0.23 (−0.37, −0.10)	0.39	0.38
Burkina Faso	PROMIS CS (39)												
Ghana	GHANA (40)												
Ghana	iLiNS−DYAD−G (41)												
Kenya	WASH−B (42)												
Madagascar	MAHAY (43)												
Malawi	iLiNS−DYAD−M (44)	111	222	36.5	−0.13 (−0.24, −0.02)	0.47	0.47	79	151	29.1	−0.05 (−0.17, 0.07)	0.46	0.41
Malawi	iLiNS−DOSE (45)	127	65	46.2	−0.17 (−0.30, −0.05)	0.33	0.33	53	22	40.9	−0.19 (−0.40, 0.02)	0.15	0.22
Mali	PROMIS CS (46)												
Zimbabwe	SHINE (HIV−) (47)												
Zimbabwe	SHINE (HIV+) (48)												
		**446**	**342**		**I² = 0.00, Tau² = 0.00**			**236**	**213**		**I² = 0.53, Tau² = 0.00**		
**Fixed**					**−0.16 (−0.23, −0.09)**						**−0.14 (−0.23, −0.06)**		
**Random**					**−0.16 (−0.23, −0.09)**						**−0.15 (−0.27, −0.03)**		

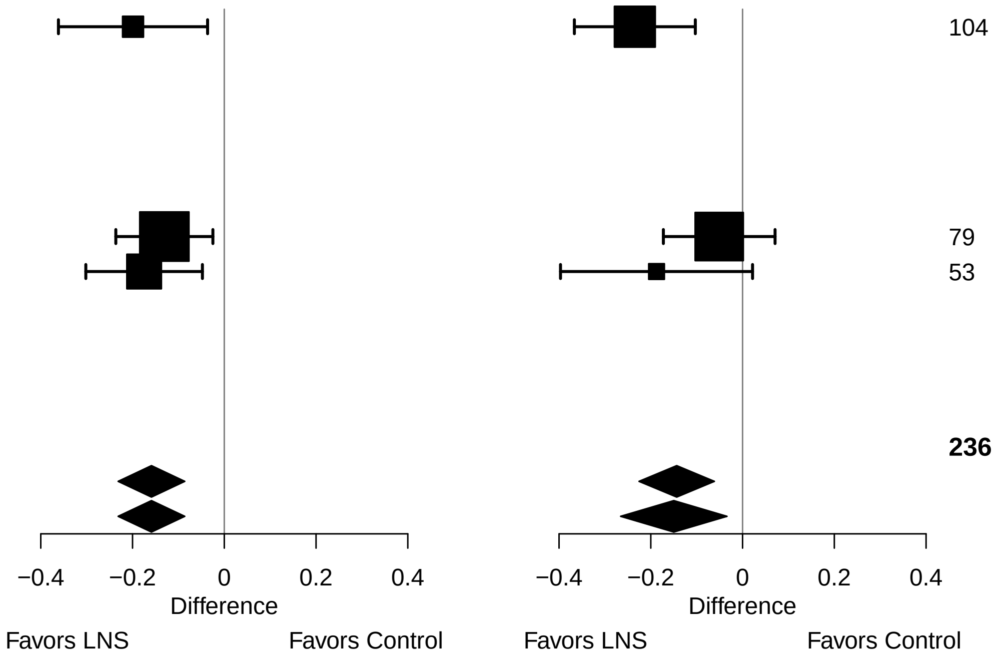

Supplemental figure 8P: Elevated zinc protoporphyrin prevalence difference

8P2: Stratified by Maternal age

P−for−interaction = 0.689
Difference in PDs = −0.02 (−0.12, 0.08)

		At least 25 y						Less than 25 y					
		**LNS**	**Control**	**Control**	**PD**	**Fixed**	**Random**	**LNS**	**Control**	**Control**	**PD**	**Fixed**	**Random**
Country	Trial	N	N	Prevalence	(95% CI)	W	W	N	N	Prevalence	(95% CI)	W	W
Bangladesh	JiVitA−4 (35)												
Bangladesh	RDNS (36)												
Bangladesh	WASH−B (37)												
Burkina Faso	iLiNS−Zinc (38)	175	50	74.1	−0.24 (−0.42, −0.07)	0.12	0.17	137	45	76.5	−0.17 (−0.37, 0.03)	0.16	0.16
Burkina Faso	PROMIS CS (39)												
Ghana	GHANA (40)												
Ghana	iLiNS−DYAD−G (41)	65	128	16.4	−0.04 (−0.15, 0.07)	0.35	0.30	35	74	37.8	−0.09 (−0.29, 0.10)	0.17	0.17
Kenya	WASH−B (42)												
Madagascar	MAHAY (43)												
Malawi	iLiNS−DYAD−M (44)	88	192	28.1	−0.07 (−0.18, 0.05)	0.32	0.29	102	183	39.3	−0.14 (−0.25, −0.02)	0.47	0.47
Malawi	iLiNS−DOSE (45)	95	48	41.7	−0.18 (−0.32, −0.04)	0.21	0.23	83	39	48.7	−0.17 (−0.34, 0.01)	0.21	0.21
Mali	PROMIS CS (46)												
Zimbabwe	SHINE (HIV−) (47)												
Zimbabwe	SHINE (HIV+) (48)												
		**423**	**418**		**I² = 0.43, Tau² = 0.00**			**357**	**341**		**I² = 0.00, Tau² = 0.00**		
**Fixed**					**−0.10 (−0.17, −0.04)**						**−0.14 (−0.22, −0.06)**		
**Random**					**−0.11 (−0.20, −0.03)**						**−0.14 (−0.22, −0.06)**		

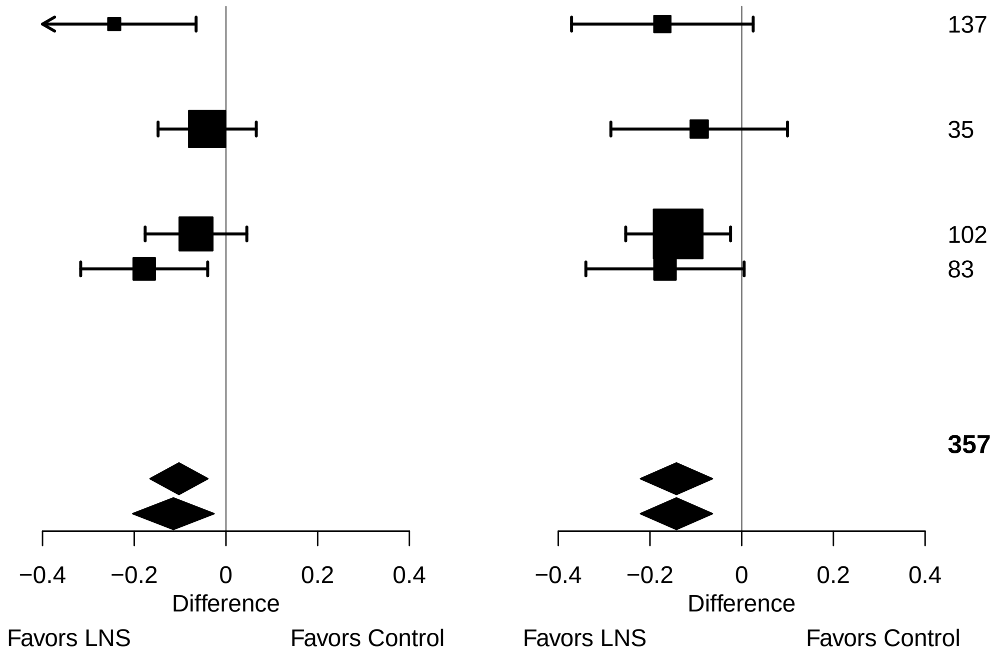

Supplemental figure 8P: Elevated zinc protoporphyrin prevalence difference

8P3: Stratified by Maternal education

P−for−interaction = 0.080
Difference in PDs = 0.13 (−0.02, 0.27)

		Primary or greater						Incomplete or no formal					
Country	Trial	LNS N	Control N	Control Prevalence	PD (95% CI)	Fixed W	Random W	LNS N	Control N	Control Prevalence	PD (95% CI)	Fixed W	Random W
Bangladesh	JiVitA−4 (35)												
Bangladesh	RDNS (36)												
Bangladesh	WASH−B (37)												
Burkina Faso	iLiNS−Zinc (38)												
Burkina Faso	PROMIS CS (39)												
Ghana	GHANA (40)												
Ghana	iLiNS−DYAD−G (41)	83	159	25.2	−0.09 (−0.20, 0.01)	0.65	0.60	17	43	20.9	0.08 (−0.16, 0.33)	0.08	0.12
Kenya	WASH−B (42)												
Madagascar	MAHAY (43)												
Malawi	iLiNS−DYAD−M (44)	31	53	45.3	−0.16 (−0.38, 0.05)	0.17	0.19	158	320	31.6	−0.09 (−0.18, −0.01)	0.63	0.55
Malawi	iLiNS−DOSE (45)	48	26	53.8	−0.28 (−0.49, −0.07)	0.18	0.21	130	61	41.0	−0.12 (−0.25, 0.00)	0.29	0.33
Mali	PROMIS CS (46)												
Zimbabwe	SHINE (HIV−) (47)												
Zimbabwe	SHINE (HIV+) (48)												
		**162**	**238**		**I² = 0.16, Tau² = 0.00**			**305**	**424**		**I² = 0.13, Tau² = 0.00**		
**Fixed**					**−0.14 (−0.23, −0.05)**						**−0.09 (−0.16, −0.02)**		
**Random**					**−0.15 (−0.25, −0.05)**						**−0.08 (−0.17, 0.00)**		

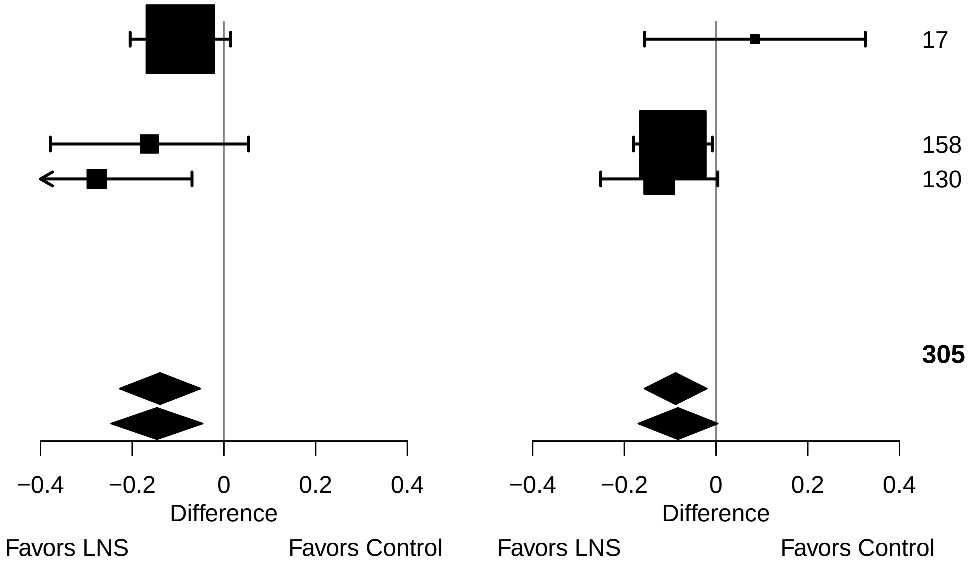

Supplemental figure 8P: Elevated zinc protoporphyrin prevalence difference

8P4: Stratified by Child sex

P−for−interaction = 0.276
Difference in PDs = 0.05 (−0.04, 0.15)

Country	Trial	LNS N	Control N	Control Prevalence	Male PD (95% CI)	Fixed W	Random W	LNS N	Control N	Control Prevalence	Female PD (95% CI)	Fixed W	Random W
Bangladesh	JiVitA−4 (35)												
Bangladesh	RDNS (36)												
Bangladesh	WASH−B (37)												
Burkina Faso	iLiNS−Zinc (38)	149	49	81.5	−0.26 (−0.41, −0.10)	0.20	0.20	163	46	68.6	−0.17 (−0.27, −0.06)	0.37	0.36
Burkina Faso	PROMIS CS (39)												
Ghana	GHANA (40)												
Ghana	iLiNS−DYAD−G (41)	63	95	26.3	−0.07 (−0.21, 0.06)	0.26	0.26	36	106	21.7	−0.05 (−0.20, 0.10)	0.16	0.16
Kenya	WASH−B (42)												
Madagascar	MAHAY (43)												
Malawi	iLiNS−DYAD−M (44)	95	183	37.2	−0.14 (−0.26, −0.02)	0.36	0.35	95	192	30.2	−0.06 (−0.17, 0.05)	0.31	0.30
Malawi	iLiNS−DOSE (45)	86	46	43.5	−0.16 (−0.32, 0.00)	0.19	0.19	95	41	46.3	−0.17 (−0.32, −0.02)	0.17	0.17
Mali	PROMIS CS (46)												
Zimbabwe	SHINE (HIV−) (47)												
Zimbabwe	SHINE (HIV+) (48)												
		**393**	**373**		**I² = 0.05, Tau² = 0.00**			**389**	**385**		**I² = 0.04, Tau² = 0.00**		
**Fixed**					**−0.15 (−0.22, −0.08)**						**−0.12 (−0.18, −0.05)**		
**Random**					**−0.15 (−0.22, −0.08)**						**−0.12 (−0.18, −0.05)**		

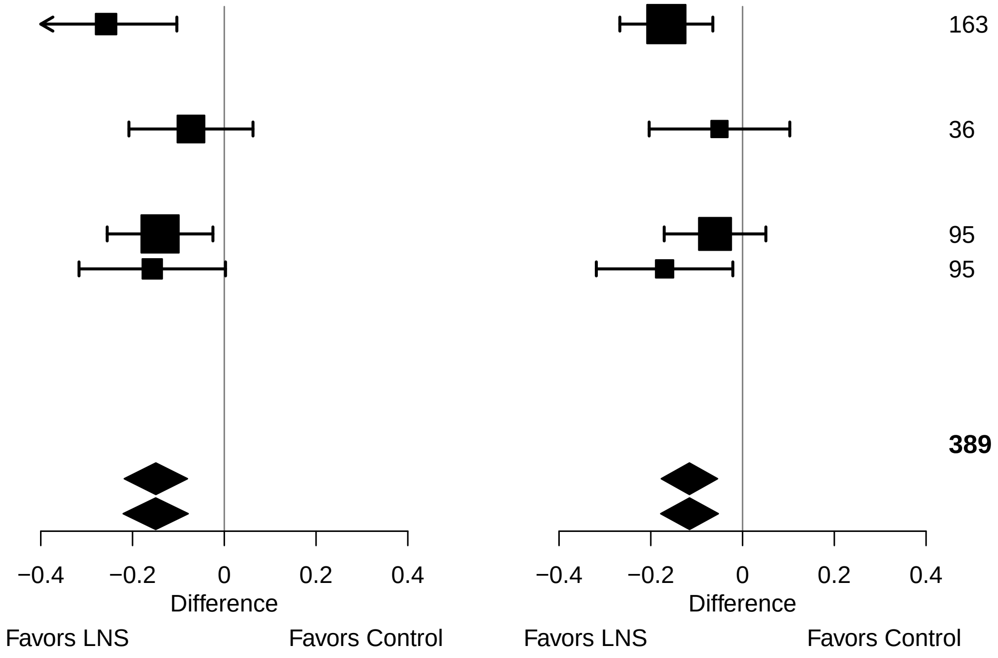

Supplemental figure 8P: Elevated zinc protoporphyrin prevalence difference

8P5: Stratified by Child birth order

P−for−interaction = 0.462
Difference in PDs = 0.05 (−0.08, 0.18)

Country	Trial	LNS N	Control N	Control Prevalence	Later born PD (95% CI)	Fixed W	Random W	LNS N	Control N	Control Prevalence	Firstborn PD (95% CI)	Fixed W	Random W
Bangladesh	JiVitA−4 (35)												
Bangladesh	RDNS (36)												
Bangladesh	WASH−B (37)												
Burkina Faso	iLiNS−Zinc (38)	246	71	74.7	−0.23 (−0.35, −0.12)	0.29	0.32	66	24	76.7	−0.13 (−0.29, 0.04)	0.45	0.45
Burkina Faso	PROMIS CS (39)												
Ghana	GHANA (40)												
Ghana	iLiNS−DYAD−G (41)												
Kenya	WASH−B (42)												
Madagascar	MAHAY (43)												
Malawi	iLiNS−DYAD−M (44)	152	299	33.4	−0.09 (−0.18, 0.00)	0.47	0.40	38	75	34.7	−0.14 (−0.31, 0.04)	0.39	0.39
Malawi	iLiNS−DOSE (45)	119	59	45.8	−0.21 (−0.34, −0.08)	0.23	0.28	33	19	36.8	−0.01 (−0.28, 0.27)	0.17	0.17
Mali	PROMIS CS (46)												
Zimbabwe	SHINE (HIV−) (47)												
Zimbabwe	SHINE (HIV+) (48)												
		**517**	**429**		**I² = 0.55, Tau² = 0.00**			**137**	**118**		**I² = 0.00, Tau² = 0.00**		
**Fixed**					**−0.16 (−0.22, −0.10)**						**−0.11 (−0.22, 0.00)**		
**Random**					**−0.17 (−0.26, −0.08)**						**−0.11 (−0.22, 0.00)**		

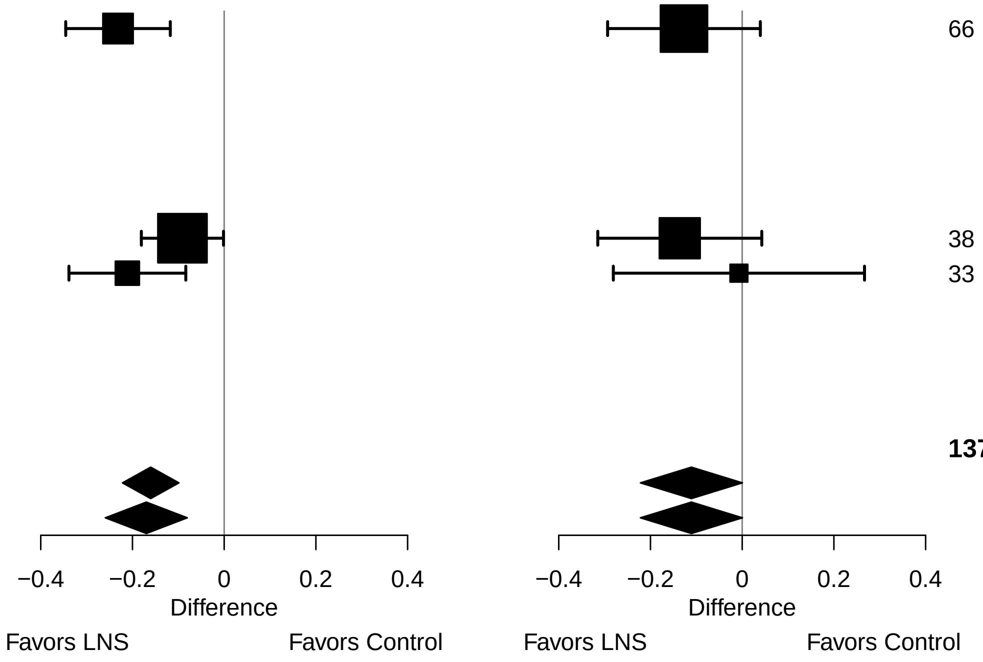

Supplemental figure 8P: Elevated zinc protoporphyrin prevalence difference

8P6: Stratified by Child baseline acute malnutrition (insufficient comparisons)

Supplemental figure 8P: Elevated zinc protoporphyrin prevalence difference

8P7: Stratified by Child baseline anemia

P−for−interaction = 0.663
Difference in PDs = 0.03 (−0.09, 0.14)

Country	Trial	Not anemic LNS N	Not anemic Control N	Not anemic Control Prevalence	Not anemic PD (95% CI)	Not anemic Fixed W	Not anemic Random W	Anemic LNS N	Anemic Control N	Anemic Control Prevalence	Anemic PD (95% CI)	Anemic Fixed W	Anemic Random W
Bangladesh	JiVitA−4 (35)												
Bangladesh	RDNS (36)												
Bangladesh	WASH−B (37)												
Burkina Faso	iLiNS−Zinc (38)												
Burkina Faso	PROMIS CS (39)												
Ghana	GHANA (40)												
Ghana	iLiNS−DYAD−G (41)	75	132	23.5	−0.11 (−0.23, 0.00)	0.45	0.45	25	70	25.7	0.10 (−0.10, 0.31)	0.14	0.26
Kenya	WASH−B (42)												
Madagascar	MAHAY (43)												
Malawi	iLiNS−DYAD−M (44)	59	121	23.1	−0.08 (−0.20, 0.05)	0.35	0.35	121	242	38.0	−0.11 (−0.21, 0.00)	0.57	0.40
Malawi	iLiNS−DOSE (45)	66	36	36.1	−0.14 (−0.31, 0.02)	0.20	0.20	115	50	50.0	−0.18 (−0.32, −0.03)	0.29	0.34
Mali	PROMIS CS (46)												
Zimbabwe	SHINE (HIV−) (47)												
Zimbabwe	SHINE (HIV+) (48)												
		**200**	**289**		**I² = 0.00, Tau² = 0.00**			**261**	**362**		**I² = 0.58, Tau² = 0.01**		
**Fixed**					**−0.11 (−0.18, −0.03)**						**−0.10 (−0.18, −0.02)**		
**Random**					**−0.11 (−0.18, −0.03)**						**−0.08 (−0.23, 0.08)**		

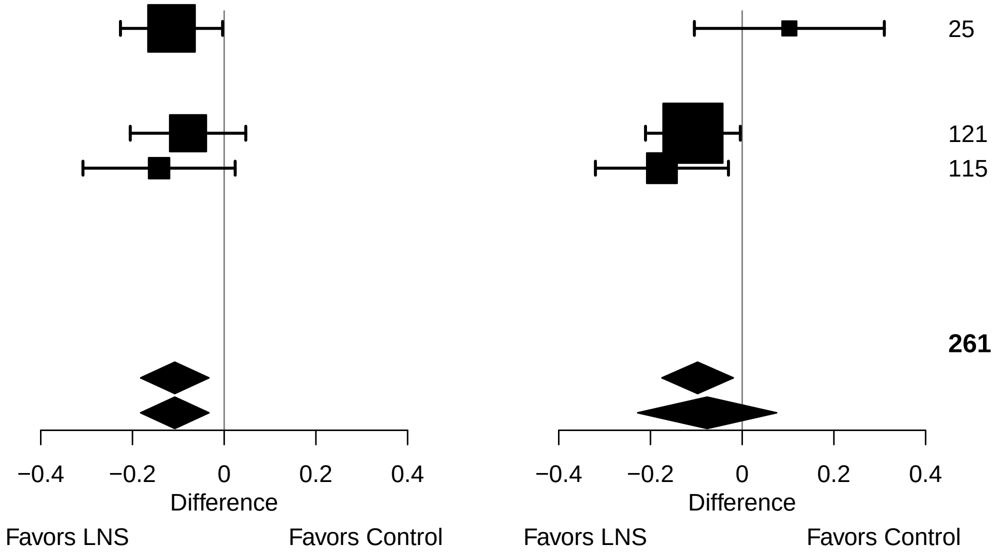

Supplemental figure 8P: Elevated zinc protoporphyrin prevalence difference

8P8: Stratified by Child high-dose vitamin A supplementation (insufficient comparisons)

Supplemental figure 8P: Elevated zinc protoporphyrin prevalence difference

8P9: Stratified by Child inflammation

P−for−interaction = 0.882
Difference in PDs = −0.01 (−0.11, 0.09)

		Not inflamed						High AGP or CRP					
Country	Trial	LNS N	Control N	Control Prevalence	PD (95% CI)	Fixed W	Random W	LNS N	Control N	Control Prevalence	PD (95% CI)	Fixed W	Random W
Bangladesh	JiVitA−4 (35)												
Bangladesh	RDNS (36)												
Bangladesh	WASH−B (37)												
Burkina Faso	iLiNS−Zinc (38)	123	30	78.8	−0.24 (−0.45, −0.04)	0.16	0.16	189	65	73.6	−0.20 (−0.32, −0.07)	0.22	0.23
Burkina Faso	PROMIS CS (39)												
Ghana	GHANA (40)												
Ghana	iLiNS−DYAD−G (41)	45	120	25.0	−0.09 (−0.24, 0.05)	0.34	0.34	55	82	23.2	−0.03 (−0.17, 0.11)	0.17	0.19
Kenya	WASH−B (42)												
Madagascar	MAHAY (43)												
Malawi	iLiNS−DYAD−M (44)	53	119	29.4	−0.05 (−0.19, 0.10)	0.33	0.33	137	256	35.5	−0.12 (−0.22, −0.03)	0.39	0.35
Malawi	iLiNS−DOSE (45)	62	26	42.3	−0.14 (−0.34, 0.07)	0.17	0.17	119	61	45.9	−0.19 (−0.31, −0.06)	0.22	0.23
Mali	PROMIS CS (46)												
Zimbabwe	SHINE (HIV−) (47)												
Zimbabwe	SHINE (HIV+) (48)												
		**283**	**295**		**I^2 = 0.00, Tau^2 = 0.00**			**500**	**464**		**I^2 = 0.16, Tau^2 = 0.00**		
**Fixed**					**−0.11 (−0.19, −0.03)**						**−0.14 (−0.20, −0.08)**		
**Random**					**−0.11 (−0.19, −0.03)**						**−0.14 (−0.20, −0.07)**		

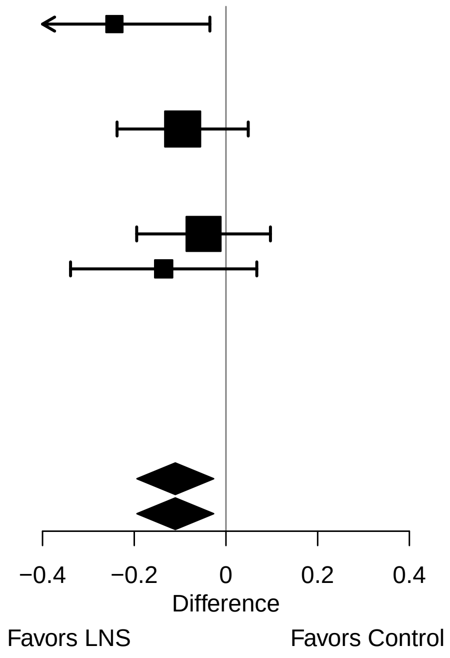

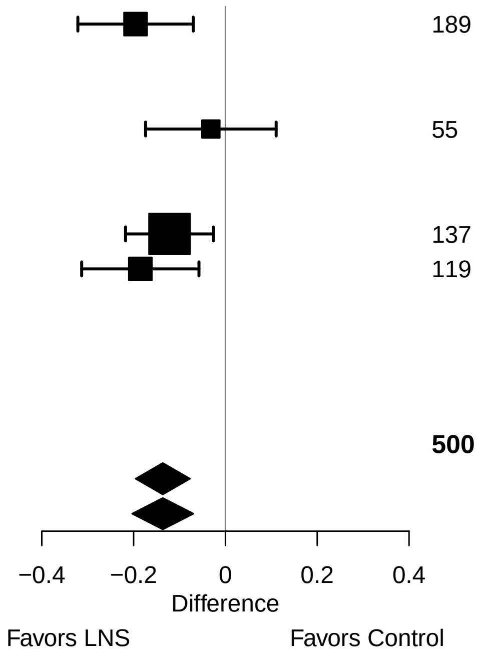

Supplemental figure 8Q: Geometric mean ratio of plasma zinc concentration

8Q1: Stratified by Maternal BMI (insufficient comparisons)

Supplemental figure 8Q: Geometric mean ratio of plasma zinc concentration

8Q2: Stratified by Maternal age

P−for−interaction = 0.003
Ratio of GMRs = 0.94 (0.90, 0.98)

		At least 25 y						Less than 25 y					
Country	Trial	LNS N	Control N	Control Median	GMR (95% CI)	Fixed W	Random W	LNS N	Control N	Control Median	GMR (95% CI)	Fixed W	Random W
Bangladesh	JiVitA−4 (35)	201	70	86.1	1.00 (0.92, 1.08)	0.12	0.12	248	73	91.3	0.93 (0.87, 1.00)	0.31	0.35
Bangladesh	RDNS (36)												
Bangladesh	WASH−B (37)												
Burkina Faso	iLiNS−Zinc (38)	177	50	67.1	1.03 (1.00, 1.06)	0.73	0.73	137	46	69.9	0.98 (0.93, 1.03)	0.64	0.57
Burkina Faso	PROMIS CS (39)												
Ghana	GHANA (40)	44	44	61.0	1.03 (0.96, 1.11)	0.15	0.15	14	19	62.9	0.87 (0.74, 1.03)	0.06	0.08
Ghana	iLiNS−DYAD−G (41)												
Kenya	WASH−B (42)												
Madagascar	MAHAY (43)												
Malawi	iLiNS−DYAD−M (44)												
Malawi	iLiNS−DOSE (45)												
Mali	PROMIS CS (46)												
Zimbabwe	SHINE (HIV−) (47)												
Zimbabwe	SHINE (HIV+) (48)												
		**422**	**164**		**I² = 0.00, Tau² = 0.00**			**399**	**138**		**I² = 0.21, Tau² = 0.00**		
**Fixed**					**1.03 (1.00, 1.05)**						**0.96 (0.92, 1.00)**		
**Random**					**1.03 (1.00, 1.05)**						**0.96 (0.91, 1.00)**		

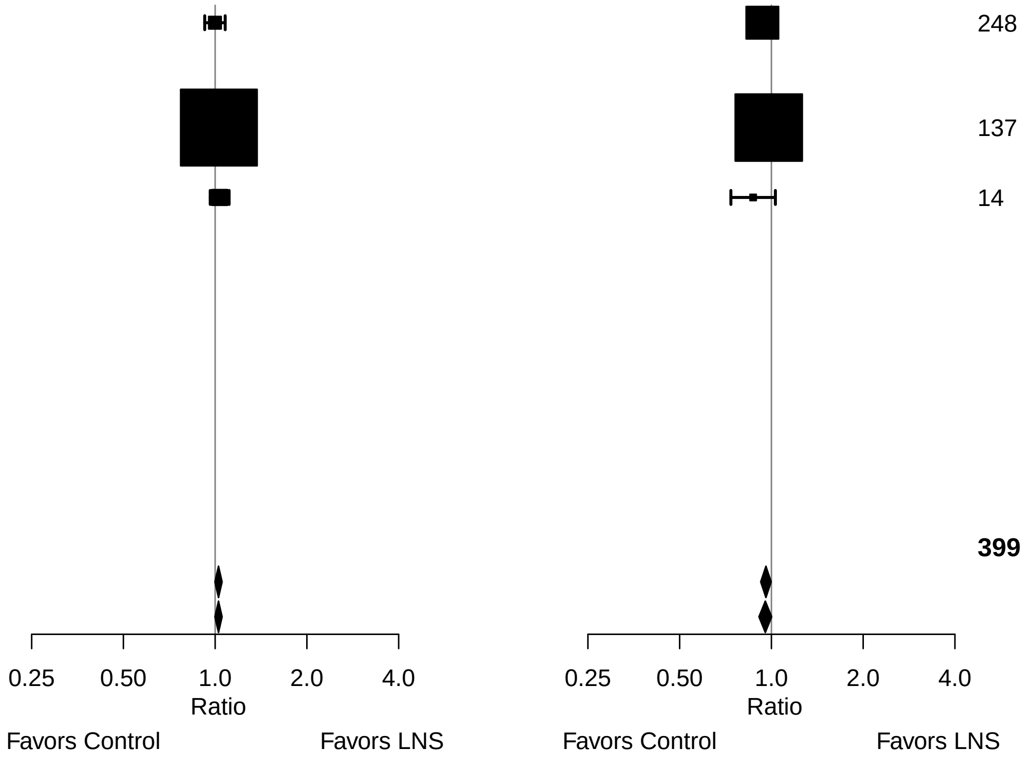

Supplemental figure 8Q: Geometric mean ratio of plasma zinc concentration

8Q3: Stratified by Maternal education (insufficient comparisons)

Supplemental figure 8Q: Geometric mean ratio of plasma zinc concentration

8Q4: Stratified by Child sex

P−for−interaction = 0.728
Ratio of GMRs = 1.01 (0.96, 1.06)

Country	Trial	Male: LNS N	Male: Control N	Male: Control Median	Male: GMR (95% CI)	Male: Fixed W	Male: Random W	Female: LNS N	Female: Control N	Female: Control Median	Female: GMR (95% CI)	Female: Fixed W	Female: Random W
Bangladesh	JiVitA−4 (35)	234	70	91.2	0.95 (0.88, 1.02)	0.25	0.30	218	74	87.0	0.98 (0.91, 1.05)	0.16	0.16
Bangladesh	RDNS (36)												
Bangladesh	WASH−B (37)												
Burkina Faso	iLiNS−Zinc (38)	149	49	67.8	1.00 (0.95, 1.05)	0.57	0.47	165	47	69.8	1.01 (0.98, 1.05)	0.76	0.76
Burkina Faso	PROMIS CS (39)												
Ghana	GHANA (40)	34	31	58.6	1.05 (0.96, 1.15)	0.17	0.23	24	38	61.2	0.96 (0.86, 1.07)	0.08	0.08
Ghana	iLiNS−DYAD−G (41)												
Kenya	WASH−B (42)												
Madagascar	MAHAY (43)												
Malawi	iLiNS−DYAD−M (44)												
Malawi	iLiNS−DOSE (45)												
Mali	PROMIS CS (46)												
Zimbabwe	SHINE (HIV−) (47)												
Zimbabwe	SHINE (HIV+) (48)												
		**417**	**150**		**I² = 0.30, Tau² = 0.00**			**407**	**159**		**I² = 0.00, Tau² = 0.00**		
**Fixed**					**0.99 (0.96, 1.03)**						**1.00 (0.97, 1.03)**		
**Random**					**0.99 (0.95, 1.04)**						**1.00 (0.97, 1.03)**		

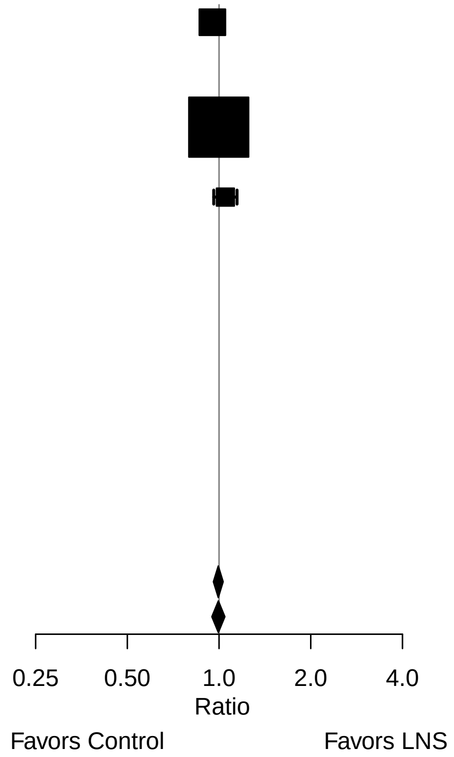

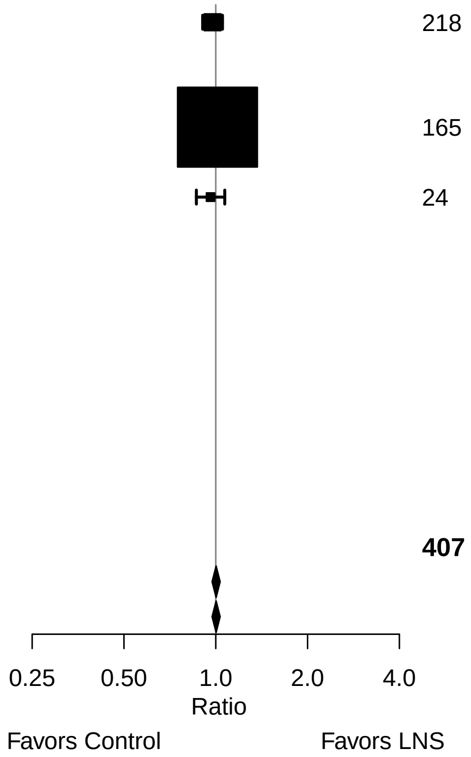

Supplemental figure 8Q: Geometric mean ratio of plasma zinc concentration

8Q5: Stratified by Child birth order

P−for−interaction = 0.131
Ratio of GMRs = 0.96 (0.91, 1.01)

		Later born						Firstborn					
		**LNS**	**Control**	**Control**	**GMR**	**Fixed**	**Random**	**LNS**	**Control**	**Control**	**GMR**	**Fixed**	**Random**
Country	Trial	N	N	Median	(95% CI)	W	W	N	N	Median	(95% CI)	W	W
Bangladesh	JiVitA−4 (35)	113	32	86.1	1.00 (0.90, 1.12)	0.08	0.08	338	111	88.8	0.95 (0.90, 1.01)	0.47	0.47
Bangladesh	RDNS (36)												
Bangladesh	WASH−B (37)												
Burkina Faso	iLiNS−Zinc (38)	248	71	66.5	1.02 (0.98, 1.06)	0.77	0.77	66	25	72.6	0.97 (0.91, 1.03)	0.41	0.41
Burkina Faso	PROMIS CS (39)												
Ghana	GHANA (40)	38	43	61.5	0.99 (0.91, 1.08)	0.15	0.15	20	23	59.8	1.00 (0.89, 1.12)	0.12	0.12
Ghana	iLiNS−DYAD−G (41)												
Kenya	WASH−B (42)												
Madagascar	MAHAY (43)												
Malawi	iLiNS−DYAD−M (44)												
Malawi	iLiNS−DOSE (45)												
Mali	PROMIS CS (46)												
Zimbabwe	SHINE (HIV−) (47)												
Zimbabwe	SHINE (HIV+) (48)												
		**399**	**146**		**I^2 = 0.00, Tau^2 = 0.00**			**424**	**159**		**I^2 = 0.00, Tau^2 = 0.00**		
**Fixed**					**1.01 (0.98, 1.05)**						**0.97 (0.93, 1.00)**		
**Random**					**1.01 (0.98, 1.05)**						**0.97 (0.93, 1.00)**		

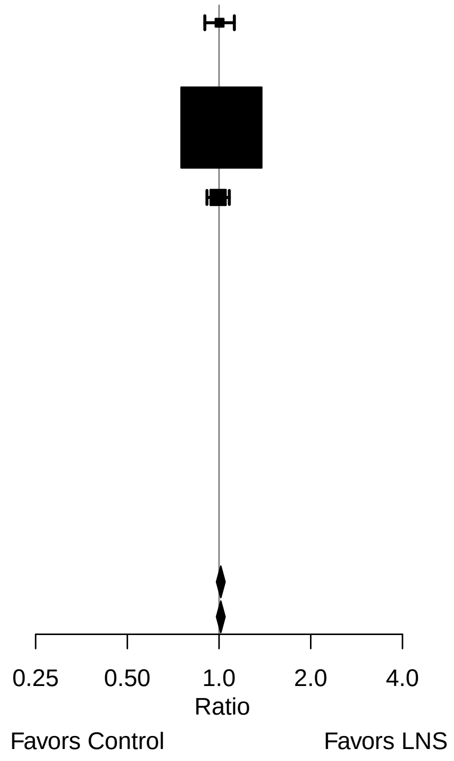

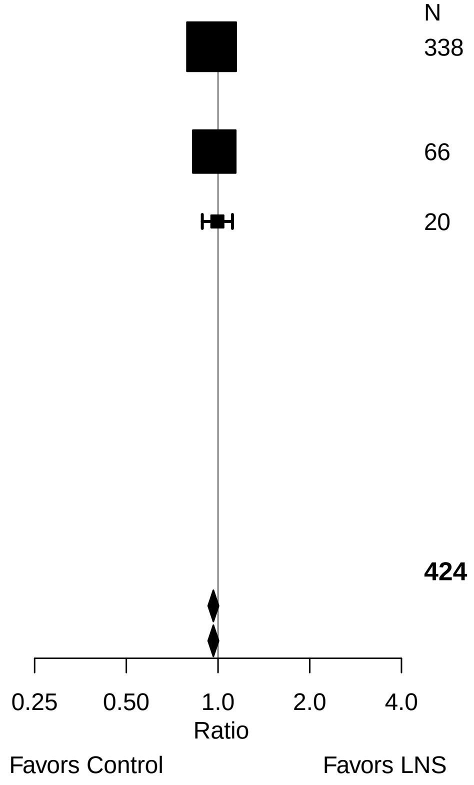

Supplemental figure 8Q: Geometric mean ratio of plasma zinc concentration

8Q6: Stratified by Child baseline acute malnutrition (insufficient comparisons)

Supplemental figure 8Q: Geometric mean ratio of plasma zinc concentration

8Q7: Stratified by Child baseline anemia (insufficient comparisons)

Supplemental figure 8Q: Geometric mean ratio of plasma zinc concentration

8Q8: Stratified by Child high-dose vitamin A supplementation (insufficient comparisons)

Supplemental figure 8Q: Geometric mean ratio of plasma zinc concentration

8Q9: Stratified by Child inflammation

P−for−interaction = 0.425
Ratio of GMRs = 1.02 (0.97, 1.06)

		Not inflamed						High AGP or CRP					
		**LNS**	**Control**	**Control**	**GMR**	**Fixed**	**Random**	**LNS**	**Control**	**Control**	**GMR**	**Fixed**	**Random**
Country	Trial	N	N	Median	(95% CI)	W	W	N	N	Median	(95% CI)	W	W
Bangladesh	JiVitA−4 (35)	184	46	88.8	0.95 (0.89, 1.01)	0.24	0.26	268	98	88.1	0.97 (0.91, 1.03)	0.29	0.35
Bangladesh	RDNS (36)												
Bangladesh	WASH−B (37)												
Burkina Faso	iLiNS−Zinc (38)	124	31	68.5	0.99 (0.94, 1.03)	0.58	0.54	190	65	69.0	1.02 (0.97, 1.06)	0.66	0.56
Burkina Faso	PROMIS CS (39)												
Ghana	GHANA (40)	48	54	59.5	1.03 (0.95, 1.11)	0.18	0.20	10	15	61.5	0.91 (0.78, 1.06)	0.05	0.09
Ghana	iLiNS−DYAD−G (41)												
Kenya	WASH−B (42)												
Madagascar	MAHAY (43)												
Malawi	iLiNS−DYAD−M (44)												
Malawi	iLiNS−DOSE (45)												
Mali	PROMIS CS (46)												
Zimbabwe	SHINE (HIV−) (47)												
Zimbabwe	SHINE (HIV+) (48)												
		**356**	**131**		**I² = 0.12, Tau² = 0.00**			**468**	**178**		**I² = 0.30, Tau² = 0.00**		
**Fixed**					**0.98 (0.95, 1.02)**						**1.00 (0.96, 1.03)**		
**Random**					**0.98 (0.95, 1.02)**						**0.99 (0.94, 1.04)**		

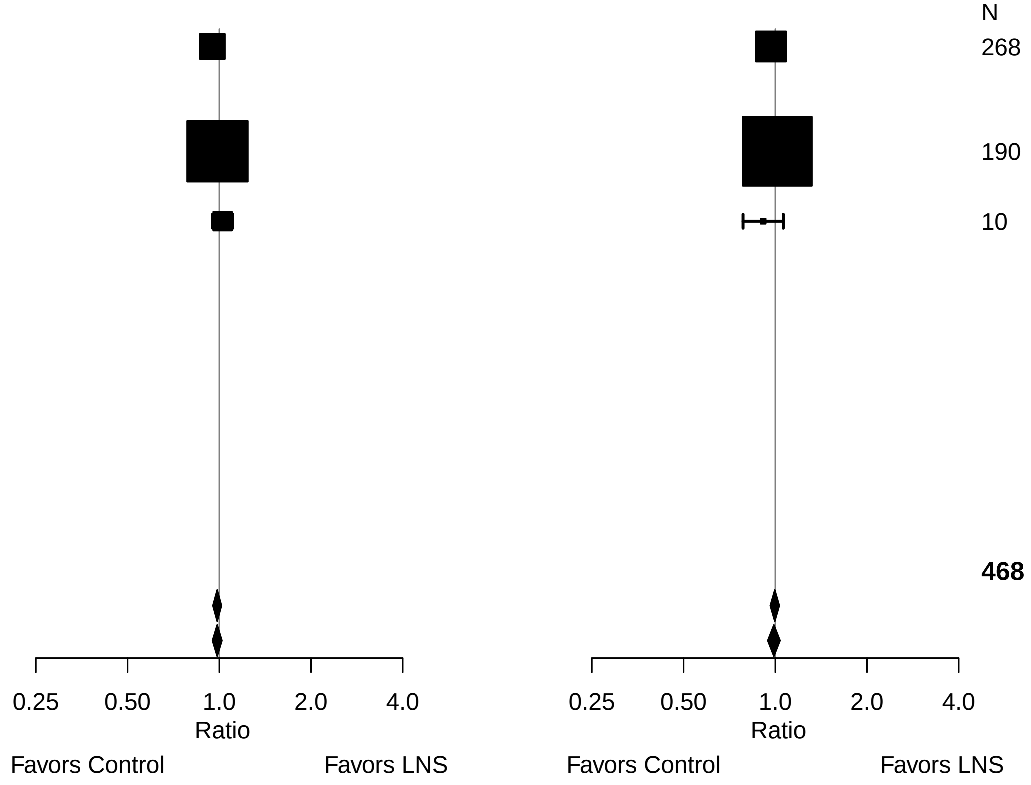

Supplemental figure 8R: Geometric mean ratio of retinol concentration

8R1: Stratified by Maternal BMI (insufficient comparisons)

Supplemental figure 8R: Geometric mean ratio of retinol concentration

8R2: Stratified by Maternal age

P−for−interaction = 0.473
Ratio of GMRs = 0.97 (0.90, 1.05)

At least 25 y

Country	Trial	**LNS** N	**Control** N	**Control** Median	**GMR** (95% CI)	**Fixed** W	**Random** W
Bangladesh	JiVitA−4 (35)	195	67	1.4	1.12 (1.03, 1.22)	0.31	0.30
Bangladesh	RDNS (36)						
Bangladesh	WASH−B (37)						
Burkina Faso	iLiNS−Zinc (38)						
Burkina Faso	PROMIS CS (39)						
Ghana	GHANA (40)	47	47	1.0	1.13 (0.97, 1.32)	0.10	0.15
Ghana	iLiNS−DYAD−G (41)	63	124	1.1	0.98 (0.91, 1.06)	0.42	0.34
Kenya	WASH−B (42)						
Madagascar	MAHAY (43)						
Malawi	iLiNS−DYAD−M (44)	33	81	1.1	1.02 (0.91, 1.15)	0.16	0.21
Malawi	iLiNS−DOSE (45)						
Mali	PROMIS CS (46)						
Zimbabwe	SHINE (HIV−) (47)						
Zimbabwe	SHINE (HIV+) (48)						
		**338**	**319**		**I² = 0.52, Tau² = 0.00**		
**Fixed**					**1.05 (1.00, 1.10)**		
**Random**					**1.05 (0.98, 1.13)**		

Less than 25 y

Country	Trial	**LNS** N	**Control** N	**Control** Median	**GMR** (95% CI)	**Fixed** W	**Random** W
Bangladesh	JiVitA−4 (35)	237	71	1.3	1.05 (0.97, 1.14)	0.49	0.49
Bangladesh	RDNS (36)						
Bangladesh	WASH−B (37)						
Burkina Faso	iLiNS−Zinc (38)						
Burkina Faso	PROMIS CS (39)						
Ghana	GHANA (40)	16	21	1.2	0.91 (0.67, 1.24)	0.04	0.04
Ghana	iLiNS−DYAD−G (41)	33	72	1.0	1.05 (0.93, 1.18)	0.24	0.24
Kenya	WASH−B (42)						
Madagascar	MAHAY (43)						
Malawi	iLiNS−DYAD−M (44)	38	82	1.0	0.99 (0.88, 1.12)	0.23	0.23
Malawi	iLiNS−DOSE (45)						
Mali	PROMIS CS (46)						
Zimbabwe	SHINE (HIV−) (47)						
Zimbabwe	SHINE (HIV+) (48)						
		**324**	**246**		**I² = 0.00, Tau² = 0.00**		
**Fixed**					**1.03 (0.97, 1.09)**		
**Random**					**1.03 (0.97, 1.09)**		

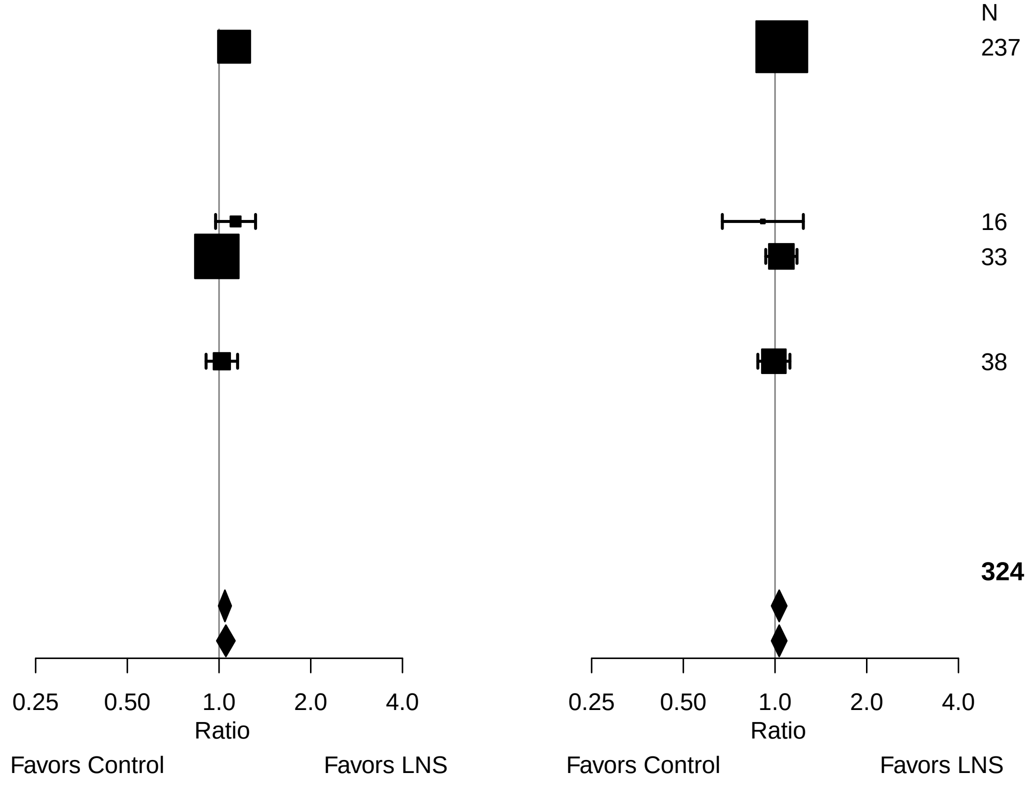

Supplemental figure 8R: Geometric mean ratio of retinol concentration

8R3: Stratified by Maternal education

P−for−interaction = 0.607
Ratio of GMRs = 0.97 (0.88, 1.08)

Country	Trial	Primary or greater: LNS N	Control N	Control Median	GMR (95% CI)	Fixed W	Random W	Incomplete or no formal: LNS N	Control N	Control Median	GMR (95% CI)	Fixed W	Random W
Bangladesh	JiVitA−4 (35)	283	90	1.4	1.08 (1.00, 1.17)	0.44	0.44	150	49	1.3	1.08 (0.95, 1.23)	0.28	0.28
Bangladesh	RDNS (36)												
Bangladesh	WASH−B (37)												
Burkina Faso	iLiNS−Zinc (38)												
Burkina Faso	PROMIS CS (39)												
Ghana	GHANA (40)												
Ghana	iLiNS−DYAD−G (41)	79	154	1.0	1.02 (0.95, 1.10)	0.52	0.52	17	42	1.0	0.95 (0.81, 1.10)	0.19	0.19
Kenya	WASH−B (42)												
Madagascar	MAHAY (43)												
Malawi	iLiNS−DYAD−M (44)	9	22	1.1	0.98 (0.76, 1.26)	0.04	0.04	61	141	1.1	1.00 (0.91, 1.09)	0.53	0.53
Malawi	iLiNS−DOSE (45)												
Mali	PROMIS CS (46)												
Zimbabwe	SHINE (HIV−) (47)												
Zimbabwe	SHINE (HIV+) (48)												
		**371**	**266**		**I² = 0.00, Tau² = 0.00**			**228**	**232**		**I² = 0.00, Tau² = 0.00**		
**Fixed**					**1.05 (0.99, 1.10)**						**1.01 (0.95, 1.08)**		
**Random**					**1.05 (0.99, 1.10)**						**1.01 (0.95, 1.08)**		

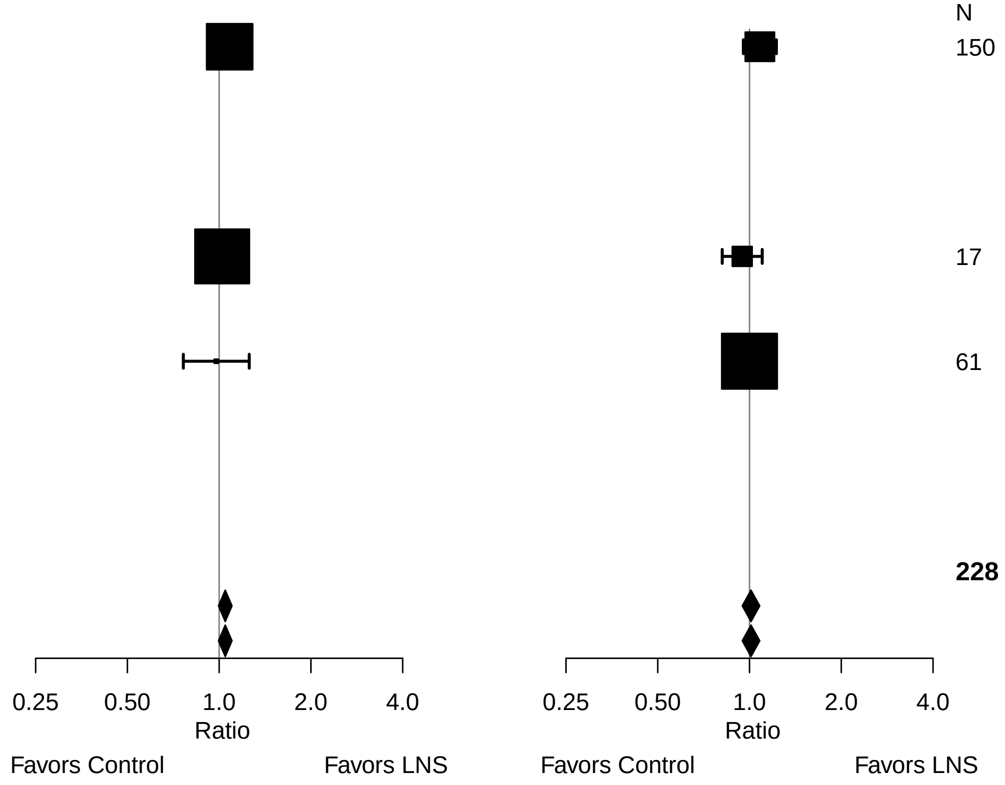

Supplemental figure 8R: Geometric mean ratio of retinol concentration

8R4: Stratified by Child sex

P−for−interaction = 0.315
Ratio of GMRs = 1.04 (0.96, 1.12)

		Male						Female					
Country	Trial	LNS N	Control N	Control Median	GMR (95% CI)	Fixed W	Random W	LNS N	Control N	Control Median	GMR (95% CI)	Fixed W	Random W
Bangladesh	JiVitA−4 (35)	222	67	1.4	1.02 (0.93, 1.11)	0.37	0.37	212	72	1.4	1.15 (1.05, 1.26)	0.38	0.33
Bangladesh	RDNS (36)												
Bangladesh	WASH−B (37)												
Burkina Faso	iLiNS−Zinc (38)												
Burkina Faso	PROMIS CS (39)												
Ghana	GHANA (40)	37	32	1.1	1.01 (0.83, 1.23)	0.07	0.07	26	42	1.0	1.13 (0.93, 1.37)	0.08	0.13
Ghana	iLiNS−DYAD−G (41)	62	93	1.0	1.06 (0.97, 1.15)	0.35	0.35	33	102	1.1	0.98 (0.89, 1.08)	0.34	0.31
Kenya	WASH−B (42)												
Madagascar	MAHAY (43)												
Malawi	iLiNS−DYAD−M (44)	39	80	1.0	1.00 (0.89, 1.12)	0.21	0.21	32	83	1.1	1.01 (0.89, 1.15)	0.19	0.23
Malawi	iLiNS−DOSE (45)												
Mali	PROMIS CS (46)												
Zimbabwe	SHINE (HIV−) (47)												
Zimbabwe	SHINE (HIV+) (48)												
		**360**	**272**		**I² = 0.00, Tau² = 0.00**			**303**	**299**		**I² = 0.55, Tau² = 0.00**		
**Fixed**					**1.03 (0.97, 1.08)**						**1.06 (1.01, 1.13)**		
**Random**					**1.03 (0.97, 1.08)**						**1.06 (0.98, 1.15)**		

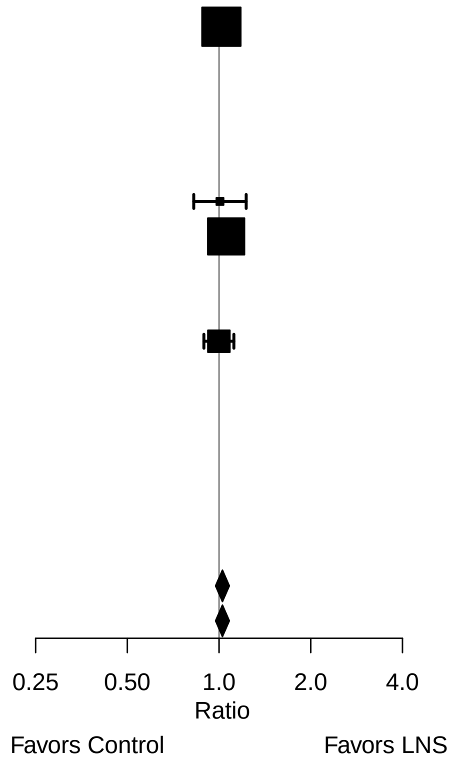

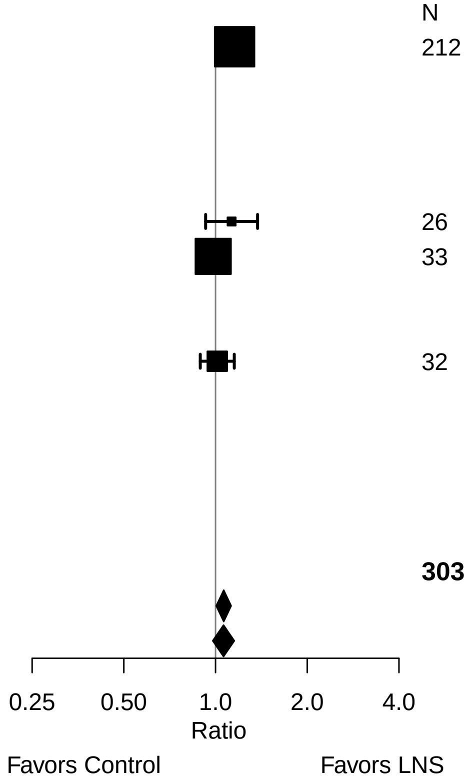

Supplemental figure 8R: Geometric mean ratio of retinol concentration

8R5: Stratified by Child birth order

P−for−interaction = 0.862
Ratio of GMRs = 0.99 (0.91, 1.09)

Country	Trial	Later born LNS N	Later born Control N	Later born Control Median	Later born GMR (95% CI)	Later born Fixed W	Later born Random W	Firstborn LNS N	Firstborn Control N	Firstborn Control Median	Firstborn GMR (95% CI)	Firstborn Fixed W	Firstborn Random W
Bangladesh	JiVitA−4 (35)	106	31	1.5	1.04 (0.89, 1.21)	0.11	0.16	327	107	1.3	1.10 (1.02, 1.17)	0.68	0.57
Bangladesh	RDNS (36)												
Bangladesh	WASH−B (37)												
Burkina Faso	iLiNS−Zinc (38)												
Burkina Faso	PROMIS CS (39)												
Ghana	GHANA (40)	42	45	1.0	1.20 (1.02, 1.40)	0.11	0.16	21	26	1.1	0.87 (0.67, 1.13)	0.05	0.07
Ghana	iLiNS−DYAD−G (41)	68	137	1.0	1.01 (0.94, 1.08)	0.47	0.37	28	59	1.0	1.01 (0.89, 1.14)	0.20	0.26
Kenya	WASH−B (42)												
Madagascar	MAHAY (43)												
Malawi	iLiNS−DYAD−M (44)	56	129	1.1	1.00 (0.91, 1.09)	0.31	0.31	15	34	1.1	1.04 (0.83, 1.30)	0.06	0.10
Malawi	iLiNS−DOSE (45)												
Mali	PROMIS CS (46)												
Zimbabwe	SHINE (HIV−) (47)												
Zimbabwe	SHINE (HIV+) (48)												
		**272**	**342**		**I² = 0.32, Tau² = 0.00**			**391**	**226**		**I² = 0.21, Tau² = 0.00**		
**Fixed**					**1.03 (0.97, 1.08)**						**1.06 (1.00, 1.12)**		
**Random**					**1.04 (0.96, 1.11)**						**1.05 (0.97, 1.13)**		

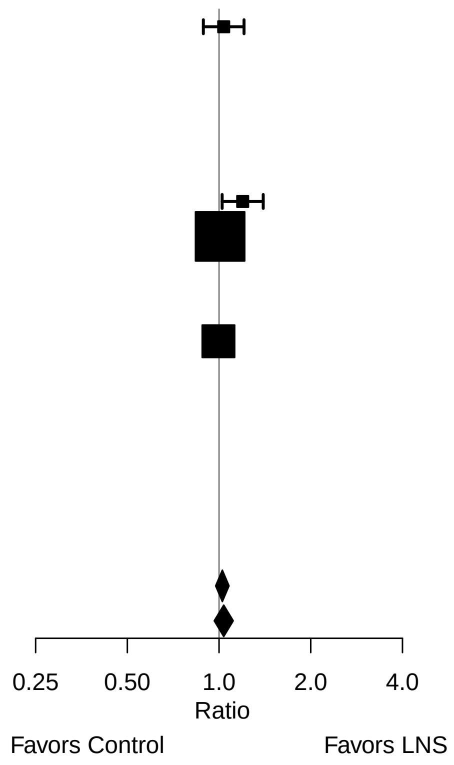

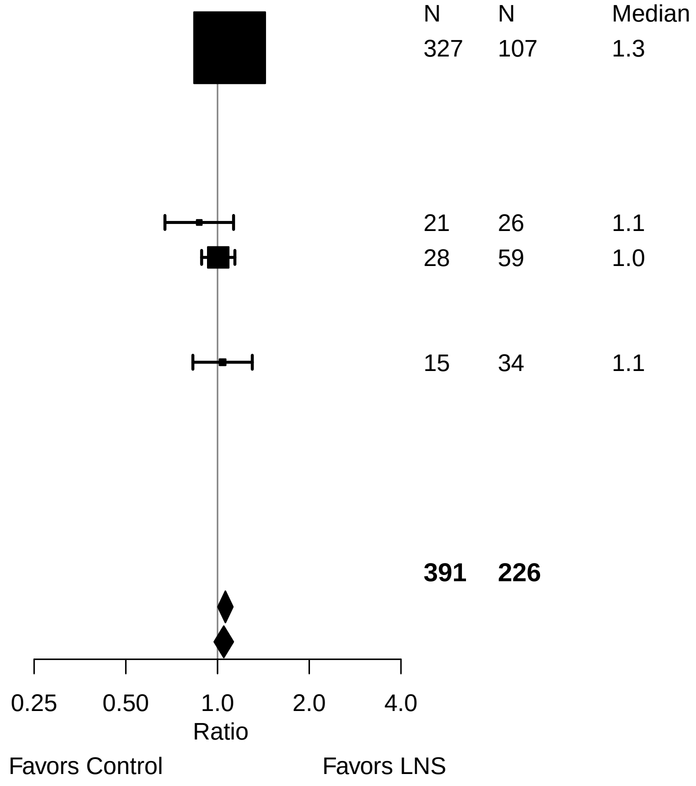

Supplemental figure 8R: Geometric mean ratio of retinol concentration

8R6: Stratified by Child baseline acute malnutrition

P−for−interaction = 0.075
Ratio of GMRs = 1.11 (0.99, 1.24)

		No						Yes					
		**LNS**	**Control**	**Control**	**GMR**	**Fixed**	**Random**	**LNS**	**Control**	**Control**	**GMR**	**Fixed**	**Random**
Country	Trial	N	N	Median	(95% CI)	W	W	N	N	Median	(95% CI)	W	W
Bangladesh	JiVitA−4 (35)	346	107	1.4	1.06 (0.99, 1.14)	0.35	0.35	86	32	1.3	1.17 (1.03, 1.32)	0.69	0.69
Bangladesh	RDNS (36)												
Bangladesh	WASH−B (37)												
Burkina Faso	iLiNS−Zinc (38)												
Burkina Faso	PROMIS CS (39)												
Ghana	GHANA (40)												
Ghana	iLiNS−DYAD−G (41)	90	182	1.0	1.01 (0.94, 1.08)	0.42	0.42	6	14	1.0	1.00 (0.81, 1.25)	0.21	0.21
Kenya	WASH−B (42)												
Madagascar	MAHAY (43)												
Malawi	iLiNS−DYAD−M (44)	58	145	1.1	1.00 (0.91, 1.09)	0.24	0.24	7	14	0.9	1.28 (0.92, 1.80)	0.09	0.09
Malawi	iLiNS−DOSE (45)												
Mali	PROMIS CS (46)												
Zimbabwe	SHINE (HIV−) (47)												
Zimbabwe	SHINE (HIV+) (48)												
		**494**	**434**		**I² = 0.00, Tau² = 0.00**			**99**	**60**		**I² = 0.00, Tau² = 0.00**		
**Fixed**					**1.02 (0.98, 1.07)**						**1.14 (1.03, 1.26)**		
**Random**					**1.02 (0.98, 1.07)**						**1.14 (1.03, 1.26)**		

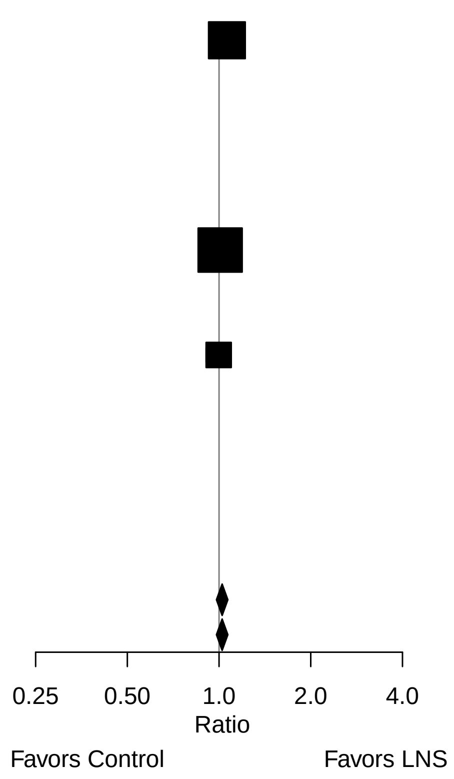

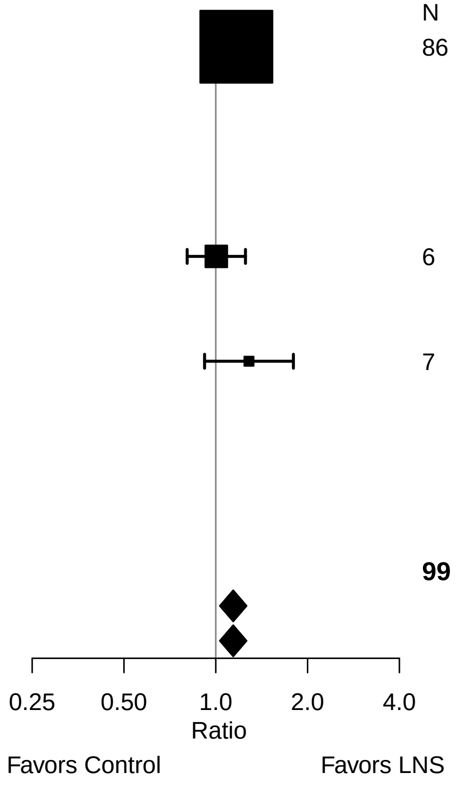

Supplemental figure 8R: Geometric mean ratio of retinol concentration

8R7: Stratified by Child baseline anemia (insufficient comparisons)

Supplemental figure 8R: Geometric mean ratio of retinol concentration

8R8: Stratified by Child high-dose vitamin A supplementation

P−for−interaction = 0.577
Ratio of GMRs = 0.98 (0.90, 1.06)

Country	Trial	Received VitA: LNS N	Control N	Control Median	GMR (95% CI)	Fixed W	Random W	Not treated: LNS N	Control N	Control Median	GMR (95% CI)	Fixed W	Random W
Bangladesh	JiVitA−4 (35)	136	36	1.4	1.10 (1.02, 1.19)	0.41	0.41	129	38	1.4	1.04 (0.93, 1.17)	0.28	0.28
Bangladesh	RDNS (36)												
Bangladesh	WASH−B (37)												
Burkina Faso	iLiNS−Zinc (38)												
Burkina Faso	PROMIS CS (39)												
Ghana	GHANA (40)	27	47	1.0	1.07 (0.90, 1.27)	0.08	0.08	36	24	1.2	1.00 (0.80, 1.25)	0.07	0.07
Ghana	iLiNS−DYAD−G (41)	71	143	1.0	1.00 (0.93, 1.08)	0.43	0.43	25	53	1.1	1.02 (0.91, 1.15)	0.26	0.26
Kenya	WASH−B (42)												
Madagascar	MAHAY (43)												
Malawi	iLiNS−DYAD−M (44)	16	42	1.0	1.01 (0.84, 1.20)	0.08	0.08	54	119	1.1	1.00 (0.91, 1.10)	0.39	0.39
Malawi	iLiNS−DOSE (45)												
Mali	PROMIS CS (46)												
Zimbabwe	SHINE (HIV−) (47)												
Zimbabwe	SHINE (HIV+) (48)												
		**250**	**268**		**I² = 0.00, Tau² = 0.00**			**244**	**234**		**I² = 0.00, Tau² = 0.00**		
**Fixed**					**1.05 (1.00, 1.10)**						**1.02 (0.96, 1.08)**		
**Random**					**1.05 (1.00, 1.10)**						**1.02 (0.96, 1.08)**		

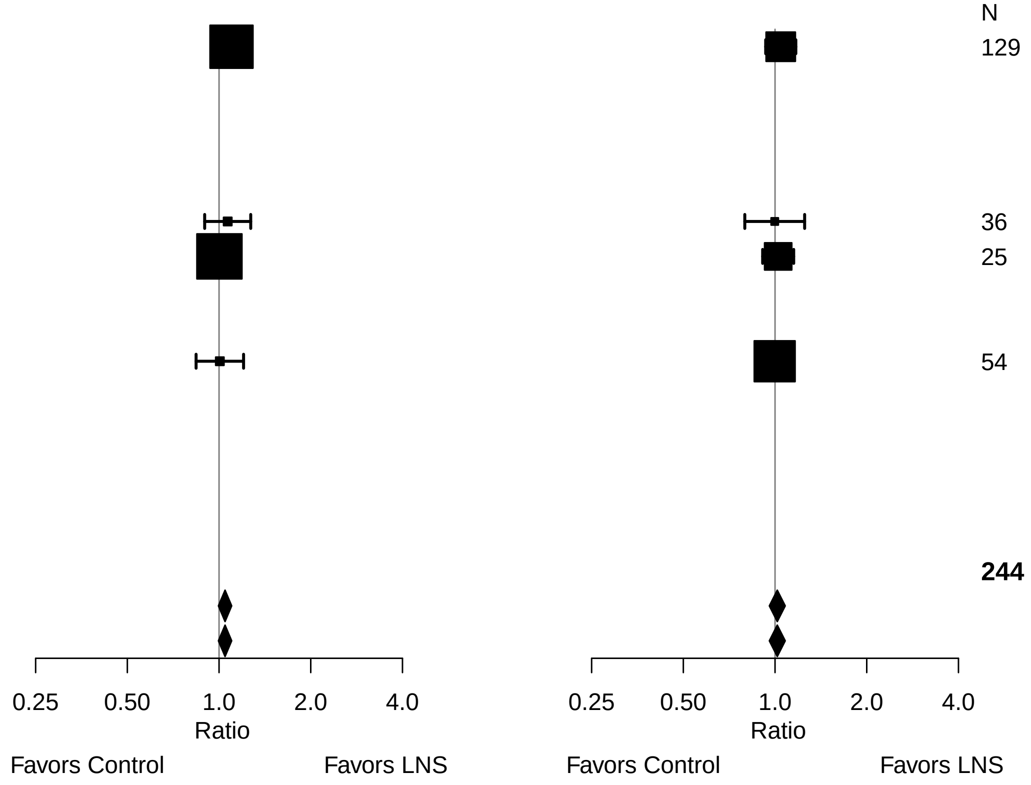

Supplemental figure 8R: Geometric mean ratio of retinol concentration

8R9: Stratified by Child inflammation

P−for−interaction = 0.481
Ratio of GMRs = 1.03 (0.95, 1.11)

		Not inflamed						High AGP or CRP					
Country	Trial	LNS N	Control N	Control Median	GMR (95% CI)	Fixed W	Random W	LNS N	Control N	Control Median	GMR (95% CI)	Fixed W	Random W
Bangladesh	JiVitA−4 (35)	177	46	1.4	1.05 (0.97, 1.15)	0.37	0.36	257	93	1.4	1.10 (1.02, 1.20)	0.43	0.42
Bangladesh	RDNS (36)												
Bangladesh	WASH−B (37)												
Burkina Faso	iLiNS−Zinc (38)												
Burkina Faso	PROMIS CS (39)												
Ghana	GHANA (40)	51	59	1.1	1.13 (0.98, 1.30)	0.13	0.14	12	15	1.0	0.86 (0.59, 1.26)	0.02	0.02
Ghana	iLiNS−DYAD−G (41)	43	118	1.0	0.99 (0.91, 1.07)	0.40	0.39	53	78	1.0	1.03 (0.93, 1.15)	0.27	0.27
Kenya	WASH−B (42)												
Madagascar	MAHAY (43)												
Malawi	iLiNS−DYAD−M (44)	20	61	1.0	0.98 (0.84, 1.15)	0.10	0.11	51	102	1.1	1.01 (0.91, 1.11)	0.28	0.29
Malawi	iLiNS−DOSE (45)												
Mali	PROMIS CS (46)												
Zimbabwe	SHINE (HIV−) (47)												
Zimbabwe	SHINE (HIV+) (48)												
		**291**	**284**		**I² = 0.02, Tau² = 0.00**			**373**	**288**		**I² = 0.06, Tau² = 0.00**		
**Fixed**					**1.03 (0.98, 1.08)**						**1.05 (1.00, 1.11)**		
**Random**					**1.03 (0.98, 1.09)**						**1.05 (0.99, 1.11)**		

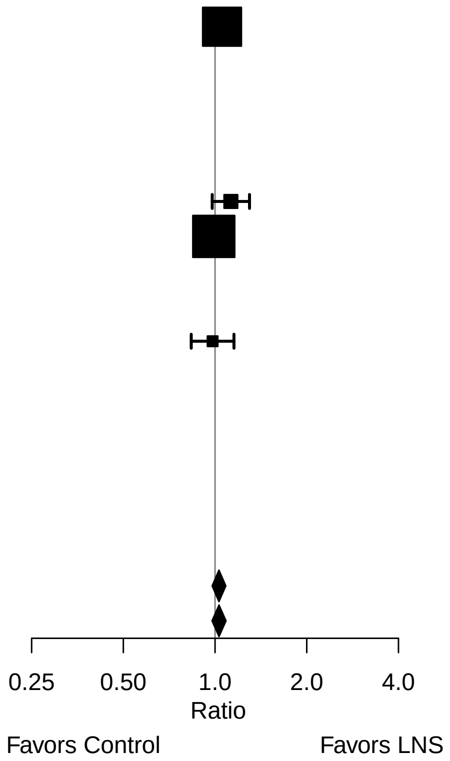

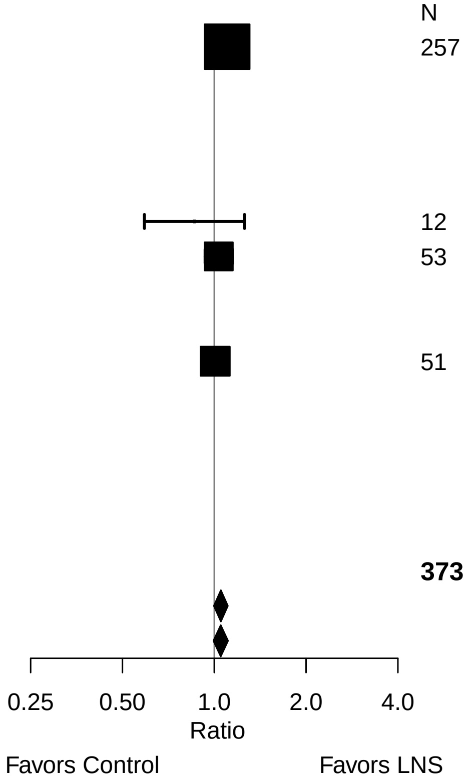

Supplemental figure 8S: Low vitamin A (retinol < 0.70 µmol/L) prevalence ratio

8S1: Stratified by Maternal BMI (insufficient comparisons)

Supplemental figure 8S: Low vitamin A (retinol < 0.70 µmol/L) prevalence ratio

8S2: Stratified by Maternal age (insufficient comparisons)

Supplemental figure 8S: Low vitamin A (retinol < 0.70 µmol/L) prevalence ratio

8S3: Stratified by Maternal education (insufficient comparisons)

Supplemental figure 8S: Low vitamin A (retinol < 0.70 µmol/L) prevalence ratio

8S4: Stratified by Child sex (insufficient comparisons)

Supplemental figure 8S: Low vitamin A (retinol < 0.70 µmol/L) prevalence ratio

8S5: Stratified by Child birth order (insufficient comparisons)

Supplemental figure 8S: Low vitamin A (retinol < 0.70 µmol/L) prevalence ratio

8S6: Stratified by Child baseline acute malnutrition (insufficient comparisons)

Supplemental figure 8S: Low vitamin A (retinol < 0.70 µmol/L) prevalence ratio

8S7: Stratified by Child baseline anemia (insufficient comparisons)

Supplemental figure 8S: Low vitamin A (retinol < 0.70 µmol/L) prevalence ratio

8S8: Stratified by Child high-dose vitamin A supplementation (insufficient comparisons)

Supplemental figure 8S: Low vitamin A (retinol < 0.70 µmol/L) prevalence ratio

8S9: Stratified by Child inflammation (insufficient comparisons)

Supplemental figure 8T: Low vitamin A (retinol < 0.70 µmol/L) prevalence difference

8T1: Stratified by Maternal BMI (insufficient comparisons)

Supplemental figure 8T: Low vitamin A (retinol < 0.70 µmol/L) prevalence difference

8T2: Stratified by Maternal age (insufficient comparisons)

Supplemental figure 8T: Low vitamin A (retinol < 0.70 µmol/L) prevalence difference

8T3: Stratified by Maternal education (insufficient comparisons)

Supplemental figure 8T: Low vitamin A (retinol < 0.70 µmol/L) prevalence difference

8T4: Stratified by Child sex (insufficient comparisons)

Supplemental figure 8T: Low vitamin A (retinol < 0.70 µmol/L) prevalence difference

8T5: Stratified by Child birth order (insufficient comparisons)

Supplemental figure 8T: Low vitamin A (retinol < 0.70 µmol/L) prevalence difference

8T6: Stratified by Child baseline acute malnutrition (insufficient comparisons)

Supplemental figure 8T: Low vitamin A (retinol < 0.70 µmol/L) prevalence difference

8T7: Stratified by Child baseline anemia (insufficient comparisons)

Supplemental figure 8T: Low vitamin A (retinol < 0.70 µmol/L) prevalence difference

8T8: Stratified by Child high-dose vitamin A supplementation (insufficient comparisons)

Supplemental figure 8T: Low vitamin A (retinol < 0.70 µmol/L) prevalence difference

8T9: Stratified by Child inflammation (insufficient comparisons)

Supplemental figure 8U: Marginal vitamin A (retinol < 1.05 µmol/L) prevalence ratio

8U1: Stratified by Maternal BMI (insufficient comparisons)

Supplemental figure 8U: Marginal vitamin A (retinol < 1.05 µmol/L) prevalence ratio

8U2: Stratified by Maternal age

P−for−interaction = 0.912
Ratio of PRs = 0.98 (0.73, 1.32)

		At least 25 y						Less than 25 y					
Country	Trial	LNS N	Control N	Control Prevalence	PR (95% CI)	Fixed W	Random W	LNS N	Control N	Control Prevalence	PR (95% CI)	Fixed W	Random W
Bangladesh	JiVitA−4 (35)	195	67	23.9	0.62 (0.35, 1.11)	0.11	0.14	237	71	15.5	0.90 (0.51, 1.59)	0.14	0.14
Bangladesh	RDNS (36)												
Bangladesh	WASH−B (37)												
Burkina Faso	iLiNS−Zinc (38)												
Burkina Faso	PROMIS CS (39)												
Ghana	GHANA (40)	47	47	53.2	0.96 (0.65, 1.41)	0.24	0.25	16	21	38.1	1.15 (0.53, 2.50)	0.07	0.07
Ghana	iLiNS−DYAD−G (41)	63	124	50.0	0.98 (0.72, 1.34)	0.39	0.35	33	72	68.1	0.85 (0.61, 1.18)	0.41	0.41
Kenya	WASH−B (42)												
Madagascar	MAHAY (43)												
Malawi	iLiNS−DYAD−M (44)	33	81	46.9	1.23 (0.84, 1.78)	0.26	0.27	38	82	50.0	1.16 (0.82, 1.64)	0.38	0.38
Malawi	iLiNS−DOSE (45)												
Mali	PROMIS CS (46)												
Zimbabwe	SHINE (HIV−) (47)												
Zimbabwe	SHINE (HIV+) (48)												
		**338**	**319**		**I² = 0.21, Tau² = 0.02**			**324**	**246**		**I² = 0.00, Tau² = 0.00**		
**Fixed**					**0.99 (0.81, 1.19)**						**0.98 (0.79, 1.22)**		
**Random**					**0.98 (0.77, 1.23)**						**0.98 (0.79, 1.22)**		

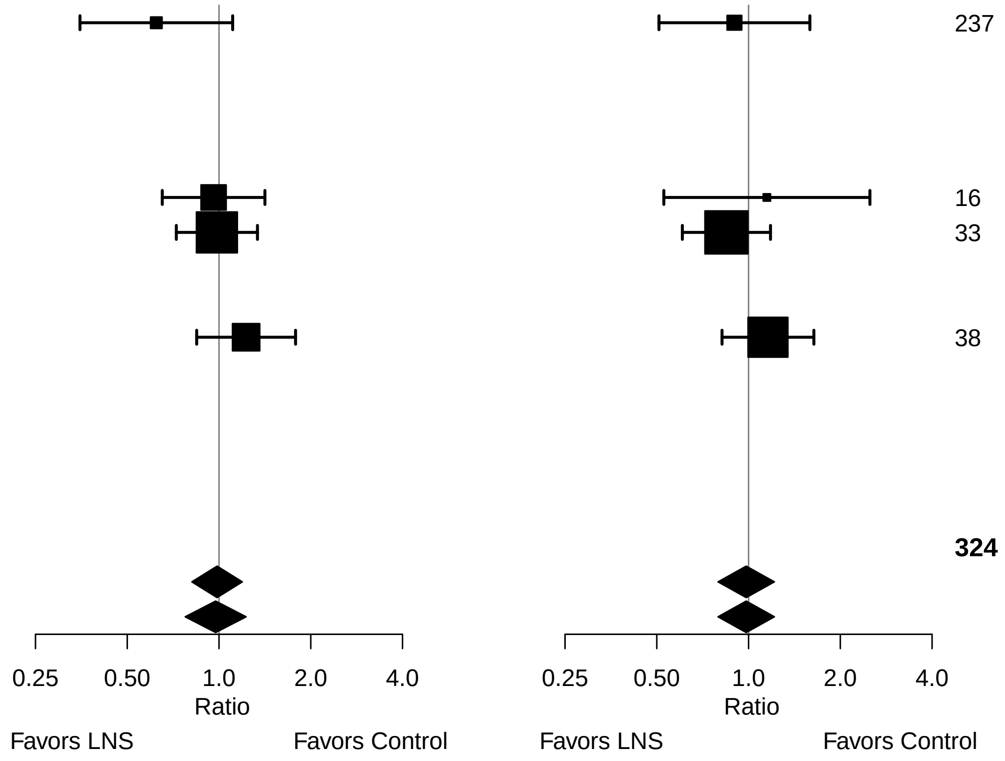

Supplemental figure 8U: Marginal vitamin A (retinol < 1.05 µmol/L) prevalence ratio

8U3: Stratified by Maternal education (insufficient comparisons)

Supplemental figure 8U: Marginal vitamin A (retinol < 1.05 µmol/L) prevalence ratio

8U4: Stratified by Child sex

P−for−interaction = 0.838
Ratio of PRs = 1.03 (0.77, 1.38)

		**Male**						**Female**					
Country	Trial	**LNS** N	**Control** N	**Control** Prevalence	**PR** (95% CI)	**Fixed** W	**Random** W	**LNS** N	**Control** N	**Control** Prevalence	**PR** (95% CI)	**Fixed** W	**Random** W
Bangladesh	JiVitA−4 (35)	222	67	17.9	1.03 (0.51, 2.10)	0.07	0.10	212	72	20.8	0.48 (0.31, 0.73)	0.25	0.25
Bangladesh	RDNS (36)												
Bangladesh	WASH−B (37)												
Burkina Faso	iLiNS−Zinc (38)												
Burkina Faso	PROMIS CS (39)												
Ghana	GHANA (40)	37	32	46.9	1.04 (0.63, 1.70)	0.14	0.18	26	42	50.0	1.00 (0.61, 1.63)	0.19	0.23
Ghana	iLiNS−DYAD−G (41)	62	93	66.7	0.75 (0.56, 1.00)	0.41	0.37	33	102	47.1	1.16 (0.80, 1.68)	0.33	0.27
Kenya	WASH−B (42)												
Madagascar	MAHAY (43)												
Malawi	iLiNS−DYAD−M (44)	39	80	55.0	1.21 (0.90, 1.63)	0.38	0.36	32	83	42.2	1.11 (0.71, 1.74)	0.23	0.25
Malawi	iLiNS−DOSE (45)												
Mali	PROMIS CS (46)												
Zimbabwe	SHINE (HIV−) (47)												
Zimbabwe	SHINE (HIV+) (48)												
		**360**	**272**		**I² = 0.44, Tau² = 0.02**			**303**	**299**		**I² = 0.74, Tau² = 0.13**		
**Fixed**					**0.96 (0.80, 1.16)**						**0.89 (0.72, 1.11)**		
**Random**					**0.97 (0.77, 1.23)**						**0.89 (0.59, 1.34)**		

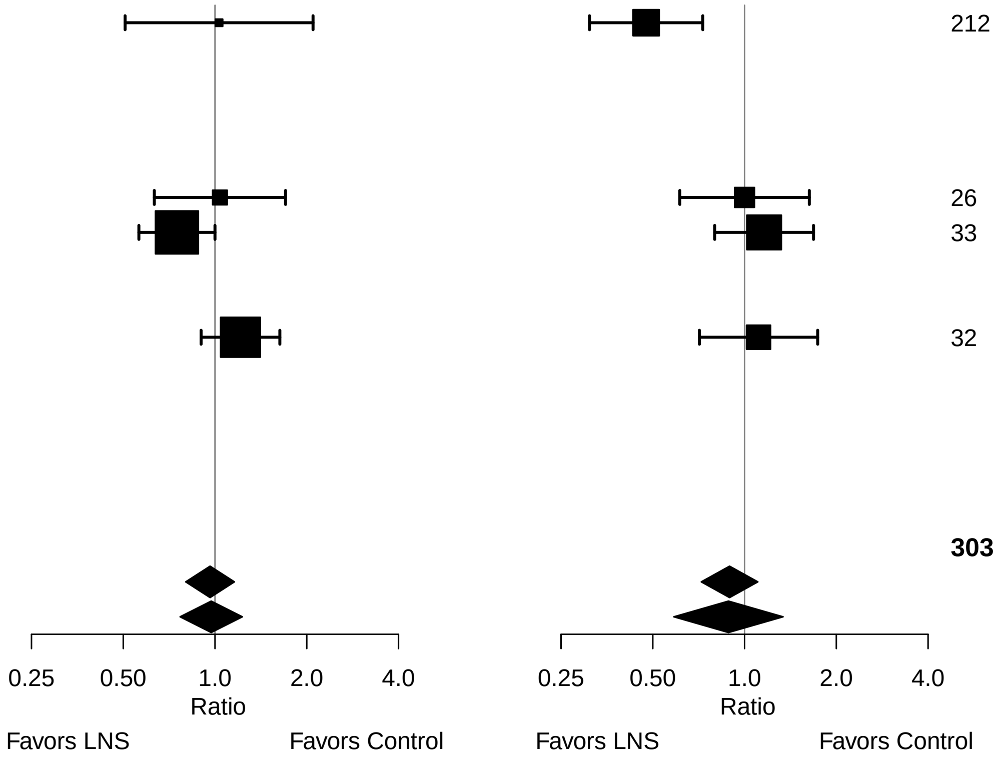

Supplemental figure 8U: Marginal vitamin A (retinol < 1.05 μmol/L) prevalence ratio

8U5: Stratified by Child birth order

P−for−interaction = 0.132
Ratio of PRs = 1.27 (0.93, 1.74)

		Later born						Firstborn					
		**LNS**	**Control**	**Control**	**PR**	**Fixed**	**Random**	**LNS**	**Control**	**Control**	**PR**	**Fixed**	**Random**
Country	Trial	N	N	Prevalence	(95% CI)	W	W	N	N	Prevalence	(95% CI)	W	W
Bangladesh	JiVitA−4 (35)	106	31	16.1	0.82 (0.29, 2.29)	0.03	0.05	327	107	20.6	0.71 (0.46, 1.10)	0.24	0.26
Bangladesh	RDNS (36)												
Bangladesh	WASH−B (37)												
Burkina Faso	iLiNS−Zinc (38)												
Burkina Faso	PROMIS CS (39)												
Ghana	GHANA (40)	42	45	51.1	0.79 (0.50, 1.26)	0.16	0.20	21	26	46.2	1.44 (0.86, 2.41)	0.17	0.21
Ghana	iLiNS−DYAD−G (41)	68	137	54.0	0.82 (0.60, 1.11)	0.36	0.35	28	59	62.7	1.14 (0.84, 1.55)	0.48	0.38
Kenya	WASH−B (42)												
Madagascar	MAHAY (43)												
Malawi	iLiNS−DYAD−M (44)	56	129	48.8	1.24 (0.94, 1.64)	0.45	0.40	15	34	47.1	0.99 (0.52, 1.90)	0.11	0.15
Malawi	iLiNS−DOSE (45)												
Mali	PROMIS CS (46)												
Zimbabwe	SHINE (HIV−) (47)												
Zimbabwe	SHINE (HIV+) (48)												
		**272**	**342**		**I² = 0.42, Tau² = 0.02**			**391**	**226**		**I² = 0.38, Tau² = 0.03**		
**Fixed**					**0.98 (0.82, 1.18)**						**1.05 (0.85, 1.29)**		
**Random**					**0.96 (0.76, 1.22)**						**1.04 (0.78, 1.38)**		

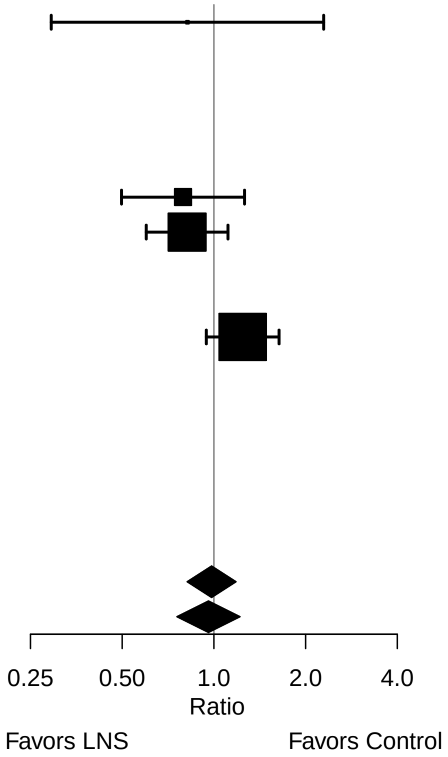

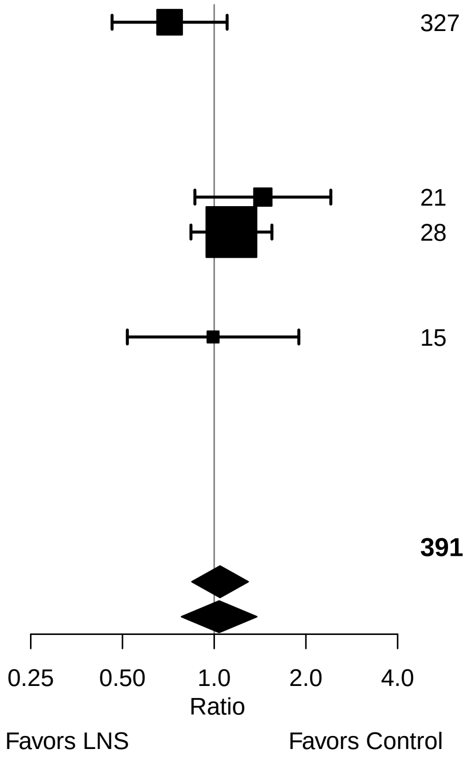

Supplemental figure 8U: Marginal vitamin A (retinol < 1.05 µmol/L) prevalence ratio

8U6: Stratified by Child baseline acute malnutrition (insufficient comparisons)

Supplemental figure 8U: Marginal vitamin A (retinol < 1.05 µmol/L) prevalence ratio

8U7: Stratified by Child baseline anemia (insufficient comparisons)

Supplemental figure 8U: Marginal vitamin A (retinol < 1.05 µmol/L) prevalence ratio

8U8: Stratified by Child high-dose vitamin A supplementation

P−for−interaction = 0.520
Ratio of PRs = 0.90 (0.64, 1.25)

		Received VitA						Not treated					
Country	Trial	LNS N	Control N	Control Prevalence	PR (95% CI)	Fixed W	Random W	LNS N	Control N	Control Prevalence	PR (95% CI)	Fixed W	Random W
Bangladesh	JiVitA−4 (35)	136	36	13.9	0.74 (0.33, 1.68)	0.05	0.05	129	38	18.4	0.67 (0.31, 1.44)	0.09	0.09
Bangladesh	RDNS (36)												
Bangladesh	WASH−B (37)												
Burkina Faso	iLiNS−Zinc (38)												
Burkina Faso	PROMIS CS (39)												
Ghana	GHANA (40)	27	47	53.2	1.11 (0.74, 1.68)	0.21	0.21	36	24	41.7	1.00 (0.54, 1.84)	0.15	0.15
Ghana	iLiNS−DYAD−G (41)	71	143	59.4	0.92 (0.72, 1.19)	0.56	0.55	25	53	49.1	0.90 (0.53, 1.51)	0.20	0.20
Kenya	WASH−B (42)												
Madagascar	MAHAY (43)												
Malawi	iLiNS−DYAD−M (44)	16	42	50.0	1.37 (0.88, 2.15)	0.18	0.18	54	119	47.1	1.14 (0.83, 1.56)	0.56	0.56
Malawi	iLiNS−DOSE (45)												
Mali	PROMIS CS (46)												
Zimbabwe	SHINE (HIV−) (47)												
Zimbabwe	SHINE (HIV+) (48)												
		**250**	**268**		**I² = 0.02, Tau² = 0.00**			**244**	**234**		**I² = 0.00, Tau² = 0.00**		
**Fixed**					**1.02 (0.84, 1.23)**						**1.01 (0.80, 1.28)**		
**Random**					**1.02 (0.84, 1.24)**						**1.01 (0.80, 1.28)**		

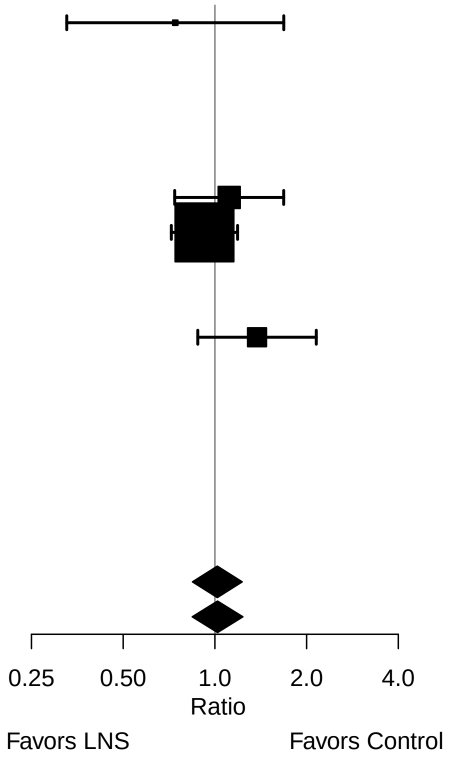

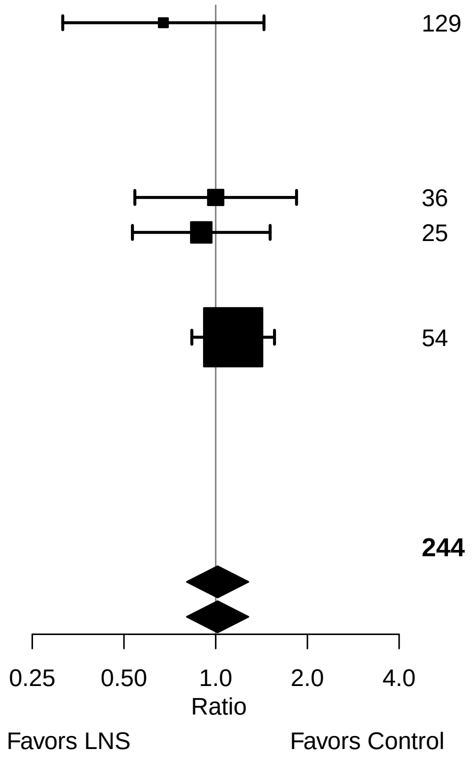

Supplemental figure 8U: Marginal vitamin A (retinol < 1.05 µmol/L) prevalence ratio

8U9: Stratified by Child inflammation

P−for−interaction = 0.775
Ratio of PRs = 0.96 (0.70, 1.30)

		Not inflamed						High AGP or CRP					
Country	Trial	LNS N	Control N	Control Prevalence	PR (95% CI)	Fixed W	Random W	LNS N	Control N	Control Prevalence	PR (95% CI)	Fixed W	Random W
Bangladesh	JiVitA−4 (35)	177	46	21.7	0.68 (0.42, 1.08)	0.21	0.30	257	93	18.3	0.77 (0.43, 1.36)	0.14	0.14
Bangladesh	RDNS (36)												
Bangladesh	WASH−B (37)												
Burkina Faso	iLiNS−Zinc (38)												
Burkina Faso	PROMIS CS (39)												
Ghana	GHANA (40)												
Ghana	iLiNS−DYAD−G (41)	43	118	55.9	0.87 (0.62, 1.23)	0.40	0.35	53	78	57.7	0.95 (0.70, 1.29)	0.47	0.47
Kenya	WASH−B (42)												
Madagascar	MAHAY (43)												
Malawi	iLiNS−DYAD−M (44)	20	61	52.5	1.43 (1.01, 2.02)	0.39	0.35	51	102	46.1	1.11 (0.79, 1.56)	0.39	0.39
Malawi	iLiNS−DOSE (45)												
Mali	PROMIS CS (46)												
Zimbabwe	SHINE (HIV−) (47)												
Zimbabwe	SHINE (HIV+) (48)												
		**240**	**225**		**I² = 0.73, Tau² = 0.10**			**361**	**273**		**I² = 0.00, Tau² = 0.00**		
**Fixed**					**1.00 (0.81, 1.24)**						**0.98 (0.79, 1.21)**		
**Random**					**0.96 (0.63, 1.48)**						**0.98 (0.79, 1.21)**		

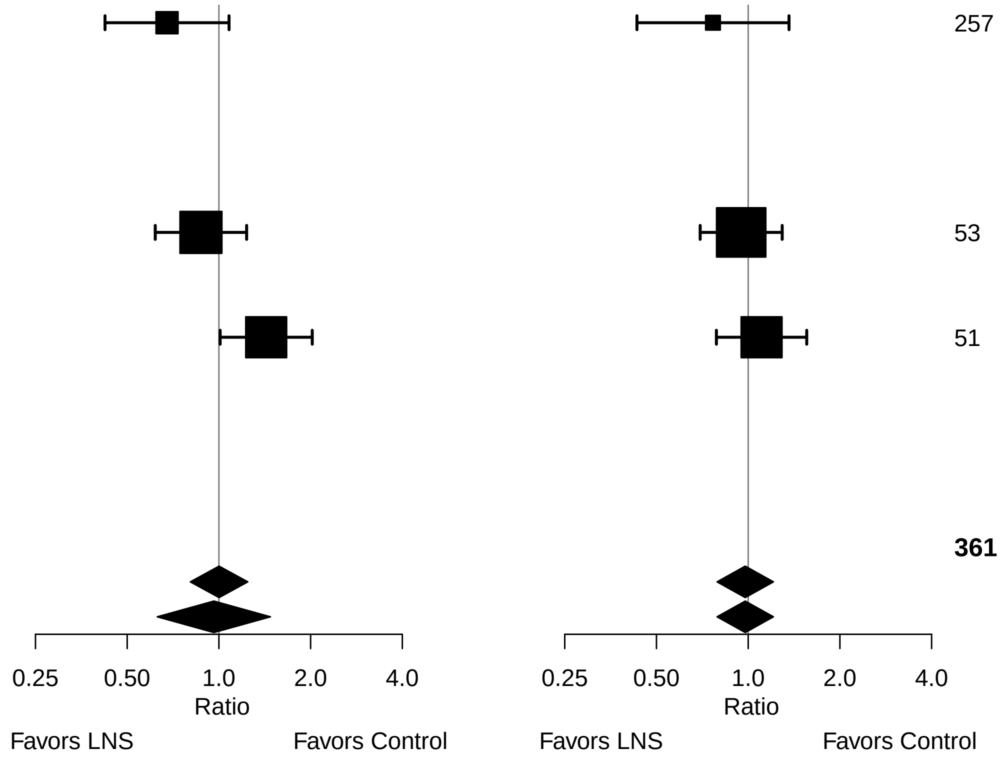

Supplemental figure 8V: Marginal vitamin A (retinol < 1.05 µmol/L) prevalence difference

8V1: Stratified by Maternal BMI (insufficient comparisons)

Supplemental figure 8V: Marginal vitamin A (retinol < 1.05 µmol/L) prevalence difference

8V2: Stratified by Maternal age

P−for−interaction = 0.659
Difference in PDs = 0.03 (−0.09, 0.14)

		At least 25 y						Less than 25 y					
Country	Trial	LNS N	Control N	Control Prevalence	PD (95% CI)	Fixed W	Random W	LNS N	Control N	Control Prevalence	PD (95% CI)	Fixed W	Random W
Bangladesh	JiVitA−4 (35)	195	67	23.9	−0.09 (−0.21, 0.03)	0.42	0.42	237	71	15.5	−0.02 (−0.10, 0.07)	0.69	0.69
Bangladesh	RDNS (36)												
Bangladesh	WASH−B (37)												
Burkina Faso	iLiNS−Zinc (38)												
Burkina Faso	PROMIS CS (39)												
Ghana	GHANA (40)	47	47	53.2	−0.02 (−0.23, 0.18)	0.15	0.15	16	21	38.1	0.06 (−0.27, 0.38)	0.05	0.05
Ghana	iLiNS−DYAD−G (41)	63	124	50.0	−0.01 (−0.16, 0.14)	0.27	0.27	33	72	68.1	−0.10 (−0.30, 0.09)	0.13	0.13
Kenya	WASH−B (42)												
Madagascar	MAHAY (43)												
Malawi	iLiNS−DYAD−M (44)	33	81	46.9	0.11 (−0.10, 0.31)	0.15	0.15	38	82	50.0	0.08 (−0.11, 0.27)	0.14	0.14
Malawi	iLiNS−DOSE (45)												
Mali	PROMIS CS (46)												
Zimbabwe	SHINE (HIV−) (47)												
Zimbabwe	SHINE (HIV+) (48)												
		**338**	**319**		**I² = 0.00, Tau² = 0.00**			**324**	**246**		**I² = 0.00, Tau² = 0.00**		
**Fixed**					**−0.03 (−0.11, 0.05)**						**−0.01 (−0.08, 0.06)**		
**Random**					**−0.03 (−0.11, 0.05)**						**−0.01 (−0.08, 0.06)**		

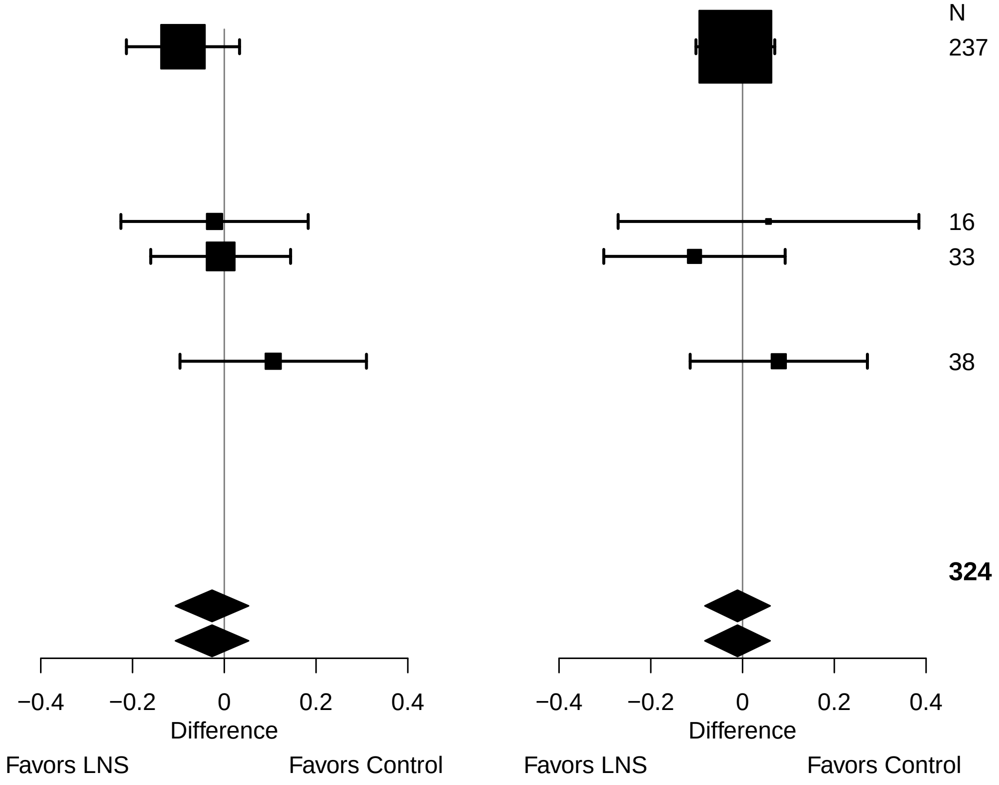

Supplemental figure 8V: Marginal vitamin A (retinol < 1.05 µmol/L) prevalence difference

8V3: Stratified by Maternal education (insufficient comparisons)

Supplemental figure 8V: Marginal vitamin A (retinol < 1.05 µmol/L) prevalence difference

8V4: Stratified by Child sex

P−for−interaction = 0.578
Difference in PDs = −0.03 (−0.14, 0.08)

Country	Trial	Male: LNS N	Male: Control N	Male: Control Prevalence	Male: PD (95% CI)	Male: Fixed W	Male: Random W	Female: LNS N	Female: Control N	Female: Control Prevalence	Female: PD (95% CI)	Female: Fixed W	Female: Random W
Bangladesh	JiVitA−4 (35)	222	67	17.9	0.01 (−0.12, 0.13)	0.42	0.33	212	72	20.8	−0.11 (−0.17, −0.05)	0.83	0.58
Bangladesh	RDNS (36)												
Bangladesh	WASH−B (37)												
Burkina Faso	iLiNS−Zinc (38)												
Burkina Faso	PROMIS CS (39)												
Ghana	GHANA (40)	37	32	46.9	0.02 (−0.22, 0.26)	0.12	0.17	26	42	50.0	0.00 (−0.25, 0.25)	0.04	0.11
Ghana	iLiNS−DYAD−G (41)	62	93	66.7	−0.17 (−0.32, −0.01)	0.28	0.28	33	102	47.1	0.07 (−0.12, 0.27)	0.07	0.16
Kenya	WASH−B (42)												
Madagascar	MAHAY (43)												
Malawi	iLiNS−DYAD−M (44)	39	80	55.0	0.12 (−0.07, 0.31)	0.19	0.23	32	83	42.2	0.05 (−0.16, 0.25)	0.06	0.15
Malawi	iLiNS−DOSE (45)												
Mali	PROMIS CS (46)												
Zimbabwe	SHINE (HIV−) (47)												
Zimbabwe	SHINE (HIV+) (48)												
		**360**	**272**		**I² = 0.47, Tau² = 0.01**			**303**	**299**		**I² = 0.43, Tau² = 0.00**		
**Fixed**					**−0.02 (−0.10, 0.06)**						**−0.08 (−0.13, −0.03)**		
**Random**					**−0.02 (−0.13, 0.10)**						**−0.04 (−0.13, 0.04)**		

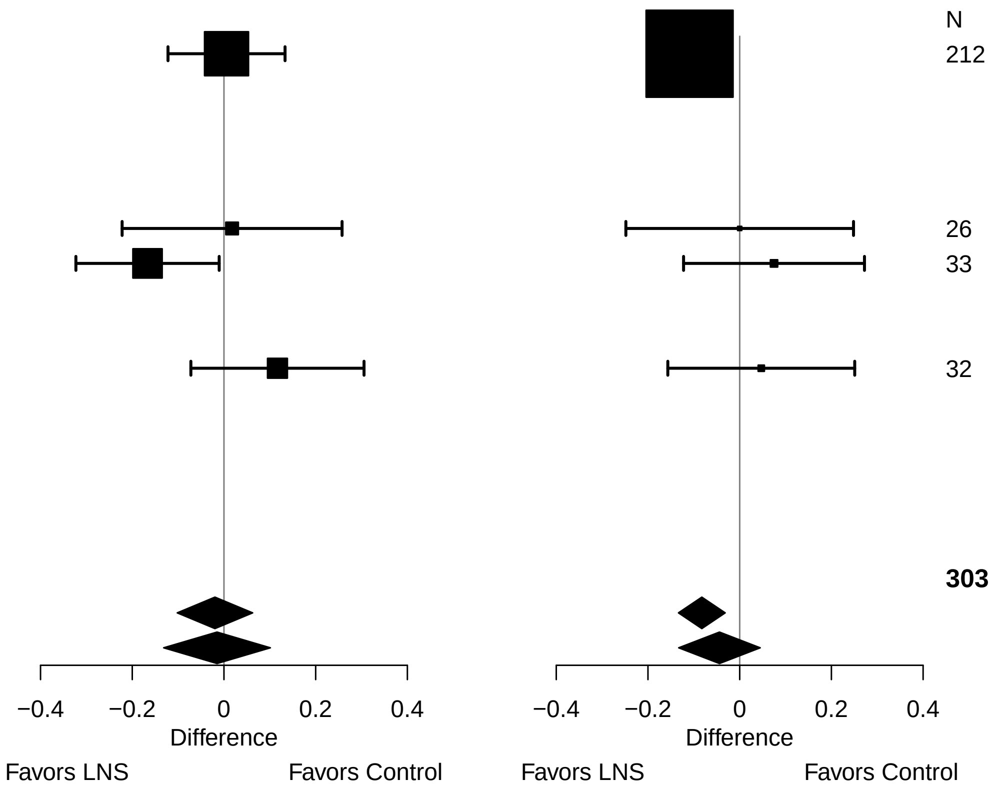

Supplemental figure 8V: Marginal vitamin A (retinol < 1.05 µmol/L) prevalence difference

8V5: Stratified by Child birth order

P−for−interaction = 0.432
Difference in PDs = 0.05 (−0.07, 0.17)

		Later born						Firstborn					
		**LNS**	**Control**	**Control**	**PD**	**Fixed**	**Random**	**LNS**	**Control**	**Control**	**PD**	**Fixed**	**Random**
Country	Trial	N	N	Prevalence	(95% CI)	W	W	N	N	Prevalence	(95% CI)	W	W
Bangladesh	JiVitA−4 (35)	106	31	16.1	−0.03 (−0.19, 0.13)	0.26	0.26	327	107	20.6	−0.06 (−0.14, 0.02)	0.77	0.60
Bangladesh	RDNS (36)												
Bangladesh	WASH−B (37)												
Burkina Faso	iLiNS−Zinc (38)												
Burkina Faso	PROMIS CS (39)												
Ghana	GHANA (40)	42	45	51.1	−0.11 (−0.32, 0.10)	0.15	0.18	21	26	46.2	0.21 (−0.08, 0.49)	0.06	0.11
Ghana	iLiNS−DYAD−G (41)	68	137	54.0	−0.10 (−0.24, 0.05)	0.31	0.29	28	59	62.7	0.09 (−0.13, 0.30)	0.11	0.19
Kenya	WASH−B (42)												
Madagascar	MAHAY (43)												
Malawi	iLiNS−DYAD−M (44)	56	129	48.8	0.12 (−0.04, 0.28)	0.27	0.27	15	34	47.1	0.00 (−0.31, 0.31)	0.06	0.10
Malawi	iLiNS−DOSE (45)												
Mali	PROMIS CS (46)												
Zimbabwe	SHINE (HIV−) (47)												
Zimbabwe	SHINE (HIV+) (48)												
		**272**	**342**		**I² = 0.38, Tau² = 0.00**			**391**	**226**		**I² = 0.28, Tau² = 0.00**		
**Fixed**					**−0.02 (−0.10, 0.06)**						**−0.02 (−0.09, 0.05)**		
**Random**					**−0.02 (−0.13, 0.08)**						**0.00 (−0.10, 0.11)**		

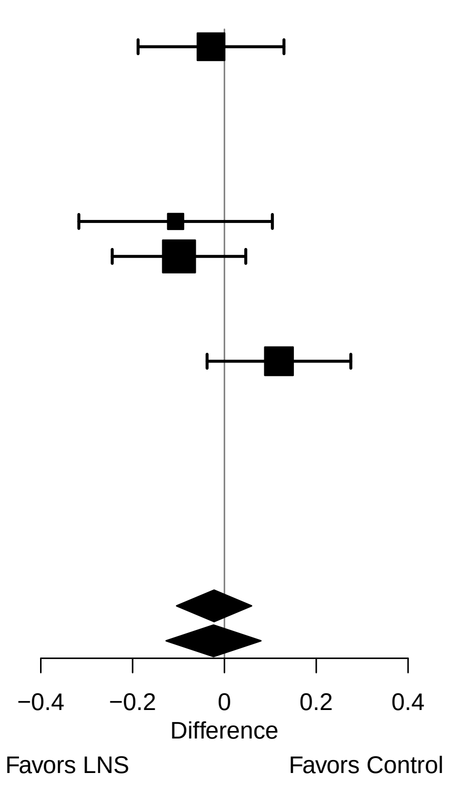

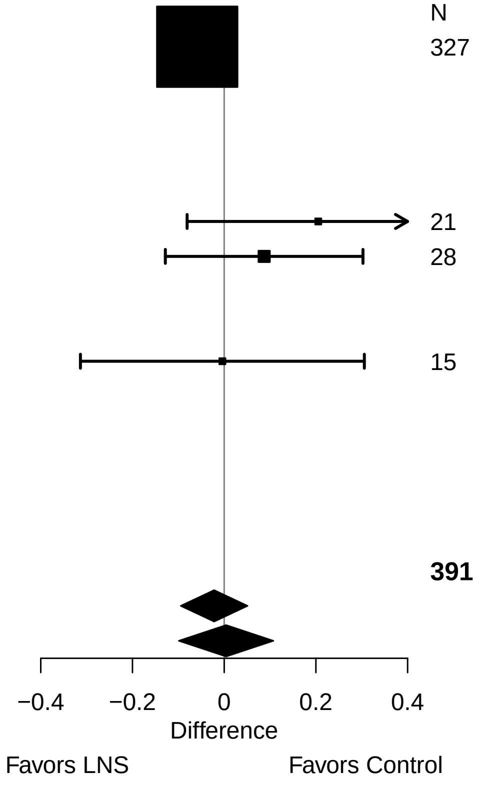

Supplemental figure 8V: Marginal vitamin A (retinol < 1.05 µmol/L) prevalence difference

8V6: Stratified by Child baseline acute malnutrition (insufficient comparisons)

Supplemental figure 8V: Marginal vitamin A (retinol < 1.05 µmol/L) prevalence difference

8V7: Stratified by Child baseline anemia (insufficient comparisons)

Supplemental figure 8V: Marginal vitamin A (retinol < 1.05 µmol/L) prevalence difference

8V8: Stratified by Child high-dose vitamin A supplementation

P−for−interaction = 0.534
Difference in PDs = −0.04 (−0.17, 0.09)

		Received VitA						Not treated					
Country	Trial	LNS N	Control N	Control Prevalence	PD (95% CI)	Fixed W	Random W	LNS N	Control N	Control Prevalence	PD (95% CI)	Fixed W	Random W
Bangladesh	JiVitA−4 (35)	136	36	13.9	−0.04 (−0.14, 0.07)	0.54	0.54	129	38	18.4	−0.06 (−0.19, 0.07)	0.46	0.46
Bangladesh	RDNS (36)												
Bangladesh	WASH−B (37)												
Burkina Faso	iLiNS−Zinc (38)												
Burkina Faso	PROMIS CS (39)												
Ghana	GHANA (40)	27	47	53.2	0.06 (−0.18, 0.30)	0.10	0.10	36	24	41.7	0.00 (−0.26, 0.26)	0.11	0.11
Ghana	iLiNS−DYAD−G (41)	71	143	59.4	−0.05 (−0.19, 0.10)	0.29	0.29	25	53	49.1	−0.05 (−0.29, 0.19)	0.13	0.13
Kenya	WASH−B (42)												
Madagascar	MAHAY (43)												
Malawi	iLiNS−DYAD−M (44)	16	42	50.0	0.19 (−0.10, 0.47)	0.07	0.07	54	119	47.1	0.07 (−0.09, 0.23)	0.29	0.29
Malawi	iLiNS−DOSE (45)												
Mali	PROMIS CS (46)												
Zimbabwe	SHINE (HIV−) (47)												
Zimbabwe	SHINE (HIV+) (48)												
		**250**	**268**		**I² = 0.00, Tau² = 0.00**			**244**	**234**		**I² = 0.00, Tau² = 0.00**		
**Fixed**					**−0.01 (−0.09, 0.06)**						**−0.02 (−0.10, 0.07)**		
**Random**					**−0.01 (−0.09, 0.06)**						**−0.02 (−0.10, 0.07)**		

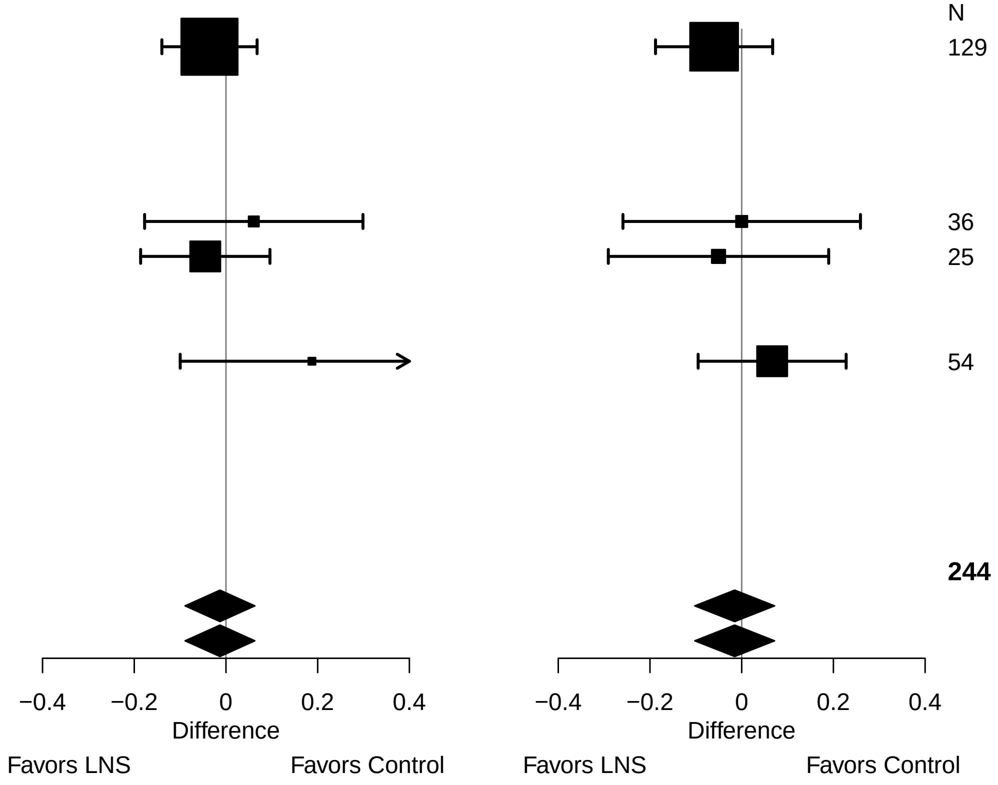

Supplemental figure 8V: Marginal vitamin A (retinol < 1.05 μmol/L) prevalence difference

8V9: Stratified by Child inflammation

P−for−interaction = 0.927
Difference in PDs = −0.01 (−0.13, 0.12)

Country	Trial	LNS N	Control N	Control Prevalence	Not inflamed PD (95% CI)	Fixed W	Random W	LNS N	Control N	Control Prevalence	High AGP or CRP PD (95% CI)	Fixed W	Random W
Bangladesh	JiVitA−4 (35)	177	46	21.7	−0.07 (−0.16, 0.02)	0.72	0.43	257	93	18.3	−0.04 (−0.14, 0.06)	0.60	0.60
Bangladesh	RDNS (36)												
Bangladesh	WASH−B (37)												
Burkina Faso	iLiNS−Zinc (38)												
Burkina Faso	PROMIS CS (39)												
Ghana	GHANA (40)												
Ghana	iLiNS−DYAD−G (41)	43	118	55.9	−0.07 (−0.25, 0.10)	0.19	0.33	53	78	57.7	−0.03 (−0.20, 0.14)	0.19	0.19
Kenya	WASH−B (42)												
Madagascar	MAHAY (43)												
Malawi	iLiNS−DYAD−M (44)	20	61	52.5	0.23 (−0.02, 0.47)	0.09	0.25	51	102	46.1	0.05 (−0.12, 0.22)	0.21	0.21
Malawi	iLiNS−DOSE (45)												
Mali	PROMIS CS (46)												
Zimbabwe	SHINE (HIV−) (47)												
Zimbabwe	SHINE (HIV+) (48)												
		**240**	**225**		**I^2 = 0.60, Tau^2 = 0.02**			**361**	**273**		**I^2 = 0.00, Tau^2 = 0.00**		
**Fixed**					**−0.04 (−0.12, 0.03)**						**−0.02 (−0.10, 0.06)**		
**Random**					**0.00 (−0.17, 0.18)**						**−0.02 (−0.10, 0.06)**		

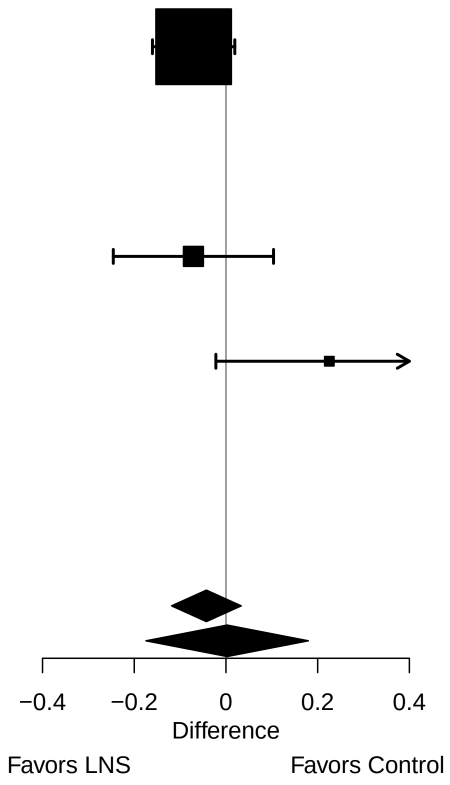

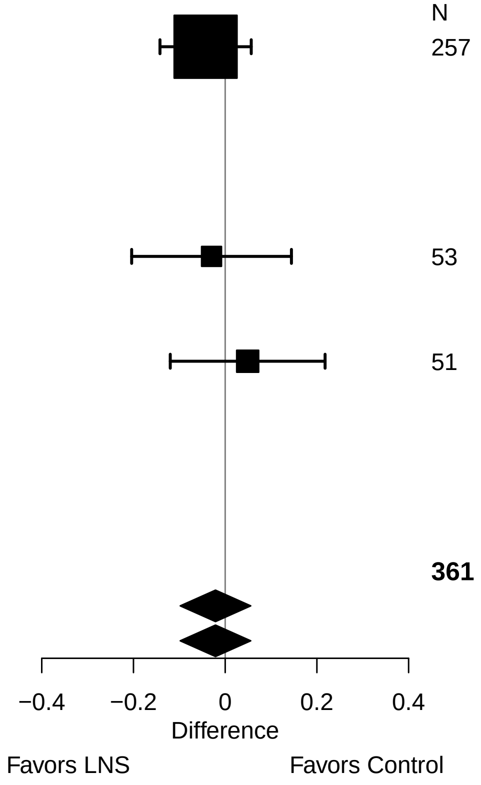

Supplemental figure 8W: Geometric mean ratio of retinol binding protein concentration

8W1: Stratified by Maternal BMI

P−for−interaction = 0.572
Ratio of GMRs = 0.99 (0.95, 1.03)

		At least 20 kg/m²						Less than 20 kg/m²					
		**LNS**	**Control**	**Control**	**GMR**	**Fixed**	**Random**	**LNS**	**Control**	**Control**	**GMR**	**Fixed**	**Random**
Country	Trial	N	N	Median	(95% CI)	W	W	N	N	Median	(95% CI)	W	W
Bangladesh	JiVitA−4 (35)												
Bangladesh	RDNS (36)	228	102	1.1	1.07 (1.00, 1.15)	0.21	0.24	300	160	1.2	0.99 (0.95, 1.03)	0.44	0.28
Bangladesh	WASH−B (37)	93	97	1.2	1.01 (0.93, 1.09)	0.16	0.20	119	81	1.1	1.09 (1.03, 1.15)	0.26	0.26
Burkina Faso	iLiNS−Zinc (38)	209	55	1.1	1.08 (1.02, 1.15)	0.31	0.28	106	41	1.1	1.10 (1.03, 1.18)	0.16	0.23
Burkina Faso	PROMIS CS (39)												
Ghana	GHANA (40)												
Ghana	iLiNS−DYAD−G (41)												
Kenya	WASH−B (42)	223	200	1.0	1.14 (1.08, 1.21)	0.32	0.28	64	51	1.0	1.14 (1.06, 1.23)	0.14	0.23
Madagascar	MAHAY (43)												
Malawi	iLiNS−DYAD−M (44)												
Malawi	iLiNS−DOSE (45)												
Mali	PROMIS CS (46)												
Zimbabwe	SHINE (HIV−) (47)												
Zimbabwe	SHINE (HIV+) (48)												
		**753**	**454**		**I² = 0.52, Tau² = 0.00**			**589**	**333**		**I² = 0.83, Tau² = 0.00**		
**Fixed**					**1.09 (1.05, 1.12)**						**1.05 (1.02, 1.08)**		
**Random**					**1.08 (1.03, 1.13)**						**1.07 (1.01, 1.15)**		

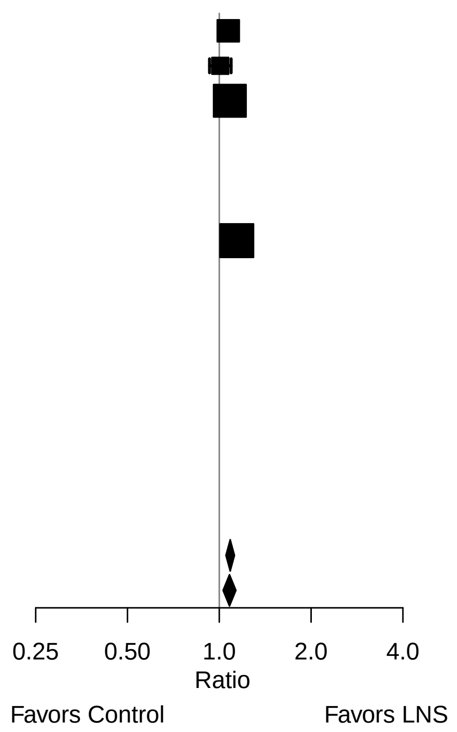

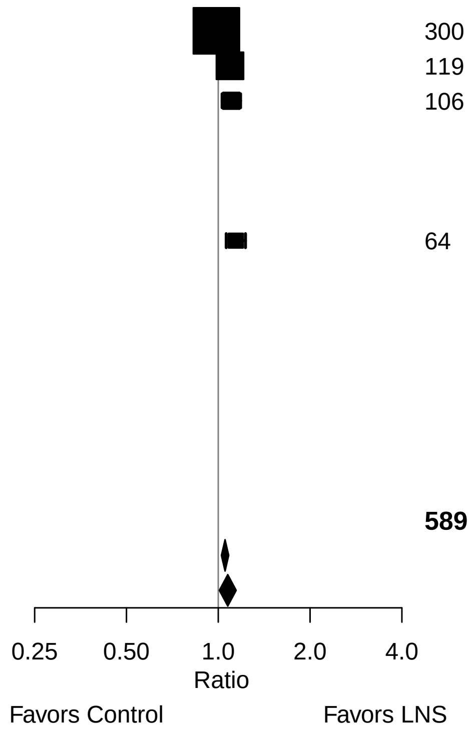

Supplemental figure 8W: Geometric mean ratio of retinol binding protein concentration

8W2: Stratified by Maternal age

P−for−interaction = 0.657
Ratio of GMRs = 0.99 (0.95, 1.03)

		At least 25 y						Less than 25 y					
Country	Trial	LNS N	Control N	Control Median	GMR (95% CI)	Fixed W	Random W	LNS N	Control N	Control Median	GMR (95% CI)	Fixed W	Random W
Bangladesh	JiVitA−4 (35)												
Bangladesh	RDNS (36)	152	81	1.2	1.07 (1.00, 1.15)	0.24	0.23	398	191	1.2	1.00 (0.96, 1.05)	0.43	0.28
Bangladesh	WASH−B (37)	79	80	1.1	1.02 (0.94, 1.11)	0.18	0.19	132	98	1.2	1.06 (0.99, 1.13)	0.20	0.22
Burkina Faso	iLiNS−Zinc (38)	178	50	1.0	1.08 (1.00, 1.17)	0.17	0.19	137	46	1.1	1.09 (1.01, 1.18)	0.15	0.19
Burkina Faso	PROMIS CS (39)												
Ghana	GHANA (40)												
Ghana	iLiNS−DYAD−G (41)												
Kenya	WASH−B (42)	178	149	1.0	1.13 (1.07, 1.20)	0.35	0.30	116	109	0.9	1.15 (1.06, 1.24)	0.16	0.20
Madagascar	MAHAY (43)	47	25	1.1	0.99 (0.87, 1.13)	0.06	0.09	36	26	1.1	1.11 (0.98, 1.26)	0.06	0.11
Malawi	iLiNS−DYAD−M (44)												
Malawi	iLiNS−DOSE (45)												
Mali	PROMIS CS (46)												
Zimbabwe	SHINE (HIV−) (47)												
Zimbabwe	SHINE (HIV+) (48)												
		**634**	**385**		**I² = 0.34, Tau² = 0.00**			**819**	**470**		**I² = 0.65, Tau² = 0.00**		
**Fixed**					**1.08 (1.04, 1.12)**						**1.05 (1.02, 1.09)**		
**Random**					**1.07 (1.03, 1.12)**						**1.07 (1.02, 1.12)**		

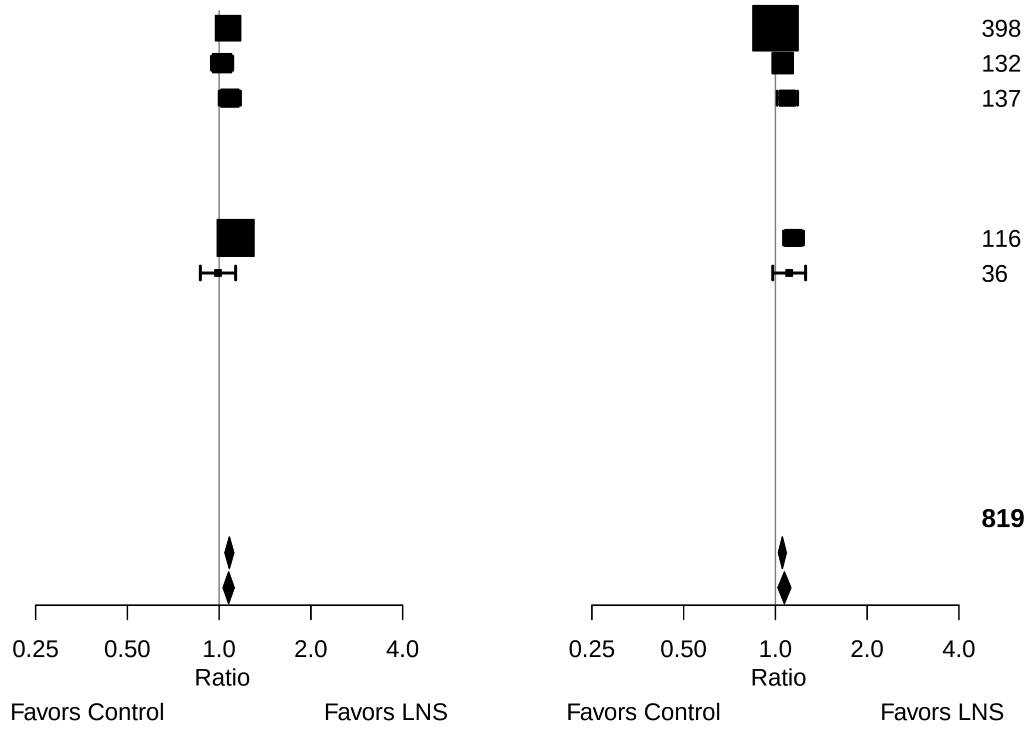

Supplemental figure 8W: Geometric mean ratio of retinol binding protein concentration

8W3: Stratified by Maternal education

P−for−interaction = 0.793
Ratio of GMRs = 1.01 (0.95, 1.07)

		**Primary or greater**						**Incomplete or no formal**					
Country	Trial	**LNS** N	**Control** N	**Control** Median	**GMR** (95% CI)	**Fixed** W	**Random** W	**LNS** N	**Control** N	**Control** Median	**GMR** (95% CI)	**Fixed** W	**Random** W
Bangladesh	JiVitA−4 (35)												
Bangladesh	RDNS (36)	406	195	1.2	1.00 (0.95, 1.06)	0.43	0.33	144	77	1.2	1.06 (0.98, 1.15)	0.26	0.26
Bangladesh	WASH−B (37)	149	139	1.2	1.05 (0.98, 1.12)	0.28	0.29	63	39	1.1	1.04 (0.93, 1.15)	0.17	0.19
Burkina Faso	iLiNS−Zinc (38)												
Burkina Faso	PROMIS CS (39)												
Ghana	GHANA (40)												
Ghana	iLiNS−DYAD−G (41)												
Kenya	WASH−B (42)	143	117	1.0	1.13 (1.05, 1.21)	0.25	0.28	154	142	0.9	1.14 (1.07, 1.22)	0.39	0.35
Madagascar	MAHAY (43)	16	16	1.1	1.15 (0.97, 1.37)	0.04	0.09	67	35	1.1	1.02 (0.93, 1.13)	0.18	0.20
Malawi	iLiNS−DYAD−M (44)												
Malawi	iLiNS−DOSE (45)												
Mali	PROMIS CS (46)												
Zimbabwe	SHINE (HIV−) (47)												
Zimbabwe	SHINE (HIV+) (48)												
		**714**	**467**		**I^2 = 0.64, Tau^2 = 0.00**			**428**	**293**		**I^2 = 0.33, Tau^2 = 0.00**		
**Fixed**					**1.05 (1.02, 1.09)**						**1.08 (1.04, 1.13)**		
**Random**					**1.06 (1.00, 1.13)**						**1.08 (1.02, 1.13)**		

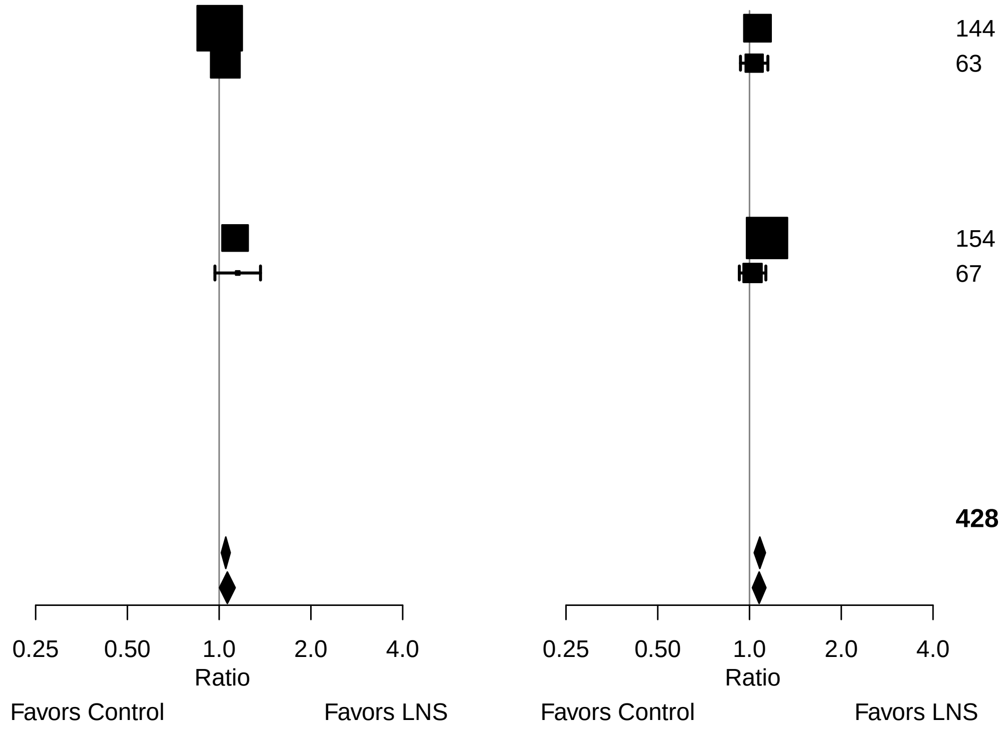

Supplemental figure 8W: Geometric mean ratio of retinol binding protein concentration

8W4: Stratified by Child sex

P−for−interaction = 0.100
Ratio of GMRs = 1.03 (0.99, 1.07)

Country	Trial	Male LNS N	Male Control N	Male Control Median	Male GMR (95% CI)	Male Fixed W	Male Random W	Female LNS N	Female Control N	Female Control Median	Female GMR (95% CI)	Female Fixed W	Female Random W
Bangladesh	JiVitA−4 (35)												
Bangladesh	RDNS (36)	266	131	1.2	1.00 (0.94, 1.07)	0.28	0.24	281	141	1.2	1.04 (0.99, 1.08)	0.41	0.33
Bangladesh	WASH−B (37)	105	85	1.2	1.00 (0.94, 1.07)	0.24	0.23	107	93	1.1	1.08 (1.02, 1.15)	0.18	0.20
Burkina Faso	iLiNS−Zinc (38)	150	49	1.0	1.07 (0.98, 1.16)	0.15	0.18	165	47	1.1	1.11 (1.04, 1.18)	0.19	0.20
Burkina Faso	PROMIS CS (39)												
Ghana	GHANA (40)												
Ghana	iLiNS−DYAD−G (41)												
Kenya	WASH−B (42)	146	125	0.9	1.14 (1.07, 1.22)	0.27	0.24	152	134	1.0	1.13 (1.06, 1.21)	0.17	0.19
Madagascar	MAHAY (43)	34	22	1.1	1.03 (0.91, 1.18)	0.07	0.11	49	29	1.1	1.06 (0.94, 1.19)	0.05	0.07
Malawi	iLiNS−DYAD−M (44)												
Malawi	iLiNS−DOSE (45)												
Mali	PROMIS CS (46)												
Zimbabwe	SHINE (HIV−) (47)												
Zimbabwe	SHINE (HIV+) (48)												
		**701**	**412**		**I^2 = 0.63, Tau^2 = 0.00**			**754**	**444**		**I^2 = 0.35, Tau^2 = 0.00**		
**Fixed**					**1.05 (1.02, 1.09)**						**1.07 (1.05, 1.10)**		
**Random**					**1.05 (1.00, 1.11)**						**1.08 (1.04, 1.11)**		

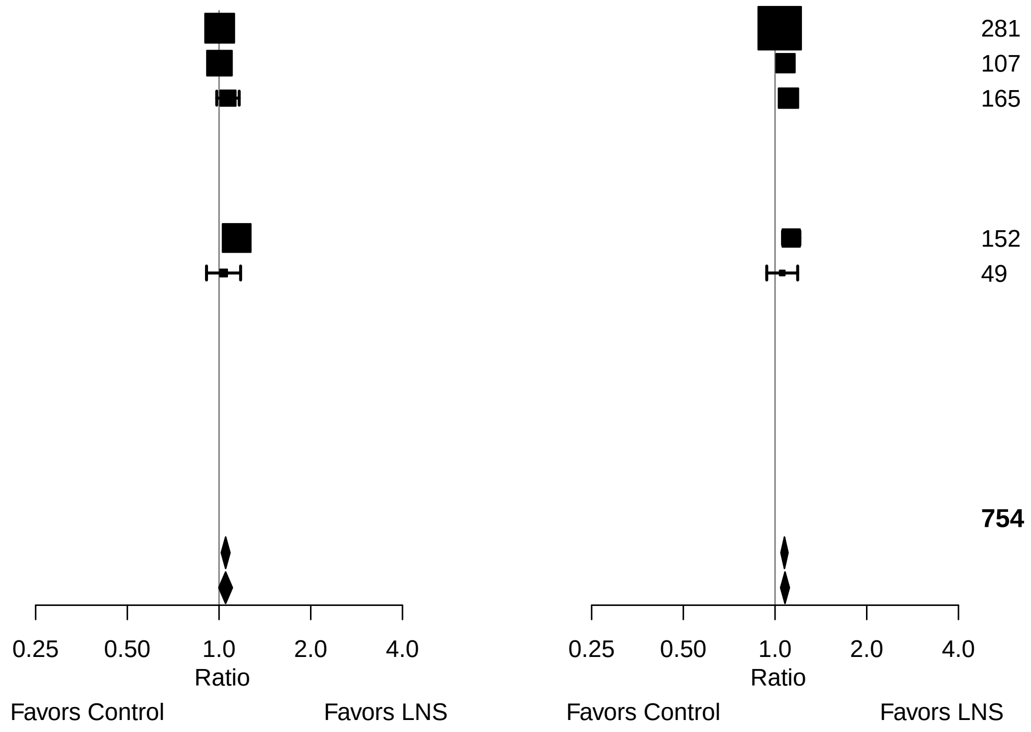

Supplemental figure 8W: Geometric mean ratio of retinol binding protein concentration

8W5: Stratified by Child birth order

P−for−interaction = 0.353
Ratio of GMRs = 0.98 (0.93, 1.03)

Country	Trial	Later born LNS N	Later born Control N	Later born Control Median	Later born GMR (95% CI)	Later born Fixed W	Later born Random W	Firstborn LNS N	Firstborn Control N	Firstborn Control Median	Firstborn GMR (95% CI)	Firstborn Fixed W	Firstborn Random W
Bangladesh	JiVitA−4 (35)												
Bangladesh	RDNS (36)	340	171	1.2	1.03 (0.98, 1.08)	0.32	0.25	210	101	1.2	1.00 (0.93, 1.08)	0.28	0.28
Bangladesh	WASH−B (37)	124	119	1.1	1.03 (0.96, 1.10)	0.16	0.19	86	57	1.2	1.07 (0.98, 1.16)	0.21	0.21
Burkina Faso	iLiNS−Zinc (38)	249	71	1.0	1.11 (1.04, 1.18)	0.16	0.20	66	25	1.1	1.04 (0.95, 1.13)	0.22	0.22
Burkina Faso	PROMIS CS (39)												
Ghana	GHANA (40)												
Ghana	iLiNS−DYAD−G (41)												
Kenya	WASH−B (42)	237	213	1.0	1.15 (1.09, 1.20)	0.30	0.24	60	46	0.9	1.10 (1.00, 1.21)	0.17	0.17
Madagascar	MAHAY (43)	61	37	1.1	1.04 (0.93, 1.15)	0.06	0.12	21	11	1.2	1.05 (0.93, 1.18)	0.11	0.11
Malawi	iLiNS−DYAD−M (44)												
Malawi	iLiNS−DOSE (45)												
Mali	PROMIS CS (46)												
Zimbabwe	SHINE (HIV−) (47)												
Zimbabwe	SHINE (HIV+) (48)												
		**1011**	**611**		**I² = 0.68, Tau² = 0.00**			**443**	**240**		**I² = 0.00, Tau² = 0.00**		
**Fixed**					**1.08 (1.05, 1.11)**						**1.05 (1.00, 1.09)**		
**Random**					**1.07 (1.03, 1.12)**						**1.05 (1.00, 1.09)**		

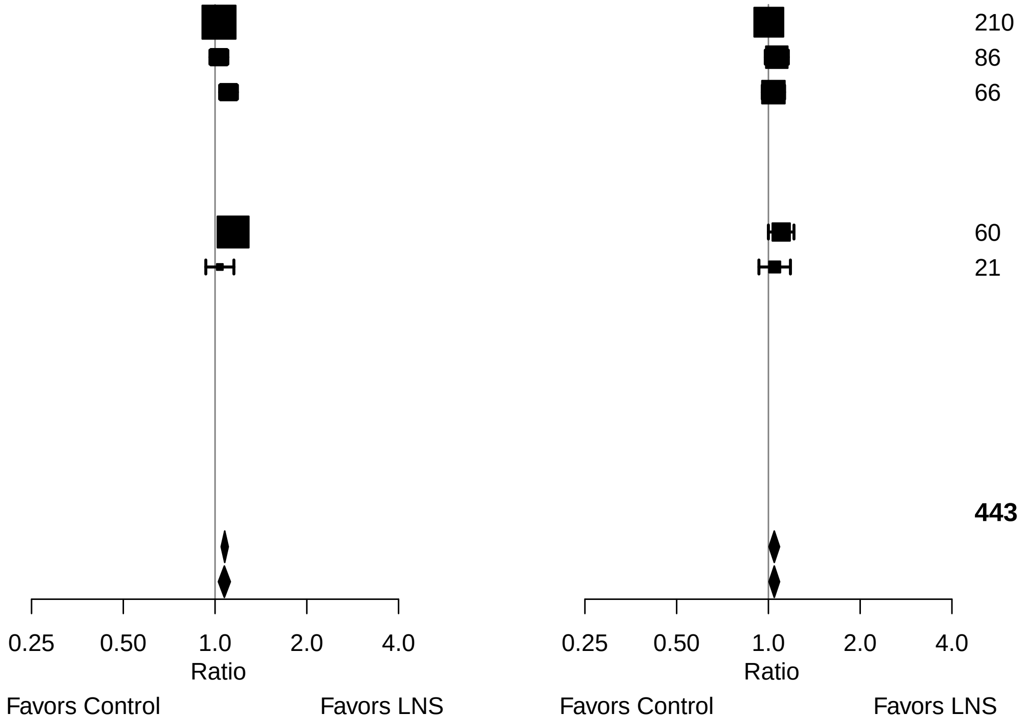

Supplemental figure 8W: Geometric mean ratio of retinol binding protein concentration

8W6: Stratified by Child baseline acute malnutrition (insufficient comparisons)

Supplemental figure 8W: Geometric mean ratio of retinol binding protein concentration

8W7: Stratified by Child baseline anemia (insufficient comparisons)

Supplemental figure 8W: Geometric mean ratio of retinol binding protein concentration

8W8: Stratified by Child high-dose vitamin A supplementation

P−for−interaction = 0.107
Ratio of GMRs = 0.94 (0.88, 1.01)

Country	Trial	Received VitA: LNS N	Control N	Control Median	GMR (95% CI)	Fixed W	Random W	Not treated: LNS N	Control N	Control Median	GMR (95% CI)	Fixed W	Random W
Bangladesh	JiVitA−4 (35)												
Bangladesh	RDNS (36)	444	223	1.2	1.02 (0.97, 1.06)	0.80	0.40	105	49	1.2	1.04 (0.94, 1.14)	0.22	0.34
Bangladesh	WASH−B (37)	16	16	1.0	1.21 (1.07, 1.37)	0.11	0.31	15	9	1.2	0.91 (0.79, 1.06)	0.09	0.24
Burkina Faso	iLiNS−Zinc (38)												
Burkina Faso	PROMIS CS (39)												
Ghana	GHANA (40)												
Ghana	iLiNS−DYAD−G (41)												
Kenya	WASH−B (42)	26	31	0.9	1.24 (1.08, 1.42)	0.09	0.29	223	180	1.0	1.11 (1.05, 1.17)	0.69	0.42
Madagascar	MAHAY (43)												
Malawi	iLiNS−DYAD−M (44)												
Malawi	iLiNS−DOSE (45)												
Mali	PROMIS CS (46)												
Zimbabwe	SHINE (HIV−) (47)												
Zimbabwe	SHINE (HIV+) (48)												
		**486**	**270**		**I² = 0.85, Tau² = 0.01**			**343**	**238**		**I² = 0.69, Tau² = 0.01**		
**Fixed**					**1.05 (1.01, 1.10)**						**1.07 (1.03, 1.12)**		
**Random**					**1.14 (1.00, 1.29)**						**1.03 (0.93, 1.15)**		

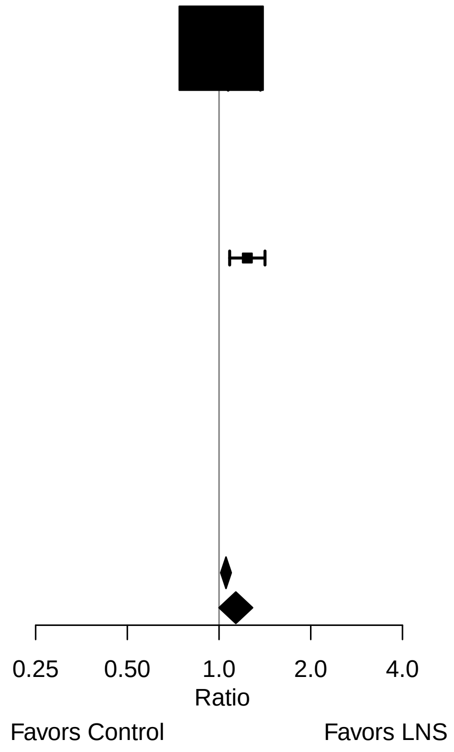

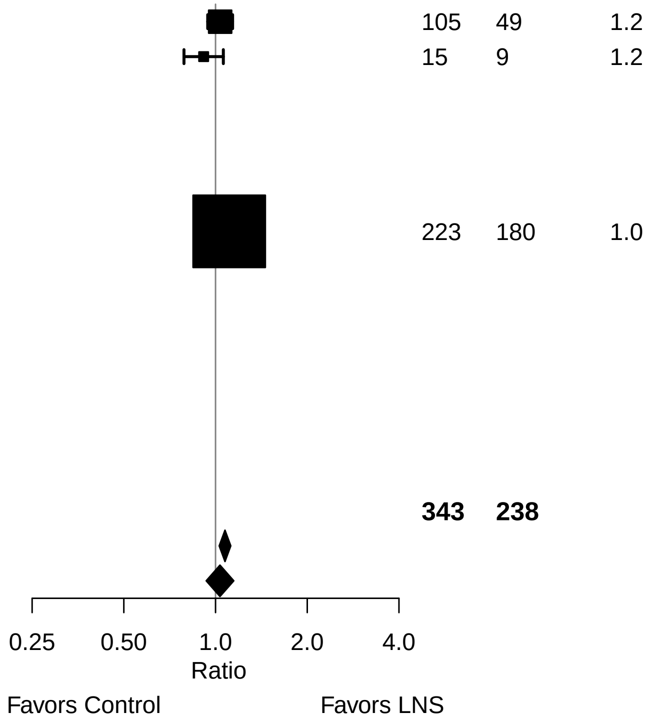

Supplemental figure 8W: Geometric mean ratio of retinol binding protein concentration

8W9: Stratified by Child inflammation

P−for−interaction = 0.088
Ratio of GMRs = 1.04 (0.99, 1.08)

		Not inflamed						High AGP or CRP					
Country	Trial	LNS N	Control N	Control Median	GMR (95% CI)	Fixed W	Random W	LNS N	Control N	Control Median	GMR (95% CI)	Fixed W	Random W
Bangladesh	JiVitA−4 (35)												
Bangladesh	RDNS (36)	370	168	1.2	1.00 (0.96, 1.04)	0.40	0.27	180	104	1.1	1.06 (0.98, 1.14)	0.22	0.22
Bangladesh	WASH−B (37)	165	136	1.2	1.05 (0.99, 1.11)	0.20	0.22	47	42	1.1	1.02 (0.91, 1.14)	0.11	0.15
Burkina Faso	iLiNS−Zinc (38)	124	31	1.1	1.13 (1.04, 1.22)	0.10	0.16	191	65	1.1	1.07 (1.01, 1.15)	0.33	0.27
Burkina Faso	PROMIS CS (39)												
Ghana	GHANA (40)												
Ghana	iLiNS−DYAD−G (41)												
Kenya	WASH−B (42)	157	135	1.0	1.09 (1.04, 1.15)	0.25	0.24	141	124	0.9	1.18 (1.10, 1.27)	0.27	0.25
Madagascar	MAHAY (43)	44	32	1.1	1.00 (0.89, 1.12)	0.05	0.10	39	19	1.1	1.12 (0.98, 1.29)	0.07	0.11
Malawi	iLiNS−DYAD−M (44)												
Malawi	iLiNS−DOSE (45)												
Mali	PROMIS CS (46)												
Zimbabwe	SHINE (HIV−) (47)												
Zimbabwe	SHINE (HIV+) (48)												
		**860**	**502**		**I^2 = 0.64, Tau^2 = 0.00**			**598**	**354**		**I^2 = 0.49, Tau^2 = 0.00**		
**Fixed**					**1.04 (1.02, 1.07)**						**1.10 (1.06, 1.14)**		
**Random**					**1.05 (1.00, 1.10)**						**1.09 (1.04, 1.15)**		

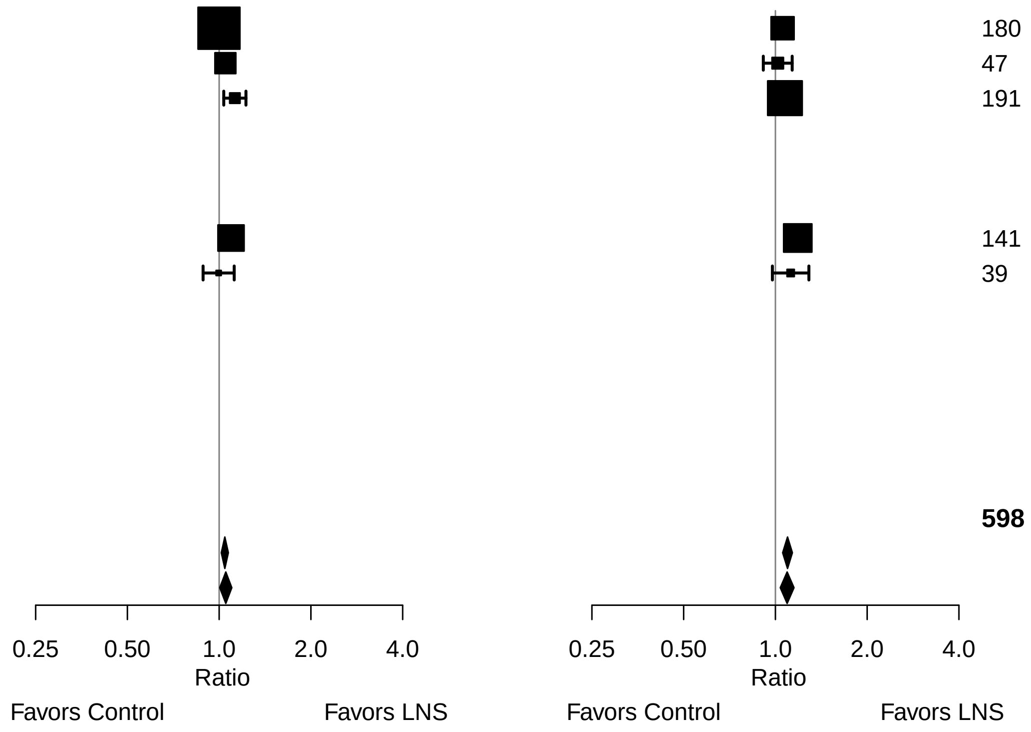

Supplemental figure 8X: Low vitamin A status (RBP < 0.70 µmol/L) prevalence ratio

8X1: Stratified by Maternal BMI (insufficient comparisons)

Supplemental figure 8X: Low vitamin A status (RBP < 0.70 µmol/L) prevalence ratio

8X2: Stratified by Maternal age (insufficient comparisons)

Supplemental figure 8X: Low vitamin A status (RBP < 0.70 µmol/L) prevalence ratio

8X3: Stratified by Maternal education (insufficient comparisons)

Supplemental figure 8X: Low vitamin A status (RBP < 0.70 µmol/L) prevalence ratio

8X4: Stratified by Child sex (insufficient comparisons)

Supplemental figure 8X: Low vitamin A status (RBP < 0.70 µmol/L) prevalence ratio

8X5: Stratified by Child birth order (insufficient comparisons)

Supplemental figure 8X: Low vitamin A status (RBP < 0.70 µmol/L) prevalence ratio

8X6: Stratified by Child baseline acute malnutrition (insufficient comparisons)

Supplemental figure 8X: Low vitamin A status (RBP < 0.70 µmol/L) prevalence ratio

8X7: Stratified by Child baseline anemia (insufficient comparisons)

Supplemental figure 8X: Low vitamin A status (RBP < 0.70 µmol/L) prevalence ratio

8X8: Stratified by Child high-dose vitamin A supplementation (insufficient comparisons)

Supplemental figure 8X: Low vitamin A status (RBP < 0.70 µmol/L) prevalence ratio

8X9: Stratified by Child inflammation (insufficient comparisons)

Supplemental figure 8Y: Low vitamin A status (RBP < 0.70 µmol/L) prevalence difference

8Y1: Stratified by Maternal BMI (insufficient comparisons)

Supplemental figure 8Y: Low vitamin A status (RBP < 0.70 µmol/L) prevalence difference

8Y2: Stratified by Maternal age (insufficient comparisons)

Supplemental figure 8Y: Low vitamin A status (RBP < 0.70 µmol/L) prevalence difference

8Y3: Stratified by Maternal education (insufficient comparisons)

Supplemental figure 8Y: Low vitamin A status (RBP < 0.70 µmol/L) prevalence difference

8Y4: Stratified by Child sex (insufficient comparisons)

Supplemental figure 8Y: Low vitamin A status (RBP < 0.70 µmol/L) prevalence difference

8Y5: Stratified by Child birth order (insufficient comparisons)

Supplemental figure 8Y: Low vitamin A status (RBP < 0.70 µmol/L) prevalence difference

8Y6: Stratified by Child baseline acute malnutrition (insufficient comparisons)

Supplemental figure 8Y: Low vitamin A status (RBP < 0.70 µmol/L) prevalence difference

8Y7: Stratified by Child baseline anemia (insufficient comparisons)

Supplemental figure 8Y: Low vitamin A status (RBP < 0.70 µmol/L) prevalence difference

8Y8: Stratified by Child high-dose vitamin A supplementation (insufficient comparisons)

Supplemental figure 8Y: Low vitamin A status (RBP < 0.70 µmol/L) prevalence difference

8Y9: Stratified by Child inflammation (insufficient comparisons)

Supplemental figure 8Z: Marginal vitamin A status (RBP < 1.05 µmol/L) prevalence ratio

8Z1: Stratified by Maternal BMI

P−for−interaction = 0.327
Ratio of PRs = 1.13 (0.89, 1.43)

		At least 20 kg/m²						Less than 20 kg/m²					
Country	Trial	LNS N	Control N	Control Prevalence	PR (95% CI)	Fixed W	Random W	LNS N	Control N	Control Prevalence	PR (95% CI)	Fixed W	Random W
Bangladesh	JiVitA−4 (35)												
Bangladesh	RDNS (36)	228	102	34.3	0.66 (0.47, 0.94)	0.18	0.18	300	160	27.5	1.01 (0.71, 1.42)	0.26	0.26
Bangladesh	WASH−B (37)	93	97	30.9	0.76 (0.46, 1.27)	0.08	0.08	119	81	40.7	0.87 (0.61, 1.23)	0.25	0.25
Burkina Faso	iLiNS−Zinc (38)	209	55	49.2	0.76 (0.55, 1.06)	0.20	0.20	106	41	48.9	0.76 (0.54, 1.07)	0.26	0.26
Burkina Faso	PROMIS CS (39)												
Ghana	GHANA (40)												
Ghana	iLiNS−DYAD−G (41)												
Kenya	WASH−B (42)	223	200	61.5	0.72 (0.59, 0.88)	0.54	0.54	64	51	56.9	0.69 (0.48, 0.98)	0.23	0.23
Madagascar	MAHAY (43)												
Malawi	iLiNS−DYAD−M (44)												
Malawi	iLiNS−DOSE (45)												
Mali	PROMIS CS (46)												
Zimbabwe	SHINE (HIV−) (47)												
Zimbabwe	SHINE (HIV+) (48)												
		**753**	**454**		**I² = 0.00, Tau² = 0.00**			**589**	**333**		**I² = 0.00, Tau² = 0.00**		
**Fixed**					**0.72 (0.62, 0.84)**						**0.82 (0.69, 0.98)**		
**Random**					**0.72 (0.62, 0.84)**						**0.82 (0.69, 0.98)**		

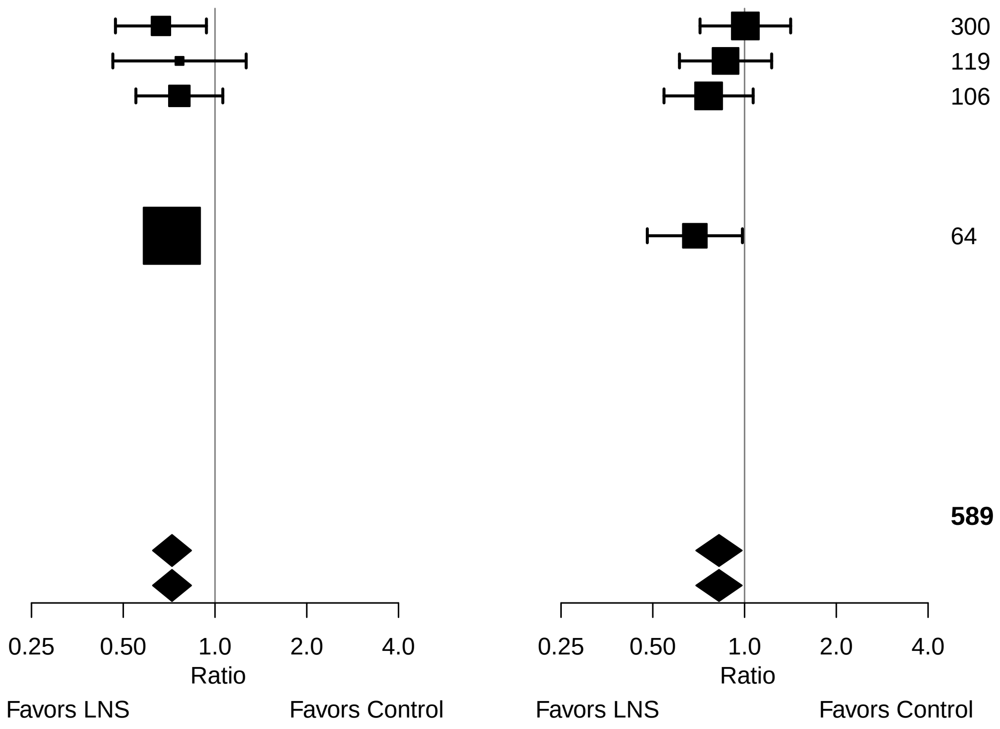

Supplemental figure 8Z: Marginal vitamin A status (RBP < 1.05 µmol/L) prevalence ratio

8Z2: Stratified by Maternal age

P−for−interaction = 0.255
Ratio of PRs = 0.89 (0.72, 1.09)

		At least 25 y						Less than 25 y					
Country	Trial	LNS N	Control N	Control Prevalence	PR (95% CI)	Fixed W	Random W	LNS N	Control N	Control Prevalence	PR (95% CI)	Fixed W	Random W
Bangladesh	JiVitA−4 (35)												
Bangladesh	RDNS (36)	152	81	29.6	0.87 (0.54, 1.39)	0.10	0.10	398	191	28.8	0.87 (0.68, 1.11)	0.35	0.34
Bangladesh	WASH−B (37)	79	80	38.8	1.05 (0.71, 1.54)	0.14	0.14	132	98	32.7	0.72 (0.45, 1.16)	0.09	0.09
Burkina Faso	iLiNS−Zinc (38)	178	50	50.0	0.72 (0.55, 0.93)	0.30	0.30	137	46	48.1	0.83 (0.61, 1.12)	0.22	0.23
Burkina Faso	PROMIS CS (39)												
Ghana	GHANA (40)												
Ghana	iLiNS−DYAD−G (41)												
Kenya	WASH−B (42)	178	149	55.0	0.82 (0.65, 1.03)	0.41	0.41	116	109	66.1	0.61 (0.47, 0.80)	0.31	0.30
Madagascar	MAHAY (43)	47	25	40.0	0.90 (0.47, 1.74)	0.05	0.05	36	26	38.5	0.72 (0.33, 1.58)	0.03	0.04
Malawi	iLiNS−DYAD−M (44)												
Malawi	iLiNS−DOSE (45)												
Mali	PROMIS CS (46)												
Zimbabwe	SHINE (HIV−) (47)												
Zimbabwe	SHINE (HIV+) (48)												
		**634**	**385**		**I² = 0.00, Tau² = 0.00**			**819**	**470**		**I² = 0.05, Tau² = 0.00**		
**Fixed**					**0.82 (0.71, 0.95)**						**0.76 (0.65, 0.87)**		
**Random**					**0.82 (0.71, 0.95)**						**0.76 (0.65, 0.88)**		

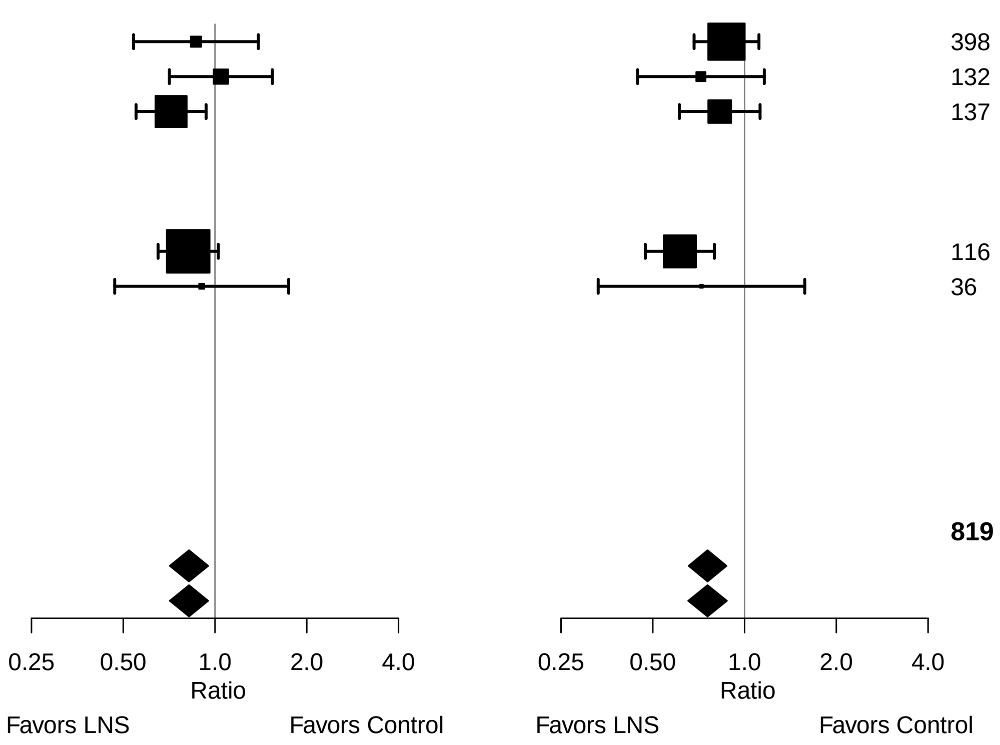

Supplemental figure 8Z: Marginal vitamin A status (RBP < 1.05 µmol/L) prevalence ratio

8Z3: Stratified by Maternal education

P−for−interaction = 0.772
Ratio of PRs = 1.04 (0.79, 1.38)

		Primary or greater						Incomplete or no formal					
Country	Trial	LNS N	Control N	Control Prevalence	PR (95% CI)	Fixed W	Random W	LNS N	Control N	Control Prevalence	PR (95% CI)	Fixed W	Random W
Bangladesh	JiVitA−4 (35)												
Bangladesh	RDNS (36)	406	195	27.2	0.87 (0.67, 1.13)	0.42	0.42	144	77	33.8	0.88 (0.54, 1.45)	0.16	0.16
Bangladesh	WASH−B (37)	149	139	35.3	0.82 (0.58, 1.15)	0.24	0.24	63	39	35.9	0.93 (0.53, 1.62)	0.12	0.12
Burkina Faso	iLiNS−Zinc (38)												
Burkina Faso	PROMIS CS (39)												
Ghana	GHANA (40)												
Ghana	iLiNS−DYAD−G (41)												
Kenya	WASH−B (42)	143	117	53.0	0.71 (0.53, 0.95)	0.33	0.33	154	142	64.8	0.73 (0.58, 0.92)	0.72	0.72
Madagascar	MAHAY (43)												
Malawi	iLiNS−DYAD−M (44)												
Malawi	iLiNS−DOSE (45)												
Mali	PROMIS CS (46)												
Zimbabwe	SHINE (HIV−) (47)												
Zimbabwe	SHINE (HIV+) (48)												
		**698**	**451**		**I² = 0.00, Tau² = 0.00**			**361**	**258**		**I² = 0.00, Tau² = 0.00**		
**Fixed**					**0.80 (0.68, 0.95)**						**0.78 (0.64, 0.94)**		
**Random**					**0.80 (0.68, 0.95)**						**0.78 (0.64, 0.94)**		

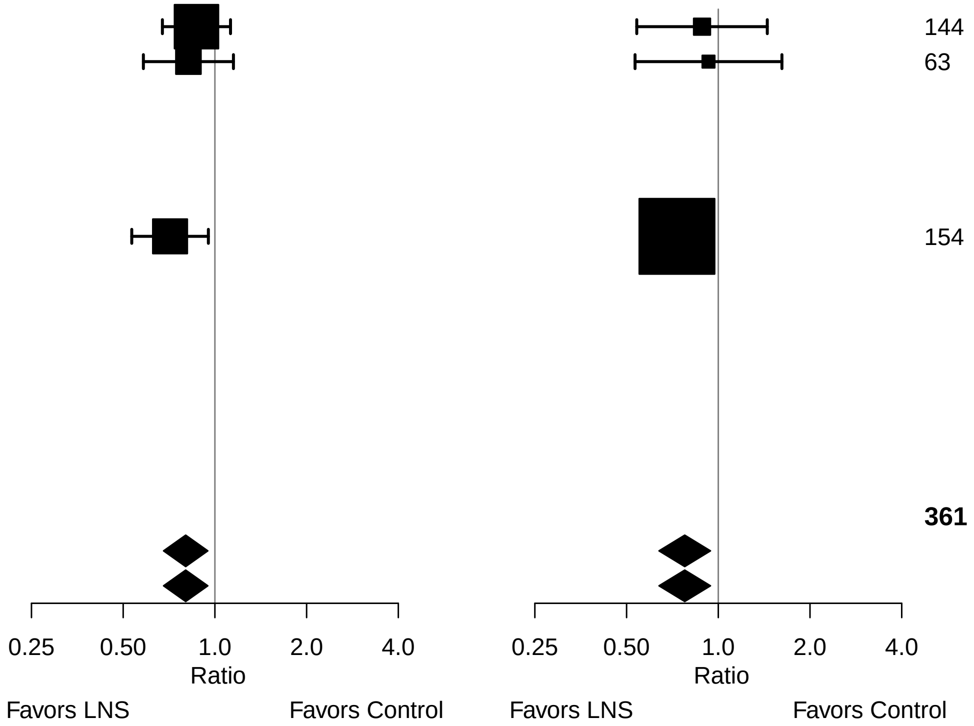

Supplemental figure 8Z: Marginal vitamin A status (RBP < 1.05 µmol/L) prevalence ratio

8Z4: Stratified by Child sex

P−for−interaction = 0.345
Ratio of PRs = 0.91 (0.74, 1.11)

		Male						Female					
Country	Trial	LNS N	Control N	Control Prevalence	PR (95% CI)	Fixed W	Random W	LNS N	Control N	Control Prevalence	PR (95% CI)	Fixed W	Random W
Bangladesh	JiVitA−4 (35)												
Bangladesh	RDNS (36)	266	131	27.5	0.96 (0.68, 1.36)	0.19	0.19	281	141	30.5	0.78 (0.58, 1.05)	0.25	0.25
Bangladesh	WASH−B (37)	105	85	32.9	1.04 (0.70, 1.55)	0.15	0.15	107	93	37.6	0.70 (0.47, 1.03)	0.14	0.14
Burkina Faso	iLiNS−Zinc (38)	150	49	57.4	0.74 (0.55, 0.99)	0.27	0.27	165	47	40.4	0.83 (0.61, 1.12)	0.23	0.23
Burkina Faso	PROMIS CS (39)												
Ghana	GHANA (40)												
Ghana	iLiNS−DYAD−G (41)												
Kenya	WASH−B (42)	146	125	61.6	0.72 (0.56, 0.93)	0.36	0.36	152	134	57.5	0.72 (0.56, 0.93)	0.33	0.33
Madagascar	MAHAY (43)	34	22	31.8	0.92 (0.41, 2.08)	0.04	0.04	49	29	44.8	0.77 (0.43, 1.38)	0.06	0.06
Malawi	iLiNS−DYAD−M (44)												
Malawi	iLiNS−DOSE (45)												
Mali	PROMIS CS (46)												
Zimbabwe	SHINE (HIV−) (47)												
Zimbabwe	SHINE (HIV+) (48)												
		**701**	**412**		**I² = 0.00, Tau² = 0.00**			**754**	**444**		**I² = 0.00, Tau² = 0.00**		
**Fixed**					**0.82 (0.70, 0.95)**						**0.76 (0.66, 0.88)**		
**Random**					**0.82 (0.70, 0.95)**						**0.76 (0.66, 0.88)**		

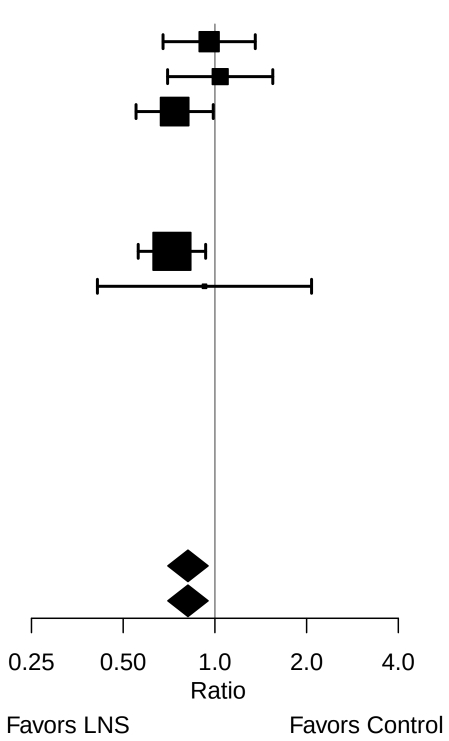

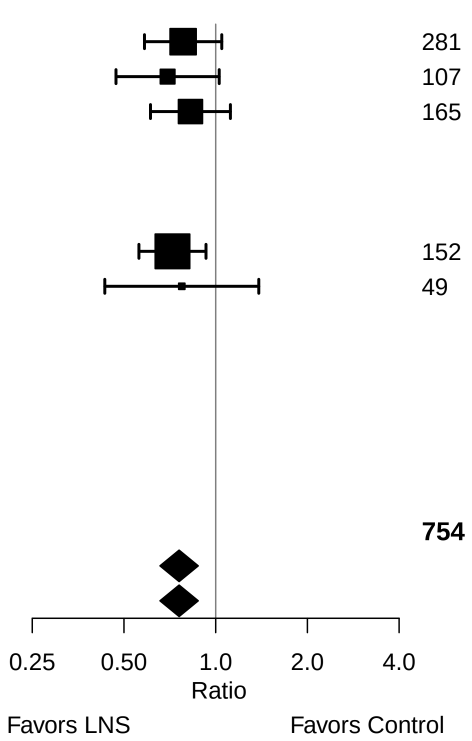

Supplemental figure 8Z: Marginal vitamin A status (RBP < 1.05 μmol/L) prevalence ratio

8Z5: Stratified by Child birth order

P−for−interaction = 0.310
Ratio of PRs = 1.13 (0.89, 1.44)

Country	Trial	Later born LNS N	Later born Control N	Later born Control Prevalence	Later born PR (95% CI)	Later born Fixed W	Later born Random W	Firstborn LNS N	Firstborn Control N	Firstborn Control Prevalence	Firstborn PR (95% CI)	Firstborn Fixed W	Firstborn Random W
Bangladesh	JiVitA−4 (35)												
Bangladesh	RDNS (36)	340	171	30.4	0.82 (0.59, 1.14)	0.14	0.14	210	101	26.7	0.96 (0.66, 1.40)	0.30	0.30
Bangladesh	WASH−B (37)	124	119	38.7	0.92 (0.69, 1.22)	0.19	0.19	86	57	29.8	0.74 (0.39, 1.41)	0.10	0.10
Burkina Faso	iLiNS−Zinc (38)	249	71	53.3	0.70 (0.55, 0.88)	0.29	0.29	66	25	38.7	1.05 (0.65, 1.71)	0.18	0.18
Burkina Faso	PROMIS CS (39)												
Ghana	GHANA (40)												
Ghana	iLiNS−DYAD−G (41)												
Kenya	WASH−B (42)	237	213	58.2	0.70 (0.57, 0.86)	0.38	0.38	60	46	65.2	0.77 (0.56, 1.05)	0.42	0.42
Madagascar	MAHAY (43)												
Malawi	iLiNS−DYAD−M (44)												
Malawi	iLiNS−DOSE (45)												
Mali	PROMIS CS (46)												
Zimbabwe	SHINE (HIV−) (47)												
Zimbabwe	SHINE (HIV+) (48)												
		**950**	**574**		**I² = 0.00, Tau² = 0.00**			**422**	**229**		**I² = 0.00, Tau² = 0.00**		
**Fixed**					**0.75 (0.67, 0.85)**						**0.86 (0.70, 1.06)**		
**Random**					**0.75 (0.67, 0.85)**						**0.86 (0.70, 1.06)**		

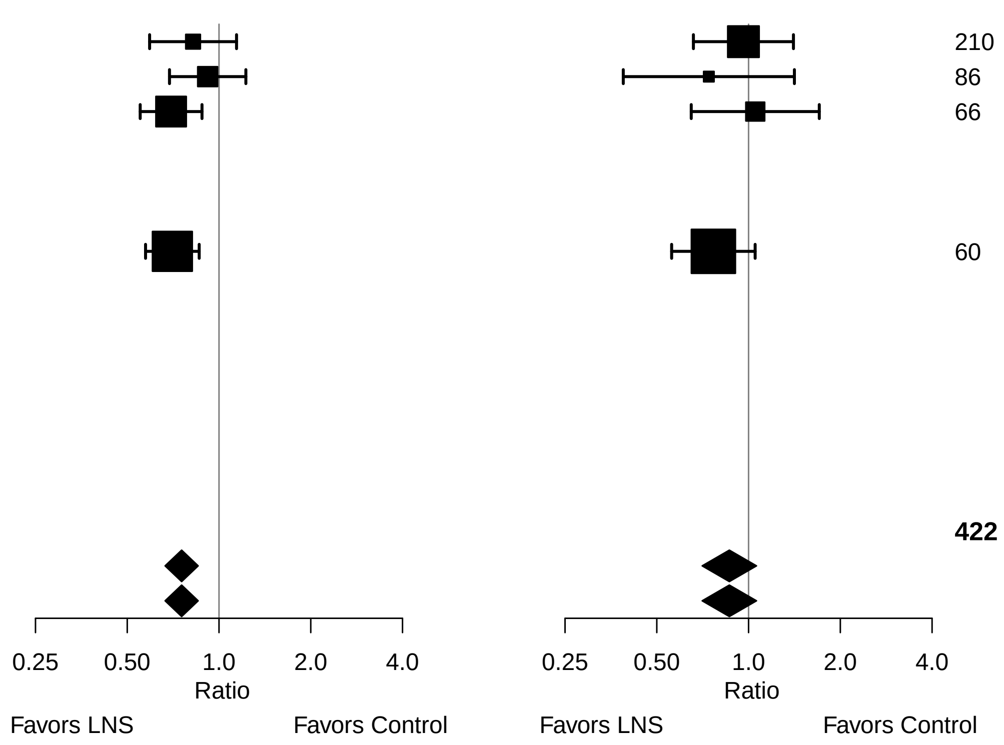

Supplemental figure 8Z: Marginal vitamin A status (RBP < 1.05 µmol/L) prevalence ratio

8Z6: Stratified by Child baseline acute malnutrition (insufficient comparisons)

Supplemental figure 8Z: Marginal vitamin A status (RBP < 1.05 µmol/L) prevalence ratio

8Z7: Stratified by Child baseline anemia (insufficient comparisons)

Supplemental figure 8Z: Marginal vitamin A status (RBP < 1.05 µmol/L) prevalence ratio

8Z8: Stratified by Child high-dose vitamin A supplementation (insufficient comparisons)

Supplemental figure 8Z: Marginal vitamin A status (RBP < 1.05 μmol/L) prevalence ratio

8Z9: Stratified by Child inflammation

P−for−interaction = 0.223
Ratio of PRs = 0.87 (0.69, 1.09)

Country	Trial	LNS N	Control N	Control Prevalence	Not inflamed PR (95% CI)	Fixed W	Random W	LNS N	Control N	Control Prevalence	High AGP or CRP PR (95% CI)	Fixed W	Random W
Bangladesh	JiVitA−4 (35)												
Bangladesh	RDNS (36)	370	168	23.8	1.02 (0.68, 1.53)	0.14	0.14	180	104	37.5	0.73 (0.54, 0.98)	0.27	0.27
Bangladesh	WASH−B (37)	165	136	31.6	0.94 (0.63, 1.40)	0.14	0.14	47	42	47.6	0.67 (0.38, 1.18)	0.08	0.08
Burkina Faso	iLiNS−Zinc (38)	124	31	50.0	0.73 (0.53, 0.99)	0.24	0.24	191	65	48.6	0.77 (0.57, 1.04)	0.27	0.27
Burkina Faso	PROMIS CS (39)												
Ghana	GHANA (40)												
Ghana	iLiNS−DYAD−G (41)												
Kenya	WASH−B (42)	157	135	56.3	0.77 (0.61, 0.97)	0.43	0.43	141	124	62.9	0.68 (0.52, 0.88)	0.34	0.34
Madagascar	MAHAY (43)	44	32	37.5	0.85 (0.42, 1.72)	0.05	0.05	39	19	42.1	0.79 (0.37, 1.69)	0.04	0.04
Malawi	iLiNS−DYAD−M (44)												
Malawi	iLiNS−DOSE (45)												
Mali	PROMIS CS (46)												
Zimbabwe	SHINE (HIV−) (47)												
Zimbabwe	SHINE (HIV+) (48)												
		**860**	**502**		**I² = 0.00, Tau² = 0.00**			**598**	**354**		**I² = 0.00, Tau² = 0.00**		
**Fixed**					**0.82 (0.70, 0.95)**						**0.72 (0.61, 0.84)**		
**Random**					**0.82 (0.70, 0.95)**						**0.72 (0.61, 0.84)**		

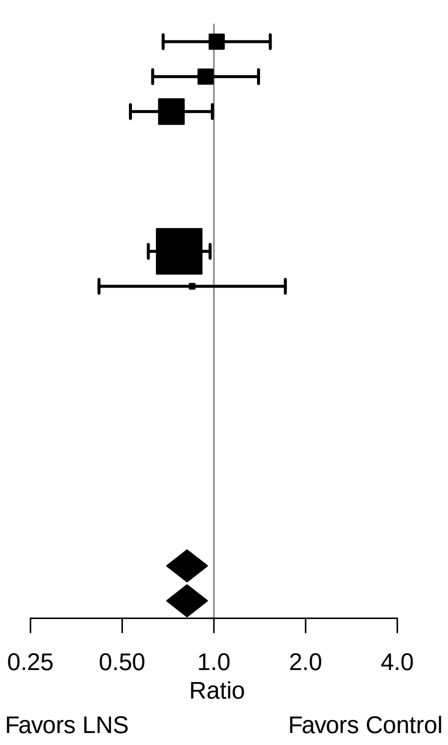

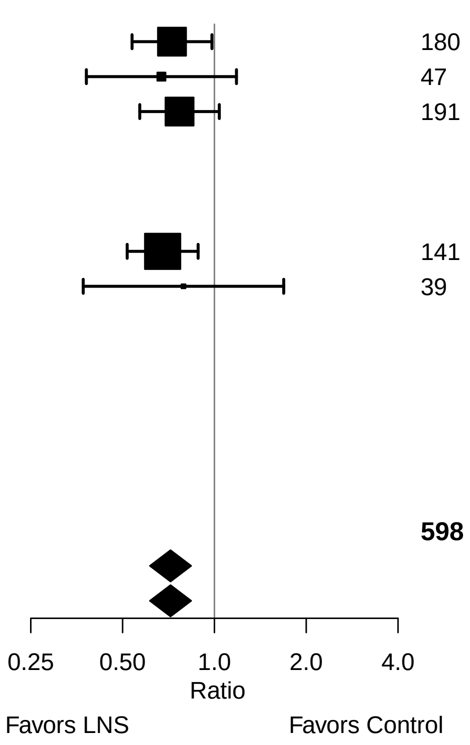

Supplemental figure 8AA: Marginal vitamin A status (RBP < 1.05 µmol/L) prevalence difference

8AA1: Stratified by Maternal BMI

P−for−interaction = 0.234
Difference in PDs = 0.05 (−0.04, 0.14)

		At least 20 kg/m²						Less than 20 kg/m²					
Country	Trial	LNS N	Control N	Control Prevalence	PD (95% CI)	Fixed W	Random W	LNS N	Control N	Control Prevalence	PD (95% CI)	Fixed W	Random W
Bangladesh	JiVitA−4 (35)												
Bangladesh	RDNS (36)	228	102	34.3	−0.12 (−0.21, −0.02)	0.35	0.35	300	160	27.5	0.00 (−0.09, 0.10)	0.44	0.38
Bangladesh	WASH−B (37)	93	97	30.9	−0.07 (−0.21, 0.06)	0.18	0.18	119	81	40.7	−0.05 (−0.19, 0.08)	0.22	0.24
Burkina Faso	iLiNS−Zinc (38)	209	55	49.2	−0.12 (−0.26, 0.02)	0.17	0.17	106	41	48.9	−0.11 (−0.26, 0.04)	0.17	0.20
Burkina Faso	PROMIS CS (39)												
Ghana	GHANA (40)												
Ghana	iLiNS−DYAD−G (41)												
Kenya	WASH−B (42)	223	200	61.5	−0.17 (−0.28, −0.07)	0.30	0.30	64	51	56.9	−0.18 (−0.34, −0.02)	0.16	0.19
Madagascar	MAHAY (43)												
Malawi	iLiNS−DYAD−M (44)												
Malawi	iLiNS−DOSE (45)												
Mali	PROMIS CS (46)												
Zimbabwe	SHINE (HIV−) (47)												
Zimbabwe	SHINE (HIV+) (48)												
		**753**	**454**		**I² = 0.00, Tau² = 0.00**			**589**	**333**		**I² = 0.30, Tau² = 0.00**		
**Fixed**					**−0.12 (−0.18, −0.07)**						**−0.06 (−0.12, 0.00)**		
**Random**					**−0.12 (−0.18, −0.07)**						**−0.07 (−0.14, 0.01)**		

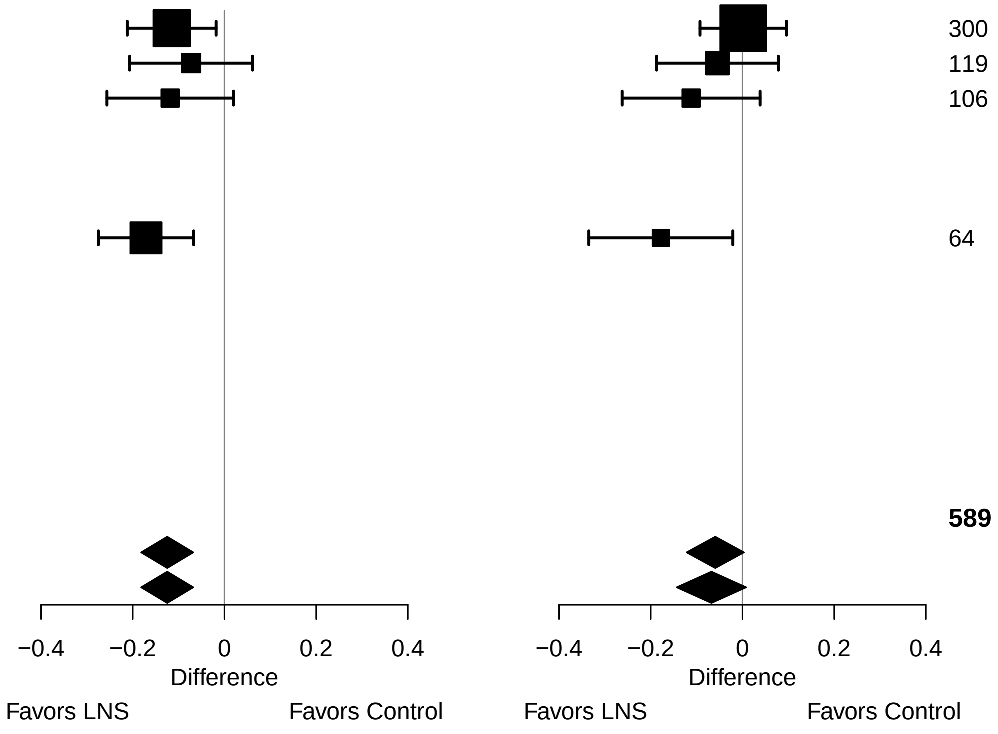

Supplemental figure 8AA: Marginal vitamin A status (RBP < 1.05 µmol/L) prevalence difference

8AA2: Stratified by Maternal age

P−for−interaction = 0.217
Difference in PDs = −0.05 (−0.14, 0.03)

		At least 25 y						Less than 25 y					
		**LNS**	**Control**	**Control**	**PD**	**Fixed**	**Random**	**LNS**	**Control**	**Control**	**PD**	**Fixed**	**Random**
Country	Trial	N	N	Prevalence	(95% CI)	W	W	N	N	Prevalence	(95% CI)	W	W
Bangladesh	JiVitA−4 (35)												
Bangladesh	RDNS (36)	152	81	29.6	−0.04 (−0.17, 0.09)	0.20	0.20	398	191	28.8	−0.04 (−0.10, 0.03)	0.53	0.33
Bangladesh	WASH−B (37)	79	80	38.8	0.02 (−0.14, 0.17)	0.15	0.15	132	98	32.7	−0.09 (−0.22, 0.04)	0.14	0.19
Burkina Faso	iLiNS−Zinc (38)	178	50	50.0	−0.14 (−0.25, −0.04)	0.34	0.34	137	46	48.1	−0.08 (−0.21, 0.06)	0.13	0.19
Burkina Faso	PROMIS CS (39)												
Ghana	GHANA (40)												
Ghana	iLiNS−DYAD−G (41)												
Kenya	WASH−B (42)	178	149	55.0	−0.10 (−0.22, 0.01)	0.26	0.26	116	109	66.1	−0.26 (−0.38, −0.13)	0.16	0.21
Madagascar	MAHAY (43)	47	25	40.0	−0.04 (−0.29, 0.22)	0.05	0.05	36	26	38.5	−0.11 (−0.35, 0.14)	0.04	0.08
Malawi	iLiNS−DYAD−M (44)												
Malawi	iLiNS−DOSE (45)												
Mali	PROMIS CS (46)												
Zimbabwe	SHINE (HIV−) (47)												
Zimbabwe	SHINE (HIV+) (48)												
		**634**	**385**		**I² = 0.00, Tau² = 0.00**			**819**	**470**		**I² = 0.56, Tau² = 0.00**		
**Fixed**					**−0.08 (−0.14, −0.02)**						**−0.09 (−0.14, −0.04)**		
**Random**					**−0.08 (−0.14, −0.02)**						**−0.11 (−0.18, −0.03)**		

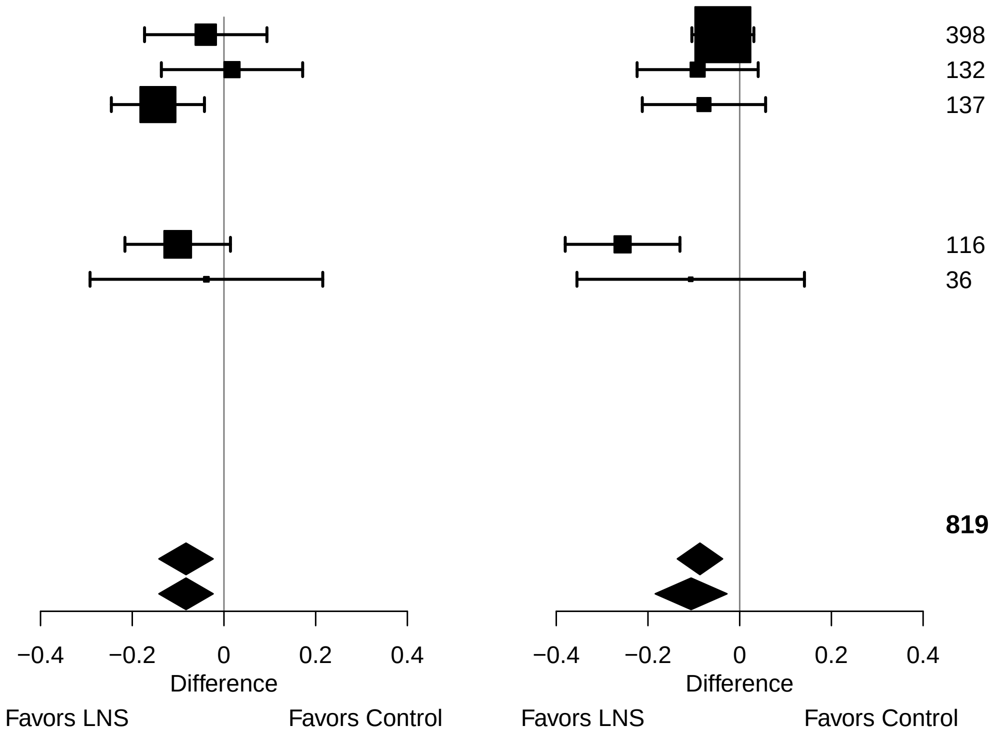

Supplemental figure 8AA: Marginal vitamin A status (RBP < 1.05 µmol/L) prevalence difference

8AA3: Stratified by Maternal education

P−for−interaction = 0.974
Difference in PDs = 0.00 (−0.10, 0.10)

		Primary or greater						Incomplete or no formal					
		**LNS**	**Control**	**Control**	**PD**	**Fixed**	**Random**	**LNS**	**Control**	**Control**	**PD**	**Fixed**	**Random**
Country	Trial	N	N	Prevalence	(95% CI)	W	W	N	N	Prevalence	(95% CI)	W	W
Bangladesh	JiVitA−4 (35)												
Bangladesh	RDNS (36)	406	195	27.2	−0.04 (−0.10, 0.03)	0.61	0.55	144	77	33.8	−0.04 (−0.20, 0.12)	0.31	0.31
Bangladesh	WASH−B (37)	149	139	35.3	−0.06 (−0.17, 0.04)	0.23	0.27	63	39	35.9	−0.03 (−0.22, 0.17)	0.21	0.22
Burkina Faso	iLiNS−Zinc (38)												
Burkina Faso	PROMIS CS (39)												
Ghana	GHANA (40)												
Ghana	iLiNS−DYAD−G (41)												
Kenya	WASH−B (42)	143	117	53.0	−0.15 (−0.28, −0.02)	0.15	0.19	154	142	64.8	−0.17 (−0.30, −0.05)	0.49	0.46
Madagascar	MAHAY (43)												
Malawi	iLiNS−DYAD−M (44)												
Malawi	iLiNS−DOSE (45)												
Mali	PROMIS CS (46)												
Zimbabwe	SHINE (HIV−) (47)												
Zimbabwe	SHINE (HIV+) (48)												
		**698**	**451**		**I² = 0.19, Tau² = 0.00**			**361**	**258**		**I² = 0.17, Tau² = 0.00**		
**Fixed**					**−0.06 (−0.11, −0.01)**						**−0.10 (−0.19, −0.01)**		
**Random**					**−0.06 (−0.13, 0.00)**						**−0.10 (−0.20, 0.00)**		

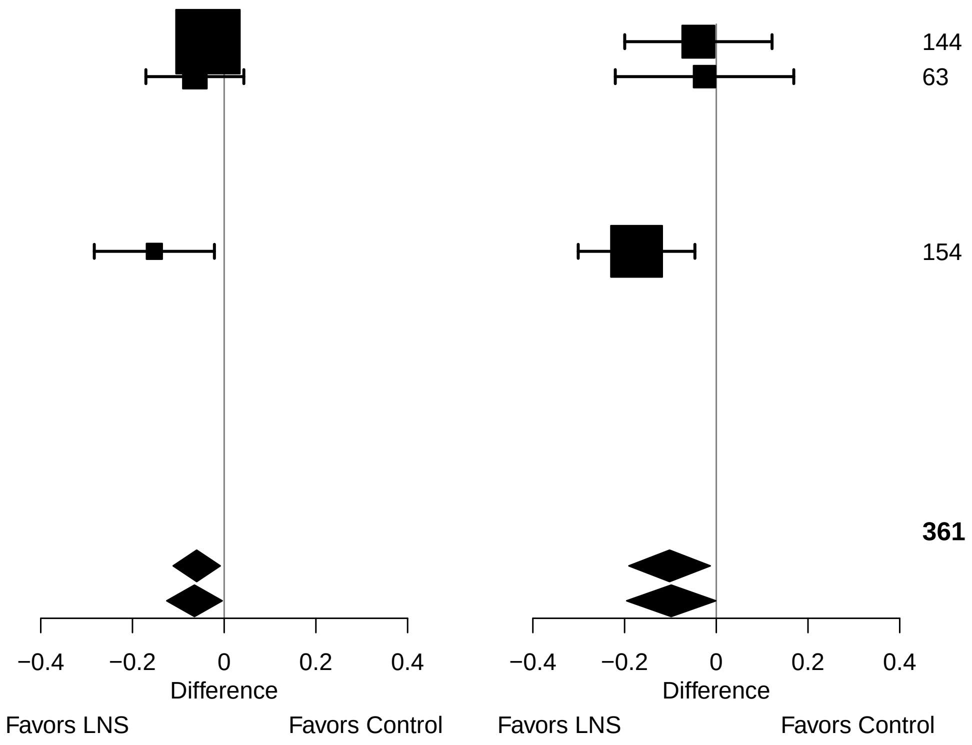

Supplemental figure 8AA: Marginal vitamin A status (RBP < 1.05 µmol/L) prevalence difference

8AA4: Stratified by Child sex

P−for−interaction = 0.393
Difference in PDs = −0.04 (−0.12, 0.05)

Country	Trial	Male LNS N	Male Control N	Male Control Prevalence	Male PD (95% CI)	Male Fixed W	Male Random W	Female LNS N	Female Control N	Female Control Prevalence	Female PD (95% CI)	Female Fixed W	Female Random W
Bangladesh	JiVitA−4 (35)												
Bangladesh	RDNS (36)	266	131	27.5	−0.01 (−0.11, 0.08)	0.38	0.31	281	141	30.5	−0.07 (−0.15, 0.01)	0.40	0.40
Bangladesh	WASH−B (37)	105	85	32.9	0.01 (−0.12, 0.15)	0.19	0.21	107	93	37.6	−0.11 (−0.23, 0.01)	0.18	0.18
Burkina Faso	iLiNS−Zinc (38)	150	49	57.4	−0.14 (−0.28, 0.00)	0.18	0.20	165	47	40.4	−0.07 (−0.18, 0.04)	0.20	0.20
Burkina Faso	PROMIS CS (39)												
Ghana	GHANA (40)												
Ghana	iLiNS−DYAD−G (41)												
Kenya	WASH−B (42)	146	125	61.6	−0.17 (−0.30, −0.04)	0.19	0.21	152	134	57.5	−0.16 (−0.28, −0.04)	0.17	0.17
Madagascar	MAHAY (43)	34	22	31.8	−0.02 (−0.27, 0.23)	0.05	0.08	49	29	44.8	−0.10 (−0.34, 0.13)	0.05	0.05
Malawi	iLiNS−DYAD−M (44)												
Malawi	iLiNS−DOSE (45)												
Mali	PROMIS CS (46)												
Zimbabwe	SHINE (HIV−) (47)												
Zimbabwe	SHINE (HIV+) (48)												
		**701**	**412**		**I² = 0.36, Tau² = 0.00**			**754**	**444**		**I² = 0.00, Tau² = 0.00**		
**Fixed**					**−0.06 (−0.12, 0.00)**						**−0.09 (−0.14, −0.04)**		
**Random**					**−0.07 (−0.14, 0.01)**						**−0.09 (−0.14, −0.04)**		

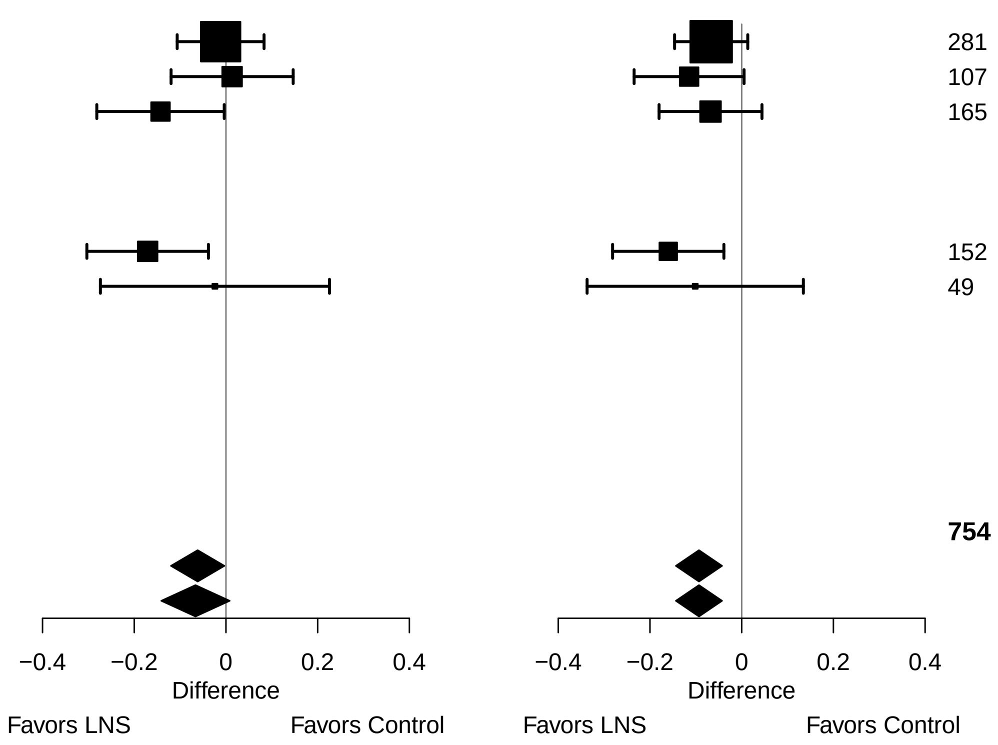

Supplemental figure 8AA: Marginal vitamin A status (RBP < 1.05 µmol/L) prevalence difference

8AA5: Stratified by Child birth order

P−for−interaction = 0.410
Difference in PDs = 0.04 (−0.05, 0.13)

Country	Trial	Later born LNS N	Later born Control N	Later born Control Prevalence	Later born PD (95% CI)	Later born Fixed W	Later born Random W	Firstborn LNS N	Firstborn Control N	Firstborn Control Prevalence	Firstborn PD (95% CI)	Firstborn Fixed W	Firstborn Random W
Bangladesh	JiVitA−4 (35)												
Bangladesh	RDNS (36)	340	171	30.4	−0.05 (−0.15, 0.04)	0.30	0.27	210	101	26.7	−0.01 (−0.11, 0.09)	0.50	0.50
Bangladesh	WASH−B (37)	124	119	38.7	−0.03 (−0.14, 0.07)	0.22	0.24	86	57	29.8	−0.08 (−0.24, 0.09)	0.19	0.19
Burkina Faso	iLiNS−Zinc (38)	249	71	53.3	−0.16 (−0.27, −0.05)	0.22	0.23	66	25	38.7	0.02 (−0.16, 0.20)	0.16	0.16
Burkina Faso	PROMIS CS (39)												
Ghana	GHANA (40)												
Ghana	iLiNS−DYAD−G (41)												
Kenya	WASH−B (42)	237	213	58.2	−0.17 (−0.27, −0.07)	0.26	0.26	60	46	65.2	−0.15 (−0.33, 0.03)	0.16	0.16
Madagascar	MAHAY (43)												
Malawi	iLiNS−DYAD−M (44)												
Malawi	iLiNS−DOSE (45)												
Mali	PROMIS CS (46)												
Zimbabwe	SHINE (HIV−) (47)												
Zimbabwe	SHINE (HIV+) (48)												
		**950**	**574**		**I² = 0.48, Tau² = 0.00**			**422**	**229**		**I² = 0.00, Tau² = 0.00**		
**Fixed**					**−0.10 (−0.15, −0.05)**						**−0.04 (−0.11, 0.03)**		
**Random**					**−0.10 (−0.17, −0.03)**						**−0.04 (−0.11, 0.03)**		

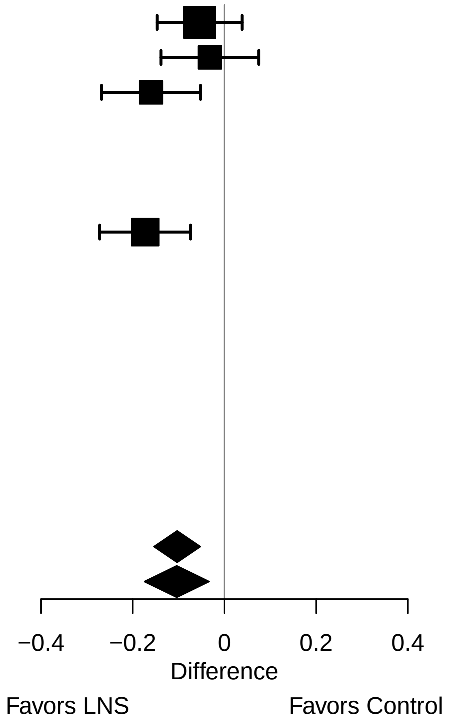

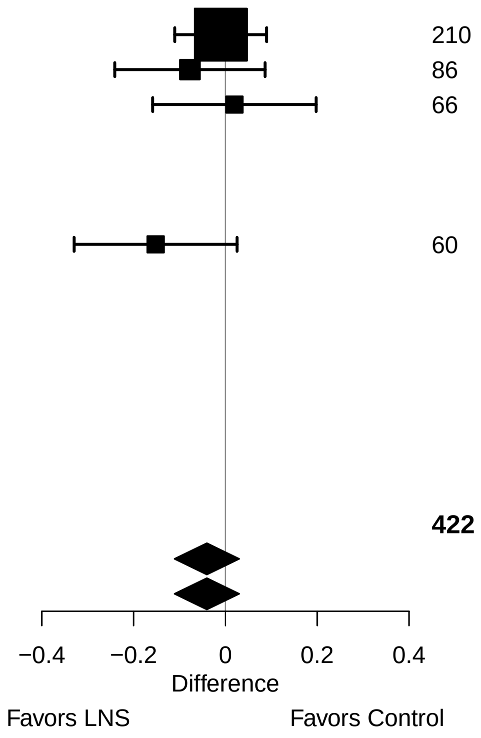

Supplemental figure 8AA: Marginal vitamin A status (RBP < 1.05 µmol/L) prevalence difference

8AA6: Stratified by Child baseline acute malnutrition (insufficient comparisons)

Supplemental figure 8AA: Marginal vitamin A status (RBP < 1.05 µmol/L) prevalence difference

8AA7: Stratified by Child baseline anemia (insufficient comparisons)

Supplemental figure 8AA: Marginal vitamin A status (RBP < 1.05 µmol/L) prevalence difference

8AA8: Stratified by Child high-dose vitamin A supplementation (insufficient comparisons)

Supplemental figure 8AA: Marginal vitamin A status (RBP < 1.05 µmol/L) prevalence difference

8AA9: Stratified by Child inflammation

P−for−interaction = 0.080
Difference in PDs = −0.08 (−0.16, 0.01)

		Not inflamed						High AGP or CRP					
Country	Trial	LNS N	Control N	Control Prevalence	PD (95% CI)	Fixed W	Random W	LNS N	Control N	Control Prevalence	PD (95% CI)	Fixed W	Random W
Bangladesh	JiVitA−4 (35)												
Bangladesh	RDNS (36)	370	168	23.8	0.01 (−0.09, 0.10)	0.35	0.32	180	104	37.5	−0.10 (−0.20, −0.01)	0.40	0.40
Bangladesh	WASH−B (37)	165	136	31.6	−0.02 (−0.14, 0.10)	0.22	0.22	47	42	47.6	−0.16 (−0.37, 0.05)	0.08	0.08
Burkina Faso	iLiNS−Zinc (38)	124	31	50.0	−0.15 (−0.29, 0.00)	0.15	0.16	191	65	48.6	−0.11 (−0.22, 0.01)	0.27	0.27
Burkina Faso	PROMIS CS (39)												
Ghana	GHANA (40)												
Ghana	iLiNS−DYAD−G (41)												
Kenya	WASH−B (42)	157	135	56.3	−0.13 (−0.25, −0.01)	0.23	0.24	141	124	62.9	−0.20 (−0.34, −0.07)	0.21	0.21
Madagascar	MAHAY (43)	44	32	37.5	−0.06 (−0.30, 0.19)	0.05	0.06	39	19	42.1	−0.09 (−0.38, 0.21)	0.04	0.04
Malawi	iLiNS−DYAD−M (44)												
Malawi	iLiNS−DOSE (45)												
Mali	PROMIS CS (46)												
Zimbabwe	SHINE (HIV−) (47)												
Zimbabwe	SHINE (HIV+) (48)												
		**860**	**502**		**I² = 0.16, Tau² = 0.00**			**598**	**354**		**I² = 0.00, Tau² = 0.00**		
**Fixed**					**−0.06 (−0.11, 0.00)**						**−0.13 (−0.19, −0.07)**		
**Random**					**−0.06 (−0.12, 0.00)**						**−0.13 (−0.19, −0.07)**		

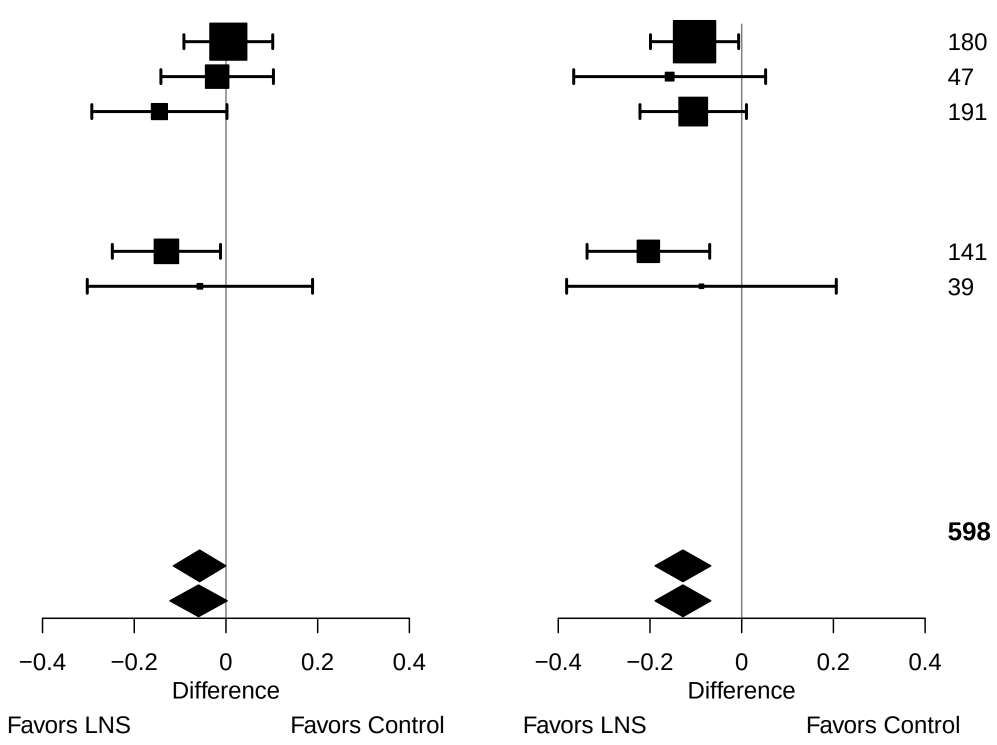

Supplement: nqab276_Supplemental_Files [file nqab276_supplemental_files.zip › 12_ipdb_suppfig8_20210707.pdf]
