# Supplementary material for: Characteristics that modify the effect of small-quantity lipid-based nutrient supplementation on child anemia and micronutrient status: an individual participant data meta-analysis of randomized controlled trials
Source: Am J Clin Nutr. 2021 Sep 29;114(Suppl 1):68S–94S. doi: 10.1093/ajcn/nqab276 (PMC8560313; doi:10.1093/ajcn/nqab276)

Supplemental figure 9: Forest plots for effects of SQ-LNS on biochemical outcomes stratified by individual-level household effect modifiers

Contents

Supplemental figure 9A: Mean difference in hemoglobin concentration **5**
9A1: Stratified by Household socio-economic status 5
9A2: Stratified by Household food insecurity 6
9A3: Stratified by Household source water quality 7
9A4: Stratified by Household sanitation 8
9A5: Stratified by Season at the time of assessment 9

Supplemental figure 9B: Anemia prevalence ratio **10**
9B1: Stratified by Household socio-economic status 10
9B2: Stratified by Household food insecurity 11
9B3: Stratified by Household source water quality 12
9B4: Stratified by Household sanitation 13
9B5: Stratified by Season at the time of assessment 14

Supplemental figure 9C: Anemia prevalence difference **15**
9C1: Stratified by Household socio-economic status 15
9C2: Stratified by Household food insecurity 16
9C3: Stratified by Household source water quality 17
9C4: Stratified by Household sanitation 18
9C5: Stratified by Season at the time of assessment 19

Supplemental figure 9D: Moderate-to-severe anemia prevalence ratio **20**
9D1: Stratified by Household socio-economic status 20
9D2: Stratified by Household food insecurity 21
9D3: Stratified by Household source water quality 22
9D4: Stratified by Household sanitation 23
9D5: Stratified by Season at the time of assessment 24

Supplemental figure 9E: Moderate-to-severe anemia prevalence difference **25**
9E1: Stratified by Household socio-economic status 25
9E2: Stratified by Household food insecurity 26
9E3: Stratified by Household source water quality 27
9E4: Stratified by Household sanitation 28
9E5: Stratified by Season at the time of assessment 29

Supplemental figure 9F: Geometric mean ratio of ferritin concentration **30**
9F1: Stratified by Household socio-economic status 30
9F2: Stratified by Household food insecurity 31
9F3: Stratified by Household source water quality 32
9F4: Stratified by Household sanitation 33
9F5: Stratified by Season at the time of assessment 34

Supplemental figure 9G: Iron deficiency (ferritin < 12 µg/L) prevalence ratio **35**
9G1: Stratified by Household socio-economic status . . . 35
9G2: Stratified by Household food insecurity . . . 36
9G3: Stratified by Household source water quality . . . 37
9G4: Stratified by Household sanitation (insufficient comparisons) . . . 38
9G5: Stratified by Season at the time of assessment . . . 39

Supplemental figure 9H: Iron deficiency (ferritin < 12 µg/L) prevalence difference **40**
9H1: Stratified by Household socio-economic status . . . 40
9H2: Stratified by Household food insecurity . . . 41
9H3: Stratified by Household source water quality . . . 42
9H4: Stratified by Household sanitation (insufficient comparisons) . . . 43
9H5: Stratified by Season at the time of assessment . . . 44

Supplemental figure 9I: Iron deficiency anemia prevalence ratio **45**
9I1: Stratified by Household socio-economic status . . . 45
9I2: Stratified by Household food insecurity (insufficient comparisons) . . . 46
9I3: Stratified by Household source water quality (insufficient comparisons) . . . 47
9I4: Stratified by Household sanitation (insufficient comparisons) . . . 48
9I5: Stratified by Season at the time of assessment (insufficient comparisons) . . . 49

Supplemental figure 9J: Iron deficiency anemia prevalence difference **50**
9J1: Stratified by Household socio-economic status . . . 50
9J2: Stratified by Household food insecurity (insufficient comparisons) . . . 51
9J3: Stratified by Household source water quality (insufficient comparisons) . . . 52
9J4: Stratified by Household sanitation (insufficient comparisons) . . . 53
9J5: Stratified by Season at the time of assessment (insufficient comparisons) . . . 54

Supplemental figure 9K: Geometric mean ratio of soluble transferrin receptor concentration **55**
9K1: Stratified by Household socio-economic status . . . 55
9K2: Stratified by Household food insecurity . . . 56
9K3: Stratified by Household source water quality . . . 57
9K4: Stratified by Household sanitation (insufficient comparisons) . . . 58
9K5: Stratified by Season at the time of assessment . . . 59

Supplemental figure 9L: Elevated soluble transferrin receptor prevalence ratio **60**
9L1: Stratified by Household socio-economic status . . . 60
9L2: Stratified by Household food insecurity . . . 61
9L3: Stratified by Household source water quality . . . 62
9L4: Stratified by Household sanitation (insufficient comparisons) . . . 63
9L5: Stratified by Season at the time of assessment . . . 64

Supplemental figure 9M: Elevated soluble transferrin receptor prevalence difference **65**
9M1: Stratified by Household socio-economic status . . . 65
9M2: Stratified by Household food insecurity . . . 66
9M3: Stratified by Household source water quality . . . 67
9M4: Stratified by Household sanitation (insufficient comparisons) . . . 68
9M5: Stratified by Season at the time of assessment . . . 69

Supplemental figure 9N: Geometric mean ratio of zinc protoporphyrin concentration **70**
9N1: Stratified by Household socio-economic status . . . 70
9N2: Stratified by Household food insecurity . . . 71

9N3: Stratified by Household source water quality (insufficient comparisons) . . . 72
9N4: Stratified by Household sanitation (insufficient comparisons) . . . 73
9N5: Stratified by Season at the time of assessment . . . 74

Supplemental figure 9O: Elevated zinc protoporphyrin prevalence ratio **75**
9O1: Stratified by Household socio-economic status . . . 75
9O2: Stratified by Household food insecurity . . . 76
9O3: Stratified by Household source water quality (insufficient comparisons) . . . 77
9O4: Stratified by Household sanitation (insufficient comparisons) . . . 78
9O5: Stratified by Season at the time of assessment . . . 79

Supplemental figure 9P: Elevated zinc protoporphyrin prevalence difference **80**
9P1: Stratified by Household socio-economic status . . . 80
9P2: Stratified by Household food insecurity . . . 81
9P3: Stratified by Household source water quality (insufficient comparisons) . . . 82
9P4: Stratified by Household sanitation (insufficient comparisons) . . . 83
9P5: Stratified by Season at the time of assessment . . . 84

Supplemental figure 9Q: Geometric mean ratio of plasma zinc concentration **85**
9Q1: Stratified by Household socio-economic status . . . 85
9Q2: Stratified by Household food insecurity (insufficient comparisons) . . . 86
9Q3: Stratified by Household source water quality (insufficient comparisons) . . . 87
9Q4: Stratified by Household sanitation (insufficient comparisons) . . . 88
9Q5: Stratified by Season at the time of assessment . . . 89

Supplemental figure 9R: Geometric mean ratio of retinol concentration **90**
9R1: Stratified by Household socio-economic status . . . 90
9R2: Stratified by Household food insecurity . . . 91
9R3: Stratified by Household source water quality (insufficient comparisons) . . . 92
9R4: Stratified by Household sanitation (insufficient comparisons) . . . 93
9R5: Stratified by Season at the time of assessment . . . 94

Supplemental figure 9S: Low vitamin A (retinol < 0.70 µmol/L) prevalence ratio **95**
9S1: Stratified by Household socio-economic status (insufficient comparisons) . . . 95
9S2: Stratified by Household food insecurity (insufficient comparisons) . . . 96
9S3: Stratified by Household source water quality (insufficient comparisons) . . . 97
9S4: Stratified by Household sanitation (insufficient comparisons) . . . 98
9S5: Stratified by Season at the time of assessment (insufficient comparisons) . . . 99

Supplemental figure 9T: Low vitamin A (retinol < 0.70 µmol/L) prevalence difference **100**
9T1: Stratified by Household socio-economic status (insufficient comparisons) . . . 100
9T2: Stratified by Household food insecurity (insufficient comparisons) . . . 101
9T3: Stratified by Household source water quality (insufficient comparisons) . . . 102
9T4: Stratified by Household sanitation (insufficient comparisons) . . . 103
9T5: Stratified by Season at the time of assessment (insufficient comparisons) . . . 104

Supplemental figure 9U: Marginal vitamin A (retinol < 1.05 µmol/L) prevalence ratio **105**
9U1: Stratified by Household socio-economic status . . . 105
9U2: Stratified by Household food insecurity (insufficient comparisons) . . . 106
9U3: Stratified by Household source water quality (insufficient comparisons) . . . 107
9U4: Stratified by Household sanitation (insufficient comparisons) . . . 108
9U5: Stratified by Season at the time of assessment . . . 109

Supplemental figure 9V: Marginal vitamin A (retinol < 1.05 µmol/L) prevalence difference **110**
9V1: Stratified by Household socio-economic status . . . 110
9V2: Stratified by Household food insecurity (insufficient comparisons) . . . 111
9V3: Stratified by Household source water quality (insufficient comparisons) . . . 112
9V4: Stratified by Household sanitation (insufficient comparisons) . . . 113
9V5: Stratified by Season at the time of assessment . . . 114

Supplemental figure 9W: Geometric mean ratio of retinol binding protein concentration **115**
9W1: Stratified by Household socio-economic status . . . 115
9W2: Stratified by Household food insecurity . . . 116
9W3: Stratified by Household source water quality . . . 117
9W4: Stratified by Household sanitation (insufficient comparisons) . . . 118
9W5: Stratified by Season at the time of assessment . . . 119

Supplemental figure 9X: Low vitamin A status (RBP < 0.70 µmol/L) prevalence ratio **120**
9X1: Stratified by Household socio-economic status (insufficient comparisons) . . . 120
9X2: Stratified by Household food insecurity (insufficient comparisons) . . . 121
9X3: Stratified by Household source water quality (insufficient comparisons) . . . 122
9X4: Stratified by Household sanitation (insufficient comparisons) . . . 123
9X5: Stratified by Season at the time of assessment (insufficient comparisons) . . . 124

Supplemental figure 9Y: Low vitamin A status (RBP < 0.70 µmol/L) prevalence difference **125**
9Y1: Stratified by Household socio-economic status (insufficient comparisons) . . . 125
9Y2: Stratified by Household food insecurity (insufficient comparisons) . . . 126
9Y3: Stratified by Household source water quality (insufficient comparisons) . . . 127
9Y4: Stratified by Household sanitation (insufficient comparisons) . . . 128
9Y5: Stratified by Season at the time of assessment (insufficient comparisons) . . . 129

Supplemental figure 9Z: Marginal vitamin A status (RBP < 1.05 µmol/L) prevalence ratio **130**
9Z1: Stratified by Household socio-economic status . . . 130
9Z2: Stratified by Household food insecurity . . . 131
9Z3: Stratified by Household source water quality . . . 132
9Z4: Stratified by Household sanitation (insufficient comparisons) . . . 133
9Z5: Stratified by Season at the time of assessment . . . 134

Supplemental figure 9AA: Marginal vitamin A status (RBP < 1.05 µmol/L) prevalence difference **135**
9AA1: Stratified by Household socio-economic status . . . 135
9AA2: Stratified by Household food insecurity . . . 136
9AA3: Stratified by Household source water quality . . . 137
9AA4: Stratified by Household sanitation (insufficient comparisons) . . . 138
9AA5: Stratified by Season at the time of assessment . . . 139

Supplemental figure 9: Forest plots for effects of SQ-LNS on biochemical outcomes stratified by individual-level household effect modifiers

These figures are forest plots showing the individual-level effect modification of intervention effects. Each figure has the estimates of intervention effect stratified within study by individual-level effect modifier category. For definitions of effect modifiers, see Box 1 in the main paper. Individual study estimates were generated from log-binomial regression for dichotomous outcomes and simple linear regression for continuous outcomes; controlling for baseline measure when available and with clustered observations using robust standard errors for cluster-randomized trials. Pooled interaction term and sub-group estimates were generated using inverse-variance weighting fixed and random effects. For continuous outcomes the intervention effect is measured by the difference in mean of the LNS group minus control. For log transformed continuous outcomes, the intervention effect is measured by the ratio of geometric means, the effect estimate is the geometric mean in the LNS group divided by the geometric mean in the control group. For dichotomous outcomes analyzed via prevalence ratios, the effect estimate is the prevalence in the LNS group divided by the prevalence in the control group. For dichotomous outcomes analyzed via prevalence differences, the effect estimate is the prevalence in the LNS group minus the prevalence in the control group.

The labels on the far left correspond to trial level information. In the middle left and on the right the values indicate the study level effect estimate, confidence interval, and weighting for deriving the pooled estimates is shown by subgroup.

Not all trials were included in all individual-level effect modification analyses, either because they did not measure the biomarker outcome or the effect modifier of interest (e.g., baseline anemia or acute malnutrition, receipt of high-dose vitamin A supplement), or because the prevalence of the binary outcome or proportion of children within one of the effect modifier subgroups was too low to allow us to generate effect estimates. If fewer than three studies contribute to a pooled estimate then the pooled estimate was not generated (e.g. if fewer than 3 studies are categorized into a study level effect modification category), and this is labeled as “insufficient comparisons”.

Ferritin, sTfR, ZPP, zinc, retinol and RBP concentrations were adjusted for inflammation (i.e., C-reactive protein (CRP) and/or α-1-acid glycoprotein (AGP) concentrations, as available), using a regression correction approach adapted from the Biomarkers Reflecting Inflammation and Nutritional Determinants of Anemia (BRINDA) project (28)

RBP, retinol binding protein.

Supplemental figure 9A: Mean difference in hemoglobin concentration

9A1: Stratified by Household socio-economic status

P−for−interaction = 0.528
Difference in MDs = −0.26 (−1.07, 0.55)

At least median

Country	Trial	LNS N	Control N	Control Mean	MD (95% CI)	Fixed W	Random W
Bangladesh	JiVitA−4 (35)	244	79	117.1	3.51 (1.31, 5.71)	0.08	0.08
Bangladesh	RDNS (36)	272	136	112.2	5.44 (2.82, 8.05)	0.06	0.08
Bangladesh	WASH−B (37)	114	93	117.5	4.52 (1.84, 7.20)	0.05	0.07
Burkina Faso	iLiNS−Zinc (38)	1232	299	88.9	8.35 (5.76, 10.95)	0.06	0.08
Burkina Faso	PROMIS CS (39)	289	293	103.1	1.10 (−1.85, 4.06)	0.04	0.07
Ghana	GHANA (40)	57	44	109.4	6.35 (1.27, 11.42)	0.02	0.04
Ghana	iLiNS−DYAD−G (41)	149	344	113.3	0.24 (−1.66, 2.15)	0.11	0.09
Kenya	WASH−B (42)	190	175	109.5	4.56 (2.31, 6.81)	0.08	0.08
Madagascar	MAHAY (43)	278	311	104.6	4.08 (0.92, 7.24)	0.04	0.06
Malawi	iLiNS−DYAD−M (44)	117	225	109.0	2.44 (−0.97, 5.86)	0.03	0.06
Malawi	iLiNS−DOSE (45)	86	32	99.9	7.41 (1.69, 13.13)	0.01	0.03
Mali	PROMIS CS (46)	505	463	96.8	5.61 (3.44, 7.78)	0.08	0.08
Zimbabwe	SHINE (HIV−) (47)	821	722	114.2	2.19 (1.05, 3.32)	0.30	0.11
Zimbabwe	SHINE (HIV+) (48)	155	138	115.0	3.00 (−0.27, 6.28)	0.04	0.06
		**4509**	**3354**		**I² = 0.70, Tau² = 3.18**		
**Fixed**					**3.41 (2.78, 4.03)**		
**Random**					**3.93 (2.73, 5.13)**		

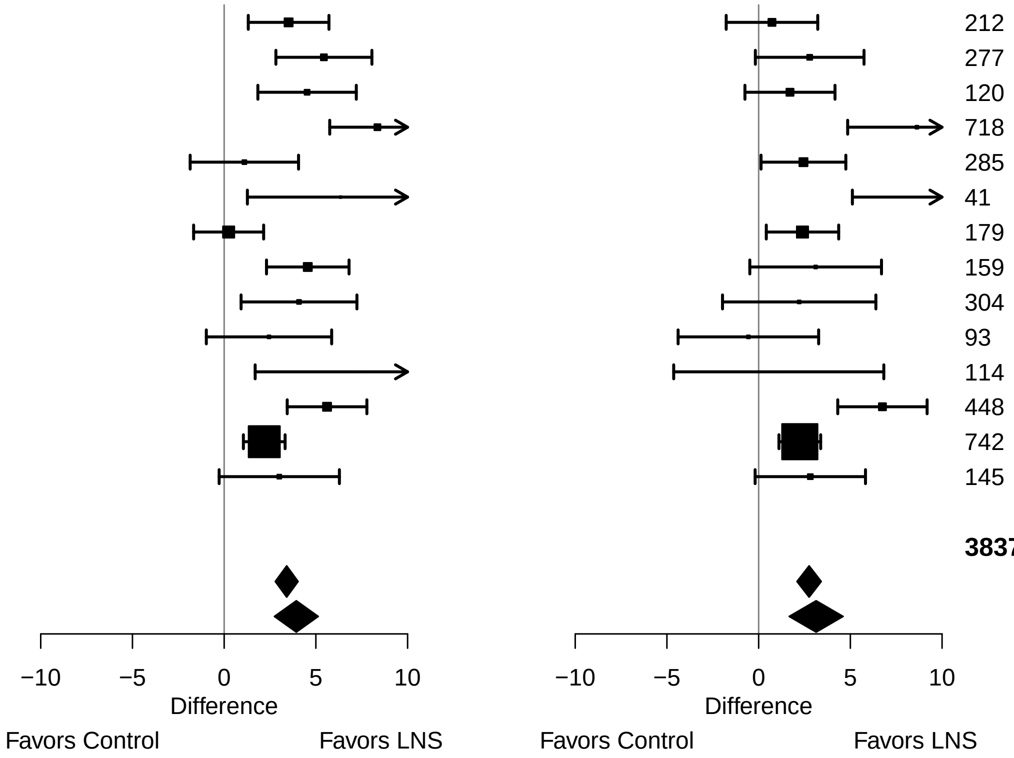

Less than median

Country	Trial	LNS N	Control N	Control Mean	MD (95% CI)	Fixed W	Random W
Bangladesh	JiVitA−4 (35)	212	67	119.0	0.73 (−1.77, 3.22)	0.07	0.08
Bangladesh	RDNS (36)	277	136	112.6	2.78 (−0.18, 5.74)	0.05	0.07
Bangladesh	WASH−B (37)	120	93	118.7	1.71 (−0.75, 4.16)	0.07	0.08
Burkina Faso	iLiNS−Zinc (38)	718	364	88.3	8.63 (4.85, 12.40)	0.03	0.06
Burkina Faso	PROMIS CS (39)	285	288	102.1	2.44 (0.13, 4.75)	0.08	0.08
Ghana	GHANA (40)	41	46	101.6	11.25 (5.11, 17.40)	0.01	0.04
Ghana	iLiNS−DYAD−G (41)	179	315	110.5	2.39 (0.42, 4.36)	0.11	0.09
Kenya	WASH−B (42)	159	125	110.3	3.11 (−0.48, 6.70)	0.03	0.07
Madagascar	MAHAY (43)	304	262	103.2	2.21 (−1.97, 6.39)	0.03	0.06
Malawi	iLiNS−DYAD−M (44)	93	206	106.8	−0.56 (−4.39, 3.27)	0.03	0.06
Malawi	iLiNS−DOSE (45)	114	39	101.8	1.10 (−4.63, 6.82)	0.01	0.04
Mali	PROMIS CS (46)	448	507	94.8	6.75 (4.31, 9.18)	0.07	0.08
Zimbabwe	SHINE (HIV−) (47)	742	760	114.4	2.24 (1.11, 3.38)	0.34	0.10
Zimbabwe	SHINE (HIV+) (48)	145	140	114.4	2.82 (−0.19, 5.83)	0.05	0.07
		**3837**	**3348**		**I² = 0.62, Tau² = 5.27**		
**Fixed**					**2.75 (2.09, 3.42)**		
**Random**					**3.14 (1.66, 4.61)**		

Supplemental figure 9A: Mean difference in hemoglobin concentration

9A2: Stratified by Household food insecurity

P−for−interaction = 0.470
Difference in MDs = 0.40 (−0.68, 1.48)

		Mild to secure						Moderate to severe					
Country	Trial	LNS N	Control N	Control Mean	MD (95% CI)	Fixed W	Random W	LNS N	Control N	Control Mean	MD (95% CI)	Fixed W	Random W
Bangladesh	JiVitA−4 (35)	339	113	118.0	2.27 (−0.02, 4.55)	0.07	0.10	117	33	117.8	2.15 (−0.82, 5.13)	0.10	0.11
Bangladesh	RDNS (36)	342	166	113.7	2.94 (−0.01, 5.89)	0.04	0.09	207	106	110.4	5.91 (2.94, 8.88)	0.10	0.11
Bangladesh	WASH−B (37)	190	145	118.6	2.75 (0.55, 4.95)	0.07	0.11	44	41	116.4	4.22 (0.41, 8.03)	0.06	0.08
Burkina Faso	iLiNS−Zinc (38)	1016	320	87.4	8.87 (6.02, 11.73)	0.04	0.09	934	343	89.6	8.22 (4.52, 11.92)	0.07	0.09
Burkina Faso	PROMIS CS (39)												
Ghana	GHANA (40)												
Ghana	iLiNS−DYAD−G (41)	230	457	111.6	1.02 (−0.56, 2.61)	0.14	0.12	97	201	112.6	1.91 (−0.81, 4.63)	0.12	0.11
Kenya	WASH−B (42)	304	273	109.7	4.27 (2.23, 6.31)	0.08	0.11	45	27	111.1	1.18 (−4.08, 6.44)	0.03	0.06
Madagascar	MAHAY (43)	359	340	101.8	5.00 (1.79, 8.20)	0.03	0.08	137	140	105.6	−0.76 (−5.57, 4.05)	0.04	0.06
Malawi	iLiNS−DYAD−M (44)	66	113	105.3	4.85 (−0.06, 9.75)	0.01	0.05	143	314	109.0	−0.21 (−3.23, 2.82)	0.10	0.10
Malawi	iLiNS−DOSE (45)	50	21	101.6	2.53 (−5.96, 11.02)	0.00	0.02	144	46	101.0	3.24 (−1.69, 8.18)	0.04	0.06
Mali	PROMIS CS (46)												
Zimbabwe	SHINE (HIV−) (47)	1253	1162	114.5	1.76 (0.88, 2.65)	0.45	0.14	264	293	113.9	3.53 (1.78, 5.28)	0.29	0.15
Zimbabwe	SHINE (HIV+) (48)	217	204	114.6	2.60 (−0.03, 5.24)	0.05	0.09	75	66	114.9	3.90 (−0.54, 8.34)	0.05	0.07
		**4366**	**3314**		**I² = 0.68, Tau² = 2.85**			**2207**	**1610**		**I² = 0.50, Tau² = 3.19**		
**Fixed**					**2.53 (1.94, 3.12)**						**3.18 (2.23, 4.13)**		
**Random**					**3.32 (2.03, 4.62)**						**3.13 (1.63, 4.63)**		

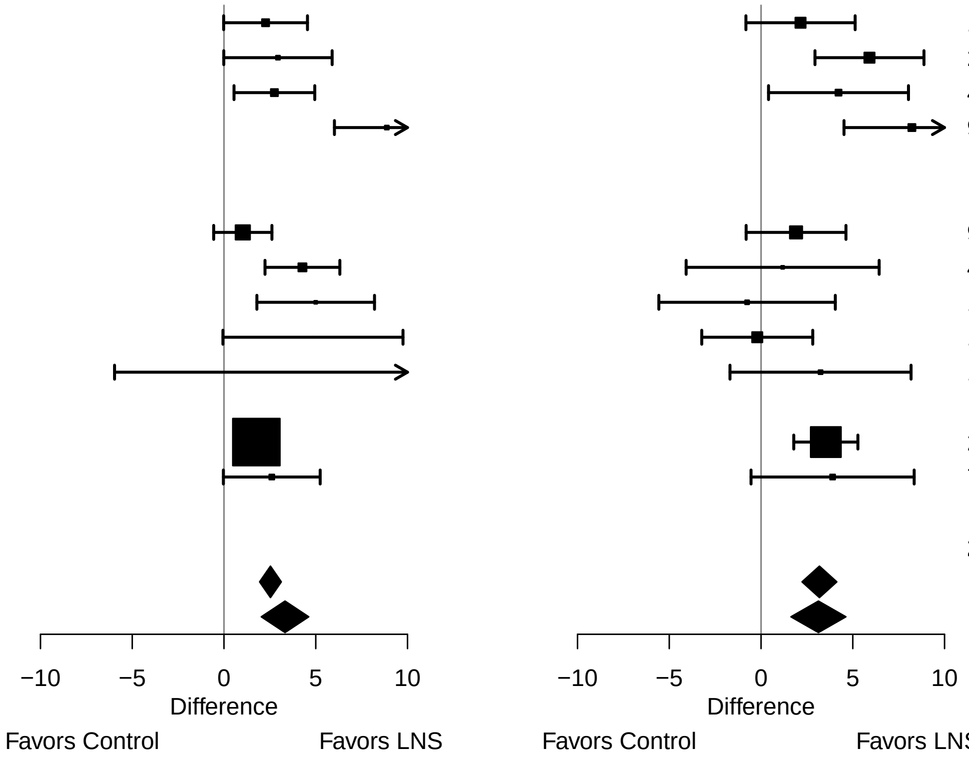

Supplemental figure 9A: Mean difference in hemoglobin concentration

9A3: Stratified by Household source water quality

P−for−interaction = 0.746
Difference in MDs = −0.21 (−1.49, 1.07)

Country	Trial	Improved LNS N	Improved Control N	Improved Control Mean	Improved MD (95% CI)	Improved Fixed W	Improved Random W	Unimproved LNS N	Unimproved Control N	Unimproved Control Mean	Unimproved MD (95% CI)	Unimproved Fixed W	Unimproved Random W
Bangladesh	JiVitA−4 (35)												
Bangladesh	RDNS (36)												
Bangladesh	WASH−B (37)	84	46	118.5	3.76 (0.24, 7.28)	0.07	0.11	21	14	118.8	1.26 (−4.36, 6.89)	0.03	0.06
Burkina Faso	iLiNS−Zinc (38)	555	141	86.9	11.38 (6.98, 15.77)	0.04	0.09	1394	519	89.0	7.53 (4.38, 10.68)	0.11	0.13
Burkina Faso	PROMIS CS (39)	373	363	102.8	1.35 (−0.79, 3.48)	0.18	0.13	201	217	102.3	2.49 (−0.34, 5.31)	0.13	0.14
Ghana	GHANA (40)												
Ghana	iLiNS−DYAD−G (41)												
Kenya	WASH−B (42)	128	121	108.8	3.46 (0.26, 6.67)	0.08	0.11	68	41	113.5	1.45 (−2.98, 5.89)	0.05	0.08
Madagascar	MAHAY (43)	137	164	105.0	0.74 (−4.25, 5.74)	0.03	0.08	433	408	103.5	3.80 (0.70, 6.90)	0.11	0.13
Malawi	iLiNS−DYAD−M (44)	191	397	108.5	0.34 (−2.33, 3.01)	0.12	0.12	19	33	101.5	10.72 (1.84, 19.59)	0.01	0.03
Malawi	iLiNS−DOSE (45)												
Mali	PROMIS CS (46)	548	567	95.3	6.90 (4.34, 9.45)	0.13	0.12	364	388	96.3	5.43 (2.66, 8.20)	0.14	0.14
Zimbabwe	SHINE (HIV−) (47)	469	436	114.6	2.18 (0.55, 3.81)	0.31	0.14	259	281	114.4	2.25 (0.53, 3.97)	0.35	0.19
Zimbabwe	SHINE (HIV+) (48)	77	77	115.0	1.05 (−3.12, 5.22)	0.05	0.10	49	46	114.7	2.36 (−1.74, 6.47)	0.06	0.09
		**2562**	**2312**		**I^2 = 0.75, Tau^2 = 8.97**			**2808**	**1947**		**I^2 = 0.46, Tau^2 = 2.60**		
**Fixed**					**2.91 (2.00, 3.82)**						**3.49 (2.46, 4.51)**		
**Random**					**3.37 (1.13, 5.61)**						**3.73 (2.14, 5.32)**		

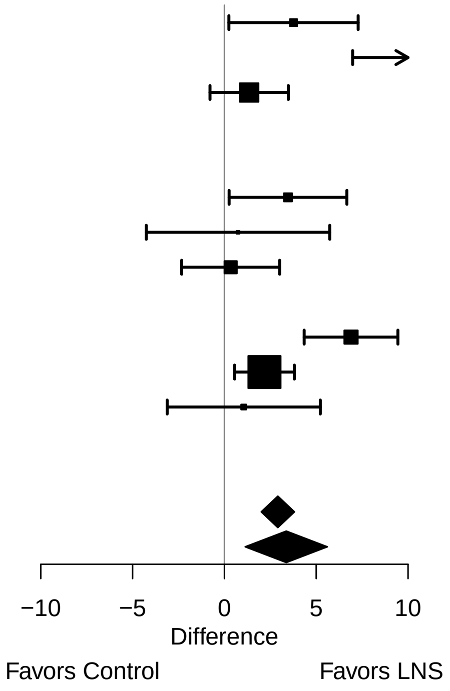

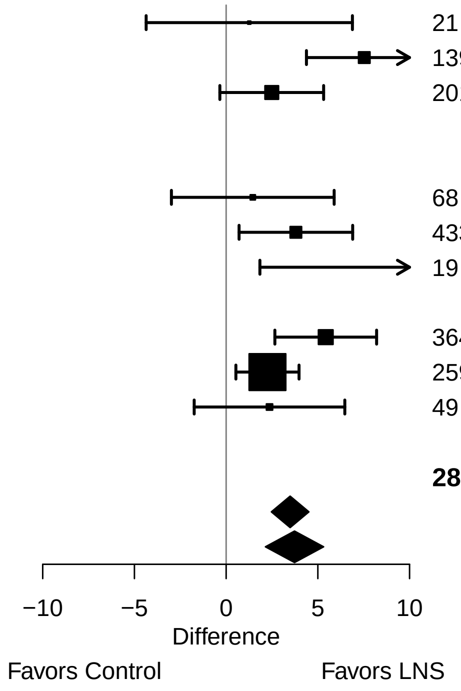

Supplemental figure 9A: Mean difference in hemoglobin concentration

9A4: Stratified by Household sanitation

P−for−interaction = 0.607
Difference in MDs = 0.32 (−0.89, 1.52)

Country	Trial	Improved LNS N	Improved Control N	Improved Control Mean	Improved MD (95% CI)	Improved Fixed W	Improved Random W	Unimproved LNS N	Unimproved Control N	Unimproved Control Mean	Unimproved MD (95% CI)	Unimproved Fixed W	Unimproved Random W
Bangladesh	JiVitA−4 (35)	363	114	117.6	3.14 (1.07, 5.21)	0.14	0.13	93	32	119.5	−1.19 (−4.97, 2.59)	0.05	0.09
Bangladesh	RDNS (36)	376	193	112.9	3.74 (1.16, 6.33)	0.09	0.12	173	79	111.2	5.01 (2.00, 8.01)	0.08	0.10
Bangladesh	WASH−B (37)												
Burkina Faso	iLiNS−Zinc (38)	43	16	88.3	9.64 (4.27, 15.01)	0.02	0.06	1906	644	88.6	8.46 (5.37, 11.55)	0.08	0.10
Burkina Faso	PROMIS CS (39)	244	229	103.0	1.15 (−1.14, 3.44)	0.11	0.13	329	350	102.3	2.15 (−0.10, 4.40)	0.15	0.11
Ghana	GHANA (40)												
Ghana	iLiNS−DYAD−G (41)	317	642	112.0	1.22 (−0.18, 2.62)	0.30	0.15	10	17	109.8	2.74 (−4.84, 10.31)	0.01	0.05
Kenya	WASH−B (42)	24	25	113.2	5.17 (−1.20, 11.55)	0.01	0.04	172	137	109.4	3.07 (0.10, 6.03)	0.08	0.10
Madagascar	MAHAY (43)												
Malawi	iLiNS−DYAD−M (44)	20	39	107.3	9.44 (0.08, 18.80)	0.01	0.02	190	391	108.1	0.32 (−2.34, 2.97)	0.11	0.11
Malawi	iLiNS−DOSE (45)												
Mali	PROMIS CS (46)	703	696	95.7	5.44 (3.17, 7.70)	0.11	0.13	219	249	96.1	8.80 (5.89, 11.71)	0.09	0.11
Zimbabwe	SHINE (HIV−) (47)	262	218	114.3	1.68 (−0.08, 3.44)	0.19	0.14	465	498	114.5	2.57 (0.97, 4.18)	0.29	0.12
Zimbabwe	SHINE (HIV+) (48)	46	30	114.1	3.56 (−1.06, 8.18)	0.03	0.07	80	92	115.3	0.40 (−3.07, 3.87)	0.06	0.10
		**2398**	**2202**		**I^2 = 0.61, Tau^2 = 3.47**			**3637**	**2489**		**I^2 = 0.78, Tau^2 = 8.45**		
**Fixed**					**2.60 (1.84, 3.37)**						**3.19 (2.33, 4.05)**		
**Random**					**3.38 (1.84, 4.92)**						**3.29 (1.22, 5.37)**		

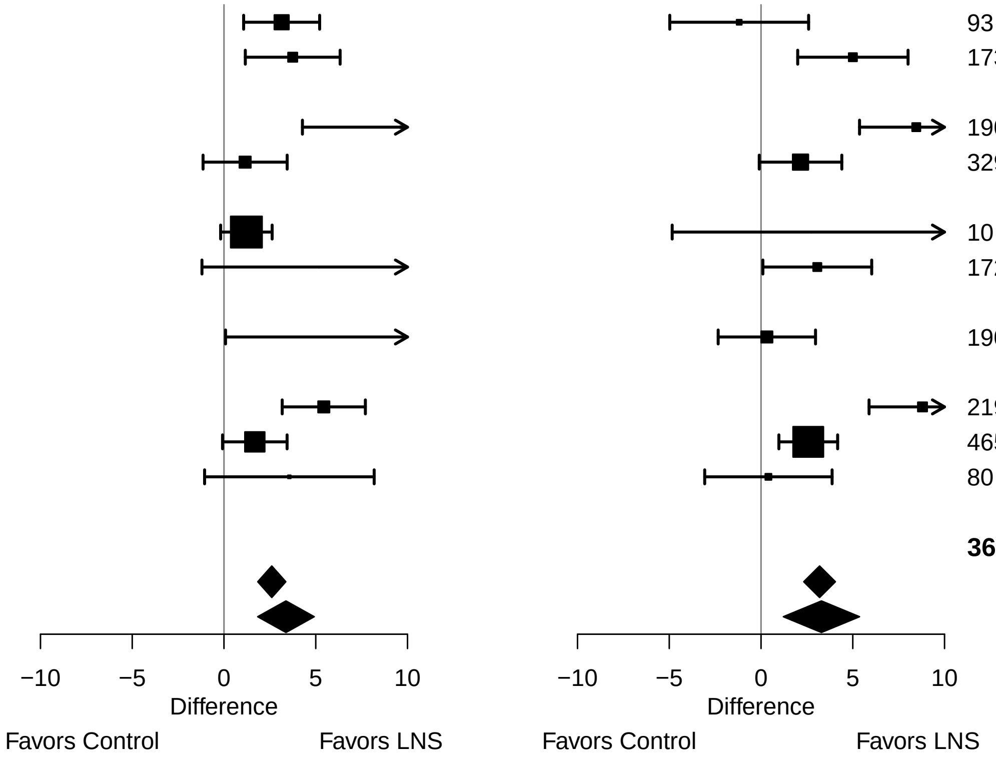

Supplemental figure 9A: Mean difference in hemoglobin concentration

9A5: Stratified by Season at the time of assessment

P−for−interaction = 0.190
Difference in MDs = −0.69 (−1.72, 0.34)

		**Dry**						**Rainy**					
Country	Trial	**LNS** N	**Control** N	**Control** Mean	**MD** (95% CI)	**Fixed** W	**Random** W	**LNS** N	**Control** N	**Control** Mean	**MD** (95% CI)	**Fixed** W	**Random** W
Bangladesh	JiVitA−4 (35)	264	72	118.6	1.35 (−1.44, 4.14)	0.04	0.08	193	74	117.4	3.14 (0.91, 5.37)	0.14	0.10
Bangladesh	RDNS (36)	341	166	110.5	5.65 (3.05, 8.25)	0.05	0.08	208	106	115.4	1.70 (−2.06, 5.46)	0.05	0.07
Bangladesh	WASH−B (37)	108	96	118.5	2.49 (0.08, 4.90)	0.06	0.09	126	90	117.7	3.70 (0.94, 6.46)	0.09	0.09
Burkina Faso	iLiNS−Zinc (38)	1398	475	88.6	8.74 (5.82, 11.65)	0.04	0.08	559	189	88.5	7.76 (3.68, 11.85)	0.04	0.07
Burkina Faso	PROMIS CS (39)	283	245	102.1	2.36 (−0.38, 5.09)	0.04	0.08	291	336	103.0	1.32 (−1.02, 3.66)	0.13	0.10
Ghana	GHANA (40)	57	55	105.7	9.59 (4.43, 14.75)	0.01	0.04	41	41	105.7	7.79 (1.53, 14.05)	0.02	0.04
Ghana	iLiNS−DYAD−G (41)	198	386	111.8	1.55 (−0.21, 3.31)	0.11	0.10	130	274	112.2	0.76 (−1.46, 2.97)	0.14	0.11
Kenya	WASH−B (42)	100	88	111.7	3.74 (0.76, 6.71)	0.04	0.08	250	212	109.0	4.05 (1.79, 6.32)	0.13	0.10
Madagascar	MAHAY (43)	517	537	103.3	2.04 (−0.87, 4.95)	0.04	0.08	83	51	110.9	4.51 (−0.26, 9.29)	0.03	0.06
Malawi	iLiNS−DYAD−M (44)	147	305	109.2	1.95 (−1.01, 4.91)	0.04	0.08	63	125	105.1	−0.61 (−5.53, 4.31)	0.03	0.05
Malawi	iLiNS−DOSE (45)	123	32	101.8	−0.23 (−6.20, 5.74)	0.01	0.04	120	50	99.8	6.93 (2.14, 11.72)	0.03	0.06
Mali	PROMIS CS (46)												
Zimbabwe	SHINE (HIV−) (47)	1558	1477	114.2	2.35 (1.53, 3.18)	0.48	0.11	124	117	115.5	−0.63 (−2.74, 1.47)	0.16	0.11
Zimbabwe	SHINE (HIV+) (48)	289	265	115.0	2.69 (0.14, 5.25)	0.05	0.08	17	20	111.2	3.26 (−2.80, 9.32)	0.02	0.04
		**5383**	**4199**		**I^2 = 0.64, Tau^2 = 4.40**			**2205**	**1685**		**I^2 = 0.59, Tau^2 = 3.95**		
**Fixed**					**2.75 (2.17, 3.32)**						**2.41 (1.58, 3.24)**		
**Random**					**3.27 (1.88, 4.66)**						**2.92 (1.46, 4.37)**		

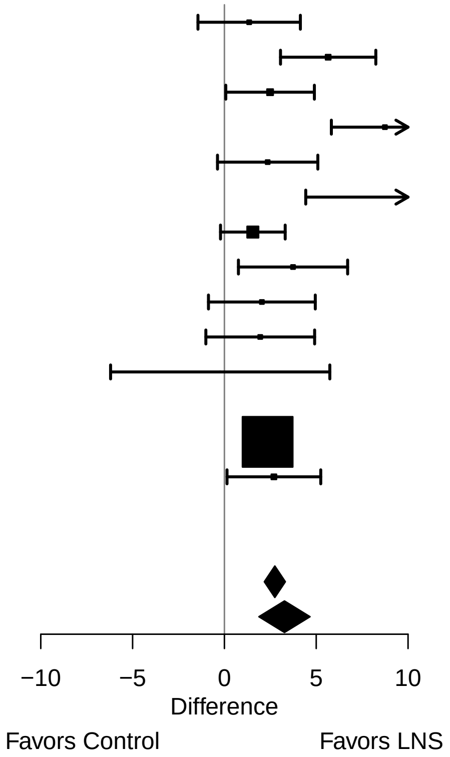

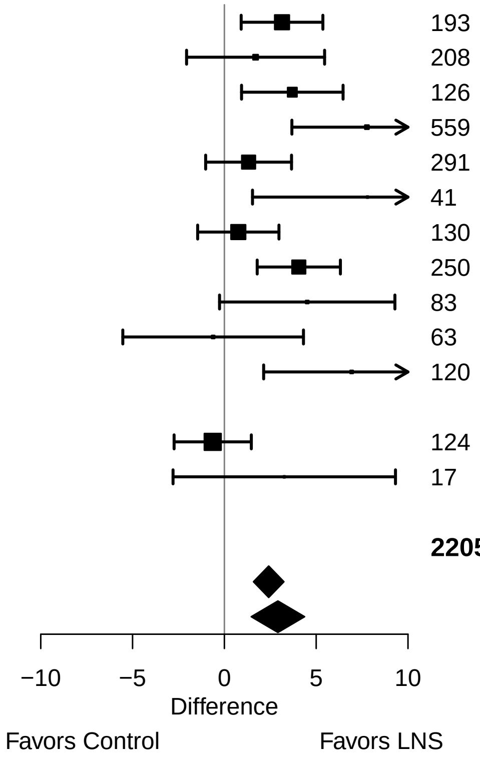

Supplemental figure 9B: Anemia prevalence ratio

9B1: Stratified by Household socio-economic status

P−for−interaction = 0.762
Ratio of PRs = 0.99 (0.95, 1.04)

		At least median							Less than median						
Country	Trial	LNS N	Control N	Control Prevalence	PR (95% CI)	Fixed W	Random W		LNS N	Control N	Control Prevalence	PR (95% CI)	Fixed W	Random W
Bangladesh	JiVitA−4 (35)	244	79	17.7	0.62 (0.37, 1.05)	0.01	0.02		212	67	13.4	1.16 (0.64, 2.10)	0.00	0.02
Bangladesh	RDNS (36)	272	136	41.9	0.57 (0.40, 0.81)	0.01	0.04		277	136	41.9	0.74 (0.56, 0.97)	0.02	0.06
Bangladesh	WASH−B (37)													
Burkina Faso	iLiNS−Zinc (38)	1232	299	90.0	0.89 (0.84, 0.95)	0.44	0.17		718	364	92.0	0.84 (0.79, 0.89)	0.40	0.13
Burkina Faso	PROMIS CS (39)	289	293	70.0	0.93 (0.80, 1.08)	0.07	0.11		285	288	70.1	0.94 (0.85, 1.04)	0.16	0.12
Ghana	GHANA (40)	57	44	45.5	0.58 (0.34, 1.00)	0.01	0.02		41	46	71.7	0.48 (0.30, 0.76)	0.01	0.03
Ghana	iLiNS−DYAD−G (41)	149	344	41.6	0.89 (0.70, 1.13)	0.03	0.07		179	315	48.6	0.83 (0.67, 1.02)	0.03	0.08
Kenya	WASH−B (42)	190	175	48.0	0.65 (0.49, 0.85)	0.02	0.06		159	125	46.4	0.73 (0.53, 1.00)	0.01	0.05
Madagascar	MAHAY (43)	278	311	64.0	0.79 (0.65, 0.95)	0.04	0.09		304	262	65.6	0.96 (0.81, 1.13)	0.06	0.10
Malawi	iLiNS−DYAD−M (44)	117	225	48.4	0.83 (0.64, 1.07)	0.02	0.06		93	206	55.8	0.98 (0.79, 1.23)	0.03	0.08
Malawi	iLiNS−DOSE (45)	86	32	78.1	0.71 (0.55, 0.93)	0.02	0.06		114	39	61.5	1.08 (0.84, 1.40)	0.02	0.07
Mali	PROMIS CS (46)	505	463	85.7	0.82 (0.75, 0.89)	0.23	0.15		448	507	86.6	0.82 (0.75, 0.89)	0.18	0.12
Zimbabwe	SHINE (HIV−) (47)	821	722	35.3	0.73 (0.64, 0.84)	0.08	0.12		742	760	34.9	0.78 (0.68, 0.91)	0.07	0.10
Zimbabwe	SHINE (HIV+) (48)	155	138	34.1	0.61 (0.41, 0.90)	0.01	0.03		145	140	40.0	0.66 (0.46, 0.93)	0.01	0.05
		**4395**	**3261**		**I² = 0.54, Tau² = 0.01**				**3717**	**3255**		**I² = 0.51, Tau² = 0.02**		
**Fixed**					**0.83 (0.80, 0.87)**							**0.85 (0.82, 0.89)**		
**Random**					**0.79 (0.73, 0.85)**							**0.85 (0.77, 0.93)**		

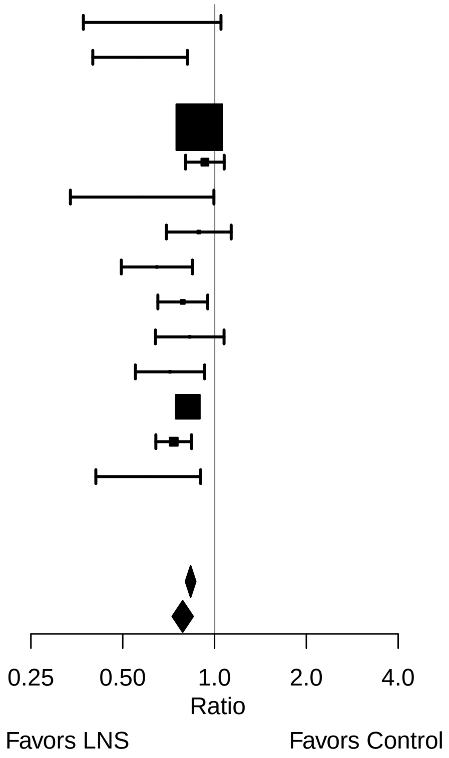

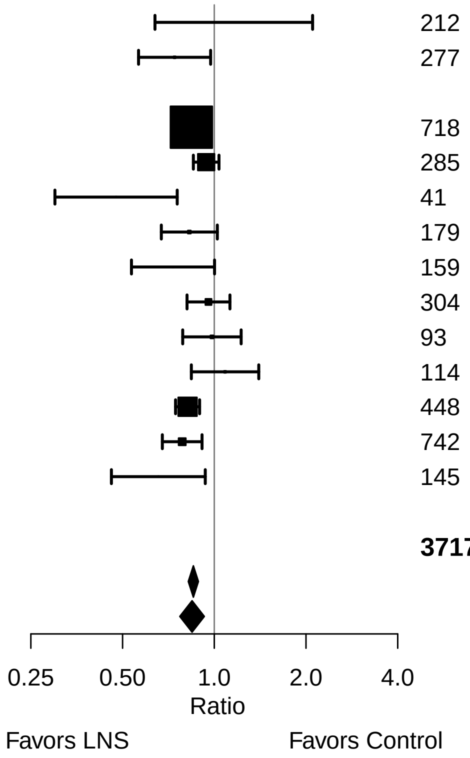

Supplemental figure 9B: Anemia prevalence ratio

9B2: Stratified by Household food insecurity

P−for−interaction = 0.949
Ratio of PRs = 1.00 (0.95, 1.05)

Country	Trial	LNS N	Control N	Control Prevalence	Mild to secure PR (95% CI)	Fixed W	Random W	LNS N	Control N	Control Prevalence	Moderate to severe PR (95% CI)	Fixed W	Random W
Bangladesh	JiVitA−4 (35)												
Bangladesh	RDNS (36)	342	166	40.4	0.65 (0.46, 0.92)	0.01	0.06	207	106	44.3	0.66 (0.51, 0.87)	0.04	0.11
Bangladesh	WASH−B (37)												
Burkina Faso	iLiNS−Zinc (38)	1016	320	91.9	0.87 (0.83, 0.92)	0.61	0.20	934	343	90.4	0.87 (0.82, 0.92)	0.71	0.20
Burkina Faso	PROMIS CS (39)												
Ghana	GHANA (40)												
Ghana	iLiNS−DYAD−G (41)	230	457	47.0	0.88 (0.73, 1.05)	0.05	0.12	97	201	40.8	0.81 (0.58, 1.12)	0.02	0.09
Kenya	WASH−B (42)	304	273	48.7	0.65 (0.54, 0.78)	0.05	0.12	45	27	33.3	1.13 (0.65, 1.96)	0.01	0.04
Madagascar	MAHAY (43)	359	340	69.4	0.83 (0.72, 0.95)	0.09	0.15	137	140	63.6	1.01 (0.82, 1.24)	0.06	0.14
Malawi	iLiNS−DYAD−M (44)	66	113	58.4	0.78 (0.57, 1.06)	0.02	0.07	143	314	50.0	0.94 (0.76, 1.15)	0.06	0.14
Malawi	iLiNS−DOSE (45)	50	21	61.9	1.12 (0.78, 1.61)	0.01	0.05	144	46	69.6	0.87 (0.69, 1.09)	0.05	0.13
Mali	PROMIS CS (46)												
Zimbabwe	SHINE (HIV−) (47)	1253	1162	34.5	0.80 (0.71, 0.90)	0.13	0.16	264	293	36.9	0.61 (0.47, 0.79)	0.04	0.11
Zimbabwe	SHINE (HIV+) (48)	217	204	35.3	0.64 (0.47, 0.87)	0.02	0.07	75	66	42.4	0.60 (0.36, 1.00)	0.01	0.05
		**3837**	**3056**		**I² = 0.55, Tau² = 0.01**			**2046**	**1536**		**I² = 0.51, Tau² = 0.02**		
**Fixed**					**0.84 (0.80, 0.87)**						**0.86 (0.81, 0.90)**		
**Random**					**0.80 (0.72, 0.88)**						**0.83 (0.73, 0.94)**		

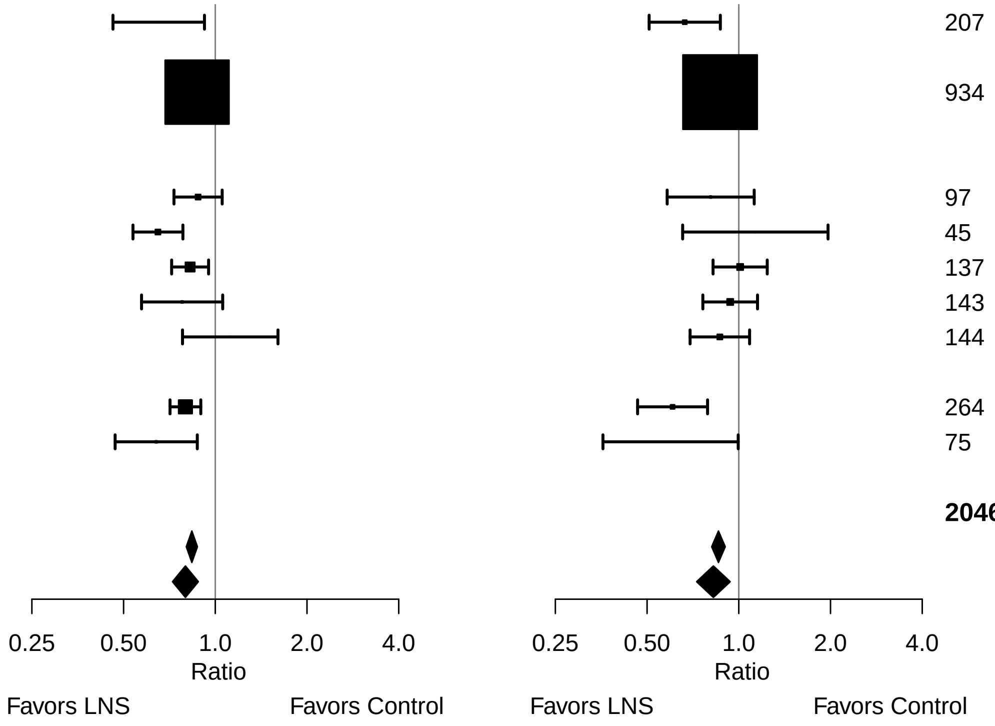

Supplemental figure 9B: Anemia prevalence ratio

9B3: Stratified by Household source water quality

P−for−interaction = 0.009
Ratio of PRs = 1.08 (1.02, 1.14)

Country	Trial	LNS N	Control N	Control Prevalence	Improved PR (95% CI)	Fixed W	Random W	LNS N	Control N	Control Prevalence	Unimproved PR (95% CI)	Fixed W	Random W
Bangladesh	JiVitA−4 (35)												
Bangladesh	RDNS (36)												
Bangladesh	WASH−B (37)												
Burkina Faso	iLiNS−Zinc (38)	555	141	96.5	0.80 (0.76, 0.85)	0.51	0.30	1394	519	89.6	0.89 (0.84, 0.95)	0.57	0.57
Burkina Faso	PROMIS CS (39)	373	363	71.1	0.92 (0.82, 1.04)	0.13	0.17	201	217	68.2	0.96 (0.81, 1.12)	0.07	0.07
Ghana	GHANA (40)												
Ghana	iLiNS−DYAD−G (41)												
Kenya	WASH−B (42)	128	121	52.9	0.72 (0.55, 0.94)	0.03	0.05	68	41	36.6	0.88 (0.51, 1.52)	0.01	0.01
Madagascar	MAHAY (43)	137	164	66.5	0.90 (0.71, 1.14)	0.03	0.07	433	408	64.2	0.87 (0.75, 1.01)	0.08	0.08
Malawi	iLiNS−DYAD−M (44)	191	397	51.1	0.91 (0.76, 1.09)	0.06	0.10	19	33	60.6	0.78 (0.45, 1.35)	0.01	0.01
Malawi	iLiNS−DOSE (45)												
Mali	PROMIS CS (46)	548	567	86.8	0.81 (0.74, 0.89)	0.20	0.21	364	388	85.6	0.82 (0.75, 0.90)	0.21	0.21
Zimbabwe	SHINE (HIV−) (47)	469	436	33.9	0.69 (0.56, 0.86)	0.04	0.07	259	281	33.8	0.82 (0.66, 1.03)	0.04	0.04
Zimbabwe	SHINE (HIV+) (48)	77	77	36.4	0.68 (0.42, 1.10)	0.01	0.02	49	46	28.3	1.01 (0.48, 2.12)	0.00	0.00
		**2478**	**2266**		**I^2 = 0.31, Tau^2 = 0.00**			**2787**	**1933**		**I^2 = 0.00, Tau^2 = 0.00**		
**Fixed**					**0.82 (0.79, 0.86)**						**0.88 (0.84, 0.92)**		
**Random**					**0.83 (0.77, 0.88)**						**0.88 (0.84, 0.92)**		

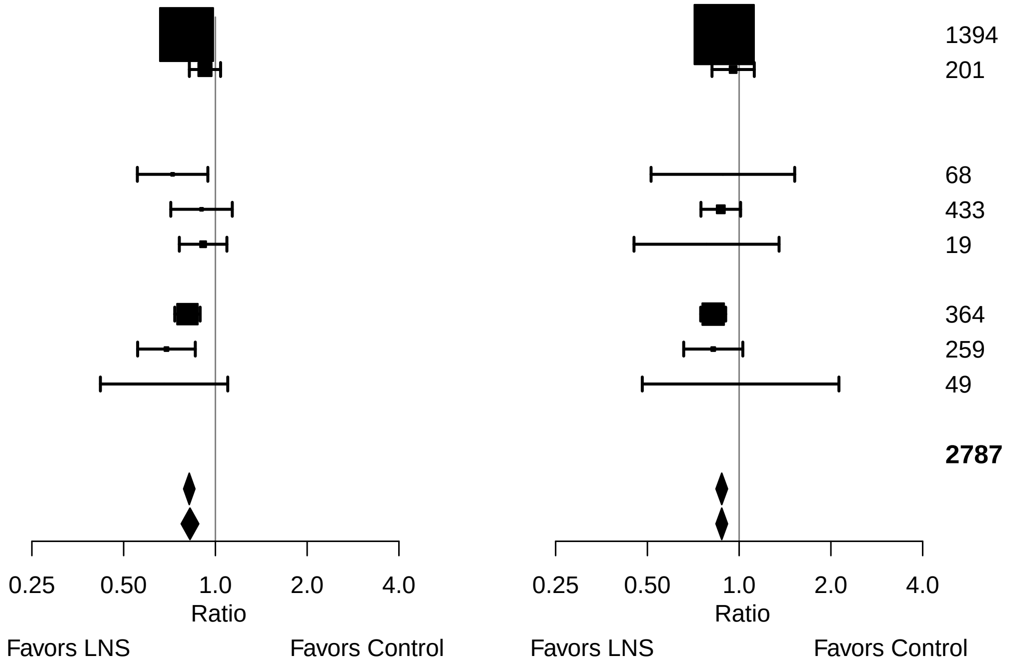

Supplemental figure 9B: Anemia prevalence ratio

9B4: Stratified by Household sanitation

P−for−interaction = 0.092
Ratio of PRs = 0.92 (0.83, 1.01)

Country	Trial	Improved LNS N	Improved Control N	Improved Control Prevalence	Improved PR (95% CI)	Improved Fixed W	Improved Random W	Unimproved LNS N	Unimproved Control N	Unimproved Control Prevalence	Unimproved PR (95% CI)	Unimproved Fixed W	Unimproved Random W
Bangladesh	JiVitA−4 (35)												
Bangladesh	RDNS (36)	376	193	38.9	0.72 (0.52, 0.98)	0.04	0.04	173	79	49.4	0.54 (0.40, 0.72)	0.07	0.14
Bangladesh	WASH−B (37)												
Burkina Faso	iLiNS−Zinc (38)												
Burkina Faso	PROMIS CS (39)	244	229	69.9	0.92 (0.81, 1.05)	0.24	0.24	329	350	70.0	0.95 (0.84, 1.07)	0.37	0.23
Ghana	GHANA (40)												
Ghana	iLiNS−DYAD−G (41)												
Kenya	WASH−B (42)	24	25	44.0	0.47 (0.20, 1.10)	0.01	0.01	172	137	49.6	0.77 (0.61, 0.98)	0.10	0.16
Madagascar	MAHAY (43)												
Malawi	iLiNS−DYAD−M (44)												
Malawi	iLiNS−DOSE (45)												
Mali	PROMIS CS (46)	703	696	86.1	0.84 (0.77, 0.91)	0.62	0.62	219	249	86.7	0.73 (0.64, 0.83)	0.34	0.22
Zimbabwe	SHINE (HIV−) (47)	262	218	31.7	0.88 (0.70, 1.10)	0.09	0.09	465	498	34.7	0.67 (0.54, 0.84)	0.11	0.17
Zimbabwe	SHINE (HIV+) (48)	46	30	30.0	0.72 (0.34, 1.52)	0.01	0.01	80	92	33.7	0.85 (0.53, 1.36)	0.02	0.08
		**1655**	**1391**		**I^2 = 0.00, Tau^2 = 0.00**			**1438**	**1405**		**I^2 = 0.74, Tau^2 = 0.02**		
**Fixed**					**0.85 (0.80, 0.91)**						**0.79 (0.73, 0.85)**		
**Random**					**0.85 (0.80, 0.91)**						**0.75 (0.64, 0.88)**		

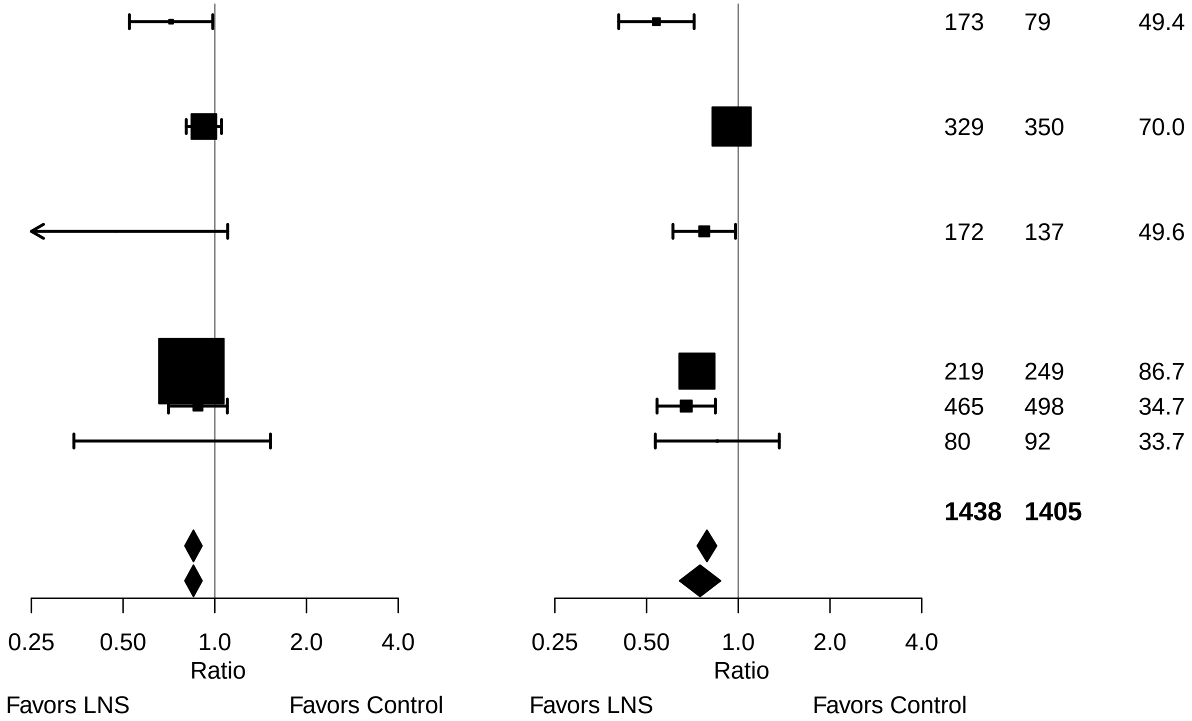

Supplemental figure 9B: Anemia prevalence ratio

9B5: Stratified by Season at the time of assessment

P−for−interaction = 0.434
Ratio of PRs = 1.03 (0.96, 1.10)

		Dry						Rainy					
Country	Trial	LNS N	Control N	Control Prevalence	PR (95% CI)	Fixed W	Random W	LNS N	Control N	Control Prevalence	PR (95% CI)	Fixed W	Random W
Bangladesh	JiVitA−4 (35)	264	72	13.9	1.09 (0.58, 2.05)	0.00	0.02	193	74	17.6	0.59 (0.31, 1.12)	0.01	0.04
Bangladesh	RDNS (36)	341	166	45.8	0.62 (0.47, 0.83)	0.02	0.07	208	106	35.8	0.72 (0.48, 1.10)	0.02	0.07
Bangladesh	WASH−B (37)	108	96	17.7	0.47 (0.23, 0.97)	0.00	0.02	126	90	14.4	0.38 (0.17, 0.87)	0.00	0.03
Burkina Faso	iLiNS−Zinc (38)	1398	475	92.6	0.86 (0.82, 0.90)	0.58	0.14	559	189	87.3	0.90 (0.83, 0.98)	0.43	0.14
Burkina Faso	PROMIS CS (39)	283	245	72.7	0.89 (0.77, 1.04)	0.07	0.12	291	336	68.2	0.97 (0.86, 1.09)	0.24	0.13
Ghana	GHANA (40)	57	55	60.0	0.50 (0.32, 0.78)	0.01	0.04	41	41	56.1	0.52 (0.30, 0.90)	0.01	0.05
Ghana	iLiNS−DYAD−G (41)	198	386	45.6	0.80 (0.64, 0.99)	0.03	0.09	130	274	43.8	0.97 (0.76, 1.23)	0.06	0.10
Kenya	WASH−B (42)	100	88	35.2	0.74 (0.53, 1.02)	0.01	0.06	250	212	52.4	0.66 (0.55, 0.81)	0.08	0.11
Madagascar	MAHAY (43)	517	537	66.7	0.94 (0.83, 1.07)	0.10	0.12	83	51	45.1	0.51 (0.32, 0.81)	0.02	0.06
Malawi	iLiNS−DYAD−M (44)	147	305	46.6	0.91 (0.72, 1.13)	0.03	0.09	63	125	64.8	0.88 (0.69, 1.13)	0.05	0.10
Malawi	iLiNS−DOSE (45)	123	32	68.8	1.01 (0.78, 1.30)	0.02	0.08	120	50	74.0	0.78 (0.62, 0.97)	0.07	0.11
Mali	PROMIS CS (46)												
Zimbabwe	SHINE (HIV−) (47)	1558	1477	36.0	0.76 (0.68, 0.84)	0.13	0.13	124	117	25.6	1.10 (0.74, 1.64)	0.02	0.07
Zimbabwe	SHINE (HIV+) (48)												
		**5094**	**3934**		**I² = 0.54, Tau² = 0.02**			**2188**	**1665**		**I² = 0.62, Tau² = 0.04**		
**Fixed**					**0.85 (0.82, 0.88)**						**0.87 (0.82, 0.92)**		
**Random**					**0.82 (0.73, 0.91)**						**0.79 (0.68, 0.91)**		

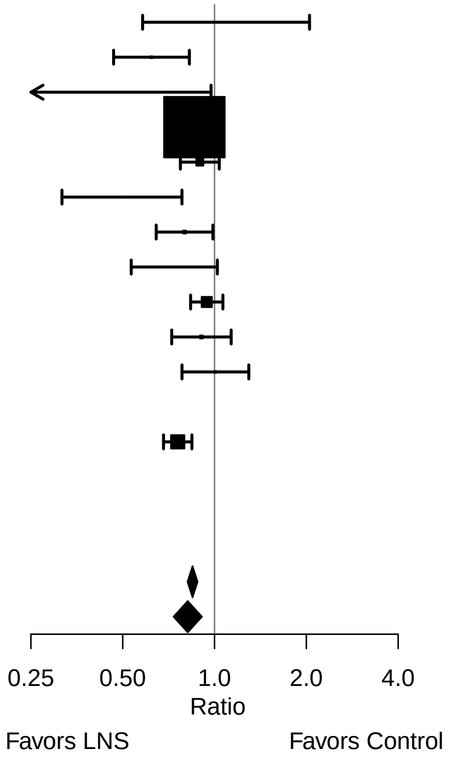

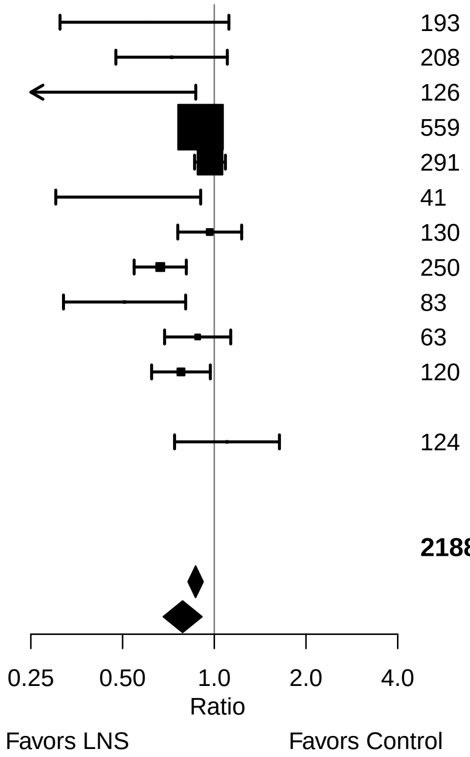

Supplemental figure 9C: Anemia prevalence difference

9C1: Stratified by Household socio-economic status

P−for−interaction = 0.499

Difference in PDs = 0.01 (−0.02, 0.04)

		**At least median**						**Less than median**					
Country	Trial	**LNS** N	**Control** N	**Control** Prevalence	**PD** (95% CI)	**Fixed** W	**Random** W	**LNS** N	**Control** N	**Control** Prevalence	**PD** (95% CI)	**Fixed** W	**Random** W
Bangladesh	JiVitA−4 (35)	244	79	17.7	−0.07 (−0.14, 0.01)	0.08	0.08	212	67	13.4	0.02 (−0.06, 0.10)	0.07	0.09
Bangladesh	RDNS (36)	272	136	41.9	−0.18 (−0.30, −0.06)	0.03	0.04	277	136	41.9	−0.11 (−0.21, −0.01)	0.05	0.08
Bangladesh	WASH−B (37)												
Burkina Faso	iLiNS−Zinc (38)	1232	299	90.0	−0.10 (−0.15, −0.05)	0.18	0.17	718	364	92.0	−0.15 (−0.20, −0.10)	0.19	0.10
Burkina Faso	PROMIS CS (39)	289	293	70.0	−0.05 (−0.15, 0.05)	0.05	0.05	285	288	70.1	−0.04 (−0.11, 0.02)	0.12	0.10
Ghana	GHANA (40)	57	44	45.5	−0.19 (−0.38, −0.01)	0.01	0.02	41	46	71.7	−0.38 (−0.57, −0.18)	0.01	0.04
Ghana	iLiNS−DYAD−G (41)	149	344	41.6	−0.05 (−0.14, 0.05)	0.05	0.06	179	315	48.6	−0.08 (−0.17, 0.01)	0.06	0.08
Kenya	WASH−B (42)	190	175	48.0	−0.17 (−0.26, −0.08)	0.05	0.06	159	125	46.4	−0.12 (−0.25, 0.01)	0.03	0.06
Madagascar	MAHAY (43)	278	311	64.0	−0.14 (−0.24, −0.03)	0.04	0.05	304	262	65.6	−0.03 (−0.13, 0.08)	0.04	0.07
Malawi	iLiNS−DYAD−M (44)	117	225	48.4	−0.08 (−0.19, 0.03)	0.04	0.04	93	206	55.8	−0.01 (−0.13, 0.11)	0.03	0.07
Malawi	iLiNS−DOSE (45)	86	32	78.1	−0.22 (−0.42, −0.03)	0.01	0.01	114	39	61.5	0.05 (−0.12, 0.22)	0.02	0.05
Mali	PROMIS CS (46)	505	463	85.7	−0.16 (−0.22, −0.10)	0.13	0.13	448	507	86.6	−0.16 (−0.23, −0.09)	0.11	0.09
Zimbabwe	SHINE (HIV−) (47)	821	722	35.3	−0.09 (−0.14, −0.05)	0.27	0.24	742	760	34.9	−0.08 (−0.12, −0.03)	0.23	0.10
Zimbabwe	SHINE (HIV+) (48)	155	138	34.1	−0.13 (−0.24, −0.03)	0.04	0.05	145	140	40.0	−0.14 (−0.25, −0.03)	0.04	0.07
		**4395**	**3261**		**I² = 0.08, Tau² = 0.00**			**3717**	**3255**		**I² = 0.64, Tau² = 0.01**		
**Fixed**					**−0.11 (−0.13, −0.09)**						**−0.09 (−0.11, −0.07)**		
**Random**					**−0.11 (−0.13, −0.09)**						**−0.09 (−0.14, −0.04)**		

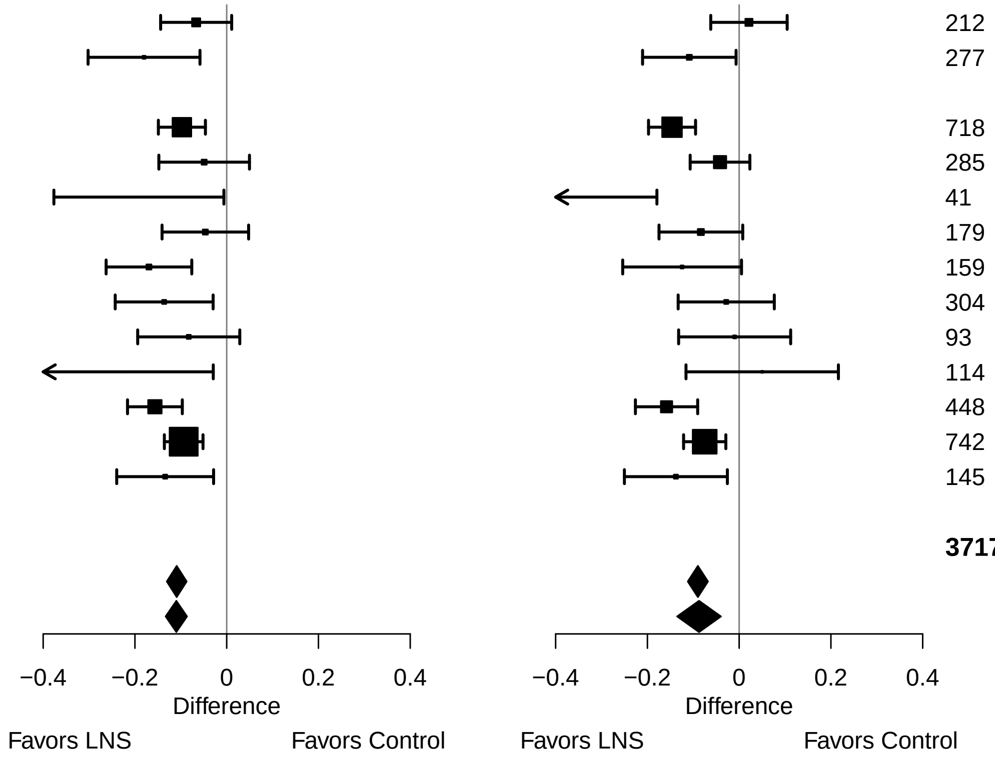

Supplemental figure 9C: Anemia prevalence difference

9C2: Stratified by Household food insecurity

P−for−interaction = 0.857
Difference in PDs = 0.00 (−0.04, 0.03)

		Mild to secure						Moderate to severe					
Country	Trial	LNS N	Control N	Control Prevalence	PD (95% CI)	Fixed W	Random W	LNS N	Control N	Control Prevalence	PD (95% CI)	Fixed W	Random W
Bangladesh	JiVitA−4 (35)												
Bangladesh	RDNS (36)	342	166	40.4	−0.14 (−0.27, −0.01)	0.03	0.05	207	106	44.3	−0.15 (−0.24, −0.05)	0.11	0.12
Bangladesh	WASH−B (37)												
Burkina Faso	iLiNS−Zinc (38)	1016	320	91.9	−0.12 (−0.17, −0.07)	0.23	0.22	934	343	90.4	−0.12 (−0.17, −0.07)	0.38	0.26
Burkina Faso	PROMIS CS (39)												
Ghana	GHANA (40)												
Ghana	iLiNS−DYAD−G (41)	230	457	47.0	−0.06 (−0.14, 0.02)	0.08	0.11	97	201	40.8	−0.08 (−0.20, 0.04)	0.07	0.09
Kenya	WASH−B (42)	304	273	48.7	−0.17 (−0.25, −0.10)	0.09	0.11	45	27	33.3	0.04 (−0.15, 0.24)	0.03	0.04
Madagascar	MAHAY (43)	359	340	69.4	−0.12 (−0.21, −0.03)	0.06	0.09	137	140	63.6	0.01 (−0.12, 0.14)	0.06	0.08
Malawi	iLiNS−DYAD−M (44)	66	113	58.4	−0.13 (−0.28, 0.02)	0.02	0.03	143	314	50.0	−0.03 (−0.13, 0.07)	0.10	0.12
Malawi	iLiNS−DOSE (45)	50	21	61.9	0.07 (−0.17, 0.32)	0.01	0.01	144	46	69.6	−0.09 (−0.25, 0.06)	0.04	0.06
Mali	PROMIS CS (46)												
Zimbabwe	SHINE (HIV−) (47)	1253	1162	34.5	−0.07 (−0.11, −0.03)	0.40	0.29	264	293	36.9	−0.15 (−0.22, −0.07)	0.18	0.18
Zimbabwe	SHINE (HIV+) (48)	217	204	35.3	−0.13 (−0.21, −0.04)	0.07	0.09	75	66	42.4	−0.17 (−0.33, −0.01)	0.04	0.05
		**3837**	**3056**		**I² = 0.27, Tau² = 0.00**			**2046**	**1536**		**I² = 0.22, Tau² = 0.00**		
**Fixed**					**−0.10 (−0.12, −0.08)**						**−0.10 (−0.14, −0.07)**		
**Random**					**−0.10 (−0.13, −0.07)**						**−0.10 (−0.14, −0.06)**		

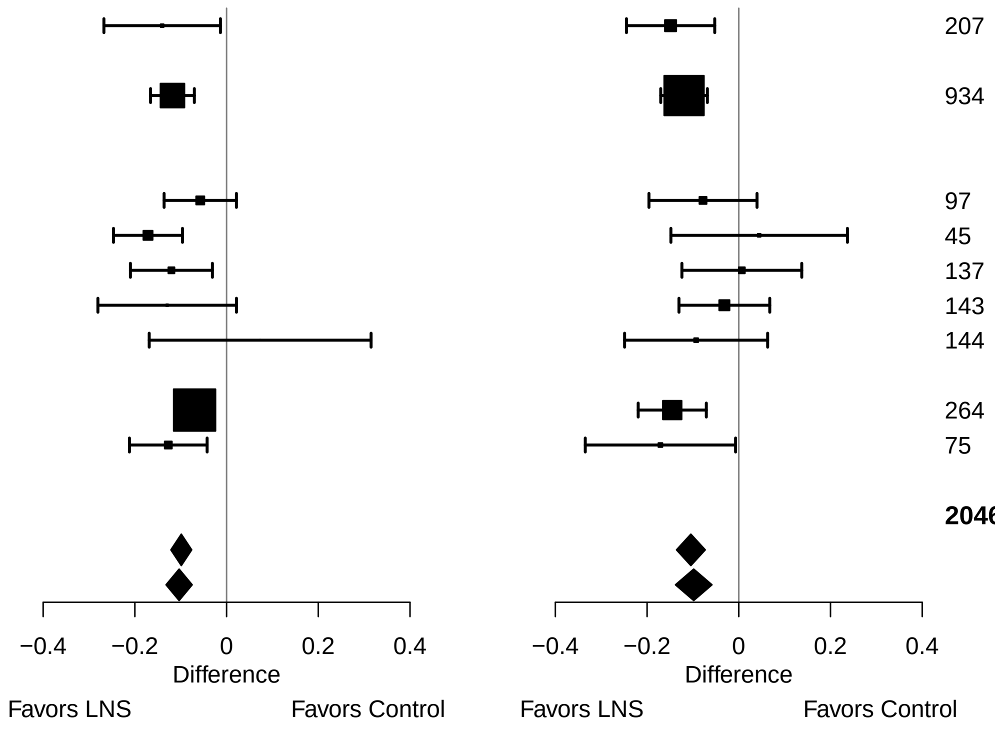

Supplemental figure 9C: Anemia prevalence difference

9C3: Stratified by Household source water quality

P−for−interaction = 0.005
Difference in PDs = 0.05 (0.02, 0.09)

Country	Trial	LNS N	Control N	Control Prevalence	Improved PD (95% CI)	Fixed W	Random W	LNS N	Control N	Control Prevalence	Unimproved PD (95% CI)	Fixed W	Random W
Bangladesh	JiVitA−4 (35)												
Bangladesh	RDNS (36)												
Bangladesh	WASH−B (37)												
Burkina Faso	iLiNS−Zinc (38)	555	141	96.5	−0.19 (−0.24, −0.14)	0.31	0.21	1394	519	89.6	−0.10 (−0.14, −0.05)	0.37	0.37
Burkina Faso	PROMIS CS (39)	373	363	71.1	−0.05 (−0.13, 0.03)	0.12	0.14	201	217	68.2	−0.03 (−0.14, 0.08)	0.08	0.08
Ghana	GHANA (40)												
Ghana	iLiNS−DYAD−G (41)												
Kenya	WASH−B (42)	128	121	52.9	−0.15 (−0.26, −0.03)	0.06	0.08	68	41	36.6	−0.04 (−0.23, 0.15)	0.03	0.03
Madagascar	MAHAY (43)	137	164	66.5	−0.07 (−0.22, 0.08)	0.03	0.06	433	408	64.2	−0.08 (−0.17, 0.01)	0.11	0.11
Malawi	iLiNS−DYAD−M (44)	191	397	51.1	−0.05 (−0.13, 0.04)	0.10	0.13	19	33	60.6	−0.13 (−0.42, 0.15)	0.01	0.01
Malawi	iLiNS−DOSE (45)												
Mali	PROMIS CS (46)	548	567	86.8	−0.17 (−0.24, −0.09)	0.15	0.16	364	388	85.6	−0.15 (−0.22, −0.08)	0.18	0.18
Zimbabwe	SHINE (HIV−) (47)	469	436	33.9	−0.10 (−0.17, −0.04)	0.19	0.17	259	281	33.8	−0.06 (−0.13, 0.01)	0.20	0.20
Zimbabwe	SHINE (HIV+) (48)	77	77	36.4	−0.12 (−0.27, 0.03)	0.03	0.06	49	46	28.3	0.00 (−0.21, 0.21)	0.02	0.02
		**2478**	**2266**		**I² = 0.53, Tau² = 0.00**			**2787**	**1933**		**I² = 0.00, Tau² = 0.00**		
**Fixed**					**−0.13 (−0.16, −0.10)**						**−0.09 (−0.12, −0.06)**		
**Random**					**−0.12 (−0.16, −0.08)**						**−0.09 (−0.12, −0.06)**		

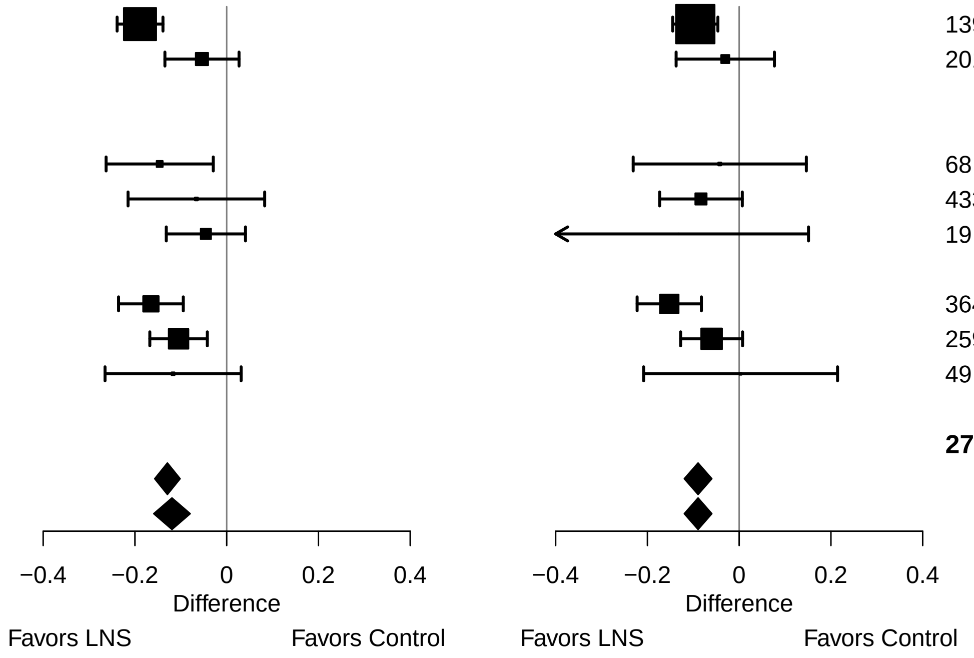

Supplemental figure 9C: Anemia prevalence difference

9C4: Stratified by Household sanitation

P−for−interaction = 0.036
Difference in PDs = −0.05 (−0.11, 0.00)

Country	Trial	Improved LNS N	Improved Control N	Improved Control Prevalence	Improved PD (95% CI)	Improved Fixed W	Improved Random W	Unimproved LNS N	Unimproved Control N	Unimproved Control Prevalence	Unimproved PD (95% CI)	Unimproved Fixed W	Unimproved Random W
Bangladesh	JiVitA−4 (35)												
Bangladesh	RDNS (36)	376	193	38.9	−0.11 (−0.22, 0.00)	0.11	0.13	173	79	49.4	−0.23 (−0.33, −0.12)	0.11	0.15
Bangladesh	WASH−B (37)												
Burkina Faso	iLiNS−Zinc (38)												
Burkina Faso	PROMIS CS (39)	244	229	69.9	−0.06 (−0.14, 0.03)	0.17	0.19	329	350	70.0	−0.03 (−0.12, 0.05)	0.19	0.18
Ghana	GHANA (40)												
Ghana	iLiNS−DYAD−G (41)												
Kenya	WASH−B (42)	24	25	44.0	−0.23 (−0.47, 0.01)	0.02	0.03	172	137	49.6	−0.11 (−0.22, −0.01)	0.12	0.16
Madagascar	MAHAY (43)												
Malawi	iLiNS−DYAD−M (44)												
Malawi	iLiNS−DOSE (45)												
Mali	PROMIS CS (46)	703	696	86.1	−0.14 (−0.20, −0.08)	0.35	0.31	219	249	86.7	−0.23 (−0.32, −0.15)	0.18	0.18
Zimbabwe	SHINE (HIV−) (47)	262	218	31.7	−0.04 (−0.11, 0.03)	0.30	0.28	465	498	34.7	−0.11 (−0.17, −0.05)	0.34	0.21
Zimbabwe	SHINE (HIV+) (48)	46	30	30.0	−0.08 (−0.27, 0.10)	0.04	0.05	80	92	33.7	−0.05 (−0.20, 0.10)	0.06	0.12
		**1655**	**1391**		**I² = 0.26, Tau² = 0.00**			**1438**	**1405**		**I² = 0.68, Tau² = 0.00**		
**Fixed**					**−0.09 (−0.13, −0.05)**						**−0.13 (−0.17, −0.09)**		
**Random**					**−0.09 (−0.13, −0.05)**						**−0.13 (−0.20, −0.06)**		

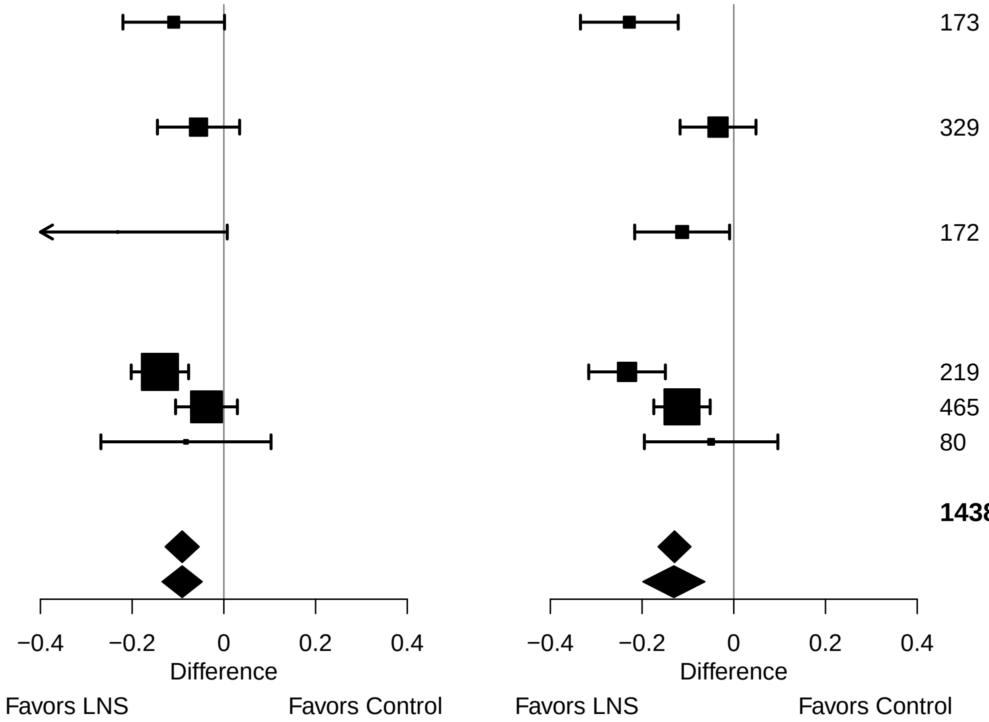

Supplemental figure 9C: Anemia prevalence difference

9C5: Stratified by Season at the time of assessment

P−for−interaction = 0.332
Difference in PDs = 0.02 (−0.02, 0.05)

Country	Trial	Dry: LNS N	Dry: Control N	Dry: Control Prevalence	Dry: PD (95% CI)	Dry: Fixed W	Dry: Random W	Rainy: LNS N	Rainy: Control N	Rainy: Control Prevalence	Rainy: PD (95% CI)	Rainy: Fixed W	Rainy: Random W
Bangladesh	JiVitA−4 (35)	264	72	13.9	0.01 (−0.08, 0.10)	0.05	0.09	193	74	17.6	−0.07 (−0.17, 0.02)	0.09	0.10
Bangladesh	RDNS (36)	341	166	45.8	−0.17 (−0.28, −0.07)	0.04	0.07	208	106	35.8	−0.10 (−0.24, 0.04)	0.04	0.06
Bangladesh	WASH−B (37)	108	96	17.7	−0.09 (−0.20, 0.01)	0.04	0.07	126	90	14.4	−0.09 (−0.17, −0.01)	0.14	0.12
Burkina Faso	iLiNS−Zinc (38)	1398	475	92.6	−0.13 (−0.17, −0.09)	0.24	0.14	559	189	87.3	−0.09 (−0.16, −0.01)	0.16	0.12
Burkina Faso	PROMIS CS (39)	283	245	72.7	−0.08 (−0.17, 0.02)	0.04	0.08	291	336	68.2	−0.02 (−0.10, 0.06)	0.14	0.11
Ghana	GHANA (40)	57	55	60.0	−0.30 (−0.48, −0.12)	0.01	0.03	41	41	56.1	−0.27 (−0.48, −0.06)	0.02	0.04
Ghana	iLiNS−DYAD−G (41)	198	386	45.6	−0.09 (−0.18, −0.01)	0.06	0.09	130	274	43.8	−0.01 (−0.12, 0.09)	0.08	0.09
Kenya	WASH−B (42)	100	88	35.2	−0.09 (−0.20, 0.02)	0.04	0.07	250	212	52.4	−0.18 (−0.26, −0.09)	0.13	0.11
Madagascar	MAHAY (43)	517	537	66.7	−0.04 (−0.12, 0.04)	0.07	0.10	83	51	45.1	−0.22 (−0.38, −0.07)	0.04	0.06
Malawi	iLiNS−DYAD−M (44)	147	305	46.6	−0.04 (−0.14, 0.05)	0.04	0.08	63	125	64.8	−0.08 (−0.22, 0.07)	0.04	0.06
Malawi	iLiNS−DOSE (45)	123	32	68.8	0.00 (−0.17, 0.17)	0.01	0.04	120	50	74.0	−0.17 (−0.32, −0.01)	0.03	0.05
Mali	PROMIS CS (46)												
Zimbabwe	SHINE (HIV−) (47)	1558	1477	36.0	−0.09 (−0.12, −0.05)	0.36	0.15	124	117	25.6	0.03 (−0.08, 0.13)	0.08	0.09
Zimbabwe	SHINE (HIV+) (48)												
		**5094**	**3934**		**I² = 0.45, Tau² = 0.00**			**2188**	**1665**		**I² = 0.45, Tau² = 0.00**		
**Fixed**					**−0.09 (−0.11, −0.07)**						**−0.08 (−0.11, −0.05)**		
**Random**					**−0.09 (−0.12, −0.05)**						**−0.09 (−0.13, −0.05)**		

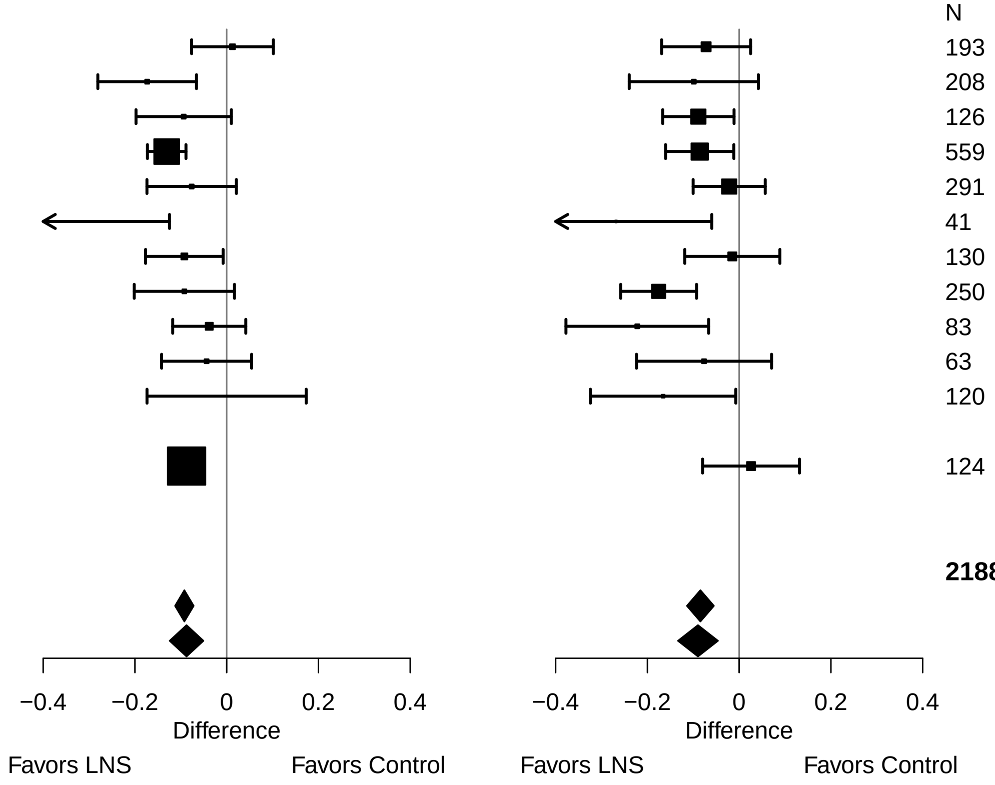

Supplemental figure 9D: Moderate-to-severe anemia prevalence ratio

9D1: Stratified by Household socio-economic status

P−for−interaction = 0.244
Ratio of PRs = 0.95 (0.88, 1.03)

		At least median							Less than median						
Country	Trial	LNS N	Control N	Control Prevalence	PR (95% CI)	Fixed W	Random W		LNS N	Control N	Control Prevalence	PR (95% CI)	Fixed W	Random W
Bangladesh	JiVitA−4 (35)													
Bangladesh	RDNS (36)	272	136	16.2	0.43 (0.24, 0.77)	0.02	0.06		277	136	16.9	0.64 (0.40, 1.03)	0.03	0.05
Bangladesh	WASH−B (37)													
Burkina Faso	iLiNS−Zinc (38)	1232	299	73.6	0.73 (0.66, 0.82)	0.51	0.16		718	364	76.1	0.69 (0.62, 0.77)	0.51	0.25
Burkina Faso	PROMIS CS (39)	289	293	34.1	1.02 (0.76, 1.38)	0.06	0.11		285	288	39.2	0.76 (0.57, 1.02)	0.07	0.11
Ghana	GHANA (40)													
Ghana	iLiNS−DYAD−G (41)	149	344	3.2	1.26 (0.47, 3.34)	0.01	0.03		179	315	7.9	0.63 (0.30, 1.33)	0.01	0.02
Kenya	WASH−B (42)	190	175	20.0	0.63 (0.41, 0.97)	0.03	0.08		159	125	23.2	0.62 (0.37, 1.05)	0.02	0.04
Madagascar	MAHAY (43)	278	311	30.2	0.73 (0.53, 0.99)	0.06	0.11		304	262	37.0	0.82 (0.61, 1.10)	0.07	0.11
Malawi	iLiNS−DYAD−M (44)	117	225	20.9	1.06 (0.70, 1.63)	0.03	0.08		93	206	26.7	1.25 (0.87, 1.80)	0.04	0.08
Malawi	iLiNS−DOSE (45)	86	32	53.1	0.55 (0.35, 0.87)	0.03	0.08		114	39	41.0	0.83 (0.55, 1.25)	0.03	0.07
Mali	PROMIS CS (46)	505	463	56.8	0.67 (0.56, 0.80)	0.18	0.14		448	507	63.3	0.61 (0.51, 0.73)	0.17	0.18
Zimbabwe	SHINE (HIV−) (47)	821	722	12.2	0.70 (0.53, 0.92)	0.07	0.12		742	760	9.5	0.63 (0.43, 0.91)	0.04	0.08
Zimbabwe	SHINE (HIV+) (48)	155	138	13.0	0.35 (0.15, 0.80)	0.01	0.03		145	140	7.1	0.87 (0.39, 1.94)	0.01	0.02
		**4094**	**3138**		**I² = 0.46, Tau² = 0.04**				**3464**	**3142**		**I² = 0.33, Tau² = 0.01**		
**Fixed**					**0.72 (0.67, 0.78)**							**0.70 (0.65, 0.76)**		
**Random**					**0.71 (0.60, 0.85)**							**0.73 (0.65, 0.82)**		

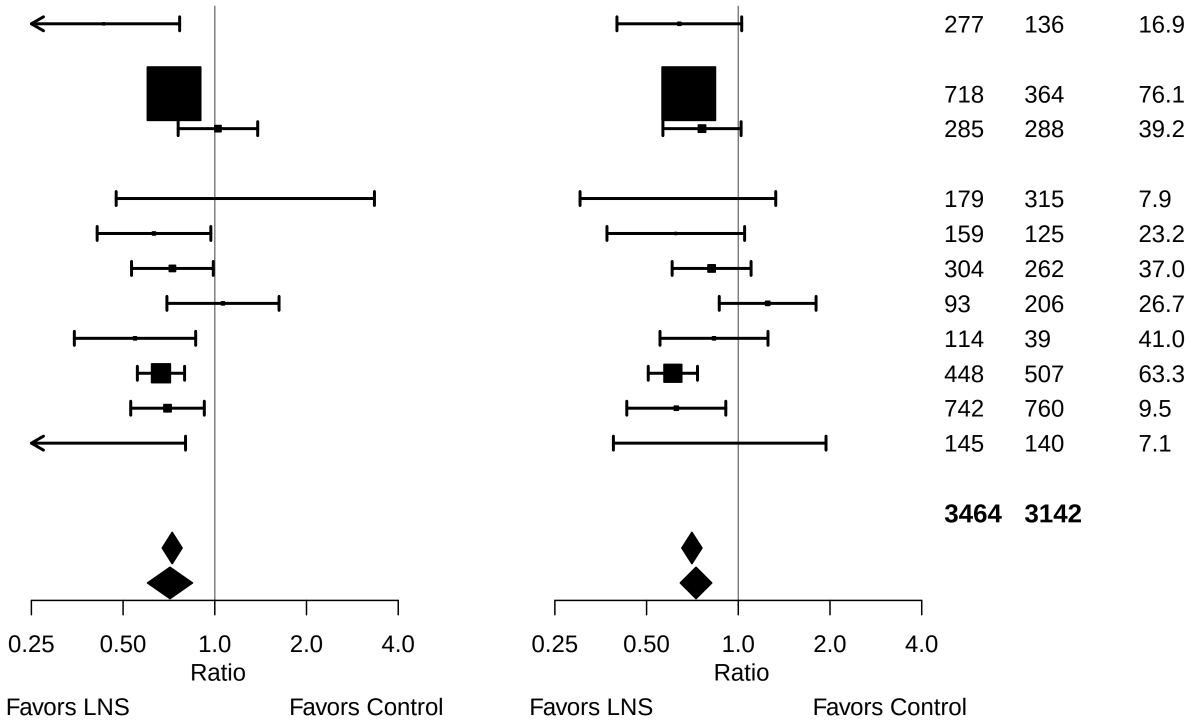

Supplemental figure 9D: Moderate-to-severe anemia prevalence ratio

9D2: Stratified by Household food insecurity

P−for−interaction = 0.938
Ratio of PRs = 1.00 (0.89, 1.11)

Country	Trial	**Mild to secure** LNS N	Control N	Control Prevalence	PR (95% CI)	Fixed W	Random W	**Moderate to severe** LNS N	Control N	Control Prevalence	PR (95% CI)	Fixed W	Random W
Bangladesh	JiVitA−4 (35)												
Bangladesh	RDNS (36)	342	166	13.3	0.71 (0.44, 1.14)	0.02	0.02	207	106	21.7	0.38 (0.24, 0.61)	0.05	0.13
Bangladesh	WASH−B (37)												
Burkina Faso	iLiNS−Zinc (38)	1016	320	77.2	0.72 (0.66, 0.79)	0.75	0.75	934	343	72.9	0.70 (0.62, 0.80)	0.64	0.19
Burkina Faso	PROMIS CS (39)												
Ghana	GHANA (40)												
Ghana	iLiNS−DYAD−G (41)												
Kenya	WASH−B (42)	304	273	21.2	0.63 (0.44, 0.92)	0.04	0.04	45	27	22.2	0.60 (0.26, 1.36)	0.02	0.08
Madagascar	MAHAY (43)	359	340	38.2	0.71 (0.54, 0.94)	0.07	0.07	137	140	32.1	1.04 (0.74, 1.48)	0.09	0.16
Malawi	iLiNS−DYAD−M (44)	66	113	28.3	0.80 (0.47, 1.37)	0.02	0.02	143	314	22.0	1.34 (0.96, 1.86)	0.10	0.16
Malawi	iLiNS−DOSE (45)	50	21	47.6	0.62 (0.34, 1.13)	0.02	0.02	144	46	43.5	0.80 (0.55, 1.16)	0.07	0.15
Mali	PROMIS CS (46)												
Zimbabwe	SHINE (HIV−) (47)	1253	1162	10.2	0.76 (0.58, 1.00)	0.08	0.08	264	293	12.6	0.51 (0.30, 0.87)	0.04	0.12
Zimbabwe	SHINE (HIV+) (48)												
		**3390**	**2395**		**I² = 0.00, Tau² = 0.00**			**1874**	**1269**		**I² = 0.77, Tau² = 0.14**		
**Fixed**					**0.72 (0.67, 0.78)**						**0.75 (0.68, 0.83)**		
**Random**					**0.72 (0.67, 0.78)**						**0.74 (0.54, 1.01)**		

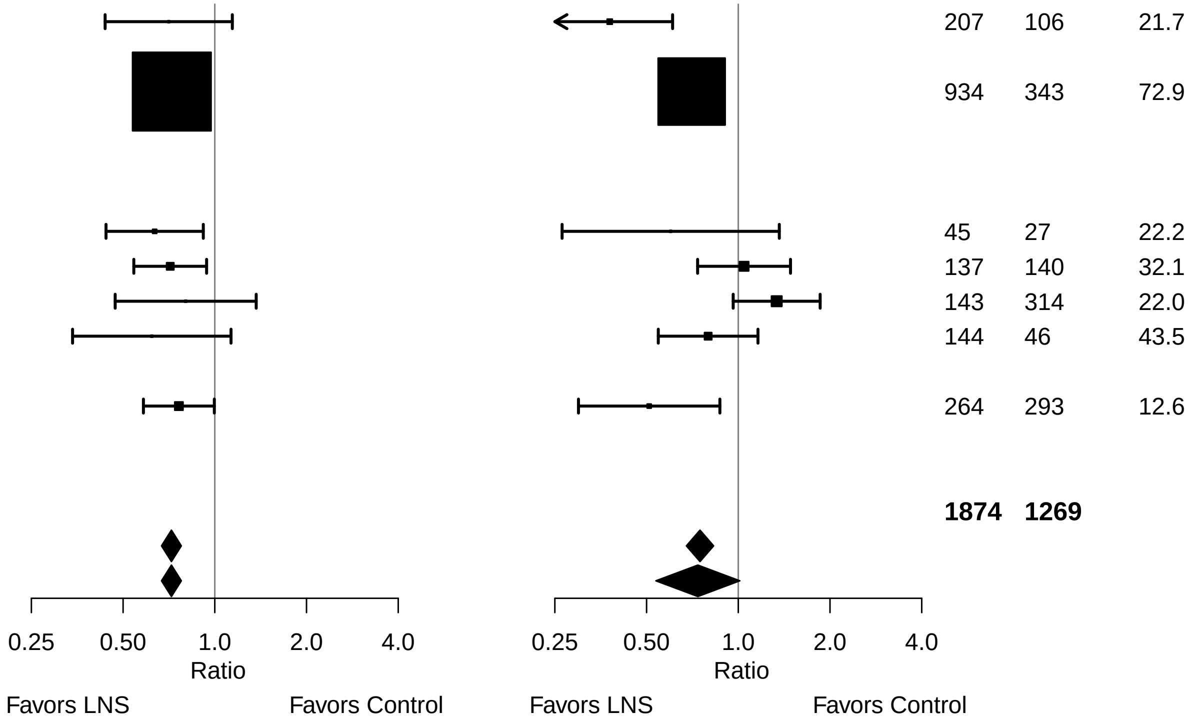

Supplemental figure 9D: Moderate-to-severe anemia prevalence ratio

9D3: Stratified by Household source water quality

P−for−interaction = 0.952
Ratio of PRs = 1.00 (0.89, 1.14)

Country	Trial	Improved LNS N	Improved Control N	Improved Control Prevalence	Improved PR (95% CI)	Improved Fixed W	Improved Random W	Unimproved LNS N	Unimproved Control N	Unimproved Control Prevalence	Unimproved PR (95% CI)	Unimproved Fixed W	Unimproved Random W
Bangladesh	JiVitA−4 (35)												
Bangladesh	RDNS (36)												
Bangladesh	WASH−B (37)												
Burkina Faso	iLiNS−Zinc (38)	555	141	75.9	0.66 (0.54, 0.80)	0.24	0.19	1394	519	74.6	0.74 (0.68, 0.81)	0.72	0.72
Burkina Faso	PROMIS CS (39)	373	363	35.8	0.94 (0.77, 1.16)	0.24	0.19	201	217	37.8	0.79 (0.57, 1.09)	0.05	0.05
Ghana	GHANA (40)												
Ghana	iLiNS−DYAD−G (41)												
Kenya	WASH−B (42)												
Madagascar	MAHAY (43)	137	164	31.1	1.01 (0.72, 1.41)	0.09	0.15	433	408	34.3	0.73 (0.55, 0.97)	0.06	0.06
Malawi	iLiNS−DYAD−M (44)	191	397	23.2	1.17 (0.88, 1.57)	0.12	0.16	19	33	30.3	0.87 (0.35, 2.16)	0.01	0.01
Malawi	iLiNS−DOSE (45)												
Mali	PROMIS CS (46)	548	567	59.8	0.62 (0.51, 0.76)	0.25	0.19	364	388	60.8	0.67 (0.56, 0.81)	0.15	0.15
Zimbabwe	SHINE (HIV−) (47)	469	436	9.4	0.70 (0.48, 1.04)	0.07	0.13	259	281	10.3	0.41 (0.22, 0.76)	0.01	0.01
Zimbabwe	SHINE (HIV+) (48)												
		**2273**	**2068**		**I² = 0.76, Tau² = 0.05**			**2670**	**1846**		**I² = 0.00, Tau² = 0.00**		
**Fixed**					**0.79 (0.71, 0.87)**						**0.73 (0.68, 0.78)**		
**Random**					**0.82 (0.66, 1.01)**						**0.73 (0.68, 0.78)**		

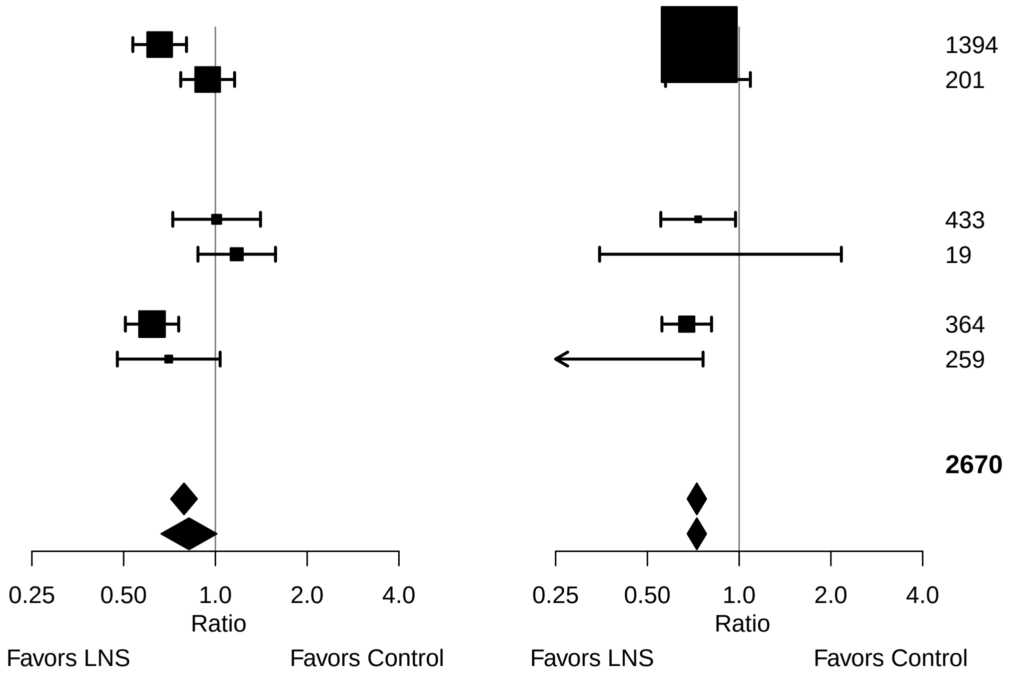

Supplemental figure 9D: Moderate-to-severe anemia prevalence ratio

9D4: Stratified by Household sanitation

P−for−interaction = 0.004
Ratio of PRs = 0.74 (0.60, 0.91)

Country	Trial	Improved LNS N	Improved Control N	Improved Control Prevalence	Improved PR (95% CI)	Improved Fixed W	Improved Random W	Unimproved LNS N	Unimproved Control N	Unimproved Control Prevalence	Unimproved PR (95% CI)	Unimproved Fixed W	Unimproved Random W
Bangladesh	JiVitA−4 (35)												
Bangladesh	RDNS (36)	376	193	13.5	0.71 (0.44, 1.15)	0.08	0.12	173	79	24.1	0.31 (0.17, 0.56)	0.08	0.19
Bangladesh	WASH−B (37)												
Burkina Faso	iLiNS−Zinc (38)												
Burkina Faso	PROMIS CS (39)	244	229	34.1	0.97 (0.73, 1.30)	0.21	0.27	329	350	38.3	0.83 (0.65, 1.07)	0.43	0.29
Ghana	GHANA (40)												
Ghana	iLiNS−DYAD−G (41)												
Kenya	WASH−B (42)												
Madagascar	MAHAY (43)												
Malawi	iLiNS−DYAD−M (44)												
Malawi	iLiNS−DOSE (45)												
Mali	PROMIS CS (46)	703	696	59.6	0.69 (0.58, 0.81)	0.64	0.50	219	249	59.4	0.48 (0.37, 0.64)	0.36	0.29
Zimbabwe	SHINE (HIV−) (47)	262	218	11.0	0.66 (0.40, 1.07)	0.07	0.11	465	498	9.2	0.54 (0.34, 0.83)	0.14	0.23
Zimbabwe	SHINE (HIV+) (48)												
		**1585**	**1336**		**I² = 0.35, Tau² = 0.01**			**1186**	**1176**		**I² = 0.79, Tau² = 0.11**		
**Fixed**					**0.74 (0.65, 0.84)**						**0.60 (0.51, 0.71)**		
**Random**					**0.75 (0.63, 0.90)**						**0.54 (0.36, 0.79)**		

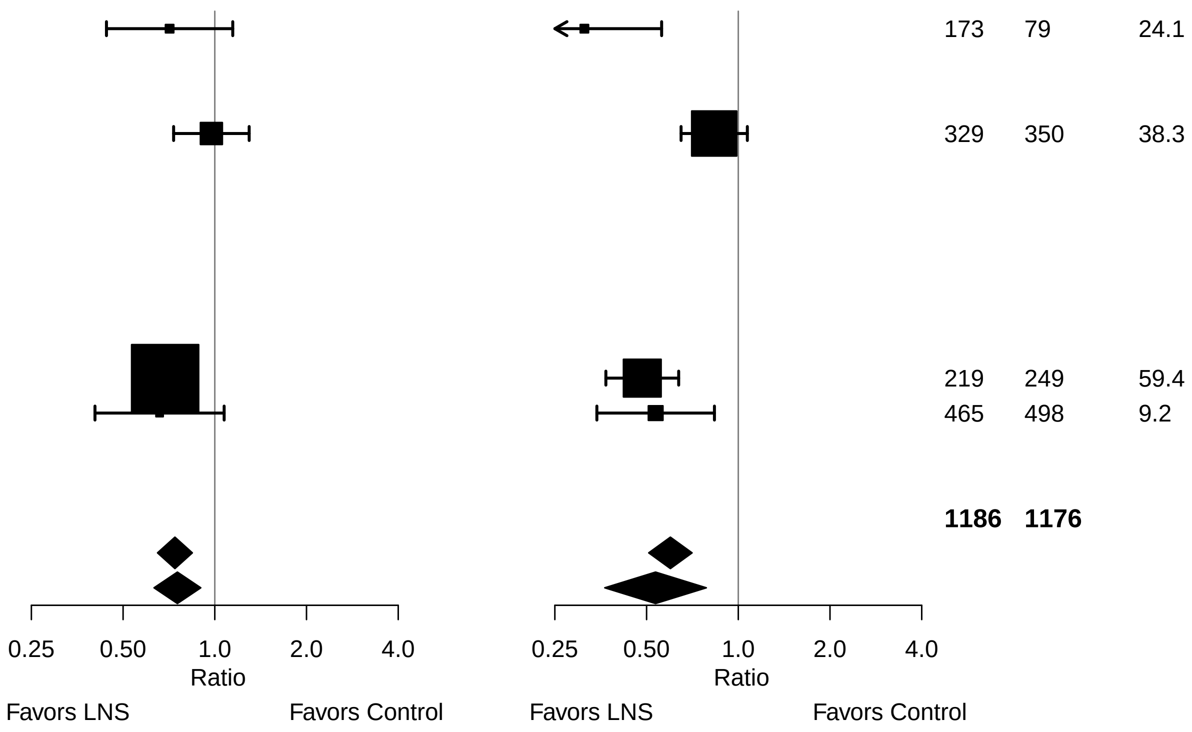

Supplemental figure 9D: Moderate-to-severe anemia prevalence ratio

9D5: Stratified by Season at the time of assessment

P−for−interaction = 0.189
Ratio of PRs = 1.08 (0.96, 1.20)

		Dry						Rainy					
Country	Trial	LNS N	Control N	Control Prevalence	PR (95% CI)	Fixed W	Random W	LNS N	Control N	Control Prevalence	PR (95% CI)	Fixed W	Random W
Bangladesh	JiVitA−4 (35)												
Bangladesh	RDNS (36)	341	166	20.5	0.44 (0.29, 0.68)	0.03	0.12	208	106	10.4	0.83 (0.38, 1.83)	0.02	0.04
Bangladesh	WASH−B (37)												
Burkina Faso	iLiNS−Zinc (38)	1398	475	75.8	0.70 (0.64, 0.77)	0.68	0.16	559	189	73.0	0.75 (0.67, 0.86)	0.59	0.30
Burkina Faso	PROMIS CS (39)	283	245	38.8	0.79 (0.58, 1.08)	0.06	0.14	291	336	35.1	0.97 (0.77, 1.22)	0.18	0.21
Ghana	GHANA (40)	57	55	34.5	0.25 (0.10, 0.63)	0.01	0.06	41	41	29.3	0.42 (0.16, 1.08)	0.01	0.03
Ghana	iLiNS−DYAD−G (41)	198	386	5.4	0.93 (0.45, 1.93)	0.01	0.07	130	274	5.5	0.70 (0.26, 1.89)	0.01	0.03
Kenya	WASH−B (42)	100	88	15.9	0.44 (0.20, 0.98)	0.01	0.07	250	212	23.6	0.68 (0.48, 0.96)	0.08	0.14
Madagascar	MAHAY (43)	517	537	34.6	0.85 (0.68, 1.07)	0.12	0.15	83	51	13.7	0.61 (0.21, 1.79)	0.01	0.02
Malawi	iLiNS−DYAD−M (44)	147	305	22.0	1.11 (0.78, 1.59)	0.05	0.13	63	125	27.2	1.23 (0.78, 1.93)	0.05	0.10
Malawi	iLiNS−DOSE (45)	123	32	40.6	0.96 (0.61, 1.51)	0.03	0.11	120	50	50.0	0.58 (0.40, 0.84)	0.07	0.13
Mali	PROMIS CS (46)												
Zimbabwe	SHINE (HIV−) (47)												
Zimbabwe	SHINE (HIV+) (48)												
		**3164**	**2289**		**I^2 = 0.64, Tau^2 = 0.11**			**1745**	**1384**		**I^2 = 0.36, Tau^2 = 0.02**		
**Fixed**					**0.73 (0.67, 0.79)**						**0.78 (0.71, 0.86)**		
**Random**					**0.71 (0.55, 0.93)**						**0.78 (0.66, 0.92)**		

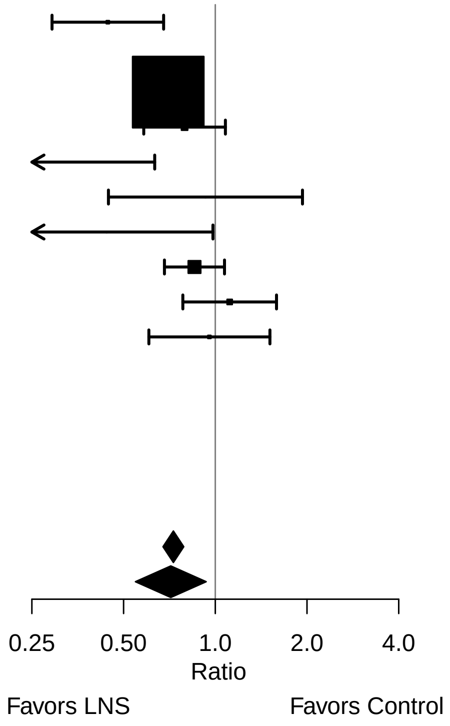

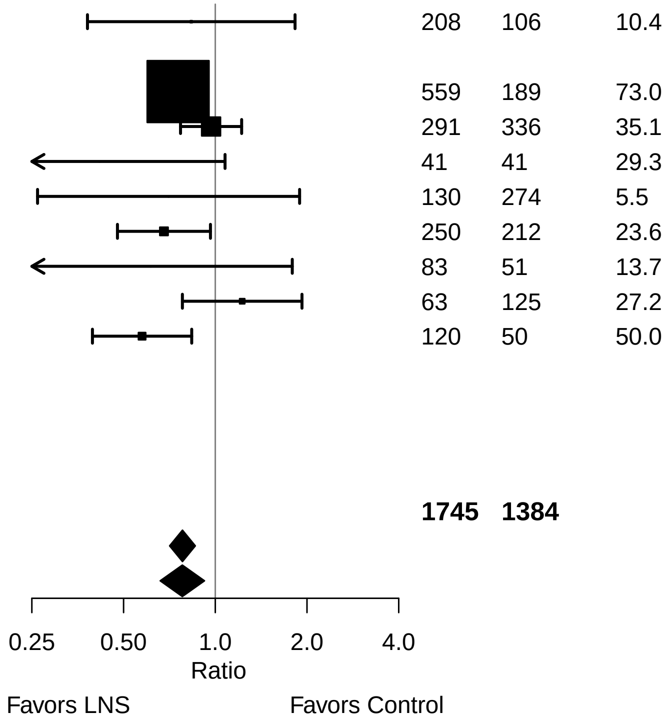

Supplemental figure 9E: Moderate-to-severe anemia prevalence difference

9E1: Stratified by Household socio-economic status

P−for−interaction = 0.490
Difference in PDs = −0.01 (−0.03, 0.02)

		At least median						Less than median					
Country	Trial	LNS N	Control N	Control Prevalence	PD (95% CI)	Fixed W	Random W	LNS N	Control N	Control Prevalence	PD (95% CI)	Fixed W	Random W
Bangladesh	JiVitA−4 (35)												
Bangladesh	RDNS (36)	272	136	16.2	−0.09 (−0.16, −0.02)	0.06	0.10	277	136	16.9	−0.06 (−0.13, 0.01)	0.07	0.10
Bangladesh	WASH−B (37)												
Burkina Faso	iLiNS−Zinc (38)	1232	299	73.6	−0.20 (−0.26, −0.13)	0.06	0.10	718	364	76.1	−0.24 (−0.30, −0.17)	0.07	0.10
Burkina Faso	PROMIS CS (39)	289	293	34.1	0.01 (−0.10, 0.11)	0.03	0.08	285	288	39.2	−0.09 (−0.19, 0.00)	0.03	0.08
Ghana	GHANA (40)												
Ghana	iLiNS−DYAD−G (41)	149	344	3.2	0.01 (−0.03, 0.04)	0.24	0.11	179	315	7.9	−0.03 (−0.08, 0.02)	0.15	0.11
Kenya	WASH−B (42)	190	175	20.0	−0.07 (−0.14, 0.00)	0.06	0.10	159	125	23.2	−0.09 (−0.19, 0.01)	0.03	0.08
Madagascar	MAHAY (43)	278	311	30.2	−0.08 (−0.16, 0.00)	0.04	0.09	304	262	37.0	−0.07 (−0.17, 0.03)	0.03	0.08
Malawi	iLiNS−DYAD−M (44)	117	225	20.9	0.01 (−0.08, 0.11)	0.04	0.08	93	206	26.7	0.07 (−0.04, 0.18)	0.03	0.08
Malawi	iLiNS−DOSE (45)	86	32	53.1	−0.24 (−0.43, −0.05)	0.01	0.04	114	39	41.0	−0.08 (−0.25, 0.09)	0.01	0.06
Mali	PROMIS CS (46)	505	463	56.8	−0.19 (−0.27, −0.11)	0.05	0.09	448	507	63.3	−0.25 (−0.33, −0.17)	0.05	0.09
Zimbabwe	SHINE (HIV−) (47)	821	722	12.2	−0.04 (−0.07, −0.01)	0.36	0.12	742	760	9.5	−0.04 (−0.06, −0.01)	0.42	0.11
Zimbabwe	SHINE (HIV+) (48)	155	138	13.0	−0.09 (−0.16, −0.01)	0.05	0.09	145	140	7.1	−0.01 (−0.06, 0.04)	0.11	0.10
		**4094**	**3138**		**I² = 0.80, Tau² = 0.00**			**3464**	**3142**		**I² = 0.84, Tau² = 0.01**		
**Fixed**					**−0.05 (−0.07, −0.04)**						**−0.06 (−0.08, −0.04)**		
**Random**					**−0.08 (−0.13, −0.03)**						**−0.08 (−0.14, −0.03)**		

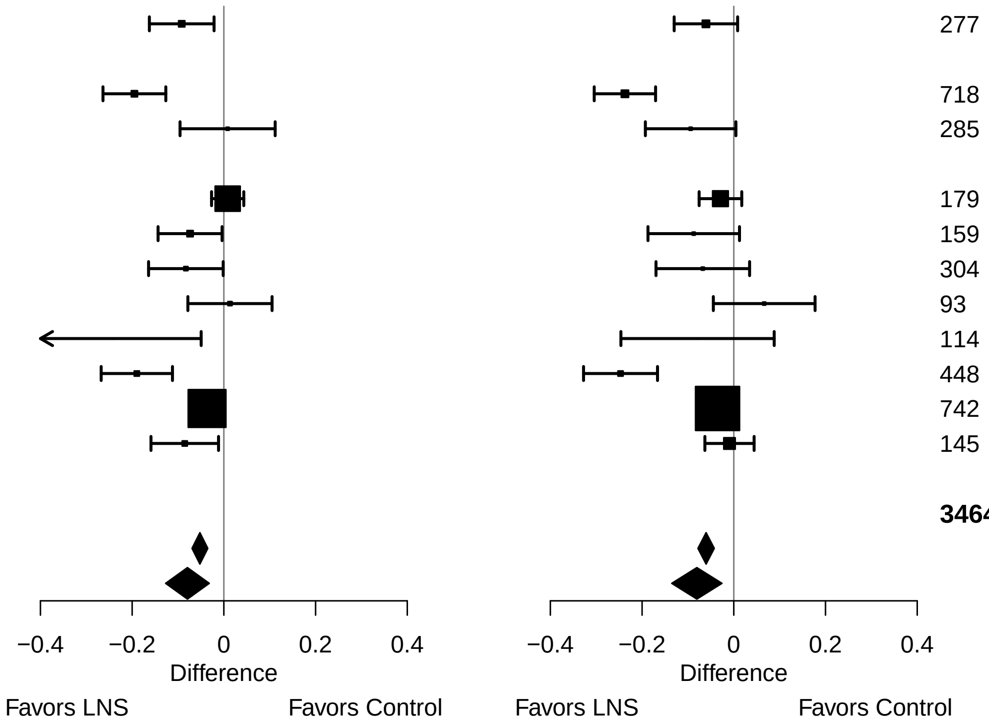

Supplemental figure 9E: Moderate-to-severe anemia prevalence difference

9E2: Stratified by Household food insecurity

P−for−interaction = 0.523
Difference in PDs = −0.01 (−0.05, 0.02)

Country	Trial	Mild to secure: LNS N	Control N	Control Prevalence	PD (95% CI)	Fixed W	Random W	Moderate to severe: LNS N	Control N	Control Prevalence	PD (95% CI)	Fixed W	Random W
Bangladesh	JiVitA−4 (35)												
Bangladesh	RDNS (36)	342	166	13.3	−0.04 (−0.10, 0.02)	0.10	0.18	207	106	21.7	−0.13 (−0.22, −0.05)	0.14	0.16
Bangladesh	WASH−B (37)												
Burkina Faso	iLiNS−Zinc (38)	1016	320	77.2	−0.21 (−0.27, −0.16)	0.10	0.17	934	343	72.9	−0.22 (−0.30, −0.14)	0.15	0.16
Burkina Faso	PROMIS CS (39)												
Ghana	GHANA (40)												
Ghana	iLiNS−DYAD−G (41)												
Kenya	WASH−B (42)	304	273	21.2	−0.08 (−0.14, −0.02)	0.09	0.17	45	27	22.2	−0.09 (−0.23, 0.05)	0.05	0.11
Madagascar	MAHAY (43)	359	340	38.2	−0.11 (−0.20, −0.02)	0.04	0.13	137	140	32.1	0.01 (−0.10, 0.13)	0.07	0.13
Malawi	iLiNS−DYAD−M (44)	66	113	28.3	−0.06 (−0.19, 0.08)	0.02	0.09	143	314	22.0	0.07 (−0.01, 0.16)	0.13	0.15
Malawi	iLiNS−DOSE (45)	50	21	47.6	−0.18 (−0.43, 0.06)	0.01	0.04	144	46	43.5	−0.09 (−0.24, 0.06)	0.04	0.11
Mali	PROMIS CS (46)												
Zimbabwe	SHINE (HIV−) (47)	1253	1162	10.2	−0.02 (−0.05, 0.00)	0.64	0.21	264	293	12.6	−0.06 (−0.11, −0.02)	0.43	0.18
Zimbabwe	SHINE (HIV+) (48)												
		**3390**	**2395**		**I² = 0.84, Tau² = 0.00**			**1874**	**1269**		**I² = 0.79, Tau² = 0.01**		
**Fixed**					**−0.05 (−0.07, −0.04)**						**−0.07 (−0.10, −0.04)**		
**Random**					**−0.09 (−0.14, −0.04)**						**−0.07 (−0.15, 0.00)**		

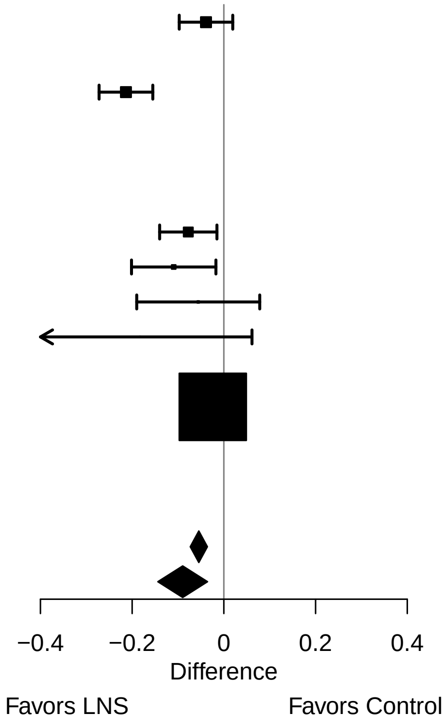

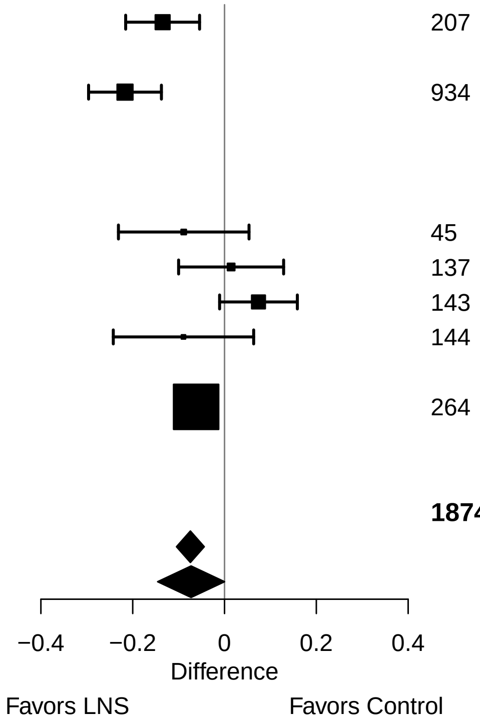

Supplemental figure 9E: Moderate-to-severe anemia prevalence difference

9E3: Stratified by Household source water quality

P−for−interaction = 0.407
Difference in PDs = −0.02 (−0.05, 0.02)

Country	Trial	Improved LNS N	Improved Control N	Improved Control Prevalence	Improved PD (95% CI)	Improved Fixed W	Improved Random W	Unimproved LNS N	Unimproved Control N	Unimproved Control Prevalence	Unimproved PD (95% CI)	Unimproved Fixed W	Unimproved Random W
Bangladesh	JiVitA−4 (35)												
Bangladesh	RDNS (36)												
Bangladesh	WASH−B (37)												
Burkina Faso	iLiNS−Zinc (38)	555	141	75.9	−0.26 (−0.40, −0.12)	0.03	0.14	1394	519	74.6	−0.19 (−0.25, −0.14)	0.26	0.23
Burkina Faso	PROMIS CS (39)	373	363	35.8	−0.02 (−0.09, 0.05)	0.12	0.17	201	217	37.8	−0.08 (−0.19, 0.03)	0.06	0.14
Ghana	GHANA (40)												
Ghana	iLiNS−DYAD−G (41)												
Kenya	WASH−B (42)												
Madagascar	MAHAY (43)	137	164	31.1	0.00 (−0.10, 0.11)	0.06	0.16	433	408	34.3	−0.09 (−0.18, −0.01)	0.10	0.17
Malawi	iLiNS−DYAD−M (44)	191	397	23.2	0.04 (−0.03, 0.11)	0.11	0.17	19	33	30.3	−0.04 (−0.30, 0.22)	0.01	0.04
Malawi	iLiNS−DOSE (45)												
Mali	PROMIS CS (46)	548	567	59.8	−0.23 (−0.31, −0.14)	0.08	0.17	364	388	60.8	−0.20 (−0.29, −0.11)	0.10	0.17
Zimbabwe	SHINE (HIV−) (47)	469	436	9.4	−0.03 (−0.06, 0.00)	0.61	0.19	259	281	10.3	−0.06 (−0.10, −0.02)	0.46	0.26
Zimbabwe	SHINE (HIV+) (48)												
		**2273**	**2068**		**I² = 0.85, Tau² = 0.01**			**2670**	**1846**		**I² = 0.75, Tau² = 0.00**		
**Fixed**					**−0.04 (−0.07, −0.02)**						**−0.11 (−0.14, −0.09)**		
**Random**					**−0.08 (−0.18, 0.02)**						**−0.12 (−0.18, −0.07)**		

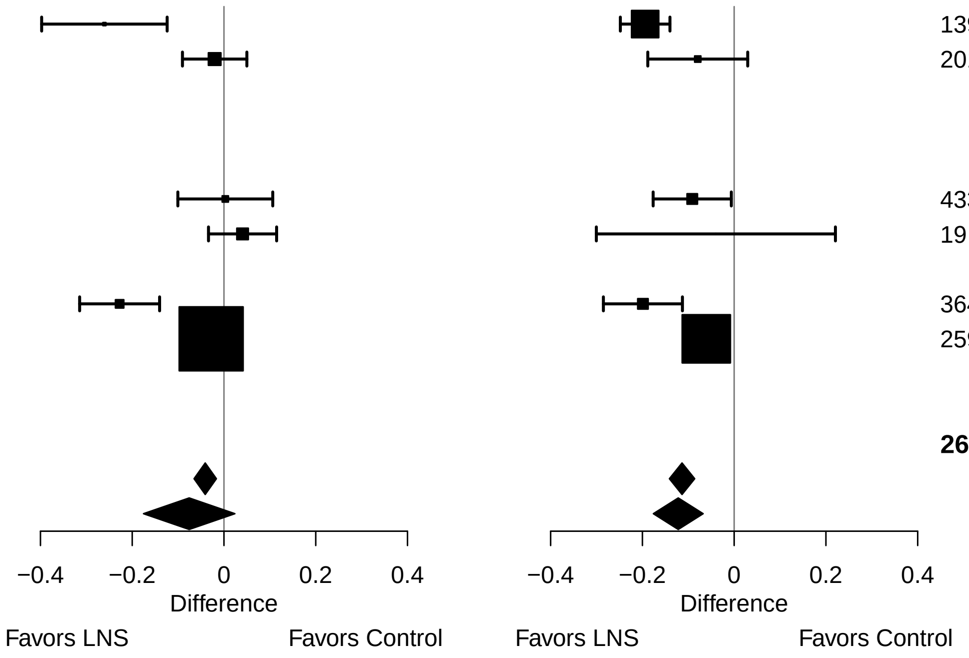

Supplemental figure 9E: Moderate-to-severe anemia prevalence difference

9E4: Stratified by Household sanitation

P−for−interaction = 0.020
Difference in PDs = −0.05 (−0.10, −0.01)

Country	Trial	Improved LNS N	Improved Control N	Improved Control Prevalence	Improved PD (95% CI)	Improved Fixed W	Improved Random W	Unimproved LNS N	Unimproved Control N	Unimproved Control Prevalence	Unimproved PD (95% CI)	Unimproved Fixed W	Unimproved Random W
Bangladesh	JiVitA−4 (35)												
Bangladesh	RDNS (36)	376	193	13.5	−0.04 (−0.10, 0.02)	0.27	0.27	173	79	24.1	−0.17 (−0.25, −0.08)	0.09	0.24
Bangladesh	WASH−B (37)												
Burkina Faso	iLiNS−Zinc (38)												
Burkina Faso	PROMIS CS (39)	244	229	34.1	−0.01 (−0.10, 0.09)	0.10	0.21	329	350	38.3	−0.06 (−0.15, 0.02)	0.09	0.24
Ghana	GHANA (40)												
Ghana	iLiNS−DYAD−G (41)												
Kenya	WASH−B (42)												
Madagascar	MAHAY (43)												
Malawi	iLiNS−DYAD−M (44)												
Malawi	iLiNS−DOSE (45)												
Mali	PROMIS CS (46)	703	696	59.6	−0.19 (−0.26, −0.11)	0.16	0.24	219	249	59.4	−0.31 (−0.40, −0.21)	0.08	0.24
Zimbabwe	SHINE (HIV−) (47)	262	218	11.0	−0.04 (−0.08, 0.01)	0.47	0.28	465	498	9.2	−0.04 (−0.07, −0.01)	0.74	0.27
Zimbabwe	SHINE (HIV+) (48)												
		**1585**	**1336**		**I^2 = 0.77, Tau^2 = 0.00**			**1186**	**1176**		**I^2 = 0.91, Tau^2 = 0.01**		
**Fixed**					**−0.06 (−0.09, −0.03)**						**−0.08 (−0.10, −0.05)**		
**Random**					**−0.07 (−0.14, 0.01)**						**−0.14 (−0.26, −0.02)**		

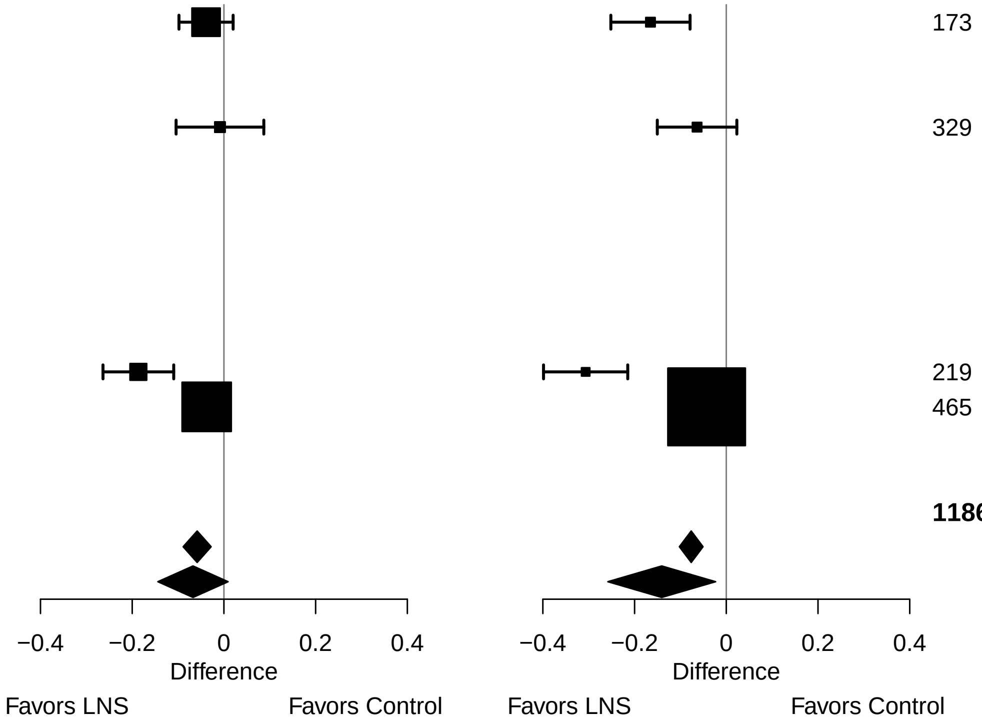

Supplemental figure 9E: Moderate-to-severe anemia prevalence difference

9E5: Stratified by Season at the time of assessment

P−for−interaction = 0.210
Difference in PDs = 0.02 (−0.01, 0.06)

Dry

Country	Trial	LNS N	Control N	Control Prevalence	PD (95% CI)	Fixed W	Random W
Bangladesh	JiVitA−4 (35)						
Bangladesh	RDNS (36)	341	166	20.5	−0.11 (−0.19, −0.04)	0.11	0.12
Bangladesh	WASH−B (37)						
Burkina Faso	iLiNS−Zinc (38)	1398	475	75.8	−0.23 (−0.29, −0.17)	0.16	0.13
Burkina Faso	PROMIS CS (39)	283	245	38.8	−0.08 (−0.18, 0.02)	0.06	0.11
Ghana	GHANA (40)	57	55	34.5	−0.26 (−0.40, −0.11)	0.03	0.08
Ghana	iLiNS−DYAD−G (41)	198	386	5.4	0.00 (−0.04, 0.03)	0.38	0.14
Kenya	WASH−B (42)	100	88	15.9	−0.09 (−0.18, 0.00)	0.07	0.11
Madagascar	MAHAY (43)	517	537	34.6	−0.05 (−0.12, 0.02)	0.10	0.12
Malawi	iLiNS−DYAD−M (44)	147	305	22.0	0.03 (−0.06, 0.11)	0.08	0.12
Malawi	iLiNS−DOSE (45)	123	32	40.6	−0.02 (−0.21, 0.17)	0.02	0.06
Mali	PROMIS CS (46)						
Zimbabwe	SHINE (HIV−) (47)						
Zimbabwe	SHINE (HIV+) (48)						
		**3164**	**2289**		**I^2 = 0.85, Tau^2 = 0.01**		
**Fixed**					**−0.07 (−0.09, −0.05)**		
**Random**					**−0.09 (−0.15, −0.03)**		

Rainy

Country	Trial	LNS N	Control N	Control Prevalence	PD (95% CI)	Fixed W	Random W
Bangladesh	JiVitA−4 (35)						
Bangladesh	RDNS (36)	208	106	10.4	−0.02 (−0.10, 0.06)	0.12	0.13
Bangladesh	WASH−B (37)						
Burkina Faso	iLiNS−Zinc (38)	559	189	73.0	−0.18 (−0.26, −0.09)	0.10	0.13
Burkina Faso	PROMIS CS (39)	291	336	35.1	−0.01 (−0.09, 0.07)	0.12	0.13
Ghana	GHANA (40)	41	41	29.3	−0.17 (−0.34, 0.00)	0.03	0.07
Ghana	iLiNS−DYAD−G (41)	130	274	5.5	−0.02 (−0.06, 0.03)	0.37	0.16
Kenya	WASH−B (42)	250	212	23.6	−0.08 (−0.15, 0.00)	0.15	0.14
Madagascar	MAHAY (43)	83	51	13.7	−0.05 (−0.18, 0.08)	0.04	0.09
Malawi	iLiNS−DYAD−M (44)	63	125	27.2	0.06 (−0.08, 0.20)	0.04	0.09
Malawi	iLiNS−DOSE (45)	120	50	50.0	−0.22 (−0.37, −0.06)	0.03	0.08
Mali	PROMIS CS (46)						
Zimbabwe	SHINE (HIV−) (47)						
Zimbabwe	SHINE (HIV+) (48)						
		**1745**	**1384**		**I^2 = 0.64, Tau^2 = 0.00**		
**Fixed**					**−0.05 (−0.08, −0.02)**		
**Random**					**−0.07 (−0.12, −0.01)**		

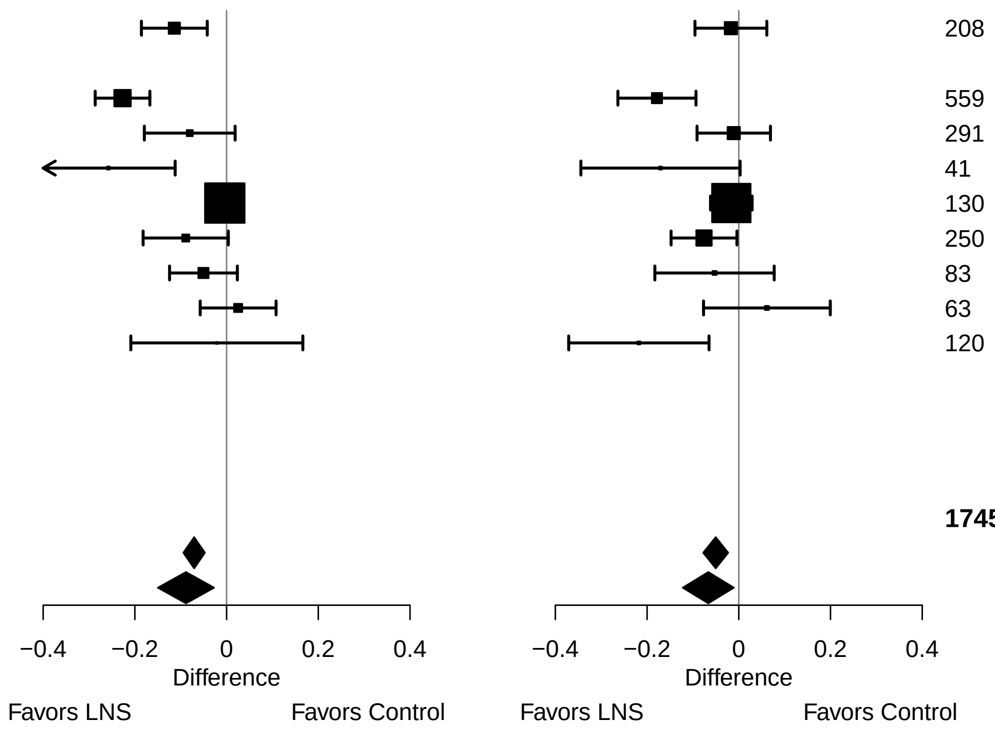

Supplemental figure 9F: Geometric mean ratio of ferritin concentration

9F1: Stratified by Household socio-economic status

P−for−interaction = 0.204
Ratio of GMRs = 0.94 (0.86, 1.03)

		At least median						Less than median					
Country	Trial	LNS N	Control N	Control Median	GMR (95% CI)	Fixed W	Random W	LNS N	Control N	Control Median	GMR (95% CI)	Fixed W	Random W
Bangladesh	JiVitA−4 (35)	243	78	21.4	1.45 (1.15, 1.83)	0.08	0.13	211	66	27.2	1.30 (1.06, 1.61)	0.12	0.15
Bangladesh	RDNS (36)	273	136	19.0	1.64 (1.47, 1.83)	0.38	0.24	277	136	22.0	1.40 (1.24, 1.59)	0.33	0.23
Bangladesh	WASH−B (37)	105	91	15.7	1.73 (1.42, 2.12)	0.11	0.15	107	87	16.2	1.74 (1.46, 2.09)	0.16	0.17
Burkina Faso	iLiNS−Zinc (38)	180	39	10.1	1.42 (1.13, 1.80)	0.08	0.13	135	57	9.6	1.79 (1.47, 2.17)	0.14	0.16
Burkina Faso	PROMIS CS (39)												
Ghana	GHANA (40)	46	39	8.5	2.72 (1.26, 5.86)	0.01	0.02	37	39	11.2	2.39 (1.15, 4.97)	0.01	0.02
Ghana	iLiNS−DYAD−G (41)												
Kenya	WASH−B (42)	163	155	9.5	1.79 (1.56, 2.04)	0.26	0.22	134	104	11.6	1.57 (1.33, 1.87)	0.18	0.18
Madagascar	MAHAY (43)	38	25	16.1	1.29 (1.02, 1.64)	0.08	0.12	43	25	11.1	1.38 (1.01, 1.87)	0.06	0.09
Malawi	iLiNS−DYAD−M (44)												
Malawi	iLiNS−DOSE (45)												
Mali	PROMIS CS (46)												
Zimbabwe	SHINE (HIV−) (47)												
Zimbabwe	SHINE (HIV+) (48)												
		**1048**	**563**		**I² = 0.39, Tau² = 0.01**			**944**	**514**		**I² = 0.43, Tau² = 0.01**		
**Fixed**					**1.63 (1.52, 1.74)**						**1.53 (1.42, 1.64)**		
**Random**					**1.60 (1.44, 1.77)**						**1.54 (1.39, 1.71)**		

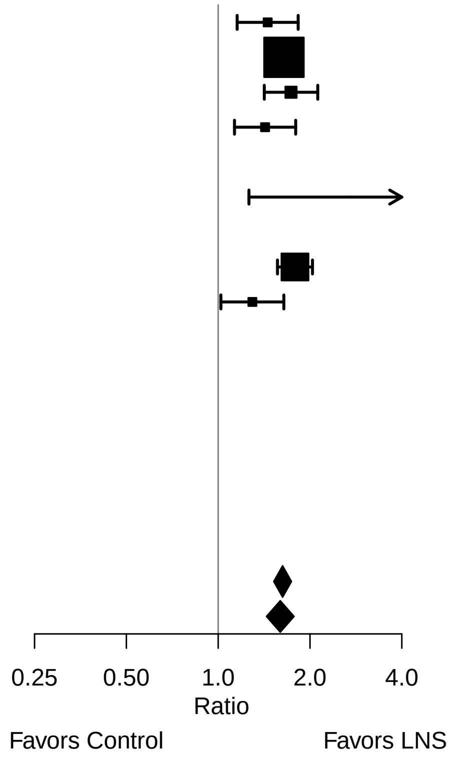

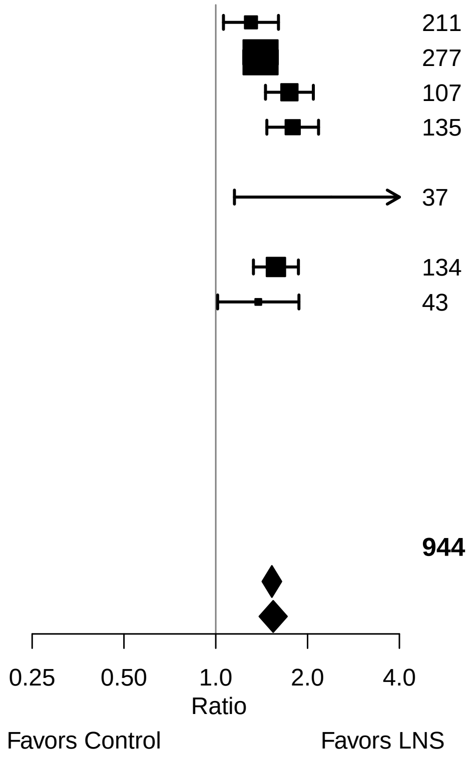

Supplemental figure 9F: Geometric mean ratio of ferritin concentration

9F2: Stratified by Household food insecurity

P−for−interaction = 0.245
Ratio of GMRs = 0.93 (0.82, 1.05)

		Mild to secure						Moderate to severe					
Country	Trial	LNS N	Control N	Control Median	GMR (95% CI)	Fixed W	Random W	LNS N	Control N	Control Median	GMR (95% CI)	Fixed W	Random W
Bangladesh	JiVitA−4 (35)	337	111	22.9	1.43 (1.19, 1.73)	0.13	0.15	117	33	33.0	1.23 (0.95, 1.58)	0.13	0.13
Bangladesh	RDNS (36)	342	166	21.3	1.50 (1.33, 1.69)	0.33	0.30	208	106	19.8	1.55 (1.36, 1.76)	0.48	0.48
Bangladesh	WASH−B (37)	171	139	14.8	1.78 (1.49, 2.12)	0.15	0.16	41	39	17.8	1.60 (1.20, 2.14)	0.10	0.10
Burkina Faso	iLiNS−Zinc (38)	160	45	9.3	1.72 (1.31, 2.26)	0.06	0.07	155	51	10.1	1.41 (1.13, 1.75)	0.17	0.17
Burkina Faso	PROMIS CS (39)												
Ghana	GHANA (40)												
Ghana	iLiNS−DYAD−G (41)												
Kenya	WASH−B (42)	259	235	10.1	1.71 (1.51, 1.94)	0.29	0.28	38	24	10.3	1.55 (1.10, 2.18)	0.07	0.07
Madagascar	MAHAY (43)	45	30	12.8	1.34 (0.93, 1.92)	0.04	0.04	19	7	14.0	1.40 (0.98, 1.99)	0.06	0.06
Malawi	iLiNS−DYAD−M (44)												
Malawi	iLiNS−DOSE (45)												
Mali	PROMIS CS (46)												
Zimbabwe	SHINE (HIV−) (47)												
Zimbabwe	SHINE (HIV+) (48)												
		**1314**	**726**		**I² = 0.18, Tau² = 0.00**			**578**	**260**		**I² = 0.00, Tau² = 0.00**		
**Fixed**					**1.60 (1.49, 1.71)**						**1.47 (1.35, 1.61)**		
**Random**					**1.60 (1.48, 1.73)**						**1.47 (1.35, 1.61)**		

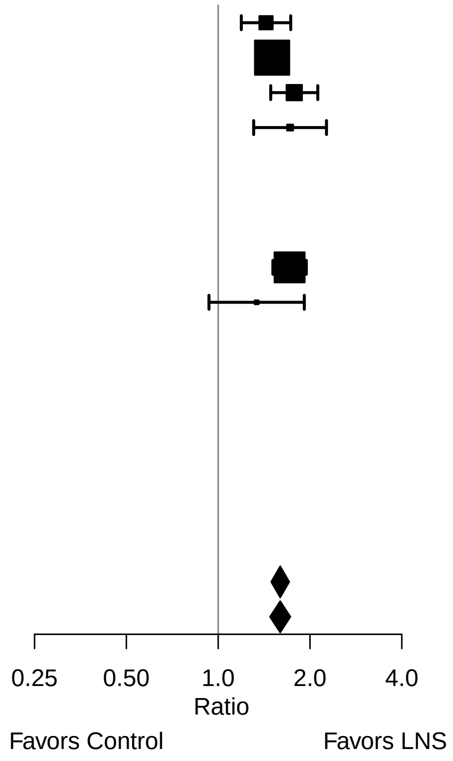

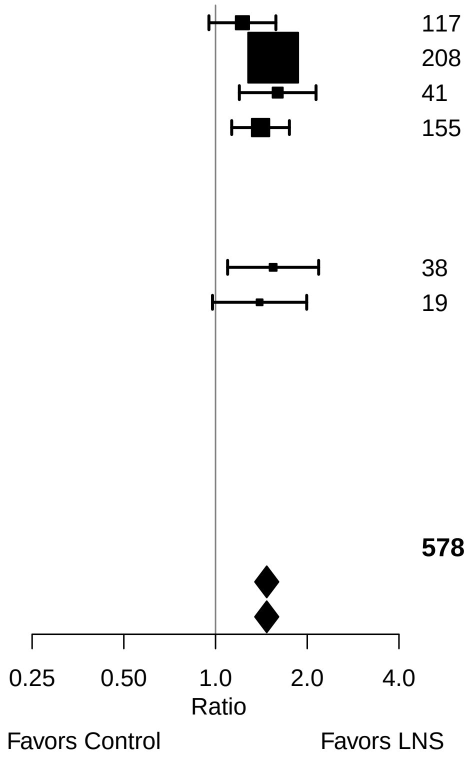

Supplemental figure 9F: Geometric mean ratio of ferritin concentration

9F3: Stratified by Household source water quality

P−for−interaction = 0.408
Ratio of GMRs = 0.93 (0.78, 1.11)

Country	Trial	LNS N	Control N	Control Median	Improved GMR (95% CI)	Fixed W	Random W	LNS N	Control N	Control Median	Unimproved GMR (95% CI)	Fixed W	Random W
Bangladesh	JiVitA−4 (35)												
Bangladesh	RDNS (36)												
Bangladesh	WASH−B (37)	75	44	15.1	1.78 (1.33, 2.39)	0.17	0.17	19	14	19.3	1.69 (1.06, 2.68)	0.08	0.08
Burkina Faso	iLiNS−Zinc (38)	97	22	6.5	1.90 (1.50, 2.41)	0.26	0.26	218	74	10.6	1.47 (1.20, 1.81)	0.40	0.40
Burkina Faso	PROMIS CS (39)												
Ghana	GHANA (40)												
Ghana	iLiNS−DYAD−G (41)												
Kenya	WASH−B (42)	111	107	10.9	1.63 (1.37, 1.94)	0.49	0.49	55	37	9.9	1.84 (1.37, 2.48)	0.19	0.19
Madagascar	MAHAY (43)	23	14	14.0	1.28 (0.83, 1.99)	0.08	0.08	55	36	11.2	1.40 (1.12, 1.75)	0.33	0.33
Malawi	iLiNS−DYAD−M (44)												
Malawi	iLiNS−DOSE (45)												
Mali	PROMIS CS (46)												
Zimbabwe	SHINE (HIV−) (47)												
Zimbabwe	SHINE (HIV+) (48)												
		**306**	**187**		**I² = 0.00, Tau² = 0.00**			**347**	**161**		**I² = 0.00, Tau² = 0.00**		
**Fixed**					**1.69 (1.50, 1.91)**						**1.53 (1.34, 1.74)**		
**Random**					**1.69 (1.50, 1.91)**						**1.53 (1.34, 1.74)**		

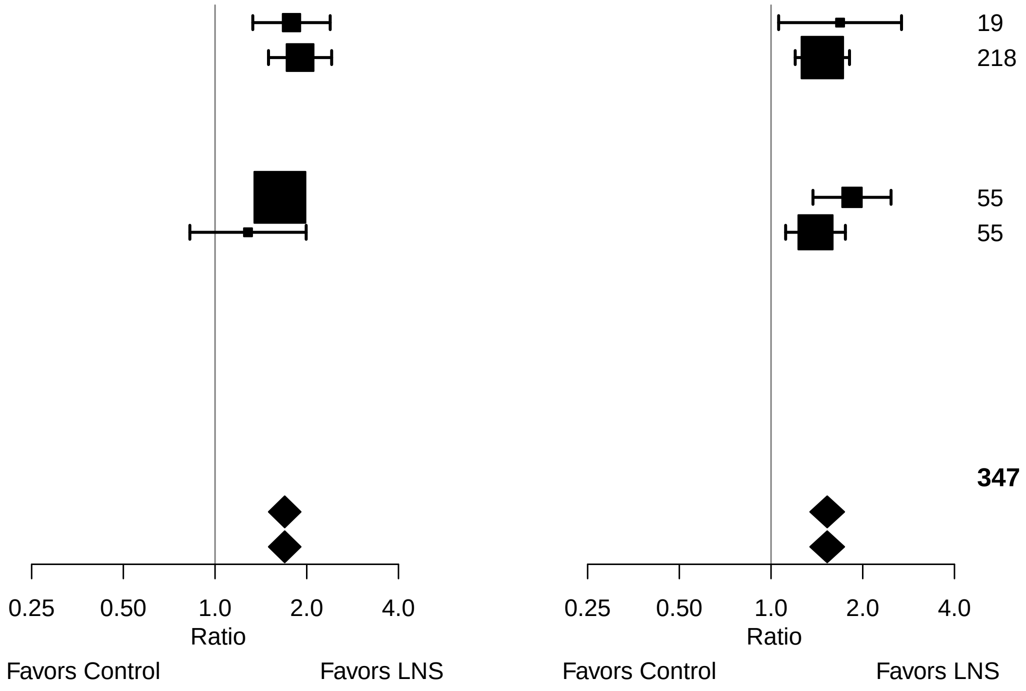

Supplemental figure 9F: Geometric mean ratio of ferritin concentration

9F4: Stratified by Household sanitation

P−for−interaction = 0.512
Ratio of GMRs = 1.05 (0.90, 1.23)

Improved

Country	Trial	LNS N	Control N	Control Median	GMR (95% CI)	Fixed W	Random W
Bangladesh	JiVitA−4 (35)	362	113	23.2	1.44 (1.21, 1.72)	0.20	0.20
Bangladesh	RDNS (36)	377	193	21.2	1.46 (1.33, 1.59)	0.77	0.77
Bangladesh	WASH−B (37)						
Burkina Faso	iLiNS−Zinc (38)						
Burkina Faso	PROMIS CS (39)						
Ghana	GHANA (40)						
Ghana	iLiNS−DYAD−G (41)						
Kenya	WASH−B (42)	21	22	10.5	1.43 (0.91, 2.22)	0.03	0.03
Madagascar	MAHAY (43)						
Malawi	iLiNS−DYAD−M (44)						
Malawi	iLiNS−DOSE (45)						
Mali	PROMIS CS (46)						
Zimbabwe	SHINE (HIV−) (47)						
Zimbabwe	SHINE (HIV+) (48)						
		**760**	**328**		**I² = 0.00, Tau² = 0.00**		
**Fixed**					**1.45 (1.34, 1.57)**		
**Random**					**1.45 (1.34, 1.57)**		

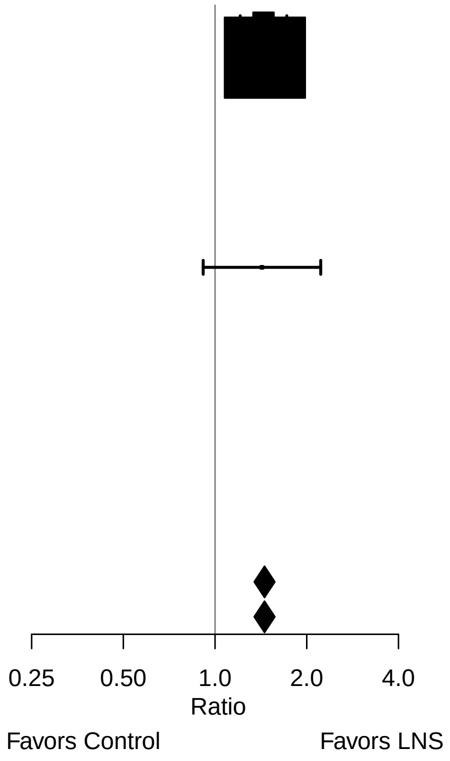

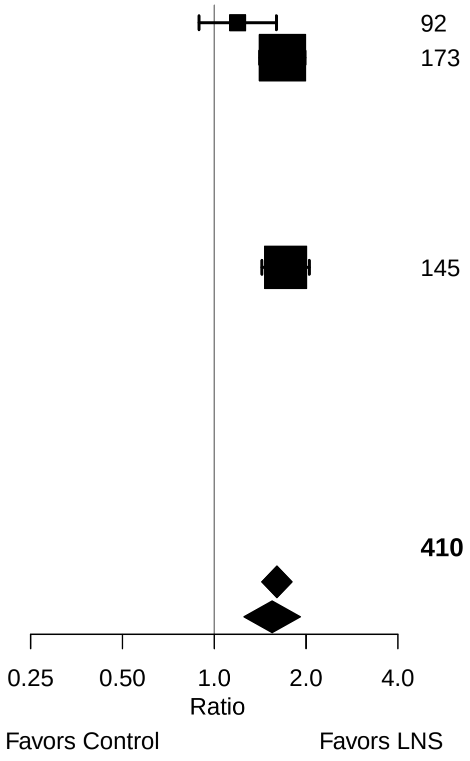

Unimproved

Country	Trial	LNS N	Control N	Control Median	GMR (95% CI)	Fixed W	Random W
Bangladesh	JiVitA−4 (35)	92	31	30.4	1.19 (0.89, 1.60)	0.15	0.26
Bangladesh	RDNS (36)	173	79	19.8	1.67 (1.41, 1.98)	0.44	0.38
Bangladesh	WASH−B (37)						
Burkina Faso	iLiNS−Zinc (38)						
Burkina Faso	PROMIS CS (39)						
Ghana	GHANA (40)						
Ghana	iLiNS−DYAD−G (41)						
Kenya	WASH−B (42)	145	122	10.6	1.71 (1.43, 2.05)	0.40	0.37
Madagascar	MAHAY (43)						
Malawi	iLiNS−DYAD−M (44)						
Malawi	iLiNS−DOSE (45)						
Mali	PROMIS CS (46)						
Zimbabwe	SHINE (HIV−) (47)						
Zimbabwe	SHINE (HIV+) (48)						
		**410**	**232**		**I² = 0.57, Tau² = 0.02**		
**Fixed**					**1.61 (1.43, 1.80)**		
**Random**					**1.55 (1.25, 1.91)**		

Supplemental figure 9F: Geometric mean ratio of ferritin concentration

9F5: Stratified by Season at the time of assessment

P−for−interaction = 0.012
Ratio of GMRs = 1.13 (1.03, 1.25)

		Dry						Rainy					
Country	Trial	LNS N	Control N	Control Median	GMR (95% CI)	Fixed W	Random W	LNS N	Control N	Control Median	GMR (95% CI)	Fixed W	Random W
Bangladesh	JiVitA−4 (35)	263	71	21.3	1.41 (1.17, 1.69)	0.17	0.20	192	73	28.2	1.39 (1.15, 1.69)	0.13	0.17
Bangladesh	RDNS (36)	341	166	21.2	1.39 (1.25, 1.54)	0.52	0.37	208	106	20.5	1.77 (1.56, 2.00)	0.32	0.25
Bangladesh	WASH−B (37)	96	93	17.7	1.50 (1.17, 1.91)	0.09	0.13	116	85	13.2	2.03 (1.73, 2.38)	0.20	0.21
Burkina Faso	iLiNS−Zinc (38)	174	61	9.2	1.54 (1.24, 1.92)	0.12	0.16	126	30	10.4	1.51 (1.19, 1.92)	0.09	0.13
Burkina Faso	PROMIS CS (39)												
Ghana	GHANA (40)	48	47	9.1	3.14 (1.53, 6.42)	0.01	0.02	35	35	11.2	1.88 (0.88, 4.01)	0.01	0.02
Ghana	iLiNS−DYAD−G (41)												
Kenya	WASH−B (42)	88	75	9.5	1.50 (1.17, 1.91)	0.09	0.13	210	184	10.6	1.78 (1.55, 2.06)	0.25	0.22
Madagascar	MAHAY (43)												
Malawi	iLiNS−DYAD−M (44)												
Malawi	iLiNS−DOSE (45)												
Mali	PROMIS CS (46)												
Zimbabwe	SHINE (HIV−) (47)												
Zimbabwe	SHINE (HIV+) (48)												
		**1010**	**513**		**I² = 0.13, Tau² = 0.00**			**887**	**513**		**I² = 0.51, Tau² = 0.01**		
**Fixed**					**1.44 (1.34, 1.55)**						**1.74 (1.62, 1.87)**		
**Random**					**1.46 (1.33, 1.62)**						**1.72 (1.54, 1.92)**		

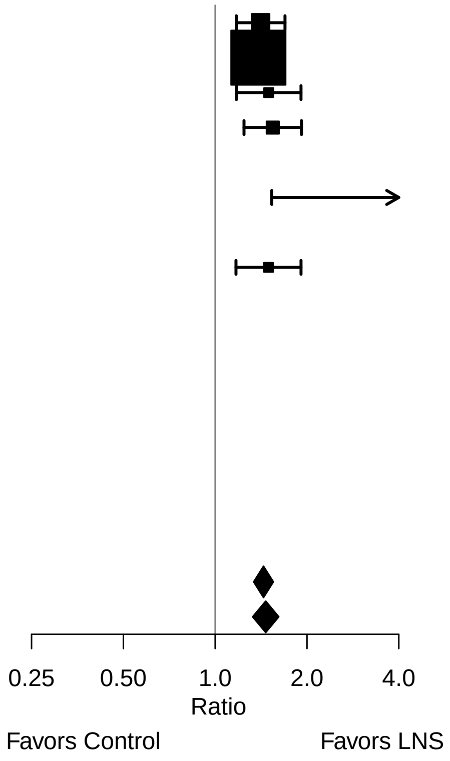

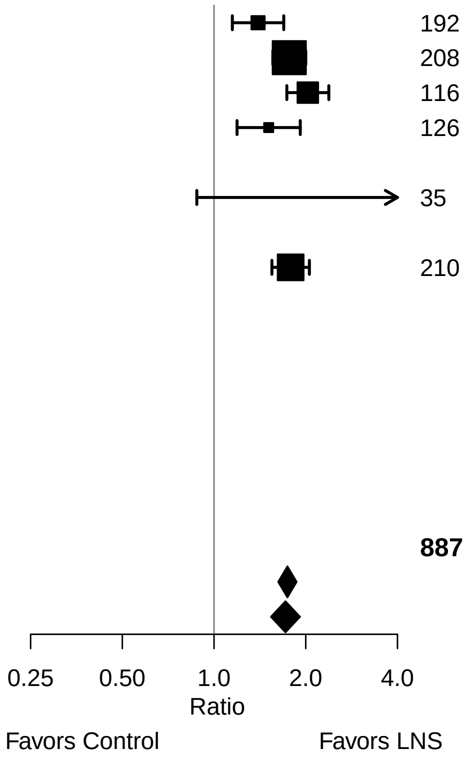

Supplemental figure 9G: Iron deficiency (ferritin < 12 µg/L) prevalence ratio

9G1: Stratified by Household socio-economic status

P−for−interaction = 0.847
Ratio of PRs = 0.98 (0.78, 1.23)

		At least median						Less than median					
Country	Trial	LNS N	Control N	Control Prevalence	PR (95% CI)	Fixed W	Random W	LNS N	Control N	Control Prevalence	PR (95% CI)	Fixed W	Random W
Bangladesh	JiVitA−4 (35)	243	78	23.1	0.43 (0.23, 0.81)	0.06	0.11	211	66	21.2	0.27 (0.11, 0.64)	0.05	0.05
Bangladesh	RDNS (36)	273	136	27.2	0.26 (0.15, 0.43)	0.08	0.14	277	136	16.9	0.47 (0.29, 0.76)	0.16	0.16
Bangladesh	WASH−B (37)	105	91	37.4	0.23 (0.10, 0.51)	0.04	0.09	107	87	33.3	0.25 (0.13, 0.49)	0.08	0.08
Burkina Faso	iLiNS−Zinc (38)	180	39	60.5	0.70 (0.55, 0.88)	0.41	0.22	135	57	57.1	0.46 (0.32, 0.65)	0.30	0.30
Burkina Faso	PROMIS CS (39)												
Ghana	GHANA (40)	46	39	59.0	0.44 (0.25, 0.77)	0.08	0.13	37	39	51.3	0.58 (0.32, 1.04)	0.11	0.11
Ghana	iLiNS−DYAD−G (41)												
Kenya	WASH−B (42)	163	155	62.6	0.37 (0.28, 0.49)	0.30	0.21	134	104	53.8	0.47 (0.32, 0.69)	0.25	0.25
Madagascar	MAHAY (43)	38	25	40.0	0.39 (0.18, 0.85)	0.04	0.09	43	25	64.0	0.33 (0.15, 0.71)	0.06	0.06
Malawi	iLiNS−DYAD−M (44)												
Malawi	iLiNS−DOSE (45)												
Mali	PROMIS CS (46)												
Zimbabwe	SHINE (HIV−) (47)												
Zimbabwe	SHINE (HIV+) (48)												
		**1048**	**563**		**I² = 0.73, Tau² = 0.08**			**944**	**514**		**I² = 0.00, Tau² = 0.00**		
**Fixed**					**0.47 (0.40, 0.54)**						**0.43 (0.36, 0.52)**		
**Random**					**0.41 (0.31, 0.54)**						**0.43 (0.36, 0.52)**		

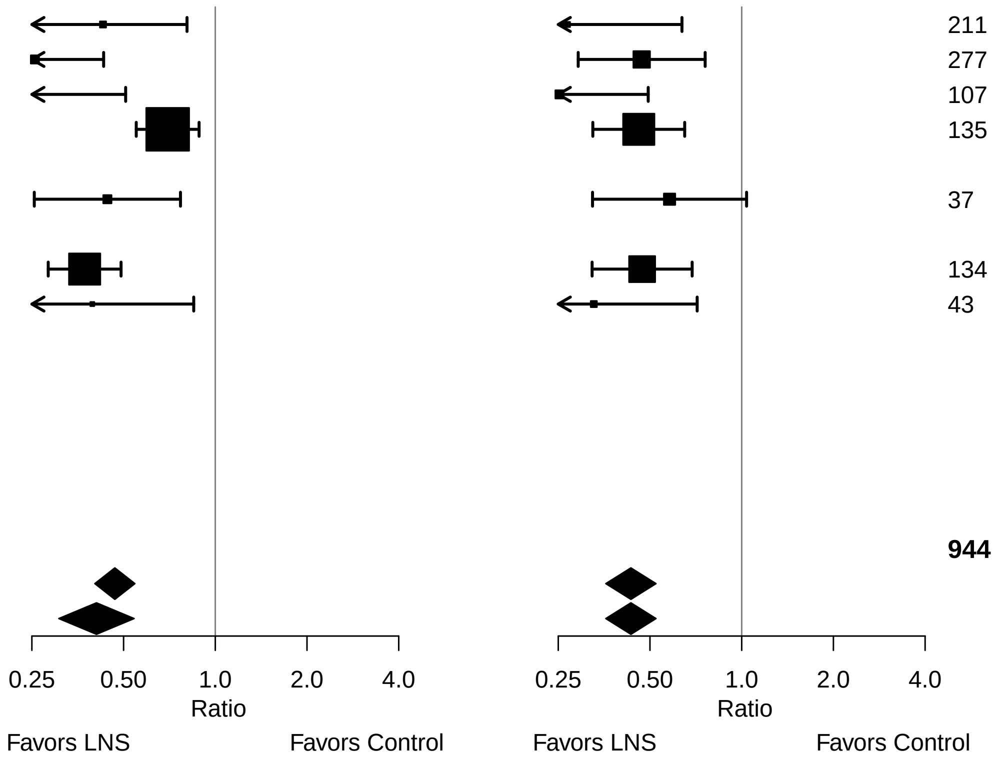

Supplemental figure 9G: Iron deficiency (ferritin < 12 μg/L) prevalence ratio

9G2: Stratified by Household food insecurity

P−for−interaction = 0.859
Ratio of PRs = 1.03 (0.77, 1.38)

		Mild to secure						Moderate to severe					
Country	Trial	LNS N	Control N	Control Prevalence	PR (95% CI)	Fixed W	Random W	LNS N	Control N	Control Prevalence	PR (95% CI)	Fixed W	Random W
Bangladesh	JiVitA−4 (35)	337	111	22.5	0.38 (0.22, 0.66)	0.09	0.16	117	33	21.2	0.28 (0.11, 0.69)	0.07	0.14
Bangladesh	RDNS (36)	342	166	21.1	0.42 (0.26, 0.65)	0.13	0.19	208	106	23.6	0.22 (0.13, 0.39)	0.19	0.22
Bangladesh	WASH−B (37)	171	139	36.7	0.19 (0.10, 0.35)	0.07	0.14	41	39	30.8	0.48 (0.19, 1.19)	0.07	0.13
Burkina Faso	iLiNS−Zinc (38)	160	45	62.7	0.55 (0.41, 0.73)	0.32	0.25	155	51	54.5	0.68 (0.48, 0.97)	0.47	0.29
Burkina Faso	PROMIS CS (39)												
Ghana	GHANA (40)												
Ghana	iLiNS−DYAD−G (41)												
Kenya	WASH−B (42)	259	235	58.3	0.41 (0.31, 0.54)	0.38	0.26	38	24	66.7	0.39 (0.22, 0.69)	0.19	0.22
Madagascar	MAHAY (43)												
Malawi	iLiNS−DYAD−M (44)												
Malawi	iLiNS−DOSE (45)												
Mali	PROMIS CS (46)												
Zimbabwe	SHINE (HIV−) (47)												
Zimbabwe	SHINE (HIV+) (48)												
		**1269**	**696**		**I^2 = 0.59, Tau^2 = 0.08**			**559**	**253**		**I^2 = 0.69, Tau^2 = 0.12**		
**Fixed**					**0.42 (0.36, 0.50)**						**0.45 (0.36, 0.58)**		
**Random**					**0.39 (0.29, 0.54)**						**0.40 (0.26, 0.61)**		

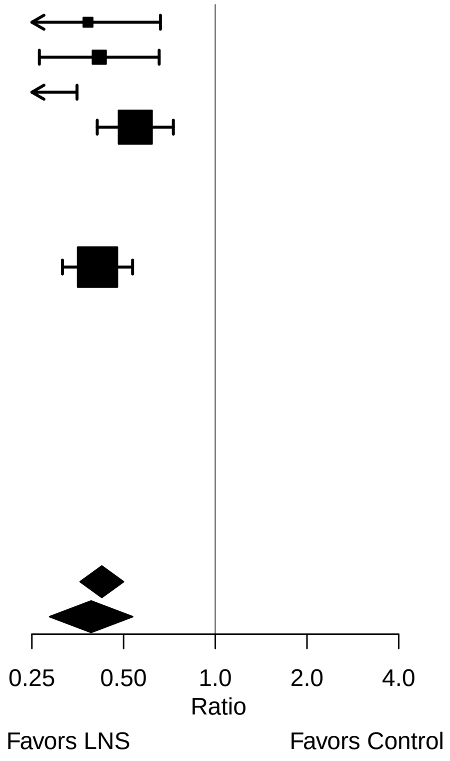

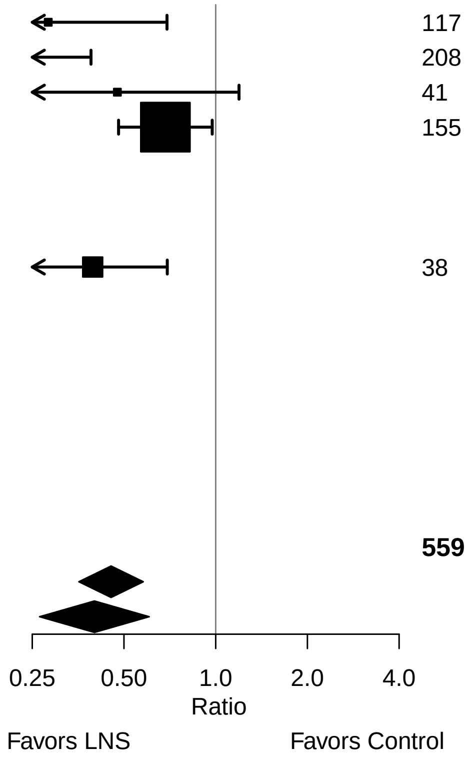

Supplemental figure 9G: Iron deficiency (ferritin < 12 µg/L) prevalence ratio

9G3: Stratified by Household source water quality

P−for−interaction = 0.976
Ratio of PRs = 1.00 (0.78, 1.29)

		Improved						Unimproved					
Country	Trial	LNS N	Control N	Control Prevalence	PR (95% CI)	Fixed W	Random W	LNS N	Control N	Control Prevalence	PR (95% CI)	Fixed W	Random W
Bangladesh	JiVitA−4 (35)												
Bangladesh	RDNS (36)												
Bangladesh	WASH−B (37)												
Burkina Faso	iLiNS−Zinc (38)	97	22	69.6	0.61 (0.47, 0.78)	0.66	0.62	218	74	55.4	0.60 (0.45, 0.81)	0.65	0.46
Burkina Faso	PROMIS CS (39)												
Ghana	GHANA (40)												
Ghana	iLiNS−DYAD−G (41)												
Kenya	WASH−B (42)	111	107	57.0	0.43 (0.29, 0.62)	0.30	0.33	55	37	64.9	0.48 (0.29, 0.79)	0.22	0.31
Madagascar	MAHAY (43)	23	14	42.9	0.51 (0.19, 1.38)	0.04	0.05	55	36	55.6	0.29 (0.15, 0.56)	0.13	0.23
Malawi	iLiNS−DYAD−M (44)												
Malawi	iLiNS−DOSE (45)												
Mali	PROMIS CS (46)												
Zimbabwe	SHINE (HIV−) (47)												
Zimbabwe	SHINE (HIV+) (48)												
		**231**	**143**		**I² = 0.16, Tau² = 0.00**			**328**	**147**		**I² = 0.51, Tau² = 0.06**		
**Fixed**					**0.54 (0.44, 0.66)**						**0.52 (0.41, 0.66)**		
**Random**					**0.54 (0.43, 0.67)**						**0.48 (0.32, 0.70)**		

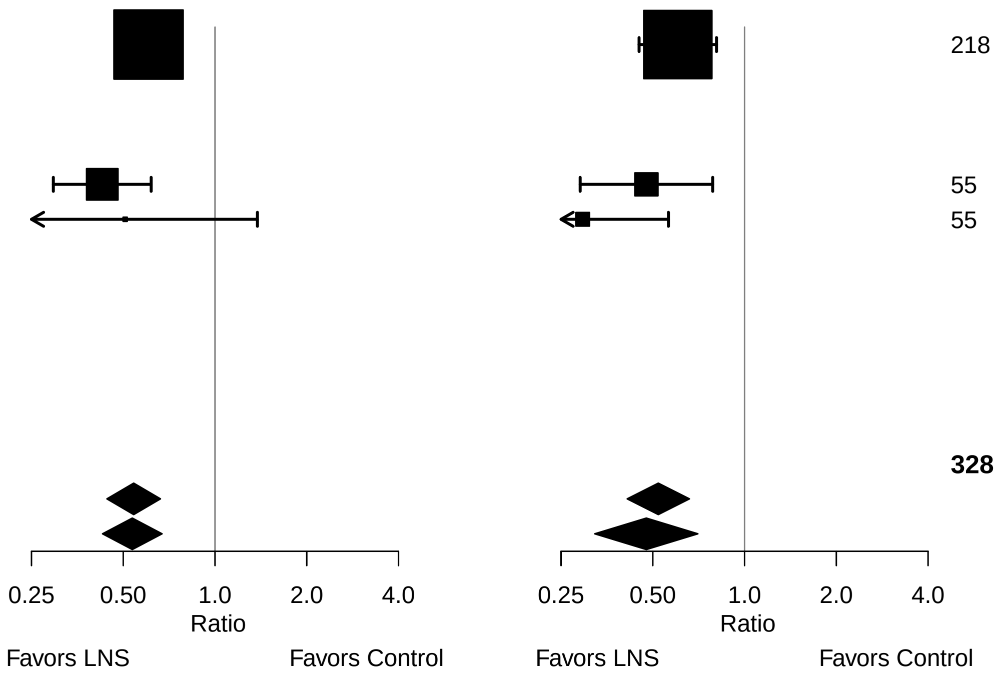

Supplemental figure 9G: Iron deficiency (ferritin < 12 µg/L) prevalence ratio

9G4: Stratified by Household sanitation (insufficient comparisons)

Supplemental figure 9G: Iron deficiency (ferritin < 12 µg/L) prevalence ratio

9G5: Stratified by Season at the time of assessment

P−for−interaction = 0.002
Ratio of PRs = 0.68 (0.53, 0.87)

Country	Trial	Dry: LNS N	Dry: Control N	Dry: Control Prevalence	Dry: PR (95% CI)	Dry: Fixed W	Dry: Random W	Rainy: LNS N	Rainy: Control N	Rainy: Control Prevalence	Rainy: PR (95% CI)	Rainy: Fixed W	Rainy: Random W
Bangladesh	JiVitA−4 (35)	263	71	21.1	0.43 (0.23, 0.81)	0.08	0.08	192	73	23.3	0.27 (0.14, 0.53)	0.08	0.16
Bangladesh	RDNS (36)	341	166	20.5	0.50 (0.35, 0.73)	0.21	0.21	208	106	24.5	0.12 (0.05, 0.26)	0.06	0.14
Bangladesh	WASH−B (37)	96	93	29.0	0.43 (0.22, 0.83)	0.07	0.07	116	85	42.4	0.12 (0.05, 0.28)	0.05	0.14
Burkina Faso	iLiNS−Zinc (38)	174	61	62.0	0.65 (0.49, 0.86)	0.36	0.36	126	30	53.3	0.53 (0.38, 0.74)	0.33	0.20
Burkina Faso	PROMIS CS (39)												
Ghana	GHANA (40)	48	47	57.4	0.51 (0.31, 0.84)	0.12	0.12	35	35	54.3	0.47 (0.25, 0.90)	0.09	0.16
Ghana	iLiNS−DYAD−G (41)												
Kenya	WASH−B (42)	88	75	64.0	0.48 (0.31, 0.74)	0.16	0.16	210	184	57.1	0.38 (0.28, 0.52)	0.39	0.20
Madagascar	MAHAY (43)												
Malawi	iLiNS−DYAD−M (44)												
Malawi	iLiNS−DOSE (45)												
Mali	PROMIS CS (46)												
Zimbabwe	SHINE (HIV−) (47)												
Zimbabwe	SHINE (HIV+) (48)												
		**1010**	**513**		**I² = 0.00, Tau² = 0.00**			**887**	**513**		**I² = 0.76, Tau² = 0.32**		
**Fixed**					**0.53 (0.45, 0.64)**						**0.37 (0.31, 0.45)**		
**Random**					**0.53 (0.45, 0.64)**						**0.29 (0.17, 0.48)**		

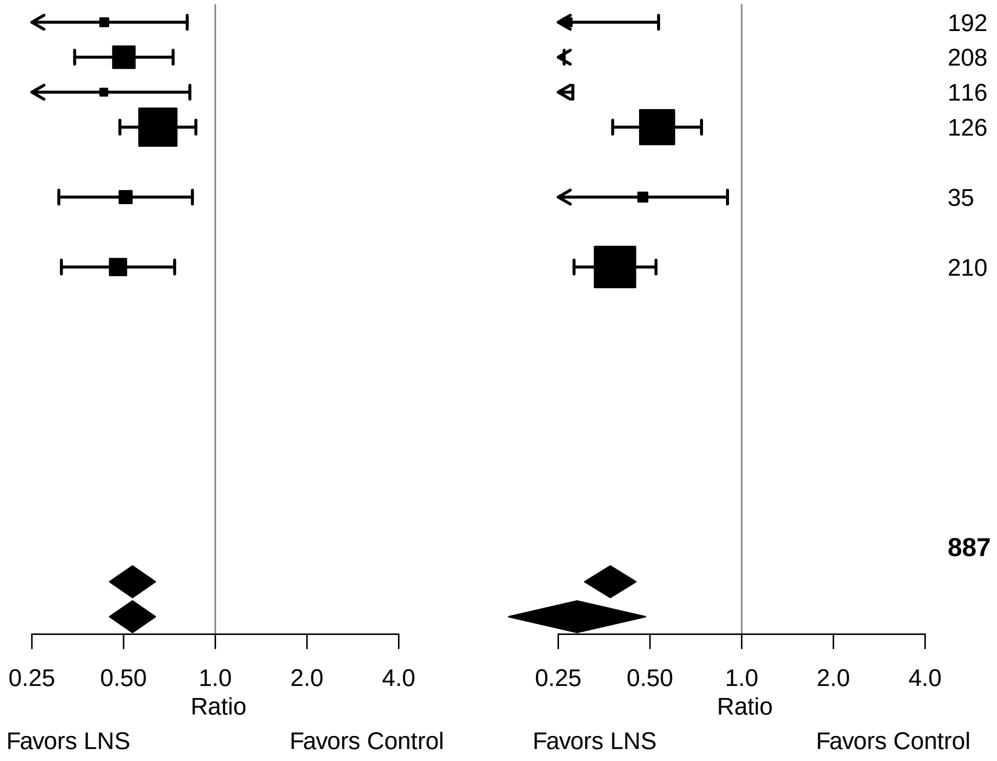

Supplemental figure 9H: Iron deficiency (ferritin < 12 µg/L) prevalence difference

9H1: Stratified by Household socio-economic status

P−for−interaction = 0.158
Difference in PDs = 0.05 (−0.02, 0.11)

		At least median						Less than median					
Country	Trial	LNS N	Control N	Control Prevalence	PD (95% CI)	Fixed W	Random W	LNS N	Control N	Control Prevalence	PD (95% CI)	Fixed W	Random W
Bangladesh	JiVitA−4 (35)	243	78	23.1	−0.13 (−0.25, −0.01)	0.12	0.15	211	66	21.2	−0.16 (−0.28, −0.03)	0.11	0.16
Bangladesh	RDNS (36)	273	136	27.2	−0.20 (−0.27, −0.13)	0.35	0.21	277	136	16.9	−0.09 (−0.15, −0.03)	0.48	0.22
Bangladesh	WASH−B (37)	105	91	37.4	−0.29 (−0.42, −0.15)	0.09	0.13	107	87	33.3	−0.25 (−0.35, −0.14)	0.16	0.17
Burkina Faso	iLiNS−Zinc (38)	180	39	60.5	−0.18 (−0.31, −0.05)	0.10	0.14	135	57	57.1	−0.31 (−0.44, −0.17)	0.09	0.14
Burkina Faso	PROMIS CS (39)												
Ghana	GHANA (40)	46	39	59.0	−0.33 (−0.53, −0.13)	0.04	0.08	37	39	51.3	−0.22 (−0.43, 0.00)	0.04	0.08
Ghana	iLiNS−DYAD−G (41)												
Kenya	WASH−B (42)	163	155	62.6	−0.39 (−0.48, −0.30)	0.21	0.18	134	104	53.8	−0.28 (−0.42, −0.15)	0.10	0.15
Madagascar	MAHAY (43)	38	25	40.0	−0.24 (−0.40, −0.09)	0.07	0.11	43	25	64.0	−0.43 (−0.67, −0.19)	0.03	0.07
Malawi	iLiNS−DYAD−M (44)												
Malawi	iLiNS−DOSE (45)												
Mali	PROMIS CS (46)												
Zimbabwe	SHINE (HIV−) (47)												
Zimbabwe	SHINE (HIV+) (48)												
		**1048**	**563**		**I² = 0.64, Tau² = 0.00**			**944**	**514**		**I² = 0.71, Tau² = 0.01**		
**Fixed**					**−0.25 (−0.29, −0.21)**						**−0.18 (−0.22, −0.13)**		
**Random**					**−0.25 (−0.32, −0.18)**						**−0.22 (−0.30, −0.15)**		

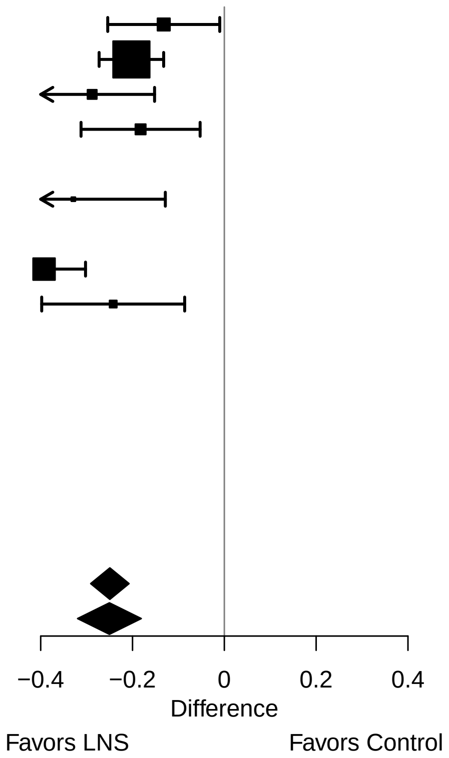

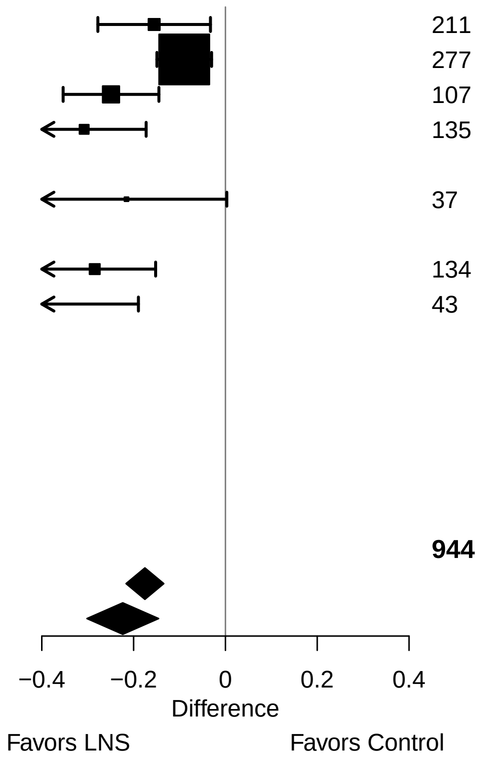

Supplemental figure 9H: Iron deficiency (ferritin < 12 µg/L) prevalence difference

9H2: Stratified by Household food insecurity

P−for−interaction = 0.908
Difference in PDs = 0.00 (−0.08, 0.07)

					Mild to secure						Moderate to severe		
		**LNS**	**Control**	**Control**	**PD**	**Fixed**	**Random**	**LNS**	**Control**	**Control**	**PD**	**Fixed**	**Random**
Country	Trial	N	N	Prevalence	(95% CI)	W	W	N	N	Prevalence	(95% CI)	W	W
Bangladesh	JiVitA−4 (35)	337	111	22.5	−0.14 (−0.24, −0.04)	0.16	0.20	117	33	21.2	−0.15 (−0.28, −0.02)	0.16	0.20
Bangladesh	RDNS (36)	342	166	21.1	−0.12 (−0.19, −0.06)	0.42	0.24	208	106	23.6	−0.18 (−0.25, −0.11)	0.58	0.44
Bangladesh	WASH−B (37)	171	139	36.7	−0.30 (−0.40, −0.19)	0.14	0.19	41	39	30.8	−0.16 (−0.34, 0.01)	0.09	0.13
Burkina Faso	iLiNS−Zinc (38)	160	45	62.7	−0.28 (−0.41, −0.14)	0.09	0.17	155	51	54.5	−0.18 (−0.34, −0.01)	0.10	0.14
Burkina Faso	PROMIS CS (39)												
Ghana	GHANA (40)												
Ghana	iLiNS−DYAD−G (41)												
Kenya	WASH−B (42)	259	235	58.3	−0.34 (−0.43, −0.25)	0.20	0.21	38	24	66.7	−0.40 (−0.61, −0.20)	0.06	0.10
Madagascar	MAHAY (43)												
Malawi	iLiNS−DYAD−M (44)												
Malawi	iLiNS−DOSE (45)												
Mali	PROMIS CS (46)												
Zimbabwe	SHINE (HIV−) (47)												
Zimbabwe	SHINE (HIV+) (48)												
		**1269**	**696**		**I² = 0.81, Tau² = 0.01**			**559**	**253**		**I² = 0.14, Tau² = 0.00**		
**Fixed**					**−0.21 (−0.25, −0.17)**						**−0.19 (−0.24, −0.14)**		
**Random**					**−0.23 (−0.32, −0.14)**						**−0.19 (−0.26, −0.13)**		

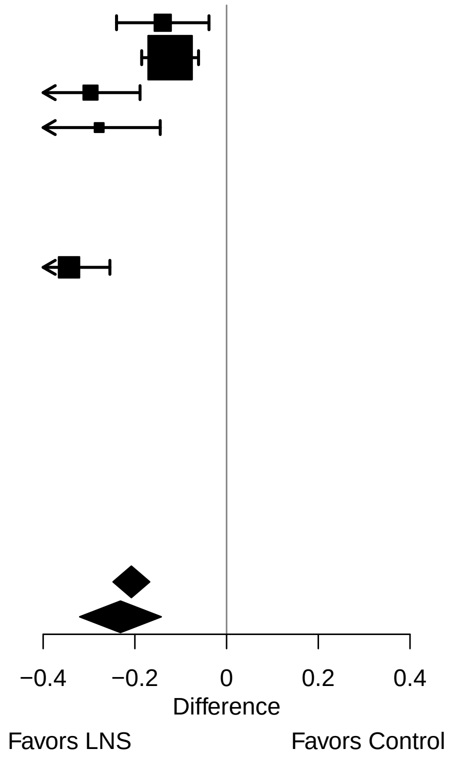

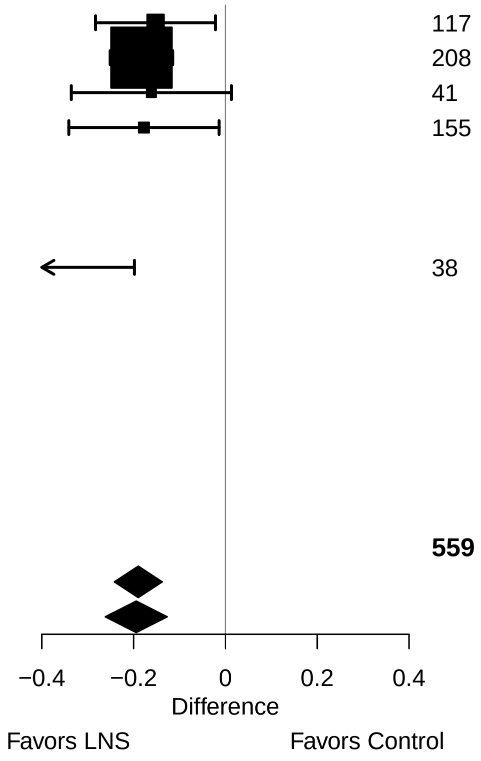

Supplemental figure 9H: Iron deficiency (ferritin < 12 µg/L) prevalence difference

9H3: Stratified by Household source water quality

P−for−interaction = 0.948
Difference in PDs = −0.01 (−0.16, 0.15)

Country	Trial	Improved: LNS N	Improved: Control N	Improved: Control Prevalence	Improved: PD (95% CI)	Improved: Fixed W	Improved: Random W	Unimproved: LNS N	Unimproved: Control N	Unimproved: Control Prevalence	Unimproved: PD (95% CI)	Unimproved: Fixed W	Unimproved: Random W
Bangladesh	JiVitA−4 (35)												
Bangladesh	RDNS (36)												
Bangladesh	WASH−B (37)												
Burkina Faso	iLiNS−Zinc (38)	97	22	69.6	−0.28 (−0.42, −0.14)	0.40	0.40	218	74	55.4	−0.22 (−0.34, −0.10)	0.52	0.45
Burkina Faso	PROMIS CS (39)												
Ghana	GHANA (40)												
Ghana	iLiNS−DYAD−G (41)												
Kenya	WASH−B (42)	111	107	57.0	−0.33 (−0.45, −0.20)	0.51	0.51	55	37	64.9	−0.34 (−0.55, −0.12)	0.17	0.21
Madagascar	MAHAY (43)	23	14	42.9	−0.21 (−0.50, 0.08)	0.09	0.09	55	36	55.6	−0.39 (−0.55, −0.24)	0.32	0.34
Malawi	iLiNS−DYAD−M (44)												
Malawi	iLiNS−DOSE (45)												
Mali	PROMIS CS (46)												
Zimbabwe	SHINE (HIV−) (47)												
Zimbabwe	SHINE (HIV+) (48)												
		**231**	**143**		**I² = 0.00, Tau² = 0.00**			**328**	**147**		**I² = 0.39, Tau² = 0.00**		
**Fixed**					**−0.30 (−0.39, −0.21)**						**−0.29 (−0.38, −0.21)**		
**Random**					**−0.30 (−0.39, −0.21)**						**−0.30 (−0.41, −0.19)**		

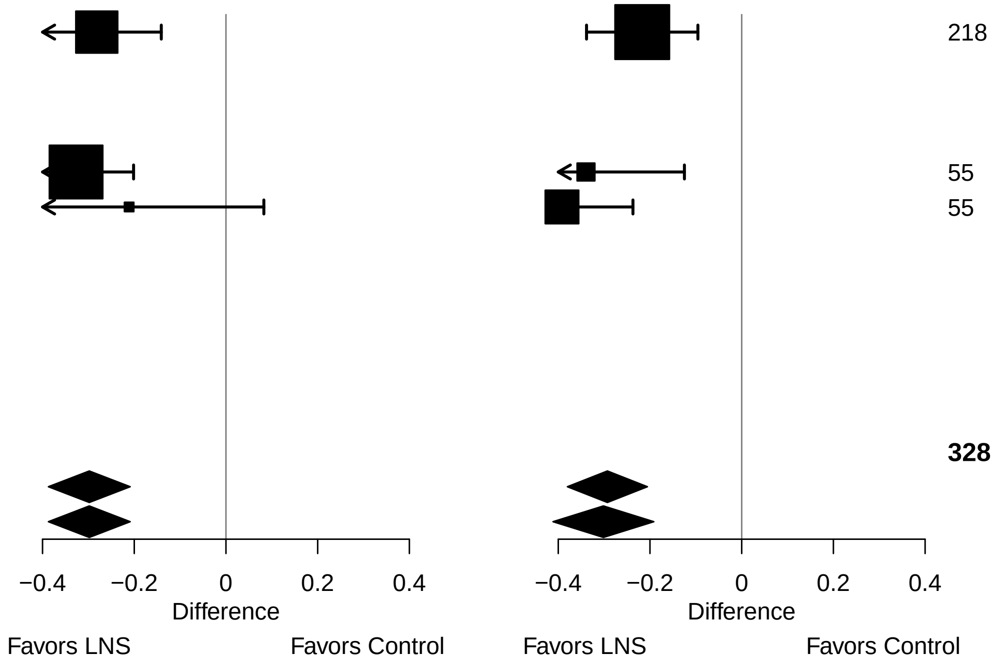

Supplemental figure 9H: Iron deficiency (ferritin < 12 µg/L) prevalence difference

9H4: Stratified by Household sanitation (insufficient comparisons)

Supplemental figure 9H: Iron deficiency (ferritin < 12 µg/L) prevalence difference

9H5: Stratified by Season at the time of assessment

P−for−interaction = 0.007
Difference in PDs = −0.09 (−0.16, −0.02)

Country	Trial	Dry: LNS N	Dry: Control N	Dry: Control Prevalence	Dry: PD (95% CI)	Dry: Fixed W	Dry: Random W	Rainy: LNS N	Rainy: Control N	Rainy: Control Prevalence	Rainy: PD (95% CI)	Rainy: Fixed W	Rainy: Random W
Bangladesh	JiVitA−4 (35)	263	71	21.1	−0.12 (−0.23, −0.01)	0.13	0.19	192	73	23.3	−0.17 (−0.28, −0.06)	0.13	0.17
Bangladesh	RDNS (36)	341	166	20.5	−0.10 (−0.16, −0.05)	0.59	0.30	208	106	24.5	−0.22 (−0.28, −0.16)	0.43	0.25
Bangladesh	WASH−B (37)	96	93	29.0	−0.17 (−0.30, −0.03)	0.10	0.16	116	85	42.4	−0.37 (−0.47, −0.27)	0.15	0.18
Burkina Faso	iLiNS−Zinc (38)	174	61	62.0	−0.22 (−0.37, −0.07)	0.08	0.14	126	30	53.3	−0.25 (−0.39, −0.12)	0.09	0.14
Burkina Faso	PROMIS CS (39)												
Ghana	GHANA (40)	48	47	57.4	−0.28 (−0.48, −0.09)	0.05	0.10	35	35	54.3	−0.29 (−0.51, −0.06)	0.03	0.07
Ghana	iLiNS−DYAD−G (41)												
Kenya	WASH−B (42)	88	75	64.0	−0.33 (−0.50, −0.16)	0.06	0.12	210	184	57.1	−0.35 (−0.45, −0.25)	0.16	0.19
Madagascar	MAHAY (43)												
Malawi	iLiNS−DYAD−M (44)												
Malawi	iLiNS−DOSE (45)												
Mali	PROMIS CS (46)												
Zimbabwe	SHINE (HIV−) (47)												
Zimbabwe	SHINE (HIV+) (48)												
		**1010**	**513**		**I² = 0.52, Tau² = 0.00**			**887**	**513**		**I² = 0.61, Tau² = 0.00**		
**Fixed**					**−0.14 (−0.18, −0.10)**						**−0.26 (−0.30, −0.22)**		
**Random**					**−0.18 (−0.25, −0.11)**						**−0.27 (−0.34, −0.21)**		

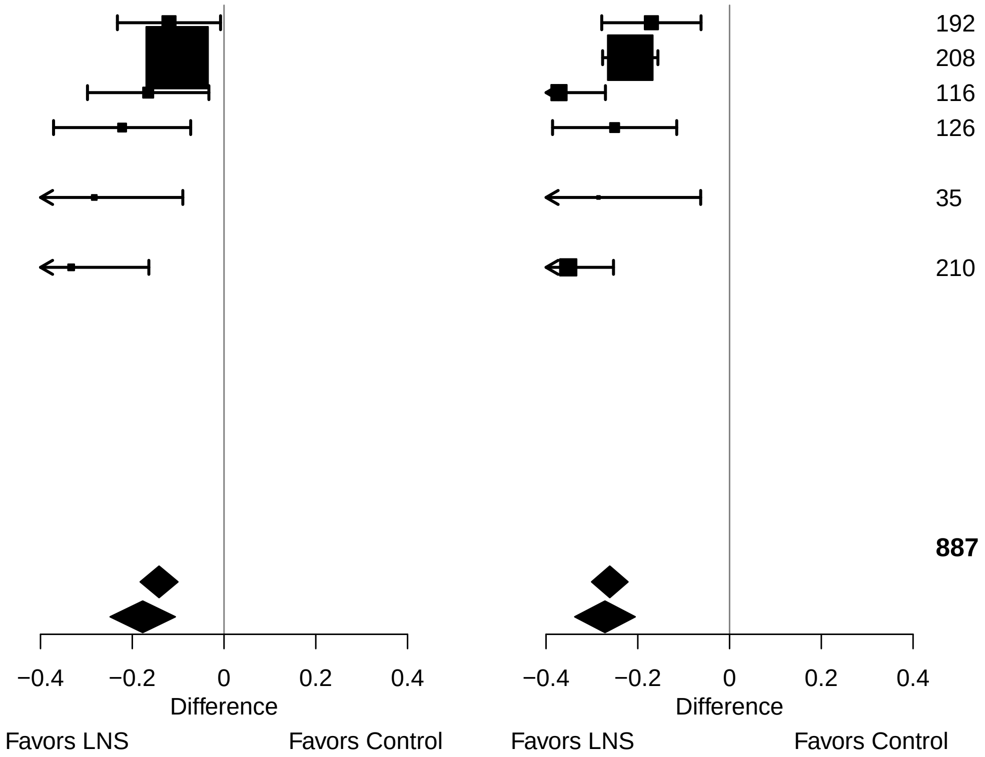

Supplemental figure 9I: Iron deficiency anemia prevalence ratio

9I1: Stratified by Household socio-economic status

P−for−interaction = 0.751
Ratio of PRs = 1.06 (0.74, 1.52)

		At least median						Less than median					
Country	Trial	LNS N	Control N	Control Prevalence	PR (95% CI)	Fixed W	Random W	LNS N	Control N	Control Prevalence	PR (95% CI)	Fixed W	Random W
Bangladesh	JiVitA−4 (35)												
Bangladesh	RDNS (36)	272	136	17.6	0.17 (0.08, 0.33)	0.13	0.29	277	136	11.8	0.37 (0.18, 0.76)	0.18	0.18
Bangladesh	WASH−B (37)												
Burkina Faso	iLiNS−Zinc (38)	188	41	58.5	0.56 (0.40, 0.77)	0.61	0.37	139	60	51.7	0.40 (0.27, 0.59)	0.62	0.62
Burkina Faso	PROMIS CS (39)												
Ghana	GHANA (40)												
Ghana	iLiNS−DYAD−G (41)												
Kenya	WASH−B (42)	163	155	38.1	0.24 (0.15, 0.40)	0.26	0.34	134	103	26.2	0.37 (0.19, 0.73)	0.20	0.20
Madagascar	MAHAY (43)												
Malawi	iLiNS−DYAD−M (44)												
Malawi	iLiNS−DOSE (45)												
Mali	PROMIS CS (46)												
Zimbabwe	SHINE (HIV−) (47)												
Zimbabwe	SHINE (HIV+) (48)												
		**623**	**332**		**I^2 = 0.86, Tau^2 = 0.32**			**550**	**299**		**I^2 = 0.00, Tau^2 = 0.00**		
**Fixed**					**0.38 (0.30, 0.49)**						**0.39 (0.29, 0.52)**		
**Random**					**0.30 (0.15, 0.60)**						**0.39 (0.29, 0.52)**		

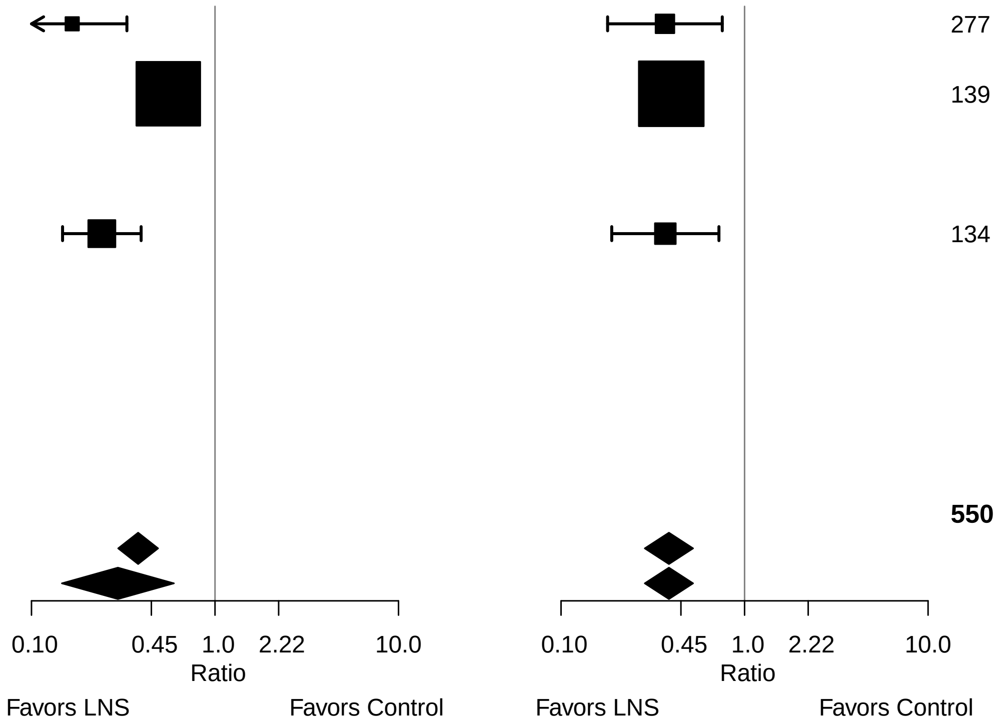

Supplemental figure 9I: Iron deficiency anemia prevalence ratio

9I2: Stratified by Household food insecurity (insufficient comparisons)

Supplemental figure 9I: Iron deficiency anemia prevalence ratio

9I3: Stratified by Household source water quality (insufficient comparisons)

Supplemental figure 9I: Iron deficiency anemia prevalence ratio

9I4: Stratified by Household sanitation (insufficient comparisons)

Supplemental figure 9I: Iron deficiency anemia prevalence ratio

9I5: Stratified by Season at the time of assessment (insufficient comparisons)

Supplemental figure 9J: Iron deficiency anemia prevalence difference

9J1: Stratified by Household socio-economic status

P−for−interaction = 0.039
Difference in PDs = 0.07 (0.00, 0.14)

Country	Trial	LNS N	Control N	Control Prevalence	At least median PD (95% CI)	Fixed W	Random W	LNS N	Control N	Control Prevalence	Less than median PD (95% CI)	Fixed W	Random W
Bangladesh	JiVitA−4 (35)												
Bangladesh	RDNS (36)	272	136	17.6	−0.15 (−0.21, −0.09)	0.60	0.43	277	136	11.8	−0.07 (−0.14, −0.01)	0.67	0.39
Bangladesh	WASH−B (37)												
Burkina Faso	iLiNS−Zinc (38)	188	41	58.5	−0.26 (−0.42, −0.10)	0.09	0.20	139	60	51.7	−0.31 (−0.45, −0.17)	0.13	0.29
Burkina Faso	PROMIS CS (39)												
Ghana	GHANA (40)												
Ghana	iLiNS−DYAD−G (41)												
Kenya	WASH−B (42)	163	155	38.1	−0.29 (−0.37, −0.21)	0.32	0.37	134	103	26.2	−0.17 (−0.28, −0.05)	0.20	0.32
Madagascar	MAHAY (43)												
Malawi	iLiNS−DYAD−M (44)												
Malawi	iLiNS−DOSE (45)												
Mali	PROMIS CS (46)												
Zimbabwe	SHINE (HIV−) (47)												
Zimbabwe	SHINE (HIV+) (48)												
		**623**	**332**		**I^2 = 0.75, Tau^2 = 0.00**			**550**	**299**		**I^2 = 0.80, Tau^2 = 0.01**		
**Fixed**					**−0.20 (−0.25, −0.16)**						**−0.12 (−0.17, −0.07)**		
**Random**					**−0.22 (−0.31, −0.13)**						**−0.17 (−0.30, −0.04)**		

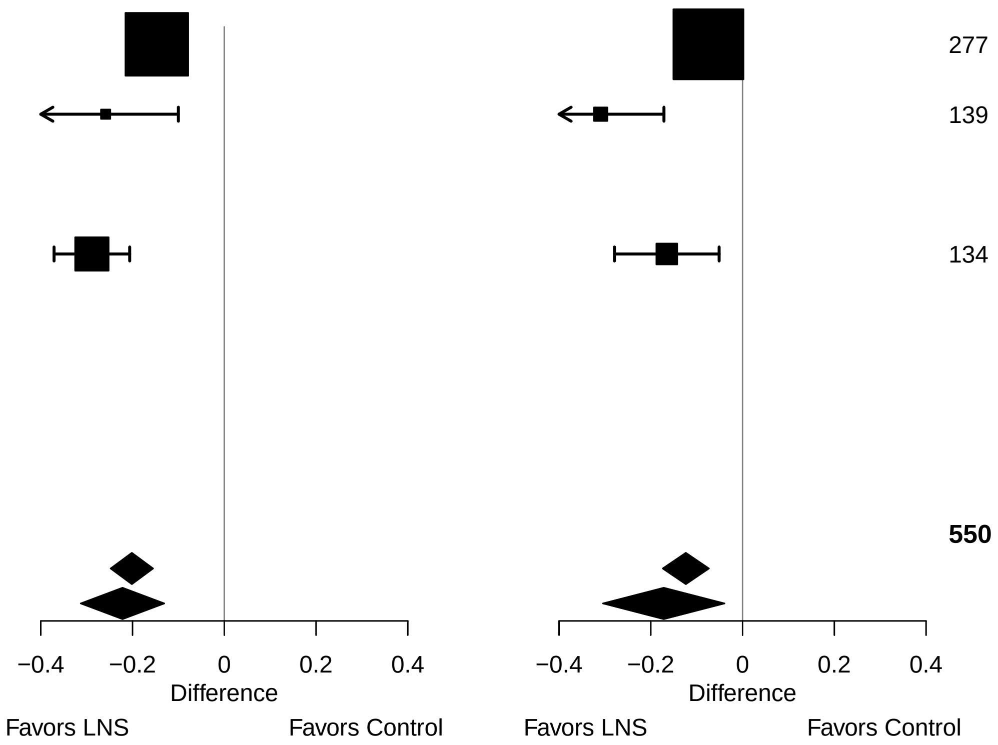

Supplemental figure 9J: Iron deficiency anemia prevalence difference

9J2: Stratified by Household food insecurity (insufficient comparisons)

Supplemental figure 9J: Iron deficiency anemia prevalence difference

9J3: Stratified by Household source water quality (insufficient comparisons)

Supplemental figure 9J: Iron deficiency anemia prevalence difference

9J4: Stratified by Household sanitation (insufficient comparisons)

Supplemental figure 9J: Iron deficiency anemia prevalence difference

9J5: Stratified by Season at the time of assessment (insufficient comparisons)

Supplemental figure 9K: Geometric mean ratio of soluble transferrin receptor concentration

9K1: Stratified by Household socio-economic status

P−for−interaction = 0.373
Ratio of GMRs = 1.03 (0.97, 1.09)

		At least median						Less than median					
		**LNS**	**Control**	**Control**	**GMR**	**Fixed**	**Random**	**LNS**	**Control**	**Control**	**GMR**	**Fixed**	**Random**
Country	Trial	N	N	Median	(95% CI)	W	W	N	N	Median	(95% CI)	W	W
Bangladesh	JiVitA−4 (35)												
Bangladesh	RDNS (36)	273	136	8.3	0.84 (0.78, 0.90)	0.34	0.34	277	136	8.2	0.90 (0.84, 0.97)	0.35	0.27
Bangladesh	WASH−B (37)	105	91	7.4	0.81 (0.73, 0.90)	0.16	0.16	107	87	7.6	0.82 (0.76, 0.89)	0.30	0.26
Burkina Faso	iLiNS−Zinc (38)	180	39	8.6	0.80 (0.71, 0.90)	0.11	0.11	135	57	10.8	0.73 (0.64, 0.84)	0.11	0.14
Burkina Faso	PROMIS CS (39)												
Ghana	GHANA (40)	47	39	9.3	0.81 (0.69, 0.94)	0.07	0.07	37	39	11.3	0.81 (0.69, 0.94)	0.08	0.11
Ghana	iLiNS−DYAD−G (41)												
Kenya	WASH−B (42)	163	155	11.0	0.78 (0.72, 0.84)	0.26	0.26	134	104	10.9	0.81 (0.72, 0.91)	0.13	0.16
Madagascar	MAHAY (43)	38	25	8.5	0.92 (0.77, 1.08)	0.06	0.06	43	25	9.2	0.89 (0.70, 1.13)	0.03	0.06
Malawi	iLiNS−DYAD−M (44)												
Malawi	iLiNS−DOSE (45)												
Mali	PROMIS CS (46)												
Zimbabwe	SHINE (HIV−) (47)												
Zimbabwe	SHINE (HIV+) (48)												
		**806**	**485**		**I² = 0.00, Tau² = 0.00**			**733**	**448**		**I² = 0.45, Tau² = 0.00**		
**Fixed**					**0.82 (0.78, 0.85)**						**0.84 (0.80, 0.87)**		
**Random**					**0.82 (0.78, 0.85)**						**0.83 (0.78, 0.88)**		

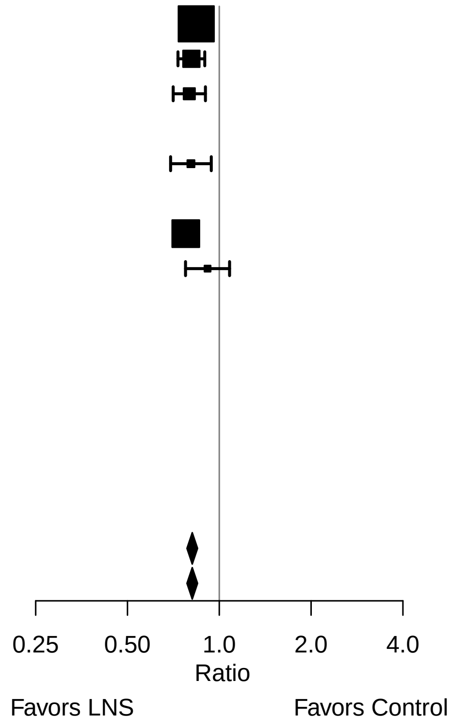

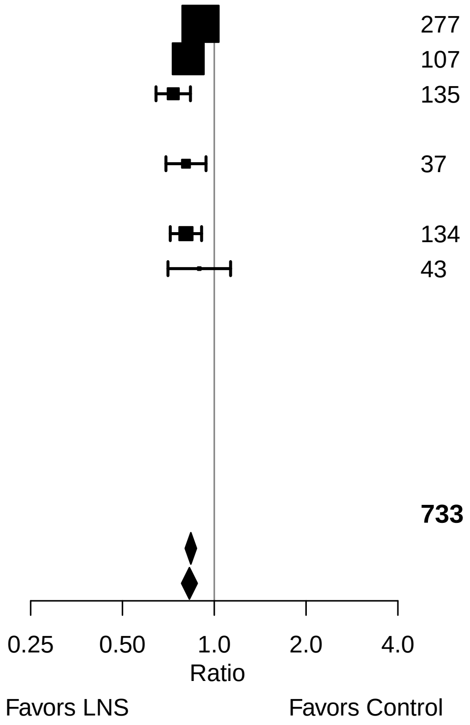

Supplemental figure 9K: Geometric mean ratio of soluble transferrin receptor concentration

9K2: Stratified by Household food insecurity

P−for−interaction = 0.189
Ratio of GMRs = 1.05 (0.98, 1.12)

		Mild to secure						Moderate to severe					
Country	Trial	LNS N	Control N	Control Median	GMR (95% CI)	Fixed W	Random W	LNS N	Control N	Control Median	GMR (95% CI)	Fixed W	Random W
Bangladesh	JiVitA−4 (35)												
Bangladesh	RDNS (36)	342	166	8.4	0.88 (0.82, 0.94)	0.33	0.26	208	106	8.0	0.86 (0.80, 0.92)	0.53	0.39
Bangladesh	WASH−B (37)	171	139	7.5	0.81 (0.74, 0.88)	0.22	0.23	41	39	7.7	0.84 (0.76, 0.94)	0.23	0.25
Burkina Faso	iLiNS−Zinc (38)	160	45	10.3	0.73 (0.66, 0.81)	0.14	0.19	155	51	9.5	0.78 (0.67, 0.91)	0.11	0.16
Burkina Faso	PROMIS CS (39)												
Ghana	GHANA (40)												
Ghana	iLiNS−DYAD−G (41)												
Kenya	WASH−B (42)	259	235	11.0	0.77 (0.71, 0.83)	0.27	0.24	38	24	9.4	0.97 (0.82, 1.14)	0.10	0.13
Madagascar	MAHAY (43)	45	30	9.1	0.87 (0.71, 1.08)	0.04	0.08	19	7	7.7	1.00 (0.77, 1.29)	0.04	0.06
Malawi	iLiNS−DYAD−M (44)												
Malawi	iLiNS−DOSE (45)												
Mali	PROMIS CS (46)												
Zimbabwe	SHINE (HIV−) (47)												
Zimbabwe	SHINE (HIV+) (48)												
		**977**	**615**		**I^2 = 0.63, Tau^2 = 0.00**			**461**	**227**		**I^2 = 0.19, Tau^2 = 0.00**		
**Fixed**					**0.81 (0.78, 0.84)**						**0.86 (0.82, 0.91)**		
**Random**					**0.80 (0.75, 0.86)**						**0.86 (0.81, 0.92)**		

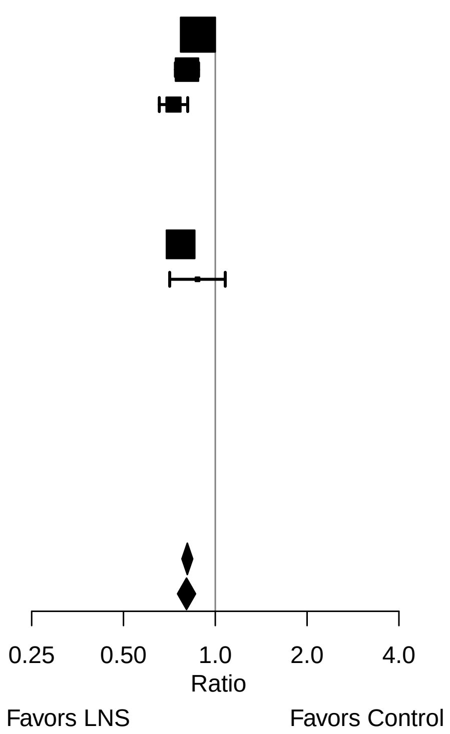

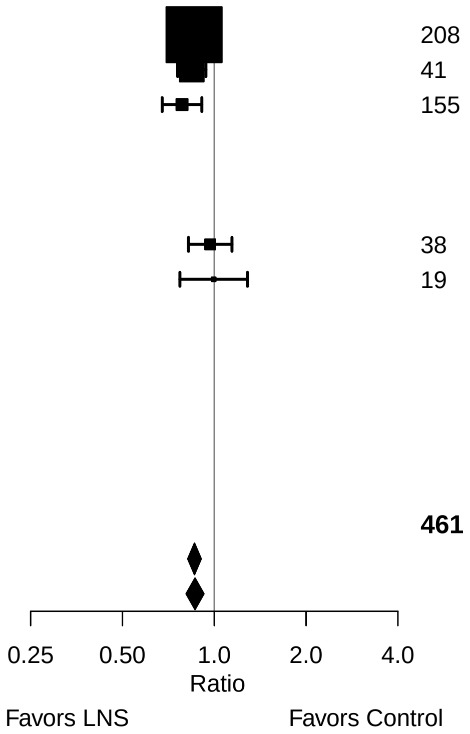

Supplemental figure 9K: Geometric mean ratio of soluble transferrin receptor concentration

9K3: Stratified by Household source water quality

P−for−interaction = 0.039
Ratio of GMRs = 1.11 (1.01, 1.22)

Country	Trial	Improved LNS N	Improved Control N	Improved Control Median	Improved GMR (95% CI)	Improved Fixed W	Improved Random W	Unimproved LNS N	Unimproved Control N	Unimproved Control Median	Unimproved GMR (95% CI)	Unimproved Fixed W	Unimproved Random W
Bangladesh	JiVitA−4 (35)												
Bangladesh	RDNS (36)												
Bangladesh	WASH−B (37)	75	44	8.1	0.70 (0.60, 0.82)	0.22	0.22	19	14	7.8	0.83 (0.70, 0.98)	0.14	0.16
Burkina Faso	iLiNS−Zinc (38)	97	22	9.9	0.74 (0.58, 0.93)	0.09	0.09	218	74	10.0	0.77 (0.70, 0.85)	0.47	0.41
Burkina Faso	PROMIS CS (39)												
Ghana	GHANA (40)												
Ghana	iLiNS−DYAD−G (41)												
Kenya	WASH−B (42)	111	107	10.8	0.76 (0.70, 0.84)	0.64	0.64	55	37	10.5	0.84 (0.73, 0.97)	0.19	0.21
Madagascar	MAHAY (43)	23	14	8.9	0.88 (0.64, 1.23)	0.05	0.05	55	36	9.0	0.91 (0.80, 1.05)	0.20	0.22
Malawi	iLiNS−DYAD−M (44)												
Malawi	iLiNS−DOSE (45)												
Mali	PROMIS CS (46)												
Zimbabwe	SHINE (HIV−) (47)												
Zimbabwe	SHINE (HIV+) (48)												
		**306**	**187**		**I² = 0.00, Tau² = 0.00**			**347**	**161**		**I² = 0.28, Tau² = 0.00**		
**Fixed**					**0.75 (0.70, 0.81)**						**0.82 (0.77, 0.87)**		
**Random**					**0.75 (0.70, 0.81)**						**0.83 (0.77, 0.89)**		

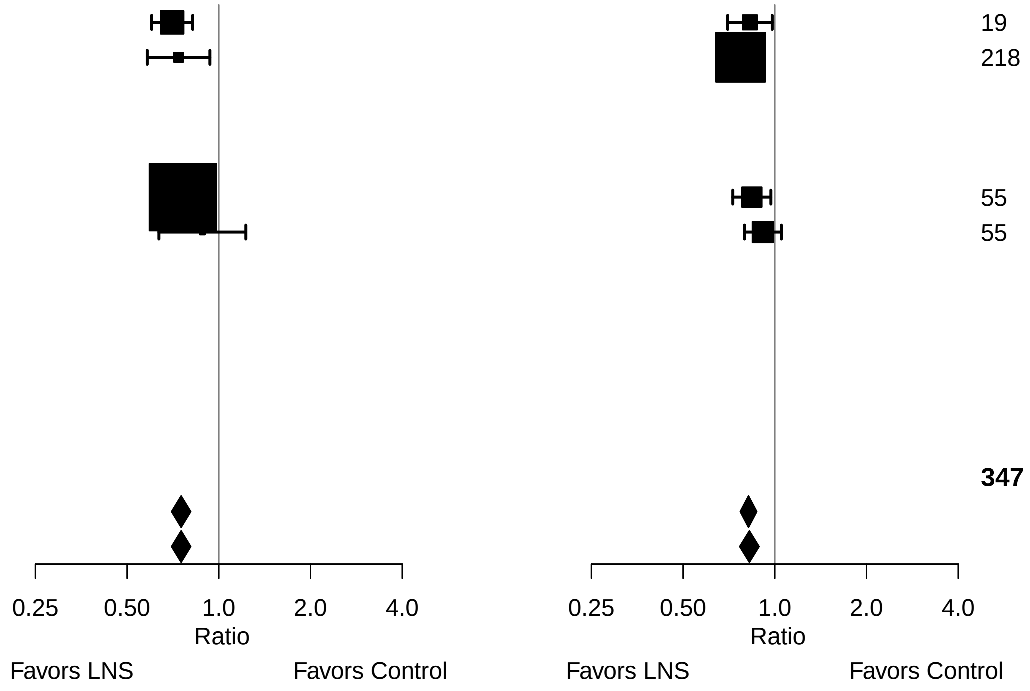

Supplemental figure 9K: Geometric mean ratio of soluble transferrin receptor concentration

9K4: Stratified by Household sanitation (insufficient comparisons)

Supplemental figure 9K: Geometric mean ratio of soluble transferrin receptor concentration

9K5: Stratified by Season at the time of assessment

P−for−interaction = 0.865
Ratio of GMRs = 1.01 (0.95, 1.07)

		Dry						Rainy					
Country	Trial	LNS N	Control N	Control Median	GMR (95% CI)	Fixed W	Random W	LNS N	Control N	Control Median	GMR (95% CI)	Fixed W	Random W
Bangladesh	JiVitA−4 (35)												
Bangladesh	RDNS (36)	341	166	8.2	0.86 (0.82, 0.91)	0.58	0.55	208	106	8.4	0.88 (0.81, 0.97)	0.26	0.25
Bangladesh	WASH−B (37)	96	93	7.6	0.82 (0.75, 0.91)	0.17	0.18	116	85	7.1	0.82 (0.75, 0.89)	0.29	0.27
Burkina Faso	iLiNS−Zinc (38)	174	61	9.6	0.77 (0.66, 0.91)	0.06	0.06	126	30	10.2	0.72 (0.61, 0.84)	0.08	0.11
Burkina Faso	PROMIS CS (39)												
Ghana	GHANA (40)	48	47	9.7	0.80 (0.69, 0.92)	0.08	0.08	36	35	9.6	0.80 (0.68, 0.94)	0.08	0.11
Ghana	iLiNS−DYAD−G (41)												
Kenya	WASH−B (42)	88	75	11.5	0.78 (0.70, 0.88)	0.12	0.13	210	184	10.6	0.79 (0.73, 0.87)	0.28	0.26
Madagascar	MAHAY (43)												
Malawi	iLiNS−DYAD−M (44)												
Malawi	iLiNS−DOSE (45)												
Mali	PROMIS CS (46)												
Zimbabwe	SHINE (HIV−) (47)												
Zimbabwe	SHINE (HIV+) (48)												
		**747**	**442**		**I² = 0.04, Tau² = 0.00**			**696**	**440**		**I² = 0.31, Tau² = 0.00**		
**Fixed**					**0.84 (0.80, 0.87)**						**0.82 (0.78, 0.86)**		
**Random**					**0.83 (0.80, 0.87)**						**0.81 (0.77, 0.86)**		

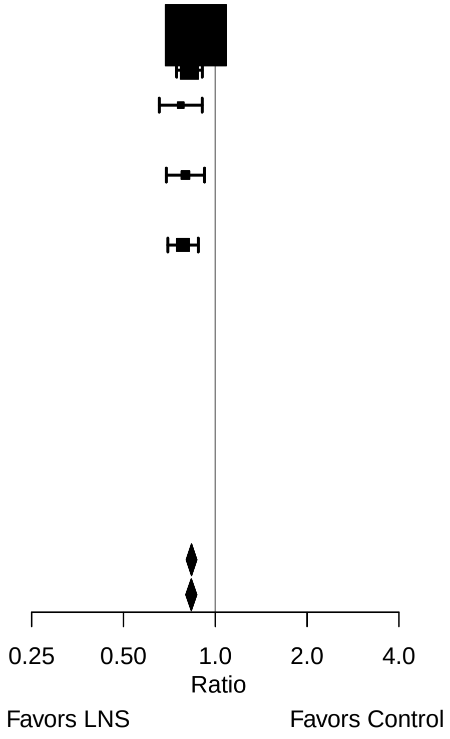

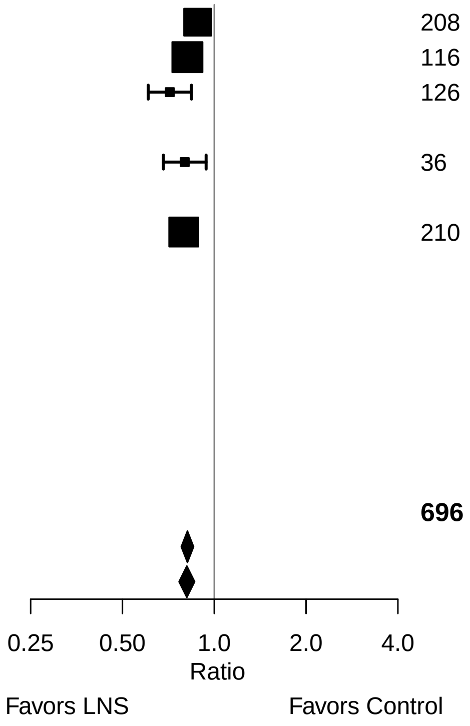

Supplemental figure 9L: Elevated soluble transferrin receptor prevalence ratio

9L1: Stratified by Household socio-economic status

P−for−interaction = 0.066
Ratio of PRs = 1.18 (0.99, 1.41)

		At least median						Less than median					
Country	Trial	LNS N	Control N	Control Prevalence	PR (95% CI)	Fixed W	Random W	LNS N	Control N	Control Prevalence	PR (95% CI)	Fixed W	Random W
Bangladesh	JiVitA−4 (35)												
Bangladesh	RDNS (36)	273	136	51.5	0.60 (0.46, 0.80)	0.18	0.19	277	136	48.5	0.80 (0.65, 0.99)	0.35	0.29
Bangladesh	WASH−B (37)	105	91	36.3	0.16 (0.08, 0.33)	0.03	0.12	107	87	26.4	0.53 (0.32, 0.89)	0.06	0.09
Burkina Faso	iLiNS−Zinc (38)	180	39	53.5	0.53 (0.36, 0.79)	0.09	0.17	135	57	66.7	0.48 (0.30, 0.76)	0.08	0.11
Burkina Faso	PROMIS CS (39)												
Ghana	GHANA (40)	47	39	59.0	0.61 (0.39, 0.97)	0.07	0.16	37	39	64.1	0.55 (0.33, 0.90)	0.07	0.09
Ghana	iLiNS−DYAD−G (41)												
Kenya	WASH−B (42)	163	155	73.5	0.63 (0.53, 0.73)	0.58	0.20	134	104	72.1	0.72 (0.58, 0.90)	0.33	0.28
Madagascar	MAHAY (43)	38	25	52.0	0.81 (0.49, 1.33)	0.06	0.16	43	25	64.0	0.80 (0.54, 1.18)	0.11	0.14
Malawi	iLiNS−DYAD−M (44)												
Malawi	iLiNS−DOSE (45)												
Mali	PROMIS CS (46)												
Zimbabwe	SHINE (HIV−) (47)												
Zimbabwe	SHINE (HIV+) (48)												
		**806**	**485**		**I² = 0.66, Tau² = 0.22**			**733**	**448**		**I² = 0.27, Tau² = 0.01**		
**Fixed**					**0.60 (0.53, 0.68)**						**0.71 (0.62, 0.80)**		
**Random**					**0.53 (0.35, 0.80)**						**0.69 (0.58, 0.81)**		

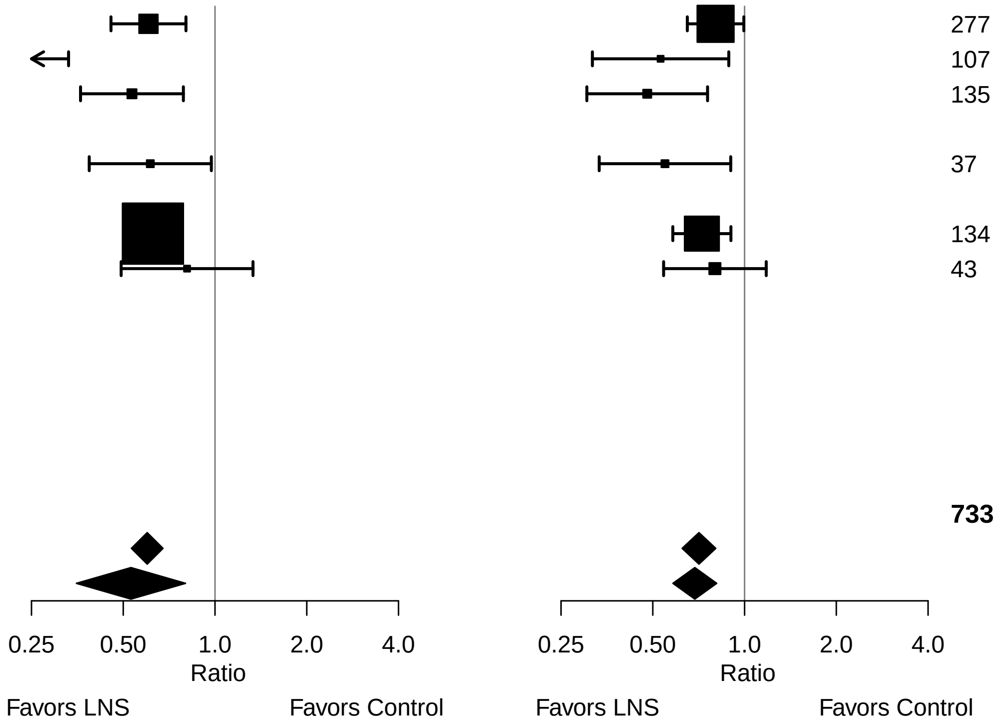

Supplemental figure 9L: Elevated soluble transferrin receptor prevalence ratio

9L2: Stratified by Household food insecurity

P−for−interaction = 0.366
Ratio of PRs = 1.10 (0.89, 1.36)

Country	Trial	Mild to secure: LNS N	Control N	Control Prevalence	PR (95% CI)	Fixed W	Random W	Moderate to severe: LNS N	Control N	Control Prevalence	PR (95% CI)	Fixed W	Random W
Bangladesh	JiVitA−4 (35)												
Bangladesh	RDNS (36)	342	166	51.8	0.73 (0.59, 0.91)	0.27	0.33	208	106	47.2	0.64 (0.49, 0.84)	0.49	0.39
Bangladesh	WASH−B (37)												
Burkina Faso	iLiNS−Zinc (38)	160	45	64.7	0.48 (0.36, 0.64)	0.16	0.27	155	51	58.2	0.54 (0.36, 0.82)	0.21	0.28
Burkina Faso	PROMIS CS (39)												
Ghana	GHANA (40)												
Ghana	iLiNS−DYAD−G (41)												
Kenya	WASH−B (42)	259	235	73.6	0.63 (0.55, 0.74)	0.57	0.39	38	24	66.7	0.95 (0.67, 1.34)	0.29	0.33
Madagascar	MAHAY (43)												
Malawi	iLiNS−DYAD−M (44)												
Malawi	iLiNS−DOSE (45)												
Mali	PROMIS CS (46)												
Zimbabwe	SHINE (HIV−) (47)												
Zimbabwe	SHINE (HIV+) (48)												
		**761**	**446**		**I² = 0.63, Tau² = 0.03**			**401**	**181**		**I² = 0.58, Tau² = 0.05**		
**Fixed**					**0.63 (0.56, 0.71)**						**0.69 (0.57, 0.84)**		
**Random**					**0.62 (0.49, 0.78)**						**0.70 (0.51, 0.95)**		

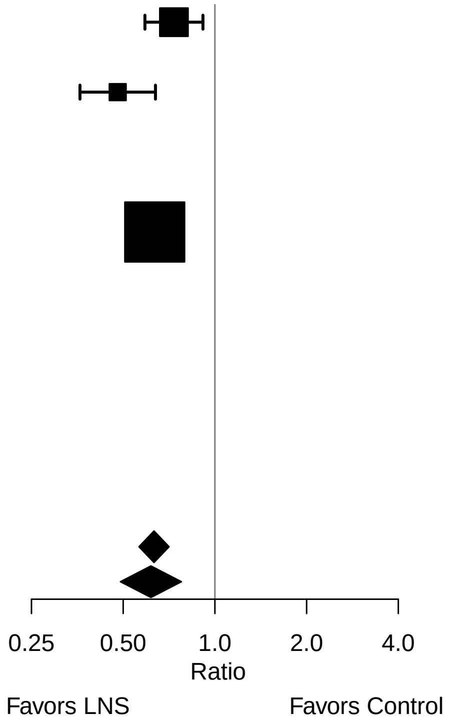

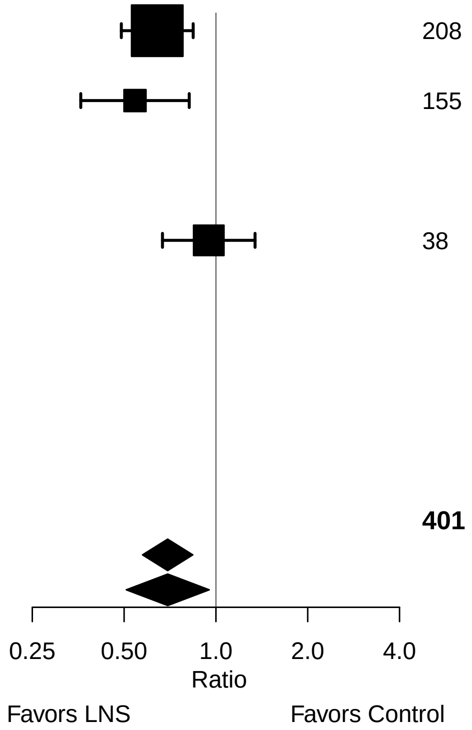

Supplemental figure 9L: Elevated soluble transferrin receptor prevalence ratio

9L3: Stratified by Household source water quality

P−for−interaction = 0.114
Ratio of PRs = 1.24 (0.95, 1.62)

Country	Trial	Improved LNS N	Improved Control N	Improved Control Prevalence	Improved PR (95% CI)	Improved Fixed W	Improved Random W	Unimproved LNS N	Unimproved Control N	Unimproved Control Prevalence	Unimproved PR (95% CI)	Unimproved Fixed W	Unimproved Random W
Bangladesh	JiVitA−4 (35)												
Bangladesh	RDNS (36)												
Bangladesh	WASH−B (37)												
Burkina Faso	iLiNS−Zinc (38)	97	22	65.2	0.42 (0.26, 0.67)	0.12	0.31	218	74	60.2	0.56 (0.44, 0.73)	0.48	0.45
Burkina Faso	PROMIS CS (39)												
Ghana	GHANA (40)												
Ghana	iLiNS−DYAD−G (41)												
Kenya	WASH−B (42)	111	107	76.6	0.59 (0.49, 0.71)	0.81	0.44	55	37	62.2	0.79 (0.54, 1.15)	0.22	0.24
Madagascar	MAHAY (43)	23	14	57.1	0.99 (0.53, 1.85)	0.07	0.25	55	36	58.3	0.72 (0.52, 0.99)	0.29	0.31
Malawi	iLiNS−DYAD−M (44)												
Malawi	iLiNS−DOSE (45)												
Mali	PROMIS CS (46)												
Zimbabwe	SHINE (HIV−) (47)												
Zimbabwe	SHINE (HIV+) (48)												
		**231**	**143**		**I² = 0.57, Tau² = 0.11**			**328**	**147**		**I² = 0.23, Tau² = 0.01**		
**Fixed**					**0.59 (0.49, 0.69)**						**0.65 (0.55, 0.78)**		
**Random**					**0.60 (0.39, 0.94)**						**0.66 (0.54, 0.81)**		

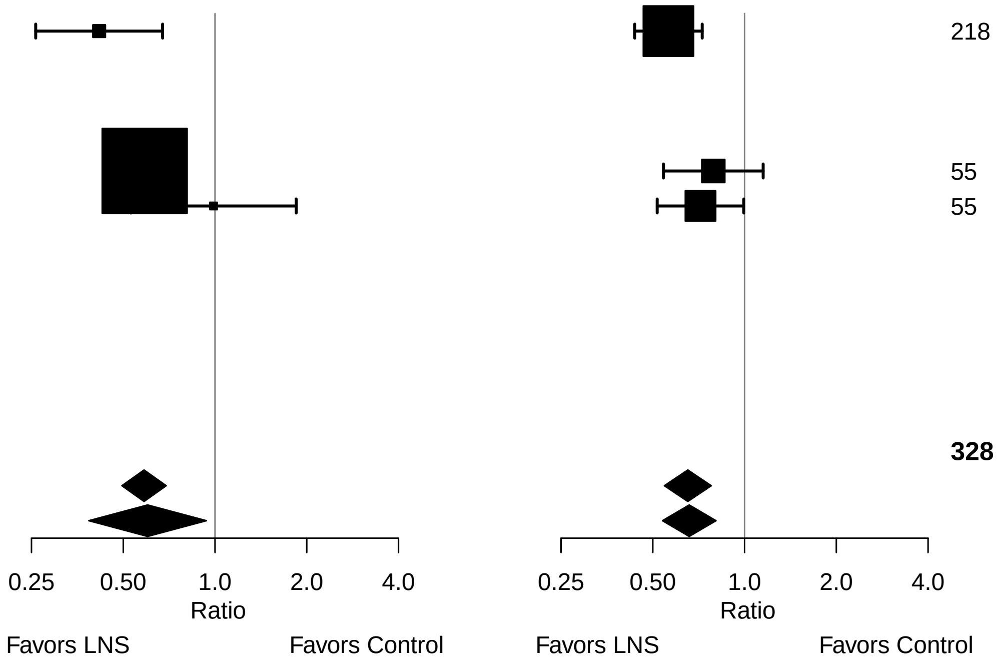

Supplemental figure 9L: Elevated soluble transferrin receptor prevalence ratio

9L4: Stratified by Household sanitation (insufficient comparisons)

Supplemental figure 9L: Elevated soluble transferrin receptor prevalence ratio

9L5: Stratified by Season at the time of assessment

P−for−interaction = 0.154
Ratio of PRs = 1.14 (0.95, 1.35)

		Dry						Rainy					
Country	Trial	LNS N	Control N	Control Prevalence	PR (95% CI)	Fixed W	Random W	LNS N	Control N	Control Prevalence	PR (95% CI)	Fixed W	Random W
Bangladesh	JiVitA−4 (35)												
Bangladesh	RDNS (36)	341	166	49.4	0.62 (0.50, 0.77)	0.39	0.38	208	106	50.9	0.82 (0.65, 1.04)	0.26	0.24
Bangladesh	WASH−B (37)	96	93	31.2	0.40 (0.26, 0.62)	0.09	0.09	116	85	31.8	0.24 (0.12, 0.50)	0.03	0.14
Burkina Faso	iLiNS−Zinc (38)	174	61	59.2	0.50 (0.34, 0.73)	0.12	0.12	126	30	70.0	0.47 (0.29, 0.75)	0.07	0.19
Burkina Faso	PROMIS CS (39)												
Ghana	GHANA (40)	48	47	66.0	0.57 (0.37, 0.86)	0.10	0.11	36	35	57.1	0.58 (0.34, 1.00)	0.05	0.17
Ghana	iLiNS−DYAD−G (41)												
Kenya	WASH−B (42)	88	75	72.0	0.63 (0.50, 0.80)	0.31	0.30	210	184	73.4	0.69 (0.59, 0.81)	0.60	0.26
Madagascar	MAHAY (43)												
Malawi	iLiNS−DYAD−M (44)												
Malawi	iLiNS−DOSE (45)												
Mali	PROMIS CS (46)												
Zimbabwe	SHINE (HIV−) (47)												
Zimbabwe	SHINE (HIV+) (48)												
		**747**	**442**		**I^2 = 0.05, Tau^2 = 0.00**			**696**	**440**		**I^2 = 0.69, Tau^2 = 0.14**		
**Fixed**					**0.58 (0.51, 0.66)**						**0.68 (0.60, 0.76)**		
**Random**					**0.58 (0.50, 0.66)**						**0.56 (0.39, 0.82)**		

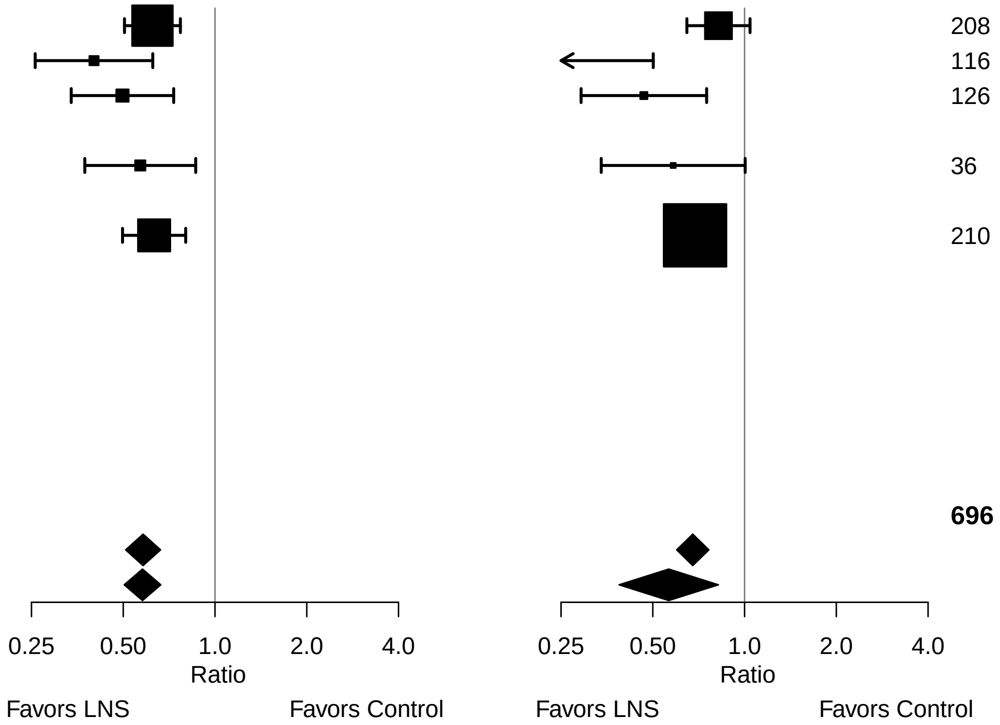

Supplemental figure 9M: Elevated soluble transferrin receptor prevalence difference

9M1: Stratified by Household socio-economic status

P−for−interaction = 0.058
Difference in PDs = 0.07 (0.00, 0.15)

		At least median						Less than median					
Country	Trial	LNS N	Control N	Control Prevalence	PD (95% CI)	Fixed W	Random W	LNS N	Control N	Control Prevalence	PD (95% CI)	Fixed W	Random W
Bangladesh	JiVitA−4 (35)												
Bangladesh	RDNS (36)	273	136	51.5	−0.20 (−0.32, −0.09)	0.22	0.22	277	136	48.5	−0.10 (−0.19, 0.00)	0.32	0.25
Bangladesh	WASH−B (37)	105	91	36.3	−0.31 (−0.42, −0.19)	0.20	0.20	107	87	26.4	−0.12 (−0.22, −0.03)	0.30	0.24
Burkina Faso	iLiNS−Zinc (38)	180	39	53.5	−0.25 (−0.45, −0.05)	0.07	0.07	135	57	66.7	−0.34 (−0.50, −0.17)	0.11	0.14
Burkina Faso	PROMIS CS (39)												
Ghana	GHANA (40)	47	39	59.0	−0.23 (−0.44, −0.02)	0.06	0.06	37	39	64.1	−0.29 (−0.51, −0.07)	0.06	0.09
Ghana	iLiNS−DYAD−G (41)												
Kenya	WASH−B (42)	163	155	73.5	−0.28 (−0.36, −0.19)	0.39	0.39	134	104	72.1	−0.20 (−0.34, −0.06)	0.15	0.18
Madagascar	MAHAY (43)	38	25	52.0	−0.10 (−0.33, 0.13)	0.05	0.05	43	25	64.0	−0.13 (−0.35, 0.09)	0.06	0.09
Malawi	iLiNS−DYAD−M (44)												
Malawi	iLiNS−DOSE (45)												
Mali	PROMIS CS (46)												
Zimbabwe	SHINE (HIV−) (47)												
Zimbabwe	SHINE (HIV+) (48)												
		**806**	**485**		**I² = 0.00, Tau² = 0.00**			**733**	**448**		**I² = 0.41, Tau² = 0.00**		
**Fixed**					**−0.25 (−0.30, −0.20)**						**−0.16 (−0.21, −0.11)**		
**Random**					**−0.25 (−0.30, −0.20)**						**−0.18 (−0.25, −0.10)**		

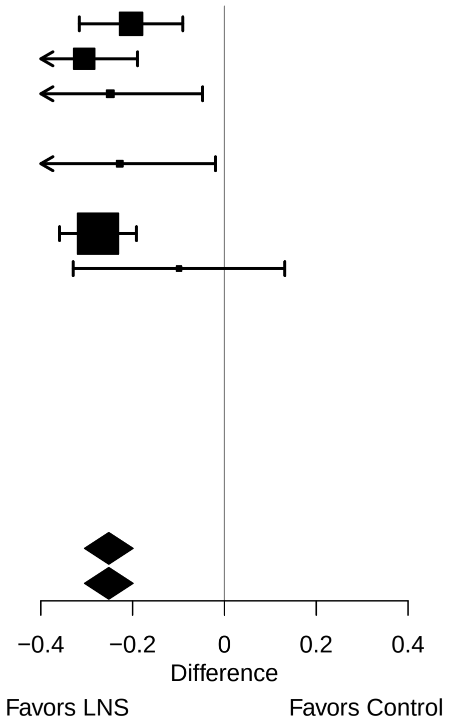

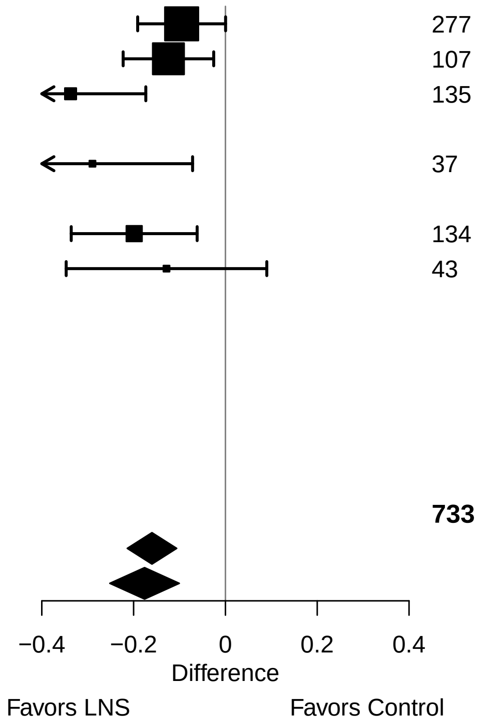

Supplemental figure 9M: Elevated soluble transferrin receptor prevalence difference

9M2: Stratified by Household food insecurity

P−for−interaction = 0.433
Difference in PDs = 0.04 (−0.07, 0.15)

Country	Trial	LNS N	Control N	Control Prevalence	Mild to secure PD (95% CI)	Fixed W	Random W	LNS N	Control N	Control Prevalence	Moderate to severe PD (95% CI)	Fixed W	Random W
Bangladesh	JiVitA−4 (35)												
Bangladesh	RDNS (36)	342	166	51.8	−0.14 (−0.24, −0.04)	0.33	0.35	208	106	47.2	−0.17 (−0.27, −0.06)	0.65	0.56
Bangladesh	WASH−B (37)												
Burkina Faso	iLiNS−Zinc (38)	160	45	64.7	−0.34 (−0.48, −0.19)	0.16	0.27	155	51	58.2	−0.27 (−0.45, −0.08)	0.21	0.26
Burkina Faso	PROMIS CS (39)												
Ghana	GHANA (40)												
Ghana	iLiNS−DYAD−G (41)												
Kenya	WASH−B (42)	259	235	73.6	−0.27 (−0.35, −0.19)	0.51	0.38	38	24	66.7	−0.04 (−0.26, 0.19)	0.14	0.18
Madagascar	MAHAY (43)												
Malawi	iLiNS−DYAD−M (44)												
Malawi	iLiNS−DOSE (45)												
Mali	PROMIS CS (46)												
Zimbabwe	SHINE (HIV−) (47)												
Zimbabwe	SHINE (HIV+) (48)												
		**761**	**446**		**I^2 = 0.67, Tau^2 = 0.01**			**401**	**181**		**I^2 = 0.16, Tau^2 = 0.00**		
**Fixed**					**−0.24 (−0.29, −0.18)**						**−0.17 (−0.26, −0.09)**		
**Random**					**−0.24 (−0.35, −0.13)**						**−0.17 (−0.27, −0.07)**		

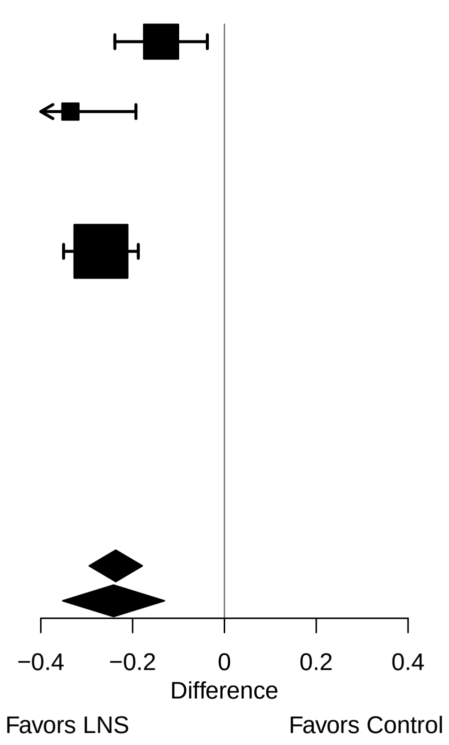

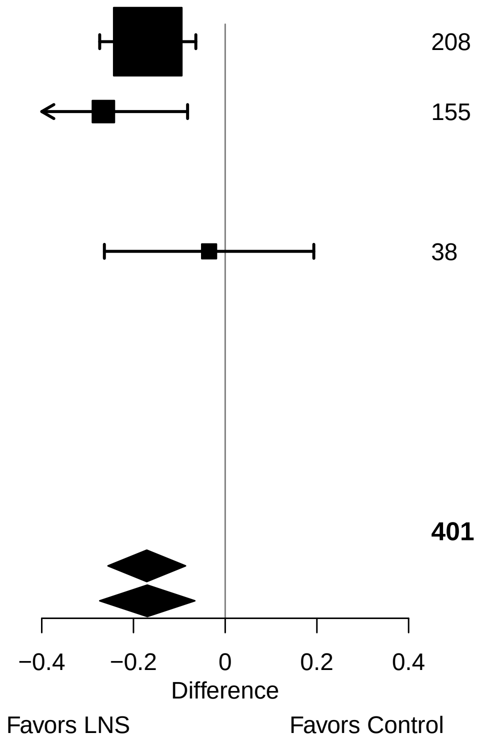

Supplemental figure 9M: Elevated soluble transferrin receptor prevalence difference

9M3: Stratified by Household source water quality

P−for−interaction = 0.231
Difference in PDs = 0.10 (−0.06, 0.25)

Country	Trial	LNS N	Control N	Control Prevalence	Improved PD (95% CI)	Fixed W	Random W	LNS N	Control N	Control Prevalence	Unimproved PD (95% CI)	Fixed W	Random W
Bangladesh	JiVitA−4 (35)												
Bangladesh	RDNS (36)												
Bangladesh	WASH−B (37)												
Burkina Faso	iLiNS−Zinc (38)	97	22	65.2	−0.35 (−0.63, −0.08)	0.12	0.25	218	74	60.2	−0.26 (−0.38, −0.15)	0.56	0.56
Burkina Faso	PROMIS CS (39)												
Ghana	GHANA (40)												
Ghana	iLiNS−DYAD−G (41)												
Kenya	WASH−B (42)	111	107	76.6	−0.32 (−0.42, −0.21)	0.81	0.57	55	37	62.2	−0.13 (−0.34, 0.08)	0.16	0.16
Madagascar	MAHAY (43)	23	14	57.1	−0.01 (−0.36, 0.35)	0.07	0.18	55	36	58.3	−0.17 (−0.33, −0.01)	0.28	0.28
Malawi	iLiNS−DYAD−M (44)												
Malawi	iLiNS−DOSE (45)												
Mali	PROMIS CS (46)												
Zimbabwe	SHINE (HIV−) (47)												
Zimbabwe	SHINE (HIV+) (48)												
		**231**	**143**		**I² = 0.30, Tau² = 0.01**			**328**	**147**		**I² = 0.00, Tau² = 0.00**		
**Fixed**					**−0.30 (−0.39, −0.20)**						**−0.22 (−0.30, −0.13)**		
**Random**					**−0.27 (−0.44, −0.10)**						**−0.22 (−0.30, −0.13)**		

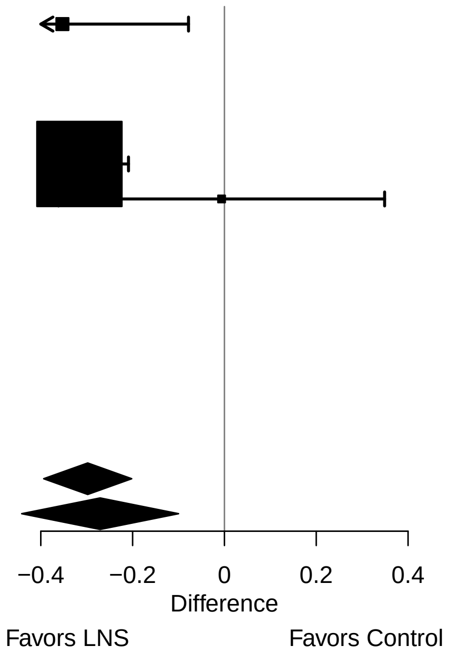

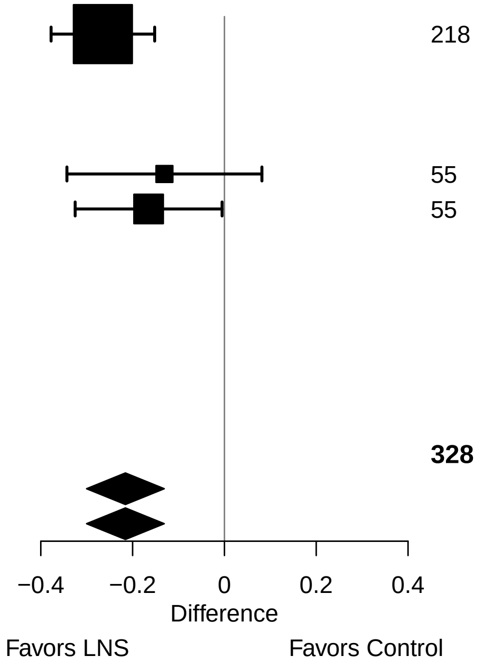

Supplemental figure 9M: Elevated soluble transferrin receptor prevalence difference

9M4: Stratified by Household sanitation (insufficient comparisons)

Supplemental figure 9M: Elevated soluble transferrin receptor prevalence difference

9M5: Stratified by Season at the time of assessment

P−for−interaction = 0.684
Difference in PDs = 0.02 (−0.06, 0.10)

Country	Trial	Dry LNS N	Dry Control N	Dry Control Prevalence	Dry PD (95% CI)	Dry Fixed W	Dry Random W	Rainy LNS N	Rainy Control N	Rainy Control Prevalence	Rainy PD (95% CI)	Rainy Fixed W	Rainy Random W
Bangladesh	JiVitA−4 (35)												
Bangladesh	RDNS (36)	341	166	49.4	−0.19 (−0.27, −0.10)	0.36	0.36	208	106	50.9	−0.09 (−0.20, 0.02)	0.25	0.25
Bangladesh	WASH−B (37)	96	93	31.2	−0.19 (−0.28, −0.09)	0.29	0.29	116	85	31.8	−0.24 (−0.35, −0.13)	0.25	0.25
Burkina Faso	iLiNS−Zinc (38)	174	61	59.2	−0.29 (−0.47, −0.11)	0.08	0.08	126	30	70.0	−0.37 (−0.59, −0.15)	0.07	0.10
Burkina Faso	PROMIS CS (39)												
Ghana	GHANA (40)	48	47	66.0	−0.28 (−0.48, −0.09)	0.07	0.07	36	35	57.1	−0.24 (−0.47, −0.01)	0.06	0.10
Ghana	iLiNS−DYAD−G (41)												
Kenya	WASH−B (42)	88	75	72.0	−0.27 (−0.38, −0.15)	0.19	0.19	210	184	73.4	−0.23 (−0.32, −0.14)	0.37	0.30
Madagascar	MAHAY (43)												
Malawi	iLiNS−DYAD−M (44)												
Malawi	iLiNS−DOSE (45)												
Mali	PROMIS CS (46)												
Zimbabwe	SHINE (HIV−) (47)												
Zimbabwe	SHINE (HIV+) (48)												
		**747**	**442**		**I² = 0.00, Tau² = 0.00**			**696**	**440**		**I² = 0.40, Tau² = 0.00**		
**Fixed**					**−0.22 (−0.27, −0.16)**						**−0.21 (−0.26, −0.15)**		
**Random**					**−0.22 (−0.27, −0.16)**						**−0.21 (−0.29, −0.13)**		

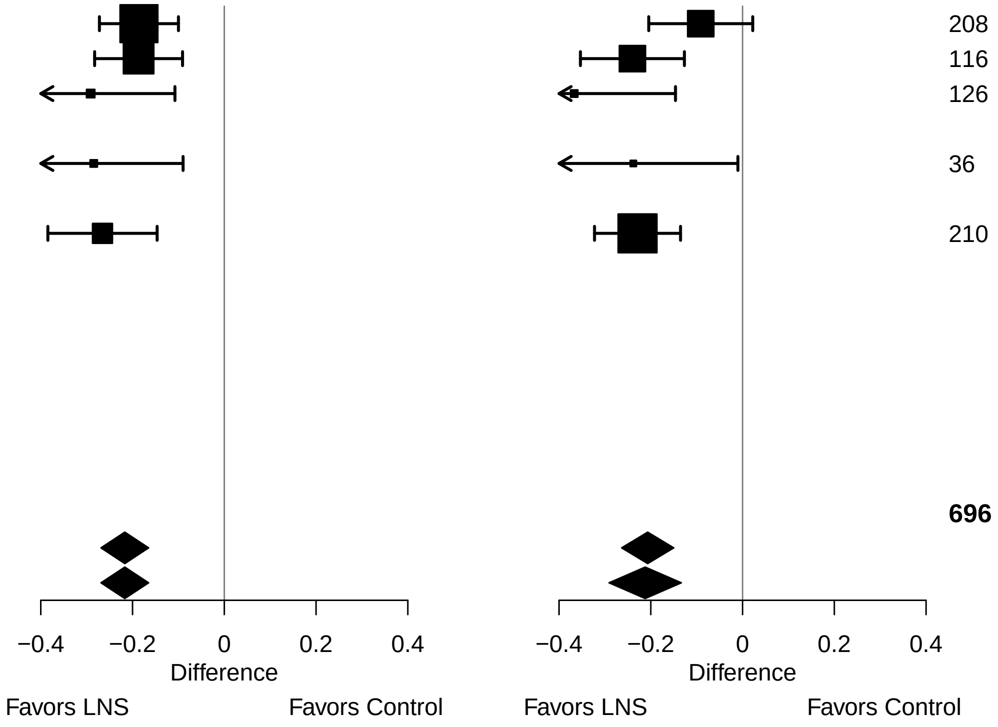

Supplemental figure 9N: Geometric mean ratio of zinc protoporphyrin concentration

9N1: Stratified by Household socio-economic status

P−for−interaction = 0.157
Ratio of GMRs = 1.09 (0.97, 1.23)

		At least median						Less than median					
Country	Trial	LNS N	Control N	Control Median	GMR (95% CI)	Fixed W	Random W	LNS N	Control N	Control Median	GMR (95% CI)	Fixed W	Random W
Bangladesh	JiVitA−4 (35)												
Bangladesh	RDNS (36)												
Bangladesh	WASH−B (37)												
Burkina Faso	iLiNS−Zinc (38)	178	39	104.0	0.70 (0.57, 0.85)	0.19	0.22	134	56	127.1	0.58 (0.49, 0.69)	0.28	0.26
Burkina Faso	PROMIS CS (39)												
Ghana	GHANA (40)												
Ghana	iLiNS−DYAD−G (41)	58	109	47.1	0.81 (0.68, 0.96)	0.26	0.26	42	93	49.3	0.95 (0.78, 1.15)	0.20	0.25
Kenya	WASH−B (42)												
Madagascar	MAHAY (43)												
Malawi	iLiNS−DYAD−M (44)	106	186	56.0	0.83 (0.72, 0.96)	0.37	0.31	84	189	51.7	0.90 (0.78, 1.04)	0.38	0.27
Malawi	iLiNS−DOSE (45)	67	42	70.4	0.65 (0.53, 0.79)	0.18	0.21	85	34	43.5	1.01 (0.80, 1.28)	0.14	0.23
Mali	PROMIS CS (46)												
Zimbabwe	SHINE (HIV−) (47)												
Zimbabwe	SHINE (HIV+) (48)												
		**409**	**376**		**I² = 0.41, Tau² = 0.01**			**345**	**372**		**I² = 0.87, Tau² = 0.05**		
**Fixed**					**0.76 (0.70, 0.83)**						**0.82 (0.75, 0.90)**		
**Random**					**0.75 (0.67, 0.85)**						**0.84 (0.65, 1.07)**		

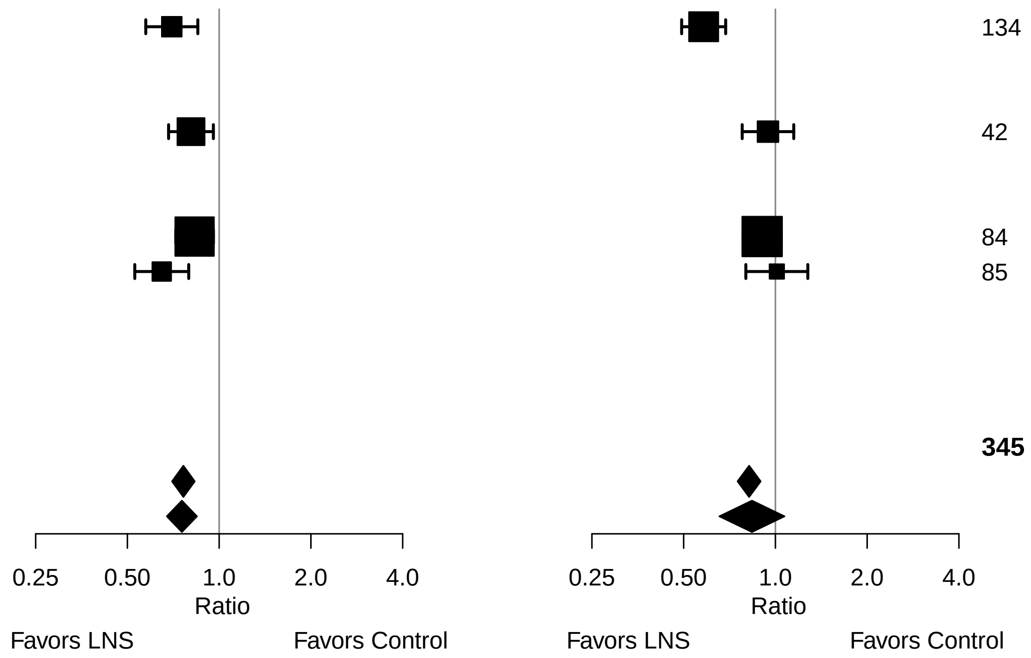

Supplemental figure 9N: Geometric mean ratio of zinc protoporphyrin concentration

9N2: Stratified by Household food insecurity

P−for−interaction = 0.193
Ratio of GMRs = 1.09 (0.96, 1.24)

Country	Trial	**Mild to secure** LNS N	Control N	Control Median	GMR (95% CI)	Fixed W	Random W	**Moderate to severe** LNS N	Control N	Control Median	GMR (95% CI)	Fixed W	Random W
Bangladesh	JiVitA−4 (35)												
Bangladesh	RDNS (36)												
Bangladesh	WASH−B (37)												
Burkina Faso	iLiNS−Zinc (38)	160	44	124.4	0.63 (0.54, 0.73)	0.33	0.30	152	51	116.5	0.66 (0.54, 0.80)	0.18	0.25
Burkina Faso	PROMIS CS (39)												
Ghana	GHANA (40)												
Ghana	iLiNS−DYAD−G (41)	79	146	47.8	0.83 (0.72, 0.95)	0.39	0.32	21	56	48.6	1.01 (0.75, 1.37)	0.08	0.17
Kenya	WASH−B (42)												
Madagascar	MAHAY (43)												
Malawi	iLiNS−DYAD−M (44)	57	100	56.2	0.81 (0.67, 0.98)	0.21	0.25	132	270	52.7	0.88 (0.78, 0.99)	0.50	0.32
Malawi	iLiNS−DOSE (45)	39	24	63.7	0.78 (0.56, 1.08)	0.07	0.13	109	47	65.2	0.83 (0.70, 0.99)	0.24	0.27
Mali	PROMIS CS (46)												
Zimbabwe	SHINE (HIV−) (47)												
Zimbabwe	SHINE (HIV+) (48)												
		**335**	**314**		**I^2 = 0.63, Tau^2 = 0.01**			**414**	**424**		**I^2 = 0.62, Tau^2 = 0.02**		
**Fixed**					**0.75 (0.69, 0.82)**						**0.83 (0.77, 0.91)**		
**Random**					**0.75 (0.66, 0.86)**						**0.83 (0.70, 0.97)**		

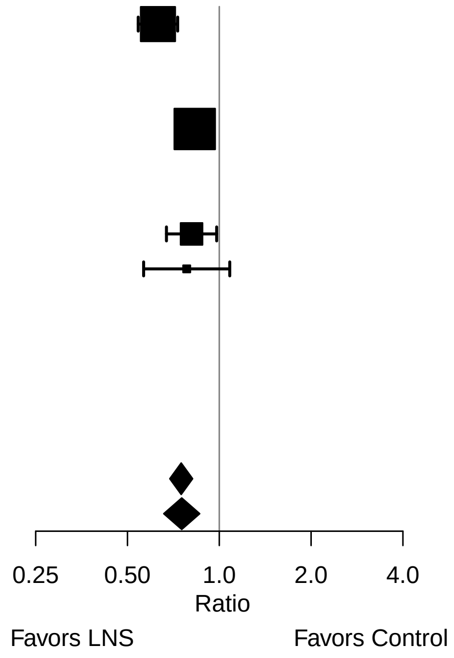

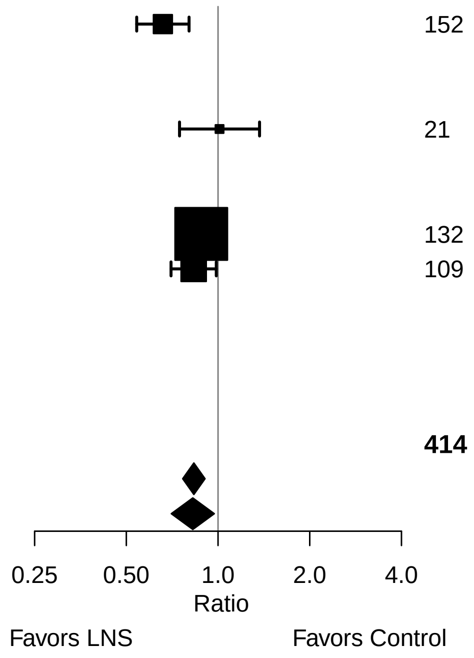

Supplemental figure 9N: Geometric mean ratio of zinc protoporphyrin concentration

9N3: Stratified by Household source water quality (insufficient comparisons)

Supplemental figure 9N: Geometric mean ratio of zinc protoporphyrin concentration

9N4: Stratified by Household sanitation (insufficient comparisons)

Supplemental figure 9N: Geometric mean ratio of zinc protoporphyrin concentration

9N5: Stratified by Season at the time of assessment

P−for−interaction = 0.079
Ratio of GMRs = 0.89 (0.77, 1.01)

Country	Trial	Dry: LNS N	Dry: Control N	Dry: Control Median	Dry: GMR (95% CI)	Dry: Fixed W	Dry: Random W	Rainy: LNS N	Rainy: Control N	Rainy: Control Median	Rainy: GMR (95% CI)	Rainy: Fixed W	Rainy: Random W
Bangladesh	JiVitA−4 (35)												
Bangladesh	RDNS (36)												
Bangladesh	WASH−B (37)												
Burkina Faso	iLiNS−Zinc (38)	173	60	127.5	0.63 (0.52, 0.76)	0.18	0.23	124	30	77.5	0.67 (0.49, 0.90)	0.12	0.16
Burkina Faso	PROMIS CS (39)												
Ghana	GHANA (40)												
Ghana	iLiNS−DYAD−G (41)	59	124	49.4	0.83 (0.70, 0.99)	0.22	0.25	41	77	46.6	0.91 (0.75, 1.11)	0.29	0.28
Kenya	WASH−B (42)												
Madagascar	MAHAY (43)												
Malawi	iLiNS−DYAD−M (44)	132	265	51.6	0.93 (0.83, 1.05)	0.44	0.29	58	109	57.9	0.73 (0.61, 0.88)	0.32	0.29
Malawi	iLiNS−DOSE (45)	103	46	65.6	0.87 (0.72, 1.06)	0.17	0.23	78	41	74.0	0.68 (0.56, 0.83)	0.27	0.27
Mali	PROMIS CS (46)												
Zimbabwe	SHINE (HIV−) (47)												
Zimbabwe	SHINE (HIV+) (48)												
		**467**	**495**		**I^2 = 0.75, Tau^2 = 0.02**			**301**	**257**		**I^2 = 0.46, Tau^2 = 0.01**		
**Fixed**					**0.84 (0.78, 0.91)**						**0.76 (0.68, 0.84)**		
**Random**					**0.82 (0.69, 0.96)**						**0.75 (0.65, 0.87)**		

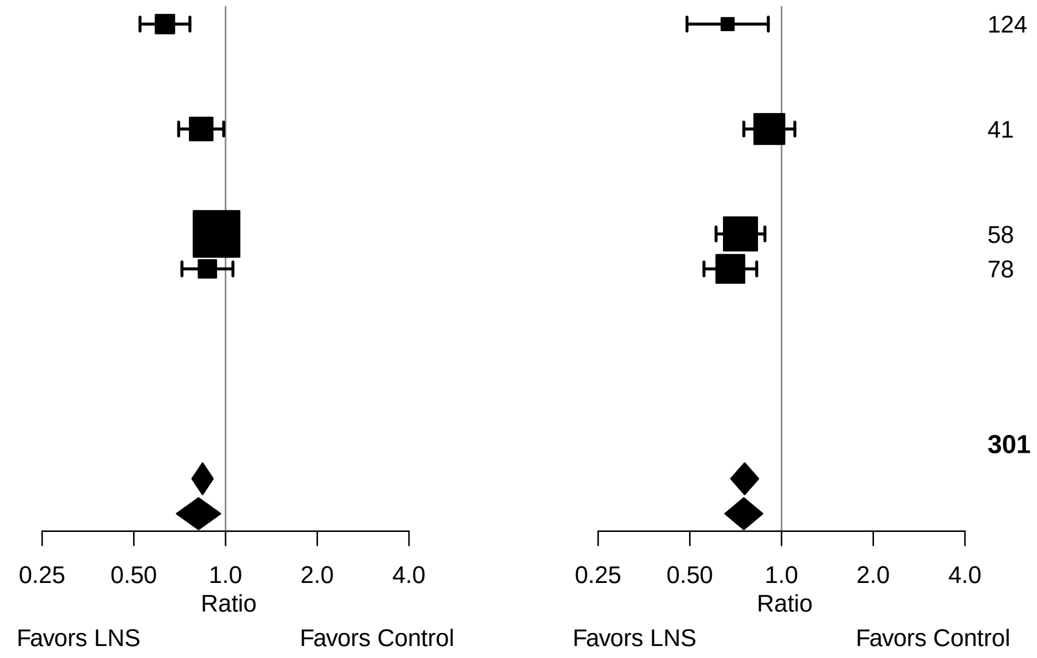

Supplemental figure 9O: Elevated zinc protoporphyrin prevalence ratio

9O1: Stratified by Household socio-economic status

P−for−interaction = 0.894
Ratio of PRs = 0.98 (0.78, 1.24)

		At least median						Less than median					
Country	Trial	LNS N	Control N	Control Prevalence	PR (95% CI)	Fixed W	Random W	LNS N	Control N	Control Prevalence	PR (95% CI)	Fixed W	Random W
Bangladesh	JiVitA−4 (35)												
Bangladesh	RDNS (36)												
Bangladesh	WASH−B (37)												
Burkina Faso	iLiNS−Zinc (38)	178	39	72.1	0.77 (0.61, 0.96)	0.62	0.60	134	56	77.4	0.63 (0.48, 0.82)	0.54	0.54
Burkina Faso	PROMIS CS (39)												
Ghana	GHANA (40)												
Ghana	iLiNS−DYAD−G (41)	58	109	24.8	0.49 (0.23, 1.05)	0.05	0.06	42	93	23.7	1.11 (0.59, 2.07)	0.10	0.10
Kenya	WASH−B (42)												
Madagascar	MAHAY (43)												
Malawi	iLiNS−DYAD−M (44)	106	186	35.5	0.64 (0.43, 0.95)	0.20	0.21	84	189	31.7	0.79 (0.51, 1.20)	0.21	0.22
Malawi	iLiNS−DOSE (45)	67	42	50.0	0.51 (0.31, 0.84)	0.13	0.14	85	34	35.3	0.77 (0.46, 1.30)	0.14	0.15
Mali	PROMIS CS (46)												
Zimbabwe	SHINE (HIV−) (47)												
Zimbabwe	SHINE (HIV+) (48)												
		**409**	**376**		**I² = 0.06, Tau² = 0.00**			**345**	**372**		**I² = 0.02, Tau² = 0.00**		
**Fixed**					**0.69 (0.58, 0.82)**						**0.72 (0.59, 0.87)**		
**Random**					**0.68 (0.56, 0.82)**						**0.72 (0.59, 0.88)**		

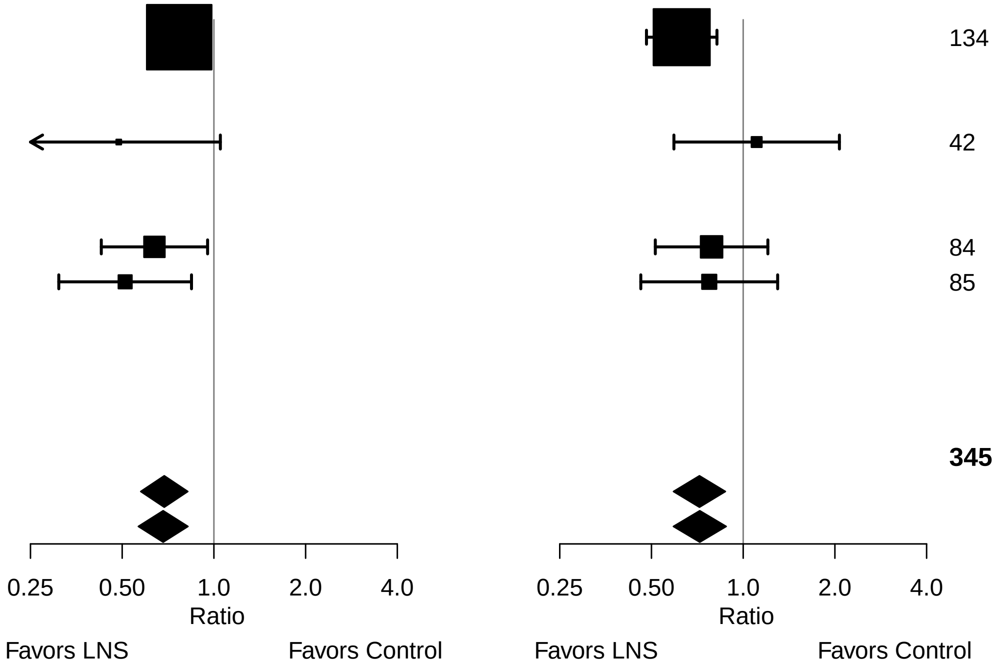

Supplemental figure 9O: Elevated zinc protoporphyrin prevalence ratio

9O2: Stratified by Household food insecurity

P−for−interaction = 0.790
Ratio of PRs = 0.97 (0.76, 1.23)

		Mild to secure						Moderate to severe					
Country	Trial	LNS N	Control N	Control Prevalence	PR (95% CI)	Fixed W	Random W	LNS N	Control N	Control Prevalence	PR (95% CI)	Fixed W	Random W
Bangladesh	JiVitA−4 (35)												
Bangladesh	RDNS (36)												
Bangladesh	WASH−B (37)												
Burkina Faso	iLiNS−Zinc (38)	160	44	76.0	0.72 (0.58, 0.90)	0.67	0.67	152	51	74.5	0.70 (0.55, 0.88)	0.55	0.55
Burkina Faso	PROMIS CS (39)												
Ghana	GHANA (40)												
Ghana	iLiNS−DYAD−G (41)	79	146	20.5	0.74 (0.40, 1.36)	0.09	0.09	21	56	33.9	0.84 (0.39, 1.82)	0.05	0.05
Kenya	WASH−B (42)												
Madagascar	MAHAY (43)												
Malawi	iLiNS−DYAD−M (44)	57	100	38.0	0.69 (0.42, 1.14)	0.13	0.13	132	270	32.2	0.68 (0.47, 0.98)	0.23	0.23
Malawi	iLiNS−DOSE (45)	39	24	45.8	0.67 (0.39, 1.16)	0.11	0.11	109	47	42.6	0.59 (0.39, 0.91)	0.17	0.17
Mali	PROMIS CS (46)												
Zimbabwe	SHINE (HIV−) (47)												
Zimbabwe	SHINE (HIV+) (48)												
		**335**	**314**		**I² = 0.00, Tau² = 0.00**			**414**	**424**		**I² = 0.00, Tau² = 0.00**		
**Fixed**					**0.71 (0.59, 0.85)**						**0.68 (0.57, 0.81)**		
**Random**					**0.71 (0.59, 0.85)**						**0.68 (0.57, 0.81)**		

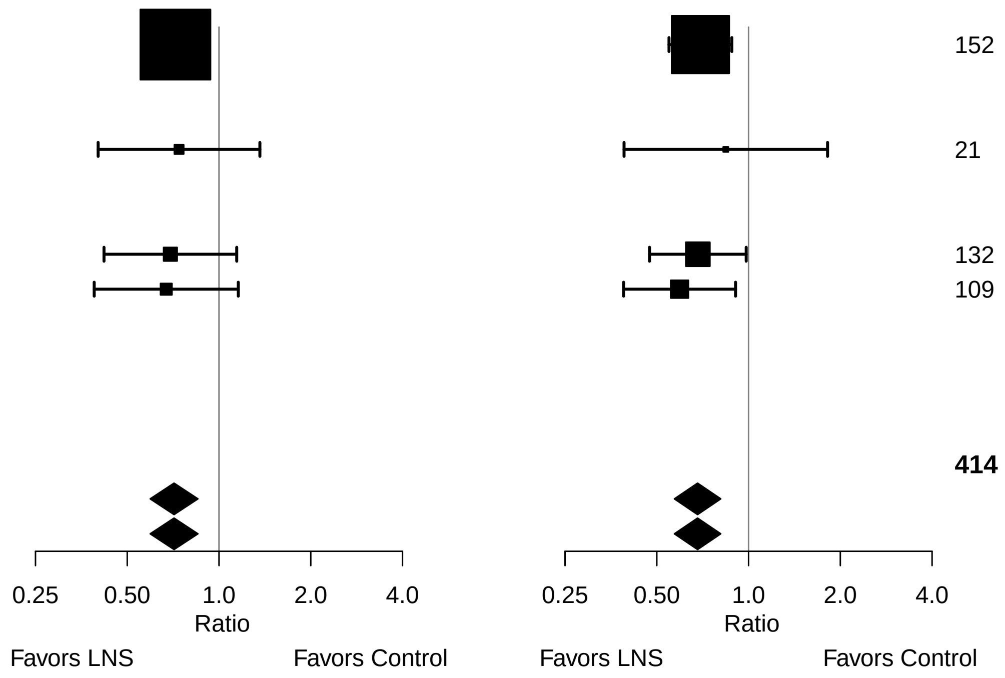

Supplemental figure 9O: Elevated zinc protoporphyrin prevalence ratio

9O3: Stratified by Household source water quality (insufficient comparisons)

Supplemental figure 9O: Elevated zinc protoporphyrin prevalence ratio

9O4: Stratified by Household sanitation (insufficient comparisons)

Supplemental figure 9O: Elevated zinc protoporphyrin prevalence ratio

9O5: Stratified by Season at the time of assessment

P−for−interaction = 0.680
Ratio of PRs = 0.94 (0.69, 1.28)

Country	Trial	Dry: LNS N	Dry: Control N	Dry: Control Prevalence	Dry: PR (95% CI)	Dry: Fixed W	Dry: Random W	Rainy: LNS N	Rainy: Control N	Rainy: Control Prevalence	Rainy: PR (95% CI)	Rainy: Fixed W	Rainy: Random W
Bangladesh	JiVitA−4 (35)												
Bangladesh	RDNS (36)												
Bangladesh	WASH−B (37)												
Burkina Faso	iLiNS−Zinc (38)	173	60	82.9	0.72 (0.58, 0.88)	0.60	0.60	124	30	63.3	0.75 (0.48, 1.15)	0.33	0.29
Burkina Faso	PROMIS CS (39)												
Ghana	GHANA (40)												
Ghana	iLiNS−DYAD−G (41)	59	124	25.0	0.41 (0.18, 0.92)	0.04	0.04	41	77	23.4	1.25 (0.67, 2.34)	0.16	0.20
Kenya	WASH−B (42)												
Madagascar	MAHAY (43)												
Malawi	iLiNS−DYAD−M (44)	132	265	33.2	0.75 (0.54, 1.06)	0.22	0.22	58	109	33.9	0.61 (0.35, 1.08)	0.19	0.23
Malawi	iLiNS−DOSE (45)	103	46	39.1	0.78 (0.51, 1.19)	0.14	0.14	78	41	51.2	0.49 (0.31, 0.76)	0.31	0.28
Mali	PROMIS CS (46)												
Zimbabwe	SHINE (HIV−) (47)												
Zimbabwe	SHINE (HIV+) (48)												
		**467**	**495**		**I² = 0.00, Tau² = 0.00**			**301**	**257**		**I² = 0.51, Tau² = 0.08**		
**Fixed**					**0.72 (0.61, 0.84)**						**0.68 (0.53, 0.87)**		
**Random**					**0.72 (0.61, 0.84)**						**0.70 (0.48, 1.03)**		

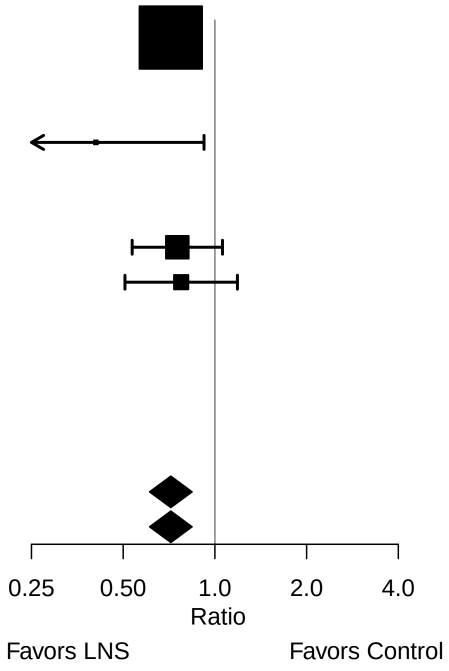

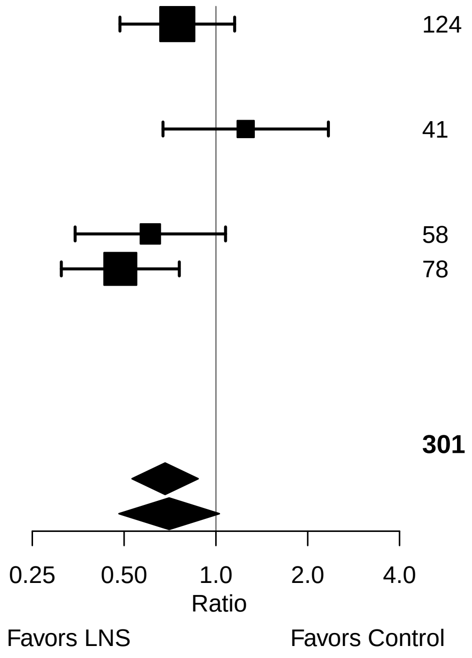

Supplemental figure 9P: Elevated zinc protoporphyrin prevalence difference

9P1: Stratified by Household socio-economic status

P−for−interaction = 0.191
Difference in PDs = 0.06 (−0.03, 0.16)

Country	Trial	LNS N (At least median)	Control N (At least median)	Control Prevalence (At least median)	At least median PD (95% CI)	Fixed W (At least median)	Random W (At least median)	LNS N (Less than median)	Control N (Less than median)	Control Prevalence (Less than median)	Less than median PD (95% CI)	Fixed W (Less than median)	Random W (Less than median)
Bangladesh	JiVitA−4 (35)												
Bangladesh	RDNS (36)												
Bangladesh	WASH−B (37)												
Burkina Faso	iLiNS−Zinc (38)	178	39	72.1	−0.17 (−0.33, −0.02)	0.18	0.18	134	56	77.4	−0.28 (−0.42, −0.14)	0.26	0.26
Burkina Faso	PROMIS CS (39)												
Ghana	GHANA (40)												
Ghana	iLiNS−DYAD−G (41)	58	109	24.8	−0.13 (−0.25, 0.00)	0.28	0.28	42	93	23.7	0.03 (−0.13, 0.18)	0.20	0.24
Kenya	WASH−B (42)												
Madagascar	MAHAY (43)												
Malawi	iLiNS−DYAD−M (44)	106	186	35.5	−0.13 (−0.24, −0.02)	0.38	0.38	84	189	31.7	−0.07 (−0.19, 0.05)	0.37	0.28
Malawi	iLiNS−DOSE (45)	67	42	50.0	−0.25 (−0.42, −0.09)	0.17	0.17	85	34	35.3	−0.08 (−0.25, 0.09)	0.17	0.22
Mali	PROMIS CS (46)												
Zimbabwe	SHINE (HIV−) (47)												
Zimbabwe	SHINE (HIV+) (48)												
		**409**	**376**		**I² = 0.00, Tau² = 0.00**			**345**	**372**		**I² = 0.66, Tau² = 0.01**		
**Fixed**					**−0.16 (−0.22, −0.09)**						**−0.10 (−0.18, −0.03)**		
**Random**					**−0.16 (−0.22, −0.09)**						**−0.10 (−0.23, 0.02)**		

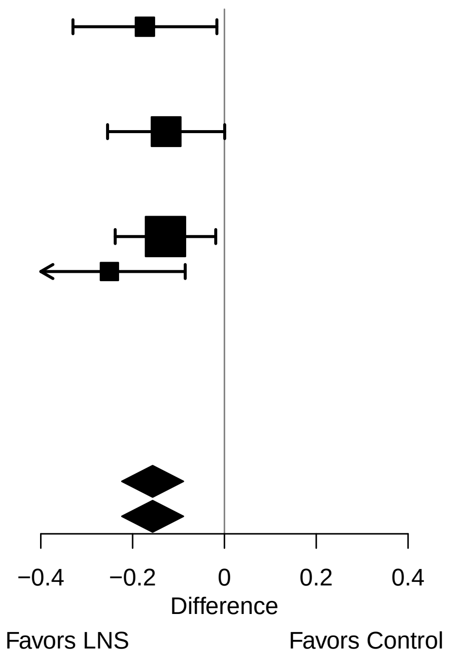

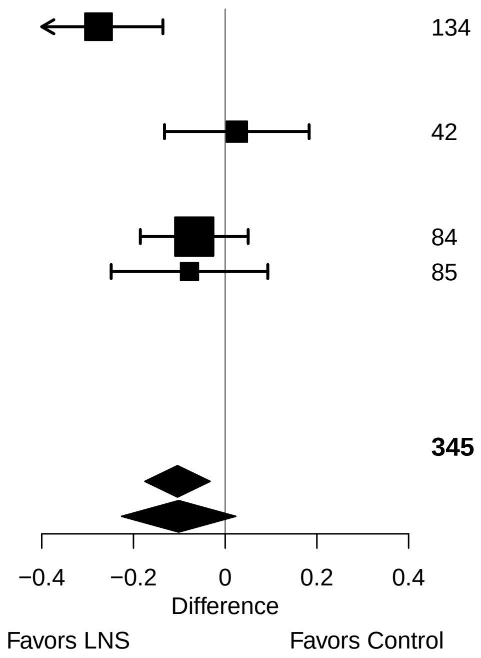

Supplemental figure 9P: Elevated zinc protoporphyrin prevalence difference

9P2: Stratified by Household food insecurity

P−for−interaction = 0.945
Difference in PDs = 0.00 (−0.11, 0.10)

Country	Trial	Mild to secure: LNS N	Control N	Control Prevalence	PD (95% CI)	Fixed W	Random W	Moderate to severe: LNS N	Control N	Control Prevalence	PD (95% CI)	Fixed W	Random W
Bangladesh	JiVitA−4 (35)												
Bangladesh	RDNS (36)												
Bangladesh	WASH−B (37)												
Burkina Faso	iLiNS−Zinc (38)	160	44	76.0	−0.21 (−0.35, −0.07)	0.26	0.26	152	51	74.5	−0.23 (−0.38, −0.08)	0.19	0.19
Burkina Faso	PROMIS CS (39)												
Ghana	GHANA (40)												
Ghana	iLiNS−DYAD−G (41)	79	146	20.5	−0.05 (−0.16, 0.05)	0.44	0.43	21	56	33.9	−0.05 (−0.29, 0.18)	0.08	0.08
Kenya	WASH−B (42)												
Madagascar	MAHAY (43)												
Malawi	iLiNS−DYAD−M (44)	57	100	38.0	−0.12 (−0.27, 0.04)	0.21	0.22	132	270	32.2	−0.10 (−0.20, −0.01)	0.50	0.50
Malawi	iLiNS−DOSE (45)	39	24	45.8	−0.15 (−0.38, 0.08)	0.09	0.10	109	47	42.6	−0.18 (−0.32, −0.04)	0.23	0.23
Mali	PROMIS CS (46)												
Zimbabwe	SHINE (HIV−) (47)												
Zimbabwe	SHINE (HIV+) (48)												
		**335**	**314**		**I² = 0.06, Tau² = 0.00**			**414**	**424**		**I² = 0.00, Tau² = 0.00**		
**Fixed**					**−0.12 (−0.19, −0.05)**						**−0.14 (−0.21, −0.07)**		
**Random**					**−0.12 (−0.19, −0.05)**						**−0.14 (−0.21, −0.07)**		

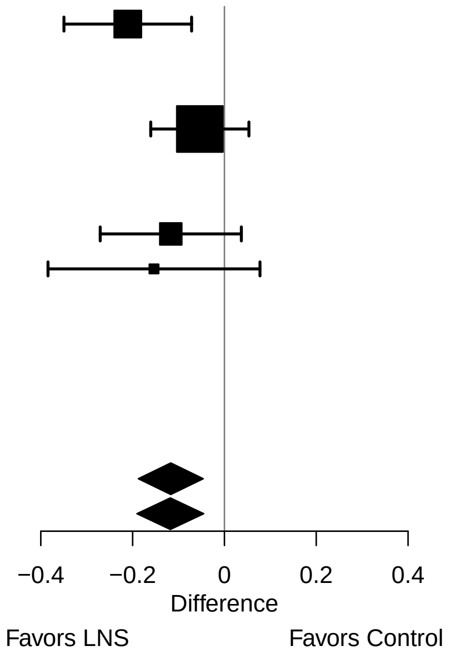

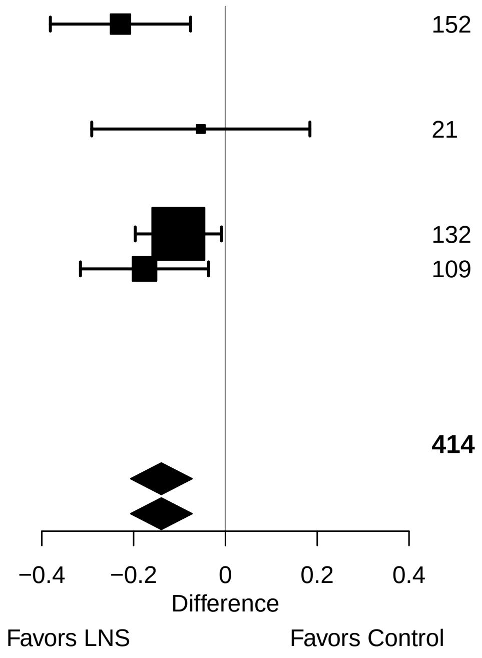

Supplemental figure 9P: Elevated zinc protoporphyrin prevalence difference

9P3: Stratified by Household source water quality (insufficient comparisons)

Supplemental figure 9P: Elevated zinc protoporphyrin prevalence difference

9P4: Stratified by Household sanitation (insufficient comparisons)

Supplemental figure 9P: Elevated zinc protoporphyrin prevalence difference

9P5: Stratified by Season at the time of assessment

P−for−interaction = 0.874
Difference in PDs = 0.01 (−0.09, 0.11)

Country	Trial	Dry LNS N	Dry Control N	Dry Control Prevalence	Dry PD (95% CI)	Dry Fixed W	Dry Random W	Rainy LNS N	Rainy Control N	Rainy Control Prevalence	Rainy PD (95% CI)	Rainy Fixed W	Rainy Random W
Bangladesh	JiVitA−4 (35)												
Bangladesh	RDNS (36)												
Bangladesh	WASH−B (37)												
Burkina Faso	iLiNS−Zinc (38)	173	60	82.9	−0.23 (−0.38, −0.09)	0.18	0.20	124	30	63.3	−0.17 (−0.41, 0.07)	0.12	0.18
Burkina Faso	PROMIS CS (39)												
Ghana	GHANA (40)												
Ghana	iLiNS−DYAD−G (41)	59	124	25.0	−0.15 (−0.27, −0.02)	0.25	0.25	41	77	23.4	0.06 (−0.11, 0.22)	0.25	0.26
Kenya	WASH−B (42)												
Madagascar	MAHAY (43)												
Malawi	iLiNS−DYAD−M (44)	132	265	33.2	−0.08 (−0.18, 0.01)	0.40	0.38	58	109	33.9	−0.13 (−0.28, 0.01)	0.34	0.29
Malawi	iLiNS−DOSE (45)	103	46	39.1	−0.08 (−0.23, 0.07)	0.17	0.18	78	41	51.2	−0.26 (−0.42, −0.11)	0.29	0.27
Mali	PROMIS CS (46)												
Zimbabwe	SHINE (HIV−) (47)												
Zimbabwe	SHINE (HIV+) (48)												
		**467**	**495**		**I^2 = 0.14, Tau^2 = 0.00**			**301**	**257**		**I^2 = 0.62, Tau^2 = 0.01**		
**Fixed**					**−0.13 (−0.19, −0.07)**						**−0.13 (−0.21, −0.04)**		
**Random**					**−0.13 (−0.20, −0.06)**						**−0.13 (−0.26, 0.01)**		

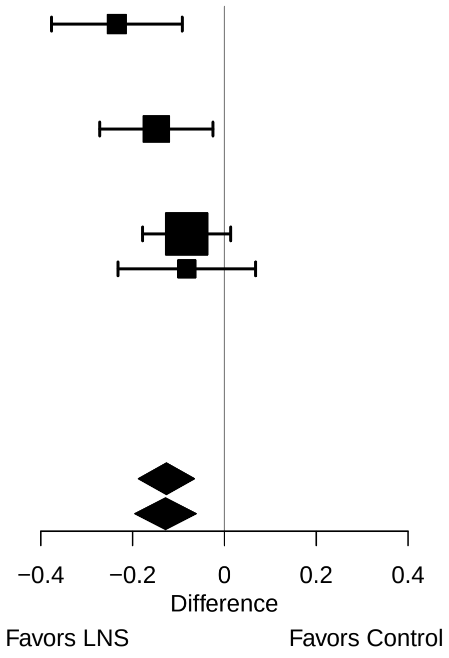

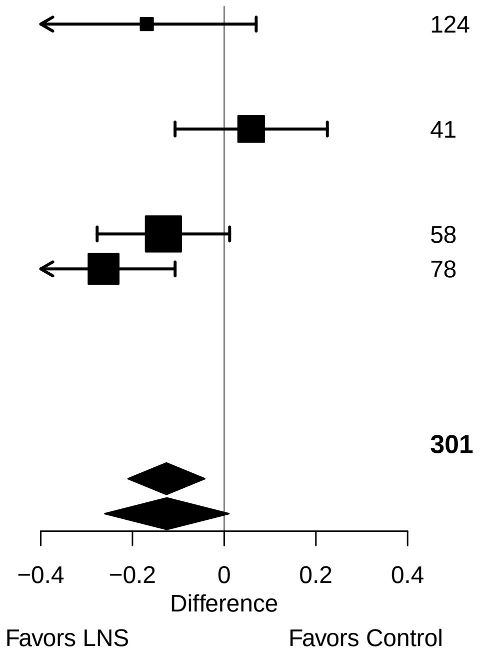

Supplemental figure 9Q: Geometric mean ratio of plasma zinc concentration

9Q1: Stratified by Household socio-economic status

P−for−interaction = 0.696
Ratio of GMRs = 0.99 (0.94, 1.04)

		At least median						Less than median					
Country	Trial	LNS N	Control N	Control Median	GMR (95% CI)	Fixed W	Random W	LNS N	Control N	Control Median	GMR (95% CI)	Fixed W	Random W
Bangladesh	JiVitA−4 (35)	242	78	88.3	0.98 (0.92, 1.05)	0.27	0.27	209	66	88.7	0.94 (0.87, 1.03)	0.20	0.20
Bangladesh	RDNS (36)												
Bangladesh	WASH−B (37)												
Burkina Faso	iLiNS−Zinc (38)	180	39	67.8	1.01 (0.96, 1.06)	0.55	0.55	134	57	68.8	1.00 (0.96, 1.05)	0.69	0.69
Burkina Faso	PROMIS CS (39)												
Ghana	GHANA (40)	34	34	61.0	0.99 (0.91, 1.08)	0.18	0.18	24	31	58.9	1.00 (0.89, 1.12)	0.11	0.11
Ghana	iLiNS−DYAD−G (41)												
Kenya	WASH−B (42)												
Madagascar	MAHAY (43)												
Malawi	iLiNS−DYAD−M (44)												
Malawi	iLiNS−DOSE (45)												
Mali	PROMIS CS (46)												
Zimbabwe	SHINE (HIV−) (47)												
Zimbabwe	SHINE (HIV+) (48)												
		**456**	**151**		**I² = 0.00, Tau² = 0.00**			**367**	**154**		**I² = 0.00, Tau² = 0.00**		
**Fixed**					**1.00 (0.96, 1.03)**						**0.99 (0.95, 1.03)**		
**Random**					**1.00 (0.96, 1.03)**						**0.99 (0.95, 1.03)**		

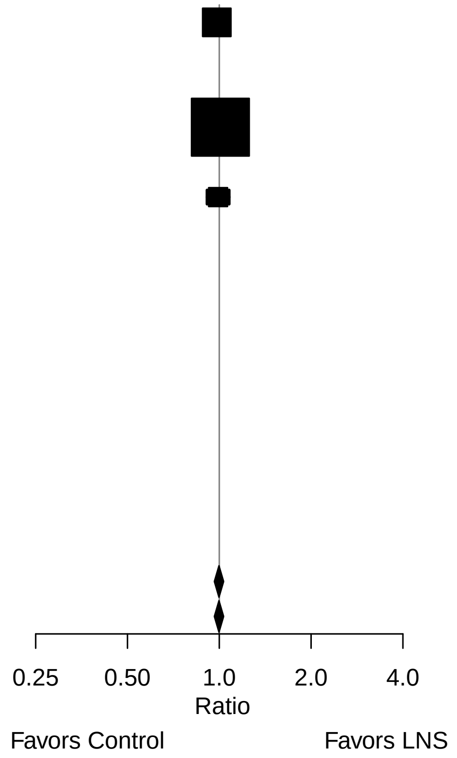

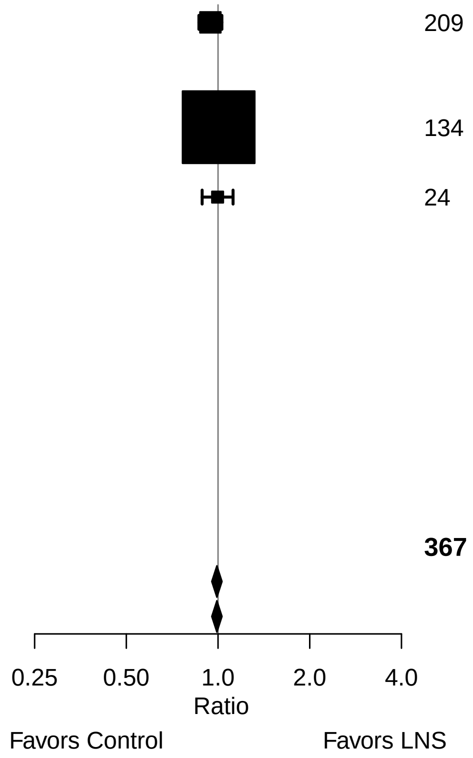

Supplemental figure 9Q: Geometric mean ratio of plasma zinc concentration

9Q2: Stratified by Household food insecurity (insufficient comparisons)

Supplemental figure 9Q: Geometric mean ratio of plasma zinc concentration

9Q3: Stratified by Household source water quality (insufficient comparisons)

Supplemental figure 9Q: Geometric mean ratio of plasma zinc concentration

9Q4: Stratified by Household sanitation (insufficient comparisons)

Supplemental figure 9Q: Geometric mean ratio of plasma zinc concentration

9Q5: Stratified by Season at the time of assessment

P−for−interaction = 0.044
Ratio of GMRs = 1.04 (1.00, 1.08)

Country	Trial	Dry: LNS N	Dry: Control N	Dry: Control Median	Dry: GMR (95% CI)	Dry: Fixed W	Dry: Random W	Rainy: LNS N	Rainy: Control N	Rainy: Control Median	Rainy: GMR (95% CI)	Rainy: Fixed W	Rainy: Random W
Bangladesh	JiVitA−4 (35)	261	71	88.0	0.98 (0.93, 1.04)	0.30	0.30	191	73	88.9	0.95 (0.87, 1.03)	0.19	0.29
Bangladesh	RDNS (36)												
Bangladesh	WASH−B (37)												
Burkina Faso	iLiNS−Zinc (38)	174	61	68.7	0.99 (0.95, 1.03)	0.61	0.61	125	30	67.3	1.05 (1.00, 1.10)	0.64	0.45
Burkina Faso	PROMIS CS (39)												
Ghana	GHANA (40)	30	36	59.7	1.03 (0.93, 1.14)	0.08	0.08	28	33	60.0	0.98 (0.90, 1.07)	0.17	0.27
Ghana	iLiNS−DYAD−G (41)												
Kenya	WASH−B (42)												
Madagascar	MAHAY (43)												
Malawi	iLiNS−DYAD−M (44)												
Malawi	iLiNS−DOSE (45)												
Mali	PROMIS CS (46)												
Zimbabwe	SHINE (HIV−) (47)												
Zimbabwe	SHINE (HIV+) (48)												
		**465**	**168**		**I^2 = 0.00, Tau^2 = 0.00**			**344**	**136**		**I^2 = 0.62, Tau^2 = 0.00**		
**Fixed**					**0.99 (0.96, 1.02)**						**1.02 (0.98, 1.06)**		
**Random**					**0.99 (0.96, 1.02)**						**1.00 (0.94, 1.07)**		

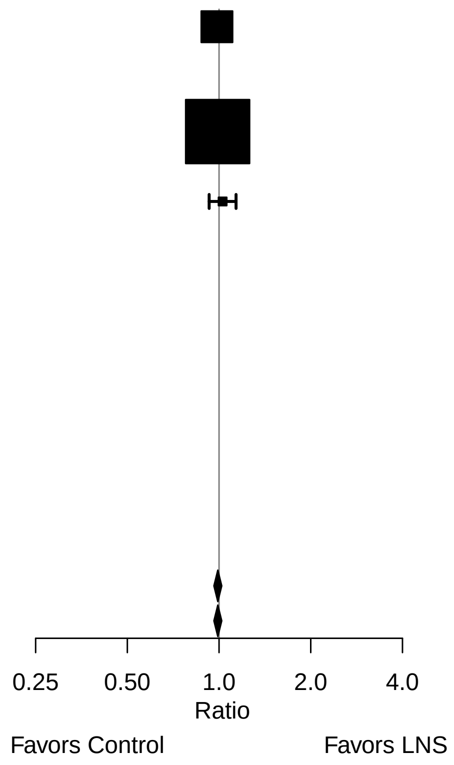

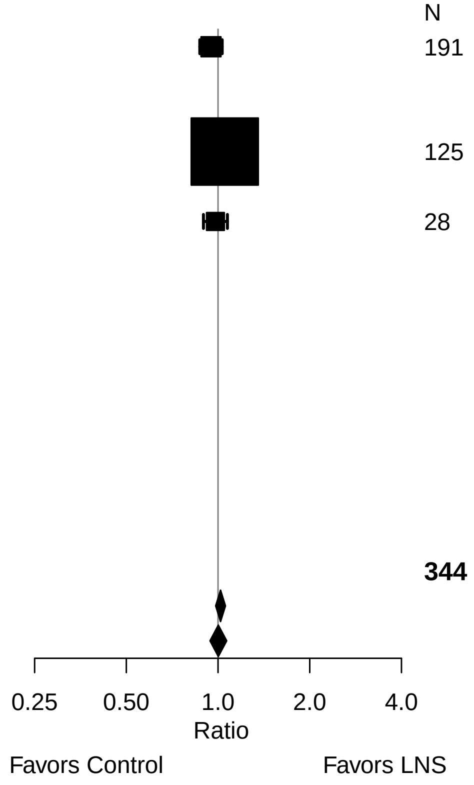

Supplemental figure 9R: Geometric mean ratio of retinol concentration

9R1: Stratified by Household socio-economic status

P−for−interaction = 0.882
Ratio of GMRs = 1.01 (0.94, 1.08)

Country	Trial	At least median: LNS N	Control N	Control Median	GMR (95% CI)	Fixed W	Random W	Less than median: LNS N	Control N	Control Median	GMR (95% CI)	Fixed W	Random W
Bangladesh	JiVitA−4 (35)	232	78	1.4	1.07 (1.00, 1.14)	0.50	0.50	201	61	1.3	1.10 (1.00, 1.21)	0.36	0.34
Bangladesh	RDNS (36)												
Bangladesh	WASH−B (37)												
Burkina Faso	iLiNS−Zinc (38)												
Burkina Faso	PROMIS CS (39)												
Ghana	GHANA (40)	38	37	1.1	1.08 (0.90, 1.29)	0.07	0.07	25	33	1.0	1.04 (0.83, 1.29)	0.07	0.08
Ghana	iLiNS−DYAD−G (41)	56	106	1.0	1.04 (0.96, 1.13)	0.31	0.31	40	90	1.0	0.96 (0.87, 1.06)	0.32	0.31
Kenya	WASH−B (42)												
Madagascar	MAHAY (43)												
Malawi	iLiNS−DYAD−M (44)	36	73	1.0	0.97 (0.85, 1.11)	0.13	0.13	35	90	1.1	1.05 (0.94, 1.17)	0.26	0.26
Malawi	iLiNS−DOSE (45)												
Mali	PROMIS CS (46)												
Zimbabwe	SHINE (HIV−) (47)												
Zimbabwe	SHINE (HIV+) (48)												
		**362**	**294**		**I^2 = 0.00, Tau^2 = 0.00**			**301**	**274**		**I^2 = 0.22, Tau^2 = 0.00**		
**Fixed**					**1.05 (1.00, 1.10)**						**1.04 (0.98, 1.10)**		
**Random**					**1.05 (1.00, 1.10)**						**1.04 (0.97, 1.10)**		

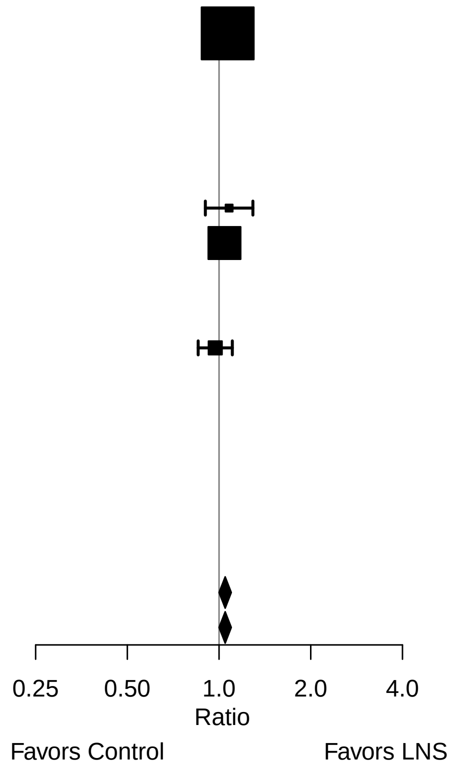

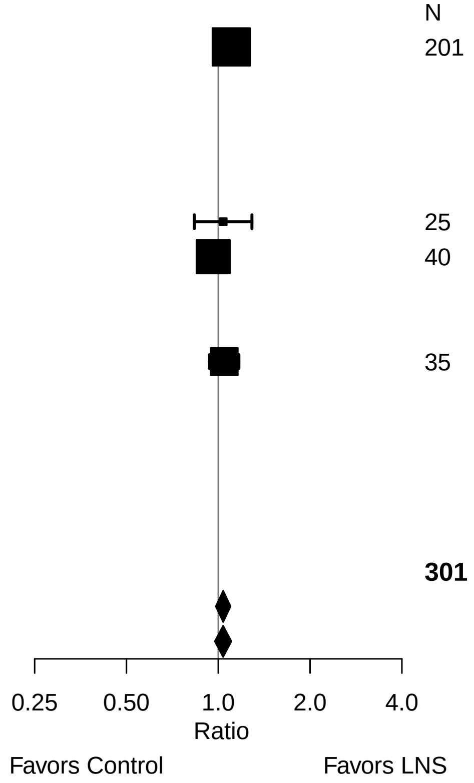

Supplemental figure 9R: Geometric mean ratio of retinol concentration

9R2: Stratified by Household food insecurity

P−for−interaction = 0.084
Ratio of GMRs = 0.93 (0.85, 1.01)

		Mild to secure						Moderate to severe					
		**LNS**	**Control**	**Control**	**GMR**	**Fixed**	**Random**	**LNS**	**Control**	**Control**	**GMR**	**Fixed**	**Random**
Country	Trial	N	N	Median	(95% CI)	W	W	N	N	Median	(95% CI)	W	W
Bangladesh	JiVitA−4 (35)	320	106	1.3	1.12 (1.04, 1.20)	0.46	0.44	113	33	1.4	0.98 (0.88, 1.10)	0.32	0.32
Bangladesh	RDNS (36)												
Bangladesh	WASH−B (37)												
Burkina Faso	iLiNS−Zinc (38)												
Burkina Faso	PROMIS CS (39)												
Ghana	GHANA (40)												
Ghana	iLiNS−DYAD−G (41)	75	144	1.0	1.01 (0.94, 1.09)	0.46	0.44	21	52	1.0	1.00 (0.88, 1.13)	0.26	0.26
Kenya	WASH−B (42)												
Madagascar	MAHAY (43)												
Malawi	iLiNS−DYAD−M (44)	19	34	1.1	1.06 (0.88, 1.28)	0.08	0.11	51	127	1.0	0.98 (0.89, 1.08)	0.42	0.42
Malawi	iLiNS−DOSE (45)												
Mali	PROMIS CS (46)												
Zimbabwe	SHINE (HIV−) (47)												
Zimbabwe	SHINE (HIV+) (48)												
		**414**	**284**		**I² = 0.43, Tau² = 0.00**			**185**	**212**		**I² = 0.00, Tau² = 0.00**		
**Fixed**					**1.06 (1.01, 1.12)**						**0.99 (0.93, 1.05)**		
**Random**					**1.06 (0.99, 1.14)**						**0.99 (0.93, 1.05)**		

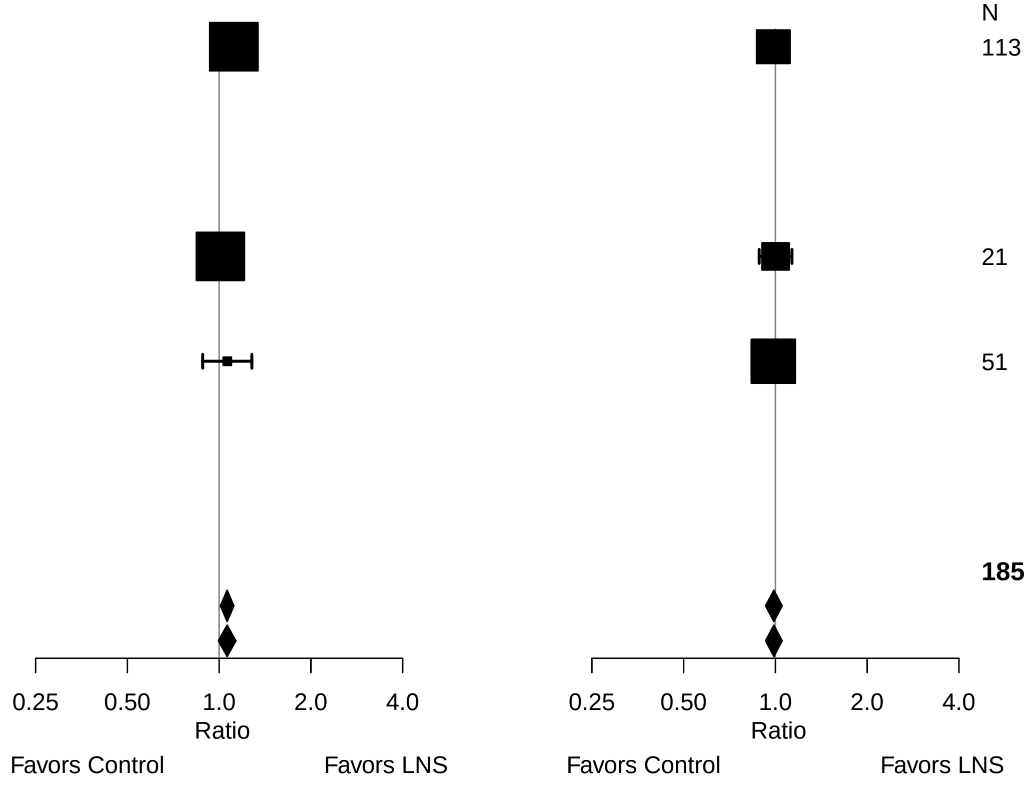

Supplemental figure 9R: Geometric mean ratio of retinol concentration

9R3: Stratified by Household source water quality (insufficient comparisons)

Supplemental figure 9R: Geometric mean ratio of retinol concentration

9R4: Stratified by Household sanitation (insufficient comparisons)

Supplemental figure 9R: Geometric mean ratio of retinol concentration

9R5: Stratified by Season at the time of assessment

P−for−interaction = 0.952
Ratio of GMRs = 1.00 (0.93, 1.08)

Country	Trial	Dry: LNS N	Dry: Control N	Dry: Control Median	Dry: GMR (95% CI)	Dry: Fixed W	Dry: Random W	Rainy: LNS N	Rainy: Control N	Rainy: Control Median	Rainy: GMR (95% CI)	Rainy: Fixed W	Rainy: Random W
Bangladesh	JiVitA−4 (35)	257	69	1.4	1.10 (1.00, 1.21)	0.30	0.28	177	70	1.3	1.04 (0.96, 1.13)	0.48	0.30
Bangladesh	RDNS (36)												
Bangladesh	WASH−B (37)												
Burkina Faso	iLiNS−Zinc (38)												
Burkina Faso	PROMIS CS (39)												
Ghana	GHANA (40)	35	41	0.9	1.15 (0.97, 1.36)	0.09	0.16	28	33	1.4	0.99 (0.84, 1.16)	0.11	0.21
Ghana	iLiNS−DYAD−G (41)	59	122	1.0	1.02 (0.94, 1.11)	0.35	0.29	37	73	1.0	0.99 (0.90, 1.09)	0.30	0.28
Kenya	WASH−B (42)												
Madagascar	MAHAY (43)												
Malawi	iLiNS−DYAD−M (44)	54	120	1.1	0.92 (0.84, 1.02)	0.27	0.27	17	43	1.0	1.30 (1.10, 1.52)	0.11	0.21
Malawi	iLiNS−DOSE (45)												
Mali	PROMIS CS (46)												
Zimbabwe	SHINE (HIV−) (47)												
Zimbabwe	SHINE (HIV+) (48)												
		**405**	**352**		**I² = 0.64, Tau² = 0.01**			**259**	**219**		**I² = 0.65, Tau² = 0.01**		
**Fixed**					**1.03 (0.98, 1.08)**						**1.04 (0.99, 1.10)**		
**Random**					**1.03 (0.95, 1.13)**						**1.06 (0.94, 1.20)**		

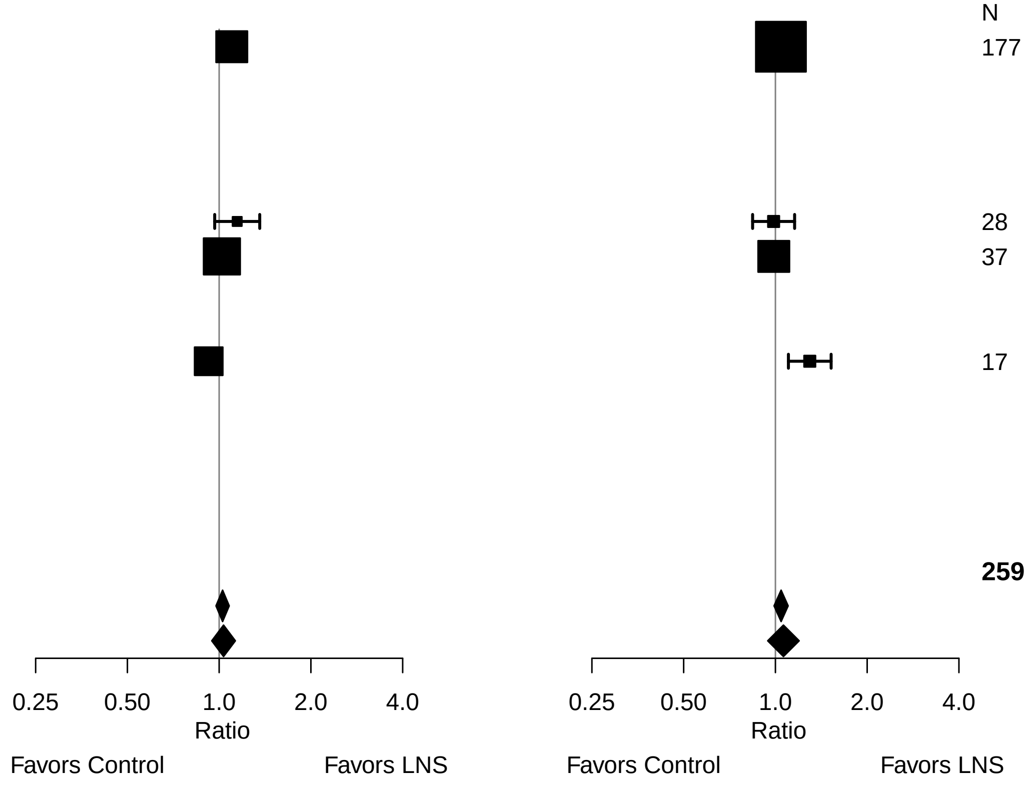

Supplemental figure 9S: Low vitamin A (retinol < 0.70 µmol/L) prevalence ratio

9S1: Stratified by Household socio-economic status (insufficient comparisons)

Supplemental figure 9S: Low vitamin A (retinol < 0.70 µmol/L) prevalence ratio

9S2: Stratified by Household food insecurity (insufficient comparisons)

Supplemental figure 9S: Low vitamin A (retinol < 0.70 µmol/L) prevalence ratio

9S3: Stratified by Household source water quality (insufficient comparisons)

Supplemental figure 9S: Low vitamin A (retinol < 0.70 µmol/L) prevalence ratio

9S4: Stratified by Household sanitation (insufficient comparisons)

Supplemental figure 9S: Low vitamin A (retinol < 0.70 µmol/L) prevalence ratio

9S5: Stratified by Season at the time of assessment (insufficient comparisons)

Supplemental figure 9T: Low vitamin A (retinol $<$ 0.70 µmol/L) prevalence difference

9T1: Stratified by Household socio-economic status (insufficient comparisons)

Supplemental figure 9T: Low vitamin A (retinol < 0.70 µmol/L) prevalence difference

9T2: Stratified by Household food insecurity (insufficient comparisons)

Supplemental figure 9T: Low vitamin A (retinol < 0.70 µmol/L) prevalence difference

9T3: Stratified by Household source water quality (insufficient comparisons)

Supplemental figure 9T: Low vitamin A (retinol < 0.70 µmol/L) prevalence difference

9T4: Stratified by Household sanitation (insufficient comparisons)

Supplemental figure 9T: Low vitamin A (retinol < 0.70 µmol/L) prevalence difference

9T5: Stratified by Season at the time of assessment (insufficient comparisons)

Supplemental figure 9U: Marginal vitamin A (retinol < 1.05 µmol/L) prevalence ratio

9U1: Stratified by Household socio-economic status

P−for−interaction = 0.579
Ratio of PRs = 1.08 (0.82, 1.44)

		At least median							Less than median						
Country	Trial	LNS N	Control N	Control Prevalence	PR (95% CI)	Fixed W	Random W		LNS N	Control N	Control Prevalence	PR (95% CI)	Fixed W	Random W	
Bangladesh	JiVitA−4 (35)	232	78	17.9	0.67 (0.42, 1.09)	0.16	0.19		201	61	21.3	0.79 (0.43, 1.48)	0.11	0.11	
Bangladesh	RDNS (36)														
Bangladesh	WASH−B (37)														
Burkina Faso	iLiNS−Zinc (38)														
Burkina Faso	PROMIS CS (39)														
Ghana	GHANA (40)	38	37	45.9	1.03 (0.64, 1.67)	0.16	0.19		25	33	51.5	1.01 (0.61, 1.67)	0.17	0.17	
Ghana	iLiNS−DYAD−G (41)	56	106	55.7	0.83 (0.60, 1.16)	0.34	0.31		40	90	57.8	1.04 (0.76, 1.41)	0.45	0.45	
Kenya	WASH−B (42)														
Madagascar	MAHAY (43)														
Malawi	iLiNS−DYAD−M (44)	36	73	52.1	1.23 (0.88, 1.71)	0.34	0.31		35	90	45.6	1.13 (0.76, 1.67)	0.27	0.27	
Malawi	iLiNS−DOSE (45)														
Mali	PROMIS CS (46)														
Zimbabwe	SHINE (HIV−) (47)														
Zimbabwe	SHINE (HIV+) (48)														
		**362**	**294**		**I² = 0.40, Tau² = 0.03**				**301**	**274**		**I² = 0.00, Tau² = 0.00**			
**Fixed**					**0.95 (0.78, 1.15)**							**1.03 (0.84, 1.26)**			
**Random**					**0.94 (0.73, 1.21)**							**1.03 (0.84, 1.26)**			

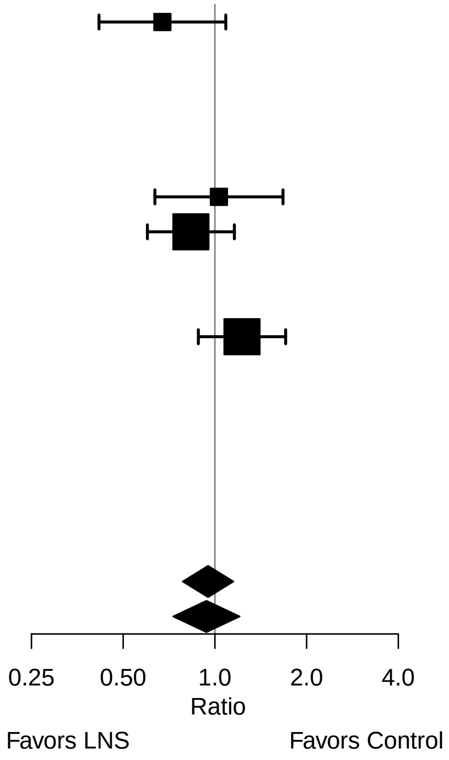

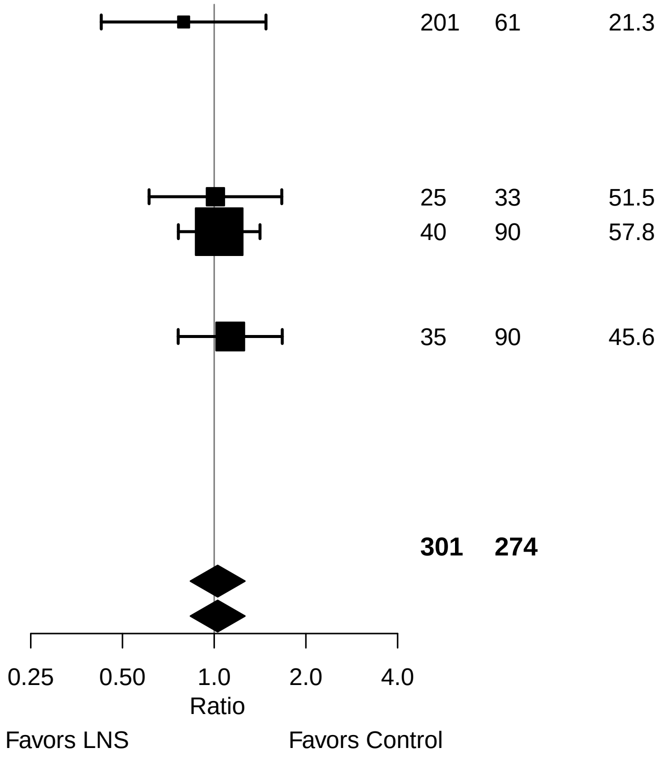

Supplemental figure 9U: Marginal vitamin A (retinol < 1.05 µmol/L) prevalence ratio

9U2: Stratified by Household food insecurity (insufficient comparisons)

Supplemental figure 9U: Marginal vitamin A (retinol < 1.05 µmol/L) prevalence ratio

9U3: Stratified by Household source water quality (insufficient comparisons)

Supplemental figure 9U: Marginal vitamin A (retinol < 1.05 µmol/L) prevalence ratio

9U4: Stratified by Household sanitation (insufficient comparisons)

Supplemental figure 9U: Marginal vitamin A (retinol < 1.05 μmol/L) prevalence ratio

9U5: Stratified by Season at the time of assessment

P−for−interaction = 0.615
Ratio of PRs = 1.09 (0.77, 1.54)

		Dry						Rainy					
Country	Trial	LNS N	Control N	Control Prevalence	PR (95% CI)	Fixed W	Random W	LNS N	Control N	Control Prevalence	PR (95% CI)	Fixed W	Random W
Bangladesh	JiVitA−4 (35)	257	69	15.9	0.61 (0.33, 1.13)	0.07	0.16	177	70	22.9	0.91 (0.51, 1.64)	0.21	0.23
Bangladesh	RDNS (36)												
Bangladesh	WASH−B (37)												
Burkina Faso	iLiNS−Zinc (38)												
Burkina Faso	PROMIS CS (39)												
Ghana	GHANA (40)	35	41	70.7	0.93 (0.68, 1.27)	0.27	0.27	28	33	21.2	1.35 (0.56, 3.25)	0.09	0.11
Ghana	iLiNS−DYAD−G (41)	59	122	59.8	0.82 (0.61, 1.11)	0.30	0.28	37	73	52.1	1.09 (0.76, 1.56)	0.57	0.52
Kenya	WASH−B (42)												
Madagascar	MAHAY (43)												
Malawi	iLiNS−DYAD−M (44)	54	120	45.8	1.45 (1.11, 1.91)	0.36	0.29	17	43	55.8	0.53 (0.24, 1.15)	0.12	0.14
Malawi	iLiNS−DOSE (45)												
Mali	PROMIS CS (46)												
Zimbabwe	SHINE (HIV−) (47)												
Zimbabwe	SHINE (HIV+) (48)												
		**405**	**352**		**I² = 0.74, Tau² = 0.09**			**259**	**219**		**I² = 0.09, Tau² = 0.01**		
**Fixed**					**1.02 (0.87, 1.20)**						**0.98 (0.75, 1.29)**		
**Random**					**0.95 (0.68, 1.34)**						**0.97 (0.72, 1.31)**		

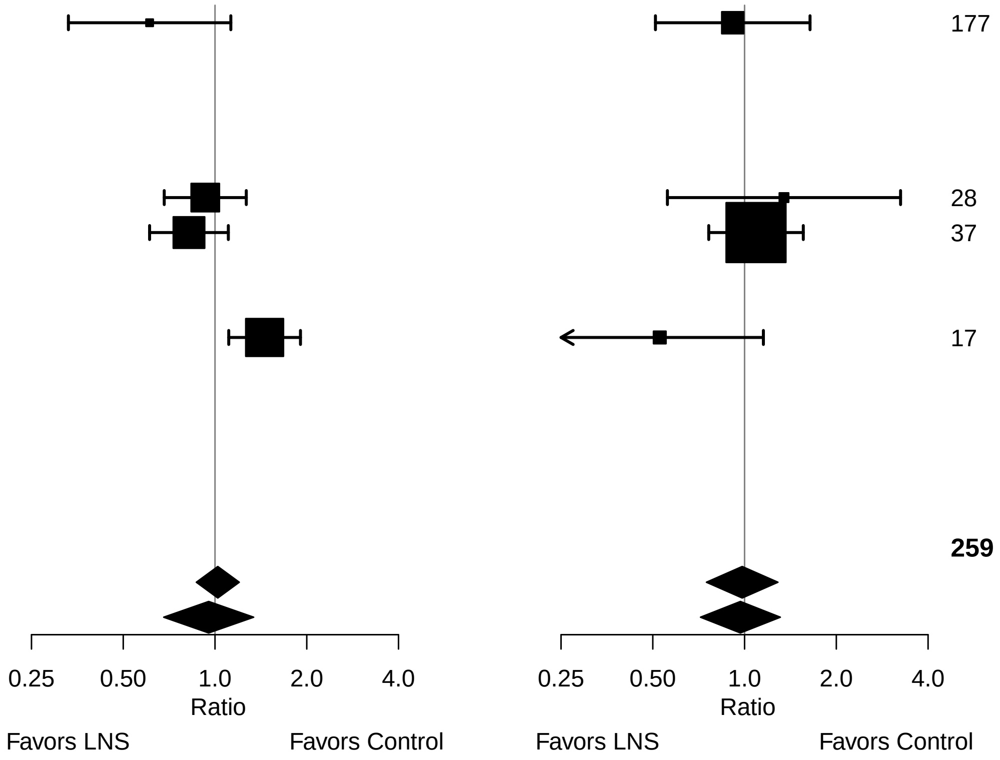

Supplemental figure 9V: Marginal vitamin A (retinol < 1.05 µmol/L) prevalence difference

9V1: Stratified by Household socio-economic status

P−for−interaction = 0.712
Difference in PDs = 0.02 (−0.09, 0.13)

		At least median							Less than median					
Country	Trial	LNS N	Control N	Control Prevalence	PD (95% CI)	Fixed W	Random W		LNS N	Control N	Control Prevalence	PD (95% CI)	Fixed W	Random W
Bangladesh	JiVitA−4 (35)	232	78	17.9	−0.06 (−0.13, 0.01)	0.70	0.62		201	61	21.3	−0.04 (−0.17, 0.08)	0.47	0.47
Bangladesh	RDNS (36)													
Bangladesh	WASH−B (37)													
Burkina Faso	iLiNS−Zinc (38)													
Burkina Faso	PROMIS CS (39)													
Ghana	GHANA (40)	38	37	45.9	0.01 (−0.21, 0.24)	0.07	0.09		25	33	51.5	0.00 (−0.26, 0.27)	0.11	0.11
Ghana	iLiNS−DYAD−G (41)	56	106	55.7	−0.09 (−0.25, 0.07)	0.14	0.17		40	90	57.8	0.02 (−0.16, 0.21)	0.22	0.22
Kenya	WASH−B (42)													
Madagascar	MAHAY (43)													
Malawi	iLiNS−DYAD−M (44)	36	73	52.1	0.12 (−0.08, 0.32)	0.09	0.12		35	90	45.6	0.06 (−0.14, 0.25)	0.20	0.20
Malawi	iLiNS−DOSE (45)													
Mali	PROMIS CS (46)													
Zimbabwe	SHINE (HIV−) (47)													
Zimbabwe	SHINE (HIV+) (48)													
		**362**	**294**		**I² = 0.09, Tau² = 0.00**				**301**	**274**		**I² = 0.00, Tau² = 0.00**		
**Fixed**					**−0.04 (−0.10, 0.02)**							**0.00 (−0.09, 0.08)**		
**Random**					**−0.04 (−0.11, 0.03)**							**0.00 (−0.09, 0.08)**		

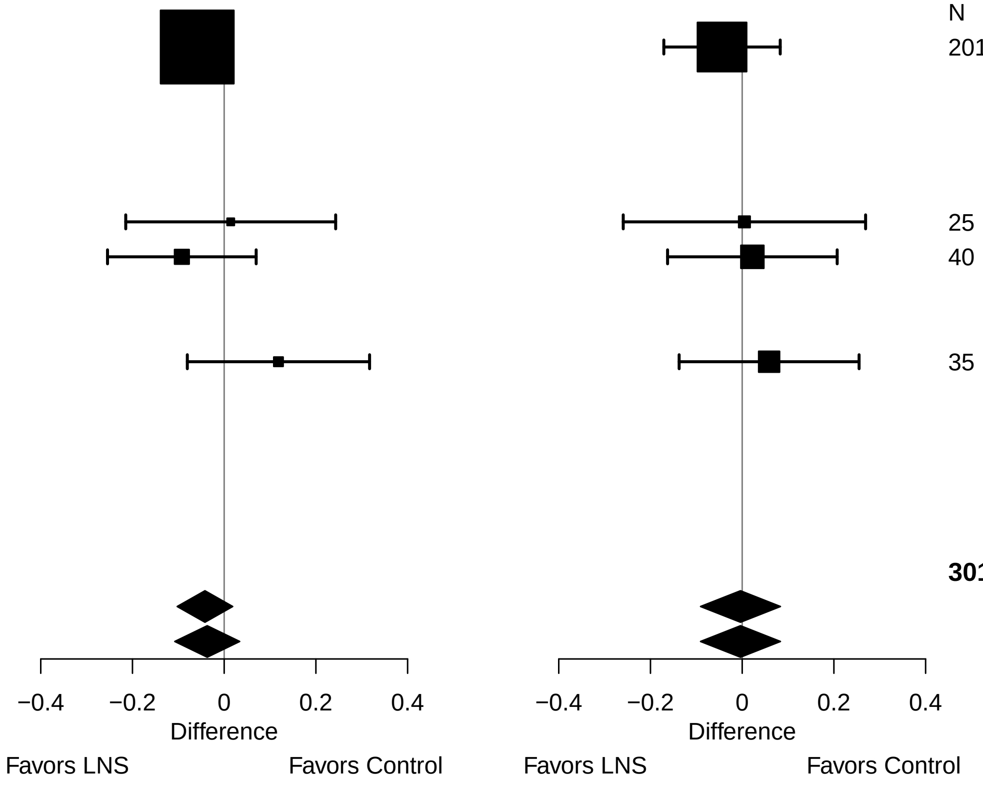

Supplemental figure 9V: Marginal vitamin A (retinol < 1.05 µmol/L) prevalence difference

9V2: Stratified by Household food insecurity (insufficient comparisons)

Supplemental figure 9V: Marginal vitamin A (retinol < 1.05 µmol/L) prevalence difference

9V3: Stratified by Household source water quality (insufficient comparisons)

Supplemental figure 9V: Marginal vitamin A (retinol < 1.05 μmol/L) prevalence difference

9V4: Stratified by Household sanitation (insufficient comparisons)

Supplemental figure 9V: Marginal vitamin A (retinol < 1.05 µmol/L) prevalence difference

9V5: Stratified by Season at the time of assessment

P−for−interaction = 0.888
Difference in PDs = 0.01 (−0.10, 0.12)

		Dry						Rainy					
Country	Trial	LNS N	Control N	Control Prevalence	PD (95% CI)	Fixed W	Random W	LNS N	Control N	Control Prevalence	PD (95% CI)	Fixed W	Random W
Bangladesh	JiVitA−4 (35)	257	69	15.9	−0.06 (−0.15, 0.03)	0.55	0.31	177	70	22.9	−0.02 (−0.15, 0.11)	0.49	0.38
Bangladesh	RDNS (36)												
Bangladesh	WASH−B (37)												
Burkina Faso	iLiNS−Zinc (38)												
Burkina Faso	PROMIS CS (39)												
Ghana	GHANA (40)	35	41	70.7	−0.05 (−0.26, 0.16)	0.10	0.19	28	33	21.2	0.07 (−0.15, 0.29)	0.18	0.22
Ghana	iLiNS−DYAD−G (41)	59	122	59.8	−0.11 (−0.26, 0.05)	0.18	0.25	37	73	52.1	0.05 (−0.15, 0.25)	0.22	0.25
Kenya	WASH−B (42)												
Madagascar	MAHAY (43)												
Malawi	iLiNS−DYAD−M (44)	54	120	45.8	0.21 (0.05, 0.37)	0.17	0.24	17	43	55.8	−0.26 (−0.54, 0.01)	0.11	0.16
Malawi	iLiNS−DOSE (45)												
Mali	PROMIS CS (46)												
Zimbabwe	SHINE (HIV−) (47)												
Zimbabwe	SHINE (HIV+) (48)												
		**405**	**352**		**I² = 0.70, Tau² = 0.01**			**259**	**219**		**I² = 0.27, Tau² = 0.01**		
**Fixed**					**−0.02 (−0.09, 0.04)**						**−0.02 (−0.11, 0.08)**		
**Random**					**0.00 (−0.14, 0.13)**						**−0.02 (−0.15, 0.10)**		

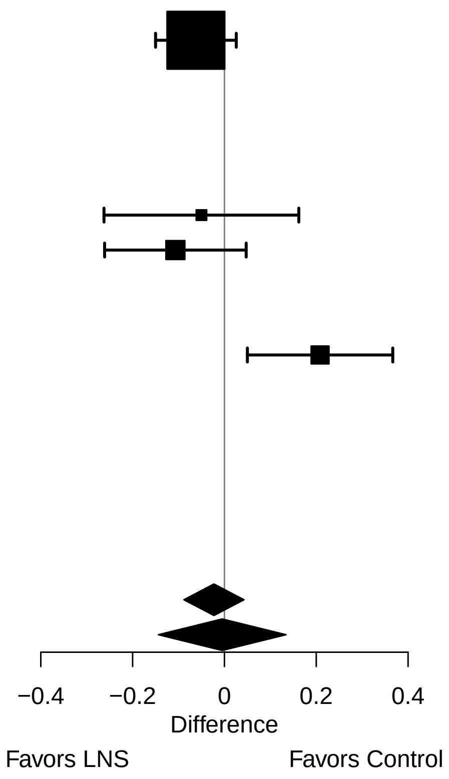

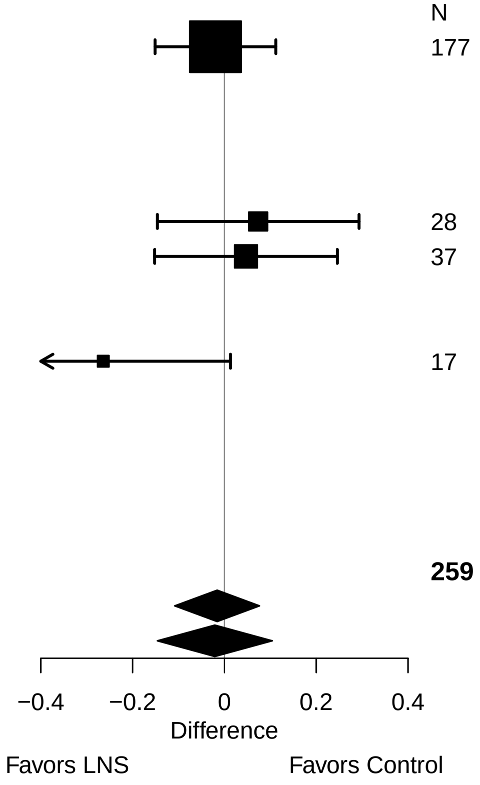

Supplemental figure 9W: Geometric mean ratio of retinol binding protein concentration

9W1: Stratified by Household socio-economic status

P−for−interaction = 0.108
Ratio of GMRs = 0.96 (0.92, 1.01)

		At least median						Less than median					
		**LNS**	**Control**	**Control**	**GMR**	**Fixed**	**Random**	**LNS**	**Control**	**Control**	**GMR**	**Fixed**	**Random**
Country	Trial	N	N	Median	(95% CI)	W	W	N	N	Median	(95% CI)	W	W
Bangladesh	JiVitA−4 (35)												
Bangladesh	RDNS (36)	273	136	1.2	1.03 (0.96, 1.11)	0.23	0.23	277	136	1.2	1.01 (0.95, 1.07)	0.28	0.25
Bangladesh	WASH−B (37)	105	91	1.1	1.08 (1.01, 1.16)	0.24	0.23	107	87	1.1	1.00 (0.94, 1.07)	0.20	0.21
Burkina Faso	iLiNS−Zinc (38)	180	39	1.1	1.08 (0.98, 1.20)	0.11	0.13	135	57	1.0	1.09 (1.03, 1.17)	0.22	0.22
Burkina Faso	PROMIS CS (39)												
Ghana	GHANA (40)												
Ghana	iLiNS−DYAD−G (41)												
Kenya	WASH−B (42)	163	155	1.0	1.16 (1.09, 1.23)	0.30	0.27	134	104	1.0	1.11 (1.04, 1.18)	0.25	0.24
Madagascar	MAHAY (43)	38	25	1.1	1.05 (0.95, 1.15)	0.12	0.14	43	25	1.1	1.04 (0.91, 1.18)	0.06	0.09
Malawi	iLiNS−DYAD−M (44)												
Malawi	iLiNS−DOSE (45)												
Mali	PROMIS CS (46)												
Zimbabwe	SHINE (HIV−) (47)												
Zimbabwe	SHINE (HIV+) (48)												
		**759**	**446**		**I² = 0.41, Tau² = 0.00**			**696**	**409**		**I² = 0.50, Tau² = 0.00**		
**Fixed**					**1.09 (1.05, 1.13)**						**1.05 (1.02, 1.08)**		
**Random**					**1.09 (1.04, 1.13)**						**1.05 (1.01, 1.10)**		

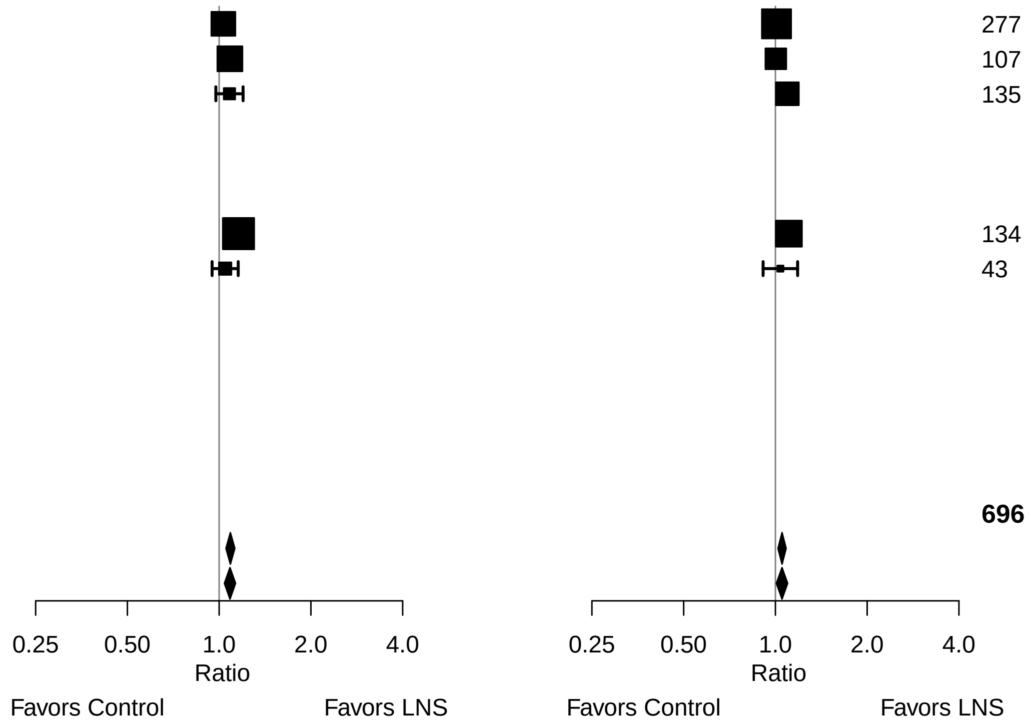

Supplemental figure 9W: Geometric mean ratio of retinol binding protein concentration

9W2: Stratified by Household food insecurity

P−for−interaction = 0.183
Ratio of GMRs = 1.04 (0.98, 1.09)

Mild to secure

Country	Trial	LNS N	Control N	Control Median	GMR (95% CI)	Fixed W	Random W
Bangladesh	JiVitA−4 (35)						
Bangladesh	RDNS (36)	342	166	1.2	1.03 (0.96, 1.10)	0.19	0.21
Bangladesh	WASH−B (37)	171	139	1.2	1.02 (0.97, 1.08)	0.29	0.27
Burkina Faso	iLiNS−Zinc (38)	160	45	1.1	1.10 (1.00, 1.21)	0.10	0.14
Burkina Faso	PROMIS CS (39)						
Ghana	GHANA (40)						
Ghana	iLiNS−DYAD−G (41)						
Kenya	WASH−B (42)	259	235	1.0	1.13 (1.07, 1.18)	0.38	0.30
Madagascar	MAHAY (43)	45	30	1.1	1.07 (0.93, 1.23)	0.05	0.08
Malawi	iLiNS−DYAD−M (44)						
Malawi	iLiNS−DOSE (45)						
Mali	PROMIS CS (46)						
Zimbabwe	SHINE (HIV−) (47)						
Zimbabwe	SHINE (HIV+) (48)						
		**977**	**615**		**I^2 = 0.53, Tau^2 = 0.00**		
**Fixed**					**1.07 (1.04, 1.10)**		
**Random**					**1.07 (1.02, 1.11)**		

Moderate to severe

Country	Trial	LNS N	Control N	Control Median	GMR (95% CI)	Fixed W	Random W
Bangladesh	JiVitA−4 (35)						
Bangladesh	RDNS (36)	208	106	1.2	1.01 (0.95, 1.07)	0.29	0.25
Bangladesh	WASH−B (37)	41	39	1.0	1.11 (1.00, 1.23)	0.09	0.18
Burkina Faso	iLiNS−Zinc (38)	155	51	1.1	1.08 (1.03, 1.13)	0.49	0.26
Burkina Faso	PROMIS CS (39)						
Ghana	GHANA (40)						
Ghana	iLiNS−DYAD−G (41)						
Kenya	WASH−B (42)	38	24	0.8	1.27 (1.13, 1.42)	0.07	0.16
Madagascar	MAHAY (43)	19	7	1.0	1.09 (0.96, 1.24)	0.06	0.15
Malawi	iLiNS−DYAD−M (44)						
Malawi	iLiNS−DOSE (45)						
Mali	PROMIS CS (46)						
Zimbabwe	SHINE (HIV−) (47)						
Zimbabwe	SHINE (HIV+) (48)						
		**461**	**227**		**I^2 = 0.68, Tau^2 = 0.00**		
**Fixed**					**1.07 (1.04, 1.11)**		
**Random**					**1.10 (1.02, 1.18)**		

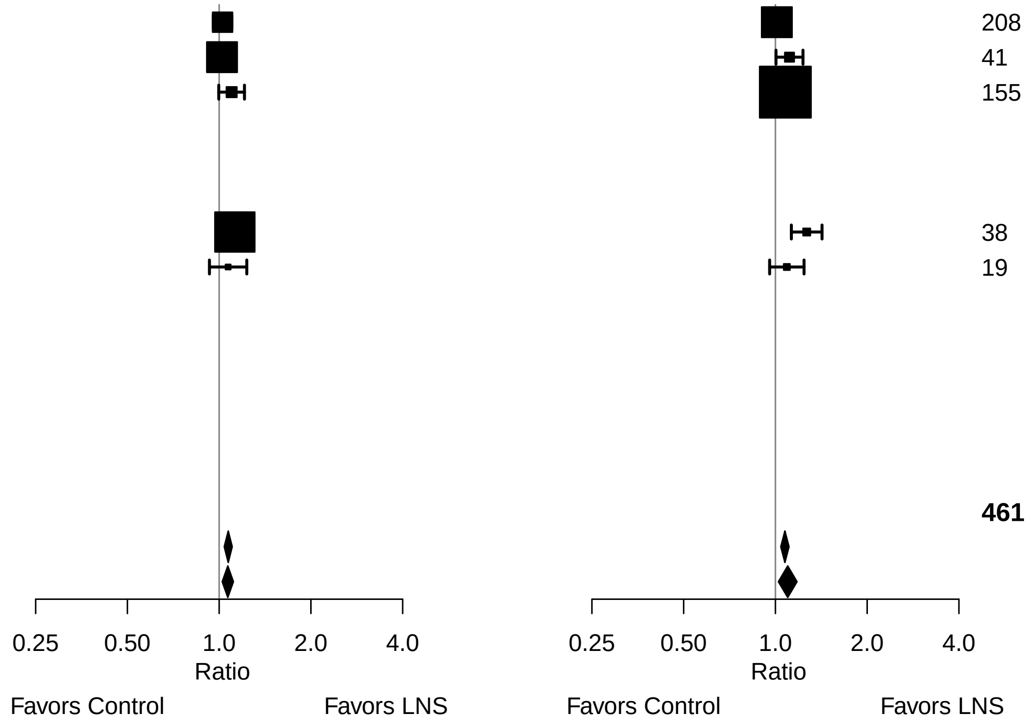

Supplemental figure 9W: Geometric mean ratio of retinol binding protein concentration

9W3: Stratified by Household source water quality

P−for−interaction = 0.959
Ratio of GMRs = 1.00 (0.92, 1.09)

Country	Trial	LNS N	Control N	Control Median	Improved GMR (95% CI)	Fixed W	Random W	LNS N	Control N	Control Median	Unimproved GMR (95% CI)	Fixed W	Random W
Bangladesh	JiVitA−4 (35)												
Bangladesh	RDNS (36)												
Bangladesh	WASH−B (37)	75	44	1.1	1.11 (1.00, 1.22)	0.29	0.29	19	14	1.0	1.05 (0.84, 1.30)	0.04	0.04
Burkina Faso	iLiNS−Zinc (38)	97	22	1.0	1.12 (0.97, 1.30)	0.13	0.18	218	74	1.1	1.09 (1.03, 1.15)	0.64	0.64
Burkina Faso	PROMIS CS (39)												
Ghana	GHANA (40)												
Ghana	iLiNS−DYAD−G (41)												
Kenya	WASH−B (42)	111	107	1.0	1.17 (1.08, 1.27)	0.45	0.36	55	37	0.9	1.17 (1.05, 1.30)	0.16	0.16
Madagascar	MAHAY (43)	23	14	1.2	0.97 (0.83, 1.13)	0.12	0.17	55	36	1.1	1.07 (0.96, 1.19)	0.16	0.16
Malawi	iLiNS−DYAD−M (44)												
Malawi	iLiNS−DOSE (45)												
Mali	PROMIS CS (46)												
Zimbabwe	SHINE (HIV−) (47)												
Zimbabwe	SHINE (HIV+) (48)												
		**306**	**187**		**I² = 0.37, Tau² = 0.00**			**347**	**161**		**I² = 0.00, Tau² = 0.00**		
**Fixed**					**1.12 (1.06, 1.18)**						**1.09 (1.05, 1.14)**		
**Random**					**1.11 (1.03, 1.19)**						**1.09 (1.05, 1.14)**		

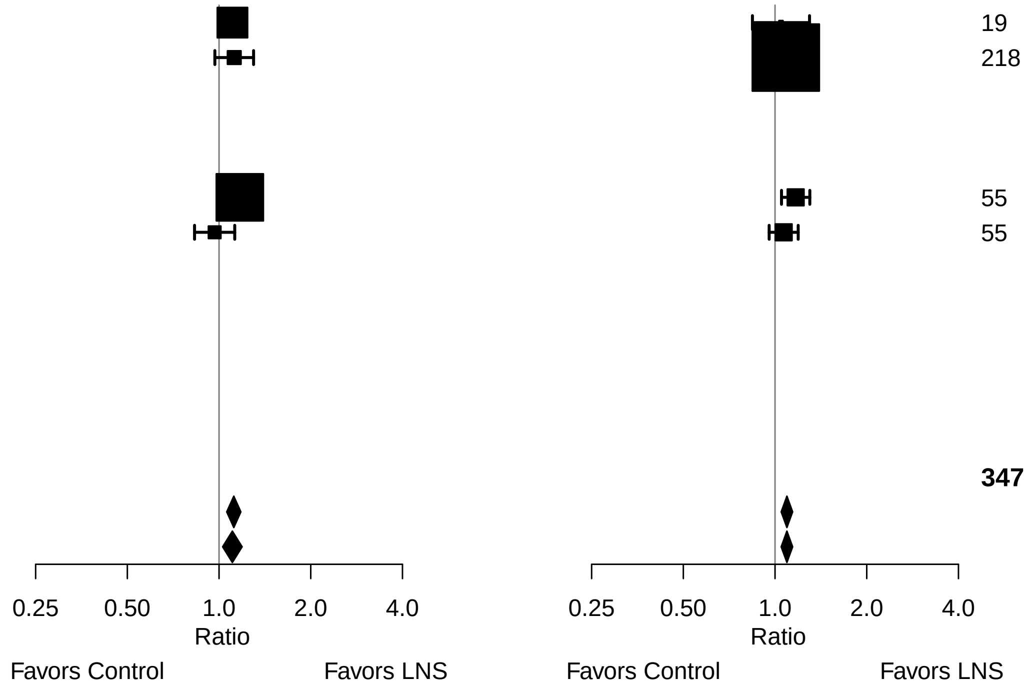

Supplemental figure 9W: Geometric mean ratio of retinol binding protein concentration

9W4: Stratified by Household sanitation (insufficient comparisons)

Supplemental figure 9W: Geometric mean ratio of retinol binding protein concentration

9W5: Stratified by Season at the time of assessment

P−for−interaction = 0.101
Ratio of GMRs = 0.96 (0.92, 1.01)

		Dry						Rainy					
Country	Trial	LNS N	Control N	Control Median	GMR (95% CI)	Fixed W	Random W	LNS N	Control N	Control Median	GMR (95% CI)	Fixed W	Random W
Bangladesh	JiVitA−4 (35)												
Bangladesh	RDNS (36)	341	166	1.2	1.03 (0.98, 1.08)	0.30	0.28	208	106	1.3	1.01 (0.94, 1.08)	0.28	0.26
Bangladesh	WASH−B (37)	96	93	1.1	1.10 (1.05, 1.15)	0.35	0.29	116	85	1.2	0.98 (0.90, 1.06)	0.20	0.24
Burkina Faso	iLiNS−Zinc (38)	174	61	1.0	1.09 (1.02, 1.15)	0.23	0.25	126	30	1.1	1.09 (1.00, 1.19)	0.17	0.22
Burkina Faso	PROMIS CS (39)												
Ghana	GHANA (40)												
Ghana	iLiNS−DYAD−G (41)												
Kenya	WASH−B (42)	88	75	1.0	1.16 (1.06, 1.26)	0.12	0.18	210	184	1.0	1.13 (1.06, 1.20)	0.35	0.28
Madagascar	MAHAY (43)												
Malawi	iLiNS−DYAD−M (44)												
Malawi	iLiNS−DOSE (45)												
Mali	PROMIS CS (46)												
Zimbabwe	SHINE (HIV−) (47)												
Zimbabwe	SHINE (HIV+) (48)												
		**699**	**395**		**I^2 = 0.53, Tau^2 = 0.00**			**660**	**405**		**I^2 = 0.71, Tau^2 = 0.00**		
**Fixed**					**1.08 (1.05, 1.11)**						**1.06 (1.02, 1.09)**		
**Random**					**1.09 (1.04, 1.14)**						**1.05 (0.98, 1.12)**		

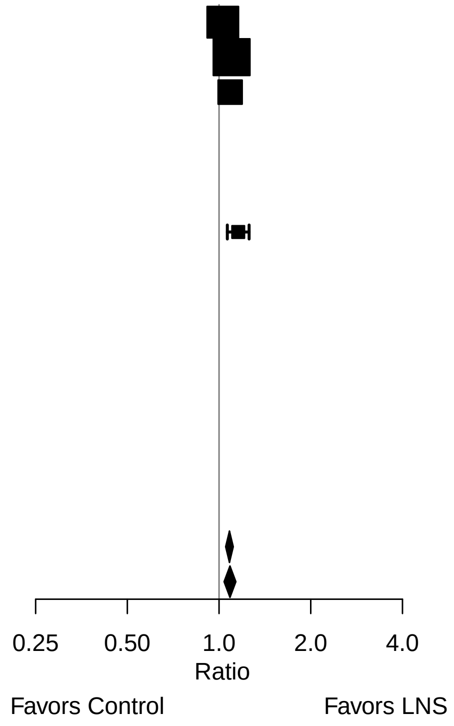

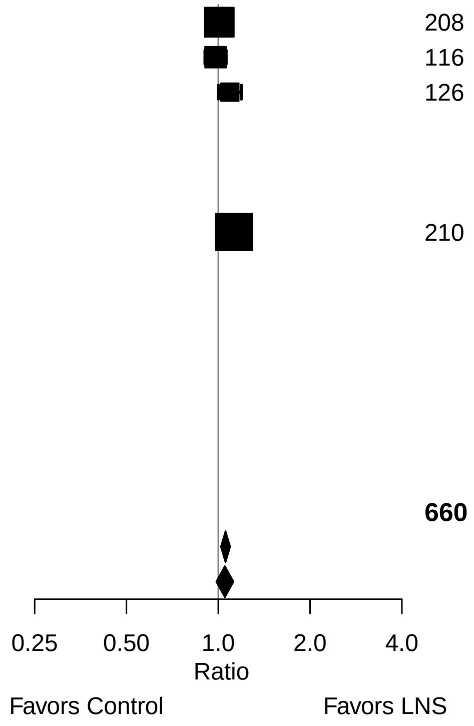

Supplemental figure 9X: Low vitamin A status (RBP < 0.70 µmol/L) prevalence ratio

9X1: Stratified by Household socio-economic status (insufficient comparisons)

Supplemental figure 9X: Low vitamin A status (RBP < 0.70 µmol/L) prevalence ratio

9X2: Stratified by Household food insecurity (insufficient comparisons)

Supplemental figure 9X: Low vitamin A status (RBP < 0.70 µmol/L) prevalence ratio

9X3: Stratified by Household source water quality (insufficient comparisons)

Supplemental figure 9X: Low vitamin A status (RBP < 0.70 µmol/L) prevalence ratio

9X4: Stratified by Household sanitation (insufficient comparisons)

Supplemental figure 9X: Low vitamin A status (RBP < 0.70 µmol/L) prevalence ratio

9X5: Stratified by Season at the time of assessment (insufficient comparisons)

Supplemental figure 9Y: Low vitamin A status (RBP < 0.70 µmol/L) prevalence difference

9Y1: Stratified by Household socio-economic status (insufficient comparisons)

Supplemental figure 9Y: Low vitamin A status (RBP < 0.70 μmol/L) prevalence difference

9Y2: Stratified by Household food insecurity (insufficient comparisons)

Supplemental figure 9Y: Low vitamin A status (RBP < 0.70 µmol/L) prevalence difference

9Y3: Stratified by Household source water quality (insufficient comparisons)

Supplemental figure 9Y: Low vitamin A status (RBP < 0.70 µmol/L) prevalence difference

9Y4: Stratified by Household sanitation (insufficient comparisons)

Supplemental figure 9Y: Low vitamin A status (RBP < 0.70 µmol/L) prevalence difference

9Y5: Stratified by Season at the time of assessment (insufficient comparisons)

Supplemental figure 9Z: Marginal vitamin A status (RBP < 1.05 μmol/L) prevalence ratio

9Z1: Stratified by Household socio-economic status

P−for−interaction = 0.026
Ratio of PRs = 1.26 (1.03, 1.56)

Country	Trial	At least median: LNS N	Control N	Control Prevalence	PR (95% CI)	Fixed W	Random W	Less than median: LNS N	Control N	Control Prevalence	PR (95% CI)	Fixed W	Random W
Bangladesh	JiVitA−4 (35)												
Bangladesh	RDNS (36)	273	136	30.9	0.75 (0.53, 1.05)	0.21	0.21	277	136	27.2	1.01 (0.71, 1.43)	0.17	0.17
Bangladesh	WASH−B (37)	105	91	34.1	0.73 (0.47, 1.12)	0.13	0.13	107	87	36.8	0.97 (0.72, 1.30)	0.24	0.24
Burkina Faso	iLiNS−Zinc (38)	180	39	44.2	0.86 (0.55, 1.35)	0.12	0.12	135	57	52.4	0.69 (0.49, 0.96)	0.19	0.19
Burkina Faso	PROMIS CS (39)												
Ghana	GHANA (40)												
Ghana	iLiNS−DYAD−G (41)												
Kenya	WASH−B (42)	163	155	63.2	0.63 (0.50, 0.79)	0.48	0.48	134	104	53.8	0.86 (0.67, 1.10)	0.35	0.35
Madagascar	MAHAY (43)	38	25	36.0	0.80 (0.41, 1.58)	0.05	0.05	43	25	40.0	0.87 (0.43, 1.78)	0.04	0.04
Malawi	iLiNS−DYAD−M (44)												
Malawi	iLiNS−DOSE (45)												
Mali	PROMIS CS (46)												
Zimbabwe	SHINE (HIV−) (47)												
Zimbabwe	SHINE (HIV+) (48)												
		**759**	**446**		**I² = 0.00, Tau² = 0.00**			**696**	**409**		**I² = 0.00, Tau² = 0.00**		
**Fixed**					**0.70 (0.60, 0.82)**						**0.87 (0.75, 1.01)**		
**Random**					**0.70 (0.60, 0.82)**						**0.87 (0.75, 1.01)**		

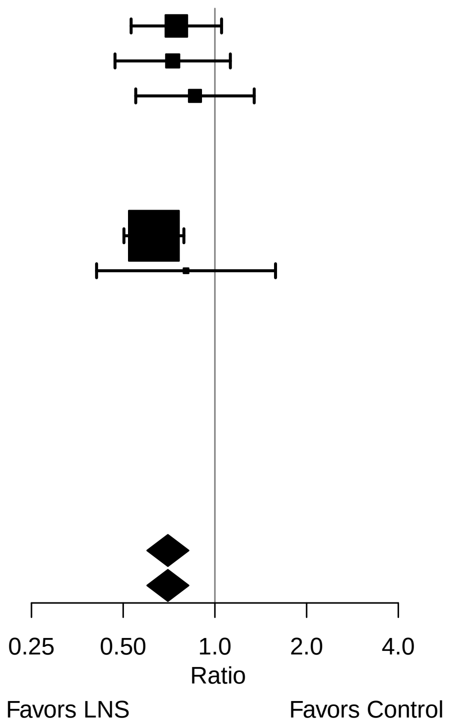

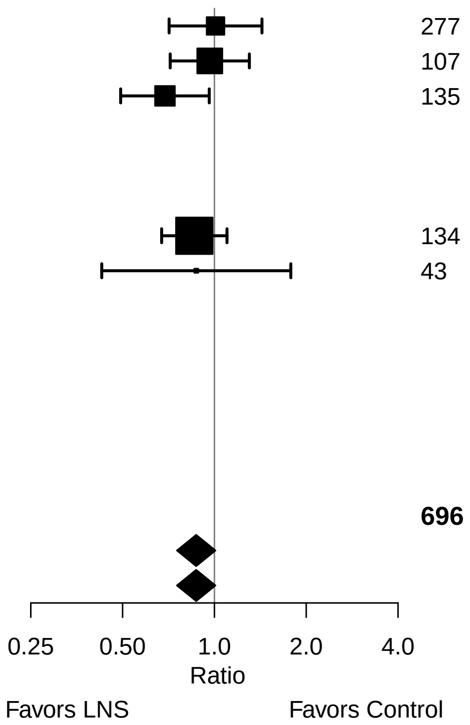

Supplemental figure 9Z: Marginal vitamin A status (RBP < 1.05 µmol/L) prevalence ratio

9Z2: Stratified by Household food insecurity

P−for−interaction = 0.208
Ratio of PRs = 0.85 (0.67, 1.09)

Mild to secure

Country	Trial	LNS N	Control N	Control Prevalence	PR (95% CI)	Fixed W	Random W
Bangladesh	JiVitA−4 (35)						
Bangladesh	RDNS (36)	342	166	28.9	0.89 (0.63, 1.26)	0.16	0.16
Bangladesh	WASH−B (37)	171	139	29.5	0.95 (0.67, 1.35)	0.16	0.16
Burkina Faso	iLiNS−Zinc (38)	160	45	49.0	0.73 (0.49, 1.08)	0.13	0.13
Burkina Faso	PROMIS CS (39)						
Ghana	GHANA (40)						
Ghana	iLiNS−DYAD−G (41)						
Kenya	WASH−B (42)	259	235	57.4	0.74 (0.61, 0.89)	0.56	0.56
Madagascar	MAHAY (43)						
Malawi	iLiNS−DYAD−M (44)						
Malawi	iLiNS−DOSE (45)						
Mali	PROMIS CS (46)						
Zimbabwe	SHINE (HIV−) (47)						
Zimbabwe	SHINE (HIV+) (48)						
		**932**	**585**		**I² = 0.00, Tau² = 0.00**		
**Fixed**					**0.79 (0.69, 0.91)**		
**Random**					**0.79 (0.69, 0.91)**		

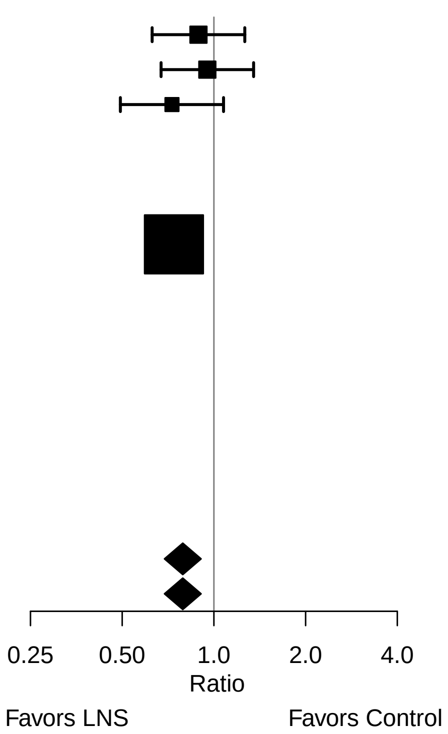

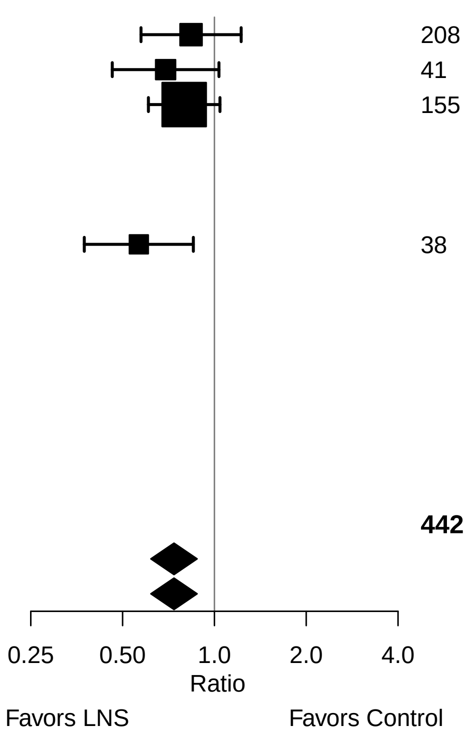

Moderate to severe

Country	Trial	LNS N	Control N	Control Prevalence	PR (95% CI)	Fixed W	Random W
Bangladesh	JiVitA−4 (35)						
Bangladesh	RDNS (36)	208	106	29.2	0.84 (0.57, 1.22)	0.21	0.21
Bangladesh	WASH−B (37)	41	39	56.4	0.69 (0.46, 1.03)	0.19	0.19
Burkina Faso	iLiNS−Zinc (38)	155	51	49.1	0.80 (0.61, 1.04)	0.42	0.42
Burkina Faso	PROMIS CS (39)						
Ghana	GHANA (40)						
Ghana	iLiNS−DYAD−G (41)						
Kenya	WASH−B (42)	38	24	79.2	0.57 (0.37, 0.85)	0.18	0.18
Madagascar	MAHAY (43)						
Malawi	iLiNS−DYAD−M (44)						
Malawi	iLiNS−DOSE (45)						
Mali	PROMIS CS (46)						
Zimbabwe	SHINE (HIV−) (47)						
Zimbabwe	SHINE (HIV+) (48)						
		**442**	**220**		**I² = 0.00, Tau² = 0.00**		
**Fixed**					**0.74 (0.62, 0.88)**		
**Random**					**0.74 (0.62, 0.88)**		

Supplemental figure 9Z: Marginal vitamin A status (RBP < 1.05 µmol/L) prevalence ratio

9Z3: Stratified by Household source water quality

P−for−interaction = 0.520
Ratio of PRs = 1.10 (0.82, 1.48)

Country	Trial	**LNS** N	**Control** N	**Control** Prevalence	**Improved PR** (95% CI)	**Fixed** W	**Random** W	**LNS** N	**Control** N	**Control** Prevalence	**Unimproved PR** (95% CI)	**Fixed** W	**Random** W
Bangladesh	JiVitA−4 (35)												
Bangladesh	RDNS (36)												
Bangladesh	WASH−B (37)	75	44	45.5	0.67 (0.40, 1.13)	0.16	0.16	19	14	50.0	0.84 (0.32, 2.22)	0.04	0.04
Burkina Faso	iLiNS−Zinc (38)	97	22	56.5	0.74 (0.51, 1.08)	0.29	0.29	218	74	47.0	0.76 (0.60, 0.96)	0.69	0.69
Burkina Faso	PROMIS CS (39)												
Ghana	GHANA (40)												
Ghana	iLiNS−DYAD−G (41)												
Kenya	WASH−B (42)	111	107	61.7	0.67 (0.51, 0.88)	0.55	0.55	55	37	67.6	0.78 (0.53, 1.14)	0.26	0.26
Madagascar	MAHAY (43)												
Malawi	iLiNS−DYAD−M (44)												
Malawi	iLiNS−DOSE (45)												
Mali	PROMIS CS (46)												
Zimbabwe	SHINE (HIV−) (47)												
Zimbabwe	SHINE (HIV+) (48)												
		**283**	**173**		**I² = 0.00, Tau² = 0.00**			**292**	**125**		**I² = 0.00, Tau² = 0.00**		
**Fixed**					**0.69 (0.56, 0.85)**						**0.77 (0.63, 0.93)**		
**Random**					**0.69 (0.56, 0.85)**						**0.77 (0.63, 0.93)**		

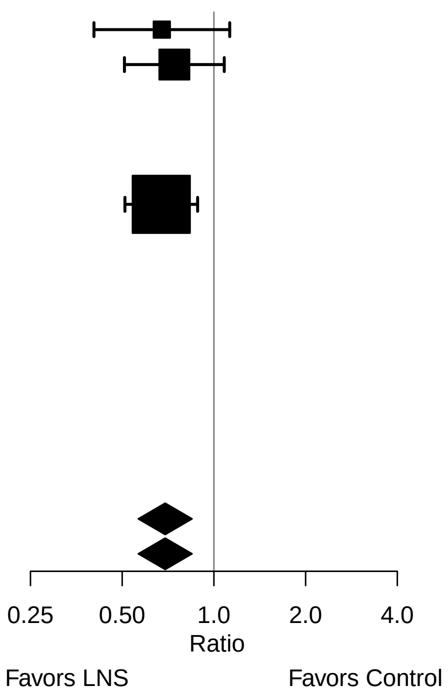

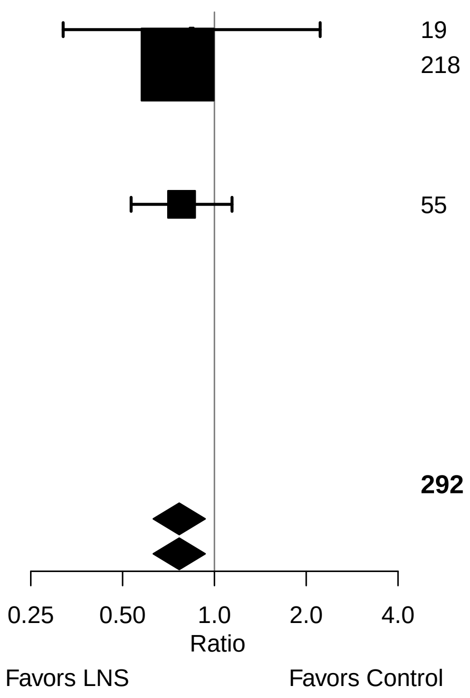

Supplemental figure 9Z: Marginal vitamin A status (RBP < 1.05 µmol/L) prevalence ratio

9Z4: Stratified by Household sanitation (insufficient comparisons)

Supplemental figure 9Z: Marginal vitamin A status (RBP < 1.05 µmol/L) prevalence ratio

9Z5: Stratified by Season at the time of assessment

P−for−interaction = 0.552
Ratio of PRs = 1.08 (0.84, 1.37)

Country	Trial	Dry: LNS N	Dry: Control N	Dry: Control Prevalence	Dry: PR (95% CI)	Dry: Fixed W	Dry: Random W	Rainy: LNS N	Rainy: Control N	Rainy: Control Prevalence	Rainy: PR (95% CI)	Rainy: Fixed W	Rainy: Random W
Bangladesh	JiVitA−4 (35)												
Bangladesh	RDNS (36)	341	166	28.9	0.85 (0.63, 1.15)	0.26	0.26	208	106	29.2	0.90 (0.59, 1.38)	0.15	0.17
Bangladesh	WASH−B (37)	96	93	44.1	0.71 (0.49, 1.02)	0.17	0.17	116	85	25.9	1.13 (0.68, 1.89)	0.11	0.12
Burkina Faso	iLiNS−Zinc (38)	174	61	50.7	0.75 (0.58, 0.97)	0.35	0.35	126	30	46.7	0.82 (0.52, 1.28)	0.14	0.15
Burkina Faso	PROMIS CS (39)												
Ghana	GHANA (40)												
Ghana	iLiNS−DYAD−G (41)												
Kenya	WASH−B (42)	88	75	56.0	0.75 (0.55, 1.03)	0.23	0.23	210	184	60.9	0.71 (0.58, 0.88)	0.60	0.57
Madagascar	MAHAY (43)												
Malawi	iLiNS−DYAD−M (44)												
Malawi	iLiNS−DOSE (45)												
Mali	PROMIS CS (46)												
Zimbabwe	SHINE (HIV−) (47)												
Zimbabwe	SHINE (HIV+) (48)												
		**699**	**395**		**I² = 0.00, Tau² = 0.00**			**660**	**405**		**I² = 0.08, Tau² = 0.00**		
**Fixed**					**0.77 (0.66, 0.89)**						**0.79 (0.67, 0.93)**		
**Random**					**0.77 (0.66, 0.89)**						**0.80 (0.67, 0.95)**		

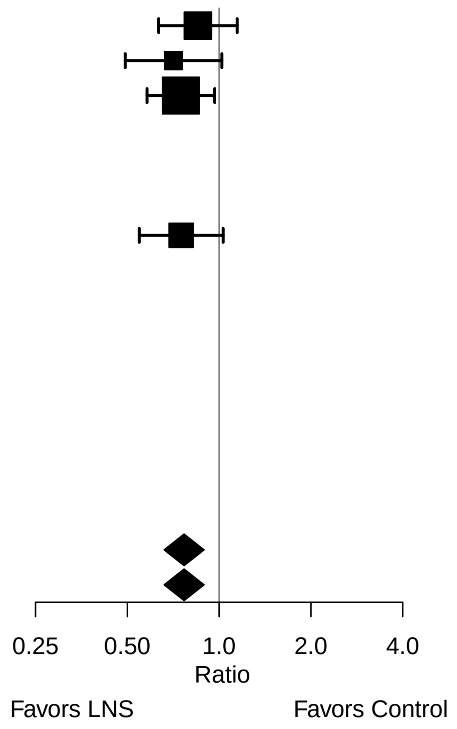

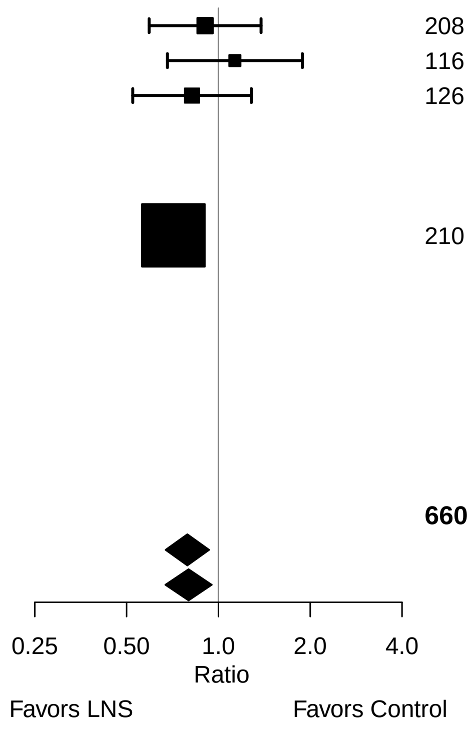

Supplemental figure 9AA: Marginal vitamin A status (RBP < 1.05 µmol/L) prevalence difference

9AA1: Stratified by Household socio-economic status

P−for−interaction = 0.076
Difference in PDs = 0.07 (−0.01, 0.15)

		At least median						Less than median					
Country	Trial	LNS N	Control N	Control Prevalence	PD (95% CI)	Fixed W	Random W	LNS N	Control N	Control Prevalence	PD (95% CI)	Fixed W	Random W
Bangladesh	JiVitA−4 (35)												
Bangladesh	RDNS (36)	273	136	30.9	−0.08 (−0.17, 0.02)	0.36	0.33	277	136	27.2	0.00 (−0.09, 0.10)	0.34	0.34
Bangladesh	WASH−B (37)	105	91	34.1	−0.09 (−0.22, 0.03)	0.21	0.22	107	87	36.8	−0.01 (−0.12, 0.10)	0.26	0.26
Burkina Faso	iLiNS−Zinc (38)	180	39	44.2	−0.07 (−0.25, 0.11)	0.10	0.12	135	57	52.4	−0.15 (−0.29, −0.01)	0.16	0.16
Burkina Faso	PROMIS CS (39)												
Ghana	GHANA (40)												
Ghana	iLiNS−DYAD−G (41)												
Kenya	WASH−B (42)	163	155	63.2	−0.23 (−0.34, −0.12)	0.27	0.26	134	104	53.8	−0.08 (−0.20, 0.05)	0.20	0.20
Madagascar	MAHAY (43)	38	25	36.0	−0.07 (−0.30, 0.16)	0.06	0.08	43	25	40.0	−0.05 (−0.32, 0.22)	0.04	0.04
Malawi	iLiNS−DYAD−M (44)												
Malawi	iLiNS−DOSE (45)												
Mali	PROMIS CS (46)												
Zimbabwe	SHINE (HIV−) (47)												
Zimbabwe	SHINE (HIV+) (48)												
		**759**	**446**		**I² = 0.26, Tau² = 0.00**			**696**	**409**		**I² = 0.00, Tau² = 0.00**		
**Fixed**					**−0.12 (−0.18, −0.06)**						**−0.04 (−0.10, 0.01)**		
**Random**					**−0.12 (−0.19, −0.05)**						**−0.04 (−0.10, 0.01)**		

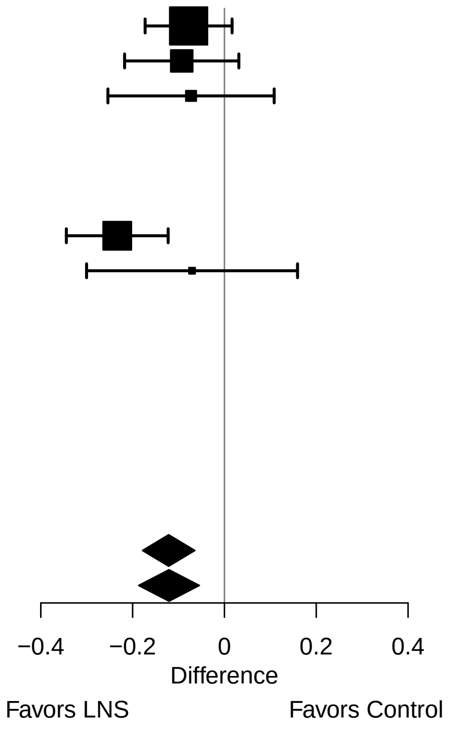

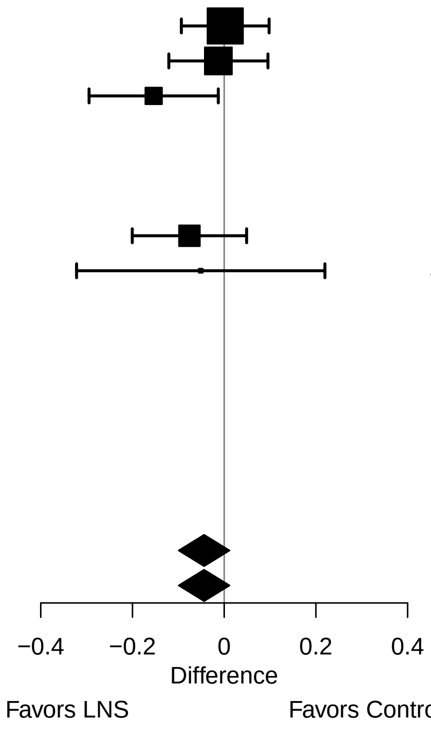

Supplemental figure 9AA: Marginal vitamin A status (RBP < 1.05 µmol/L) prevalence difference

9AA2: Stratified by Household food insecurity

P−for−interaction = 0.231
Difference in PDs = −0.06 (−0.15, 0.04)

		Mild to secure						Moderate to severe					
Country	Trial	LNS N	Control N	Control Prevalence	PD (95% CI)	Fixed W	Random W	LNS N	Control N	Control Prevalence	PD (95% CI)	Fixed W	Random W
Bangladesh	JiVitA−4 (35)												
Bangladesh	RDNS (36)	342	166	28.9	−0.03 (−0.13, 0.07)	0.29	0.28	208	106	29.2	−0.05 (−0.15, 0.06)	0.44	0.34
Bangladesh	WASH−B (37)	171	139	29.5	−0.01 (−0.11, 0.09)	0.28	0.27	41	39	56.4	−0.17 (−0.36, 0.02)	0.13	0.20
Burkina Faso	iLiNS−Zinc (38)	160	45	49.0	−0.12 (−0.29, 0.04)	0.10	0.14	155	51	49.1	−0.10 (−0.22, 0.02)	0.34	0.31
Burkina Faso	PROMIS CS (39)												
Ghana	GHANA (40)												
Ghana	iLiNS−DYAD−G (41)												
Kenya	WASH−B (42)	259	235	57.4	−0.15 (−0.24, −0.06)	0.33	0.30	38	24	79.2	−0.34 (−0.59, −0.10)	0.08	0.15
Madagascar	MAHAY (43)												
Malawi	iLiNS−DYAD−M (44)												
Malawi	iLiNS−DOSE (45)												
Mali	PROMIS CS (46)												
Zimbabwe	SHINE (HIV−) (47)												
Zimbabwe	SHINE (HIV+) (48)												
		**932**	**585**		**I² = 0.40, Tau² = 0.00**			**442**	**220**		**I² = 0.45, Tau² = 0.01**		
**Fixed**					**−0.07 (−0.13, −0.02)**						**−0.11 (−0.18, −0.04)**		
**Random**					**−0.08 (−0.14, −0.01)**						**−0.13 (−0.25, −0.02)**		

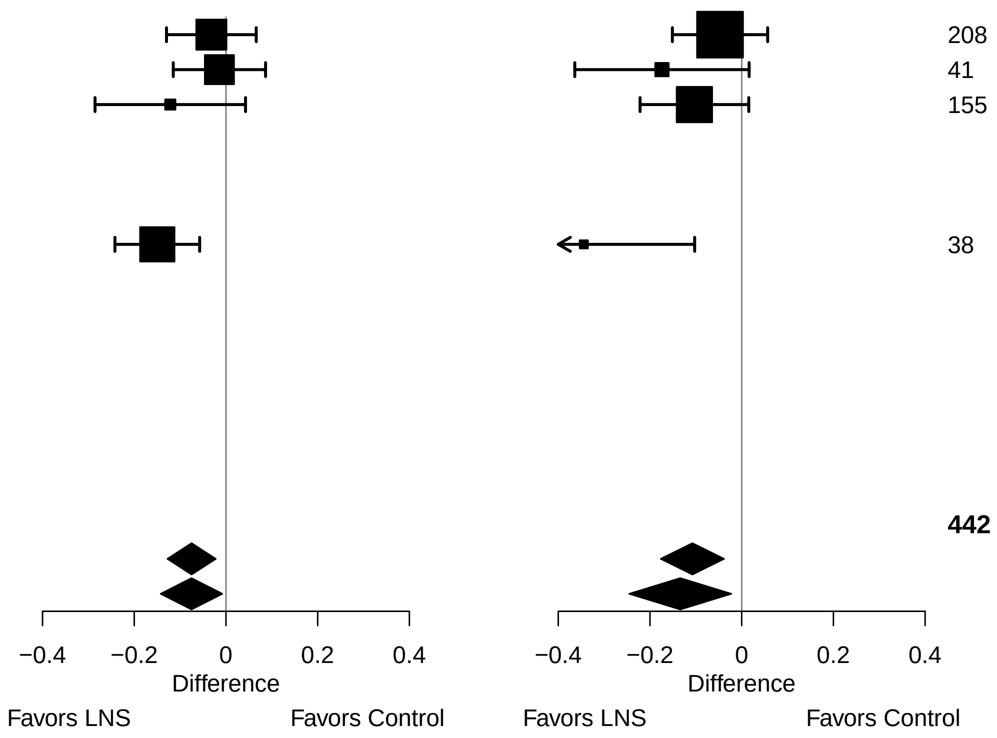

Supplemental figure 9AA: Marginal vitamin A status (RBP < 1.05 μmol/L) prevalence difference

9AA3: Stratified by Household source water quality

P−for−interaction = 0.499
Difference in PDs = 0.05 (−0.10, 0.21)

Country	Trial	Improved: LNS N	Improved: Control N	Improved: Control Prevalence	Improved: PD (95% CI)	Improved: Fixed W	Improved: Random W	Unimproved: LNS N	Unimproved: Control N	Unimproved: Control Prevalence	Unimproved: PD (95% CI)	Unimproved: Fixed W	Unimproved: Random W
Bangladesh	JiVitA−4 (35)												
Bangladesh	RDNS (36)												
Bangladesh	WASH−B (37)	75	44	45.5	−0.15 (−0.35, 0.05)	0.22	0.22	19	14	50.0	−0.08 (−0.52, 0.36)	0.03	0.03
Burkina Faso	iLiNS−Zinc (38)	97	22	56.5	−0.15 (−0.34, 0.03)	0.27	0.27	218	74	47.0	−0.11 (−0.20, −0.02)	0.84	0.84
Burkina Faso	PROMIS CS (39)												
Ghana	GHANA (40)												
Ghana	iLiNS−DYAD−G (41)												
Kenya	WASH−B (42)	111	107	61.7	−0.20 (−0.33, −0.07)	0.51	0.51	55	37	67.6	−0.15 (−0.38, 0.08)	0.13	0.13
Madagascar	MAHAY (43)												
Malawi	iLiNS−DYAD−M (44)												
Malawi	iLiNS−DOSE (45)												
Mali	PROMIS CS (46)												
Zimbabwe	SHINE (HIV−) (47)												
Zimbabwe	SHINE (HIV+) (48)												
		**283**	**173**		**I^2 = 0.00, Tau^2 = 0.00**			**292**	**125**		**I^2 = 0.00, Tau^2 = 0.00**		
**Fixed**					**−0.18 (−0.27, −0.08)**						**−0.11 (−0.20, −0.03)**		
**Random**					**−0.18 (−0.27, −0.08)**						**−0.11 (−0.20, −0.03)**		

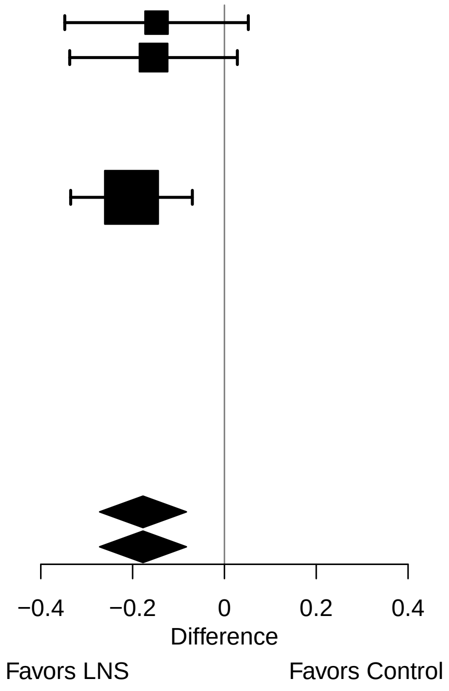

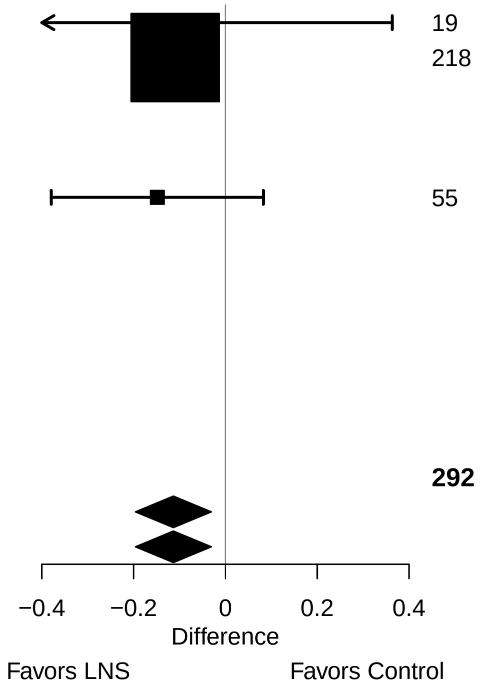

Supplemental figure 9AA: Marginal vitamin A status (RBP < 1.05 µmol/L) prevalence difference

9AA4: Stratified by Household sanitation (insufficient comparisons)

Supplemental figure 9AA: Marginal vitamin A status (RBP < 1.05 μmol/L) prevalence difference

9AA5: Stratified by Season at the time of assessment

P−for−interaction = 0.397
Difference in PDs = 0.04 (−0.05, 0.12)

Country	Trial	Dry LNS N	Dry Control N	Dry Control Prevalence	Dry PD (95% CI)	Dry Fixed W	Dry Random W	Rainy LNS N	Rainy Control N	Rainy Control Prevalence	Rainy PD (95% CI)	Rainy Fixed W	Rainy Random W
Bangladesh	JiVitA−4 (35)												
Bangladesh	RDNS (36)	341	166	28.9	−0.04 (−0.12, 0.04)	0.46	0.46	208	106	29.2	−0.03 (−0.15, 0.09)	0.29	0.28
Bangladesh	WASH−B (37)	96	93	44.1	−0.13 (−0.26, 0.00)	0.17	0.17	116	85	25.9	0.03 (−0.10, 0.17)	0.22	0.24
Burkina Faso	iLiNS−Zinc (38)	174	61	50.7	−0.13 (−0.24, −0.02)	0.24	0.24	126	30	46.7	−0.09 (−0.26, 0.08)	0.14	0.18
Burkina Faso	PROMIS CS (39)												
Ghana	GHANA (40)												
Ghana	iLiNS−DYAD−G (41)												
Kenya	WASH−B (42)	88	75	56.0	−0.14 (−0.30, 0.02)	0.12	0.12	210	184	60.9	−0.18 (−0.29, −0.06)	0.34	0.30
Madagascar	MAHAY (43)												
Malawi	iLiNS−DYAD−M (44)												
Malawi	iLiNS−DOSE (45)												
Mali	PROMIS CS (46)												
Zimbabwe	SHINE (HIV−) (47)												
Zimbabwe	SHINE (HIV+) (48)												
		**699**	**395**		**I^2 = 0.00, Tau^2 = 0.00**			**660**	**405**		**I^2 = 0.52, Tau^2 = 0.00**		
**Fixed**					**−0.09 (−0.14, −0.04)**						**−0.07 (−0.14, −0.01)**		
**Random**					**−0.09 (−0.14, −0.04)**						**−0.07 (−0.16, 0.02)**		

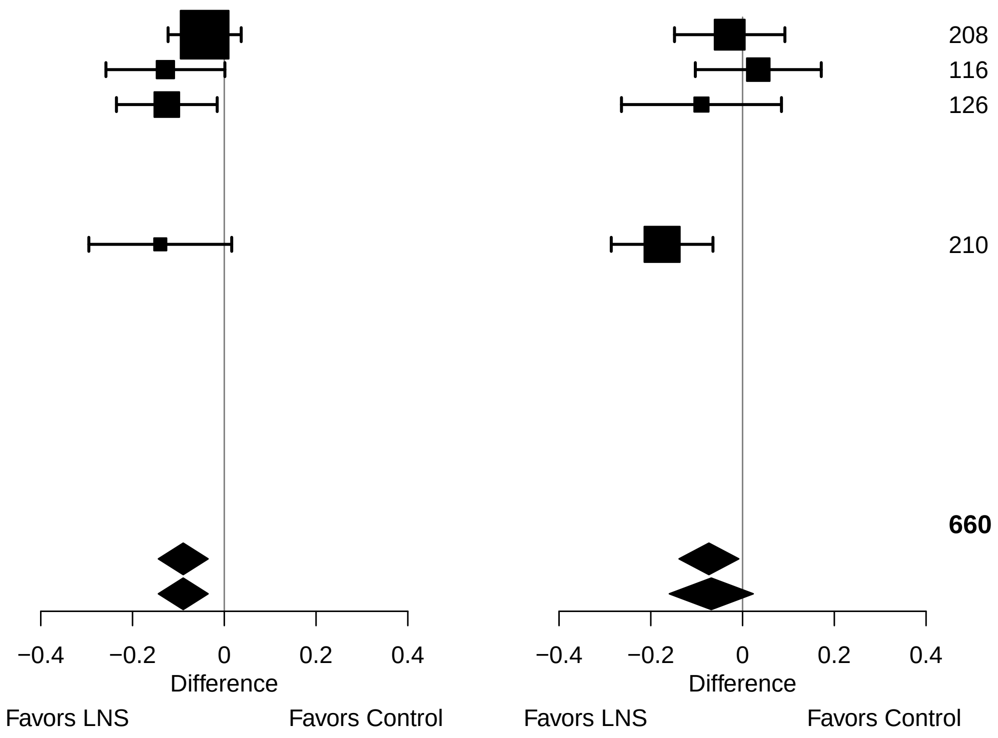

Supplement: nqab276_Supplemental_Files [file nqab276_supplemental_files.zip › 13_ipdb_suppfig9_20210707.pdf]
