# Supplementary material for: Characteristics that modify the effect of small-quantity lipid-based nutrient supplementation on child anemia and micronutrient status: an individual participant data meta-analysis of randomized controlled trials
Source: Am J Clin Nutr. 2021 Sep 29;114(Suppl 1):68S–94S. doi: 10.1093/ajcn/nqab276 (PMC8560313; doi:10.1093/ajcn/nqab276)

Supplemental figure 10: Sensitivity analyses of effect modification of SQ-LNS on biochemical outcomes by individual-level effect modifiers

Contents

Supplemental figure 10A: Difference in mean differences in hemoglobin concentration **5**
10A1: By maternal effect modifiers 5
10A2: By child effect modifiers 6
10A3: By household effect modifiers 7

Supplemental figure 10B: Ratio of anemia prevalence ratios **8**
10B1: By maternal effect modifiers 8
10B2: By child effect modifiers 9
10B3: By household effect modifiers 10

Supplemental figure 10C: Difference in anemia prevalence differences **11**
10C1: By maternal effect modifiers 11
10C2: By child effect modifiers 12
10C3: By household effect modifiers 13

Supplemental figure 10D: Ratio of moderate-to-severe anemia prevalence ratios **14**
10D1: By maternal effect modifiers 14
10D2: By child effect modifiers 15
10D3: By household effect modifiers 16

Supplemental figure 10E: Difference in moderate-to-severe anemia prevalence differences **17**
10E1: By maternal effect modifiers 17
10E2: By child effect modifiers 18
10E3: By household effect modifiers 19

Supplemental figure 10F: Ratio of geometric mean ratios of ferritin concentration **20**
10F1: By maternal effect modifiers 20
10F2: By child effect modifiers 21
10F3: By household effect modifiers 22

Supplemental figure 10G: Ratio of iron deficiency (ferritin < 12 µg/L) prevalence ratios **23**

10G1: By maternal effect modifiers . . . 23
10G2: By child effect modifiers . . . 24
10G3: By household effect modifiers . . . 25

Supplemental figure 10H: Difference in iron deficiency (ferritin < 12 µg/L) prevalence differences **26**
10H1: By maternal effect modifiers . . . 26
10H2: By child effect modifiers . . . 27
10H3: By household effect modifiers . . . 28

Supplemental figure 10I: Ratio of iron deficiency anemia prevalence ratios **29**
10I1: By maternal effect modifiers . . . 29
10I2: By child effect modifiers . . . 30
10I3: By household effect modifiers . . . 31

Supplemental figure 10J: Difference in iron deficiency anemia prevalence differences **32**
10J1: By maternal effect modifiers . . . 32
10J2: By child effect modifiers . . . 33
10J3: By household effect modifiers . . . 34

Supplemental figure 10K: Ratio of geometric mean ratios of soluble transferrin receptor concentration **35**
10K1: By maternal effect modifiers . . . 35
10K2: By child effect modifiers . . . 36
10K3: By household effect modifiers . . . 37

Supplemental figure 10L: Ratio of elevated soluble transferrin receptor prevalence ratios **38**
10L1: By maternal effect modifiers . . . 38
10L2: By child effect modifiers . . . 39
10L3: By household effect modifiers . . . 40

Supplemental figure 10M: Difference in elevated soluble transferrin receptor prevalence differences **41**
10M1: By maternal effect modifiers . . . 41
10M2: By child effect modifiers . . . 42
10M3: By household effect modifiers . . . 43

Supplemental figure 10N: Ratio of geometric mean ratios of zinc protoporphyrin concentration **44**
10N1: By maternal effect modifiers . . . 44
10N2: By child effect modifiers . . . 45
10N3: By household effect modifiers . . . 46

Supplemental figure 10O: Ratio of elevated zinc protoporphyrin prevalence ratios **47**
10O1: By maternal effect modifiers . . . 47
10O2: By child effect modifiers . . . 48
10O3: By household effect modifiers . . . 49

Supplemental figure 10P: Difference in elevated zinc protoporphyrin prevalence differences **50**
10P1: By maternal effect modifiers 50
10P2: By child effect modifiers 51
10P3: By household effect modifiers 52

Supplemental figure 10Q: Ratio of geometric mean ratios of plasma zinc concentration **53**
10Q1: By maternal effect modifiers 53
10Q2: By child effect modifiers 54
10Q3: By household effect modifiers 55

Supplemental figure 10R: Ratio of geometric mean ratios of retinol concentration **56**
10R1: By maternal effect modifiers 56
10R2: By child effect modifiers 57
10R3: By household effect modifiers 58

Supplemental figure 10S: Ratio of low vitamin A (retinol < 0.70 µmol/L) prevalence ratios **59**
10S1: By maternal effect modifiers (insufficient comparisons) 59
10S2: By child effect modifiers (insufficient comparisons) 60
10S3: By household effect modifiers (insufficient comparisons) 61

Supplemental figure 10T: Difference in low vitamin A (retinol < 0.70 µmol/L) prevalence differences **62**
10T1: By maternal effect modifiers (insufficient comparisons) 62
10T2: By child effect modifiers (insufficient comparisons) 63
10T3: By household effect modifiers (insufficient comparisons) 64

Supplemental figure 10U: Ratio of marginal vitamin A (retinol < 1.05 µmol/L) prevalence ratios **65**
10U1: By maternal effect modifiers 65
10U2: By child effect modifiers 66
10U3: By household effect modifiers 67

Supplemental figure 10V: Difference in marginal vitamin A (retinol < 1.05 µmol/L) prevalence differences **68**
10V1: By maternal effect modifiers 68
10V2: By child effect modifiers 69
10V3: By household effect modifiers 70

Supplemental figure 10W: Ratio of geometric mean ratio of retinol binding protein concentrations **71**
10W1: By maternal effect modifiers 71
10W2: By child effect modifiers 72
10W3: By household effect modifiers 73

Supplemental figure 10X: Ratio of Low vitamin A status (RBP < 0.70 µmol/L) prevalence ratios **74**
10X1: By maternal effect modifiers (insufficient comparisons) 74
10X2: By child effect modifiers (insufficient comparisons) 75

10X3: By household effect modifiers (insufficient comparisons) . . . 76

Supplemental figure 10Y: Difference in low vitamin A status (RBP < 0.70 µmol/L) prevalence differences 77
10Y1: By maternal effect modifiers (insufficient comparisons) . . . 77
10Y2: By child effect modifiers (insufficient comparisons) . . . 78
10Y3: By household effect modifiers (insufficient comparisons) . . . 79

Supplemental figure 10Z: Ratio of marginal vitamin A status (RBP < 1.05 µmol/L) prevalence ratios 80
10Z1: By maternal effect modifiers . . . 80
10Z2: By child effect modifiers . . . 81
10Z3: By household effect modifiers . . . 82

Supplemental figure 10AA: Difference in marginal vitamin A status (RBP < 1.05 µmol/L) prevalence differences 83
10AA1: By maternal effect modifiers . . . 83
10AA2: By child effect modifiers . . . 84
10AA3: By household effect modifiers . . . 85

These figures show the pooled estimates of effect modification by different sensitivity analyses. For continuous outcomes, the intervention effect is measured by the difference in mean of the LNS group minus control. For log transformed continuous outcomes, the intervention effect is measured by the ratio of geometric means, the effect estimate is the geometric mean in the LNS group divided by the geometric mean in the control group. For dichotomous outcomes analyzed via prevalence ratios, the effect estimate is the prevalence in the LNS group divided by the prevalence in the control group. For dichotomous outcomes analyzed via prevalence differences, the effect estimate is the prevalence in the LNS group minus the prevalence in the control group.

The labels on the left y-axis indicate which outcome is assessed. The different columns correspond to sensitivity analyses in which intervention group categorization differs. All-trial analysis includes all trials; Child-LNS-only excludes trial arms that provided both maternal and child LNS; Multi-component analysis separates comparisons within trials that included multi-component interventions, so that the SQ-LNS vs. no SQ-LNS comparisons were conducted separately between pairs of arms that included the same non-nutrition components (e.g. SQ-LNS+WASH vs. WASH; SQ-LNS vs. Control); Passive arms excluded analysis excludes passive control arms. Depending on the sensitivity analysis, there may not have been enough comparisons available to generate a pooled estimate.

Ferritin, sTfR, ZPP, zinc, retinol and RBP concentrations were adjusted for inflammation (i.e., C-reactive protein (CRP) and/or α-1-acid glycoprotein (AGP) concentrations, as available), using a regression correction approach adapted from the Biomarkers Reflecting Inflammation and Nutritional Determinants of Anemia (BRINDA) project (28)

sTfR, soluble transferrin receptor; ZPP, zinc protoporphyrin; RBP, retinol binding protein.

Supplemental figure 10A: Difference in mean differences in hemoglobin concentration

10A1: By maternal effect modifiers

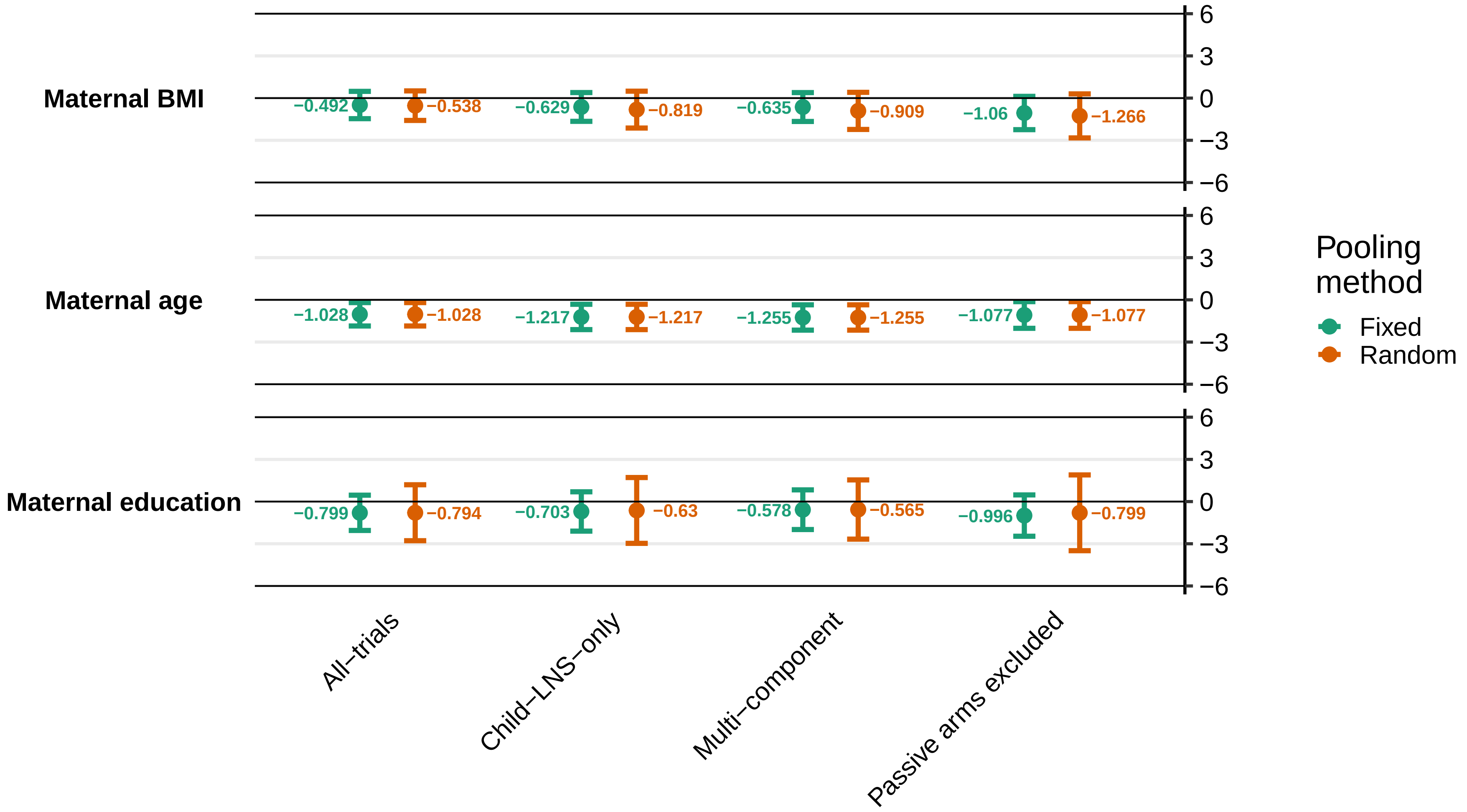

Supplemental figure 10A: Difference in mean differences in hemoglobin concentration

10A2: By child effect modifiers

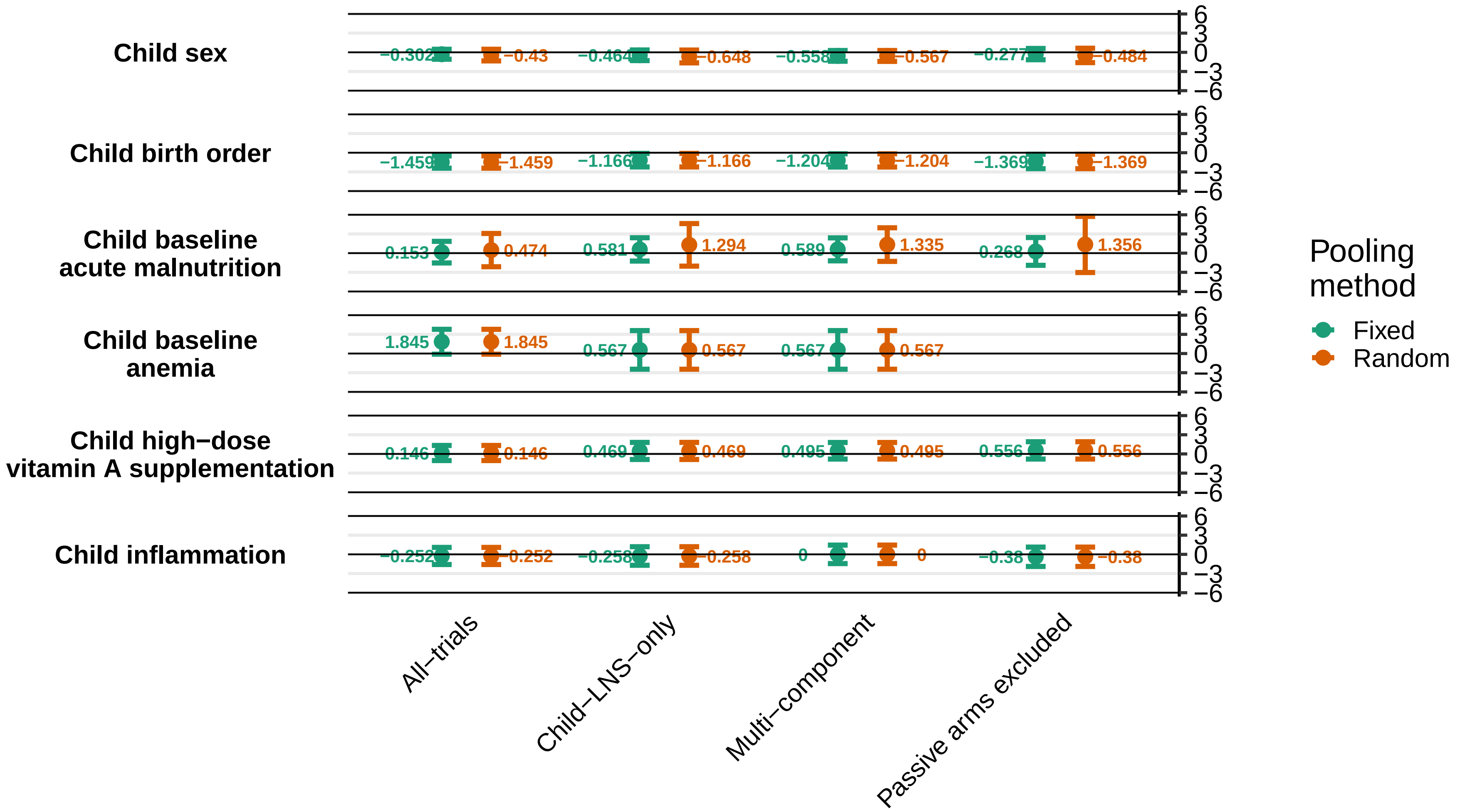

Supplemental figure 10A: Difference in mean differences in hemoglobin concentration

10A3: By household effect modifiers

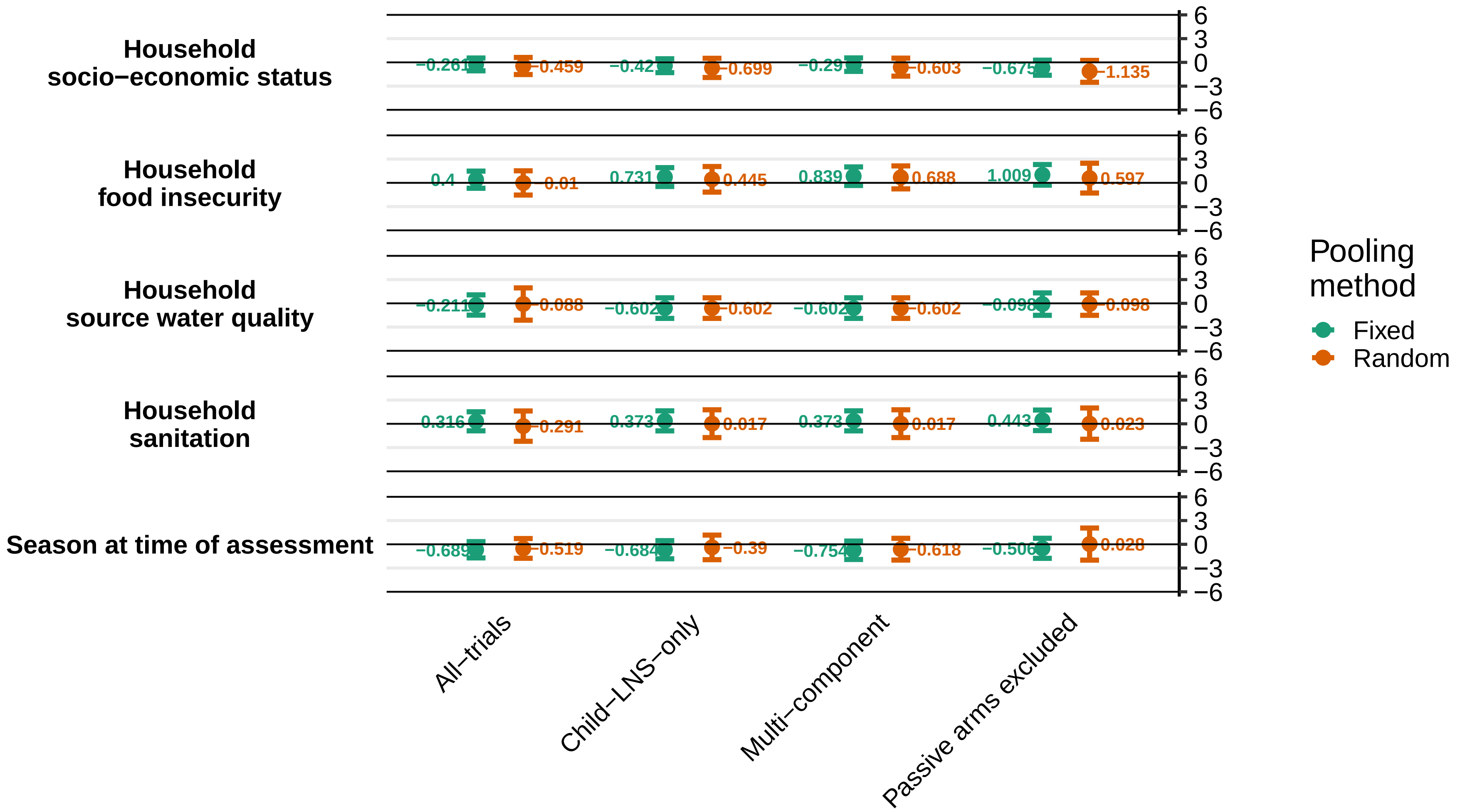

Supplemental figure 10B: Ratio of anemia prevalence ratios

10B1: By maternal effect modifiers

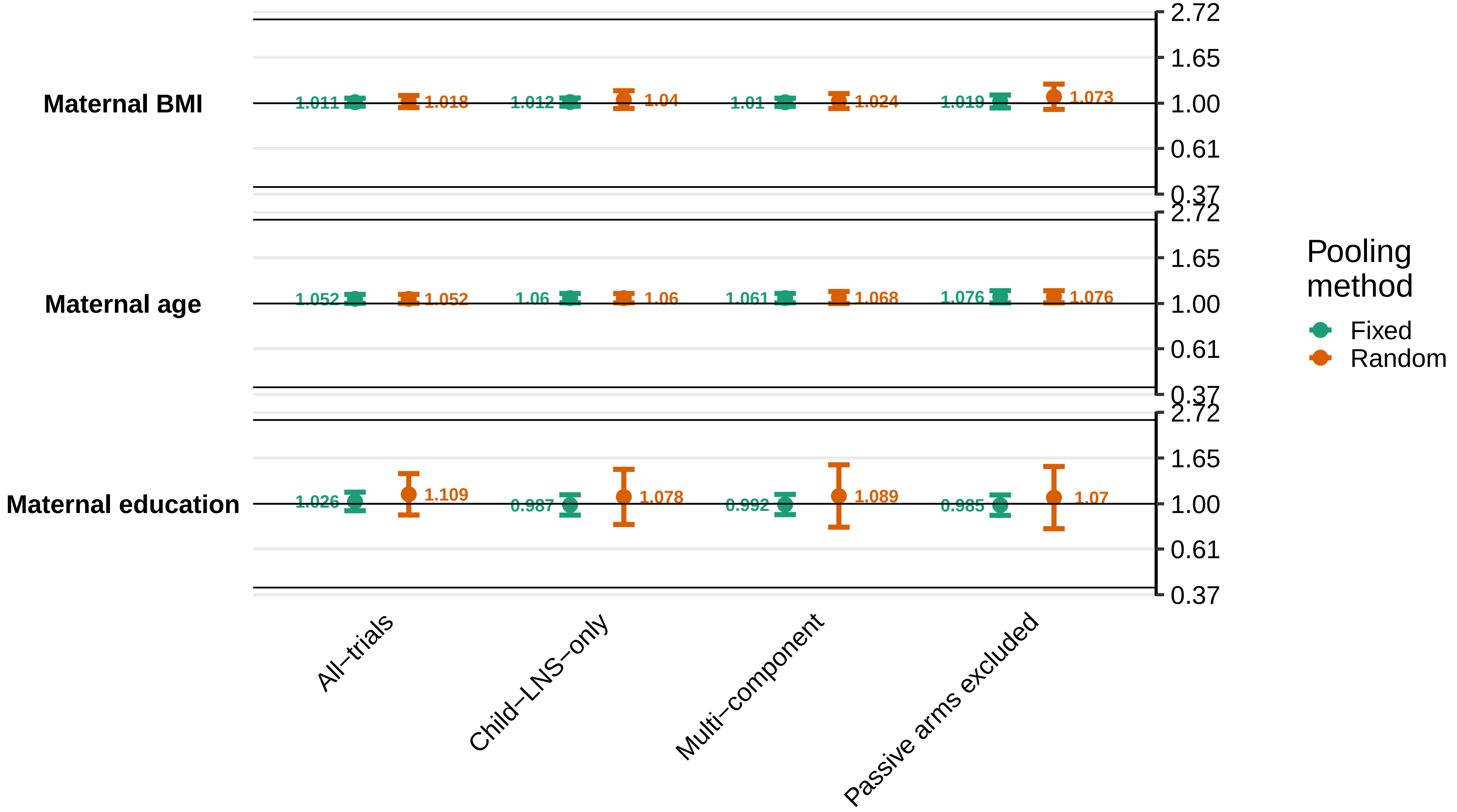

Supplemental figure 10B: Ratio of anemia prevalence ratios

10B2: By child effect modifiers

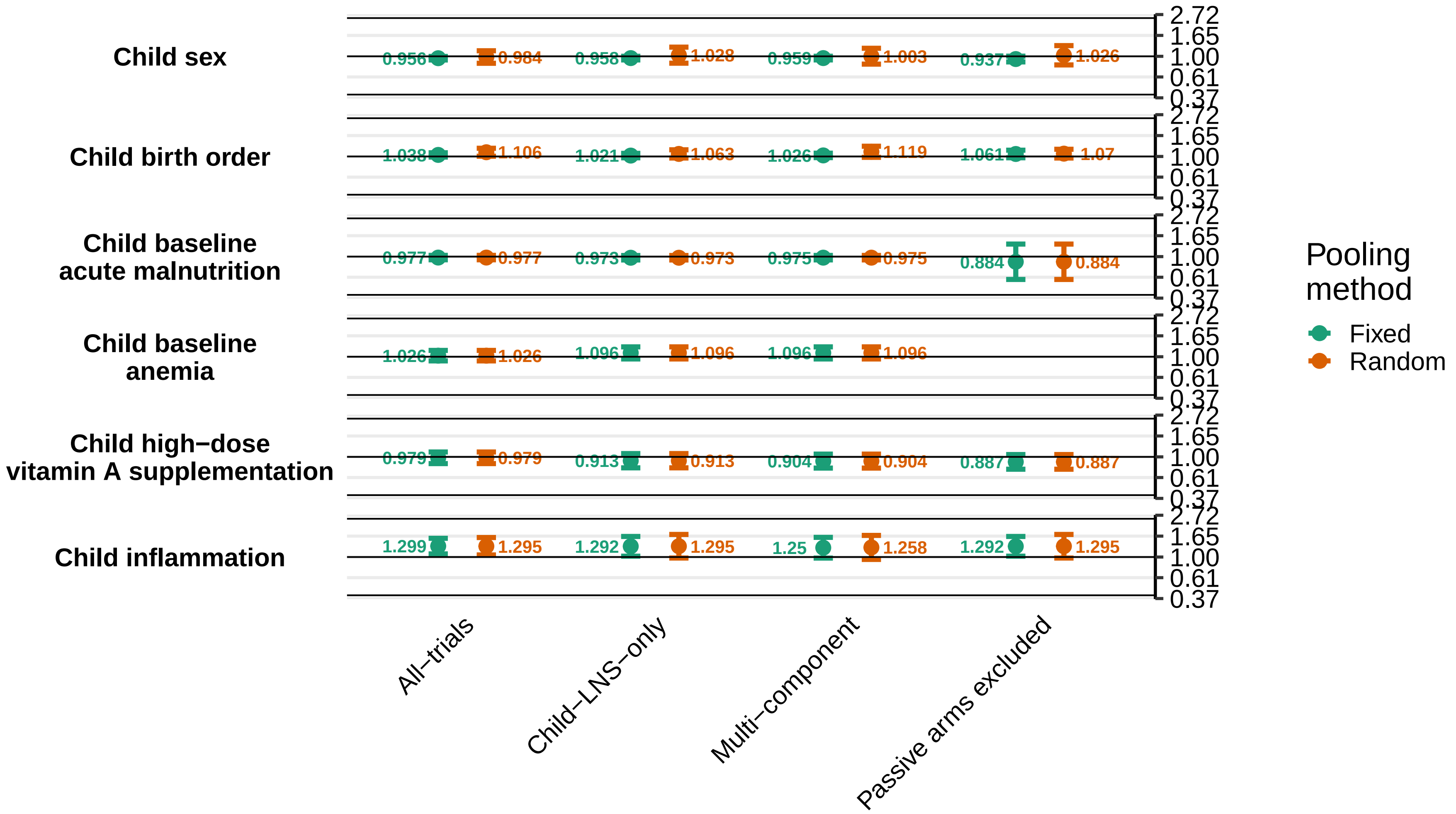

Supplemental figure 10B: Ratio of anemia prevalence ratios

10B3: By household effect modifiers

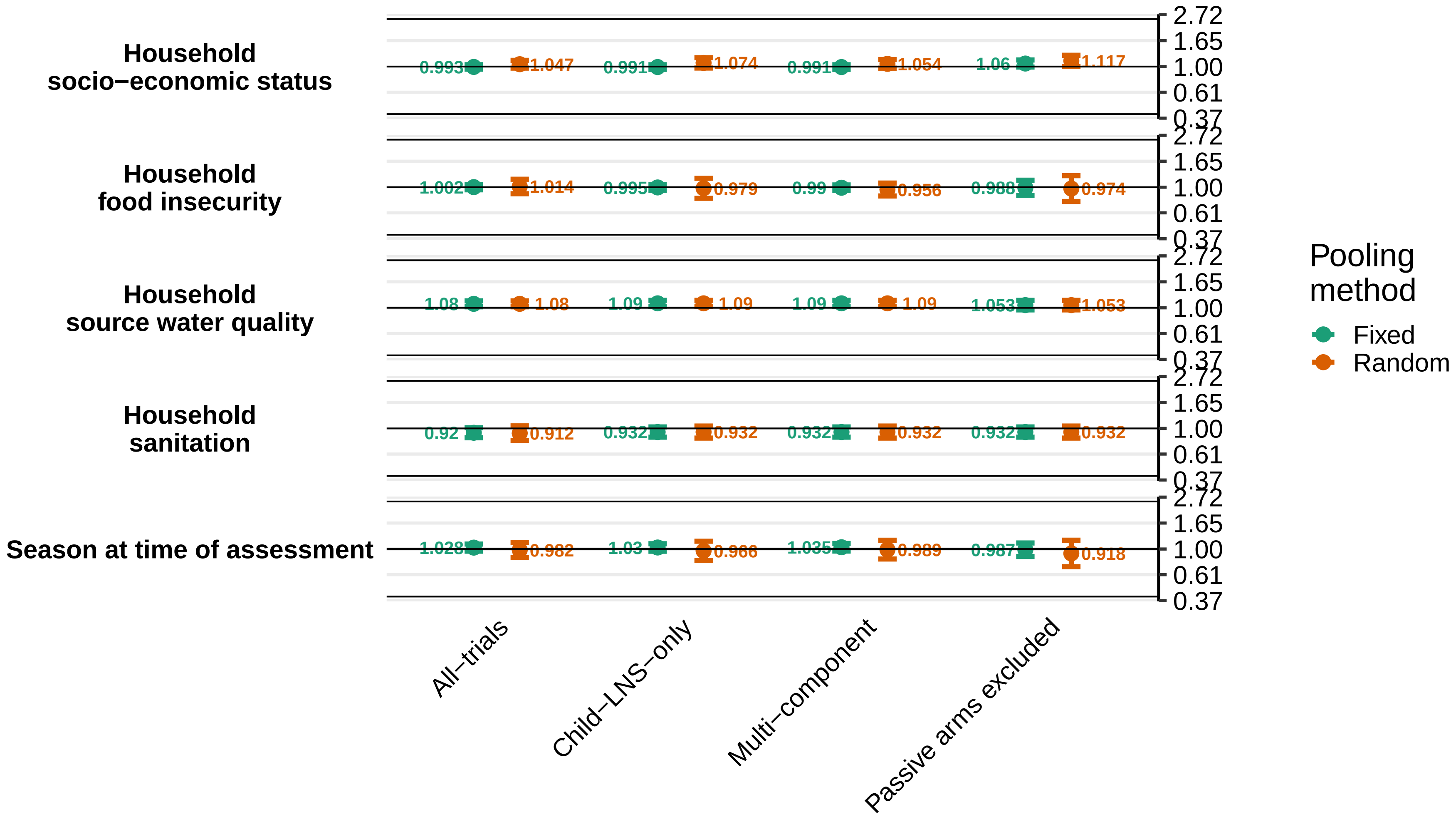

Supplemental figure 10C: Difference in anemia prevalence differences

10C1: By maternal effect modifiers

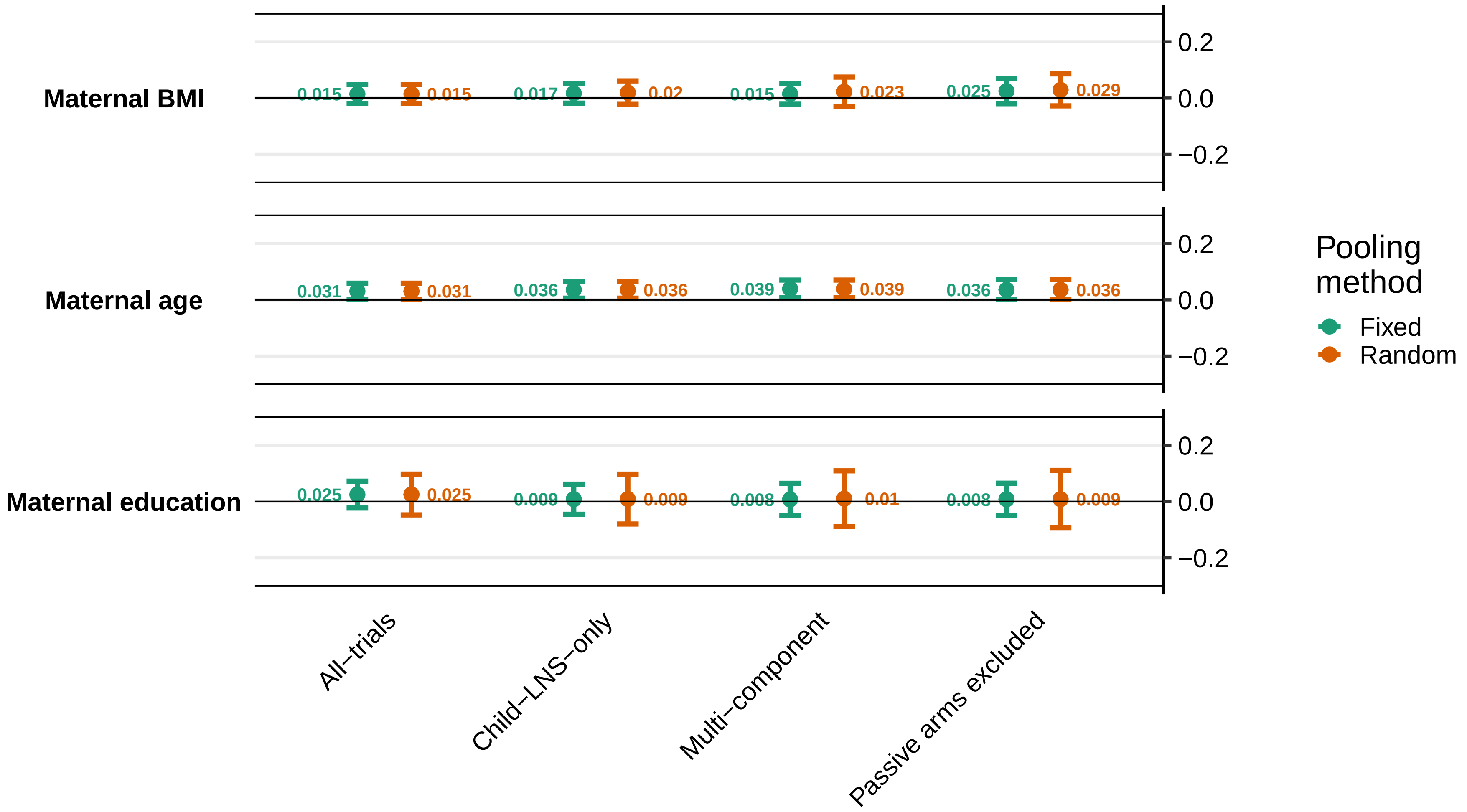

Supplemental figure 10C: Difference in anemia prevalence differences

10C2: By child effect modifiers

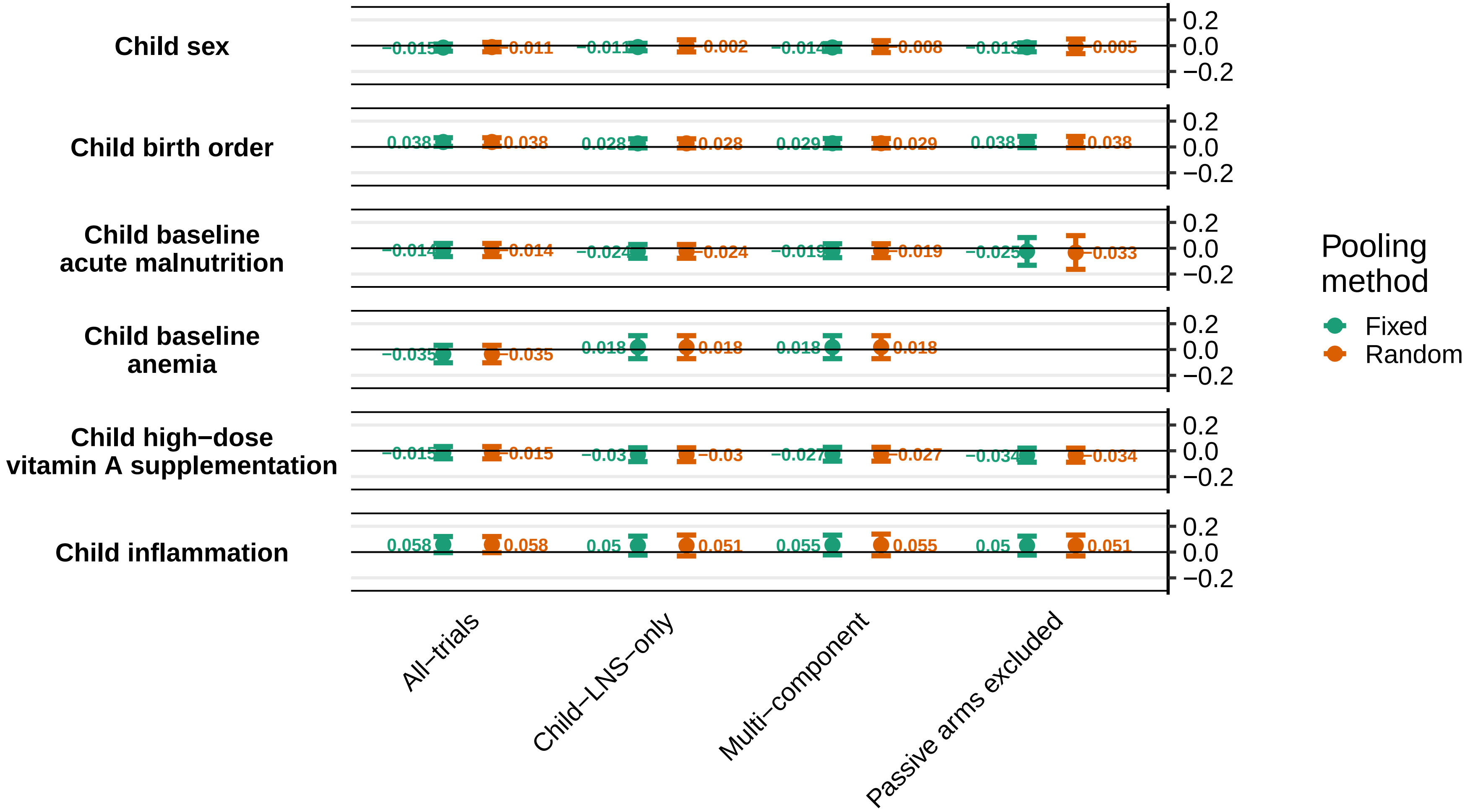

Supplemental figure 10C: Difference in anemia prevalence differences

10C3: By household effect modifiers

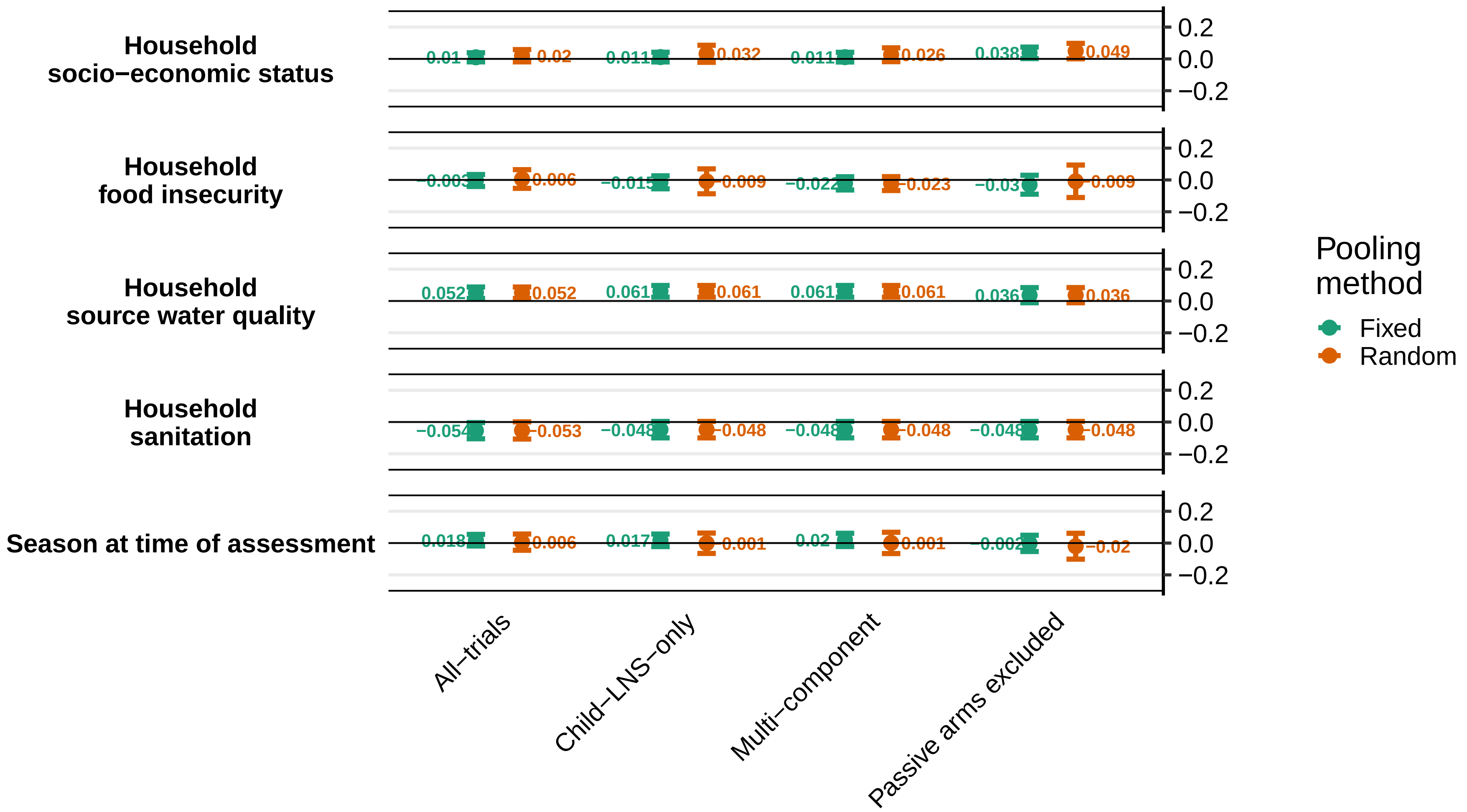

Supplemental figure 10D: Ratio of moderate-to-severe anemia prevalence ratios

10D1: By maternal effect modifiers

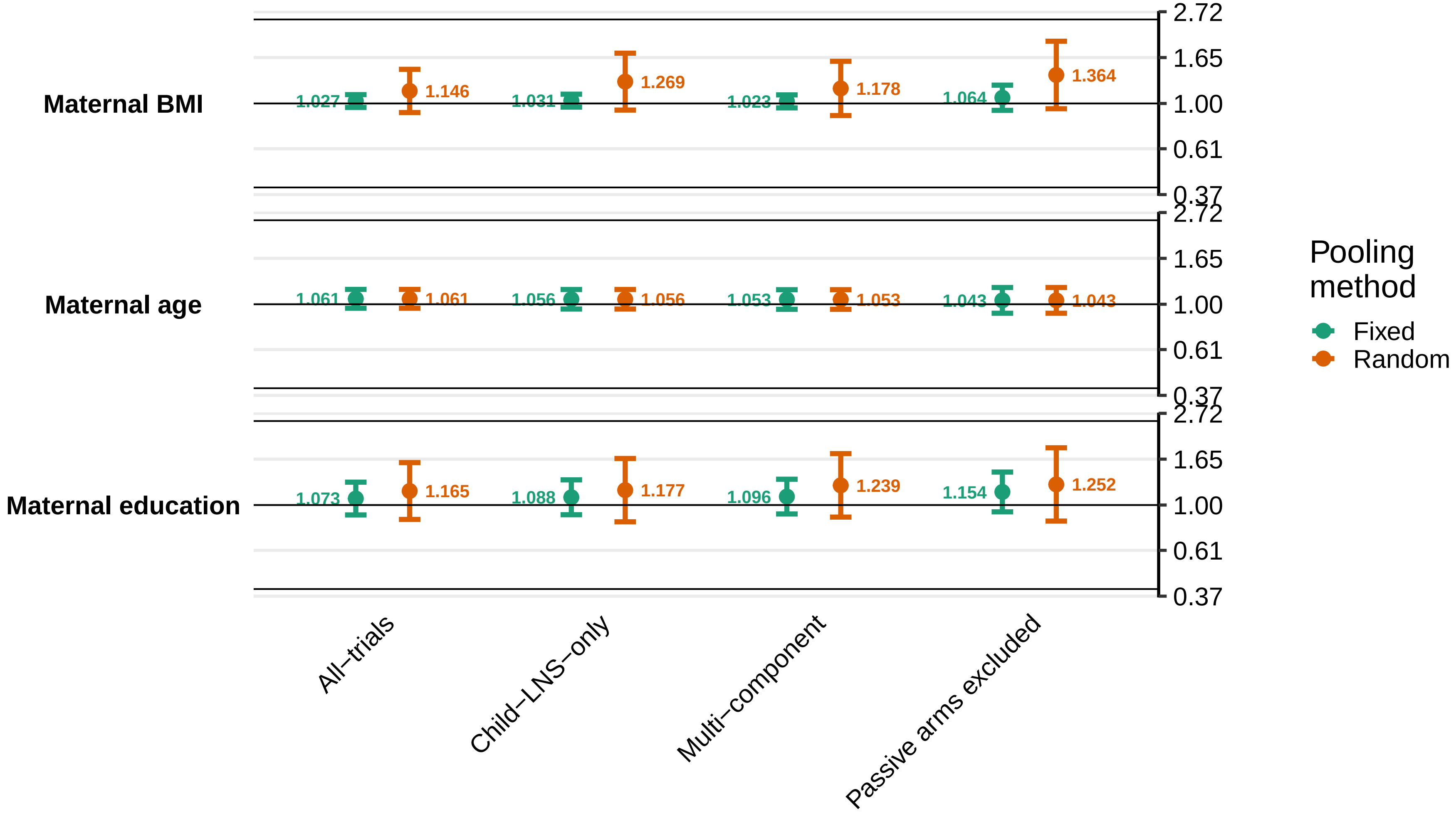

Supplemental figure 10D: Ratio of moderate-to-severe anemia prevalence ratios

10D2: By child effect modifiers

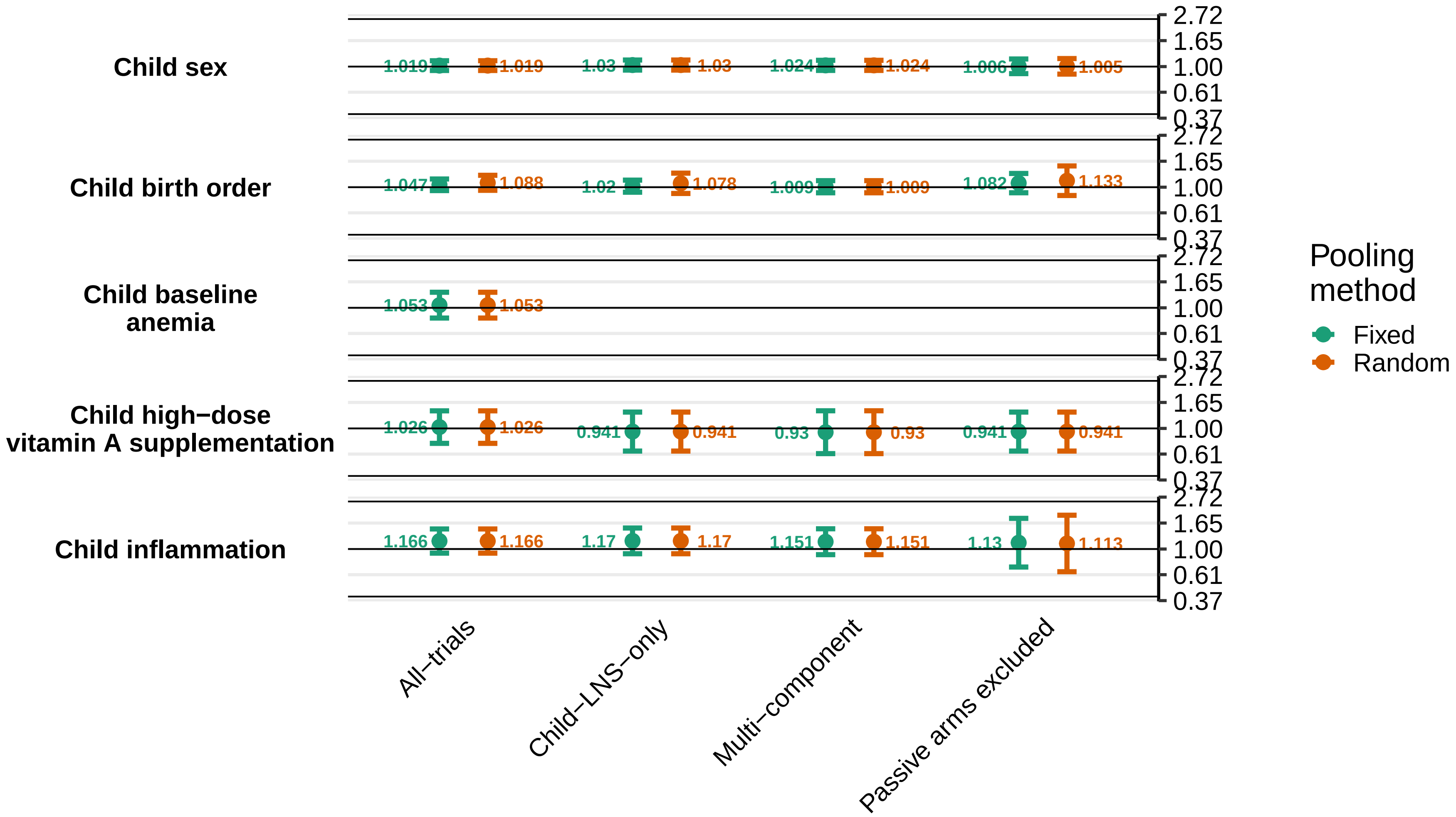

Supplemental figure 10D: Ratio of moderate-to-severe anemia prevalence ratios

10D3: By household effect modifiers

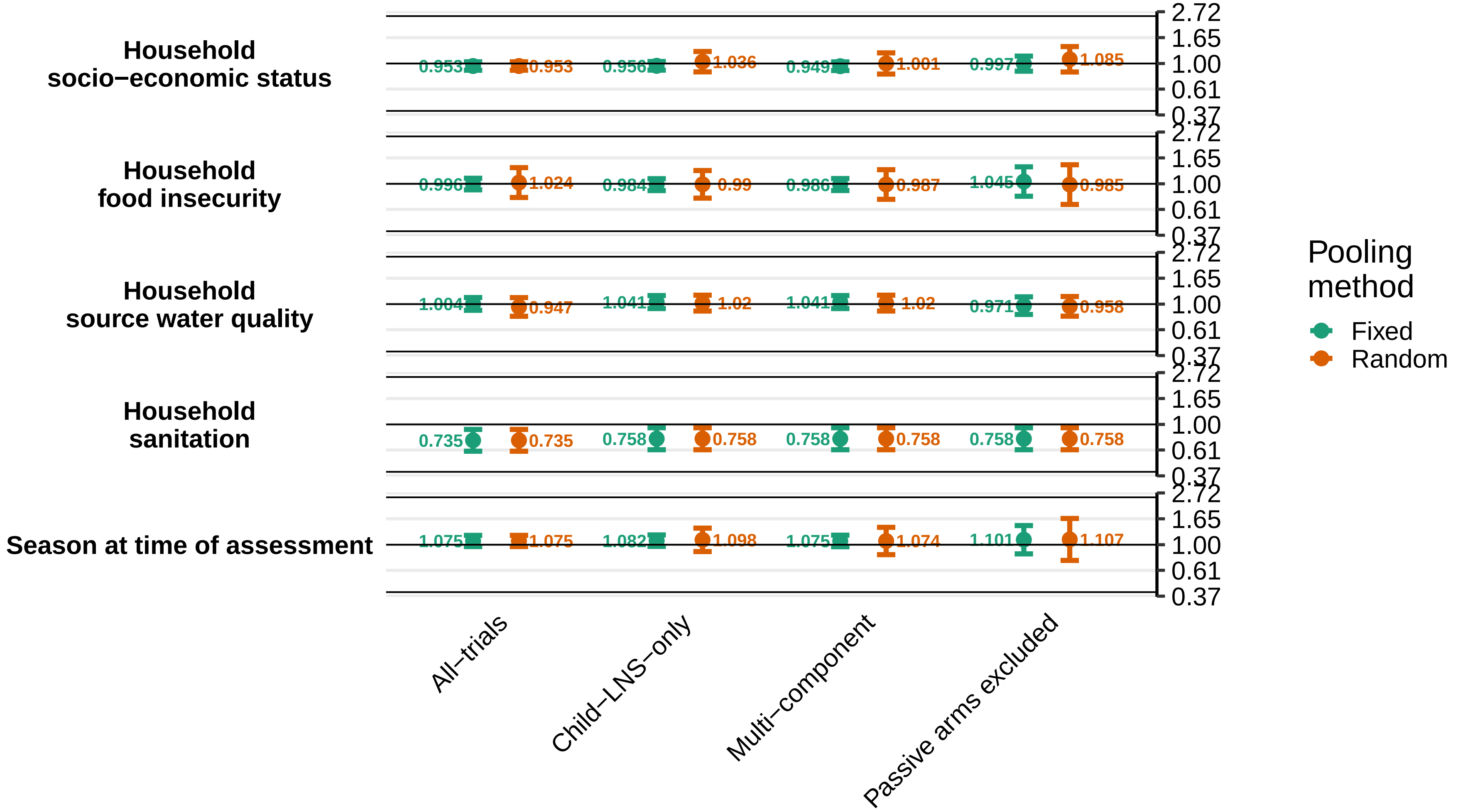

Supplemental figure 10E: Difference in moderate-to-severe anemia prevalence differences

10E1: By maternal effect modifiers

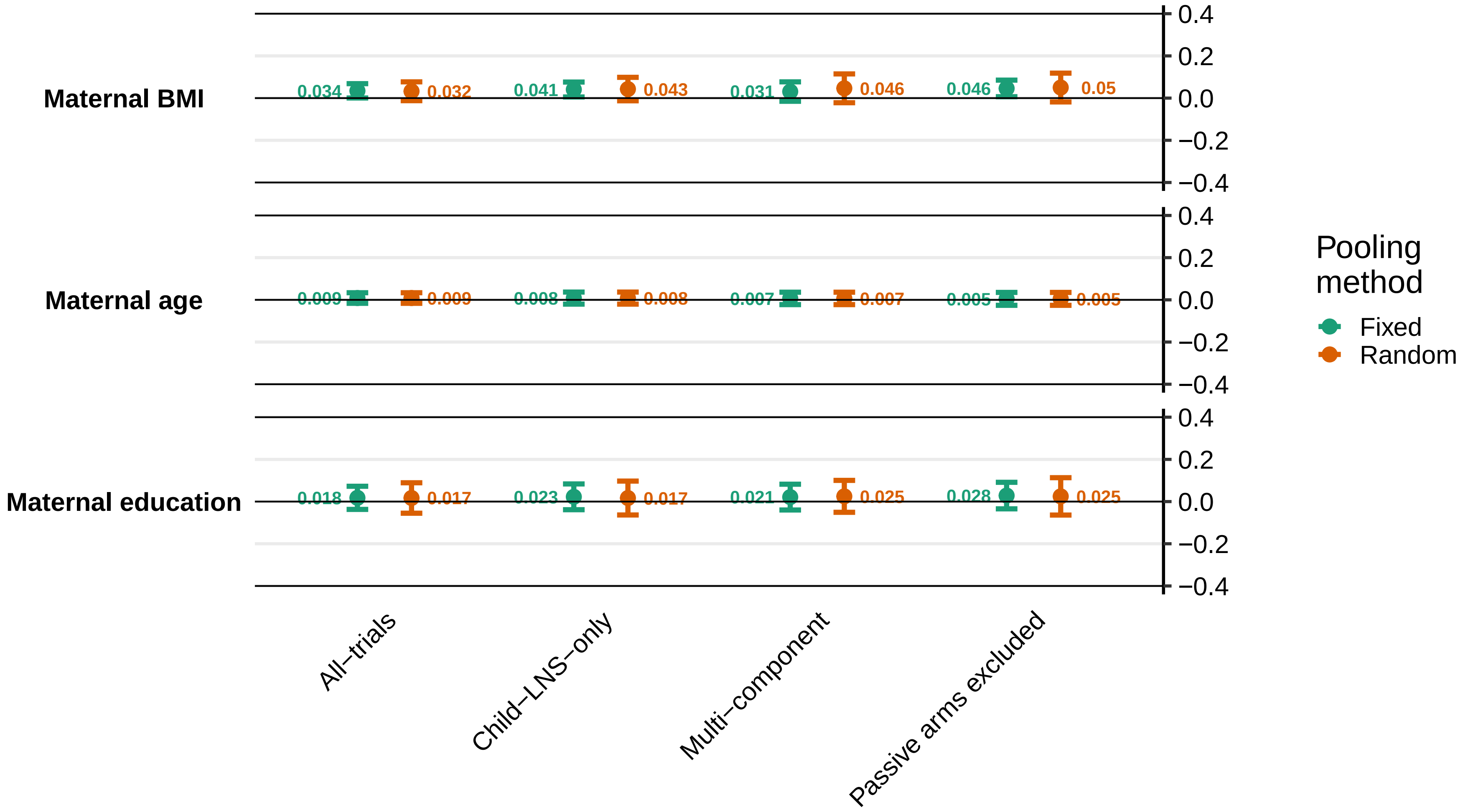

Supplemental figure 10E: Difference in moderate-to-severe anemia prevalence differences

10E2: By child effect modifiers

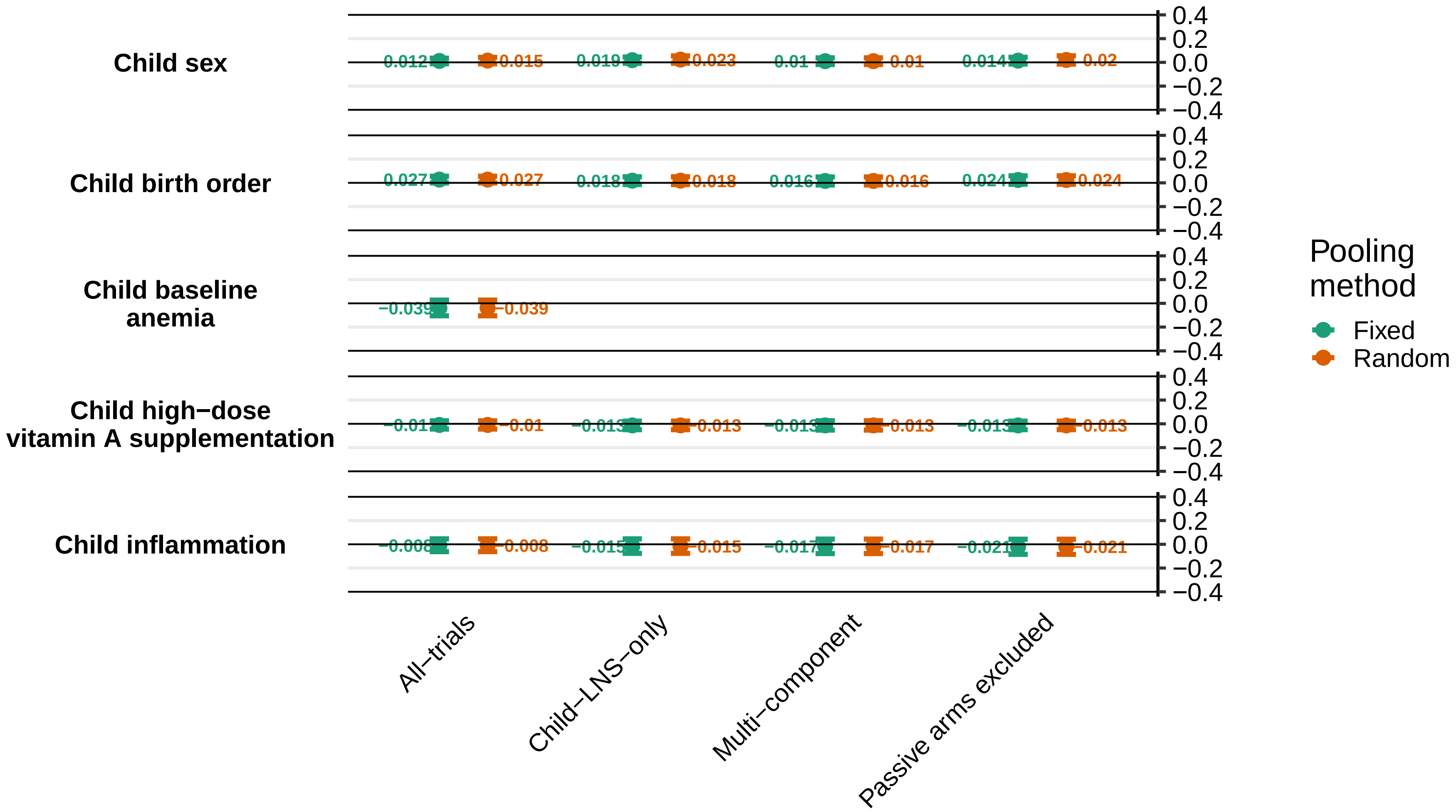

Supplemental figure 10E: Difference in moderate-to-severe anemia prevalence differences

10E3: By household effect modifiers

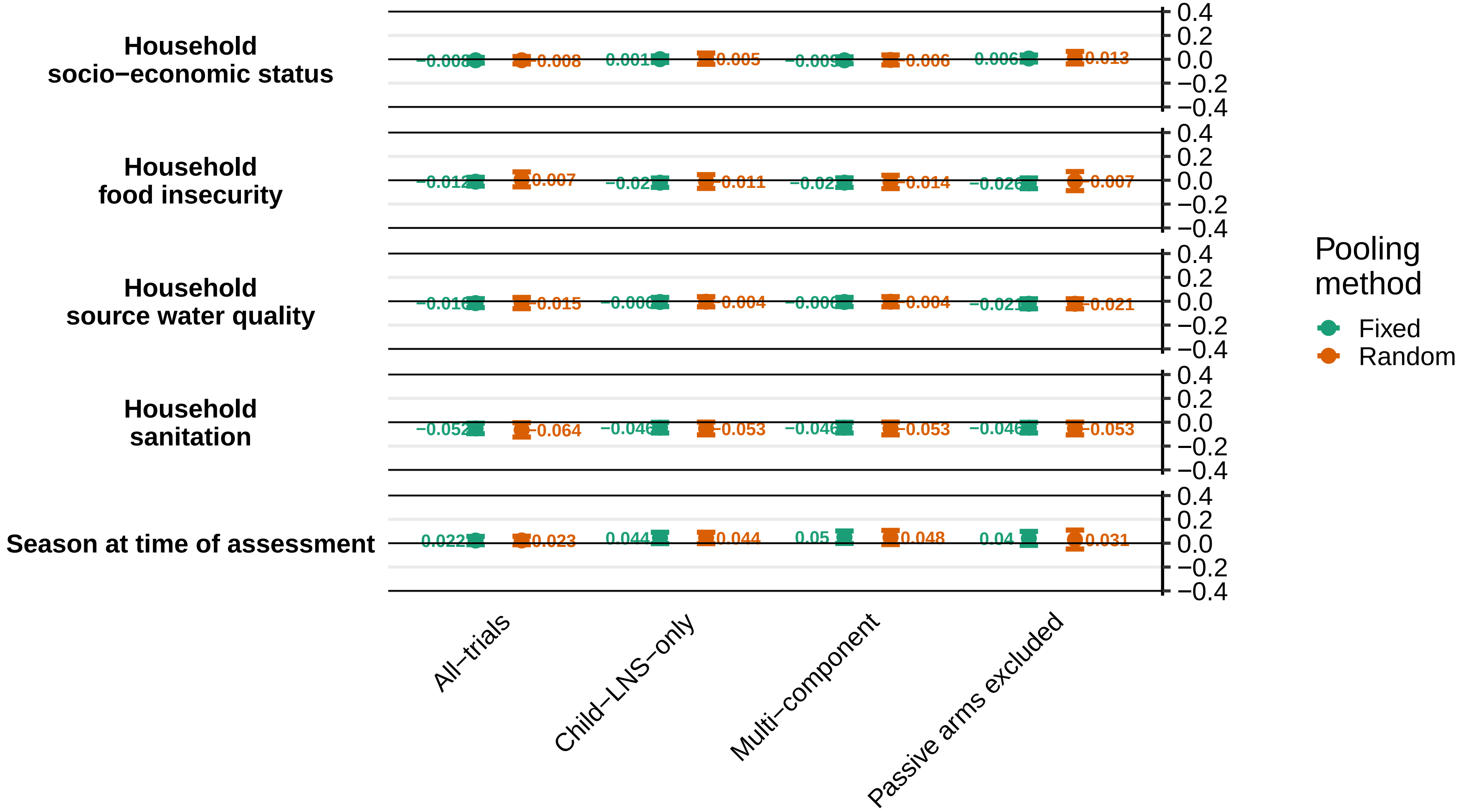

Supplemental figure 10F: Ratio of geometric mean ratios of ferritin concentration

10F1: By maternal effect modifiers

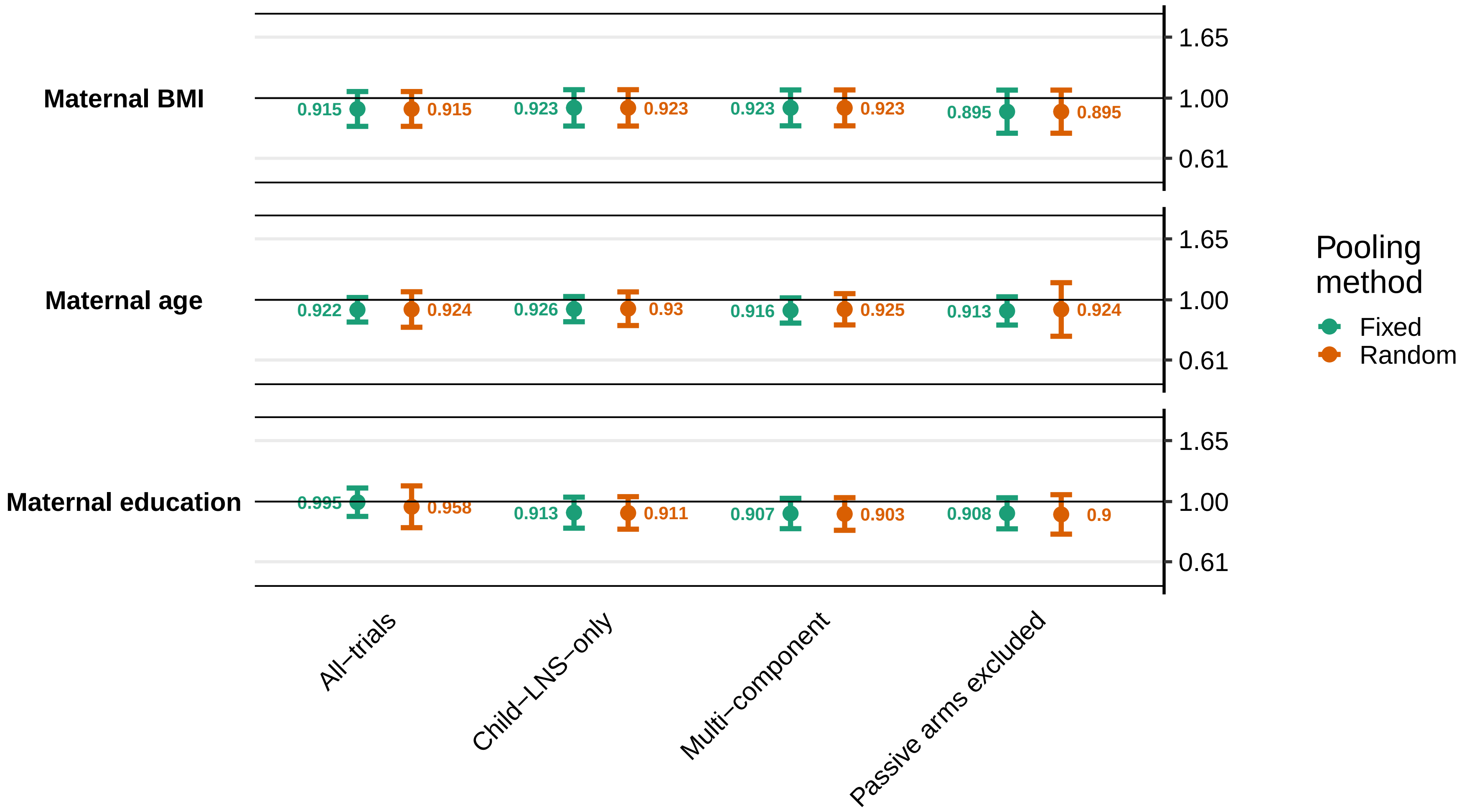

Supplemental figure 10F: Ratio of geometric mean ratios of ferritin concentration

10F2: By child effect modifiers

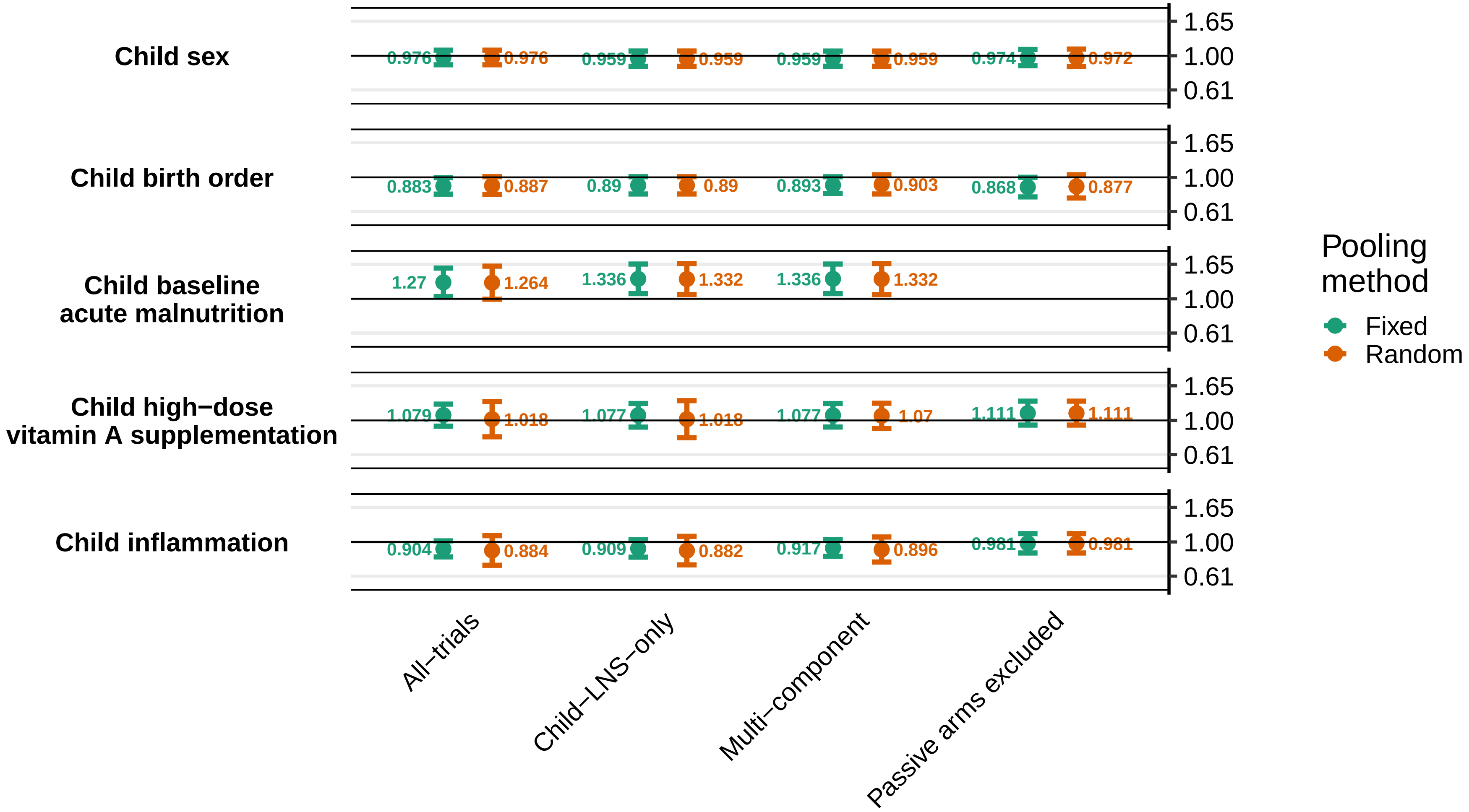

Supplemental figure 10F: Ratio of geometric mean ratios of ferritin concentration

10F3: By household effect modifiers

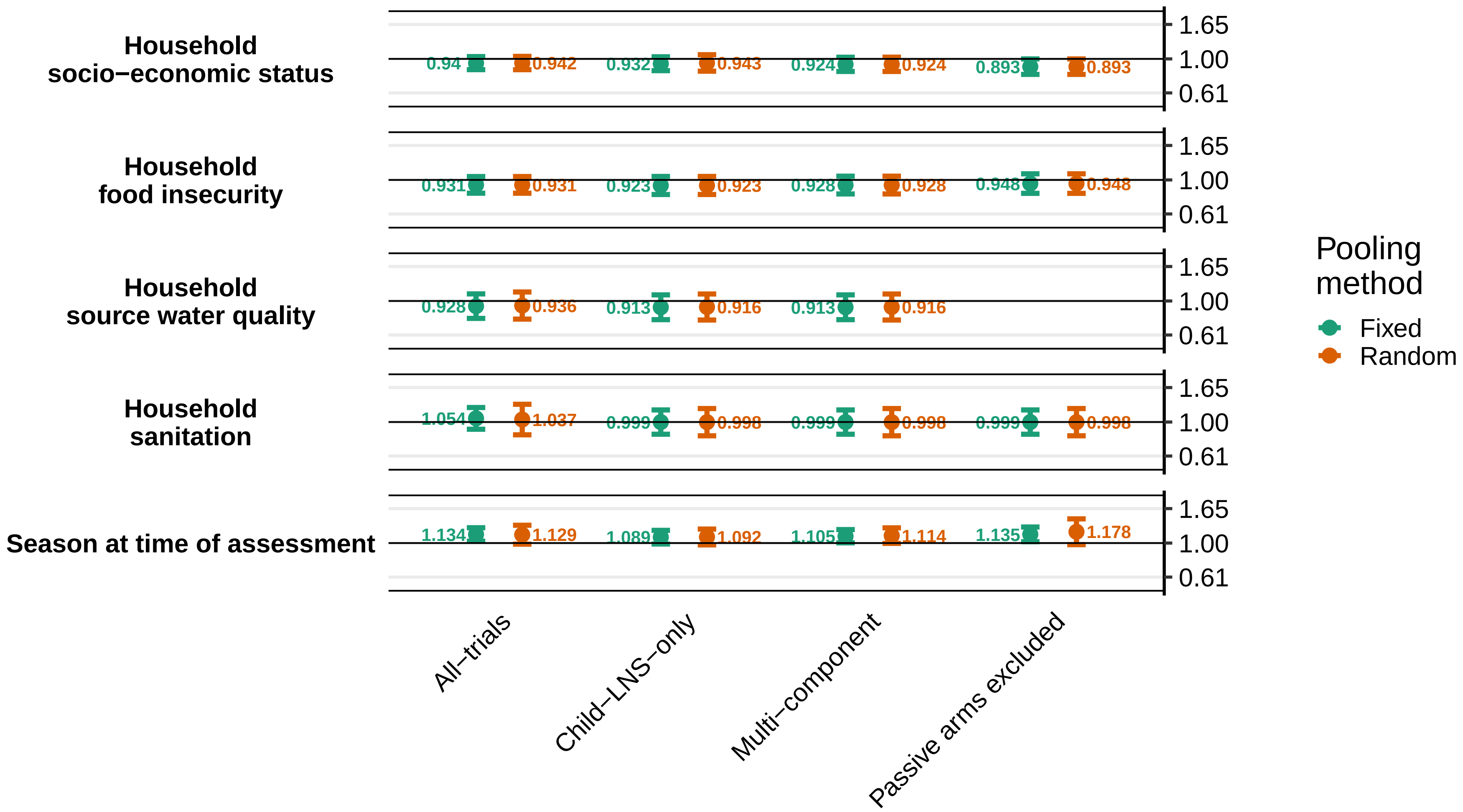

Supplemental figure 10G: Ratio of iron deficiency (ferritin < 12 µg/L) prevalence ratios

10G1: By maternal effect modifiers

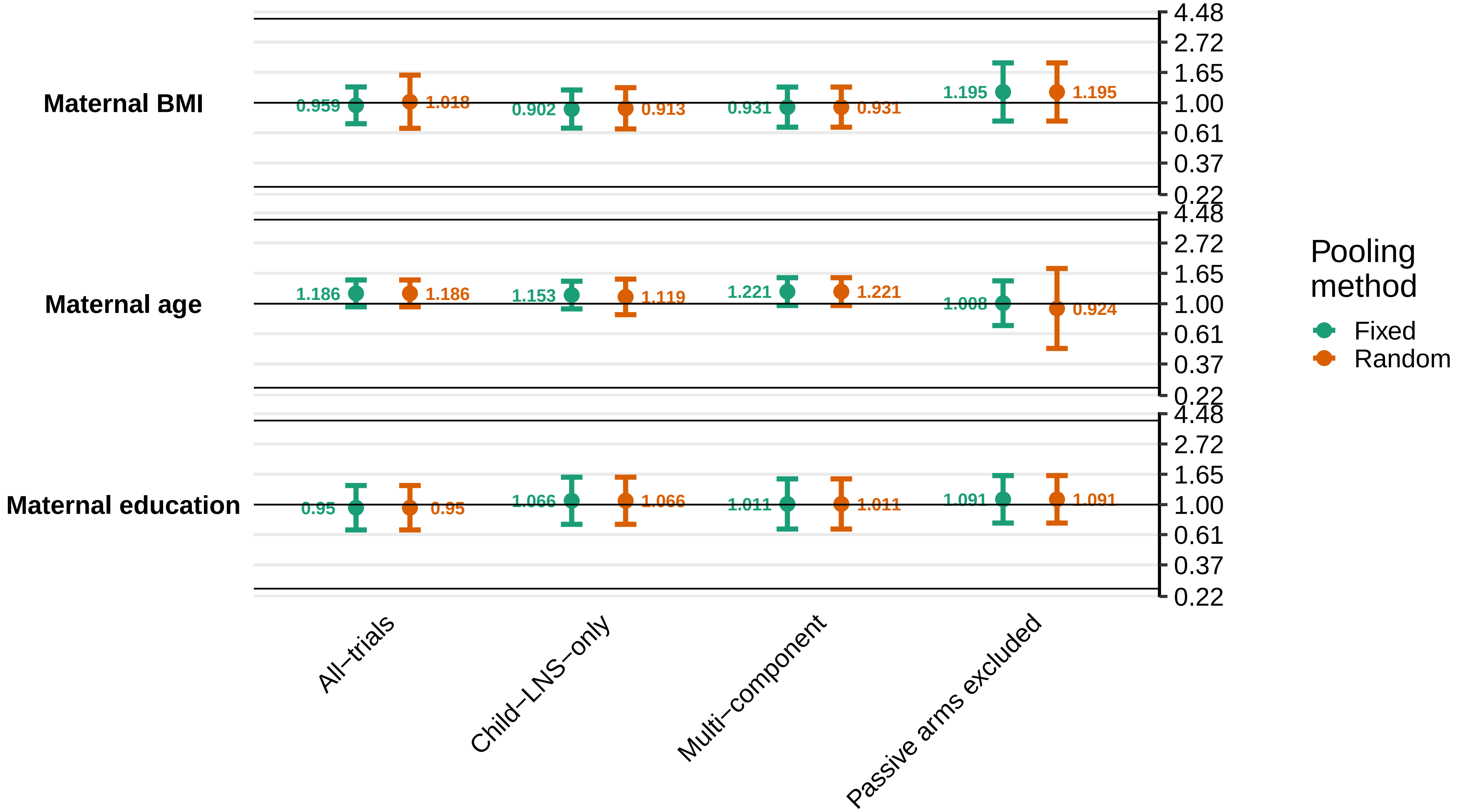

Supplemental figure 10G: Ratio of iron deficiency (ferritin < 12 µg/L) prevalence ratios

10G2: By child effect modifiers

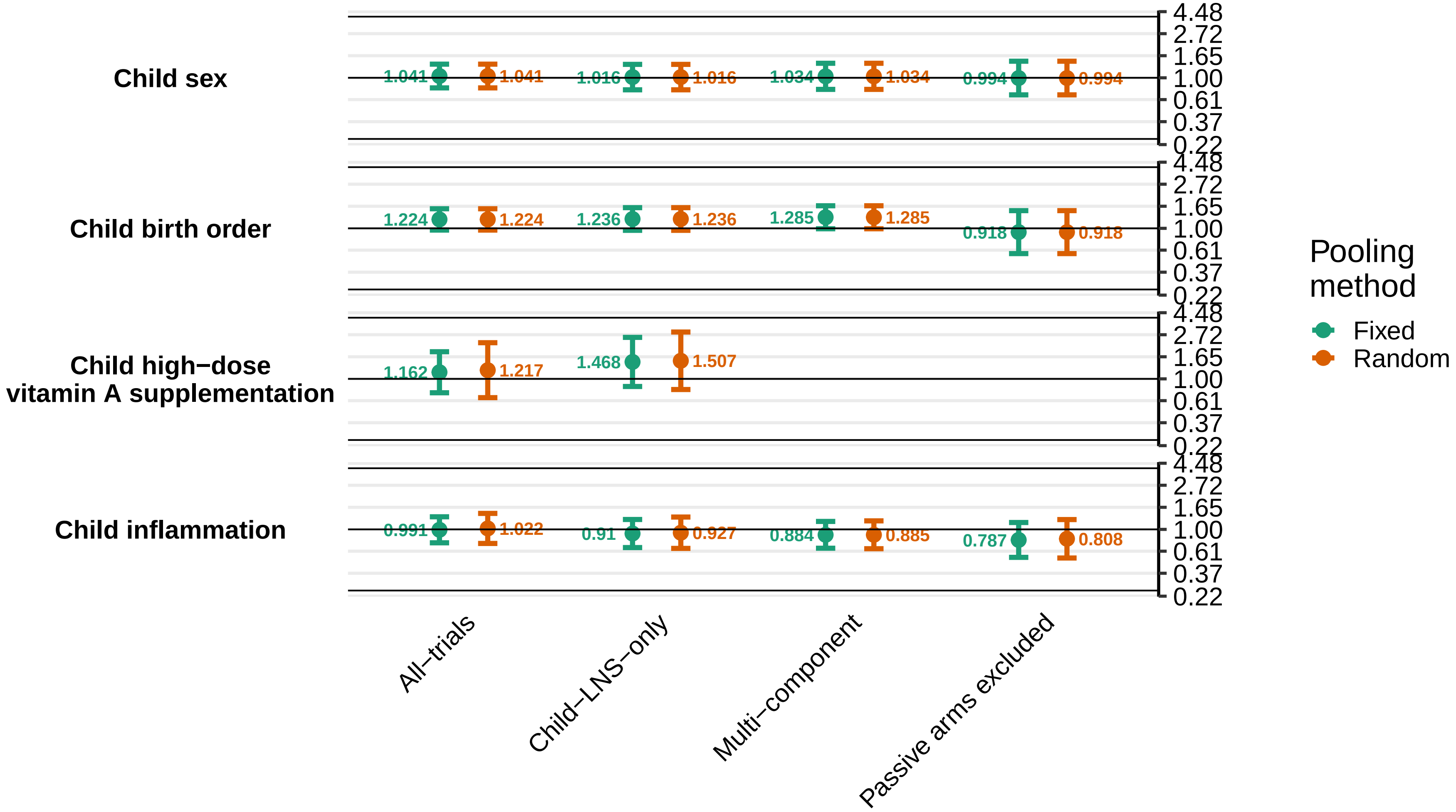

Supplemental figure 10G: Ratio of iron deficiency (ferritin < 12 µg/L) prevalence ratios

10G3: By household effect modifiers

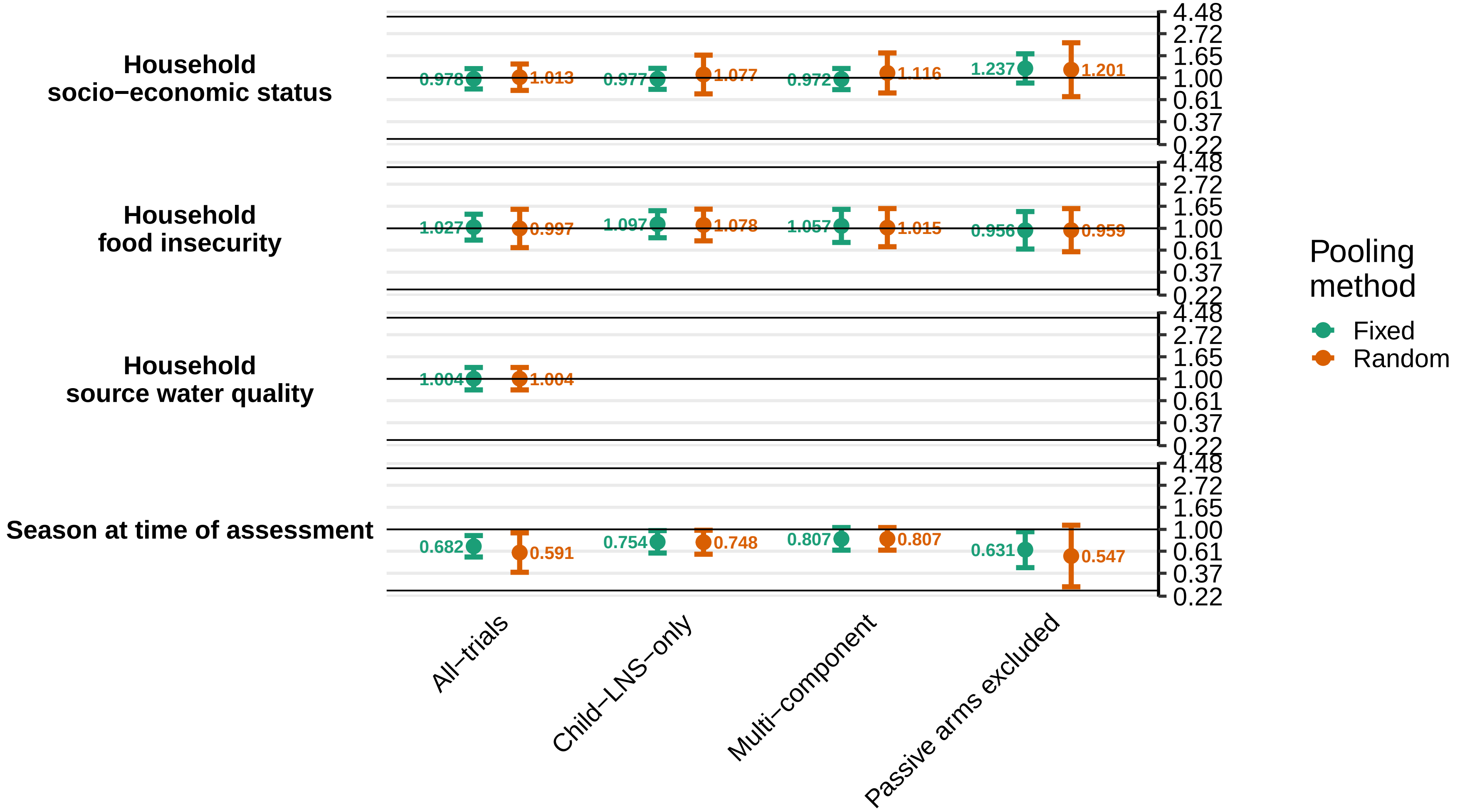

Supplemental figure 10H: Difference in iron deficiency (ferritin < 12 µg/L) prevalence differences

10H1: By maternal effect modifiers

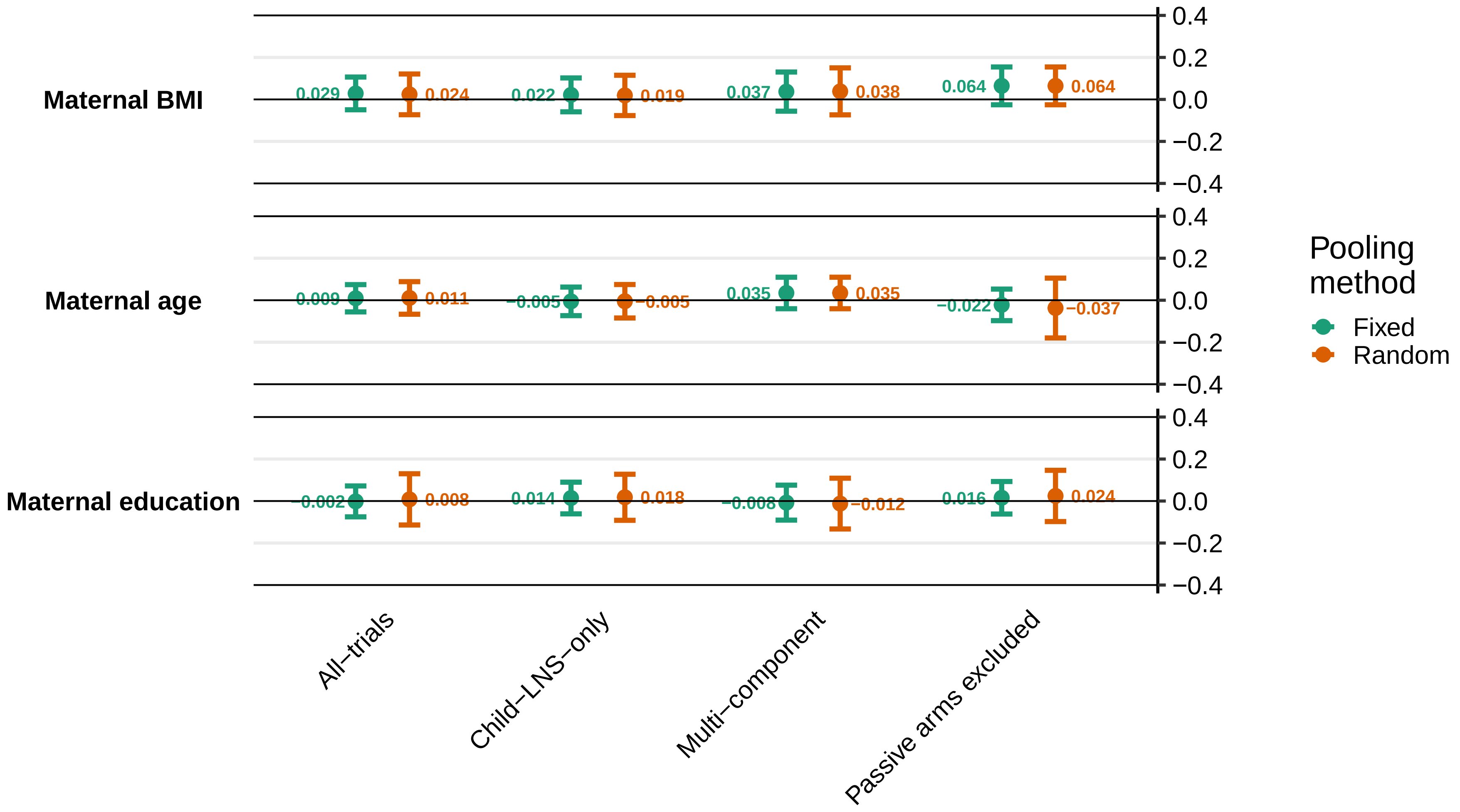

Supplemental figure 10H: Difference in iron deficiency (ferritin < 12 µg/L) prevalence differences

10H2: By child effect modifiers

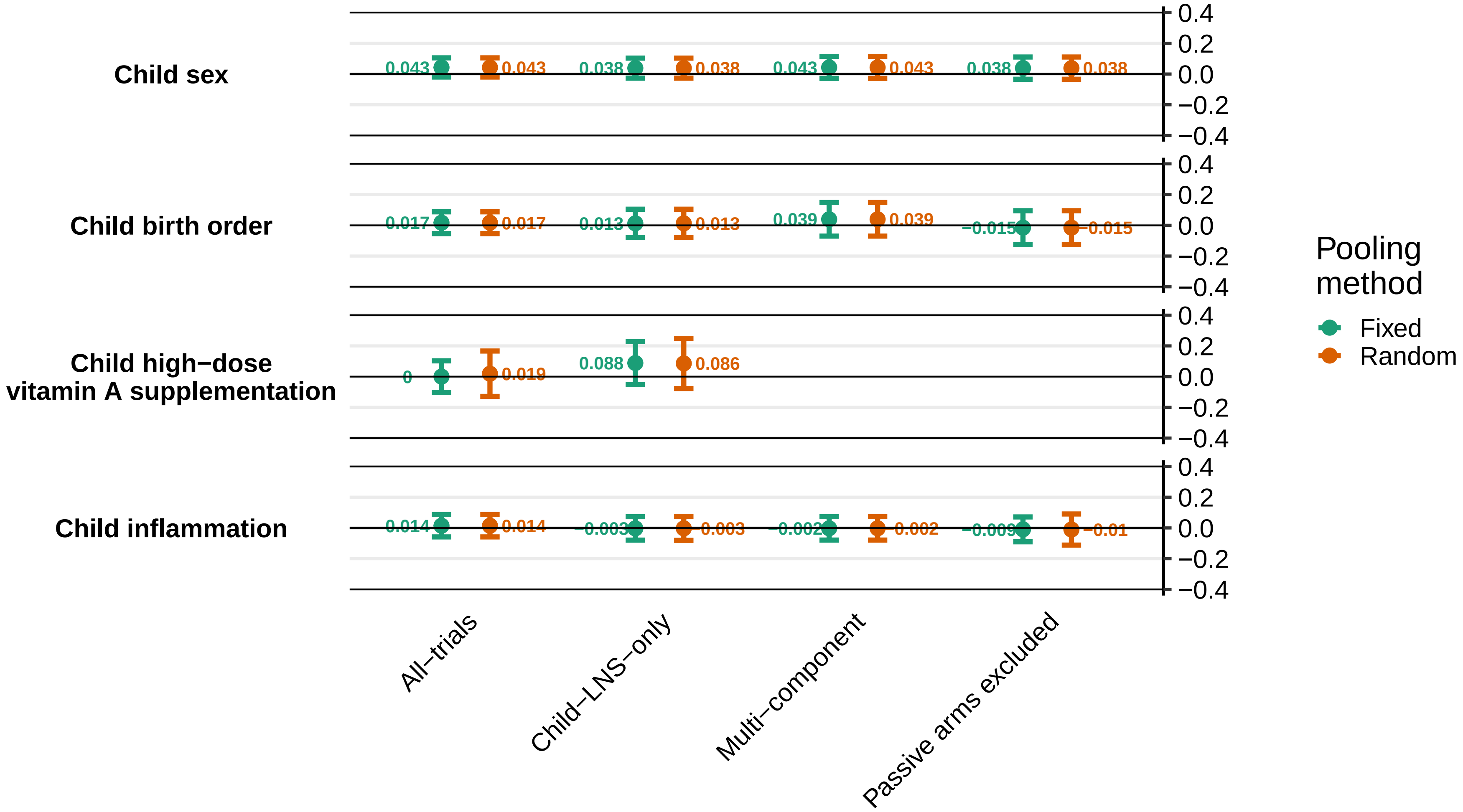

Supplemental figure 10H: Difference in iron deficiency (ferritin < 12 µg/L) prevalence differences

10H3: By household effect modifiers

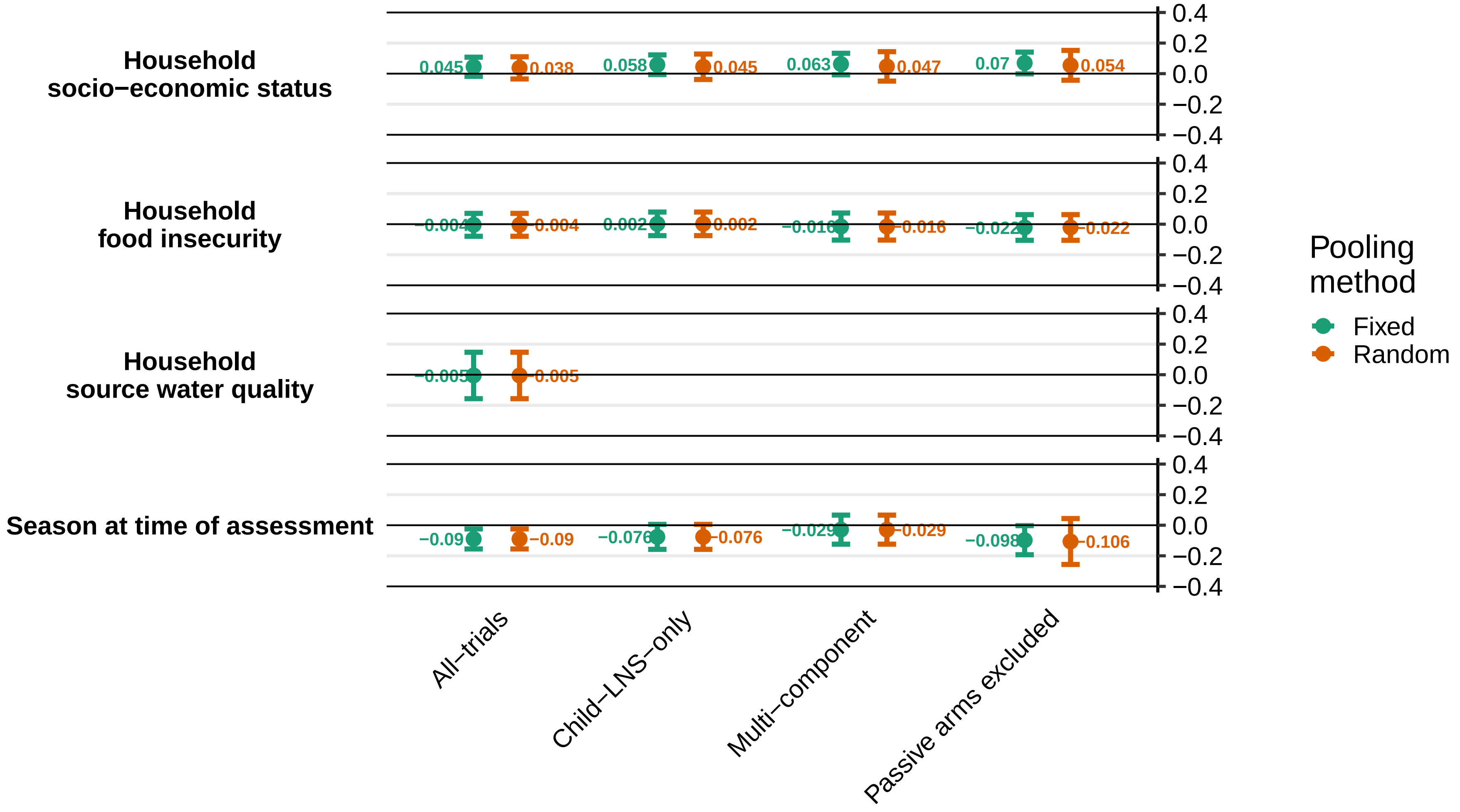

Supplemental figure 10I: Ratio of iron deficiency anemia prevalence ratios

10I1: By maternal effect modifiers

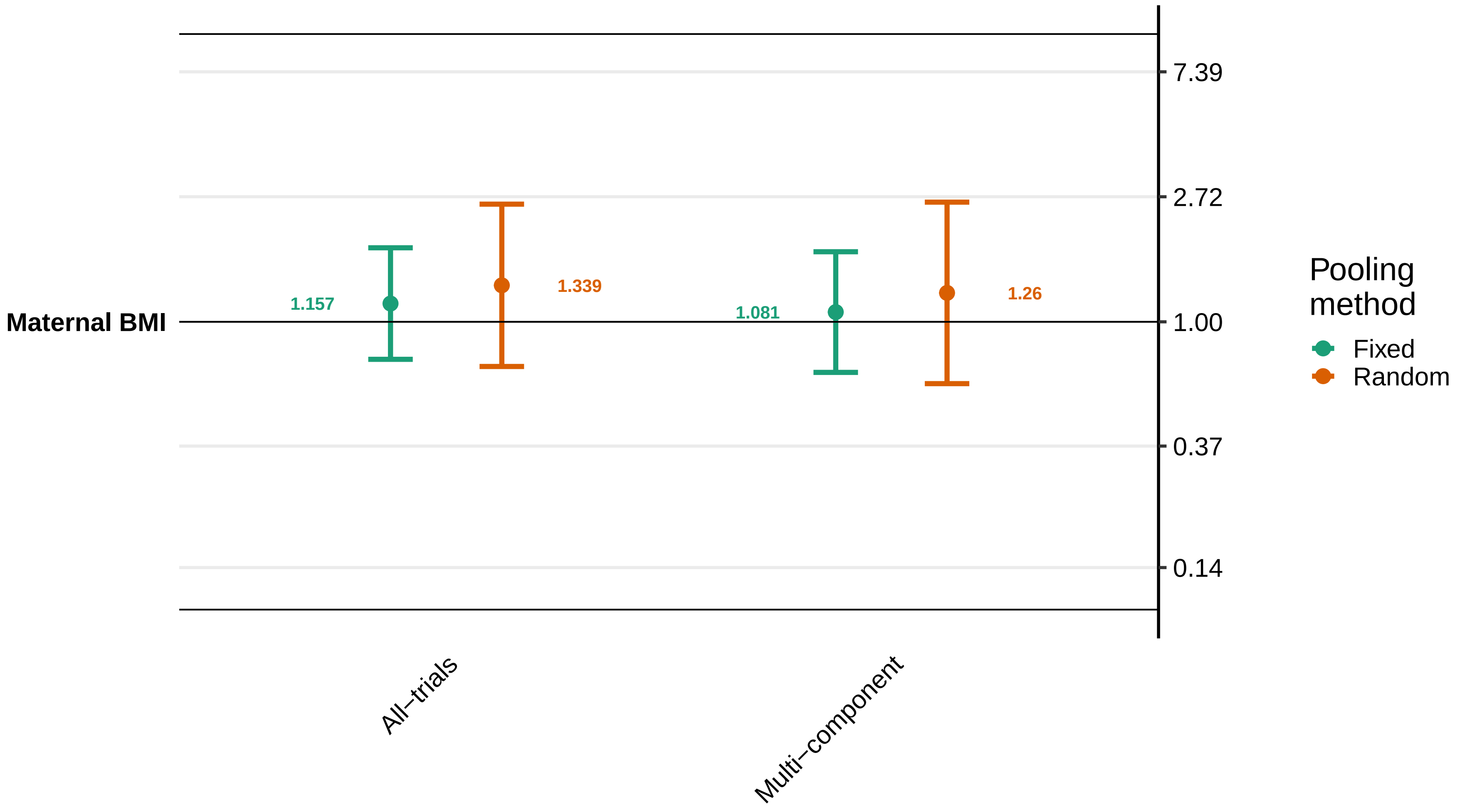

Supplemental figure 10I: Ratio of iron deficiency anemia prevalence ratios

10I2: By child effect modifiers

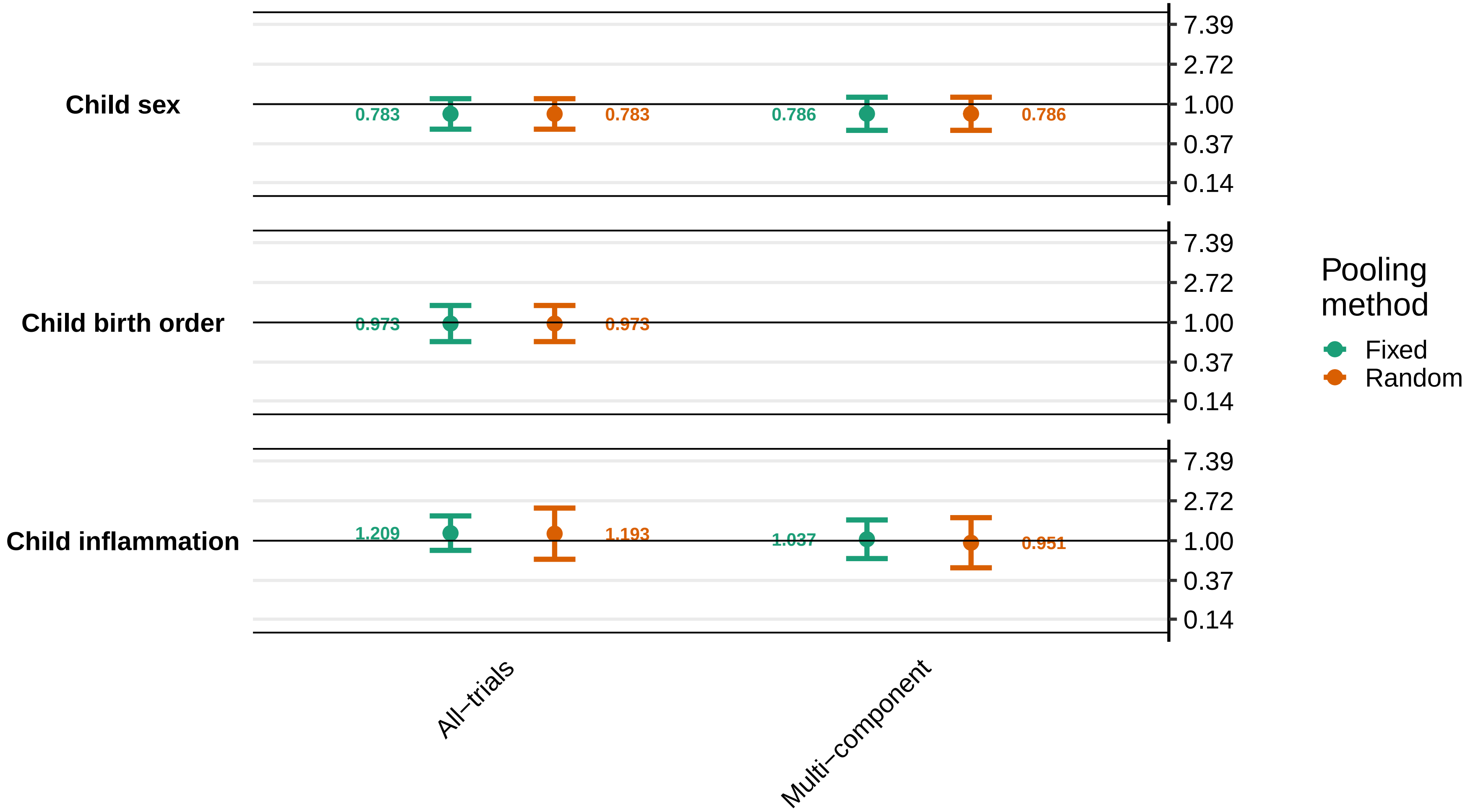

Supplemental figure 10I: Ratio of iron deficiency anemia prevalence ratios

10I3: By household effect modifiers

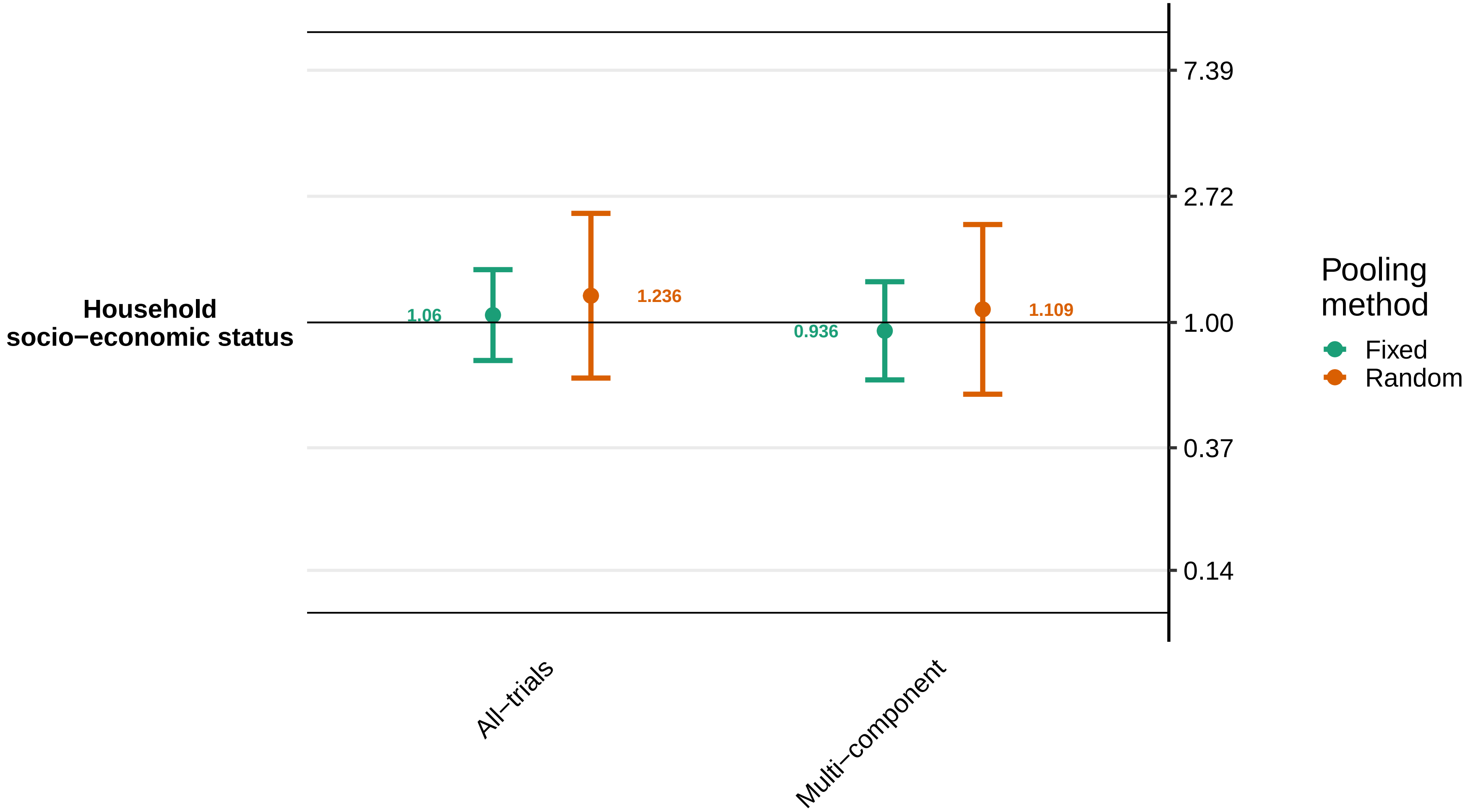

Supplemental figure 10J: Difference in iron deficiency anemia prevalence differences

10J1: By maternal effect modifiers

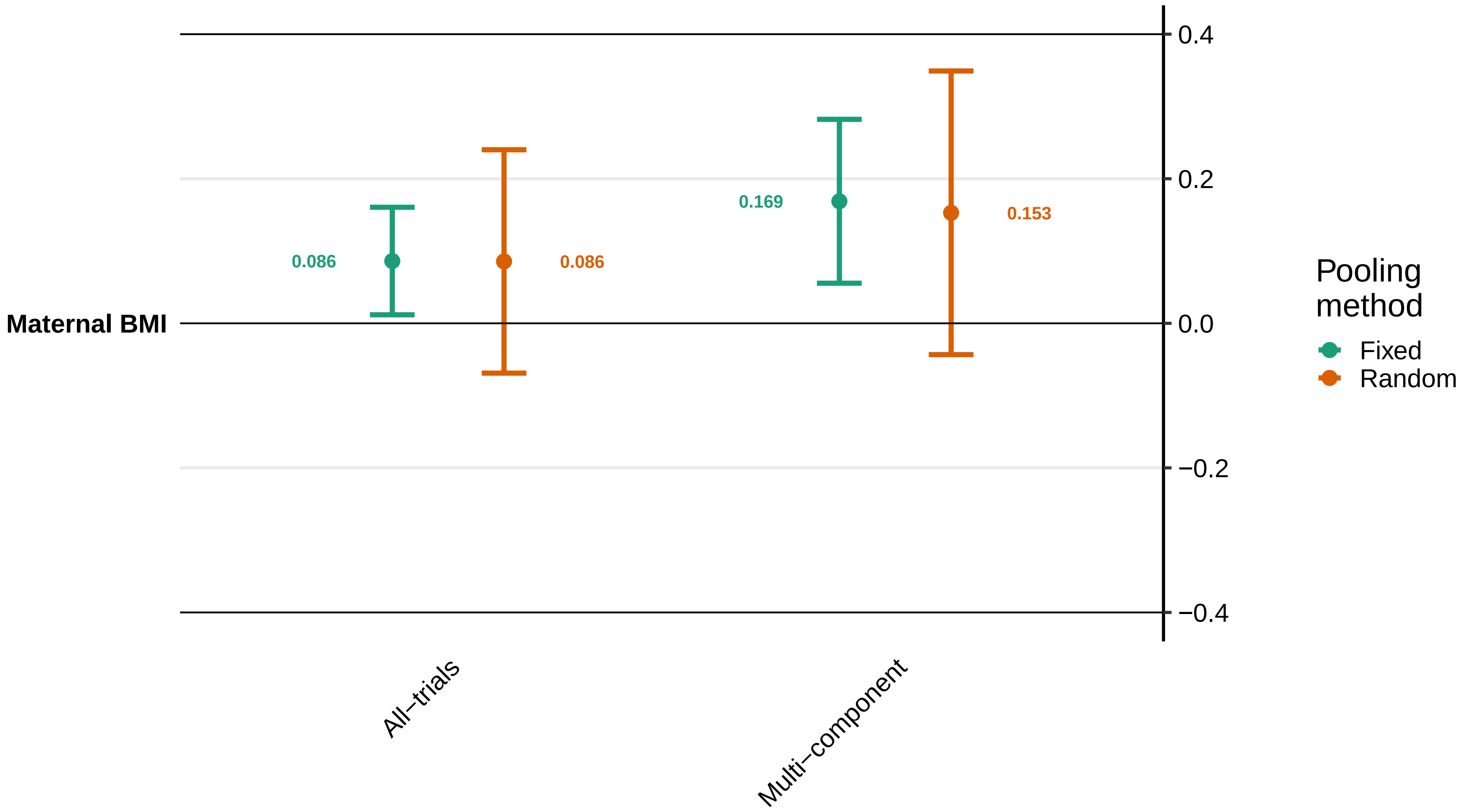

Supplemental figure 10J: Difference in iron deficiency anemia prevalence differences

10J2: By child effect modifiers

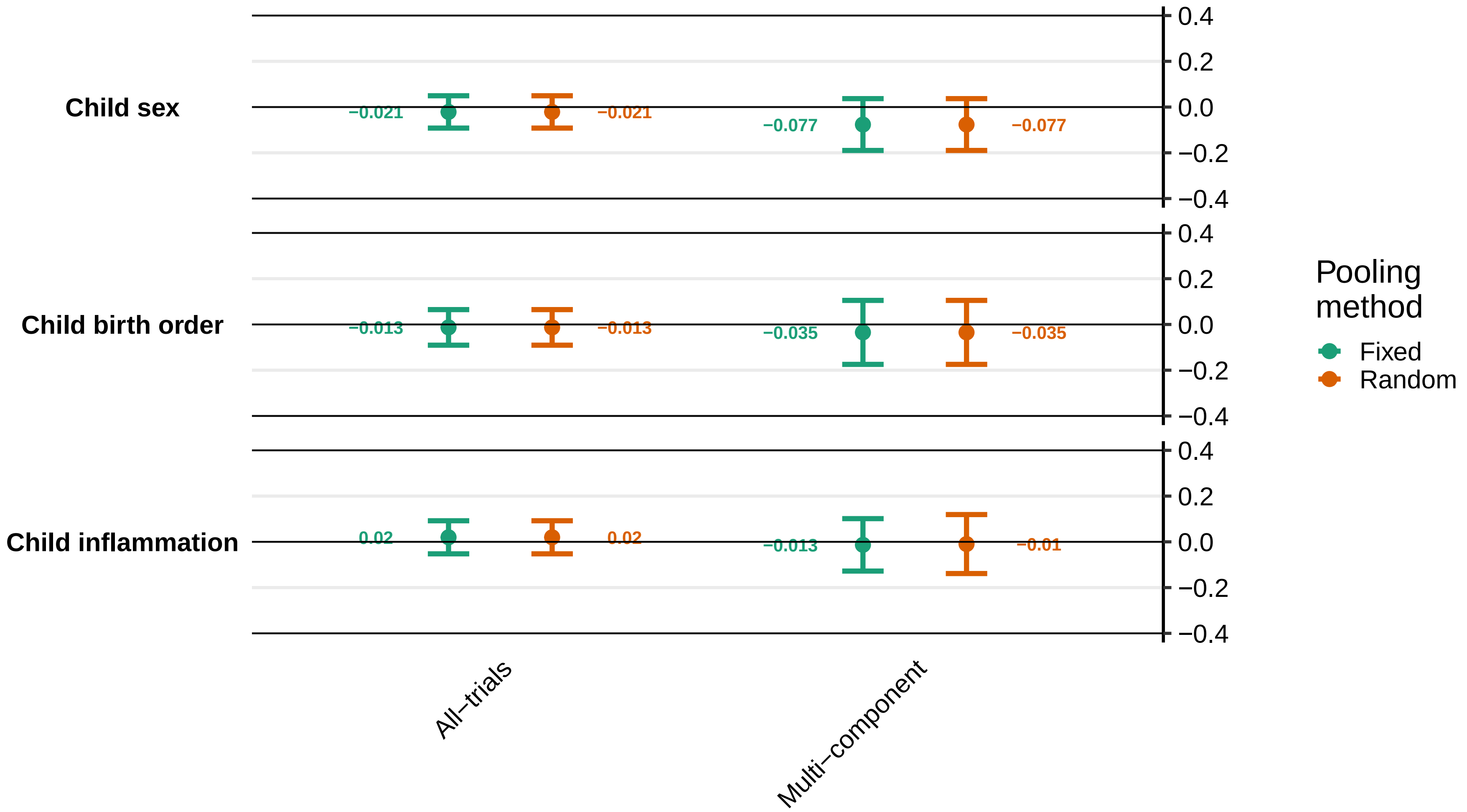

Supplemental figure 10J: Difference in iron deficiency anemia prevalence differences

10J3: By household effect modifiers

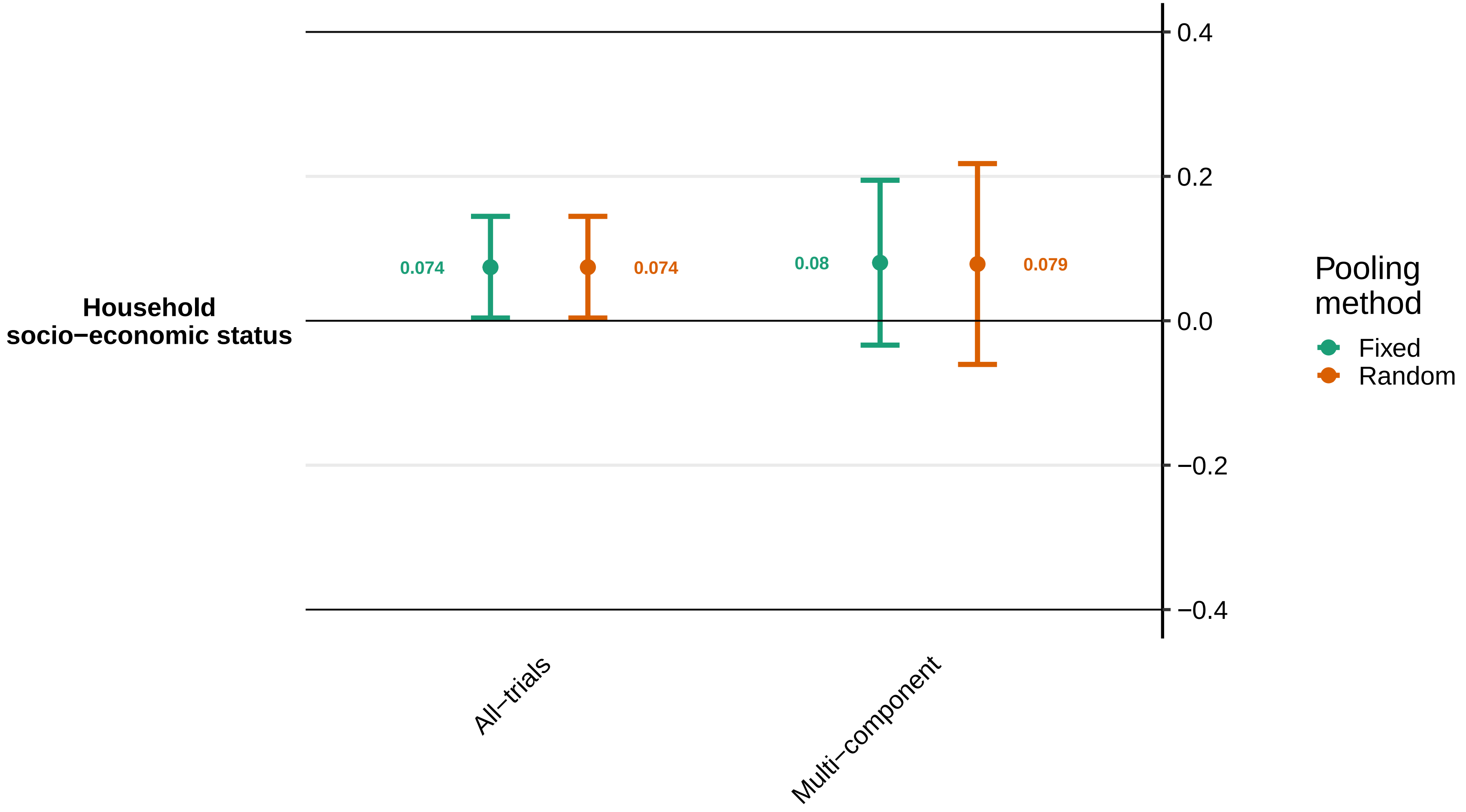

Supplemental figure 10K: Ratio of geometric mean ratios of soluble transferrin receptor concentration

10K1: By maternal effect modifiers

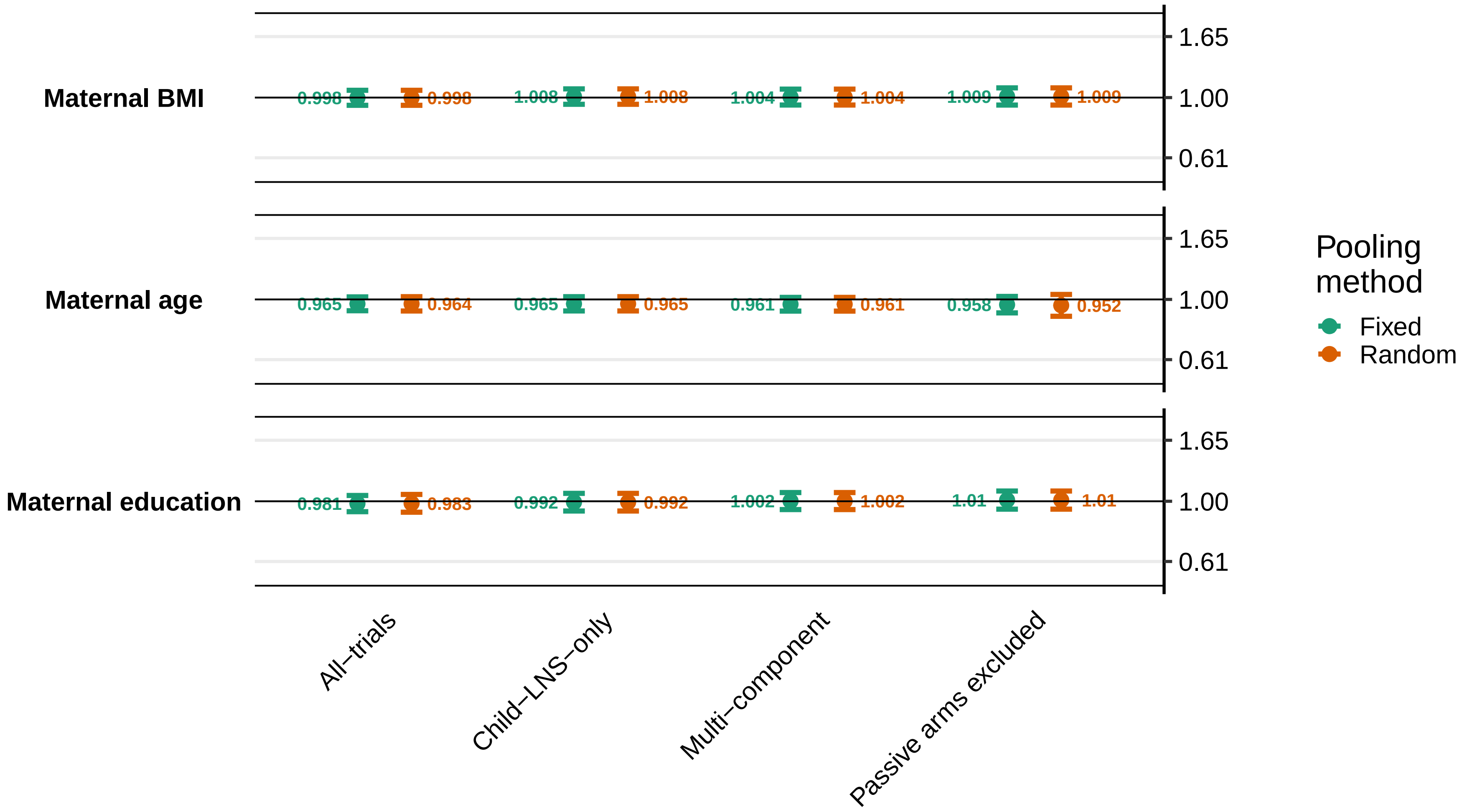

Supplemental figure 10K: Ratio of geometric mean ratios of soluble transferrin receptor concentration

10K2: By child effect modifiers

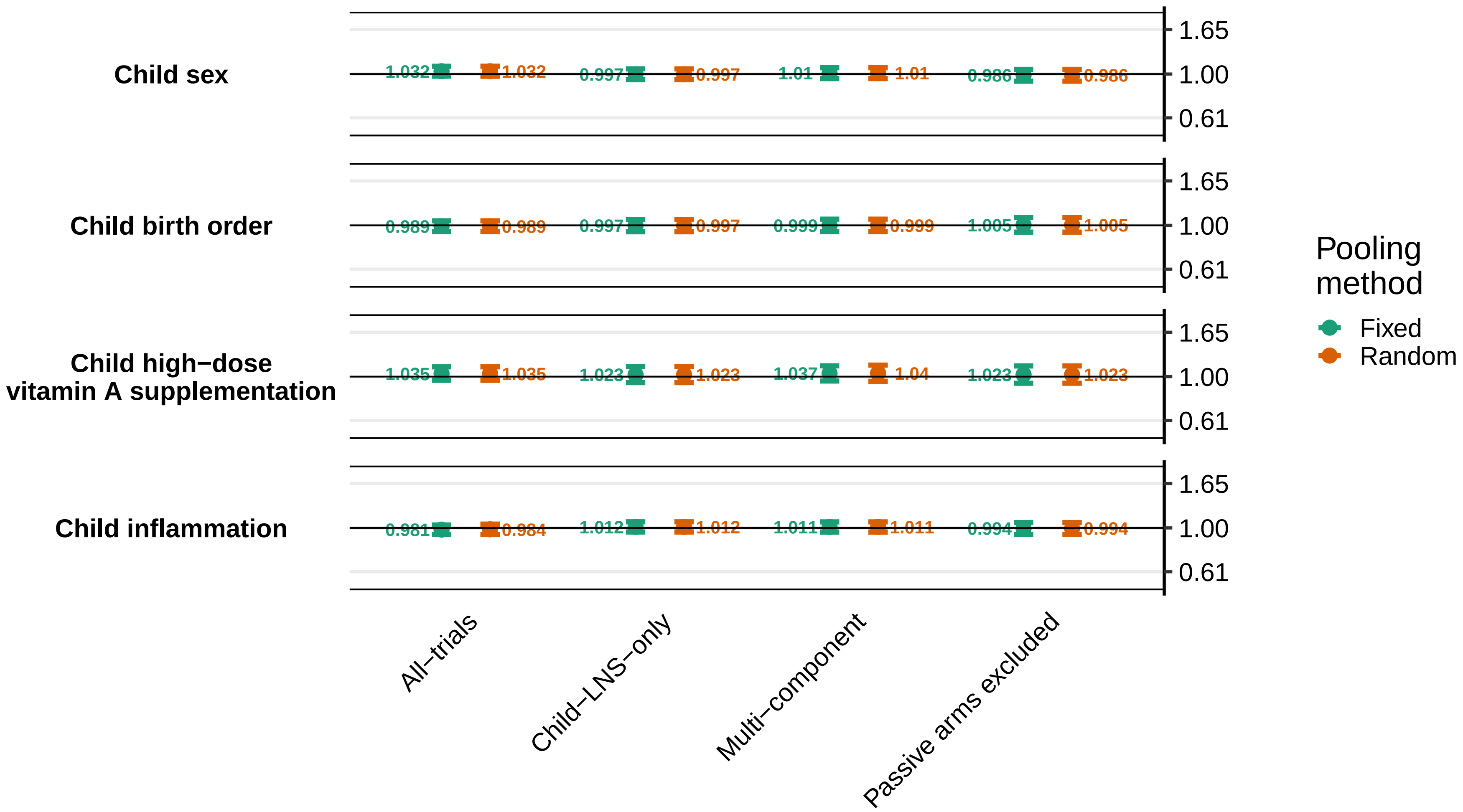

Supplemental figure 10K: Ratio of geometric mean ratios of soluble transferrin receptor concentration

10K3: By household effect modifiers

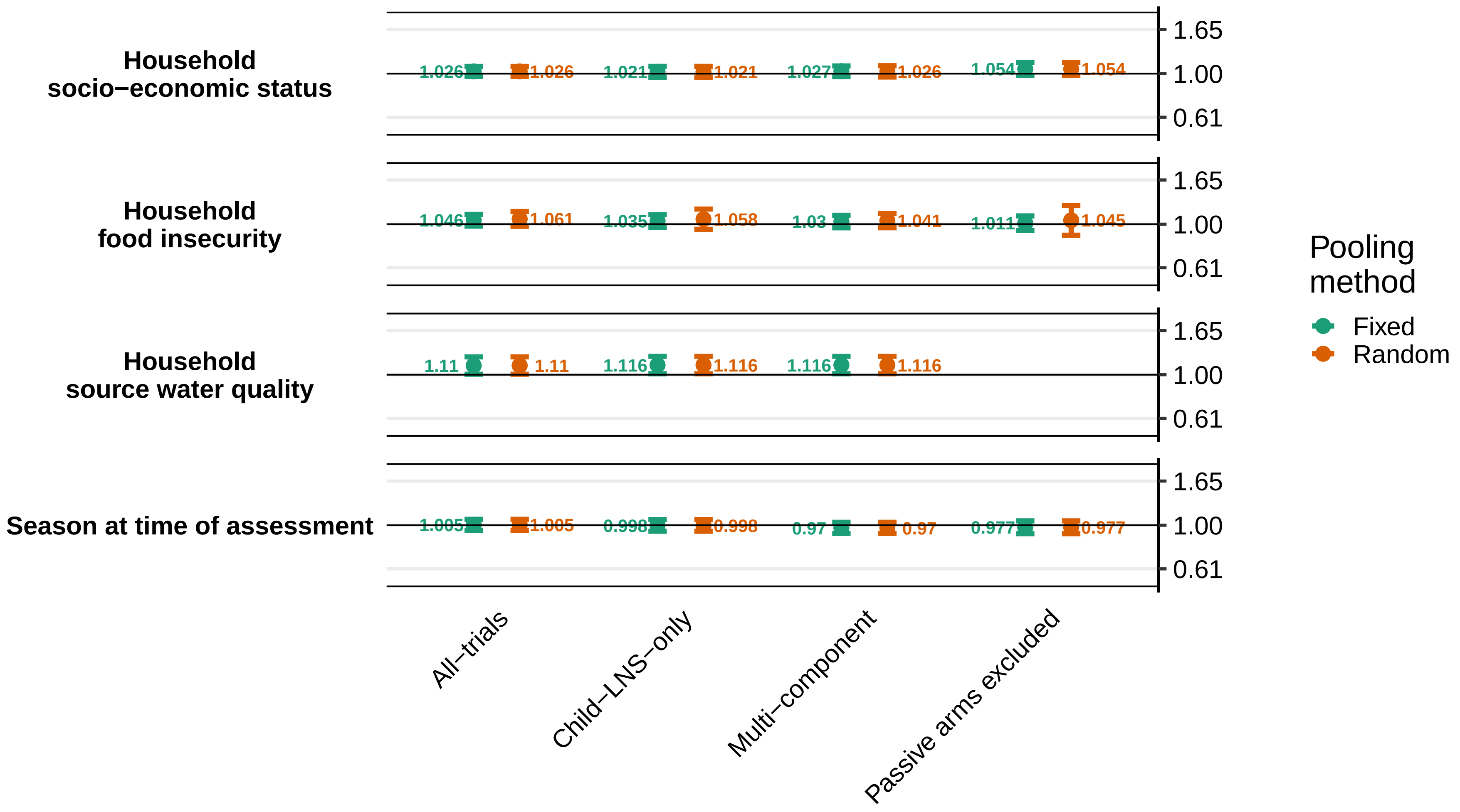

Supplemental figure 10L: Ratio of elevated soluble transferrin receptor prevalence ratios

10L1: By maternal effect modifiers

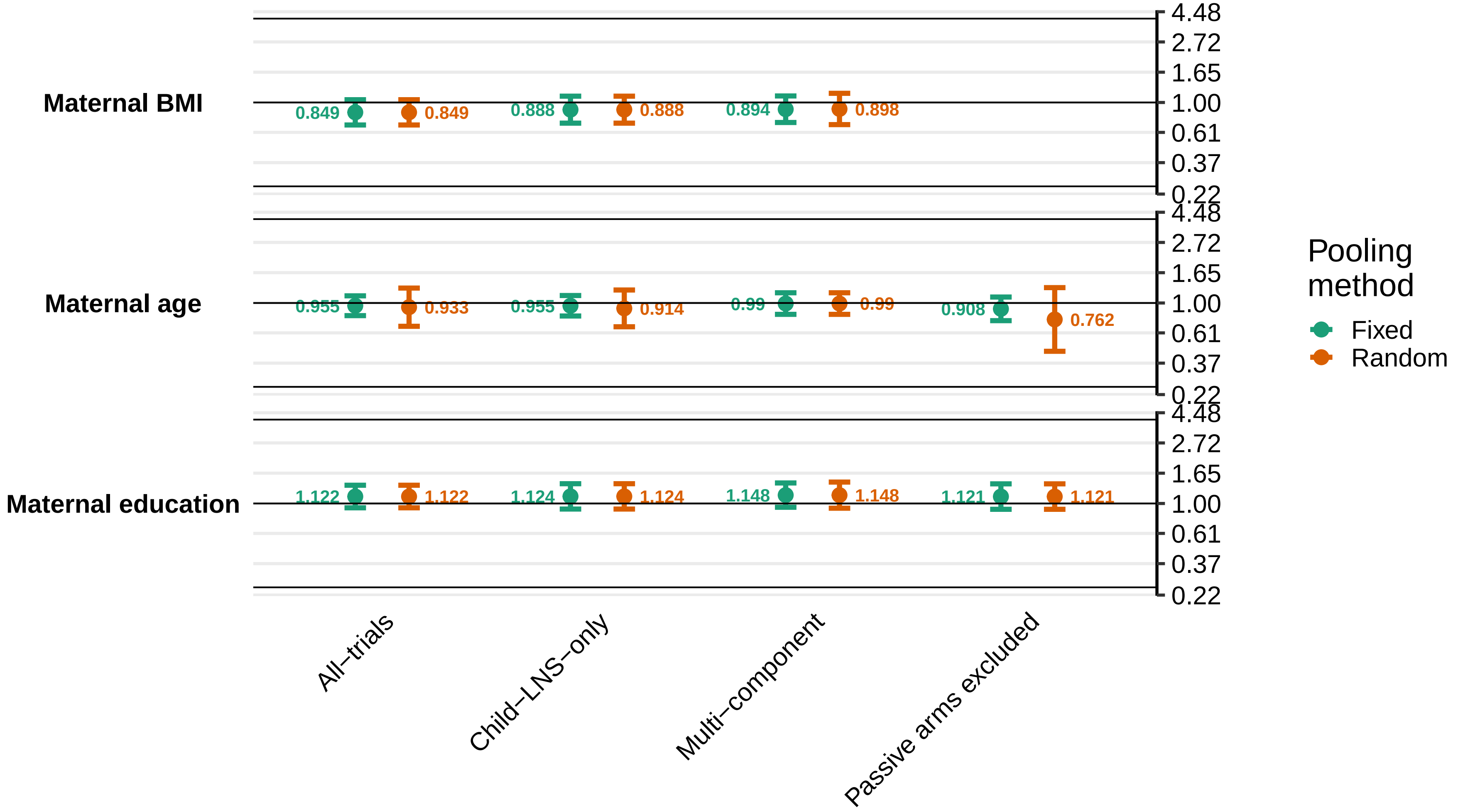

Supplemental figure 10L: Ratio of elevated soluble transferrin receptor prevalence ratios

10L2: By child effect modifiers

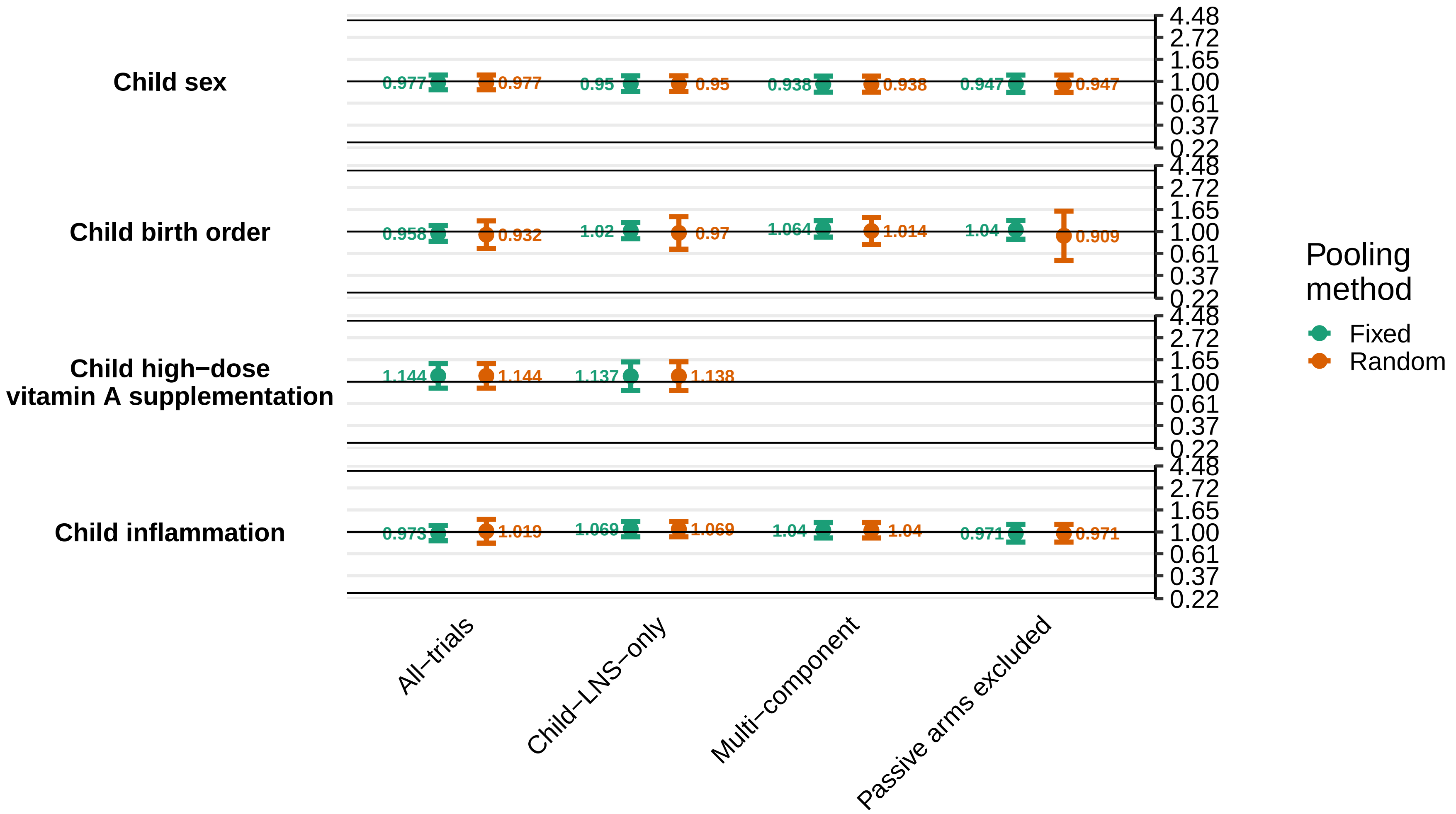

Supplemental figure 10L: Ratio of elevated soluble transferrin receptor prevalence ratios

10L3: By household effect modifiers

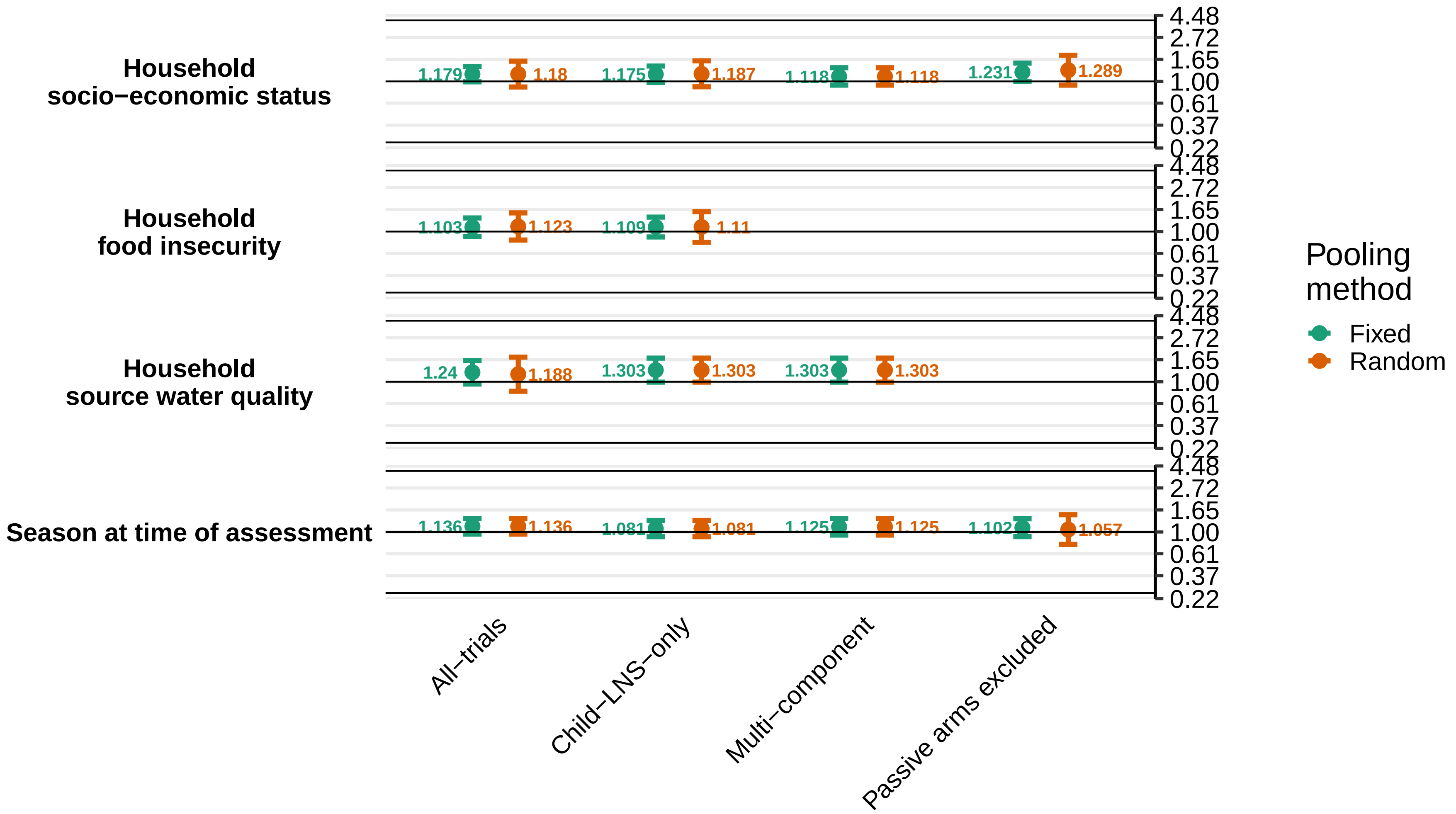

Supplemental figure 10M: Difference in elevated soluble transferrin receptor prevalence differences

10M1: By maternal effect modifiers

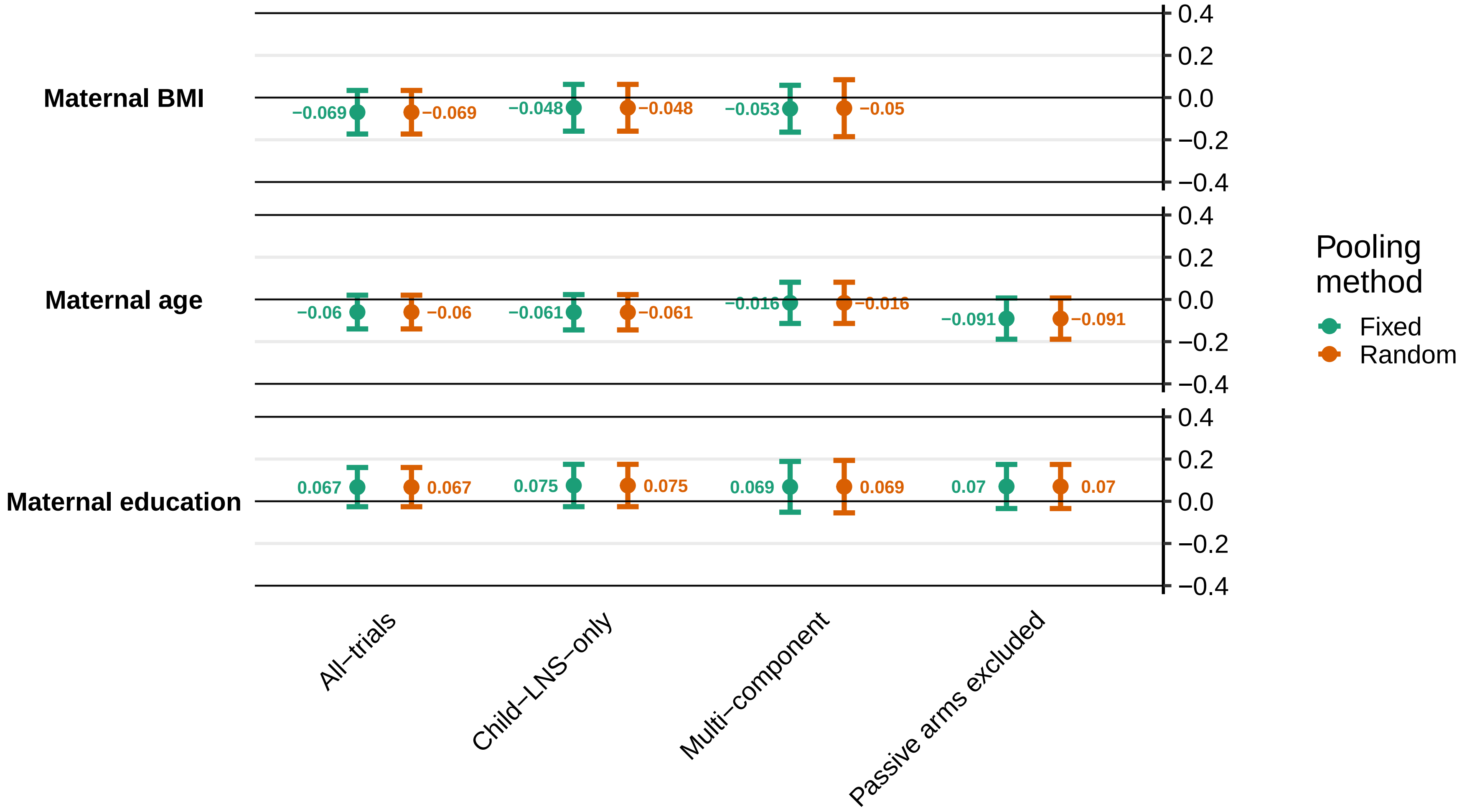

Supplemental figure 10M: Difference in elevated soluble transferrin receptor prevalence differences

10M2: By child effect modifiers

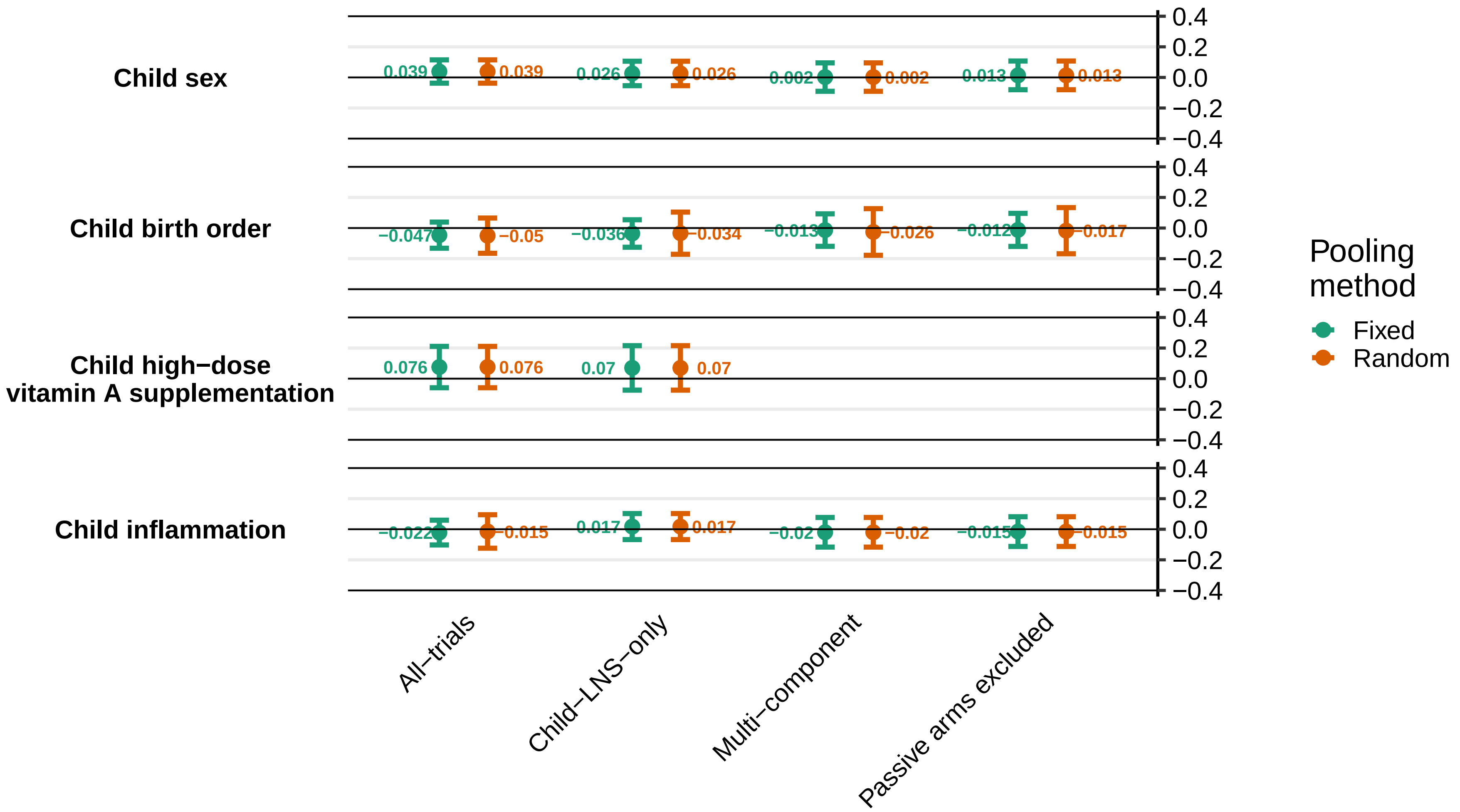

Supplemental figure 10M: Difference in elevated soluble transferrin receptor prevalence differences

10M3: By household effect modifiers

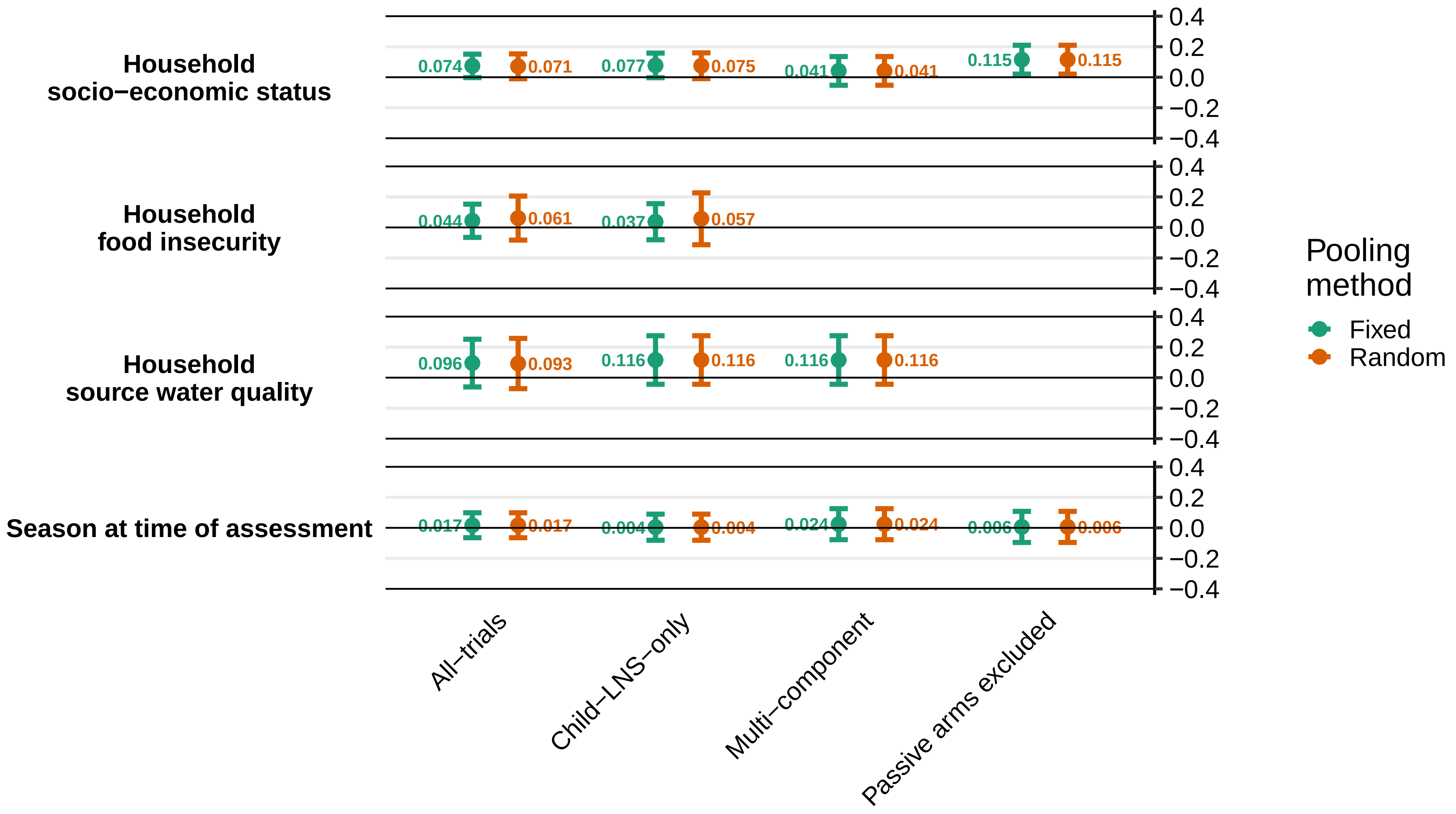

Supplemental figure 10N: Ratio of geometric mean ratios of zinc protoporphyrin concentration

10N1: By maternal effect modifiers

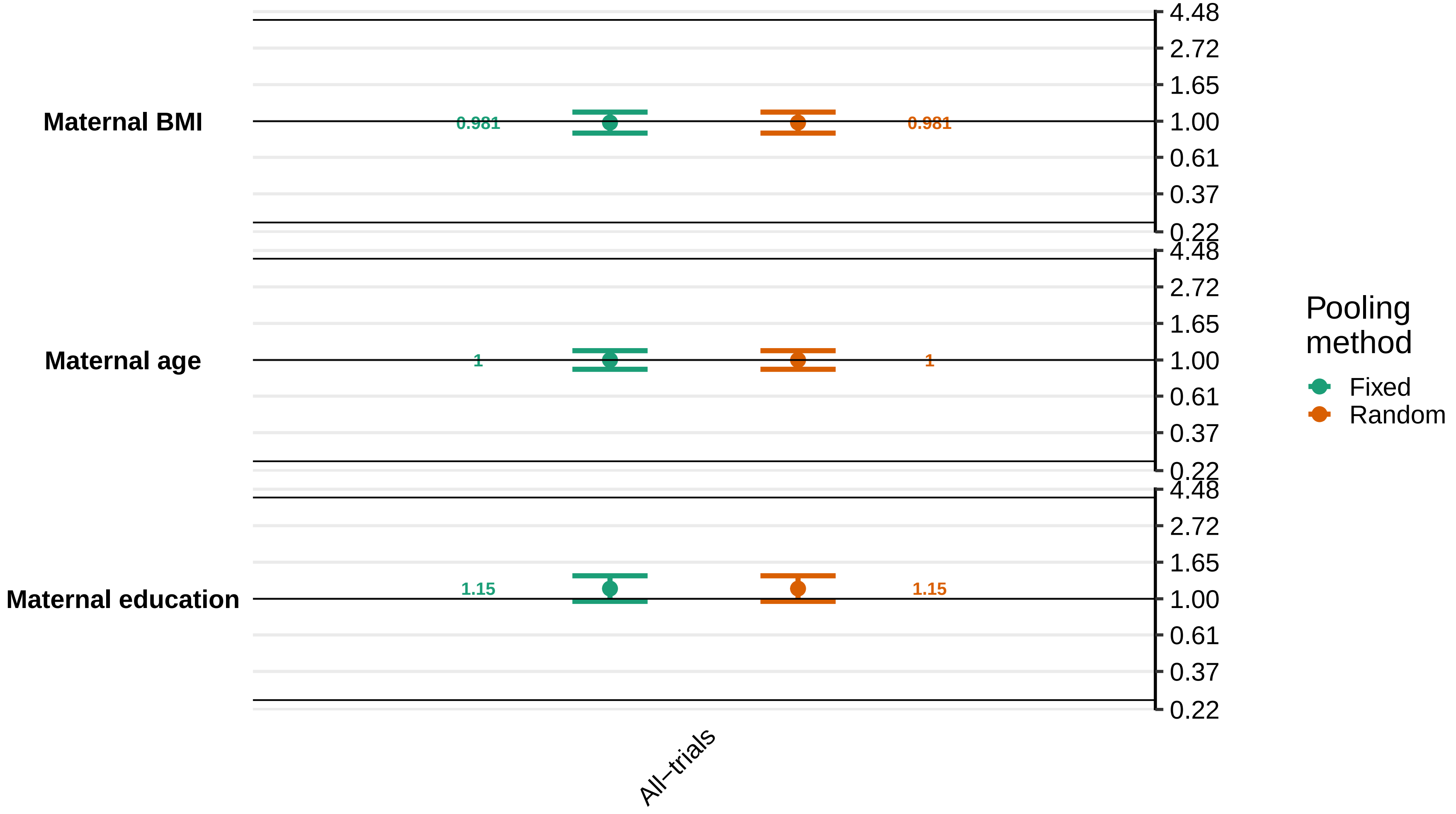

Supplemental figure 10N: Ratio of geometric mean ratios of zinc protoporphyrin concentration

10N2: By child effect modifiers

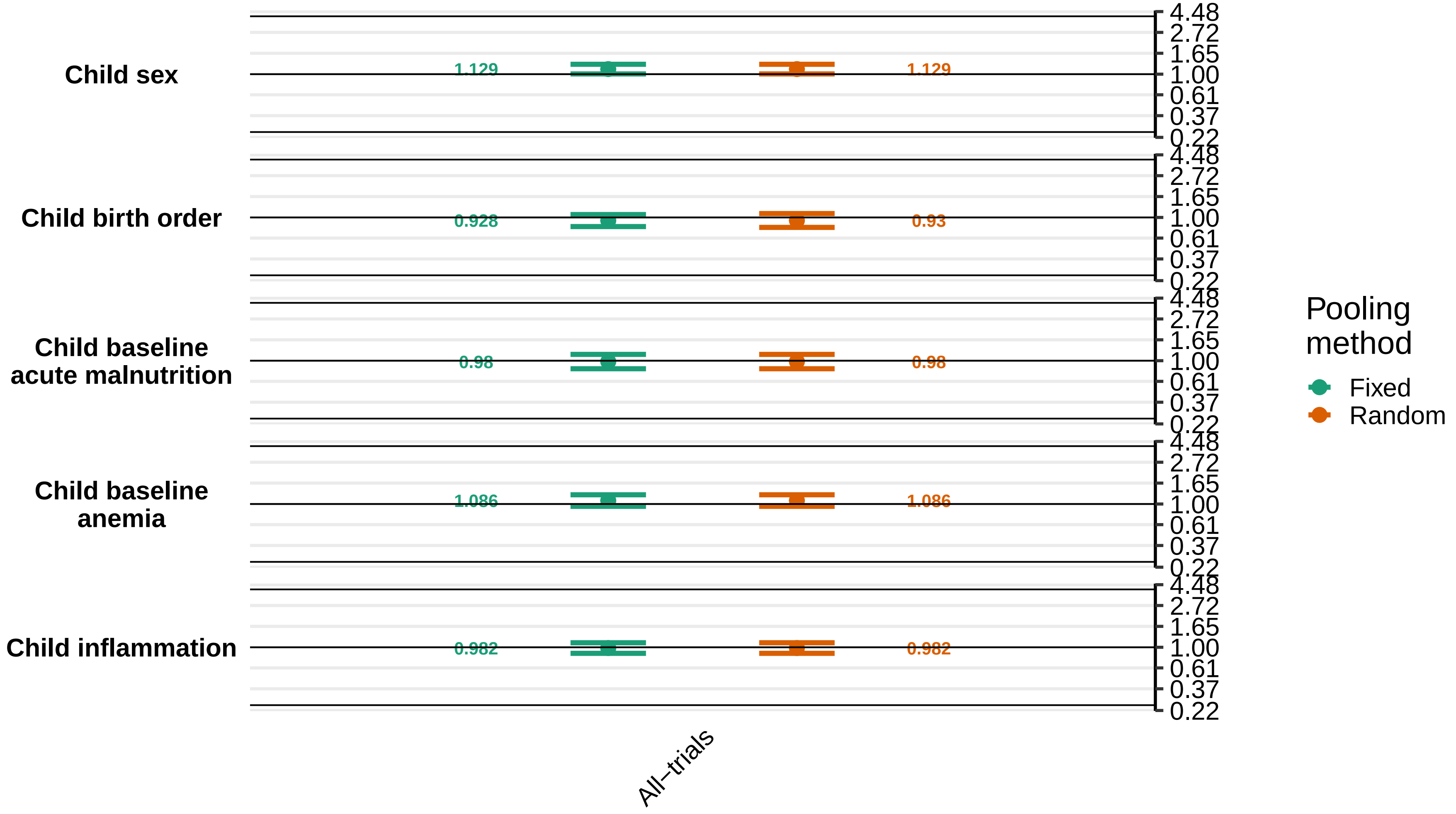

Supplemental figure 10N: Ratio of geometric mean ratios of zinc protoporphyrin concentration

10N3: By household effect modifiers

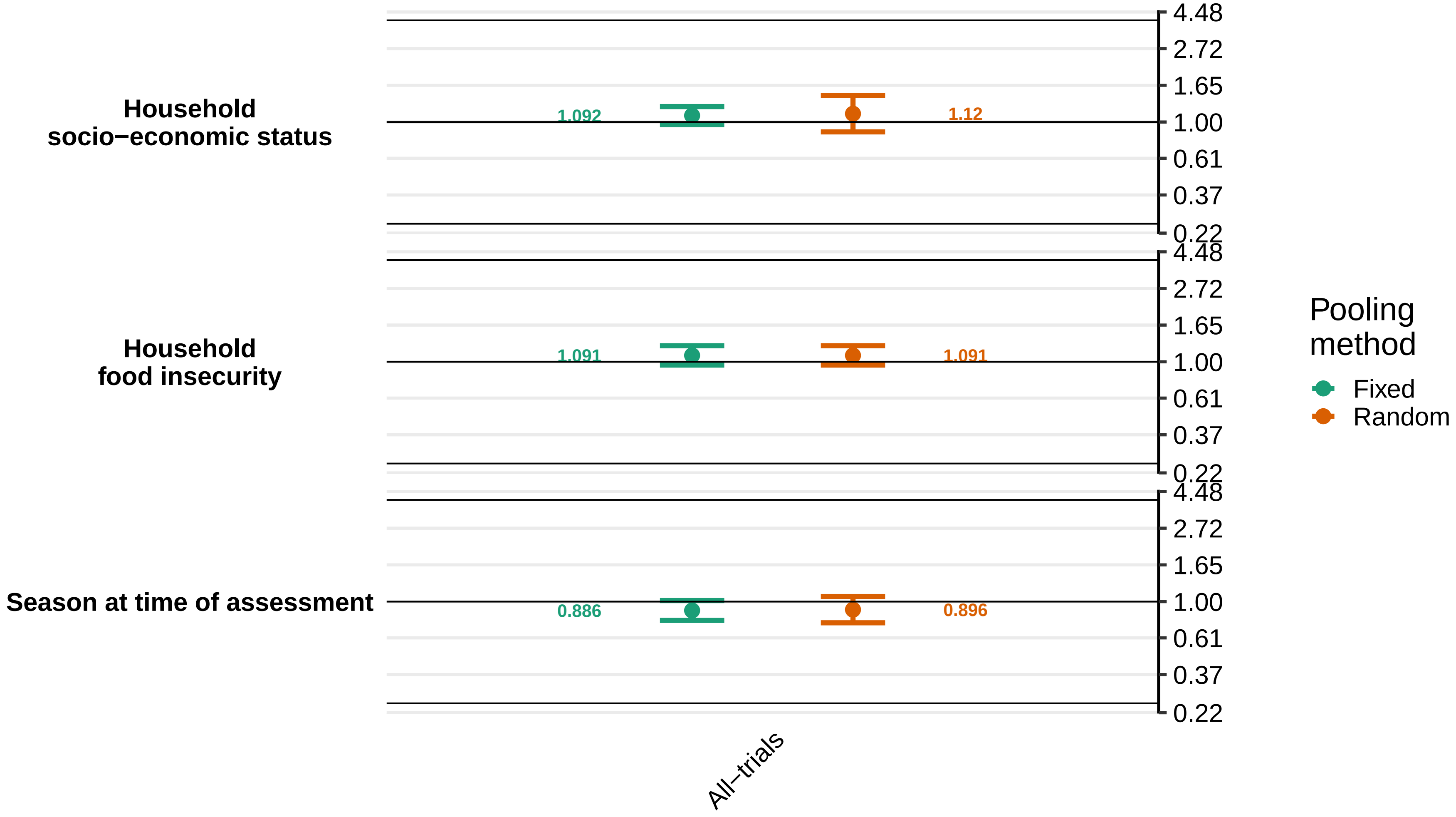

Supplemental figure 10O: Ratio of elevated zinc protoporphyrin prevalence ratios

10O1: By maternal effect modifiers

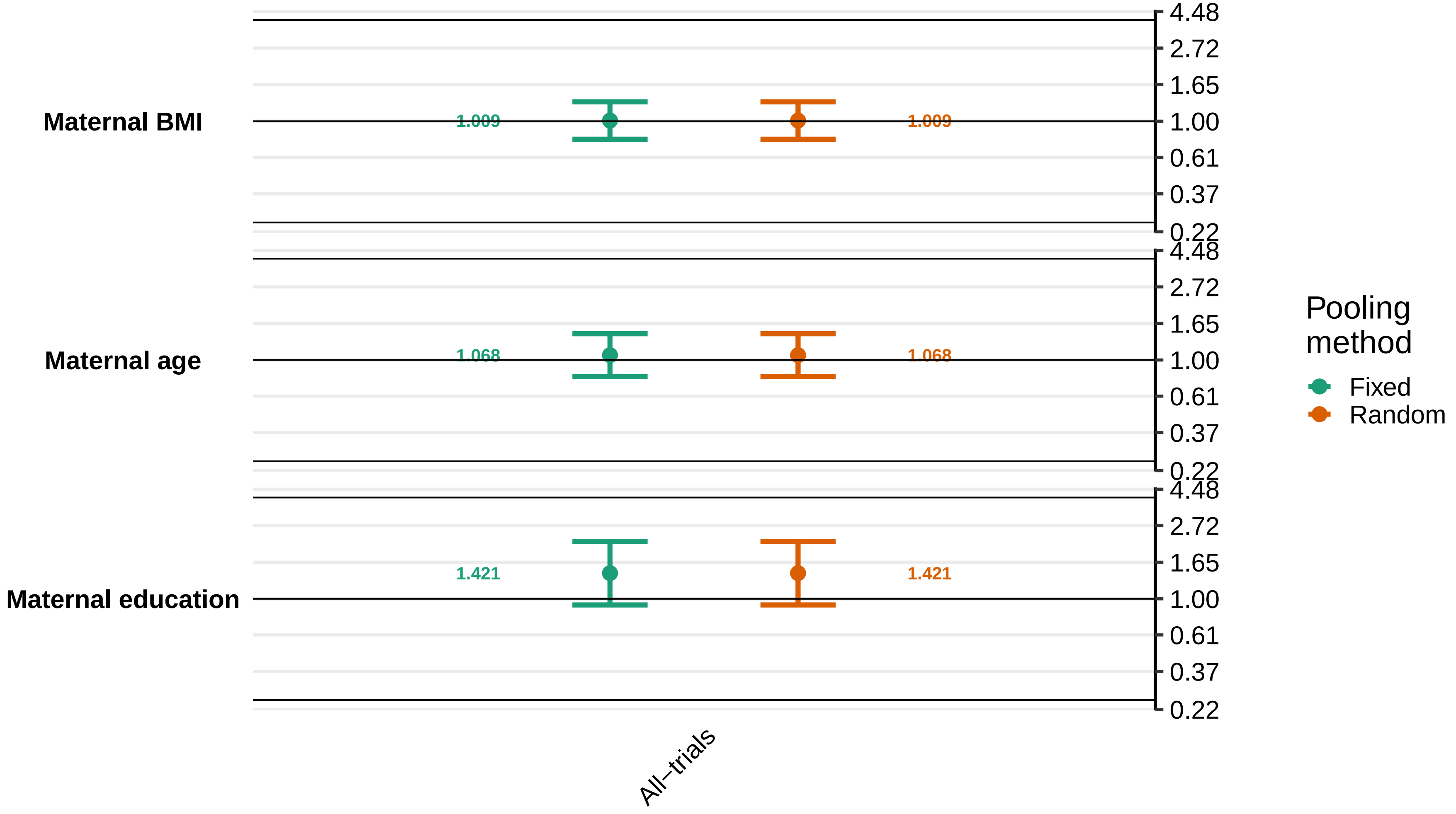

Supplemental figure 10O: Ratio of elevated zinc protoporphyrin prevalence ratios

10O2: By child effect modifiers

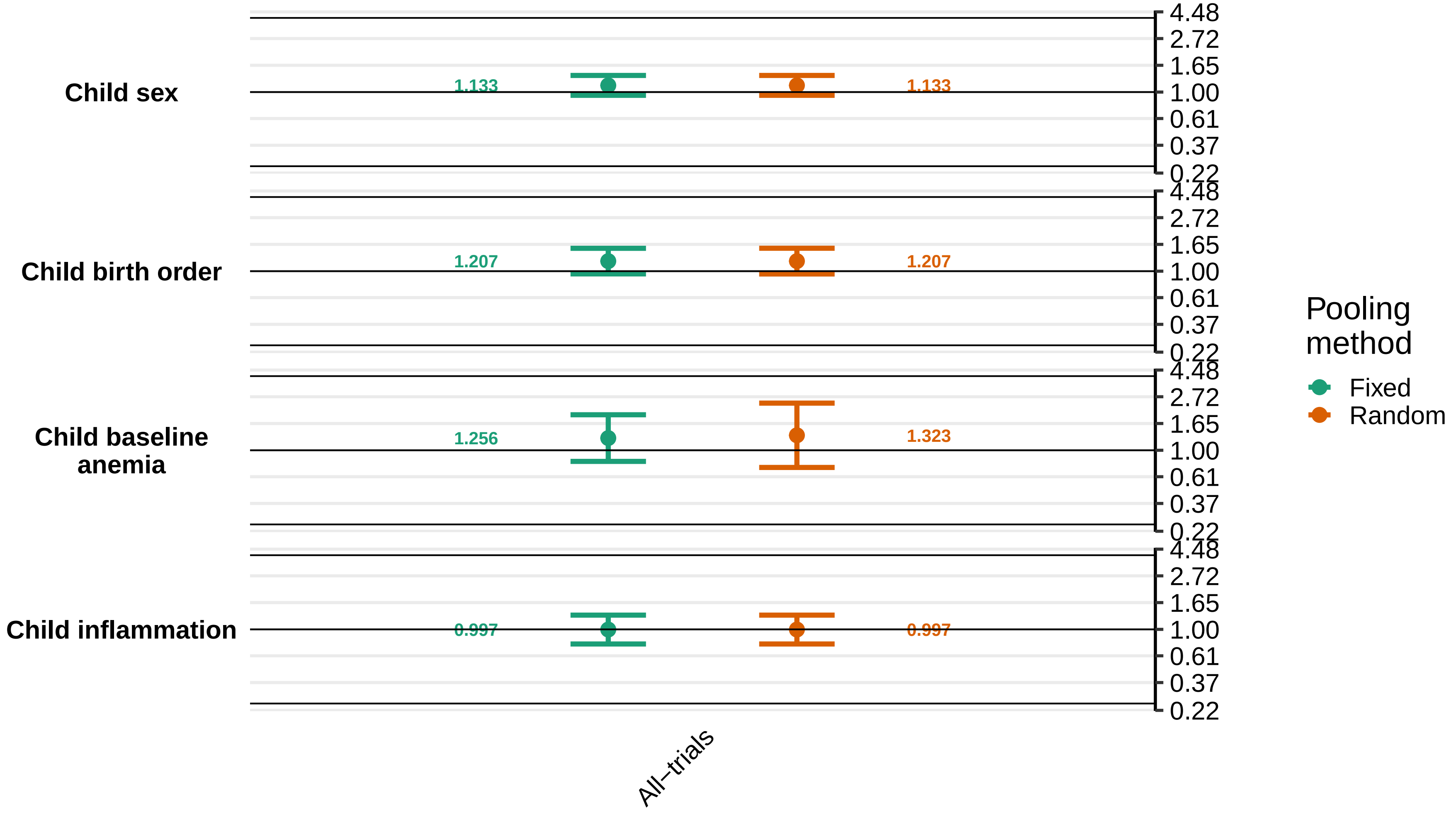

Supplemental figure 10O: Ratio of elevated zinc protoporphyrin prevalence ratios

10O3: By household effect modifiers

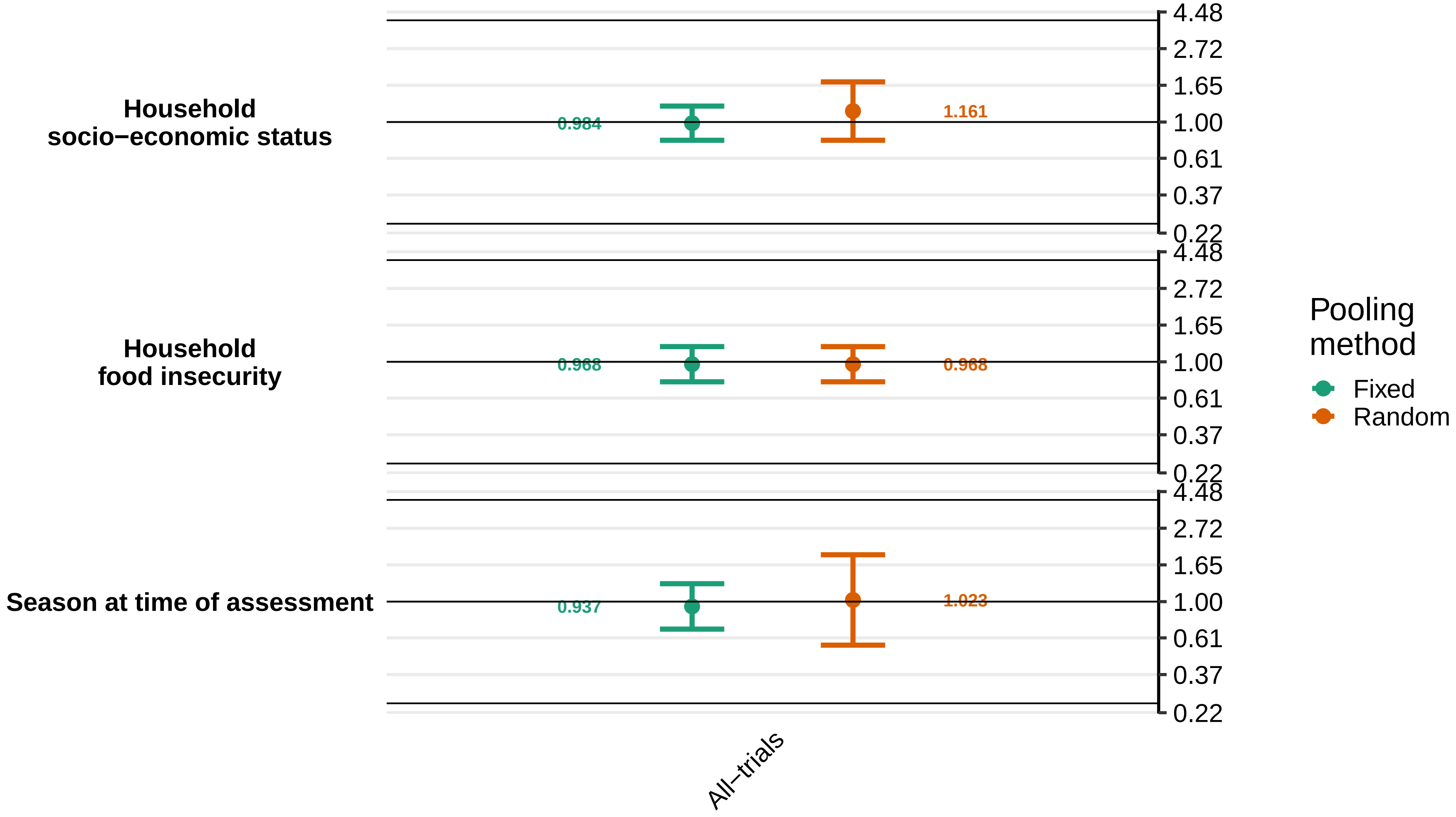

Supplemental figure 10P: Difference in elevated zinc protoporphyrin prevalence differences

10P1: By maternal effect modifiers

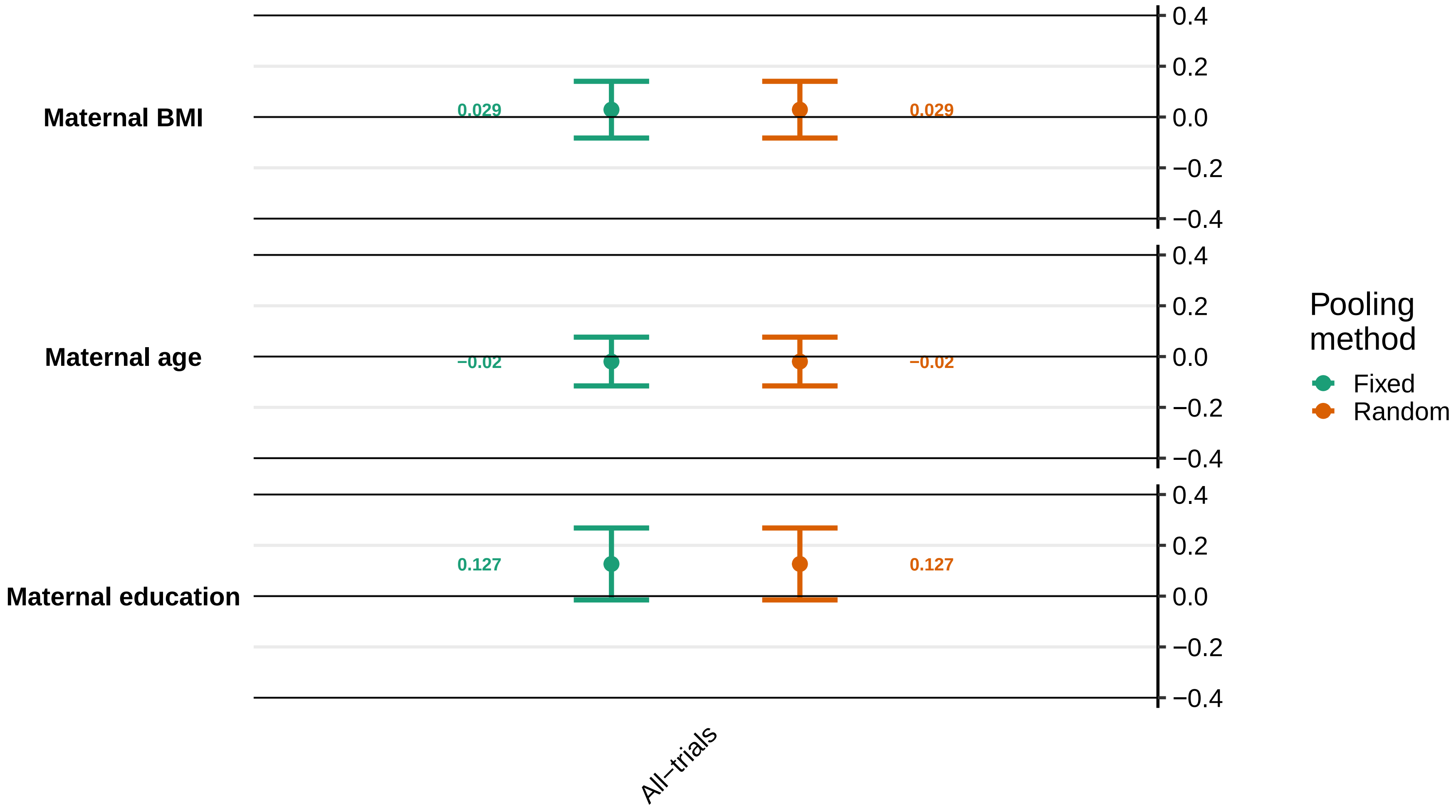

Supplemental figure 10P: Difference in elevated zinc protoporphyrin prevalence differences

10P2: By child effect modifiers

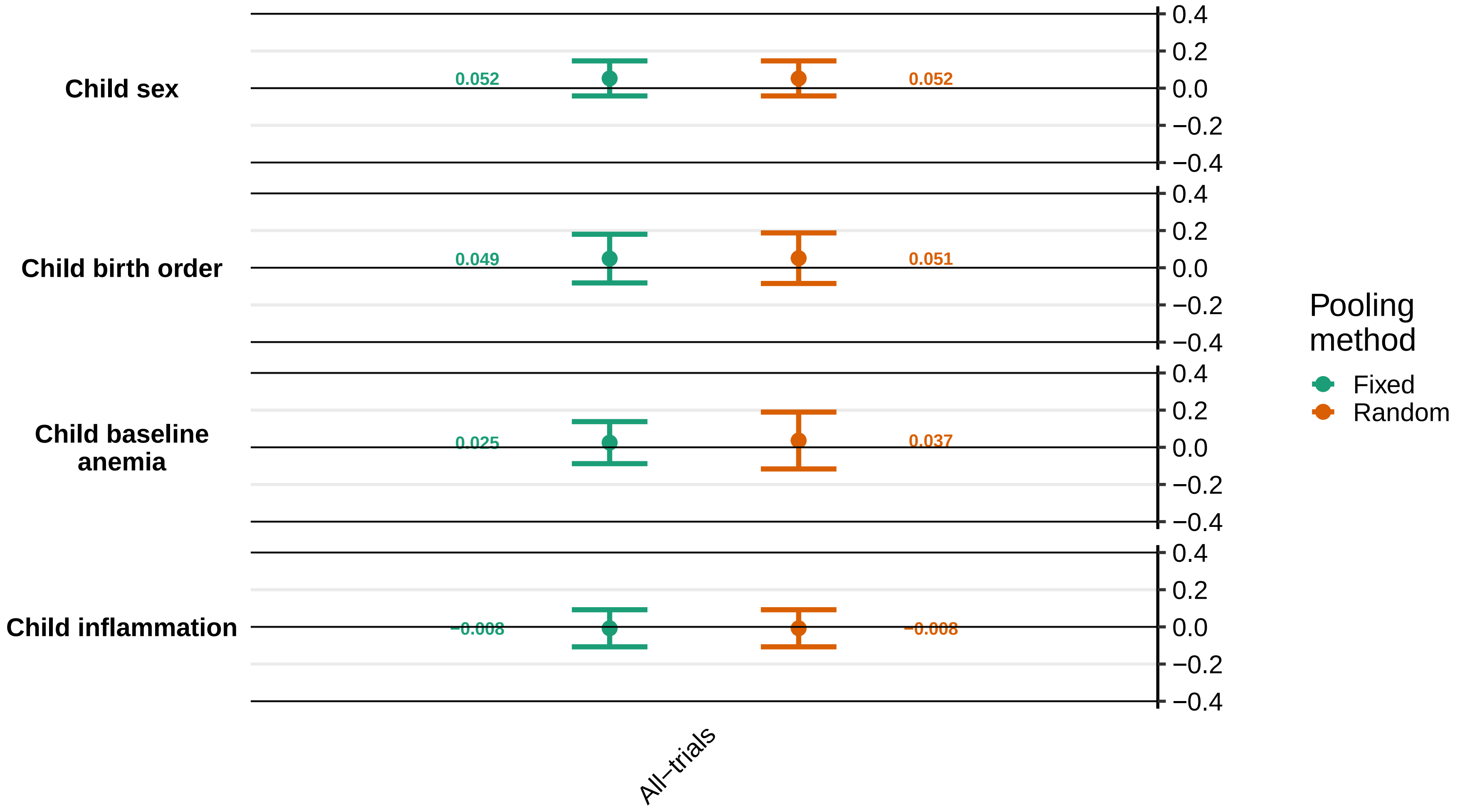

Supplemental figure 10P: Difference in elevated zinc protoporphyrin prevalence differences

10P3: By household effect modifiers

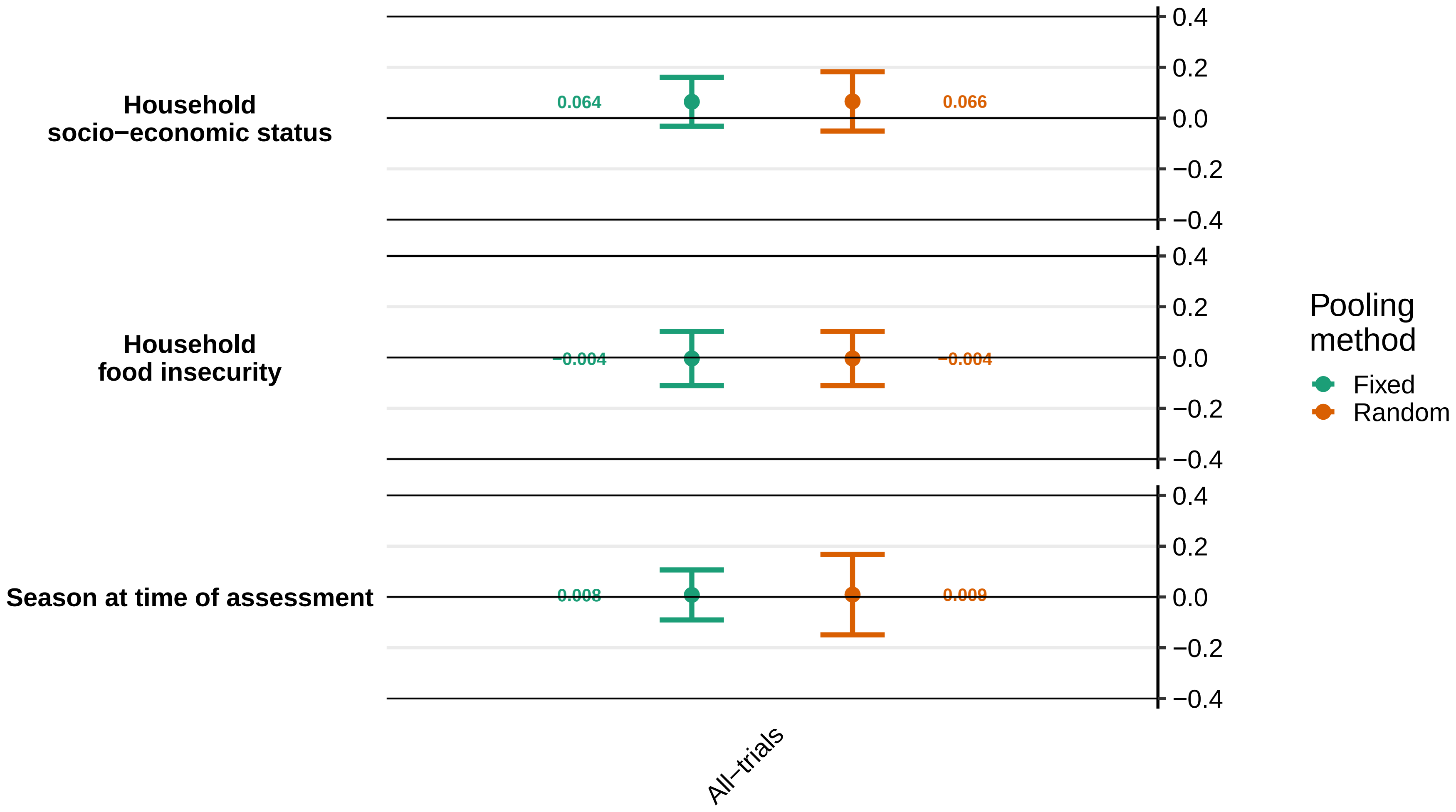

Supplemental figure 10Q: Ratio of geometric mean ratios of plasma zinc concentration

10Q1: By maternal effect modifiers

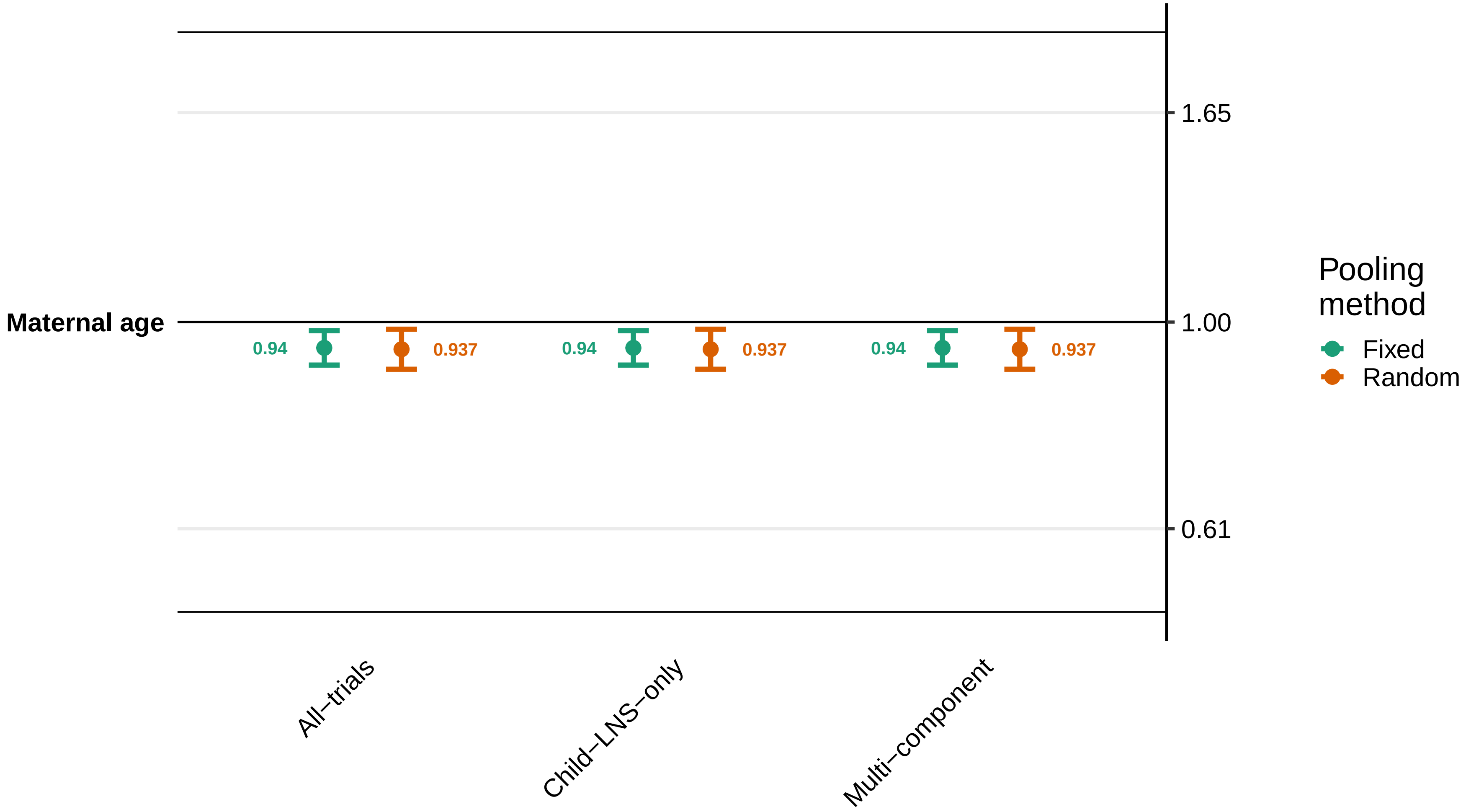

Supplemental figure 10Q: Ratio of geometric mean ratios of plasma zinc concentration

10Q2: By child effect modifiers

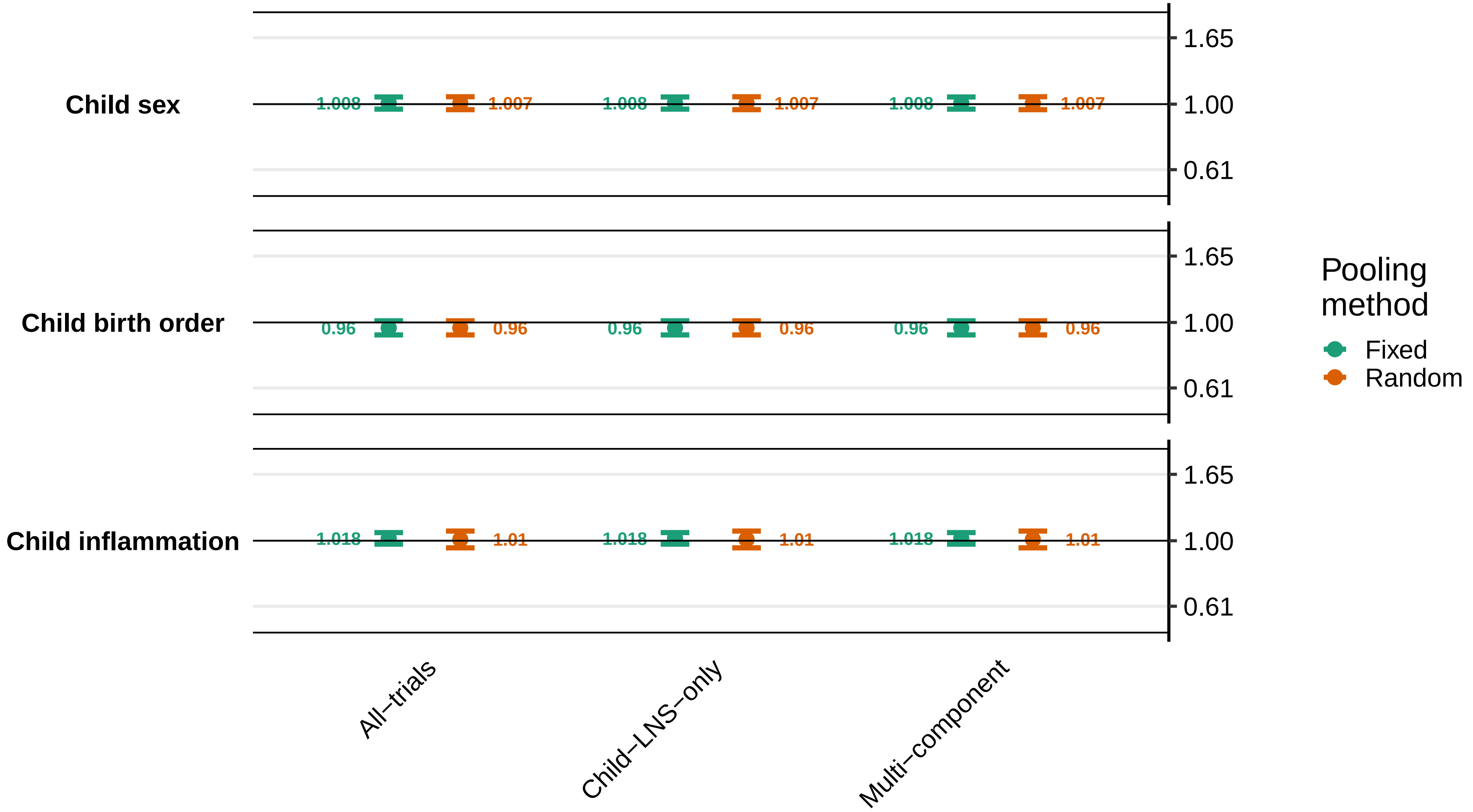

Supplemental figure 10Q: Ratio of geometric mean ratios of plasma zinc concentration

10Q3: By household effect modifiers

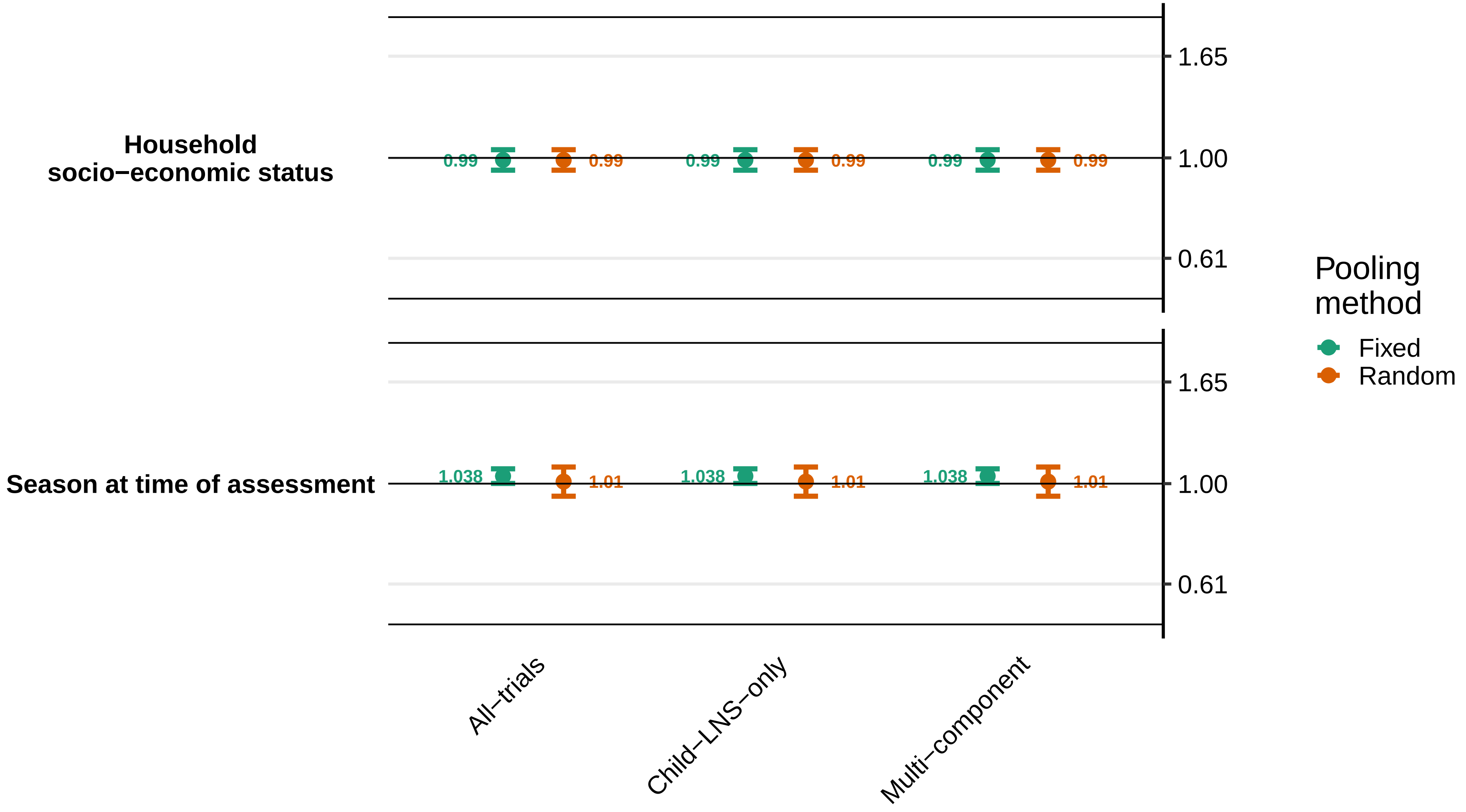

Supplemental figure 10R: Ratio of geometric mean ratios of retinol concentration

10R1: By maternal effect modifiers

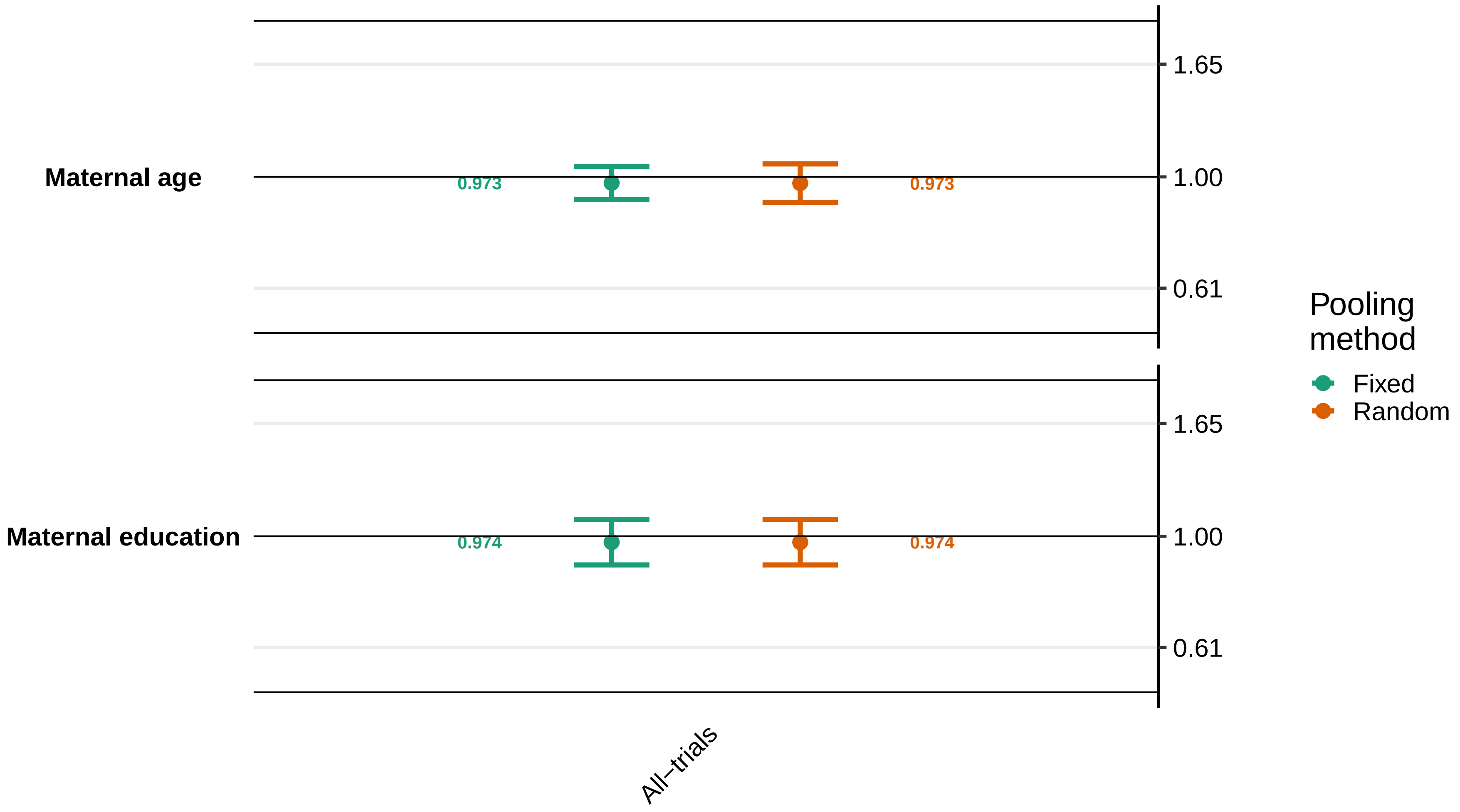

Supplemental figure 10R: Ratio of geometric mean ratios of retinol concentration

10R2: By child effect modifiers

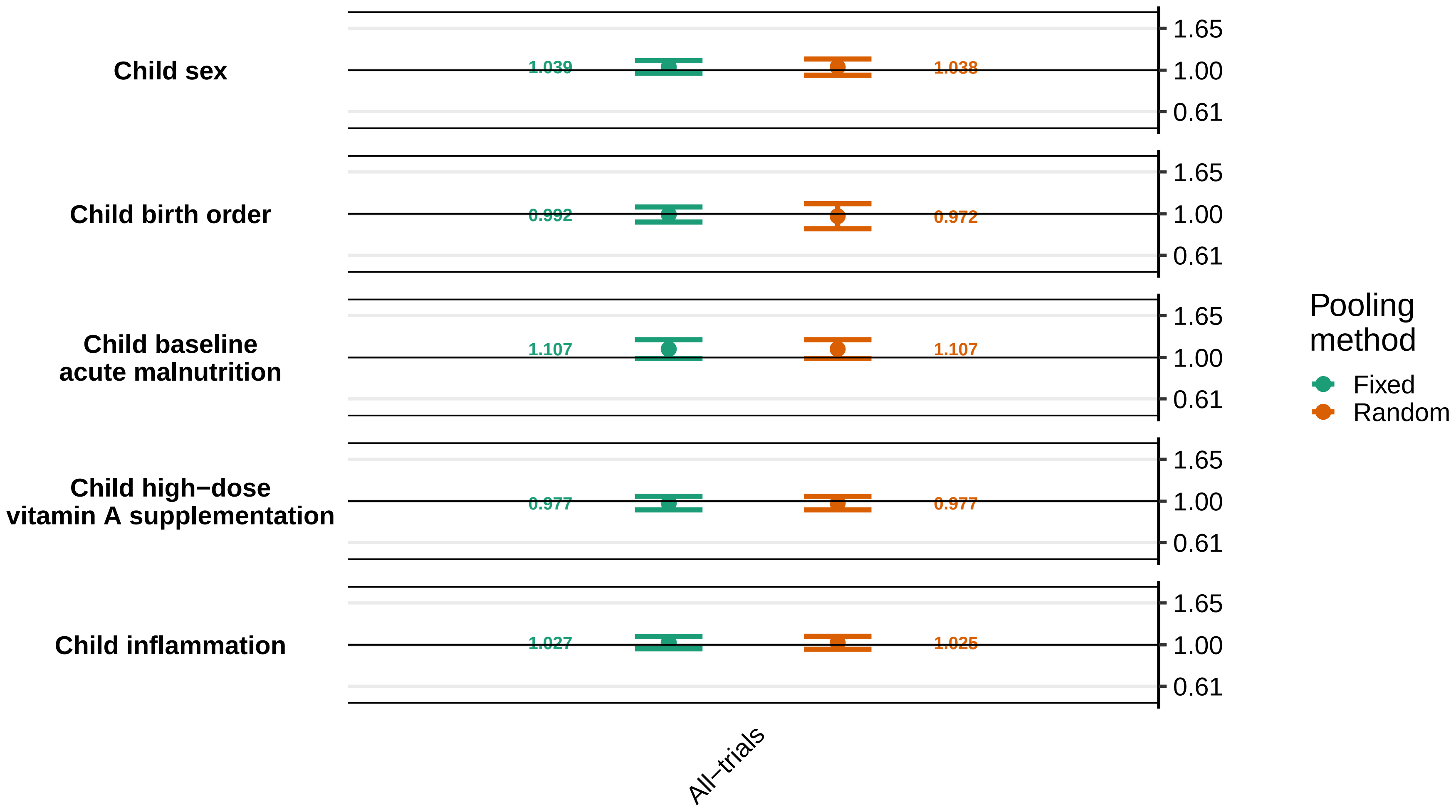

Supplemental figure 10R: Ratio of geometric mean ratios of retinol concentration

10R3: By household effect modifiers

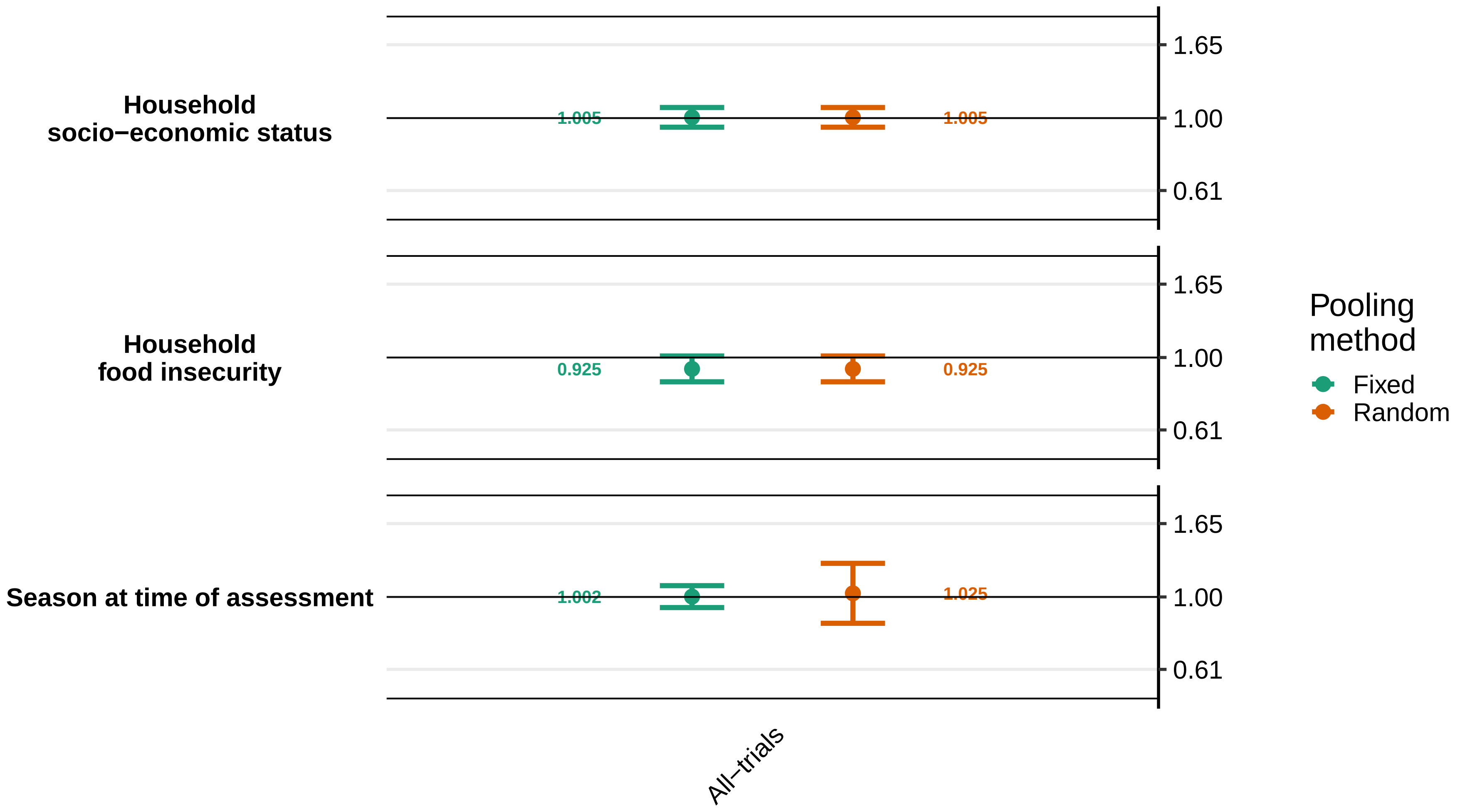

Supplemental figure 10S: Ratio of low vitamin A (retinol < 0.70 µmol/L) prevalence ratios

10S1: By maternal effect modifiers (insufficient comparisons)

Supplemental figure 10S: Ratio of low vitamin A (retinol < 0.70 µmol/L) prevalence ratios

10S2: By child effect modifiers (insufficient comparisons)

Supplemental figure 10S: Ratio of low vitamin A (retinol < 0.70 µmol/L) prevalence ratios

10S3: By household effect modifiers (insufficient comparisons)

Supplemental figure 10T: Difference in low vitamin A (retinol < 0.70 µmol/L) prevalence differences

10T1: By maternal effect modifiers (insufficient comparisons)

Supplemental figure 10T: Difference in low vitamin A (retinol < 0.70 µmol/L) prevalence differences

10T2: By child effect modifiers (insufficient comparisons)

Supplemental figure 10T: Difference in low vitamin A (retinol < 0.70 µmol/L) prevalence differences

10T3: By household effect modifiers (insufficient comparisons)

Supplemental figure 10U: Ratio of marginal vitamin A (retinol < 1.05 µmol/L) prevalence ratios

10U1: By maternal effect modifiers

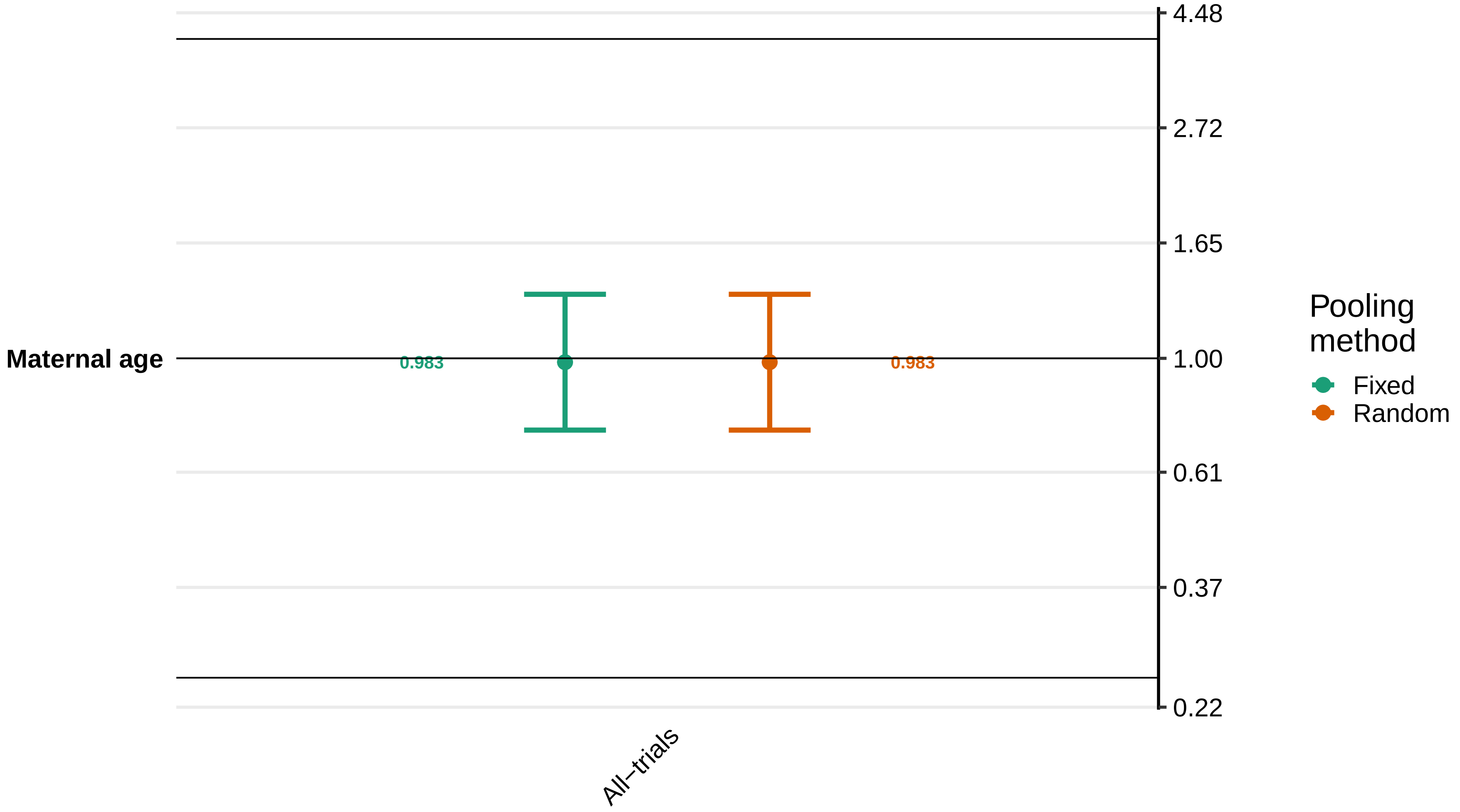

Supplemental figure 10U: Ratio of marginal vitamin A (retinol < 1.05 µmol/L) prevalence ratios

10U2: By child effect modifiers

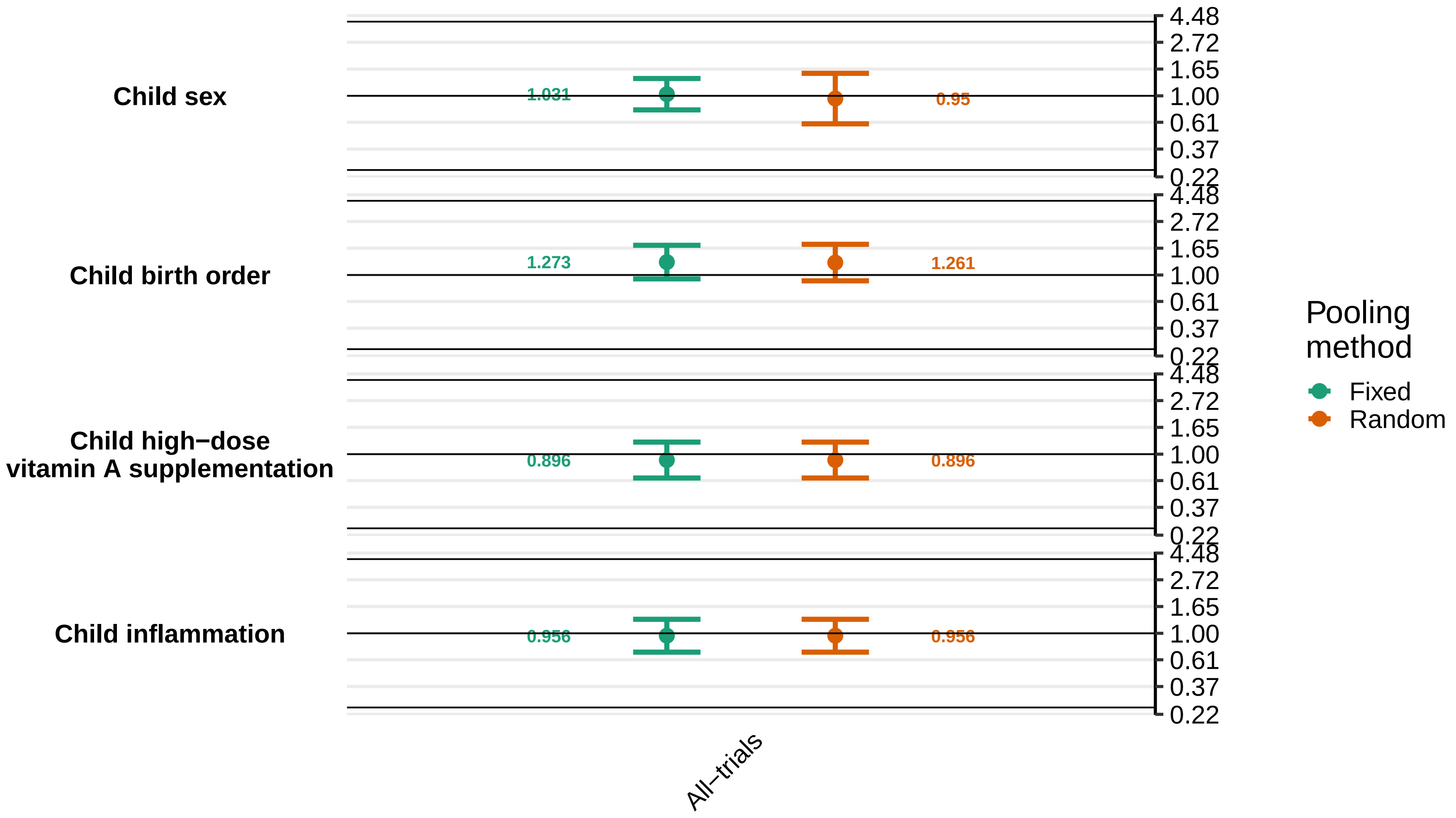

Supplemental figure 10U: Ratio of marginal vitamin A (retinol < 1.05 µmol/L) prevalence ratios

10U3: By household effect modifiers

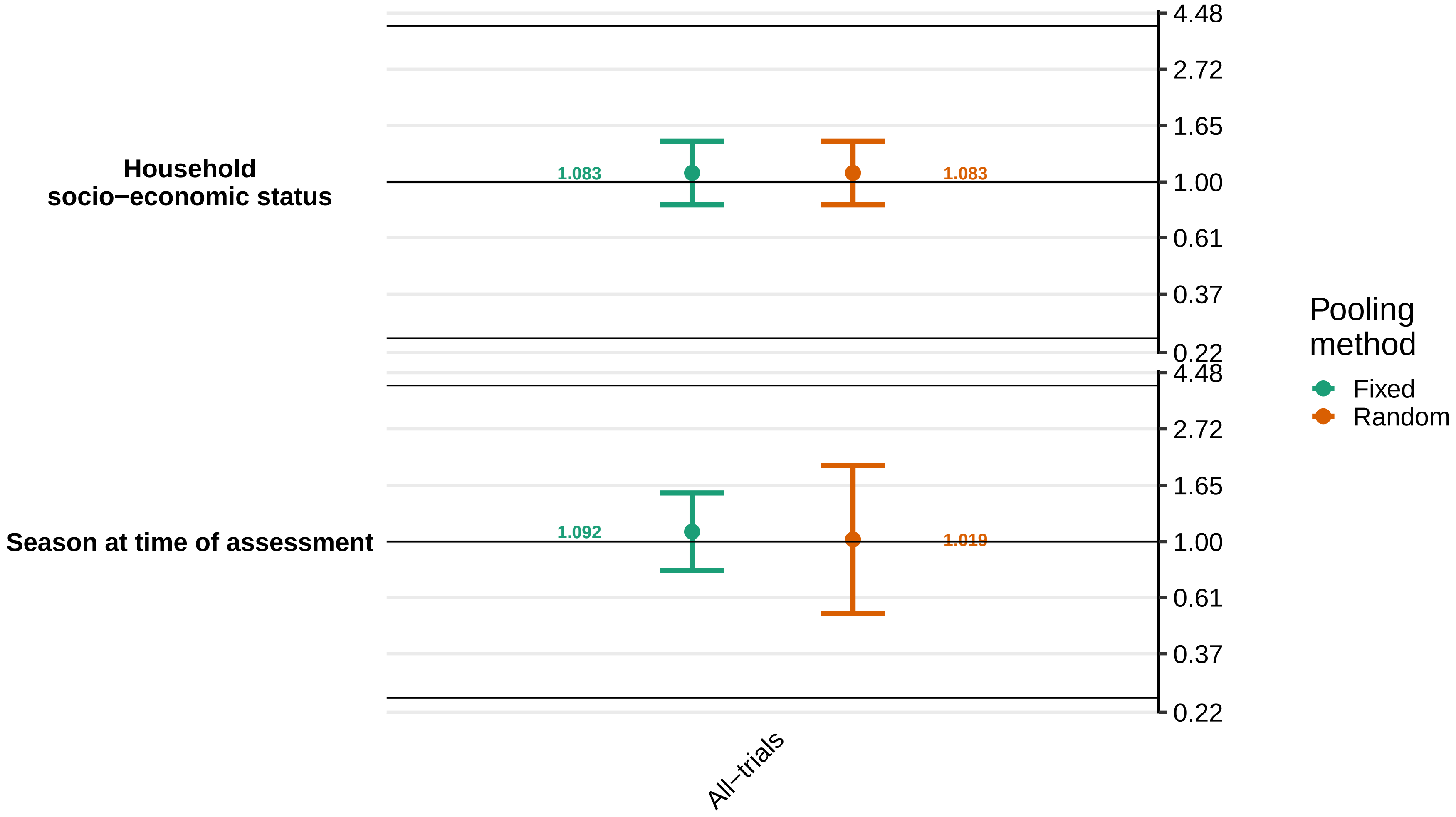

Supplemental figure 10V: Difference in marginal vitamin A (retinol < 1.05 µmol/L) prevalence differences

10V1: By maternal effect modifiers

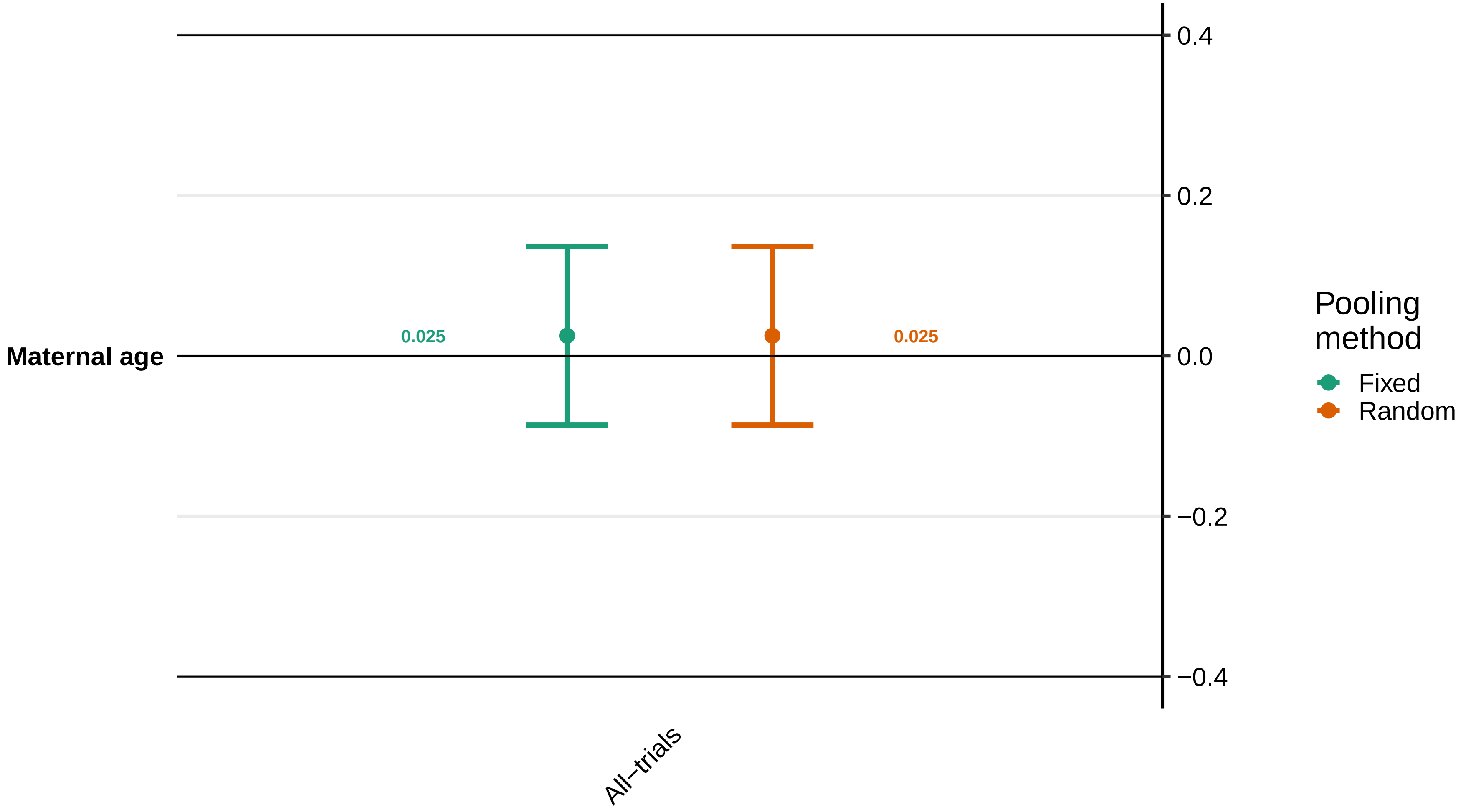

Supplemental figure 10V: Difference in marginal vitamin A (retinol < 1.05 µmol/L) prevalence differences

10V2: By child effect modifiers

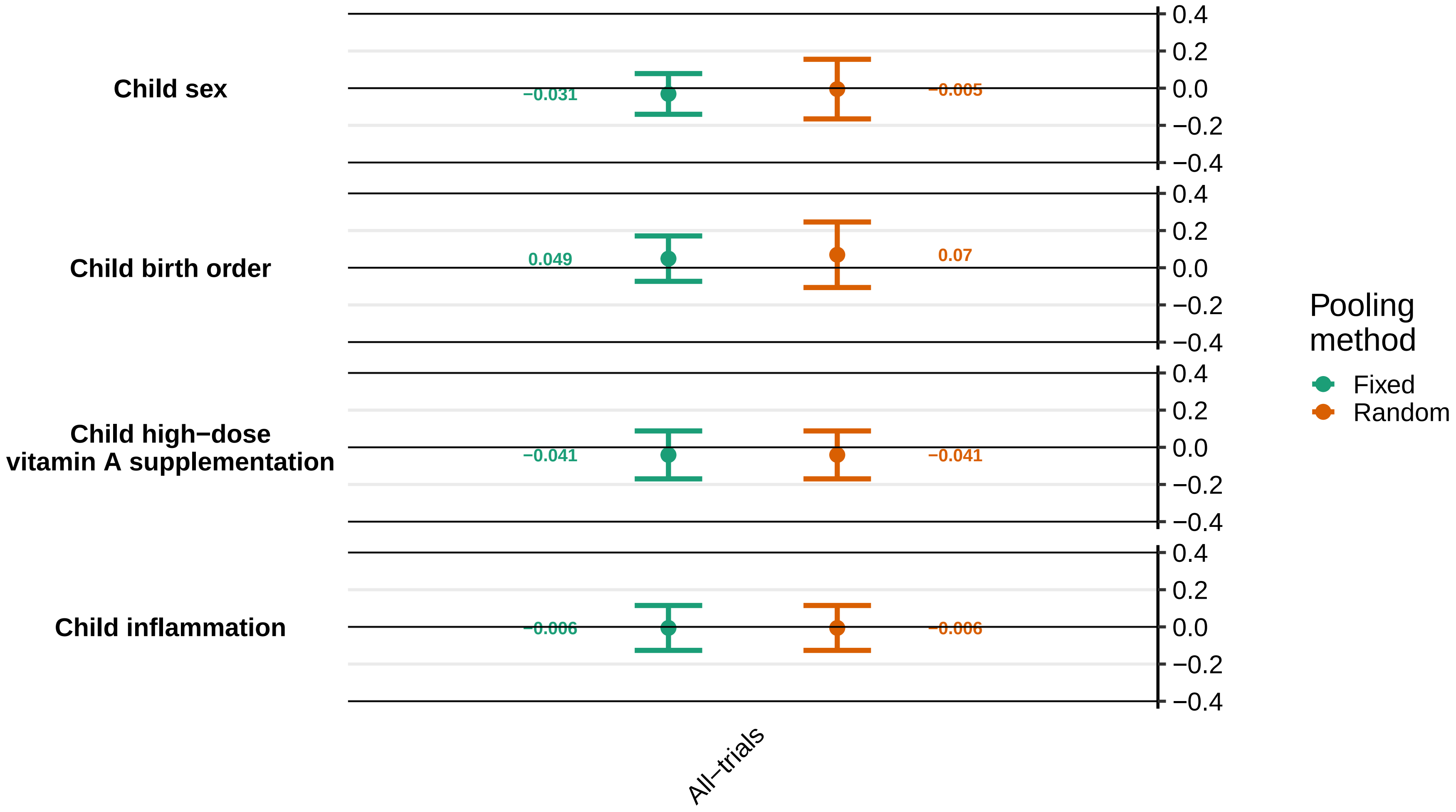

Supplemental figure 10V: Difference in marginal vitamin A (retinol < 1.05 µmol/L) prevalence differences

10V3: By household effect modifiers

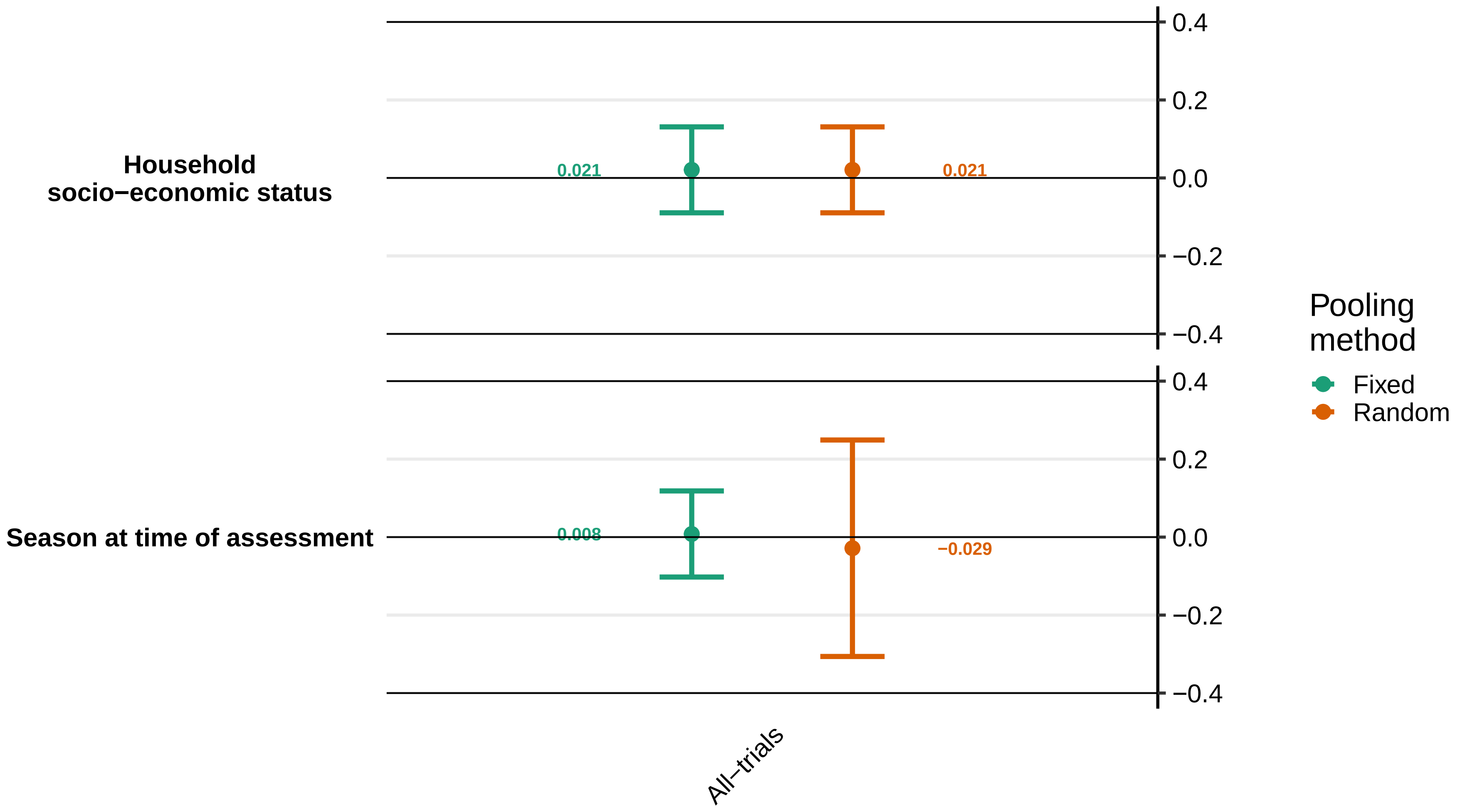

Supplemental figure 10W: Ratio of geometric mean ratio of retinol binding protein concentrations

10W1: By maternal effect modifiers

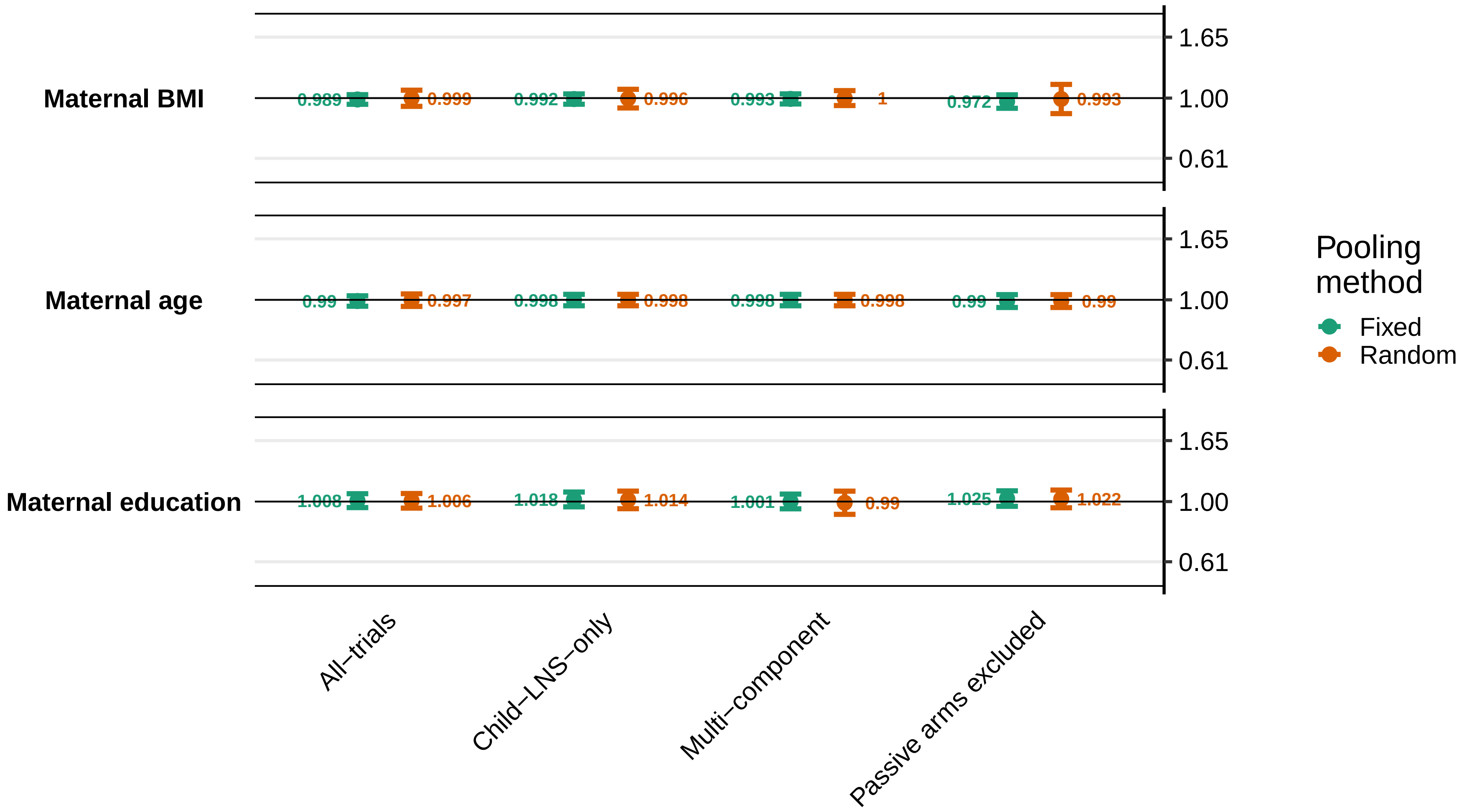

Supplemental figure 10W: Ratio of geometric mean ratio of retinol binding protein concentrations

10W2: By child effect modifiers

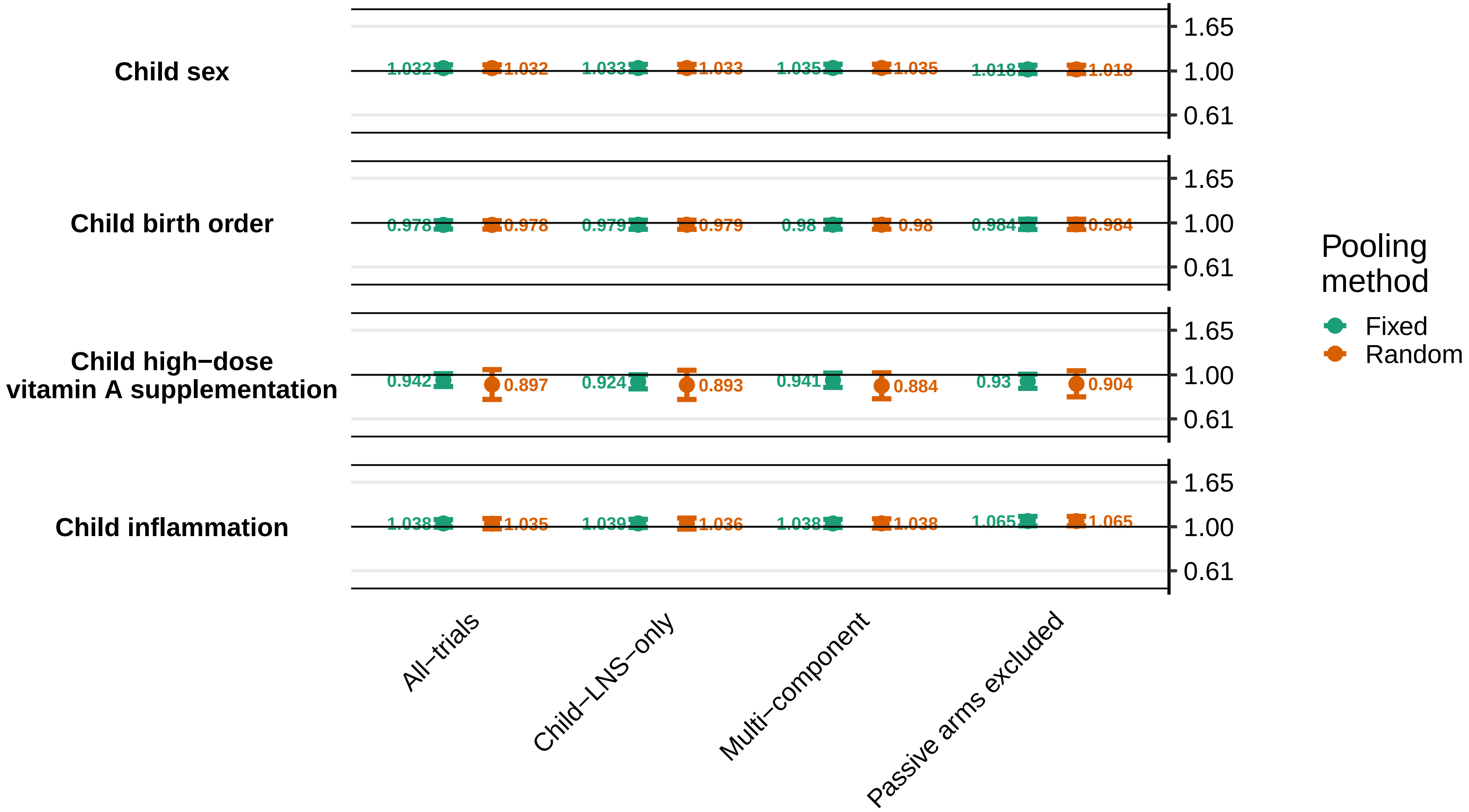

Supplemental figure 10W: Ratio of geometric mean ratio of retinol binding protein concentrations

10W3: By household effect modifiers

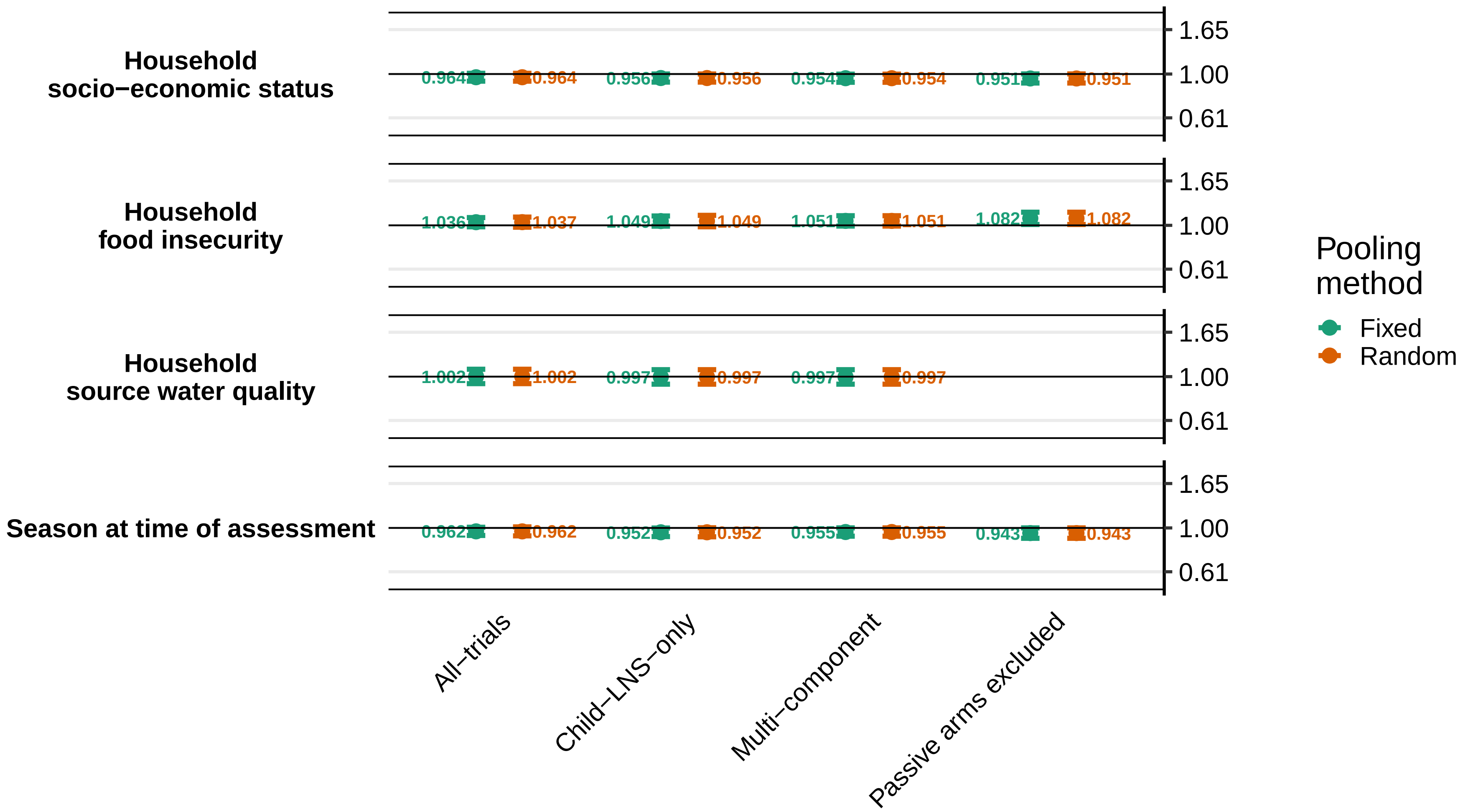

Supplemental figure 10X: Ratio of Low vitamin A status (RBP < 0.70 µmol/L) prevalence ratios

10X1: By maternal effect modifiers (insufficient comparisons)

Supplemental figure 10X: Ratio of Low vitamin A status (RBP < 0.70 µmol/L) prevalence ratios

10X2: By child effect modifiers (insufficient comparisons)

Supplemental figure 10X: Ratio of Low vitamin A status (RBP < 0.70 µmol/L) prevalence ratios

10X3: By household effect modifiers (insufficient comparisons)

Supplemental figure 10Y: Difference in low vitamin A status (RBP < 0.70 µmol/L) prevalence differences

10Y1: By maternal effect modifiers (insufficient comparisons)

Supplemental figure 10Y: Difference in low vitamin A status (RBP < 0.70 µmol/L) prevalence differences

10Y2: By child effect modifiers (insufficient comparisons)

Supplemental figure 10Y: Difference in low vitamin A status (RBP $<$ 0.70 µmol/L) prevalence differences

10Y3: By household effect modifiers (insufficient comparisons)

Supplemental figure 10Z: Ratio of marginal vitamin A status (RBP < 1.05 µmol/L) prevalence ratios

10Z1: By maternal effect modifiers

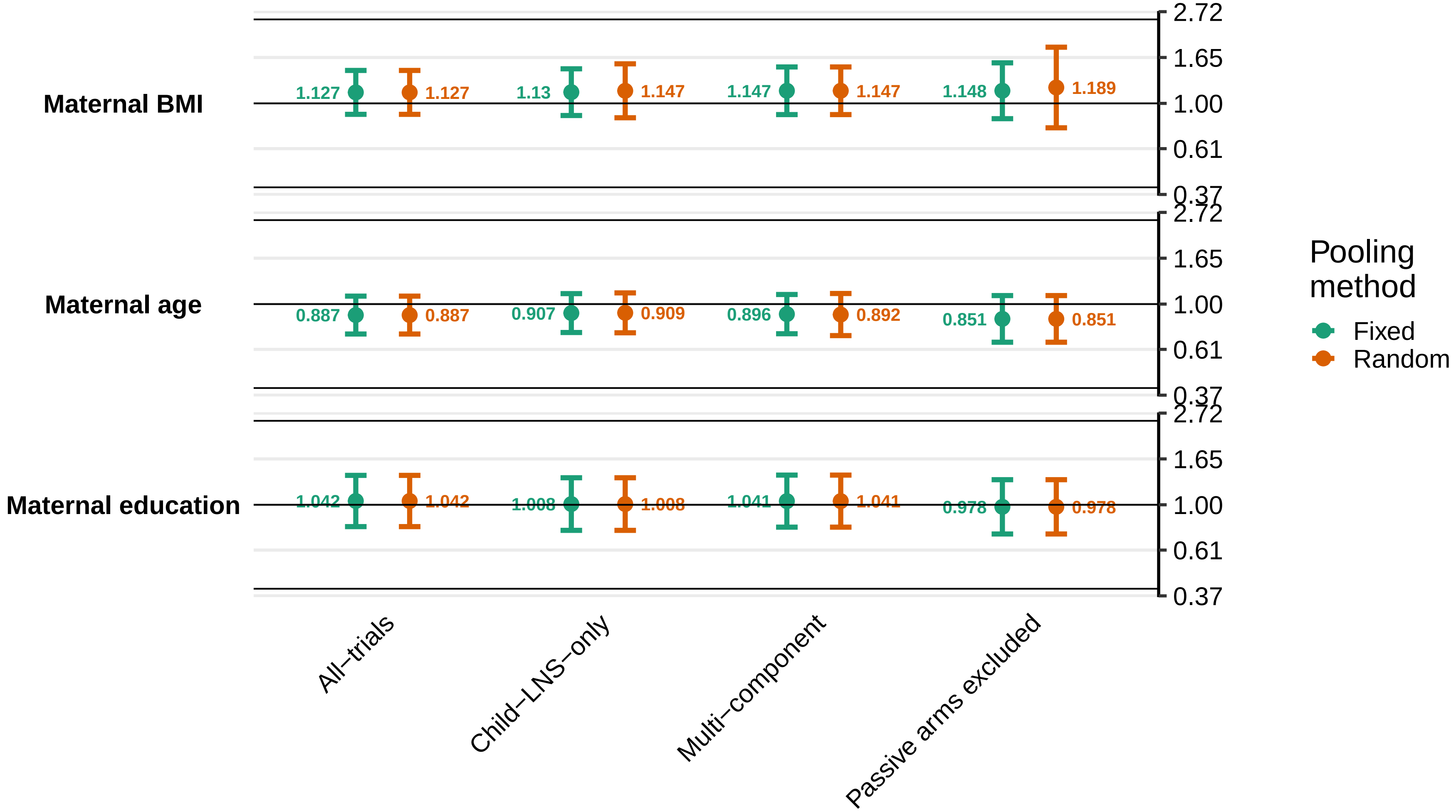

Supplemental figure 10Z: Ratio of marginal vitamin A status (RBP < 1.05 µmol/L) prevalence ratios

10Z2: By child effect modifiers

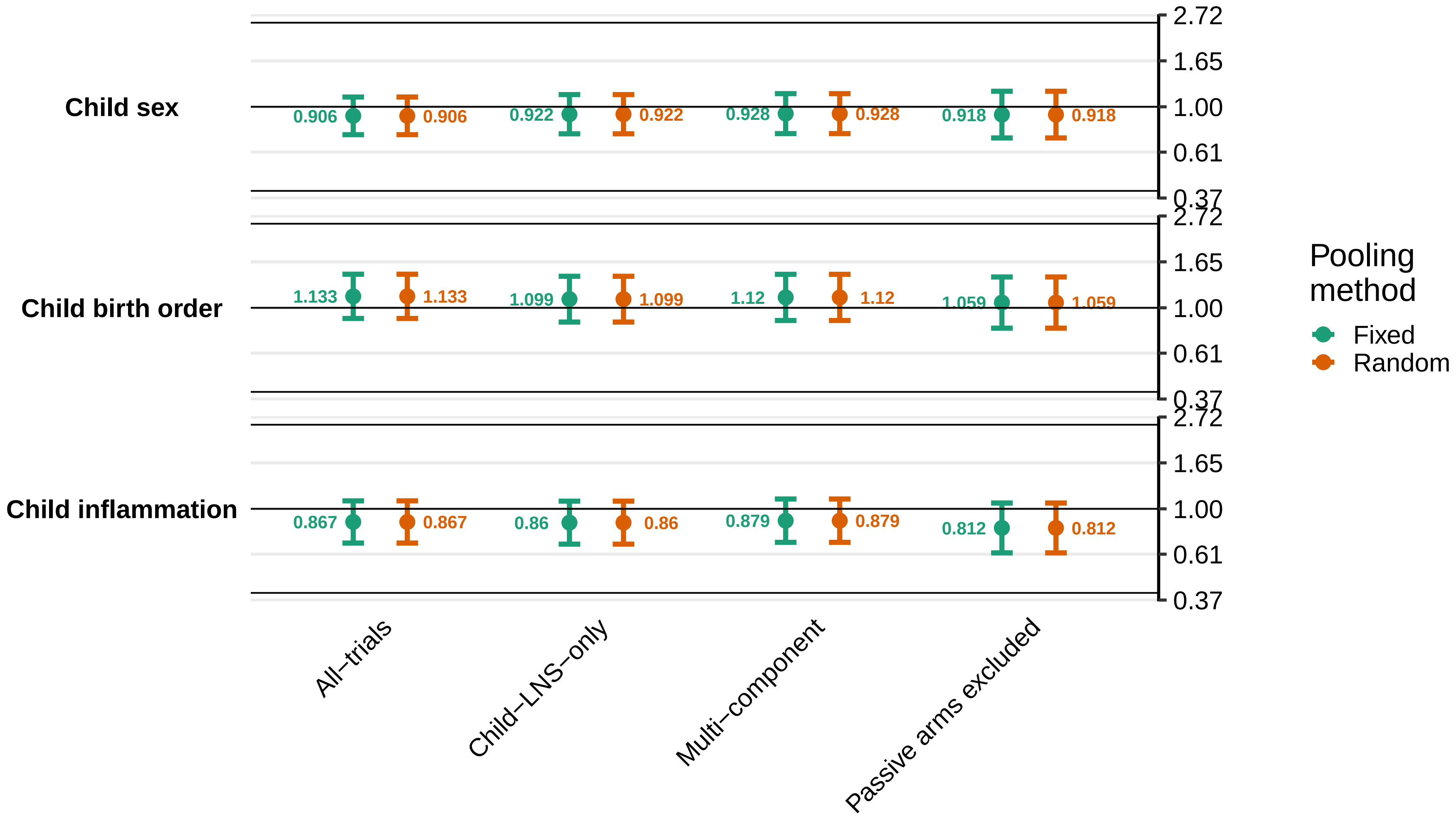

Supplemental figure 10Z: Ratio of marginal vitamin A status (RBP < 1.05 µmol/L) prevalence ratios

10Z3: By household effect modifiers

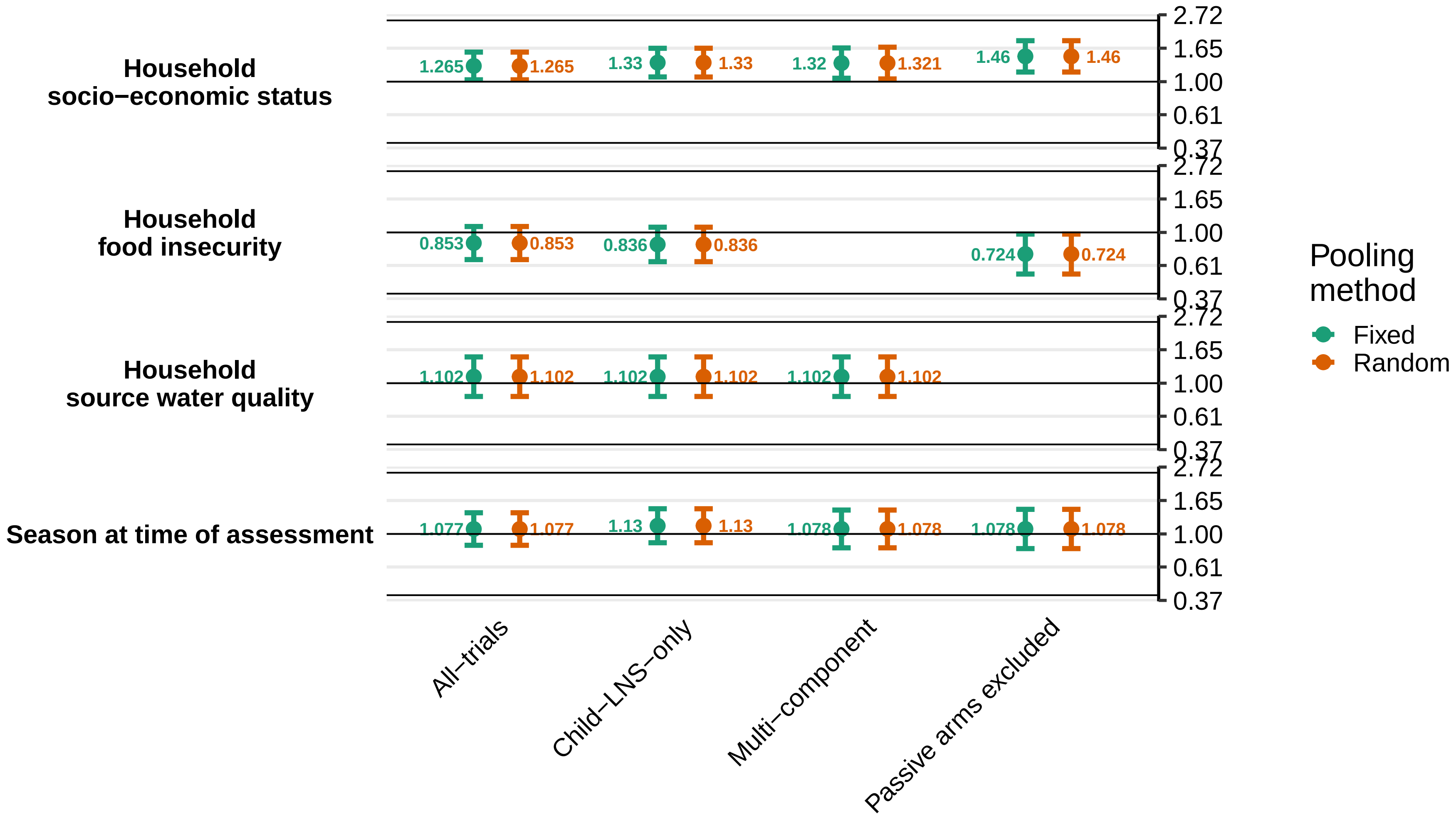

Supplemental figure 10AA: Difference in marginal vitamin A status (RBP < 1.05 µmol/L) prevalence differences

10AA1: By maternal effect modifiers

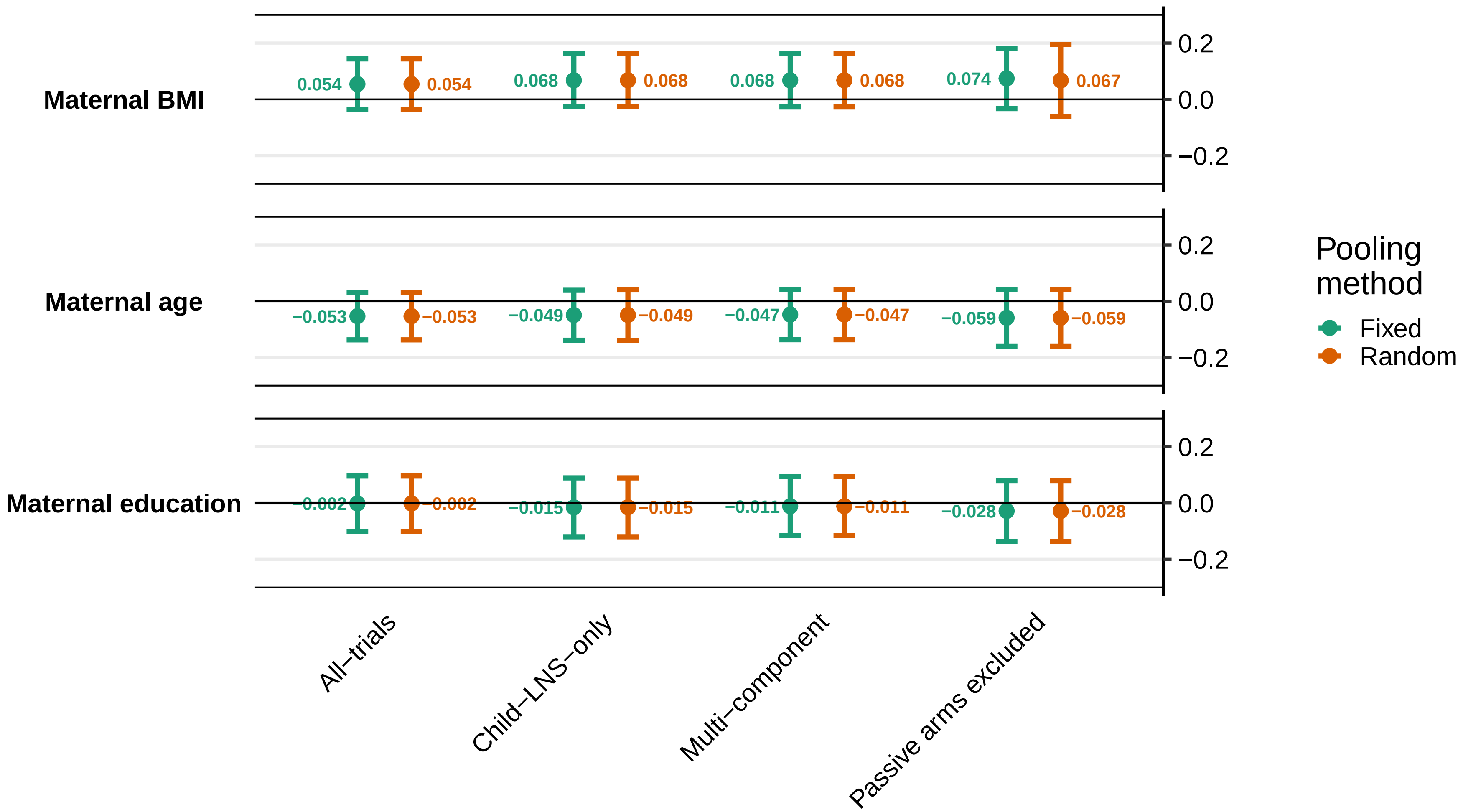

Supplemental figure 10AA: Difference in marginal vitamin A status (RBP < 1.05 µmol/L) prevalence differences

10AA2: By child effect modifiers

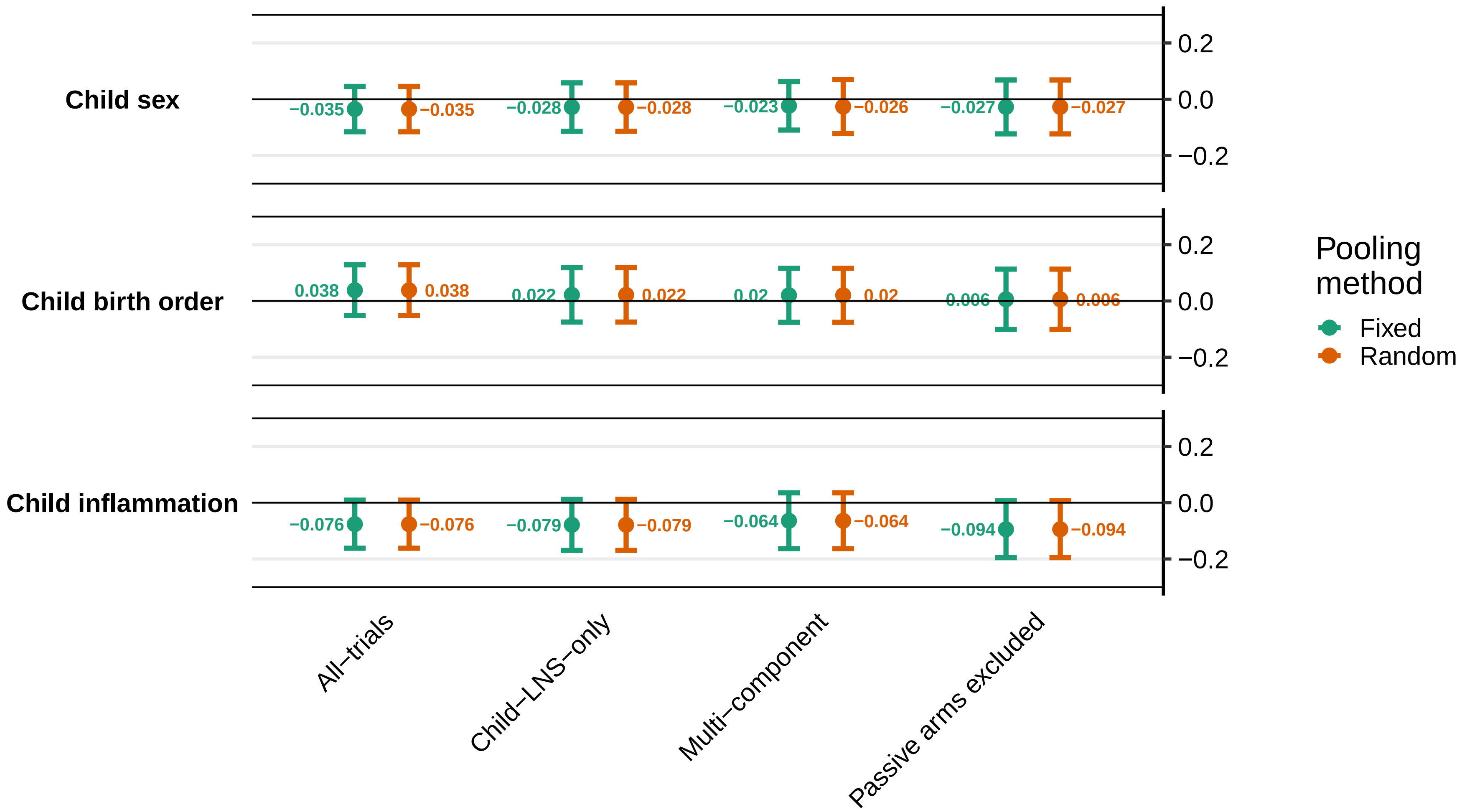

Supplemental figure 10AA: Difference in marginal vitamin A status (RBP < 1.05 µmol/L) prevalence differences

10AA3: By household effect modifiers

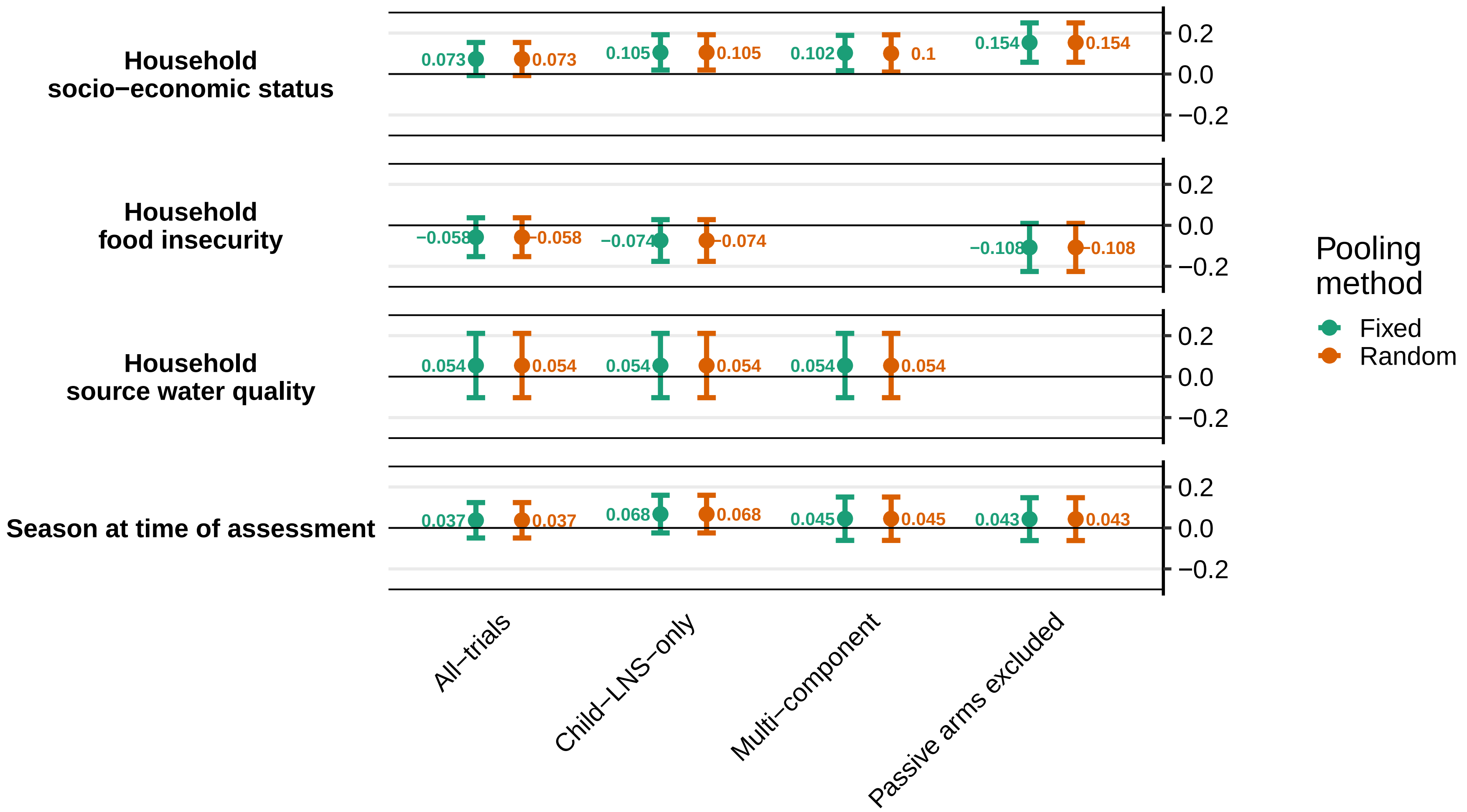

Supplement: nqab276_Supplemental_Files [file nqab276_supplemental_files.zip › 14_ipdb_suppfig10_20210401.pdf]
