# Supplementary material for: Characteristics that modify the effect of small-quantity lipid-based nutrient supplementation on child anemia and micronutrient status: an individual participant data meta-analysis of randomized controlled trials
Source: Am J Clin Nutr. 2021 Sep 29;114(Suppl 1):68S–94S. doi: 10.1093/ajcn/nqab276 (PMC8560313; doi:10.1093/ajcn/nqab276)
Supplement: nqab276_Supplemental_Files [file nqab276_supplemental_files.zip › 15_ipdb_suppfig11_20210401.pdf]

Online Supplemental Material

Characteristics that modify the effect of small-quantity lipid-based nutrient supplementation on child anemia and micronutrient status: an individual participant data meta-analysis of randomized controlled trials

Wessells *et al.* (2021)

Supplemental Figure 11. Overview of individual-level effect modification

	**Plasma zinc**	**Retinol**	**Marginal retinol**		**RBP**	**Marginal RBP**	
**Effect modifier**	GMR	GMR	PR	PD	GMR	PR	PD
Maternal BMI<br>lower (vs. higher)							
Maternal age<br>older (vs. younger)							
Maternal education<br>lower (vs. higher)							
Child sex<br>male (vs. female)							
Child birth order<br>later-born (vs. first-born)							
Child baseline malnutrition<br>acute (vs. non-acute)							
Child baseline anemia<br>anemic (vs. non-anemic)							
Child high-dose vitamin A<br>received (vs. did not receive)							
Child inflammation<br>CRP/AGP high (vs. not-high)							(C)
SES<br>lower (vs. higher)							
Food security<br>insecure (vs. secure)							
Water quality<br>unimproved (vs. improved)							
Sanitation<br>unimproved (vs. improved)							
Season<br>rainy (vs. dry)							

The reference group is the group expected to have the greatest potential to benefit. Green indicates stronger effect in the reference subgroup while blue indicates a stronger effect in the opposite subgroup. Subgroup definitions are provided in Box 2. Dark color indicates p-for-interaction < 0.05; light color indicates 0.05 < p < 0.1. The letter "C" indicates that the apparent effect modification is due to the cutoff effect; when "C" is in parentheses, it is partially explained by the cutoff effect. AGP, α-1-acid glycoprotein; CRP, C-reactive protein; GMR, geometric mean ratio; PD, prevalence difference; PR, prevalence ratio; RBP, retinol binding protein; SES, socio-economic status.
